# Integrative Bioinformatic Characterization of the HDAC6-Driven Cytoskeleton–Wnt Signaling Interface in Hepatocellular Carcinoma: Implications for Immune Modulation and Therapeutic Targeting

**DOI:** 10.3390/ijms27125201

**Published:** 2026-06-09

**Authors:** Ergul Bayram, Giuseppe Broggi, Durmus Ayan

**Affiliations:** 1 Medical Biochemistry, Nigde Omer Halisdemir University Research and Training Hospital, Nigde 51200, Türkiye; eyaylagul@hotmail.com; 2 Department of Medical and Surgical Sciences and Advanced Technologies “G.F. Ingrassia”, Anatomic Pathology, University of Catania, 95123 Catania, Italy; giuseppe.broggi@phd.unict.it; 3 Faculty of Medicine, Medical Biochemistry, Nigde Omer Halisdemir University, Nigde 51200, Türkiye

**Keywords:** hepatocellular carcinoma (HCC), HDAC6, TUBA1A, CTNNB1, bioinformatics

## Abstract

Hepatocellular carcinoma (HCC) remains a leading cause of cancer-related mortality worldwide, characterized by marked molecular heterogeneity, late-stage diagnosis, and limited therapeutic options. Emerging evidence highlights the interplay between cytoskeletal dynamics, epigenetic regulation, and oncogenic signaling pathways in hepatocarcinogenesis. Histone deacetylase 6 (HDAC6), a key regulator of cytoplasmic protein acetylation, modulates α-tubulin stability, while CTNNB1 (β-catenin) serves as a central effector of the Wnt signaling pathway. However, the existence and functional relevance of a coordinated HDAC6–TUBA1A–CTNNB1 regulatory axis in HCC remain insufficiently explored. We conducted a comprehensive integrative bioinformatic analysis using multiple publicly available datasets and platforms, including TCGA, GEO, GEPIA3, TNMplot, UALCAN, TIMER2.0, STRING, ENCORI, HPA, TargetScan, miRDB, CRISPRdb, GSCALite, and exoRBase. Gene expression, promoter methylation, survival associations, immune infiltration, regulatory RNA interactions, and therapeutic targetability were systematically evaluated. HDAC6 expression was significantly downregulated in HCC tissues, whereas TUBA1A and CTNNB1 were upregulated. Reduced HDAC6 expression was associated with poorer survival outcomes, while TUBA1A and CTNNB1 showed no significant prognostic value. Methylation analysis revealed gene-specific epigenetic alterations, including hypomethylation of CTNNB1 and differential methylation patterns in HDAC6 and TUBA1A. Immune infiltration analysis demonstrated that HDAC6 expression positively correlated with cytotoxic immune cell populations and negatively with immunosuppressive subsets. Regulatory network analyses identified lncRNA–miRNA–mRNA interactions, particularly involving SNHG1. Furthermore, in silico CRISPR targetability and extracellular vesicle (EV) transcript profiling suggested potential translational applicability of this axis. Our findings support a hypothesis of the existence of a dysregulated HDAC6–α-tubulin–β-catenin axis in HCC, linking cytoskeletal remodeling with oncogenic signaling and immune modulation. This axis may indicate a promising candidate for biomarker development and targeted therapeutic strategies, warranting further experimental validation.

## 1. Introduction

Hepatocellular carcinoma (HCC) is among the most prevalent and lethal malignancies worldwide, with high mortality rates driven by late diagnosis, therapeutic resistance, and extensive molecular heterogeneity [1,2,3]. Despite advances in surveillance and treatment strategies, clinical outcomes remain poor, underscoring the urgent need to identify robust biomarkers and novel therapeutic targets. A deeper understanding of the molecular mechanisms underlying HCC pathogenesis is therefore critical for improving patient management [4].

HCC development is governed by a complex interplay of epigenetic dysregulation, cytoskeletal remodeling, oncogenic signaling pathways, and tumor microenvironment interactions [5,6,7,8]. These processes collectively influence tumor proliferation, invasion, metastasis, and resistance to therapy [9,10,11]. Among the key regulators implicated in these mechanisms is histone deacetylase 6 (HDAC6), a unique member of the class IIb HDAC family primarily localized in the cytoplasm. Unlike nuclear HDACs, HDAC6 targets non-histone substrates, including α-tubulin, heat shock protein 90 (Hsp90), and cortactin, thereby regulating microtubule dynamics, protein folding, and cellular motility [12,13,14].

Through its deacetylase activity, HDAC6 plays a pivotal role in controlling cytoskeletal organization and intracellular trafficking. These functions are closely linked to cancer progression, particularly in processes such as cell migration, invasion, and chemoresistance [15,16,17]. Consequently, HDAC6 has emerged as an attractive target in cancer therapeutics, with increasing interest in selective inhibitors and combinatorial treatment strategies aimed at modulating interconnected oncogenic pathways [18].

TUBA1A, a major α-tubulin isoform, is a critical structural component of microtubules and a direct substrate of HDAC6. Deacetylation of TUBA1A by HDAC6 affects microtubule stability and dynamics, influencing key cellular processes including mitosis, intracellular transport, and cell motility [19]. Dysregulation of microtubule dynamics has been strongly associated with tumor progression and metastatic potential in various cancers, including HCC [20,21]. However, the specific role of TUBA1A in hepatocarcinogenesis remains insufficiently characterized.

In parallel, CTNNB1, which encodes β-catenin, is a central component of the Wnt/β-catenin signaling pathway—a well-established driver of HCC [22]. Aberrant activation of this pathway promotes hepatocyte proliferation, metabolic reprogramming, epithelial–mesenchymal transition, and immune evasion [23,24]. Notably, HDAC6 has been shown to modulate β-catenin activity through post-translational deacetylation, suggesting a mechanistic link between cytoskeletal regulation and oncogenic signaling [25].

Recent studies have begun to highlight potential interactions among HDAC6, TUBA1A, and CTNNB1, suggesting the existence of a coordinated regulatory network that integrates cytoskeletal dynamics with signal transduction [15]. However, the extent to which this axis contributes to HCC development, progression, and therapeutic response remains poorly understood.

Although HDAC6, tubulin-associated cytoskeletal remodeling, and CTNNB1/Wnt signaling have each been individually implicated in cancer biology, their integrated regulatory relationship in HCC has not been systematically characterized within a unified multi-omics bioinformatic framework. The novelty of the present study therefore lies in evaluating HDAC6, TUBA1A, and CTNNB1 as an interconnected regulatory axis rather than as isolated molecular events, integrating transcriptomic, epigenetic, protein-level, survival, immune infiltration, interactome, non-coding RNA, extracellular vesicle, CRISPR targetability, and drug-sensitivity evidence. This approach provides a hypothesis-generating translational framework for prioritizing candidate mechanisms and therapeutic vulnerabilities in HCC.

In this study, we hypothesized that dysregulation of HDAC6 contributes to hepatocarcinogenesis through a coordinated HDAC6–TUBA1A–CTNNB1 regulatory axis. To test this hypothesis, we performed a comprehensive multi-omics bioinformatic analysis to investigate gene expression patterns, epigenetic modifications, survival associations, immune microenvironment interactions, and regulatory RNA networks. Additionally, we evaluated the translational potential of this axis, including its suitability for CRISPR-based targeting and its detectability in extracellular vesicles.

## 2. Results

### 2.1. GEPIA3-Based Functional and Clinical Interpretation of HDAC6, TUBA1A and CTNN (CTNNB1)

**Figure 1:**
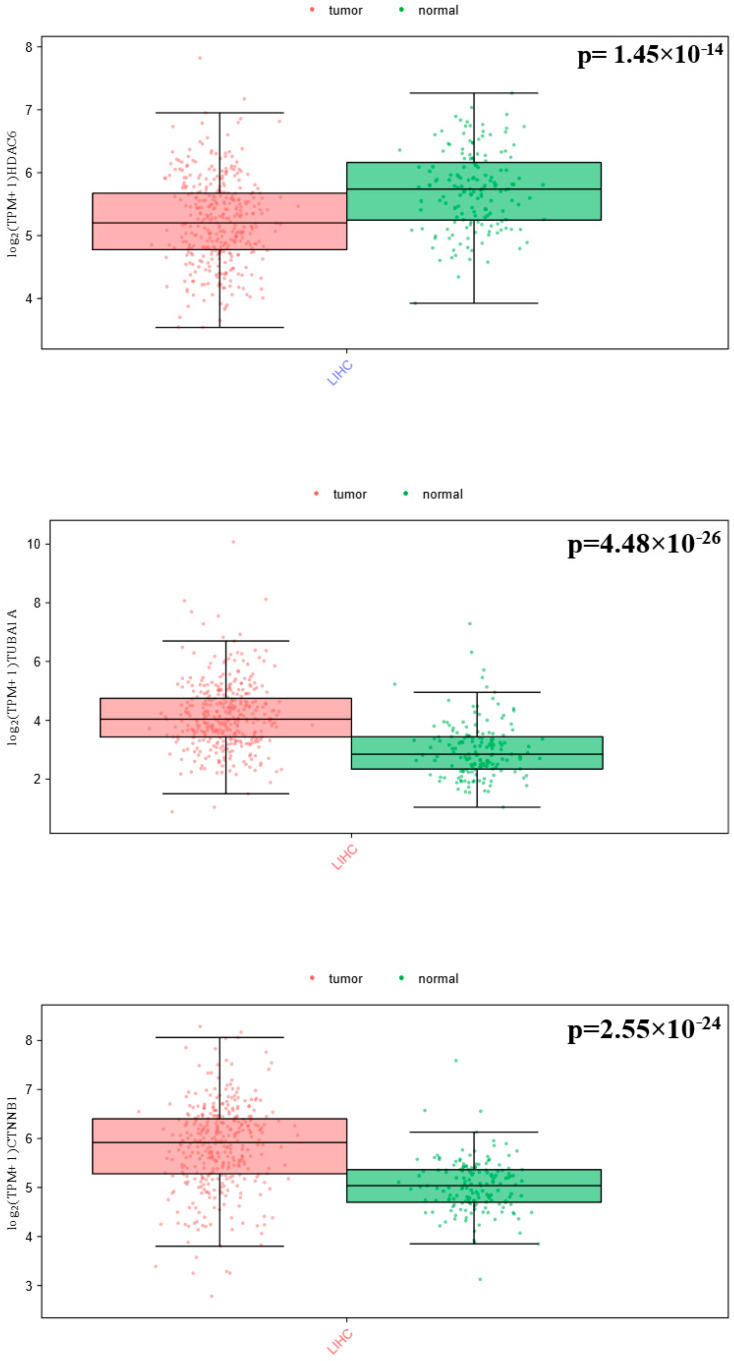
GEPIA3 tumor–normal expression analysis of HDAC6, TUBA1A, and CTNNB1 in LIHC. Box plots display differential gene expression between hepatocellular carcinoma (tumor, red) and normal adjacent tissue (green). HDAC6 is significantly downregulated in tumors (*p* = 1.45 × 10^−14^), while TUBA1A and CTNNB1 show significant upregulation in tumor samples (*p* = 4.48 × 10^−26^ and *p* = 2.55 × 10^−24^, respectively). Expression values are shown as log_2_(TPM + 1).

In the GEPIA3 tumor–normal expression analysis of the LIHC cohort, all three genes, HDAC6, TUBA1A, and CTNNB1, demonstrated statistically significant differential expression between hepatocellular carcinoma tissues and normal liver samples. HDAC6 levels were statistically downregulated in tumor tissues compared with normal adjacent tissue (*p* = 1.45 × 10^−12^) in HCC. In contrast, TUBA1A expression was statistically upregulated in tumor samples (*p* = 4.48 × 10^−26^). Similarly, CTNNB1 (β-catenin) showed significantly higher expression in tumor tissues relative to normal adjacent tissue (*p* = 2.55 × 10^−24^) (Figure 1).

### 2.2. Human Protein Atlas Immunohistochemistry as Protein-Level Validation

**Figure 2:**
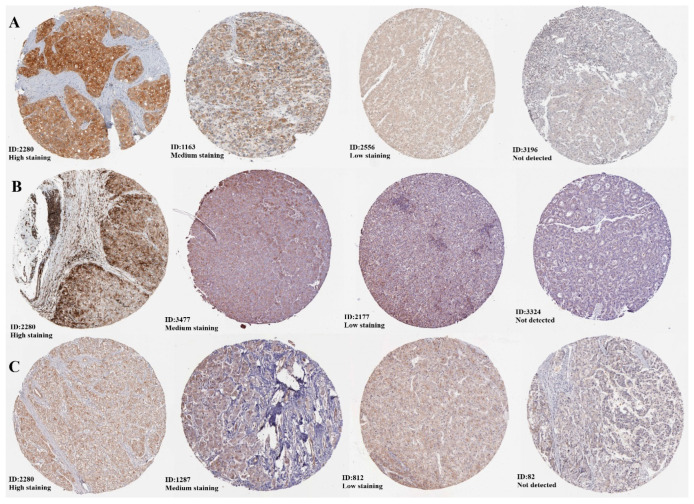
Immunohistochemical staining of HDAC6 (**A**), TUBA1A (**B**), and CTNNB1 (**C**) in LIHC. Representative tumor sections show variable protein expression across cases. HDAC6 (**A**), TUBA1A (**B**), and CTNNB1 (**C**) display high, medium, low, or undetectable staining intensities in LIHC (Scale bar = 200 μm). The ID (Identification) represents a number obtained from each patient sample.

Immunohistochemical analysis of LIHC tissue microarrays revealed heterogeneous yet quantifiable protein expression patterns for HDAC6, TUBA1A, and CTNNB1. HDAC6 (A) exhibited variable cytoplasmic staining, ranging from strong and diffuse to undetectable across different specimens. TUBA1A (B) demonstrated a similar gradient. CTNNB1 (C) also showed variable cytoplasmic immunoreactivity, ranging from strong and diffuse to undetectable across samples (Figure 2). Official HGNC gene symbols (HDAC6, TUBA1A, and CTNNB1) were queried to assess protein expression patterns in normal liver and hepatocellular carcinoma tissues. Immunohistochemical (IHC) staining images were assessed based on the HPA-standardized annotation criteria, which encompass staining intensity (categorized as negative, low, medium, or high), staining distribution, and subcellular localization. For HDAC6, anti-HDAC6 antibodies (catalog numbers: CAB004236, HPA003714 and HPA026321) were used. For CTNNB1, anti-CTNNB1 antibodies (catalog numbers: HPA0291160, CAB000108 and CAB001950) were used. For TUBA1A, anti-TUBA1A antibodies (catalog numbers: CAB008686 and HPA039247) were used.

### 2.3. Normal and Tumor Comparison Results via TNMplot

**Figure 3:**
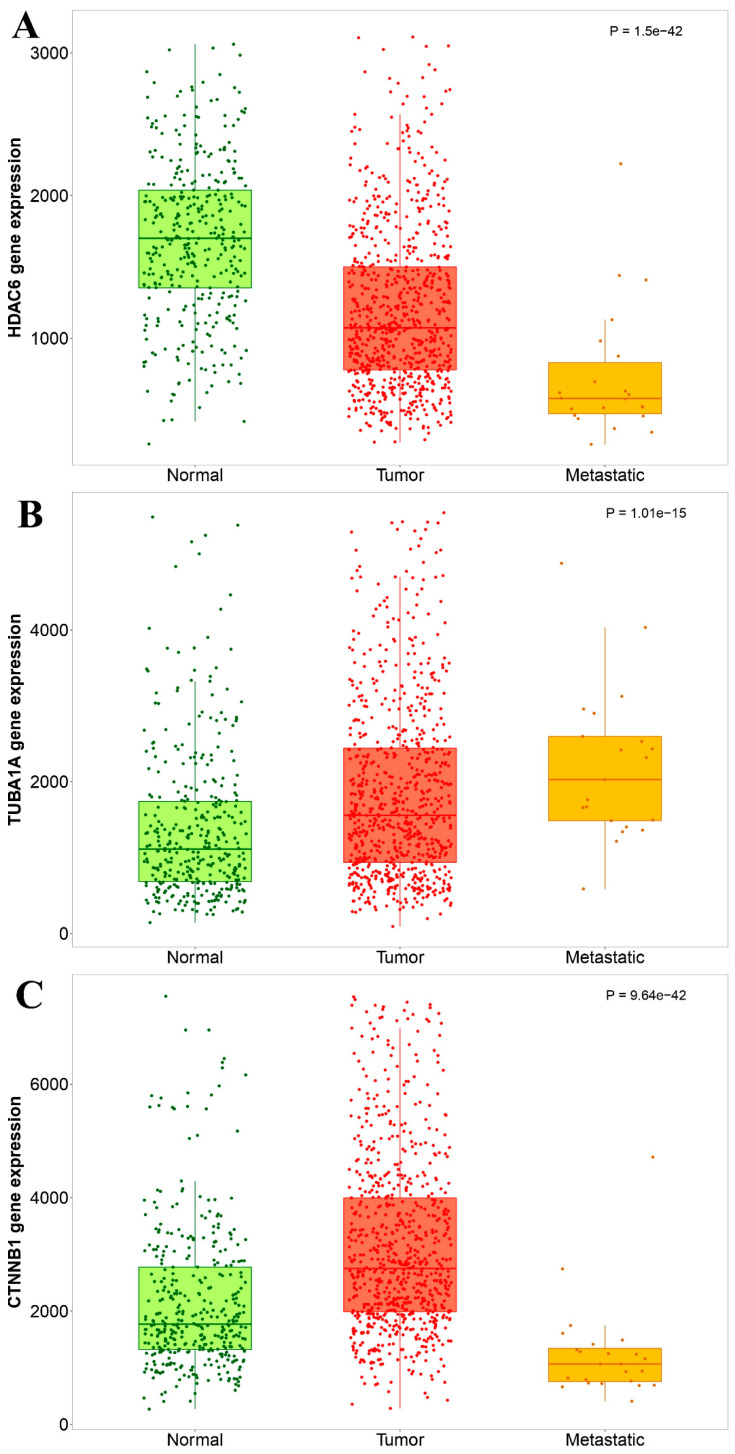
Differential expression of HDAC6, TUBA1A, and CTNNB1 across normal, primary tumor, and metastatic liver cancer tissues. (**A**) HDAC6 (**B**) TUBA1A (**C**) CTNNB1. Each dot represents an individual sample; boxplots display median, interquartile range, and overall distribution. Colors indicate sample groups (green = normal, red = tumor, yellow = metastatic). Differences in gene expression across normal, tumor, and metastatic tissues were first evaluated using the Kruskal–Wallis test, a non-parametric method appropriate for comparing more than two independent groups. The test indicated significant overall differences among the groups for all three genes (HDAC6: *p* = 1.5 × 10^–42^; TUBA1A: *p* = 1.01 × 10^–15^; CTNNB1: *p* = 9.64 × 10^–42^). Following the significant Kruskal–Wallis results, Dunn’s post hoc test was applied to perform pairwise comparisons between groups with adjustment for multiple testing.

HDAC6 expression was downregulated in tumor and metastatic samples compared with normal tissue (*p* = 4.35 × 10^−40^ and *p* = 3.61 × 10^−12^, respectively). HDAC6 expression was consequently found to be downregulated in metastatic liver tissues in comparison to tumors (*p* = 0.0013).TUBA1A expression was upregulated in tumor and metastatic groups relative to normal samples (*p* = 4.18 × 10^−15^ and *p* = 3.94 × 10^−6^, respectively). Furthermore, the expression of TUBA1A was significantly increased in metastatic liver tissues compared to primary tumors (*p* = 0.014). CTNNB1 expression was upregulated in primary tumor tissue compared to normal tissue (*p* = 7.99 × 10^−35^), while CTNNB1 expression was downregulated in metastatic liver tissues compared to normal tissue (*p* = 0.00014). Additionally, CTNNB1 expression was upregulated in tumor tissue compared to metastatic liver tissues (*p* = 8.37 × 10^−14^) (Figure 3).

### 2.4. Comparative Promoter Methylation Analysis by Tissue Type and TP53 Mutation Status

**Figure 4:**
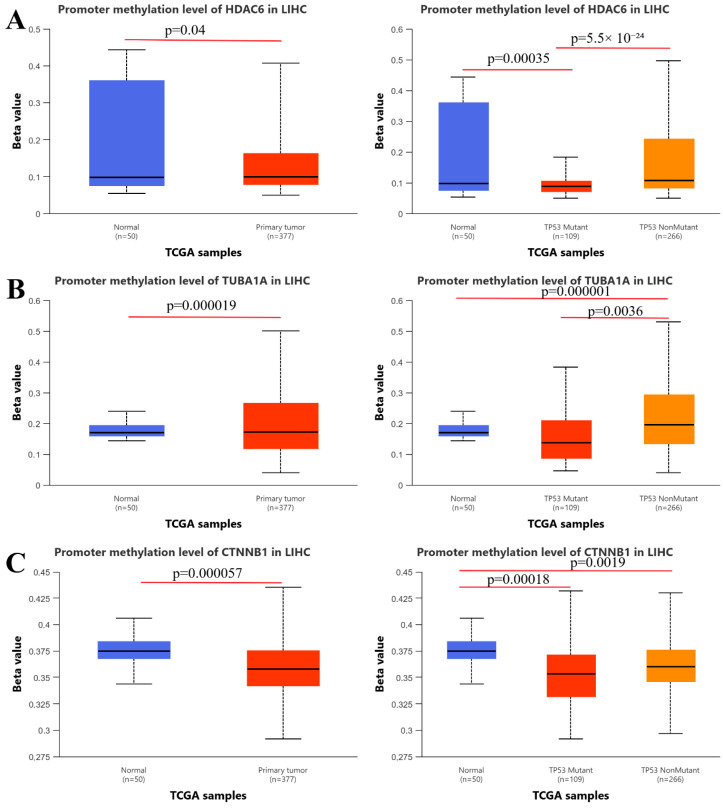
Promoter methylation profiles of HDAC6, TUBA1A, and CTNNB1 in LIHC based on UALCAN analysis. (**A**) Promoter methylation levels of HDAC6 in normal liver tissues (n = 50) versus primary LIHC tumors (n = 377) and stratified further by TP53 mutation status (TP53-mutant, n = 109; TP53-nonmutant, n = 266). (**B**) Promoter methylation levels of TUBA1A in normal versus LIHC tumor tissues and across TP53-mutant and TP53-nonmutant subgroups. (**C**) Promoter methylation levels of CTNNB1 in normal and LIHC tumor tissues, and according to TP53 mutation status.

UALCAN analysis revealed significant alterations in promoter methylation levels of HDAC6, TUBA1A, and CTNNB1 between normal adjacent tissue and tumor tissues, as well as across TP53 mutation categories in LIHC. Promoter methylation of HDAC6 was higher (hypermethylated) in normal tissues compared with primary tumors, which exhibited significant hypomethylation (*p* = 0.04). In the TP53-stratified analysis, TP53-mutant tumors showed pronounced hypomethylation relative to normal samples (*p* = 0.00035) while TP53-nonmutant tumors showed hypermethylation relative to TP53-mutant tumors (5.5 × 10^−24^). TUBA1A was hypermethylated in tumor tissues compared with normal adjacent tissue (*p* = 0.000019). TP53-nonmutant tumors exhibited significantly higher levels of hypermethylation in comparison to both normal samples and TP53-mutant tumors, with *p*-values of 0.000001 and 0.0036, respectively.CTNNB1 promoter regions were significantly hypomethylated in tumors compared with normal adjacent tissue (*p* = 0.000057). TP53-mutant and TP53-nonmutant groups both showed significant hypomethylation relative to normal adjacent tissue (*p* = 0.00018 and *p* = 0.0019, respectively) (Figure 4).

### 2.5. HDAC6, TUBA1A, and CTNNB1 Expression According to KM-Plotter Survival Analysis

**Figure 5:**
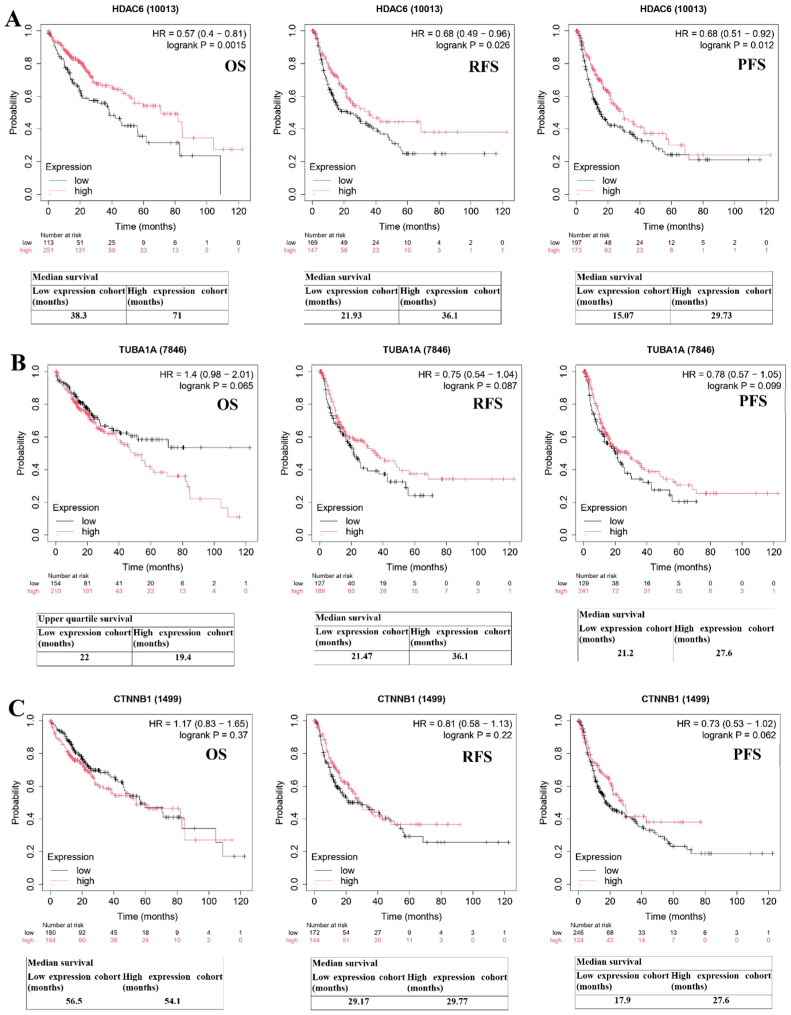
KM-Plotter survival analysis of HDAC6, TUBA1A, and CTNNB1 in LIHC. (**A**) Overall survival (OS), relapse-free survival (RFS), and progression-free survival (PFS) curves for HDAC6 based on high and low expression groups. (**B**) OS, RFS, and PFS curves for TUBA1A according to expression levels. (**C**) OS, RFS, and PFS curves for CTNNB1 comparing high- and low-expression cohorts. Hazard ratios (HRs) and log-rank *p*-values were obtained from KM-Plotter.

For HDAC6, high expression was associated with longer overall survival (OS) (HR = 0.57, *p* = 0.0015), relapse-free survival (RFS) (HR = 0.68, *p* = 0.026), and progression-free survival (PFS) (HR = 0.68, *p* = 0.012) (Figure 5A). For TUBA1A, no statistically significant differences were observed for OS (HR = 1.4, *p* = 0.065), RFS (HR = 0.75, *p* = 0.087), or PFS (HR = 0.78, *p* = 0.099) (Figure 5B). For CTNNB1, OS (HR = 1.17, *p* = 0.37), RFS (HR = 0.81, *p* = 0.22), and PFS (HR = 0.73, *p* = 0.062) showed no significant differences between high- and low-expression groups (Figure 5C).

### 2.6. STRING-Based Functional Interaction Mapping of HDAC6, TUBA1A, and CTNNB1

**Figure 6:**
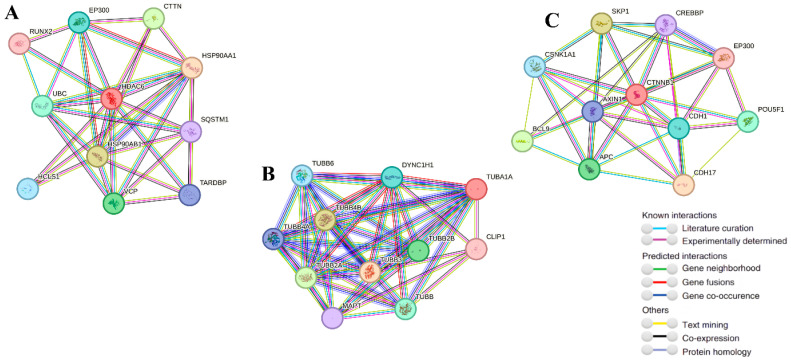
STRING Protein–Protein Interaction Networks of HDAC6 (**A**), TUBA1A (**B**), and CTNNB1 (**C**).

The STRING database was used to identify proteins interacting with HDAC6, TUBA1A, and CTNNB1 (Figure 6). The HDAC6 network includes interactions with proteins such as HSP90AA1, HSP90AB1, SQSTM1, VCP, EP300, CTTN, UBC, RUNX2, TARDBP, and HCLS1 (Figure 6A). The TUBA1A network contains multiple tubulin family members (TUBB6, TUBB4A, TUBB4B, TUBB2B, TUBB2A, TUBB3, TUBB) as well as MAPT, CLIP1, and DYNC1H1 (Figure 6B). The CTNNB1 network consists of interacting proteins including AXIN1, APC, EP300, CREBBP, SKP1, CSNK1A1, BCL9, CDH1, CDH17, and POU5F1 Figure 6C. Known and predicted interactions were generated based on literature curation, experimental evidence, gene neighborhood, gene fusions, gene co-occurrence, co-expression, text mining, and protein homology. The combination score of genes is shown in Table 1.

### 2.7. The miRNAs Associated with HDAC6, TUBA1A and CTNNB1

**Figure 7:**
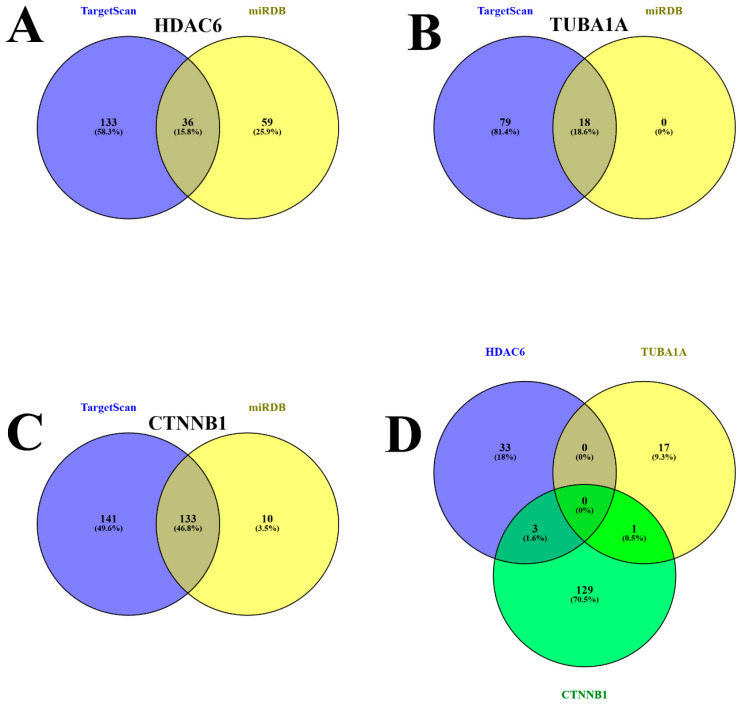
A three-way Venn diagram illustrates the shared and unique predicted miRNAs targeting HDAC6 (**A**), TUBA1A (**B**), and CTNNB1 (**C**). The diagram shows the number of miRNAs predicted to target HDAC6 (blue), TUBA1A (yellow), and CTNNB1 (green), with unique and overlapping miRNA counts displayed in each section (**D**).

The miRNAs associated with HDAC6, TUBA1A and CTNNB1 with a combination of TargetScanHuman8.0 and miRDB databases are shown in Figure 7A–C and Table 2. Overlap of predicted miRNAs targeting HDAC6, TUBA1A, and CTNNB1 based on TargetScan and miRDB Venn diagrams shows the number of predicted miRNAs identified uniquely by TargetScan (blue), uniquely by miRDB (yellow), and those shared by both databases (overlap). (A) HDAC6: 133 miRNAs identified exclusively by TargetScan, 59 miRNAs identified solely by miRDB, and 36 miRNAs identified by both databases. (B) TUBA1A: 79 miRNAs identified exclusively by TargetScan, no miRNAs identified solely by miRDB, and 18 miRNAs identified by both databases. (C) CTNNB1: 141 miRNAs identified exclusively by TargetScan, 10 miRNAs identified solely by miRDB, and 133 miRNAs identified by both databases. While common miRNAs in HDAC and CTNNB1 are hsa-miR-200a-3p, hsa-miR-141-3p, hsa-miR-548n, common miRNA in TUBA1A and CTNNB1is hsa-miR-330-3p. miRNAs that were associated with at least two genes were selected for expression analysis.

Common miRNAs are shown in Figure 7D. HDAC6 had 33 unique miRNAs, TUBA1A had 17, and CTNNB1 had 129 unique predicted miRNAs. The overlaps included 3 miRNAs shared between HDAC6 and CTNNB1, and 1 miRNA shared between TUBA1A and CTNNB1. No miRNAs were shared across all three genes.

### 2.8. Disease Association-Based Network of lncRNAs Linked to Liver Neoplasms

**Figure 8:**
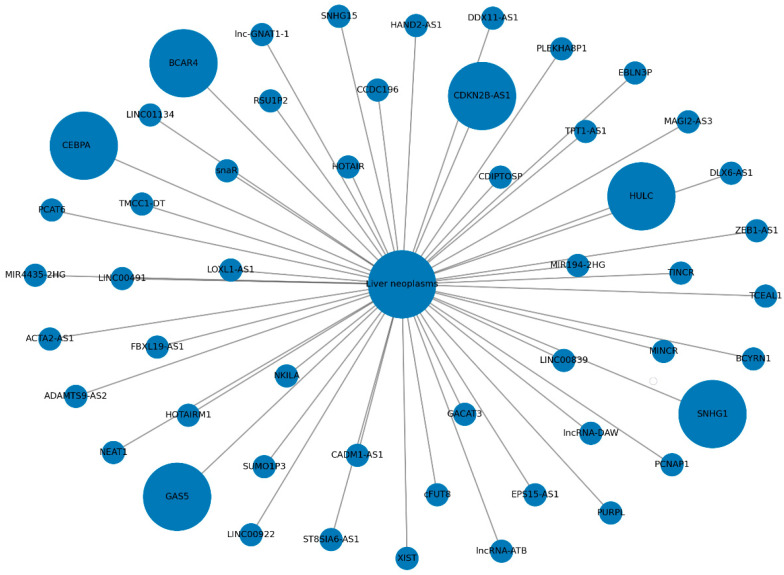
A network of liver neoplasm-associated lncRNAs is constructed based on disease association scores. The larger nodes have a score of 0.985791, while the smaller ones have a score of 0.731059.

The network illustrates lncRNAs associated with liver neoplasms, with “Liver neoplasms” placed as the central node. Each surrounding node represents an individual lncRNA identified from disease–lncRNA association data. Node sizes are scaled proportionally to the disease association score (larger nodes indicate stronger reported associations), while all nodes are connected directly to the central liver neoplasm node. The network layout was generated using a force-directed spring algorithm to visualize relative distances without implying quantitative interaction strength. The figure highlights highly ranked lncRNAs, including HULC, GAS5, CEBPA, CDKN2B-AS1, BCAR4, and SNHG1, which show the highest association scores with liver neoplasms (score = 0.985791), while all other lncRNAs cluster around an association score of 0.731059 (Figure 8). The lncRNAs with the highest scores (score = 0.985791) were selected for expression analysis.

### 2.9. miRNA–mRNA Interaction Network and miRNA Expression in LIHC

**Figure 9:**
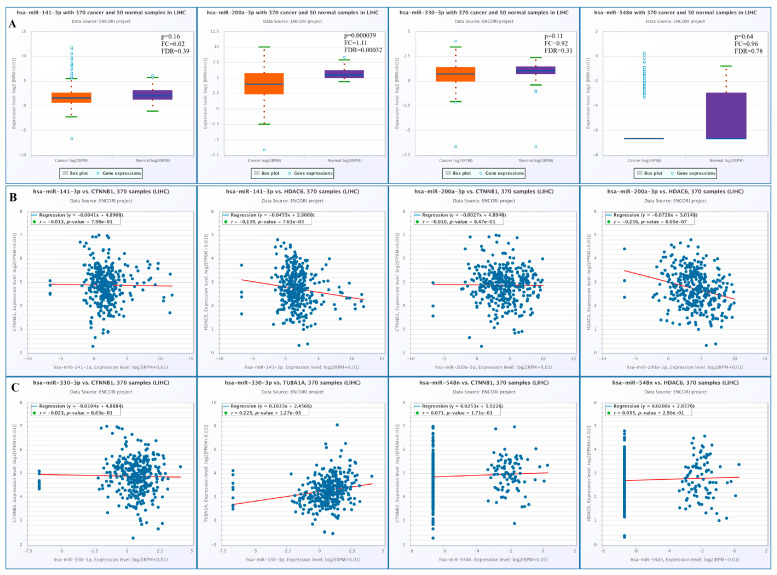
Differential expression and correlation analyses of selected miRNAs with HDAC6, TUBA1A, and CTNNB1 in LIHC (ENCORI). (**A**) Boxplots showing the expression levels of hsa-miR-141-3p, hsa-miR-200a-3p, hsa-miR-330-3p, and hsa-miR-548n in 370 LIHC tumor samples and 50 normal liver samples. Fold-change (FC), *p*-values, and FDR values are displayed for each comparison. (**B**) Correlation analyses between hsa-miR-141-3p or hsa-miR-200a-3p and the mRNA expression levels of CTNNB1, HDAC6, and TUBA1A (n = 370 LIHC samples). Each plot includes Pearson’s correlation coefficient (r), linear regression line, and corresponding *p*-value. (**C**) Correlation analyses of hsa-miR-330-3p and hsa-miR-548n with CTNNB1, HDAC6, and TUBA1A in LIHC samples (n = 370). Plots display regression fit, Pearson r, and statistical significance.

In LIHC samples, hsa-miR-200a-3p exhibited significantly elevated expression in tumor tissue compared to normal liver tissue (*p* = 0.000039, FDR = 0.000021). Conversely, hsa-miR-141-3p, hsa-miR-330-3p, and hsa-miR-548n did not exhibit significant differences between tumor and normal tissues. Correlation analysis revealed that the association between hsa-miR-141-3p and HDAC6 expression was not significant (r = −0.139, *p* = 7.61 × 10^−3^), whereas hsa-miR-200a-3p demonstrated a weak negative correlation with HDAC6 (r = −0.2256, *p* = 6.08 × 10^−7^). Additionally, hsa-miR-330-3p showed a mild positive correlation with TUBA1A (r = 0.2253, *p* = 1.27 × 10^−6^). No significant correlations were identified between hsa-miR-548n and HDAC6, TUBA1A, or CTNNB1. Across all analyses, the correlations between the examined miRNAs and CTNNB1 expression were not statistically significant (Figure 9).

### 2.10. LncRNA–mRNA Interaction Network and LncRNA Expression in LIHC

**Figure 10:**
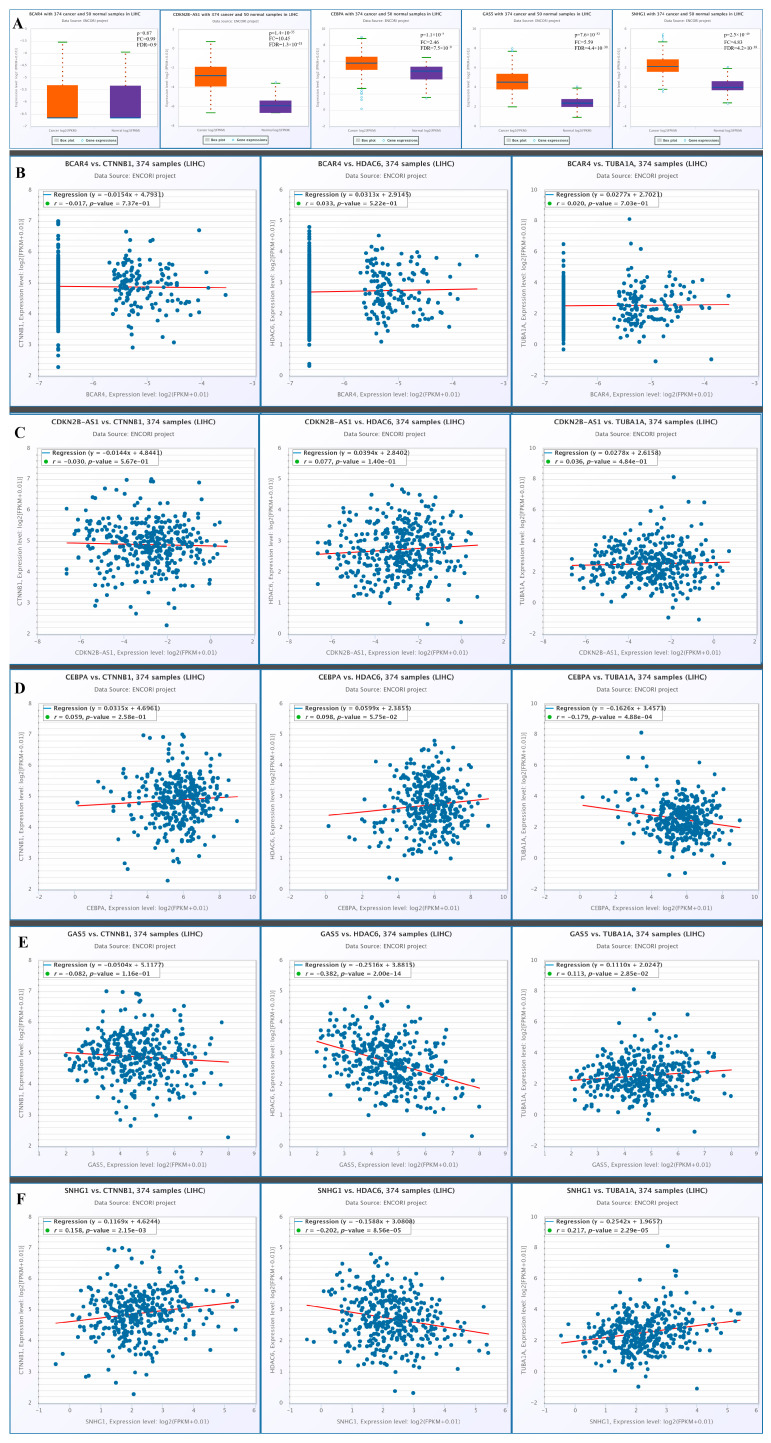
Differential expression and correlation analyses of BCAR4, CDKN2B-AS1, CEBPA, GAS5, and SNHG1 with HDAC6, TUBA1A, and CTNNB1 in LIHC (ENCORI). (**A**) Boxplots showing the expression levels of BCAR4, CDKN2B-AS1, CEBPA, GAS5, and SNHG1 in 374 LIHC tumor samples and 50 normal liver tissues. Fold-change (FC), *p*-values, and FDR values are presented for each comparison. (**B**) Correlation plots between BCAR4 expression and CTNNB1, HDAC6, and TUBA1A mRNA levels in LIHC (n = 374). Pearson’s correlation coefficient (r), regression line, and *p*-value are shown for each pair. (**C**) Correlation plots evaluating the association of CDKN2B-AS1 with CTNNB1, HDAC6, and TUBA1A expression in LIHC samples. Each panel displays the regression line, r value, and statistical significance. (**D**) Correlations between CEBPA expression and CTNNB1, HDAC6, and TUBA1A in LIHC (n = 374), presented with corresponding Pearson’s r values and *p*-values. (**E**) Correlation analyses of GAS5 with CTNNB1, HDAC6, and TUBA1A, including regression fit, r coefficient, and *p*-value. (**F**) Correlation plots for SNHG1 expression versus CTNNB1, HDAC6, and TUBA1A mRNA levels in LIHC. Scatter distributions, regression line, Pearson’s correlation coefficients, and *p*-values are shown for each comparison.

**Figure 11:**
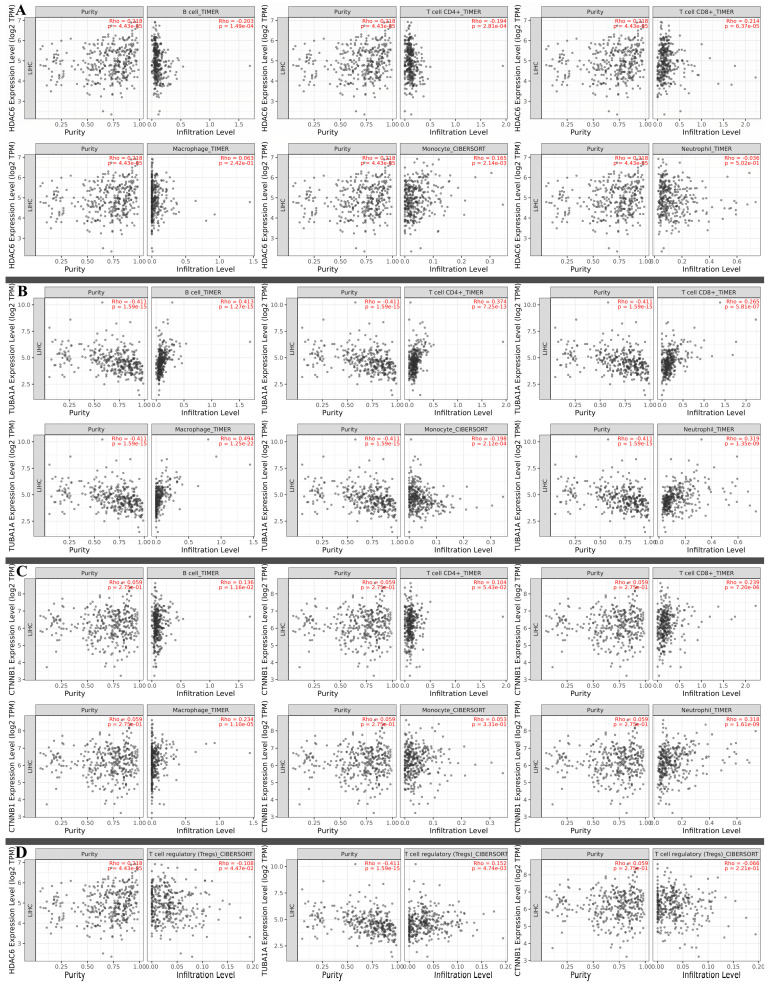
Immune infiltration correlations of HDAC6, TUBA1A, and CTNNB1 expression in LIHC (TIMER). (**A**) Correlation between HDAC6 expression and the infiltration levels of B cells, CD4^+^ T cells, CD8^+^ T cells, macrophages, monocytes, and neutrophils in LIHC. Scatterplots display Spearman’s correlation coefficient (rho) and associated *p*-values for each immune cell type, with tumor purity included for comparison. (**B**) Correlation between TUBA1A expression and immune cell infiltration levels in LIHC. Spearman’s rho and *p*-values are provided for all immune subsets’ infiltration estimates. (**C**) Correlation between CTNNB1 expression and the infiltration levels of the same immune cell populations in LIHC, presented with corresponding correlation coefficients and statistical significance. (**D**) Correlation of HDAC6, TUBA1A, and CTNNB1 genes with T cell regulatory (Treg) cells.

Expression levels of the long non-coding RNAs (lncRNAs) BCAR4, CDKN2B-AS1, CEBPA, GAS5, and SNHG1 were assessed in LIHC tumor tissues compared to normal liver samples. Among these lncRNAs, CDKN2B-AS1, CEBPA, GAS5, and SNHG1 exhibited significantly elevated expression in tumor samples, whereas BCAR4 did not show a significant difference between tumor and normal tissues (Figure 10A). Correlation analyses conducted on 374 LIHC samples indicated that BCAR4 and CDKN2B-AS1 had no significant association with the expression of CTNNB1, HDAC6, or TUBA1A (Figure 10B and Figure 10C). CEBPA was positively correlated solely with TUBA1A (Figure 11D). GAS5 exhibited a weak negative correlation with HDAC6 and a positive correlation with TUBA1A, with no correlation observed between CTNNB1 and GAS5 (Figure 10E). SNHG1 demonstrated weak negative correlations with HDAC6, while showing positive correlations with both CTNNB1 and TUBA1A (Figure 10F).

### 2.11. Immune Infiltration Patterns Associated with HDAC6, TUBA1A, and CTNNB1 Expression in LIHC

HDAC6 expression showed significant positive correlations with CD8^+^ T cells (rho = 0.214, *p* = 6.37 × 10^−5^) and monocytes (rho = 0.165, *p* = 2.14 × 10^−3^), while a significant negative correlation was observed with B cell (rho = −0.203, *p* = 1.49 × 10^−4^) and CD4^+^ T cells (rho = −0.194, *p* = 2.81 × 10^−4^). No significant association was found with macrophages (rho = 0.063, *p* = 2.42 × 10^−1^) and neutrophils (rho = −0.036, *p* = 5.02 × 10^−1^) (Figure 11A). TUBA1A expression was significantly positively correlated with CD4^+^ T cells (rho = 0.374, *p* = 7.25 × 10^−13^), macrophages (rho = 0.494, *p* = 1.25 × 10^−22^), CD8^+^ T cells (rho = 0.265, *p* = 5.81 × 10^−7^), neutrophils (rho = 0.319, *p* = 1.35 × 10^−9^) and B cells (rho = 0.413, *p* = 1.27 × 10^−15^) while TUBA1A showed a significant negative correlation with monocytes (rho = −0.198, *p* = 2.12 × 10^−4^) (Figure 11B). CTNNB1 expression demonstrated significant positive correlations with CD8^+^ T cells (rho = 0.239, *p* = 7.20 × 10^−6^), macrophages (rho = 0.234, *p* = 1.10 × 10^−5^) and neutrophils (rho = 0.318, *p* = 1.61 × 10^−9^). No significant correlation was found with CD4^+^ T cells (rho = 0.104, *p* = 5.43 × 10^−2^) and monocytes (rho = 0.053, *p* = 3.31 × 10^−1^) (Figure 11C). HDAC6 expression showed significant negative correlations with Tregs (rho = −0.108, *p* = 4.47 × 10^−2^), while TUBA1A showed a significant positive correlation with Tregs (rho = 0.152, *p* = 4.74 × 10^−3^). No significant correlation was found between CTNNB1 and Tregs (rho = −0.066, *p* = 2.21 × 10^−1^) (Figure 11D).

### 2.12. Differential Expression and Probe-Level Validation of HDAC6, TUBA1A, and CTNNB1

**Figure 12:**
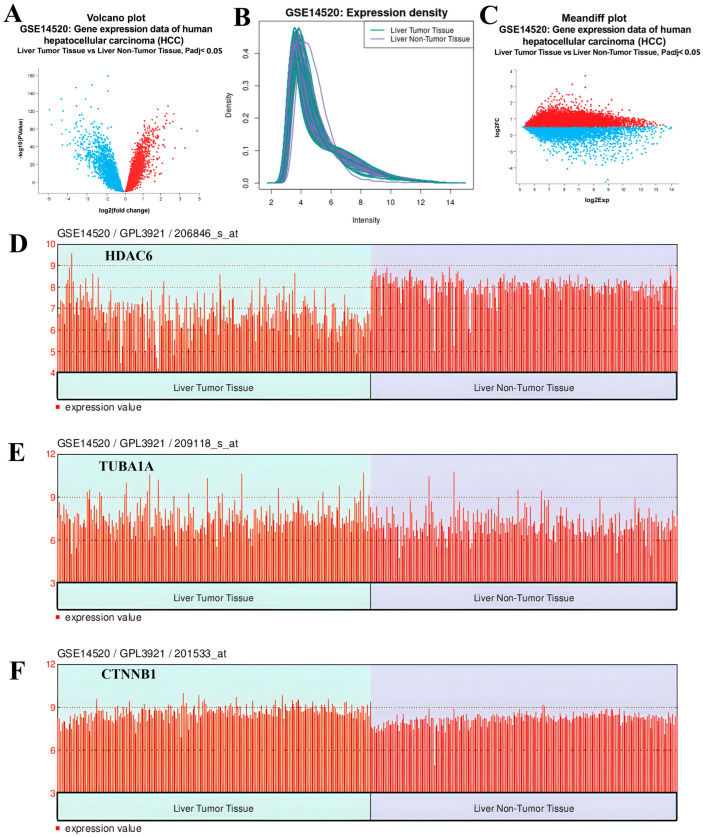
Differential expression analysis and probe-level validation of HDAC6, TUBA1A, and CTNNB1 in the GSE14520 HCC cohort. (**A**) Volcano plot of genome-wide differential expression analysis comparing liver tumor tissues to non-tumor liver tissues (GSE14520). Significantly upregulated genes (Padj < 0.05) are shown in red and downregulated genes in blue. (**B**) Density plot showing the distribution of expression intensities across liver tumor and non-tumor samples, demonstrating comparable global signal distribution following preprocessing and normalization. (**C**) Meandiff plot illustrates the relationship between log2 expression values and log2 fold change (log2FC), highlighting differentially expressed transcripts between tumor and non-tumor groups. (**D**) Probe-level expression profile of HDAC6. (**E**) Probe-level expression profile of TUBA1A. (**F**) Probe-level expression profile of CTNNB1.

Genome-wide differential expression profiling within the GSE14520 cohort demonstrated a distinct separation between tumor and non-tumor liver tissues, as evidenced by the volcano, density, and meandiff plots (Figure 12A–C). The inclusion of gene-specific effect sizes further elucidated the unique transcriptional patterns of HDAC6, TUBA1A, and CTNNB1 in HCC. HDAC6 was notably downregulated in tumor tissues (logFC = −1.23, adj *p* = 1.92 × 10^−58^). This significant reduction was also apparent at the probe level (Figure 12D), where HDAC6 expression was diminished in tumor samples compared to non-tumor liver tissues. Conversely, TUBA1A was significantly upregulated in tumor tissues (logFC = 0.48, adj *p* = 5.83 × 10^−8^), as shown in probe-level patterns (Figure 12E). Similarly, CTNNB1 was upregulated in HCC (logFC = 0.48, adj *p* = 2.65 × 10^−24^), with probe-level visualization presented in Figure 12F.

### 2.13. In Silico CRISPR-Cas9 Targetability Analysis of HDAC6, CTNNB1, and TUBA1A

**Figure 13:**
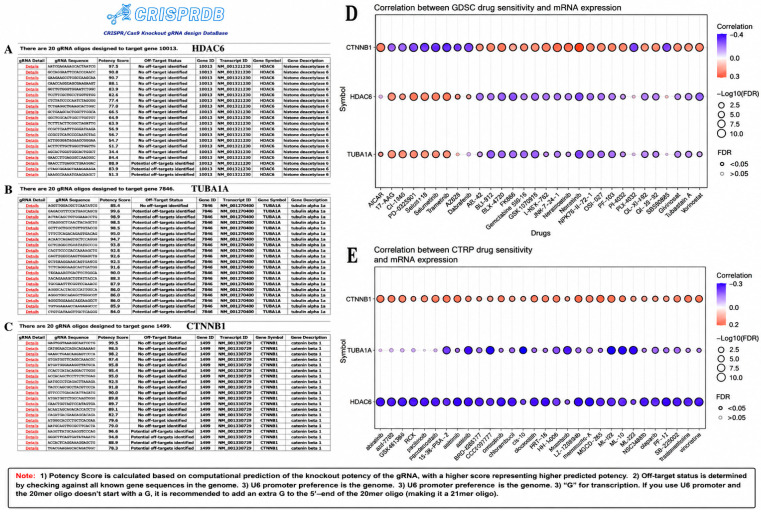
In silico CRISPR gRNA targetability profiles of HDAC6, CTNNB1, and TUBA1A. CRISPRdb-derived candidate guide RNAs are shown for HDAC6 (**A**), TUBA1A (**B**), and CTNNB1 (**C**), including gRNA sequences, predicted potency scores, and off-target status. CTNNB1 and HDAC6 exhibit multiple high-potency, low off-target guides, indicating favorable genome-editing feasibility. In contrast, TUBA1A guides, despite high predicted potency, display frequent off-target predictions, suggesting increased specificity constraints. These comparative profiles highlight differential CRISPR targetability within the HDAC6-centered regulatory axis in hepatocellular carcinoma. (**D**) Correlation analysis between GDSC (Genomics of Drug Sensitivity in Cancer) drug sensitivity and mRNA expression levels of CTNNB1, HDAC6, and TUBA1A. Bubble plots represent the correlation between gene expression and drug response across different anticancer compounds. The color gradient indicates the correlation coefficient (blue: negative correlation, red: positive correlation), while bubble size represents statistical significance expressed as −log10(FDR). Filled circles indicate statistically significant correlations (FDR ≤ 0.05). (**E**) Correlation analysis between CTRP (Cancer Therapeutics Response Portal) drug sensitivity and mRNA expression levels of CTNNB1, HDAC6, and TUBA1A. Similar to panel D, bubble color represents the correlation coefficient and bubble size reflects −log10(FDR) values. Filled circles indicate statistically significant associations (FDR ≤ 0.05), highlighting potential relationships between gene expression and drug response profiles across CTRP compounds. Positive correlation indicates that higher gene expression may lead to drug resistance. Negative correlation indicates that higher gene expression may cause drug sensitivity.

CRISPRdb-based in silico screening identified 20 candidate gRNA oligonucleotides targeting each of the HDAC6, CTNNB1, and TUBA1A loci, enabling comparative evaluation of predicted editing efficiency and off-target risk across the HDAC6-centered regulatory axis. For CTNNB1, the majority of candidate guides demonstrated very high predicted potency scores (up to 99.5) with no detectable off-target activity, whereas only a small subset showed potential off-target signals. These findings indicate a favorable specificity and efficiency profile for CRISPR-mediated modulation of CTNNB1. Similarly, HDAC6-targeting gRNAs exhibited high on-target potency (up to 97.5), and most candidates were predicted to have no off-target interactions, although a limited number displayed potential off-target sites. This pattern is consistent with the technical feasibility of precise genome targeting for HDAC6 using carefully selected guide sequences. In contrast, TUBA1A-directed guides showed consistently high potency scores (reaching 99.6) but were predominantly associated with predicted off-target activity, suggesting a comparatively higher specificity challenge for CRISPR-based targeting of TUBA1A relative to HDAC6 and CTNNB1 (Figure 13). In the correlation analysis conducted using the GDSC database, significant relationships were observed between the mRNA expression levels of the CTNNB1, HDAC6, and TUBA1A genes and sensitivity to various anticancer drugs. The analysis results revealed that, in particular, the expression levels of HDAC6 and TUBA1A showed negative correlations with many drugs, whereas the expression of CTNNB1 displayed positive correlations with certain drugs (Figure 13D. In a similar analysis conducted using the CTRP database, significant correlations were also identified between gene expression and drug sensitivity. It was found that the expression of the HDAC6 and TUBA1A genes showed a strong negative correlation with many drugs, whereas CTNNB1 expression mostly exhibited a tendency toward positive correlation (Figure 13E).

### 2.14. Extracellular Vesicle-Derived Expression Patterns of HDAC6 Axis-Related Molecules Across Disease Groups

**Figure 14:**
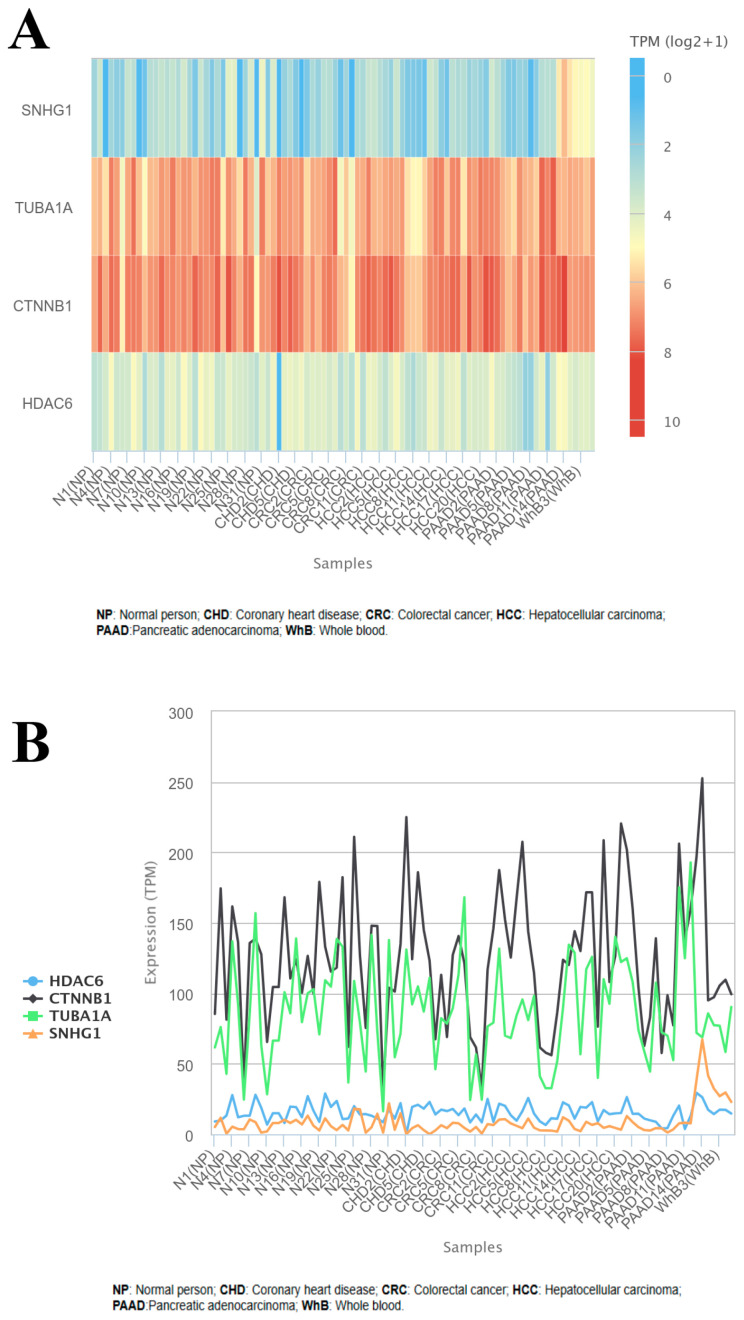
Extracellular vesicle-derived expression profiles of SNHG1, TUBA1A, CTNNB1, and HDAC6 across clinical sample groups. (**A**) Heatmap illustrating normalized transcript abundance (TPM, log2+1) of SNHG1, TUBA1A, CTNNB1, and HDAC6 in extracellular vesicle (EV) samples derived from normal individuals (NP), coronary heart disease (CHD), colorectal cancer (CRC), hepatocellular carcinoma (HCC), pancreatic adenocarcinoma (PAAD), and whole blood (WhB). Distinct expression gradients are observed, with CTNNB1 and TUBA1A displaying comparatively higher EV-associated expression, whereas HDAC6 and SNHG1 remain relatively low across most groups. (**B**) Line plot depicting quantitative TPM variation in the same molecules across individual EV samples.

The heatmap depicted in Panel A illustrates the normalized expression distribution of the SNHG1, TUBA1A, CTNNB1, and HDAC6 genes in extracellular vesicle (EV) samples derived from various clinical groups. A visual examination indicates that the expression levels of CTNNB1 and TUBA1A are significantly elevated across most sample groups, whereas the levels of HDAC6 and SNHG1 remain comparatively low. The line graph in Panel B corroborates the quantitative TPM variation in these molecules across the samples. This analysis is characterized by CTNNB1 demonstrating the highest and most variable expression, TUBA1A exhibiting a moderate-to-high level with a fluctuating pattern, HDAC6 maintaining a stable trajectory within the low-to-moderate range, and SNHG1 generally displaying low expression, with increases noted in certain disease samples (Figure 14).

## 3. Discussion

The present study was designed to address whether a coordinated HDAC6–TUBA1A–CTNNB1 regulatory axis exists in HCC and whether this axis may influence tumor progression and therapeutic vulnerability. This study utilizes an integrative bioinformatic approach focused on HDAC6, α-Tubulin, and β-Catenin. It combines differential gene expression analysis, epigenetic profiling, pan-cancer comparisons, protein–protein interaction networks, promoter methylation status, LncRNA regulatory mapping, miRNA regulatory mapping, and tumor immune infiltration analysis.Through this comprehensive analysis, a coherent molecular framework is identified, centered on HDAC6 and its relationship with TUBA1A (α-Tubulin) and CTNNB1(β-Catenin), for which it is responsible for deacetylation. These three genes collectively define a cytoskeletal–epigenetic–oncogenic axis with potential biological and translational relevance in HCC. Importantly, the proposed HDAC6–TUBA1A–CTNNB1 axis should not be interpreted as a completely new standalone pathway, but rather as an integrative regulatory framework that connects previously recognized biological processes—HDAC6-mediated cytoskeletal regulation, tubulin dynamics, and CTNNB1/Wnt-related signaling—within the specific molecular context of HCC.

Our integrative analysis revealed a distinct and complementary expression pattern: HDAC6 was significantly downregulated, whereas TUBA1A and CTNNB1 were consistently upregulated in HCC tumor tissues at both gene and probe levels. These findings were corroborated through multiple analytical layers, including GSE14520 expression density plots, volcano and mean-difference analyses, and independent probe-level confirmations. This comprehensive approach suggests that the observed transcriptional shifts are not artifacts of microarray probe variability but represent genuine biological alterations. The contrasting expression profiles observed in the present study, namely, reduced HDAC6 expression together with increased TUBA1A and CTNNB1 expression, should not necessarily be interpreted as biologically contradictory. Rather than representing a linear signaling cascade requiring concordant regulation, the proposed HDAC6–TUBA1A–CTNNB1 axis may reflect a context-dependent and compensatory molecular network. Reduced HDAC6 expression may alter cytoskeletal regulatory balance and microtubule-associated dynamics, potentially favoring adaptive remodeling processes. In this context, increased TUBA1A expression may represent compensatory cytoskeletal reorganization associated with tumor plasticity, migration, or altered cellular structural requirements. Simultaneously, CTNNB1 upregulation may reflect activation of oncogenic Wnt/β-catenin signaling programs that cooperate with cytoskeletal remodeling to support HCC progression. Accordingly, these directionally distinct expression changes may converge functionally within a broader dysregulated molecular framework rather than represent mutually exclusive biological processes.

The differential expression patterns of HDAC6 and CTNNB1 were downregulated in metastatic tumors compared with tumor and normal tissues, while TUBA1A expression was upregulated in metastatic tumors compared with tumor and normal tissues. This may suggest that these genes may play distinct roles in molecular events during hepatocarcinogenesis and metastatic progression. Given that histone acetylation and deacetylation govern critical epigenetic programs, disruptions in these processes have been widely associated with malignant transformation, tumor evolution, and reduced responsiveness to chemotherapeutic agents [15]. The concurrent observation of reduced HDAC6 expression alongside elevated TUBA1A levels may suggest an increase in microtubule acetylation, potentially leading to a more stable cytoskeletal state that could facilitate invasion. Alterations in microtubule dynamics have been previously documented in various solid tumors, where they are associated with epithelial–mesenchymal transition and cell migration [8,12]. In this context, the expression pattern observed in our study may reflect potential biological processes associated with cytoskeletal reprogramming in HCC.

The elevation in CTNNB1 levels may indicate that the Wnt/β-catenin signaling pathway may be active within tumor tissue. This pathway is recognized for its association with mechanisms such as proliferation, metabolic reprogramming, and epithelial–mesenchymal transition during the process of hepatocarcinogenesis [9,26]. In our study, the identification of reduced HDAC6 expression may suggest a mechanism that indirectly enhances pro-tumoral signaling in HCC, not only through microtubule/tubulin acetylation but also via Tau-centered acetylation networks. Notably, the primary function of HDAC6 on Tau is to reverse Tau acetylation, particularly in the MTBR region, and it has been demonstrated that HDAC6 can effectively deacetylate disease-associated Tau acetylation such as ac-K280 and ac-K311 [27].

Furthermore, the presence of K311 within MTBR and its physical interaction with HDAC6 may imply that the absence of HDAC6 could facilitate the accumulation of the “acetylated/functional” forms of Tau. In this regard, diminished levels of HDAC6 may potentiate the effects of Tau related to acetylation and/or acetyltransferase activity. It has been documented that Tau directly acetylates β-catenin (the product of CTNNB1) at K49, thereby inhibiting the phosphorylation of β-catenin and its ubiquitin-dependent degradation. This process stabilizes β-catenin, promotes its nuclear translocation, and enhances its transcriptional activity. Notably, this K49-acetylation-dependent activation of β-catenin has been shown to increase the expression of “survival” genes such as bcl2 and survivin, thereby inhibiting apoptosis and promoting cellular survival [28]. Therefore, reduced HDAC6 expression in HCC may be associated with conditions favoring Wnt/β-catenin pathway activation, including the inhibition of apoptosis, through the modulation of Tau-regulated β-catenin acetylation and stabilization. This interpretation offers a biologically coherent framework that accounts for the observed pattern of decreased HDAC6, although this interpretation remains speculative and hypothesis-generating, and increased CTNNB1 in our study, not merely as an increase in β-catenin expression, but as a model of post-translational acetylation-based enhancement. However, to directly confirm this mechanism within the context of HCC, experimental validation is necessary, including assessments of Tau acetylation levels (e.g., ac-K280/ac-K311), β-catenin K49 acetylation, and target genes such as BCL2 and BIRC5/survivin. Furthermore, the relative decrease in CTNNB1 expression in metastatic tissues may suggest that tumor cells develop distinct signaling dependencies as the disease progresses. This observation aligns with current models proposing that Wnt signaling may be dynamically regulated in a stage-specific manner.

The observation that reduced expression of HDAC6 correlates with decreased survival rates implies that this gene may function in a tumor suppressor-like capacity within the context of HCC. Notably, it has been documented that HDAC6 can manifest both oncogenic and protective roles, contingent upon the specific context in various cancer types [17]. Our study further suggests that alterations in HDAC6 promoter methylation may suggest an epigenetic foundation for this suppression. This observation implies that the equilibrium of post-translational acetylation in HCC could be pivotal in tumor progression.

In the existing literature, HDAC6 is identified as the primary enzyme responsible for the deacetylation of alpha-tubulin (encoded by TUBA1A). In our study, the observed downregulation of HDAC6 and upregulation of TUBA1A may result in microtubule hyperacetylation in HCC cells, potentially inducing epithelial–mesenchymal transition (EMT) and cellular migration [15]. In contrast, the elevated expression levels of TUBA1A in both tumor and metastatic tissues may correspond with the cytoskeletal remodeling requirements associated with enhanced proliferation and cellular motility. Hypomethylation according to UALCAN may affect the upregulation of TUBA1A. Increased TUBA1A expression was linked to mTOR and p38 MAPK pathway activity, pathways that modulate tumor cell proliferation, growth, and survival [29]. Despite these results, the lack of a significant correlation between TUBA1A expression levels and OS, RFS, or PFS suggests that TUBA1A may be involved in tumor progression signaling pathways, but it does not independently predict long-term clinical outcomes.

CTNNB1, a central component of the Wnt/β-catenin signaling pathway [30,31,32,33], was significantly upregulated in primary tumor tissues but showed reduced expression in metastatic samples, which may have indicated a context-dependent modulation of Wnt signaling during disease progression. Although promoter hypomethylation was consistent with increased CTNNB1 expression in tumor tissues, no significant associations were observed between CTNNB1 expression levels and OS, RFS, or PFS, suggesting that CTNNB1 expression did not appear to function as an independent prognostic indicator in HCC. Given the regulatory role of HDAC6 in cytoplasmic deacetylation and signal transduction, its interaction with CTNNB1 represents an important molecular interface in hepatocellular carcinoma [34]. Protein interaction analysis suggested EP300 as a shared interactor of both HDAC6 and CTNNB1. HDAC6 is a cytoplasmic deacetylase that counterbalances acetyltransferases such as EP300 and thereby regulates acetylation-dependent signaling pathways and transcriptional programs in cancer cells [35]. Prior evidence indicates that increased EP300 activity can be associated with enhanced TP53 acetylation together with suppression of canonical Wnt/β-catenin and Hippo-related transcriptional regulators [36]; in this context, the concurrent downregulation of HDAC6 and CTNNB1 observed in metastatic hepatocellular carcinoma may reflect a stage-specific reorganization of EP300-associated regulatory networks, leading to reduced reliance on canonical β-catenin-driven programs during metastatic progression. Collectively, these findings may reflect a network-based model in which HDAC6, CTNNB1, and TUBA1A function within interconnected regulatory circuits rather than as isolated molecular entities. This integrated perspective highlights the importance of pathway-level interpretation for understanding molecular crosstalk and potential vulnerabilities in hepatocellular carcinoma.

Although HDAC6, TUBA1A, and CTNNB1 collectively participate in interconnected biological pathways relevant to HCC, our survival analyses suggest that their clinical prognostic relevance is not equivalent. Among these genes, HDAC6 demonstrated a more consistent association with survival outcomes, whereas TUBA1A and CTNNB1 showed limited or non-significant prognostic value. This discrepancy may reflect differences between mechanistic involvement in tumor biology and independent prognostic utility. HDAC6, a multifunctional deacetylase involved in cytoskeletal remodeling, protein homeostasis, stress responses, and oncogenic signaling, may exert broader regulatory effects that more directly influence tumor behavior and patient outcome. In contrast, although TUBA1A and CTNNB1 are biologically implicated in cytoskeletal dynamics and Wnt/β-catenin signaling, respectively, their altered expression alone may not sufficiently capture disease aggressiveness or survival heterogeneity in HCC. Particularly for CTNNB1, functional consequences may depend more strongly on mutational status, pathway activation context, or post-transcriptional regulation than transcript abundance itself. Therefore, while TUBA1A and CTNNB1 may contribute to HCC pathogenesis and network-level tumor biology, our findings do not support interpreting them as having prognostic relevance comparable to HDAC6.

Analysis of STRING-based interactions among HDAC6, TUBA1A, and CTNNB1 has elucidated their integration within dense, cancer-relevant networks that encompass cytoskeletal regulation, protein quality control, and transcriptional control. HDAC6 exhibits high-confidence associations with chaperones and protein quality control components (HSP90AA1, HSP90AB1, VCP, UBC, SQSTM1), as well as partners related to the cytoskeleton and transcription (cortactin, RUNX2, TARDBP). This aligns with its role at the intersection of microtubule dynamics, aggresomal–autophagy pathways, and stress adaptation in tumor cells [37,38]. TUBA1A is predominantly associated with various β-tubulin isoforms (TUBB, TUBB2A/B, TUBB3, TUBB4A/B, TUBB6), as well as with MAPT, DYNC1H1, and CLIP1, underscoring its significance in microtubule organization, intracellular transport, and mitotic spindle assembly. The dysregulation of tubulin isotypes and microtubule dynamics is widely acknowledged as a catalyst for chromosomal instability, uncontrolled proliferation, and metastatic dissemination in cancer cells, highlighting the critical role of microtubule architecture in tumor biology [39,40,41]. These interactions indicate that abnormal TUBA1A activity may contribute to malignant progression by altering the cytoskeletal structure and function in a manner that enhances proliferative and metastatic potential. Although the STRING-derived protein–protein interaction network identified multiple predicted and experimentally supported interactors, particular emphasis may be placed on proteins with potentially greater biological relevance to the proposed HDAC6–TUBA1A–CTNNB1 axis. Among these, EP300 (p300) may be of particular interest due to its reported role as a transcriptional coactivator and chromatin regulator involved in β-catenin-associated transcriptional programs and HCC progression [42], potentially providing an epigenetic context for CTNNB1-related oncogenic signaling. Likewise, APC and AXIN1, key components of the β-catenin destruction complex, may provide mechanistic context for CTNNB1-associated signaling dynamics through their established involvement in β-catenin phosphorylation, degradation, and pathway regulation; disruption of this regulatory system has been implicated in hepatocarcinogenesis [22]. Furthermore, the enrichment of tubulin-associated proteins may reinforce the cytoskeletal dimension of the proposed regulatory axis, particularly considering that HDAC6 has been reported to function as a microtubule-associated deacetylase involved in α-tubulin acetylation, microtubule stability, and cellular motility [43]. Collectively, these interactors may suggest a biologically plausible framework linking epigenetic regulation, cytoskeletal remodeling, and Wnt/β-catenin signaling in HCC; however, these observations should be interpreted cautiously and require further experimental validation to establish functional relevance.

The comprehensive profiling of miRNAs reveals a coordinated post-transcriptional regulatory axis that converges on HDAC6, TUBA1A, and CTNNB1 in LIHC. miR-141-3p and miR-200a-3p, which are well documented for their roles in inhibiting EMT and Wnt/β-catenin-mediated oncogenic signaling [44,45,46], demonstrated inverse relationships with both HDAC6 and CTNNB1. This observation suggests that these miRNAs may influence epigenetic remodeling and Wnt-related transcription within a unified regulatory framework. miR-330 exhibits context-dependent functions in HCC, acting as either a tumor suppressor or promoter contingent upon its downstream targets. Specifically, miR-330-3p can inhibit tumor growth by repressing the oncogene MAP2K1, while it also promotes malignancy through the downregulation of tumor suppressors BTG1 and OCT1. Similarly, miR-330-5p suppresses CHEK1 but facilitates tumor progression by repressing SPRY2 [47]. In our LIHC dataset, the observed positive correlation between miR-330-3p and TUBA1A may suggest the pro-tumorigenic nature of this pathway, consistent with miR-330-3p’s role in supporting cytoskeletal remodeling and proliferative capacity in HCC. Collectively, these findings imply that miR-330 may interact with the TUBA1A axis to modulate tumor progression in a context-dependent manner.

The expression patterns of BCAR4, CDKN2B-AS1, CEBPA, GAS5, and SNHG1 in LIHC, along with their correlations with HDAC6, TUBA1A, and CTNNB1, suggest that these lncRNAs intersect with epigenetic regulation, cytoskeletal organization, and Wnt-related transcriptional activity. Oncogenic lncRNAs such as BCAR4, CDKN2B-AS1, CEBPA, and SNHG1, known for promoting proliferation, migration, and invasion, demonstrated associations consistent with their potential to reinforce pro-tumorigenic pathways linked to all three genes [48,49,50,51]. In our study, SNHG1 showed elevated lncRNA levels. SNHG1 functions as a multifunctional oncogene affecting cell cycle, apoptosis, migration, metabolism, immune response, and chemotherapy sensitivity in HCC [52]. SNHG1 positively correlates with TUBA1A and CTNNB1, while negatively correlating with HDAC6. This may suggest SNHG1 increases alongside factors supporting TUBA1A (cytoskeletal organization) and CTNNB1 (Wnt/β-catenin), while opposing HDAC6 (deacetylation and stress response). Rather than viewing SNHG1’s effect in HCC as singular, it should be considered as an interplay between epigenetic regulation (HDAC6), cytoskeletal remodeling (TUBA1A), and Wnt-related transcription (CTNNB1). In contrast, the tumor-suppressive lncRNA GAS5 exhibited inverse correlations that align with its reported pro-apoptotic effects [53].

The immune infiltration profile associated with HDAC6 is characterized by an increased presence of CD8^+^ T-cells and monocytes, alongside a decreased association with B-cells, CD4^+^ T-cells, and Tregs. This suggests that HDAC6 expression may influence the balance between effector and suppressive immune populations within the tumor microenvironment. Consistent with previous findings that HDAC6 inhibition enhances CD8^+^ T-cell infiltration, reduces Treg function, and promotes M1 macrophage recruitment [54], our results support the possibility that HDAC6 may function as an immunoregulatory node with potential implications for tumor immune responsiveness. The significant correlation between HDAC6 and the immune microenvironment suggests that our findings extend beyond tumor cells, implicating an immune escape mechanism. The literature extensively documents the regulatory effects of HDAC6 on T-cell activation and cytokine release. In our study, the positive correlation between HDAC6 expression and OS further substantiates the hypothesis that this gene may contribute to tumor immunity.

Beyond descriptive immune infiltration patterns, the observed associations may also be interpreted within a broader immunobiological framework. HDAC6 has been implicated in multiple processes potentially relevant to immune regulation, including inflammatory signaling, immune cell activation, antigen presentation, cytokine-associated responses, and cytoskeleton-dependent cellular dynamics that may influence immune cell trafficking and tumor–immune interactions [54].

In addition, HDAC6-associated signaling may intersect with Wnt/β-catenin pathway activity in HCC, although the direction and functional consequences of this interaction may be context-dependent. Importantly, aberrant Wnt/β-catenin or CTNNB1 activation has been associated with immune-excluded tumor phenotypes, impaired antitumor immune surveillance, and resistance to immune checkpoint-based therapeutic strategies in hepatocellular carcinoma [55,56,57]. Accordingly, the immune-related associations observed in this study may reflect indirect or context-dependent regulatory effects operating through broader signaling networks rather than direct immune-specific mechanisms. Nevertheless, because these findings are derived from computational immune inference analyses, they should be interpreted cautiously and regarded as hypothesis-generating pending experimental confirmation in relevant biological systems.

Correlation-based immune infiltration analyses do not establish direct immune modulation or causal regulatory effects within the tumor microenvironment. Rather, they indicate potential associations that may reflect broader tumor biological or microenvironmental contexts. Future validation using higher-resolution approaches, including single-cell RNA sequencing and spatial transcriptomics, may help clarify cell type-specific expression profiles, spatial organization, and the mechanistic relevance of these candidate genes in immune regulation within hepatocellular carcinoma.

In accordance with previous findings that TUBA1A in gastric cancer is associated with immunosuppressive M2-macrophage infiltration and poor prognosis [29], our study found that TUBA1A shows a significant positive correlation with extensive immune cell infiltration (CD4^+^/CD8^+^ T cells, macrophages, neutrophils, B cells, and Tregs), suggesting that this gene may be a pro-tumoral regulator driving the immune microenvironment in HCC. While CTNNB1-mutant HCC is characterized by diminished chemokine expression and extensive immune exclusion [57], our findings indicate that elevated CTNNB1 expression in LIHC is positively correlated with CD8^+^ T cells, macrophages, and neutrophils. This suggests that CTNNB1 may have a more immune-permissive effect in the non-mutant context. This contrast may align with evidence of a mutation-dependent divergence in CTNNB1-driven immune regulation in HCC.

Importantly, the extracellular vesicle-associated detection of HDAC6, TUBA1A, CTNNB1, and SNHG1 supports a hypothesis of a biologically plausible framework linking the HDAC6-centered regulatory network to gene delivery-oriented therapeutic concepts in hepatocellular carcinoma. Extracellular vesicles have emerged as naturally derived nanocarriers capable of transporting nucleic acids and regulatory molecules with favorable biocompatibility and cellular uptake characteristics, positioning them as promising platforms for targeted molecular intervention [58]. Within this context, the differential EV enrichment observed across the HDAC6–TUBA1A–CTNNB1 axis may reflect tumor-associated vesicular signaling patterns and potentially provide insight into disease-related molecular communication in HCC. However, these findings should be interpreted cautiously, as the present analysis remains exploratory and does not establish a functional or therapeutically actionable EV-mediated mechanism. Although the observed patterns raise the possibility that EV-associated regulatory pathways may warrant further investigation, any consideration of EV-mediated delivery of gene-regulatory cargos, including small interfering RNAs or genome-editing constructs targeting HDAC6, CTNNB1, or TUBA1A, remains highly speculative and requires substantial experimental validation. Future studies will need to assess biological function, delivery feasibility, targeting specificity, safety, and therapeutic efficacy before such approaches can be considered translationally relevant in HCC. In addition, data obtained from the GDSC and CTRP databases suggest that expression levels of these genes may be associated with differential responses to certain therapeutic agents, with HDAC6 and TUBA1A potentially linked to drug resistance-related profiles and CTNNB1 to distinct sensitivity patterns. However, these associations should likewise be interpreted cautiously and require experimental confirmation.

To facilitate biological interpretation, the findings of this integrative analysis may be conceptually summarized as follows: reduced HDAC6 expression in HCC may contribute to dysregulated cytoskeletal dynamics through altered tubulin-associated processes, while concomitant changes in TUBA1A and CTNNB1 may be linked to aberrant Wnt/β-catenin signaling, cellular plasticity, and tumor progression. Rather than representing isolated molecular events, these alterations may constitute an interconnected regulatory network influencing tumor behavior, immune interactions, and therapeutic vulnerability. Nevertheless, these relationships should be considered hypothesis-generating and require experimental validation.

Importantly, HDAC6 should not be interpreted as a singular master regulator of cytoskeletal remodeling or Wnt/β-catenin signaling in HCC. Rather, our findings lend support to a model in which HDAC6 may function as one context-dependent regulatory component embedded within a broader and highly interconnected molecular network involving multiple cytoskeletal regulators, signaling mediators, epigenetic mechanisms, and pathway crosstalk.

From a translational perspective, the molecular relationships identified in this study may support future therapeutic prioritization strategies targeting HDAC6-associated cytoskeletal regulation and Wnt/β-catenin signaling in HCC. Selective HDAC6 inhibitors, including agents investigated in oncology and inflammatory disease settings, have demonstrated potential to modulate cytoskeletal dynamics, tumor cell motility, immune responses, and oncogenic signaling pathways. Likewise, therapeutic approaches targeting aberrant Wnt/β-catenin signaling—including pathway inhibitors, β-catenin-associated regulatory modulators, and combination strategies—have attracted growing interest in liver cancer research. However, the clinical translation of these approaches remains challenging due to pathway redundancy, tumor heterogeneity, compensatory signaling networks, context-dependent biological responses, and potential toxicity associated with systemic pathway modulation. In this context, integrative bioinformatic studies may help prioritize biologically relevant molecular dependencies, identify candidate biomarker-defined patient subsets, and generate mechanistically informed hypotheses for future functional studies and biomarker-guided clinical trials. Nevertheless, these translational implications should be interpreted cautiously pending experimental and clinical validation.

Collectively, these results may reflect that HDAC6 and CTNNB1 represent technically favorable CRISPR-targetable genes, whereas TUBA1A targeting may require enhanced guide optimization or alternative delivery/editing strategies. Although CRISPR-Cas9 targetability analysis suggested that HDAC6, TUBA1A, and CTNNB1 may represent technically targetable candidates, these findings should be interpreted cautiously within a translational framework. The in silico gRNA prediction performed in this study was intended to provide an initial assessment of gene targetability and feasibility rather than evidence of immediate therapeutic applicability in HCC. Translation into clinically relevant interventions would require extensive experimental validation, including optimization of guide RNA efficiency and specificity, evaluation of off-target effects, tumor-selective delivery strategies, and consideration of tumor heterogeneity and safety profiles. Therefore, the CRISPR-related findings should be regarded as preliminary, hypothesis-generating observations that support future functional and translational investigations. This in silico evidence lends support to the translational feasibility of gene-delivery-mediated therapeutic modulation within the HDAC6-centered molecular network in hepatocellular carcinoma.

### Strengths and Limitations

Nevertheless, several limitations should be acknowledged. First, this study is predominantly based on retrospective bioinformatic analyses of publicly available datasets, and therefore the observed associations should be interpreted cautiously, as retrospective analyses inherently limit the ability to infer causality or establish direct biological mechanisms. Although the use of multiple independent resources (including TCGA, GEO, and HPA) strengthens robustness through cross-validation, heterogeneity among databases—including differences in patient populations, sequencing platforms, sample processing, normalization procedures, and analytical pipelines—may influence result comparability and interpretation. Second, although transcriptomic, methylation, protein-expression, survival, and immune infiltration analyses were integrated, these data were not derived from fully matched multi-omics samples within the same patient cohort. Accordingly, inferred relationships between promoter methylation, gene expression, immune contexture, and protein abundance should be interpreted as complementary rather than direct sample-level biological confirmation. Third, while significant associations were identified between gene expression and immune infiltration patterns, correlation-based analyses do not establish direct immune modulation or mechanistic crosstalk within the tumor microenvironment. Future studies using single-cell RNA sequencing, spatial transcriptomics, or functional immune assays may provide greater mechanistic resolution. Finally, the absence of experimental validation remains an important limitation. Functional confirmation through in vitro and in vivo approaches, including gene knockdown/overexpression experiments, CRISPR-based perturbation studies, extracellular vesicle characterization, and mechanistic assays, will be necessary to validate the biological and translational relevance of the identified regulatory axis. Despite these limitations, this study provides a comprehensive hypothesis-generating framework and identifies biologically plausible candidate pathways and biomarkers relevant to HCC progression.

## 4. Materials and Methods

### 4.1. GEPIA3 Platform Description and Analytical Framework

Gene Expression Profiling Interactive Analysis 3 (GEPIA3) (https://gepia3.bioinfoliu.com/, accessed on 17 November 2025) is a publicly available web-based platform designed for large-scale, pan-cancer analyses of gene expression, survival outcomes, drug-sensitivity associations and interaction networks, built upon uniformly processed RNA-seq datasets derived from The Cancer Genome Atlas (TCGA) and other major repositories [59]. In this study, GEPIA3 was used to compare mRNA expression levels of HDAC6, TUBA1A and CTNN genes between hepatocellular carcinoma and normal liver tissues. Gene expression values were retrieved as normalized transcript abundance estimates reported in log_2_(TPM+1) units following GEPIA3’s standardized normalization pipeline for RNA-seq harmonization. Differential expression analyses between tumor and normal tissues were performed through the platform-integrated expression comparison modules using GEPIA3 default analytical parameters. Statistical comparisons and visualization settings were retained according to the platform’s built-in implementation to ensure methodological consistency and facilitate independent reproducibility using identical public resources. When applicable, statistical significance was interpreted according to the significance metrics and thresholds reported directly by the platform. To complement transcriptomic findings at the protein level, expression analyses were further conducted using the Human Protein Atlas (HPA; https://www.proteinatlas.org; accessed on 17 November 2025), including the Pathology Atlas and Tissue Atlas modules. The same HGNC-approved gene symbols (HDAC6, TUBA1A, and CTNNB1) were queried to investigate protein expression profiles in normal liver and hepatocellular carcinoma tissues. Immunohistochemistry (IHC)-based staining images and annotations were evaluated for staining intensity, localization, and distribution patterns according to HPA standardized scoring procedures. Protein expression patterns were interpreted descriptively using platform-provided staining classifications (e.g., negative, low, medium, or high staining intensity) without additional independent image quantification. RNA expression data available through HPA were reported as transcripts per million (TPM) following the platform’s internal normalization pipeline. Because HPA functions primarily as a curated descriptive resource, no additional independent statistical modeling was performed beyond the platform-reported outputs. All analyses were performed according to publicly available methodological recommendations to maximize reproducibility and inter-study comparability [60].

### 4.2. Normal and Tumor Comparisons via TNMplot Database

Transcriptomic expression analyses were performed using the TNMplot platform (TNMplot.com; http://tnmplot.com; accessed on 17 November 2025), a publicly accessible web-based resource integrating harmonized transcriptomic datasets from The Cancer Genome Atlas (TCGA), the Genotype-Tissue Expression (GTEx) project, and Gene Expression Omnibus (GEO) repositories to enable comparative analyses among normal, tumor, and metastatic tissues [61]. Official HGNC gene symbols (HDAC6, TUBA1A, and CTNNB1) were used as input identifiers for all analyses. Unless otherwise stated, analyses were conducted using the platform’s default analytical settings to ensure reproducibility and consistency with publicly available workflows. The liver hepatocellular carcinoma (LIHC) dataset served as the primary disease context for focused analyses, while pan-cancer screening across all available malignancies was additionally performed to assess broader expression trends.

For each queried gene, expression distributions were examined across available normal, primary tumor, and metastatic tissue categories. Both paired and unpaired tumor–normal comparisons available within the TNMplot interface were explored where applicable, and metastatic tissues were included when available in the corresponding dataset. Gene expression values were analyzed according to TNMplot’s harmonized transcriptomic processing pipeline, which integrates platform-specific normalization procedures implemented by the database developers to facilitate cross-study comparability.

Differences in gene expression among normal, tumor, and metastatic groups were statistically evaluated using the Kruskal–Wallis test, a non-parametric method appropriate for comparisons among more than two independent groups without assumptions of normal distribution. When statistically significant overall differences were observed, pairwise comparisons were conducted using Dunn’s post hoc test with built-in correction for multiple testing as implemented by the TNMplot platform. Statistical significance thresholds were interpreted according to the platform-generated outputs, and *p* < 0.05 was considered statistically meaningful unless otherwise specified. Data visualizations and exported outputs were obtained directly from the platform to preserve methodological consistency and facilitate reproducibility.

### 4.3. TIMER 2.0-Based Immune Infiltration Analysis

Tumor immune infiltration analyses were conducted using the Tumor Immune Estimation Resource 2.0 (TIMER 2.0; http://timer.cistrome.org/; accessed on 17 November 2025), a publicly available web-based platform designed to estimate immune cell infiltration patterns from TCGA-derived transcriptomic datasets using multiple computational deconvolution algorithms. Official HGNC gene symbols (HDAC6, TUBA1A, and CTNNB1) were used as input identifiers, and analyses were performed within the liver hepatocellular carcinoma (LIHC) cohort. Unless otherwise specified, TIMER 2.0 default analytical parameters and platform-defined settings were retained to maximize methodological transparency and reproducibility.

Immune infiltration estimates were evaluated using the integrated deconvolution algorithms available in TIMER 2.0, including TIMER, EPIC, MCP-counter, CIBERSORT, CIBERSORT-ABS, quanTIseq, xCell, and TIDE, thereby enabling cross-method assessment of immune contexture. Correlations between gene expression levels and inferred abundance of major immune cell populations—including B cells, CD4^+^ T lymphocytes, CD8^+^ T lymphocytes, macrophages, neutrophils, and dendritic cells—were investigated using the platform’s *Gene module* and immune estimation framework.

Associations between gene expression and immune infiltration were statistically evaluated using partial Spearman correlation analysis with adjustment for tumor purity, as implemented within the TIMER 2.0 analytical pipeline. Correlation coefficients (ρ) and corresponding *p*-values were obtained directly from the platform-generated outputs. Statistical significance was interpreted according to TIMER 2.0 integrated significance testing and default reporting framework, with two-sided *p* < 0.05 considered statistically meaningful unless otherwise specified [62]. To minimize analytical variability and improve reproducibility, identical cohort selection, gene identifiers, and default platform settings were systematically maintained across all analyses. Exported correlation matrices and immune infiltration plots were directly retrieved from TIMER 2.0 without external reprocessing or modification. Because immune infiltration values are computationally inferred through deconvolution models rather than experimentally quantified, immune-related findings were interpreted as biologically plausible and hypothesis-generating associations rather than direct evidence of causal immune modulation.

### 4.4. UALCAN Methylation Analysis

Promoter methylation patterns of HDAC6, TUBA1A, and CTNN (CTNNB1) genes in HCC were investigated using the UALCAN web resource (http://ualcan.path.uab.edu/; accessed on 17 November 2025), which provides TCGA-derived epigenomic profiles for liver hepatocellular carcinoma (LIHC). Promoter methylation status was assessed through the “TCGA Methylation” module of UALCAN using data generated from the Illumina HumanMethylation450 BeadChip (450K) platform, which quantifies CpG methylation levels across the genome. For each queried gene, methylation levels were retrieved as β-values, representing the ratio of methylated probe signal intensity to the combined methylated and unmethylated signal intensity, ranging from 0 (unmethylated) to 1 (fully methylated). These β-values were used to compare promoter methylation distributions between primary tumor tissues and normal liver controls. Statistical comparisons between tumor and normal tissue groups were performed using the two-tailed Student’s *t*-test integrated within the UALCAN analytical framework. Corresponding *p*-values were directly obtained from platform-generated outputs, and two-sided *p* < 0.05 was considered statistically significant unless otherwise specified. Default cohort filtering, normalization procedures, and visualization settings provided by UALCAN were preserved to ensure consistency across analyses and facilitate independent reproducibility using identical public resources. Default filtering and normalization parameters recommended by UALCAN for cancer-type-specific methylation profiling were adopted to ensure reproducibility [63]. To improve methodological transparency, all analyses were conducted using explicitly reported cohort selection (TCGA-LIHC), standardized gene identifiers, platform-retained default settings, and reproducible methylation metrics. Exported methylation boxplots and associated statistical outputs were retrieved directly from UALCAN without external reprocessing or modification. Given the observational and computational nature of TCGA-derived methylation analyses, the identified epigenetic associations were interpreted as biologically relevant but hypothesis-generating evidence requiring future mechanistic validation. Promoter methylation and gene expression analyses were performed independently using complementary TCGA-based bioinformatic resources. Methylation findings (including TP53-stratified comparisons) were interpreted alongside transcriptional expression trends to explore potential epigenetic associations; however, direct sample-level methylation–expression correlation analyses were not performed. Accordingly, inferred relationships between promoter methylation and transcript abundance were interpreted cautiously.

### 4.5. Kaplan–Meier Plotter Workflow for Gene-Expression–Survival Analyses

Survival outcomes were assessed using the Kaplan–Meier Plotter platform (KMplot; https://kmplot.com/analysis accessed on 17 November 2025). Only cases with complete and validated survival information were included in the analysis. Overall survival (OS) served as the principal clinical endpoint, while progression-free or disease-free survival (PFS/DFS) was examined when corresponding datasets were available. Overall survival (OS) was defined as the principal survival endpoint, while progression-free survival (PFS) and/or disease-free survival (DFS) analyses were additionally evaluated when corresponding datasets and annotations were available within the platform. Gene-expression-based stratification of patients into high- and low-expression groups was performed according to KMplot’s internally implemented cut-off selection procedures, using platform-retained default settings unless otherwise specified. For microarray-derived datasets in which multiple probes corresponded to a single gene, the JetSet best probe selection algorithm was applied to select the most reliable probe representation for downstream survival analyses. Kaplan–Meier survival curves were generated using the integrated KMplot analytical pipeline, and statistical differences between survival distributions were evaluated using the two-sided log-rank test. Effect size estimation was performed through univariate Cox proportional hazards regression models, from which hazard ratios (HRs) and corresponding 95% confidence intervals (95% CIs) were obtained. Statistical significance values (*p*-values), HRs, and confidence intervals were retrieved directly from the platform outputs. Unless otherwise specified, two-sided *p* < 0.05 was considered statistically significant. To improve transparency and reproducibility, cohort selection, gene identifiers, survival endpoints, and platform-retained analytical settings were explicitly standardized across all analyses. Survival plots and associated statistical outputs were exported directly from KMplot without additional computational manipulation or independent model fitting. Given the retrospective and observational nature of transcriptome-associated survival analyses, identified prognostic associations were interpreted as biologically informative and hypothesis-generating rather than definitive evidence of causal clinical impact [64,65,66,67].

### 4.6. STRING Database-Based Analysis of Proteins Interacting with HDAC6, TUBA1A, and CTNN (CTNNB1)

Protein–protein interaction (PPI) networks for HDAC6, TUBA1A, and CTNN (CTNNB1) were generated using the STRING database (version 12.0; https://string-db.org; accessed on 17 November 2025). Interactors were retrieved for Homo sapiens, and only high-confidence interactions (minimum interaction score: 0.700) were included. First-shell interactors were selected, and edges supported by experimental evidence or curated databases were prioritized. GO and KEGG pathway enrichment analyses were performed using STRING’s integrated functional enrichment tool, and significance was evaluated using FDR-adjusted *p*-values < 0.05. Networks were exported in high-resolution format using STRING’s publication-ready output settings [68,69]. PPI networks were generated using the “full STRING network” configuration, enabling visualization of both direct and indirect protein associations. To improve biological specificity and reduce low-confidence interactions, only high-confidence interactions meeting a minimum required interaction score of 0.700 were retained. Active interaction sources were restricted to experimental evidence, curated databases, and co-expression data, while text mining-derived interactions were excluded to minimize literature-driven bias and improve network robustness. First-shell interacting proteins were included in the analysis, with the maximum number of interactors set to 50 for each queried gene to ensure network interpretability while preserving biologically relevant interaction complexity. Disconnected nodes were hidden from the final visualization. To maximize transparency and reproducibility, all analyses were performed using explicitly reported platform version, organism selection, gene identifiers, interaction evidence filters, confidence thresholds, interactor limits, and enrichment settings. Network outputs and enrichment tables were exported directly from STRING using publication-quality visualization settings without additional network manipulation or external computational reconstruction. Because interactome topology may vary substantially depending on confidence thresholds, evidence sources, and filtering criteria, the identified interaction networks were interpreted as context-dependent and hypothesis-generating molecular frameworks rather than definitive mechanistic models, thereby minimizing overinterpretation of network-derived biological conclusions.

### 4.7. Prediction of HDAC6, TUBA1A, and CTNN (CTNNB1) miRNA Interactions Using TargetScan 8.0 and miRDB

Predicted miRNA–mRNA interactions were explored using the TargetScanHuman database (Release 8.0; https://www.targetscan.org; accessed on 17 November 2025). This version of TargetScan identifies potential binding sites in mammalian transcripts by detecting canonical 8mer, 7mer and 6mer seed matches and prioritizes candidate miRNAs using an updated context++ scoring approach. Release 8.0 incorporates an enhanced biochemical model that refines target ranking by integrating seed pairing, site accessibility, 3′-UTR features and evolutionary conservation. Family-level prediction files were downloaded, and for each gene of interest, the cumulative weighted context++ score (CWCS) and, when available, the probability of conserved targeting (PCT) were extracted. Since lower (more negative) CWCS values indicate greater predicted inhibitory activity, these metrics were used as the basis for interpreting miRNA regulatory strength [70].

To complement the TargetScan analysis, miRNA target predictions were also retrieved from miRDB (http://mirdb.org; accessed on 17 November 2025), a machine learning-based resource trained on high-throughput CLIP-Seq experimental datasets. miRDB assigns each predicted interaction a numerical “target score” (ranging from 50 to 100), which reflects the confidence of miRNA binding based on its MirTarget computational model. For each candidate gene, miRNAs with a target score ≥ 80 were considered high-confidence regulators. All predictions were downloaded using default parameters, and overlapping miRNAs identified by both TargetScan and miRDB were prioritized for downstream analyses [71]. To improve robustness and reduce false-positive predictions, overlapping miRNAs identified by both TargetScan and miRDB were prioritized for subsequent analyses. All prediction tables were exported directly from the respective platforms without additional computational modeling or external re-ranking procedures. Because computational miRNA prediction algorithms rely on sequence complementarity, conservation features, and probabilistic scoring frameworks rather than direct biological validation, identified regulatory interactions were interpreted as biologically plausible and hypothesis-generating candidates requiring future experimental confirmation.

### 4.8. LncRNAs Associated with LIHC Identified Through the LncRNADisease Database

The LncRNADisease database (http://www.cuilab.cn/lncrnadisease, accessed on 17 November 2025) was employed to retrieve long non-coding RNAs associated with LIHC. This resource not only compiles experimentally validated links between lncRNAs and human diseases but also offers computational tools for predicting potential lncRNA–disease relationships. Moreover, the platform integrates multilayer interaction data for each lncRNA, including its connections with DNA, RNA, proteins, and miRNAs [72]. The disease term “liver hepatocellular carcinoma (LIHC)” and related hepatocellular carcinoma-associated keywords were queried within the database to retrieve lncRNAs reported to be associated with HCC pathogenesis. Candidate lncRNAs were prioritized according to database-reported evidence, including experimentally supported disease associations and platform-integrated computational annotations when available. For each candidate lncRNA, associated molecular interaction information—including links to miRNAs, proteins, DNA elements, and regulatory pathways—was examined to facilitate biological contextualization within the proposed HDAC6-centered regulatory framework. Because LncRNADisease primarily functions as a curated integrative resource rather than a statistical inference platform, no additional independent statistical modeling or quantitative filtering procedures were applied beyond the platform-provided evidence framework. Data extraction, prioritization, and interpretation were performed according to publicly accessible platform annotations and database outputs. To improve methodological transparency and reproducibility, all analyses were conducted using explicitly reported disease terms, standardized molecular identifiers, database-retained default settings, and platform-generated outputs without external computational manipulation.

Given the observational and annotation-based structure of LncRNADisease, identified lncRNA associations were interpreted as biologically relevant and hypothesis-generating evidence intended to support regulatory network exploration rather than definitive mechanistic validation of lncRNA involvement in hepatocellular carcinoma.

### 4.9. ENCORI-Based Identification of CLIP-Validated miRNA–mRNA Interactions

miRNA–mRNA regulatory interactions for HDAC6, TUBA1A and CTNN (CTNNB1) were investigated using the ENCORI (also known as starBase) database (https://starbase.sysu.edu.cn; accessed on 17 November 2025). ENCORI integrates large-scale CLIP-seq datasets (including AGO-CLIP, PAR-CLIP, HITS-CLIP and iCLIP), RNA–RNA interaction data, degradome sequencing results and multiple public transcriptomic resources, enabling experimentally supported identification of miRNA binding sites. For each target gene, miRNA interactors were retrieved through the “miRNA–mRNA” module, which reports only those binding events with direct CLIP-seq evidence. Interactions were filtered using the platform’s standard settings, requiring the presence of at least one supporting CLIP experiment and ENCORI’s built-in significance criteria [73]. miRNAs consistently detected across multiple CLIP datasets were considered high-confidence regulators. All data tables were exported in text format and subsequently cross-validated against computational predictions from TargetScan and miRDB. To reduce false-positive predictions and improve interaction confidence, only miRNA–mRNA interactions supported by at least one CLIP-supported experimental dataset were retained, in accordance with the platform’s built-in filtering structure. Platform-provided significance and evidence criteria were preserved using default settings unless otherwise stated. miRNAs reproducibly identified across multiple CLIP-supported datasets or supported by convergent evidence were prioritized as higher-confidence candidate regulators for downstream interpretation. All interaction tables and associated evidence metrics were exported directly from ENCORI without additional computational re-ranking or external statistical modeling. To improve robustness, experimentally supported interactions retrieved from ENCORI were subsequently cross-validated against computational predictions generated using TargetScanHuman Release 8.0 and miRDB, and overlapping miRNA candidates were prioritized for integrative downstream analyses. Because ENCORI-derived interactions reflect experimentally supported molecular associations inferred from transcriptome-wide binding evidence rather than gene-specific functional perturbation studies, identified miRNA–mRNA interactions were interpreted as biologically plausible and hypothesis-generating regulatory candidates, requiring future experimental validation to confirm context-specific functional relevance in hepatocellular carcinoma.

### 4.10. Gene Expression Data Acquisition from GEO

Gene expression datasets were sourced from the Gene Expression Omnibus (GEO) repository (https://www.ncbi.nlm.nih.gov/geo/; accessed on 17 November 2025) [74]. The data were derived from the publicly accessible GSE14520 cohort, which includes gene-expression microarray data generated on the GPL3921 Affymetrix Human Genome U133A platform [75,76,77,78,79,80,81,82,83,84,85,86]. Raw and platform-specific processed files (MINiML/SOFT formats) were retrieved from GEO and standardized using the appropriate annotation packages for each microarray platform. Differential expression analysis was performed using the GEO2R interface, which employs the limma (Linear Models for Microarray Data) framework implemented in R. To minimize false-positive findings due to multiple comparisons, raw *p*-values generated by limma were adjusted using the Benjamini–Hochberg false discovery rate (FDR) correction procedure. Genes demonstrating an absolute log_2_ fold change (|log_2_FC|) > 1 together with an FDR-adjusted *p* < 0.05 were considered significantly differentially expressed and retained for downstream interpretation. Normalization, probe-level preprocessing, and expression summarization procedures were conducted according to the preprocessing framework integrated into GEO2R and the original GEO platform annotations. Genes demonstrating an absolute log2 fold-change exceeding 1, along with an FDR-adjusted *p*-value below 0.05, were identified as differentially expressed. Raw and processed expression files (including platform-specific annotation resources and MINiML/SOFT files when applicable) were reviewed to ensure consistency of probe-to-gene mapping. Exported differential expression outputs were obtained directly from GEO2R without external statistical remodeling or reparameterization. To maximize methodological transparency and reproducibility, accession identifiers, platform information, statistical thresholds, correction methods, and filtering criteria were explicitly reported, thereby enabling independent replication using identical publicly accessible resources. Because GEO-derived transcriptomic analyses are observational and retrospective in nature, identified expression differences were interpreted as biologically relevant and hypothesis-generating findings requiring orthogonal validation in independent experimental systems. Comprehensive details for all datasets, including GEO accession identifiers, sample numbers, and array platforms, are provided in Supplementary Table S1.

### 4.11. CRISPR gRNA Design and Targetability Analysis

To evaluate the feasibility of CRISPR-Cas9-mediated modulation of target genes, we employed the CRISPRdb online resource (https://crisprdb.org/cgi-bin/search_gRNA.cgi, accessed on 17 November 2025) to identify and score candidate guide RNA (gRNA) sequences targeting key genes of interest, including HDAC6, TUBA1A, and CTNNB1. CRISPRdb integrates genomic context and off-target prediction to facilitate the selection of efficient and specific gRNAs for downstream functional studies. Briefly, the coding sequences (CDS) for each gene were retrieved from the Ensembl database (GRCh38/hg38 assembly). These sequences were submitted to the CRISPRdb search interface with default parameters optimized for SpCas9 targeting. Candidate gRNAs were filtered based on the presence of the canonical NGG protospacer adjacent motif (PAM). For each gene, the top 20 gRNA candidates with the highest on-target scores and lowest predicted off-target burden were extracted. On-target scores were obtained directly from CRISPRdb output, reflecting predicted Cas9 cleavage efficiency in the selected genomic context. Off-target assessment was based on genome-wide similarity searches with up to three mismatches outside the seed region, and sequences with significant off-target hits (defined as ≤3 mismatches in coding regions of other genes) were excluded from the final selection. Selected gRNAs were further evaluated for GC content and secondary structure propensity, ensuring optimal Cas9 binding and minimal formation of inhibitory hairpins. For each gene, final gRNAs were prioritized based on a composite ranking of on-target score, off-target risk, and sequence features known to influence CRISPR efficiency. This systematic in silico evaluation provides a foundation for subsequent experimental validation and supports the translational potential of CRISPR-based gene targeting approaches in hepatocellular carcinoma [87]. To improve methodological transparency and reproducibility, genome assembly version, PAM constraints, filtering criteria, target prioritization logic, and platform-retained settings were explicitly reported. Because CRISPR targetability analyses rely on computational prediction frameworks rather than experimental genome-editing validation, identified gRNA candidates were interpreted as translationally relevant and hypothesis-generating resources intended to support future functional validation studies rather than definitive evidence of gene-editing efficacy.

### 4.12. Drug Sensitivity Analysis of Genes

Drug sensitivity analysis was performed using the GSCALite platform (http://bioinfo.life.hust.edu.cn/web/GSCALite/ accessed on 17 November 2025, an integrated web-based tool that enables the exploration of gene expression, pathway activity, and drug response relationships across multiple cancer datasets. The analysis was conducted to evaluate the association between the mRNA expression levels of the selected genes and the sensitivity of cancer cell lines to various anticancer compounds. Drug response data were obtained from two large pharmacogenomic resources integrated within GSCALite: the Genomics of Drug Sensitivity in Cancer (GDSC) database and the Cancer Therapeutics Response Portal (CTRP). These datasets provide drug sensitivity information for numerous cancer cell lines along with corresponding transcriptomic profiles. Correlation analyses between gene expression levels and drug sensitivity were calculated using the built-in Spearman correlation method within the GSCALite platform. Drug response was represented by the half-maximal inhibitory concentration (IC50) values. The strength and direction of the association between gene expression and drug sensitivity were quantified using correlation coefficients. Statistical significance was evaluated using false discovery rate (FDR) correction to account for multiple testing. The results were visualized as bubble plots, where the color gradient represents the correlation coefficient (positive or negative association), and bubble size corresponds to the statistical significance expressed as −log10(FDR). Only statistically significant associations (FDR ≤ 0.05) were considered meaningful for interpretation [88]. To improve reproducibility, all analyses were conducted using explicitly reported gene identifiers, integrated pharmacogenomic datasets (GDSC and CTRP), predefined correlation methods, significance thresholds, and platform-retained default settings. Because pharmacogenomic sensitivity analyses are based on observational correlations derived from cancer cell-line datasets rather than prospective therapeutic testing, identified gene–drug associations were interpreted as biologically informative and hypothesis-generating translational candidates requiring future experimental and clinical validation.

### 4.13. Extracellular Vesicle-Derived RNA Analysis (exoRBase Database)

The exoRBase database (http://www.exorbase.org, accessed on 17 November 2025) was utilized to assess RNA profiles derived from extracellular vesicles (EVs) in circulation, which are associated with genes implicated in hepatocellular carcinoma. exoRBase is a publicly accessible resource founded on high-throughput RNA sequencing studies, encompassing expression data for exosomal mRNA, lncRNA, and circRNA obtained from human blood samples. In this investigation, the exosomal presence and expression profiles of HDAC6, TUBA1A, CTNNB1, and LncRNA SNHG1 were examined using the search module of exoRBase. During the analysis, comparisons were conducted between hepatocellular carcinoma (HCC) patient groups and healthy control samples. The normalized expression values presented in the database were scrutinized to evaluate trends in differential expression, and statistical significance information was directly acquired from the platform outputs. To interpret the biological and translational significance of the exosomal RNAs, the potential roles of the identified molecules in intercellular communication, liquid biopsy biomarker potential, and gene transfer mediated by extracellular vesicles were evaluated in conjunction with literature data. All data obtained from exoRBase were integrated with other bioinformatics analyses to delineate the vesicular components of the HDAC6-centered regulatory network in circulation [89]. Exported expression profiles and platform-generated statistical outputs were obtained directly from exoRBase without additional computational remodeling, normalization adjustment, or external statistical reanalysis. To improve methodological transparency and reproducibility, explicitly reported gene identifiers, disease categories, database source, access date, and platform-retained settings were systematically maintained across all analyses. Because exoRBase analyses are derived from observational transcriptomic datasets of circulating extracellular vesicles rather than experimentally validated vesicle-tracing studies, identified EV-associated RNA signatures were interpreted as biologically plausible and hypothesis-generating evidence requiring future experimental validation to establish mechanistic and clinical relevance in hepatocellular carcinoma.

### 4.14. Statistical Analysis

To enhance transparency and reproducibility, all analyses were performed using publicly accessible datasets and web-based analytical platforms with explicitly reported settings, thresholds, accession identifiers, and methodological parameters where applicable. The analytical workflow was designed to facilitate independent verification using the same publicly available resources. Statistical analyses were performed using the embedded analytical pipelines of publicly available bioinformatic platforms and databases. Gene expression comparisons conducted through TNMplot were evaluated using the Kruskal–Wallis test for comparisons among normal, primary tumor, and metastatic tissues, followed by Dunn’s post hoc test with adjustment for multiple comparisons when appropriate. Differential expression analyses of GEO microarray datasets were performed using the GEO2R interface based on the limma (Linear Models for Microarray Data) framework implemented in R, with *p*-values adjusted for multiple testing using the Benjamini–Hochberg false discovery rate (FDR) procedure. Genes with an absolute log2 fold change (|log2FC|) > 1 and FDR-adjusted *p* < 0.05 were considered significantly differentially expressed.

Immune infiltration analyses performed using TIMER 2.0 employed partial Spearman correlation analysis with adjustment for tumor purity to assess the association between gene expression and immune cell abundance. Statistical significance was determined according to the platform’s default parameters. Promoter methylation analyses in UALCAN were based on TCGA-derived Illumina HumanMethylation450 data, and tumor versus normal comparisons were conducted using two-tailed Student’s *t*-tests within the platform.

Survival analyses using the Kaplan–Meier Plotter platform were conducted by generating Kaplan–Meier curves and evaluating differences between expression-defined groups using two-sided log-rank tests. Hazard ratios (HRs) and 95% confidence intervals (CIs) were estimated through univariate Cox proportional hazards regression models. For protein–protein interaction enrichment analyses in STRING, functional enrichment significance was assessed using false discovery rate (FDR)-adjusted *p*-values, and pathways with FDR < 0.05 were considered statistically significant.

Drug sensitivity analyses performed through GSCALite evaluated associations between gene expression and anticancer drug response using Spearman correlation coefficients based on IC50 values obtained from CTRP and GDSC datasets. Multiple-testing correction was performed using FDR adjustment, and associations with FDR ≤ 0.05 were considered statistically meaningful. For computational miRNA interaction analyses (TargetScan, miRDB, and ENCORI), candidate regulators were prioritized according to platform-specific confidence metrics, including cumulative weighted context++ score (CWCS), probability of conserved targeting (PCT), target prediction scores, and CLIP-supported interaction evidence, without additional independent statistical modeling. Unless otherwise specified, a two-sided *p* < 0.05 was considered statistically significant.

## 5. Conclusions

In conclusion, this comprehensive multi-layered bioinformatic analysis elucidates a coherent HDAC6–TUBA1A–CTNNB1 regulatory axis that interconnects cytoplasmic deacetylation dynamics, microtubule remodeling, and Wnt/β-catenin-mediated oncogenic signaling in HCC. The observed pattern of HDAC6 downregulation, coupled with the upregulation of TUBA1A and CTNNB1, alongside epigenetic modifications, non-coding RNA regulation, immune microenvironment associations, and protein-interaction network connectivity, supports a model wherein post-translational acetylation balance and cytoskeletal reprogramming contribute to tumor progression and stage-dependent signaling adaptation. These findings position HDAC6 as a context-dependent immunoregulatory and potentially tumor-suppressive node, while suggesting that TUBA1A and CTNNB1 are involved in proliferative, migratory, and microenvironment-modulating processes without serving as independent prognostic determinants.

Furthermore, the enrichment patterns of extracellular vesicles and analyses of CRISPR-targetability advance this molecular framework towards translational strategies focused on gene delivery. This suggests that HDAC6 and CTNNB1 could be viable therapeutic targets within the vesicle-mediated regulatory interface. Upon comprehensive evaluation, the HDAC6/α-Tubulin/β-Catenin regulatory axis may be a promising target for therapeutic intervention. Although the current findings are hypothesis-generating and necessitate thorough experimental validation, they offer a pathway-level perspective that may guide future biomarker development, immune-modulatory approaches, and precision gene-editing interventions in HCC.

## Figures and Tables

**Table 1:** Combine Score Results of gene–gene interactions.

Gene-1	Gene-2	Protein Annotation	Combine Score
CTNN	HDAC6	Src substrate cortactin	0.996
EP300	HDAC6	Histone acetyltransferase p300	0.990
HCLS1	HDAC6	Hematopoietic lineage cell-specific protein	0.989
HSP90AA1	HDAC6	Heat shock protein HSP 90-alpha	0.999
HSP90AB1	HDAC6	Heat shock protein HSP 90-beta	0.998
RUNX2	HDAC6	Runt-related transcription factor 2	0.964
SQSTM1	HDAC6	Sequestosome-1	0.979
TARDBP	HDAC6	TAR DNA-binding protein 43	0.981
UBC	HDAC6	Polyubiquitin-C; [Ubiquitin]	0.995
VCP	HDAC6	Transitional endoplasmic reticulum ATPase	0.995
TUBB4B	TUBA1A	Tubulin beta-4B chain	0.999
TUBB4A	TUBA1A	Tubulin beta-4A chain	0.979
TUBB3	TUBA1A	Tubulin beta-3 chain	0.999
TUBB2B	TUBA1A	Tubulin beta-2B chain	0.997
TUBB2A	TUBA1A	Tubulin beta-2A chain	0.999
TUBB	TUBA1A	Tubulin beta chain	0.996
MAPT	TUBA1A	Microtubule-associated protein tau	0.978
DYNC1H1	TUBA1A	Cytoplasmic dynein 1 heavy chain 1	0.985
CLIP1	TUBA1A	CAP-Gly domain-containing linker protein 1	0.972
TUBB6	TUBA1A	Tubulin beta-6 chain	0.981
APC	CTNNB1	Adenomatous polyposis coli protein	0.999
AXIN1	CTNNB1	Axin-1	0.999
BCL9	CTNNB1	B-cell CLL/lymphoma 9 protein	0.999
CDH1	CTNNB1	Cadherin-1	0.999
CDH17	CTNNB1	Cadherin-17	0.999
CREBBP	CTNNB1	CREB-binding protein	0.999
CSNK1A1	CTNNB1	Casein kinase I isoform alpha	0.999
EP300	CTNNB1	Histone acetyltransferase p300	0.999
POU5F1	CTNNB1	POU domain, class 5, transcription factor 1	0.999
SKP1	CTNNB1	S-phase kinase-associated protein 1	0.999

**Table 2:** The miRNAs are associated with HDAC6, TUBA1A and CTNNB1, with a combination of TargetScanHuman8.0 and miRDB databases.

Predicted miRNAs for HDAC6 (n = 36)	
miRDB databases TargetScanHuman8.0	hsa-miR-3663-5p, hsa-miR-4430, hsa-miR-3652, hsa-miR-7151-3p, hsa-miR-6870-3p, hsa-miR-506-5p, hsa-miR-518a-5p, hsa-miR-527, hsa-miR-6763-5p, hsa-miR-3150a-3p, hsa-miR-200a-3p, hsa-miR-141-3p, hsa-miR-548n, hsa-miR-4753-3p, hsa-miR-2117, hsa-miR-6721-5p, hsa-miR-4690-5p, hsa-miR-4749-3p, hsa-miR-4492, hsa-miR-762, hsa-miR-5001-5p, hsa-miR-4498, hsa-miR-1587, hsa-miR-3620-5p, hsa-miR-4656, hsa-miR-378g, hsa-miR-6845-5p, hsa-miR-6762-5p, hsa-miR-1227-5p, hsa-miR-4469, hsa-miR-6736-3p, hsa-miR-433-3p, hsa-miR-4258, hsa-miR-4763-5p, hsa-miR-6872-3p, hsa-miR-1915-5p
Predicted miRNAs for TUBA1A (n = 18)	
miRDB databases TargetScanHuman8.0	hsa-miR-424-5p, hsa-miR-15a-5p, hsa-miR-15b-5p, hsa-miR-497-5p, hsa-miR-6838-5p, hsa-miR-195-5p, hsa-miR-16-5p, hsa-miR-221-3p, hsa-miR-222-3p, hsa-miR-3065-3p, hsa-miR-5003-3p, hsa-miR-2278, hsa-miR-1303, hsa-miR-1179, hsa-miR-330-3p, hsa-miR-4524b-3p, hsa-miR-499a-5p, hsa-miR-3658
	Predicted miRNAs for CTNNB1 (n = 133)
miRDB databases TargetScanHuman8.0	hsa-miR-330-3p, hsa-miR-5582-3p, hsa-miR-3119, hsa-miR-892b, hsa-miR-5591-3p, hsa-miR-2681-5p, hsa-miR-548L, hsa-miR-548n, hsa-miR-548az-5p, hsa-miR-548t-5p, hsa-miR-1910-3p, hsa-miR-6511a-5p, hsa-miR-3137, hsa-miR-148b-5p, hsa-miR-6874-3p, hsa-miR-148a-5p, hsa-miR-3691-3p, hsa-miR-3162-3p, hsa-miR-1224-3p, hsa-miR-3688-5p, hsa-miR-891a-3p, hsa-miR-3973, hsa-miR-4251, hsa-miR-4329, hsa-miR-6761-5p, hsa-miR-3200-5p, hsa-miR-4643, hsa-miR-3692-3p, hsa-miR-548at-5p, hsa-miR-561-3p, hsa-miR-3613-3p, hsa-miR-4470, hsa-miR-4429, hsa-miR-320c, hsa-miR-320d, hsa-miR-320b, hsa-miR-548g-5p, hsa-miR-548x-5p, hsa-miR-548aj-5p, hsa-miR-548f-5p, hsa-miR-548aw, hsa-miR-1468-3p, hsa-miR-4684-3p, hsa-miR-495-5p, hsa-miR-8081, hsa-miR-545-3p, hsa-miR-3120-3p, hsa-miR-33a-3p, hsa-miR-6762-3p, hsa-miR-1276, hsa-miR-340-5p, hsa-miR-4999-5p, hsa-miR-5000-5p, hsa-miR-1972, hsa-miR-6787-3p, hsa-miR-6715b-5p, hsa-miR-4269, hsa-miR-4742-5p, hsa-miR-4514, hsa-miR-4692, hsa-miR-4453, hsa-miR-4538, hsa-miR-512-5p, hsa-miR-4496, hsa-miR-642b-5p, hsa-miR-3682-5p, hsa-miR-589-3p, hsa-miR-6816-3p, hsa-miR-4668-3p, hsa-miR-5702, hsa-miR-6837-3p, hsa-miR-581, hsa-miR-640, hsa-miR-6069, hsa-miR-4708-3p, hsa-miR-5683, hsa-miR-6504-3p, hsa-miR-885-5p, hsa-miR-6512-5p, hsa-miR-624-5p, hsa-miR-138-1-3p, hsa-miR-6807-3p, hsa-miR-509-3-5p, hsa-miR-509-5p, hsa-miR-4418, hsa-miR-935, hsa-miR-141-3p, hsa-miR-200a-3p, hsa-miR-1537-5p, hsa-miR-4718, hsa-miR-302f, hsa-miR-4255, hsa-miR-183-3p, hsa-miR-5586-3p, hsa-miR-7849-3p, hsa-miR-892c-5p, hsa-miR-98-3p, hsa-let-7b-3p, hsa-let-7a-3p, hsa-let-7f-1-3p, hsa-miR-381-3p, hsa-miR-4666a-3p, hsa-miR-300, hsa-miR-1185-1-3p, hsa-miR-1185-2-3p, hsa-let-7f-2-3p, hsa-miR-4789-5p, hsa-miR-138-2-3p, hsa-miR-4796-5p, hsa-miR-4482-3p, hsa-miR-3619-3p, hsa-miR-4776-5p, hsa-miR-4535, hsa-miR-4503, hsa-miR-6792-5p, hsa-miR-7856-5p, hsa-miR-4733-5p, hsa-miR-21-3p, hsa-miR-548g-3p, hsa-miR-4490, hsa-miR-548an, hsa-miR-6513-5p, hsa-miR-5089-5p, hsa-miR-450b-3p, hsa-miR-769-3p, hsa-miR-6728-5p, hsa-miR-3529-3p, hsa-miR-657, hsa-miR-761, hsa-miR-214-3p, hsa-miR-3619-5p, hsa-miR-6809-3p, hsa-miR-153-5p

## Data Availability

The data used in this study were obtained from the public database TCGA and others.

## Supplementary Materials

The following supporting information can be downloaded at: https://www.mdpi.com/article/10.3390/ijms27125201/s1.

## Abbreviations

The following abbreviations are used in this manuscript:

HCC	Hepatocellular carcinoma
HDAC6	Histone deacetylase 6
TUBA1A	Tubulin α-1A
CTNNB1	Catenin β-1
TCGA	The Cancer Genome Atlas
GEO	Gene Expression Omnibus
GEPIA3	Gene Expression Profiling Interactive Analysis 3
HPA	Human Protein Atlas
TPM	Transcripts per million
IHC	Immunohistochemistry
TNMplot	Tumor–Normal–Metastasis plot platform
TIMER2.	Tumor Immune Estimation Resource 2.0
UALCA	University of Alabama at Birmingham Cancer Data Analysis Portal
KMplot	Kaplan–Meier Plotter
OS	Overall survival
RFS	Relapse-free survival
PFS	Progression-free survival
PPI	Protein–protein interaction
STRING	Search Tool for the Retrieval of Interacting Genes/Proteins
GO	Gene Ontology
KEGG	Kyoto Encyclopedia of Genes and Genomes
FDR	False discovery rate
lncRNA	Long non-coding RNA
miRNA	MicroRNA
