# Supplementary material for: Integrative Bioinformatic Characterization of the HDAC6-Driven Cytoskeleton–Wnt Signaling Interface in Hepatocellular Carcinoma: Implications for Immune Modulation and Therapeutic Targeting
Source: Int J Mol Sci. 2026 Jun 9;27(12):5201. doi: 10.3390/ijms27125201 (PMC13299183; doi:10.3390/ijms27125201)
Supplement: Supplementary file 1 [file ijms-4311074-supplementary.pdf › ijms-4311074-supplementary.pdf]

FID	adj.P.Val	P.Value	t
209365_s_at	4.91e-159	2.21e-163	-43.724.886
205019_s_at	7.26e-152	6.52e-156	-41.676.583
218002_s_at	4.84e-151	6.53e-155	-41.406.705
220114_s_at	2.77e-138	4.97e-142	-38.021.262
205866_at	5.10e-133	1.14e-136	-36.657.972
207609_s_at	5.69e-130	1.53e-133	-35.874.618
204428_s_at	4.20e-127	1.32e-130	-35.146.705
203554_x_at	1.19e-118	4.26e-122	330.759
207804_s_at	3.28e-117	1.33e-120	-32.718.022
220496_at	3.44e-117	1.55e-120	-32.702.144
205911_at	1.01e-116	4.99e-120	-32.580.598
205225_at	1.58e-116	8.52e-120	-32.525.215
211762_s_at	3.15e-116	1.84e-119	32.445.647
207608_x_at	2.07e-114	1.30e-117	-3.200.598
210481_s_at	4.25e-114	2.86e-117	-3.192.493
206797_at	9.34e-114	6.71e-117	-31.837.449
200910_at	5.63e-112	4.30e-115	31.411.463
219918_s_at	8.33e-112	6.74e-115	31.365.551
201088_at	1.27e-111	1.09e-114	31.316.786
222077_s_at	2.48e-108	2.23e-111	3.054.219
207262_at	6.92e-108	6.52e-111	-30.433.666
209189_at	2.31e-107	2.29e-110	-30.307.172
212554_at	2.78e-107	2.87e-110	30.284.109
214710_s_at	4.77e-106	5.14e-109	29.993.831
218009_s_at	9.24e-106	1.04e-108	29.923.278
201890_at	3.30e-105	3.85e-108	29.791.615
207584_at	5.78e-105	7.01e-108	-2.973.173
203316_s_at	5.39e-104	6.77e-107	29.504.757
220432_s_at	1.40e-103	1.83e-106	-29.405.528
206755_at	6.26e-103	8.43e-106	-29.253.104
206938_at	5.09e-102	7.09e-105	-29.041.229
220491_at	3.82e-101	5.49e-104	-28.837.819
207828_s_at	3.87e-101	5.73e-104	28.833.637
218039_at	1.43e-100	2.18e-103	28.701.309
201693_s_at	4.08e-100	6.41e-103	-28.594.416
213629_x_at	6.89e-100	1.11e-102	-28.539.682
209773_s_at	3.03e-99	5.03e-102	28.390.705
220070_at	3.45e-99	5.88e-102	-28.375.285
205554_s_at	4.16e-99	7.28e-102	-28.354.256
209714_s_at	1.15e-98	2.06e-101	28.251.428
202503_s_at	4.12e-98	7.59e-101	28.123.123
214431_at	7.51e-98	1.42e-100	28.061.683
201291_s_at	8.51e-98	1.64e-100	28.047.079

211609_x_at	1.48e-97	2.92e-100	2.799.055
204023_at	1.88e-97	3.81e-100	27.964.346
201128_s_at	2.23e-97	4.62e-100	2.794.545
201292_at	5.14e-97	1.08e-99	2.786.156
203213_at	1.46e-96	3.15e-99	27.756.748
214420_s_at	2.00e-96	4.40e-99	-27.724.074
218728_s_at	3.27e-96	7.35e-99	2.767.371
209531_at	3.82e-96	8.75e-99	-2.765.657
206380_s_at	1.28e-95	3.00e-98	-27.536.061
202690_s_at	1.47e-95	3.51e-98	27.520.557
201577_at	1.57e-95	3.82e-98	27.512.311
209687_at	2.13e-95	5.26e-98	-27.480.907
210524_x_at	2.44e-95	6.14e-98	-27.465.844
210106_at	4.58e-95	1.17e-97	-27.402.545
217946_s_at	4.76e-95	1.24e-97	27.397.018
200052_s_at	9.64e-95	2.55e-97	27.326.407
204955_at	2.99e-94	8.05e-97	-27.214.313
204026_s_at	3.48e-94	9.53e-97	2.719.779
206680_at	3.83e-94	1.07e-96	-27.186.814
211978_x_at	5.81e-94	1.64e-96	2.714.456
209614_at	8.10e-94	2.33e-96	-27.110.625
208079_s_at	1.66e-93	4.86e-96	27.038.876
204170_s_at	1.72e-93	5.09e-96	27.034.332
203666_at	2.13e-93	6.39e-96	-2.701.214
217165_x_at	2.19e-93	6.70e-96	-27.007.633
211378_x_at	6.59e-93	2.04e-95	26.899.084
209609_s_at	1.15e-92	3.62e-95	26.843.314
206102_at	1.43e-92	4.55e-95	26.821.027
220186_s_at	1.90e-92	6.15e-95	-267.918
221848_at	2.88e-92	9.45e-95	-26.749.979
207995_s_at	4.18e-92	1.39e-94	-26.712.507
57539_at	5.09e-92	1.72e-94	-26.692.058
211489_at	9.04e-92	3.08e-94	-26.635.068
203429_s_at	2.65e-91	9.15e-94	26.529.472
209335_at	4.73e-91	1.66e-93	-26.471.799
218350_s_at	1.05e-90	3.72e-93	26.393.447
222083_at	1.70e-90	6.11e-93	-26.345.381
212247_at	1.75e-90	6.37e-93	26.341.406
215712_s_at	2.14e-90	7.89e-93	-2.632.056
202861_at	7.01e-90	2.61e-92	-26.204.636
201694_s_at	1.40e-89	5.30e-92	-26.136.349
208644_at	1.84e-89	7.03e-92	26.108.962
222291_at	3.07e-89	1.18e-91	-26.058.532
200882_s_at	3.81e-89	1.49e-91	26.036.468

211765_x_at	4.41e-89	1.74e-91	26.021.228
201293_x_at	4.64e-89	1.86e-91	26.015.122
213375_s_at	5.73e-89	2.32e-91	-25.993.675
217546_at	9.02e-89	3.68e-91	-2.594.888
201714_at	2.33e-88	9.64e-91	2.585.605
212661_x_at	2.43e-88	1.01e-90	2.585.126
204767_s_at	6.11e-88	2.58e-90	25.761.109
205449_at	6.29e-88	2.68e-90	257.573
209836_x_at	8.80e-88	3.79e-90	25.723.939
208822_s_at	1.00e-87	4.37e-90	25.710.248
209219_at	2.50e-87	1.10e-89	25.621.429
336_at	2.87e-87	1.28e-89	-25.607.003
36829_at	4.81e-87	2.16e-89	-25.556.303
206610_s_at	4.98e-87	2.26e-89	-25.552.086
209220_at	1.94e-86	8.86e-89	25.420.462
214180_at	2.37e-86	1.10e-88	-25.399.901
205307_s_at	3.44e-86	1.61e-88	-253.633
204745_x_at	3.49e-86	1.64e-88	-25.361.071
205436_s_at	4.03e-86	1.92e-88	25.346.118
209044_x_at	4.58e-86	2.20e-88	25.332.917
212551_at	5.14e-86	2.50e-88	25.320.951
214419_s_at	5.50e-86	2.69e-88	-25.313.651
218585_s_at	1.22e-85	6.02e-88	25.236.256
218594_at	1.63e-85	8.13e-88	25.207.524
203120_at	1.94e-85	9.75e-88	25.189.985
207718_x_at	2.84e-85	1.44e-87	-25.152.604
206453_s_at	3.21e-85	1.65e-87	-25.139.771
214906_x_at	5.81e-85	3.00e-87	-25.082.126
218595_s_at	6.44e-85	3.35e-87	25.071.434
211072_x_at	6.45e-85	3.39e-87	25.070.373
201664_at	6.80e-85	3.60e-87	25.064.624
201090_x_at	8.94e-85	4.78e-87	2.503.751
202366_at	9.58e-85	5.16e-87	-25.030.101
211231_x_at	9.65e-85	5.24e-87	-25.028.555
200829_x_at	2.55e-84	1.40e-86	24.934.602
201663_s_at	5.98e-84	3.30e-86	24.852.101
213646_x_at	6.69e-84	3.73e-86	2.484.063
208369_s_at	8.25e-84	4.63e-86	-24.819.834
202469_s_at	9.15e-84	5.18e-86	24.809.057
217820_s_at	1.17e-83	6.66e-86	2.478.498
210105_s_at	1.37e-83	7.87e-86	-24.768.953
204326_x_at	2.64e-83	1.53e-85	-2.470.541
201377_at	2.66e-83	1.55e-85	24.703.916
219787_s_at	2.90e-83	1.71e-85	24.694.833

213661_at	3.13e-83	1.86e-85	-24.686.879
218544_s_at	4.96e-83	2.96e-85	-24.642.119
208581_x_at	5.51e-83	3.31e-85	-2.463.139
202244_at	5.81e-83	3.52e-85	24.625.518
209447_at	7.37e-83	4.50e-85	-24.602.102
201327_s_at	7.98e-83	4.91e-85	24.593.768
205984_at	8.06e-83	5.07e-85	-24.590.785
207027_at	8.06e-83	5.08e-85	-24.590.479
200788_s_at	8.06e-83	5.09e-85	24.590.422
201930_at	8.06e-83	5.10e-85	24.590.133
218622_at	9.60e-83	6.12e-85	24.572.707
205789_at	1.27e-82	8.13e-85	-24.545.575
211058_x_at	1.54e-82	9.93e-85	24.526.467
210460_s_at	1.70e-82	1.11e-84	24.516.239
202261_at	3.92e-82	2.57e-84	24.435.565
202954_at	4.42e-82	2.91e-84	24.423.543
219037_at	9.11e-82	6.06e-84	24.353.679
213832_at	9.72e-82	6.50e-84	-243.469
205531_s_at	1.35e-81	9.11e-84	-24.314.732
200826_at	1.47e-81	9.95e-84	24.306.282
206461_x_at	1.47e-81	1.00e-83	-243.057
202992_at	1.52e-81	1.05e-83	-24.301.518
206754_s_at	1.80e-81	1.25e-83	-24.284.837
207076_s_at	3.01e-81	2.09e-83	-24.235.229
213175_s_at	3.45e-81	2.41e-83	24.221.683
220446_s_at	4.09e-81	2.89e-83	-24.204.625
209218_at	4.58e-81	3.25e-83	24.193.263
217197_x_at	4.58e-81	3.27e-83	-24.192.718
213706_at	5.93e-81	4.26e-83	-24.167.472
207165_at	9.78e-81	7.07e-83	24.119.131
201897_s_at	1.20e-80	8.76e-83	2.409.874
216336_x_at	1.42e-80	1.04e-82	-24.082.102
210377_at	1.92e-80	1.42e-82	-24.052.938
218755_at	3.78e-80	2.80e-82	23.987.919
214621_at	3.80e-80	2.83e-82	-23.986.853
220878_at	3.99e-80	2.99e-82	-2.398.159
200652_at	5.92e-80	4.46e-82	2.394.349
201587_s_at	6.56e-80	4.98e-82	23.933.137
211456_x_at	8.09e-80	6.18e-82	-23.912.595
207069_s_at	1.28e-79	9.83e-82	-23.868.361
207244_x_at	1.34e-79	1.03e-81	-2.386.351
218285_s_at	1.44e-79	1.12e-81	-23.855.739
202422_s_at	1.68e-79	1.32e-81	23.840.521
200843_s_at	1.71e-79	1.34e-81	23.838.534

209825_s_at	1.91e-79	1.51e-81	23.827.232
212185_x_at	2.03e-79	1.61e-81	-23.821.281
218424_s_at	2.04e-79	1.63e-81	-23.820.263
217319_x_at	2.64e-79	2.13e-81	-2.379.486
210565_at	2.90e-79	2.34e-81	-23.785.674
203714_s_at	3.26e-79	2.65e-81	23.773.751
205998_x_at	3.92e-79	3.20e-81	-23.755.932
212591_at	5.44e-79	4.47e-81	23.724.151
206541_at	5.65e-79	4.67e-81	-23.719.966
212639_x_at	7.92e-79	6.58e-81	23.687.313
211896_s_at	8.16e-79	6.82e-81	-23.683.974
205773_at	8.85e-79	7.43e-81	-23.675.731
219954_s_at	9.07e-79	7.65e-81	-23.672.946
211813_x_at	1.09e-78	9.23e-81	-23.655.158
211295_x_at	1.86e-78	1.58e-80	-23.603.782
217755_at	1.95e-78	1.68e-80	23.598.353
203358_s_at	2.39e-78	2.06e-80	23.578.562
208937_s_at	2.42e-78	2.10e-80	-23.577.006
203547_at	2.59e-78	2.25e-80	-23.570.182
203500_at	3.09e-78	2.70e-80	-23.552.946
204641_at	3.68e-78	3.23e-80	23.535.844
202544_at	4.16e-78	3.68e-80	23.523.603
201518_at	8.10e-78	7.21e-80	23.459.716
221923_s_at	1.40e-77	1.25e-79	23.407.496
201555_at	1.58e-77	1.42e-79	23.395.299
211594_s_at	1.58e-77	1.42e-79	23.394.921
215014_at	5.36e-77	4.86e-79	-23.278.261
208694_at	7.59e-77	6.92e-79	23.244.857
209251_x_at	8.09e-77	7.41e-79	232.383
220975_s_at	8.34e-77	7.67e-79	-23.234.979
204825_at	8.66e-77	8.01e-79	23.230.853
212859_x_at	1.12e-76	1.04e-78	-23.206.091
220801_s_at	1.13e-76	1.05e-78	-2.320.488
209268_at	1.64e-76	1.54e-78	23.168.965
205819_at	2.16e-76	2.04e-78	-23.142.149
202846_s_at	2.46e-76	2.33e-78	2.312.957
220116_at	2.50e-76	2.38e-78	-23.127.603
210766_s_at	3.16e-76	3.03e-78	23.104.754
212832_s_at	3.19e-76	3.06e-78	23.103.564
202963_at	5.82e-76	5.62e-78	23.045.946
1494_f_at	1.01e-75	9.81e-78	-2.299.315
202243_s_at	1.22e-75	1.19e-77	22.975.039
201268_at	1.26e-75	1.23e-77	22.971.478
214320_x_at	1.37e-75	1.35e-77	-22.962.722

214512_s_at	1.55e-75	1.53e-77	22.951.007
209382_at	1.73e-75	1.71e-77	22.940.221
202095_s_at	1.98e-75	1.98e-77	22.926.594
201841_s_at	2.51e-75	2.51e-77	22.903.939
201893_x_at	3.08e-75	3.10e-77	-22.883.978
205392_s_at	3.29e-75	3.32e-77	-22.877.523
206629_at	3.57e-75	3.62e-77	-22.869.329
206867_at	3.79e-75	3.87e-77	-22.863.099
218768_at	4.04e-75	4.14e-77	22.856.669
217047_s_at	4.13e-75	4.25e-77	-22.854.168
210143_at	4.72e-75	4.88e-77	-22.841.076
201252_at	4.82e-75	5.00e-77	22.838.715
205053_at	5.17e-75	5.39e-77	22.831.544
219485_s_at	6.63e-75	6.94e-77	22.807.578
201656_at	1.05e-74	1.10e-76	2.276.364
213820_s_at	1.06e-74	1.12e-76	-22.762.219
211750_x_at	1.41e-74	1.50e-76	22.734.753
208821_at	1.56e-74	1.66e-76	22.724.989
208815_x_at	1.72e-74	1.84e-76	22.715.329
212766_s_at	2.08e-74	2.24e-76	22.696.669
203509_at	2.12e-74	2.29e-76	-22.694.509
202870_s_at	2.65e-74	2.86e-76	22.673.271
202324_s_at	3.05e-74	3.32e-76	22.659.394
214142_at	3.70e-74	4.04e-76	-22.640.697
219313_at	4.51e-74	4.95e-76	-22.621.479
209696_at	5.12e-74	5.63e-76	-22.609.164
208696_at	5.14e-74	5.70e-76	22.608.135
217978_s_at	5.14e-74	5.70e-76	22.608.107
202184_s_at	6.60e-74	7.36e-76	22.583.902
218233_s_at	7.58e-74	8.48e-76	22.570.426
213071_at	1.00e-73	1.12e-75	-22.543.732
209540_at	1.01e-73	1.13e-75	-22.542.979
213491_x_at	1.18e-73	1.34e-75	22.527.367
206753_at	1.25e-73	1.42e-75	-22.521.465
218662_s_at	1.36e-73	1.55e-75	22.513.119
203755_at	1.39e-73	1.59e-75	22.510.705
200703_at	1.42e-73	1.63e-75	22.508.711
212296_at	1.50e-73	1.73e-75	22.502.635
218918_at	1.55e-73	1.79e-75	-22.499.562
221188_s_at	1.55e-73	1.80e-75	-22.499.294
208370_s_at	1.86e-73	2.18e-75	-22.481.127
201731_s_at	1.88e-73	2.21e-75	22.479.849
207201_s_at	2.02e-73	2.38e-75	-22.472.759
218252_at	2.11e-73	2.49e-75	22.468.476

201112_s_at	2.20e-73	2.61e-75	22.464.114
218552_at	2.58e-73	3.07e-75	-22.448.659
213222_at	3.30e-73	3.94e-75	22.425.037
206030_at	3.61e-73	4.33e-75	-22.415.999
201342_at	4.79e-73	5.76e-75	22.388.998
209160_at	5.07e-73	6.12e-75	22.383.271
213355_at	7.54e-73	9.14e-75	-22.345.295
200064_at	8.08e-73	9.84e-75	22.338.336
218162_at	8.91e-73	1.09e-74	-22.328.803
209960_at	1.13e-72	1.38e-74	-2.230.626
202973_x_at	1.14e-72	1.41e-74	-22.304.565
201326_at	1.55e-72	1.92e-74	2.227.517
210691_s_at	1.55e-72	1.92e-74	22.275.064
213399_x_at	1.57e-72	1.96e-74	22.273.285
202651_at	2.30e-72	2.87e-74	22.237.102
219293_s_at	2.47e-72	3.10e-74	2.222.982
222036_s_at	3.19e-72	4.01e-74	22.205.347
209541_at	3.24e-72	4.09e-74	-22.203.513
210559_s_at	3.53e-72	4.47e-74	2.219.509
218361_at	4.42e-72	5.62e-74	2.217.344
212724_at	4.61e-72	5.88e-74	-2.216.913
214274_s_at	5.42e-72	6.93e-74	-22.153.568
210942_s_at	5.80e-72	7.45e-74	-22.146.737
218296_x_at	6.62e-72	8.53e-74	2.213.392
203721_s_at	7.42e-72	9.60e-74	22.122.772
213194_at	1.58e-71	2.06e-73	22.050.714
200068_s_at	1.71e-71	2.23e-73	22.042.905
203270_at	1.72e-71	2.24e-73	22.042.517
202025_x_at	1.85e-71	2.42e-73	-22.035.158
46323_at	1.93e-71	2.54e-73	22.030.874
202972_s_at	1.96e-71	2.59e-73	-22.028.705
207877_s_at	2.19e-71	2.89e-73	22.018.335
204244_s_at	2.63e-71	3.49e-73	22.000.569
207407_x_at	2.65e-71	3.54e-73	-21.999.346
218883_s_at	2.72e-71	3.63e-73	21.996.823
200062_s_at	2.84e-71	3.83e-73	21.991.865
205858_at	2.84e-71	3.83e-73	-21.991.792
213911_s_at	3.54e-71	4.78e-73	21.970.811
210589_s_at	4.89e-71	6.63e-73	21.939.936
207184_at	5.16e-71	7.02e-73	-2.193.457
201771_at	6.77e-71	9.24e-73	21.908.566
206916_x_at	6.91e-71	9.47e-73	-2.190.625
210052_s_at	7.25e-71	9.96e-73	21.901.503
202144_s_at	8.38e-71	1.16e-72	21.887.412

218672_at	9.68e-71	1.34e-72	21.873.541
214413_at	1.12e-70	1.56e-72	-21.859.016
213931_at	1.27e-70	1.76e-72	-21.847.433
200842_s_at	1.35e-70	1.88e-72	21.841.337
202487_s_at	1.40e-70	1.96e-72	21.837.603
200765_x_at	1.42e-70	1.99e-72	21.836.003
214890_s_at	1.42e-70	2.00e-72	-21.835.768
218532_s_at	1.83e-70	2.59e-72	-21.811.022
209852_x_at	2.08e-70	2.96e-72	2.179.857
210909_x_at	2.71e-70	3.86e-72	-21.773.482
204092_s_at	2.71e-70	3.87e-72	21.773.235
201477_s_at	3.51e-70	5.02e-72	21.748.486
218782_s_at	3.60e-70	5.18e-72	21.745.612
205442_at	4.20e-70	6.06e-72	-21.730.723
219148_at	4.37e-70	6.31e-72	21.726.879
208926_at	4.37e-70	6.34e-72	21.726.535
222303_at	4.39e-70	6.39e-72	-21.725.773
213626_at	5.24e-70	7.65e-72	-21.708.685
211303_x_at	5.54e-70	8.11e-72	-21.703.241
209421_at	5.64e-70	8.28e-72	21.701.186
201453_x_at	6.97e-70	1.03e-71	21.680.936
200792_at	7.10e-70	1.05e-71	21.678.863
206305_s_at	8.44e-70	1.25e-71	-2.166.223
205361_s_at	1.06e-69	1.58e-71	2.164.008
218355_at	1.36e-69	2.03e-71	21.616.686
209035_at	1.49e-69	2.22e-71	21.607.896
208034_s_at	1.59e-69	2.38e-71	-21.601.451
218541_s_at	2.13e-69	3.21e-71	-21.573.207
201956_s_at	2.13e-69	3.21e-71	21.573.144
202824_s_at	2.15e-69	3.26e-71	21.571.826
216058_s_at	2.51e-69	3.81e-71	-2.155.707
200783_s_at	3.11e-69	4.74e-71	21.536.326
218576_s_at	3.57e-69	5.44e-71	21.523.214
212371_at	3.86e-69	5.91e-71	21.515.457
202543_s_at	4.56e-69	7.02e-71	2.149.922
218905_at	4.56e-69	7.03e-71	21.499.042
202107_s_at	6.32e-69	9.76e-71	21.468.052
203218_at	6.63e-69	1.03e-70	2.146.327
217133_x_at	6.77e-69	1.05e-70	-21.460.941
204478_s_at	7.53e-69	1.17e-70	21.450.618
221498_at	7.53e-69	1.18e-70	21.450.396
212271_at	8.12e-69	1.27e-70	21.442.993
204822_at	9.73e-69	1.53e-70	21.425.621
33494_at	1.09e-68	1.71e-70	-21.414.832

212067_s_at	1.20e-68	1.89e-70	-21.405.594
205644_s_at	1.20e-68	1.91e-70	21.404.711
207102_at	1.81e-68	2.88e-70	-21.365.783
210095_s_at	1.82e-68	2.91e-70	-2.136.485
218061_at	1.92e-68	3.07e-70	21.359.799
206450_at	2.59e-68	4.15e-70	-21.331.283
221570_s_at	2.90e-68	4.67e-70	21.320.155
204905_s_at	3.89e-68	6.26e-70	2.129.236
201584_s_at	4.80e-68	7.76e-70	21.272.155
201531_at	6.14e-68	9.95e-70	-21.248.632
207735_at	6.34e-68	1.03e-69	-21.245.245
218239_s_at	6.40e-68	1.04e-69	21.244.174
205440_s_at	7.81e-68	1.28e-69	-2.122.505
202882_x_at	8.79e-68	1.44e-69	21.213.607
201425_at	9.10e-68	1.50e-69	-21.210.098
205813_s_at	9.78e-68	1.61e-69	-21.203.059
212168_at	1.08e-67	1.78e-69	21.193.743
206052_s_at	2.20e-67	3.64e-69	21.126.066
215450_at	2.49e-67	4.14e-69	2.111.393
221928_at	2.91e-67	4.85e-69	-21.099.024
211621_at	2.97e-67	4.97e-69	-21.096.701
205530_at	3.36e-67	5.63e-69	-21.084.882
206550_s_at	3.45e-67	5.80e-69	21.082.132
222056_s_at	5.01e-67	8.43e-69	-21.046.702
218510_x_at	5.23e-67	8.83e-69	-2.104.233
209236_at	5.37e-67	9.09e-69	-21.039.606
220218_at	5.58e-67	9.47e-69	-21.035.699
43427_at	5.73e-67	9.75e-69	-21.032.955
202580_x_at	5.91e-67	1.01e-68	21.029.845
208693_s_at	6.53e-67	1.12e-68	21.020.185
205657_at	7.06e-67	1.21e-68	-21.012.444
218561_s_at	7.88e-67	1.35e-68	21.001.908
201111_at	8.93e-67	1.54e-68	20.989.765
221139_s_at	1.00e-66	1.73e-68	-20.978.699
202705_at	1.16e-66	2.02e-68	20.964.297
213684_s_at	1.18e-66	2.04e-68	-20.963.039
200713_s_at	1.35e-66	2.35e-68	20.949.869
218983_at	1.41e-66	2.47e-68	-20.945.278
204224_s_at	1.42e-66	2.48e-68	-20.944.616
204134_at	1.43e-66	2.51e-68	-2.094.348
201381_x_at	1.56e-66	2.75e-68	20.935.023
206930_at	2.06e-66	3.63e-68	-20.908.698
218921_at	3.13e-66	5.54e-68	-20.868.836
202395_at	3.41e-66	6.05e-68	20.860.446

202475_at	4.28e-66	7.62e-68	20.838.728
219733_s_at	4.44e-66	7.91e-68	-20.835.133
206856_at	4.59e-66	8.20e-68	-2.083.176
204127_at	5.14e-66	9.21e-68	20.820.797
203073_at	5.24e-66	9.41e-68	208.188
212857_x_at	6.05e-66	1.09e-67	20.804.942
207041_at	6.34e-66	1.14e-67	-20.800.295
218984_at	6.55e-66	1.19e-67	20.796.982
216381_x_at	7.33e-66	1.33e-67	-20.786.124
200021_at	7.55e-66	1.37e-67	20.783.082
211458_s_at	8.17e-66	1.49e-67	-20.775.322
205582_s_at	8.54e-66	1.56e-67	-20.770.973
202246_s_at	1.13e-65	2.08e-67	20.743.854
206727_at	1.29e-65	2.36e-67	-20.731.867
52940_at	1.30e-65	2.39e-67	-20.730.677
202145_at	1.41e-65	2.61e-67	-20.722.528
210097_s_at	1.60e-65	2.96e-67	2.071.051
220112_at	1.99e-65	3.70e-67	-20.689.468
204700_x_at	2.02e-65	3.75e-67	20.688.092
219277_s_at	2.15e-65	4.02e-67	-20.681.656
219481_at	2.36e-65	4.41e-67	20.672.861
201764_at	2.37e-65	4.45e-67	20.672.056
213478_at	2.41e-65	4.52e-67	-20.670.432
219343_at	2.62e-65	4.94e-67	-20.662.173
213330_s_at	3.01e-65	5.67e-67	20.649.067
208808_s_at	3.10e-65	5.85e-67	20.646.053
211931_s_at	3.25e-65	6.15e-67	2.064.138
212143_s_at	3.46e-65	6.58e-67	-2.063.504
204719_at	3.78e-65	7.20e-67	-20.626.542
208029_s_at	4.11e-65	7.85e-67	20.618.327
204159_at	4.41e-65	8.43e-67	20.611.553
205498_at	4.86e-65	9.32e-67	-20.602.138
205306_x_at	4.96e-65	9.53e-67	-20.599.977
211138_s_at	5.78e-65	1.11e-66	-20.585.278
201472_at	8.80e-65	1.70e-66	20.545.374
222039_at	9.48e-65	1.83e-66	20.538.113
207202_s_at	1.02e-64	1.99e-66	-2.053.059
201074_at	1.09e-64	2.13e-66	20.524.061
49452_at	1.19e-64	2.33e-66	-20.515.681
219029_at	1.35e-64	2.63e-66	20.504.005
202309_at	1.36e-64	2.65e-66	-2.050.316
207168_s_at	2.06e-64	4.04e-66	20.463.526
209774_x_at	2.10e-64	4.13e-66	-20.461.499
202589_at	2.16e-64	4.26e-66	20.458.419

216237_s_at	2.16e-64	4.28e-66	20.458.076
218269_at	2.36e-64	4.68e-66	20.449.522
201275_at	2.37e-64	4.71e-66	20.448.963
208869_s_at	2.50e-64	4.97e-66	-20.443.944
201378_s_at	3.00e-64	5.98e-66	20.426.425
219978_s_at	3.08e-64	6.15e-66	20.423.808
202029_x_at	3.14e-64	6.28e-66	20.421.774
221685_s_at	3.28e-64	6.58e-66	20.417.414
201177_s_at	3.51e-64	7.05e-66	20.410.785
200853_at	3.99e-64	8.05e-66	20.398.351
206140_at	4.71e-64	9.52e-66	-2.038.246
211015_s_at	5.57e-64	1.13e-65	20.366.361
202892_at	5.77e-64	1.17e-65	20.362.888
201590_x_at	6.03e-64	1.23e-65	20.358.478
214039_s_at	7.03e-64	1.43e-65	20.343.714
212051_at	7.03e-64	1.44e-65	20.343.592
210233_at	7.14e-64	1.46e-65	-20.341.895
213320_at	7.71e-64	1.58e-65	20.334.361
213801_x_at	8.63e-64	1.78e-65	20.323.496
201201_at	8.90e-64	1.83e-65	20.320.407
203411_s_at	9.06e-64	1.87e-65	2.031.858
202276_at	9.83e-64	2.03e-65	20.310.645
221529_s_at	1.10e-63	2.28e-65	20.299.773
213503_x_at	1.14e-63	2.36e-65	20.296.553
210427_x_at	1.31e-63	2.73e-65	20.282.754
203800_s_at	1.57e-63	3.28e-65	20.265.448
209284_s_at	1.62e-63	3.39e-65	20.262.252
200594_x_at	1.82e-63	3.81e-65	20.251.246
205942_s_at	1.92e-63	4.03e-65	-20.245.918
200771_at	1.98e-63	4.16e-65	20.243.013
203682_s_at	2.14e-63	4.51e-65	-20.235.426
202810_at	2.23e-63	4.71e-65	20.231.206
222250_s_at	2.26e-63	4.80e-65	2.022.958
214765_s_at	2.56e-63	5.44e-65	-20.217.678
204763_s_at	2.66e-63	5.66e-65	-2.021.387
213608_s_at	2.68e-63	5.72e-65	20.212.928
211758_x_at	2.89e-63	6.18e-65	20.205.581
207773_x_at	2.90e-63	6.21e-65	-20.205.132
204289_at	3.14e-63	6.75e-65	-20.197.303
218092_s_at	3.19e-63	6.87e-65	20.195.632
217797_at	3.46e-63	7.47e-65	20.187.726
202345_s_at	4.30e-63	9.28e-65	2.016.714
201485_s_at	4.40e-63	9.51e-65	20.164.799
205790_at	4.56e-63	9.88e-65	-20.161.213

204162_at	4.83e-63	1.05e-64	20.155.478
202679_at	5.15e-63	1.12e-64	20.149.166
201795_at	5.67e-63	1.24e-64	20.139.873
213088_s_at	5.69e-63	1.24e-64	20.139.473
220524_at	6.20e-63	1.36e-64	-20.131.056
210337_s_at	6.38e-63	1.40e-64	20.128.171
212560_at	8.36e-63	1.84e-64	-20.102.409
202635_s_at	8.99e-63	1.98e-64	2.009.542
212141_at	1.01e-62	2.23e-64	20.084.434
218909_at	1.14e-62	2.53e-64	20.072.262
202081_at	1.17e-62	2.59e-64	-2.007.012
218238_at	1.20e-62	2.67e-64	20.067.375
219014_at	1.81e-62	4.04e-64	-20.028.018
217917_s_at	1.91e-62	4.25e-64	20.023.184
212160_at	1.93e-62	4.31e-64	20.021.868
219543_at	1.95e-62	4.38e-64	-20.020.455
204146_at	1.97e-62	4.42e-64	20.019.573
206706_at	2.12e-62	4.78e-64	-20.012.097
216958_s_at	2.23e-62	5.03e-64	-20.007.312
211761_s_at	2.42e-62	5.47e-64	19.999.379
208367_x_at	2.61e-62	5.92e-64	-19.991.938
202768_at	2.86e-62	6.49e-64	-19.983.188
214440_at	3.06e-62	6.94e-64	-19.976.804
212494_at	3.37e-62	7.67e-64	-19.967.385
221588_x_at	3.54e-62	8.08e-64	-19.962.486
218253_s_at	3.55e-62	8.11e-64	19.962.091
209712_at	4.88e-62	1.12e-63	-19.931.701
203362_s_at	4.91e-62	1.13e-63	19.931.063
200750_s_at	5.09e-62	1.17e-63	19.927.416
206469_x_at	5.60e-62	1.29e-63	-19.918.213
209787_s_at	5.97e-62	1.38e-63	19.911.858
209054_s_at	6.99e-62	1.62e-63	19.896.789
215231_at	7.27e-62	1.68e-63	-19.892.933
221590_s_at	7.52e-62	1.75e-63	-19.889.507
200757_s_at	7.90e-62	1.84e-63	19.884.718
202413_s_at	7.90e-62	1.84e-63	19.884.535
208021_s_at	8.11e-62	1.89e-63	19.881.843
215363_x_at	8.31e-62	1.94e-63	-19.879.394
200037_s_at	8.64e-62	2.02e-63	19.875.512
201266_at	1.01e-61	2.37e-63	19.860.483
200889_s_at	1.04e-61	2.44e-63	19.858.029
205176_s_at	1.14e-61	2.70e-63	19.848.404
200999_s_at	1.16e-61	2.73e-63	19.847.069
202737_s_at	1.18e-61	2.78e-63	19.845.453

213007_at	1.42e-61	3.36e-63	19.827.706
202772_at	1.55e-61	3.68e-63	-19.818.933
204301_at	1.82e-61	4.33e-63	-19.803.484
209760_at	1.82e-61	4.34e-63	-19.803.454
205194_at	2.19e-61	5.24e-63	19.785.573
203550_s_at	2.34e-61	5.61e-63	19.779.135
205042_at	2.56e-61	6.14e-63	-19.770.505
211441_x_at	2.61e-61	6.28e-63	-19.768.346
205999_x_at	2.89e-61	6.97e-63	-19.758.563
209053_s_at	3.02e-61	7.29e-63	1.975.429
220604_x_at	3.06e-61	7.39e-63	-19.753.005
203738_at	3.12e-61	7.56e-63	19.750.824
218213_s_at	3.37e-61	8.18e-63	19.743.364
206354_at	3.70e-61	8.99e-63	-19.734.401
221732_at	3.79e-61	9.22e-63	19.732.056
203008_x_at	3.85e-61	9.39e-63	19.730.293
204768_s_at	4.23e-61	1.03e-62	19.721.246
205860_x_at	4.38e-61	1.07e-62	-19.717.793
200877_at	4.57e-61	1.12e-62	19.713.654
202868_s_at	5.20e-61	1.28e-62	19.701.186
220367_s_at	5.28e-61	1.30e-62	19.699.358
214864_s_at	5.28e-61	1.30e-62	-1.969.934
213321_at	5.98e-61	1.48e-62	-19.687.405
212861_at	6.11e-61	1.51e-62	19.685.172
209542_x_at	7.10e-61	1.76e-62	-19.670.756
37201_at	7.36e-61	1.83e-62	-19.667.268
201681_s_at	8.21e-61	2.04e-62	19.656.718
215178_x_at	9.09e-61	2.26e-62	-19.646.947
219147_s_at	1.01e-60	2.51e-62	-19.637.071
219059_s_at	1.01e-60	2.52e-62	-1.963.699
201202_at	1.07e-60	2.68e-62	19.631.142
217558_at	1.12e-60	2.82e-62	-19.626.234
201199_s_at	1.68e-60	4.22e-62	19.587.997
208795_s_at	1.71e-60	4.30e-62	1.958.625
216661_x_at	1.74e-60	4.39e-62	-19.584.181
212688_at	2.37e-60	5.99e-62	19.554.888
57163_at	2.91e-60	7.36e-62	19.535.293
202666_s_at	2.96e-60	7.50e-62	19.533.463
203908_at	2.96e-60	7.54e-62	-19.533.057
200627_at	3.19e-60	8.11e-62	19.526.104
203725_at	3.37e-60	8.60e-62	-19.520.595
205476_at	3.54e-60	9.05e-62	19.515.756
210063_at	5.14e-60	1.32e-61	-19.480.178
203484_at	5.19e-60	1.33e-61	19.479.212

208659_at	5.31e-60	1.36e-61	19.476.825
219588_s_at	5.61e-60	1.44e-61	19.471.472
209015_s_at	5.69e-60	1.47e-61	19.469.995
206840_at	6.56e-60	1.69e-61	-19.456.384
209470_s_at	6.87e-60	1.78e-61	-19.451.689
203764_at	6.87e-60	1.78e-61	19.451.594
217918_at	8.75e-60	2.27e-61	1.942.848
213097_s_at	9.27e-60	2.41e-61	19.422.924
200608_s_at	1.03e-59	2.68e-61	19.412.814
202813_at	1.12e-59	2.93e-61	19.404.506
201985_at	1.22e-59	3.18e-61	19.396.544
205363_at	1.36e-59	3.56e-61	-19.385.971
217880_at	1.36e-59	3.57e-61	19.385.704
206210_s_at	1.62e-59	4.25e-61	-19.369.225
203022_at	1.63e-59	4.29e-61	19.368.325
203077_s_at	1.64e-59	4.31e-61	19.367.792
213226_at	1.68e-59	4.44e-61	19.364.897
217127_at	1.69e-59	4.47e-61	-19.364.305
201874_at	1.78e-59	4.73e-61	19.359.045
208805_at	1.94e-59	5.14e-61	19.351.135
213008_at	2.27e-59	6.04e-61	19.335.737
212405_s_at	2.42e-59	6.44e-61	19.329.736
218788_s_at	2.45e-59	6.53e-61	19.328.395
211442_x_at	2.45e-59	6.56e-61	-19.327.975
218308_at	2.80e-59	7.50e-61	19.315.286
203544_s_at	2.81e-59	7.53e-61	19.314.862
206424_at	2.85e-59	7.65e-61	-19.313.344
208103_s_at	3.56e-59	9.57e-61	19.292.201
208767_s_at	3.93e-59	1.06e-60	19.282.498
218663_at	4.14e-59	1.12e-60	19.277.391
214501_s_at	4.52e-59	1.22e-60	19.269.008
202153_s_at	5.26e-59	1.42e-60	19.254.523
202647_s_at	5.38e-59	1.46e-60	19.252.105
213009_s_at	6.37e-59	1.73e-60	1.923.596
202836_s_at	6.59e-59	1.79e-60	19.232.638
212765_at	6.60e-59	1.80e-60	19.232.342
219275_at	6.84e-59	1.87e-60	19.228.677
202983_at	8.39e-59	2.30e-60	19.209.174
200095_x_at	1.03e-58	2.82e-60	19.189.586
209786_at	1.28e-58	3.51e-60	19.168.995
211623_s_at	1.28e-58	3.51e-60	19.168.974
206068_s_at	1.37e-58	3.76e-60	-19.162.351
202168_at	1.45e-58	4.00e-60	1.915.643
221834_at	1.56e-58	4.31e-60	-19.149.321

215640_at	1.58e-58	4.38e-60	-19.147.879
211980_at	1.70e-58	4.70e-60	19.141.157
201479_at	1.71e-58	4.73e-60	19.140.517
209368_at	1.71e-58	4.76e-60	-19.140.049
214261_s_at	1.78e-58	4.95e-60	-1.913.622
202823_at	1.87e-58	5.21e-60	19.131.434
208758_at	1.88e-58	5.26e-60	1.913.044
206846_s_at	1.92e-58	5.37e-60	-19.128.574
205909_at	1.93e-58	5.41e-60	19.127.766
217519_at	2.11e-58	5.92e-60	-19.119.231
209711_at	2.12e-58	5.95e-60	-19.118.784
219212_at	2.12e-58	5.98e-60	19.118.277
202779_s_at	2.19e-58	6.17e-60	19.115.317
208938_at	2.49e-58	7.04e-60	19.102.852
203131_at	2.60e-58	7.36e-60	-19.098.668
221970_s_at	2.66e-58	7.54e-60	19.096.313
218512_at	3.09e-58	8.77e-60	19.081.933
200988_s_at	3.32e-58	9.42e-60	19.075.139
211110_s_at	3.76e-58	1.07e-59	-19.063.139
209974_s_at	4.01e-58	1.14e-59	19.056.671
204744_s_at	4.36e-58	1.25e-59	19.048.623
218726_at	4.38e-58	1.25e-59	19.048.024
218979_at	4.59e-58	1.32e-59	19.043.437
204997_at	4.88e-58	1.40e-59	-19.037.567
208147_s_at	6.45e-58	1.85e-59	-1.901.086
208700_s_at	7.37e-58	2.12e-59	1.899.812
216025_x_at	8.07e-58	2.33e-59	-18.989.378
214830_at	8.30e-58	2.40e-59	18.986.571
202322_s_at	8.39e-58	2.43e-59	18.985.385
201135_at	9.57e-58	2.77e-59	-18.972.722
204977_at	9.87e-58	2.86e-59	18.969.577
214421_x_at	1.05e-57	3.05e-59	-18.963.763
222231_s_at	1.12e-57	3.26e-59	18.957.409
208678_at	1.26e-57	3.68e-59	1.894.567
211542_x_at	1.32e-57	3.85e-59	18.941.422
202321_at	1.41e-57	4.11e-59	18.935.201
214737_x_at	1.44e-57	4.21e-59	18.932.909
218251_at	1.56e-57	4.56e-59	18.925.385
210328_at	1.71e-57	5.03e-59	-18.916.087
201608_s_at	1.82e-57	5.37e-59	1.890.996
200817_x_at	1.91e-57	5.64e-59	18.905.256
208816_x_at	2.06e-57	6.09e-59	1.889.799
205740_s_at	2.11e-57	6.22e-59	1.889.588
221505_at	2.53e-57	7.50e-59	18.878.137

220061_at	2.53e-57	7.51e-59	-18.877.985
221654_s_at	2.57e-57	7.62e-59	18.876.615
210250_x_at	2.60e-57	7.73e-59	18.875.295
208689_s_at	2.85e-57	8.48e-59	18.866.518
200639_s_at	3.58e-57	1.07e-58	18.844.613
200943_at	3.69e-57	1.10e-58	18.841.496
202020_s_at	3.98e-57	1.19e-58	18.834.335
200834_s_at	4.09e-57	1.22e-58	18.831.533
213279_at	4.16e-57	1.25e-58	-18.829.808
210168_at	4.31e-57	1.29e-58	-1.882.631
200944_s_at	5.45e-57	1.64e-58	18.803.811
216033_s_at	5.72e-57	1.72e-58	-18.799.112
217774_s_at	5.98e-57	1.80e-58	18.794.741
213165_at	6.48e-57	1.96e-58	18.786.952
219157_at	6.58e-57	1.99e-58	-18.785.229
200708_at	6.63e-57	2.01e-58	-18.784.301
204704_s_at	6.63e-57	2.01e-58	-18.784.262
214415_at	6.77e-57	2.06e-58	-18.782.224
219030_at	6.87e-57	2.09e-58	1.878.057
202200_s_at	7.80e-57	2.38e-58	18.768.458
202634_at	8.24e-57	2.52e-58	18.763.025
214889_at	9.70e-57	2.97e-58	-18.747.391
218556_at	1.06e-56	3.24e-58	18.738.892
203155_at	1.19e-56	3.66e-58	18.727.387
204511_at	1.20e-56	3.68e-58	-18.726.804
201743_at	1.20e-56	3.71e-58	-18.726.263
89977_at	1.37e-56	4.23e-58	-18.713.621
212519_at	1.41e-56	4.34e-58	18.711.271
200025_s_at	1.42e-56	4.37e-58	18.710.483
204962_s_at	1.61e-56	4.99e-58	18.697.969
200057_s_at	1.63e-56	5.06e-58	18.696.584
203336_s_at	1.88e-56	5.83e-58	18.683.125
207812_s_at	2.02e-56	6.29e-58	18.675.983
212407_at	2.03e-56	6.32e-58	18.675.535
202220_at	2.09e-56	6.53e-58	18.672.388
219060_at	2.13e-56	6.66e-58	18.670.504
213828_x_at	2.21e-56	6.92e-58	18.666.884
201041_s_at	2.66e-56	8.32e-58	-18.649.275
201347_x_at	3.05e-56	9.56e-58	-18.636.124
206364_at	3.34e-56	1.05e-57	18.627.217
209610_s_at	3.42e-56	1.07e-57	18.624.973
200770_s_at	4.03e-56	1.27e-57	18.609.216
205807_s_at	4.35e-56	1.37e-57	18.601.738
203432_at	4.42e-56	1.40e-57	18.600.104

221873_at	4.43e-56	1.40e-57	18.599.668
218272_at	4.60e-56	1.46e-57	-18.596.039
55065_at	5.03e-56	1.60e-57	18.587.308
202306_at	5.03e-56	1.60e-57	18.587.181
218816_at	5.13e-56	1.63e-57	18.585.273
200891_s_at	5.49e-56	1.75e-57	185.787
202223_at	7.73e-56	2.46e-57	1.854.592
205034_at	7.95e-56	2.54e-57	18.543.144
213720_s_at	8.01e-56	2.56e-57	18.542.265
218578_at	8.27e-56	2.65e-57	18.539.055
217286_s_at	8.40e-56	2.69e-57	1.853.746
216295_s_at	8.90e-56	2.86e-57	18.531.785
214414_x_at	9.06e-56	2.91e-57	-18.530.057
213310_at	9.25e-56	2.98e-57	18.527.909
201578_at	9.58e-56	3.09e-57	1.852.442
209469_at	9.97e-56	3.22e-57	-18.520.507
210983_s_at	1.08e-55	3.49e-57	18.512.666
220148_at	1.12e-55	3.61e-57	-18.509.478
209523_at	1.16e-55	3.75e-57	18.505.869
45288_at	1.32e-55	4.29e-57	-1.849.314
206239_s_at	1.38e-55	4.48e-57	18.489.085
218059_at	1.38e-55	4.50e-57	18.488.614
212626_x_at	1.63e-55	5.31e-57	18.472.754
200932_s_at	1.66e-55	5.41e-57	1.847.109
220017_x_at	1.69e-55	5.53e-57	-18.468.964
205384_at	2.02e-55	6.62e-57	-18.451.773
202961_s_at	2.03e-55	6.67e-57	18.451.134
219539_at	2.21e-55	7.27e-57	18.442.903
201458_s_at	3.37e-55	1.11e-56	1.840.252
201594_s_at	3.37e-55	1.11e-56	18.402.489
205259_at	3.53e-55	1.16e-56	-18.398.075
214734_at	3.63e-55	1.20e-56	-18.395.143
207203_s_at	3.76e-55	1.24e-56	-18.391.766
201127_s_at	3.82e-55	1.26e-56	18.390.256
209093_s_at	3.91e-55	1.30e-56	18.387.777
206262_at	4.22e-55	1.40e-56	-18.380.509
213881_x_at	4.24e-55	1.41e-56	1.837.978
212872_s_at	4.31e-55	1.43e-56	18.378.134
212226_s_at	5.10e-55	1.70e-56	-18.361.966
203138_at	5.10e-55	1.70e-56	18.361.832
221523_s_at	5.48e-55	1.83e-56	18.354.818
203732_at	5.62e-55	1.88e-56	18.352.317
212561_at	6.04e-55	2.02e-56	18.345.336
214829_at	6.53e-55	2.19e-56	-18.337.756

208979_at	7.58e-55	2.55e-56	18.323.393
215785_s_at	7.73e-55	2.60e-56	-18.321.408
211440_x_at	8.36e-55	2.81e-56	-18.313.844
201405_s_at	8.46e-55	2.85e-56	18.312.594
215578_at	8.51e-55	2.88e-56	-18.311.795
213427_at	9.11e-55	3.08e-56	18.305.233
203671_at	9.81e-55	3.32e-56	-18.297.997
208882_s_at	9.83e-55	3.33e-56	18.297.722
213804_at	9.90e-55	3.36e-56	-18.296.942
201077_s_at	1.07e-54	3.65e-56	18.288.984
218931_at	1.27e-54	4.33e-56	-18.272.831
211699_x_at	1.38e-54	4.70e-56	-18.264.888
216308_x_at	1.39e-54	4.75e-56	-18.263.995
204483_at	1.42e-54	4.84e-56	-18.262.165
208755_x_at	1.47e-54	5.03e-56	18.258.367
203262_s_at	1.51e-54	5.18e-56	18.255.675
208691_at	1.80e-54	6.19e-56	18.238.679
207544_s_at	1.94e-54	6.67e-56	-18.231.519
217022_s_at	1.95e-54	6.69e-56	-18.231.212
207421_at	2.00e-54	6.90e-56	-18.228.244
203856_at	2.05e-54	7.07e-56	1.822.602
200735_x_at	2.08e-54	7.19e-56	18.224.292
212541_at	2.20e-54	7.62e-56	1.821.879
210720_s_at	2.25e-54	7.78e-56	18.216.857
201406_at	2.35e-54	8.14e-56	18.212.557
201857_at	2.69e-54	9.35e-56	18.199.254
205189_s_at	2.79e-54	9.69e-56	-18.195.822
222043_at	2.81e-54	9.78e-56	-18.195.012
201261_x_at	2.84e-54	9.91e-56	-18.193.753
213577_at	2.96e-54	1.03e-55	18.189.671
207560_at	3.05e-54	1.06e-55	-18.186.871
214193_s_at	3.40e-54	1.19e-55	18.176.345
35820_at	3.43e-54	1.20e-55	18.175.296
217414_x_at	3.50e-54	1.23e-55	-18.173.402
214475_x_at	3.51e-54	1.23e-55	-18.172.775
205433_at	3.54e-54	1.24e-55	-18.172.035
218460_at	3.55e-54	1.25e-55	18.171.508
209709_s_at	3.57e-54	1.26e-55	18.170.934
218772_x_at	3.65e-54	1.29e-55	1.816.857
208383_s_at	3.66e-54	1.30e-55	-18.168.169
212461_at	3.76e-54	1.33e-55	18.165.532
210386_s_at	3.83e-54	1.36e-55	18.163.598
219632_s_at	3.88e-54	1.38e-55	-18.162.243
212530_at	4.05e-54	1.44e-55	18.158.075

209647_s_at	4.24e-54	1.51e-55	18.153.651
211946_s_at	4.53e-54	1.61e-55	18.147.294
213451_x_at	5.11e-54	1.82e-55	-18.135.635
218117_at	5.15e-54	1.84e-55	18.134.705
219960_s_at	5.49e-54	1.96e-55	1.812.855
217014_s_at	7.14e-54	2.56e-55	-18.103.232
210944_s_at	7.14e-54	2.56e-55	-18.103.114
218365_s_at	7.28e-54	2.61e-55	18.101.136
201524_x_at	7.77e-54	2.79e-55	1.809.488
213726_x_at	1.06e-53	3.82e-55	18.064.961
210768_x_at	1.06e-53	3.83e-55	18.064.645
207122_x_at	1.10e-53	3.98e-55	-18.060.971
208820_at	1.21e-53	4.36e-55	18.052.126
212811_x_at	1.28e-53	4.63e-55	18.046.471
206316_s_at	1.32e-53	4.78e-55	1.804.338
218708_at	1.33e-53	4.84e-55	1.804.232
218193_s_at	1.37e-53	4.96e-55	18.039.961
200787_s_at	1.37e-53	4.98e-55	18.039.593
204462_s_at	1.41e-53	5.14e-55	-18.036.466
206539_s_at	1.45e-53	5.28e-55	-18.033.934
215253_s_at	1.58e-53	5.76e-55	-18.025.525
212984_at	1.58e-53	5.77e-55	1.802.535
213699_s_at	1.71e-53	6.24e-55	1.801.791
221555_x_at	1.79e-53	6.55e-55	-18.013.335
209103_s_at	1.80e-53	6.61e-55	18.012.373
204541_at	1.90e-53	6.99e-55	-1.800.713
203012_x_at	1.98e-53	7.28e-55	1.800.322
201457_x_at	2.04e-53	7.50e-55	18.000.307
202326_at	2.06e-53	7.59e-55	1.799.919
208834_x_at	2.09e-53	7.70e-55	17.997.882
207668_x_at	2.17e-53	8.02e-55	17.993.936
211248_s_at	2.37e-53	8.75e-55	-1.798.557
219281_at	2.38e-53	8.79e-55	-17.985.129
203087_s_at	2.38e-53	8.83e-55	17.984.756
203436_at	2.46e-53	9.12e-55	17.981.622
200830_at	2.76e-53	1.03e-54	17.970.374
211940_x_at	3.12e-53	1.16e-54	17.958.621
212094_at	3.18e-53	1.18e-54	17.956.708
205073_at	3.19e-53	1.19e-54	-17.956.287
201459_at	3.20e-53	1.20e-54	17.955.804
212099_at	3.20e-53	1.20e-54	-17.955.672
208799_at	3.51e-53	1.31e-54	17.946.742
216333_x_at	3.68e-53	1.38e-54	-17.942.276
209458_x_at	3.73e-53	1.40e-54	-17.940.737

218324_s_at	3.79e-53	1.42e-54	17.939.058
211745_x_at	3.82e-53	1.43e-54	-17.938.322
203064_s_at	3.84e-53	1.44e-54	17.937.735
217973_at	3.95e-53	1.49e-54	-17.934.906
204018_x_at	4.30e-53	1.62e-54	-1.792.664
200710_at	4.84e-53	1.83e-54	-17.915.197
211890_x_at	5.25e-53	1.98e-54	-17.907.276
211385_x_at	5.36e-53	2.03e-54	-1.790.515
208775_at	5.44e-53	2.06e-54	17.903.671
204788_s_at	5.47e-53	2.07e-54	1.790.306
213905_x_at	5.54e-53	2.10e-54	-17.901.636
201947_s_at	5.72e-53	2.18e-54	17.898.407
212412_at	6.34e-53	2.41e-54	-17.888.568
208639_x_at	6.40e-53	2.44e-54	17.887.499
207842_s_at	6.95e-53	2.65e-54	17.879.446
210243_s_at	7.42e-53	2.84e-54	17.873.066
213610_s_at	7.51e-53	2.87e-54	17.871.898
202579_x_at	7.52e-53	2.88e-54	17.871.593
200614_at	7.60e-53	2.92e-54	17.870.431
201092_at	7.78e-53	2.99e-54	1.786.817
208743_s_at	7.91e-53	3.04e-54	17.866.386
201027_s_at	7.91e-53	3.04e-54	1.786.631
212737_at	8.24e-53	3.17e-54	17.862.356
203629_s_at	8.51e-53	3.28e-54	17.859.119
205728_at	8.52e-53	3.29e-54	-17.858.888
201940_at	8.55e-53	3.31e-54	17.858.415
203406_at	9.34e-53	3.62e-54	17.849.866
216564_at	9.92e-53	3.85e-54	-1.784.395
203160_s_at	1.21e-52	4.70e-54	17.824.834
214003_x_at	1.35e-52	5.26e-54	17.814.008
204290_s_at	1.43e-52	5.55e-54	-17.808.802
215017_s_at	1.48e-52	5.76e-54	17.805.307
209326_at	1.59e-52	6.19e-54	17.798.307
214764_at	1.66e-52	6.48e-54	17.793.924
218935_at	1.68e-52	6.55e-54	-17.792.986
201486_at	1.69e-52	6.61e-54	17.792.133
208825_x_at	1.84e-52	7.20e-54	1.778.393
213241_at	1.86e-52	7.30e-54	17.782.599
217829_s_at	2.05e-52	8.06e-54	17.773.111
208977_x_at	2.09e-52	8.22e-54	17.771.164
211470_s_at	2.12e-52	8.34e-54	17.769.762
213092_x_at	2.26e-52	8.90e-54	17.763.596
221833_at	2.37e-52	9.33e-54	-17.759.036
212563_at	2.41e-52	9.50e-54	17.757.336

222116_s_at	2.42e-52	9.57e-54	17.756.607
200863_s_at	2.42e-52	9.58e-54	17.756.521
202420_s_at	2.63e-52	1.04e-53	1.774.853
202633_at	2.90e-52	1.15e-53	17.739.043
214061_at	2.91e-52	1.15e-53	1.773.864
202591_s_at	2.91e-52	1.15e-53	17.738.603
214454_at	3.34e-52	1.33e-53	-17.725.165
204264_at	3.61e-52	1.44e-53	-1.771.765
204318_s_at	3.61e-52	1.44e-53	17.717.362
207738_s_at	3.79e-52	1.51e-53	17.712.788
211577_s_at	4.04e-52	1.61e-53	-17.706.571
202758_s_at	4.31e-52	1.72e-53	17.700.195
200875_s_at	4.61e-52	1.85e-53	17.693.585
219873_at	4.62e-52	1.85e-53	-17.693.257
212137_at	4.83e-52	1.94e-53	17.688.982
209567_at	4.94e-52	1.99e-53	17.686.655
203046_s_at	4.96e-52	1.99e-53	17.686.208
218014_at	5.06e-52	2.04e-53	17.684.113
212445_s_at	5.07e-52	2.04e-53	1.768.394
206287_s_at	5.07e-52	2.05e-53	-17.683.751
221922_at	5.10e-52	2.06e-53	17.683.132
209247_s_at	5.15e-52	2.08e-53	17.682.084
209645_s_at	5.17e-52	2.09e-53	-17.681.628
208738_x_at	5.44e-52	2.21e-53	17.676.606
210087_s_at	5.62e-52	2.28e-53	17.673.344
209460_at	5.64e-52	2.29e-53	-17.672.834
218049_s_at	5.86e-52	2.38e-53	1.766.913
201068_s_at	5.91e-52	2.41e-53	17.668.136
202027_at	6.02e-52	2.46e-53	17.666.272
218187_s_at	6.20e-52	2.53e-53	17.663.412
203284_s_at	6.39e-52	2.61e-53	17.660.371
214719_at	6.45e-52	2.64e-53	-17.659.371
219177_at	6.69e-52	2.74e-53	17.655.822
208766_s_at	7.22e-52	2.96e-53	17.648.329
219061_s_at	7.34e-52	3.01e-53	17.646.696
200763_s_at	8.21e-52	3.37e-53	17.635.879
201478_s_at	9.64e-52	3.96e-53	17.620.352
202754_at	1.02e-51	4.19e-53	17.614.921
219413_at	1.03e-51	4.23e-53	-1.761.406
202655_at	1.05e-51	4.34e-53	17.611.698
220108_at	1.07e-51	4.41e-53	-17.610.092
202874_s_at	1.22e-51	5.05e-53	17.597.077
203502_at	1.23e-51	5.08e-53	17.596.556
213988_s_at	1.25e-51	5.18e-53	-17.594.712

203983_at	1.33e-51	5.51e-53	17.588.821
208716_s_at	1.33e-51	5.51e-53	17.588.657
209459_s_at	1.40e-51	5.82e-53	-17.583.558
212467_at	1.40e-51	5.82e-53	17.583.503
209889_at	1.51e-51	6.30e-53	-1.757.588
218336_at	1.53e-51	6.37e-53	17.574.893
218741_at	1.54e-51	6.44e-53	17.573.734
210543_s_at	1.60e-51	6.71e-53	17.569.886
208843_s_at	1.64e-51	6.86e-53	17.567.762
205565_s_at	1.66e-51	6.95e-53	-17.566.392
220865_s_at	1.77e-51	7.43e-53	17.560.027
213134_x_at	1.91e-51	8.02e-53	17.552.744
205548_s_at	1.97e-51	8.29e-53	17.549.553
203119_at	2.00e-51	8.41e-53	17.548.156
203105_s_at	2.01e-51	8.46e-53	17.547.536
201091_s_at	2.03e-51	8.55e-53	17.546.588
212155_at	2.10e-51	8.88e-53	17.542.976
211492_s_at	2.10e-51	8.88e-53	-17.542.908
218982_s_at	2.12e-51	8.96e-53	1.754.204
203776_at	2.19e-51	9.26e-53	17.538.909
221509_at	2.28e-51	9.68e-53	17.534.615
215299_x_at	2.41e-51	1.02e-52	-17.529.178
200593_s_at	2.48e-51	1.05e-52	1.752.661
218757_s_at	2.48e-51	1.06e-52	17.526.324
201515_s_at	2.84e-51	1.21e-52	17.513.293
200987_x_at	2.86e-51	1.22e-52	17.512.525
212331_at	2.96e-51	1.26e-52	-17.509.184
206093_x_at	3.04e-51	1.30e-52	-17.506.548
218869_at	3.05e-51	1.30e-52	-17.506.176
203832_at	3.27e-51	1.40e-52	17.499.173
218504_at	3.27e-51	1.40e-52	-1.749.915
212370_x_at	3.41e-51	1.46e-52	17.494.886
217750_s_at	3.60e-51	1.54e-52	17.489.747
209304_x_at	3.72e-51	1.60e-52	-17.486.567
207420_at	3.91e-51	1.68e-52	-17.481.577
220365_at	4.24e-51	1.82e-52	-17.473.765
209408_at	4.41e-51	1.90e-52	17.469.755
213084_x_at	4.89e-51	2.11e-52	17.459.742
208939_at	5.10e-51	2.20e-52	17.455.644
211933_s_at	5.18e-51	2.24e-52	17.453.955
205871_at	5.41e-51	2.34e-52	-17.449.782
209572_s_at	5.58e-51	2.42e-52	17.446.686
203228_at	5.63e-51	2.44e-52	17.445.753
212539_at	5.66e-51	2.46e-52	17.445.034

213476_x_at	5.83e-51	2.53e-52	17.442.188
204033_at	6.53e-51	2.84e-52	17.431.179
81737_at	7.06e-51	3.08e-52	-17.423.529
209903_s_at	7.25e-51	3.16e-52	17.420.889
204263_s_at	7.48e-51	3.27e-52	-17.417.815
203615_x_at	7.95e-51	3.47e-52	-17.411.846
201755_at	8.26e-51	3.61e-52	17.408.091
202558_s_at	8.57e-51	3.75e-52	17.404.398
218024_at	8.81e-51	3.86e-52	-17.401.692
201390_s_at	9.00e-51	3.95e-52	17.399.499
200065_s_at	9.30e-51	4.08e-52	17.396.299
212022_s_at	9.49e-51	4.17e-52	17.394.254
203214_x_at	9.53e-51	4.19e-52	17.393.757
201941_at	1.01e-50	4.43e-52	17.388.441
217972_at	1.03e-50	4.53e-52	17.386.392
201343_at	1.06e-50	4.69e-52	17.383.069
209849_s_at	1.09e-50	4.84e-52	1.737.999
205890_s_at	1.13e-50	5.01e-52	17.376.716
208726_s_at	1.23e-50	5.47e-52	17.368.232
213404_s_at	1.23e-50	5.47e-52	17.368.198
207185_at	1.24e-50	5.52e-52	-17.367.319
212287_at	1.43e-50	6.34e-52	17.354.047
201973_s_at	1.47e-50	6.54e-52	17.350.952
48031_r_at	1.49e-50	6.64e-52	-17.349.613
201289_at	1.49e-50	6.66e-52	-17.349.266
208707_at	1.61e-50	7.20e-52	-17.341.787
214946_x_at	1.65e-50	7.38e-52	17.339.344
201622_at	1.66e-50	7.41e-52	17.338.931
218185_s_at	1.71e-50	7.64e-52	17.336.046
220949_s_at	1.73e-50	7.73e-52	17.334.904
215791_at	1.74e-50	7.78e-52	-1.733.427
202872_at	1.88e-50	8.44e-52	17.326.479
219129_s_at	2.07e-50	9.30e-52	17.317.145
201020_at	2.16e-50	9.69e-52	17.313.126
210301_at	2.18e-50	9.83e-52	-17.311.785
207574_s_at	2.21e-50	9.94e-52	-17.310.718
213874_at	2.35e-50	1.06e-51	-17.304.428
210889_s_at	2.57e-50	1.16e-51	-17.295.685
34868_at	2.58e-50	1.17e-51	1.729.541
219791_s_at	2.61e-50	1.18e-51	-17.294.155
204273_at	2.63e-50	1.19e-51	-1.729.353
208635_x_at	2.63e-50	1.19e-51	172.933
208319_s_at	2.63e-50	1.19e-51	17.293.209
213581_at	2.71e-50	1.23e-51	17.290.057

221486_at	2.79e-50	1.27e-51	17.287.127
200890_s_at	2.84e-50	1.29e-51	17.285.367
203693_s_at	2.93e-50	1.34e-51	17.282.297
50374_at	2.97e-50	1.36e-51	17.280.838
213247_at	3.04e-50	1.39e-51	-17.278.602
202911_at	3.04e-50	1.39e-51	17.278.545
212055_at	3.07e-50	1.40e-51	17.277.547
212597_s_at	3.19e-50	1.46e-51	17.273.714
205664_at	3.25e-50	1.49e-51	17.271.691
200661_at	3.28e-50	1.51e-51	17.270.731
200063_s_at	3.33e-50	1.53e-51	17.269.207
202003_s_at	3.55e-50	1.63e-51	-1.726.298
210452_x_at	3.56e-50	1.64e-51	-17.262.569
210844_x_at	3.59e-50	1.65e-51	1.726.188
200717_x_at	3.72e-50	1.71e-51	17.258.259
213518_at	3.88e-50	1.79e-51	17.254.055
206069_s_at	3.92e-50	1.81e-51	-17.253.023
201875_s_at	4.07e-50	1.88e-51	17.249.247
214086_s_at	4.36e-50	2.02e-51	17.242.539
200960_x_at	4.52e-50	2.09e-51	17.238.976
207275_s_at	4.54e-50	2.11e-51	-17.238.453
203840_at	5.48e-50	2.54e-51	1.722.028
219188_s_at	5.61e-50	2.61e-51	-17.217.911
203145_at	6.24e-50	2.90e-51	17.207.576
214728_x_at	7.08e-50	3.29e-51	17.195.338
202041_s_at	7.32e-50	3.41e-51	1.719.202
203854_at	7.88e-50	3.68e-51	-17.184.758
207243_s_at	8.39e-50	3.91e-51	1.717.871
207491_at	8.56e-50	4.00e-51	-17.176.692
204057_at	8.68e-50	4.06e-51	-17.175.158
201527_at	8.76e-50	4.10e-51	17.174.205
221552_at	8.81e-50	4.13e-51	-17.173.543
200640_at	8.83e-50	4.14e-51	17.173.278
209187_at	9.36e-50	4.39e-51	171.676
206436_at	1.01e-49	4.76e-51	-17.159.879
209613_s_at	1.02e-49	4.81e-51	-17.158.722
200957_s_at	1.03e-49	4.85e-51	17.158.018
201898_s_at	1.11e-49	5.25e-51	17.150.451
201943_s_at	1.12e-49	5.27e-51	1.715.003
210859_x_at	1.12e-49	5.30e-51	17.149.453
219820_at	1.22e-49	5.78e-51	-17.141.098
202918_s_at	1.27e-49	6.00e-51	17.137.425
210764_s_at	1.36e-49	6.42e-51	-17.131.037
213356_x_at	1.43e-49	6.78e-51	17.125.668

201260_s_at	1.46e-49	6.90e-51	17.123.998
217809_at	1.51e-49	7.15e-51	1.712.059
203258_at	1.52e-49	7.23e-51	17.119.545
201725_at	1.61e-49	7.65e-51	17.114.022
201303_at	1.98e-49	9.43e-51	17.093.863
218316_at	2.06e-49	9.81e-51	17.090.087
218243_at	2.18e-49	1.04e-50	17.084.442
207218_at	2.21e-49	1.06e-50	-17.082.827
211297_s_at	2.21e-49	1.06e-50	17.082.751
220751_s_at	2.23e-49	1.07e-50	-17.082.075
203477_at	2.42e-49	1.16e-50	17.073.933
201516_at	2.56e-49	1.23e-50	17.068.523
209104_s_at	2.91e-49	1.39e-50	17.056.185
206085_s_at	3.15e-49	1.51e-50	-17.048.253
203622_s_at	3.25e-49	1.56e-50	17.045.325
201432_at	3.26e-49	1.57e-50	-17.044.929
202960_s_at	3.32e-49	1.60e-50	-17.043.026
38487_at	3.44e-49	1.66e-50	-17.039.377
212486_s_at	3.87e-49	1.87e-50	-17.027.994
209172_s_at	3.88e-49	1.87e-50	17.027.704
215157_x_at	4.40e-49	2.13e-50	17.015.419
209688_s_at	4.45e-49	2.15e-50	17.014.144
218681_s_at	5.21e-49	2.52e-50	16.998.919
212345_s_at	5.49e-49	2.66e-50	16.993.748
218842_at	5.79e-49	2.81e-50	16.988.579
218229_s_at	5.89e-49	2.86e-50	16.986.715
216813_at	6.08e-49	2.95e-50	-16.983.595
217770_at	6.41e-49	3.12e-50	16.978.401
205633_s_at	6.88e-49	3.35e-50	-16.971.514
209899_s_at	6.92e-49	3.37e-50	16.970.786
46256_at	7.38e-49	3.60e-50	-16.964.576
218391_at	7.70e-49	3.76e-50	16.960.396
200949_x_at	8.48e-49	4.14e-50	16.950.963
221522_at	8.62e-49	4.21e-50	16.949.288
201772_at	8.78e-49	4.30e-50	16.947.414
205342_s_at	9.00e-49	4.41e-50	16.944.859
208699_x_at	9.76e-49	4.79e-50	16.936.982
203654_s_at	9.92e-49	4.87e-50	16.935.305
219862_s_at	1.04e-48	5.10e-50	16.930.858
210907_s_at	1.05e-48	5.17e-50	16.929.589
209642_at	1.07e-48	5.26e-50	16.927.874
211628_x_at	1.10e-48	5.40e-50	16.925.238
217970_s_at	1.16e-48	5.70e-50	16.920.064
211275_s_at	1.18e-48	5.81e-50	16.918.265

202916_s_at	1.18e-48	5.84e-50	16.917.755
202733_at	1.29e-48	6.38e-50	16.909.246
202847_at	1.33e-48	6.59e-50	-16.906.096
221589_s_at	1.35e-48	6.68e-50	-16.904.758
201379_s_at	1.38e-48	6.84e-50	16.902.383
202154_x_at	1.44e-48	7.16e-50	16.898.059
206344_at	1.45e-48	7.23e-50	-16.897.133
221488_s_at	1.51e-48	7.52e-50	16.893.285
205355_at	1.54e-48	7.64e-50	-16.891.778
207621_s_at	1.57e-48	7.80e-50	-16.889.686
212621_at	1.60e-48	7.97e-50	16.887.672
217916_s_at	1.74e-48	8.67e-50	16.879.525
219363_s_at	1.87e-48	9.34e-50	16.872.312
218046_s_at	1.90e-48	9.47e-50	16.870.942
201241_at	2.07e-48	1.03e-49	16.862.479
44673_at	2.08e-48	1.04e-49	-16.861.553
209309_at	2.12e-48	1.06e-49	-1.685.968
213528_at	2.13e-48	1.07e-49	16.859.451
219004_s_at	2.16e-48	1.08e-49	16.857.869
208883_at	2.18e-48	1.10e-49	16.856.709
206561_s_at	2.24e-48	1.13e-49	1.685.413
200092_s_at	2.34e-48	1.18e-49	16.849.665
209612_s_at	2.42e-48	1.22e-49	-16.846.535
215563_s_at	2.57e-48	1.29e-49	-16.840.762
206709_x_at	2.74e-48	1.38e-49	-16.834.408
218853_s_at	2.91e-48	1.47e-49	16.828.475
206514_s_at	3.08e-48	1.56e-49	-16.822.679
210751_s_at	3.16e-48	1.60e-49	-16.820.327
208931_s_at	3.39e-48	1.72e-49	16.813.368
213941_x_at	3.44e-48	1.74e-49	16.811.905
211056_s_at	3.52e-48	1.78e-49	-16.809.619
208688_x_at	3.57e-48	1.81e-49	16.808.008
211989_at	3.59e-48	1.83e-49	16.807.456
205335_s_at	3.60e-48	1.83e-49	16.807.171
202126_at	3.65e-48	1.86e-49	16.805.784
200873_s_at	4.14e-48	2.11e-49	1.679.339
221827_at	4.68e-48	2.39e-49	16.781.493
203521_s_at	4.97e-48	2.54e-49	16.775.483
213562_s_at	5.34e-48	2.73e-49	16.768.489
217919_s_at	5.42e-48	2.77e-49	16.766.979
200709_at	5.84e-48	2.99e-49	16.759.723
200662_s_at	5.98e-48	3.06e-49	16.757.257
207840_at	6.36e-48	3.26e-49	-16.751.168
205661_s_at	6.70e-48	3.44e-49	16.746.071

203957_at	6.73e-48	3.45e-49	1.674.564
215330_at	7.07e-48	3.63e-49	-16.740.757
202264_s_at	7.58e-48	3.90e-49	1.673.386
203182_s_at	7.72e-48	3.98e-49	16.732.031
200020_at	7.79e-48	4.02e-49	16.731.045
210473_s_at	7.93e-48	4.09e-49	-16.729.187
202926_at	8.60e-48	4.44e-49	16.721.343
217852_s_at	9.21e-48	4.76e-49	16.714.604
210891_s_at	9.34e-48	4.83e-49	16.713.146
212335_at	9.71e-48	5.03e-49	16.709.317
212871_at	1.01e-47	5.26e-49	16.704.982
207820_at	1.06e-47	5.48e-49	-16.700.915
221769_at	1.07e-47	5.55e-49	-16.699.649
219253_at	1.30e-47	6.74e-49	16.680.834
201508_at	1.37e-47	7.13e-49	-16.675.368
205627_at	1.49e-47	7.74e-49	-16.667.398
200029_at	1.52e-47	7.93e-49	16.665.055
214853_s_at	1.57e-47	8.19e-49	16.661.956
219933_at	1.75e-47	9.16e-49	1.665.112
205812_s_at	1.77e-47	9.23e-49	16.650.389
208022_s_at	1.87e-47	9.79e-49	-16.644.596
209814_at	1.90e-47	9.94e-49	-16.643.117
209891_at	1.90e-47	9.97e-49	16.642.852
204150_at	2.03e-47	1.06e-48	-16.636.794
219713_at	2.10e-47	1.10e-48	-166.332
217835_x_at	2.13e-47	1.12e-48	16.631.852
210739_x_at	2.18e-47	1.15e-48	-16.629.424
204050_s_at	2.24e-47	1.18e-48	1.662.675
201583_s_at	2.26e-47	1.19e-48	16.625.728
201198_s_at	2.31e-47	1.22e-48	16.623.539
208051_s_at	2.72e-47	1.44e-48	16.607.486
201697_s_at	2.82e-47	1.49e-48	16.604.098
202642_s_at	3.14e-47	1.66e-48	16.593.322
210438_x_at	3.57e-47	1.89e-48	16.580.978
204488_at	3.65e-47	1.93e-48	16.578.606
204634_at	3.76e-47	1.99e-48	16.575.653
201797_s_at	3.79e-47	2.01e-48	16.574.894
212129_at	3.94e-47	2.09e-48	16.571.129
206024_at	4.08e-47	2.17e-48	-16.567.514
201521_s_at	4.16e-47	2.21e-48	16.565.564
219161_s_at	4.53e-47	2.41e-48	16.557.178
218133_s_at	5.49e-47	2.92e-48	1.653.852
211501_s_at	5.92e-47	3.16e-48	16.530.961
214478_at	6.38e-47	3.40e-48	-16.523.736

218349_s_at	6.99e-47	3.73e-48	16.514.747
219675_s_at	7.56e-47	4.04e-48	16.506.949
207096_at	8.51e-47	4.55e-48	-16.495.429
201451_x_at	8.56e-47	4.58e-48	-1.649.478
219119_at	8.71e-47	4.66e-48	16.492.969
204931_at	8.79e-47	4.71e-48	-1.649.199
218429_s_at	8.90e-47	4.77e-48	-16.490.739
219137_s_at	9.10e-47	4.89e-48	16.488.448
201773_at	9.89e-47	5.31e-48	16.480.356
202691_at	1.00e-46	5.40e-48	16.478.771
212825_at	1.06e-46	5.72e-48	16.473.043
208952_s_at	1.09e-46	5.89e-48	16.470.242
200828_s_at	1.13e-46	6.10e-48	16.466.898
215380_s_at	1.16e-46	6.27e-48	16.464.194
201025_at	1.28e-46	6.92e-48	16.454.531
206571_s_at	1.35e-46	7.30e-48	16.449.346
209853_s_at	1.38e-46	7.47e-48	16.447.196
209237_s_at	1.39e-46	7.50e-48	-16.446.713
209275_s_at	1.44e-46	7.80e-48	16.442.995
200936_at	1.65e-46	8.92e-48	16.429.868
213485_s_at	1.70e-46	9.21e-48	16.426.772
219453_at	1.75e-46	9.51e-48	-16.423.612
200837_at	1.80e-46	9.76e-48	16.421.097
201271_s_at	1.81e-46	9.87e-48	16.420.055
200028_s_at	1.83e-46	9.94e-48	16.419.291
212390_at	1.87e-46	1.02e-47	-16.417.033
216194_s_at	1.90e-46	1.03e-47	16.415.444
203408_s_at	2.03e-46	1.11e-47	-16.408.844
201963_at	2.17e-46	1.19e-47	-16.402.031
218107_at	2.28e-46	1.24e-47	16.397.424
218497_s_at	2.39e-46	1.31e-47	16.392.539
218538_s_at	2.46e-46	1.34e-47	16.389.929
204880_at	2.47e-46	1.35e-47	-16.389.205
201144_s_at	2.56e-46	1.41e-47	16.385.588
204231_s_at	2.62e-46	1.44e-47	-16.383.537
214506_at	2.65e-46	1.45e-47	-16.382.371
201566_x_at	2.73e-46	1.50e-47	-16.379.285
211963_s_at	2.74e-46	1.51e-47	16.378.713
201592_at	2.84e-46	1.56e-47	16.375.173
217718_s_at	2.95e-46	1.63e-47	16.371.397
216696_s_at	3.10e-46	1.71e-47	-1.636.646
201582_at	3.14e-46	1.73e-47	16.365.206
219553_at	3.25e-46	1.79e-47	16.361.886
213929_at	3.48e-46	1.92e-47	-16.355.024

205167_s_at	3.51e-46	1.94e-47	1.635.416
214211_at	3.51e-46	1.94e-47	16.354.157
200638_s_at	3.63e-46	2.01e-47	16.350.765
206188_at	3.66e-46	2.03e-47	16.349.845
211759_x_at	3.89e-46	2.16e-47	16.343.708
213068_at	3.91e-46	2.17e-47	-16.343.268
201672_s_at	3.91e-46	2.17e-47	16.343.117
209313_at	3.95e-46	2.20e-47	16.342.058
212799_at	3.99e-46	2.22e-47	16.340.883
201760_s_at	4.12e-46	2.30e-47	16.337.773
210959_s_at	4.66e-46	2.60e-47	-16.325.588
206979_at	4.76e-46	2.66e-47	-16.323.578
212144_at	4.84e-46	2.70e-47	-16.321.879
200005_at	5.24e-46	2.93e-47	16.313.981
218360_at	5.28e-46	2.96e-47	16.313.178
218715_at	5.78e-46	3.24e-47	16.304.226
210268_at	5.86e-46	3.28e-47	-16.302.916
202217_at	5.93e-46	3.33e-47	-16.301.578
204444_at	5.96e-46	3.35e-47	16.301.083
220239_at	6.21e-46	3.49e-47	16.296.992
213293_s_at	6.28e-46	3.53e-47	-16.295.739
209902_at	6.38e-46	3.59e-47	16.294.137
217885_at	6.57e-46	3.70e-47	16.291.325
206178_at	7.02e-46	3.96e-47	-16.284.738
203142_s_at	7.14e-46	4.02e-47	16.283.039
200040_at	7.17e-46	4.05e-47	16.282.464
212918_at	7.21e-46	4.07e-47	16.281.908
221050_s_at	7.34e-46	4.15e-47	16.279.995
205695_at	8.37e-46	4.73e-47	-16.267.214
214435_x_at	8.73e-46	4.94e-47	1.626.301
208868_s_at	9.38e-46	5.32e-47	-1.625.591
212923_s_at	1.01e-45	5.74e-47	-16.248.463
205097_at	1.02e-45	5.77e-47	16.247.865
211557_x_at	1.03e-45	5.87e-47	-16.246.178
205943_at	1.05e-45	5.94e-47	-16.245.002
203373_at	1.15e-45	6.56e-47	-1.623.537
215084_s_at	1.17e-45	6.69e-47	16.233.453
218732_at	1.21e-45	6.90e-47	16.230.458
204866_at	1.22e-45	6.95e-47	16.229.725
210013_at	1.22e-45	6.97e-47	-16.229.436
203562_at	1.35e-45	7.69e-47	-16.219.875
209066_x_at	1.36e-45	7.76e-47	1.621.893
211714_x_at	1.41e-45	8.09e-47	16.214.949
203302_at	1.43e-45	8.21e-47	16.213.421

206119_at	1.44e-45	8.23e-47	-16.213.192
201441_at	1.45e-45	8.31e-47	16.212.316
203462_x_at	1.48e-45	8.48e-47	16.210.347
205326_at	1.75e-45	1.01e-46	-16.193.546
219433_at	1.75e-45	1.01e-46	16.193.468
206955_at	1.77e-45	1.02e-46	-16.192.282
222145_at	1.85e-45	1.07e-46	-16.188
210125_s_at	1.89e-45	1.09e-46	16.185.913
204223_at	1.92e-45	1.11e-46	-16.184.049
202382_s_at	1.95e-45	1.13e-46	16.182.656
202238_s_at	2.08e-45	1.20e-46	-16.176.221
206058_at	2.08e-45	1.20e-46	-16.176.013
217737_x_at	2.10e-45	1.22e-46	16.175.148
214494_s_at	2.25e-45	1.31e-46	-16.168.174
209194_at	2.26e-45	1.31e-46	1.616.766
212713_at	2.30e-45	1.34e-46	-1.616.591
201944_at	2.37e-45	1.38e-46	16.163.054
201804_x_at	2.39e-45	1.39e-46	16.161.977
201726_at	2.44e-45	1.42e-46	16.160.033
200007_at	2.59e-45	1.51e-46	16.154.234
211491_at	2.82e-45	1.64e-46	-16.145.883
39763_at	3.08e-45	1.80e-46	-16.137.023
203584_at	3.12e-45	1.82e-46	16.135.618
201565_s_at	3.28e-45	1.91e-46	-16.130.858
211720_x_at	3.34e-45	1.95e-46	16.128.844
201833_at	3.46e-45	2.02e-46	16.125.326
200014_s_at	3.49e-45	2.04e-46	16.124.439
212556_at	3.61e-45	2.11e-46	16.121.167
205690_s_at	3.66e-45	2.14e-46	16.119.642
209841_s_at	3.69e-45	2.16e-46	-16.118.794
211068_x_at	3.92e-45	2.30e-46	16.112.765
63825_at	4.05e-45	2.38e-46	-16.109.575
48030_i_at	4.36e-45	2.56e-46	-16.102.267
215165_x_at	4.38e-45	2.57e-46	16.101.802
203593_at	4.60e-45	2.70e-46	16.096.932
206449_s_at	4.66e-45	2.75e-46	-1.609.544
212592_at	5.27e-45	3.11e-46	-16.083.415
220083_x_at	5.50e-45	3.24e-46	16.079.154
215110_at	5.55e-45	3.27e-46	-16.078.216
210592_s_at	5.69e-45	3.36e-46	-16.075.678
214696_at	6.11e-45	3.61e-46	-16.068.694
221203_s_at	6.77e-45	4.00e-46	16.058.524
218915_at	6.77e-45	4.01e-46	16.058.432
219185_at	6.78e-45	4.02e-46	-16.058.216

215314_at	6.82e-45	4.04e-46	-16.057.585
212268_at	6.88e-45	4.08e-46	16.056.625
201397_at	7.36e-45	4.37e-46	-16.049.961
205155_s_at	7.84e-45	4.66e-46	-16.043.703
209253_at	7.99e-45	4.75e-46	-16.041.783
207332_s_at	8.74e-45	5.20e-46	16.032.904
205339_at	8.81e-45	5.25e-46	16.032.044
216505_x_at	8.91e-45	5.31e-46	16.030.828
212423_at	8.91e-45	5.32e-46	-16.030.778
217834_s_at	8.96e-45	5.35e-46	16.030.148
203830_at	9.03e-45	5.39e-46	16.029.393
206949_s_at	9.45e-45	5.65e-46	16.024.819
214430_at	9.57e-45	5.72e-46	16.023.535
204510_at	1.04e-44	6.20e-46	16.015.736
213380_x_at	1.09e-44	6.53e-46	-16.010.662
201036_s_at	1.11e-44	6.68e-46	-16.008.343
208964_s_at	1.13e-44	6.75e-46	16.007.378
204240_s_at	1.13e-44	6.78e-46	16.006.921
213243_at	1.17e-44	7.01e-46	16.003.617
212973_at	1.20e-44	7.24e-46	16.000.487
211098_x_at	1.20e-44	7.25e-46	1.600.039
201247_at	1.25e-44	7.55e-46	15.996.397
200934_at	1.32e-44	7.94e-46	15.991.494
205404_at	1.37e-44	8.24e-46	-15.987.886
203696_s_at	1.38e-44	8.35e-46	15.986.546
205201_at	1.45e-44	8.75e-46	-15.981.974
218640_s_at	1.46e-44	8.85e-46	15.980.798
201400_at	1.49e-44	8.99e-46	15.979.256
204391_x_at	1.64e-44	9.92e-46	1.596.963
205075_at	1.70e-44	1.03e-45	-15.966.259
200821_at	1.73e-44	1.05e-45	1.596.392
214695_at	1.75e-44	1.06e-45	15.963.052
205913_at	1.84e-44	1.12e-45	-15.957.781
217768_at	1.87e-44	1.13e-45	15.956.483
211733_x_at	1.87e-44	1.14e-45	-15.956.216
207809_s_at	1.90e-44	1.16e-45	15.954.527
202798_at	1.93e-44	1.18e-45	-15.952.967
220755_s_at	2.01e-44	1.22e-45	15.949.083
212021_s_at	2.01e-44	1.22e-45	15.948.984
202505_at	2.14e-44	1.30e-45	15.942.815
217232_x_at	2.17e-44	1.32e-45	-15.941.306
212810_s_at	2.24e-44	1.37e-45	15.938.227
208131_s_at	2.58e-44	1.58e-45	-15.923.987
203075_at	2.66e-44	1.63e-45	15.921.008

209464_at	2.67e-44	1.64e-45	1.592.052
215689_s_at	2.73e-44	1.67e-45	-15.918.462
213168_at	2.74e-44	1.68e-45	1.591.804
205079_s_at	3.04e-44	1.87e-45	-15.907.561
207468_s_at	3.17e-44	1.95e-45	-15.903.587
210428_s_at	3.39e-44	2.08e-45	15.896.955
203564_at	3.39e-44	2.09e-45	15.896.663
213890_x_at	3.44e-44	2.12e-45	15.895.295
207554_x_at	3.64e-44	2.24e-45	-15.889.554
208310_s_at	3.68e-44	2.27e-45	15.888.506
201968_s_at	3.71e-44	2.29e-45	-15.887.649
209132_s_at	3.99e-44	2.47e-45	1.588.033
217013_at	4.01e-44	2.48e-45	-15.879.712
212186_at	4.03e-44	2.49e-45	15.879.262
218069_at	4.08e-44	2.53e-45	15.877.925
208327_at	4.11e-44	2.55e-45	-15.877.122
209508_x_at	4.14e-44	2.56e-45	-1.587.647
211219_s_at	4.20e-44	2.61e-45	-15.874.867
203572_s_at	4.22e-44	2.62e-45	15.874.371
217879_at	4.42e-44	2.74e-45	15.869.808
200658_s_at	4.63e-44	2.88e-45	15.865.077
216640_s_at	4.83e-44	3.00e-45	15.860.943
209484_s_at	4.88e-44	3.03e-45	15.859.929
213617_s_at	4.88e-44	3.04e-45	15.859.678
208794_s_at	4.93e-44	3.08e-45	15.858.602
201033_x_at	5.09e-44	3.17e-45	15.855.525
203967_at	5.10e-44	3.19e-45	15.855.148
218709_s_at	5.21e-44	3.25e-45	15.853.127
214220_s_at	5.25e-44	3.28e-45	15.852.249
203473_at	5.30e-44	3.32e-45	-15.851.169
218688_at	5.39e-44	3.38e-45	-15.849.467
221741_s_at	5.39e-44	3.38e-45	15.849.345
218968_s_at	5.45e-44	3.42e-45	15.848.237
210142_x_at	5.80e-44	3.64e-45	15.842.025
211928_at	5.83e-44	3.66e-45	15.841.529
202596_at	5.90e-44	3.70e-45	15.840.321
201899_s_at	5.99e-44	3.77e-45	15.838.649
200986_at	6.00e-44	3.78e-45	-15.838.406
203606_at	6.05e-44	3.81e-45	15.837.603
202465_at	6.48e-44	4.09e-45	-15.830.704
213261_at	6.61e-44	4.17e-45	-15.828.707
213301_x_at	6.69e-44	4.22e-45	15.827.507
211696_x_at	6.81e-44	4.30e-45	-15.825.615
222158_s_at	6.83e-44	4.32e-45	15.825.268

219258_at	6.95e-44	4.39e-45	15.823.563
210609_s_at	7.11e-44	4.50e-45	15.821.216
205222_at	7.12e-44	4.51e-45	-15.821.075
203355_s_at	7.52e-44	4.77e-45	-15.815.582
217837_s_at	8.37e-44	5.31e-45	15.804.978
213043_s_at	8.55e-44	5.43e-45	15.802.783
213616_at	8.89e-44	5.64e-45	15.798.967
213599_at	9.51e-44	6.04e-45	15.792.254
208641_s_at	9.95e-44	6.33e-45	15.787.721
212132_at	1.01e-43	6.46e-45	15.785.722
220767_at	1.05e-43	6.67e-45	-15.782.504
201237_at	1.06e-43	6.77e-45	15.781.119
217959_s_at	1.14e-43	7.28e-45	15.773.889
215177_s_at	1.17e-43	7.47e-45	15.771.334
209259_s_at	1.20e-43	7.64e-45	15.769.175
209019_s_at	1.20e-43	7.69e-45	-15.768.572
214328_s_at	1.25e-43	7.98e-45	15.764.918
200856_x_at	1.28e-43	8.22e-45	-15.761.936
208856_x_at	1.31e-43	8.37e-45	15.760.189
206976_s_at	1.37e-43	8.78e-45	1.575.553
200618_at	1.37e-43	8.81e-45	15.755.197
51146_at	1.38e-43	8.84e-45	-15.754.847
201754_at	1.45e-43	9.28e-45	15.749.985
217851_s_at	1.45e-43	9.30e-45	1.574.977
205830_at	1.56e-43	1.00e-44	15.742.274
220085_at	1.64e-43	1.05e-44	15.737.548
212402_at	1.66e-43	1.07e-44	-15.735.964
219666_at	1.80e-43	1.16e-44	-15.728.072
218543_s_at	1.80e-43	1.16e-44	15.727.843
208394_x_at	1.85e-43	1.19e-44	15.725.136
218257_s_at	1.87e-43	1.21e-44	15.724.132
218343_s_at	1.87e-43	1.21e-44	15.724.122
202396_at	1.90e-43	1.23e-44	15.722.416
212320_at	1.96e-43	1.27e-44	15.719.443
212816_s_at	1.96e-43	1.27e-44	-15.719.207
221008_s_at	2.01e-43	1.30e-44	-15.716.854
208880_s_at	2.11e-43	1.37e-44	15.711.593
203692_s_at	2.19e-43	1.42e-44	1.570.811
205614_x_at	2.32e-43	1.51e-44	-15.702.198
221746_at	2.53e-43	1.64e-44	15.693.698
201569_s_at	2.65e-43	1.72e-44	15.688.985
220032_at	2.68e-43	1.74e-44	-15.687.974
202557_at	2.77e-43	1.80e-44	15.684.539
221677_s_at	2.79e-43	1.82e-44	1.568.368

214283_at	3.03e-43	1.97e-44	15.675.716
216977_x_at	3.07e-43	2.00e-44	15.674.312
200046_at	3.17e-43	2.07e-44	156.709
207874_s_at	3.33e-43	2.17e-44	-15.666.157
203566_s_at	3.51e-43	2.29e-44	-15.660.953
213627_at	3.52e-43	2.31e-44	15.660.408
37022_at	3.65e-43	2.39e-44	-1.565.674
212058_at	3.71e-43	2.43e-44	15.655.195
206385_s_at	3.75e-43	2.46e-44	-15.653.951
53720_at	3.76e-43	2.47e-44	-15.653.668
202187_s_at	3.90e-43	2.56e-44	15.650.109
209644_x_at	4.25e-43	2.79e-44	15.641.514
212846_at	4.30e-43	2.83e-44	1.564.022
216593_s_at	4.30e-43	2.83e-44	15.640.133
221761_at	4.31e-43	2.84e-44	15.640.003
211060_x_at	4.44e-43	2.92e-44	15.636.955
209306_s_at	4.56e-43	3.01e-44	15.634.144
208855_s_at	4.76e-43	3.14e-44	15.630.025
201585_s_at	4.97e-43	3.28e-44	15.625.676
209586_s_at	5.72e-43	3.78e-44	15.611.643
201429_s_at	5.73e-43	3.79e-44	15.611.394
216863_s_at	5.74e-43	3.80e-44	15.611.244
208754_s_at	6.00e-43	3.97e-44	15.606.709
200004_at	6.06e-43	4.01e-44	15.605.776
209596_at	6.32e-43	4.19e-44	-15.601.495
221436_s_at	6.46e-43	4.29e-44	15.599.205
213695_at	6.48e-43	4.30e-44	-15.598.902
218104_at	6.86e-43	4.56e-44	15.593.236
207256_at	6.98e-43	4.64e-44	-15.591.411
204844_at	7.31e-43	4.86e-44	-15.586.839
208852_s_at	7.94e-43	5.28e-44	15.578.583
212240_s_at	8.02e-43	5.34e-44	-15.577.497
201894_s_at	8.35e-43	5.56e-44	15.573.514
211972_x_at	8.46e-43	5.64e-44	15.572.135
218710_at	8.57e-43	5.72e-44	15.570.779
217815_at	8.65e-43	5.78e-44	15.569.787
200966_x_at	8.71e-43	5.82e-44	1.556.905
218314_s_at	8.71e-43	5.83e-44	15.568.954
212072_s_at	8.78e-43	5.87e-44	15.568.125
205345_at	9.78e-43	6.55e-44	15.557.434
202185_at	9.85e-43	6.60e-44	1.555.662
217778_at	1.01e-42	6.75e-44	15.554.462
207832_at	1.03e-42	6.90e-44	-15.552.198
200024_at	1.11e-42	7.43e-44	15.544.985

209085_x_at	1.17e-42	7.85e-44	15.539.443
221691_x_at	1.22e-42	8.22e-44	15.534.933
205141_at	1.28e-42	8.64e-44	-15.529.985
201145_at	1.31e-42	8.82e-44	15.527.996
209536_s_at	1.32e-42	8.91e-44	15.526.954
202233_s_at	1.33e-42	8.98e-44	1.552.616
208093_s_at	1.38e-42	9.31e-44	-15.522.645
203715_at	1.45e-42	9.78e-44	15.517.811
204067_at	1.45e-42	9.81e-44	-1.551.747
205761_s_at	1.53e-42	1.03e-43	15.512.387
217897_at	1.56e-42	1.05e-43	-15.510.358
200726_at	1.59e-42	1.08e-43	15.508.094
204003_s_at	1.63e-42	1.11e-43	15.505.708
203091_at	1.66e-42	1.13e-43	15.503.727
202338_at	1.66e-42	1.13e-43	15.503.638
221483_s_at	1.70e-42	1.15e-43	15.501.371
209116_x_at	1.79e-42	1.22e-43	-15.496.189
217850_at	1.83e-42	1.24e-43	15.494.149
202265_at	1.89e-42	1.28e-43	15.490.814
218966_at	1.99e-42	1.35e-43	15.485.814
203571_s_at	2.00e-42	1.36e-43	-15.485.301
221868_at	2.03e-42	1.39e-43	-15.483.292
221830_at	2.06e-42	1.41e-43	15.481.928
203820_s_at	2.14e-42	1.46e-43	15.478.305
202268_s_at	2.15e-42	1.47e-43	15.477.792
208955_at	2.20e-42	1.50e-43	154.755
203790_s_at	2.21e-42	1.51e-43	-15.474.661
209135_at	2.28e-42	1.56e-43	15.471.854
214687_x_at	2.28e-42	1.56e-43	15.471.591
221758_at	2.38e-42	1.63e-43	-1.546.714
219231_at	2.39e-42	1.64e-43	15.466.743
208056_s_at	2.43e-42	1.67e-43	-15.465.095
221647_s_at	2.49e-42	1.71e-43	15.462.501
202141_s_at	2.52e-42	1.73e-43	15.461.446
213101_s_at	2.59e-42	1.78e-43	15.458.703
201554_x_at	2.64e-42	1.81e-43	15.456.718
213405_at	2.76e-42	1.90e-43	15.452.097
202862_at	2.84e-42	1.95e-43	-15.449.423
200610_s_at	2.84e-42	1.96e-43	15.449.235
206101_at	2.97e-42	2.05e-43	-15.444.633
204589_at	3.05e-42	2.11e-43	15.441.924
208864_s_at	3.09e-42	2.14e-43	1.544.052
205849_s_at	3.15e-42	2.18e-43	15.438.714
219192_at	3.17e-42	2.19e-43	15.438.068

213027_at	3.26e-42	2.25e-43	15.435.208
204608_at	3.36e-42	2.32e-43	-15.432.145
218184_at	3.39e-42	2.35e-43	1.543.104
210680_s_at	3.50e-42	2.42e-43	-15.428.057
212092_at	3.82e-42	2.65e-43	15.419.253
207977_s_at	3.99e-42	2.77e-43	-15.414.924
218189_s_at	4.00e-42	2.78e-43	15.414.448
221572_s_at	4.10e-42	2.85e-43	15.412.004
200701_at	4.26e-42	2.96e-43	1.540.829
218699_at	4.48e-42	3.12e-43	15.403.095
213572_s_at	4.53e-42	3.15e-43	1.540.195
218831_s_at	4.55e-42	3.17e-43	-15.401.482
202381_at	4.91e-42	3.42e-43	15.393.805
216320_x_at	5.22e-42	3.64e-43	-15.387.662
200016_x_at	5.36e-42	3.74e-43	15.385.127
214097_at	5.44e-42	3.80e-43	15.383.541
208778_s_at	5.70e-42	3.98e-43	15.378.798
206074_s_at	5.78e-42	4.04e-43	15.377.449
44783_s_at	5.78e-42	4.05e-43	15.377.225
211682_x_at	5.81e-42	4.07e-43	-15.376.774
209607_x_at	5.89e-42	4.13e-43	-15.375.258
205012_s_at	6.04e-42	4.23e-43	-1.537.276
221839_s_at	6.43e-42	4.51e-43	15.366.547
207396_s_at	6.46e-42	4.53e-43	15.366.007
201818_at	6.48e-42	4.55e-43	15.365.568
201807_at	6.99e-42	4.91e-43	15.358.096
201475_x_at	7.32e-42	5.14e-43	15.353.449
211764_s_at	7.65e-42	5.38e-43	15.348.983
202908_at	7.81e-42	5.50e-43	15.346.785
212270_x_at	7.85e-42	5.53e-43	15.346.307
217836_s_at	7.91e-42	5.58e-43	15.345.418
202237_at	8.26e-42	5.82e-43	-15.341.145
218514_at	8.33e-42	5.88e-43	15.340.178
220037_s_at	8.51e-42	6.01e-43	-15.338.044
212474_at	9.31e-42	6.58e-43	1.532.904
219913_s_at	9.39e-42	6.64e-43	15.328.168
216457_s_at	9.68e-42	6.85e-43	15.325.063
206809_s_at	9.73e-42	6.89e-43	15.324.498
201618_x_at	1.05e-41	7.45e-43	15.316.763
201138_s_at	1.10e-41	7.82e-43	15.311.881
200741_s_at	1.11e-41	7.85e-43	1.531.154
219083_at	1.14e-41	8.08e-43	1.530.872
221826_at	1.14e-41	8.10e-43	15.308.488
213306_at	1.17e-41	8.35e-43	-15.305.437

218045_x_at	1.22e-41	8.66e-43	-15.301.833
202757_at	1.24e-41	8.83e-43	152.999
201549_x_at	1.28e-41	9.11e-43	15.296.769
220224_at	1.31e-41	9.36e-43	-15.294.049
209976_s_at	1.50e-41	1.07e-42	-15.280.545
202461_at	1.74e-41	1.24e-42	15.265.806
212807_s_at	1.78e-41	1.27e-42	15.263.832
201892_s_at	1.78e-41	1.27e-42	15.263.666
212147_at	1.80e-41	1.29e-42	15.262.587
221230_s_at	1.87e-41	1.34e-42	15.258.551
218136_s_at	1.90e-41	1.36e-42	-15.256.821
212165_at	1.92e-41	1.38e-42	15.255.635
209657_s_at	2.00e-41	1.43e-42	15.251.774
218976_at	2.01e-41	1.44e-42	-15.251.231
207537_at	2.14e-41	1.53e-42	-15.245.042
216223_at	2.17e-41	1.56e-42	-15.243.584
208674_x_at	2.31e-41	1.66e-42	1.523.733
222037_at	2.31e-41	1.66e-42	15.237.116
201049_s_at	2.34e-41	1.68e-42	15.235.803
202701_at	2.50e-41	1.80e-42	-15.229.223
212696_s_at	2.66e-41	1.92e-42	15.222.844
210272_at	2.67e-41	1.93e-42	-15.222.449
200607_s_at	2.75e-41	1.98e-42	15.219.503
202939_at	3.32e-41	2.40e-42	1.520.065
200950_at	3.37e-41	2.43e-42	15.199.102
208206_s_at	3.41e-41	2.47e-42	-15.197.835
202340_x_at	3.73e-41	2.69e-42	-15.189.013
202811_at	3.74e-41	2.70e-42	15.188.724
208660_at	3.75e-41	2.72e-42	15.188.176
208934_s_at	3.83e-41	2.78e-42	15.186.083
203594_at	4.05e-41	2.94e-42	15.180.501
201786_s_at	4.35e-41	3.15e-42	15.173.351
209305_s_at	4.45e-41	3.23e-42	-15.170.959
201471_s_at	4.90e-41	3.56e-42	15.161.377
221514_at	4.96e-41	3.60e-42	15.160.088
207121_s_at	5.30e-41	3.85e-42	151.535
217504_at	5.76e-41	4.19e-42	-15.145.171
208788_at	5.97e-41	4.34e-42	15.141.521
219990_at	5.97e-41	4.35e-42	15.141.435
209455_at	6.10e-41	4.44e-42	1.513.924
200723_s_at	6.20e-41	4.52e-42	15.137.523
208684_at	6.51e-41	4.75e-42	15.132.542
212537_x_at	6.64e-41	4.85e-42	15.130.504
220060_s_at	6.95e-41	5.08e-42	15.125.972

222105_s_at	8.01e-41	5.85e-42	15.111.775
202212_at	8.01e-41	5.86e-42	15.111.624
219299_at	8.01e-41	5.86e-42	15.111.616
222357_at	8.13e-41	5.95e-42	-15.110.149
219649_at	8.74e-41	6.41e-42	15.102.825
221584_s_at	8.77e-41	6.43e-42	-15.102.425
203025_at	9.17e-41	6.73e-42	15.097.898
222128_at	9.21e-41	6.76e-42	-15.097.428
218181_s_at	9.39e-41	6.90e-42	15.095.444
209627_s_at	9.48e-41	6.97e-42	15.094.455
218783_at	9.69e-41	7.13e-42	15.092.142
209026_x_at	9.78e-41	7.20e-42	15.091.178
210293_s_at	9.93e-41	7.31e-42	15.089.615
208086_s_at	9.96e-41	7.34e-42	-15.089.205
222372_at	1.01e-40	7.42e-42	-15.088.171
202845_s_at	1.02e-40	7.53e-42	1.508.676
217840_at	1.04e-40	7.68e-42	15.084.702
207007_at	1.08e-40	7.96e-42	-15.081.119
44120_at	1.11e-40	8.23e-42	1.507.786
212828_at	1.12e-40	8.32e-42	15.076.761
212133_at	1.18e-40	8.75e-42	15.071.755
203799_at	1.19e-40	8.79e-42	-15.071.267
215322_at	1.19e-40	8.82e-42	-15.070.953
201339_s_at	1.20e-40	8.93e-42	-15.069.678
201652_at	1.25e-40	9.28e-42	15.065.915
207667_s_at	1.31e-40	9.69e-42	-15.061.573
204615_x_at	1.31e-40	9.72e-42	15.061.225
200675_at	1.32e-40	9.78e-42	-15.060.658
205284_at	1.34e-40	9.96e-42	15.058.773
204781_s_at	1.36e-40	1.01e-41	-15.057.018
206738_at	1.49e-40	1.11e-41	-15.048.031
212929_s_at	1.57e-40	1.17e-41	15.043.011
208640_at	1.57e-40	1.17e-41	15.042.507
207945_s_at	1.59e-40	1.18e-41	1.504.164
214837_at	1.72e-40	1.29e-41	-15.033.285
210115_at	1.74e-40	1.30e-41	15.032.287
215690_x_at	1.78e-40	1.33e-41	15.029.817
201240_s_at	1.82e-40	1.36e-41	15.027.569
202241_at	1.85e-40	1.39e-41	-15.025.921
33323_r_at	2.02e-40	1.51e-41	15.017.323
214235_at	2.12e-40	1.59e-41	-15.012.438
203410_at	2.15e-40	1.61e-41	15.010.876
208881_x_at	2.25e-40	1.69e-41	15.006.087
203968_s_at	2.33e-40	1.75e-41	15.002.744

209482_at	2.38e-40	1.79e-41	15.000.442
209703_x_at	2.40e-40	1.80e-41	-14.999.514
218037_at	2.42e-40	1.82e-41	1.499.885
202602_s_at	2.53e-40	1.90e-41	14.994.121
200990_at	2.54e-40	1.91e-41	1.499.369
200764_s_at	2.71e-40	2.04e-41	14.987.089
222382_x_at	2.72e-40	2.05e-41	14.986.858
204442_x_at	2.75e-40	2.08e-41	-14.985.517
212997_s_at	2.83e-40	2.14e-41	14.982.648
208650_s_at	2.92e-40	2.21e-41	14.979.351
211016_x_at	2.97e-40	2.24e-41	14.977.701
202421_at	3.27e-40	2.47e-41	14.967.963
219521_at	3.45e-40	2.61e-41	-14.962.794
204459_at	3.62e-40	2.74e-41	14.957.862
217647_at	3.86e-40	2.92e-41	-14.951.347
201273_s_at	4.01e-40	3.04e-41	14.947.387
200888_s_at	4.34e-40	3.29e-41	1.493.947
200027_at	4.52e-40	3.43e-41	1.493.541
204649_at	4.58e-40	3.47e-41	1.493.409
200082_s_at	4.62e-40	3.51e-41	14.933.003
221992_at	4.65e-40	3.53e-41	-14.932.411
200812_at	4.71e-40	3.58e-41	14.930.979
200043_at	4.89e-40	3.72e-41	14.927.111
1431_at	5.11e-40	3.89e-41	-14.922.785
217256_x_at	5.26e-40	4.00e-41	14.919.875
218041_x_at	5.48e-40	4.17e-41	-1.491.567
220858_at	5.79e-40	4.42e-41	-14.910.067
220924_s_at	5.80e-40	4.43e-41	-14.909.812
218852_at	6.14e-40	4.68e-41	14.904.142
218911_at	6.17e-40	4.72e-41	14.903.465
218611_at	6.62e-40	5.06e-41	14.896.391
207837_at	6.70e-40	5.13e-41	-14.895.151
201948_at	6.82e-40	5.22e-41	1.489.333
218829_s_at	6.85e-40	5.25e-41	148.928
200996_at	6.87e-40	5.26e-41	14.892.481
202913_at	6.93e-40	5.31e-41	14.891.526
219979_s_at	7.12e-40	5.46e-41	14.888.803
218331_s_at	7.31e-40	5.61e-41	14.886.116
208638_at	7.36e-40	5.65e-41	14.885.381
208724_s_at	7.43e-40	5.71e-41	14.884.321
200913_at	7.48e-40	5.75e-41	14.883.653
209654_at	7.62e-40	5.86e-41	14.881.748
221482_s_at	7.70e-40	5.92e-41	14.880.661
204559_s_at	7.97e-40	6.14e-41	14.877.063

200994_at	8.34e-40	6.42e-41	14.872.547
217884_at	8.72e-40	6.72e-41	14.867.975
212800_at	8.84e-40	6.82e-41	14.866.567
201139_s_at	9.00e-40	6.95e-41	14.864.706
203758_at	9.03e-40	6.98e-41	-14.864.289
209123_at	9.20e-40	7.11e-41	-14.862.361
220554_at	9.24e-40	7.15e-41	-14.861.834
200031_s_at	9.37e-40	7.25e-41	14.860.451
202724_s_at	9.38e-40	7.26e-41	-14.860.304
218334_at	9.73e-40	7.54e-41	14.856.516
205876_at	9.85e-40	7.64e-41	-14.855.205
205234_at	9.96e-40	7.73e-41	-14.854.056
203418_at	1.01e-39	7.81e-41	14.852.981
219880_at	1.03e-39	7.97e-41	1.485.093
203909_at	1.06e-39	8.22e-41	14.847.805
218423_x_at	1.10e-39	8.55e-41	14.843.872
211202_s_at	1.11e-39	8.60e-41	14.843.357
211494_s_at	1.14e-39	8.89e-41	-14.839.999
209796_s_at	1.17e-39	9.08e-41	14.837.864
204288_s_at	1.17e-39	9.13e-41	-14.837.353
203743_s_at	1.18e-39	9.18e-41	14.836.782
208736_at	1.26e-39	9.83e-41	14.829.904
201473_at	1.26e-39	9.84e-41	-1.482.983
202604_x_at	1.28e-39	9.99e-41	14.828.348
218698_at	1.30e-39	1.02e-40	14.826.477
202946_s_at	1.31e-39	1.02e-40	14.825.782
209817_at	1.31e-39	1.03e-40	14.825.594
203013_at	1.37e-39	1.07e-40	14.821.215
207255_at	1.45e-39	1.14e-40	-14.815.271
219324_at	1.46e-39	1.15e-40	14.814.604
203034_s_at	1.48e-39	1.16e-40	14.813.128
218689_at	1.54e-39	1.21e-40	1.480.942
222369_at	1.56e-39	1.23e-40	14.807.746
205221_at	1.69e-39	1.33e-40	-1.479.995
205078_at	1.72e-39	1.35e-40	14.798.056
203545_at	1.73e-39	1.36e-40	14.797.305
208897_s_at	1.78e-39	1.40e-40	14.794.176
218598_at	1.87e-39	1.47e-40	14.789.246
222316_at	1.87e-39	1.48e-40	-14.789.199
200631_s_at	1.95e-39	1.54e-40	14.784.976
203344_s_at	2.06e-39	1.63e-40	14.779.418
203739_at	2.09e-39	1.65e-40	14.778.217
207740_s_at	2.14e-39	1.69e-40	14.775.636
217196_s_at	2.16e-39	1.70e-40	14.774.757

201758_at	2.23e-39	1.76e-40	14.771.316
219797_at	2.32e-39	1.84e-40	14.767.266
212635_at	2.35e-39	1.86e-40	14.765.939
200017_at	2.43e-39	1.92e-40	14.762.706
202567_at	2.46e-39	1.94e-40	14.761.481
200038_s_at	2.64e-39	2.09e-40	14.754.088
212023_s_at	2.70e-39	2.14e-40	14.752.001
200872_at	2.75e-39	2.18e-40	14.750.034
212543_at	2.80e-39	2.23e-40	-14.747.964
209656_s_at	2.86e-39	2.27e-40	-14.745.877
218542_at	3.06e-39	2.44e-40	14.738.882
208954_s_at	3.27e-39	2.60e-40	14.732.175
208999_at	3.30e-39	2.63e-40	14.731.181
217998_at	3.33e-39	2.65e-40	-14.730.333
205826_at	3.52e-39	2.80e-40	-14.724.845
221920_s_at	3.60e-39	2.87e-40	-14.722.489
200998_s_at	3.71e-39	2.96e-40	14.719.447
208912_s_at	3.75e-39	2.99e-40	14.718.178
202427_s_at	3.76e-39	3.01e-40	14.717.754
204720_s_at	4.07e-39	3.25e-40	1.470.991
219541_at	4.43e-39	3.54e-40	-14.701.253
208715_at	4.65e-39	3.72e-40	1.469.626
208876_s_at	4.65e-39	3.72e-40	14.696.242
218247_s_at	4.68e-39	3.75e-40	14.695.572
216088_s_at	4.80e-39	3.84e-40	14.693.055
203098_at	4.95e-39	3.97e-40	146.898
214584_x_at	4.97e-39	3.98e-40	-14.689.473
203428_s_at	5.17e-39	4.15e-40	14.685.432
213246_at	5.25e-39	4.22e-40	14.683.743
203612_at	5.52e-39	4.43e-40	14.678.706
218653_at	5.67e-39	4.56e-40	-14.675.959
204460_s_at	5.90e-39	4.75e-40	1.467.182
203774_at	6.12e-39	4.93e-40	14.668.049
201491_at	6.36e-39	5.12e-40	14.664.257
211729_x_at	6.59e-39	5.31e-40	14.660.626
204835_at	6.63e-39	5.35e-40	14.659.852
221931_s_at	6.73e-39	5.42e-40	14.658.397
210896_s_at	6.85e-39	5.53e-40	14.656.539
215221_at	7.08e-39	5.72e-40	-14.653.102
203164_at	7.38e-39	5.96e-40	14.648.892
208818_s_at	8.19e-39	6.62e-40	-14.638.382
200080_s_at	8.27e-39	6.69e-40	14.637.338
212773_s_at	8.29e-39	6.71e-40	14.637.041
202097_at	8.61e-39	6.97e-40	14.633.182

219512_at	8.83e-39	7.15e-40	14.630.572
203819_s_at	9.27e-39	7.51e-40	14.625.634
210527_x_at	9.47e-39	7.68e-40	14.623.417
219035_s_at	9.62e-39	7.81e-40	14.621.742
212042_x_at	9.84e-39	7.99e-40	146.194
219108_x_at	9.99e-39	8.12e-40	14.617.816
207409_at	1.03e-38	8.33e-40	-14.615.182
210521_s_at	1.04e-38	8.50e-40	-14.613.213
203455_s_at	1.05e-38	8.52e-40	-14.612.988
219934_s_at	1.06e-38	8.62e-40	-14.611.819
200909_s_at	1.06e-38	8.62e-40	14.611.759
208682_s_at	1.07e-38	8.76e-40	1.461.014
201886_at	1.08e-38	8.81e-40	-1.460.957
201328_at	1.14e-38	9.30e-40	-14.604.077
208126_s_at	1.14e-38	9.32e-40	-14.603.864
218924_s_at	1.22e-38	9.95e-40	-14.597.271
201398_s_at	1.23e-38	1.01e-39	14.595.931
202189_x_at	1.24e-38	1.02e-39	14.595.059
210580_x_at	1.27e-38	1.04e-39	-14.592.997
201676_x_at	1.28e-38	1.05e-39	14.591.892
203956_at	1.35e-38	1.11e-39	14.586.422
203630_s_at	1.37e-38	1.13e-39	14.584.886
213119_at	1.37e-38	1.13e-39	14.584.732
204349_at	1.43e-38	1.17e-39	1.458.066
201001_s_at	1.44e-38	1.18e-39	14.580.157
218140_x_at	1.44e-38	1.19e-39	14.579.637
213448_at	1.53e-38	1.25e-39	14.574.032
218298_s_at	1.59e-38	1.31e-39	-14.569.732
212725_s_at	1.60e-38	1.32e-39	14.569.161
209186_at	1.60e-38	1.32e-39	14.569.096
201657_at	1.63e-38	1.34e-39	14.567.377
218956_s_at	1.65e-38	1.36e-39	14.565.704
217846_at	1.81e-38	1.49e-39	14.556.497
205107_s_at	1.86e-38	1.53e-39	14.553.659
208747_s_at	1.89e-38	1.56e-39	-14.551.977
201629_s_at	1.95e-38	1.61e-39	14.548.689
211746_x_at	2.02e-38	1.67e-39	14.545.297
204046_at	2.05e-38	1.70e-39	-14.543.556
210653_s_at	2.13e-38	1.76e-39	-14.539.563
208893_s_at	2.16e-38	1.78e-39	-14.538.397
208922_s_at	2.20e-38	1.82e-39	-14.536.534
219007_at	2.26e-38	1.88e-39	14.533.347
218209_s_at	2.29e-38	1.90e-39	14.532.276
58696_at	2.35e-38	1.95e-39	14.529.418

211038_s_at	2.37e-38	1.97e-39	-14.528.529
211715_s_at	2.39e-38	1.99e-39	-14.527.584
204521_at	2.43e-38	2.02e-39	14.525.995
201575_at	2.46e-38	2.04e-39	14.524.774
208921_s_at	2.58e-38	2.14e-39	14.519.979
210023_s_at	2.58e-38	2.15e-39	14.519.578
204839_at	2.62e-38	2.18e-39	14.518.016
211270_x_at	2.71e-38	2.26e-39	14.514.706
218721_s_at	2.88e-38	2.40e-39	14.508.308
200773_x_at	3.20e-38	2.67e-39	14.497.844
208824_x_at	3.60e-38	3.00e-39	14.485.893
220161_s_at	3.60e-38	3.01e-39	-14.485.715
209509_s_at	3.61e-38	3.01e-39	144.855
218901_at	3.78e-38	3.16e-39	-14.480.669
217900_at	3.80e-38	3.18e-39	14.480.076
218847_at	3.94e-38	3.30e-39	14.476.396
212568_s_at	4.24e-38	3.55e-39	14.468.922
217976_s_at	4.30e-38	3.60e-39	1.446.741
207098_s_at	4.31e-38	3.61e-39	14.467.173
206542_s_at	4.36e-38	3.66e-39	-14.465.873
202576_s_at	4.42e-38	3.70e-39	-14.464.625
203881_s_at	4.61e-38	3.87e-39	-14.460.236
203466_at	4.77e-38	4.01e-39	14.456.727
201517_at	4.92e-38	4.14e-39	1.445.346
205930_at	5.01e-38	4.22e-39	14.451.561
211662_s_at	5.15e-38	4.33e-39	14.448.877
208817_at	5.23e-38	4.41e-39	-1.444.711
208739_x_at	5.39e-38	4.54e-39	14.444.155
218567_x_at	5.72e-38	4.82e-39	14.438.022
212050_at	6.01e-38	5.07e-39	14.432.917
213056_at	6.02e-38	5.08e-39	-14.432.766
209374_s_at	6.15e-38	5.19e-39	-14.430.475
201699_at	6.18e-38	5.22e-39	14.429.972
212334_at	6.67e-38	5.64e-39	14.422.208
214264_s_at	6.68e-38	5.64e-39	14.422.073
218988_at	6.84e-38	5.79e-39	14.419.537
221560_at	7.39e-38	6.26e-39	14.411.637
215103_at	7.68e-38	6.50e-39	-14.407.793
215870_s_at	8.08e-38	6.85e-39	-14.402.504
58900_at	8.33e-38	7.06e-39	-14.399.381
202852_s_at	9.24e-38	7.83e-39	14.388.846
218973_at	9.33e-38	7.92e-39	14.387.806
215754_at	9.33e-38	7.92e-39	-14.387.774
209748_at	9.58e-38	8.13e-39	14.385.046

221727_at	1.01e-37	8.56e-39	14.379.901
219219_at	1.02e-37	8.64e-39	14.378.972
211922_s_at	1.03e-37	8.74e-39	-14.377.783
213733_at	1.10e-37	9.34e-39	-14.371.081
203642_s_at	1.13e-37	9.62e-39	-14.368.014
221229_s_at	1.14e-37	9.73e-39	1.436.691
208875_s_at	1.15e-37	9.80e-39	14.366.202
221494_x_at	1.19e-37	1.01e-38	14.362.824
219737_s_at	1.20e-37	1.03e-38	-14.361.621
216323_x_at	1.34e-37	1.15e-38	14.350.143
207660_at	1.37e-37	1.17e-38	-1.434.824
205568_at	1.43e-37	1.22e-38	-14.343.938
213206_at	1.47e-37	1.25e-38	1.434.114
205429_s_at	1.48e-37	1.26e-38	14.340.305
207330_at	1.50e-37	1.28e-38	-14.338.816
221556_at	1.52e-37	1.30e-38	-14.337.249
208854_s_at	1.52e-37	1.30e-38	14.337.157
209980_s_at	1.53e-37	1.31e-38	-14.336.653
211684_s_at	1.53e-37	1.31e-38	143.366
217726_at	1.53e-37	1.31e-38	14.336.492
212429_s_at	1.57e-37	1.35e-38	14.333.975
210326_at	1.64e-37	1.41e-38	-14.329.639
221984_s_at	1.64e-37	1.41e-38	14.329.574
211971_s_at	1.83e-37	1.57e-38	14.318.353
208753_s_at	1.94e-37	1.67e-38	1.431.222
209301_at	1.99e-37	1.71e-38	-14.309.503
221058_s_at	2.14e-37	1.85e-38	14.301.994
200048_s_at	2.14e-37	1.85e-38	14.301.981
200045_at	2.33e-37	2.01e-38	14.293.442
201385_at	2.38e-37	2.06e-38	1.429.098
208780_x_at	2.38e-37	2.06e-38	14.290.896
212332_at	2.44e-37	2.11e-38	-142.885
203415_at	2.56e-37	2.21e-38	14.283.714
218631_at	2.69e-37	2.33e-38	-14.278.367
218270_at	2.71e-37	2.35e-38	14.277.612
202298_at	2.97e-37	2.57e-38	14.268.422
220968_s_at	3.00e-37	2.60e-38	-14.267.316
213473_at	3.00e-37	2.60e-38	14.267.294
209064_x_at	3.17e-37	2.75e-38	1.426.142
218843_at	3.19e-37	2.77e-38	-14.260.768
218836_at	3.22e-37	2.80e-38	14.259.739
202815_s_at	3.42e-37	2.97e-38	-14.253.717
202658_at	3.42e-37	2.97e-38	14.253.532
208763_s_at	3.49e-37	3.03e-38	-14.251.531

213671_s_at	3.81e-37	3.31e-38	14.242.622
210927_x_at	4.08e-37	3.55e-38	14.235.612
203648_at	4.26e-37	3.71e-38	1.423.116
220691_at	4.38e-37	3.82e-38	-14.228.175
201662_s_at	4.43e-37	3.86e-38	14.227.076
206259_at	4.43e-37	3.86e-38	-14.227.028
202959_at	4.43e-37	3.86e-38	-14.226.965
209916_at	4.45e-37	3.88e-38	-14.226.404
210211_s_at	4.52e-37	3.94e-38	14.224.852
202915_s_at	4.65e-37	4.06e-38	14.221.949
216600_x_at	4.68e-37	4.09e-38	-14.221.113
201980_s_at	4.73e-37	4.13e-38	14.220.095
208609_s_at	4.87e-37	4.26e-38	-14.217.075
221662_s_at	4.87e-37	4.26e-38	-14.217.017
206944_at	5.07e-37	4.44e-38	-14.212.734
205086_s_at	5.14e-37	4.50e-38	14.211.435
219478_at	5.24e-37	4.59e-38	-14.209.385
219269_at	5.28e-37	4.63e-38	-14.208.452
221809_at	5.82e-37	5.11e-38	-14.198.501
202723_s_at	5.87e-37	5.15e-38	-14.197.639
209449_at	5.96e-37	5.23e-38	1.419.603
210501_x_at	6.28e-37	5.52e-38	14.190.684
205675_at	6.55e-37	5.76e-38	-1.418.623
203499_at	7.29e-37	6.42e-38	-14.175.306
204699_s_at	7.60e-37	6.69e-38	14.171.045
202555_s_at	7.66e-37	6.74e-38	-14.170.277
208127_s_at	8.05e-37	7.09e-38	1.416.507
215659_at	8.69e-37	7.66e-38	-1.415.729
205757_at	8.72e-37	7.69e-38	-14.156.813
212419_at	9.41e-37	8.30e-38	-14.149.081
202830_s_at	9.63e-37	8.50e-38	-14.146.602
219980_at	1.02e-36	8.98e-38	-14.141.113
219200_at	1.04e-36	9.21e-38	14.138.448
201262_s_at	1.04e-36	9.23e-38	-14.138.288
214766_s_at	1.06e-36	9.35e-38	14.137.001
211531_x_at	1.06e-36	9.37e-38	-14.136.715
202748_at	1.07e-36	9.43e-38	14.136.062
208697_s_at	1.08e-36	9.57e-38	14.134.554
200790_at	1.08e-36	9.60e-38	14.134.221
217791_s_at	1.10e-36	9.75e-38	14.132.711
211717_at	1.10e-36	9.77e-38	14.132.438
218568_at	1.11e-36	9.81e-38	14.132.044
218523_at	1.14e-36	1.01e-37	-14.129.217
201840_at	1.21e-36	1.07e-37	14.123.035

221791_s_at	1.21e-36	1.08e-37	14.122.613
211987_at	1.21e-36	1.08e-37	14.122.192
202967_at	1.22e-36	1.09e-37	1.412.143
219118_at	1.31e-36	1.17e-37	14.114.221
200807_s_at	1.32e-36	1.18e-37	14.113.526
201558_at	1.40e-36	1.25e-37	14.107.243
206515_at	1.41e-36	1.26e-37	-14.106.819
218082_s_at	1.47e-36	1.31e-37	14.102.504
200718_s_at	1.56e-36	1.39e-37	14.096.527
206995_x_at	1.57e-36	1.40e-37	-14.095.433
51176_at	1.61e-36	1.44e-37	1.409.288
218670_at	1.65e-36	1.48e-37	14.090.186
204752_x_at	1.70e-36	1.52e-37	14.087.189
219544_at	1.75e-36	1.57e-37	14.084.062
213632_at	1.79e-36	1.60e-37	-14.082.134
210434_x_at	1.80e-36	1.61e-37	1.408.128
209406_at	1.81e-36	1.62e-37	14.080.609
218903_s_at	2.07e-36	1.85e-37	14.067.113
213391_at	2.11e-36	1.90e-37	14.064.751
208114_s_at	2.18e-36	1.95e-37	14.061.718
209027_s_at	2.21e-36	1.99e-37	14.060.121
202372_at	2.41e-36	2.16e-37	14.051.299
208749_x_at	2.42e-36	2.18e-37	14.050.721
218081_at	2.50e-36	2.25e-37	14.047.492
221520_s_at	2.58e-36	2.32e-37	14.044.203
222122_s_at	2.64e-36	2.38e-37	14.041.647
208510_s_at	2.86e-36	2.57e-37	14.033.634
221497_x_at	2.86e-36	2.58e-37	14.033.438
203139_at	2.88e-36	2.60e-37	-14.032.753
202726_at	2.90e-36	2.62e-37	14.031.863
203088_at	2.91e-36	2.63e-37	-14.031.479
211483_x_at	2.92e-36	2.64e-37	-14.031.125
201351_s_at	2.93e-36	2.65e-37	14.030.715
207239_s_at	2.93e-36	2.65e-37	14.030.673
202352_s_at	3.02e-36	2.73e-37	1.402.754
204299_at	3.06e-36	2.77e-37	14.026.202
209250_at	3.12e-36	2.82e-37	14.024.179
218028_at	3.16e-36	2.86e-37	14.022.732
214221_at	3.26e-36	2.95e-37	14.019.549
208113_x_at	3.27e-36	2.96e-37	14.019.145
214426_x_at	3.36e-36	3.05e-37	14.016.371
217092_x_at	3.36e-36	3.05e-37	14.016.179
212689_s_at	3.37e-36	3.06e-37	14.015.764
204675_at	3.45e-36	3.13e-37	-14.013.432

208713_at	3.49e-36	3.17e-37	14.012.357
219988_s_at	3.65e-36	3.32e-37	14.007.517
212742_at	3.70e-36	3.37e-37	14.006.105
215773_x_at	3.85e-36	3.51e-37	14.001.949
204160_s_at	3.90e-36	3.55e-37	14.000.583
205343_at	4.14e-36	3.77e-37	13.994.476
209442_x_at	4.17e-36	3.81e-37	-13.993.609
217956_s_at	4.28e-36	3.91e-37	13.990.937
209679_s_at	4.34e-36	3.96e-37	13.989.509
200740_s_at	4.38e-36	4.00e-37	1.398.843
215920_s_at	4.43e-36	4.04e-37	-13.987.391
202474_s_at	4.59e-36	4.19e-37	13.983.648
220307_at	4.68e-36	4.28e-37	-13.981.559
221804_s_at	4.95e-36	4.53e-37	13.975.741
211208_s_at	4.99e-36	4.57e-37	13.974.939
212852_s_at	5.01e-36	4.58e-37	13.974.555
221524_s_at	5.06e-36	4.64e-37	13.973.386
217720_at	5.31e-36	4.86e-37	13.968.518
218859_s_at	5.31e-36	4.87e-37	13.968.439
208963_x_at	5.54e-36	5.08e-37	13.963.962
203565_s_at	5.57e-36	5.11e-37	13.963.404
221815_at	5.58e-36	5.12e-37	-13.963.168
204438_at	5.64e-36	5.18e-37	-13.962.069
212896_at	5.73e-36	5.27e-37	13.960.275
218480_at	6.14e-36	5.64e-37	13.953.268
218613_at	6.34e-36	5.84e-37	-13.949.824
38918_at	6.52e-36	6.01e-37	13.946.902
202193_at	6.52e-36	6.01e-37	13.946.845
209265_s_at	6.92e-36	6.38e-37	13.940.733
206527_at	6.98e-36	6.44e-37	-13.939.772
202706_s_at	7.00e-36	6.46e-37	13.939.472
201407_s_at	7.00e-36	6.46e-37	13.939.369
209545_s_at	7.14e-36	6.59e-37	13.937.308
204477_at	7.22e-36	6.67e-37	13.936.109
213974_at	7.27e-36	6.72e-37	-13.935.402
202214_s_at	7.63e-36	7.06e-37	13.930.357
216306_x_at	8.14e-36	7.53e-37	13.923.663
209397_at	8.20e-36	7.59e-37	13.922.964
219240_s_at	9.00e-36	8.34e-37	13.913.265
207097_s_at	9.01e-36	8.34e-37	-13.913.188
212250_at	9.52e-36	8.82e-37	139.075
202969_at	9.62e-36	8.92e-37	13.906.302
208837_at	9.69e-36	8.99e-37	13.905.505
213513_x_at	1.01e-35	9.33e-37	13.901.679

201277_s_at	1.02e-35	9.47e-37	13.900.162
210852_s_at	1.04e-35	9.66e-37	-13.898.161
216304_x_at	1.06e-35	9.82e-37	13.896.466
206441_s_at	1.11e-35	1.03e-36	13.891.749
41387_r_at	1.15e-35	1.07e-36	-1.388.759
220199_s_at	1.18e-35	1.10e-36	13.885.228
215823_x_at	1.19e-35	1.11e-36	13.884.223
213598_at	1.19e-35	1.11e-36	13.883.638
212922_s_at	1.25e-35	1.17e-36	13.878.553
218392_x_at	1.25e-35	1.17e-36	-13.878.482
220335_x_at	1.27e-35	1.19e-36	-13.877.111
212281_s_at	1.28e-35	1.19e-36	1.387.662
201098_at	1.29e-35	1.20e-36	13.875.808
206606_at	1.31e-35	1.23e-36	-13.873.584
211744_s_at	1.31e-35	1.23e-36	13.873.454
201456_s_at	1.34e-35	1.26e-36	13.871.277
205821_at	1.44e-35	1.35e-36	-13.863.992
202857_at	1.46e-35	1.37e-36	1.386.219
219178_at	1.48e-35	1.38e-36	13.861.418
205060_at	1.48e-35	1.38e-36	13.861.378
200076_s_at	1.49e-35	1.40e-36	13.860.012
208777_s_at	1.51e-35	1.42e-36	13.858.618
202998_s_at	1.52e-35	1.43e-36	13.857.909
210573_s_at	1.54e-35	1.45e-36	13.856.679
212803_at	1.56e-35	1.47e-36	-13.855.314
202408_s_at	1.62e-35	1.53e-36	13.851.187
222271_at	1.67e-35	1.57e-36	-13.848.476
203664_s_at	1.70e-35	1.60e-36	13.846.635
203196_at	1.71e-35	1.61e-36	13.845.977
221926_s_at	1.71e-35	1.61e-36	-13.845.873
214359_s_at	1.73e-35	1.63e-36	13.844.486
204165_at	1.75e-35	1.65e-36	13.843.309
201500_s_at	1.78e-35	1.68e-36	13.841.636
218399_s_at	1.82e-35	1.71e-36	13.839.307
216472_at	1.84e-35	1.74e-36	-13.837.885
208975_s_at	1.88e-35	1.78e-36	13.835.579
212627_s_at	1.95e-35	1.84e-36	13.831.969
202332_at	1.98e-35	1.87e-36	13.830.086
222121_at	2.06e-35	1.95e-36	-13.825.925
209252_at	2.09e-35	1.97e-36	13.824.746
203422_at	2.09e-35	1.98e-36	13.824.636
213654_at	2.13e-35	2.02e-36	13.822.371
201976_s_at	2.13e-35	2.02e-36	-13.822.335
210213_s_at	2.15e-35	2.04e-36	13.821.379

200055_at	2.20e-35	2.08e-36	13.819.208
219238_at	2.29e-35	2.17e-36	-13.814.821
218682_s_at	2.32e-35	2.20e-36	13.813.482
201730_s_at	2.32e-35	2.21e-36	13.813.256
209307_at	2.34e-35	2.22e-36	1.381.255
204126_s_at	2.34e-35	2.22e-36	1.381.247
215714_s_at	2.36e-35	2.24e-36	13.811.587
221637_s_at	2.41e-35	2.29e-36	13.809.486
211424_x_at	2.45e-35	2.33e-36	-13.807.699
221605_s_at	2.48e-35	2.37e-36	-13.806.188
201850_at	2.63e-35	2.50e-36	1.380.039
218875_s_at	2.63e-35	2.51e-36	13.800.117
203773_x_at	2.67e-35	2.55e-36	13.798.419
214835_s_at	2.74e-35	2.61e-36	-13.795.869
217722_s_at	2.83e-35	2.70e-36	13.792.607
216908_x_at	2.88e-35	2.75e-36	-13.790.531
203209_at	2.93e-35	2.80e-36	13.788.664
210472_at	2.99e-35	2.86e-36	-13.786.699
212163_at	3.09e-35	2.96e-36	13.783.185
202331_at	3.14e-35	3.00e-36	-137.817
208675_s_at	3.36e-35	3.21e-36	13.774.645
218115_at	3.45e-35	3.31e-36	1.377.171
204407_at	3.48e-35	3.33e-36	13.770.829
207620_s_at	3.49e-35	3.35e-36	13.770.465
203298_s_at	3.55e-35	3.41e-36	13.768.566
206468_s_at	3.60e-35	3.45e-36	13.767.287
204709_s_at	3.70e-35	3.55e-36	13.764.387
204373_s_at	3.82e-35	3.67e-36	13.761.073
200744_s_at	3.85e-35	3.70e-36	13.760.251
204066_s_at	4.10e-35	3.94e-36	13.753.634
61734_at	4.41e-35	4.24e-36	-13.746.076
217761_at	4.45e-35	4.29e-36	-13.744.979
204695_at	4.60e-35	4.43e-36	13.741.611
209933_s_at	4.60e-35	4.43e-36	-13.741.595
200674_s_at	4.69e-35	4.52e-36	13.739.621
220770_s_at	4.78e-35	4.61e-36	13.737.476
219027_s_at	4.78e-35	4.61e-36	-13.737.439
201417_at	4.94e-35	4.76e-36	13.734.075
212088_at	5.10e-35	4.92e-36	-13.730.785
215424_s_at	5.12e-35	4.95e-36	13.730.139
218570_at	5.21e-35	5.03e-36	13.728.392
219462_at	5.47e-35	5.29e-36	-1.372.333
218886_at	5.52e-35	5.34e-36	13.722.335
209543_s_at	5.79e-35	5.60e-36	13.717.393

205733_at	5.83e-35	5.64e-36	13.716.651
219519_s_at	5.92e-35	5.74e-36	-13.714.923
207469_s_at	6.23e-35	6.03e-36	13.709.747
220317_at	6.29e-35	6.09e-36	-13.708.695
212426_s_at	6.41e-35	6.22e-36	13.706.566
217521_at	6.49e-35	6.30e-36	-13.705.246
217727_x_at	6.50e-35	6.31e-36	13.705.164
221573_at	6.52e-35	6.33e-36	13.704.707
208759_at	6.64e-35	6.45e-36	1.370.283
211150_s_at	6.64e-35	6.46e-36	13.702.704
63009_at	7.28e-35	7.08e-36	13.693.224
214308_s_at	7.28e-35	7.08e-36	-13.693.218
201589_at	7.29e-35	7.10e-36	13.692.914
218322_s_at	7.34e-35	7.15e-36	-13.692.287
209348_s_at	7.45e-35	7.26e-36	-13.690.593
218586_at	7.59e-35	7.40e-36	13.688.722
201157_s_at	7.60e-35	7.41e-36	13.688.527
202188_at	8.04e-35	7.85e-36	13.682.622
210597_x_at	8.59e-35	8.39e-36	-13.675.734
202693_s_at	8.67e-35	8.47e-36	13.674.794
202294_at	8.72e-35	8.53e-36	13.674.061
209772_s_at	8.73e-35	8.53e-36	13.673.961
211981_at	8.77e-35	8.58e-36	13.673.371
210334_x_at	9.10e-35	8.91e-36	13.669.559
209975_at	9.29e-35	9.09e-36	-13.667.414
202441_at	9.39e-35	9.20e-36	-13.666.181
220999_s_at	9.60e-35	9.41e-36	-13.663.911
202116_at	9.63e-35	9.44e-36	13.663.541
200755_s_at	9.82e-35	9.63e-36	13.661.516
218717_s_at	9.95e-35	9.76e-36	-13.660.055
201179_s_at	1.00e-34	9.82e-36	13.659.471
209735_at	1.05e-34	1.03e-35	-13.654.125
202166_s_at	1.06e-34	1.04e-35	13.653.278
213711_at	1.09e-34	1.07e-35	-13.650.514
213205_s_at	1.14e-34	1.12e-35	-13.645.725
214075_at	1.16e-34	1.14e-35	13.643.752
200748_s_at	1.16e-34	1.14e-35	13.643.671
208878_s_at	1.19e-34	1.18e-35	13.640.772
207224_s_at	1.20e-34	1.18e-35	-13.640.106
206363_at	1.22e-34	1.20e-35	-13.638.889
200976_s_at	1.25e-34	1.23e-35	13.636.362
33322_i_at	1.27e-34	1.25e-35	13.634.572
202442_at	1.29e-34	1.28e-35	13.632.303
201513_at	1.29e-34	1.28e-35	13.632.204

211977_at	1.31e-34	1.30e-35	13.630.758
204799_at	1.34e-34	1.32e-35	13.628.553
211382_s_at	1.36e-34	1.34e-35	13.627.206
204108_at	1.37e-34	1.35e-35	13.626.363
218292_s_at	1.42e-34	1.41e-35	-13.622.145
208424_s_at	1.44e-34	1.42e-35	13.621.147
214805_at	1.46e-34	1.45e-35	-13.619.226
215772_x_at	1.56e-34	1.54e-35	-13.612.736
214751_at	1.59e-34	1.58e-35	13.610.313
31807_at	1.64e-34	1.63e-35	13.607.359
206353_at	1.65e-34	1.64e-35	-13.606.551
200995_at	1.66e-34	1.65e-35	13.605.649
203095_at	1.67e-34	1.66e-35	13.604.997
209124_at	1.68e-34	1.66e-35	-13.604.901
206066_s_at	1.82e-34	1.81e-35	13.596.178
201504_s_at	1.84e-34	1.83e-35	13.594.961
201636_at	1.93e-34	1.93e-35	13.589.847
222216_s_at	1.98e-34	1.97e-35	13.587.614
200632_s_at	1.99e-34	1.98e-35	13.587
212459_x_at	2.04e-34	2.04e-35	-13.583.976
208374_s_at	2.11e-34	2.10e-35	13.580.867
203076_s_at	2.15e-34	2.14e-35	13.578.864
211726_s_at	2.29e-34	2.28e-35	-13.572.318
202171_at	2.34e-34	2.33e-35	13.569.887
208389_s_at	2.46e-34	2.46e-35	-13.564.498
218412_s_at	2.50e-34	2.49e-35	13.563.024
212709_at	2.51e-34	2.50e-35	13.562.622
204331_s_at	2.52e-34	2.52e-35	13.561.779
216153_x_at	2.53e-34	2.53e-35	-13.561.596
213437_at	2.54e-34	2.54e-35	-13.561.128
222163_s_at	2.54e-34	2.55e-35	13.560.941
218910_at	2.58e-34	2.58e-35	13.559.509
201251_at	2.58e-34	2.59e-35	13.559.252
206007_at	2.61e-34	2.62e-35	-13.558.095
210396_s_at	2.63e-34	2.64e-35	13.557.299
216559_x_at	2.86e-34	2.87e-35	1.354.859
219345_at	2.87e-34	2.88e-35	13.548.103
210798_x_at	2.88e-34	2.90e-35	-13.547.497
214665_s_at	3.00e-34	3.02e-35	-13.543.334
213149_at	3.11e-34	3.12e-35	13.539.775
215201_at	3.12e-34	3.14e-35	-13.539.086
200003_s_at	3.30e-34	3.32e-35	13.533.446
205083_at	3.31e-34	3.33e-35	-13.533.022
221561_at	3.42e-34	3.44e-35	13.529.731

209355_s_at	3.55e-34	3.58e-35	-13.525.701
211727_s_at	3.59e-34	3.62e-35	13.524.482
205229_s_at	3.73e-34	3.77e-35	1.352.033
55081_at	3.74e-34	3.77e-35	13.520.254
202293_at	3.78e-34	3.82e-35	13.518.978
221798_x_at	4.07e-34	4.11e-35	13.511.321
221504_s_at	4.14e-34	4.18e-35	13.509.532
218271_s_at	4.16e-34	4.21e-35	13.508.793
218536_at	4.29e-34	4.33e-35	13.505.768
217968_at	4.33e-34	4.38e-35	13.504.762
204976_s_at	4.34e-34	4.39e-35	13.504.382
217238_s_at	4.37e-34	4.43e-35	-13.503.523
218735_s_at	4.43e-34	4.49e-35	13.502.162
213435_at	4.82e-34	4.89e-35	13.493.343
209978_s_at	4.83e-34	4.90e-35	-13.492.985
219349_s_at	4.90e-34	4.97e-35	13.491.467
221485_at	5.24e-34	5.32e-35	13.484.492
217714_x_at	5.24e-34	5.33e-35	13.484.384
205126_at	5.76e-34	5.85e-35	13.474.674
203974_at	5.81e-34	5.90e-35	13.473.747
215498_s_at	5.88e-34	5.98e-35	-13.472.338
201065_s_at	5.99e-34	6.09e-35	13.470.405
218106_s_at	6.02e-34	6.12e-35	13.469.859
205173_x_at	6.10e-34	6.21e-35	13.468.395
204457_s_at	6.37e-34	6.49e-35	-13.463.821
219233_s_at	6.39e-34	6.51e-35	-13.463.476
210366_at	6.57e-34	6.70e-35	-1.346.051
205091_x_at	6.99e-34	7.13e-35	13.454.088
201991_s_at	7.26e-34	7.41e-35	13.450.052
204705_x_at	7.31e-34	7.46e-35	-13.449.371
216903_s_at	7.84e-34	8.00e-35	-13.442.053
212200_at	7.84e-34	8.01e-35	13.441.968
214423_x_at	7.87e-34	8.04e-35	-13.441.575
214069_at	8.00e-34	8.18e-35	-13.439.742
218503_at	8.09e-34	8.28e-35	13.438.585
204606_at	8.09e-34	8.28e-35	-13.438.563
219371_s_at	8.15e-34	8.34e-35	-13.437.739
204566_at	8.16e-34	8.35e-35	13.437.607
202209_at	8.18e-34	8.38e-35	13.437.229
213253_at	8.33e-34	8.54e-35	13.435.333
212264_s_at	8.68e-34	8.90e-35	13.430.983
220688_s_at	9.07e-34	9.31e-35	13.426.345
217757_at	9.25e-34	9.50e-35	-13.424.229
221736_at	9.74e-34	1.00e-34	13.418.863

201075_s_at	9.81e-34	1.01e-34	13.418.036
221619_s_at	1.06e-33	1.08e-34	13.410.436
209080_x_at	1.06e-33	1.09e-34	1.341.005
201330_at	1.08e-33	1.11e-34	13.407.772
205046_at	1.10e-33	1.13e-34	13.406.448
218712_at	1.10e-33	1.13e-34	13.406.232
221899_at	1.17e-33	1.20e-34	-13.399.603
205463_s_at	1.18e-33	1.22e-34	1.339.834
214486_x_at	1.20e-33	1.24e-34	-13.396.317
218163_at	1.23e-33	1.27e-34	13.394.301
201870_at	1.24e-33	1.28e-34	13.393.223
200738_s_at	1.27e-33	1.31e-34	13.390.544
203062_s_at	1.34e-33	1.38e-34	13.385.234
202006_at	1.36e-33	1.40e-34	13.383.663
214226_at	1.36e-33	1.41e-34	-13.383.051
209165_at	1.38e-33	1.43e-34	1.338.173
219526_at	1.41e-33	1.46e-34	13.379.326
206878_at	1.42e-33	1.47e-34	-13.378.728
201770_at	1.45e-33	1.50e-34	13.376.554
218021_at	1.48e-33	1.54e-34	-1.337.416
210072_at	1.53e-33	1.59e-34	-13.370.732
51774_s_at	1.55e-33	1.60e-34	-13.369.731
216284_at	1.61e-33	1.67e-34	-13.365.732
219162_s_at	1.62e-33	1.68e-34	13.364.944
220786_s_at	1.67e-33	1.73e-34	-13.361.656
202715_at	1.68e-33	1.75e-34	13.360.616
219216_at	1.73e-33	1.79e-34	13.358.119
203079_s_at	1.73e-33	1.79e-34	13.358.021
200926_at	1.85e-33	1.92e-34	13.350.789
210296_s_at	1.86e-33	1.93e-34	13.350.389
202483_s_at	1.87e-33	1.95e-34	13.349.519
218098_at	1.89e-33	1.97e-34	13.348.538
221475_s_at	1.93e-33	2.01e-34	13.346.148
217841_s_at	2.04e-33	2.13e-34	13.340.352
218088_s_at	2.04e-33	2.13e-34	13.340.088
209977_at	2.04e-33	2.13e-34	-13.340.021
213453_x_at	2.11e-33	2.20e-34	1.333.691
207752_x_at	2.25e-33	2.35e-34	-13.329.819
209076_s_at	2.26e-33	2.36e-34	13.329.458
214076_at	2.33e-33	2.44e-34	-1.332.615
209628_at	2.39e-33	2.50e-34	13.323.499
216241_s_at	2.50e-33	2.61e-34	13.318.791
218638_s_at	2.54e-33	2.66e-34	13.316.885
218438_s_at	2.65e-33	2.77e-34	13.312.755

210216_x_at	2.69e-33	2.82e-34	13.311.041
218137_s_at	2.75e-33	2.88e-34	13.308.793
200977_s_at	2.86e-33	3.00e-34	13.304.502
212436_at	2.90e-33	3.05e-34	13.302.806
207298_at	3.03e-33	3.17e-34	-13.298.502
215397_x_at	3.04e-33	3.19e-34	-13.298.068
206743_s_at	3.04e-33	3.19e-34	-13.297.952
213664_at	3.14e-33	3.30e-34	-13.294.512
200737_at	3.28e-33	3.45e-34	13.289.786
211590_x_at	3.34e-33	3.51e-34	-13.287.891
215768_at	3.36e-33	3.53e-34	-1.328.733
211600_at	3.45e-33	3.63e-34	-13.284.454
200044_at	3.52e-33	3.70e-34	1.328.239
203208_s_at	3.66e-33	3.85e-34	1.327.833
210759_s_at	3.69e-33	3.89e-34	13.277.345
205843_x_at	3.76e-33	3.97e-34	-13.275.199
207188_at	3.79e-33	3.99e-34	-13.274.536
203243_s_at	3.99e-33	4.21e-34	-13.269.016
209646_x_at	4.09e-33	4.32e-34	-13.266.271
209660_at	4.26e-33	4.50e-34	-1.326.206
204119_s_at	4.78e-33	5.05e-34	-13.249.949
212439_at	4.88e-33	5.15e-34	1.324.788
217784_at	4.96e-33	5.24e-34	13.246.134
222200_s_at	5.20e-33	5.50e-34	-13.241.069
215158_s_at	5.23e-33	5.53e-34	13.240.476
203960_s_at	5.41e-33	5.73e-34	13.236.737
213245_at	5.57e-33	5.90e-34	-13.233.731
209429_x_at	5.60e-33	5.94e-34	13.233.056
202275_at	5.74e-33	6.08e-34	13.230.527
215506_s_at	5.92e-33	6.28e-34	-13.227.147
218997_at	6.10e-33	6.47e-34	-13.224.097
201853_s_at	6.26e-33	6.64e-34	13.221.363
1053_at	6.33e-33	6.72e-34	13.220.115
218434_s_at	6.41e-33	6.80e-34	13.218.768
203786_s_at	6.46e-33	6.87e-34	-13.217.788
211357_s_at	6.58e-33	6.99e-34	-13.215.925
219053_s_at	6.85e-33	7.28e-34	13.211.671
201238_s_at	6.86e-33	7.29e-34	13.211.516
214212_x_at	6.88e-33	7.33e-34	-13.211.019
207378_at	6.97e-33	7.42e-34	-1.320.966
208836_at	7.08e-33	7.54e-34	13.208.045
221825_at	7.25e-33	7.72e-34	13.205.542
201809_s_at	7.46e-33	7.96e-34	-13.202.406
211610_at	7.50e-33	8.00e-34	-13.201.786

200754_x_at	7.60e-33	8.11e-34	13.200.418
200841_s_at	7.64e-33	8.15e-34	13.199.837
210027_s_at	7.76e-33	8.29e-34	13.198.125
221893_s_at	7.96e-33	8.50e-34	13.195.412
209188_x_at	8.16e-33	8.72e-34	13.192.787
204315_s_at	8.24e-33	8.81e-34	13.191.764
205218_at	8.32e-33	8.90e-34	13.190.637
212934_at	8.39e-33	8.98e-34	13.189.771
218961_s_at	8.54e-33	9.14e-34	13.187.807
219306_at	8.67e-33	9.28e-34	13.186.221
209174_s_at	8.69e-33	9.31e-34	13.185.918
202373_s_at	9.05e-33	9.70e-34	13.181.604
200916_at	9.13e-33	9.80e-34	13.180.608
212584_at	9.60e-33	1.03e-33	1.317.537
218073_s_at	9.79e-33	1.05e-33	13.173.231
210426_x_at	9.94e-33	1.07e-33	-13.171.661
215499_at	1.00e-32	1.08e-33	-13.170.757
221540_x_at	1.01e-32	1.09e-33	13.169.857
210257_x_at	1.01e-32	1.09e-33	13.169.443
211941_s_at	1.02e-32	1.10e-33	-13.168.458
218224_at	1.04e-32	1.12e-33	13.166.769
219146_at	1.05e-32	1.13e-33	13.165.456
209323_at	1.09e-32	1.17e-33	13.161.538
200066_at	1.11e-32	1.20e-33	1.315.963
214681_at	1.11e-32	1.20e-33	-13.159.563
210888_s_at	1.13e-32	1.22e-33	-13.157.815
219427_at	1.15e-32	1.24e-33	-13.155.749
218535_s_at	1.16e-32	1.25e-33	13.155.111
215535_s_at	1.22e-32	1.31e-33	13.149.759
201659_s_at	1.24e-32	1.34e-33	13.147.434
200030_s_at	1.27e-32	1.37e-33	13.145.629
201794_s_at	1.27e-32	1.37e-33	13.145.515
204366_s_at	1.27e-32	1.37e-33	13.145.381
218051_s_at	1.27e-32	1.37e-33	13.145.283
215220_s_at	1.29e-32	1.40e-33	1.314.333
214307_at	1.30e-32	1.41e-33	-13.142.474
202228_s_at	1.31e-32	1.42e-33	13.141.354
217496_s_at	1.34e-32	1.45e-33	13.139.159
214282_at	1.36e-32	1.48e-33	-13.137.526
218125_s_at	1.37e-32	1.49e-33	-131.366
204993_at	1.38e-32	1.50e-33	13.135.904
206898_at	1.38e-32	1.50e-33	-1.313.588
41220_at	1.39e-32	1.51e-33	13.135.232
202964_s_at	1.45e-32	1.58e-33	13.130.755

200825_s_at	1.47e-32	1.60e-33	13.129.429
203211_s_at	1.47e-32	1.60e-33	13.128.925
203897_at	1.49e-32	1.62e-33	-13.127.886
40189_at	1.57e-32	1.71e-33	13.122.009
32088_at	1.60e-32	1.74e-33	1.312.031
208652_at	1.60e-32	1.74e-33	13.120.133
202686_s_at	1.64e-32	1.78e-33	-13.117.838
204753_s_at	1.71e-32	1.86e-33	-13.113.189
214234_s_at	1.74e-32	1.90e-33	-13.111.022
213377_x_at	1.76e-32	1.92e-33	13.110.096
218839_at	1.77e-32	1.94e-33	13.109.126
208962_s_at	1.78e-32	1.94e-33	131.089
203435_s_at	1.82e-32	1.98e-33	-13.106.509
215385_at	1.82e-32	1.99e-33	-13.106.079
202750_s_at	1.86e-32	2.03e-33	13.103.961
215321_at	1.90e-32	2.07e-33	-13.101.905
211969_at	1.98e-32	2.16e-33	13.097.425
207654_x_at	1.99e-32	2.18e-33	13.096.699
214700_x_at	2.00e-32	2.19e-33	13.096.342
203354_s_at	2.03e-32	2.22e-33	-13.094.614
37384_at	2.07e-32	2.27e-33	13.092.401
208842_s_at	2.13e-32	2.34e-33	13.089.311
201896_s_at	2.14e-32	2.34e-33	13.088.961
210466_s_at	2.16e-32	2.37e-33	13.088.028
202919_at	2.23e-32	2.44e-33	13.084.657
219294_at	2.23e-32	2.45e-33	13.084.207
209626_s_at	2.26e-32	2.48e-33	13.083.087
218356_at	2.27e-32	2.50e-33	13.082.415
203522_at	2.31e-32	2.54e-33	-13.080.504
218395_at	2.47e-32	2.72e-33	13.073.462
213923_at	2.50e-32	2.75e-33	13.072.078
204128_s_at	2.51e-32	2.76e-33	13.071.824
221434_s_at	2.53e-32	2.79e-33	13.070.792
203181_x_at	2.57e-32	2.83e-33	13.069.284
219125_s_at	2.57e-32	2.84e-33	13.068.989
218923_at	2.61e-32	2.88e-33	-13.067.518
35666_at	2.64e-32	2.91e-33	13.066.336
214424_s_at	2.68e-32	2.96e-33	-13.064.574
201258_at	2.74e-32	3.03e-33	13.062.159
219394_at	2.81e-32	3.11e-33	13.059.405
202111_at	2.85e-32	3.15e-33	1.305.785
203730_s_at	2.87e-32	3.17e-33	13.057.231
221495_s_at	3.40e-32	3.76e-33	-1.303.933
201061_s_at	3.47e-32	3.84e-33	-13.037.142

216338_s_at	3.53e-32	3.90e-33	13.035.313
202740_at	3.58e-32	3.96e-33	-13.033.873
202786_at	3.59e-32	3.97e-33	13.033.423
208946_s_at	4.20e-32	4.66e-33	13.016.717
53987_at	4.21e-32	4.67e-33	-13.016.543
41047_at	4.23e-32	4.69e-33	13.015.943
215559_at	4.48e-32	4.97e-33	-13.009.818
208752_x_at	4.49e-32	4.98e-33	130.097
201994_at	4.49e-32	4.98e-33	1.300.959
219562_at	4.53e-32	5.03e-33	-13.008.612
218857_s_at	4.53e-32	5.04e-33	13.008.491
210570_x_at	4.55e-32	5.05e-33	13.008.116
31835_at	4.65e-32	5.17e-33	-13.005.821
210378_s_at	4.66e-32	5.18e-33	13.005.537
206345_s_at	4.79e-32	5.33e-33	-13.002.501
213567_at	5.03e-32	5.60e-33	12.997.328
219431_at	5.30e-32	5.90e-33	-12.991.845
218560_s_at	5.37e-32	5.98e-33	12.990.366
209751_s_at	5.44e-32	6.06e-33	12.988.991
201855_s_at	5.52e-32	6.16e-33	-12.987.244
203846_at	5.65e-32	6.31e-33	12.984.757
212438_at	5.69e-32	6.35e-33	12.983.994
200855_at	5.81e-32	6.49e-33	-12.981.713
201044_x_at	5.83e-32	6.51e-33	-12.981.426
202161_at	5.93e-32	6.62e-33	12.979.601
204688_at	5.98e-32	6.69e-33	12.978.543
203372_s_at	6.05e-32	6.76e-33	-12.977.418
201946_s_at	6.34e-32	7.09e-33	12.972.348
39249_at	6.41e-32	7.17e-33	-12.971.186
218507_at	6.72e-32	7.52e-33	12.966.138
212110_at	6.77e-32	7.58e-33	-12.965.372
209279_s_at	6.83e-32	7.65e-33	12.964.351
202043_s_at	7.15e-32	8.02e-33	12.959.433
202433_at	7.38e-32	8.28e-33	12.956.049
203242_s_at	7.42e-32	8.32e-33	-12.955.522
205401_at	7.69e-32	8.62e-33	12.951.716
201704_at	7.90e-32	8.87e-33	12.948.801
212456_at	7.94e-32	8.92e-33	-12.948.154
201523_x_at	7.99e-32	8.98e-33	12.947.458
57532_at	8.09e-32	9.09e-33	12.946.176
204944_at	8.14e-32	9.15e-33	12.945.449
218894_s_at	8.15e-32	9.17e-33	12.945.258
213553_x_at	8.64e-32	9.73e-33	-12.939.021
218103_at	8.65e-32	9.74e-33	12.938.907

219076_s_at	8.68e-32	9.77e-33	-12.938.495
212716_s_at	9.37e-32	1.06e-32	12.930.356
201548_s_at	9.39e-32	1.06e-32	12.930.102
203520_s_at	9.41e-32	1.06e-32	12.929.861
AFFX-HUMGAPDH/M3	9.46e-32	1.07e-32	12.929.208
219000_s_at	9.60e-32	1.08e-32	12.927.564
205393_s_at	9.62e-32	1.09e-32	1.292.734
219087_at	9.74e-32	1.10e-32	-12.925.979
211316_x_at	1.01e-31	1.14e-32	-12.922.512
205364_at	1.03e-31	1.17e-32	-12.919.675
210320_s_at	1.04e-31	1.17e-32	12.919.406
214113_s_at	1.04e-31	1.17e-32	12.919.229
219410_at	1.05e-31	1.18e-32	-12.918.291
200712_s_at	1.12e-31	1.27e-32	12.910.896
208611_s_at	1.13e-31	1.28e-32	12.910.271
220933_s_at	1.15e-31	1.31e-32	-12.907.862
205082_s_at	1.17e-31	1.33e-32	-12.906.107
200053_at	1.17e-31	1.33e-32	-1.290.589
202165_at	1.21e-31	1.37e-32	12.903
210154_at	1.21e-31	1.37e-32	12.902.973
203317_at	1.21e-31	1.37e-32	-1.290.255
200975_at	1.21e-31	1.38e-32	12.902.283
202088_at	1.26e-31	1.43e-32	1.289.832
212060_at	1.27e-31	1.44e-32	1.289.749
218210_at	1.27e-31	1.45e-32	12.897.082
222155_s_at	1.28e-31	1.45e-32	12.896.489
208695_s_at	1.28e-31	1.46e-32	12.896.065
203459_s_at	1.39e-31	1.58e-32	12.887.681
210911_at	1.45e-31	1.65e-32	-12.882.904
205352_at	1.47e-31	1.67e-32	12.881.862
216267_s_at	1.47e-31	1.68e-32	12.881.231
204480_s_at	1.48e-31	1.69e-32	12.880.685
201362_at	1.49e-31	1.70e-32	12.879.883
208289_s_at	1.62e-31	1.84e-32	-12.871.305
202603_at	1.65e-31	1.88e-32	12.869.082
207956_x_at	1.66e-31	1.90e-32	12.868.126
202074_s_at	1.69e-31	1.93e-32	12.866.524
202722_s_at	1.69e-31	1.94e-32	12.866.223
210646_x_at	1.72e-31	1.96e-32	12.864.662
220746_s_at	1.74e-31	2.00e-32	1.286.299
212790_x_at	1.81e-31	2.07e-32	12.859.092
217801_at	1.81e-31	2.08e-32	12.858.795
210024_s_at	1.87e-31	2.14e-32	12.855.739
204173_at	1.87e-31	2.14e-32	12.855.569

215129_at	2.05e-31	2.35e-32	-12.845.535
212566_at	2.06e-31	2.36e-32	12.845.148
219117_s_at	2.07e-31	2.37e-32	12.844.768
202015_x_at	2.07e-31	2.38e-32	-12.844.274
207988_s_at	2.12e-31	2.43e-32	12.842.066
202556_s_at	2.16e-31	2.48e-32	128.401
214272_at	2.30e-31	2.64e-32	-12.833.432
208679_s_at	2.35e-31	2.70e-32	12.830.875
208619_at	2.36e-31	2.72e-32	12.830.306
221521_s_at	2.46e-31	2.82e-32	1.282.613
208471_at	2.46e-31	2.83e-32	-12.826.109
1598_g_at	2.62e-31	3.02e-32	-12.819.205
216782_at	2.62e-31	3.02e-32	-12.819.187
204351_at	2.66e-31	3.07e-32	12.817.356
214949_at	2.67e-31	3.07e-32	12.817.248
207138_at	2.72e-31	3.14e-32	-12.814.872
212248_at	2.77e-31	3.20e-32	12.812.995
203702_s_at	2.77e-31	3.20e-32	1.281.293
209797_at	2.82e-31	3.25e-32	12.811.167
201264_at	2.83e-31	3.26e-32	12.810.797
218575_at	3.03e-31	3.50e-32	12.803.331
210327_s_at	3.10e-31	3.59e-32	-12.800.776
204163_at	3.18e-31	3.67e-32	-12.798.294
215482_s_at	3.18e-31	3.68e-32	12.798.108
213180_s_at	3.23e-31	3.73e-32	12.796.587
212553_at	3.29e-31	3.81e-32	12.794.302
207622_s_at	3.36e-31	3.89e-32	12.792.047
203279_at	3.49e-31	4.04e-32	-12.788.182
64440_at	3.49e-31	4.05e-32	-12.787.927
211135_x_at	3.54e-31	4.11e-32	-12.786.397
210404_x_at	3.55e-31	4.11e-32	-12.786.263
37408_at	3.60e-31	4.18e-32	-127.846
207039_at	3.83e-31	4.44e-32	12.778.018
218091_at	3.90e-31	4.53e-32	12.776.079
220753_s_at	3.91e-31	4.54e-32	-12.775.794
200716_x_at	3.93e-31	4.56e-32	12.775.167
201114_x_at	3.99e-31	4.64e-32	12.773.398
203342_at	4.06e-31	4.72e-32	12.771.535
203653_s_at	4.16e-31	4.84e-32	12.768.919
219695_at	4.30e-31	5.00e-32	-12.765.478
200050_at	4.38e-31	5.09e-32	12.763.511
218068_s_at	4.46e-31	5.20e-32	12.761.357
215931_s_at	4.73e-31	5.51e-32	12.755.127
205249_at	4.73e-31	5.52e-32	-12.755.015

205471_s_at	4.80e-31	5.60e-32	-12.753.452
212612_at	4.83e-31	5.63e-32	12.752.863
210028_s_at	5.04e-31	5.88e-32	1.274.828
209418_s_at	5.22e-31	6.09e-32	1.274.456
203472_s_at	5.22e-31	6.09e-32	-12.744.506
202678_at	5.24e-31	6.12e-32	12.744.075
205328_at	5.38e-31	6.29e-32	-12.741.075
212581_x_at	5.48e-31	6.40e-32	12.739.188
211965_at	5.57e-31	6.51e-32	-12.737.357
202736_s_at	5.71e-31	6.68e-32	12.734.728
205769_at	5.78e-31	6.76e-32	-12.733.432
213300_at	5.97e-31	6.99e-32	-12.729.826
203487_s_at	6.00e-31	7.03e-32	12.729.286
201667_at	6.25e-31	7.33e-32	1.272.482
218695_at	6.27e-31	7.35e-32	1.272.449
202263_at	6.46e-31	7.57e-32	12.721.293
221242_at	6.52e-31	7.65e-32	-12.720.268
218667_at	6.56e-31	7.70e-32	12.719.504
202289_s_at	6.67e-31	7.83e-32	12.717.734
207819_s_at	6.69e-31	7.86e-32	-12.717.402
208774_at	6.87e-31	8.08e-32	12.714.468
205200_at	6.90e-31	8.11e-32	-1.271.403
209422_at	7.07e-31	8.31e-32	12.711.377
218130_at	7.22e-31	8.49e-32	12.709.122
39248_at	7.30e-31	8.59e-32	-1.270.794
218700_s_at	7.34e-31	8.64e-32	12.707.303
205412_at	7.45e-31	8.77e-32	-12.705.731
217848_s_at	7.45e-31	8.77e-32	12.705.659
201876_at	7.65e-31	9.02e-32	12.702.727
213730_x_at	7.71e-31	9.09e-32	12.701.824
212542_s_at	8.03e-31	9.47e-32	1.269.749
206263_at	8.04e-31	9.49e-32	-12.697.326
203893_at	8.37e-31	9.88e-32	12.692.973
218718_at	8.77e-31	1.04e-31	-12.687.978
215722_s_at	8.85e-31	1.04e-31	12.687.039
208668_x_at	8.89e-31	1.05e-31	12.686.465
216652_s_at	8.98e-31	1.06e-31	12.685.347
213228_at	9.07e-31	1.07e-31	-1.268.429
218626_at	9.97e-31	1.18e-31	12.674.108
209210_s_at	1.05e-30	1.24e-31	-12.668.848
213397_x_at	1.08e-30	1.27e-31	-12.665.915
201563_at	1.10e-30	1.30e-31	-12.663.444
208863_s_at	1.10e-30	1.31e-31	12.663.041
218351_at	1.11e-30	1.32e-31	12.661.992

213158_at	1.12e-30	1.33e-31	-12.661.532
213869_x_at	1.13e-30	1.34e-31	126.605
205739_x_at	1.15e-30	1.37e-31	12.658.349
221795_at	1.16e-30	1.38e-31	-12.657.361
200820_at	1.17e-30	1.39e-31	12.656.689
202559_x_at	1.19e-30	1.41e-31	12.654.693
215235_at	1.20e-30	1.43e-31	12.653.819
220110_s_at	1.21e-30	1.43e-31	-12.653.291
218286_s_at	1.24e-30	1.47e-31	12.650.552
202590_s_at	1.24e-30	1.48e-31	-12.649.888
218946_at	1.25e-30	1.49e-31	12.648.956
214801_at	1.26e-30	1.50e-31	12.648.409
210054_at	1.30e-30	1.55e-31	12.644.873
206264_at	1.31e-30	1.56e-31	-12.644.039
201197_at	1.35e-30	1.61e-31	12.641.182
213509_x_at	1.35e-30	1.61e-31	-12.640.741
200058_s_at	1.38e-30	1.65e-31	12.638.257
209648_x_at	1.42e-30	1.70e-31	12.635.352
179_at	1.45e-30	1.73e-31	-12.633.332
214133_at	1.45e-30	1.73e-31	-12.633.003
205264_at	1.53e-30	1.82e-31	12.627.617
219732_at	1.54e-30	1.83e-31	12.626.922
220765_s_at	1.54e-30	1.84e-31	-12.626.825
200667_at	1.62e-30	1.94e-31	-1.262.118
219449_s_at	1.73e-30	2.08e-31	12.613.778
203035_s_at	1.87e-30	2.24e-31	1.260.542
202060_at	1.91e-30	2.29e-31	12.603.324
212796_s_at	1.94e-30	2.32e-31	-12.601.793
218407_x_at	2.00e-30	2.39e-31	12.598.543
206138_s_at	2.07e-30	2.48e-31	12.594.552
209267_s_at	2.08e-30	2.49e-31	-1.259.411
204514_at	2.09e-30	2.51e-31	12.593.613
209071_s_at	2.10e-30	2.52e-31	12.592.922
211920_at	2.17e-30	2.60e-31	-12.589.561
210479_s_at	2.19e-30	2.63e-31	-12.588.603
208672_s_at	2.23e-30	2.68e-31	12.586.422
213937_s_at	2.28e-30	2.74e-31	12.583.865
218618_s_at	2.29e-30	2.75e-31	12.583.612
213515_x_at	2.29e-30	2.75e-31	-12.583.597
211143_x_at	2.34e-30	2.82e-31	-12.581.114
210811_s_at	2.42e-30	2.92e-31	12.577.373
203802_x_at	2.51e-30	3.02e-31	12.573.646
208801_at	2.52e-30	3.03e-31	12.573.183
201669_s_at	2.54e-30	3.06e-31	12.572.285

205208_at	2.55e-30	3.07e-31	-12.571.718
213920_at	2.58e-30	3.12e-31	-1.257.031
204088_at	2.61e-30	3.15e-31	12.569.157
216913_s_at	2.63e-30	3.17e-31	12.568.292
200857_s_at	2.63e-30	3.18e-31	-12.568.176
215691_x_at	2.74e-30	3.31e-31	12.563.944
207435_s_at	2.76e-30	3.33e-31	-1.256.323
205878_at	3.04e-30	3.67e-31	-12.552.774
203658_at	3.22e-30	3.89e-31	-12.546.429
207513_s_at	3.27e-30	3.95e-31	12.544.788
204120_s_at	3.29e-30	3.98e-31	-12.544.046
40665_at	3.35e-30	4.06e-31	-1.254.203
218621_at	3.36e-30	4.07e-31	-12.541.783
201612_at	3.38e-30	4.09e-31	-1.254.109
203542_s_at	3.40e-30	4.12e-31	-12.540.409
202670_at	3.50e-30	4.25e-31	-12.537.135
210776_x_at	3.60e-30	4.37e-31	12.534.071
219175_s_at	3.68e-30	4.47e-31	12.531.638
205395_s_at	3.77e-30	4.58e-31	12.529.064
32837_at	3.83e-30	4.65e-31	-12.527.337
221014_s_at	3.90e-30	4.74e-31	-12.525.426
221770_at	3.90e-30	4.74e-31	1.252.526
41386_i_at	3.92e-30	4.76e-31	-12.524.832
217975_at	3.98e-30	4.84e-31	12.523.138
32836_at	3.99e-30	4.85e-31	12.522.845
211028_s_at	3.99e-30	4.85e-31	-12.522.787
209698_at	4.12e-30	5.01e-31	12.519.342
206130_s_at	4.13e-30	5.03e-31	-12.519.034
219931_s_at	4.25e-30	5.17e-31	12.515.954
201259_s_at	4.32e-30	5.26e-31	12.514.103
53202_at	4.38e-30	5.34e-31	12.512.549
206697_s_at	4.40e-30	5.37e-31	-12.511.999
201614_s_at	4.45e-30	5.43e-31	12.510.729
204203_at	4.55e-30	5.55e-31	12.508.406
201214_s_at	4.62e-30	5.64e-31	1.250.659
202535_at	4.66e-30	5.69e-31	12.505.681
211519_s_at	4.78e-30	5.84e-31	12.502.978
200971_s_at	4.81e-30	5.88e-31	12.502.189
210622_x_at	4.91e-30	6.00e-31	-12.499.999
211948_x_at	5.01e-30	6.12e-31	12.497.901
91684_g_at	5.17e-30	6.32e-31	1.249.445
211970_x_at	5.26e-30	6.43e-31	12.492.586
213864_s_at	5.40e-30	6.62e-31	12.489.549
208802_at	5.47e-30	6.70e-31	12.488.188

202347_s_at	5.68e-30	6.96e-31	12.484.136
213600_at	5.68e-30	6.96e-31	12.484.071
208892_s_at	5.68e-30	6.97e-31	-12.483.989
212881_at	5.71e-30	7.01e-31	1.248.338
206204_at	5.74e-30	7.04e-31	-12.482.846
212779_at	5.81e-30	7.13e-31	-12.481.575
214002_at	5.84e-30	7.17e-31	-12.480.955
212983_at	5.85e-30	7.18e-31	12.480.697
217117_x_at	5.99e-30	7.36e-31	-124.781
210410_s_at	5.99e-30	7.37e-31	12.477.989
216942_s_at	6.02e-30	7.40e-31	12.477.452
218978_s_at	6.03e-30	7.41e-31	-12.477.302
201231_s_at	6.14e-30	7.56e-31	12.475.218
216952_s_at	6.36e-30	7.83e-31	12.471.418
214742_at	6.36e-30	7.84e-31	12.471.372
212169_at	6.38e-30	7.86e-31	1.247.107
204528_s_at	6.49e-30	7.99e-31	12.469.216
214198_s_at	6.52e-30	8.04e-31	12.468.604
217958_at	6.54e-30	8.07e-31	12.468.244
221027_s_at	6.56e-30	8.09e-31	-12.467.929
212738_at	6.73e-30	8.31e-31	124.651
204934_s_at	6.76e-30	8.34e-31	-12.464.633
221041_s_at	6.92e-30	8.54e-31	1.246.212
212712_at	7.04e-30	8.69e-31	12.460.218
202323_s_at	7.04e-30	8.71e-31	12.460.045
201834_at	7.06e-30	8.73e-31	12.459.807
210619_s_at	7.07e-30	8.74e-31	-12.459.645
209343_at	7.32e-30	9.06e-31	-12.455.791
208626_s_at	7.32e-30	9.06e-31	1.245.571
212020_s_at	7.36e-30	9.11e-31	12.455.151
221737_at	7.48e-30	9.26e-31	12.453.389
203396_at	7.48e-30	9.26e-31	12.453.368
208884_s_at	7.49e-30	9.28e-31	12.453.204
203414_at	7.60e-30	9.42e-31	12.451.586
211627_x_at	7.70e-30	9.55e-31	-12.450.128
218235_s_at	8.11e-30	1.01e-30	12.444.468
212722_s_at	8.13e-30	1.01e-30	12.444.214
209257_s_at	8.14e-30	1.01e-30	12.443.939
219102_at	8.27e-30	1.03e-30	-1.244.223
204613_at	8.49e-30	1.05e-30	-12.439.424
205324_s_at	8.49e-30	1.06e-30	1.243.931
203114_at	8.56e-30	1.06e-30	12.438.453
215049_x_at	8.70e-30	1.08e-30	-12.436.598
218353_at	8.87e-30	1.10e-30	12.434.506

208610_s_at	8.87e-30	1.10e-30	-12.434.441
206853_s_at	8.88e-30	1.11e-30	12.434.279
211951_at	9.22e-30	1.15e-30	12.430.236
206173_x_at	9.30e-30	1.16e-30	12.429.255
207157_s_at	9.30e-30	1.16e-30	12.429.237
218291_at	9.58e-30	1.19e-30	12.426.003
209385_s_at	9.83e-30	1.23e-30	-12.423.145
206613_s_at	9.84e-30	1.23e-30	1.242.305
208787_at	9.87e-30	1.23e-30	12.422.722
212306_at	1.02e-29	1.27e-30	12.419.481
201002_s_at	1.02e-29	1.28e-30	12.418.614
204294_at	1.08e-29	1.34e-30	-12.413.309
201386_s_at	1.08e-29	1.35e-30	12.412.946
202541_at	1.09e-29	1.37e-30	12.411.565
200955_at	1.12e-29	1.40e-30	12.408.997
221634_at	1.16e-29	1.45e-30	12.404.849
210371_s_at	1.18e-29	1.48e-30	12.403.149
209546_s_at	1.19e-29	1.49e-30	-12.402.006
212815_at	1.23e-29	1.54e-30	12.398.911
208877_at	1.24e-29	1.55e-30	12.398.033
203371_s_at	1.24e-29	1.56e-30	12.397.206
203407_at	1.26e-29	1.58e-30	-12.396
222006_at	1.27e-29	1.59e-30	12.395.061
200956_s_at	1.28e-29	1.61e-30	12.393.986
217839_at	1.28e-29	1.61e-30	12.393.951
203519_s_at	1.30e-29	1.64e-30	12.392.134
205063_at	1.31e-29	1.64e-30	12.391.561
201209_at	1.37e-29	1.72e-30	12.386.965
201460_at	1.38e-29	1.73e-30	12.386.111
213462_at	1.40e-29	1.76e-30	1.238.418
209028_s_at	1.42e-29	1.79e-30	12.382.426
213179_at	1.43e-29	1.80e-30	12.381.556
209233_at	1.46e-29	1.84e-30	12.379.272
206815_at	1.48e-29	1.87e-30	-12.378.003
214794_at	1.52e-29	1.91e-30	12.375.333
203078_at	1.54e-29	1.94e-30	12.374.031
212747_at	1.57e-29	1.99e-30	-12.371.262
208445_s_at	1.58e-29	1.99e-30	12.370.868
207808_s_at	1.60e-29	2.02e-30	-12.369.538
200804_at	1.61e-29	2.03e-30	-1.236.905
215140_at	1.61e-29	2.03e-30	-12.368.853
206205_at	1.61e-29	2.04e-30	12.368.437
213422_s_at	1.64e-29	2.08e-30	-12.366.389
207338_s_at	1.72e-29	2.18e-30	12.361.272

213793_s_at	1.72e-29	2.18e-30	12.361.242
207697_x_at	1.76e-29	2.23e-30	-12.358.622
215716_s_at	1.84e-29	2.32e-30	12.354.282
64486_at	1.87e-29	2.36e-30	12.352.393
212919_at	1.87e-29	2.37e-30	1.235.236
217398_x_at	1.91e-29	2.42e-30	12.350.088
220626_at	1.91e-29	2.42e-30	-12.349.775
219041_s_at	1.98e-29	2.51e-30	12.346.007
213696_s_at	2.01e-29	2.55e-30	12.344.247
218482_at	2.06e-29	2.61e-30	12.341.697
218804_at	2.06e-29	2.62e-30	-12.341.537
202502_at	2.08e-29	2.63e-30	-12.340.735
210416_s_at	2.08e-29	2.65e-30	1.234.029
218004_at	2.11e-29	2.68e-30	-12.338.851
218557_at	2.22e-29	2.82e-30	-12.333.228
218367_x_at	2.22e-29	2.83e-30	1.233.318
219010_at	2.24e-29	2.85e-30	12.332.427
220643_s_at	2.28e-29	2.89e-30	12.330.571
215783_s_at	2.32e-29	2.95e-30	-12.328.508
200808_s_at	2.32e-29	2.95e-30	12.328.396
219376_at	2.35e-29	2.99e-30	1.232.705
39549_at	2.37e-29	3.02e-30	12.325.962
204271_s_at	2.40e-29	3.06e-30	-12.324.707
201745_at	2.49e-29	3.18e-30	12.320.398
201038_s_at	2.51e-29	3.20e-30	12.319.839
209149_s_at	2.53e-29	3.22e-30	12.318.937
212282_at	2.61e-29	3.33e-30	12.315.377
213074_at	2.68e-29	3.42e-30	12.312.432
203089_s_at	2.69e-29	3.44e-30	12.312.024
213102_at	2.77e-29	3.54e-30	1.230.876
211936_at	2.78e-29	3.56e-30	12.308.299
209581_at	2.85e-29	3.64e-30	-12.305.693
243_g_at	2.86e-29	3.66e-30	12.305.226
205811_at	2.89e-29	3.70e-30	12.304.011
37512_at	2.93e-29	3.75e-30	-12.302.589
209434_s_at	2.96e-29	3.79e-30	12.301.551
208905_at	2.97e-29	3.80e-30	12.301.055
206055_s_at	2.97e-29	3.81e-30	12.300.943
221614_s_at	3.01e-29	3.86e-30	-12.299.367
217986_s_at	3.06e-29	3.93e-30	12.297.535
214363_s_at	3.15e-29	4.05e-30	12.294.363
211101_x_at	3.19e-29	4.09e-30	-12.293.152
200678_x_at	3.21e-29	4.12e-30	12.292.523
214064_at	3.25e-29	4.17e-30	-12.291.026

202251_at	3.38e-29	4.35e-30	12.286.586
201079_at	3.43e-29	4.41e-30	12.284.991
217730_at	3.47e-29	4.45e-30	12.283.945
220994_s_at	3.51e-29	4.52e-30	12.282.463
204755_x_at	3.53e-29	4.53e-30	-12.282.035
221515_s_at	3.68e-29	4.73e-30	12.277.492
213428_s_at	3.81e-29	4.90e-30	-12.273.597
208771_s_at	3.88e-29	5.00e-30	12.271.453
202720_at	3.92e-29	5.05e-30	12.270.352
212967_x_at	3.98e-29	5.13e-30	12.268.672
213762_x_at	4.03e-29	5.20e-30	1.226.722
219778_at	4.06e-29	5.23e-30	-12.266.585
200963_x_at	4.16e-29	5.36e-30	12.263.853
220005_at	4.17e-29	5.39e-30	-12.263.387
211342_x_at	4.33e-29	5.59e-30	12.259.392
212447_at	4.38e-29	5.66e-30	12.258.046
205292_s_at	4.57e-29	5.91e-30	12.253.406
204900_x_at	4.61e-29	5.95e-30	12.252.584
218281_at	4.64e-29	5.99e-30	12.251.845
211968_s_at	4.70e-29	6.07e-30	12.250.428
212087_s_at	4.74e-29	6.13e-30	12.249.292
221492_s_at	4.75e-29	6.14e-30	12.249.141
204605_at	4.82e-29	6.24e-30	1.224.741
64488_at	4.96e-29	6.43e-30	12.244.243
209655_s_at	4.97e-29	6.43e-30	-12.244.172
209565_at	5.05e-29	6.55e-30	12.242.251
204859_s_at	5.31e-29	6.88e-30	12.236.803
202257_s_at	5.37e-29	6.96e-30	12.235.592
204341_at	5.41e-29	7.01e-30	12.234.781
210878_s_at	5.42e-29	7.04e-30	12.234.405
218487_at	5.64e-29	7.33e-30	-12.230.038
205024_s_at	5.73e-29	7.45e-30	12.228.292
202211_at	5.83e-29	7.57e-30	12.226.449
202106_at	5.84e-29	7.58e-30	12.226.331
212718_at	5.87e-29	7.63e-30	12.225.687
38269_at	6.03e-29	7.84e-30	12.222.683
206872_at	6.18e-29	8.04e-30	-12.219.937
204370_at	6.22e-29	8.09e-30	12.219.326
208783_s_at	6.22e-29	8.09e-30	1.221.928
207220_at	6.22e-29	8.10e-30	-12.219.121
221531_at	6.34e-29	8.25e-30	1.221.709
201136_at	6.56e-29	8.55e-30	12.213.249
219796_s_at	6.60e-29	8.60e-30	-12.212.622
209143_s_at	6.63e-29	8.64e-30	12.212.141

214882_s_at	6.68e-29	8.71e-30	12.211.316
213414_s_at	6.68e-29	8.71e-30	12.211.229
209180_at	6.73e-29	8.78e-30	1.221.044
204787_at	6.76e-29	8.82e-30	-1.220.992
204188_s_at	7.08e-29	9.25e-30	-12.204.712
214005_at	7.57e-29	9.89e-30	12.197.473
214500_at	7.66e-29	1.00e-29	1.219.619
201688_s_at	7.67e-29	1.00e-29	12.196.036
212381_at	7.67e-29	1.00e-29	12.195.992
204228_at	7.80e-29	1.02e-29	1.219.401
52164_at	7.87e-29	1.03e-29	-1.219.304
217786_at	7.91e-29	1.04e-29	121.925
218933_at	8.30e-29	1.09e-29	12.187.245
201774_s_at	8.32e-29	1.09e-29	12.186.973
209668_x_at	8.45e-29	1.11e-29	-12.185.256
203048_s_at	8.53e-29	1.12e-29	12.184.148
216041_x_at	8.60e-29	1.13e-29	12.183.259
211784_s_at	8.63e-29	1.13e-29	12.182.837
208828_at	8.65e-29	1.13e-29	12.182.538
203423_at	8.88e-29	1.17e-29	-12.179.627
214845_s_at	9.42e-29	1.24e-29	12.173.104
217766_s_at	9.57e-29	1.26e-29	12.171.379
218080_x_at	9.65e-29	1.27e-29	12.170.473
204484_at	9.65e-29	1.27e-29	12.170.425
210153_s_at	9.68e-29	1.27e-29	12.170.068
209780_at	1.02e-28	1.34e-29	12.164.247
210628_x_at	1.07e-28	1.40e-29	-12.159.336
212378_at	1.07e-28	1.41e-29	12.159.175
211983_x_at	1.07e-28	1.41e-29	12.158.706
203549_s_at	1.08e-28	1.42e-29	12.158.029
200032_s_at	1.10e-28	1.44e-29	12.156.273
203747_at	1.10e-28	1.45e-29	-12.155.898
220486_x_at	1.11e-28	1.47e-29	12.154.544
212180_at	1.12e-28	1.48e-29	12.153.673
210053_at	1.13e-28	1.49e-29	1.215.269
216051_x_at	1.15e-28	1.52e-29	-12.150.963
218545_at	1.16e-28	1.53e-29	12.150.277
204432_at	1.16e-28	1.53e-29	12.150.206
205768_s_at	1.21e-28	1.60e-29	-1.214.524
212086_x_at	1.23e-28	1.62e-29	12.143.615
218053_at	1.27e-28	1.68e-29	12.140.124
41512_at	1.27e-28	1.68e-29	12.140.025
212239_at	1.27e-28	1.68e-29	-12.140.013
221700_s_at	1.28e-28	1.70e-29	12.138.815

218450_at	1.30e-28	1.73e-29	-12.136.864
204639_at	1.31e-28	1.73e-29	12.136.623
211284_s_at	1.34e-28	1.77e-29	12.134.131
206496_at	1.37e-28	1.82e-29	-12.131.405
212009_s_at	1.41e-28	1.87e-29	12.127.906
218358_at	1.43e-28	1.89e-29	12.126.889
200854_at	1.50e-28	1.98e-29	-12.121.757
205231_s_at	1.52e-28	2.02e-29	-12.119.868
207826_s_at	1.54e-28	2.05e-29	-12.118.371
218315_s_at	1.55e-28	2.06e-29	12.117.777
206247_at	1.56e-28	2.08e-29	12.116.821
213047_x_at	1.57e-28	2.08e-29	12.116.608
203460_s_at	1.58e-28	2.10e-29	12.115.546
202683_s_at	1.58e-28	2.11e-29	12.115.159
210119_at	1.65e-28	2.19e-29	-1.211.082
200601_at	1.65e-28	2.20e-29	12.110.416
62987_r_at	1.71e-28	2.28e-29	-12.106.556
201546_at	1.74e-28	2.32e-29	12.104.545
212648_at	1.78e-28	2.36e-29	12.102.603
209018_s_at	1.87e-28	2.49e-29	-12.096.857
203107_x_at	1.88e-28	2.50e-29	12.096.514
212791_at	1.88e-28	2.50e-29	12.096.472
218479_s_at	1.89e-28	2.52e-29	12.095.696
203380_x_at	1.94e-28	2.59e-29	-12.092.501
220429_at	1.95e-28	2.60e-29	-12.092.141
208470_s_at	2.00e-28	2.67e-29	-12.089.183
218048_at	2.02e-28	2.70e-29	12.088.301
201599_at	2.04e-28	2.72e-29	-12.087.209
206075_s_at	2.05e-28	2.74e-29	12.086.441
205047_s_at	2.06e-28	2.75e-29	12.086.073
204252_at	2.10e-28	2.81e-29	12.083.631
214279_s_at	2.19e-28	2.93e-29	-12.079.349
201954_at	2.22e-28	2.97e-29	12.077.734
218615_s_at	2.25e-28	3.01e-29	1.207.629
204849_at	2.25e-28	3.01e-29	12.076.207
50965_at	2.28e-28	3.05e-29	-12.074.897
204818_at	2.37e-28	3.18e-29	-12.070.324
200693_at	2.37e-28	3.18e-29	12.070.297
221510_s_at	2.38e-28	3.19e-29	12.069.891
214773_x_at	2.41e-28	3.23e-29	120.685
208677_s_at	2.41e-28	3.24e-29	12.068.284
219054_at	2.44e-28	3.27e-29	-12.067.281
205077_s_at	2.51e-28	3.37e-29	1.206.393
208692_at	2.55e-28	3.43e-29	12.062.068

213752_at	2.64e-28	3.55e-29	-12.058.388
211450_s_at	2.68e-28	3.61e-29	12.056.514
200665_s_at	2.69e-28	3.62e-29	12.056.079
221835_at	2.73e-28	3.68e-29	-12.054.473
213275_x_at	2.74e-28	3.68e-29	-12.054.254
209293_x_at	2.81e-28	3.78e-29	-12.051.483
212017_at	2.81e-28	3.78e-29	12.051.432
219181_at	2.81e-28	3.79e-29	-12.051.225
218676_s_at	2.82e-28	3.80e-29	-12.050.975
212685_s_at	2.91e-28	3.92e-29	12.047.346
201586_s_at	2.93e-28	3.95e-29	12.046.655
216687_x_at	2.97e-28	4.01e-29	-12.045.067
213545_x_at	3.14e-28	4.24e-29	12.038.827
215416_s_at	3.15e-28	4.24e-29	12.038.755
211552_s_at	3.18e-28	4.29e-29	-12.037.589
219615_s_at	3.18e-28	4.29e-29	-1.203.758
396_f_at	3.30e-28	4.45e-29	-12.033.493
203222_s_at	3.38e-28	4.57e-29	12.030.741
203159_at	3.41e-28	4.61e-29	12.029.614
221205_at	3.43e-28	4.63e-29	-12.029.186
201921_at	3.68e-28	4.97e-29	12.021.384
213811_x_at	3.73e-28	5.05e-29	12.019.759
201749_at	3.75e-28	5.07e-29	-12.019.231
209153_s_at	3.79e-28	5.12e-29	12.018.132
217564_s_at	3.81e-28	5.16e-29	-12.017.401
218108_at	3.85e-28	5.21e-29	12.016.339
217396_at	3.86e-28	5.23e-29	-12.015.938
206734_at	3.87e-28	5.24e-29	12.015.687
208793_x_at	3.95e-28	5.36e-29	12.013.283
221263_s_at	3.95e-28	5.36e-29	12.013.273
214931_s_at	4.06e-28	5.51e-29	12.010.156
202001_s_at	4.20e-28	5.70e-29	12.006.586
212845_at	4.25e-28	5.77e-29	-1.200.514
215304_at	4.78e-28	6.50e-29	-11.992.136
204434_at	4.85e-28	6.59e-29	11.990.682
213822_s_at	4.86e-28	6.61e-29	11.990.304
200864_s_at	4.88e-28	6.64e-29	11.989.803
200597_at	4.96e-28	6.75e-29	1.198.794
210329_s_at	4.99e-28	6.79e-29	-11.987.363
208657_s_at	5.02e-28	6.83e-29	11.986.637
222201_s_at	5.09e-28	6.93e-29	11.985.028
206200_s_at	5.34e-28	7.27e-29	11.979.893
213786_at	5.39e-28	7.35e-29	11.978.685
206065_s_at	5.44e-28	7.41e-29	-11.977.756

215631_s_at	5.44e-28	7.41e-29	11.977.749
217965_s_at	5.45e-28	7.43e-29	11.977.442
203002_at	5.67e-28	7.74e-29	-11.973.006
201492_s_at	5.79e-28	7.90e-29	11.970.783
205774_at	5.80e-28	7.92e-29	-11.970.508
222029_x_at	5.93e-28	8.10e-29	11.967.983
201756_at	5.94e-28	8.12e-29	11.967.724
212623_at	5.97e-28	8.16e-29	11.967.219
212172_at	6.00e-28	8.20e-29	-11.966.626
202143_s_at	6.06e-28	8.29e-29	11.965.523
209803_s_at	6.21e-28	8.50e-29	11.962.743
212295_s_at	6.54e-28	8.95e-29	11.957.071
219016_at	6.60e-28	9.03e-29	11.956.031
215029_at	6.89e-28	9.44e-29	-11.951.211
205332_at	6.99e-28	9.57e-29	-11.949.715
218208_at	7.36e-28	1.01e-28	-11.943.893
206292_s_at	7.47e-28	1.02e-28	-11.942.367
208676_s_at	7.54e-28	1.03e-28	11.941.202
221200_at	7.54e-28	1.03e-28	-11.941.185
203334_at	7.54e-28	1.03e-28	11.941.183
209522_s_at	7.55e-28	1.04e-28	-11.940.956
201232_s_at	7.74e-28	1.06e-28	1.193.825
207414_s_at	7.74e-28	1.06e-28	-11.938.202
218328_at	7.76e-28	1.07e-28	-11.937.937
207707_s_at	7.85e-28	1.08e-28	11.936.505
210784_x_at	7.93e-28	1.09e-28	-11.935.388
217823_s_at	8.11e-28	1.12e-28	11.932.915
217927_at	8.14e-28	1.12e-28	11.932.425
217736_s_at	8.38e-28	1.15e-28	11.929.244
219395_at	8.39e-28	1.15e-28	-11.929.089
212053_at	8.51e-28	1.17e-28	-11.927.443
203060_s_at	8.54e-28	1.18e-28	-11.927.009
203979_at	9.08e-28	1.25e-28	-11.920.318
207392_x_at	9.21e-28	1.27e-28	-11.918.756
201314_at	9.36e-28	1.29e-28	11.916.884
207474_at	9.73e-28	1.34e-28	-11.912.593
213754_s_at	9.88e-28	1.36e-28	11.910.868
221671_x_at	9.94e-28	1.37e-28	-11.910.213
38521_at	1.05e-27	1.44e-28	-11.904.454
221063_x_at	1.10e-27	1.52e-28	-11.898.716
212493_s_at	1.13e-27	1.56e-28	11.895.995
210825_s_at	1.14e-27	1.57e-28	-11.895.206
213734_at	1.15e-27	1.59e-28	11.894.081
203517_at	1.15e-27	1.59e-28	11.894.072

221651_x_at	1.16e-27	1.61e-28	-1.189.265
201651_s_at	1.19e-27	1.65e-28	11.889.714
215447_at	1.20e-27	1.66e-28	-1.188.897
205102_at	1.20e-27	1.67e-28	-11.888.683
48531_at	1.23e-27	1.70e-28	11.886.259
205628_at	1.32e-27	1.83e-28	1.187.849
209840_s_at	1.34e-27	1.85e-28	-11.877.255
200626_s_at	1.38e-27	1.91e-28	1.187.361
201180_s_at	1.39e-27	1.93e-28	11.872.765
201729_s_at	1.42e-27	1.97e-28	11.870.366
202232_s_at	1.44e-27	1.99e-28	11.869.025
204920_at	1.49e-27	2.06e-28	-11.865.267
214657_s_at	1.50e-27	2.08e-28	-11.864.348
203434_s_at	1.53e-27	2.13e-28	-11.861.839
202384_s_at	1.54e-27	2.14e-28	11.861.111
222049_s_at	1.55e-27	2.15e-28	-11.860.712
212265_at	1.57e-27	2.18e-28	11.859.226
202560_s_at	1.59e-27	2.21e-28	11.857.598
221636_s_at	1.59e-27	2.22e-28	-11.857.392
205596_s_at	1.62e-27	2.25e-28	11.855.591
202844_s_at	1.65e-27	2.30e-28	11.853.475
212015_x_at	1.65e-27	2.30e-28	1.185.318
201736_s_at	1.68e-27	2.33e-28	11.851.669
221956_at	1.68e-27	2.34e-28	-11.851.426
213164_at	1.69e-27	2.35e-28	-11.850.807
200992_at	1.69e-27	2.36e-28	11.850.697
207688_s_at	1.70e-27	2.38e-28	11.849.623
214838_at	1.71e-27	2.39e-28	11.849.305
203722_at	1.71e-27	2.39e-28	-11.849.185
219099_at	1.78e-27	2.49e-28	1.184.466
221039_s_at	1.81e-27	2.53e-28	11.842.964
217707_x_at	1.87e-27	2.62e-28	-11.839.011
213214_x_at	1.89e-27	2.64e-28	11.838.054
200997_at	1.90e-27	2.65e-28	11.837.636
200071_at	1.94e-27	2.71e-28	11.835.154
205700_at	1.96e-27	2.75e-28	-11.833.723
212116_at	2.00e-27	2.79e-28	11.831.863
212138_at	2.00e-27	2.80e-28	11.831.805
207291_at	2.00e-27	2.81e-28	-1.183.139
221607_x_at	2.02e-27	2.83e-28	11.830.325
204129_at	2.06e-27	2.88e-28	11.828.505
214955_at	2.06e-27	2.89e-28	-11.828.161
208941_s_at	2.10e-27	2.94e-28	11.826.296
217962_at	2.13e-27	2.99e-28	11.824.434

205649_s_at	2.17e-27	3.04e-28	-11.822.534
208091_s_at	2.22e-27	3.11e-28	11.819.906
203608_at	2.32e-27	3.26e-28	-11.814.814
204141_at	2.33e-27	3.27e-28	11.814.457
208393_s_at	2.39e-27	3.35e-28	11.811.828
217487_x_at	2.41e-27	3.39e-28	-11.810.658
32032_at	2.45e-27	3.45e-28	11.808.579
203645_s_at	2.48e-27	3.48e-28	-11.807.558
207483_s_at	2.48e-27	3.48e-28	11.807.514
201345_s_at	2.51e-27	3.53e-28	11.806.038
213526_s_at	2.56e-27	3.60e-28	1.180.375
212038_s_at	2.56e-27	3.61e-28	11.803.713
201606_s_at	2.59e-27	3.65e-28	1.180.233
211375_s_at	2.69e-27	3.79e-28	11.798.333
209175_at	2.70e-27	3.81e-28	11.797.611
212898_at	2.71e-27	3.82e-28	11.797.423
203165_s_at	2.75e-27	3.88e-28	11.795.745
218352_at	2.84e-27	4.00e-28	11.792.153
219165_at	2.92e-27	4.12e-28	-11.788.994
219329_s_at	2.96e-27	4.18e-28	11.787.267
200695_at	3.05e-27	4.31e-28	11.783.929
218245_at	3.06e-27	4.32e-28	-11.783.846
203987_at	3.06e-27	4.32e-28	11.783.808
214433_s_at	3.08e-27	4.35e-28	-11.783.003
207200_at	3.10e-27	4.38e-28	-11.782.168
212348_s_at	3.10e-27	4.39e-28	11.782.079
206245_s_at	3.12e-27	4.41e-28	11.781.498
203878_s_at	3.13e-27	4.44e-28	1.178.084
202800_at	3.14e-27	4.44e-28	11.780.636
211340_s_at	3.15e-27	4.46e-28	11.780.339
220925_at	3.18e-27	4.51e-28	11.779.103
205749_at	3.26e-27	4.62e-28	-11.776.296
213073_at	3.28e-27	4.65e-28	11.775.694
202337_at	3.29e-27	4.66e-28	11.775.443
212193_s_at	3.29e-27	4.66e-28	11.775.375
212544_at	3.31e-27	4.70e-28	1.177.447
202104_s_at	3.40e-27	4.82e-28	-11.771.619
202034_x_at	3.47e-27	4.93e-28	11.769.151
221258_s_at	3.52e-27	5.00e-28	11.767.618
202299_s_at	3.60e-27	5.11e-28	11.765.092
207974_s_at	3.69e-27	5.24e-28	11.762.391
217981_s_at	4.00e-27	5.68e-28	11.753.494
201661_s_at	4.02e-27	5.71e-28	11.752.891
217987_at	4.14e-27	5.88e-28	11.749.574

213578_at	4.17e-27	5.93e-28	11.748.653
202330_s_at	4.22e-27	6.00e-28	11.747.375
202721_s_at	4.28e-27	6.10e-28	11.745.631
201066_at	4.30e-27	6.12e-28	11.745.152
217948_at	4.39e-27	6.25e-28	11.742.915
202649_x_at	4.73e-27	6.74e-28	11.734.624
201035_s_at	4.80e-27	6.84e-28	-11.732.842
205883_at	4.82e-27	6.87e-28	-11.732.456
213507_s_at	4.98e-27	7.10e-28	11.728.733
209456_s_at	5.01e-27	7.14e-28	-11.728.152
218385_at	5.10e-27	7.28e-28	11.726.023
205960_at	5.13e-27	7.32e-28	-11.725.455
213146_at	5.16e-27	7.37e-28	-11.724.718
207436_x_at	5.38e-27	7.69e-28	-11.720.001
201595_s_at	5.66e-27	8.08e-28	11.714.429
213918_s_at	5.68e-27	8.11e-28	11.713.999
217907_at	6.12e-27	8.75e-28	1.170.565
206410_at	6.20e-27	8.87e-28	-11.704.087
201550_x_at	6.44e-27	9.21e-28	11.699.902
207023_x_at	6.53e-27	9.34e-28	11.698.403
209502_s_at	6.70e-27	9.59e-28	-11.695.498
210519_s_at	6.79e-27	9.72e-28	1.169.395
217999_s_at	6.91e-27	9.90e-28	-11.691.983
220147_s_at	6.96e-27	9.98e-28	11.691.046
204121_at	7.00e-27	1.00e-27	-11.690.425
201443_s_at	7.04e-27	1.01e-27	11.689.845
203133_at	7.29e-27	1.05e-27	11.685.934
202808_at	7.41e-27	1.06e-27	-11.683.959
209020_at	7.47e-27	1.07e-27	11.683.087
202231_at	7.71e-27	1.11e-27	11.679.575
201802_at	7.74e-27	1.11e-27	11.679.099
200993_at	7.76e-27	1.11e-27	11.678.822
208891_at	7.83e-27	1.13e-27	-11.677.682
202956_at	7.95e-27	1.14e-27	11.675.993
201763_s_at	7.95e-27	1.14e-27	11.675.951
213631_x_at	8.04e-27	1.16e-27	-11.674.683
206010_at	8.04e-27	1.16e-27	-11.674.632
205935_at	8.19e-27	1.18e-27	-11.672.586
205730_s_at	8.25e-27	1.19e-27	-11.671.779
202536_at	8.26e-27	1.19e-27	11.671.508
211707_s_at	8.69e-27	1.25e-27	11.665.885
212230_at	8.82e-27	1.27e-27	-11.664.145
221031_s_at	8.94e-27	1.29e-27	11.662.701
219676_at	9.13e-27	1.32e-27	11.660.268

205922_at	9.19e-27	1.33e-27	11.659.517
204247_s_at	9.29e-27	1.34e-27	11.658.274
214948_s_at	9.48e-27	1.37e-27	11.656.026
208813_at	9.51e-27	1.37e-27	-11.655.568
213359_at	9.61e-27	1.39e-27	-11.654.445
222099_s_at	9.70e-27	1.40e-27	11.653.386
213523_at	9.92e-27	1.43e-27	11.650.827
210753_s_at	1.01e-26	1.46e-27	-11.648.793
201358_s_at	1.01e-26	1.47e-27	11.648.324
201643_x_at	1.05e-26	1.51e-27	11.644.828
201248_s_at	1.08e-26	1.56e-27	11.641.709
200042_at	1.08e-26	1.56e-27	1.164.122
201603_at	1.13e-26	1.64e-27	11.636.168
208833_s_at	1.13e-26	1.64e-27	11.635.646
216232_s_at	1.18e-26	1.71e-27	11.631.529
218679_s_at	1.25e-26	1.82e-27	11.624.556
221009_s_at	1.36e-26	1.97e-27	-11.615.738
201689_s_at	1.39e-26	2.01e-27	11.613.244
221532_s_at	1.41e-26	2.04e-27	11.611.335
205133_s_at	1.43e-26	2.07e-27	11.610.058
220647_s_at	1.45e-26	2.11e-27	11.607.984
202537_s_at	1.46e-26	2.11e-27	1.160.767
221517_s_at	1.48e-26	2.15e-27	11.605.879
206593_s_at	1.50e-26	2.18e-27	1.160.404
218008_at	1.55e-26	2.26e-27	11.600.359
201543_s_at	1.59e-26	2.30e-27	11.598.023
219341_at	1.64e-26	2.38e-27	-11.594.477
212988_x_at	1.64e-26	2.39e-27	11.593.942
202968_s_at	1.65e-26	2.41e-27	11.593.203
210652_s_at	1.75e-26	2.54e-27	11.587.014
205995_x_at	1.88e-26	2.74e-27	11.578.598
211271_x_at	1.91e-26	2.78e-27	11.577.221
206618_at	1.91e-26	2.78e-27	-11.577.014
209043_at	1.93e-26	2.82e-27	11.575.613
201483_s_at	1.95e-26	2.85e-27	11.574.471
218132_s_at	1.99e-26	2.89e-27	11.572.584
207071_s_at	2.04e-26	2.98e-27	-11.569.348
203328_x_at	2.06e-26	3.00e-27	11.568.458
204098_at	2.08e-26	3.04e-27	11.567.273
222035_s_at	2.08e-26	3.04e-27	11.567.179
222230_s_at	2.14e-26	3.13e-27	11.564.036
205528_s_at	2.15e-26	3.13e-27	-11.563.716
217796_s_at	2.15e-26	3.14e-27	11.563.332
201942_s_at	2.16e-26	3.16e-27	11.562.778

219494_at	2.21e-26	3.24e-27	115.601
207354_at	2.24e-26	3.27e-27	-11.559.005
220906_at	2.24e-26	3.28e-27	-11.558.794
212676_at	2.25e-26	3.29e-27	11.558.382
213409_s_at	2.25e-26	3.29e-27	-11.558.241
201416_at	2.26e-26	3.31e-27	1.155.771
212448_at	2.31e-26	3.38e-27	11.555.331
214590_s_at	2.32e-26	3.39e-27	11.554.804
214669_x_at	2.39e-26	3.51e-27	-115.512
211932_at	2.41e-26	3.53e-27	11.550.398
219525_at	2.47e-26	3.63e-27	-11.547.424
218643_s_at	2.49e-26	3.66e-27	11.546.524
218374_s_at	2.53e-26	3.72e-27	11.544.586
211945_s_at	2.54e-26	3.73e-27	11.544.431
217685_at	2.55e-26	3.74e-27	-11.543.947
213186_at	2.56e-26	3.76e-27	11.543.411
203207_s_at	2.59e-26	3.80e-27	11.542.159
204868_at	2.60e-26	3.82e-27	11.541.538
220507_s_at	2.63e-26	3.87e-27	-11.540.312
209387_s_at	2.69e-26	3.96e-27	11.537.587
204091_at	2.79e-26	4.11e-27	11.533.417
200039_s_at	2.88e-26	4.24e-27	1.152.998
205054_at	2.89e-26	4.26e-27	11.529.476
204017_at	2.93e-26	4.32e-27	11.527.975
202971_s_at	2.98e-26	4.40e-27	1.152.592
219493_at	3.01e-26	4.43e-27	11.525.117
212428_at	3.07e-26	4.52e-27	11.522.836
209291_at	3.09e-26	4.55e-27	-1.152.201
209082_s_at	3.09e-26	4.56e-27	-11.521.798
214148_at	3.18e-26	4.69e-27	-11.518.591
207628_s_at	3.24e-26	4.78e-27	11.516.535
205562_at	3.47e-26	5.12e-27	11.508.844
211318_s_at	3.50e-26	5.17e-27	11.507.879
209944_at	3.50e-26	5.18e-27	11.507.653
204225_at	3.53e-26	5.22e-27	11.506.773
207072_at	3.60e-26	5.32e-27	-11.504.616
207821_s_at	3.62e-26	5.35e-27	11.504.027
212228_s_at	3.63e-26	5.36e-27	-11.503.678
219715_s_at	3.66e-26	5.42e-27	11.502.588
200933_x_at	3.89e-26	5.76e-27	11.495.728
200625_s_at	3.96e-26	5.87e-27	11.493.669
212131_at	4.03e-26	5.96e-27	11.491.875
213276_at	4.11e-26	6.09e-27	-11.489.498
266_s_at	4.20e-26	6.22e-27	11.487.048

206226_at	4.24e-26	6.29e-27	-11.485.875
219066_at	4.25e-26	6.30e-27	11.485.683
219396_s_at	4.63e-26	6.87e-27	-11.475.976
209678_s_at	4.70e-26	6.97e-27	11.474.317
205304_s_at	4.71e-26	6.99e-27	-11.474.081
218977_s_at	4.71e-26	7.00e-27	11.473.921
213452_at	4.74e-26	7.03e-27	11.473.341
201717_at	4.78e-26	7.10e-27	11.472.355
209912_s_at	4.81e-26	7.15e-27	11.471.541
209392_at	4.89e-26	7.26e-27	1.146.975
209549_s_at	5.00e-26	7.44e-27	11.467.072
221622_s_at	5.03e-26	7.47e-27	11.466.526
221287_at	5.04e-26	7.50e-27	-11.466.154
204766_s_at	5.10e-26	7.59e-27	11.464.732
220926_s_at	5.12e-26	7.61e-27	11.464.462
219372_at	5.19e-26	7.73e-27	11.462.772
209925_at	5.26e-26	7.83e-27	11.461.322
212867_at	5.30e-26	7.90e-27	11.460.297
217832_at	5.40e-26	8.05e-27	11.458.179
218483_s_at	5.47e-26	8.15e-27	-11.456.803
206966_s_at	5.51e-26	8.22e-27	-11.455.857
205756_s_at	5.66e-26	8.44e-27	-11.452.842
218632_at	5.72e-26	8.53e-27	-11.451.746
200700_s_at	5.72e-26	8.53e-27	11.451.648
217742_s_at	5.81e-26	8.67e-27	11.449.856
212578_x_at	5.87e-26	8.76e-27	1.144.873
215693_x_at	6.06e-26	9.05e-27	11.445.053
220794_at	6.14e-26	9.17e-27	-11.443.535
201992_s_at	6.23e-26	9.32e-27	11.441.807
205394_at	6.45e-26	9.64e-27	11.438.022
214774_x_at	6.66e-26	9.96e-27	11.434.372
210005_at	6.68e-26	9.99e-27	11.433.976
214167_s_at	6.75e-26	1.01e-26	11.432.743
204303_s_at	6.81e-26	1.02e-26	-11.431.787
217982_s_at	7.01e-26	1.05e-26	1.142.844
203109_at	7.02e-26	1.05e-26	11.428.233
221562_s_at	7.13e-26	1.07e-26	-11.426.443
209666_s_at	7.25e-26	1.09e-26	11.424.554
203525_s_at	7.30e-26	1.09e-26	11.423.681
207606_s_at	7.41e-26	1.11e-26	11.422.081
216224_s_at	7.43e-26	1.11e-26	-11.421.695
210225_x_at	7.48e-26	1.12e-26	-11.420.991
208928_at	7.88e-26	1.18e-26	-11.415.076
204227_s_at	8.06e-26	1.21e-26	-11.412.381

38964_r_at	8.06e-26	1.21e-26	-11.412.363
211964_at	8.30e-26	1.25e-26	11.409.042
211737_x_at	8.36e-26	1.26e-26	-11.408.262
201243_s_at	8.41e-26	1.26e-26	1.140.749
202412_s_at	8.55e-26	1.29e-26	11.405.604
210009_s_at	8.62e-26	1.30e-26	11.404.658
219644_at	9.13e-26	1.37e-26	11.398.243
202467_s_at	9.28e-26	1.40e-26	11.396.403
212841_s_at	9.33e-26	1.40e-26	-1.139.574
217073_x_at	9.34e-26	1.41e-26	-11.395.556
201588_at	9.42e-26	1.42e-26	11.394.553
220948_s_at	9.64e-26	1.45e-26	11.391.986
201290_at	9.65e-26	1.45e-26	11.391.738
201051_at	9.88e-26	1.49e-26	11.389.086
200619_at	1.00e-25	1.51e-26	11.387.472
207762_at	1.04e-25	1.56e-26	-11.383.584
214812_s_at	1.04e-25	1.57e-26	1.138.332
209171_at	1.05e-25	1.58e-26	11.382.282
219248_at	1.10e-25	1.66e-26	11.376.656
215452_x_at	1.13e-25	1.70e-26	11.374.241
218089_at	1.14e-25	1.73e-26	11.372.544
220206_at	1.14e-25	1.73e-26	11.372.504
45828_at	1.16e-25	1.75e-26	11.370.767
212836_at	1.16e-25	1.76e-26	11.370.522
217857_s_at	1.17e-25	1.77e-26	11.369.834
201440_at	1.24e-25	1.88e-26	11.362.873
203058_s_at	1.33e-25	2.01e-26	-11.355.377
204882_at	1.34e-25	2.03e-26	-11.354.127
208800_at	1.35e-25	2.05e-26	11.353.195
200893_at	1.37e-25	2.07e-26	11.352.059
212523_s_at	1.38e-25	2.09e-26	11.350.832
215526_at	1.39e-25	2.11e-26	-11.350.065
204762_s_at	1.41e-25	2.14e-26	-11.348.536
215706_x_at	1.43e-25	2.16e-26	11.346.997
203327_at	1.44e-25	2.18e-26	11.346.154
201690_s_at	1.45e-25	2.20e-26	1.134.511
204569_at	1.45e-25	2.20e-26	11.344.945
218095_s_at	1.47e-25	2.23e-26	11.343.775
202036_s_at	1.49e-25	2.27e-26	-11.341.847
215873_x_at	1.52e-25	2.31e-26	11.339.756
222186_at	1.54e-25	2.34e-26	-11.338.291
206451_at	1.54e-25	2.35e-26	1.133.791
201263_at	1.55e-25	2.36e-26	11.337.275
214470_at	1.56e-25	2.37e-26	-11.336.774

213984_at	1.60e-25	2.43e-26	11.334.033
218439_s_at	1.60e-25	2.44e-26	11.333.666
218196_at	1.64e-25	2.49e-26	11.331.139
205566_at	1.68e-25	2.55e-26	-11.328.493
204450_x_at	1.72e-25	2.62e-26	-11.325.487
212293_at	1.73e-25	2.64e-26	11.324.527
206767_at	1.79e-25	2.73e-26	-11.320.753
221541_at	1.80e-25	2.75e-26	-1.132.012
208156_x_at	1.81e-25	2.76e-26	11.319.696
217814_at	1.85e-25	2.82e-26	11.317.319
204929_s_at	1.85e-25	2.82e-26	11.317.069
204775_at	1.85e-25	2.83e-26	11.316.976
219527_at	1.86e-25	2.84e-26	-11.316.375
215121_x_at	1.86e-25	2.85e-26	-11.316.172
219195_at	1.91e-25	2.91e-26	-11.313.589
218172_s_at	1.97e-25	3.00e-26	11.310.058
220854_at	2.00e-25	3.05e-26	-11.308.198
217301_x_at	2.00e-25	3.06e-26	11.307.789
202365_at	2.03e-25	3.10e-26	11.306.581
221858_at	2.06e-25	3.15e-26	11.304.655
207108_s_at	2.08e-25	3.19e-26	11.303.324
210839_s_at	2.16e-25	3.31e-26	11.299.013
213287_s_at	2.18e-25	3.33e-26	11.298.307
207761_s_at	2.26e-25	3.46e-26	-11.294.105
204621_s_at	2.27e-25	3.47e-26	-11.293.683
218158_s_at	2.33e-25	3.58e-26	11.290.349
207353_s_at	2.36e-25	3.61e-26	-11.289.213
218862_at	2.51e-25	3.85e-26	-11.282.141
209358_at	2.52e-25	3.86e-26	11.281.611
208985_s_at	2.55e-25	3.92e-26	11.279.996
208936_x_at	2.56e-25	3.93e-26	11.279.799
214672_at	2.59e-25	3.97e-26	11.278.419
209600_s_at	2.60e-25	3.99e-26	-1.127.804
218138_at	2.68e-25	4.12e-26	11.274.225
218160_at	2.70e-25	4.14e-26	11.273.725
212342_at	2.72e-25	4.18e-26	11.272.637
205043_at	2.74e-25	4.21e-26	-11.271.918
206952_at	2.75e-25	4.23e-26	-1.127.137
214173_x_at	2.86e-25	4.39e-26	1.126.707
208301_at	2.90e-25	4.47e-26	-11.265.107
206293_at	2.94e-25	4.52e-26	-11.263.866
201696_at	2.95e-25	4.54e-26	-11.263.331
207498_s_at	3.00e-25	4.63e-26	-11.261.256
204168_at	3.02e-25	4.65e-26	-11.260.549

212010_s_at	3.05e-25	4.70e-26	11.259.461
209070_s_at	3.07e-25	4.73e-26	11.258.737
213374_x_at	3.07e-25	4.74e-26	-11.258.593
205656_at	3.07e-25	4.74e-26	11.258.509
201172_x_at	3.12e-25	4.81e-26	11.256.797
206132_at	3.15e-25	4.87e-26	-11.255.514
208949_s_at	3.17e-25	4.90e-26	11.254.798
204603_at	3.59e-25	5.54e-26	11.240.759
213981_at	3.67e-25	5.67e-26	-11.238.197
200624_s_at	3.72e-25	5.75e-26	11.236.608
218799_at	3.77e-25	5.83e-26	11.235.029
219375_at	3.77e-25	5.83e-26	-11.234.958
212100_s_at	3.78e-25	5.85e-26	11.234.675
219818_s_at	3.85e-25	5.96e-26	1.123.248
219803_at	3.86e-25	5.97e-26	-11.232.412
219600_s_at	3.92e-25	6.06e-26	11.230.568
213927_at	3.94e-25	6.10e-26	11.229.977
221942_s_at	3.96e-25	6.14e-26	-11.229.155
201245_s_at	4.03e-25	6.24e-26	11.227.392
212751_at	4.04e-25	6.26e-26	11.227.005
209927_s_at	4.16e-25	6.44e-26	11.223.704
220248_x_at	4.32e-25	6.70e-26	11.219.255
204226_at	4.38e-25	6.79e-26	112.177
206702_at	4.43e-25	6.87e-26	-11.216.424
211615_s_at	4.45e-25	6.91e-26	11.215.794
221983_at	4.57e-25	7.10e-26	11.212.668
216259_at	4.57e-25	7.10e-26	-11.212.647
216515_x_at	4.57e-25	7.10e-26	11.212.633
202667_s_at	4.61e-25	7.16e-26	11.211.709
210988_s_at	4.66e-25	7.25e-26	11.210.361
201574_at	4.69e-25	7.30e-26	11.209.608
219902_at	4.76e-25	7.40e-26	-11.207.991
218464_s_at	4.78e-25	7.44e-26	11.207.381
212520_s_at	4.85e-25	7.55e-26	11.205.722
215662_at	5.03e-25	7.83e-26	-1.120.162
210112_at	5.06e-25	7.88e-26	11.200.834
218499_at	5.06e-25	7.89e-26	11.200.771
220106_at	5.12e-25	7.97e-26	-11.199.549
214022_s_at	5.13e-25	8.00e-26	-11.199.165
32029_at	5.16e-25	8.05e-26	-11.198.448
204537_s_at	5.23e-25	8.15e-26	11.197.065
206118_at	5.32e-25	8.30e-26	-11.195.015
212949_at	5.48e-25	8.56e-26	1.119.152
203276_at	5.50e-25	8.59e-26	11.191.098

49327_at	5.65e-25	8.82e-26	-11.188.026
220202_s_at	6.14e-25	9.59e-26	11.178.513
203335_at	6.17e-25	9.64e-26	-11.178.024
205037_at	6.17e-25	9.64e-26	1.117.795
209181_s_at	6.29e-25	9.83e-26	11.175.712
206666_at	6.34e-25	9.91e-26	-1.117.487
200663_at	6.43e-25	1.01e-25	11.173.182
204000_at	6.47e-25	1.01e-25	11.172.383
212839_s_at	6.58e-25	1.03e-25	11.170.495
211767_at	6.62e-25	1.04e-25	11.169.862
209864_at	6.66e-25	1.04e-25	11.169.042
206592_s_at	6.85e-25	1.07e-25	1.116.589
203927_at	6.88e-25	1.08e-25	11.165.315
219222_at	7.03e-25	1.10e-25	-11.162.752
210612_s_at	7.07e-25	1.11e-25	11.162.082
215004_s_at	7.16e-25	1.12e-25	11.160.569
213773_x_at	7.16e-25	1.12e-25	11.160.547
211962_s_at	7.16e-25	1.12e-25	-11.160.538
218996_at	7.17e-25	1.13e-25	11.160.367
218072_at	7.22e-25	1.13e-25	11.159.606
205777_at	7.63e-25	1.20e-25	11.153.293
34846_at	7.63e-25	1.20e-25	-11.153.185
203655_at	7.64e-25	1.20e-25	11.153.124
212399_s_at	7.91e-25	1.24e-25	11.149.146
218299_at	8.17e-25	1.28e-25	-11.145.387
218767_at	8.22e-25	1.29e-25	11.144.623
218448_at	8.51e-25	1.34e-25	11.140.696
209302_at	8.54e-25	1.34e-25	11.140.274
204988_at	8.55e-25	1.34e-25	-11.140.108
204694_at	8.60e-25	1.35e-25	11.139.367
218262_at	8.86e-25	1.39e-25	11.135.939
216071_x_at	8.97e-25	1.41e-25	11.134.499
219193_at	8.97e-25	1.41e-25	1.113.442
201463_s_at	9.17e-25	1.45e-25	11.131.892
213488_at	9.40e-25	1.48e-25	-11.129.065
213420_at	9.44e-25	1.49e-25	11.128.543
46142_at	9.50e-25	1.50e-25	-11.127.804
210215_at	9.91e-25	1.56e-25	-11.122.977
207470_at	1.03e-24	1.63e-25	-1.111.826
201468_s_at	1.04e-24	1.64e-25	11.117.517
215054_at	1.05e-24	1.65e-25	-11.116.709
33760_at	1.06e-24	1.68e-25	-11.114.919
201476_s_at	1.07e-24	1.70e-25	11.113.641
201623_s_at	1.08e-24	1.71e-25	11.112.812

215999_at	1.13e-24	1.78e-25	-11.108.122
218684_at	1.14e-24	1.79e-25	11.107.227
216840_s_at	1.15e-24	1.83e-25	-11.105.272
218065_s_at	1.16e-24	1.83e-25	11.104.833
209081_s_at	1.16e-24	1.84e-25	-11.104.538
216095_x_at	1.19e-24	1.88e-25	11.102.155
213361_at	1.19e-24	1.89e-25	11.101.396
210689_at	1.21e-24	1.92e-25	-11.099.816
200641_s_at	1.25e-24	1.98e-25	11.096.098
213188_s_at	1.30e-24	2.07e-25	11.091.187
216062_at	1.31e-24	2.08e-25	-11.090.292
219078_at	1.34e-24	2.12e-25	11.088.276
221780_s_at	1.36e-24	2.16e-25	11.086.236
204476_s_at	1.38e-24	2.19e-25	-11.084.473
201481_s_at	1.44e-24	2.28e-25	11.080.044
218564_at	1.44e-24	2.28e-25	11.079.965
214753_at	1.44e-24	2.29e-25	-11.079.428
218149_s_at	1.45e-24	2.31e-25	-11.078.404
218593_at	1.46e-24	2.32e-25	11.078.044
212637_s_at	1.47e-24	2.34e-25	11.076.807
214465_at	1.49e-24	2.37e-25	-11.075.457
212625_at	1.49e-24	2.38e-25	11.075.215
218398_at	1.53e-24	2.43e-25	11.072.471
204171_at	1.53e-24	2.43e-25	11.072.427
209653_at	1.54e-24	2.45e-25	11.071.702
205354_at	1.55e-24	2.46e-25	-11.071.105
212249_at	1.55e-24	2.48e-25	-11.070.523
212892_at	1.56e-24	2.48e-25	1.107.026
219191_s_at	1.59e-24	2.53e-25	-11.067.996
209463_s_at	1.59e-24	2.54e-25	11.067.572
215620_at	1.65e-24	2.62e-25	-11.063.876
202806_at	1.67e-24	2.67e-25	11.061.876
208024_s_at	1.71e-24	2.72e-25	-11.059.664
209138_x_at	1.72e-24	2.74e-25	-11.059.014
204831_at	1.72e-24	2.75e-25	11.058.496
209667_at	1.72e-24	2.75e-25	-11.058.367
201469_s_at	1.77e-24	2.83e-25	11.055.115
206574_s_at	1.78e-24	2.84e-25	11.054.923
64883_at	1.78e-24	2.85e-25	11.054.559
221676_s_at	1.81e-24	2.89e-25	11.052.679
215171_s_at	1.82e-24	2.91e-25	11.052.182
205844_at	1.82e-24	2.91e-25	-11.051.999
220075_s_at	1.83e-24	2.93e-25	-1.105.116
203343_at	1.97e-24	3.16e-25	11.042.789

221474_at	1.98e-24	3.17e-25	11.042.106
202397_at	2.05e-24	3.29e-25	11.038.046
209695_at	2.05e-24	3.29e-25	11.037.982
212266_s_at	2.08e-24	3.34e-25	-11.036.302
219559_at	2.13e-24	3.41e-25	-11.033.865
220235_s_at	2.16e-24	3.47e-25	11.032.021
218263_s_at	2.17e-24	3.48e-25	1.103.155
207830_s_at	2.17e-24	3.48e-25	11.031.531
208651_x_at	2.18e-24	3.50e-25	11.030.865
202453_s_at	2.20e-24	3.53e-25	11.029.933
208972_s_at	2.24e-24	3.60e-25	11.027.841
203162_s_at	2.28e-24	3.66e-25	1.102.569
221580_s_at	2.30e-24	3.69e-25	11.024.986
212674_s_at	2.30e-24	3.70e-25	11.024.565
202957_at	2.38e-24	3.82e-25	-11.020.948
204291_at	2.38e-24	3.83e-25	11.020.518
214677_x_at	2.40e-24	3.85e-25	-11.019.976
209662_at	2.40e-24	3.86e-25	11.019.848
208953_at	2.52e-24	4.05e-25	11.014.191
213094_at	2.57e-24	4.13e-25	-11.011.866
217938_s_at	2.57e-24	4.14e-25	11.011.631
201533_at	2.65e-24	4.26e-25	11.008.318
212793_at	2.65e-24	4.27e-25	-11.008.074
208861_s_at	2.70e-24	4.35e-25	11.005.928
221136_at	2.75e-24	4.44e-25	-11.003.784
215379_x_at	2.76e-24	4.45e-25	-1.100.334
201422_at	2.77e-24	4.47e-25	11.002.894
218124_at	2.81e-24	4.54e-25	-11.001.226
213504_at	2.84e-24	4.58e-25	1.100.016
204593_s_at	2.89e-24	4.66e-25	10.998.229
206873_at	2.93e-24	4.73e-25	-10.996.314
212746_s_at	2.97e-24	4.79e-25	10.994.965
210253_at	3.02e-24	4.87e-25	10.993.054
218458_at	3.02e-24	4.89e-25	10.992.694
211994_at	3.05e-24	4.93e-25	10.991.571
202620_s_at	3.14e-24	5.07e-25	10.988.517
208839_s_at	3.28e-24	5.30e-25	10.983.434
203867_s_at	3.34e-24	5.40e-25	10.981.277
202404_s_at	3.39e-24	5.48e-25	10.979.455
201433_s_at	3.40e-24	5.50e-25	10.979.118
216396_s_at	3.43e-24	5.55e-25	-10.977.979
210978_s_at	3.50e-24	5.66e-25	10.975.806
200018_at	3.52e-24	5.70e-25	10.974.938
218027_at	3.60e-24	5.83e-25	10.972.394

212687_at	3.64e-24	5.90e-25	10.971.026
34697_at	3.77e-24	6.10e-25	-10.967.189
36936_at	3.83e-24	6.21e-25	1.096.527
202937_x_at	3.96e-24	6.43e-25	10.961.242
201054_at	4.04e-24	6.56e-25	10.958.901
205089_at	4.11e-24	6.67e-25	10.956.982
201665_x_at	4.14e-24	6.71e-25	10.956.216
218703_at	4.17e-24	6.77e-25	1.095.529
212255_s_at	4.28e-24	6.95e-25	10.952.278
209154_at	4.39e-24	7.13e-25	10.949.251
203364_s_at	4.40e-24	7.16e-25	10.948.875
216274_s_at	4.44e-24	7.21e-25	10.947.979
214295_at	4.52e-24	7.36e-25	-1.094.573
219404_at	4.55e-24	7.40e-25	10.945.069
200847_s_at	4.59e-24	7.47e-25	-10.943.915
201660_at	4.61e-24	7.50e-25	10.943.532
201503_at	4.65e-24	7.56e-25	10.942.542
213083_at	4.69e-24	7.64e-25	10.941.361
218311_at	4.72e-24	7.68e-25	10.940.797
211100_x_at	4.73e-24	7.70e-25	-10.940.434
214967_at	4.74e-24	7.73e-25	-10.940.081
203904_x_at	4.75e-24	7.75e-25	-10.939.762
212257_s_at	4.81e-24	7.83e-25	-109.385
217016_x_at	4.86e-24	7.93e-25	-10.937.057
201562_s_at	5.11e-24	8.34e-25	-10.931.321
201399_s_at	5.25e-24	8.57e-25	10.928.131
219342_at	5.27e-24	8.60e-25	10.927.694
211430_s_at	5.38e-24	8.79e-25	-10.925.271
205074_at	5.54e-24	9.04e-25	10.921.998
216212_s_at	5.59e-24	9.14e-25	10.920.789
201093_x_at	5.64e-24	9.22e-25	-10.919.729
201817_at	5.66e-24	9.25e-25	10.919.347
207153_s_at	5.84e-24	9.54e-25	10.915.809
204041_at	5.86e-24	9.58e-25	-10.915.338
220383_at	5.97e-24	9.77e-25	-10.913.077
212986_s_at	6.01e-24	9.82e-25	10.912.434
37462_i_at	6.02e-24	9.85e-25	10.912.114
206133_at	6.04e-24	9.89e-25	-10.911.652
216623_x_at	6.13e-24	1.00e-24	1.091.004
203914_x_at	6.18e-24	1.01e-24	-10.909.087
205719_s_at	6.26e-24	1.02e-24	-10.907.561
211009_s_at	6.27e-24	1.03e-24	10.907.182
208956_x_at	6.66e-24	1.09e-24	10.900.319
221797_at	6.69e-24	1.10e-24	10.899.704

206756_at	6.77e-24	1.11e-24	-10.898.406
210633_x_at	6.79e-24	1.11e-24	10.898.038
204461_x_at	6.98e-24	1.15e-24	10.894.751
217746_s_at	7.00e-24	1.15e-24	10.894.452
201570_at	7.02e-24	1.15e-24	10.893.958
202753_at	7.18e-24	1.18e-24	10.891.409
215126_at	7.24e-24	1.19e-24	-10.890.381
212361_s_at	7.24e-24	1.19e-24	10.890.354
221547_at	7.26e-24	1.19e-24	10.890.079
219824_at	7.34e-24	1.21e-24	-10.888.773
208107_s_at	7.54e-24	1.24e-24	10.885.617
207719_x_at	7.55e-24	1.24e-24	10.885.492
218486_at	7.57e-24	1.25e-24	-10.885.052
204578_at	7.60e-24	1.25e-24	10.884.623
200720_s_at	7.65e-24	1.26e-24	10.883.774
54037_at	7.83e-24	1.29e-24	10.881.139
203744_at	7.88e-24	1.30e-24	10.880.274
202606_s_at	7.92e-24	1.30e-24	10.879.756
202094_at	7.92e-24	1.30e-24	10.879.711
213296_at	8.18e-24	1.35e-24	-10.875.873
218070_s_at	8.31e-24	1.37e-24	10.874.124
222375_at	8.37e-24	1.38e-24	-10.873.221
203869_at	8.41e-24	1.39e-24	108.726
212964_at	8.48e-24	1.40e-24	10.871.679
203600_s_at	8.51e-24	1.41e-24	10.871.152
211487_x_at	8.64e-24	1.43e-24	10.869.367
203936_s_at	8.75e-24	1.44e-24	10.867.984
200051_at	8.93e-24	1.47e-24	10.865.587
212532_s_at	9.19e-24	1.52e-24	10.862.186
36553_at	9.20e-24	1.52e-24	-10.862.015
202449_s_at	9.24e-24	1.53e-24	-10.861.485
210499_s_at	9.41e-24	1.56e-24	-10.859.367
218335_x_at	9.50e-24	1.57e-24	10.858.225
209479_at	9.53e-24	1.58e-24	-10.857.879
209297_at	9.55e-24	1.58e-24	-10.857.547
206698_at	9.64e-24	1.60e-24	10.856.479
200059_s_at	9.67e-24	1.60e-24	10.856.121
214527_s_at	9.70e-24	1.61e-24	10.855.635
207644_at	1.03e-23	1.70e-24	-10.849.031
210594_x_at	1.04e-23	1.72e-24	10.848.048
207883_s_at	1.04e-23	1.72e-24	-10.847.938
221191_at	1.06e-23	1.75e-24	-10.845.767
218011_at	1.06e-23	1.76e-24	10.845.192
212194_s_at	1.08e-23	1.79e-24	10.843.102

214836_x_at	1.08e-23	1.80e-24	-10.842.678
218151_x_at	1.08e-23	1.80e-24	10.842.514
201816_s_at	1.09e-23	1.82e-24	10.841.584
209503_s_at	1.10e-23	1.82e-24	10.841.315
209875_s_at	1.10e-23	1.82e-24	10.841.106
208095_s_at	1.11e-23	1.84e-24	10.839.947
211944_at	1.11e-23	1.84e-24	10.839.749
218329_at	1.11e-23	1.85e-24	10.839.625
210660_at	1.13e-23	1.88e-24	-10.837.515
216583_x_at	1.18e-23	1.97e-24	10.832.425
201528_at	1.19e-23	1.98e-24	10.831.424
201096_s_at	1.19e-23	1.98e-24	10.831.309
209649_at	1.19e-23	1.99e-24	10.831.258
205158_at	1.22e-23	2.03e-24	-10.828.729
204597_x_at	1.24e-23	2.07e-24	10.826.515
215573_at	1.26e-23	2.10e-24	-10.824.553
202424_at	1.27e-23	2.12e-24	1.082.388
209694_at	1.28e-23	2.13e-24	-10.823.007
221452_s_at	1.29e-23	2.15e-24	10.822.108
212364_at	1.31e-23	2.18e-24	-10.820.556
203338_at	1.35e-23	2.25e-24	10.816.812
221699_s_at	1.35e-23	2.26e-24	10.816.318
202125_s_at	1.39e-23	2.32e-24	10.813.146
210541_s_at	1.41e-23	2.36e-24	10.811.206
204123_at	1.43e-23	2.38e-24	10.810.149
221725_at	1.50e-23	2.51e-24	10.804.267
218123_at	1.52e-23	2.54e-24	10.802.656
32042_at	1.54e-23	2.58e-24	10.800.821
214119_s_at	1.58e-23	2.64e-24	10.798.305
206323_x_at	1.59e-23	2.65e-24	-10.797.627
209176_at	1.59e-23	2.66e-24	10.797.404
200947_s_at	1.63e-23	2.73e-24	-10.794.199
208765_s_at	1.63e-23	2.74e-24	10.794.071
210453_x_at	1.68e-23	2.81e-24	10.790.936
215641_at	1.68e-23	2.81e-24	-10.790.842
220935_s_at	1.68e-23	2.82e-24	10.790.663
213753_x_at	1.69e-23	2.84e-24	-1.078.979
204161_s_at	1.73e-23	2.91e-24	10.787.036
218087_s_at	1.77e-23	2.98e-24	-10.784.377
211942_x_at	1.80e-23	3.01e-24	10.782.924
204970_s_at	1.82e-23	3.06e-24	10.781.285
205025_at	1.82e-23	3.06e-24	-10.781.283
203333_at	1.84e-23	3.09e-24	10.780.192
220558_x_at	1.84e-23	3.10e-24	-10.779.795

209894_at	1.85e-23	3.11e-24	-10.779.202
57540_at	1.87e-23	3.15e-24	-1.077.791
202729_s_at	1.88e-23	3.16e-24	-10.777.508
202066_at	1.89e-23	3.17e-24	107.771
218962_s_at	1.92e-23	3.23e-24	10.774.955
204847_at	1.99e-23	3.35e-24	10.770.631
211862_x_at	2.00e-23	3.37e-24	-10.769.939
203112_s_at	2.04e-23	3.43e-24	10.767.879
218989_x_at	2.06e-23	3.47e-24	10.766.496
212605_s_at	2.09e-23	3.51e-24	10.765.175
200684_s_at	2.09e-23	3.52e-24	10.764.978
208746_x_at	2.22e-23	3.74e-24	10.757.892
204894_s_at	2.23e-23	3.76e-24	-10.757.102
219303_at	2.24e-23	3.77e-24	10.756.986
202743_at	2.30e-23	3.88e-24	10.753.683
219903_s_at	2.32e-23	3.92e-24	-10.752.435
214879_x_at	2.35e-23	3.96e-24	-10.751.335
201196_s_at	2.37e-23	4.00e-24	10.750.176
207431_s_at	2.37e-23	4.00e-24	10.750.154
212130_x_at	2.37e-23	4.01e-24	10.749.819
213560_at	2.40e-23	4.05e-24	-10.748.621
209161_at	2.42e-23	4.08e-24	10.747.746
206789_s_at	2.42e-23	4.09e-24	10.747.541
216803_at	2.44e-23	4.12e-24	-10.746.585
220084_at	2.47e-23	4.17e-24	-10.745.129
218665_at	2.52e-23	4.26e-24	-10.742.693
215905_s_at	2.52e-23	4.27e-24	10.742.473
200937_s_at	2.58e-23	4.36e-24	10.739.968
211144_x_at	2.61e-23	4.41e-24	-10.738.611
201363_s_at	2.62e-23	4.43e-24	10.738.134
212251_at	2.70e-23	4.57e-24	10.734.581
216804_s_at	2.71e-23	4.59e-24	-10.734.105
57516_at	2.72e-23	4.61e-24	1.073.366
219531_at	2.73e-23	4.63e-24	10.733.134
206632_s_at	2.73e-23	4.63e-24	10.733.011
202037_s_at	2.74e-23	4.64e-24	-10.732.766
201888_s_at	2.80e-23	4.74e-24	-10.730.274
212675_s_at	2.80e-23	4.76e-24	10.729.973
202491_s_at	2.82e-23	4.78e-24	10.729.297
206237_s_at	2.87e-23	4.87e-24	-10.727.306
212145_at	2.90e-23	4.92e-24	10.726.076
216017_s_at	2.96e-23	5.03e-24	-1.072.355
220675_s_at	2.97e-23	5.05e-24	-10.722.984
209534_x_at	3.00e-23	5.09e-24	-10.721.999

201512_s_at	3.02e-23	5.14e-24	10.720.974
202173_s_at	3.10e-23	5.27e-24	10.717.907
213328_at	3.12e-23	5.30e-24	-10.717.311
213090_s_at	3.15e-23	5.36e-24	10.716.062
218516_s_at	3.25e-23	5.52e-24	10.712.617
221538_s_at	3.25e-23	5.54e-24	10.712.239
219561_at	3.28e-23	5.58e-24	-10.711.282
215923_s_at	3.41e-23	5.81e-24	-10.706.654
212091_s_at	3.74e-23	6.37e-24	-10.696
210568_s_at	3.75e-23	6.39e-24	10.695.653
202611_s_at	3.82e-23	6.50e-24	10.693.541
200034_s_at	3.88e-23	6.62e-24	10.691.506
202240_at	3.89e-23	6.62e-24	10.691.411
201695_s_at	3.89e-23	6.62e-24	-10.691.382
200596_s_at	3.95e-23	6.73e-24	10.689.533
215108_x_at	3.97e-23	6.78e-24	10.688.734
218501_at	4.00e-23	6.82e-24	10.688.002
218305_at	4.00e-23	6.83e-24	10.687.773
212596_s_at	4.05e-23	6.92e-24	10.686.345
209604_s_at	4.11e-23	7.02e-24	-10.684.702
212416_at	4.13e-23	7.05e-24	10.684.091
200967_at	4.15e-23	7.09e-24	10.683.429
200722_s_at	4.22e-23	7.21e-24	10.681.553
219646_at	4.30e-23	7.35e-24	10.679.234
217143_s_at	4.37e-23	7.48e-24	-10.677.219
202860_at	4.41e-23	7.55e-24	10.676.113
213975_s_at	4.49e-23	7.68e-24	10.674.113
203746_s_at	4.49e-23	7.69e-24	10.674.037
215034_s_at	4.51e-23	7.72e-24	10.673.536
200595_s_at	4.57e-23	7.82e-24	10.672.089
213112_s_at	4.66e-23	7.98e-24	10.669.648
220343_at	4.66e-23	7.98e-24	-10.669.648
213151_s_at	4.71e-23	8.07e-24	10.668.415
213402_at	4.71e-23	8.07e-24	10.668.367
222021_x_at	4.82e-23	8.27e-24	-10.665.525
214690_at	5.00e-23	8.58e-24	10.661.207
200049_at	5.01e-23	8.59e-24	10.661.093
216118_at	5.02e-23	8.61e-24	-10.660.845
218534_s_at	5.03e-23	8.63e-24	10.660.492
202762_at	5.04e-23	8.64e-24	10.660.345
222368_at	5.06e-23	8.68e-24	-10.659.845
201791_s_at	5.07e-23	8.71e-24	10.659.536
216769_x_at	5.11e-23	8.78e-24	-10.658.579
202460_s_at	5.20e-23	8.94e-24	-10.656.487

203043_at	5.23e-23	8.98e-24	-1.065.585
222130_s_at	5.33e-23	9.16e-24	10.653.633
212569_at	5.48e-23	9.43e-24	10.650.254
209238_at	5.65e-23	9.72e-24	10.646.661
221602_s_at	5.66e-23	9.74e-24	-10.646.432
221586_s_at	5.70e-23	9.82e-24	10.645.507
201009_s_at	5.72e-23	9.85e-24	-1.064.508
205937_at	5.73e-23	9.87e-24	10.644.869
221661_at	5.74e-23	9.89e-24	-1.064.458
209096_at	5.98e-23	1.03e-23	10.639.761
201414_s_at	6.05e-23	1.04e-23	10.638.418
219442_at	6.26e-23	1.08e-23	-10.634.361
202480_s_at	6.38e-23	1.10e-23	1.063.214
215809_at	6.42e-23	1.11e-23	-10.631.488
212062_at	6.50e-23	1.12e-23	10.629.859
219698_s_at	6.60e-23	1.14e-23	10.628.217
206070_s_at	6.70e-23	1.16e-23	-10.626.307
46167_at	6.84e-23	1.18e-23	10.623.911
209436_at	6.85e-23	1.18e-23	-10.623.709
201620_at	6.94e-23	1.20e-23	-10.622.176
221652_s_at	6.95e-23	1.20e-23	10.621.936
210678_s_at	7.01e-23	1.21e-23	-10.620.839
212644_s_at	7.13e-23	1.23e-23	1.061.887
202540_s_at	7.22e-23	1.25e-23	10.617.419
213794_s_at	7.23e-23	1.25e-23	10.617.127
218000_s_at	7.31e-23	1.26e-23	-10.615.891
200074_s_at	7.39e-23	1.28e-23	10.614.625
39548_at	7.80e-23	1.35e-23	10.608.154
203322_at	7.86e-23	1.36e-23	10.607.298
203673_at	8.04e-23	1.39e-23	-10.604.556
201632_at	8.08e-23	1.40e-23	10.604.039
206340_at	8.09e-23	1.40e-23	-10.603.832
211710_x_at	8.09e-23	1.40e-23	10.603.792
202194_at	8.43e-23	1.46e-23	10.598.941
215874_at	8.48e-23	1.47e-23	-10.598.191
216920_s_at	8.56e-23	1.48e-23	-105.971
212034_s_at	8.69e-23	1.51e-23	10.595.336
219635_at	8.77e-23	1.52e-23	10.594.225
221739_at	9.08e-23	1.58e-23	10.590.089
210746_s_at	9.08e-23	1.58e-23	-10.590.036
213044_at	9.15e-23	1.59e-23	10.589.192
213474_at	9.23e-23	1.60e-23	10.588.039
217168_s_at	9.34e-23	1.62e-23	-10.586.665
206682_at	9.45e-23	1.64e-23	-10.585.265

207405_s_at	9.60e-23	1.67e-23	1.058.344
201434_at	9.63e-23	1.67e-23	10.582.995
206139_at	9.69e-23	1.69e-23	10.582.262
201097_s_at	9.85e-23	1.71e-23	10.580.314
212820_at	9.99e-23	1.74e-23	10.578.662
217903_at	9.99e-23	1.74e-23	1.057.858
201507_at	1.01e-22	1.75e-23	10.577.572
200599_s_at	1.01e-22	1.76e-23	10.577.151
215629_s_at	1.03e-22	1.79e-23	10.574.912
205798_at	1.05e-22	1.82e-23	-10.573.036
204352_at	1.05e-22	1.83e-23	10.572.358
202078_at	1.06e-22	1.85e-23	1.057.114
220304_s_at	1.07e-22	1.86e-23	-10.570.571
202878_s_at	1.07e-22	1.87e-23	10.570.228
203341_at	1.08e-22	1.89e-23	10.568.819
212948_at	1.09e-22	1.90e-23	-10.568.394
220295_x_at	1.11e-22	1.93e-23	10.566.206
218240_at	1.11e-22	1.94e-23	10.565.934
201154_x_at	1.12e-22	1.96e-23	10.564.846
209372_x_at	1.12e-22	1.96e-23	10.564.591
202024_at	1.13e-22	1.97e-23	10.563.807
218477_at	1.13e-22	1.98e-23	10.563.386
212122_at	1.15e-22	2.00e-23	10.561.944
205480_s_at	1.15e-22	2.02e-23	-10.561.284
220780_at	1.16e-22	2.03e-23	-1.056.062
208562_s_at	1.17e-22	2.04e-23	-10.559.634
217785_s_at	1.17e-22	2.05e-23	10.559.492
205506_at	1.18e-22	2.06e-23	10.558.518
205303_at	1.20e-22	2.10e-23	-10.556.525
211548_s_at	1.21e-22	2.13e-23	-10.555.027
201576_s_at	1.24e-22	2.17e-23	10.552.771
203663_s_at	1.25e-22	2.19e-23	1.055.133
220773_s_at	1.25e-22	2.20e-23	-1.055.112
214697_s_at	1.31e-22	2.30e-23	10.545.847
214093_s_at	1.31e-22	2.30e-23	10.545.549
215093_at	1.34e-22	2.36e-23	10.542.885
37986_at	1.36e-22	2.38e-23	-10.541.817
203090_at	1.38e-22	2.43e-23	10.539.403
61732_r_at	1.39e-22	2.44e-23	-10.538.769
218828_at	1.44e-22	2.52e-23	-10.534.928
219555_s_at	1.44e-22	2.53e-23	10.534.719
205227_at	1.50e-22	2.63e-23	-10.530.041
208757_at	1.52e-22	2.68e-23	10.527.978
200061_s_at	1.59e-22	2.80e-23	10.522.743

204949_at	1.60e-22	2.81e-23	-10.522.356
217942_at	1.62e-22	2.85e-23	10.520.519
219940_s_at	1.62e-22	2.86e-23	10.520.242
208530_s_at	1.65e-22	2.91e-23	-10.518.223
220018_at	1.71e-22	3.01e-23	10.514.357
218378_s_at	1.71e-22	3.01e-23	10.514.352
217950_at	1.72e-22	3.02e-23	10.513.623
209087_x_at	1.73e-22	3.05e-23	10.512.517
204204_at	1.75e-22	3.08e-23	-10.511.508
221877_at	1.77e-22	3.12e-23	10.510.104
212943_at	1.81e-22	3.19e-23	10.507.201
204027_s_at	1.81e-22	3.20e-23	10.507.124
208120_x_at	1.83e-22	3.23e-23	-10.505.703
206177_s_at	1.84e-22	3.24e-23	-10.505.517
212622_at	1.84e-22	3.24e-23	10.505.473
213939_s_at	1.87e-22	3.30e-23	-10.503.363
211355_x_at	1.87e-22	3.30e-23	-10.503.259
202898_at	1.89e-22	3.34e-23	-10.501.931
209623_at	1.90e-22	3.35e-23	-10.501.589
212082_s_at	1.92e-22	3.39e-23	10.500.104
205235_s_at	1.96e-22	3.46e-23	10.497.762
203427_at	1.96e-22	3.47e-23	10.497.373
204617_s_at	1.99e-22	3.52e-23	1.049.584
209962_at	2.01e-22	3.56e-23	-10.494.373
204458_at	2.02e-22	3.57e-23	-10.494.055
209466_x_at	2.02e-22	3.58e-23	-10.493.863
210463_x_at	2.04e-22	3.60e-23	10.492.983
203403_s_at	2.04e-22	3.61e-23	10.492.848
218344_s_at	2.10e-22	3.71e-23	10.489.458
202904_s_at	2.13e-22	3.76e-23	10.487.831
221487_s_at	2.15e-22	3.80e-23	10.486.632
217790_s_at	2.16e-22	3.83e-23	10.485.947
210665_at	2.16e-22	3.83e-23	10.485.887
201239_s_at	2.16e-22	3.83e-23	10.485.844
213668_s_at	2.16e-22	3.84e-23	10.485.621
204265_s_at	2.19e-22	3.89e-23	-1.048.399
207426_s_at	2.20e-22	3.90e-23	10.483.739
213532_at	2.21e-22	3.92e-23	10.482.985
205820_s_at	2.23e-22	3.96e-23	-10.481.873
221712_s_at	2.28e-22	4.04e-23	10.479.432
205353_s_at	2.35e-22	4.17e-23	-10.475.675
217911_s_at	2.38e-22	4.23e-23	10.473.979
207416_s_at	2.45e-22	4.35e-23	-10.470.771
211725_s_at	2.50e-22	4.45e-23	1.046.808

211152_s_at	2.52e-22	4.48e-23	10.467.452
205865_at	2.53e-22	4.50e-23	10.466.718
218562_s_at	2.53e-22	4.51e-23	-10.466.625
217816_s_at	2.53e-22	4.51e-23	10.466.591
215483_at	2.56e-22	4.55e-23	-10.465.377
210149_s_at	2.62e-22	4.67e-23	10.462.527
209384_at	2.63e-22	4.69e-23	-10.462.042
209935_at	2.65e-22	4.72e-23	10.461.093
210124_x_at	2.66e-22	4.74e-23	10.460.629
219364_at	2.67e-22	4.76e-23	-10.460.167
218381_s_at	2.68e-22	4.78e-23	10.459.693
202089_s_at	2.69e-22	4.80e-23	10.459.084
207756_at	2.75e-22	4.91e-23	-10.456.431
203781_at	2.76e-22	4.92e-23	10.456.228
209115_at	2.78e-22	4.97e-23	10.455.092
211356_x_at	2.85e-22	5.09e-23	-10.452.153
211063_s_at	2.86e-22	5.11e-23	10.451.869
218719_s_at	2.86e-22	5.12e-23	1.045.167
221744_at	2.89e-22	5.17e-23	10.450.437
212089_at	2.93e-22	5.23e-23	1.044.901
218452_at	2.93e-22	5.24e-23	10.448.834
215737_x_at	2.93e-22	5.25e-23	-10.448.703
212599_at	2.96e-22	5.30e-23	-10.447.524
203557_s_at	3.02e-22	5.40e-23	-10.445.185
44111_at	3.04e-22	5.44e-23	10.444.417
205383_s_at	3.12e-22	5.58e-23	-10.441.406
210830_s_at	3.13e-22	5.60e-23	10.441.008
209448_at	3.17e-22	5.68e-23	1.043.925
215548_s_at	3.22e-22	5.76e-23	10.437.701
216537_s_at	3.24e-22	5.80e-23	-10.436.817
209156_s_at	3.34e-22	5.99e-23	-10.433.028
209084_s_at	3.42e-22	6.13e-23	1.043.029
209280_at	3.63e-22	6.52e-23	-10.423.091
205405_at	3.76e-22	6.74e-23	-10.419.151
201233_at	3.88e-22	6.96e-23	1.041.531
200642_at	3.91e-22	7.02e-23	-10.414.214
220613_s_at	3.92e-22	7.03e-23	-10.414.055
219140_s_at	3.98e-22	7.14e-23	-10.412.213
204607_at	4.07e-22	7.31e-23	-10.409.479
220139_at	4.08e-22	7.33e-23	-1.040.923
203210_s_at	4.18e-22	7.50e-23	10.406.405
203104_at	4.22e-22	7.59e-23	-10.405.075
220512_at	4.29e-22	7.71e-23	-10.403.206
212139_at	4.31e-22	7.74e-23	10.402.647

204651_at	4.37e-22	7.86e-23	10.400.917
214055_x_at	4.40e-22	7.91e-23	10.400.217
215462_at	4.40e-22	7.91e-23	-10.400.129
205765_at	4.42e-22	7.95e-23	-10.399.599
212577_at	4.46e-22	8.03e-23	10.398.406
203870_at	4.47e-22	8.05e-23	10.398.084
219564_at	4.61e-22	8.30e-23	-10.394.508
205406_s_at	4.69e-22	8.44e-23	10.392.482
200002_at	4.77e-22	8.60e-23	10.390.225
65493_at	4.81e-22	8.66e-23	10.389.365
205140_at	4.81e-22	8.67e-23	10.389.341
205048_s_at	4.83e-22	8.71e-23	10.388.747
208886_at	4.83e-22	8.71e-23	103.887
203233_at	4.83e-22	8.71e-23	-10.388.669
209128_s_at	4.94e-22	8.92e-23	10.385.905
210862_s_at	4.95e-22	8.93e-23	-10.385.753
201722_s_at	4.98e-22	9.00e-23	10.384.885
204531_s_at	5.01e-22	9.04e-23	10.384.297
209830_s_at	5.05e-22	9.12e-23	-10.383.247
200022_at	5.11e-22	9.24e-23	10.381.761
40465_at	5.21e-22	9.41e-23	1.037.961
206913_at	5.39e-22	9.74e-23	-10.375.546
200805_at	5.39e-22	9.75e-23	10.375.365
209386_at	5.45e-22	9.86e-23	1.037.401
206256_at	5.50e-22	9.95e-23	-1.037.298
201642_at	5.52e-22	9.99e-23	1.037.249
208838_at	5.55e-22	1.01e-22	10.371.772
217740_x_at	5.58e-22	1.01e-22	10.371.268
203103_s_at	5.58e-22	1.01e-22	10.371.063
205044_at	5.67e-22	1.03e-22	-10.369.233
202495_at	5.70e-22	1.03e-22	1.036.857
200096_s_at	5.72e-22	1.04e-22	10.368.179
205741_s_at	5.72e-22	1.04e-22	10.368.148
220590_at	5.97e-22	1.08e-22	-10.363.067
206187_at	6.03e-22	1.09e-22	-10.361.729
217509_x_at	6.03e-22	1.09e-22	-10.361.699
216387_x_at	6.10e-22	1.11e-22	10.360.287
202406_s_at	6.41e-22	1.16e-22	10.354.444
214033_at	6.45e-22	1.17e-22	10.353.741
219570_at	6.50e-22	1.18e-22	10.352.787
217765_at	6.52e-22	1.18e-22	10.352.407
212433_x_at	6.55e-22	1.19e-22	10.351.771
206350_at	6.69e-22	1.22e-22	-10.349.204
216604_s_at	6.72e-22	1.22e-22	-1.034.856

201540_at	6.77e-22	1.23e-22	-10.347.779
219421_at	6.78e-22	1.23e-22	10.347.533
214043_at	6.78e-22	1.23e-22	-1.034.743
219479_at	6.80e-22	1.24e-22	10.347.043
204618_s_at	6.88e-22	1.25e-22	10.345.786
202374_s_at	7.09e-22	1.29e-22	10.342.082
216607_s_at	7.11e-22	1.29e-22	10.341.801
221260_s_at	7.41e-22	1.35e-22	10.336.859
202021_x_at	7.45e-22	1.36e-22	10.336.152
204790_at	7.48e-22	1.36e-22	-10.335.564
222273_at	7.58e-22	1.38e-22	10.333.952
215554_at	7.76e-22	1.41e-22	-10.331.234
204998_s_at	7.80e-22	1.42e-22	-10.330.489
212579_at	7.95e-22	1.45e-22	10.328.209
203049_s_at	8.07e-22	1.47e-22	10.326.386
33307_at	8.42e-22	1.54e-22	10.321.334
205119_s_at	8.43e-22	1.54e-22	-10.321.162
215537_x_at	8.45e-22	1.54e-22	10.320.912
217805_at	8.47e-22	1.55e-22	10.320.644
210966_x_at	8.84e-22	1.62e-22	1.031.542
201344_at	9.30e-22	1.70e-22	10.309.342
212337_at	9.47e-22	1.73e-22	10.307.213
218942_at	9.48e-22	1.73e-22	10.307.105
218609_s_at	9.58e-22	1.75e-22	10.305.762
AFFX-HUMGAPDH/M3	9.64e-22	1.76e-22	10.304.988
211255_x_at	9.69e-22	1.77e-22	10.304.349
203117_s_at	9.71e-22	1.78e-22	-10.304.076
202177_at	9.73e-22	1.78e-22	-10.303.754
206854_s_at	9.77e-22	1.79e-22	10.303.342
217904_s_at	9.80e-22	1.79e-22	-10.302.917
201175_at	9.93e-22	1.82e-22	10.301.344
202128_at	9.95e-22	1.82e-22	10.301.032
65588_at	1.01e-21	1.85e-22	10.299.102
202204_s_at	1.02e-21	1.87e-22	-10.297.918
219991_at	1.02e-21	1.87e-22	-10.297.675
216883_x_at	1.02e-21	1.88e-22	10.297.383
203269_at	1.05e-21	1.92e-22	10.294.984
219765_at	1.05e-21	1.93e-22	1.029.435
208722_s_at	1.10e-21	2.02e-22	10.288.879
209813_x_at	1.10e-21	2.02e-22	-10.288.841
215867_x_at	1.12e-21	2.05e-22	10.286.871
201298_s_at	1.13e-21	2.07e-22	1.028.573
219056_at	1.14e-21	2.09e-22	10.284.565
42361_g_at	1.16e-21	2.12e-22	10.282.864

203503_s_at	1.17e-21	2.14e-22	-10.281.717
213826_s_at	1.22e-21	2.24e-22	10.276.448
37943_at	1.22e-21	2.25e-22	10.276.092
218094_s_at	1.23e-21	2.27e-22	10.274.933
215095_at	1.24e-21	2.28e-22	-10.274.345
210610_at	1.25e-21	2.30e-22	-10.273.363
213347_x_at	1.29e-21	2.38e-22	10.269.468
203212_s_at	1.31e-21	2.42e-22	10.267.432
221904_at	1.36e-21	2.51e-22	10.263.024
221952_x_at	1.40e-21	2.57e-22	10.260.035
202776_at	1.44e-21	2.65e-22	10.256.402
203543_s_at	1.45e-21	2.67e-22	-10.255.644
220736_at	1.45e-21	2.67e-22	-10.255.445
213072_at	1.45e-21	2.67e-22	10.255.413
220668_s_at	1.49e-21	2.75e-22	10.251.886
221571_at	1.52e-21	2.80e-22	10.250.004
202351_at	1.53e-21	2.82e-22	1.024.898
215244_at	1.53e-21	2.82e-22	-10.248.968
219008_at	1.56e-21	2.89e-22	10.246.186
204957_at	1.59e-21	2.93e-22	10.244.412
213129_s_at	1.62e-21	2.99e-22	-10.242.035
212531_at	1.62e-21	3.00e-22	1.024.165
214150_x_at	1.66e-21	3.06e-22	10.239.049
209380_s_at	1.67e-21	3.08e-22	10.238.284
218590_at	1.67e-21	3.09e-22	10.238.007
218339_at	1.68e-21	3.11e-22	1.023.716
211373_s_at	1.75e-21	3.24e-22	10.232.262
205863_at	1.79e-21	3.31e-22	-10.229.823
204728_s_at	1.80e-21	3.34e-22	10.228.928
221729_at	1.80e-21	3.34e-22	10.228.915
213511_s_at	1.81e-21	3.36e-22	10.228.083
212244_at	1.82e-21	3.36e-22	10.227.936
210821_x_at	1.86e-21	3.44e-22	10.225.286
218498_s_at	1.86e-21	3.45e-22	10.225.048
213166_x_at	1.87e-21	3.46e-22	1.022.449
208705_s_at	1.90e-21	3.52e-22	-10.222.624
219848_s_at	1.98e-21	3.67e-22	1.021.758
213075_at	2.12e-21	3.93e-22	10.209.192
201972_at	2.17e-21	4.02e-22	10.206.628
202907_s_at	2.20e-21	4.07e-22	10.204.965
216319_at	2.21e-21	4.09e-22	-10.204.385
201359_at	2.23e-21	4.13e-22	1.020.331
212567_s_at	2.25e-21	4.18e-22	10.201.958
206153_at	2.28e-21	4.23e-22	-10.200.529

201072_s_at	2.31e-21	4.28e-22	10.198.978
216602_s_at	2.36e-21	4.39e-22	10.195.978
215930_s_at	2.38e-21	4.42e-22	-10.195.122
217665_at	2.45e-21	4.54e-22	10.191.885
203976_s_at	2.45e-21	4.56e-22	10.191.433
205169_at	2.50e-21	4.66e-22	10.188.935
209000_s_at	2.52e-21	4.68e-22	10.188.407
209362_at	2.53e-21	4.71e-22	10.187.485
214314_s_at	2.59e-21	4.81e-22	10.184.982
205340_at	2.61e-21	4.86e-22	10.183.872
201329_s_at	2.62e-21	4.88e-22	-10.183.241
212643_at	2.66e-21	4.95e-22	10.181.564
209063_x_at	2.76e-21	5.14e-22	10.177.242
200948_at	2.76e-21	5.15e-22	-1.017.694
213983_s_at	2.80e-21	5.21e-22	10.175.443
204886_at	2.82e-21	5.26e-22	10.174.288
222233_s_at	2.83e-21	5.28e-22	101.739
219355_at	2.89e-21	5.39e-22	1.017.136
219961_s_at	2.90e-21	5.41e-22	10.171.086
200728_at	2.90e-21	5.41e-22	10.170.941
218441_s_at	2.94e-21	5.48e-22	10.169.413
211806_s_at	2.95e-21	5.51e-22	-1.016.888
209014_at	2.95e-21	5.52e-22	10.168.632
212164_at	2.98e-21	5.56e-22	10.167.604
212242_at	3.01e-21	5.63e-22	10.166.241
218415_at	3.02e-21	5.65e-22	10.165.729
202149_at	3.05e-21	5.69e-22	10.164.869
204013_s_at	3.11e-21	5.81e-22	-10.162.442
219038_at	3.14e-21	5.87e-22	10.161.166
211984_at	3.22e-21	6.01e-22	-10.158.273
213030_s_at	3.31e-21	6.19e-22	10.154.774
200069_at	3.31e-21	6.20e-22	10.154.674
219821_s_at	3.31e-21	6.20e-22	-10.154.672
212847_at	3.36e-21	6.28e-22	10.153.004
211465_x_at	3.38e-21	6.33e-22	-10.152.113
221711_s_at	3.45e-21	6.47e-22	10.149.554
212142_at	3.46e-21	6.49e-22	1.014.912
221779_at	3.50e-21	6.57e-22	10.147.742
211262_at	3.51e-21	6.57e-22	-1.014.764
203094_at	3.51e-21	6.58e-22	10.147.543
200746_s_at	3.51e-21	6.59e-22	10.147.387
59697_at	3.55e-21	6.67e-22	10.145.912
204833_at	3.58e-21	6.73e-22	10.144.861
211819_s_at	3.59e-21	6.74e-22	-1.014.453

219105_x_at	3.64e-21	6.84e-22	10.142.804
215451_s_at	3.65e-21	6.84e-22	-10.142.743
211354_s_at	3.68e-21	6.91e-22	-10.141.601
201579_at	3.73e-21	7.00e-22	10.140.045
218249_at	3.82e-21	7.18e-22	10.137.068
208744_x_at	3.84e-21	7.22e-22	10.136.356
212848_s_at	3.86e-21	7.26e-22	-10.135.643
210283_x_at	3.87e-21	7.29e-22	10.135.237
220646_s_at	3.90e-21	7.34e-22	-10.134.338
222064_s_at	4.02e-21	7.56e-22	1.013.073
217996_at	4.05e-21	7.63e-22	-10.129.691
39402_at	4.10e-21	7.71e-22	-10.128.362
212476_at	4.12e-21	7.76e-22	10.127.598
209045_at	4.30e-21	8.09e-22	10.122.594
200918_s_at	4.30e-21	8.10e-22	-10.122.487
205370_x_at	4.41e-21	8.30e-22	-10.119.559
211200_s_at	4.53e-21	8.54e-22	10.116.108
218873_at	4.60e-21	8.67e-22	10.114.312
210230_at	4.61e-21	8.70e-22	-101.139
40829_at	4.67e-21	8.81e-22	-10.112.417
200819_s_at	4.70e-21	8.87e-22	10.111.545
204602_at	4.74e-21	8.94e-22	10.110.605
200067_x_at	4.79e-21	9.04e-22	10.109.333
205526_s_at	4.80e-21	9.06e-22	10.109.059
201828_x_at	4.82e-21	9.11e-22	10.108.361
207601_at	4.90e-21	9.26e-22	-10.106.434
217356_s_at	5.01e-21	9.46e-22	1.010.379
218588_s_at	5.04e-21	9.52e-22	10.103.003
212061_at	5.07e-21	9.58e-22	10.102.296
214848_at	5.09e-21	9.62e-22	10.101.746
210313_at	5.09e-21	9.63e-22	-10.101.679
212227_x_at	5.14e-21	9.73e-22	10.100.471
210470_x_at	5.19e-21	9.82e-22	10.099.287
201119_s_at	5.21e-21	9.85e-22	10.098.884
201784_s_at	5.22e-21	9.87e-22	10.098.663
203449_s_at	5.27e-21	9.98e-22	1.009.738
203855_at	5.39e-21	1.02e-21	10.094.763
209994_s_at	5.42e-21	1.03e-21	-10.093.977
222378_at	5.51e-21	1.04e-21	-10.092.026
200705_s_at	5.53e-21	1.05e-21	10.091.425
206651_s_at	5.53e-21	1.05e-21	-10.091.387
202282_at	5.59e-21	1.06e-21	-10.090.189
202219_at	5.59e-21	1.06e-21	10.090.033
203607_at	5.66e-21	1.07e-21	1.008.849

217733_s_at	5.71e-21	1.08e-21	10.087.501
202914_s_at	5.94e-21	1.13e-21	10.082.762
212330_at	6.00e-21	1.14e-21	1.008.147
208511_at	6.06e-21	1.15e-21	10.080.224
220954_s_at	6.12e-21	1.16e-21	-10.079.006
204372_s_at	6.13e-21	1.17e-21	10.078.693
210447_at	6.14e-21	1.17e-21	10.078.532
204580_at	6.22e-21	1.18e-21	10.076.895
204367_at	6.31e-21	1.20e-21	10.075.271
202364_at	6.33e-21	1.20e-21	-10.074.788
203292_s_at	6.45e-21	1.23e-21	10.072.495
207843_x_at	6.51e-21	1.24e-21	-10.071.269
218830_at	6.69e-21	1.27e-21	10.067.953
210058_at	6.73e-21	1.28e-21	10.067.211
218260_at	6.74e-21	1.28e-21	10.066.993
201484_at	6.82e-21	1.30e-21	10.065.667
213156_at	6.96e-21	1.33e-21	-10.063.103
217027_x_at	7.07e-21	1.35e-21	10.061.189
218500_at	7.09e-21	1.35e-21	10.060.877
209616_s_at	7.16e-21	1.36e-21	-10.059.681
217873_at	7.19e-21	1.37e-21	10.059.154
209121_x_at	7.27e-21	1.39e-21	-10.057.797
219530_at	7.35e-21	1.40e-21	10.056.368
213362_at	7.46e-21	1.42e-21	-10.054.531
203740_at	7.57e-21	1.44e-21	10.052.719
212945_s_at	7.58e-21	1.45e-21	10.052.647
207111_at	7.61e-21	1.45e-21	-10.052.032
216569_at	7.66e-21	1.46e-21	-10.051.176
203641_s_at	7.69e-21	1.47e-21	-10.050.778
201427_s_at	7.72e-21	1.48e-21	-1.005.019
206535_at	7.73e-21	1.48e-21	-10.050.009
206548_at	7.82e-21	1.49e-21	-10.048.668
201230_s_at	7.86e-21	1.50e-21	10.047.944
200739_s_at	7.87e-21	1.50e-21	10.047.855
203149_at	8.13e-21	1.55e-21	10.043.923
219623_at	8.15e-21	1.56e-21	10.043.466
207788_s_at	8.58e-21	1.64e-21	-10.037.314
203229_s_at	8.65e-21	1.65e-21	10.036.323
201710_at	8.74e-21	1.67e-21	10.035.059
217159_x_at	8.76e-21	1.68e-21	-10.034.636
209295_at	8.84e-21	1.69e-21	-10.033.605
201509_at	8.89e-21	1.70e-21	10.032.878
210387_at	8.89e-21	1.70e-21	10.032.855
219915_s_at	9.06e-21	1.74e-21	-10.030.452

201094_at	9.08e-21	1.74e-21	10.030.143
201670_s_at	9.09e-21	1.74e-21	10.029.991
211976_at	9.10e-21	1.75e-21	10.029.837
213712_at	9.11e-21	1.75e-21	10.029.778
213601_at	9.15e-21	1.76e-21	-10.029.094
214306_at	9.26e-21	1.78e-21	10.027.733
216238_s_at	9.37e-21	1.80e-21	-10.026.248
217388_s_at	9.44e-21	1.81e-21	-10.025.265
216379_x_at	9.50e-21	1.82e-21	10.024.555
218470_at	9.59e-21	1.84e-21	10.023.319
209665_at	9.85e-21	1.89e-21	10.020.018
204759_at	1.00e-20	1.93e-21	-10.017.932
211888_x_at	1.00e-20	1.93e-21	-10.017.666
203609_s_at	1.01e-20	1.94e-21	-10.016.872
220661_s_at	1.02e-20	1.96e-21	1.001.557
200784_s_at	1.08e-20	2.08e-21	-1.000.838
201256_at	1.09e-20	2.09e-21	10.007.848
203583_at	1.09e-20	2.10e-21	10.007.664
221247_s_at	1.09e-20	2.10e-21	10.007.451
222016_s_at	1.13e-20	2.17e-21	10.003.516
212366_at	1.16e-20	2.24e-21	9.999.623
209475_at	1.19e-20	2.30e-21	-9.996.493
203771_s_at	1.20e-20	2.31e-21	9.995.699
208974_x_at	1.21e-20	2.34e-21	9.994.355
218023_s_at	1.22e-20	2.34e-21	9.994.216
219120_at	1.24e-20	2.40e-21	9.991.461
204055_s_at	1.25e-20	2.41e-21	-9.990.878
218992_at	1.25e-20	2.41e-21	9.990.524
219731_at	1.25e-20	2.42e-21	9.990.308
213480_at	1.25e-20	2.42e-21	9.990.255
210708_x_at	1.25e-20	2.42e-21	-9.990.145
204388_s_at	1.26e-20	2.44e-21	-9.989.181
202167_s_at	1.29e-20	2.49e-21	9.986.642
218734_at	1.29e-20	2.50e-21	9.986.533
211569_s_at	1.32e-20	2.54e-21	-9.984.175
214427_at	1.33e-20	2.57e-21	9.983.056
205283_at	1.33e-20	2.57e-21	9.982.795
211827_s_at	1.33e-20	2.58e-21	-9.982.487
208730_x_at	1.34e-20	2.59e-21	-9.981.961
202152_x_at	1.36e-20	2.63e-21	-9.980.378
211115_x_at	1.36e-20	2.63e-21	9.979.953
91816_f_at	1.38e-20	2.68e-21	9.977.902
219268_at	1.41e-20	2.72e-21	-9.976.017
200657_at	1.44e-20	2.79e-21	9.973.019

218870_at	1.45e-20	2.80e-21	-9.972.342
212666_at	1.45e-20	2.81e-21	9.972.085
201412_at	1.48e-20	2.86e-21	9.970.055
204832_s_at	1.48e-20	2.86e-21	9.969.832
215418_at	1.48e-20	2.88e-21	-996.931
205336_at	1.49e-20	2.88e-21	-9.968.974
209022_at	1.51e-20	2.92e-21	9.967.524
207736_s_at	1.52e-20	2.95e-21	-9.966.308
220137_at	1.53e-20	2.96e-21	9.965.744
212930_at	1.54e-20	2.99e-21	9.964.599
202760_s_at	1.55e-20	3.01e-21	-9.963.727
213298_at	1.55e-20	3.01e-21	-9.963.593
203409_at	1.59e-20	3.08e-21	-996.091
200644_at	1.61e-20	3.12e-21	9.959.386
215088_s_at	1.63e-20	3.17e-21	9.957.332
211026_s_at	1.64e-20	3.19e-21	-9.956.723
205061_s_at	1.67e-20	3.24e-21	9.954.948
205839_s_at	1.67e-20	3.25e-21	-9.954.384
220240_s_at	1.68e-20	3.26e-21	9.954.072
211454_x_at	1.68e-20	3.26e-21	-9.953.888
219015_s_at	1.68e-20	3.27e-21	9.953.629
222102_at	1.72e-20	3.35e-21	-9.950.633
212608_s_at	1.75e-20	3.41e-21	9.948.764
212365_at	1.76e-20	3.43e-21	-9.947.993
203963_at	1.77e-20	3.45e-21	9.947.281
212959_s_at	1.78e-20	3.46e-21	9.946.887
206000_at	1.80e-20	3.50e-21	9.945.525
215806_x_at	1.82e-20	3.55e-21	-9.943.681
212526_at	1.85e-20	3.60e-21	-9.942.074
222103_at	1.86e-20	3.62e-21	9.941.426
206507_at	1.86e-20	3.62e-21	9.941.326
219401_at	1.86e-20	3.62e-21	9.941.321
204359_at	1.87e-20	3.65e-21	-9.940.391
203356_at	1.87e-20	3.66e-21	9.940.127
202673_at	1.91e-20	3.73e-21	9.937.813
221596_s_at	1.93e-20	3.78e-21	9.936.204
201922_at	1.94e-20	3.79e-21	9.935.739
206272_at	1.96e-20	3.84e-21	9.934.275
209827_s_at	1.99e-20	3.88e-21	-9.932.941
202539_s_at	1.99e-20	3.89e-21	9.932.478
217749_at	2.02e-20	3.94e-21	9.930.958
222296_at	2.03e-20	3.98e-21	-9.929.873
213454_at	2.07e-20	4.05e-21	992.774
209771_x_at	2.08e-20	4.07e-21	9.927.112

203991_s_at	2.09e-20	4.08e-21	9.926.666
200725_x_at	2.10e-20	4.11e-21	9.925.852
207799_x_at	2.11e-20	4.14e-21	-9.925.086
220107_s_at	2.12e-20	4.16e-21	-9.924.442
216849_at	2.13e-20	4.18e-21	-9.923.922
211858_x_at	2.14e-20	4.20e-21	9.923.374
203286_at	2.18e-20	4.27e-21	9.921.111
218860_at	2.24e-20	4.40e-21	9.917.659
38149_at	2.25e-20	4.41e-21	-9.917.209
202447_at	2.26e-20	4.43e-21	-991.684
218425_at	2.30e-20	4.52e-21	9.914.312
201864_at	2.36e-20	4.63e-21	9.911.269
54632_at	2.41e-20	4.74e-21	9.908.576
218057_x_at	2.42e-20	4.76e-21	9.908.105
213469_at	2.45e-20	4.81e-21	9.906.695
218549_s_at	2.46e-20	4.83e-21	9.906.172
208620_at	2.48e-20	4.86e-21	9.905.394
221895_at	2.56e-20	5.03e-21	99.013
214662_at	2.63e-20	5.17e-21	9.897.941
49077_at	2.64e-20	5.18e-21	9.897.583
205357_s_at	2.66e-20	5.22e-21	-9.896.698
213302_at	2.68e-20	5.26e-21	9.895.801
209370_s_at	2.68e-20	5.26e-21	-989.574
206835_at	2.71e-20	5.34e-21	-9.894.054
222154_s_at	2.73e-20	5.38e-21	9.893.104
202763_at	2.74e-20	5.39e-21	9.892.763
200772_x_at	2.74e-20	5.40e-21	9.892.678
214280_x_at	2.78e-20	5.46e-21	9.891.137
202138_x_at	2.86e-20	5.64e-21	9.887.366
209012_at	2.88e-20	5.67e-21	9.886.566
212238_at	2.91e-20	5.72e-21	9.885.468
214164_x_at	2.91e-20	5.74e-21	98.852
209089_at	2.99e-20	5.89e-21	9.881.923
205650_s_at	3.00e-20	5.90e-21	-9.881.697
214979_at	3.08e-20	6.07e-21	-9.878.355
218459_at	3.10e-20	6.11e-21	9.877.527
204333_s_at	3.11e-20	6.14e-21	9.876.976
216389_s_at	3.19e-20	6.29e-21	-9.873.891
208986_at	3.21e-20	6.34e-21	9.872.961
218156_s_at	3.25e-20	6.41e-21	9.871.565
214592_s_at	3.25e-20	6.42e-21	-9.871.532
209880_s_at	3.26e-20	6.44e-21	-9.871.085
203448_s_at	3.27e-20	6.46e-21	9.870.724
222139_at	3.31e-20	6.54e-21	9.869.263

209680_s_at	3.35e-20	6.61e-21	9.867.869
206929_s_at	3.38e-20	6.67e-21	-9.866.767
219407_s_at	3.40e-20	6.72e-21	-9.865.971
213082_s_at	3.40e-20	6.72e-21	9.865.806
212170_at	3.41e-20	6.73e-21	9.865.612
219065_s_at	3.44e-20	6.80e-21	9.864.421
208485_x_at	3.44e-20	6.81e-21	-9.864.342
215734_at	3.46e-20	6.84e-21	-986.364
217745_s_at	3.47e-20	6.87e-21	9.863.124
208851_s_at	3.50e-20	6.93e-21	9.862.124
213048_s_at	3.56e-20	7.05e-21	9.860.017
204643_s_at	3.56e-20	7.06e-21	9.859.856
204040_at	3.58e-20	7.09e-21	9.859.389
217173_s_at	3.59e-20	7.11e-21	-9.858.998
214375_at	3.60e-20	7.13e-21	-9.858.564
218342_s_at	3.62e-20	7.18e-21	9.857.823
204008_at	3.63e-20	7.21e-21	985.735
202777_at	3.65e-20	7.23e-21	9.856.871
216791_at	3.66e-20	7.27e-21	-9.856.266
218481_at	3.72e-20	7.38e-21	9.854.361
218947_s_at	3.77e-20	7.47e-21	985.289
221082_s_at	3.77e-20	7.49e-21	9.852.624
212891_s_at	3.85e-20	7.64e-21	9.850.117
203924_at	3.91e-20	7.77e-21	-9.848.107
212504_at	4.05e-20	8.05e-21	-9.843.835
220160_s_at	4.06e-20	8.06e-21	9.843.658
213519_s_at	4.10e-20	8.15e-21	-9.842.269
209553_at	4.14e-20	8.23e-21	9.841.126
207714_s_at	4.15e-20	8.26e-21	9.840.644
206049_at	4.16e-20	8.28e-21	-9.840.407
209625_at	4.21e-20	8.37e-21	9.839.071
206807_s_at	4.21e-20	8.38e-21	-9.838.925
222253_s_at	4.30e-20	8.55e-21	-9.836.391
220951_s_at	4.32e-20	8.60e-21	-9.835.643
213316_at	4.33e-20	8.62e-21	9.835.427
218646_at	4.39e-20	8.75e-21	98.336
204201_s_at	4.41e-20	8.78e-21	-983.318
217825_s_at	4.45e-20	8.87e-21	9.831.882
203198_at	4.46e-20	8.89e-21	-9.831.594
217106_x_at	4.47e-20	8.90e-21	9.831.451
212380_at	4.56e-20	9.09e-21	9.828.931
216702_x_at	4.57e-20	9.11e-21	-9.828.668
213896_x_at	4.62e-20	9.22e-21	9.827.168
201821_s_at	4.65e-20	9.28e-21	9.826.335

202300_at	4.67e-20	9.31e-21	9.825.918
203884_s_at	4.73e-20	9.45e-21	9.824.169
204449_at	4.78e-20	9.55e-21	9.822.908
204010_s_at	4.79e-20	9.56e-21	-9.822.686
203735_x_at	4.83e-20	9.65e-21	-9.821.552
39854_r_at	4.87e-20	9.73e-21	-9.820.585
218687_s_at	4.92e-20	9.83e-21	9.819.255
201637_s_at	4.93e-20	9.86e-21	9.818.942
219071_x_at	5.03e-20	1.01e-20	9.816.453
209177_at	5.12e-20	1.02e-20	9.814.263
218244_at	5.13e-20	1.03e-20	9.814.162
202218_s_at	5.16e-20	1.03e-20	9.813.355
206445_s_at	5.29e-20	1.06e-20	9.810.143
204107_at	5.48e-20	1.10e-20	9.805.785
201184_s_at	5.54e-20	1.11e-20	9.804.414
201131_s_at	5.61e-20	1.12e-20	-9.802.853
209533_s_at	5.63e-20	1.13e-20	9.802.451
201573_s_at	5.67e-20	1.14e-20	9.801.659
208598_s_at	5.67e-20	1.14e-20	9.801.586
203023_at	5.67e-20	1.14e-20	9.801.445
216532_x_at	5.74e-20	1.15e-20	9.799.966
213080_x_at	5.84e-20	1.17e-20	9.797.799
218157_x_at	5.88e-20	1.18e-20	9.797.017
201389_at	5.99e-20	1.20e-20	9.794.637
209425_at	6.16e-20	1.24e-20	-9.791.139
222010_at	6.19e-20	1.24e-20	9.790.594
208811_s_at	6.27e-20	1.26e-20	9.788.965
209445_x_at	6.29e-20	1.26e-20	9.788.587
215269_at	6.32e-20	1.27e-20	-9.787.967
212995_x_at	6.35e-20	1.28e-20	9.787.253
204800_s_at	6.39e-20	1.28e-20	-9.786.599
207104_x_at	6.42e-20	1.29e-20	-9.785.874
202899_s_at	6.54e-20	1.31e-20	9.783.666
219428_s_at	6.56e-20	1.32e-20	9.783.228
205291_at	6.61e-20	1.33e-20	-9.782.335
37966_at	6.70e-20	1.35e-20	9.780.596
209404_s_at	6.73e-20	1.35e-20	Eyl.78
209971_x_at	6.76e-20	1.36e-20	9.779.352
206544_x_at	6.80e-20	1.37e-20	-9.778.747
221261_x_at	6.80e-20	1.37e-20	9.778.611
221015_s_at	6.82e-20	1.37e-20	-9.778.216
213687_s_at	6.86e-20	1.38e-20	9.777.589
215032_at	6.86e-20	1.38e-20	-97.775
209209_s_at	6.93e-20	1.40e-20	-9.776.195

207429_at	6.99e-20	1.41e-20	-9.775.163
210558_at	7.03e-20	1.42e-20	-9.774.381
202315_s_at	7.08e-20	1.43e-20	9.773.575
208343_s_at	7.08e-20	1.43e-20	9.773.534
221311_x_at	7.08e-20	1.43e-20	9.773.368
203225_s_at	7.12e-20	1.44e-20	977.265
202039_at	7.13e-20	1.44e-20	-9.772.558
212485_at	7.15e-20	1.44e-20	9.772.074
212503_s_at	7.20e-20	1.45e-20	-9.771.283
215203_at	7.29e-20	1.47e-20	-9.769.691
208850_s_at	7.32e-20	1.48e-20	97.691
221718_s_at	7.37e-20	1.49e-20	-9.768.313
218627_at	7.40e-20	1.50e-20	9.767.708
221536_s_at	7.54e-20	1.52e-20	9.765.393
213387_at	7.65e-20	1.55e-20	9.763.645
212481_s_at	7.74e-20	1.57e-20	9.762.162
219540_at	7.86e-20	1.59e-20	9.760.254
203059_s_at	8.00e-20	1.62e-20	-9.758.101
204776_at	8.00e-20	1.62e-20	9.758.077
216248_s_at	8.14e-20	1.65e-20	-9.755.827
212670_at	8.24e-20	1.67e-20	-9.754.305
205181_at	8.27e-20	1.67e-20	9.753.905
206120_at	8.31e-20	1.68e-20	-9.753.297
218405_at	8.32e-20	1.69e-20	9.753.044
212711_at	8.51e-20	1.72e-20	9.750.232
210782_x_at	8.58e-20	1.74e-20	-9.749.204
201026_at	8.60e-20	1.74e-20	9.748.929
201306_s_at	8.67e-20	1.76e-20	974.787
207856_s_at	8.69e-20	1.76e-20	9.747.528
209964_s_at	8.79e-20	1.78e-20	-9.746.197
210755_at	8.79e-20	1.78e-20	-9.746.068
200897_s_at	8.83e-20	1.79e-20	9.745.555
201685_s_at	8.98e-20	1.82e-20	9.743.436
218145_at	9.00e-20	1.83e-20	9.743.091
212827_at	9.13e-20	1.85e-20	-9.741.309
218402_s_at	9.17e-20	1.86e-20	9.740.762
218854_at	9.19e-20	1.87e-20	-9.740.428
203283_s_at	9.33e-20	1.90e-20	9.738.511
203760_s_at	9.45e-20	1.92e-20	-9.736.904
205478_at	9.47e-20	1.92e-20	-9.736.709
210990_s_at	9.52e-20	1.94e-20	-9.735.993
206741_at	9.56e-20	1.94e-20	9.735.427
204595_s_at	9.64e-20	1.96e-20	9.734.396
204999_s_at	9.73e-20	1.98e-20	-9.733.199

203146_s_at	9.77e-20	1.99e-20	-9.732.723
212422_at	9.79e-20	1.99e-20	9.732.399
220480_at	9.80e-20	2.00e-20	-9.732.227
210762_s_at	9.84e-20	2.00e-20	-9.731.783
206110_at	9.84e-20	2.01e-20	9.731.651
210962_s_at	9.87e-20	2.01e-20	-9.731.249
209417_s_at	1.01e-19	2.06e-20	9.728.389
206814_at	1.02e-19	2.08e-20	-9.727.138
216179_x_at	1.02e-19	2.09e-20	-9.726.598
222281_s_at	1.03e-19	2.10e-20	9.725.678
206643_at	1.05e-19	2.14e-20	-9.723.409
218944_at	1.07e-19	2.17e-20	9.721.648
209122_at	1.08e-19	2.21e-20	-9.719.557
212723_at	1.09e-19	2.22e-20	9.718.916
206213_at	1.09e-19	2.23e-20	-9.718.407
200036_s_at	1.11e-19	2.26e-20	9.716.957
219515_at	1.11e-19	2.27e-20	9.716.364
209158_s_at	1.12e-19	2.28e-20	9.715.861
210648_x_at	1.12e-19	2.28e-20	9.715.809
220622_at	1.12e-19	2.29e-20	9.715.366
200885_at	1.12e-19	2.29e-20	9.715.325
217940_s_at	1.19e-19	2.44e-20	-9.707.601
212483_at	1.22e-19	2.50e-20	9.704.662
218765_at	1.23e-19	2.52e-20	-9.703.614
216628_at	1.23e-19	2.52e-20	-970.323
206833_s_at	1.27e-19	2.61e-20	-9.699.341
217304_at	1.28e-19	2.61e-20	-9.699.078
208612_at	1.28e-19	2.63e-20	9.698.157
214438_at	1.29e-19	2.65e-20	-9.697.298
213086_s_at	1.30e-19	2.66e-20	9.696.855
204350_s_at	1.31e-19	2.68e-20	9.695.975
208191_x_at	1.36e-19	2.78e-20	-9.691.107
210979_at	1.36e-19	2.79e-20	-9.690.899
215136_s_at	1.40e-19	2.86e-20	9.687.691
213656_s_at	1.40e-19	2.88e-20	-9.687.031
209260_at	1.42e-19	2.91e-20	9.685.693
202202_s_at	1.42e-19	2.92e-20	9.685.289
213013_at	1.42e-19	2.92e-20	-9.685.095
210186_s_at	1.46e-19	3.01e-20	9.681.661
213017_at	1.47e-19	3.03e-20	9.680.827
219230_at	1.49e-19	3.05e-20	-9.679.769
213906_at	1.50e-19	3.08e-20	9.678.737
201698_s_at	1.51e-19	3.10e-20	967.793
205066_s_at	1.57e-19	3.23e-20	-9.672.629

214450_at	1.58e-19	3.25e-20	-9.672.019
221660_at	1.61e-19	3.31e-20	-9.669.706
204754_at	1.62e-19	3.34e-20	-9.668.771
205260_s_at	1.66e-19	3.41e-20	9.666.087
217677_at	1.67e-19	3.44e-20	-9.665.102
203345_s_at	1.67e-19	3.44e-20	9.664.997
204383_at	1.68e-19	3.46e-20	9.664.167
221508_at	1.68e-19	3.46e-20	9.664.105
214027_x_at	1.72e-19	3.54e-20	-9.661.248
221195_at	1.75e-19	3.61e-20	-9.658.984
206481_s_at	1.77e-19	3.64e-20	-965.782
222279_at	1.77e-19	3.65e-20	9.657.697
213329_at	1.77e-19	3.65e-20	9.657.649
221891_x_at	1.79e-19	3.70e-20	9.656.048
217926_at	1.80e-19	3.71e-20	9.655.577
213170_at	1.80e-19	3.72e-20	9.655.372
208322_s_at	1.80e-19	3.72e-20	-9.655.347
216844_at	1.83e-19	3.78e-20	-9.653.315
220871_at	1.88e-19	3.87e-20	-9.650.287
206417_at	1.90e-19	3.92e-20	-9.648.831
205040_at	1.93e-19	3.97e-20	-9.647.077
200001_at	1.96e-19	4.04e-20	9.644.939
221934_s_at	1.96e-19	4.05e-20	964.482
202415_s_at	1.99e-19	4.11e-20	9.642.992
218071_s_at	2.00e-19	4.13e-20	9.642.458
218220_at	2.02e-19	4.18e-20	9.640.895
215187_at	2.05e-19	4.23e-20	-9.639.304
212695_at	2.07e-19	4.29e-20	-9.637.682
201924_at	2.08e-19	4.29e-20	-9.637.567
212680_x_at	2.09e-19	4.31e-20	9.636.891
204652_s_at	2.09e-19	4.32e-20	9.636.776
206869_at	2.10e-19	4.35e-20	-9.635.774
49485_at	2.17e-19	4.49e-20	9.632.033
222001_x_at	2.18e-19	4.52e-20	9.631.217
216928_at	2.21e-19	4.58e-20	-9.629.423
216574_s_at	2.25e-19	4.66e-20	9.627.338
222336_at	2.25e-19	4.67e-20	-9.627.194
202903_at	2.27e-19	4.70e-20	9.626.336
204238_s_at	2.28e-19	4.72e-20	962.587
208078_s_at	2.29e-19	4.76e-20	-9.624.792
221095_s_at	2.33e-19	4.83e-20	-9.622.904
212491_s_at	2.36e-19	4.90e-20	9.621.203
222244_s_at	2.41e-19	5.00e-20	9.618.659
219687_at	2.42e-19	5.03e-20	961.796

221884_at	2.51e-19	5.21e-20	9.613.536
218461_at	2.52e-19	5.22e-20	9.613.228
211336_x_at	2.53e-19	5.25e-20	-961.242
212019_at	2.53e-19	5.26e-20	-9.612.247
213360_s_at	2.56e-19	5.31e-20	9.611.046
216625_at	2.58e-19	5.37e-20	-96.098
203074_at	2.58e-19	5.37e-20	-9.609.798
201285_at	2.59e-19	5.39e-20	9.609.295
218319_at	2.59e-19	5.39e-20	960.926
220292_at	2.70e-19	5.62e-20	-9.604.119
212837_at	2.71e-19	5.64e-20	9.603.593
217752_s_at	2.74e-19	5.71e-20	-9.602.151
208247_at	2.77e-19	5.77e-20	-9.600.832
209025_s_at	2.86e-19	5.94e-20	9.597.111
214943_s_at	2.87e-19	5.97e-20	9.596.534
210553_x_at	2.90e-19	6.04e-20	-9.595.158
210376_x_at	2.97e-19	6.18e-20	-9.592.304
201059_at	3.03e-19	6.31e-20	9.589.591
218258_at	3.06e-19	6.38e-20	9.588.326
32625_at	3.08e-19	6.41e-20	-9.587.693
203291_at	3.09e-19	6.44e-20	9.587.109
217150_s_at	3.10e-19	6.45e-20	-9.586.899
219315_s_at	3.11e-19	6.48e-20	-9.586.267
205446_s_at	3.12e-19	6.50e-20	9.585.868
201938_at	3.16e-19	6.59e-20	9.584.284
202451_at	3.17e-19	6.61e-20	958.382
200801_x_at	3.18e-19	6.64e-20	9.583.345
207627_s_at	3.20e-19	6.67e-20	9.582.739
212475_at	3.28e-19	6.84e-20	9.579.562
211921_x_at	3.28e-19	6.85e-20	9.579.337
221123_x_at	3.39e-19	7.07e-20	-9.575.424
205846_at	3.39e-19	7.08e-20	-9.575.289
207769_s_at	3.43e-19	7.17e-20	9.573.736
201514_s_at	3.43e-19	7.17e-20	9.573.721
218550_s_at	3.45e-19	7.21e-20	9.572.965
202668_at	3.47e-19	7.25e-20	9.572.316
202314_at	3.49e-19	7.30e-20	9.571.505
202671_s_at	3.49e-19	7.31e-20	9.571.345
209285_s_at	3.52e-19	7.35e-20	9.570.567
218936_s_at	3.56e-19	7.45e-20	9.568.986
204493_at	3.57e-19	7.48e-20	9.568.494
213554_s_at	3.59e-19	7.51e-20	9.567.925
221045_s_at	3.59e-19	7.52e-20	-9.567.758
210986_s_at	3.67e-19	7.69e-20	956.507

201529_s_at	3.71e-19	7.77e-20	9.563.791
200651_at	3.79e-19	7.94e-20	9.561.073
215997_s_at	3.82e-19	8.00e-20	956.009
213018_at	3.90e-19	8.18e-20	9.557.308
217225_x_at	3.94e-19	8.26e-20	9.556.053
220143_x_at	3.96e-19	8.29e-20	-9.555.588
214114_x_at	4.00e-19	8.39e-20	95.542
218906_x_at	4.01e-19	8.41e-20	955.388
220165_at	4.03e-19	8.45e-20	-9.553.268
218505_at	4.07e-19	8.54e-20	-9.551.989
202389_s_at	4.13e-19	8.66e-20	9.550.206
205380_at	4.14e-19	8.70e-20	9.549.633
215585_at	4.15e-19	8.71e-20	-9.549.507
218465_at	4.17e-19	8.75e-20	9.548.847
212363_x_at	4.25e-19	8.92e-20	9.546.444
209036_s_at	4.25e-19	8.93e-20	9.546.326
205301_s_at	4.25e-19	8.94e-20	9.546.217
209366_x_at	4.37e-19	9.19e-20	-9.542.761
205648_at	4.38e-19	9.20e-20	-954.264
209731_at	4.38e-19	9.20e-20	-9.542.613
207637_at	4.39e-19	9.23e-20	-9.542.273
222113_s_at	4.42e-19	9.30e-20	9.541.346
212440_at	4.44e-19	9.34e-20	9.540.754
222212_s_at	4.44e-19	9.35e-20	9.540.611
214144_at	4.47e-19	9.42e-20	9.539.759
208324_at	4.53e-19	9.54e-20	-9.538.091
204068_at	4.56e-19	9.60e-20	9.537.353
214708_at	4.61e-19	9.70e-20	9.535.984
202266_at	4.62e-19	9.73e-20	9.535.639
208942_s_at	4.62e-19	9.73e-20	-9.535.615
219149_x_at	4.80e-19	1.01e-19	9.530.698
205670_at	4.82e-19	1.02e-19	9.530.101
212415_at	4.91e-19	1.03e-19	9.528.008
212510_at	5.01e-19	1.06e-19	9.525.325
205002_at	5.04e-19	1.06e-19	-9.524.479
214358_at	5.06e-19	1.07e-19	9.523.984
209544_at	5.20e-19	1.10e-19	952.056
217471_at	5.42e-19	1.14e-19	-9.515.327
221558_s_at	5.44e-19	1.15e-19	9.514.979
209940_at	5.46e-19	1.15e-19	-951.449
213643_s_at	5.61e-19	1.18e-19	-9.511.077
202023_at	5.63e-19	1.19e-19	9.510.531
221810_at	5.64e-19	1.19e-19	9.510.268
216384_x_at	5.74e-19	1.21e-19	9.508.012

208683_at	5.77e-19	1.22e-19	9.507.392
201727_s_at	5.78e-19	1.22e-19	9.507.168
210022_at	5.87e-19	1.24e-19	9.505.258
204845_s_at	5.89e-19	1.25e-19	-9.504.649
219362_at	5.92e-19	1.25e-19	9.504.067
222311_s_at	5.94e-19	1.26e-19	-9.503.597
214336_s_at	5.95e-19	1.26e-19	9.503.275
220140_s_at	6.06e-19	1.28e-19	9.501.044
215068_s_at	6.06e-19	1.28e-19	9.500.952
201024_x_at	6.10e-19	1.29e-19	9.500.092
202906_s_at	6.19e-19	1.31e-19	9.498.349
217977_at	6.21e-19	1.32e-19	-949.788
209056_s_at	6.25e-19	1.32e-19	9.497.138
215227_x_at	6.27e-19	1.33e-19	9.496.546
219379_x_at	6.31e-19	1.34e-19	-9.495.879
211768_at	6.31e-19	1.34e-19	-949.586
201557_at	6.35e-19	1.35e-19	-9.494.988
204780_s_at	6.35e-19	1.35e-19	-9.494.837
210299_s_at	6.39e-19	1.36e-19	-9.494.167
204846_at	6.44e-19	1.37e-19	-9.493.211
218513_at	6.52e-19	1.38e-19	9.491.545
214505_s_at	6.68e-19	1.42e-19	-9.488.518
214372_x_at	6.73e-19	1.43e-19	-9.487.429
202789_at	6.77e-19	1.44e-19	9.486.664
213867_x_at	6.78e-19	1.44e-19	9.486.507
208860_s_at	6.83e-19	1.45e-19	9.485.645
203675_at	6.87e-19	1.46e-19	9.484.801
219639_x_at	6.87e-19	1.46e-19	-9.484.761
209518_at	6.98e-19	1.49e-19	9.482.746
201318_s_at	7.05e-19	1.50e-19	9.481.595
219487_at	7.07e-19	1.50e-19	9.481.188
205072_s_at	7.13e-19	1.52e-19	9.480.001
210399_x_at	7.22e-19	1.54e-19	-9.478.438
218377_s_at	7.39e-19	1.57e-19	-947.548
220789_s_at	7.41e-19	1.58e-19	9.475.092
34689_at	7.46e-19	1.59e-19	-9.474.326
212640_at	7.48e-19	1.59e-19	9.473.922
214387_x_at	7.54e-19	1.61e-19	-9.472.898
200070_at	7.59e-19	1.62e-19	9.471.946
217512_at	7.85e-19	1.67e-19	-9.467.771
210093_s_at	7.97e-19	1.70e-19	9.465.787
213758_at	8.12e-19	1.73e-19	-9.463.504
204154_at	8.12e-19	1.73e-19	-9.463.442
203762_s_at	8.12e-19	1.73e-19	9.463.388

200078_s_at	8.18e-19	1.75e-19	9.462.503
205754_at	8.24e-19	1.76e-19	-9.461.509
33736_at	8.29e-19	1.77e-19	9.460.797
205732_s_at	8.38e-19	1.79e-19	9.459.311
208950_s_at	8.56e-19	1.83e-19	-9.456.612
219809_at	8.59e-19	1.84e-19	945.613
218795_at	8.80e-19	1.88e-19	9.453.134
203272_s_at	9.00e-19	1.92e-19	9.450.302
215464_s_at	9.05e-19	1.93e-19	9.449.561
215855_s_at	9.05e-19	1.93e-19	-9.449.536
217043_s_at	9.05e-19	1.94e-19	9.449.519
208398_s_at	9.05e-19	1.94e-19	9.449.445
218144_s_at	9.06e-19	1.94e-19	-9.449.328
206302_s_at	9.14e-19	1.95e-19	9.448.251
201935_s_at	9.16e-19	1.96e-19	9.447.889
205781_at	9.32e-19	1.99e-19	-9.445.727
208698_s_at	9.38e-19	2.01e-19	9.444.809
214846_s_at	9.52e-19	2.04e-19	9.442.986
203556_at	9.66e-19	2.07e-19	9.441.162
203246_s_at	9.90e-19	2.12e-19	943.802
205898_at	9.94e-19	2.13e-19	-9.437.509
220725_x_at	1.00e-18	2.14e-19	-9.436.587
215602_at	1.02e-18	2.19e-19	-943.416
217748_at	1.03e-18	2.21e-19	9.432.722
218325_s_at	1.04e-18	2.23e-19	943.185
217960_s_at	1.04e-18	2.23e-19	9.431.637
219863_at	1.05e-18	2.25e-19	-9.430.381
200751_s_at	1.05e-18	2.26e-19	9.429.803
204912_at	1.06e-18	2.27e-19	-9.429.439
201493_s_at	1.06e-18	2.28e-19	9.428.961
202426_s_at	1.07e-18	2.31e-19	-9.427.418
202462_s_at	1.10e-18	2.36e-19	9.424.563
220479_at	1.10e-18	2.37e-19	-9.424.115
215942_s_at	1.11e-18	2.38e-19	9.423.669
208308_s_at	1.11e-18	2.39e-19	9.423.049
216507_at	1.13e-18	2.43e-19	-9.421.134
211133_x_at	1.15e-18	2.47e-19	-9.418.806
203015_s_at	1.15e-18	2.47e-19	9.418.615
206094_x_at	1.16e-18	2.49e-19	-9.418.017
55662_at	1.16e-18	2.49e-19	9.417.584
205431_s_at	1.16e-18	2.50e-19	-9.417.232
211435_at	1.16e-18	2.51e-19	-9.416.937
209363_s_at	1.22e-18	2.63e-19	9.410.903
208687_x_at	1.24e-18	2.66e-19	9.409.413

203050_at	1.25e-18	2.68e-19	9.408.376
216644_at	1.25e-18	2.70e-19	-9.407.707
218888_s_at	1.26e-18	2.71e-19	9.407.205
218766_s_at	1.27e-18	2.74e-19	9.405.558
206183_s_at	1.30e-18	2.79e-19	-9.403.343
205398_s_at	1.30e-18	2.81e-19	9.402.599
204405_x_at	1.31e-18	2.82e-19	9.402.022
215947_s_at	1.34e-18	2.89e-19	939.896
213778_x_at	1.36e-18	2.94e-19	-9.397.091
202703_at	1.37e-18	2.95e-19	9.396.264
211843_x_at	1.39e-18	2.99e-19	-9.394.758
213124_at	1.39e-18	3.00e-19	9.394.429
202262_x_at	1.40e-18	3.03e-19	9.392.963
200806_s_at	1.41e-18	3.05e-19	9.392.211
220734_s_at	1.41e-18	3.06e-19	-9.392.003
213729_at	1.45e-18	3.13e-19	9.389.119
203941_at	1.46e-18	3.15e-19	9.388.164
216836_s_at	1.46e-18	3.16e-19	-9.387.613
202286_s_at	1.47e-18	3.17e-19	-9.387.205
202303_x_at	1.48e-18	3.21e-19	9.385.896
213588_x_at	1.53e-18	3.31e-19	9.381.775
203474_at	1.54e-18	3.33e-19	-9.381.329
213649_at	1.55e-18	3.36e-19	-9.380.111
205545_x_at	1.58e-18	3.43e-19	9.377.432
203359_s_at	1.59e-18	3.44e-19	9.376.979
217997_at	1.59e-18	3.45e-19	-9.376.869
211317_s_at	1.59e-18	3.45e-19	-9.376.671
209226_s_at	1.60e-18	3.46e-19	9.376.484
200019_s_at	1.60e-18	3.47e-19	9.375.976
209821_at	1.65e-18	3.58e-19	-9.371.984
219130_at	1.67e-18	3.62e-19	937.048
209282_at	1.67e-18	3.63e-19	9.370.314
207795_s_at	1.72e-18	3.72e-19	-9.367.022
209842_at	1.73e-18	3.75e-19	-9.366.036
201871_s_at	1.74e-18	3.77e-19	9.365.354
201281_at	1.76e-18	3.82e-19	9.363.739
208791_at	1.77e-18	3.85e-19	-9.362.875
220987_s_at	1.78e-18	3.87e-19	9.362.143
210694_s_at	1.80e-18	3.91e-19	9.360.782
218930_s_at	1.86e-18	4.03e-19	9.356.935
211395_x_at	1.86e-18	4.04e-19	-9.356.659
207124_s_at	1.90e-18	4.13e-19	9.353.958
221223_x_at	1.90e-18	4.13e-19	-9.353.885
206121_at	1.92e-18	4.18e-19	-9.352.515

212151_at	1.95e-18	4.24e-19	-9.350.771
203701_s_at	1.96e-18	4.25e-19	9.350.253
52285_f_at	1.97e-18	4.28e-19	9.349.369
200958_s_at	1.99e-18	4.33e-19	9.347.982
219953_s_at	2.02e-18	4.39e-19	9.346.252
219591_at	2.02e-18	4.39e-19	-9.346.175
201297_s_at	2.03e-18	4.42e-19	9.345.259
222140_s_at	2.03e-18	4.43e-19	9.345.145
213748_at	2.04e-18	4.43e-19	-9.344.968
200858_s_at	2.05e-18	4.47e-19	9.343.833
214181_x_at	2.14e-18	4.66e-19	-9.338.795
213140_s_at	2.15e-18	4.69e-19	-9.337.916
218079_s_at	2.17e-18	4.72e-19	9.336.999
213535_s_at	2.21e-18	4.82e-19	9.334.468
208397_x_at	2.21e-18	4.82e-19	-9.334.316
208647_at	2.21e-18	4.83e-19	9.334.276
208454_s_at	2.21e-18	4.83e-19	9.334.165
215006_at	2.24e-18	4.90e-19	9.332.446
219330_at	2.25e-18	4.91e-19	9.331.985
217453_at	2.26e-18	4.93e-19	-9.331.426
207126_x_at	2.29e-18	4.99e-19	-9.330.057
203351_s_at	2.29e-18	4.99e-19	9.329.939
207785_s_at	2.36e-18	5.16e-19	9.325.693
220708_at	2.38e-18	5.19e-19	-9.324.939
211490_at	2.39e-18	5.21e-19	-9.324.462
200606_at	2.42e-18	5.28e-19	9.322.931
201744_s_at	2.43e-18	5.30e-19	-9.322.267
201010_s_at	2.46e-18	5.38e-19	-9.320.367
217292_at	2.48e-18	5.42e-19	-9.319.569
214203_s_at	2.51e-18	5.48e-19	-9.318.079
215125_s_at	2.52e-18	5.51e-19	-9.317.473
202685_s_at	2.55e-18	5.58e-19	-9.315.762
210146_x_at	2.59e-18	5.67e-19	-9.313.813
202770_s_at	2.62e-18	5.72e-19	9.312.616
218086_at	2.65e-18	5.81e-19	-9.310.796
205763_s_at	2.71e-18	5.94e-19	9.307.866
205472_s_at	2.76e-18	6.04e-19	-9.305.824
203158_s_at	2.78e-18	6.09e-19	9.304.755
218889_at	2.78e-18	6.09e-19	9.304.715
206414_s_at	2.80e-18	6.13e-19	9.304.016
213785_at	2.87e-18	6.29e-19	9.300.597
203497_at	2.89e-18	6.34e-19	9.299.629
215386_at	2.92e-18	6.41e-19	-9.298.305
218167_at	2.97e-18	6.52e-19	9.296.039

215200_x_at	3.05e-18	6.70e-19	-9.292.689
209454_s_at	3.10e-18	6.81e-19	9.290.609
202752_x_at	3.11e-18	6.81e-19	-9.290.504
215708_s_at	3.11e-18	6.83e-19	9.290.247
219226_at	3.13e-18	6.87e-19	9.289.471
206116_s_at	3.17e-18	6.97e-19	9.287.706
211345_x_at	3.18e-18	6.99e-19	9.287.281
210779_x_at	3.19e-18	7.00e-19	9.287.113
209753_s_at	3.24e-18	7.11e-19	9.285.109
200689_x_at	3.27e-18	7.18e-19	9.283.935
209119_x_at	3.29e-18	7.22e-19	-9.283.141
206016_at	3.32e-18	7.29e-19	9.281.839
207993_s_at	3.33e-18	7.32e-19	-9.281.438
212201_at	3.33e-18	7.32e-19	9.281.378
200604_s_at	3.34e-18	7.35e-19	9.280.795
204382_at	3.37e-18	7.42e-19	9.279.597
220642_x_at	3.43e-18	7.55e-19	9.277.395
201671_x_at	3.43e-18	7.56e-19	9.277.368
214103_s_at	3.46e-18	7.62e-19	-9.276.257
200682_s_at	3.50e-18	7.70e-19	9.274.957
203552_at	3.51e-18	7.74e-19	9.274.336
212124_at	3.53e-18	7.78e-19	9.273.683
205802_at	3.54e-18	7.80e-19	9.273.374
218917_s_at	3.55e-18	7.82e-19	9.273.067
213342_at	3.55e-18	7.82e-19	9.272.986
38158_at	3.55e-18	7.83e-19	9.272.817
201792_at	3.56e-18	7.84e-19	-9.272.627
208002_s_at	3.56e-18	7.85e-19	9.272.449
203842_s_at	3.58e-18	7.89e-19	-9.271.791
202054_s_at	3.59e-18	7.92e-19	-9.271.424
201409_s_at	3.63e-18	8.02e-19	9.269.827
208680_at	3.66e-18	8.08e-19	9.268.762
218926_at	3.84e-18	8.47e-19	9.262.879
204532_x_at	3.85e-18	8.50e-19	-9.262.345
204413_at	3.86e-18	8.52e-19	926.211
220617_s_at	3.86e-18	8.52e-19	9.262.036
204194_at	3.87e-18	8.56e-19	9.261.542
222024_s_at	3.89e-18	8.59e-19	-9.261.106
221954_at	3.89e-18	8.59e-19	9.261.022
216122_at	3.91e-18	8.65e-19	-9.260.118
200621_at	3.92e-18	8.66e-19	-9.260.021
222162_s_at	3.92e-18	8.66e-19	-9.260.019
213189_at	3.97e-18	8.78e-19	9.258.324
212616_at	3.99e-18	8.83e-19	-9.257.546

213262_at	4.05e-18	8.97e-19	9.255.499
213969_x_at	4.05e-18	8.97e-19	9.255.492
220346_at	4.05e-18	8.98e-19	-9.255.452
211979_at	4.08e-18	9.04e-19	9.254.538
37424_at	4.09e-18	9.07e-19	9.254.154
217932_at	4.11e-18	9.10e-19	9.253.752
209511_at	4.11e-18	9.11e-19	9.253.512
222235_s_at	4.14e-18	9.18e-19	9.252.646
221715_at	4.24e-18	9.39e-19	-924.973
203636_at	4.26e-18	9.44e-19	9.249.072
212075_s_at	4.37e-18	9.68e-19	9.245.823
208135_at	4.37e-18	9.68e-19	-9.245.798
215176_x_at	4.48e-18	9.94e-19	-9.242.401
203385_at	4.66e-18	1.03e-18	-9.237.349
202119_s_at	4.69e-18	1.04e-18	9.236.711
214908_s_at	4.72e-18	1.05e-18	9.235.717
212215_at	4.74e-18	1.05e-18	9.235.202
219397_at	4.74e-18	1.05e-18	9.235.083
206998_x_at	4.80e-18	1.07e-18	-9.233.502
203971_at	4.85e-18	1.08e-18	-923.224
204355_at	4.88e-18	1.08e-18	9.231.416
216658_at	4.89e-18	1.09e-18	-9.231.196
213199_at	5.00e-18	1.11e-18	9.228.229
201803_at	5.03e-18	1.12e-18	9.227.537
214676_x_at	5.08e-18	1.13e-18	-922.607
218052_s_at	5.13e-18	1.14e-18	9.224.793
222203_s_at	5.20e-18	1.16e-18	9.223.181
211595_s_at	5.24e-18	1.17e-18	9.222.026
213662_at	5.30e-18	1.18e-18	-922.057
215206_at	5.40e-18	1.20e-18	-9.218.104
208898_at	5.41e-18	1.20e-18	9.217.902
205639_at	5.54e-18	1.23e-18	-921.494
209921_at	5.56e-18	1.24e-18	9.214.446
212804_s_at	5.69e-18	1.27e-18	9.211.473
219549_s_at	5.78e-18	1.29e-18	9.209.477
218673_s_at	5.88e-18	1.31e-18	920.719
205550_s_at	5.90e-18	1.32e-18	9.206.609
213795_s_at	5.98e-18	1.33e-18	9.204.868
222100_at	6.04e-18	1.35e-18	-9.203.643
217881_s_at	6.06e-18	1.35e-18	920.311
203582_s_at	6.08e-18	1.36e-18	9.202.756
217516_x_at	6.14e-18	1.37e-18	-9.201.588
212403_at	6.16e-18	1.38e-18	9.200.971
213520_at	6.20e-18	1.38e-18	9.200.177

202653_s_at	6.24e-18	1.39e-18	9.199.358
201912_s_at	6.25e-18	1.40e-18	9.199.033
48612_at	6.30e-18	1.41e-18	-9.198.062
204622_x_at	6.31e-18	1.41e-18	-9.197.779
207864_at	6.40e-18	1.43e-18	-9.196.067
208549_x_at	6.47e-18	1.45e-18	9.194.622
217767_at	6.79e-18	1.52e-18	-9.188.293
203719_at	6.81e-18	1.52e-18	9.188.072
202641_at	6.82e-18	1.52e-18	9.187.799
218812_s_at	6.88e-18	1.54e-18	-9.186.714
212098_at	6.96e-18	1.56e-18	9.185.087
220607_x_at	7.00e-18	1.57e-18	9.184.377
208498_s_at	7.27e-18	1.63e-18	-9.179.526
220289_s_at	7.28e-18	1.63e-18	9.179.373
204416_x_at	7.35e-18	1.64e-18	-9.178.114
203894_at	7.38e-18	1.65e-18	9.177.534
219063_at	7.40e-18	1.66e-18	9.177.146
207156_at	7.41e-18	1.66e-18	9.176.854
213540_at	7.42e-18	1.66e-18	-9.176.786
201420_s_at	7.44e-18	1.67e-18	9.176.293
210701_at	7.61e-18	1.71e-18	-9.173.353
201497_x_at	7.61e-18	1.71e-18	-9.173.337
221883_at	7.65e-18	1.71e-18	9.172.768
204448_s_at	7.70e-18	1.73e-18	9.171.919
216770_at	8.03e-18	1.80e-18	-9.166.461
211582_x_at	8.07e-18	1.81e-18	-9.165.762
222309_at	8.09e-18	1.81e-18	9.165.509
219774_at	8.11e-18	1.82e-18	9.165.037
215318_at	8.18e-18	1.84e-18	-9.163.993
214140_at	8.18e-18	1.84e-18	9.163.979
218758_s_at	8.31e-18	1.87e-18	9.161.961
215633_x_at	8.37e-18	1.88e-18	-9.160.938
218131_s_at	8.44e-18	1.90e-18	9.159.826
213573_at	8.50e-18	1.91e-18	9.158.893
217294_s_at	8.61e-18	1.94e-18	9.157.268
212256_at	8.75e-18	1.97e-18	9.155.208
205067_at	8.87e-18	2.00e-18	-9.153.333
201936_s_at	8.90e-18	2.00e-18	9.152.954
211090_s_at	9.06e-18	2.04e-18	9.150.587
200785_s_at	9.14e-18	2.06e-18	-9.149.439
33132_at	9.16e-18	2.06e-18	9.149.185
36129_at	9.24e-18	2.08e-18	-9.148.017
214895_s_at	9.26e-18	2.09e-18	9.147.651
206419_at	9.33e-18	2.10e-18	-9.146.726

205933_at	9.33e-18	2.10e-18	-9.146.627
204574_s_at	9.50e-18	2.14e-18	-9.144.302
203580_s_at	9.61e-18	2.17e-18	9.142.866
212865_s_at	9.67e-18	2.18e-18	-9.142.038
221163_s_at	9.67e-18	2.18e-18	-9.141.982
218775_s_at	9.70e-18	2.19e-18	-9.141.559
207386_at	9.70e-18	2.19e-18	-9.141.558
221648_s_at	9.71e-18	2.19e-18	-9.141.335
217871_s_at	9.85e-18	2.22e-18	913.947
218496_at	9.86e-18	2.23e-18	913.935
205894_at	9.92e-18	2.24e-18	-9.138.618
207857_at	9.99e-18	2.26e-18	-9.137.564
202452_at	9.99e-18	2.26e-18	-9.137.552
200622_x_at	1.00e-17	2.27e-18	9.136.939
203040_s_at	1.02e-17	2.30e-18	9.135.374
201276_at	1.03e-17	2.33e-18	9.133.394
204097_s_at	1.03e-17	2.34e-18	9.133.081
203843_at	1.04e-17	2.36e-18	9.131.854
216291_at	1.06e-17	2.40e-18	-9.129.595
211999_at	1.06e-17	2.40e-18	9.129.385
213281_at	1.07e-17	2.42e-18	-9.128.582
219640_at	1.10e-17	2.49e-18	912.503
221781_s_at	1.11e-17	2.52e-18	9.123.483
200623_s_at	1.13e-17	2.56e-18	9.121.235
203853_s_at	1.14e-17	2.59e-18	9.120.054
209781_s_at	1.15e-17	2.60e-18	9.119.381
216748_at	1.16e-17	2.62e-18	-9.118.263
204547_at	1.19e-17	2.70e-18	9.114.404
205084_at	1.19e-17	2.70e-18	9.114.261
218752_at	1.20e-17	2.71e-18	9.114.094
205854_at	1.21e-17	2.75e-18	9.112.347
217992_s_at	1.21e-17	2.75e-18	9.111.982
203801_at	1.21e-17	2.75e-18	9.111.971
215726_s_at	1.23e-17	2.79e-18	-9.110.223
213493_at	1.24e-17	2.80e-18	-9.109.699
205382_s_at	1.24e-17	2.81e-18	-9.109.412
205302_at	1.25e-17	2.83e-18	-9.108.363
203885_at	1.25e-17	2.84e-18	9.108.025
221193_s_at	1.27e-17	2.89e-18	9.105.856
203319_s_at	1.28e-17	2.91e-18	9.104.692
213971_s_at	1.31e-17	2.98e-18	9.101.883
209507_at	1.31e-17	2.98e-18	910.175
215470_at	1.31e-17	2.98e-18	9.101.594
209674_at	1.34e-17	3.03e-18	909.945

201132_at	1.35e-17	3.06e-18	9.098.536
203278_s_at	1.36e-17	3.09e-18	9.097.061
211563_s_at	1.38e-17	3.14e-18	9.095.208
201371_s_at	1.38e-17	3.15e-18	9.094.752
210346_s_at	1.39e-17	3.15e-18	-9.094.565
219374_s_at	1.39e-17	3.16e-18	9.094.355
200084_at	1.39e-17	3.16e-18	909.417
202000_at	1.39e-17	3.17e-18	9.093.874
216175_at	1.41e-17	3.20e-18	-909.269
202520_s_at	1.41e-17	3.20e-18	9.092.495
217719_at	1.42e-17	3.23e-18	9.091.541
211214_s_at	1.42e-17	3.23e-18	-9.091.456
212212_s_at	1.46e-17	3.32e-18	9.087.772
214613_at	1.46e-17	3.33e-18	-9.087.434
219602_s_at	1.46e-17	3.33e-18	9.087.422
221708_s_at	1.49e-17	3.38e-18	9.085.394
220342_x_at	1.49e-17	3.39e-18	908.518
218277_s_at	1.49e-17	3.41e-18	9.084.512
206674_at	1.50e-17	3.42e-18	-9.083.899
203538_at	1.52e-17	3.47e-18	9.082.258
214048_at	1.54e-17	3.51e-18	-9.080.791
220238_s_at	1.55e-17	3.53e-18	9.079.877
208764_s_at	1.60e-17	3.65e-18	9.075.738
205521_at	1.61e-17	3.68e-18	9.074.699
212858_at	1.62e-17	3.69e-18	907.422
213843_x_at	1.63e-17	3.73e-18	9.072.878
202538_s_at	1.63e-17	3.73e-18	9.072.789
219032_x_at	1.64e-17	3.74e-18	9.072.403
208546_x_at	1.64e-17	3.75e-18	9.072.213
202888_s_at	1.65e-17	3.76e-18	-9.071.844
204011_at	1.65e-17	3.76e-18	-9.071.805
221807_s_at	1.65e-17	3.76e-18	9.071.743
201418_s_at	1.65e-17	3.77e-18	9.071.651
201532_at	1.67e-17	3.83e-18	9.069.506
203975_s_at	1.68e-17	3.85e-18	906.893
201962_s_at	1.68e-17	3.85e-18	9.068.792
206244_at	1.68e-17	3.85e-18	-9.068.737
204445_s_at	1.69e-17	3.86e-18	-9.068.401
37152_at	1.70e-17	3.90e-18	9.067.176
47608_at	1.71e-17	3.92e-18	9.066.335
213444_at	1.73e-17	3.97e-18	-9.064.816
216866_s_at	1.74e-17	3.99e-18	-9.064.302
217716_s_at	1.77e-17	4.06e-18	9.062.008
215798_at	1.77e-17	4.06e-18	-9.061.811

219296_at	1.78e-17	4.07e-18	9.061.549
210391_at	1.82e-17	4.17e-18	-9.058.484
211137_s_at	1.82e-17	4.18e-18	905.822
218599_at	1.84e-17	4.22e-18	-9.057.036
210059_s_at	1.85e-17	4.24e-18	905.646
213366_x_at	1.85e-17	4.24e-18	9.056.237
204019_s_at	1.86e-17	4.26e-18	-9.055.562
208628_s_at	1.89e-17	4.34e-18	9.053.314
213970_at	1.94e-17	4.44e-18	9.050.219
221401_at	1.98e-17	4.54e-18	-9.047.417
203694_s_at	2.01e-17	4.61e-18	9.045.371
203360_s_at	2.02e-17	4.64e-18	9.044.728
220939_s_at	2.05e-17	4.71e-18	9.042.691
220553_s_at	2.11e-17	4.84e-18	903.916
218232_at	2.11e-17	4.85e-18	-9.038.861
209576_at	2.12e-17	4.87e-18	9.038.409
220201_at	2.13e-17	4.91e-18	9.037.398
202459_s_at	2.14e-17	4.93e-18	-9.036.925
221184_at	2.14e-17	4.93e-18	-9.036.839
212424_at	2.16e-17	4.97e-18	9.035.697
45633_at	2.18e-17	5.01e-18	9.034.822
47069_at	2.18e-17	5.02e-18	-9.034.569
208649_s_at	2.19e-17	5.05e-18	9.033.732
205219_s_at	2.19e-17	5.05e-18	9.033.712
AFFX-HSAC07/X0035	2.23e-17	5.14e-18	9.031.466
213352_at	2.24e-17	5.15e-18	9.031.102
205654_at	2.24e-17	5.16e-18	-9.030.992
202955_s_at	2.27e-17	5.22e-18	9.029.362
220651_s_at	2.27e-17	5.23e-18	9.029.069
214177_s_at	2.28e-17	5.27e-18	9.028.265
221836_s_at	2.32e-17	5.35e-18	9.026.131
218803_at	2.39e-17	5.52e-18	9.022.143
220840_s_at	2.43e-17	5.62e-18	901.997
218226_s_at	2.44e-17	5.62e-18	9.019.772
202570_s_at	2.47e-17	5.70e-18	9.018.076
212946_at	2.47e-17	5.71e-18	-9.017.918
217865_at	2.48e-17	5.73e-18	-9.017.366
212374_at	2.49e-17	5.76e-18	9.016.687
201071_x_at	2.50e-17	5.77e-18	9.016.504
213989_x_at	2.52e-17	5.81e-18	9.015.506
204313_s_at	2.52e-17	5.81e-18	9.015.499
219340_s_at	2.55e-17	5.88e-18	-9.013.908
39835_at	2.55e-17	5.89e-18	9.013.863
212460_at	2.57e-17	5.95e-18	9.012.492

215778_x_at	2.60e-17	6.01e-18	-9.011.116
37586_at	2.63e-17	6.09e-18	900.953
221125_s_at	2.66e-17	6.15e-18	9.008.247
216881_x_at	2.70e-17	6.25e-18	-9.006.038
218605_at	2.70e-17	6.26e-18	9.005.968
211224_s_at	2.87e-17	6.64e-18	-8.998.203
207515_s_at	2.90e-17	6.71e-18	8.996.884
211156_at	2.90e-17	6.72e-18	-8.996.646
218318_s_at	2.96e-17	6.86e-18	8.994.089
210556_at	2.99e-17	6.92e-18	-899.284
200907_s_at	2.99e-17	6.93e-18	8.992.691
219443_at	3.00e-17	6.95e-18	899.232
207961_x_at	3.09e-17	7.15e-18	-8.988.557
204133_at	3.13e-17	7.26e-18	8.986.739
208052_x_at	3.17e-17	7.36e-18	-8.984.929
221714_s_at	3.19e-17	7.40e-18	-8.984.093
200683_s_at	3.21e-17	7.45e-18	8.983.352
214702_at	3.23e-17	7.49e-18	-8.982.625
210507_s_at	3.23e-17	7.50e-18	-8.982.392
208940_at	3.25e-17	7.55e-18	8.981.524
50314_i_at	3.25e-17	7.55e-18	8.981.516
220826_at	3.28e-17	7.61e-18	-8.980.554
201801_s_at	3.28e-17	7.61e-18	8.980.501
221730_at	3.30e-17	7.67e-18	8.979.481
219703_at	3.33e-17	7.73e-18	8.978.547
211036_x_at	3.35e-17	7.78e-18	8.977.732
214096_s_at	3.42e-17	7.94e-18	-8.975.034
216859_x_at	3.42e-17	7.95e-18	-8.974.787
201788_at	3.44e-17	8.00e-18	8.974.058
209861_s_at	3.46e-17	8.03e-18	8.973.516
214612_x_at	3.48e-17	8.09e-18	897.258
208517_x_at	3.49e-17	8.12e-18	8.972.156
215316_at	3.49e-17	8.13e-18	-8.971.981
211574_s_at	3.50e-17	8.14e-18	8.971.834
214574_x_at	3.53e-17	8.22e-18	-8.970.499
222150_s_at	3.54e-17	8.23e-18	-8.970.321
216578_at	3.61e-17	8.39e-18	-8.967.815
207542_s_at	3.74e-17	8.70e-18	-8.963.104
200630_x_at	3.75e-17	8.74e-18	896.248
210827_s_at	3.76e-17	8.76e-18	8.962.316
216010_x_at	3.76e-17	8.77e-18	-8.962.098
213290_at	3.79e-17	8.82e-18	-896.128
207648_at	3.80e-17	8.87e-18	-8.960.652
201444_s_at	3.83e-17	8.93e-18	895.973

206683_at	3.86e-17	9.01e-18	8.958.567
209942_x_at	3.88e-17	9.04e-18	895.812
215211_at	3.98e-17	9.29e-18	-8.954.532
212678_at	4.01e-17	9.36e-18	8.953.667
208646_at	4.02e-17	9.37e-18	8.953.429
1405_i_at	4.05e-17	9.45e-18	-8.952.392
207541_s_at	4.05e-17	9.46e-18	8.952.228
214706_at	4.07e-17	9.50e-18	8.951.698
220059_at	4.10e-17	9.58e-18	-8.950.663
220029_at	4.16e-17	9.71e-18	8.948.822
205477_s_at	4.19e-17	9.79e-18	-8.947.789
203829_at	4.19e-17	9.80e-18	8.947.685
201630_s_at	4.21e-17	9.85e-18	8.947.032
207063_at	4.25e-17	9.94e-18	-8.945.771
205290_s_at	4.27e-17	9.98e-18	8.945.269
221142_s_at	4.27e-17	9.99e-18	-8.945.152
200892_s_at	4.31e-17	1.01e-17	8.943.886
219673_at	4.40e-17	1.03e-17	8.941.129
209932_s_at	4.42e-17	1.03e-17	8.940.702
214965_at	4.45e-17	1.04e-17	-8.939.775
204451_at	4.46e-17	1.04e-17	-8.939.506
211457_at	4.48e-17	1.05e-17	-893.877
218522_s_at	4.50e-17	1.05e-17	8.938.213
209317_at	4.53e-17	1.06e-17	8.937.312
214152_at	4.64e-17	1.09e-17	-8.934.222
201601_x_at	4.65e-17	1.09e-17	-8.933.887
221641_s_at	4.66e-17	1.09e-17	8.933.402
203887_s_at	4.67e-17	1.09e-17	-8.933.265
201352_at	4.68e-17	1.10e-17	8.933.028
214663_at	4.69e-17	1.10e-17	8.932.604
211708_s_at	4.69e-17	1.10e-17	8.932.497
209127_s_at	4.76e-17	1.12e-17	8.930.522
214909_s_at	4.79e-17	1.12e-17	8.929.745
205371_s_at	4.80e-17	1.13e-17	-8.929.536
206335_at	4.81e-17	1.13e-17	-8.929.238
212801_at	4.84e-17	1.14e-17	89.285
218784_s_at	4.85e-17	1.14e-17	8.928.085
208720_s_at	4.86e-17	1.14e-17	892.782
203695_s_at	4.86e-17	1.14e-17	8.927.683
202564_x_at	4.86e-17	1.14e-17	8.927.651
215956_at	4.87e-17	1.14e-17	-8.927.504
211703_s_at	4.97e-17	1.17e-17	8.924.884
213184_at	5.01e-17	1.18e-17	892.375
201464_x_at	5.01e-17	1.18e-17	-8.923.606

213951_s_at	5.07e-17	1.19e-17	8.922.187
209882_at	5.09e-17	1.20e-17	8.921.658
204103_at	5.10e-17	1.20e-17	-8.921.314
202360_at	5.20e-17	1.22e-17	891.872
219298_at	5.30e-17	1.25e-17	-8.916.317
211484_s_at	5.32e-17	1.25e-17	-8.915.745
210950_s_at	5.40e-17	1.27e-17	89.136
219215_s_at	5.41e-17	1.27e-17	8.913.515
212658_at	5.45e-17	1.28e-17	8.912.437
221897_at	5.53e-17	1.30e-17	8.910.474
208351_s_at	5.54e-17	1.30e-17	8.910.353
36552_at	5.70e-17	1.34e-17	8.906.486
203553_s_at	5.78e-17	1.36e-17	8.904.694
33850_at	5.79e-17	1.36e-17	8.904.424
209692_at	5.79e-17	1.37e-17	-8.904.318
205165_at	5.88e-17	1.39e-17	8.902.413
220602_s_at	5.91e-17	1.39e-17	8.901.763
206932_at	5.93e-17	1.40e-17	-8.901.124
200768_s_at	6.01e-17	1.42e-17	8.899.522
218723_s_at	6.10e-17	1.44e-17	8.897.559
219079_at	6.21e-17	1.47e-17	8.895.078
211042_x_at	6.22e-17	1.47e-17	8.894.788
222033_s_at	6.26e-17	1.48e-17	8.893.908
212118_at	6.31e-17	1.49e-17	8.893.007
218444_at	6.31e-17	1.49e-17	8.892.963
218170_at	6.39e-17	1.51e-17	-8.891.186
206958_s_at	6.40e-17	1.51e-17	8.891.017
208792_s_at	6.54e-17	1.55e-17	-8.888.165
208853_s_at	6.58e-17	1.56e-17	8.887.357
205874_at	6.58e-17	1.56e-17	8.887.311
221146_at	6.58e-17	1.56e-17	-8.887.188
218602_s_at	6.60e-17	1.56e-17	8.886.855
214224_s_at	6.61e-17	1.56e-17	8.886.686
208249_s_at	6.69e-17	1.58e-17	-8.885.054
208480_s_at	6.71e-17	1.59e-17	-8.884.683
200613_at	6.72e-17	1.59e-17	8.884.458
200617_at	6.87e-17	1.63e-17	8.881.406
213133_s_at	6.88e-17	1.63e-17	-8.881.307
211909_x_at	6.89e-17	1.63e-17	-8.881.029
203405_at	6.91e-17	1.64e-17	8.880.631
214327_x_at	6.97e-17	1.65e-17	-8.879.497
207300_s_at	7.00e-17	1.66e-17	-8.878.972
207549_x_at	7.01e-17	1.66e-17	8.878.794
201593_s_at	7.05e-17	1.67e-17	8.877.874

205474_at	7.07e-17	1.68e-17	8.877.504
222206_s_at	7.11e-17	1.69e-17	8.876.726
217152_at	7.15e-17	1.70e-17	-887.601
212691_at	7.25e-17	1.72e-17	8.874.158
218419_s_at	7.33e-17	1.74e-17	8.872.634
209214_s_at	7.44e-17	1.77e-17	8.870.645
205114_s_at	7.45e-17	1.77e-17	-8.870.517
206470_at	7.47e-17	1.77e-17	8.870.078
203576_at	7.48e-17	1.78e-17	8.869.907
200721_s_at	7.51e-17	1.78e-17	8.869.464
221381_s_at	7.56e-17	1.80e-17	8.868.539
209338_at	7.65e-17	1.82e-17	8.866.857
201738_at	7.72e-17	1.83e-17	8.865.714
215134_at	7.90e-17	1.88e-17	8.862.699
219069_at	7.96e-17	1.89e-17	8.861.624
208920_at	7.97e-17	1.90e-17	8.861.405
209465_x_at	8.03e-17	1.91e-17	-8.860.425
204277_s_at	8.11e-17	1.93e-17	-8.859.094
207573_x_at	8.15e-17	1.94e-17	8.858.478
218974_at	8.19e-17	1.95e-17	8.857.744
219793_at	8.24e-17	1.96e-17	8.856.932
201999_s_at	8.26e-17	1.97e-17	8.856.696
206662_at	8.28e-17	1.97e-17	-8.856.338
210549_s_at	8.42e-17	2.00e-17	-8.854.131
205204_at	8.43e-17	2.01e-17	8.853.918
35776_at	8.44e-17	2.01e-17	-8.853.736
203818_s_at	8.44e-17	2.01e-17	8.853.615
219985_at	8.58e-17	2.04e-17	-8.851.526
202357_s_at	8.62e-17	2.05e-17	-8.850.914
202288_at	8.83e-17	2.10e-17	8.847.699
216481_at	8.87e-17	2.11e-17	-8.847.106
209091_s_at	8.88e-17	2.12e-17	8.846.852
211581_x_at	9.01e-17	2.15e-17	-8.845.001
211927_x_at	9.11e-17	2.17e-17	8.843.486
221428_s_at	9.25e-17	2.21e-17	8.841.413
211572_s_at	9.30e-17	2.22e-17	-884.072
204669_s_at	9.38e-17	2.24e-17	883.956
205116_at	9.60e-17	2.29e-17	-8.836.557
219753_at	9.77e-17	2.33e-17	-8.834.191
211743_s_at	9.83e-17	2.35e-17	-8.833.312
200968_s_at	9.87e-17	2.36e-17	8.832.805
219507_at	1.00e-16	2.39e-17	8.830.986
201244_s_at	1.00e-16	2.39e-17	8.830.855
211774_s_at	1.01e-16	2.43e-17	-8.829.043

209521_s_at	1.02e-16	2.43e-17	-8.828.754
53968_at	1.02e-16	2.44e-17	8.828.445
212153_at	1.02e-16	2.44e-17	8.828.287
205862_at	1.02e-16	2.45e-17	8.827.889
203860_at	1.02e-16	2.45e-17	-8.827.766
221616_s_at	1.02e-16	2.45e-17	-8.827.647
213410_at	1.03e-16	2.46e-17	8.827.344
217249_x_at	1.03e-16	2.47e-17	882.691
215849_x_at	1.04e-16	2.49e-17	-8.825.856
216929_x_at	1.04e-16	2.49e-17	-8.825.659
205987_at	1.05e-16	2.51e-17	-8.824.677
218579_s_at	1.08e-16	2.58e-17	8.821.165
215215_s_at	1.08e-16	2.58e-17	8.820.969
218867_s_at	1.08e-16	2.58e-17	8.820.882
210534_s_at	1.08e-16	2.58e-17	8.820.775
200946_x_at	1.08e-16	2.60e-17	-8.819.987
219998_at	1.10e-16	2.64e-17	8.818.137
216342_x_at	1.11e-16	2.65e-17	8.817.293
202272_s_at	1.11e-16	2.67e-17	88.166
213335_s_at	1.13e-16	2.71e-17	-8.814.714
222211_x_at	1.13e-16	2.71e-17	-8.814.594
204058_at	1.13e-16	2.71e-17	8.814.389
205213_at	1.14e-16	2.73e-17	-881.363
214192_at	1.14e-16	2.74e-17	-8.813.199
220792_at	1.16e-16	2.78e-17	-8.811.187
36907_at	1.16e-16	2.79e-17	-8.810.746
218135_at	1.16e-16	2.79e-17	8.810.625
219805_at	1.16e-16	2.79e-17	8.810.485
201951_at	1.16e-16	2.79e-17	881.045
201076_at	1.17e-16	2.81e-17	8.809.552
221620_s_at	1.18e-16	2.83e-17	8.809.006
221864_at	1.18e-16	2.83e-17	-8.808.944
219579_at	1.19e-16	2.87e-17	-8.806.762
219633_at	1.20e-16	2.90e-17	-8.805.771
201881_s_at	1.21e-16	2.91e-17	8.804.966
213596_at	1.21e-16	2.92e-17	-8.804.847
209956_s_at	1.21e-16	2.92e-17	-8.804.714
217617_at	1.22e-16	2.93e-17	-8.804.184
209336_at	1.23e-16	2.96e-17	8.803.087
213311_s_at	1.23e-16	2.96e-17	-8.802.833
201537_s_at	1.23e-16	2.97e-17	8.802.316
218077_s_at	1.24e-16	3.00e-17	8.801.186
214804_at	1.24e-16	3.00e-17	8.801.126
202982_s_at	1.27e-16	3.06e-17	-8.798.571

203508_at	1.29e-16	3.12e-17	-8.795.999
204208_at	1.30e-16	3.15e-17	8.794.859
212049_at	1.31e-16	3.16e-17	8.794.176
218003_s_at	1.31e-16	3.16e-17	8.794.089
214903_at	1.32e-16	3.18e-17	-8.793.553
208803_s_at	1.32e-16	3.18e-17	8.793.309
214670_at	1.33e-16	3.20e-17	-8.792.698
202936_s_at	1.33e-16	3.21e-17	8.792.062
214549_x_at	1.33e-16	3.22e-17	-8.791.909
215621_s_at	1.34e-16	3.24e-17	-8.791.164
200060_s_at	1.35e-16	3.25e-17	879.046
215083_at	1.35e-16	3.27e-17	-8.789.643
202190_at	1.36e-16	3.28e-17	8.789.548
202934_at	1.37e-16	3.32e-17	8.787.849
204848_x_at	1.39e-16	3.36e-17	-8.786.158
213292_s_at	1.42e-16	3.43e-17	878.338
220800_s_at	1.43e-16	3.45e-17	8.782.567
213123_at	1.44e-16	3.47e-17	8.781.776
221853_s_at	1.44e-16	3.48e-17	8.781.426
213848_at	1.45e-16	3.50e-17	8.780.651
219569_s_at	1.46e-16	3.54e-17	8.779.435
220750_s_at	1.47e-16	3.55e-17	8.778.779
219080_s_at	1.48e-16	3.58e-17	8.777.702
212108_at	1.54e-16	3.74e-17	8.772.145
218789_s_at	1.55e-16	3.74e-17	-8.771.949
214374_s_at	1.57e-16	3.80e-17	-8.770.107
209411_s_at	1.57e-16	3.81e-17	8.769.669
213464_at	1.59e-16	3.86e-17	-876.792
213957_s_at	1.61e-16	3.90e-17	8.766.454
204371_s_at	1.65e-16	3.99e-17	-8.763.516
205573_s_at	1.66e-16	4.03e-17	8.762.211
208943_s_at	1.74e-16	4.21e-17	-8.756.299
209061_at	1.74e-16	4.23e-17	8.755.739
222209_s_at	1.74e-16	4.23e-17	8.755.713
222028_at	1.74e-16	4.23e-17	8.755.708
222054_at	1.75e-16	4.25e-17	-8.755.247
202313_at	1.75e-16	4.26e-17	-8.754.977
220183_s_at	1.75e-16	4.26e-17	-8.754.859
217703_x_at	1.76e-16	4.28e-17	-8.754.313
210621_s_at	1.78e-16	4.31e-17	8.753.224
212720_at	1.82e-16	4.42e-17	8.750.027
206211_at	1.84e-16	4.47e-17	-874.848
213130_at	1.84e-16	4.48e-17	8.748.219
204189_at	1.84e-16	4.48e-17	-8.748.191

218384_at	1.85e-16	4.50e-17	-8.747.567
210800_at	1.87e-16	4.54e-17	-8.746.401
209795_at	1.87e-16	4.54e-17	-8.746.339
219043_s_at	1.87e-16	4.55e-17	8.746.114
210538_s_at	1.88e-16	4.57e-17	874.546
201082_s_at	1.91e-16	4.65e-17	8.743.269
205090_s_at	1.93e-16	4.70e-17	8.741.975
203745_at	1.96e-16	4.78e-17	8.739.747
37425_g_at	1.98e-16	4.82e-17	8.738.476
201388_at	1.99e-16	4.84e-17	8.737.873
212235_at	2.03e-16	4.95e-17	8.735.059
211172_x_at	2.07e-16	5.05e-17	-8.732.326
213524_s_at	2.10e-16	5.11e-17	-8.730.814
213555_at	2.11e-16	5.14e-17	8.730.111
210732_s_at	2.11e-16	5.15e-17	8.729.686
221172_at	2.12e-16	5.18e-17	-8.729.103
218310_at	2.13e-16	5.18e-17	8.728.854
212521_s_at	2.13e-16	5.19e-17	-8.728.836
209069_s_at	2.21e-16	5.38e-17	8.724.037
220990_s_at	2.23e-16	5.43e-17	8.722.748
207645_s_at	2.25e-16	5.49e-17	8.721.173
217894_at	2.28e-16	5.55e-17	8.719.757
203194_s_at	2.28e-16	5.55e-17	8.719.735
208750_s_at	2.28e-16	5.56e-17	8.719.672
203137_at	2.28e-16	5.57e-17	8.719.293
211260_at	2.35e-16	5.75e-17	-8.715.186
210074_at	2.38e-16	5.82e-17	8.713.612
210049_at	2.38e-16	5.82e-17	-8.713.473
218612_s_at	2.41e-16	5.89e-17	8.712.049
39817_s_at	2.44e-16	5.96e-17	8.710.299
208919_s_at	2.45e-16	5.98e-17	-8.710.041
218467_at	2.45e-16	5.98e-17	8.709.973
217803_at	2.46e-16	6.00e-17	8.709.391
206945_at	2.46e-16	6.02e-17	-8.709.073
209629_s_at	2.48e-16	6.07e-17	8.707.966
219495_s_at	2.51e-16	6.14e-17	870.636
204081_at	2.51e-16	6.15e-17	-8.706.261
209345_s_at	2.53e-16	6.20e-17	8.705.191
202369_s_at	2.53e-16	6.20e-17	-8.705.187
203110_at	2.56e-16	6.27e-17	-8.703.755
202176_at	2.57e-16	6.29e-17	8.703.291
209420_s_at	2.59e-16	6.34e-17	-8.702.284
212333_at	2.59e-16	6.34e-17	8.702.258
205188_s_at	2.59e-16	6.34e-17	8.702.177

204565_at	2.61e-16	6.39e-17	-8.701.218
202675_at	2.64e-16	6.46e-17	-869.972
213265_at	2.64e-16	6.47e-17	-8.699.494
205298_s_at	2.64e-16	6.47e-17	8.699.484
221941_at	2.64e-16	6.48e-17	-8.699.189
211644_x_at	2.67e-16	6.54e-17	-8.698.075
217822_at	2.71e-16	6.66e-17	8.695.746
214112_s_at	2.71e-16	6.66e-17	8.695.657
207517_at	2.75e-16	6.74e-17	-8.693.975
215444_s_at	2.76e-16	6.76e-17	8.693.582
215147_at	2.77e-16	6.79e-17	-8.693.039
207217_s_at	2.78e-16	6.82e-17	-8.692.488
219648_at	2.79e-16	6.85e-17	-8.691.832
209196_at	2.87e-16	7.05e-17	8.688.034
207643_s_at	2.91e-16	7.16e-17	-868.612
215408_at	2.98e-16	7.31e-17	-8.683.261
221749_at	2.98e-16	7.32e-17	8.683.079
205519_at	2.99e-16	7.34e-17	8.682.761
217008_s_at	3.07e-16	7.55e-17	-8.678.926
52651_at	3.11e-16	7.64e-17	-8.677.463
218919_at	3.11e-16	7.64e-17	8.677.386
202838_at	3.11e-16	7.65e-17	-867.715
209804_at	3.12e-16	7.68e-17	8.676.733
206827_s_at	3.13e-16	7.70e-17	-8.676.388
208288_at	3.13e-16	7.71e-17	-8.676.141
222019_at	3.14e-16	7.72e-17	8.675.958
200013_at	3.18e-16	7.84e-17	867.399
216499_at	3.18e-16	7.84e-17	-8.673.985
205784_x_at	3.19e-16	7.85e-17	-8.673.806
214881_s_at	3.19e-16	7.86e-17	8.673.596
204807_at	3.21e-16	7.90e-17	8.672.965
36084_at	3.21e-16	7.91e-17	8.672.716
209292_at	3.23e-16	7.97e-17	-8.671.794
205215_at	3.25e-16	8.01e-17	8.671.059
221814_at	3.28e-16	8.08e-17	-8.669.952
214819_at	3.29e-16	8.12e-17	-8.669.365
210854_x_at	3.35e-16	8.25e-17	8.667.123
211882_x_at	3.37e-16	8.31e-17	-8.666.262
220043_s_at	3.38e-16	8.33e-17	-8.665.872
221419_s_at	3.38e-16	8.33e-17	-8.665.828
218283_at	3.39e-16	8.36e-17	8.665.461
206099_at	3.41e-16	8.42e-17	-8.664.398
216806_at	3.46e-16	8.55e-17	8.662.481
207251_at	3.47e-16	8.56e-17	-8.662.316

216495_x_at	3.52e-16	8.69e-17	-8.660.233
213069_at	3.53e-16	8.71e-17	865.996
201122_x_at	3.53e-16	8.73e-17	-8.659.687
219631_at	3.58e-16	8.85e-17	8.657.817
209230_s_at	3.60e-16	8.91e-17	8.657.003
218737_at	3.61e-16	8.94e-17	8.656.559
215360_at	3.70e-16	9.16e-17	-8.653.305
206286_s_at	3.76e-16	9.31e-17	8.651.136
202258_s_at	3.78e-16	9.35e-17	-8.650.576
210932_s_at	3.78e-16	9.36e-17	-8.650.427
200989_at	3.79e-16	9.39e-17	8.650.017
208873_s_at	3.88e-16	9.61e-17	-8.646.809
211256_x_at	3.95e-16	9.78e-17	864.458
216411_s_at	3.97e-16	9.82e-17	8.644.027
205711_x_at	3.97e-16	9.83e-17	8.643.833
220723_s_at	3.98e-16	9.86e-17	-8.643.389
213705_at	4.00e-16	9.90e-17	-8.642.935
212127_at	4.02e-16	9.97e-17	8.641.962
209592_s_at	4.03e-16	9.98e-17	8.641.841
215609_at	4.12e-16	1.02e-16	-8.638.874
219516_at	4.12e-16	1.02e-16	-8.638.857
202052_s_at	4.14e-16	1.03e-16	8.637.932
213052_at	4.16e-16	1.03e-16	8.637.276
202033_s_at	4.18e-16	1.04e-16	8.636.661
203433_at	4.20e-16	1.04e-16	-8.636.063
221776_s_at	4.26e-16	1.06e-16	8.634.272
220251_at	4.30e-16	1.07e-16	8.633.033
32091_at	4.30e-16	1.07e-16	8.632.765
219048_at	4.38e-16	1.09e-16	8.630.273
207265_s_at	4.40e-16	1.09e-16	862.966
211280_s_at	4.41e-16	1.09e-16	-8.629.542
218495_at	4.48e-16	1.11e-16	8.627.248
210931_at	4.48e-16	1.11e-16	-8.627.247
37577_at	4.48e-16	1.11e-16	8.627.181
200089_s_at	4.53e-16	1.13e-16	8.625.775
209713_s_at	4.54e-16	1.13e-16	-8.625.461
208016_s_at	4.59e-16	1.14e-16	-862.403
216598_s_at	4.59e-16	1.14e-16	-8.623.878
217771_at	4.66e-16	1.16e-16	8.621.855
219538_at	4.67e-16	1.16e-16	8.621.628
206634_at	4.74e-16	1.18e-16	-8.619.576
211423_s_at	4.78e-16	1.19e-16	-8.618.513
206520_x_at	4.78e-16	1.19e-16	-8.618.421
208870_x_at	4.79e-16	1.19e-16	8.617.999

205289_at	4.82e-16	1.20e-16	8.617.102
212512_s_at	4.87e-16	1.21e-16	8.615.748
214168_s_at	4.88e-16	1.22e-16	8.615.383
204731_at	5.10e-16	1.27e-16	-8.609.576
203929_s_at	5.19e-16	1.29e-16	8.607.159
201467_s_at	5.22e-16	1.30e-16	8.606.478
202628_s_at	5.23e-16	1.30e-16	-8.606.116
200098_s_at	5.31e-16	1.32e-16	8.604.009
205041_s_at	5.36e-16	1.34e-16	-8.602.756
213483_at	5.39e-16	1.34e-16	8.602.072
211885_x_at	5.41e-16	1.35e-16	-8.601.439
209605_at	5.43e-16	1.35e-16	-8.601.094
202409_at	5.43e-16	1.35e-16	-8.600.984
203143_s_at	5.54e-16	1.38e-16	-8.598.378
221447_s_at	5.59e-16	1.40e-16	-8.597.081
213065_at	5.64e-16	1.41e-16	859.589
213760_s_at	5.65e-16	1.41e-16	-8.595.486
213126_at	5.67e-16	1.42e-16	8.595.038
211296_x_at	5.68e-16	1.42e-16	8.594.924
204867_at	5.91e-16	1.48e-16	-8.589.583
205895_s_at	6.01e-16	1.50e-16	8.587.186
203486_s_at	6.19e-16	1.55e-16	8.583.194
219286_s_at	6.21e-16	1.55e-16	8.582.857
216892_at	6.25e-16	1.56e-16	-8.581.963
214885_at	6.32e-16	1.58e-16	-8.580.334
209838_at	6.33e-16	1.58e-16	8.580.078
219283_at	6.33e-16	1.58e-16	8.580.027
202619_s_at	6.40e-16	1.60e-16	8.578.543
213142_x_at	6.49e-16	1.62e-16	-8.576.703
207180_s_at	6.49e-16	1.62e-16	8.576.675
203676_at	6.54e-16	1.64e-16	8.575.621
222275_at	6.55e-16	1.64e-16	8.575.454
214143_x_at	6.55e-16	1.64e-16	857.543
210208_x_at	6.62e-16	1.66e-16	8.574.027
211830_s_at	6.77e-16	1.69e-16	-8.570.995
200743_s_at	6.88e-16	1.72e-16	8.568.685
213647_at	6.90e-16	1.73e-16	8.568.333
211277_x_at	6.91e-16	1.73e-16	-8.568.201
209433_s_at	6.94e-16	1.74e-16	8.567.575
204540_at	6.94e-16	1.74e-16	856.741
215307_at	6.96e-16	1.74e-16	8.567.097
209426_s_at	7.00e-16	1.76e-16	-8.566.248
204353_s_at	7.02e-16	1.76e-16	8.565.887
205168_at	7.10e-16	1.78e-16	-8.564.248

200609_s_at	7.14e-16	1.79e-16	8.563.558
200756_x_at	7.26e-16	1.82e-16	8.561.249
200090_at	7.26e-16	1.82e-16	8.561.232
216542_x_at	7.35e-16	1.85e-16	-8.559.579
212120_at	7.50e-16	1.88e-16	8.556.866
203482_at	7.58e-16	1.90e-16	8.555.321
216402_at	7.60e-16	1.91e-16	-8.555.092
217758_s_at	7.67e-16	1.93e-16	8.553.789
205539_at	7.69e-16	1.93e-16	-8.553.347
212149_at	7.75e-16	1.95e-16	8.552.402
200015_s_at	7.76e-16	1.95e-16	8.552.128
219716_at	7.90e-16	1.99e-16	-8.549.743
210613_s_at	7.90e-16	1.99e-16	8.549.698
218282_at	7.92e-16	1.99e-16	854.927
218119_at	7.95e-16	2.00e-16	8.548.754
219452_at	8.20e-16	2.06e-16	-854.466
207163_s_at	8.23e-16	2.07e-16	-8.544.058
221766_s_at	8.25e-16	2.08e-16	-8.543.808
220828_s_at	8.36e-16	2.10e-16	-854.193
212499_s_at	8.38e-16	2.11e-16	8.541.518
211832_s_at	8.43e-16	2.12e-16	-8.540.703
204059_s_at	8.50e-16	2.14e-16	8.539.638
201604_s_at	8.51e-16	2.15e-16	8.539.365
219814_at	8.56e-16	2.16e-16	8.538.553
213860_x_at	8.66e-16	2.18e-16	8.536.968
207589_at	8.72e-16	2.20e-16	-8.536.147
202749_at	8.73e-16	2.20e-16	8.535.908
212632_at	8.74e-16	2.21e-16	8.535.659
202530_at	8.76e-16	2.21e-16	8.535.352
221925_s_at	8.80e-16	2.22e-16	8.534.806
205285_s_at	8.82e-16	2.23e-16	-8.534.424
210417_s_at	8.86e-16	2.23e-16	8.533.864
200981_x_at	8.92e-16	2.25e-16	8.532.902
200823_x_at	8.95e-16	2.26e-16	8.532.375
208096_s_at	8.99e-16	2.27e-16	8.531.827
203347_s_at	9.38e-16	2.37e-16	8.525.969
212117_at	9.40e-16	2.38e-16	8.525.662
201668_x_at	9.44e-16	2.38e-16	8.525.148
203598_s_at	9.49e-16	2.40e-16	852.433
203889_at	9.54e-16	2.41e-16	-8.523.594
213024_at	9.66e-16	2.44e-16	8.522.008
203558_at	9.67e-16	2.45e-16	852.174
212367_at	9.69e-16	2.45e-16	8.521.498
221750_at	9.89e-16	2.50e-16	8.518.695

201639_s_at	1.01e-15	2.54e-16	8.516.488
219289_at	1.01e-15	2.54e-16	8.516.475
209381_x_at	1.01e-15	2.56e-16	8.515.368
210177_at	1.02e-15	2.57e-16	-8.515.105
218192_at	1.02e-15	2.57e-16	851.508
218692_at	1.02e-15	2.57e-16	-8.514.995
211617_at	1.03e-15	2.60e-16	-8.513.478
202708_s_at	1.04e-15	2.63e-16	8.512.003
214767_s_at	1.07e-15	2.70e-16	-8.508.154
208039_at	1.07e-15	2.70e-16	-8.508.144
214456_x_at	1.08e-15	2.73e-16	-8.506.737
212961_x_at	1.09e-15	2.77e-16	8.504.718
213769_at	1.11e-15	2.83e-16	-850.226
215588_x_at	1.12e-15	2.84e-16	-8.501.749
201724_s_at	1.12e-15	2.84e-16	8.501.552
206549_at	1.13e-15	2.87e-16	-8.499.954
201319_at	1.13e-15	2.88e-16	849.979
202677_at	1.14e-15	2.89e-16	8.499.154
205250_s_at	1.14e-15	2.90e-16	8.498.839
214873_at	1.14e-15	2.90e-16	-8.498.671
210638_s_at	1.16e-15	2.94e-16	8.497.021
203446_s_at	1.16e-15	2.94e-16	8.496.844
210231_x_at	1.16e-15	2.95e-16	8.496.422
201966_at	1.17e-15	2.97e-16	8.495.714
213318_s_at	1.18e-15	3.00e-16	8.494.174
222177_s_at	1.20e-15	3.04e-16	-8.492.416
201634_s_at	1.20e-15	3.05e-16	8.491.824
218659_at	1.20e-15	3.06e-16	8.491.482
219347_at	1.22e-15	3.09e-16	8.490.227
65635_at	1.23e-15	3.14e-16	-8.488.118
218161_s_at	1.24e-15	3.15e-16	8.487.621
211538_s_at	1.24e-15	3.15e-16	8.487.407
207700_s_at	1.24e-15	3.16e-16	8.487.358
216986_s_at	1.26e-15	3.21e-16	-8.484.915
205020_s_at	1.27e-15	3.24e-16	8.483.949
209258_s_at	1.27e-15	3.24e-16	8.483.805
218357_s_at	1.28e-15	3.26e-16	8.483.099
203850_s_at	1.28e-15	3.27e-16	-8.482.539
220054_at	1.33e-15	3.38e-16	-8.478.235
220920_at	1.33e-15	3.38e-16	-8.478.151
212877_at	1.33e-15	3.38e-16	8.477.877
220232_at	1.33e-15	3.39e-16	-8.477.545
209934_s_at	1.33e-15	3.39e-16	8.477.541
201489_at	1.36e-15	3.48e-16	-8.474.257

213070_at	1.37e-15	3.49e-16	8.473.707
205263_at	1.37e-15	3.50e-16	847.334
209568_s_at	1.39e-15	3.56e-16	-8.471.236
214340_at	1.44e-15	3.67e-16	-8.466.923
206045_s_at	1.45e-15	3.69e-16	-8.466.092
206926_s_at	1.45e-15	3.71e-16	-8.465.427
202252_at	1.47e-15	3.75e-16	8.464.138
220641_at	1.48e-15	3.77e-16	-8.463.468
218267_at	1.48e-15	3.78e-16	8.463.084
203888_at	1.51e-15	3.85e-16	-846.057
221569_at	1.51e-15	3.86e-16	-8.460.128
204986_s_at	1.52e-15	3.89e-16	8.459.094
214011_s_at	1.56e-15	3.98e-16	8.456.025
201060_x_at	1.56e-15	3.99e-16	-8.455.545
216092_s_at	1.57e-15	4.02e-16	-8.454.525
208642_s_at	1.59e-15	4.05e-16	8.453.447
201356_at	1.60e-15	4.09e-16	8.452.208
217649_at	1.61e-15	4.12e-16	-8.451.327
202978_s_at	1.61e-15	4.12e-16	8.451.309
216019_x_at	1.62e-15	4.14e-16	-8.450.754
215607_x_at	1.63e-15	4.17e-16	-844.955
214429_at	1.64e-15	4.21e-16	8.448.419
221347_at	1.66e-15	4.24e-16	-8.447.475
205310_at	1.66e-15	4.24e-16	8.447.408
218134_s_at	1.66e-15	4.24e-16	8.447.394
205845_at	1.66e-15	4.24e-16	-8.447.331
215064_at	1.66e-15	4.24e-16	-844.731
217826_s_at	1.67e-15	4.27e-16	8.446.318
216233_at	1.68e-15	4.30e-16	-8.445.415
216996_s_at	1.69e-15	4.33e-16	8.444.618
211162_x_at	1.71e-15	4.37e-16	8.443.175
203394_s_at	1.71e-15	4.38e-16	-8.442.958
216207_x_at	1.71e-15	4.38e-16	-8.442.874
212136_at	1.71e-15	4.39e-16	844.257
201644_at	1.72e-15	4.40e-16	8.442.367
200972_at	1.73e-15	4.44e-16	8.441.233
212225_at	1.74e-15	4.46e-16	-8.440.618
221527_s_at	1.77e-15	4.53e-16	8.438.347
211553_x_at	1.77e-15	4.55e-16	-8.437.821
214907_at	1.78e-15	4.58e-16	-8.437.022
201929_s_at	1.80e-15	4.63e-16	843.551
219314_s_at	1.81e-15	4.65e-16	8.434.822
202172_at	1.85e-15	4.75e-16	8.432.035
202858_at	1.86e-15	4.77e-16	8.431.382

220618_s_at	1.86e-15	4.78e-16	-8.431.099
218991_at	1.88e-15	4.82e-16	8.429.995
219077_s_at	1.89e-15	4.86e-16	-8.428.927
204857_at	1.92e-15	4.93e-16	-8.426.912
213237_at	1.92e-15	4.93e-16	8.426.856
203779_s_at	1.94e-15	5.00e-16	8.425.174
219311_at	1.95e-15	5.00e-16	842.492
216609_at	1.96e-15	5.03e-16	8.424.281
212955_s_at	1.98e-15	5.10e-16	842.234
210321_at	1.99e-15	5.13e-16	-8.421.608
209432_s_at	2.03e-15	5.23e-16	841.901
201983_s_at	2.03e-15	5.23e-16	-8.418.971
220260_at	2.05e-15	5.28e-16	8.417.629
215812_s_at	2.05e-15	5.29e-16	8.417.405
207724_s_at	2.06e-15	5.30e-16	8.417.061
213592_at	2.07e-15	5.34e-16	-8.416.191
36742_at	2.12e-15	5.46e-16	-8.413.141
218941_at	2.13e-15	5.49e-16	8.412.455
203063_at	2.14e-15	5.52e-16	8.411.695
219380_x_at	2.14e-15	5.52e-16	-8.411.602
203312_x_at	2.17e-15	5.58e-16	8.410.044
212196_at	2.20e-15	5.67e-16	-8.407.985
219405_at	2.20e-15	5.67e-16	8.407.928
203096_s_at	2.21e-15	5.70e-16	-8.407.248
212241_at	2.22e-15	5.73e-16	8.406.585
216446_at	2.23e-15	5.74e-16	-8.406.215
220941_s_at	2.26e-15	5.83e-16	-8.404.262
204837_at	2.26e-15	5.83e-16	-8.404.066
201229_s_at	2.28e-15	5.87e-16	8.403.164
204812_at	2.29e-15	5.90e-16	8.402.528
219166_at	2.30e-15	5.93e-16	8.401.952
203224_at	2.31e-15	5.96e-16	8.401.264
36545_s_at	2.32e-15	5.98e-16	8.400.679
200896_x_at	2.32e-15	5.99e-16	8.400.476
218932_at	2.36e-15	6.09e-16	83.983
207651_at	2.36e-15	6.10e-16	-8.397.955
210657_s_at	2.36e-15	6.11e-16	-8.397.852
204441_s_at	2.37e-15	6.13e-16	8.397.379
219576_at	2.38e-15	6.16e-16	-8.396.751
204354_at	2.39e-15	6.17e-16	8.396.422
201255_x_at	2.39e-15	6.17e-16	8.396.366
200815_s_at	2.41e-15	6.24e-16	8.394.944
218949_s_at	2.42e-15	6.26e-16	8.394.609
216157_at	2.43e-15	6.28e-16	-8.394.053

202013_s_at	2.44e-15	6.31e-16	8.393.345
216779_at	2.45e-15	6.35e-16	-8.392.583
213990_s_at	2.46e-15	6.37e-16	-8.392.086
211185_s_at	2.53e-15	6.55e-16	8.388.424
218827_s_at	2.59e-15	6.71e-16	8.385.008
200729_s_at	2.59e-15	6.72e-16	8.384.853
205804_s_at	2.61e-15	6.76e-16	-8.384.129
203638_s_at	2.61e-15	6.76e-16	-8.384.125
205788_s_at	2.72e-15	7.04e-16	8.378.446
220574_at	2.74e-15	7.10e-16	-8.377.418
211995_x_at	2.74e-15	7.10e-16	8.377.417
209443_at	2.74e-15	7.10e-16	-8.377.302
218013_x_at	2.76e-15	7.17e-16	8.376.041
209388_at	2.80e-15	7.25e-16	8.374.484
212893_at	2.80e-15	7.26e-16	837.439
212393_at	2.81e-15	7.30e-16	8.373.562
39966_at	2.86e-15	7.42e-16	8.371.346
212555_at	2.87e-15	7.46e-16	-8.370.665
203178_at	2.88e-15	7.47e-16	-8.370.398
220020_at	2.88e-15	7.48e-16	-8.370.296
210381_s_at	2.88e-15	7.49e-16	-8.370.112
218300_at	2.91e-15	7.55e-16	8.368.988
217898_at	2.93e-15	7.62e-16	8.367.755
211598_x_at	2.96e-15	7.70e-16	-8.366.236
211848_s_at	2.97e-15	7.71e-16	-8.366.161
213274_s_at	2.98e-15	7.74e-16	-8.365.633
208945_s_at	3.00e-15	7.81e-16	836.433
216272_x_at	3.01e-15	7.84e-16	-8.363.916
205270_s_at	3.02e-15	7.85e-16	-8.363.624
214916_x_at	3.02e-15	7.86e-16	-8.363.574
221995_s_at	3.07e-15	7.98e-16	-8.361.498
217941_s_at	3.13e-15	8.13e-16	8.358.819
207525_s_at	3.14e-15	8.18e-16	8.358.086
217861_s_at	3.14e-15	8.18e-16	8.358.008
213252_at	3.16e-15	8.24e-16	-8.357.057
202573_at	3.19e-15	8.31e-16	8.355.812
202269_x_at	3.21e-15	8.36e-16	-8.355.101
220047_at	3.21e-15	8.36e-16	8.355.011
210493_s_at	3.22e-15	8.38e-16	-8.354.739
200075_s_at	3.26e-15	8.50e-16	8.352.732
213838_at	3.29e-15	8.58e-16	8.351.588
203763_at	3.31e-15	8.63e-16	8.350.686
218389_s_at	3.33e-15	8.68e-16	8.349.952
205687_at	3.34e-15	8.71e-16	8.349.475

202689_at	3.36e-15	8.76e-16	8.348.735
210330_at	3.37e-15	8.78e-16	-8.348.392
202769_at	3.49e-15	9.11e-16	8.343.312
202455_at	3.50e-15	9.13e-16	8.343.019
211080_s_at	3.50e-15	9.15e-16	8.342.773
207388_s_at	3.51e-15	9.17e-16	-8.342.497
212354_at	3.51e-15	9.17e-16	8.342.439
219883_at	3.55e-15	9.26e-16	-8.341.075
202031_s_at	3.55e-15	9.28e-16	8.340.876
213700_s_at	3.57e-15	9.34e-16	8.339.992
211043_s_at	3.58e-15	9.35e-16	8.339.742
222133_s_at	3.68e-15	9.61e-16	8.336.055
218848_at	3.69e-15	9.65e-16	8.335.449
219295_s_at	3.71e-15	9.70e-16	8.334.771
201274_at	3.73e-15	9.74e-16	8.334.176
218463_s_at	3.83e-15	1.00e-15	8.330.455
201742_x_at	3.85e-15	1.01e-15	832.969
36499_at	3.87e-15	1.01e-15	8.328.915
204402_at	3.90e-15	1.02e-15	8.327.724
214196_s_at	3.91e-15	1.02e-15	832.759
214032_at	3.91e-15	1.02e-15	-8.327.382
204655_at	3.93e-15	1.03e-15	-8.326.685
200615_s_at	3.94e-15	1.03e-15	8.326.221
220945_x_at	3.97e-15	1.04e-15	8.325.435
201823_s_at	4.02e-15	1.05e-15	8.323.434
213465_s_at	4.05e-15	1.06e-15	8.322.615
217831_s_at	4.09e-15	1.07e-15	8.321.076
218323_at	4.15e-15	1.09e-15	8.319.139
214724_at	4.17e-15	1.09e-15	-8.318.502
212396_s_at	4.17e-15	1.09e-15	831.839
206810_at	4.17e-15	1.09e-15	-8.318.228
212684_at	4.30e-15	1.13e-15	831.406
218674_at	4.32e-15	1.13e-15	8.313.477
215731_s_at	4.34e-15	1.14e-15	8.312.908
215868_x_at	4.37e-15	1.15e-15	-8.311.906
215557_at	4.38e-15	1.15e-15	-8.311.505
221110_x_at	4.48e-15	1.18e-15	-8.308.369
221023_s_at	4.52e-15	1.19e-15	-8.307.256
214918_at	4.53e-15	1.19e-15	-8.306.911
202213_s_at	4.53e-15	1.19e-15	8.306.818
208596_s_at	4.55e-15	1.20e-15	-8.306.193
215341_at	4.55e-15	1.20e-15	-8.306.149
201286_at	4.57e-15	1.20e-15	-8.305.482
221513_s_at	4.61e-15	1.21e-15	8.304.426

200690_at	4.62e-15	1.21e-15	8.304.024
208337_s_at	4.62e-15	1.21e-15	8.304.019
216332_at	4.64e-15	1.22e-15	-8.303.492
204517_at	4.65e-15	1.22e-15	8.303.156
220221_at	4.66e-15	1.23e-15	-830.271
219923_at	4.68e-15	1.23e-15	8.302.142
211410_x_at	4.68e-15	1.23e-15	-8.302.063
212878_s_at	4.70e-15	1.24e-15	8.301.496
203726_s_at	4.76e-15	1.25e-15	8.299.784
201014_s_at	4.86e-15	1.28e-15	8.296.817
204748_at	4.88e-15	1.28e-15	-8.296.338
53076_at	4.88e-15	1.29e-15	8.296.201
205110_s_at	4.90e-15	1.29e-15	8.295.746
203468_at	4.92e-15	1.30e-15	-8.295.108
211939_x_at	4.94e-15	1.30e-15	8.294.498
212231_at	4.95e-15	1.30e-15	-8.294.222
214146_s_at	4.95e-15	1.31e-15	-8.294.052
201934_at	5.00e-15	1.32e-15	8.292.913
40612_at	5.02e-15	1.32e-15	-8.292.156
219132_at	5.09e-15	1.34e-15	-8.290.403
216601_at	5.13e-15	1.35e-15	-8.289.287
203640_at	5.22e-15	1.38e-15	-8.286.684
217915_s_at	5.37e-15	1.42e-15	8.282.888
209303_at	5.38e-15	1.42e-15	8.282.526
207532_at	5.43e-15	1.43e-15	-8.281.215
220091_at	5.44e-15	1.44e-15	828.095
209047_at	5.50e-15	1.45e-15	-8.279.473
205516_x_at	5.50e-15	1.45e-15	8.279.373
219650_at	5.50e-15	1.45e-15	8.279.311
217067_s_at	5.55e-15	1.46e-15	-8.278.261
214373_at	5.57e-15	1.47e-15	8.277.532
55093_at	5.63e-15	1.49e-15	8.276.244
221734_at	5.66e-15	1.50e-15	8.275.438
217288_at	5.69e-15	1.50e-15	-8.274.616
209947_at	5.76e-15	1.52e-15	8.272.949
218491_s_at	5.76e-15	1.52e-15	-8.272.945
210582_s_at	5.81e-15	1.54e-15	8.271.728
201361_at	5.81e-15	1.54e-15	8.271.656
215372_x_at	5.85e-15	1.55e-15	-8.270.713
204358_s_at	5.85e-15	1.55e-15	-8.270.652
219274_at	5.92e-15	1.57e-15	-8.269.107
210085_s_at	5.93e-15	1.57e-15	8.268.673
204887_s_at	6.02e-15	1.59e-15	826.663
206499_s_at	6.07e-15	1.61e-15	8.265.617

203581_at	6.09e-15	1.61e-15	8.265.036
215209_at	6.21e-15	1.64e-15	-8.262.387
203053_at	6.22e-15	1.65e-15	8.262.048
218199_s_at	6.37e-15	1.69e-15	8.258.706
216882_s_at	6.38e-15	1.69e-15	-8.258.617
217868_s_at	6.39e-15	1.69e-15	8.258.208
212229_s_at	6.43e-15	1.71e-15	-8.257.346
201814_at	6.45e-15	1.71e-15	8.256.919
205312_at	6.48e-15	1.72e-15	-8.256.391
215405_at	6.55e-15	1.74e-15	-8.254.866
210496_at	6.64e-15	1.76e-15	-8.252.959
206701_x_at	6.69e-15	1.77e-15	-825.189
221935_s_at	6.71e-15	1.78e-15	8.251.396
32723_at	6.78e-15	1.80e-15	8.249.886
206626_x_at	6.98e-15	1.85e-15	8.246.023
219957_at	7.04e-15	1.87e-15	-82.447
205273_s_at	7.10e-15	1.89e-15	8.243.503
221042_s_at	7.13e-15	1.89e-15	-824.297
203172_at	7.21e-15	1.92e-15	-8.241.332
221253_s_at	7.22e-15	1.92e-15	8.241.203
218845_at	7.24e-15	1.92e-15	8.240.743
215375_x_at	7.25e-15	1.93e-15	-8.240.639
207658_s_at	7.26e-15	1.93e-15	-8.240.319
209278_s_at	7.30e-15	1.94e-15	-8.239.483
213256_at	7.38e-15	1.96e-15	8.238.097
200780_x_at	7.47e-15	1.99e-15	8.236.417
210952_at	7.53e-15	2.00e-15	-8.235.286
219817_at	7.59e-15	2.02e-15	8.234.131
214205_x_at	7.67e-15	2.04e-15	8.232.643
212373_at	7.70e-15	2.05e-15	8.232.019
216870_x_at	7.71e-15	2.05e-15	8.231.881
200870_at	7.88e-15	2.10e-15	8.228.869
203803_at	7.95e-15	2.12e-15	-8.227.637
202376_at	7.97e-15	2.12e-15	-8.227.136
215967_s_at	7.98e-15	2.12e-15	-8.227.062
217289_s_at	8.03e-15	2.14e-15	-8.226.078
220815_at	8.05e-15	2.14e-15	-8.225.793
213879_at	8.07e-15	2.15e-15	8.225.416
202809_s_at	8.07e-15	2.15e-15	8.225.323
209215_at	8.16e-15	2.18e-15	8.223.785
207233_s_at	8.26e-15	2.20e-15	-8.222.038
205525_at	8.28e-15	2.21e-15	-8.221.758
204518_s_at	8.33e-15	2.22e-15	8.220.974
211730_s_at	8.36e-15	2.23e-15	8.220.418

221057_at	8.39e-15	2.24e-15	-821.979
210372_s_at	8.46e-15	2.26e-15	-8.218.646
222011_s_at	8.48e-15	2.26e-15	8.218.333
208904_s_at	8.65e-15	2.31e-15	8.215.656
218569_s_at	8.65e-15	2.31e-15	8.215.599
208973_at	8.67e-15	2.32e-15	8.215.142
217336_at	8.68e-15	2.32e-15	8.214.987
208163_s_at	8.75e-15	2.34e-15	-8.213.953
218102_at	8.81e-15	2.35e-15	-8.213.001
201346_at	8.85e-15	2.36e-15	-8.212.323
214548_x_at	8.86e-15	2.37e-15	8.212.172
212233_at	8.89e-15	2.38e-15	8.211.567
203511_s_at	8.95e-15	2.39e-15	8.210.681
208961_s_at	8.98e-15	2.40e-15	-8.210.236
209675_s_at	8.98e-15	2.40e-15	8.210.164
219725_at	8.98e-15	2.40e-15	8.210.069
201952_at	9.00e-15	2.41e-15	8.209.814
212994_at	9.00e-15	2.41e-15	8.209.703
215775_at	9.03e-15	2.42e-15	-8.209.223
218370_s_at	9.11e-15	2.44e-15	8.208.078
204362_at	9.19e-15	2.46e-15	8.206.792
203241_at	9.26e-15	2.48e-15	8.205.755
214270_s_at	9.26e-15	2.48e-15	-8.205.729
215894_at	9.30e-15	2.49e-15	-8.205.098
207345_at	9.41e-15	2.52e-15	-820.347
210870_s_at	9.45e-15	2.53e-15	-8.202.874
212538_at	9.45e-15	2.53e-15	820.287
206612_at	9.45e-15	2.53e-15	-8.202.721
206771_at	9.49e-15	2.54e-15	8.202.137
204311_at	9.64e-15	2.58e-15	-8.199.959
203756_at	9.67e-15	2.59e-15	-8.199.539
204544_at	9.98e-15	2.68e-15	-8.195.168
202030_at	1.00e-14	2.69e-15	-8.194.448
209150_s_at	1.00e-14	2.69e-15	8.194.388
201872_s_at	1.01e-14	2.70e-15	8.194.047
221656_s_at	1.02e-14	2.73e-15	-8.192.341
201350_at	1.05e-14	2.81e-15	8.188.201
203913_s_at	1.05e-14	2.83e-15	-8.187.402
211062_s_at	1.07e-14	2.88e-15	-8.185.159
204109_s_at	1.07e-14	2.89e-15	8.184.699
211780_x_at	1.08e-14	2.89e-15	8.184.453
202121_s_at	1.08e-14	2.89e-15	8.184.409
202594_at	1.08e-14	2.90e-15	8.183.816
210637_at	1.10e-14	2.95e-15	-8.181.498

214658_at	1.10e-14	2.96e-15	8.181.364
212273_x_at	1.10e-14	2.96e-15	8.181.332
218462_at	1.11e-14	2.98e-15	8.180.412
221583_s_at	1.12e-14	3.00e-15	-8.179.155
220395_at	1.13e-14	3.03e-15	-8.178.154
201186_at	1.14e-14	3.06e-15	8.176.625
207315_at	1.15e-14	3.09e-15	-8.175.281
220396_at	1.15e-14	3.10e-15	-8.174.801
208196_x_at	1.16e-14	3.11e-15	-8.174.237
217378_x_at	1.17e-14	3.15e-15	-8.172.468
219210_s_at	1.17e-14	3.16e-15	8.172.234
216289_at	1.18e-14	3.17e-15	-8.171.775
205484_at	1.18e-14	3.17e-15	-8.171.564
219548_at	1.18e-14	3.18e-15	8.171.047
201080_at	1.19e-14	3.20e-15	8.170.511
213653_at	1.19e-14	3.21e-15	8.169.807
215582_x_at	1.21e-14	3.26e-15	-8.167.916
218280_x_at	1.21e-14	3.27e-15	8.167.444
210887_s_at	1.21e-14	3.27e-15	-8.167.318
215807_s_at	1.23e-14	3.31e-15	-8.165.675
211358_s_at	1.24e-14	3.34e-15	8.164.587
216872_at	1.28e-14	3.44e-15	-8.160.359
205055_at	1.28e-14	3.46e-15	8.159.572
205762_s_at	1.28e-14	3.46e-15	8.159.373
216626_at	1.30e-14	3.50e-15	-8.157.948
221377_s_at	1.30e-14	3.50e-15	-8.157.879
216483_s_at	1.30e-14	3.51e-15	8.157.543
215254_at	1.31e-14	3.53e-15	-8.156.794
202201_at	1.32e-14	3.56e-15	-8.155.613
212323_s_at	1.32e-14	3.56e-15	-8.155.386
219510_at	1.33e-14	3.58e-15	8.154.806
207363_at	1.34e-14	3.62e-15	-8.153.298
222132_s_at	1.35e-14	3.65e-15	8.152.088
220288_at	1.36e-14	3.67e-15	-8.151.499
203438_at	1.37e-14	3.71e-15	8.149.935
213500_at	1.37e-14	3.71e-15	8.149.834
205892_s_at	1.37e-14	3.71e-15	-8.149.668
204385_at	1.38e-14	3.72e-15	-8.149.318
220833_at	1.39e-14	3.76e-15	-8.147.993
206002_at	1.40e-14	3.78e-15	8.147.422
202985_s_at	1.42e-14	3.83e-15	814.541
211825_s_at	1.44e-14	3.89e-15	-8.143.104
206123_at	1.44e-14	3.90e-15	-8.143.065
214963_at	1.44e-14	3.90e-15	8.142.857

208723_at	1.46e-14	3.94e-15	8.141.407
211074_at	1.46e-14	3.94e-15	-8.141.318
214124_x_at	1.46e-14	3.95e-15	-8.141.165
211721_s_at	1.46e-14	3.96e-15	8.140.855
217672_x_at	1.47e-14	3.99e-15	8.139.714
220722_s_at	1.48e-14	4.00e-15	-8.139.517
220376_at	1.49e-14	4.03e-15	-8.138.417
220585_at	1.50e-14	4.07e-15	8.136.921
214799_at	1.51e-14	4.10e-15	-8.136.103
213977_s_at	1.51e-14	4.10e-15	8.135.869
211814_s_at	1.52e-14	4.11e-15	8.135.626
216176_at	1.52e-14	4.12e-15	-8.135.309
204418_x_at	1.52e-14	4.13e-15	-8.134.981
201365_at	1.53e-14	4.16e-15	-81.339
214202_at	1.54e-14	4.18e-15	8.133.145
203946_s_at	1.55e-14	4.20e-15	8.132.555
203126_at	1.56e-14	4.22e-15	8.131.902
201535_at	1.56e-14	4.22e-15	-8.131.833
214354_x_at	1.60e-14	4.33e-15	-8.128.295
213193_x_at	1.60e-14	4.35e-15	-8.127.728
212148_at	1.60e-14	4.35e-15	-8.127.678
221212_x_at	1.63e-14	4.42e-15	-8.125.383
212650_at	1.65e-14	4.49e-15	-812.323
201707_at	1.66e-14	4.50e-15	8.123.068
91682_at	1.67e-14	4.53e-15	-8.122.175
220631_at	1.67e-14	4.54e-15	8.121.698
214388_at	1.68e-14	4.57e-15	-8.120.706
215788_at	1.68e-14	4.57e-15	-8.120.691
219322_s_at	1.68e-14	4.58e-15	8.120.543
221550_at	1.70e-14	4.61e-15	8.119.525
205973_at	1.70e-14	4.63e-15	-811.904
213933_at	1.72e-14	4.67e-15	-8.117.784
205372_at	1.72e-14	4.68e-15	8.117.381
217100_s_at	1.75e-14	4.76e-15	8.115.033
203938_s_at	1.78e-14	4.83e-15	-8.113.043
219292_at	1.78e-14	4.85e-15	8.112.605
210245_at	1.79e-14	4.88e-15	-811.169
208030_s_at	1.80e-14	4.90e-15	8.111.161
219488_at	1.81e-14	4.92e-15	-811.052
216978_x_at	1.82e-14	4.95e-15	-8.109.721
219696_at	1.82e-14	4.95e-15	8.109.645
205486_at	1.82e-14	4.97e-15	-8.109.038
212921_at	1.84e-14	5.01e-15	8.108.077
205115_s_at	1.84e-14	5.02e-15	8.107.878

216032_s_at	1.87e-14	5.11e-15	810.538
204817_at	1.89e-14	5.16e-15	810.401
218440_at	1.89e-14	5.17e-15	-8.103.706
213121_at	1.90e-14	5.19e-15	-8.102.975
213313_at	1.91e-14	5.20e-15	8.102.878
205049_s_at	1.91e-14	5.22e-15	-8.102.325
211537_x_at	1.95e-14	5.32e-15	8.099.758
207325_x_at	1.97e-14	5.38e-15	8.098.103
201174_s_at	1.98e-14	5.41e-15	-8.097.238
204143_s_at	2.00e-14	5.46e-15	-8.095.952
204472_at	2.01e-14	5.48e-15	-8.095.416
221821_s_at	2.03e-14	5.53e-15	8.094.188
207855_s_at	2.03e-14	5.54e-15	8.093.887
215971_at	2.06e-14	5.63e-15	-8.091.816
211031_s_at	2.09e-14	5.71e-15	8.089.776
204824_at	2.10e-14	5.75e-15	-8.088.892
205003_at	2.11e-14	5.78e-15	8.088.157
201118_at	2.14e-14	5.85e-15	8.086.348
221215_s_at	2.15e-14	5.87e-15	-8.085.916
211935_at	2.17e-14	5.95e-15	-808.412
212453_at	2.24e-14	6.14e-15	8.079.666
217882_at	2.25e-14	6.14e-15	8.079.562
212894_at	2.25e-14	6.15e-15	807.947
201402_at	2.31e-14	6.32e-15	-8.075.521
222129_at	2.32e-14	6.34e-15	807.518
212471_at	2.33e-14	6.37e-15	8.074.419
201008_s_at	2.34e-14	6.40e-15	-8.073.835
220904_at	2.37e-14	6.48e-15	-8.072.104
205685_at	2.38e-14	6.51e-15	-8.071.464
214182_at	2.39e-14	6.55e-15	8.070.633
220761_s_at	2.39e-14	6.56e-15	8.070.381
211503_s_at	2.39e-14	6.56e-15	-8.070.328
34221_at	2.46e-14	6.75e-15	8.066.309
202260_s_at	2.47e-14	6.76e-15	806.618
207380_x_at	2.47e-14	6.76e-15	-8.066.167
217358_at	2.47e-14	6.77e-15	-8.066.044
201183_s_at	2.47e-14	6.77e-15	8.065.929
203431_s_at	2.48e-14	6.80e-15	-8.065.354
214241_at	2.48e-14	6.80e-15	-8.065.266
216047_x_at	2.53e-14	6.95e-15	-8.062.246
211349_at	2.62e-14	7.19e-15	-8.057.554
212730_at	2.72e-14	7.47e-15	8.052.145
221177_at	2.73e-14	7.51e-15	-8.051.481
208982_at	2.79e-14	7.66e-15	8.048.598

214339_s_at	2.80e-14	7.70e-15	-804.786
201805_at	2.81e-14	7.72e-15	8.047.601
213486_at	2.81e-14	7.73e-15	-8.047.451
201141_at	2.83e-14	7.78e-15	8.046.557
208067_x_at	2.87e-14	7.90e-15	-8.044.425
203672_x_at	2.89e-14	7.95e-15	-8.043.428
205062_x_at	2.91e-14	8.00e-15	-8.042.649
212216_at	2.91e-14	8.00e-15	8.042.591
213902_at	2.93e-14	8.05e-15	8.041.659
212899_at	2.94e-14	8.08e-15	8.041.195
220658_s_at	2.94e-14	8.09e-15	8.041.089
203728_at	2.94e-14	8.09e-15	8.041.084
207382_at	3.02e-14	8.30e-15	-803.735
206455_s_at	3.04e-14	8.36e-15	-8.036.402
202865_at	3.06e-14	8.44e-15	-8.035.115
213830_at	3.07e-14	8.44e-15	-8.035.067
202929_s_at	3.07e-14	8.45e-15	-8.034.935
219528_s_at	3.07e-14	8.45e-15	-8.034.813
212780_at	3.09e-14	8.51e-15	8.033.961
206073_at	3.11e-14	8.57e-15	-8.032.855
217532_x_at	3.13e-14	8.62e-15	-8.032.178
219263_at	3.15e-14	8.67e-15	-8.031.254
212862_at	3.15e-14	8.69e-15	8.031.025
210266_s_at	3.16e-14	8.71e-15	8.030.689
208099_x_at	3.21e-14	8.84e-15	-802.858
205696_s_at	3.21e-14	8.86e-15	-8.028.239
215719_x_at	3.23e-14	8.91e-15	-8.027.391
206737_at	3.23e-14	8.92e-15	-8.027.309
201488_x_at	3.27e-14	9.02e-15	8.025.672
213122_at	3.30e-14	9.12e-15	-8.024.213
214788_x_at	3.31e-14	9.14e-15	-802.392
214642_x_at	3.31e-14	9.14e-15	8.023.808
205752_s_at	3.32e-14	9.16e-15	-8.023.523
220459_at	3.33e-14	9.20e-15	8.022.889
212507_at	3.35e-14	9.26e-15	8.022.036
202759_s_at	3.35e-14	9.27e-15	-8.021.926
217403_s_at	3.47e-14	9.59e-15	8.017.183
202785_at	3.49e-14	9.63e-15	8.016.497
218680_x_at	3.49e-14	9.64e-15	8.016.416
216629_at	3.55e-14	9.82e-15	-8.013.847
206406_at	3.58e-14	9.89e-15	-8.012.798
44654_at	3.58e-14	9.89e-15	8.012.774
203437_at	3.58e-14	9.89e-15	8.012.755
200681_at	3.58e-14	9.89e-15	8.012.721

213248_at	3.58e-14	9.91e-15	8.012.468
215223_s_at	3.58e-14	9.92e-15	8.012.413
213144_at	3.61e-14	1.00e-14	8.011.257
209208_at	3.66e-14	1.01e-14	-8.009.545
206170_at	3.69e-14	1.02e-14	-8.008.355
212064_x_at	3.70e-14	1.02e-14	8.007.984
204276_at	3.72e-14	1.03e-14	-8.007.225
217808_s_at	3.73e-14	1.03e-14	8.006.685
213135_at	3.78e-14	1.05e-14	-8.004.624
203944_x_at	3.78e-14	1.05e-14	8.004.579
219351_at	3.79e-14	1.05e-14	8.004.241
216647_at	3.83e-14	1.06e-14	-8.002.749
212372_at	3.84e-14	1.06e-14	-8.002.376
203506_s_at	3.87e-14	1.07e-14	8.001.468
214073_at	3.88e-14	1.08e-14	8.001.017
210666_at	3.95e-14	1.10e-14	-799.828
213859_x_at	4.01e-14	1.11e-14	7.996.407
216229_x_at	4.09e-14	1.13e-14	-7.993.521
203997_at	4.10e-14	1.14e-14	-7.993.057
203190_at	4.10e-14	1.14e-14	7.993.007
204500_s_at	4.11e-14	1.14e-14	7.992.569
212978_at	4.14e-14	1.15e-14	7.991.537
204786_s_at	4.18e-14	1.16e-14	-799.038
204283_at	4.21e-14	1.17e-14	798.917
209593_s_at	4.23e-14	1.18e-14	798.844
222050_at	4.34e-14	1.21e-14	-7.984.833
212167_s_at	4.36e-14	1.21e-14	7.984.175
209294_x_at	4.37e-14	1.21e-14	-7.984.038
213215_at	4.43e-14	1.23e-14	-7.981.914
202085_at	4.46e-14	1.24e-14	-7.981.075
208948_s_at	4.46e-14	1.24e-14	7.980.865
200878_at	4.48e-14	1.24e-14	-7.980.362
205240_at	4.51e-14	1.25e-14	7.979.351
204078_at	4.58e-14	1.27e-14	7.977.284
211947_s_at	4.59e-14	1.28e-14	7.976.727
60474_at	4.61e-14	1.28e-14	797.615
202638_s_at	4.63e-14	1.29e-14	7.975.608
219912_s_at	4.64e-14	1.29e-14	-7.975.197
207625_s_at	4.65e-14	1.30e-14	7.974.774
203780_at	4.66e-14	1.30e-14	7.974.426
220138_at	4.68e-14	1.30e-14	-797.392
206325_at	4.69e-14	1.31e-14	-7.973.568
33197_at	4.74e-14	1.32e-14	-7.972.103
201901_s_at	4.79e-14	1.33e-14	797.072

213798_s_at	4.81e-14	1.34e-14	7.970.097
208439_s_at	4.84e-14	1.35e-14	-7.969.079
219208_at	4.86e-14	1.36e-14	7.968.406
204038_s_at	4.87e-14	1.36e-14	-7.968.164
208451_s_at	4.90e-14	1.37e-14	-7.967.255
214162_at	4.90e-14	1.37e-14	-796.711
219897_at	4.93e-14	1.37e-14	-7.966.371
77508_r_at	4.94e-14	1.38e-14	-7.965.961
201624_at	4.96e-14	1.38e-14	7.965.378
212764_at	4.97e-14	1.39e-14	7.965.038
220311_at	4.98e-14	1.39e-14	7.964.785
215614_at	4.98e-14	1.39e-14	-7.964.693
221842_s_at	5.11e-14	1.43e-14	7.961.126
219393_s_at	5.15e-14	1.44e-14	-7.959.946
208101_s_at	5.17e-14	1.44e-14	7.959.566
210031_at	5.17e-14	1.44e-14	-7.959.498
202277_at	5.21e-14	1.46e-14	7.958.255
215000_s_at	5.27e-14	1.47e-14	795.676
221823_at	5.30e-14	1.48e-14	7.955.979
219020_at	5.31e-14	1.48e-14	7.955.476
211960_s_at	5.35e-14	1.50e-14	7.954.515
218948_at	5.36e-14	1.50e-14	7.954.119
214581_x_at	5.41e-14	1.51e-14	7.952.978
215182_x_at	5.43e-14	1.52e-14	-7.952.371
219373_at	5.44e-14	1.52e-14	7.951.969
200749_at	5.44e-14	1.52e-14	7.951.908
220816_at	5.48e-14	1.53e-14	-7.950.894
218154_at	5.54e-14	1.55e-14	7.949.311
211406_at	5.59e-14	1.56e-14	794.821
210060_at	5.60e-14	1.57e-14	-7.947.707
218165_at	5.66e-14	1.59e-14	794.617
205333_s_at	5.68e-14	1.59e-14	7.945.807
210101_x_at	5.68e-14	1.59e-14	7.945.632
218652_s_at	5.69e-14	1.59e-14	7.945.374
215350_at	5.72e-14	1.60e-14	-7.944.726
203177_x_at	5.81e-14	1.63e-14	7.942.406
219335_at	5.82e-14	1.63e-14	7.942.284
205969_at	5.84e-14	1.64e-14	-7.941.624
201502_s_at	5.84e-14	1.64e-14	-7.941.559
212279_at	5.86e-14	1.64e-14	7.941.255
210985_s_at	5.87e-14	1.65e-14	-7.940.864
213227_at	5.95e-14	1.67e-14	-7.939.052
202782_s_at	5.96e-14	1.67e-14	-7.938.757
205164_at	5.96e-14	1.67e-14	-7.938.729

219243_at	5.96e-14	1.67e-14	-7.938.588
207922_s_at	5.97e-14	1.67e-14	7.938.491
204262_s_at	6.01e-14	1.69e-14	7.937.464
219271_at	6.05e-14	1.70e-14	-7.936.426
219798_s_at	6.10e-14	1.71e-14	7.935.313
206216_at	6.12e-14	1.72e-14	-7.934.845
205296_at	6.16e-14	1.73e-14	7.933.885
218683_at	6.23e-14	1.75e-14	7.932.182
204363_at	6.24e-14	1.75e-14	-793.191
222326_at	6.30e-14	1.77e-14	-7.930.708
201125_s_at	6.36e-14	1.79e-14	7.929.141
208826_x_at	6.37e-14	1.79e-14	7.929.036
221533_at	6.37e-14	1.79e-14	-7.928.923
221502_at	6.39e-14	1.80e-14	7.928.493
203481_at	6.51e-14	1.83e-14	7.925.787
215208_x_at	6.52e-14	1.83e-14	-7.925.662
218711_s_at	6.57e-14	1.85e-14	-792.459
201011_at	6.60e-14	1.86e-14	7.923.931
212340_at	6.63e-14	1.87e-14	7.923.131
40273_at	6.68e-14	1.88e-14	-7.922.129
210467_x_at	6.69e-14	1.88e-14	792.178
204239_s_at	6.77e-14	1.91e-14	-7.920.172
203113_s_at	6.85e-14	1.93e-14	7.918.361
210289_at	6.90e-14	1.94e-14	-7.917.407
221692_s_at	6.90e-14	1.94e-14	-7.917.348
205759_s_at	6.98e-14	1.97e-14	-7.915.718
204838_s_at	7.02e-14	1.98e-14	7.914.923
205161_s_at	7.05e-14	1.99e-14	-7.914.203
210038_at	7.11e-14	2.00e-14	-7.913.064
207791_s_at	7.12e-14	2.01e-14	791.284
40020_at	7.16e-14	2.02e-14	791.197
208224_at	7.20e-14	2.03e-14	-791.109
217187_at	7.23e-14	2.04e-14	-7.910.644
212492_s_at	7.27e-14	2.05e-14	-7.909.651
206621_s_at	7.29e-14	2.06e-14	7.909.311
218604_at	7.35e-14	2.07e-14	7.908.164
211005_at	7.39e-14	2.08e-14	-7.907.391
206275_s_at	7.51e-14	2.12e-14	-7.905.133
222251_s_at	7.51e-14	2.12e-14	7.904.943
206802_at	7.52e-14	2.12e-14	-7.904.829
214126_at	7.57e-14	2.14e-14	7.903.936
218237_s_at	7.59e-14	2.14e-14	7.903.511
214877_at	7.68e-14	2.17e-14	7.901.787
201419_at	7.91e-14	2.23e-14	789.759

204725_s_at	7.95e-14	2.25e-14	7.896.711
204025_s_at	8.04e-14	2.27e-14	7.895.193
203851_at	8.04e-14	2.27e-14	-7.895.124
205430_at	8.17e-14	2.31e-14	-7.892.764
202970_at	8.18e-14	2.31e-14	7.892.724
220530_at	8.18e-14	2.31e-14	-7.892.623
202500_at	8.27e-14	2.34e-14	7.891.085
203879_at	8.29e-14	2.34e-14	-7.890.705
217773_s_at	8.34e-14	2.36e-14	7.889.824
216867_s_at	8.43e-14	2.39e-14	7.888.253
218722_s_at	8.49e-14	2.40e-14	7.887.189
203560_at	8.49e-14	2.40e-14	7.887.154
205504_at	8.55e-14	2.42e-14	-7.886.111
202114_at	8.57e-14	2.43e-14	7.885.756
205277_at	8.66e-14	2.45e-14	-7.884.268
209510_at	8.67e-14	2.46e-14	7.884.106
203604_at	8.73e-14	2.47e-14	7.883.087
200072_s_at	8.75e-14	2.48e-14	7.882.714
221980_at	8.78e-14	2.49e-14	-78.822
220973_s_at	8.83e-14	2.50e-14	7.881.409
214007_s_at	8.86e-14	2.51e-14	7.880.993
217466_x_at	8.86e-14	2.51e-14	7.880.946
211437_at	9.00e-14	2.55e-14	-787.867
202042_at	9.05e-14	2.57e-14	7.877.799
221909_at	9.06e-14	2.57e-14	7.877.666
209729_at	9.06e-14	2.57e-14	7.877.662
203017_s_at	9.11e-14	2.58e-14	7.876.844
208846_s_at	9.14e-14	2.59e-14	7.876.343
56748_at	9.16e-14	2.60e-14	-7.875.987
201984_s_at	9.19e-14	2.61e-14	-7.875.582
214394_x_at	9.21e-14	2.62e-14	787.513
200973_s_at	9.45e-14	2.68e-14	7.871.551
52741_at	9.51e-14	2.70e-14	-7.870.556
219994_at	9.59e-14	2.72e-14	-7.869.419
206459_s_at	9.60e-14	2.73e-14	-7.869.252
212931_at	9.64e-14	2.74e-14	7.868.637
211677_x_at	9.80e-14	2.79e-14	-7.866.191
205797_s_at	9.85e-14	2.80e-14	-7.865.491
216576_x_at	9.88e-14	2.81e-14	-7.864.983
212886_at	9.92e-14	2.82e-14	-7.864.392
214934_at	9.93e-14	2.82e-14	7.864.228
200077_s_at	9.94e-14	2.83e-14	7.864.035
222285_at	9.98e-14	2.84e-14	-7.863.477
204567_s_at	9.99e-14	2.84e-14	7.863.284

205721_at	1.04e-13	2.97e-14	-7.857.263
204579_at	1.07e-13	3.03e-14	7.854.011
203495_at	1.07e-13	3.05e-14	7.853.059
201990_s_at	1.08e-13	3.09e-14	-7.851.473
209725_at	1.09e-13	3.11e-14	7.850.238
200742_s_at	1.10e-13	3.13e-14	784.957
208015_at	1.10e-13	3.13e-14	-7.849.332
211797_s_at	1.10e-13	3.14e-14	7.848.928
218995_s_at	1.13e-13	3.21e-14	-784.572
213315_x_at	1.15e-13	3.27e-14	7.843.385
215897_at	1.15e-13	3.28e-14	-7.842.991
221010_s_at	1.16e-13	3.32e-14	-7.841.149
217137_x_at	1.16e-13	3.32e-14	-7.841.116
218846_at	1.19e-13	3.38e-14	7.838.563
204124_at	1.19e-13	3.40e-14	-7.837.923
214425_at	1.19e-13	3.41e-14	-7.837.419
213314_at	1.20e-13	3.42e-14	783.695
216266_s_at	1.20e-13	3.42e-14	7.836.822
212097_at	1.20e-13	3.42e-14	7.836.739
216720_at	1.20e-13	3.43e-14	-7.836.479
213803_at	1.21e-13	3.45e-14	7.835.685
219058_x_at	1.21e-13	3.46e-14	-7.835.193
218607_s_at	1.22e-13	3.49e-14	7.833.898
215618_at	1.23e-13	3.51e-14	-7.833.194
207892_at	1.24e-13	3.53e-14	-7.832.432
33768_at	1.24e-13	3.54e-14	-7.832.113
218619_s_at	1.27e-13	3.62e-14	7.828.907
220589_s_at	1.28e-13	3.66e-14	-7.827.005
212125_at	1.30e-13	3.70e-14	7.825.555
210750_s_at	1.30e-13	3.73e-14	-7.824.544
209322_s_at	1.32e-13	3.77e-14	-7.823.112
220382_s_at	1.32e-13	3.77e-14	-7.823.052
213079_at	1.34e-13	3.82e-14	7.820.988
211760_s_at	1.35e-13	3.87e-14	7.819.387
212013_at	1.35e-13	3.87e-14	-781.929
213543_at	1.37e-13	3.91e-14	-7.817.653
214678_x_at	1.39e-13	3.98e-14	7.815.334
204551_s_at	1.40e-13	3.99e-14	-7.814.698
213022_s_at	1.40e-13	4.00e-14	7.814.334
217945_at	1.41e-13	4.04e-14	7.812.873
209371_s_at	1.43e-13	4.08e-14	-7.811.555
209476_at	1.43e-13	4.10e-14	7.810.979
203239_s_at	1.44e-13	4.13e-14	7.809.895
220034_at	1.47e-13	4.21e-14	-7.806.999

210510_s_at	1.50e-13	4.29e-14	7.804.512
220888_s_at	1.50e-13	4.29e-14	-7.804.489
204716_at	1.52e-13	4.35e-14	7.802.426
201117_s_at	1.54e-13	4.41e-14	7.800.635
205232_s_at	1.54e-13	4.42e-14	-7.800.071
213001_at	1.57e-13	4.50e-14	-77.977
214733_s_at	1.58e-13	4.52e-14	7.796.974
221187_s_at	1.58e-13	4.53e-14	-7.796.504
209689_at	1.58e-13	4.54e-14	7.796.165
204086_at	1.59e-13	4.56e-14	7.795.709
31874_at	1.63e-13	4.68e-14	7.791.988
219838_at	1.64e-13	4.71e-14	7.791.032
209277_at	1.64e-13	4.72e-14	-7.790.886
206229_x_at	1.65e-13	4.74e-14	-7.790.116
211803_at	1.69e-13	4.84e-14	-7.787.056
202304_at	1.70e-13	4.87e-14	-7.786.211
208218_s_at	1.70e-13	4.87e-14	-7.786.171
217221_x_at	1.70e-13	4.88e-14	7.785.897
221028_s_at	1.71e-13	4.90e-14	-7.785.298
205936_s_at	1.71e-13	4.91e-14	-7.785.151
215673_at	1.72e-13	4.93e-14	-7.784.492
218614_at	1.73e-13	4.96e-14	-7.783.579
217251_x_at	1.75e-13	5.04e-14	-7.781.299
210849_s_at	1.77e-13	5.09e-14	7.779.881
211833_s_at	1.78e-13	5.11e-14	7.779.282
210398_x_at	1.78e-13	5.13e-14	-7.778.901
217794_at	1.79e-13	5.15e-14	777.835
213880_at	1.80e-13	5.18e-14	7.777.253
204430_s_at	1.81e-13	5.21e-14	777.652
201751_at	1.81e-13	5.22e-14	7.776.307
217658_at	1.81e-13	5.22e-14	-7.776.223
214769_at	1.81e-13	5.22e-14	7.776.201
202155_s_at	1.82e-13	5.23e-14	7.776.035
207684_at	1.85e-13	5.32e-14	-7.773.546
206724_at	1.85e-13	5.34e-14	7.772.997
218920_at	1.90e-13	5.48e-14	-7.769.184
208189_s_at	1.92e-13	5.54e-14	-7.767.668
204785_x_at	1.98e-13	5.71e-14	7.763.436
213054_at	1.98e-13	5.71e-14	7.763.421
202563_at	1.98e-13	5.71e-14	-7.763.347
220041_at	1.99e-13	5.74e-14	7.762.609
203657_s_at	2.00e-13	5.78e-14	-7.761.733
206828_at	2.01e-13	5.80e-14	-7.761.051
203221_at	2.01e-13	5.81e-14	7.760.989

210324_at	2.01e-13	5.81e-14	-7.760.887
202582_s_at	2.02e-13	5.83e-14	7.760.437
218955_at	2.02e-13	5.83e-14	7.760.404
202179_at	2.04e-13	5.90e-14	7.758.702
202893_at	2.05e-13	5.93e-14	7.758.001
201073_s_at	2.07e-13	5.97e-14	7.756.864
219389_at	2.08e-13	6.00e-14	7.756.172
220730_at	2.09e-13	6.05e-14	-775.508
203623_at	2.10e-13	6.06e-14	7.754.852
213264_at	2.13e-13	6.15e-14	-7.752.598
203548_s_at	2.14e-13	6.18e-14	7.752.031
217777_s_at	2.14e-13	6.19e-14	7.751.807
204739_at	2.15e-13	6.20e-14	-7.751.434
213589_s_at	2.16e-13	6.25e-14	7.750.434
216131_at	2.19e-13	6.32e-14	-7.748.793
213670_x_at	2.23e-13	6.44e-14	7.746.031
218190_s_at	2.23e-13	6.44e-14	7.745.972
208561_at	2.24e-13	6.47e-14	-7.745.486
220046_s_at	2.24e-13	6.49e-14	-7.744.894
212953_x_at	2.25e-13	6.50e-14	7.744.733
200959_at	2.26e-13	6.54e-14	7.743.802
206831_s_at	2.26e-13	6.55e-14	-774.366
205178_s_at	2.27e-13	6.57e-14	-7.743.154
202108_at	2.30e-13	6.65e-14	-7.741.523
214792_x_at	2.30e-13	6.65e-14	-774.132
203189_s_at	2.30e-13	6.66e-14	7.741.113
219257_s_at	2.31e-13	6.70e-14	7.740.346
206630_at	2.32e-13	6.71e-14	-7.740.082
211801_x_at	2.33e-13	6.75e-14	7.739.371
218442_at	2.34e-13	6.79e-14	7.738.521
210228_at	2.35e-13	6.81e-14	-7.738.084
217670_at	2.36e-13	6.85e-14	-7.737.125
203806_s_at	2.38e-13	6.90e-14	7.736.137
200011_s_at	2.38e-13	6.90e-14	7.736.127
209290_s_at	2.38e-13	6.90e-14	-7.736.004
221335_x_at	2.41e-13	6.98e-14	7.734.376
216209_at	2.43e-13	7.04e-14	-7.733.152
222148_s_at	2.44e-13	7.08e-14	7.732.309
203720_s_at	2.45e-13	7.10e-14	7.732.044
213715_s_at	2.45e-13	7.10e-14	-7.731.882
220371_s_at	2.46e-13	7.14e-14	77.312
215921_at	2.50e-13	7.26e-14	-7.728.659
209478_at	2.51e-13	7.30e-14	7.727.936
212631_at	2.55e-13	7.40e-14	7.726.043

212835_at	2.55e-13	7.41e-14	7.725.846
207093_s_at	2.56e-13	7.43e-14	-7.725.378
211900_x_at	2.60e-13	7.56e-14	-7.722.955
222182_s_at	2.61e-13	7.57e-14	7.722.631
37004_at	2.61e-13	7.59e-14	-7.722.411
215507_x_at	2.62e-13	7.61e-14	-7.722.038
220893_at	2.62e-13	7.63e-14	-7.721.643
201561_s_at	2.63e-13	7.65e-14	7.721.257
201032_at	2.63e-13	7.65e-14	-7.721.108
211186_s_at	2.63e-13	7.66e-14	-772.093
211071_s_at	2.64e-13	7.69e-14	7.720.423
212469_at	2.66e-13	7.75e-14	7.719.398
217266_at	2.69e-13	7.83e-14	7.717.766
56256_at	2.69e-13	7.84e-14	-7.717.708
206509_at	2.70e-13	7.86e-14	-7.717.356
209798_at	2.71e-13	7.88e-14	7.716.915
208608_s_at	2.72e-13	7.91e-14	7.716.322
219852_s_at	2.76e-13	8.03e-14	-7.714.164
211334_at	2.77e-13	8.08e-14	-7.713.279
202069_s_at	2.79e-13	8.12e-14	7.712.605
214428_x_at	2.79e-13	8.12e-14	-7.712.524
207655_s_at	2.79e-13	8.13e-14	-7.712.328
213552_at	2.81e-13	8.19e-14	7.711.325
209784_s_at	2.82e-13	8.21e-14	7.711.001
207008_at	2.85e-13	8.32e-14	-7.709.058
220074_at	2.86e-13	8.34e-14	-7.708.731
210504_at	2.92e-13	8.50e-14	-7.705.923
221913_at	2.92e-13	8.50e-14	-7.705.894
202468_s_at	2.94e-13	8.57e-14	7.704.741
213085_s_at	2.98e-13	8.70e-14	7.702.679
211997_x_at	3.02e-13	8.81e-14	7.700.776
217167_x_at	3.04e-13	8.87e-14	-7.699.757
202799_at	3.04e-13	8.88e-14	-7.699.573
211871_x_at	3.05e-13	8.92e-14	7.699.066
207983_s_at	3.06e-13	8.94e-14	7.698.587
39318_at	3.07e-13	8.95e-14	-7.698.443
221470_s_at	3.07e-13	8.95e-14	-769.844
222073_at	3.07e-13	8.96e-14	-7.698.266
202049_s_at	3.08e-13	8.99e-14	7.697.851
222312_s_at	3.09e-13	9.04e-14	-7.697.114
219681_s_at	3.09e-13	9.04e-14	7.697.042
205643_s_at	3.10e-13	9.08e-14	-7.696.476
203667_at	3.12e-13	9.12e-14	7.695.849
201597_at	3.13e-13	9.14e-14	7.695.436

220431_at	3.13e-13	9.14e-14	-7.695.434
215493_x_at	3.13e-13	9.16e-14	7.695.103
217045_x_at	3.17e-13	9.27e-14	-7.693.489
208645_s_at	3.19e-13	9.32e-14	7.692.628
212736_at	3.19e-13	9.32e-14	-7.692.559
219842_at	3.24e-13	9.48e-14	7.690.099
208453_s_at	3.27e-13	9.57e-14	7.688.855
208523_x_at	3.29e-13	9.63e-14	7.687.894
206506_s_at	3.29e-13	9.65e-14	768.766
202283_at	3.33e-13	9.74e-14	-7.686.196
212956_at	3.34e-13	9.77e-14	-7.685.771
205183_at	3.34e-13	9.78e-14	-7.685.683
211417_x_at	3.35e-13	9.81e-14	-7.685.276
208264_s_at	3.37e-13	9.88e-14	7.684.208
204389_at	3.38e-13	9.90e-14	-7.683.832
213438_at	3.38e-13	9.90e-14	-7.683.817
204890_s_at	3.39e-13	9.95e-14	-7.683.138
212594_at	3.40e-13	9.97e-14	7.682.825
220245_at	3.40e-13	9.98e-14	-7.682.781
204435_at	3.41e-13	9.99e-14	7.682.607
207365_x_at	3.41e-13	1.00e-13	-7.682.265
219022_at	3.43e-13	1.01e-13	7.681.622
218678_at	3.43e-13	1.01e-13	7.681.445
213669_at	3.46e-13	1.02e-13	-7.680.241
210987_x_at	3.47e-13	1.02e-13	7.679.817
219826_at	3.51e-13	1.03e-13	767.815
210267_at	3.58e-13	1.05e-13	-7.675.153
206492_at	3.60e-13	1.06e-13	767.443
203973_s_at	3.62e-13	1.06e-13	-7.673.468
204314_s_at	3.63e-13	1.06e-13	7.673.288
221902_at	3.65e-13	1.07e-13	-7.672.182
219170_at	3.68e-13	1.08e-13	-767.121
218893_at	3.72e-13	1.09e-13	-7.669.337
219242_at	3.73e-13	1.10e-13	7.669.154
217696_at	3.78e-13	1.11e-13	-7.667.057
208161_s_at	3.78e-13	1.11e-13	-7.666.922
221250_s_at	3.79e-13	1.11e-13	7.666.627
213150_at	3.81e-13	1.12e-13	7.666.065
218457_s_at	3.81e-13	1.12e-13	7.665.911
202886_s_at	3.82e-13	1.12e-13	-7.665.617
221065_s_at	3.83e-13	1.13e-13	-7.665.267
204302_s_at	3.83e-13	1.13e-13	-7.664.925
211747_s_at	3.85e-13	1.13e-13	7.664.458
216188_at	3.88e-13	1.14e-13	-7.663.043

220967_s_at	3.90e-13	1.15e-13	766.237
205907_s_at	3.91e-13	1.15e-13	-7.662.072
219312_s_at	3.95e-13	1.16e-13	7.660.442
215741_x_at	3.97e-13	1.17e-13	-7.659.865
219006_at	3.97e-13	1.17e-13	7.659.782
220344_at	3.98e-13	1.17e-13	-7.659.478
207081_s_at	4.01e-13	1.18e-13	7.658.187
216046_at	4.02e-13	1.18e-13	-7.657.904
216038_x_at	4.08e-13	1.20e-13	7.655.552
208559_at	4.09e-13	1.21e-13	-7.655.301
41329_at	4.09e-13	1.21e-13	7.655.274
214290_s_at	4.11e-13	1.21e-13	7.654.326
218032_at	4.12e-13	1.21e-13	7.654.206
219870_at	4.13e-13	1.22e-13	-7.653.815
215138_s_at	4.15e-13	1.22e-13	-765.304
200753_x_at	4.17e-13	1.23e-13	7.652.223
205529_s_at	4.19e-13	1.24e-13	-7.651.607
201978_s_at	4.26e-13	1.26e-13	-7.649.056
220416_at	4.26e-13	1.26e-13	-764.899
212435_at	4.30e-13	1.27e-13	7.647.909
207320_x_at	4.31e-13	1.27e-13	7.647.314
206957_at	4.34e-13	1.28e-13	-7.646.474
203530_s_at	4.38e-13	1.29e-13	764.496
220000_at	4.40e-13	1.30e-13	-7.644.427
211886_s_at	4.42e-13	1.31e-13	-7.643.484
204408_at	4.44e-13	1.31e-13	7.642.795
205541_s_at	4.45e-13	1.32e-13	-7.642.591
213521_at	4.50e-13	1.33e-13	-7.640.965
207578_s_at	4.52e-13	1.34e-13	-7.640.162
206261_at	4.53e-13	1.34e-13	7.639.785
213677_s_at	4.54e-13	1.34e-13	7.639.683
202890_at	4.56e-13	1.35e-13	-7.639
219111_s_at	4.59e-13	1.36e-13	763.798
201465_s_at	4.64e-13	1.37e-13	-7.636.226
210379_s_at	4.67e-13	1.38e-13	7.635.547
213645_at	4.67e-13	1.38e-13	-7.635.305
217140_s_at	4.69e-13	1.39e-13	7.634.693
220035_at	4.84e-13	1.43e-13	-7.630.143
217335_at	4.87e-13	1.44e-13	-7.629.246
214271_x_at	4.87e-13	1.44e-13	7.629.092
219416_at	4.88e-13	1.45e-13	7.628.892
32811_at	4.88e-13	1.45e-13	-7.628.779
221601_s_at	4.88e-13	1.45e-13	-7.628.735
216826_at	4.90e-13	1.45e-13	-7.628.127

213039_at	4.91e-13	1.46e-13	7.627.723
213704_at	4.92e-13	1.46e-13	7.627.642
221290_s_at	4.93e-13	1.46e-13	-7.627.306
213021_at	4.93e-13	1.46e-13	7.627.239
203275_at	5.03e-13	1.49e-13	-7.624.311
218810_at	5.13e-13	1.52e-13	-7.621.412
207033_at	5.16e-13	1.53e-13	-7.620.485
215844_at	5.17e-13	1.53e-13	-7.620.298
205031_at	5.22e-13	1.55e-13	-7.618.852
219502_at	5.23e-13	1.55e-13	7.618.589
219496_at	5.26e-13	1.56e-13	-7.617.555
216932_at	5.28e-13	1.57e-13	-761.711
210871_x_at	5.28e-13	1.57e-13	7.617.014
209092_s_at	5.32e-13	1.58e-13	-7.615.811
217018_at	5.37e-13	1.60e-13	-7.614.425
202719_s_at	5.45e-13	1.62e-13	7.612.382
220065_at	5.49e-13	1.63e-13	-7.611.336
217386_at	5.51e-13	1.64e-13	-7.610.722
205801_s_at	5.52e-13	1.64e-13	7.610.351
208899_x_at	5.53e-13	1.64e-13	7.610.153
206628_at	5.54e-13	1.65e-13	-7.609.936
201928_at	5.60e-13	1.66e-13	7.608.239
202695_s_at	5.68e-13	1.69e-13	7.606.075
216112_at	5.73e-13	1.70e-13	-7.604.874
217480_x_at	5.77e-13	1.72e-13	-7.603.784
220942_x_at	5.82e-13	1.73e-13	-7.602.475
205924_at	5.87e-13	1.75e-13	7.601.199
206679_at	5.89e-13	1.75e-13	-7.600.832
208781_x_at	6.04e-13	1.80e-13	7.596.912
206231_at	6.06e-13	1.80e-13	-7.596.523
205248_at	6.11e-13	1.82e-13	7.595.267
203603_s_at	6.12e-13	1.82e-13	-7.594.974
205276_s_at	6.13e-13	1.83e-13	-759.481
217530_at	6.14e-13	1.83e-13	-759.438
214291_at	6.19e-13	1.85e-13	7.593.208
216250_s_at	6.25e-13	1.86e-13	-7.591.774
203515_s_at	6.32e-13	1.88e-13	7.590.306
209757_s_at	6.32e-13	1.88e-13	7.590.119
37433_at	6.33e-13	1.89e-13	7.590.022
60528_at	6.34e-13	1.89e-13	-7.589.751
212536_at	6.38e-13	1.90e-13	7.588.762
215504_x_at	6.39e-13	1.91e-13	-758.845
212217_at	6.55e-13	1.95e-13	7.584.906
215226_at	6.56e-13	1.96e-13	-7.584.707

212184_s_at	6.65e-13	1.99e-13	-7.582.481
202072_at	6.67e-13	1.99e-13	75.821
206711_at	6.77e-13	2.02e-13	-7.579.967
221987_s_at	6.80e-13	2.03e-13	7.579.299
220605_s_at	6.86e-13	2.05e-13	-7.578.033
214110_s_at	6.87e-13	2.05e-13	7.577.621
213108_at	6.90e-13	2.06e-13	-7.577.041
212683_at	6.91e-13	2.07e-13	757.671
206214_at	7.02e-13	2.10e-13	7.574.533
220807_at	7.05e-13	2.10e-13	-7.573.927
209560_s_at	7.11e-13	2.12e-13	7.572.637
219812_at	7.13e-13	2.13e-13	-7.572.053
219707_at	7.14e-13	2.14e-13	-7.571.833
211693_at	7.16e-13	2.14e-13	-7.571.562
202227_s_at	7.17e-13	2.14e-13	7.571.279
220172_at	7.27e-13	2.17e-13	7.569.166
220497_at	7.31e-13	2.19e-13	-756.844
209641_s_at	7.32e-13	2.19e-13	-7.568.175
204024_at	7.39e-13	2.21e-13	7.566.744
203157_s_at	7.48e-13	2.24e-13	7.565.014
201776_s_at	7.49e-13	2.24e-13	7.564.734
220844_at	7.56e-13	2.26e-13	-7.563.342
203688_at	7.57e-13	2.27e-13	-7.563.111
215840_at	7.60e-13	2.28e-13	-7.562.489
218214_at	7.63e-13	2.28e-13	7.561.917
87100_at	7.68e-13	2.30e-13	-7.560.924
213229_at	7.71e-13	2.31e-13	-7.560.355
211755_s_at	7.72e-13	2.31e-13	7.560.028
203852_s_at	7.82e-13	2.34e-13	7.558.259
211001_at	7.87e-13	2.36e-13	-7.557.162
217274_x_at	7.92e-13	2.37e-13	-7.556.354
211786_at	7.92e-13	2.37e-13	-7.556.299
202529_at	7.97e-13	2.39e-13	7.555.281
207576_x_at	7.98e-13	2.39e-13	-7.555.056
214151_s_at	8.06e-13	2.42e-13	-7.553.628
207911_s_at	8.07e-13	2.42e-13	-7.553.425
204703_at	8.17e-13	2.45e-13	-755.162
220577_at	8.21e-13	2.46e-13	-7.550.771
202109_at	8.25e-13	2.47e-13	755.016
217303_s_at	8.26e-13	2.48e-13	-7.549.991
217405_x_at	8.31e-13	2.49e-13	-7.549.055
203805_s_at	8.32e-13	2.50e-13	7.548.774
211840_s_at	8.36e-13	2.51e-13	-7.548.049
217622_at	8.48e-13	2.55e-13	-754.597

218654_s_at	8.52e-13	2.56e-13	7.545.232
206374_at	8.54e-13	2.57e-13	7.544.901
218386_x_at	8.65e-13	2.60e-13	7.542.997
206115_at	8.66e-13	2.60e-13	-7.542.841
202159_at	8.69e-13	2.61e-13	7.542.268
217963_s_at	8.71e-13	2.62e-13	754.183
215262_at	8.85e-13	2.66e-13	-7.539.591
222191_s_at	8.89e-13	2.67e-13	7.538.881
217070_at	9.01e-13	2.71e-13	-7.536.796
217080_s_at	9.03e-13	2.72e-13	-7.536.459
204619_s_at	9.06e-13	2.73e-13	753.593
221693_s_at	9.07e-13	2.73e-13	7.535.801
203337_x_at	9.11e-13	2.74e-13	7.535.165
220122_at	9.14e-13	2.75e-13	7.534.688
217395_at	9.14e-13	2.75e-13	-7.534.628
207889_at	9.15e-13	2.76e-13	-7.534.366
204395_s_at	9.16e-13	2.76e-13	-7.534.268
210629_x_at	9.24e-13	2.78e-13	-7.533.023
201413_at	9.24e-13	2.78e-13	-7.532.898
203847_s_at	9.29e-13	2.80e-13	753.208
215686_x_at	9.32e-13	2.81e-13	-7.531.638
221364_at	9.44e-13	2.85e-13	-7.529.671
222165_x_at	9.45e-13	2.85e-13	752.948
205606_at	9.47e-13	2.85e-13	-7.529.195
202554_s_at	9.47e-13	2.86e-13	7.529.151
213460_x_at	9.50e-13	2.86e-13	752.876
219612_s_at	9.57e-13	2.89e-13	-7.527.601
210555_s_at	9.58e-13	2.89e-13	-7.527.346
208896_at	9.60e-13	2.90e-13	7.527.059
215779_s_at	9.65e-13	2.91e-13	7.526.332
209638_x_at	9.71e-13	2.93e-13	-7.525.402
221328_at	9.76e-13	2.94e-13	-7.524.645
221433_at	9.87e-13	2.98e-13	7.522.976
206918_s_at	9.88e-13	2.98e-13	7.522.794
201437_s_at	1.00e-12	3.02e-13	7.520.712
213492_at	1.00e-12	3.03e-13	7.520.304
212694_s_at	1.01e-12	3.04e-13	-7.519.882
206580_s_at	1.01e-12	3.04e-13	-7.519.781
222220_s_at	1.01e-12	3.05e-13	-7.519.264
215284_at	1.01e-12	3.06e-13	-7.519.132
208527_x_at	1.02e-12	3.08e-13	7.518.017
221973_at	1.03e-12	3.10e-13	7.516.943
220922_s_at	1.03e-12	3.11e-13	-7.516.633
207090_x_at	1.03e-12	3.11e-13	7.516.449

209527_at	1.03e-12	3.11e-13	7.516.361
218866_s_at	1.03e-12	3.11e-13	751.633
205988_at	1.03e-12	3.12e-13	-7.516.039
212630_at	1.03e-12	3.13e-13	7.515.596
212966_at	1.04e-12	3.14e-13	7.515.274
220960_x_at	1.05e-12	3.17e-13	7.513.886
211212_s_at	1.06e-12	3.21e-13	7.511.964
209736_at	1.06e-12	3.21e-13	7.511.829
37860_at	1.06e-12	3.21e-13	7.511.714
214709_s_at	1.07e-12	3.22e-13	7.511.215
38447_at	1.07e-12	3.23e-13	-7.510.805
222306_at	1.07e-12	3.24e-13	-7.510.678
219598_s_at	1.07e-12	3.24e-13	751.049
61297_at	1.07e-12	3.25e-13	-7.510.106
202122_s_at	1.08e-12	3.28e-13	7.508.812
209467_s_at	1.09e-12	3.30e-13	-7.507.773
218596_at	1.09e-12	3.30e-13	75.076
213334_x_at	1.09e-12	3.32e-13	7.507.132
208741_at	1.10e-12	3.34e-13	-7.505.826
220956_s_at	1.11e-12	3.35e-13	7.505.569
207937_x_at	1.11e-12	3.35e-13	-750.555
203127_s_at	1.11e-12	3.35e-13	7.505.497
203935_at	1.12e-12	3.40e-13	7.503.495
207695_s_at	1.12e-12	3.40e-13	7.503.309
205076_s_at	1.14e-12	3.44e-13	750.147
221148_at	1.15e-12	3.48e-13	-7.499.873
217819_at	1.15e-12	3.50e-13	7.499.058
214194_at	1.16e-12	3.51e-13	7.498.512
222337_at	1.16e-12	3.52e-13	-7.498.477
204360_s_at	1.16e-12	3.53e-13	-7.497.895
210789_x_at	1.19e-12	3.60e-13	-7.494.946
202850_at	1.19e-12	3.60e-13	-7.494.864
207189_s_at	1.19e-12	3.61e-13	-7.494.452
212391_x_at	1.19e-12	3.63e-13	7.493.901
220927_s_at	1.21e-12	3.66e-13	-7.492.427
217678_at	1.21e-12	3.67e-13	7.492.243
40016_g_at	1.21e-12	3.69e-13	-7.491.428
38671_at	1.22e-12	3.70e-13	7.491.003
202672_s_at	1.22e-12	3.71e-13	-7.490.527
219509_at	1.22e-12	3.72e-13	-7.490.292
210497_x_at	1.23e-12	3.73e-13	748.979
209914_s_at	1.23e-12	3.73e-13	-7.489.726
219724_s_at	1.25e-12	3.81e-13	-748.672
211678_s_at	1.25e-12	3.81e-13	7.486.508

206893_at	1.27e-12	3.87e-13	-7.484.355
221901_at	1.29e-12	3.92e-13	-7.482.208
203689_s_at	1.29e-12	3.93e-13	7.481.913
200676_s_at	1.31e-12	3.98e-13	7.480.177
213104_at	1.31e-12	3.99e-13	7.479.725
203082_at	1.32e-12	4.01e-13	7.478.981
209583_s_at	1.32e-12	4.03e-13	7.478.401
210733_at	1.32e-12	4.03e-13	7.478.358
219971_at	1.33e-12	4.05e-13	-7.477.589
208436_s_at	1.33e-12	4.05e-13	-7.477.421
209573_s_at	1.34e-12	4.07e-13	-7.476.887
213675_at	1.34e-12	4.08e-13	-7.476.459
202368_s_at	1.35e-12	4.11e-13	-7.475.444
216191_s_at	1.36e-12	4.13e-13	-7.474.549
222101_s_at	1.36e-12	4.14e-13	-7.474.406
204534_at	1.36e-12	4.15e-13	-7.474.128
218016_s_at	1.36e-12	4.15e-13	7.473.891
218705_s_at	1.38e-12	4.20e-13	7.472.122
34225_at	1.38e-12	4.22e-13	7.471.427
202414_at	1.39e-12	4.24e-13	-7.470.791
214403_x_at	1.40e-12	4.27e-13	-746.982
218417_s_at	1.40e-12	4.28e-13	7.469.289
218218_at	1.40e-12	4.29e-13	7.469.161
212826_s_at	1.41e-12	4.29e-13	746.898
217458_at	1.41e-12	4.30e-13	-746.878
215198_s_at	1.42e-12	4.33e-13	-7.467.617
52005_at	1.43e-12	4.35e-13	7.466.881
212214_at	1.43e-12	4.37e-13	7.466.468
216383_at	1.43e-12	4.37e-13	7.466.276
216733_s_at	1.44e-12	4.40e-13	-7.465.266
214840_at	1.45e-12	4.45e-13	-7.463.771
206197_at	1.45e-12	4.45e-13	-7.463.712
208360_s_at	1.46e-12	4.47e-13	-7.463.082
203238_s_at	1.46e-12	4.47e-13	7.463.039
203625_x_at	1.47e-12	4.49e-13	7.462.453
211588_s_at	1.48e-12	4.51e-13	-7.461.552
218724_s_at	1.48e-12	4.54e-13	7.460.711
201684_s_at	1.50e-12	4.60e-13	7.458.753
201825_s_at	1.51e-12	4.62e-13	74.582
209430_at	1.53e-12	4.67e-13	7.456.519
214405_at	1.53e-12	4.68e-13	-7.456.138
215100_at	1.54e-12	4.71e-13	-7.455.265
210799_at	1.55e-12	4.75e-13	-7.454.011
207721_x_at	1.57e-12	4.80e-13	7.452.515

217583_at	1.57e-12	4.80e-13	-7.452.464
204646_at	1.57e-12	4.80e-13	-7.452.394
203245_s_at	1.57e-12	4.81e-13	7.451.957
200887_s_at	1.59e-12	4.87e-13	7.450.349
210039_s_at	1.59e-12	4.89e-13	-7.449.748
221747_at	1.61e-12	4.93e-13	-7.448.303
202307_s_at	1.62e-12	4.97e-13	7.447.179
204190_at	1.63e-12	5.01e-13	-7.446.165
202632_at	1.64e-12	5.02e-13	-7.445.672
206751_s_at	1.65e-12	5.06e-13	-7.444.609
206956_at	1.65e-12	5.06e-13	7.444.477
208037_s_at	1.66e-12	5.09e-13	-7.443.716
214349_at	1.68e-12	5.14e-13	-744.224
212885_at	1.68e-12	5.14e-13	7.442.166
216290_x_at	1.68e-12	5.15e-13	-7.441.972
91920_at	1.68e-12	5.15e-13	-7.441.923
218297_at	1.68e-12	5.16e-13	-7.441.723
217653_x_at	1.69e-12	5.20e-13	-7.440.551
218533_s_at	1.70e-12	5.22e-13	7.439.922
221621_at	1.71e-12	5.25e-13	7.439.013
209229_s_at	1.71e-12	5.27e-13	7.438.605
213590_at	1.73e-12	5.31e-13	-7.437.404
217676_at	1.73e-12	5.33e-13	-7.436.948
211114_x_at	1.74e-12	5.36e-13	7.435.975
221083_at	1.75e-12	5.39e-13	-7.435.281
205369_x_at	1.75e-12	5.39e-13	-7.435.264
216584_at	1.75e-12	5.39e-13	-7.435.133
204794_at	1.76e-12	5.42e-13	-7.434.384
215545_at	1.77e-12	5.43e-13	-7.434.097
212788_x_at	1.77e-12	5.45e-13	7.433.555
202513_s_at	1.78e-12	5.48e-13	7.432.744
207440_at	1.81e-12	5.56e-13	-7.430.622
213761_at	1.83e-12	5.64e-13	7.428.316
219142_at	1.84e-12	5.67e-13	-7.427.665
210601_at	1.87e-12	5.74e-13	-7.425.759
220671_at	1.87e-12	5.77e-13	-7.425.066
216738_at	1.88e-12	5.78e-13	-7.424.726
212693_at	1.89e-12	5.82e-13	-7.423.708
203501_at	1.89e-12	5.83e-13	7.423.632
212756_s_at	1.91e-12	5.87e-13	7.422.459
215192_at	1.91e-12	5.90e-13	-7.421.768
217640_x_at	1.92e-12	5.92e-13	7.421.306
208456_s_at	1.94e-12	5.99e-13	7.419.512
201615_x_at	1.94e-12	5.99e-13	-7.419.426

211966_at	1.97e-12	6.07e-13	7.417.578
208734_x_at	1.97e-12	6.08e-13	7.417.331
212774_at	1.98e-12	6.12e-13	7.416.308
215046_at	1.99e-12	6.14e-13	-741.579
202067_s_at	2.00e-12	6.16e-13	-7.415.215
208913_at	2.02e-12	6.24e-13	741.345
205489_at	2.03e-12	6.26e-13	-7.412.829
204361_s_at	2.04e-12	6.28e-13	7.412.369
207438_s_at	2.04e-12	6.30e-13	7.412.024
218574_s_at	2.05e-12	6.33e-13	7.411.275
205961_s_at	2.06e-12	6.35e-13	7.410.817
201480_s_at	2.07e-12	6.39e-13	740.996
209002_s_at	2.07e-12	6.40e-13	-7.409.609
205803_s_at	2.08e-12	6.43e-13	7.408.913
211088_s_at	2.09e-12	6.45e-13	-7.408.547
217988_at	2.18e-12	6.73e-13	-7.402.163
219762_s_at	2.18e-12	6.74e-13	7.401.947
203016_s_at	2.18e-12	6.74e-13	7.401.823
220699_s_at	2.18e-12	6.75e-13	-7.401.744
217275_at	2.19e-12	6.77e-13	-7.401.259
219112_at	2.20e-12	6.80e-13	7.400.697
201761_at	2.21e-12	6.83e-13	7.399.994
211579_at	2.24e-12	6.93e-13	-7.397.854
203480_s_at	2.26e-12	6.98e-13	-7.396.723
209155_s_at	2.29e-12	7.08e-13	7.394.527
203656_at	2.29e-12	7.09e-13	7.394.371
201380_at	2.29e-12	7.09e-13	7.394.329
208152_s_at	2.30e-12	7.11e-13	7.393.977
211210_x_at	2.30e-12	7.12e-13	-7.393.781
212856_at	2.31e-12	7.16e-13	-7.392.864
212614_at	2.32e-12	7.17e-13	7.392.691
212547_at	2.34e-12	7.23e-13	7.391.368
213682_at	2.35e-12	7.26e-13	7.390.778
215332_s_at	2.35e-12	7.26e-13	-7.390.731
202470_s_at	2.37e-12	7.33e-13	7.389.333
200982_s_at	2.37e-12	7.33e-13	-7.389.332
221179_at	2.38e-12	7.36e-13	-7.388.722
207844_at	2.40e-12	7.43e-13	-7.387.472
222317_at	2.40e-12	7.44e-13	-7.387.195
205872_x_at	2.45e-12	7.60e-13	-7.384.092
217713_x_at	2.45e-12	7.60e-13	-7.384.084
209179_s_at	2.46e-12	7.63e-13	7.383.388
200902_at	2.49e-12	7.72e-13	7.381.662
213813_x_at	2.50e-12	7.76e-13	-7.380.891

203795_s_at	2.50e-12	7.76e-13	7.380.822
216745_x_at	2.52e-12	7.83e-13	-7.379.527
201374_x_at	2.53e-12	7.84e-13	-7.379.442
215421_at	2.53e-12	7.87e-13	-7.378.849
202407_s_at	2.54e-12	7.87e-13	7.378.773
203731_s_at	2.54e-12	7.89e-13	737.849
207796_x_at	2.56e-12	7.95e-13	-7.377.303
213501_at	2.57e-12	7.97e-13	-7.376.897
AFFX-HUMGAPDH/M3	2.57e-12	7.98e-13	7.376.684
213634_s_at	2.57e-12	7.99e-13	7.376.579
205281_s_at	2.59e-12	8.06e-13	7.375.188
35265_at	2.62e-12	8.13e-13	-7.373.965
214915_at	2.63e-12	8.17e-13	-7.373.159
214758_at	2.64e-12	8.19e-13	-7.372.782
213154_s_at	2.66e-12	8.26e-13	7.371.636
210578_at	2.67e-12	8.32e-13	-7.370.552
209497_s_at	2.68e-12	8.33e-13	7.370.298
209222_s_at	2.68e-12	8.34e-13	7.370.178
210086_at	2.68e-12	8.35e-13	-7.369.963
206386_at	2.69e-12	8.38e-13	-7.369.342
219439_at	2.70e-12	8.41e-13	7.368.816
202105_at	2.73e-12	8.50e-13	7.367.262
200964_at	2.73e-12	8.50e-13	7.367.236
214647_s_at	2.74e-12	8.54e-13	7.366.644
219232_s_at	2.77e-12	8.62e-13	7.365.233
217812_at	2.77e-12	8.62e-13	7.365.217
211988_at	2.80e-12	8.71e-13	7.363.695
205967_at	2.82e-12	8.77e-13	7.362.556
218871_x_at	2.82e-12	8.78e-13	7.362.469
213982_s_at	2.85e-12	8.87e-13	736.092
207253_s_at	2.87e-12	8.94e-13	735.967
208983_s_at	2.94e-12	9.16e-13	7.356.129
205760_s_at	2.95e-12	9.20e-13	7.355.435
211495_x_at	2.95e-12	9.20e-13	-7.355.374
201322_at	2.97e-12	9.27e-13	7.354.295
212309_at	3.00e-12	9.35e-13	7.353.005
214489_at	3.00e-12	9.37e-13	-7.352.749
212535_at	3.00e-12	9.37e-13	-7.352.655
221370_at	3.03e-12	9.44e-13	-7.351.534
220210_at	3.03e-12	9.45e-13	-7.351.399
210241_s_at	3.03e-12	9.46e-13	-7.351.266
214855_s_at	3.04e-12	9.49e-13	7.350.733
219055_at	3.10e-12	9.68e-13	7.347.822
221860_at	3.11e-12	9.72e-13	-7.347.168

216328_at	3.12e-12	9.73e-13	-7.347.004
216741_at	3.13e-12	9.77e-13	-7.346.408
207929_at	3.15e-12	9.85e-13	-7.345.188
210235_s_at	3.16e-12	9.88e-13	7.344.665
221994_at	3.17e-12	9.91e-13	-7.344.231
216228_s_at	3.19e-12	9.96e-13	7.343.573
201908_at	3.24e-12	1.01e-12	7.341.049
221752_at	3.24e-12	1.01e-12	7.341.003
40093_at	3.26e-12	1.02e-12	7.339.933
202391_at	3.27e-12	1.02e-12	-7.339.561
220006_at	3.28e-12	1.03e-12	-7.338.968
216441_at	3.30e-12	1.03e-12	-7.338.087
221731_x_at	3.35e-12	1.05e-12	733.583
201995_at	3.36e-12	1.05e-12	7.335.608
208414_s_at	3.36e-12	1.05e-12	-7.335.372
207366_at	3.37e-12	1.05e-12	-73.351
221679_s_at	3.38e-12	1.06e-12	-7.334.635
214071_at	3.45e-12	1.08e-12	7.331.602
220737_at	3.46e-12	1.08e-12	-7.331.022
202947_s_at	3.47e-12	1.09e-12	-7.330.429
202873_at	3.48e-12	1.09e-12	7.329.999
220962_s_at	3.48e-12	1.09e-12	-7.329.971
210737_at	3.48e-12	1.09e-12	-7.329.909
209832_s_at	3.49e-12	1.09e-12	7.329.398
208951_at	3.53e-12	1.11e-12	-7.327.877
214058_at	3.55e-12	1.11e-12	-7.326.969
206973_at	3.57e-12	1.12e-12	-7.326.131
205325_at	3.59e-12	1.13e-12	-7.325.057
212140_at	3.59e-12	1.13e-12	7.325.011
AFFX-HUMISGF3A/M9	3.62e-12	1.13e-12	7.324.075
214842_s_at	3.64e-12	1.14e-12	-7.323.145
203883_s_at	3.66e-12	1.15e-12	7.322.413
202704_at	3.66e-12	1.15e-12	-7.322.104
210869_s_at	3.66e-12	1.15e-12	7.322.047
215352_at	3.73e-12	1.17e-12	-7.319.452
207019_s_at	3.75e-12	1.18e-12	-73.187
206608_s_at	3.77e-12	1.18e-12	-7.317.835
208284_x_at	3.77e-12	1.18e-12	-7.317.521
209424_s_at	3.79e-12	1.19e-12	-7.316.843
212920_at	3.90e-12	1.22e-12	7.312.567
211048_s_at	3.90e-12	1.23e-12	7.312.401
221272_s_at	3.94e-12	1.24e-12	-731.097
213141_at	3.97e-12	1.25e-12	-730.988
217020_at	4.00e-12	1.26e-12	-7.308.641

208491_s_at	4.06e-12	1.27e-12	-7.306.497
212234_at	4.07e-12	1.28e-12	7.305.995
212915_at	4.11e-12	1.29e-12	-7.304.498
202157_s_at	4.11e-12	1.29e-12	-7.304.382
218649_x_at	4.11e-12	1.29e-12	7.304.294
218443_s_at	4.17e-12	1.31e-12	7.302.356
202948_at	4.18e-12	1.31e-12	-7.301.821
210405_x_at	4.20e-12	1.32e-12	-7.301.081
213737_x_at	4.20e-12	1.32e-12	-7.301.027
216667_at	4.20e-12	1.32e-12	-7.300.995
207446_at	4.24e-12	1.33e-12	-7.299.763
206896_s_at	4.24e-12	1.33e-12	-7.299.672
207698_at	4.32e-12	1.36e-12	-7.296.901
222237_s_at	4.37e-12	1.38e-12	7.294.849
221698_s_at	4.38e-12	1.38e-12	-7.294.786
202856_s_at	4.38e-12	1.38e-12	7.294.656
208579_x_at	4.39e-12	1.38e-12	729.429
222000_at	4.40e-12	1.39e-12	7.293.858
216679_at	4.48e-12	1.41e-12	-7.291.026
213408_s_at	4.54e-12	1.43e-12	7.289.061
218763_at	4.62e-12	1.45e-12	7.286.602
213955_at	4.69e-12	1.48e-12	-7.284.295
208731_at	4.75e-12	1.50e-12	728.211
207024_at	4.78e-12	1.51e-12	-7.281.154
207563_s_at	4.79e-12	1.51e-12	7.280.797
217821_s_at	4.84e-12	1.53e-12	7.279.314
213904_at	4.88e-12	1.54e-12	7.278.076
214972_at	4.88e-12	1.54e-12	-7.277.913
215759_at	4.90e-12	1.54e-12	-7.277.485
212126_at	4.94e-12	1.56e-12	7.276.304
204317_at	4.94e-12	1.56e-12	7.276.157
207800_at	4.94e-12	1.56e-12	-7.276.073
221196_x_at	4.96e-12	1.56e-12	7.275.627
204105_s_at	5.00e-12	1.58e-12	7.274.198
48106_at	5.06e-12	1.60e-12	7.272.552
203835_at	5.07e-12	1.60e-12	-7.272.204
210172_at	5.10e-12	1.61e-12	-7.271.107
201455_s_at	5.14e-12	1.62e-12	7.269.849
206207_at	5.15e-12	1.63e-12	-7.269.693
217364_x_at	5.15e-12	1.63e-12	7.269.612
213994_s_at	5.17e-12	1.63e-12	-7.268.945
222241_at	5.26e-12	1.66e-12	-7.266.417
208613_s_at	5.29e-12	1.67e-12	7.265.547
200099_s_at	5.30e-12	1.67e-12	7.265.328

200698_at	5.44e-12	1.72e-12	7.261.214
201121_s_at	5.45e-12	1.72e-12	-7.260.874
207571_x_at	5.50e-12	1.74e-12	-7.259.496
213445_at	5.50e-12	1.74e-12	7.259.436
219925_at	5.53e-12	1.75e-12	-7.258.746
207480_s_at	5.54e-12	1.75e-12	7.258.357
207763_at	5.57e-12	1.76e-12	-7.257.615
222023_at	5.57e-12	1.76e-12	-7.257.438
215901_at	5.59e-12	1.77e-12	-7.257.058
200799_at	5.64e-12	1.79e-12	7.255.514
218796_at	5.65e-12	1.79e-12	7.255.343
221748_s_at	5.68e-12	1.80e-12	-7.254.377
219480_at	5.74e-12	1.82e-12	-7.252.837
210323_at	5.82e-12	1.84e-12	-7.250.837
221176_x_at	5.82e-12	1.84e-12	-7.250.689
211647_x_at	5.88e-12	1.86e-12	-7.249.178
209500_x_at	5.92e-12	1.88e-12	-7.248.107
209286_at	5.94e-12	1.88e-12	-7.247.639
205750_at	5.95e-12	1.89e-12	-7.247.327
91580_at	5.95e-12	1.89e-12	-7.247.179
217754_at	5.97e-12	1.89e-12	7.246.866
217576_x_at	5.99e-12	1.90e-12	-7.246.273
203737_s_at	6.04e-12	1.91e-12	724.501
205129_at	6.04e-12	1.92e-12	7.244.994
202356_s_at	6.04e-12	1.92e-12	7.244.901
219081_at	6.06e-12	1.92e-12	7.244.366
217223_s_at	6.06e-12	1.92e-12	7.244.281
211924_s_at	6.08e-12	1.93e-12	-7.243.789
202174_s_at	6.16e-12	1.96e-12	7.241.804
219928_s_at	6.19e-12	1.96e-12	7.241.155
204006_s_at	6.20e-12	1.97e-12	-7.240.707
211541_s_at	6.22e-12	1.98e-12	-7.240.288
208042_at	6.26e-12	1.99e-12	7.239.438
202925_s_at	6.26e-12	1.99e-12	7.239.252
213739_at	6.26e-12	1.99e-12	7.239.232
207394_at	6.31e-12	2.00e-12	723.807
216271_x_at	6.38e-12	2.03e-12	-7.236.249
206939_at	6.41e-12	2.04e-12	-7.235.514
217692_at	6.47e-12	2.06e-12	-7.234.293
213263_s_at	6.47e-12	2.06e-12	-7.234.243
219390_at	6.47e-12	2.06e-12	7.234.203
221471_at	6.49e-12	2.06e-12	7.233.672
213691_at	6.53e-12	2.08e-12	-7.232.719
212222_at	6.53e-12	2.08e-12	7.232.689

36475_at	6.56e-12	2.09e-12	-7.231.993
200874_s_at	6.58e-12	2.09e-12	7.231.397
217549_at	6.63e-12	2.11e-12	-723.034
209632_at	6.66e-12	2.12e-12	-722.971
218600_at	6.71e-12	2.14e-12	-7.228.388
222151_s_at	6.74e-12	2.15e-12	7.227.671
216083_s_at	6.89e-12	2.19e-12	-7.224.327
213152_s_at	6.93e-12	2.21e-12	-7.223.516
203250_at	6.96e-12	2.22e-12	7.222.826
208544_at	7.05e-12	2.24e-12	-7.220.914
205320_at	7.15e-12	2.28e-12	-7.218.663
206713_at	7.16e-12	2.28e-12	-7.218.495
206283_s_at	7.18e-12	2.29e-12	-7.218.083
210011_s_at	7.20e-12	2.29e-12	7.217.569
210397_at	7.30e-12	2.33e-12	-7.215.386
201927_s_at	7.31e-12	2.33e-12	721.515
219888_at	7.32e-12	2.33e-12	7.215.039
204906_at	7.35e-12	2.34e-12	-7.214.281
203496_s_at	7.40e-12	2.36e-12	7.213.254
201331_s_at	7.45e-12	2.38e-12	-7.212.289
218520_at	7.45e-12	2.38e-12	7.212.206
218188_s_at	7.47e-12	2.38e-12	7.211.888
219051_x_at	7.48e-12	2.39e-12	-7.211.538
205269_at	7.57e-12	2.42e-12	-720.978
207463_x_at	7.61e-12	2.43e-12	7.208.883
219189_at	7.63e-12	2.44e-12	720.851
210226_at	7.71e-12	2.46e-12	-7.206.939
204297_at	7.71e-12	2.46e-12	7.206.906
204590_x_at	7.85e-12	2.51e-12	7.204.119
204629_at	7.91e-12	2.53e-12	7.202.863
215539_at	7.93e-12	2.53e-12	-720.249
201989_s_at	7.94e-12	2.54e-12	-7.202.368
209718_at	8.01e-12	2.56e-12	7.200.929
207191_s_at	8.01e-12	2.56e-12	-7.200.901
201494_at	8.05e-12	2.57e-12	-720.007
214323_s_at	8.06e-12	2.58e-12	7.199.905
218445_at	8.09e-12	2.59e-12	7.199.358
219135_s_at	8.11e-12	2.59e-12	-719.902
207187_at	8.19e-12	2.62e-12	-7.197.469
214260_at	8.19e-12	2.62e-12	7.197.342
215051_x_at	8.23e-12	2.63e-12	-7.196.731
218420_s_at	8.26e-12	2.64e-12	7.196.129
218838_s_at	8.30e-12	2.65e-12	-7.195.381
209052_s_at	8.36e-12	2.67e-12	7.194.265

218756_s_at	8.36e-12	2.68e-12	7.194.197
213028_at	8.38e-12	2.68e-12	7.193.752
214817_at	8.40e-12	2.69e-12	-7.193.477
216743_at	8.51e-12	2.73e-12	-719.139
209919_x_at	8.56e-12	2.74e-12	-7.190.396
202346_at	8.58e-12	2.75e-12	7.190.144
210187_at	8.59e-12	2.75e-12	7.189.891
222214_at	8.60e-12	2.76e-12	7.189.687
214422_at	8.60e-12	2.76e-12	-7.189.627
220972_s_at	8.66e-12	2.78e-12	-7.188.633
202717_s_at	8.68e-12	2.78e-12	7.188.226
211956_s_at	8.70e-12	2.79e-12	7.187.849
205058_at	8.73e-12	2.80e-12	-7.187.303
212838_at	8.80e-12	2.82e-12	-7.186.057
211384_s_at	8.84e-12	2.83e-12	-7.185.379
202794_at	9.02e-12	2.89e-12	-7.182.347
207614_s_at	9.05e-12	2.90e-12	7.181.788
209394_at	9.06e-12	2.91e-12	-7.181.544
222137_at	9.28e-12	2.98e-12	-7.177.796
217353_at	9.31e-12	2.99e-12	7.177.414
212937_s_at	9.31e-12	2.99e-12	-7.177.294
204883_s_at	9.32e-12	2.99e-12	7.177.116
200611_s_at	9.36e-12	3.01e-12	7.176.401
217026_at	9.43e-12	3.03e-12	-7.175.386
213490_s_at	9.47e-12	3.04e-12	7.174.673
212446_s_at	9.47e-12	3.04e-12	7.174.581
219358_s_at	9.47e-12	3.04e-12	-7.174.535
202935_s_at	9.59e-12	3.08e-12	717.274
220533_at	9.60e-12	3.09e-12	-7.172.444
202112_at	9.65e-12	3.10e-12	7.171.736
203169_at	9.68e-12	3.11e-12	7.171.214
209843_s_at	9.68e-12	3.11e-12	-7.171.095
216373_at	9.70e-12	3.12e-12	-7.170.855
202073_at	9.73e-12	3.13e-12	717.031
215616_s_at	9.74e-12	3.13e-12	-717.014
214356_s_at	9.75e-12	3.14e-12	7.170.011
220044_x_at	9.83e-12	3.16e-12	7.168.695
213865_at	9.86e-12	3.17e-12	-7.168.186
204022_at	9.92e-12	3.19e-12	716.724
211322_s_at	9.96e-12	3.21e-12	-7.166.642
202163_s_at	9.96e-12	3.21e-12	7.166.598
205492_s_at	9.97e-12	3.21e-12	-7.166.454
215875_at	9.97e-12	3.21e-12	-7.166.348
217035_at	1.00e-11	3.22e-12	-7.165.806

218761_at	1.00e-11	3.22e-12	7.165.725
218078_s_at	1.00e-11	3.23e-12	71.657
205848_at	1.00e-11	3.23e-12	-7.165.277
220439_at	1.01e-11	3.25e-12	-7.164.395
213118_at	1.01e-11	3.25e-12	7.164.377
215597_x_at	1.03e-11	3.30e-12	-7.161.992
208390_s_at	1.03e-11	3.33e-12	-7.160.645
217071_s_at	1.04e-11	3.34e-12	-7.160.339
210929_s_at	1.05e-11	3.37e-12	-7.158.953
215946_x_at	1.05e-11	3.38e-12	-715.831
218219_s_at	1.06e-11	3.41e-12	7.157.071
210922_at	1.07e-11	3.45e-12	-7.155.319
215275_at	1.07e-11	3.46e-12	-7.154.983
208014_x_at	1.07e-11	3.47e-12	-71.546
202588_at	1.08e-11	3.47e-12	-7.154.453
213749_at	1.09e-11	3.51e-12	-7.152.974
211171_s_at	1.10e-11	3.55e-12	-7.150.859
218872_at	1.11e-11	3.57e-12	7.150.279
220109_at	1.11e-11	3.57e-12	-715.004
204826_at	1.11e-11	3.58e-12	714.972
216178_x_at	1.11e-11	3.58e-12	7.149.559
221754_s_at	1.11e-11	3.59e-12	7.149.319
213373_s_at	1.13e-11	3.66e-12	7.146.406
215608_at	1.13e-11	3.66e-12	-7.146.404
220386_s_at	1.14e-11	3.67e-12	7.145.966
217346_at	1.14e-11	3.69e-12	7.145.093
206903_at	1.15e-11	3.73e-12	-7.143.495
212302_at	1.16e-11	3.76e-12	7.142.068
222120_at	1.16e-11	3.76e-12	7.142.058
212883_at	1.17e-11	3.77e-12	-7.141.847
215246_at	1.17e-11	3.79e-12	-7.140.948
219901_at	1.17e-11	3.79e-12	7.140.927
204965_at	1.18e-11	3.81e-12	-7.140.053
213663_s_at	1.18e-11	3.83e-12	-7.139.435
205453_at	1.19e-11	3.86e-12	-7.138.242
214107_x_at	1.20e-11	3.88e-12	7.137.298
205972_at	1.20e-11	3.89e-12	-7.137.206
219116_s_at	1.21e-11	3.93e-12	7.135.377
215163_at	1.22e-11	3.94e-12	7.135.223
215293_s_at	1.22e-11	3.95e-12	7.134.679
218194_at	1.22e-11	3.96e-12	7.134.132
216362_at	1.22e-11	3.97e-12	-7.133.985
219168_s_at	1.23e-11	3.99e-12	-7.133.019
216728_at	1.23e-11	4.00e-12	-7.132.761

221981_s_at	1.24e-11	4.03e-12	-7.131.602
204943_at	1.25e-11	4.04e-12	-7.131.076
209269_s_at	1.25e-11	4.05e-12	-7.130.899
217348_x_at	1.26e-11	4.08e-12	-7.129.844
205175_s_at	1.26e-11	4.09e-12	-7.129.289
220790_s_at	1.27e-11	4.10e-12	-7.128.834
214806_at	1.27e-11	4.11e-12	7.128.405
210408_s_at	1.27e-11	4.12e-12	-7.128.398
212977_at	1.28e-11	4.15e-12	7.126.972
213223_at	1.28e-11	4.16e-12	7.126.728
204836_at	1.29e-11	4.18e-12	-7.126.153
204486_at	1.30e-11	4.22e-12	-7.124.632
215308_at	1.30e-11	4.22e-12	-7.124.577
210788_s_at	1.30e-11	4.23e-12	7.124.124
217407_x_at	1.30e-11	4.23e-12	7.124.105
204668_at	1.31e-11	4.25e-12	7.123.623
221112_at	1.31e-11	4.27e-12	-7.122.813
217408_at	1.34e-11	4.34e-12	7.120.293
217394_at	1.34e-11	4.36e-12	-7.119.528
203267_s_at	1.34e-11	4.37e-12	7.119.237
214814_at	1.34e-11	4.37e-12	-711.916
213800_at	1.35e-11	4.39e-12	-7.118.536
210144_at	1.35e-11	4.39e-12	7.118.491
215001_s_at	1.35e-11	4.40e-12	7.118.227
208097_s_at	1.38e-11	4.48e-12	7.115.364
220835_s_at	1.38e-11	4.49e-12	-7.115.134
207900_at	1.39e-11	4.53e-12	-7.113.651
219905_at	1.40e-11	4.56e-12	-7.112.597
200800_s_at	1.40e-11	4.56e-12	7.112.541
55692_at	1.41e-11	4.59e-12	7.111.586
212944_at	1.41e-11	4.60e-12	-7.111.372
210606_x_at	1.41e-11	4.60e-12	-7.111.357
209615_s_at	1.41e-11	4.60e-12	7.111.154
204667_at	1.42e-11	4.61e-12	7.111.075
213958_at	1.42e-11	4.62e-12	-7.110.674
216269_s_at	1.43e-11	4.65e-12	-7.109.568
208728_s_at	1.43e-11	4.66e-12	7.109.385
202626_s_at	1.44e-11	4.68e-12	7.108.495
205191_at	1.44e-11	4.69e-12	710.839
220341_s_at	1.44e-11	4.70e-12	-7.108.045
222143_s_at	1.45e-11	4.72e-12	7.107.489
205522_at	1.45e-11	4.72e-12	7.107.462
215891_s_at	1.45e-11	4.72e-12	710.723
203454_s_at	1.46e-11	4.77e-12	7.105.771

209516_at	1.46e-11	4.77e-12	710.568
202922_at	1.48e-11	4.83e-12	-7.103.859
214784_x_at	1.50e-11	4.90e-12	7.101.437
217769_s_at	1.52e-11	4.96e-12	7.099.806
207771_at	1.52e-11	4.97e-12	-7.099.343
204594_s_at	1.53e-11	4.99e-12	7.098.803
209949_at	1.53e-11	4.99e-12	7.098.771
209024_s_at	1.53e-11	5.00e-12	7.098.446
205122_at	1.56e-11	5.08e-12	7.096.121
203111_s_at	1.56e-11	5.10e-12	-7.095.438
204293_at	1.57e-11	5.14e-12	7.094.298
203677_s_at	1.58e-11	5.16e-12	7.093.578
217017_at	1.59e-11	5.20e-12	-7.092.567
35201_at	1.60e-11	5.24e-12	7.091.272
211313_s_at	1.61e-11	5.27e-12	-709.047
212601_at	1.62e-11	5.30e-12	-7.089.642
205470_s_at	1.63e-11	5.34e-12	-7.088.425
207782_s_at	1.64e-11	5.36e-12	7.087.758
208253_at	1.64e-11	5.37e-12	-7.087.547
207450_s_at	1.66e-11	5.42e-12	-7.085.929
211688_x_at	1.66e-11	5.43e-12	-7.085.822
222310_at	1.68e-11	5.48e-12	-7.084.421
221719_s_at	1.69e-11	5.54e-12	-708.267
211639_x_at	1.71e-11	5.58e-12	-7.081.471
212081_x_at	1.71e-11	5.60e-12	7.080.993
201016_at	1.71e-11	5.60e-12	7.080.895
217264_s_at	1.73e-11	5.67e-12	-7.078.994
207839_s_at	1.74e-11	5.69e-12	707.848
211742_s_at	1.75e-11	5.72e-12	-7.077.869
214508_x_at	1.76e-11	5.75e-12	-7.076.893
201641_at	1.78e-11	5.84e-12	-707.448
209733_at	1.79e-11	5.87e-12	7.073.846
210394_x_at	1.81e-11	5.93e-12	7.072.322
217326_x_at	1.82e-11	5.97e-12	-7.071.042
200912_s_at	1.83e-11	5.99e-12	7.070.741
212843_at	1.84e-11	6.04e-12	-7.069.401
216409_at	1.84e-11	6.04e-12	-7.069.359
203857_s_at	1.86e-11	6.08e-12	-706.834
207939_x_at	1.86e-11	6.10e-12	7.067.709
216805_at	1.86e-11	6.11e-12	7.067.561
221773_at	1.87e-11	6.14e-12	7.066.785
200778_s_at	1.88e-11	6.17e-12	7.066.164
218904_s_at	1.90e-11	6.22e-12	7.064.769
219327_s_at	1.90e-11	6.23e-12	-7.064.631

201410_at	1.90e-11	6.24e-12	7.064.364
212659_s_at	1.90e-11	6.24e-12	-7.064.221
211052_s_at	1.91e-11	6.28e-12	7.063.276
214020_x_at	1.92e-11	6.31e-12	7.062.559
202518_at	1.92e-11	6.32e-12	7.062.424
203964_at	1.92e-11	6.32e-12	7.062.409
207744_at	1.93e-11	6.35e-12	-7.061.679
202113_s_at	1.93e-11	6.35e-12	7.061.566
213604_at	1.94e-11	6.36e-12	-7.061.431
213883_s_at	1.94e-11	6.37e-12	7.061.236
41856_at	1.96e-11	6.42e-12	-7.059.895
221034_s_at	1.98e-11	6.49e-12	-7.058.233
210380_s_at	1.98e-11	6.49e-12	-7.058.228
204295_at	1.98e-11	6.49e-12	-7.058.195
213440_at	1.98e-11	6.52e-12	7.057.566
216069_at	1.99e-11	6.53e-12	-7.057.319
217943_s_at	1.99e-11	6.53e-12	7.057.284
214233_at	2.01e-11	6.62e-12	-7.055.214
200869_at	2.03e-11	6.66e-12	7.054.159
208960_s_at	2.03e-11	6.67e-12	-7.054.005
217905_at	2.03e-11	6.69e-12	7.053.637
216936_at	2.04e-11	6.72e-12	-7.052.917
215919_s_at	2.05e-11	6.73e-12	-7.052.583
214721_x_at	2.06e-11	6.77e-12	7.051.785
210234_at	2.06e-11	6.78e-12	-7.051.619
204648_at	2.06e-11	6.78e-12	-7.051.513
216350_s_at	2.06e-11	6.80e-12	-7.051.138
216471_x_at	2.07e-11	6.81e-12	7.050.779
204556_s_at	2.08e-11	6.84e-12	-705.025
215071_s_at	2.09e-11	6.88e-12	7.049.228
217376_at	2.10e-11	6.93e-12	-7.048.181
212902_at	2.11e-11	6.95e-12	7.047.733
216546_s_at	2.11e-11	6.95e-12	-7.047.691
217673_x_at	2.14e-11	7.04e-12	7.045.594
35156_at	2.14e-11	7.05e-12	-7.045.429
214449_s_at	2.14e-11	7.06e-12	7.045.206
204070_at	2.16e-11	7.13e-12	-7.043.791
206271_at	2.18e-11	7.18e-12	-7.042.702
220781_at	2.19e-11	7.21e-12	-7.041.991
218295_s_at	2.21e-11	7.29e-12	7.040.262
221286_s_at	2.21e-11	7.30e-12	-7.040.034
205184_at	2.22e-11	7.31e-12	7.039.816
220052_s_at	2.23e-11	7.35e-12	7.039.106
200803_s_at	2.23e-11	7.35e-12	-7.039.039

207746_at	2.23e-11	7.36e-12	7.038.876
213549_at	2.24e-11	7.38e-12	7.038.406
214239_x_at	2.25e-11	7.42e-12	7.037.628
202514_at	2.25e-11	7.44e-12	7.037.189
205542_at	2.26e-11	7.46e-12	7.036.732
210104_at	2.27e-11	7.48e-12	7.036.295
210785_s_at	2.28e-11	7.51e-12	-7.035.577
217320_at	2.28e-11	7.54e-12	-703.515
209963_s_at	2.29e-11	7.55e-12	-7.034.879
203175_at	2.29e-11	7.55e-12	7.034.808
221738_at	2.29e-11	7.56e-12	7.034.594
202545_at	2.29e-11	7.57e-12	7.034.477
209981_at	2.31e-11	7.62e-12	-7.033.445
221199_at	2.31e-11	7.63e-12	-7.033.224
210314_x_at	2.32e-11	7.66e-12	-7.032.669
217393_x_at	2.32e-11	7.69e-12	7.032.062
200831_s_at	2.36e-11	7.81e-12	7.029.623
218388_at	2.37e-11	7.85e-12	7.028.878
213351_s_at	2.41e-11	7.96e-12	7.026.623
208219_at	2.41e-11	7.98e-12	-7.026.328
203845_at	2.43e-11	8.05e-12	-7.024.927
203452_at	2.44e-11	8.08e-12	7.024.383
216066_at	2.45e-11	8.11e-12	-702.376
221974_at	2.48e-11	8.21e-12	7.021.885
205815_at	2.51e-11	8.31e-12	7.019.922
220989_s_at	2.52e-11	8.34e-12	-701.945
216123_x_at	2.55e-11	8.45e-12	-7.017.443
217957_at	2.58e-11	8.55e-12	-7.015.561
220198_s_at	2.62e-11	8.69e-12	7.013.127
203349_s_at	2.65e-11	8.78e-12	7.011.379
217664_at	2.66e-11	8.81e-12	-7.010.934
213058_at	2.67e-11	8.84e-12	7.010.385
209471_s_at	2.67e-11	8.86e-12	7.010.022
220538_at	2.68e-11	8.90e-12	-7.009.356
201349_at	2.70e-11	8.95e-12	7.008.542
212708_at	2.71e-11	8.98e-12	7.007.924
219671_at	2.72e-11	9.03e-12	-7.007.146
206457_s_at	2.73e-11	9.06e-12	-7.006.518
209379_s_at	2.75e-11	9.11e-12	700.571
216189_at	2.75e-11	9.12e-12	-7.005.468
215449_at	2.75e-11	9.13e-12	-7.005.305
207387_s_at	2.78e-11	9.24e-12	-7.003.515
200724_at	2.79e-11	9.27e-12	-7.003.071
210108_at	2.80e-11	9.29e-12	7.002.665

220788_s_at	2.81e-11	9.33e-12	7.002.039
211732_x_at	2.81e-11	9.33e-12	-7.001.966
217930_s_at	2.81e-11	9.35e-12	-700.172
219448_at	2.82e-11	9.37e-12	700.137
216340_s_at	2.84e-11	9.42e-12	-7.000.466
217902_s_at	2.85e-11	9.46e-12	6.999.833
209826_at	2.86e-11	9.49e-12	-6.999.437
206659_at	2.86e-11	9.50e-12	6.999.147
208823_s_at	2.89e-11	9.61e-12	6.997.477
204928_s_at	2.90e-11	9.65e-12	6.996.746
210667_s_at	2.90e-11	9.66e-12	-6.996.678
221088_s_at	2.91e-11	9.67e-12	6.996.452
1316_at	2.91e-11	9.67e-12	-6.996.387
215826_x_at	2.93e-11	9.73e-12	-6.995.433
206735_at	2.94e-11	9.78e-12	-6.994.635
217424_at	2.94e-11	9.79e-12	-6.994.549
36554_at	2.96e-11	9.86e-12	-6.993.422
200088_x_at	2.97e-11	9.89e-12	6.992.977
216898_s_at	2.99e-11	9.93e-12	-6.992.253
214937_x_at	3.00e-11	9.98e-12	6.991.471
212571_at	3.05e-11	1.01e-11	6.988.929
218211_s_at	3.07e-11	1.02e-11	-698.778
217401_at	3.08e-11	1.03e-11	-6.987.188
217560_at	3.09e-11	1.03e-11	-6.986.852
220393_at	3.11e-11	1.04e-11	-698.566
215092_s_at	3.12e-11	1.04e-11	-6.985.152
212917_x_at	3.14e-11	1.05e-11	6.984.305
218284_at	3.16e-11	1.05e-11	698.343
215863_at	3.19e-11	1.06e-11	-6.981.626
209299_x_at	3.23e-11	1.07e-11	6.979.972
204420_at	3.28e-11	1.09e-11	-6.977.544
207083_s_at	3.30e-11	1.10e-11	-6.976.341
213757_at	3.30e-11	1.10e-11	-6.976.243
212307_s_at	3.32e-11	1.11e-11	697.516
38710_at	3.34e-11	1.12e-11	6.974.229
208466_at	3.34e-11	1.12e-11	-6.974.195
205724_at	3.39e-11	1.13e-11	-697.219
212769_at	3.39e-11	1.13e-11	6.972.028
217299_s_at	3.41e-11	1.14e-11	6.971.167
222330_at	3.41e-11	1.14e-11	-6.970.901
214493_s_at	3.42e-11	1.14e-11	-6.970.825
208392_x_at	3.42e-11	1.14e-11	-6.970.587
208633_s_at	3.42e-11	1.14e-11	-6.970.523
215636_at	3.46e-11	1.16e-11	-6.968.596

212114_at	3.47e-11	1.16e-11	6.968.341
212121_at	3.47e-11	1.16e-11	6.968.233
219679_s_at	3.50e-11	1.17e-11	6.966.989
209055_s_at	3.51e-11	1.17e-11	6.966.505
214735_at	3.54e-11	1.18e-11	-6.965.086
216074_x_at	3.55e-11	1.18e-11	6.964.805
209289_at	3.59e-11	1.20e-11	-6.962.733
207336_at	3.66e-11	1.22e-11	-6.959.964
219131_at	3.66e-11	1.22e-11	695.972
208122_x_at	3.70e-11	1.24e-11	-6.957.948
214703_s_at	3.77e-11	1.26e-11	-6.955.187
215026_x_at	3.78e-11	1.26e-11	-6.954.858
205799_s_at	3.79e-11	1.27e-11	-6.954.424
202542_s_at	3.79e-11	1.27e-11	6.954.385
216848_at	3.79e-11	1.27e-11	-6.954.232
205441_at	3.82e-11	1.28e-11	-6.952.909
206223_at	3.82e-11	1.28e-11	-6.952.773
220441_at	3.85e-11	1.29e-11	-6.951.703
213688_at	3.85e-11	1.29e-11	-6.951.551
220155_s_at	3.86e-11	1.29e-11	6.951.375
211223_at	3.86e-11	1.29e-11	-6.951.174
206962_x_at	3.87e-11	1.29e-11	-6.950.934
218141_at	3.87e-11	1.30e-11	6.950.695
213295_at	3.87e-11	1.30e-11	-6.950.647
212706_at	3.88e-11	1.30e-11	6.950.334
220812_s_at	3.89e-11	1.30e-11	-6.949.821
215652_at	3.91e-11	1.31e-11	-6.948.978
207616_s_at	3.91e-11	1.31e-11	-6.948.967
208083_s_at	3.92e-11	1.31e-11	-6.948.537
213386_at	3.93e-11	1.32e-11	6.948.373
210583_at	3.93e-11	1.32e-11	-6.948.269
213162_at	3.93e-11	1.32e-11	-6.948.201
215438_x_at	3.94e-11	1.32e-11	6.947.904
214238_at	3.94e-11	1.32e-11	-6.947.751
206360_s_at	3.97e-11	1.33e-11	-6.946.685
208712_at	4.01e-11	1.35e-11	-6.944.778
220966_x_at	4.03e-11	1.35e-11	694.393
220976_s_at	4.04e-11	1.35e-11	-6.943.819
213602_s_at	4.05e-11	1.36e-11	-6.943.291
202197_at	4.08e-11	1.37e-11	6.942.229
219746_at	4.08e-11	1.37e-11	-6.942.069
214582_at	4.12e-11	1.38e-11	-694.069
219288_at	4.13e-11	1.39e-11	694.004
208207_at	4.14e-11	1.39e-11	-6.939.814

201723_s_at	4.17e-11	1.40e-11	6.938.577
212782_x_at	4.18e-11	1.41e-11	6.938.084
216984_x_at	4.19e-11	1.41e-11	-6.937.724
215199_at	4.20e-11	1.41e-11	-6.937.285
218207_s_at	4.22e-11	1.42e-11	-6.936.708
219630_at	4.23e-11	1.42e-11	6.936.385
205212_s_at	4.24e-11	1.42e-11	-6.935.925
220372_at	4.24e-11	1.43e-11	-6.935.739
219325_s_at	4.26e-11	1.43e-11	-6.935.217
221867_at	4.29e-11	1.44e-11	-6.933.969
214748_at	4.30e-11	1.45e-11	-6.933.567
206733_at	4.31e-11	1.45e-11	-693.311
212437_at	4.31e-11	1.45e-11	6.933.068
212996_s_at	4.32e-11	1.45e-11	693.277
214460_at	4.36e-11	1.47e-11	-6.931.429
216184_s_at	4.36e-11	1.47e-11	-6.931.348
213717_at	4.37e-11	1.47e-11	-6.930.984
219881_s_at	4.37e-11	1.47e-11	6.930.823
218175_at	4.39e-11	1.48e-11	-6.930.001
216036_x_at	4.40e-11	1.48e-11	-6.929.948
204419_x_at	4.40e-11	1.48e-11	-6.929.946
218787_x_at	4.40e-11	1.48e-11	6.929.759
208478_s_at	4.41e-11	1.49e-11	6.929.431
216252_x_at	4.41e-11	1.49e-11	-6.929.278
220952_s_at	4.43e-11	1.49e-11	6.928.454
214800_x_at	4.45e-11	1.50e-11	6.928.076
200023_s_at	4.45e-11	1.50e-11	6.928.033
219350_s_at	4.45e-11	1.50e-11	6.927.973
210026_s_at	4.46e-11	1.50e-11	6.927.413
203326_x_at	4.47e-11	1.51e-11	-6.927.124
215256_x_at	4.47e-11	1.51e-11	-6.927.017
203969_at	4.48e-11	1.51e-11	-6.926.603
211337_s_at	4.49e-11	1.51e-11	6.926.383
214683_s_at	4.52e-11	1.53e-11	-6.925.259
206572_x_at	4.55e-11	1.53e-11	6.924.332
221929_at	4.55e-11	1.54e-11	-6.924.109
206775_at	4.57e-11	1.54e-11	-6.923.463
204823_at	4.57e-11	1.54e-11	6.923.375
210540_s_at	4.60e-11	1.55e-11	6.922.289
207001_x_at	4.65e-11	1.57e-11	-6.920.746
221796_at	4.65e-11	1.57e-11	-6.920.696
206155_at	4.66e-11	1.57e-11	-6.920.476
214768_x_at	4.66e-11	1.57e-11	-6.920.367
215039_at	4.67e-11	1.58e-11	-6.920.037

202761_s_at	4.68e-11	1.58e-11	-6.919.743
201856_s_at	4.73e-11	1.60e-11	6.918.051
200884_at	4.74e-11	1.60e-11	6.917.712
215937_at	4.74e-11	1.60e-11	-6.917.566
218426_s_at	4.76e-11	1.61e-11	6.916.878
221464_at	4.80e-11	1.62e-11	-6.915.672
222047_s_at	4.80e-11	1.62e-11	6.915.455
201166_s_at	4.85e-11	1.64e-11	6.913.988
218288_s_at	4.85e-11	1.64e-11	-6.913.752
202962_at	4.89e-11	1.66e-11	-6.912.397
208845_at	4.90e-11	1.66e-11	6.912.339
207177_at	4.92e-11	1.66e-11	6.911.643
217404_s_at	4.92e-11	1.67e-11	6.911.454
206233_at	4.97e-11	1.68e-11	6.909.972
215884_s_at	5.03e-11	1.70e-11	6.907.867
207851_s_at	5.05e-11	1.71e-11	-6.907.489
215671_at	5.05e-11	1.71e-11	-6.907.288
209431_s_at	5.06e-11	1.71e-11	6.907.131
220864_s_at	5.07e-11	1.72e-11	6.906.735
201270_x_at	5.08e-11	1.72e-11	6.906.447
213203_at	5.08e-11	1.72e-11	6.906.411
218660_at	5.10e-11	1.73e-11	-6.905.689
222252_x_at	5.12e-11	1.73e-11	-6.905.087
216392_s_at	5.16e-11	1.75e-11	6.903.775
215479_at	5.17e-11	1.75e-11	-6.903.612
205348_s_at	5.18e-11	1.76e-11	6.903.183
212529_at	5.19e-11	1.76e-11	6.902.762
218645_at	5.24e-11	1.78e-11	690.133
213574_s_at	5.24e-11	1.78e-11	6.901.218
221151_at	5.28e-11	1.79e-11	-6.900.041
209383_at	5.28e-11	1.79e-11	6.899.921
201712_s_at	5.28e-11	1.79e-11	6.899.914
205059_s_at	5.31e-11	1.80e-11	6.899.184
211636_at	5.32e-11	1.80e-11	-6.898.827
204155_s_at	5.35e-11	1.82e-11	-689.789
209276_s_at	5.40e-11	1.83e-11	-6.896.515
208984_x_at	5.41e-11	1.84e-11	6.896.074
220965_s_at	5.43e-11	1.84e-11	-6.895.381
207666_x_at	5.44e-11	1.85e-11	6.895.218
210542_s_at	5.49e-11	1.86e-11	-6.893.688
212496_s_at	5.49e-11	1.87e-11	-6.893.651
213385_at	5.54e-11	1.88e-11	-6.892.254
201556_s_at	5.64e-11	1.91e-11	-6.889.529
219088_s_at	5.64e-11	1.92e-11	6.889.487

200922_at	5.64e-11	1.92e-11	6.889.418
207786_at	5.65e-11	1.92e-11	6.888.973
214300_s_at	5.65e-11	1.92e-11	-688.893
207459_x_at	5.68e-11	1.93e-11	-6.888.312
201907_x_at	5.69e-11	1.93e-11	688.788
203588_s_at	5.72e-11	1.94e-11	6.887.132
209499_x_at	5.73e-11	1.95e-11	-6.886.693
218751_s_at	5.77e-11	1.96e-11	-68.857
216571_at	5.78e-11	1.97e-11	-688.538
212115_at	5.80e-11	1.97e-11	688.475
203400_s_at	5.81e-11	1.98e-11	-6.884.556
220648_at	5.84e-11	1.99e-11	-6.883.619
209271_at	5.84e-11	1.99e-11	6.883.574
210544_s_at	5.89e-11	2.00e-11	-6.882.376
221892_at	5.89e-11	2.01e-11	-6.882.172
220834_at	5.93e-11	2.02e-11	-6.881.197
200696_s_at	6.05e-11	2.06e-11	-6.878.091
215268_at	6.15e-11	2.10e-11	-687.534
215410_at	6.17e-11	2.10e-11	-6.874.965
207304_at	6.21e-11	2.12e-11	6.873.695
208935_s_at	6.22e-11	2.12e-11	6.873.523
221964_at	6.23e-11	2.12e-11	6.873.334
207810_at	6.26e-11	2.14e-11	-6.872.371
206919_at	6.31e-11	2.15e-11	6.871.108
202581_at	6.38e-11	2.17e-11	6.869.517
211773_s_at	6.48e-11	2.21e-11	6.866.888
209520_s_at	6.62e-11	2.26e-11	68.636
210890_x_at	6.63e-11	2.26e-11	-6.863.352
208607_s_at	6.64e-11	2.27e-11	-6.862.998
221579_s_at	6.68e-11	2.28e-11	6.862.132
210098_s_at	6.75e-11	2.30e-11	6.860.435
204658_at	6.82e-11	2.33e-11	6.858.709
222270_at	6.88e-11	2.35e-11	68.573
211298_s_at	6.94e-11	2.37e-11	-6.855.867
212275_s_at	6.95e-11	2.37e-11	-685.582
205717_x_at	6.95e-11	2.37e-11	-6.855.605
208203_x_at	6.99e-11	2.39e-11	-6.854.686
51228_at	6.99e-11	2.39e-11	6.854.681
208968_s_at	7.01e-11	2.39e-11	6.854.276
212074_at	7.01e-11	2.40e-11	6.854.158
222338_x_at	7.04e-11	2.40e-11	6.853.582
209740_s_at	7.08e-11	2.42e-11	-685.263
213936_x_at	7.08e-11	2.42e-11	-6.852.514
215435_at	7.20e-11	2.46e-11	6.849.894

209539_at	7.22e-11	2.47e-11	-6.849.509
213894_at	7.23e-11	2.47e-11	6.849.184
221908_at	7.27e-11	2.49e-11	6.848.327
211687_x_at	7.34e-11	2.51e-11	-6.846.865
204829_s_at	7.37e-11	2.52e-11	-684.608
214779_s_at	7.46e-11	2.55e-11	6.844.291
214063_s_at	7.46e-11	2.55e-11	-684.427
216173_at	7.53e-11	2.57e-11	-684.279
211534_x_at	7.57e-11	2.59e-11	-6.841.736
204585_s_at	7.61e-11	2.60e-11	-6.841.022
47083_at	7.62e-11	2.61e-11	6.840.735
212763_at	7.65e-11	2.62e-11	6.840.111
209881_s_at	7.72e-11	2.64e-11	-6.838.651
203765_at	7.75e-11	2.65e-11	6.838.005
202979_s_at	7.87e-11	2.69e-11	6.835.647
207669_at	7.91e-11	2.71e-11	-6.834.677
217341_at	7.96e-11	2.73e-11	-683.378
207776_s_at	8.02e-11	2.75e-11	-6.832.588
210221_at	8.06e-11	2.76e-11	-6.831.615
217708_x_at	8.12e-11	2.78e-11	-6.830.412
217501_at	8.23e-11	2.82e-11	-6.828.433
203378_at	8.26e-11	2.83e-11	-6.827.815
214847_s_at	8.28e-11	2.84e-11	-6.827.342
217322_x_at	8.41e-11	2.88e-11	6.824.808
206716_at	8.42e-11	2.89e-11	-6.824.691
218797_s_at	8.44e-11	2.89e-11	68.243
209839_at	8.44e-11	2.89e-11	6.824.277
211681_s_at	8.48e-11	2.91e-11	-6.823.423
213066_at	8.49e-11	2.91e-11	-6.823.282
220369_at	8.51e-11	2.92e-11	-6.822.799
213755_s_at	8.56e-11	2.94e-11	-6.821.976
213370_s_at	8.62e-11	2.96e-11	6.820.836
221240_s_at	8.64e-11	2.97e-11	-6.820.331
214741_at	8.66e-11	2.97e-11	6.819.972
217179_x_at	8.70e-11	2.99e-11	-6.819.328
212502_at	8.70e-11	2.99e-11	6.819.238
201939_at	8.71e-11	2.99e-11	-681.899
65630_at	8.72e-11	3.00e-11	6.818.783
216244_at	8.75e-11	3.01e-11	-681.829
201812_s_at	8.81e-11	3.03e-11	6.817.077
215439_x_at	8.86e-11	3.04e-11	-6.816.306
212587_s_at	8.96e-11	3.08e-11	-6.814.444
221563_at	9.02e-11	3.10e-11	-6.813.363
208776_at	9.02e-11	3.10e-11	6.813.266

211614_at	9.04e-11	3.11e-11	-6.812.886
206892_at	9.05e-11	3.11e-11	-6.812.745
215604_x_at	9.09e-11	3.13e-11	-6.811.997
219450_at	9.17e-11	3.15e-11	-6.810.601
218835_at	9.19e-11	3.16e-11	-681.031
202991_at	9.22e-11	3.17e-11	6.809.702
210156_s_at	9.25e-11	3.18e-11	6.809.201
221245_s_at	9.31e-11	3.20e-11	6.808.084
217324_at	9.35e-11	3.22e-11	-6.807.385
214743_at	9.40e-11	3.23e-11	6.806.602
214596_at	9.46e-11	3.26e-11	6.805.568
206883_x_at	9.48e-11	3.26e-11	-6.805.145
221293_s_at	9.49e-11	3.27e-11	-6.805.048
214617_at	9.52e-11	3.28e-11	-680.445
201424_s_at	9.53e-11	3.28e-11	6.804.301
200653_s_at	9.54e-11	3.29e-11	-6.804.034
203147_s_at	9.61e-11	3.31e-11	-6.802.852
215912_at	9.61e-11	3.31e-11	-6.802.833
204312_x_at	9.70e-11	3.34e-11	680.131
216451_at	9.82e-11	3.38e-11	-679.947
205216_s_at	9.89e-11	3.41e-11	-6.798.264
217717_s_at	9.92e-11	3.42e-11	6.797.782
205297_s_at	9.93e-11	3.42e-11	-6.797.553
214688_at	9.94e-11	3.43e-11	-6.797.455
206206_at	9.94e-11	3.43e-11	-6.797.426
218642_s_at	1.00e-10	3.45e-11	6.796.452
216460_at	1.00e-10	3.46e-11	-6.796.109
221449_s_at	1.00e-10	3.46e-11	-6.795.944
214712_at	1.00e-10	3.46e-11	-6.795.872
202180_s_at	1.00e-10	3.47e-11	6.795.525
211612_s_at	1.01e-10	3.47e-11	-679.537
217971_at	1.01e-10	3.48e-11	6.794.951
210796_x_at	1.02e-10	3.53e-11	-6.792.861
206471_s_at	1.03e-10	3.57e-11	6.790.979
200079_s_at	1.04e-10	3.57e-11	6.790.712
212301_at	1.04e-10	3.58e-11	6.790.442
216899_s_at	1.04e-10	3.59e-11	6.789.818
218856_at	1.05e-10	3.62e-11	6.788.857
206486_at	1.05e-10	3.62e-11	-6.788.684
210002_at	1.05e-10	3.63e-11	-6.788.069
214035_x_at	1.05e-10	3.64e-11	-6.787.896
214980_at	1.05e-10	3.64e-11	-6.787.839
222204_s_at	1.08e-10	3.75e-11	-6.783.274
209992_at	1.09e-10	3.76e-11	6.782.581

214872_at	1.09e-10	3.77e-11	-6.782.315
217157_x_at	1.09e-10	3.78e-11	-6.781.894
201847_at	1.11e-10	3.84e-11	6.779.236
207423_s_at	1.12e-10	3.87e-11	-6.778.154
215230_x_at	1.12e-10	3.88e-11	677.781
213571_s_at	1.12e-10	3.88e-11	6.777.675
212341_at	1.13e-10	3.93e-11	6.775.788
215821_x_at	1.14e-10	3.93e-11	-6.775.664
208270_s_at	1.14e-10	3.94e-11	6.775.362
211157_at	1.15e-10	3.97e-11	-6.774.123
213710_s_at	1.16e-10	4.01e-11	-6.772.232
214566_at	1.19e-10	4.10e-11	-6.768.711
207131_x_at	1.19e-10	4.11e-11	-6.768.426
202521_at	1.19e-10	4.12e-11	676.797
219801_at	1.20e-10	4.16e-11	6.766.665
220802_at	1.21e-10	4.18e-11	-6.765.899
208602_x_at	1.21e-10	4.18e-11	-676.585
200692_s_at	1.21e-10	4.19e-11	6.765.367
218174_s_at	1.22e-10	4.21e-11	-6.764.561
218809_at	1.22e-10	4.21e-11	676.456
217753_s_at	1.22e-10	4.22e-11	6.764.384
207593_at	1.22e-10	4.22e-11	-6.764.337
218472_s_at	1.23e-10	4.25e-11	6.763.087
213442_x_at	1.23e-10	4.28e-11	-6.762.093
216780_at	1.24e-10	4.29e-11	-6.761.593
205038_at	1.24e-10	4.29e-11	-6.761.479
217315_s_at	1.25e-10	4.34e-11	-6.759.829
214265_at	1.27e-10	4.40e-11	-6.757.473
216168_at	1.28e-10	4.44e-11	-6.756.192
203191_at	1.28e-10	4.45e-11	-6.755.668
216245_at	1.30e-10	4.50e-11	-6.753.919
AFFX-r2-Ec-bioB-M_at	1.31e-10	4.53e-11	-6.752.788
211179_at	1.31e-10	4.54e-11	-6.752.634
212083_at	1.31e-10	4.54e-11	6.752.601
210658_s_at	1.32e-10	4.58e-11	6.751.006
210348_at	1.32e-10	4.60e-11	-6.750.533
205268_s_at	1.32e-10	4.60e-11	-6.750.372
206970_at	1.33e-10	4.61e-11	-6.750.221
211645_x_at	1.34e-10	4.67e-11	-6.747.936
211425_x_at	1.35e-10	4.68e-11	6.747.754
222147_s_at	1.35e-10	4.71e-11	6.746.811
217735_s_at	1.36e-10	4.72e-11	6.746.528
203579_s_at	1.36e-10	4.72e-11	6.746.376
213305_s_at	1.36e-10	4.72e-11	6.746.205

44696_at	1.36e-10	4.73e-11	6.746.092
206143_at	1.37e-10	4.76e-11	6.745.167
210677_at	1.37e-10	4.77e-11	6.744.748
212772_s_at	1.38e-10	4.79e-11	-6.743.894
34471_at	1.38e-10	4.82e-11	-674.313
218748_s_at	1.39e-10	4.83e-11	6.742.663
217482_at	1.39e-10	4.85e-11	-6.742.097
208911_s_at	1.39e-10	4.85e-11	6.741.996
202011_at	1.40e-10	4.87e-11	6.741.433
201572_x_at	1.41e-10	4.89e-11	-6.740.611
216734_s_at	1.41e-10	4.91e-11	-6.739.994
202508_s_at	1.43e-10	4.98e-11	6.737.654
206911_at	1.43e-10	4.98e-11	-6.737.639
220016_at	1.44e-10	5.01e-11	-67.369
219047_s_at	1.44e-10	5.01e-11	6.736.756
202319_at	1.44e-10	5.02e-11	6.736.432
206792_x_at	1.45e-10	5.04e-11	-6.735.935
216187_x_at	1.46e-10	5.09e-11	-6.734.433
211113_s_at	1.46e-10	5.10e-11	673.409
204960_at	1.46e-10	5.10e-11	-6.733.821
219854_at	1.47e-10	5.11e-11	673.352
206965_at	1.47e-10	5.13e-11	-6.733.115
215432_at	1.49e-10	5.20e-11	6.730.823
220892_s_at	1.49e-10	5.21e-11	-6.730.625
210632_s_at	1.49e-10	5.21e-11	-6.730.413
203179_at	1.50e-10	5.24e-11	-6.729.576
207348_s_at	1.50e-10	5.24e-11	6.729.472
217148_x_at	1.51e-10	5.27e-11	-6.728.819
201116_s_at	1.52e-10	5.29e-11	6.728.029
201967_at	1.53e-10	5.33e-11	-6.726.922
201170_s_at	1.53e-10	5.35e-11	-672.615
205901_at	1.54e-10	5.37e-11	-6.725.726
219772_s_at	1.54e-10	5.37e-11	6.725.705
210338_s_at	1.57e-10	5.47e-11	6.722.673
218484_at	1.57e-10	5.48e-11	6.722.373
204183_s_at	1.57e-10	5.50e-11	6.721.992
218109_s_at	1.58e-10	5.53e-11	6.721.078
205017_s_at	1.58e-10	5.53e-11	-6.720.944
212710_at	1.59e-10	5.56e-11	6.720.182
205704_s_at	1.59e-10	5.57e-11	6.719.946
219441_s_at	1.60e-10	5.59e-11	-6.719.338
217052_x_at	1.61e-10	5.61e-11	-6.718.643
219858_s_at	1.61e-10	5.64e-11	6.717.932
220511_s_at	1.62e-10	5.65e-11	-6.717.466

202047_s_at	1.62e-10	5.66e-11	6.717.293
220776_at	1.63e-10	5.69e-11	-6.716.479
210640_s_at	1.64e-10	5.75e-11	-6.714.595
208589_at	1.65e-10	5.78e-11	-6.713.919
208040_s_at	1.65e-10	5.78e-11	-6.713.794
204947_at	1.66e-10	5.81e-11	6.713.185
204327_s_at	1.66e-10	5.81e-11	6.712.958
208032_s_at	1.66e-10	5.83e-11	-6.712.594
206648_at	1.67e-10	5.85e-11	6.711.843
219203_at	1.67e-10	5.86e-11	6.711.654
201746_at	1.69e-10	5.93e-11	6.709.712
212913_at	1.70e-10	5.94e-11	6.709.402
215595_x_at	1.70e-10	5.96e-11	-6.708.931
216956_s_at	1.71e-10	6.00e-11	-6.707.756
214750_at	1.72e-10	6.03e-11	-670.712
216902_s_at	1.72e-10	6.03e-11	-6.707.061
203812_at	1.74e-10	6.10e-11	-6.705.261
205253_at	1.75e-10	6.15e-11	-6.703.936
221704_s_at	1.77e-10	6.20e-11	-6.702.562
208538_at	1.77e-10	6.22e-11	-6.702.039
205971_s_at	1.78e-10	6.25e-11	-6.701.388
211215_x_at	1.83e-10	6.42e-11	-6.697.093
213565_s_at	1.85e-10	6.48e-11	-6.695.492
217864_s_at	1.85e-10	6.50e-11	6.695.038
221005_s_at	1.86e-10	6.51e-11	6.694.704
220726_at	1.86e-10	6.53e-11	-6.694.278
205616_at	1.86e-10	6.53e-11	-6.694.245
205014_at	1.88e-10	6.62e-11	-6.692.186
212987_at	1.89e-10	6.63e-11	6.691.728
212989_at	1.89e-10	6.65e-11	669.139
217459_at	1.90e-10	6.68e-11	-6.690.631
208809_s_at	1.90e-10	6.68e-11	6.690.618
214153_at	1.93e-10	6.77e-11	-6.688.357
206623_at	1.93e-10	6.79e-11	-6.687.976
206054_at	1.95e-10	6.85e-11	-6.686.486
212260_at	1.96e-10	6.89e-11	668.557
217177_s_at	1.97e-10	6.91e-11	-6.685.148
218473_s_at	1.97e-10	6.92e-11	6.684.859
206430_at	1.97e-10	6.93e-11	-6.684.667
218731_s_at	1.98e-10	6.96e-11	6.684.065
203395_s_at	1.99e-10	6.98e-11	-668.352
204505_s_at	2.03e-10	7.13e-11	-6.680.208
217493_x_at	2.04e-10	7.18e-11	-6.679.096
218408_at	2.04e-10	7.19e-11	6.678.857

220282_at	2.06e-10	7.23e-11	-6.677.863
202788_at	2.06e-10	7.25e-11	6.677.456
216059_at	2.07e-10	7.29e-11	-667.654
208671_at	2.07e-10	7.29e-11	-6.676.453
200845_s_at	2.08e-10	7.32e-11	-6.675.957
206390_x_at	2.10e-10	7.37e-11	-6.674.694
216688_at	2.13e-10	7.49e-11	-6.672.258
202361_at	2.13e-10	7.49e-11	6.672.198
208262_x_at	2.13e-10	7.49e-11	-6.672.138
211583_x_at	2.14e-10	7.55e-11	-6.670.831
216965_x_at	2.15e-10	7.57e-11	-6.670.381
220097_s_at	2.16e-10	7.59e-11	6.669.929
210131_x_at	2.17e-10	7.64e-11	6.668.871
213449_at	2.18e-10	7.70e-11	6.667.755
217305_s_at	2.19e-10	7.72e-11	-6.667.214
222183_x_at	2.19e-10	7.73e-11	-6.667.111
214933_at	2.20e-10	7.76e-11	-6.666.479
213674_x_at	2.22e-10	7.82e-11	-6.665.166
220912_at	2.22e-10	7.83e-11	-6.664.989
207728_at	2.23e-10	7.85e-11	-6.664.498
207693_at	2.23e-10	7.86e-11	-6.664.388
220113_x_at	2.23e-10	7.87e-11	-6.664.145
215484_at	2.24e-10	7.89e-11	-6.663.699
220555_s_at	2.26e-10	7.97e-11	-6.662.235
213127_s_at	2.28e-10	8.04e-11	6.660.711
207349_s_at	2.28e-10	8.04e-11	-6.660.646
221645_s_at	2.29e-10	8.08e-11	6.659.936
216198_at	2.29e-10	8.10e-11	-6.659.612
209011_at	2.29e-10	8.10e-11	6.659.565
211075_s_at	2.32e-10	8.20e-11	6.657.571
216821_at	2.34e-10	8.27e-11	-665.621
210162_s_at	2.34e-10	8.27e-11	-6.656.104
221820_s_at	2.36e-10	8.34e-11	-6.654.735
202572_s_at	2.36e-10	8.35e-11	6.654.623
220696_at	2.38e-10	8.39e-11	-6.653.797
200865_at	2.38e-10	8.42e-11	-6.653.202
212546_s_at	2.41e-10	8.52e-11	6.651.408
201567_s_at	2.41e-10	8.52e-11	6.651.313
205160_at	2.41e-10	8.53e-11	-6.651.076
209354_at	2.41e-10	8.53e-11	-6.651.075
215699_x_at	2.44e-10	8.63e-11	6.649.198
209298_s_at	2.45e-10	8.66e-11	-6.648.633
208003_s_at	2.45e-10	8.67e-11	-6.648.584
221060_s_at	2.45e-10	8.67e-11	-6.648.577

210008_s_at	2.46e-10	8.70e-11	6.647.874
216138_at	2.47e-10	8.73e-11	-6.647.444
212219_at	2.51e-10	8.88e-11	6.644.647
218155_x_at	2.51e-10	8.90e-11	-6.644.326
217154_s_at	2.52e-10	8.92e-11	-6.643.975
217019_at	2.56e-10	9.06e-11	6.641.388
213095_x_at	2.57e-10	9.09e-11	-6.640.887
200648_s_at	2.57e-10	9.11e-11	6.640.531
200978_at	2.62e-10	9.28e-11	6.637.461
218749_s_at	2.69e-10	9.52e-11	-6.633.379
204234_s_at	2.70e-10	9.57e-11	663.258
200732_s_at	2.70e-10	9.58e-11	-6.632.348
203928_x_at	2.71e-10	9.61e-11	6.631.787
204571_x_at	2.71e-10	9.62e-11	6.631.721
201658_at	2.72e-10	9.64e-11	6.631.339
204543_at	2.74e-10	9.70e-11	-6.630.333
203184_at	2.74e-10	9.71e-11	-6.630.239
221646_s_at	2.76e-10	9.78e-11	-6.629.081
201257_x_at	2.76e-10	9.79e-11	6.628.896
211846_s_at	2.77e-10	9.81e-11	-6.628.462
202710_at	2.77e-10	9.82e-11	6.628.349
218750_at	2.77e-10	9.84e-11	6.628.051
222358_x_at	2.83e-10	1.00e-10	-6.624.909
212509_s_at	2.83e-10	1.01e-10	6.624.547
201691_s_at	2.84e-10	1.01e-10	6.624.203
208957_at	2.86e-10	1.01e-10	6.623.004
214898_x_at	2.87e-10	1.02e-10	-6.622.531
209818_s_at	2.88e-10	1.02e-10	6.621.773
221918_at	2.91e-10	1.03e-10	6.620.024
210306_at	2.92e-10	1.04e-10	6.619.499
206401_s_at	2.93e-10	1.04e-10	6.619.118
213204_at	2.96e-10	1.05e-10	6.617.148
210431_at	2.97e-10	1.05e-10	-6.616.968
221768_at	2.97e-10	1.05e-10	-6.616.776
220879_at	2.98e-10	1.06e-10	-6.616.058
203282_at	2.99e-10	1.06e-10	-6.615.353
204793_at	3.00e-10	1.07e-10	-6.615.126
207062_at	3.01e-10	1.07e-10	-6.614.254
200093_s_at	3.06e-10	1.09e-10	6.611.571
216524_x_at	3.07e-10	1.09e-10	-6.611.155
215336_at	3.07e-10	1.09e-10	-6.611.057
205847_at	3.08e-10	1.09e-10	-6.610.745
205152_at	3.09e-10	1.10e-10	-6.610.332
215245_x_at	3.09e-10	1.10e-10	6.610.231

201571_s_at	3.09e-10	1.10e-10	-6.610.205
208490_x_at	3.12e-10	1.11e-10	6.608.292
201084_s_at	3.13e-10	1.11e-10	660.794
211045_s_at	3.14e-10	1.12e-10	-660.754
215603_x_at	3.14e-10	1.12e-10	-660.744
215393_s_at	3.15e-10	1.12e-10	-6.606.778
200928_s_at	3.16e-10	1.12e-10	-6.606.442
215824_at	3.17e-10	1.13e-10	-6.605.779
200942_s_at	3.23e-10	1.15e-10	6.602.559
216031_x_at	3.23e-10	1.15e-10	-6.602.491
220418_at	3.25e-10	1.16e-10	-6.601.867
209329_x_at	3.25e-10	1.16e-10	6.601.566
216975_x_at	3.25e-10	1.16e-10	-6.601.388
207790_at	3.27e-10	1.17e-10	6.600.502
206036_s_at	3.28e-10	1.17e-10	-6.599.969
203453_at	3.29e-10	1.17e-10	-6.599.551
210020_x_at	3.30e-10	1.18e-10	-6.599.173
216356_x_at	3.30e-10	1.18e-10	-6.598.971
203947_at	3.33e-10	1.19e-10	6.597.706
212704_at	3.33e-10	1.19e-10	6.597.401
213285_at	3.33e-10	1.19e-10	-6.597.245
203911_at	3.34e-10	1.19e-10	6.596.912
206590_x_at	3.35e-10	1.20e-10	-6.596.287
205908_s_at	3.37e-10	1.20e-10	-6.595.521
211413_s_at	3.38e-10	1.21e-10	-6.594.816
204871_at	3.38e-10	1.21e-10	6.594.794
201790_s_at	3.39e-10	1.21e-10	6.594.498
206320_s_at	3.40e-10	1.21e-10	-6.594.084
200938_s_at	3.44e-10	1.23e-10	-6.592.168
208556_at	3.46e-10	1.23e-10	-6.591.302
210596_at	3.47e-10	1.24e-10	6.590.713
220290_at	3.49e-10	1.25e-10	-6.589.695
215038_s_at	3.50e-10	1.25e-10	6.589.242
213459_at	3.51e-10	1.25e-10	6.588.852
218777_at	3.51e-10	1.25e-10	6.588.681
210910_s_at	3.52e-10	1.26e-10	658.831
212411_at	3.52e-10	1.26e-10	6.587.933
208656_s_at	3.54e-10	1.26e-10	-6.587.308
220809_at	3.58e-10	1.28e-10	-6.585.464
209281_s_at	3.61e-10	1.29e-10	6.584.073
216656_at	3.67e-10	1.31e-10	-6.581.242
216750_at	3.69e-10	1.32e-10	-6.580.401
202302_s_at	3.70e-10	1.32e-10	6.579.658
203536_s_at	3.71e-10	1.33e-10	6.579.355

219622_at	3.72e-10	1.33e-10	-6.578.995
220057_at	3.72e-10	1.33e-10	6.578.946
200809_x_at	3.74e-10	1.34e-10	6.577.832
217824_at	3.76e-10	1.35e-10	6.577.043
36612_at	3.79e-10	1.36e-10	6.575.748
212174_at	3.81e-10	1.36e-10	-6.574.964
221080_s_at	3.82e-10	1.37e-10	-6.574.445
215592_at	3.84e-10	1.37e-10	-6.573.635
203529_at	3.85e-10	1.38e-10	6.573.044
215593_at	3.86e-10	1.38e-10	-6.572.848
207213_s_at	3.88e-10	1.39e-10	-6.571.764
203749_s_at	3.89e-10	1.39e-10	6.571.596
207312_at	3.89e-10	1.39e-10	-6.571.373
212522_at	3.93e-10	1.41e-10	-6.569.808
205146_x_at	3.93e-10	1.41e-10	6.569.682
218528_s_at	3.94e-10	1.41e-10	6.569.218
208879_x_at	3.97e-10	1.42e-10	6.568.036
208520_at	3.99e-10	1.43e-10	-6.567.269
215678_at	4.02e-10	1.44e-10	-6.565.961
50400_at	4.03e-10	1.44e-10	-6.565.665
202068_s_at	4.06e-10	1.46e-10	-6.564.284
202393_s_at	4.06e-10	1.46e-10	-6.564.271
214437_s_at	4.07e-10	1.46e-10	-6.563.957
215552_s_at	4.08e-10	1.46e-10	-6.563.467
216992_s_at	4.14e-10	1.49e-10	-656.094
219470_x_at	4.14e-10	1.49e-10	6.560.905
215907_at	4.14e-10	1.49e-10	-6.560.855
206688_s_at	4.15e-10	1.49e-10	6.560.548
204664_at	4.15e-10	1.49e-10	-6.560.503
221706_s_at	4.16e-10	1.49e-10	6.559.985
208895_s_at	4.19e-10	1.50e-10	6.558.872
215384_s_at	4.21e-10	1.51e-10	-6.558.137
215373_x_at	4.21e-10	1.51e-10	-6.557.974
209346_s_at	4.23e-10	1.52e-10	-655.724
204083_s_at	4.24e-10	1.52e-10	6.556.921
217011_at	4.25e-10	1.53e-10	-6.556.537
216114_at	4.25e-10	1.53e-10	-6.556.473
218490_s_at	4.28e-10	1.54e-10	6.555.469
220525_s_at	4.29e-10	1.54e-10	6.554.929
200867_at	4.32e-10	1.55e-10	6.553.691
215139_at	4.33e-10	1.56e-10	-6.553.357
221079_s_at	4.34e-10	1.56e-10	6.552.881
216789_at	4.35e-10	1.56e-10	-6.552.627
209990_s_at	4.36e-10	1.57e-10	6.552.053

216575_at	4.41e-10	1.59e-10	-6.550.238
212048_s_at	4.41e-10	1.59e-10	6.550.163
206600_s_at	4.42e-10	1.59e-10	-6.549.817
205601_s_at	4.43e-10	1.59e-10	-6.549.489
215546_at	4.45e-10	1.60e-10	-6.548.713
212574_x_at	4.45e-10	1.60e-10	-6.548.607
201435_s_at	4.47e-10	1.61e-10	6.547.933
207370_at	4.47e-10	1.61e-10	-6.547.824
216557_x_at	4.49e-10	1.62e-10	-6.547.135
217586_x_at	4.50e-10	1.62e-10	-6.546.567
204397_at	4.51e-10	1.63e-10	-6.546.246
203153_at	4.52e-10	1.63e-10	-6.546.099
214859_at	4.52e-10	1.63e-10	-6.546.051
218529_at	4.52e-10	1.63e-10	6.545.909
217360_x_at	4.59e-10	1.65e-10	-6.543.482
217473_x_at	4.59e-10	1.65e-10	-6.543.431
211352_s_at	4.62e-10	1.66e-10	6.542.339
221782_at	4.69e-10	1.69e-10	6.539.936
211852_s_at	4.69e-10	1.69e-10	6.539.866
218697_at	4.70e-10	1.69e-10	6.539.399
203814_s_at	4.71e-10	1.70e-10	-6.539.121
210166_at	4.71e-10	1.70e-10	6.538.862
219839_x_at	4.73e-10	1.71e-10	-653.834
203122_at	4.73e-10	1.71e-10	6.538.307
206642_at	4.76e-10	1.72e-10	-6.537.339
210344_at	4.77e-10	1.72e-10	-6.536.933
218805_at	4.80e-10	1.73e-10	-6.535.885
209888_s_at	4.81e-10	1.73e-10	-6.535.609
201487_at	4.82e-10	1.74e-10	6.535.004
221155_x_at	4.82e-10	1.74e-10	-6.534.937
204507_s_at	4.84e-10	1.75e-10	6.534.526
207357_s_at	4.84e-10	1.75e-10	6.534.277
215644_at	4.85e-10	1.75e-10	-6.534.018
221400_at	5.01e-10	1.81e-10	-6.528.617
217949_s_at	5.01e-10	1.81e-10	-6.528.599
217344_at	5.01e-10	1.81e-10	6.528.464
202795_x_at	5.04e-10	1.82e-10	6.527.677
214131_at	5.04e-10	1.82e-10	-6.527.579
216060_s_at	5.05e-10	1.83e-10	-6.527.199
201701_s_at	5.06e-10	1.83e-10	-6.526.958
213169_at	5.07e-10	1.83e-10	-6.526.601
201224_s_at	5.10e-10	1.84e-10	-6.525.691
213191_at	5.10e-10	1.84e-10	6.525.583
220851_at	5.12e-10	1.85e-10	-6.524.727

213016_at	5.13e-10	1.86e-10	6.524.472
221587_s_at	5.17e-10	1.87e-10	6.523.285
222282_at	5.17e-10	1.87e-10	-6.523.188
219237_s_at	5.23e-10	1.89e-10	-6.521.235
201945_at	5.23e-10	1.89e-10	-6.521.199
200798_x_at	5.24e-10	1.90e-10	-6.520.945
215553_x_at	5.25e-10	1.90e-10	-6.520.599
217611_at	5.25e-10	1.90e-10	-6.520.541
202583_s_at	5.26e-10	1.90e-10	6.520.326
213174_at	5.26e-10	1.90e-10	6.520.273
221113_s_at	5.27e-10	1.91e-10	-6.519.967
215583_at	5.27e-10	1.91e-10	-6.519.964
206963_s_at	5.28e-10	1.91e-10	-651.961
207146_at	5.28e-10	1.91e-10	-6.519.583
203686_at	5.28e-10	1.91e-10	-6.519.424
201040_at	5.30e-10	1.92e-10	-6.518.756
218981_at	5.33e-10	1.93e-10	6.517.856
210442_at	5.33e-10	1.93e-10	-6.517.822
220814_at	5.35e-10	1.94e-10	-6.517.204
219972_s_at	5.38e-10	1.95e-10	6.516.374
221963_x_at	5.41e-10	1.96e-10	6.515.265
206870_at	5.44e-10	1.97e-10	-6.514.591
214469_at	5.47e-10	1.98e-10	6.513.527
214296_x_at	5.55e-10	2.01e-10	-6.511.111
205607_s_at	5.57e-10	2.02e-10	651.049
213915_at	5.63e-10	2.04e-10	-6.508.748
221831_at	5.63e-10	2.04e-10	-6.508.651
220123_at	5.63e-10	2.04e-10	6.508.615
209618_at	5.65e-10	2.05e-10	6.508.019
212705_x_at	5.67e-10	2.06e-10	-6.507.614
214535_s_at	5.68e-10	2.06e-10	-6.507.329
208025_s_at	5.68e-10	2.06e-10	6.507.295
211536_x_at	5.68e-10	2.06e-10	6.507.281
213582_at	5.68e-10	2.06e-10	6.507.153
218884_s_at	5.73e-10	2.08e-10	6.505.593
212357_at	5.75e-10	2.09e-10	6.505.141
201192_s_at	5.75e-10	2.09e-10	-6.505.048
214490_at	5.78e-10	2.10e-10	-6.504.114
204550_x_at	5.79e-10	2.10e-10	-6.503.962
201235_s_at	5.79e-10	2.10e-10	-6.503.762
207344_at	5.81e-10	2.11e-10	-6.503.323
212359_s_at	5.82e-10	2.12e-10	-6.502.926
201541_s_at	5.85e-10	2.13e-10	-65.021
207278_s_at	5.85e-10	2.13e-10	-6.502.092

205452_at	5.91e-10	2.15e-10	6.500.369
215560_x_at	5.92e-10	2.15e-10	-6.500.015
202613_at	5.92e-10	2.15e-10	-6.499.965
201887_at	5.94e-10	2.16e-10	-6.499.573
205099_s_at	5.95e-10	2.16e-10	-6.499.264
208524_at	5.95e-10	2.16e-10	-6.499.148
40837_at	6.02e-10	2.19e-10	-6.497.212
220435_at	6.03e-10	2.19e-10	6.497.016
219832_s_at	6.03e-10	2.19e-10	-6.496.936
208814_at	6.03e-10	2.19e-10	6.496.931
210275_s_at	6.07e-10	2.21e-10	-6.495.829
209344_at	6.07e-10	2.21e-10	649.573
210126_at	6.09e-10	2.22e-10	-649.521
213125_at	6.18e-10	2.25e-10	6.492.613
207699_at	6.19e-10	2.25e-10	-6.492.533
213924_at	6.32e-10	2.30e-10	6.488.956
215333_x_at	6.34e-10	2.31e-10	-6.488.401
209762_x_at	6.34e-10	2.31e-10	6.488.331
208768_x_at	6.35e-10	2.31e-10	6.488.297
200906_s_at	6.36e-10	2.32e-10	6.487.948
204251_s_at	6.36e-10	2.32e-10	-6.487.762
200636_s_at	6.37e-10	2.32e-10	6.487.611
204392_at	6.38e-10	2.33e-10	6.487.215
219489_s_at	6.39e-10	2.33e-10	-6.487.053
210754_s_at	6.46e-10	2.36e-10	6.485.141
220839_at	6.47e-10	2.36e-10	-6.484.833
208132_x_at	6.48e-10	2.36e-10	6.484.592
219728_at	6.49e-10	2.37e-10	-6.484.379
216063_at	6.52e-10	2.38e-10	-6.483.595
217444_at	6.53e-10	2.38e-10	-6.483.307
208211_s_at	6.54e-10	2.39e-10	-6.483.146
215904_at	6.56e-10	2.40e-10	-6.482.438
218275_at	6.57e-10	2.40e-10	-648.227
214894_x_at	6.58e-10	2.40e-10	-6.481.897
207760_s_at	6.58e-10	2.40e-10	6.481.892
215183_at	6.60e-10	2.41e-10	-6.481.409
213812_s_at	6.62e-10	2.42e-10	-6.480.816
207805_s_at	6.64e-10	2.43e-10	6.480.299
219641_at	6.69e-10	2.45e-10	6.479.059
213968_at	6.70e-10	2.45e-10	-6.478.881
207652_s_at	6.74e-10	2.46e-10	-6.477.831
221052_at	6.76e-10	2.47e-10	6.477.412
215987_at	6.77e-10	2.47e-10	-6.477.153
213965_s_at	6.81e-10	2.49e-10	-6.476.208

209435_s_at	6.89e-10	2.52e-10	6.474.098
214629_x_at	6.91e-10	2.53e-10	-6.473.588
220180_at	6.94e-10	2.54e-10	-6.472.826
214163_at	7.00e-10	2.56e-10	6.471.375
218019_s_at	7.02e-10	2.57e-10	6.471.061
205321_at	7.02e-10	2.57e-10	6.470.867
209337_at	7.04e-10	2.58e-10	6.470.375
206174_s_at	7.06e-10	2.58e-10	6.470.038
210955_at	7.07e-10	2.59e-10	-6.469.843
205111_s_at	7.09e-10	2.59e-10	6.469.286
220056_at	7.09e-10	2.60e-10	646.922
212733_at	7.09e-10	2.60e-10	6.469.211
200846_s_at	7.09e-10	2.60e-10	6.469.137
215142_at	7.15e-10	2.62e-10	-6.467.847
210793_s_at	7.15e-10	2.62e-10	6.467.716
202004_x_at	7.17e-10	2.63e-10	6.467.317
210536_s_at	7.17e-10	2.63e-10	-6.467.221
204919_at	7.28e-10	2.67e-10	-6.464.788
208944_at	7.29e-10	2.67e-10	6.464.393
202418_at	7.34e-10	2.69e-10	646.322
211567_at	7.38e-10	2.70e-10	-6.462.457
203019_x_at	7.41e-10	2.72e-10	6.461.685
205224_at	7.44e-10	2.73e-10	6.461.113
212202_s_at	7.44e-10	2.73e-10	6.461.003
216624_s_at	7.46e-10	2.74e-10	-646.049
206581_at	7.48e-10	2.74e-10	-6.460.122
207441_at	7.51e-10	2.75e-10	-6.459.442
215027_at	7.54e-10	2.77e-10	-6.458.713
209505_at	7.57e-10	2.78e-10	-6.458.087
210117_at	7.64e-10	2.80e-10	6.456.537
211217_s_at	7.65e-10	2.81e-10	-6.456.349
214974_x_at	7.70e-10	2.82e-10	6.455.233
211228_s_at	7.74e-10	2.84e-10	645.436
208518_s_at	7.82e-10	2.87e-10	-6.452.645
215949_x_at	7.85e-10	2.88e-10	-6.451.832
204693_at	7.89e-10	2.90e-10	6.451.049
215098_at	7.98e-10	2.93e-10	-6.449.167
217952_x_at	8.07e-10	2.96e-10	6.447.294
201137_s_at	8.10e-10	2.98e-10	-6.446.578
207164_s_at	8.10e-10	2.98e-10	6.446.531
208831_x_at	8.11e-10	2.98e-10	6.446.467
202783_at	8.11e-10	2.98e-10	-6.446.317
213034_at	8.11e-10	2.98e-10	-6.446.295
221528_s_at	8.19e-10	3.01e-10	6.444.606

206772_at	8.24e-10	3.03e-10	644.365
221930_at	8.25e-10	3.03e-10	-6.443.414
208543_at	8.27e-10	3.04e-10	-6.442.955
222218_s_at	8.47e-10	3.11e-10	-6.439.052
213421_x_at	8.51e-10	3.13e-10	6.438.317
202875_s_at	8.52e-10	3.13e-10	6.438.087
210968_s_at	8.56e-10	3.15e-10	-6.437.165
AFFX-r2-Ec-bioC-5_at	8.56e-10	3.15e-10	-6.437.101
219986_s_at	8.61e-10	3.17e-10	-6.436.251
212252_at	8.68e-10	3.19e-10	-6.434.929
205305_at	8.71e-10	3.21e-10	-6.434.203
218547_at	8.74e-10	3.22e-10	6.433.691
208588_at	8.74e-10	3.22e-10	-643.369
211893_x_at	8.76e-10	3.23e-10	-6.433.221
203662_s_at	8.82e-10	3.25e-10	-6.432.111
202051_s_at	8.83e-10	3.25e-10	643.195
206284_x_at	8.86e-10	3.26e-10	6.431.245
215445_x_at	8.92e-10	3.28e-10	-6.430.211
209669_s_at	9.01e-10	3.32e-10	6.428.467
217255_at	9.10e-10	3.35e-10	-6.426.788
32062_at	9.21e-10	3.39e-10	6.424.825
220595_at	9.22e-10	3.40e-10	-6.424.624
210357_s_at	9.23e-10	3.40e-10	6.424.434
209152_s_at	9.25e-10	3.41e-10	6.424.074
215934_at	9.31e-10	3.43e-10	-6.422.893
206964_at	9.32e-10	3.43e-10	6.422.755
220208_at	9.34e-10	3.44e-10	-6.422.376
205645_at	9.38e-10	3.46e-10	-6.421.516
215075_s_at	9.38e-10	3.46e-10	6.421.487
202035_s_at	9.53e-10	3.52e-10	-6.418.891
31799_at	9.53e-10	3.52e-10	6.418.863
205536_at	9.56e-10	3.53e-10	-6.418.332
220727_at	9.61e-10	3.55e-10	-6.417.476
203068_at	9.67e-10	3.57e-10	6.416.409
220560_at	9.71e-10	3.58e-10	-6.415.691
211505_s_at	9.72e-10	3.59e-10	6.415.557
214084_x_at	9.73e-10	3.59e-10	-6.415.257
201988_s_at	9.76e-10	3.60e-10	-6.414.719
208420_x_at	9.78e-10	3.61e-10	6.414.417
214100_x_at	9.86e-10	3.64e-10	6.412.993
216884_at	9.93e-10	3.67e-10	-6.411.788
218952_at	9.95e-10	3.67e-10	6.411.562
216509_x_at	1.00e-09	3.71e-10	-6.410.042
221124_s_at	1.03e-09	3.82e-10	-6.404.979

220090_at	1.05e-09	3.87e-10	-6.402.752
206134_at	1.05e-09	3.89e-10	6.401.995
200860_s_at	1.06e-09	3.90e-10	6.401.622
201357_s_at	1.07e-09	3.94e-10	6.400.007
202026_at	1.07e-09	3.94e-10	-639.993
221235_s_at	1.07e-09	3.96e-10	6.399.222
206875_s_at	1.07e-09	3.97e-10	6.398.725
213378_s_at	1.09e-09	4.02e-10	6.396.668
202945_at	1.09e-09	4.02e-10	-6.396.532
206098_at	1.09e-09	4.03e-10	6.395.948
222002_at	1.09e-09	4.04e-10	-6.395.814
218865_at	1.10e-09	4.05e-10	-6.395.177
213093_at	1.10e-09	4.08e-10	6.394.064
201466_s_at	1.12e-09	4.14e-10	-6.391.562
203531_at	1.12e-09	4.14e-10	6.391.483
207482_at	1.13e-09	4.17e-10	-6.390.377
218696_at	1.13e-09	4.19e-10	6.389.779
206677_at	1.13e-09	4.19e-10	-6.389.581
210886_x_at	1.14e-09	4.21e-10	-6.388.861
216919_at	1.15e-09	4.25e-10	-6.387.445
206338_at	1.15e-09	4.25e-10	-6.387.096
201095_at	1.15e-09	4.27e-10	6.386.665
216459_x_at	1.15e-09	4.27e-10	-6.386.605
211369_at	1.16e-09	4.30e-10	-638.536
211509_s_at	1.16e-09	4.31e-10	-6.384.988
206238_s_at	1.17e-09	4.34e-10	-6.383.835
212619_at	1.17e-09	4.35e-10	6.383.205
214777_at	1.18e-09	4.36e-10	-6.383.036
214809_at	1.19e-09	4.41e-10	-6.380.965
65718_at	1.19e-09	4.42e-10	-6.380.818
202728_s_at	1.19e-09	4.43e-10	-6.380.521
220448_at	1.19e-09	4.43e-10	-6.380.433
220243_at	1.20e-09	4.44e-10	-6.380.065
206160_at	1.20e-09	4.44e-10	-6.379.782
214603_at	1.20e-09	4.45e-10	6.379.741
205986_at	1.20e-09	4.46e-10	-6.379.175
210129_s_at	1.20e-09	4.47e-10	-6.378.798
205877_s_at	1.21e-09	4.48e-10	-6.378.418
205203_at	1.21e-09	4.49e-10	-6.378.089
211868_x_at	1.21e-09	4.50e-10	-6.377.692
207564_x_at	1.22e-09	4.51e-10	6.377.196
208585_at	1.22e-09	4.52e-10	-6.377.029
204828_at	1.22e-09	4.53e-10	6.376.742
212564_at	1.22e-09	4.53e-10	6.376.688

216172_at	1.22e-09	4.53e-10	-637.664
208933_s_at	1.22e-09	4.53e-10	6.376.635
219982_s_at	1.22e-09	4.54e-10	6.376.411
220227_at	1.22e-09	4.54e-10	-6.376.313
211953_s_at	1.22e-09	4.54e-10	6.376.244
203952_at	1.23e-09	4.56e-10	637.561
216316_x_at	1.23e-09	4.58e-10	-6.374.875
210786_s_at	1.23e-09	4.59e-10	-6.374.502
220045_at	1.24e-09	4.60e-10	-6.374.145
207950_s_at	1.25e-09	4.65e-10	-6.372.097
221211_s_at	1.25e-09	4.65e-10	6.372.066
214771_x_at	1.26e-09	4.67e-10	-6.371.382
209243_s_at	1.26e-09	4.69e-10	6.370.872
203331_s_at	1.26e-09	4.69e-10	-6.370.802
220808_at	1.26e-09	4.70e-10	-6.370.369
218526_s_at	1.27e-09	4.71e-10	6.369.965
200097_s_at	1.27e-09	4.72e-10	6.369.726
217688_at	1.27e-09	4.73e-10	-6.369.432
203357_s_at	1.27e-09	4.73e-10	6.369.219
202730_s_at	1.28e-09	4.77e-10	6.368.089
222159_at	1.29e-09	4.80e-10	-6.367.083
216158_at	1.30e-09	4.86e-10	-6.364.996
202093_s_at	1.31e-09	4.86e-10	636.468
213999_at	1.31e-09	4.87e-10	-6.364.342
212649_at	1.31e-09	4.89e-10	6.363.735
41644_at	1.32e-09	4.91e-10	-6.363.269
204144_s_at	1.33e-09	4.94e-10	-6.362.107
205569_at	1.33e-09	4.94e-10	6.362.042
221844_x_at	1.33e-09	4.96e-10	-6.361.445
207433_at	1.34e-09	5.01e-10	-6.359.755
212562_s_at	1.34e-09	5.01e-10	-6.359.707
214951_at	1.34e-09	5.01e-10	-6.359.604
202533_s_at	1.35e-09	5.02e-10	6.359.545
214860_at	1.35e-09	5.02e-10	-6.359.506
212441_at	1.35e-09	5.03e-10	635.895
213750_at	1.35e-09	5.04e-10	-6.358.674
208265_at	1.35e-09	5.05e-10	-6.358.496
217219_at	1.36e-09	5.06e-10	-6.358.164
210388_at	1.36e-09	5.07e-10	-6.357.625
208176_at	1.38e-09	5.16e-10	-635.489
206064_s_at	1.38e-09	5.17e-10	-6.354.572
207863_at	1.39e-09	5.17e-10	-6.354.393
218963_s_at	1.39e-09	5.19e-10	635.396
220566_at	1.39e-09	5.20e-10	-6.353.448

219565_at	1.40e-09	5.21e-10	6.353.074
201205_at	1.40e-09	5.24e-10	-6.352.347
218971_s_at	1.40e-09	5.24e-10	6.352.309
211252_x_at	1.41e-09	5.28e-10	-6.350.974
209759_s_at	1.42e-09	5.29e-10	-6.350.497
222207_x_at	1.42e-09	5.32e-10	-6.349.798
203551_s_at	1.44e-09	5.37e-10	6.347.965
205293_x_at	1.44e-09	5.38e-10	-6.347.882
203065_s_at	1.44e-09	5.40e-10	6.347.284
203297_s_at	1.45e-09	5.42e-10	6.346.686
211794_at	1.46e-09	5.47e-10	-6.345.083
220354_at	1.47e-09	5.49e-10	6.344.426
215926_x_at	1.47e-09	5.49e-10	-6.344.374
219508_at	1.47e-09	5.51e-10	6.343.781
213844_at	1.48e-09	5.52e-10	6.343.534
203620_s_at	1.49e-09	5.56e-10	-6.342.238
221429_x_at	1.50e-09	5.61e-10	-634.073
218511_s_at	1.50e-09	5.63e-10	-6.340.323
213268_at	1.51e-09	5.64e-10	6.339.849
215681_at	1.51e-09	5.65e-10	-6.339.718
221254_s_at	1.51e-09	5.67e-10	-6.338.958
221156_x_at	1.52e-09	5.68e-10	-6.338.616
210048_at	1.53e-09	5.74e-10	6.336.975
215983_s_at	1.54e-09	5.76e-10	-6.336.433
215848_at	1.54e-09	5.76e-10	-6.336.345
202385_s_at	1.54e-09	5.77e-10	6.335.911
204717_s_at	1.54e-09	5.79e-10	633.557
201134_x_at	1.55e-09	5.81e-10	6.334.899
217082_at	1.55e-09	5.82e-10	-6.334.679
216963_s_at	1.55e-09	5.82e-10	-6.334.647
209959_at	1.55e-09	5.82e-10	-6.334.524
213689_x_at	1.56e-09	5.85e-10	-6.333.701
206071_s_at	1.56e-09	5.86e-10	-6.333.558
216834_at	1.56e-09	5.86e-10	-6.333.558
217445_s_at	1.59e-09	5.95e-10	6.330.811
209742_s_at	1.59e-09	5.97e-10	-6.330.187
202625_at	1.60e-09	6.00e-10	6.329.503
217074_at	1.60e-09	6.02e-10	-632.901
216875_x_at	1.60e-09	6.02e-10	-6.328.957
218581_at	1.60e-09	6.02e-10	6.328.951
211218_at	1.60e-09	6.02e-10	-6.328.881
210094_s_at	1.60e-09	6.02e-10	6.328.833
219159_s_at	1.61e-09	6.03e-10	-6.328.664
213064_at	1.62e-09	6.06e-10	-6.327.731

AFFX-r2-Ec-bioB-3_at	1.63e-09	6.11e-10	-6.326.497
216251_s_at	1.63e-09	6.11e-10	6.326.391
214077_x_at	1.63e-09	6.12e-10	-6.326.242
200647_x_at	1.64e-09	6.18e-10	6.324.599
213575_at	1.64e-09	6.18e-10	-6.324.598
216028_at	1.65e-09	6.18e-10	6.324.475
217725_x_at	1.65e-09	6.19e-10	6.324.242
209529_at	1.65e-09	6.20e-10	6.323.878
204873_at	1.66e-09	6.24e-10	63.229
203646_at	1.67e-09	6.27e-10	-6.321.974
216841_s_at	1.67e-09	6.28e-10	6.321.876
213076_at	1.68e-09	6.31e-10	632.102
202312_s_at	1.68e-09	6.32e-10	-6.320.703
212910_at	1.69e-09	6.35e-10	6.320.047
222192_s_at	1.69e-09	6.36e-10	6.319.782
215780_s_at	1.69e-09	6.37e-10	6.319.537
208066_s_at	1.69e-09	6.37e-10	6.319.322
216496_s_at	1.70e-09	6.40e-10	-6.318.734
47553_at	1.71e-09	6.42e-10	6.318.074
212457_at	1.73e-09	6.49e-10	6.316.173
214896_at	1.73e-09	6.51e-10	-6.315.704
221161_at	1.73e-09	6.53e-10	-6.315.303
201713_s_at	1.76e-09	6.62e-10	6.312.988
64064_at	1.76e-09	6.63e-10	-6.312.765
212770_at	1.76e-09	6.63e-10	6.312.763
209789_at	1.76e-09	6.63e-10	-6.312.558
208484_at	1.77e-09	6.66e-10	-6.312.019
37796_at	1.77e-09	6.67e-10	-6.311.549
212938_at	1.79e-09	6.73e-10	-6.310.251
208627_s_at	1.79e-09	6.75e-10	6.309.651
203273_s_at	1.79e-09	6.75e-10	630.961
208410_x_at	1.80e-09	6.79e-10	-6.308.591
204346_s_at	1.81e-09	6.81e-10	6.308.046
204863_s_at	1.81e-09	6.82e-10	-6.307.874
211950_at	1.81e-09	6.82e-10	-6.307.806
203093_s_at	1.82e-09	6.86e-10	6.306.872
201062_at	1.83e-09	6.88e-10	-6.306.395
222262_s_at	1.83e-09	6.89e-10	6.306.248
207259_at	1.83e-09	6.90e-10	-6.305.932
207866_at	1.83e-09	6.91e-10	-6.305.741
221735_at	1.83e-09	6.91e-10	6.305.707
32137_at	1.83e-09	6.92e-10	6.305.453
203937_s_at	1.85e-09	6.99e-10	-6.303.825
202718_at	1.87e-09	7.07e-10	-6.301.902

210663_s_at	1.89e-09	7.12e-10	-6.300.743
204642_at	1.89e-09	7.12e-10	-6.300.595
203173_s_at	1.89e-09	7.13e-10	-6.300.448
219642_s_at	1.89e-09	7.13e-10	-630.032
209146_at	1.89e-09	7.15e-10	-6.299.893
210958_s_at	1.89e-09	7.16e-10	-6.299.796
202270_at	1.90e-09	7.16e-10	-6.299.663
212585_at	1.91e-09	7.21e-10	6.298.531
208388_at	1.91e-09	7.23e-10	-629.809
74694_s_at	1.93e-09	7.30e-10	6.296.496
215995_x_at	1.93e-09	7.31e-10	-6.296.164
215996_at	1.94e-09	7.31e-10	-6.296.132
214402_s_at	1.94e-09	7.33e-10	6.295.691
207119_at	1.95e-09	7.38e-10	-6.294.626
211845_at	1.95e-09	7.38e-10	-6.294.565
206990_at	1.97e-09	7.46e-10	-629.281
216463_at	1.98e-09	7.48e-10	-6.292.224
204256_at	1.98e-09	7.49e-10	-6.292.137
210791_s_at	1.98e-09	7.50e-10	-6.291.969
200087_s_at	1.98e-09	7.50e-10	6.291.864
212960_at	2.00e-09	7.56e-10	-6.290.498
219874_at	2.00e-09	7.58e-10	6.290.075
200925_at	2.01e-09	7.60e-10	6.289.656
210469_at	2.01e-09	7.60e-10	6.289.613
216121_at	2.04e-09	7.74e-10	-6.286.582
214054_at	2.06e-09	7.78e-10	-6.285.673
210763_x_at	2.06e-09	7.80e-10	-628.533
202010_s_at	2.06e-09	7.80e-10	6.285.318
209440_at	2.06e-09	7.80e-10	6.285.281
216597_at	2.06e-09	7.82e-10	-6.284.848
206219_s_at	2.09e-09	7.90e-10	-6.283.076
212914_at	2.09e-09	7.91e-10	-6.282.815
213337_s_at	2.11e-09	7.99e-10	-6.281.129
205659_at	2.11e-09	7.99e-10	-6.281.082
207591_s_at	2.11e-09	8.01e-10	-6.280.633
205669_at	2.11e-09	8.01e-10	-6.280.633
211837_s_at	2.12e-09	8.03e-10	-6.280.261
207238_s_at	2.12e-09	8.04e-10	-628.018
217802_s_at	2.12e-09	8.04e-10	6.280.052
205427_at	2.12e-09	8.05e-10	6.279.977
205726_at	2.13e-09	8.07e-10	-6.279.445
213839_at	2.13e-09	8.09e-10	-6.279.141
203970_s_at	2.14e-09	8.11e-10	-6.278.709
202515_at	2.14e-09	8.11e-10	6.278.643

221197_s_at	2.14e-09	8.12e-10	-6.278.385
220377_at	2.15e-09	8.16e-10	-6.277.556
204620_s_at	2.15e-09	8.17e-10	627.744
216159_s_at	2.15e-09	8.17e-10	-6.277.339
205758_at	2.16e-09	8.19e-10	-6.277.019
215953_at	2.18e-09	8.27e-10	-6.275.276
202316_x_at	2.19e-09	8.31e-10	6.274.609
221872_at	2.20e-09	8.36e-10	6.273.554
205272_s_at	2.20e-09	8.36e-10	-6.273.531
217872_at	2.21e-09	8.40e-10	6.272.733
204182_s_at	2.21e-09	8.41e-10	-6.272.443
209597_s_at	2.21e-09	8.41e-10	-6.272.414
212135_s_at	2.22e-09	8.42e-10	627.227
215353_at	2.22e-09	8.42e-10	-6.272.217
220490_at	2.22e-09	8.45e-10	-6.271.607
209173_at	2.23e-09	8.46e-10	6.271.469
208709_s_at	2.25e-09	8.56e-10	626.949
220676_at	2.26e-09	8.59e-10	-6.268.902
218217_at	2.27e-09	8.62e-10	6.268.222
200894_s_at	2.28e-09	8.67e-10	6.267.384
208762_at	2.28e-09	8.67e-10	6.267.337
207657_x_at	2.28e-09	8.68e-10	6.267.213
213825_at	2.28e-09	8.68e-10	-626.716
218142_s_at	2.28e-09	8.68e-10	-6.267.116
220831_at	2.28e-09	8.68e-10	-6.267.031
213136_at	2.28e-09	8.69e-10	6.267.025
201454_s_at	2.30e-09	8.75e-10	6.265.858
207311_at	2.30e-09	8.77e-10	-6.265.438
205162_at	2.31e-09	8.80e-10	6.264.882
207511_s_at	2.31e-09	8.81e-10	6.264.568
216397_s_at	2.32e-09	8.82e-10	6.264.384
213593_s_at	2.36e-09	8.97e-10	-6.261.495
214902_x_at	2.36e-09	8.99e-10	-6.261.262
218597_s_at	2.36e-09	9.00e-10	6.261.028
202327_s_at	2.38e-09	9.07e-10	-6.259.762
222197_s_at	2.39e-09	9.11e-10	-6.258.926
203470_s_at	2.39e-09	9.12e-10	-625.881
207350_s_at	2.39e-09	9.12e-10	6.258.728
210179_at	2.40e-09	9.13e-10	-6.258.491
211211_x_at	2.40e-09	9.16e-10	-6.258.063
215044_s_at	2.40e-09	9.16e-10	6.257.937
216949_s_at	2.43e-09	9.28e-10	-625.577
215700_x_at	2.44e-09	9.31e-10	-6.255.261
213607_x_at	2.45e-09	9.35e-10	-6.254.578

218236_s_at	2.46e-09	9.36e-10	6.254.252
216530_at	2.46e-09	9.38e-10	-6.253.993
216979_at	2.47e-09	9.42e-10	-6.253.225
211205_x_at	2.47e-09	9.44e-10	6.252.971
201267_s_at	2.48e-09	9.46e-10	6.252.579
201510_at	2.49e-09	9.49e-10	6.252.076
213613_s_at	2.50e-09	9.54e-10	-6.251.167
213882_at	2.50e-09	9.55e-10	6.250.978
214038_at	2.51e-09	9.60e-10	6.250.107
217928_s_at	2.52e-09	9.63e-10	6.249.451
207999_s_at	2.53e-09	9.65e-10	-624.918
213291_s_at	2.53e-09	9.65e-10	624.912
213709_at	2.53e-09	9.66e-10	6.248.905
217789_at	2.53e-09	9.67e-10	624.875
219420_s_at	2.55e-09	9.73e-10	6.247.738
206508_at	2.57e-09	9.82e-10	-6.246.186
217377_x_at	2.58e-09	9.87e-10	-6.245.334
201043_s_at	2.59e-09	9.88e-10	6.245.135
219357_at	2.59e-09	9.91e-10	6.244.667
220053_at	2.60e-09	9.93e-10	-6.244.216
217367_s_at	2.61e-09	9.96e-10	-6.243.709
203668_at	2.61e-09	9.97e-10	-6.243.594
208193_at	2.61e-09	9.97e-10	-6.243.554
218539_at	2.61e-09	9.98e-10	6.243.491
200769_s_at	2.62e-09	1.00e-09	6.242.879
215197_at	2.62e-09	1.00e-09	-624.268
215266_at	2.63e-09	1.00e-09	-6.242.274
206936_x_at	2.64e-09	1.01e-09	-6.241.597
204570_at	2.64e-09	1.01e-09	-6.241.408
208198_x_at	2.64e-09	1.01e-09	-6.241.389
217373_x_at	2.65e-09	1.01e-09	-6.241.003
206172_at	2.66e-09	1.02e-09	-6.240.267
209369_at	2.66e-09	1.02e-09	-6.239.841
219158_s_at	2.67e-09	1.02e-09	6.239.172
39582_at	2.68e-09	1.03e-09	-6.238.617
209860_s_at	2.68e-09	1.03e-09	6.238.379
221072_at	2.70e-09	1.03e-09	-6.237.649
215989_at	2.72e-09	1.04e-09	-6.236.283
217058_at	2.72e-09	1.04e-09	-6.235.813
215688_at	2.73e-09	1.04e-09	-6.235.648
220349_s_at	2.73e-09	1.04e-09	-6.235.627
219356_s_at	2.73e-09	1.05e-09	6.235.143
212376_s_at	2.74e-09	1.05e-09	623.507
208497_x_at	2.74e-09	1.05e-09	-6.234.552

205851_at	2.75e-09	1.06e-09	6.233.963
202341_s_at	2.77e-09	1.06e-09	-6.233.049
217706_at	2.77e-09	1.06e-09	-623.303
206227_at	2.78e-09	1.07e-09	-6.232.352
214052_x_at	2.79e-09	1.07e-09	623.131
218020_s_at	2.81e-09	1.08e-09	6.230.341
219017_at	2.81e-09	1.08e-09	6.230.195
220120_s_at	2.82e-09	1.08e-09	-6.229.938
211532_x_at	2.83e-09	1.09e-09	-6.228.886
214371_at	2.85e-09	1.09e-09	-6.227.962
204907_s_at	2.85e-09	1.09e-09	-622.795
216891_at	2.85e-09	1.10e-09	-6.227.599
43934_at	2.86e-09	1.10e-09	-6.227.307
217184_s_at	2.87e-09	1.10e-09	-6.226.468
202676_x_at	2.87e-09	1.10e-09	6.226.382
200876_s_at	2.88e-09	1.11e-09	6.225.836
201982_s_at	2.88e-09	1.11e-09	-6.225.772
201810_s_at	2.89e-09	1.11e-09	-6.225.239
211908_x_at	2.90e-09	1.11e-09	-6.224.657
209810_at	2.91e-09	1.12e-09	-6.223.958
204757_s_at	2.93e-09	1.12e-09	-6.223.207
217557_s_at	2.93e-09	1.13e-09	-6.222.837
218644_at	2.95e-09	1.13e-09	-6.221.968
214656_x_at	2.99e-09	1.15e-09	-6.219.332
212375_at	2.99e-09	1.15e-09	6.219.108
204612_at	3.01e-09	1.16e-09	6.218.435
202259_s_at	3.01e-09	1.16e-09	-6.218.154
204820_s_at	3.02e-09	1.16e-09	-6.217.708
215663_at	3.02e-09	1.16e-09	-6.217.694
203442_x_at	3.02e-09	1.16e-09	-6.217.304
214439_x_at	3.03e-09	1.17e-09	6.216.813
210092_at	3.04e-09	1.17e-09	6.216.543
213424_at	3.04e-09	1.17e-09	6.216.537
207503_at	3.04e-09	1.17e-09	-6.216.474
204558_at	3.07e-09	1.18e-09	6.214.913
216434_at	3.10e-09	1.19e-09	-6.213.066
213716_s_at	3.11e-09	1.20e-09	6.212.652
206747_at	3.11e-09	1.20e-09	-6.212.598
207934_at	3.11e-09	1.20e-09	-6.212.504
218056_at	3.11e-09	1.20e-09	6.212.345
211566_x_at	3.11e-09	1.20e-09	6.212.308
35617_at	3.12e-09	1.20e-09	6.212.039
205456_at	3.12e-09	1.20e-09	-6.212.035
214783_s_at	3.12e-09	1.20e-09	6.211.907

214589_at	3.12e-09	1.20e-09	-6.211.836
216132_at	3.12e-09	1.20e-09	-6.211.783
204727_at	3.13e-09	1.20e-09	6.211.375
AFFX-r2-Ec-bioC-3_at	3.13e-09	1.21e-09	-6.211.271
208217_at	3.13e-09	1.21e-09	-6.211.075
215456_at	3.13e-09	1.21e-09	-6.210.912
219123_at	3.14e-09	1.21e-09	6.210.708
200915_x_at	3.15e-09	1.21e-09	6.210.076
209033_s_at	3.18e-09	1.22e-09	6.208.554
206042_x_at	3.18e-09	1.23e-09	6.208.134
219186_at	3.21e-09	1.24e-09	-6.206.553
212702_s_at	3.21e-09	1.24e-09	6.206.519
201955_at	3.21e-09	1.24e-09	6.206.439
205617_at	3.24e-09	1.25e-09	-6.205.144
206175_x_at	3.24e-09	1.25e-09	6.204.917
216330_s_at	3.24e-09	1.25e-09	-6.204.896
207270_x_at	3.29e-09	1.27e-09	-6.202.283
219337_at	3.31e-09	1.28e-09	-6.201.472
216676_x_at	3.31e-09	1.28e-09	-6.201.087
201109_s_at	3.32e-09	1.28e-09	-6.200.839
217149_x_at	3.36e-09	1.30e-09	-6.198.482
209403_at	3.37e-09	1.30e-09	-6.198.184
207766_at	3.38e-09	1.30e-09	-6.197.823
209966_x_at	3.39e-09	1.31e-09	-6.197.046
202525_at	3.39e-09	1.31e-09	-6.196.982
207113_s_at	3.39e-09	1.31e-09	-6.196.892
201067_at	3.40e-09	1.32e-09	6.196.385
207106_s_at	3.40e-09	1.32e-09	-6.196.352
209851_at	3.41e-09	1.32e-09	-6.195.745
216591_s_at	3.42e-09	1.32e-09	6.195.501
34764_at	3.45e-09	1.33e-09	6.194.068
209845_at	3.47e-09	1.34e-09	6.192.998
210679_x_at	3.51e-09	1.36e-09	-6.191.139
219594_at	3.51e-09	1.36e-09	6.190.905
216147_at	3.52e-09	1.36e-09	-6.190.591
213038_at	3.53e-09	1.37e-09	618.996
220859_at	3.58e-09	1.38e-09	-6.187.574
212156_at	3.58e-09	1.39e-09	-6.187.449
216206_x_at	3.58e-09	1.39e-09	-6.187.348
210673_x_at	3.59e-09	1.39e-09	-6.186.943
221775_x_at	3.59e-09	1.39e-09	6.186.917
209079_x_at	3.64e-09	1.41e-09	-6.184.684
202630_at	3.64e-09	1.41e-09	6.184.617
221772_s_at	3.67e-09	1.42e-09	-6.183.277

214056_at	3.70e-09	1.43e-09	-6.181.912
206378_at	3.70e-09	1.43e-09	-6.181.826
221526_x_at	3.70e-09	1.43e-09	6.181.646
201519_at	3.70e-09	1.43e-09	6.181.598
215428_at	3.71e-09	1.44e-09	-6.181.048
207687_at	3.77e-09	1.46e-09	-6.178.449
209515_s_at	3.77e-09	1.46e-09	-6.178.147
212243_at	3.78e-09	1.46e-09	617.807
202476_s_at	3.79e-09	1.47e-09	-6.177.419
204875_s_at	3.87e-09	1.50e-09	6.173.825
213692_s_at	3.88e-09	1.50e-09	-6.173.508
221020_s_at	3.89e-09	1.51e-09	6.172.978
212610_at	3.90e-09	1.51e-09	6.172.438
202162_s_at	3.92e-09	1.52e-09	6.171.485
221549_at	3.94e-09	1.53e-09	6.170.652
206882_at	4.00e-09	1.55e-09	-6.167.909
221271_at	4.04e-09	1.57e-09	-6.166.346
202801_at	4.05e-09	1.57e-09	-616.602
204834_at	4.05e-09	1.57e-09	-6.165.989
207252_at	4.07e-09	1.58e-09	-6.165.018
211954_s_at	4.10e-09	1.59e-09	6.163.537
206480_at	4.11e-09	1.60e-09	-6.163.146
217469_at	4.11e-09	1.60e-09	-6.163.087
217579_x_at	4.11e-09	1.60e-09	-6.163.049
213980_s_at	4.12e-09	1.60e-09	6.162.922
216768_x_at	4.12e-09	1.60e-09	-6.162.713
217297_s_at	4.12e-09	1.60e-09	6.162.625
209968_s_at	4.14e-09	1.61e-09	-6.161.715
205559_s_at	4.15e-09	1.61e-09	6.161.631
221857_s_at	4.15e-09	1.61e-09	6.161.326
216161_at	4.17e-09	1.62e-09	-6.160.443
216755_at	4.18e-09	1.62e-09	-6.160.147
210140_at	4.19e-09	1.63e-09	-6.159.662
210285_x_at	4.22e-09	1.64e-09	6.158.445
211943_x_at	4.22e-09	1.64e-09	-6.158.409
210076_x_at	4.22e-09	1.64e-09	6.158.291
206616_s_at	4.26e-09	1.66e-09	-615.683
221025_x_at	4.27e-09	1.66e-09	6.156.346
212660_at	4.31e-09	1.68e-09	6.154.686
217783_s_at	4.31e-09	1.68e-09	6.154.622
216050_at	4.32e-09	1.68e-09	-6.154.244
220747_at	4.32e-09	1.68e-09	-6.154.113
214986_x_at	4.35e-09	1.69e-09	6.153.068
205712_at	4.35e-09	1.69e-09	-6.152.992

212449_s_at	4.35e-09	1.70e-09	6.152.834
202644_s_at	4.36e-09	1.70e-09	6.152.538
217049_x_at	4.37e-09	1.70e-09	-6.152.097
202419_at	4.39e-09	1.71e-09	-6.151.348
203469_s_at	4.40e-09	1.71e-09	-6.150.841
215222_x_at	4.42e-09	1.72e-09	-6.150.274
210520_at	4.47e-09	1.74e-09	-6.148.406
218720_x_at	4.48e-09	1.74e-09	6.147.915
207824_s_at	4.52e-09	1.76e-09	6.146.197
220796_x_at	4.59e-09	1.79e-09	-614.369
205622_at	4.60e-09	1.79e-09	6.143.198
62212_at	4.61e-09	1.80e-09	-6.142.851
202333_s_at	4.61e-09	1.80e-09	614.281
214288_s_at	4.61e-09	1.80e-09	6.142.783
205071_x_at	4.62e-09	1.80e-09	6.142.497
201003_x_at	4.72e-09	1.84e-09	6.138.632
215701_at	4.75e-09	1.85e-09	-6.137.746
221380_at	4.76e-09	1.86e-09	-6.137.042
211396_at	4.76e-09	1.86e-09	-6.136.997
220677_s_at	4.77e-09	1.86e-09	-6.136.959
200781_s_at	4.77e-09	1.86e-09	6.136.862
206280_at	4.78e-09	1.87e-09	-6.136.309
217899_at	4.79e-09	1.87e-09	6.135.985
217416_x_at	4.79e-09	1.87e-09	-6.135.956
215179_x_at	4.80e-09	1.87e-09	-6.135.693
203056_s_at	4.81e-09	1.88e-09	-6.135.137
202931_x_at	4.83e-09	1.89e-09	6.134.469
206749_at	4.84e-09	1.89e-09	-6.134.057
207273_at	4.85e-09	1.89e-09	-613.387
208832_at	4.86e-09	1.90e-09	6.133.352
213303_x_at	4.87e-09	1.90e-09	-6.132.939
213723_s_at	4.87e-09	1.90e-09	-6.132.868
219794_at	4.87e-09	1.90e-09	-6.132.793
201007_at	4.92e-09	1.92e-09	-6.131.272
211689_s_at	4.96e-09	1.94e-09	-6.129.875
210769_at	4.98e-09	1.94e-09	-6.129.159
205500_at	5.00e-09	1.95e-09	-6.128.285
219360_s_at	5.01e-09	1.96e-09	6.127.814
216811_at	5.02e-09	1.96e-09	-6.127.613
220921_at	5.04e-09	1.97e-09	-6.126.966
219664_s_at	5.04e-09	1.97e-09	-6.126.847
218624_s_at	5.07e-09	1.98e-09	6.125.959
209806_at	5.09e-09	1.99e-09	6.125.077
206180_x_at	5.12e-09	2.00e-09	-6.124.042

221336_at	5.12e-09	2.00e-09	-612.394
41858_at	5.15e-09	2.02e-09	612.302
202781_s_at	5.16e-09	2.02e-09	-6.122.631
219889_at	5.20e-09	2.03e-09	6.121.454
201895_at	5.21e-09	2.04e-09	-6.121.094
204726_at	5.24e-09	2.05e-09	6.119.874
206437_at	5.26e-09	2.06e-09	-6.119.371
208297_s_at	5.26e-09	2.06e-09	-6.119.317
210389_x_at	5.27e-09	2.06e-09	6.118.961
202110_at	5.32e-09	2.08e-09	6.117.173
209884_s_at	5.33e-09	2.09e-09	6.116.947
219265_at	5.33e-09	2.09e-09	6.116.775
221740_x_at	5.34e-09	2.09e-09	6.116.655
219777_at	5.37e-09	2.10e-09	-6.115.727
206060_s_at	5.40e-09	2.11e-09	-6.114.689
204509_at	5.40e-09	2.12e-09	-6.114.422
208417_at	5.42e-09	2.12e-09	-6.114.037
216579_at	5.43e-09	2.13e-09	-6.113.678
211360_s_at	5.43e-09	2.13e-09	-6.113.522
207070_at	5.45e-09	2.14e-09	-6.112.962
215403_at	5.46e-09	2.14e-09	-6.112.704
213240_s_at	5.47e-09	2.15e-09	-6.112.091
211982_x_at	5.48e-09	2.15e-09	6.112.013
203268_s_at	5.48e-09	2.15e-09	6.111.922
218266_s_at	5.48e-09	2.15e-09	-611.173
214472_at	5.54e-09	2.18e-09	6.109.844
222299_x_at	5.57e-09	2.19e-09	-6.108.932
216901_s_at	5.58e-09	2.19e-09	-6.108.599
204891_s_at	5.60e-09	2.20e-09	-6.108.082
215120_s_at	5.60e-09	2.20e-09	-610.795
203353_s_at	5.62e-09	2.21e-09	6.107.324
201107_s_at	5.67e-09	2.23e-09	-6.105.825
215425_at	5.69e-09	2.23e-09	6.105.192
211498_s_at	5.71e-09	2.24e-09	-6.104.715
221440_s_at	5.76e-09	2.26e-09	-6.103.127
218346_s_at	5.77e-09	2.27e-09	-6.102.814
202156_s_at	5.77e-09	2.27e-09	-6.102.612
215952_s_at	5.78e-09	2.27e-09	6.102.332
204673_at	5.79e-09	2.27e-09	-6.102.103
218802_at	5.81e-09	2.28e-09	6.101.455
202456_s_at	5.83e-09	2.29e-09	-6.100.761
216199_s_at	5.83e-09	2.29e-09	6.100.681
215063_x_at	5.84e-09	2.29e-09	-6.100.559
215122_at	5.86e-09	2.31e-09	-6.099.773

204077_x_at	5.88e-09	2.31e-09	-6.099.142
208232_x_at	5.90e-09	2.32e-09	-60.988
211234_x_at	5.90e-09	2.32e-09	-6.098.522
210292_s_at	5.95e-09	2.34e-09	-6.097.302
201709_s_at	5.95e-09	2.34e-09	-609.705
215069_at	6.03e-09	2.37e-09	-6.094.691
218623_at	6.07e-09	2.39e-09	-6.093.678
209240_at	6.09e-09	2.40e-09	6.093.155
209704_at	6.09e-09	2.40e-09	6.093.112
219775_s_at	6.09e-09	2.40e-09	-6.093.076
206495_s_at	6.09e-09	2.40e-09	6.092.983
219446_at	6.11e-09	2.41e-09	6.092.321
219819_s_at	6.15e-09	2.42e-09	6.091.284
213176_s_at	6.17e-09	2.43e-09	-6.090.648
215383_x_at	6.18e-09	2.43e-09	-6.090.425
214083_at	6.18e-09	2.44e-09	-6.090.251
218559_s_at	6.19e-09	2.44e-09	-6.090.103
214568_at	6.19e-09	2.44e-09	-6.090.069
211856_x_at	6.20e-09	2.44e-09	-6.089.662
208188_at	6.25e-09	2.46e-09	-6.088.288
220364_at	6.30e-09	2.48e-09	-6.087.044
219418_at	6.30e-09	2.48e-09	6.086.926
217237_at	6.33e-09	2.50e-09	-6.085.986
202199_s_at	6.35e-09	2.50e-09	6.085.391
212353_at	6.36e-09	2.51e-09	6.085.251
214197_s_at	6.38e-09	2.51e-09	6.084.752
202148_s_at	6.38e-09	2.51e-09	6.084.693
208717_at	6.39e-09	2.52e-09	6.084.299
212787_at	6.40e-09	2.53e-09	608.391
211142_x_at	6.42e-09	2.53e-09	-6.083.477
210588_x_at	6.42e-09	2.53e-09	6.083.459
200047_s_at	6.43e-09	2.54e-09	6.083.055
215427_s_at	6.43e-09	2.54e-09	-6.083.022
211194_s_at	6.51e-09	2.57e-09	-6.080.943
221629_x_at	6.51e-09	2.57e-09	608.089
206925_at	6.53e-09	2.58e-09	-6.080.486
200789_at	6.56e-09	2.59e-09	-6.079.563
217033_x_at	6.62e-09	2.61e-09	-6.078.015
214832_at	6.63e-09	2.62e-09	-6.077.816
208578_at	6.63e-09	2.62e-09	-6.077.726
202516_s_at	6.65e-09	2.63e-09	6.077.157
206019_at	6.67e-09	2.64e-09	6.076.512
221246_x_at	6.69e-09	2.64e-09	-6.076.059
212745_s_at	6.70e-09	2.65e-09	6.075.648

220473_s_at	6.74e-09	2.66e-09	6.074.656
205163_at	6.78e-09	2.68e-09	-607.356
202601_s_at	6.84e-09	2.70e-09	6.072.043
221318_at	6.84e-09	2.71e-09	-607.202
206759_at	6.91e-09	2.73e-09	-6.070.405
218985_at	6.94e-09	2.75e-09	-6.069.447
201616_s_at	7.06e-09	2.79e-09	-6.066.644
210974_s_at	7.07e-09	2.79e-09	6.066.349
203483_at	7.34e-09	2.91e-09	-6.059.609
218038_at	7.36e-09	2.91e-09	6.059.151
216678_at	7.36e-09	2.91e-09	-6.059.097
216210_x_at	7.38e-09	2.92e-09	6.058.687
222160_at	7.39e-09	2.93e-09	-6.058.361
201321_s_at	7.42e-09	2.94e-09	605.774
217739_s_at	7.46e-09	2.95e-09	-6.056.835
218801_at	7.47e-09	2.96e-09	605.647
217144_at	7.49e-09	2.97e-09	-6.056.043
203424_s_at	7.50e-09	2.97e-09	-6.055.733
216401_x_at	7.53e-09	2.98e-09	-6.055.105
215977_x_at	7.53e-09	2.98e-09	-6.054.997
207641_at	7.55e-09	2.99e-09	-6.054.616
213648_at	7.56e-09	2.99e-09	6.054.406
221006_s_at	7.57e-09	3.00e-09	6.054.173
219225_at	7.58e-09	3.00e-09	6.053.774
203768_s_at	7.68e-09	3.04e-09	-605.145
221402_at	7.70e-09	3.05e-09	-6.051.144
208458_at	7.75e-09	3.07e-09	-6.049.995
213847_at	7.76e-09	3.07e-09	-6.049.721
212969_x_at	7.77e-09	3.08e-09	-6.049.515
221141_x_at	7.77e-09	3.08e-09	-6.049.341
206910_x_at	7.79e-09	3.09e-09	-6.048.912
206599_at	7.89e-09	3.13e-09	-6.046.691
216048_s_at	7.92e-09	3.14e-09	6.046.102
215148_s_at	7.95e-09	3.15e-09	604.541
206122_at	7.98e-09	3.17e-09	-6.044.631
206961_s_at	8.04e-09	3.19e-09	6.043.344
209402_s_at	8.05e-09	3.19e-09	-6.043.069
211740_at	8.10e-09	3.21e-09	-604.206
204304_s_at	8.15e-09	3.24e-09	-6.040.807
208238_x_at	8.17e-09	3.24e-09	-6.040.527
206310_at	8.17e-09	3.24e-09	-6.040.518
216799_at	8.17e-09	3.24e-09	-6.040.439
215123_at	8.17e-09	3.24e-09	-6.040.348
211986_at	8.17e-09	3.25e-09	6.040.318

209330_s_at	8.25e-09	3.28e-09	6.038.609
201023_at	8.32e-09	3.30e-09	6.037.132
44822_s_at	8.33e-09	3.31e-09	6.036.835
214050_at	8.35e-09	3.32e-09	-6.036.473
209865_at	8.37e-09	3.33e-09	-6.036.072
209617_s_at	8.40e-09	3.34e-09	6.035.472
203637_s_at	8.40e-09	3.34e-09	603.542
204798_at	8.40e-09	3.34e-09	6.035.304
200802_at	8.47e-09	3.37e-09	6.033.901
211166_at	8.50e-09	3.38e-09	-6.033.199
207427_at	8.51e-09	3.38e-09	-6.032.995
215932_at	8.51e-09	3.39e-09	-6.032.907
201221_s_at	8.52e-09	3.39e-09	-6.032.732
219290_x_at	8.54e-09	3.40e-09	-6.032.382
39891_at	8.58e-09	3.41e-09	6.031.557
217036_at	8.64e-09	3.44e-09	-6.030.303
221053_s_at	8.67e-09	3.45e-09	6.029.716
205187_at	8.70e-09	3.46e-09	6.028.956
210418_s_at	8.71e-09	3.47e-09	6.028.756
222044_at	8.75e-09	3.48e-09	6.027.945
207367_at	8.81e-09	3.51e-09	-6.026.703
216886_at	8.85e-09	3.52e-09	-6.025.968
221568_s_at	8.85e-09	3.53e-09	-6.025.852
204474_at	8.88e-09	3.54e-09	602.528
201766_at	8.90e-09	3.55e-09	-6.024.849
210244_at	8.96e-09	3.57e-09	-6.023.689
209538_at	8.99e-09	3.58e-09	6.023.024
222018_at	9.00e-09	3.58e-09	6.022.948
204852_s_at	9.03e-09	3.60e-09	-6.022.315
206235_at	9.04e-09	3.60e-09	-6.022.093
202595_s_at	9.06e-09	3.61e-09	6.021.658
204045_at	9.10e-09	3.63e-09	6.020.933
212869_x_at	9.12e-09	3.64e-09	-6.020.456
204808_s_at	9.27e-09	3.70e-09	6.017.562
212690_at	9.32e-09	3.72e-09	601.661
215168_at	9.35e-09	3.73e-09	-6.016.107
208887_at	9.36e-09	3.73e-09	6.015.774
216264_s_at	9.38e-09	3.74e-09	-6.015.518
213947_s_at	9.41e-09	3.75e-09	6.014.913
214901_at	9.45e-09	3.77e-09	601.406
211672_s_at	9.46e-09	3.77e-09	6.013.922
209857_s_at	9.49e-09	3.79e-09	-601.327
213910_at	9.52e-09	3.80e-09	-601.284
215551_at	9.53e-09	3.80e-09	-6.012.565

203992_s_at	9.65e-09	3.85e-09	6.010.337
201787_at	9.66e-09	3.86e-09	6.010.093
206033_s_at	9.67e-09	3.86e-09	-6.009.998
217078_s_at	9.72e-09	3.88e-09	-6.009.051
201130_s_at	9.72e-09	3.88e-09	-6.009.018
208286_x_at	9.72e-09	3.88e-09	-60.089
203216_s_at	9.81e-09	3.92e-09	600.743
213530_at	9.82e-09	3.92e-09	600.709
203621_at	9.86e-09	3.94e-09	6.006.456
201625_s_at	9.87e-09	3.94e-09	-6.006.295
209248_at	9.90e-09	3.96e-09	-6.005.614
215837_x_at	9.95e-09	3.98e-09	-6.004.788
210826_x_at	9.97e-09	3.99e-09	6.004.314
204237_at	1.00e-08	4.00e-09	6.003.797
219128_at	1.00e-08	4.01e-09	-6.003.377
204587_at	1.01e-08	4.04e-09	6.001.938
200687_s_at	1.01e-08	4.05e-09	6.001.723
209375_at	1.02e-08	4.06e-09	-600.108
219386_s_at	1.02e-08	4.07e-09	600.051
201885_s_at	1.02e-08	4.07e-09	6.000.472
217964_at	1.02e-08	4.09e-09	-5.999.969
202448_s_at	1.02e-08	4.09e-09	-5.999.918
213625_at	1.02e-08	4.09e-09	5.999.695
203180_at	1.03e-08	4.12e-09	-5.998.707
202643_s_at	1.03e-08	4.13e-09	599.799
222098_s_at	1.04e-08	4.15e-09	-5.997.231
210949_s_at	1.05e-08	4.20e-09	5.995.348
208447_s_at	1.05e-08	4.21e-09	5.994.883
34858_at	1.05e-08	4.21e-09	5.994.641
204220_at	1.05e-08	4.22e-09	-5.994.388
221175_at	1.05e-08	4.22e-09	-5.994.308
43977_at	1.05e-08	4.22e-09	5.994.278
206819_at	1.06e-08	4.25e-09	-5.993.163
215610_at	1.06e-08	4.25e-09	-5.992.995
221068_at	1.06e-08	4.26e-09	-599.267
214545_s_at	1.06e-08	4.26e-09	-5.992.667
210576_at	1.06e-08	4.26e-09	-5.992.548
212006_at	1.07e-08	4.27e-09	5.992.186
203102_s_at	1.09e-08	4.35e-09	5.988.912
207400_at	1.09e-08	4.35e-09	-5.988.828
207907_at	1.11e-08	4.43e-09	-598.563
217662_x_at	1.11e-08	4.44e-09	-5.985.304
209496_at	1.11e-08	4.44e-09	-5.985.284
218868_at	1.11e-08	4.45e-09	598.486

217174_s_at	1.12e-08	4.47e-09	-598.402
203135_at	1.12e-08	4.48e-09	5.983.951
200929_at	1.12e-08	4.50e-09	5.983.132
219046_s_at	1.12e-08	4.50e-09	-5.982.939
31845_at	1.16e-08	4.64e-09	5.977.462
217162_at	1.17e-08	4.68e-09	-5.976.061
210204_s_at	1.17e-08	4.69e-09	-5.975.733
217421_at	1.17e-08	4.70e-09	-5.975.199
213037_x_at	1.17e-08	4.71e-09	5.975.049
204137_at	1.20e-08	4.82e-09	5.970.966
219776_s_at	1.20e-08	4.82e-09	-5.970.769
204342_at	1.20e-08	4.83e-09	5.970.651
217041_at	1.21e-08	4.85e-09	-5.969.876
210331_at	1.21e-08	4.86e-09	-5.969.616
203651_at	1.22e-08	4.89e-09	5.968.437
210298_x_at	1.22e-08	4.90e-09	-5.968.091
213690_s_at	1.22e-08	4.90e-09	-5.968.011
208455_at	1.23e-08	4.93e-09	-5.967.029
200649_at	1.23e-08	4.93e-09	-5.966.835
218617_at	1.23e-08	4.94e-09	596.672
207908_at	1.23e-08	4.96e-09	-5.966.062
210771_at	1.23e-08	4.96e-09	-596.583
216842_x_at	1.24e-08	4.98e-09	-5.965.315
216756_at	1.24e-08	4.99e-09	-596.496
200833_s_at	1.25e-08	5.01e-09	5.964.208
218055_s_at	1.25e-08	5.01e-09	5.964.167
217510_at	1.25e-08	5.02e-09	-5.963.904
220645_at	1.25e-08	5.03e-09	-5.963.422
207114_at	1.26e-08	5.07e-09	-5.962.149
221988_at	1.27e-08	5.09e-09	5.961.293
206834_at	1.27e-08	5.09e-09	-5.961.197
211381_x_at	1.27e-08	5.10e-09	-5.960.892
206234_s_at	1.27e-08	5.13e-09	-5.959.992
210748_at	1.28e-08	5.14e-09	-5.959.748
202048_s_at	1.28e-08	5.16e-09	5.958.902
215346_at	1.28e-08	5.16e-09	-5.958.772
217859_s_at	1.29e-08	5.20e-09	5.957.724
221479_s_at	1.30e-08	5.23e-09	-5.956.679
218085_at	1.30e-08	5.23e-09	595.652
215985_at	1.30e-08	5.23e-09	595.647
205080_at	1.31e-08	5.27e-09	-595.534
212178_s_at	1.31e-08	5.29e-09	5.954.593
217729_s_at	1.32e-08	5.31e-09	-5.953.809
207457_s_at	1.32e-08	5.31e-09	-5.953.711

217565_at	1.32e-08	5.32e-09	-5.953.643
218026_at	1.32e-08	5.32e-09	5.953.445
201176_s_at	1.32e-08	5.33e-09	5.953.357
208057_s_at	1.32e-08	5.34e-09	-5.952.983
203919_at	1.33e-08	5.36e-09	-5.952.198
217616_at	1.33e-08	5.37e-09	-5.951.726
216795_at	1.34e-08	5.40e-09	-5.950.877
219272_at	1.35e-08	5.43e-09	-5.949.977
207794_at	1.35e-08	5.43e-09	-5.949.838
221666_s_at	1.35e-08	5.44e-09	5.949.509
220714_at	1.35e-08	5.46e-09	-594.898
214118_x_at	1.35e-08	5.46e-09	594.879
212358_at	1.36e-08	5.51e-09	-5.947.416
200629_at	1.39e-08	5.61e-09	5.944.118
202712_s_at	1.40e-08	5.65e-09	5.942.926
214186_s_at	1.41e-08	5.70e-09	-5.941.238
220171_x_at	1.42e-08	5.73e-09	5.940.531
215628_x_at	1.42e-08	5.75e-09	-5.939.769
208172_s_at	1.45e-08	5.85e-09	-5.936.729
221356_x_at	1.45e-08	5.85e-09	-5.936.617
216345_at	1.45e-08	5.87e-09	-5.936.207
208580_x_at	1.45e-08	5.88e-09	5.935.968
216589_at	1.46e-08	5.89e-09	5.935.505
205666_at	1.47e-08	5.92e-09	5.934.578
212073_at	1.47e-08	5.95e-09	5.933.784
208229_at	1.47e-08	5.95e-09	5.933.638
203115_at	1.49e-08	6.03e-09	5.931.314
206381_at	1.50e-08	6.05e-09	-5.930.821
202517_at	1.50e-08	6.06e-09	-593.062
213441_x_at	1.50e-08	6.06e-09	-5.930.493
220454_s_at	1.51e-08	6.10e-09	-5.929.466
214263_x_at	1.51e-08	6.10e-09	-5.929.292
208106_x_at	1.52e-08	6.14e-09	-5.928.181
218287_s_at	1.52e-08	6.16e-09	5.927.484
210797_s_at	1.52e-08	6.16e-09	-5.927.452
205230_at	1.52e-08	6.17e-09	-5.927.437
210174_at	1.53e-08	6.19e-09	5.926.772
218882_s_at	1.54e-08	6.23e-09	5.925.538
218146_at	1.55e-08	6.28e-09	-5.924.195
210044_s_at	1.56e-08	6.34e-09	-5.922.614
211709_s_at	1.56e-08	6.34e-09	-5.922.602
213103_at	1.57e-08	6.34e-09	-5.922.353
203235_at	1.57e-08	6.36e-09	-5.921.855
216403_at	1.59e-08	6.43e-09	-5.920.048

220157_x_at	1.59e-08	6.46e-09	591.925
33814_at	1.60e-08	6.46e-09	5.919.034
205479_s_at	1.60e-08	6.47e-09	5.918.816
221306_at	1.60e-08	6.48e-09	-5.918.673
204233_s_at	1.61e-08	6.51e-09	5.917.827
221259_s_at	1.61e-08	6.53e-09	-5.917.279
217183_at	1.62e-08	6.56e-09	-5.916.567
201534_s_at	1.62e-08	6.56e-09	-5.916.305
221391_at	1.62e-08	6.58e-09	-5.915.939
215892_at	1.63e-08	6.59e-09	-5.915.618
220536_at	1.63e-08	6.60e-09	-591.548
202731_at	1.63e-08	6.63e-09	5.914.537
217811_at	1.64e-08	6.67e-09	5.913.514
201987_at	1.65e-08	6.70e-09	5.912.669
202853_s_at	1.67e-08	6.78e-09	5.910.487
214042_s_at	1.68e-08	6.80e-09	5.910.028
209377_s_at	1.68e-08	6.80e-09	-5.910.003
200715_x_at	1.68e-08	6.81e-09	5.909.917
219440_at	1.68e-08	6.82e-09	-5.909.486
202950_at	1.68e-08	6.84e-09	-5.909.063
210163_at	1.70e-08	6.90e-09	5.907.418
218474_s_at	1.71e-08	6.94e-09	5.906.494
209756_s_at	1.71e-08	6.95e-09	-5.906.227
213431_x_at	1.71e-08	6.95e-09	590.608
205261_at	1.71e-08	6.96e-09	5.906.011
211416_x_at	1.71e-08	6.96e-09	-5.905.869
221484_at	1.72e-08	6.98e-09	590.537
218998_at	1.72e-08	7.00e-09	590.488
206144_at	1.73e-08	7.02e-09	-5.904.421
218394_at	1.73e-08	7.02e-09	-5.904.299
208648_at	1.73e-08	7.05e-09	5.903.643
211912_at	1.74e-08	7.08e-09	-5.902.996
211122_s_at	1.74e-08	7.09e-09	5.902.677
201220_x_at	1.75e-08	7.10e-09	-5.902.339
212962_at	1.75e-08	7.11e-09	-5.902.249
214294_at	1.75e-08	7.12e-09	-5.901.959
208306_x_at	1.75e-08	7.13e-09	-5.901.592
204527_at	1.75e-08	7.14e-09	5.901.496
206699_x_at	1.76e-08	7.14e-09	-5.901.413
206373_at	1.76e-08	7.14e-09	5.901.374
200782_at	1.77e-08	7.20e-09	5.899.894
216941_s_at	1.77e-08	7.21e-09	5.899.754
221774_x_at	1.77e-08	7.22e-09	5.899.424
205424_at	1.78e-08	7.23e-09	-589.923

207880_at	1.78e-08	7.26e-09	-5.898.478
222190_s_at	1.79e-08	7.27e-09	-5.898.252
215012_at	1.79e-08	7.28e-09	-5.897.929
212657_s_at	1.79e-08	7.28e-09	-5.897.828
210820_x_at	1.82e-08	7.39e-09	5.895.213
208210_at	1.82e-08	7.40e-09	-5.895.082
208241_at	1.82e-08	7.40e-09	-589.497
204671_s_at	1.82e-08	7.40e-09	5.894.939
217104_at	1.82e-08	7.43e-09	5.894.303
219377_at	1.83e-08	7.44e-09	-5.894.133
220164_s_at	1.83e-08	7.45e-09	-589.383
216739_at	1.84e-08	7.48e-09	-589.314
214861_at	1.85e-08	7.54e-09	-5.891.624
207115_x_at	1.85e-08	7.55e-09	-5.891.338
207727_s_at	1.86e-08	7.60e-09	5.890.376
218121_at	1.87e-08	7.63e-09	-5.889.572
218518_at	1.87e-08	7.64e-09	5.889.407
214335_at	1.88e-08	7.65e-09	-5.889.036
221458_at	1.88e-08	7.66e-09	-5.888.786
211386_at	1.88e-08	7.67e-09	-5.888.614
219524_s_at	1.90e-08	7.77e-09	-5.886.429
219710_at	1.91e-08	7.78e-09	-5.886.189
64942_at	1.91e-08	7.79e-09	-5.885.966
203397_s_at	1.91e-08	7.81e-09	-5.885.321
212349_at	1.92e-08	7.85e-09	5.884.435
210081_at	1.93e-08	7.88e-09	-5.883.929
220203_at	1.94e-08	7.91e-09	-5.883.233
202687_s_at	1.94e-08	7.91e-09	-5.883.152
209723_at	1.94e-08	7.94e-09	-5.882.438
202796_at	1.94e-08	7.94e-09	-5.882.436
209141_at	1.96e-08	8.00e-09	5.881.209
220775_s_at	1.97e-08	8.05e-09	5.880.024
216498_at	1.99e-08	8.15e-09	-5.877.871
65517_at	2.00e-08	8.18e-09	5.877.079
206653_at	2.01e-08	8.21e-09	5.876.512
209915_s_at	2.02e-08	8.23e-09	-587.598
210892_s_at	2.02e-08	8.24e-09	5.875.957
204029_at	2.02e-08	8.25e-09	5.875.547
207894_s_at	2.02e-08	8.26e-09	-5.875.323
221966_at	2.04e-08	8.33e-09	-5.873.819
205990_s_at	2.04e-08	8.35e-09	5.873.458
213177_at	2.05e-08	8.38e-09	-5.872.943
217711_at	2.06e-08	8.41e-09	-5.872.227
205693_at	2.06e-08	8.42e-09	-5.872.041

216749_at	2.06e-08	8.42e-09	-5.871.995
216102_at	2.06e-08	8.42e-09	-5.871.933
204921_at	2.08e-08	8.50e-09	-5.870.243
202880_s_at	2.11e-08	8.62e-09	586.784
208438_s_at	2.11e-08	8.63e-09	-5.867.529
220905_at	2.11e-08	8.64e-09	-58.673
212863_x_at	2.11e-08	8.65e-09	5.867.278
219456_s_at	2.12e-08	8.66e-09	-5.866.913
206928_at	2.12e-08	8.68e-09	5.866.563
208889_s_at	2.12e-08	8.69e-09	-5.866.308
203141_s_at	2.13e-08	8.71e-09	5.865.981
220587_s_at	2.13e-08	8.74e-09	5.865.349
214634_at	2.14e-08	8.76e-09	-5.864.882
216830_at	2.16e-08	8.84e-09	-5.863.226
219455_at	2.16e-08	8.84e-09	-5.863.204
201838_s_at	2.17e-08	8.87e-09	586.263
213041_s_at	2.21e-08	9.04e-09	-5.859.357
214888_at	2.21e-08	9.06e-09	-5.858.975
205584_at	2.22e-08	9.08e-09	-5.858.545
216085_at	2.22e-08	9.09e-09	-5.858.301
203195_s_at	2.24e-08	9.18e-09	5.856.517
202680_at	2.26e-08	9.26e-09	5.855.094
219663_s_at	2.26e-08	9.28e-09	-5.854.597
218743_at	2.26e-08	9.29e-09	5.854.484
220704_at	2.27e-08	9.30e-09	-5.854.311
202716_at	2.27e-08	9.31e-09	5.853.991
206359_at	2.29e-08	9.41e-09	-5.852.128
205824_at	2.31e-08	9.47e-09	-5.850.988
213942_at	2.31e-08	9.48e-09	-5.850.819
215043_s_at	2.32e-08	9.50e-09	5.850.331
214641_at	2.32e-08	9.50e-09	-5.850.323
215746_at	2.32e-08	9.53e-09	-5.849.835
211066_x_at	2.33e-08	9.56e-09	-5.849.302
207730_x_at	2.34e-08	9.59e-09	-5.848.763
204112_s_at	2.35e-08	9.66e-09	-5.847.487
219745_at	2.35e-08	9.66e-09	5.847.464
204037_at	2.35e-08	9.67e-09	-5.847.303
205400_at	2.37e-08	9.72e-09	-5.846.371
203680_at	2.37e-08	9.72e-09	-5.846.267
215579_at	2.37e-08	9.73e-09	-5.846.217
214457_at	2.37e-08	9.74e-09	-5.845.946
203426_s_at	2.37e-08	9.75e-09	-5.845.717
221435_x_at	2.40e-08	9.86e-09	-5.843.795
220212_s_at	2.40e-08	9.86e-09	5.843.786

205697_at	2.41e-08	9.90e-09	5.843.057
217384_x_at	2.41e-08	9.92e-09	-5.842.603
217270_s_at	2.41e-08	9.93e-09	-584.257
218563_at	2.42e-08	9.93e-09	5.842.482
203443_at	2.42e-08	9.94e-09	-5.842.385
208130_s_at	2.44e-08	1.00e-08	-5.840.835
65591_at	2.44e-08	1.00e-08	5.840.737
211389_x_at	2.45e-08	1.01e-08	-5.839.732
205142_x_at	2.46e-08	1.01e-08	5.839.214
216280_s_at	2.47e-08	1.02e-08	-5.838.198
203611_at	2.47e-08	1.02e-08	583.805
212993_at	2.47e-08	1.02e-08	5.838.021
218431_at	2.48e-08	1.02e-08	5.837.517
209630_s_at	2.49e-08	1.02e-08	5.837.098
219917_at	2.49e-08	1.02e-08	5.837.051
38290_at	2.50e-08	1.03e-08	5.835.773
212090_at	2.52e-08	1.04e-08	5.834.297
220397_at	2.52e-08	1.04e-08	5.834.286
201387_s_at	2.53e-08	1.04e-08	5.833.847
201765_s_at	2.53e-08	1.04e-08	5.833.653
206803_at	2.53e-08	1.04e-08	-5.833.652
213429_at	2.53e-08	1.04e-08	5.833.508
209918_at	2.55e-08	1.05e-08	-5.832.412
215501_s_at	2.57e-08	1.06e-08	-5.831.007
215261_at	2.58e-08	1.06e-08	-5.830.472
215624_at	2.58e-08	1.06e-08	-5.830.261
208166_at	2.58e-08	1.06e-08	-5.830.169
203391_at	2.59e-08	1.07e-08	-58.296
209006_s_at	2.60e-08	1.07e-08	-5.828.687
211438_at	2.61e-08	1.08e-08	-5.828.084
206567_s_at	2.62e-08	1.08e-08	5.827.047
202178_at	2.64e-08	1.09e-08	-5.826.195
217370_x_at	2.65e-08	1.09e-08	5.825.016
208923_at	2.68e-08	1.10e-08	5.823.519
216202_s_at	2.69e-08	1.11e-08	582.257
206107_at	2.69e-08	1.11e-08	-5.822.223
210090_at	2.72e-08	1.12e-08	-5.820.538
218176_at	2.72e-08	1.12e-08	5.820.283
208285_at	2.72e-08	1.12e-08	-5.820.168
209957_s_at	2.72e-08	1.12e-08	-5.820.153
204212_at	2.73e-08	1.13e-08	5.819.787
206933_s_at	2.75e-08	1.13e-08	-5.818.618
211587_x_at	2.75e-08	1.13e-08	-5.818.554
221819_at	2.77e-08	1.14e-08	5.817.355

210370_s_at	2.77e-08	1.14e-08	-5.817.041
215525_at	2.78e-08	1.15e-08	-5.816.724
201580_s_at	2.78e-08	1.15e-08	5.816.491
215294_s_at	2.79e-08	1.15e-08	5.815.865
210201_x_at	2.80e-08	1.16e-08	5.815.083
218159_at	2.80e-08	1.16e-08	5.814.936
207460_at	2.80e-08	1.16e-08	-5.814.765
201970_s_at	2.81e-08	1.16e-08	5.814.524
215377_at	2.81e-08	1.16e-08	-5.814.464
211466_at	2.82e-08	1.16e-08	-5.813.967
221012_s_at	2.83e-08	1.17e-08	5.813.152
212057_at	2.83e-08	1.17e-08	-5.812.728
213023_at	2.84e-08	1.17e-08	5.812.455
214017_s_at	2.88e-08	1.19e-08	5.810.106
206730_at	2.88e-08	1.19e-08	-5.809.925
203009_at	2.90e-08	1.20e-08	5.808.559
221097_s_at	2.91e-08	1.20e-08	-5.808.123
212199_at	2.91e-08	1.21e-08	5.807.659
212501_at	2.92e-08	1.21e-08	-5.807.127
210865_at	2.95e-08	1.22e-08	-580.541
221617_at	2.96e-08	1.22e-08	-5.805.015
220305_at	2.96e-08	1.22e-08	5.804.757
218954_s_at	2.96e-08	1.23e-08	58.047
210336_x_at	2.96e-08	1.23e-08	-5.804.526
215702_s_at	2.99e-08	1.24e-08	-5.802.806
203590_at	3.00e-08	1.24e-08	-5.802.359
205416_s_at	3.00e-08	1.24e-08	5.801.979
1294_at	3.01e-08	1.25e-08	-5.801.475
207198_s_at	3.02e-08	1.25e-08	5.800.709
204765_at	3.03e-08	1.25e-08	580.064
211748_x_at	3.03e-08	1.26e-08	-5.800.351
219720_s_at	3.04e-08	1.26e-08	-5.799.495
216570_x_at	3.05e-08	1.27e-08	5.798.882
214019_at	3.06e-08	1.27e-08	-579.859
AFFX-r2-Bs-phe-3_at	3.07e-08	1.27e-08	-5.798.082
202170_s_at	3.11e-08	1.29e-08	5.795.636
210210_at	3.12e-08	1.30e-08	5.794.681
210738_s_at	3.13e-08	1.30e-08	-5.794.402
219768_at	3.15e-08	1.31e-08	-5.793.146
208981_at	3.17e-08	1.32e-08	5.791.757
211955_at	3.17e-08	1.32e-08	5.791.695
217253_at	3.18e-08	1.32e-08	-5.791.598
204089_x_at	3.18e-08	1.32e-08	5.791.151
205338_s_at	3.19e-08	1.32e-08	-5.790.735

218393_s_at	3.21e-08	1.33e-08	5.789.674
202506_at	3.21e-08	1.33e-08	-5.789.509
207175_at	3.23e-08	1.34e-08	-5.788.732
215660_s_at	3.24e-08	1.35e-08	-5.787.697
201734_at	3.25e-08	1.35e-08	-5.787.079
216151_at	3.25e-08	1.35e-08	-5.787.069
200786_at	3.26e-08	1.35e-08	5.786.575
208329_at	3.27e-08	1.36e-08	-5.786.058
203986_at	3.28e-08	1.36e-08	-5.785.615
217095_x_at	3.28e-08	1.36e-08	-5.785.542
219726_at	3.28e-08	1.36e-08	-5.785.298
214485_at	3.28e-08	1.36e-08	-5.785.254
207408_at	3.30e-08	1.37e-08	-5.784.243
207504_at	3.31e-08	1.37e-08	-5.784.082
218898_at	3.32e-08	1.38e-08	5.783.437
202471_s_at	3.32e-08	1.38e-08	5.783.345
216023_at	3.32e-08	1.38e-08	-5.783.283
220187_at	3.33e-08	1.38e-08	-5.782.814
219013_at	3.33e-08	1.39e-08	5.782.519
210623_at	3.35e-08	1.39e-08	-5.781.536
203827_at	3.36e-08	1.40e-08	5.781.194
215587_x_at	3.36e-08	1.40e-08	-5.781.102
217120_s_at	3.36e-08	1.40e-08	-578.071
207737_at	3.37e-08	1.40e-08	-5.780.309
219438_at	3.38e-08	1.40e-08	-5.780.023
217198_x_at	3.40e-08	1.41e-08	-5.778.787
201246_s_at	3.42e-08	1.42e-08	5.777.714
206744_s_at	3.42e-08	1.42e-08	-5.777.677
207603_at	3.42e-08	1.42e-08	-5.777.605
215649_s_at	3.45e-08	1.43e-08	-5.776.245
205027_s_at	3.46e-08	1.44e-08	5.775.628
205359_at	3.47e-08	1.44e-08	-5.774.964
205085_at	3.49e-08	1.45e-08	5.774.131
204125_at	3.49e-08	1.45e-08	-5.773.802
212785_s_at	3.51e-08	1.46e-08	-5.772.964
201496_x_at	3.54e-08	1.47e-08	-57.713
203305_at	3.55e-08	1.48e-08	5.770.938
217933_s_at	3.55e-08	1.48e-08	-5.770.829
221362_at	3.57e-08	1.49e-08	-5.769.911
215300_s_at	3.57e-08	1.49e-08	-5.769.637
217667_at	3.57e-08	1.49e-08	-5.769.548
212517_at	3.57e-08	1.49e-08	5.769.499
213324_at	3.58e-08	1.49e-08	5.769.035
220521_s_at	3.58e-08	1.49e-08	-576.897

220089_at	3.61e-08	1.50e-08	-5.767.589
221201_s_at	3.62e-08	1.51e-08	-5.767.182
212190_at	3.63e-08	1.51e-08	5.766.557
209738_x_at	3.63e-08	1.51e-08	-5.766.348
221511_x_at	3.64e-08	1.52e-08	-5.765.913
208031_s_at	3.66e-08	1.53e-08	-5.764.904
220514_at	3.67e-08	1.53e-08	-576.456
212154_at	3.68e-08	1.54e-08	5.763.882
220096_at	3.69e-08	1.54e-08	-5.763.643
207374_at	3.70e-08	1.54e-08	-5.762.879
206762_at	3.71e-08	1.55e-08	-5.762.365
206327_s_at	3.71e-08	1.55e-08	-5.762.262
218706_s_at	3.72e-08	1.55e-08	-5.761.818
206349_at	3.73e-08	1.56e-08	-5.761.517
205682_x_at	3.73e-08	1.56e-08	-5.761.209
203287_at	3.74e-08	1.56e-08	5.760.832
217006_x_at	3.75e-08	1.57e-08	-5.760.085
220983_s_at	3.76e-08	1.57e-08	-5.760.001
204473_s_at	3.76e-08	1.57e-08	5.759.746
216832_at	3.77e-08	1.57e-08	-5.759.508
212748_at	3.77e-08	1.58e-08	5.759.204
210409_at	3.77e-08	1.58e-08	5.759.063
208415_x_at	3.79e-08	1.58e-08	-5.758.486
213770_at	3.80e-08	1.59e-08	-5.757.915
221990_at	3.80e-08	1.59e-08	-5.757.564
205771_s_at	3.84e-08	1.60e-08	-5.755.935
220420_at	3.85e-08	1.61e-08	-5.755.365
215732_s_at	3.85e-08	1.61e-08	-5.755.116
218371_s_at	3.86e-08	1.61e-08	5.754.991
202379_s_at	3.87e-08	1.62e-08	-5.754.172
205547_s_at	3.88e-08	1.62e-08	-5.753.861
214989_x_at	3.91e-08	1.64e-08	-575.224
204670_x_at	3.94e-08	1.65e-08	-575.107
213525_at	3.94e-08	1.65e-08	-5.751.003
211427_s_at	3.96e-08	1.66e-08	-5.750.062
201250_s_at	3.96e-08	1.66e-08	574.992
206668_s_at	3.98e-08	1.66e-08	5.749.346
202164_s_at	3.99e-08	1.67e-08	5.748.575
206090_s_at	4.00e-08	1.67e-08	-5.748.347
219251_s_at	4.02e-08	1.68e-08	-5.747.317
214570_x_at	4.04e-08	1.69e-08	-5.746.414
219387_at	4.06e-08	1.70e-08	5.745.513
214463_x_at	4.09e-08	1.71e-08	5.743.913
214920_at	4.10e-08	1.72e-08	5.743.418

211959_at	4.12e-08	1.73e-08	-5.742.624
221118_at	4.13e-08	1.73e-08	-574.225
213926_s_at	4.13e-08	1.73e-08	5.742.031
200752_s_at	4.13e-08	1.73e-08	5.741.982
202370_s_at	4.14e-08	1.73e-08	5.741.833
205468_s_at	4.14e-08	1.73e-08	-5.741.766
216385_at	4.16e-08	1.74e-08	-5.740.734
219763_at	4.19e-08	1.75e-08	5.739.672
217468_at	4.20e-08	1.76e-08	-5.739
209606_at	4.21e-08	1.77e-08	-573.856
212085_at	4.22e-08	1.77e-08	5.738.173
211892_s_at	4.22e-08	1.77e-08	-5.737.912
208231_at	4.23e-08	1.77e-08	-5.737.631
205251_at	4.24e-08	1.78e-08	-5.737.205
215972_at	4.26e-08	1.79e-08	-5.736.395
201000_at	4.29e-08	1.80e-08	5.735.065
217079_at	4.30e-08	1.80e-08	-5.734.612
221238_at	4.32e-08	1.81e-08	-5.733.941
206776_x_at	4.33e-08	1.82e-08	-5.733.276
201808_s_at	4.36e-08	1.83e-08	-5.731.991
204771_s_at	4.40e-08	1.85e-08	5.730.346
217147_s_at	4.42e-08	1.86e-08	-5.729.385
215794_x_at	4.42e-08	1.86e-08	-572.932
213978_at	4.43e-08	1.86e-08	-5.729.063
209599_s_at	4.44e-08	1.86e-08	5.728.799
214579_at	4.44e-08	1.86e-08	-5.728.641
217924_at	4.47e-08	1.88e-08	5.727.344
213895_at	4.52e-08	1.90e-08	-5.725.264
208378_x_at	4.52e-08	1.90e-08	-5.725.201
201156_s_at	4.54e-08	1.91e-08	5.724.464
210311_at	4.56e-08	1.91e-08	-5.723.821
201977_s_at	4.56e-08	1.91e-08	-5.723.759
213861_s_at	4.57e-08	1.92e-08	572.328
216655_s_at	4.58e-08	1.93e-08	-5.722.746
211405_x_at	4.58e-08	1.93e-08	-5.722.671
213390_at	4.59e-08	1.93e-08	5.722.258
205205_at	4.59e-08	1.93e-08	5.722.124
219620_x_at	4.60e-08	1.94e-08	-5.721.824
210554_s_at	4.63e-08	1.95e-08	-5.720.765
210595_at	4.65e-08	1.95e-08	-5.720.015
203617_x_at	4.65e-08	1.96e-08	-5.719.971
207268_x_at	4.65e-08	1.96e-08	5.719.743
208735_s_at	4.65e-08	1.96e-08	-5.719.738
209734_at	4.70e-08	1.98e-08	-5.717.864

211543_s_at	4.71e-08	1.98e-08	5.717.597
205112_at	4.71e-08	1.98e-08	5.717.373
207531_at	4.72e-08	1.99e-08	-5.717.104
202248_at	4.73e-08	1.99e-08	-5.716.452
205806_at	4.74e-08	1.99e-08	-5.716.348
219997_s_at	4.75e-08	2.00e-08	5.715.946
217901_at	4.76e-08	2.01e-08	571.523
202843_at	4.77e-08	2.01e-08	5.715.081
200645_at	4.77e-08	2.01e-08	-5.715.055
216145_at	4.79e-08	2.02e-08	-5.714.318
217843_s_at	4.79e-08	2.02e-08	5.714.301
207626_s_at	4.80e-08	2.02e-08	-5.713.911
202697_at	4.81e-08	2.03e-08	5.713.522
209563_x_at	4.83e-08	2.04e-08	-5.712.601
202123_s_at	4.83e-08	2.04e-08	5.712.506
213050_at	4.84e-08	2.04e-08	-5.712.025
207955_at	4.86e-08	2.05e-08	-5.711.539
211002_s_at	4.86e-08	2.05e-08	-5.711.367
215488_at	4.88e-08	2.06e-08	-5.710.819
218118_s_at	4.88e-08	2.06e-08	5.710.788
203100_s_at	4.88e-08	2.06e-08	5.710.626
207379_at	4.90e-08	2.07e-08	-570.971
219572_at	4.92e-08	2.08e-08	5.709.041
217046_s_at	4.93e-08	2.08e-08	-5.708.831
215146_s_at	4.94e-08	2.09e-08	5.708.147
209895_at	4.95e-08	2.09e-08	5.707.815
204519_s_at	4.96e-08	2.09e-08	-5.707.524
AFFX-ThrX-5_at	4.98e-08	2.10e-08	-5.706.888
214910_s_at	4.99e-08	2.10e-08	-5.706.518
217798_at	4.99e-08	2.11e-08	5.706.455
202481_at	4.99e-08	2.11e-08	-5.706.417
214313_s_at	5.05e-08	2.13e-08	5.703.986
210908_s_at	5.06e-08	2.14e-08	5.703.692
219970_at	5.11e-08	2.16e-08	-5.702.001
202198_s_at	5.12e-08	2.16e-08	-5.701.379
208476_s_at	5.13e-08	2.17e-08	-5.700.997
221387_at	5.14e-08	2.17e-08	-5.700.805
209333_at	5.20e-08	2.20e-08	-5.698.714
202510_s_at	5.20e-08	2.20e-08	-5.698.645
209856_x_at	5.22e-08	2.21e-08	5.697.983
222031_at	5.24e-08	2.21e-08	-5.697.357
215378_at	5.24e-08	2.21e-08	-5.697.293
220841_s_at	5.25e-08	2.22e-08	-5.696.916
205951_at	5.25e-08	2.22e-08	-5.696.637

221007_s_at	5.27e-08	2.23e-08	5.696.137
203775_at	5.31e-08	2.24e-08	-5.694.822
202569_s_at	5.33e-08	2.25e-08	5.693.966
217641_at	5.33e-08	2.25e-08	-5.693.944
211249_at	5.33e-08	2.26e-08	-5.693.867
203561_at	5.35e-08	2.26e-08	5.693.344
204197_s_at	5.38e-08	2.28e-08	-5.692.059
220253_s_at	5.41e-08	2.29e-08	5.691.046
220012_at	5.42e-08	2.29e-08	5.690.933
201852_x_at	5.42e-08	2.29e-08	-5.690.914
212741_at	5.49e-08	2.32e-08	-5.688.522
218387_s_at	5.50e-08	2.33e-08	5.688.057
207967_at	5.51e-08	2.33e-08	-5.687.774
203639_s_at	5.51e-08	2.33e-08	-5.687.699
218330_s_at	5.51e-08	2.33e-08	-5.687.587
215202_at	5.51e-08	2.33e-08	-5.687.566
211251_x_at	5.55e-08	2.35e-08	5.686.211
202216_x_at	5.55e-08	2.35e-08	5.686.132
211926_s_at	5.59e-08	2.37e-08	5.685.074
210030_at	5.62e-08	2.38e-08	-568.384
205381_at	5.65e-08	2.39e-08	-5.682.959
207492_at	5.66e-08	2.40e-08	-5.682.423
205538_at	5.67e-08	2.40e-08	5.682.343
217258_x_at	5.68e-08	2.41e-08	-5.681.864
217096_at	5.70e-08	2.42e-08	-56.811
211469_s_at	5.72e-08	2.42e-08	-5.680.671
211398_at	5.72e-08	2.42e-08	-5.680.659
208901_s_at	5.73e-08	2.43e-08	56.802
203605_at	5.75e-08	2.44e-08	5.679.443
214789_x_at	5.82e-08	2.47e-08	-5.677.326
209118_s_at	5.83e-08	2.47e-08	5.676.851
207169_x_at	5.84e-08	2.48e-08	5.676.757
205660_at	5.84e-08	2.48e-08	-5.676.621
220117_at	5.85e-08	2.48e-08	-5.676.288
207050_at	5.86e-08	2.49e-08	-5.675.939
211996_s_at	5.90e-08	2.50e-08	-5.674.751
206464_at	5.92e-08	2.51e-08	-5.673.903
210998_s_at	5.95e-08	2.53e-08	-5.672.996
207545_s_at	5.95e-08	2.53e-08	-5.672.961
206152_at	5.96e-08	2.53e-08	-5.672.868
216276_s_at	5.96e-08	2.53e-08	-567.275
208535_x_at	5.98e-08	2.54e-08	-5.672.187
206493_at	6.01e-08	2.55e-08	-5.671.064
216242_x_at	6.01e-08	2.55e-08	5.671.014

220599_s_at	6.02e-08	2.56e-08	-5.670.683
203252_at	6.03e-08	2.56e-08	5.670.626
220682_s_at	6.03e-08	2.56e-08	5.670.508
221939_at	6.05e-08	2.57e-08	5.669.882
208910_s_at	6.10e-08	2.59e-08	5.668.205
201960_s_at	6.13e-08	2.61e-08	-5.667.381
220778_x_at	6.14e-08	2.61e-08	-5.666.993
210270_at	6.17e-08	2.62e-08	-5.666.182
207960_at	6.19e-08	2.63e-08	-5.665.573
206285_at	6.20e-08	2.63e-08	-5.665.345
208658_at	6.20e-08	2.63e-08	5.665.342
222149_x_at	6.22e-08	2.65e-08	-5.664.534
216701_at	6.26e-08	2.66e-08	-5.663.438
202629_at	6.27e-08	2.67e-08	5.662.946
214796_at	6.29e-08	2.68e-08	-5.662.444
219156_at	6.29e-08	2.68e-08	-5.662.422
203817_at	6.29e-08	2.68e-08	-5.662.317
219318_x_at	6.30e-08	2.68e-08	-5.662.174
215089_s_at	6.34e-08	2.70e-08	5.661.058
220331_at	6.35e-08	2.70e-08	-5.660.699
204306_s_at	6.40e-08	2.72e-08	5.659.231
214998_at	6.42e-08	2.73e-08	-5.658.596
218648_at	6.42e-08	2.73e-08	5.658.572
210731_s_at	6.44e-08	2.74e-08	5.657.927
201703_s_at	6.45e-08	2.75e-08	-5.657.567
222360_at	6.48e-08	2.76e-08	5.656.826
219807_x_at	6.48e-08	2.76e-08	5.656.648
220908_at	6.52e-08	2.78e-08	-5.655.713
49306_at	6.53e-08	2.78e-08	565.543
208477_at	6.54e-08	2.79e-08	-565.512
200691_s_at	6.54e-08	2.79e-08	5.654.902
33646_g_at	6.55e-08	2.79e-08	5.654.689
209393_s_at	6.61e-08	2.82e-08	5.652.931
207216_at	6.65e-08	2.84e-08	-5.651.766
207227_x_at	6.66e-08	2.84e-08	-565.162
204985_s_at	6.77e-08	2.89e-08	5.648.483
202775_s_at	6.78e-08	2.89e-08	5.648.354
210041_s_at	6.85e-08	2.92e-08	5.646.336
216997_x_at	6.89e-08	2.94e-08	-5.645.216
202562_s_at	6.90e-08	2.94e-08	5.645.082
203281_s_at	6.90e-08	2.94e-08	-5.644.939
211370_s_at	6.90e-08	2.94e-08	5.644.859
210643_at	6.91e-08	2.95e-08	-5.644.724
206056_x_at	6.93e-08	2.96e-08	-5.644.023

220419_s_at	6.93e-08	2.96e-08	5.643.961
217593_at	6.93e-08	2.96e-08	-564.391
216634_at	6.94e-08	2.96e-08	-564.369
202645_s_at	6.94e-08	2.97e-08	5.643.606
219103_at	6.96e-08	2.97e-08	564.319
208174_x_at	6.96e-08	2.97e-08	-5.643.141
218164_at	6.98e-08	2.98e-08	-564.268
203318_s_at	6.99e-08	2.98e-08	5.642.464
221165_s_at	7.03e-08	3.00e-08	-5.641.375
206640_x_at	7.03e-08	3.00e-08	5.641.163
212150_at	7.08e-08	3.03e-08	5.639.809
207981_s_at	7.11e-08	3.04e-08	-5.639.001
216612_x_at	7.14e-08	3.05e-08	-5.638.406
220149_at	7.14e-08	3.05e-08	5.638.227
212400_at	7.15e-08	3.06e-08	-5.638.076
202927_at	7.18e-08	3.07e-08	5.637.359
202688_at	7.18e-08	3.07e-08	-5.637.137
205592_at	7.20e-08	3.08e-08	-5.636.725
207015_s_at	7.21e-08	3.08e-08	-5.636.522
214122_at	7.21e-08	3.08e-08	-5.636.454
213570_at	7.23e-08	3.09e-08	5.635.805
207919_at	7.25e-08	3.10e-08	-5.635.237
203996_s_at	7.31e-08	3.13e-08	-5.633.716
203332_s_at	7.35e-08	3.14e-08	-563.288
211390_at	7.39e-08	3.16e-08	-5.631.764
208465_at	7.41e-08	3.17e-08	-5.631.127
205959_at	7.42e-08	3.18e-08	-5.630.979
47530_at	7.43e-08	3.18e-08	5.630.606
200598_s_at	7.47e-08	3.20e-08	5.629.752
221627_at	7.48e-08	3.20e-08	-5.629.323
205170_at	7.51e-08	3.21e-08	-5.628.704
205308_at	7.53e-08	3.23e-08	5.628.014
221598_s_at	7.60e-08	3.26e-08	5.626.379
201750_s_at	7.61e-08	3.26e-08	-5.626.189
204733_at	7.62e-08	3.26e-08	-5.625.999
213372_at	7.62e-08	3.26e-08	5.625.884
210236_at	7.70e-08	3.30e-08	5.623.923
217499_x_at	7.71e-08	3.30e-08	5.623.661
214174_s_at	7.74e-08	3.32e-08	-562.288
211301_at	7.75e-08	3.32e-08	-5.622.766
216694_at	7.78e-08	3.33e-08	-562.197
203534_at	7.80e-08	3.34e-08	5.621.416
203080_s_at	7.81e-08	3.35e-08	-5.621.051
204305_at	7.86e-08	3.37e-08	-5.620.079

206472_s_at	7.88e-08	3.38e-08	5.619.568
215682_at	7.91e-08	3.39e-08	-5.618.672
213238_at	7.94e-08	3.40e-08	5.618.097
222171_s_at	7.96e-08	3.41e-08	-5.617.592
207581_s_at	8.00e-08	3.43e-08	-5.616.718
210747_at	8.00e-08	3.43e-08	-5.616.583
209924_at	8.03e-08	3.44e-08	5.615.944
AFFX-r2-Ec-bioB-5_at	8.03e-08	3.45e-08	-5.615.871
215055_at	8.04e-08	3.45e-08	-5.615.628
205252_at	8.04e-08	3.45e-08	561.561
221829_s_at	8.07e-08	3.47e-08	5.614.807
221296_at	8.09e-08	3.47e-08	-5.614.363
216412_x_at	8.14e-08	3.49e-08	-5.613.274
213917_at	8.16e-08	3.50e-08	-5.612.738
217366_at	8.18e-08	3.51e-08	-5.612.292
206057_x_at	8.20e-08	3.52e-08	-5.611.853
219304_s_at	8.20e-08	3.52e-08	-5.611.747
210815_s_at	8.26e-08	3.55e-08	5.610.358
212195_at	8.26e-08	3.55e-08	-5.610.341
205612_at	8.27e-08	3.55e-08	-561.027
221548_s_at	8.27e-08	3.55e-08	5.610.175
201959_s_at	8.29e-08	3.56e-08	-5.609.731
203712_at	8.29e-08	3.56e-08	5.609.579
217257_at	8.31e-08	3.57e-08	-5.609.235
216099_at	8.35e-08	3.59e-08	-5.608.376
208007_at	8.38e-08	3.60e-08	-5.607.501
212942_s_at	8.39e-08	3.61e-08	5.607.277
204016_at	8.46e-08	3.64e-08	-5.605.858
217081_at	8.46e-08	3.64e-08	-5.605.804
205591_at	8.46e-08	3.64e-08	-5.605.654
206906_at	8.52e-08	3.67e-08	-5.604.378
219788_at	8.54e-08	3.67e-08	-5.604.052
212590_at	8.56e-08	3.68e-08	5.603.599
211173_at	8.56e-08	3.68e-08	-5.603.532
215533_s_at	8.58e-08	3.69e-08	5.603.092
219267_at	8.59e-08	3.70e-08	5.602.856
219234_x_at	8.61e-08	3.71e-08	5.602.346
221167_s_at	8.61e-08	3.71e-08	-5.602.271
206575_at	8.62e-08	3.71e-08	-5.602.024
209670_at	8.62e-08	3.71e-08	-5.601.997
222020_s_at	8.63e-08	3.71e-08	-5.601.948
206355_at	8.73e-08	3.76e-08	5.599.746
210957_s_at	8.73e-08	3.76e-08	-5.599.584
217402_at	8.76e-08	3.77e-08	-5.599.002

208073_x_at	8.85e-08	3.81e-08	5.597.095
221237_s_at	8.92e-08	3.84e-08	-559.559
206220_s_at	8.94e-08	3.85e-08	-5.595.122
200010_at	8.95e-08	3.86e-08	5.595.003
210197_at	8.97e-08	3.87e-08	-5.594.496
212645_x_at	9.01e-08	3.88e-08	5.593.774
200941_at	9.01e-08	3.88e-08	-559.376
203568_s_at	9.01e-08	3.88e-08	-5.593.719
204874_x_at	9.03e-08	3.89e-08	-5.593.263
206329_at	9.04e-08	3.90e-08	-5.592.904
220099_s_at	9.08e-08	3.91e-08	-5.592.174
209003_at	9.09e-08	3.92e-08	-5.591.903
217598_at	9.15e-08	3.94e-08	5.590.781
212798_s_at	9.17e-08	3.95e-08	5.590.308
208235_x_at	9.17e-08	3.96e-08	5.590.187
219869_s_at	9.21e-08	3.97e-08	-5.589.473
1861_at	9.22e-08	3.98e-08	5.589.095
212936_at	9.26e-08	4.00e-08	558.838
203369_x_at	9.26e-08	4.00e-08	-5.588.338
209569_x_at	9.27e-08	4.00e-08	-5.588.104
216500_at	9.28e-08	4.00e-08	-5.587.968
222180_at	9.29e-08	4.01e-08	-5.587.635
210056_at	9.31e-08	4.02e-08	-5.587.191
220312_at	9.33e-08	4.03e-08	-5.586.784
213878_at	9.34e-08	4.04e-08	5.586.552
206589_at	9.37e-08	4.05e-08	-5.586.056
219884_at	9.43e-08	4.07e-08	-5.584.837
220601_at	9.43e-08	4.07e-08	-5.584.766
214016_s_at	9.44e-08	4.08e-08	-5.584.534
216086_at	9.56e-08	4.13e-08	-5.582.145
204455_at	9.59e-08	4.14e-08	5.581.651
214857_at	9.60e-08	4.15e-08	5.581.327
213681_at	9.60e-08	4.15e-08	5.581.292
219717_at	9.63e-08	4.16e-08	558.072
203569_s_at	9.65e-08	4.17e-08	5.580.461
203217_s_at	9.76e-08	4.22e-08	-5.578.275
213829_x_at	9.76e-08	4.22e-08	-5.578.167
213137_s_at	9.79e-08	4.23e-08	5.577.654
217239_x_at	9.83e-08	4.25e-08	-557.694
214319_at	9.83e-08	4.25e-08	5.576.921
209979_at	9.83e-08	4.25e-08	-5.576.898
220629_at	9.84e-08	4.26e-08	-5.576.555
221396_at	9.85e-08	4.26e-08	-5.576.423
215668_s_at	9.87e-08	4.27e-08	-5.575.961

204632_at	9.90e-08	4.28e-08	-5.575.501
215042_at	9.90e-08	4.28e-08	-5.575.456
203188_at	9.94e-08	4.30e-08	5.574.582
211836_s_at	9.95e-08	4.31e-08	-5.574.393
215366_at	9.96e-08	4.31e-08	-557.425
211027_s_at	9.99e-08	4.32e-08	-5.573.659
203401_at	1.00e-07	4.33e-08	5.573.262
212253_x_at	1.00e-07	4.35e-08	-5.572.575
206705_at	1.01e-07	4.37e-08	-5.571.772
204198_s_at	1.01e-07	4.38e-08	-557.123
201021_s_at	1.01e-07	4.39e-08	5.571.049
200991_s_at	1.02e-07	4.40e-08	5.570.419
208366_at	1.02e-07	4.41e-08	-5.570.038
201607_at	1.03e-07	4.45e-08	5.568.317
220532_s_at	1.03e-07	4.46e-08	-5.567.982
211855_s_at	1.03e-07	4.48e-08	5.567.275
211471_s_at	1.04e-07	4.49e-08	-5.566.482
218010_x_at	1.04e-07	4.50e-08	5.566.317
221658_s_at	1.04e-07	4.50e-08	-5.566.275
207006_s_at	1.04e-07	4.53e-08	-5.565.007
215207_x_at	1.05e-07	4.54e-08	5.564.584
202709_at	1.05e-07	4.56e-08	-5.563.763
219581_at	1.05e-07	4.57e-08	5.563.533
214386_at	1.05e-07	4.57e-08	-5.563.463
204889_s_at	1.06e-07	4.59e-08	-5.562.662
207871_s_at	1.06e-07	4.60e-08	-5.562.308
217354_s_at	1.06e-07	4.61e-08	5.561.723
216907_x_at	1.07e-07	4.66e-08	-555.956
213759_at	1.08e-07	4.68e-08	-5.558.987
221180_at	1.08e-07	4.70e-08	-5.558.198
220449_at	1.09e-07	4.71e-08	-555.759
219284_at	1.09e-07	4.74e-08	5.556.736
200895_s_at	1.09e-07	4.74e-08	5.556.413
216311_at	1.10e-07	4.76e-08	-5.555.616
209716_at	1.10e-07	4.80e-08	-5.554.305
216309_x_at	1.11e-07	4.80e-08	5.554.241
213818_x_at	1.13e-07	4.90e-08	-5.550.242
209556_at	1.13e-07	4.90e-08	5.550.214
204118_at	1.13e-07	4.91e-08	-5.550.112
208980_s_at	1.13e-07	4.92e-08	554.949
217520_x_at	1.13e-07	4.93e-08	5.549.193
216665_s_at	1.14e-07	4.96e-08	-5.548.049
217991_x_at	1.14e-07	4.97e-08	-5.547.878
221100_at	1.14e-07	4.98e-08	-5.547.457

207527_at	1.15e-07	4.98e-08	-5.547.411
201442_s_at	1.16e-07	5.02e-08	-5.545.678
220316_at	1.16e-07	5.03e-08	-5.545.434
217878_s_at	1.16e-07	5.05e-08	5.544.802
213312_at	1.16e-07	5.05e-08	5.544.792
212197_x_at	1.17e-07	5.09e-08	-5.543.386
221866_at	1.18e-07	5.12e-08	-5.542.268
204241_at	1.18e-07	5.13e-08	-5.541.769
208664_s_at	1.18e-07	5.14e-08	5.541.446
204773_at	1.18e-07	5.16e-08	-5.540.835
218198_at	1.19e-07	5.17e-08	-554.017
207144_s_at	1.19e-07	5.18e-08	-5.540.011
212615_at	1.19e-07	5.19e-08	-5.539.768
202612_s_at	1.20e-07	5.21e-08	-5.538.927
212646_at	1.20e-07	5.21e-08	-5.538.883
203573_s_at	1.20e-07	5.22e-08	5.538.566
222119_s_at	1.20e-07	5.23e-08	5.538.208
214699_x_at	1.20e-07	5.24e-08	-5.537.827
210639_s_at	1.20e-07	5.24e-08	5.537.806
208489_at	1.21e-07	5.28e-08	-5.536.282
210695_s_at	1.22e-07	5.29e-08	-5.535.874
207475_at	1.22e-07	5.31e-08	-553.523
218320_s_at	1.23e-07	5.34e-08	5.534.351
215052_at	1.23e-07	5.34e-08	-5.534.181
200616_s_at	1.23e-07	5.34e-08	5.534.108
222041_at	1.23e-07	5.35e-08	-5.533.918
202137_s_at	1.23e-07	5.35e-08	553.391
203263_s_at	1.23e-07	5.36e-08	5.533.568
221171_at	1.23e-07	5.37e-08	-5.533.278
220445_s_at	1.23e-07	5.37e-08	5.533.213
219198_at	1.24e-07	5.39e-08	5.532.661
207301_at	1.24e-07	5.40e-08	-553.213
207352_s_at	1.24e-07	5.41e-08	-5.531.733
212319_at	1.24e-07	5.43e-08	-553.128
201165_s_at	1.25e-07	5.45e-08	5.530.413
207112_s_at	1.27e-07	5.52e-08	-5.527.982
204229_at	1.27e-07	5.53e-08	-5.527.538
212850_s_at	1.27e-07	5.54e-08	5.527.511
209023_s_at	1.27e-07	5.54e-08	5.527.459
216614_at	1.28e-07	5.57e-08	-5.526.451
221921_s_at	1.28e-07	5.57e-08	-5.526.381
212300_at	1.28e-07	5.58e-08	5.525.877
215115_x_at	1.30e-07	5.67e-08	-5.523.021
214707_x_at	1.30e-07	5.67e-08	-5.522.955

213949_s_at	1.30e-07	5.67e-08	-5.522.907
205535_s_at	1.30e-07	5.68e-08	-5.522.789
215852_x_at	1.31e-07	5.70e-08	-5.522.029
211046_at	1.31e-07	5.70e-08	-552.194
202621_at	1.31e-07	5.72e-08	5.521.271
204581_at	1.31e-07	5.72e-08	-5.521.239
213447_at	1.31e-07	5.73e-08	5.521.116
209584_x_at	1.31e-07	5.73e-08	-5.520.941
208177_at	1.31e-07	5.74e-08	-5.520.843
215569_at	1.32e-07	5.77e-08	-5.519.807
210273_at	1.32e-07	5.79e-08	-5.519.027
200654_at	1.33e-07	5.80e-08	551.867
222260_at	1.33e-07	5.81e-08	-5.518.309
219011_at	1.33e-07	5.83e-08	-5.517.742
221960_s_at	1.33e-07	5.84e-08	5.517.545
216247_at	1.34e-07	5.85e-08	-5.517.187
203234_at	1.34e-07	5.85e-08	5.517.157
215067_x_at	1.34e-07	5.86e-08	-5.516.734
200731_s_at	1.34e-07	5.88e-08	-5.516.142
202819_s_at	1.35e-07	5.89e-08	5.515.928
214455_at	1.35e-07	5.91e-08	5.515.159
208209_s_at	1.35e-07	5.91e-08	-5.515.123
213035_at	1.35e-07	5.91e-08	5.515.116
216776_at	1.35e-07	5.92e-08	-5.515.042
209312_x_at	1.36e-07	5.94e-08	-5.514.374
209105_at	1.36e-07	5.94e-08	5.514.125
201598_s_at	1.37e-07	5.98e-08	5.512.904
218025_s_at	1.37e-07	5.99e-08	-5.512.797
218222_x_at	1.37e-07	6.01e-08	5.512.093
211174_s_at	1.37e-07	6.01e-08	-5.512.045
207403_at	1.37e-07	6.01e-08	-5.511.947
208557_at	1.37e-07	6.02e-08	-5.511.813
221878_at	1.38e-07	6.04e-08	5.511.055
208061_at	1.38e-07	6.05e-08	-5.510.958
208064_s_at	1.38e-07	6.07e-08	-5.510.268
210303_at	1.40e-07	6.15e-08	-5.507.793
205859_at	1.41e-07	6.17e-08	-5.507.218
203392_s_at	1.41e-07	6.17e-08	5.507.008
201837_s_at	1.42e-07	6.21e-08	5.505.962
212406_s_at	1.42e-07	6.21e-08	5.505.884
221888_at	1.42e-07	6.22e-08	-5.505.661
216837_at	1.42e-07	6.24e-08	-550.505
44702_at	1.42e-07	6.25e-08	-5.504.823
214900_at	1.42e-07	6.25e-08	-5.504.753

202891_at	1.43e-07	6.26e-08	-5.504.375
205707_at	1.43e-07	6.26e-08	-5.504.312
210758_at	1.43e-07	6.26e-08	550.427
220731_s_at	1.43e-07	6.27e-08	5.504.098
206092_x_at	1.43e-07	6.27e-08	-5.504.066
205417_s_at	1.44e-07	6.31e-08	5.502.852
207731_at	1.44e-07	6.34e-08	-5.502.078
208432_s_at	1.44e-07	6.34e-08	-5.502.063
221416_at	1.46e-07	6.39e-08	-5.500.585
205211_s_at	1.46e-07	6.43e-08	-5.499.406
210103_s_at	1.48e-07	6.48e-08	-5.498.002
211053_at	1.48e-07	6.49e-08	-5.497.624
213294_at	1.48e-07	6.52e-08	5.496.727
210645_s_at	1.49e-07	6.54e-08	5.496.305
220118_at	1.49e-07	6.54e-08	-5.496.292
208330_at	1.49e-07	6.55e-08	-5.495.857
215630_at	1.50e-07	6.58e-08	-5.495.083
216467_s_at	1.50e-07	6.58e-08	-5.495.049
200840_at	1.50e-07	6.58e-08	5.495.045
210599_at	1.50e-07	6.60e-08	-5.494.583
207523_at	1.50e-07	6.60e-08	-5.494.529
221407_at	1.50e-07	6.61e-08	-5.494.231
221424_s_at	1.50e-07	6.61e-08	-5.494.066
218289_s_at	1.51e-07	6.62e-08	5.493.834
204322_at	1.51e-07	6.62e-08	-5.493.773
207991_x_at	1.53e-07	6.71e-08	-5.491.422
210716_s_at	1.54e-07	6.76e-08	5.489.971
219044_at	1.54e-07	6.78e-08	-5.489.441
220886_at	1.54e-07	6.79e-08	-5.489.176
217936_at	1.54e-07	6.79e-08	5.488.979
210406_s_at	1.55e-07	6.81e-08	5.488.648
204654_s_at	1.58e-07	6.96e-08	-5.484.462
216794_at	1.58e-07	6.96e-08	-5.484.397
210915_x_at	1.59e-07	7.01e-08	-5.483.108
213529_at	1.59e-07	7.02e-08	-5.482.907
201859_at	1.60e-07	7.03e-08	-5.482.509
205594_at	1.60e-07	7.04e-08	5.482.386
208242_at	1.61e-07	7.07e-08	-5.481.513
213277_at	1.61e-07	7.07e-08	-5.481.421
221535_at	1.61e-07	7.08e-08	5.481.296
204657_s_at	1.61e-07	7.10e-08	-5.480.594
204147_s_at	1.63e-07	7.18e-08	5.478.677
213198_at	1.63e-07	7.18e-08	5.478.651
221657_s_at	1.64e-07	7.21e-08	5.477.642

203918_at	1.64e-07	7.22e-08	-5.477.535
209528_s_at	1.64e-07	7.23e-08	-5.477.353
212771_at	1.65e-07	7.27e-08	-5.476.304
204715_at	1.65e-07	7.27e-08	547.624
220477_s_at	1.66e-07	7.30e-08	5.475.511
205915_x_at	1.66e-07	7.31e-08	-5.475.081
215328_at	1.66e-07	7.32e-08	-547.502
204811_s_at	1.66e-07	7.32e-08	-5.475.003
201408_at	1.66e-07	7.33e-08	5.474.604
215541_s_at	1.66e-07	7.34e-08	-5.474.406
203310_at	1.66e-07	7.34e-08	54.743
202195_s_at	1.67e-07	7.35e-08	-5.474.122
221838_at	1.67e-07	7.36e-08	5.473.847
206584_at	1.67e-07	7.36e-08	5.473.778
209998_at	1.67e-07	7.37e-08	5.473.714
33304_at	1.68e-07	7.40e-08	54.728
207611_at	1.68e-07	7.41e-08	-5.472.526
201217_x_at	1.68e-07	7.43e-08	5.471.999
200797_s_at	1.69e-07	7.46e-08	-5.471.313
220062_s_at	1.69e-07	7.47e-08	5.471.145
201680_x_at	1.69e-07	7.47e-08	5.471.103
212545_s_at	1.70e-07	7.49e-08	-547.055
219937_at	1.70e-07	7.49e-08	5.470.517
217491_x_at	1.71e-07	7.54e-08	5.469.289
207030_s_at	1.71e-07	7.55e-08	5.469.102
203154_s_at	1.71e-07	7.55e-08	5.468.988
209837_at	1.72e-07	7.58e-08	5.468.237
213341_at	1.72e-07	7.61e-08	5.467.464
41037_at	1.74e-07	7.67e-08	5.466.088
203097_s_at	1.74e-07	7.68e-08	-5.465.857
218776_s_at	1.74e-07	7.71e-08	5.465.132
205410_s_at	1.76e-07	7.77e-08	-546.367
214404_x_at	1.76e-07	7.77e-08	-5.463.651
219062_s_at	1.76e-07	7.77e-08	-5.463.575
206396_at	1.76e-07	7.80e-08	-5.462.916
213091_at	1.76e-07	7.80e-08	-5.462.876
202142_at	1.76e-07	7.80e-08	5.462.876
208706_s_at	1.76e-07	7.80e-08	-5.462.863
207978_s_at	1.77e-07	7.82e-08	-5.462.402
37566_at	1.77e-07	7.83e-08	-5.462.168
208043_at	1.77e-07	7.83e-08	-5.462.097
AFFX-r2-Ec-bioD-5_at	1.77e-07	7.84e-08	-5.462.047
211478_s_at	1.78e-07	7.89e-08	5.460.783
177_at	1.79e-07	7.94e-08	-5.459.437

209829_at	1.80e-07	7.96e-08	-5.459.117
207508_at	1.80e-07	7.97e-08	5.458.898
214098_at	1.80e-07	7.97e-08	5.458.744
221911_at	1.81e-07	8.00e-08	5.458.115
214074_s_at	1.81e-07	8.02e-08	5.457.647
207499_x_at	1.81e-07	8.03e-08	-5.457.441
207633_s_at	1.82e-07	8.05e-08	-5.456.872
214615_at	1.83e-07	8.12e-08	-5.455.396
215162_at	1.84e-07	8.15e-08	-5.454.523
206361_at	1.84e-07	8.16e-08	5.454.242
217817_at	1.84e-07	8.17e-08	5.454.129
203192_at	1.85e-07	8.19e-08	5.453.588
209347_s_at	1.85e-07	8.20e-08	-5.453.396
218616_at	1.85e-07	8.20e-08	5.453.379
214775_at	1.86e-07	8.22e-08	-5.452.897
222297_x_at	1.86e-07	8.22e-08	545.287
206974_at	1.86e-07	8.25e-08	-5.452.354
37028_at	1.86e-07	8.25e-08	-5.452.243
204872_at	1.89e-07	8.39e-08	-5.449.028
206667_s_at	1.89e-07	8.40e-08	5.448.905
215600_x_at	1.91e-07	8.46e-08	-5.447.509
210615_at	1.91e-07	8.47e-08	-5.447.188
201505_at	1.91e-07	8.49e-08	5.446.847
203831_at	1.94e-07	8.60e-08	-5.444.324
212754_s_at	1.94e-07	8.61e-08	-5.444.262
202923_s_at	1.94e-07	8.63e-08	-5.443.811
205609_at	1.95e-07	8.64e-08	5.443.502
203563_at	1.97e-07	8.76e-08	54.408
210980_s_at	1.98e-07	8.77e-08	5.440.679
203253_s_at	1.98e-07	8.77e-08	5.440.678
215978_x_at	1.98e-07	8.81e-08	-5.439.771
222109_at	1.99e-07	8.83e-08	-5.439.413
205135_s_at	1.99e-07	8.86e-08	5.438.753
220031_at	2.01e-07	8.95e-08	-5.436.884
208402_at	2.01e-07	8.95e-08	-5.436.867
218774_at	2.02e-07	8.95e-08	-5.436.778
209106_at	2.02e-07	8.96e-08	5.436.584
211992_at	2.03e-07	9.00e-08	54.358
207817_at	2.04e-07	9.05e-08	-5.434.795
211734_s_at	2.04e-07	9.07e-08	-5.434.335
201998_at	2.05e-07	9.11e-08	-5.433.423
212177_at	2.05e-07	9.12e-08	-5.433.143
206358_at	2.05e-07	9.13e-08	-5.433.019
202953_at	2.06e-07	9.17e-08	-5.432.206

218221_at	2.06e-07	9.18e-08	5.432
207264_at	2.08e-07	9.24e-08	5.430.768
205039_s_at	2.09e-07	9.29e-08	-5.429.806
221917_s_at	2.09e-07	9.31e-08	-5.429.336
201953_at	2.10e-07	9.33e-08	-5.428.952
214369_s_at	2.10e-07	9.34e-08	-5.428.729
220453_at	2.10e-07	9.34e-08	-5.428.674
211235_s_at	2.10e-07	9.34e-08	-5.428.617
210057_at	2.11e-07	9.40e-08	-5.427.487
210137_s_at	2.14e-07	9.51e-08	-5.425.182
201171_at	2.14e-07	9.55e-08	5.424.556
215076_s_at	2.15e-07	9.59e-08	-5.423.674
213434_at	2.16e-07	9.61e-08	-5.423.229
216163_at	2.16e-07	9.61e-08	-5.423.215
208571_at	2.17e-07	9.65e-08	-5.422.395
207964_x_at	2.18e-07	9.69e-08	-5.421.653
208847_s_at	2.18e-07	9.70e-08	-5.421.515
217021_at	2.19e-07	9.73e-08	-5.420.859
219002_at	2.19e-07	9.74e-08	5.420.739
205098_at	2.19e-07	9.75e-08	-5.420.451
218047_at	2.19e-07	9.77e-08	-5.420.135
210259_s_at	2.20e-07	9.78e-08	-5.419.906
216493_s_at	2.20e-07	9.78e-08	5.419.898
221130_s_at	2.20e-07	9.81e-08	-5.419.288
214269_at	2.21e-07	9.83e-08	-5.418.936
206690_at	2.21e-07	9.84e-08	-5.418.765
205356_at	2.22e-07	9.89e-08	5.417.864
221799_at	2.23e-07	9.93e-08	5.416.941
221655_x_at	2.23e-07	9.95e-08	-5.416.731
208303_s_at	2.24e-07	9.97e-08	-5.416.216
205932_s_at	2.24e-07	9.98e-08	5.416.111
205226_at	2.25e-07	1.00e-07	5.415.405
200822_x_at	2.25e-07	1.00e-07	541.499
218375_at	2.26e-07	1.01e-07	-5.414.511
203711_s_at	2.26e-07	1.01e-07	-5.414.409
206845_s_at	2.28e-07	1.02e-07	5.412.649
221912_s_at	2.28e-07	1.02e-07	5.412.351
217715_x_at	2.30e-07	1.03e-07	-5.410.693
204782_at	2.30e-07	1.03e-07	-5.410.357
215727_x_at	2.30e-07	1.03e-07	-5.410.331
208228_s_at	2.33e-07	1.04e-07	-5.408.172
201783_s_at	2.36e-07	1.05e-07	5.405.741
208088_s_at	2.37e-07	1.06e-07	-5.405.286
206781_at	2.37e-07	1.06e-07	-5.405.278

222359_x_at	2.37e-07	1.06e-07	-5.405.172
214318_s_at	2.38e-07	1.06e-07	5.404.053
211985_s_at	2.39e-07	1.07e-07	-5.403.579
210960_at	2.40e-07	1.07e-07	-5.402.762
205722_s_at	2.40e-07	1.07e-07	-5.402.239
205593_s_at	2.40e-07	1.07e-07	540.201
212351_at	2.41e-07	1.08e-07	5.401.334
204031_s_at	2.43e-07	1.09e-07	5.400.037
216520_s_at	2.44e-07	1.09e-07	-5.399.494
215492_x_at	2.44e-07	1.09e-07	-5.399.351
208742_s_at	2.45e-07	1.09e-07	5.398.487
214345_at	2.46e-07	1.10e-07	-5.397.311
222075_s_at	2.47e-07	1.10e-07	5.396.862
206384_at	2.49e-07	1.11e-07	-539.517
217571_at	2.51e-07	1.12e-07	-5.393.955
203271_s_at	2.51e-07	1.12e-07	5.393.948
211795_s_at	2.51e-07	1.12e-07	-5.393.788
208710_s_at	2.52e-07	1.13e-07	5.393.081
218778_x_at	2.52e-07	1.13e-07	-5.393.042
220863_at	2.52e-07	1.13e-07	-5.393.004
220284_at	2.53e-07	1.13e-07	-5.392.217
217574_at	2.53e-07	1.13e-07	-5.392.038
206863_x_at	2.53e-07	1.13e-07	-5.392.035
217413_s_at	2.54e-07	1.14e-07	-5.391.142
208440_at	2.54e-07	1.14e-07	-539.108
219904_at	2.54e-07	1.14e-07	5.390.986
203905_at	2.54e-07	1.14e-07	5.390.965
213502_x_at	2.55e-07	1.14e-07	-539.059
217115_at	2.55e-07	1.14e-07	-5.390.329
202713_s_at	2.56e-07	1.14e-07	5.389.896
209685_s_at	2.56e-07	1.15e-07	-5.389.694
205331_s_at	2.56e-07	1.15e-07	-5.389.466
221581_s_at	2.57e-07	1.15e-07	-5.388.643
211555_s_at	2.58e-07	1.16e-07	-5.388.059
212653_s_at	2.60e-07	1.16e-07	-5.386.735
217446_x_at	2.60e-07	1.16e-07	-5.386.696
203289_s_at	2.60e-07	1.17e-07	-5.386.478
59999_at	2.60e-07	1.17e-07	5.386.096
204661_at	2.61e-07	1.17e-07	-5.385.934
217920_at	2.62e-07	1.17e-07	5.385.194
219110_at	2.62e-07	1.17e-07	5.384.871
207086_x_at	2.62e-07	1.18e-07	5.384.539
214578_s_at	2.63e-07	1.18e-07	5.384.158
217235_x_at	2.63e-07	1.18e-07	-5.383.971

216784_at	2.66e-07	1.19e-07	-5.382.029
214088_s_at	2.66e-07	1.19e-07	-5.381.873
212968_at	2.67e-07	1.20e-07	-5.381.486
200736_s_at	2.68e-07	1.20e-07	5.380.319
218636_s_at	2.68e-07	1.20e-07	5.380.284
205652_s_at	2.69e-07	1.21e-07	5.379.742
208614_s_at	2.69e-07	1.21e-07	5.379.472
204030_s_at	2.72e-07	1.22e-07	-5.377.335
211339_s_at	2.73e-07	1.22e-07	-5.376.986
204192_at	2.74e-07	1.23e-07	-5.376.259
213036_x_at	2.75e-07	1.23e-07	-5.375.667
213208_at	2.75e-07	1.23e-07	5.375.574
211728_s_at	2.75e-07	1.24e-07	5.375.136
215632_at	2.75e-07	1.24e-07	-5.375.105
209164_s_at	2.77e-07	1.25e-07	5.373.629
204968_at	2.78e-07	1.25e-07	5.373.398
215160_x_at	2.78e-07	1.25e-07	537.319
219700_at	2.78e-07	1.25e-07	5.373.087
214715_x_at	2.78e-07	1.25e-07	-5.372.868
203882_at	2.80e-07	1.26e-07	-5.371.603
218097_s_at	2.81e-07	1.26e-07	5.370.999
221476_s_at	2.81e-07	1.26e-07	5.370.936
217779_s_at	2.82e-07	1.27e-07	5.370.617
202305_s_at	2.82e-07	1.27e-07	537.024
205992_s_at	2.83e-07	1.27e-07	-5.369.579
206166_s_at	2.83e-07	1.27e-07	-5.369.526
204995_at	2.85e-07	1.28e-07	5.368.439
205896_at	2.85e-07	1.28e-07	5.368.149
207685_at	2.85e-07	1.28e-07	-5.367.886
215132_at	2.86e-07	1.29e-07	-536.737
219259_at	2.86e-07	1.29e-07	-5.367.301
214474_at	2.86e-07	1.29e-07	5.367.155
200935_at	2.86e-07	1.29e-07	5.367.148
214350_at	2.88e-07	1.30e-07	-536.593
205899_at	2.90e-07	1.30e-07	-5.364.971
207801_s_at	2.90e-07	1.30e-07	5.364.754
215025_at	2.92e-07	1.31e-07	-5.363.374
216639_at	2.93e-07	1.32e-07	-5.362.989
218881_s_at	2.93e-07	1.32e-07	-5.362.864
221696_s_at	2.93e-07	1.32e-07	-5.362.736
218373_at	2.95e-07	1.33e-07	-5.361.491
207885_at	2.95e-07	1.33e-07	-5.361.359
212188_at	2.96e-07	1.33e-07	-5.360.697
204701_s_at	2.96e-07	1.33e-07	5.360.356

203321_s_at	2.98e-07	1.34e-07	535.943
212783_at	2.98e-07	1.34e-07	-5.359.129
214004_s_at	2.99e-07	1.35e-07	5.358.333
217481_x_at	3.00e-07	1.35e-07	-5.358.149
207242_s_at	3.01e-07	1.36e-07	-5.357.459
216214_at	3.06e-07	1.38e-07	-5.354.365
205667_at	3.06e-07	1.38e-07	5.353.945
205425_at	3.06e-07	1.38e-07	5.353.757
207927_at	3.07e-07	1.38e-07	-5.353.536
202053_s_at	3.07e-07	1.38e-07	-5.353.388
38037_at	3.07e-07	1.39e-07	-5.353.238
214377_s_at	3.07e-07	1.39e-07	-5.353.055
216372_at	3.09e-07	1.39e-07	-5.351.918
219518_s_at	3.10e-07	1.40e-07	5.351.703
201830_s_at	3.12e-07	1.41e-07	5.350.311
218152_at	3.13e-07	1.41e-07	5.349.268
35150_at	3.15e-07	1.42e-07	-5.348.361
203618_at	3.16e-07	1.42e-07	-5.347.802
211126_s_at	3.20e-07	1.45e-07	5.345.038
217534_at	3.20e-07	1.45e-07	-5.345.029
213436_at	3.21e-07	1.45e-07	5.344.647
213367_at	3.23e-07	1.46e-07	5.343.589
210809_s_at	3.23e-07	1.46e-07	5.343.204
200901_s_at	3.23e-07	1.46e-07	5.343.095
219755_at	3.25e-07	1.47e-07	5.342.092
211796_s_at	3.27e-07	1.47e-07	-5.341.191
215674_at	3.27e-07	1.48e-07	-5.341.018
215838_at	3.27e-07	1.48e-07	-5.340.871
210928_at	3.27e-07	1.48e-07	-5.340.786
208782_at	3.27e-07	1.48e-07	-5.340.703
204495_s_at	3.28e-07	1.48e-07	5.340.352
220929_at	3.28e-07	1.48e-07	-53.402
216716_at	3.29e-07	1.49e-07	-5.339.791
206672_at	3.30e-07	1.49e-07	-5.338.928
221099_at	3.30e-07	1.49e-07	-5.338.726
216649_at	3.32e-07	1.50e-07	-5.337.966
215298_at	3.32e-07	1.50e-07	-5.337.786
220358_at	3.35e-07	1.51e-07	-5.336.132
209890_at	3.36e-07	1.52e-07	5.335.582
202203_s_at	3.38e-07	1.53e-07	-5.334.216
209793_at	3.38e-07	1.53e-07	-5.334.049
203907_s_at	3.39e-07	1.53e-07	5.333.677
202290_at	3.40e-07	1.54e-07	5.333.016
216429_at	3.40e-07	1.54e-07	-5.333

205755_at	3.40e-07	1.54e-07	-5.332.822
217411_s_at	3.41e-07	1.54e-07	-5.332.635
220583_at	3.41e-07	1.55e-07	-5.332.147
215388_s_at	3.43e-07	1.55e-07	-5.331.112
206547_s_at	3.44e-07	1.56e-07	-5.330.847
200818_at	3.44e-07	1.56e-07	5.330.569
212432_at	3.44e-07	1.56e-07	-5.330.507
208155_x_at	3.45e-07	1.56e-07	533.032
210561_s_at	3.45e-07	1.56e-07	-5.329.846
220179_at	3.46e-07	1.57e-07	-5.329.321
202999_s_at	3.47e-07	1.57e-07	-5.328.995
215911_x_at	3.48e-07	1.58e-07	-532.819
216715_at	3.49e-07	1.58e-07	-5.327.968
218907_s_at	3.49e-07	1.58e-07	5.327.718
217323_at	3.50e-07	1.58e-07	-5.327.352
205265_s_at	3.51e-07	1.59e-07	-5.326.737
209904_at	3.51e-07	1.59e-07	5.326.661
215005_at	3.51e-07	1.59e-07	-5.326.323
216204_at	3.52e-07	1.60e-07	-5.325.705
208180_s_at	3.53e-07	1.60e-07	5.325.531
AFFX-HSAC07/X0035	3.53e-07	1.60e-07	532.542
211374_x_at	3.54e-07	1.61e-07	-5.324.697
208403_x_at	3.54e-07	1.61e-07	-5.324.547
201439_at	3.56e-07	1.61e-07	5.323.892
221618_s_at	3.56e-07	1.61e-07	5.323.841
214236_at	3.57e-07	1.62e-07	-5.323.329
213821_s_at	3.57e-07	1.62e-07	-5.323.191
37965_at	3.58e-07	1.63e-07	5.322.313
205245_at	3.61e-07	1.64e-07	-5.320.784
207713_s_at	3.62e-07	1.64e-07	5.320.425
211379_x_at	3.63e-07	1.65e-07	5.319.953
216981_x_at	3.63e-07	1.65e-07	-5.319.524
221489_s_at	3.64e-07	1.65e-07	-5.319.389
214368_at	3.65e-07	1.66e-07	-5.318.708
208276_at	3.65e-07	1.66e-07	-5.318.705
209579_s_at	3.65e-07	1.66e-07	-531.843
204827_s_at	3.67e-07	1.67e-07	5.317.709
206265_s_at	3.68e-07	1.67e-07	-5.317.195
209254_at	3.68e-07	1.67e-07	-5.317.175
209356_x_at	3.68e-07	1.67e-07	-5.316.953
222225_at	3.69e-07	1.67e-07	-5.316.645
214701_s_at	3.69e-07	1.68e-07	-5.316.554
217337_at	3.69e-07	1.68e-07	-5.316.502
217567_at	3.70e-07	1.68e-07	-5.316.057

218844_at	3.70e-07	1.68e-07	-5.315.946
220209_at	3.71e-07	1.68e-07	-5.315.467
210975_x_at	3.71e-07	1.69e-07	5.315.016
211839_s_at	3.73e-07	1.69e-07	-5.314.448
216404_at	3.74e-07	1.70e-07	-5.313.842
209530_at	3.75e-07	1.71e-07	-5.313.013
210170_at	3.76e-07	1.71e-07	-5.312.567
215883_at	3.77e-07	1.72e-07	-5.311.961
214870_x_at	3.78e-07	1.72e-07	-531.152
211578_s_at	3.80e-07	1.73e-07	531.029
213425_at	3.81e-07	1.73e-07	5.310.163
209263_x_at	3.81e-07	1.73e-07	-5.309.843
220569_at	3.82e-07	1.74e-07	-5.309.605
218101_s_at	3.82e-07	1.74e-07	5.309.325
220894_x_at	3.82e-07	1.74e-07	-5.309.144
208167_s_at	3.85e-07	1.75e-07	-5.307.728
213973_at	3.86e-07	1.76e-07	-5.307.187
212119_at	3.89e-07	1.77e-07	5.305.794
207228_at	3.91e-07	1.78e-07	-5.304.936
211474_s_at	3.92e-07	1.78e-07	5.304.513
203874_s_at	3.93e-07	1.79e-07	5.303.587
215784_at	3.94e-07	1.80e-07	-5.303.036
215104_at	3.97e-07	1.81e-07	-53.016
216757_at	3.99e-07	1.82e-07	-5.300.847
212500_at	4.00e-07	1.82e-07	5.300.233
214556_at	4.02e-07	1.83e-07	-5.299.355
211439_at	4.04e-07	1.84e-07	-5.298.401
220758_s_at	4.04e-07	1.84e-07	-5.298.254
205016_at	4.04e-07	1.84e-07	-5.298.203
201140_s_at	4.04e-07	1.84e-07	5.298.146
65884_at	4.06e-07	1.85e-07	5.297.142
219424_at	4.06e-07	1.85e-07	-5.297.118
207536_s_at	4.07e-07	1.86e-07	-5.296.766
215096_s_at	4.07e-07	1.86e-07	-5.296.678
203288_at	4.11e-07	1.87e-07	5.294.935
221841_s_at	4.11e-07	1.87e-07	-529.486
218993_at	4.11e-07	1.88e-07	-5.294.642
89476_r_at	4.12e-07	1.88e-07	-5.294.288
215653_at	4.13e-07	1.89e-07	5.293.546
206454_s_at	4.13e-07	1.89e-07	-5.293.521
221480_at	4.16e-07	1.90e-07	529.235
219252_s_at	4.18e-07	1.91e-07	-5.291.564
219332_at	4.18e-07	1.91e-07	-5.291.438
220131_at	4.18e-07	1.91e-07	-5.291.206

203920_at	4.18e-07	1.91e-07	-5.291.201
210920_x_at	4.19e-07	1.91e-07	-5.290.867
216152_at	4.21e-07	1.92e-07	-5.290.037
214602_at	4.26e-07	1.95e-07	-5.287.446
202344_at	4.28e-07	1.95e-07	5.286.745
34408_at	4.28e-07	1.96e-07	5.286.489
206009_at	4.29e-07	1.96e-07	-5.286.379
216249_at	4.29e-07	1.96e-07	-5.286.016
221496_s_at	4.30e-07	1.96e-07	-5.285.773
202311_s_at	4.30e-07	1.96e-07	5.285.735
210681_s_at	4.30e-07	1.96e-07	-5.285.682
221534_at	4.30e-07	1.96e-07	5.285.677
200814_at	4.30e-07	1.97e-07	5.285.523
219179_at	4.30e-07	1.97e-07	-5.285.368
208230_s_at	4.32e-07	1.98e-07	-5.284.509
201018_at	4.33e-07	1.98e-07	5.284.131
200911_s_at	4.34e-07	1.98e-07	5.283.882
215048_at	4.37e-07	2.00e-07	-5.282.429
208721_s_at	4.39e-07	2.01e-07	5.281.346
212909_at	4.41e-07	2.02e-07	5.280.424
217313_at	4.42e-07	2.02e-07	5.279.981
215958_at	4.43e-07	2.02e-07	-5.279.839
219597_s_at	4.43e-07	2.03e-07	5.279.416
216484_x_at	4.47e-07	2.04e-07	527.797
213239_at	4.47e-07	2.05e-07	5.277.718
210113_s_at	4.47e-07	2.05e-07	-5.277.612
209636_at	4.50e-07	2.06e-07	5.276.255
206649_s_at	4.53e-07	2.07e-07	5.275.328
218400_at	4.53e-07	2.07e-07	5.275.042
200775_s_at	4.54e-07	2.08e-07	5.274.625
216235_s_at	4.57e-07	2.09e-07	-5.273.428
210992_x_at	4.62e-07	2.11e-07	-5.271.421
208529_at	4.62e-07	2.11e-07	-5.271.342
206356_s_at	4.64e-07	2.12e-07	5.270.572
204456_s_at	4.66e-07	2.13e-07	-5.269.429
208179_x_at	4.67e-07	2.14e-07	-5.269.014
220425_x_at	4.67e-07	2.14e-07	-526.891
213051_at	4.70e-07	2.15e-07	5.267.759
221965_at	4.71e-07	2.16e-07	5.267.162
204732_s_at	4.73e-07	2.17e-07	5.266.686
222012_at	4.73e-07	2.17e-07	-5.266.362
206080_at	4.74e-07	2.17e-07	-5.266.191
203652_at	4.74e-07	2.17e-07	5.265.989
65472_at	4.75e-07	2.18e-07	5.265.683

205716_at	4.76e-07	2.18e-07	5.265.127
204772_s_at	4.77e-07	2.19e-07	5.264.572
206169_x_at	4.80e-07	2.20e-07	-5.263.332
204254_s_at	4.81e-07	2.21e-07	5.263.097
216758_at	4.82e-07	2.21e-07	-5.262.773
216346_at	4.82e-07	2.21e-07	-5.262.576
208287_at	4.83e-07	2.22e-07	-5.262.173
202127_at	4.83e-07	2.22e-07	5.262.168
204139_x_at	4.85e-07	2.22e-07	-5.261.521
213304_at	4.86e-07	2.23e-07	5.261.131
215345_x_at	4.86e-07	2.23e-07	-5.261.125
222168_at	4.89e-07	2.24e-07	-5.259.638
217131_at	4.93e-07	2.26e-07	-525.816
213498_at	4.93e-07	2.26e-07	-5.257.986
212220_at	4.94e-07	2.27e-07	5.257.513
204568_at	4.95e-07	2.27e-07	5.257.188
201254_x_at	4.96e-07	2.28e-07	5.256.781
212187_x_at	4.96e-07	2.28e-07	-5.256.693
211756_at	4.96e-07	2.28e-07	5.256.611
204563_at	4.99e-07	2.29e-07	-5.255.725
216120_s_at	5.00e-07	2.30e-07	5.255.087
212277_at	5.02e-07	2.31e-07	-5.254.321
320_at	5.03e-07	2.31e-07	5.253.851
214268_s_at	5.05e-07	2.32e-07	-5.253.372
214685_at	5.12e-07	2.35e-07	-5.250.663
215566_x_at	5.12e-07	2.35e-07	525.043
203171_s_at	5.12e-07	2.35e-07	5.250.423
203825_at	5.12e-07	2.35e-07	5.250.341
212179_at	5.12e-07	2.35e-07	-5.250.318
218454_at	5.16e-07	2.37e-07	5.249.006
216044_x_at	5.16e-07	2.37e-07	-5.248.948
205611_at	5.16e-07	2.37e-07	-5.248.934
217291_at	5.16e-07	2.37e-07	-5.248.673
214507_s_at	5.18e-07	2.38e-07	5.248.076
221669_s_at	5.23e-07	2.40e-07	-5.246.186
208965_s_at	5.23e-07	2.41e-07	-5.246.078
214970_s_at	5.25e-07	2.42e-07	-5.245.308
202818_s_at	5.26e-07	2.42e-07	-5.245.009
209691_s_at	5.28e-07	2.43e-07	-5.244.228
90610_at	5.29e-07	2.43e-07	-5.243.947
205341_at	5.32e-07	2.45e-07	-5.242.804
214921_at	5.37e-07	2.47e-07	-524.068
208298_at	5.39e-07	2.48e-07	-5.239.903
216407_at	5.40e-07	2.49e-07	-5.239.718

59375_at	5.41e-07	2.49e-07	-523.923
201370_s_at	5.42e-07	2.49e-07	5.239.024
200796_s_at	5.42e-07	2.50e-07	-5.238.772
221459_at	5.44e-07	2.51e-07	-5.238.057
209659_s_at	5.45e-07	2.51e-07	5.237.959
219827_at	5.50e-07	2.53e-07	-5.235.868
207473_at	5.51e-07	2.54e-07	-523.576
219808_at	5.52e-07	2.54e-07	-52.353
217182_at	5.52e-07	2.54e-07	-5.235.112
206362_x_at	5.55e-07	2.56e-07	-523.407
209910_at	5.59e-07	2.58e-07	5.232.583
214572_s_at	5.59e-07	2.58e-07	-523.258
210420_at	5.61e-07	2.59e-07	-5.231.959
205313_at	5.61e-07	2.59e-07	-5.231.931
211017_s_at	5.62e-07	2.59e-07	-5.231.435
216282_x_at	5.63e-07	2.60e-07	-5.231.214
209120_at	5.64e-07	2.60e-07	-5.230.805
218200_s_at	5.65e-07	2.61e-07	5.230.319
200879_s_at	5.66e-07	2.61e-07	-5.230.208
222055_at	5.67e-07	2.62e-07	-5.229.579
211022_s_at	5.68e-07	2.62e-07	-5.229.422
222315_at	5.73e-07	2.64e-07	5.227.795
221723_s_at	5.73e-07	2.64e-07	-5.227.549
202980_s_at	5.75e-07	2.65e-07	5.227.054
204850_s_at	5.75e-07	2.65e-07	-5.226.927
216878_x_at	5.76e-07	2.66e-07	-5.226.407
220467_at	5.78e-07	2.67e-07	-5.225.919
202869_at	5.78e-07	2.67e-07	-5.225.911
207547_s_at	5.80e-07	2.68e-07	-5.225.028
215917_at	5.81e-07	2.68e-07	-5.224.667
220094_s_at	5.82e-07	2.69e-07	5.224.551
200603_at	5.82e-07	2.69e-07	5.224.505
210310_s_at	5.84e-07	2.70e-07	-5.223.806
211325_x_at	5.84e-07	2.70e-07	5.223.564
218817_at	5.85e-07	2.70e-07	5.223.475
218042_at	5.85e-07	2.70e-07	5.223.437
206488_s_at	5.85e-07	2.70e-07	5.223.254
204854_at	5.86e-07	2.71e-07	-5.222.983
220934_s_at	5.86e-07	2.71e-07	5.222.746
216939_s_at	5.91e-07	2.73e-07	-5.221.203
200766_at	5.92e-07	2.73e-07	5.221.017
213323_s_at	5.96e-07	2.75e-07	-5.219.645
204966_at	5.96e-07	2.76e-07	-5.219.419
203366_at	5.99e-07	2.77e-07	-5.218.566

207613_s_at	5.99e-07	2.77e-07	-5.218.521
213197_at	5.99e-07	2.77e-07	-5.218.412
220039_s_at	5.99e-07	2.77e-07	5.218.283
217738_at	6.00e-07	2.77e-07	-5.218.099
215725_at	6.03e-07	2.79e-07	-5.217.158
206788_s_at	6.05e-07	2.80e-07	-521.656
217827_s_at	6.09e-07	2.82e-07	5.215.144
207860_at	6.10e-07	2.82e-07	-5.214.687
219580_s_at	6.12e-07	2.83e-07	5.213.995
217433_at	6.15e-07	2.84e-07	-521.326
200685_at	6.16e-07	2.85e-07	-5.212.913
212972_x_at	6.17e-07	2.85e-07	-5.212.581
210019_at	6.17e-07	2.85e-07	-5.212.567
220877_at	6.18e-07	2.86e-07	-5.212.158
206923_at	6.19e-07	2.86e-07	-5.211.873
207566_at	6.19e-07	2.87e-07	-5.211.584
217280_x_at	6.21e-07	2.87e-07	-5.211.203
221190_s_at	6.21e-07	2.87e-07	5.211.128
219672_at	6.22e-07	2.88e-07	-5.210.614
217619_x_at	6.26e-07	2.90e-07	-5.209.534
207303_at	6.27e-07	2.91e-07	-5.209.034
209690_s_at	6.29e-07	2.91e-07	-5.208.588
220009_at	6.31e-07	2.92e-07	-5.207.844
206983_at	6.35e-07	2.94e-07	5.206.701
215687_x_at	6.35e-07	2.94e-07	-5.206.539
218554_s_at	6.35e-07	2.94e-07	-5.206.398
206703_at	6.35e-07	2.94e-07	-5.206.397
218294_s_at	6.36e-07	2.95e-07	5.206.019
202489_s_at	6.39e-07	2.96e-07	5.205.231
221885_at	6.41e-07	2.97e-07	-520.459
205172_x_at	6.41e-07	2.97e-07	5.204.455
214015_at	6.42e-07	2.98e-07	-5.204.333
220923_s_at	6.45e-07	2.99e-07	-5.203.315
200677_at	6.46e-07	3.00e-07	5.203.022
208292_at	6.46e-07	3.00e-07	-520.295
211775_x_at	6.47e-07	3.00e-07	-5.202.737
220674_at	6.47e-07	3.00e-07	-5.202.467
211261_at	6.48e-07	3.01e-07	-5.202.212
205502_at	6.49e-07	3.01e-07	520.208
211929_at	6.49e-07	3.01e-07	5.201.818
204267_x_at	6.52e-07	3.03e-07	5.201.085
216294_s_at	6.53e-07	3.03e-07	-5.200.667
216889_s_at	6.57e-07	3.05e-07	-5.199.467
212308_at	6.60e-07	3.06e-07	5.198.677

215106_at	6.60e-07	3.06e-07	-5.198.527
205190_at	6.60e-07	3.07e-07	5.198.435
215170_s_at	6.61e-07	3.07e-07	5.198.344
219693_at	6.62e-07	3.07e-07	5.197.991
211451_s_at	6.64e-07	3.08e-07	-5.197.379
221210_s_at	6.64e-07	3.08e-07	5.197.298
218122_s_at	6.64e-07	3.08e-07	5.197.268
215520_at	6.64e-07	3.08e-07	-5.197.247
208592_s_at	6.64e-07	3.09e-07	-5.197.143
202751_at	6.65e-07	3.09e-07	5.196.891
210880_s_at	6.66e-07	3.09e-07	-5.196.689
215925_s_at	6.66e-07	3.10e-07	-519.649
219857_at	6.69e-07	3.11e-07	-5.195.697
217005_at	6.72e-07	3.12e-07	-5.194.859
204113_at	6.76e-07	3.14e-07	5.193.447
208102_s_at	6.76e-07	3.15e-07	-519.343
220433_at	6.79e-07	3.16e-07	-5.192.706
213002_at	6.81e-07	3.17e-07	5.191.944
215975_x_at	6.83e-07	3.18e-07	-5.191.404
206303_s_at	6.84e-07	3.18e-07	5.191.113
208711_s_at	6.88e-07	3.20e-07	-5.189.962
211145_x_at	6.89e-07	3.21e-07	-5.189.671
215735_s_at	6.90e-07	3.21e-07	-5.189.434
207237_at	6.97e-07	3.24e-07	-5.187.261
221979_at	7.01e-07	3.26e-07	5.186.059
204274_at	7.02e-07	3.27e-07	5.185.952
221284_s_at	7.06e-07	3.28e-07	-5.184.851
202553_s_at	7.10e-07	3.31e-07	5.183.476
211121_s_at	7.16e-07	3.33e-07	-5.181.976
216726_at	7.16e-07	3.33e-07	-5.181.901
202636_at	7.16e-07	3.33e-07	518.186
208996_s_at	7.16e-07	3.34e-07	-5.181.737
204888_s_at	7.19e-07	3.35e-07	-5.181.083
207489_at	7.20e-07	3.35e-07	-5.180.778
57715_at	7.23e-07	3.37e-07	-518.001
221086_s_at	7.23e-07	3.37e-07	-5.179.989
208118_x_at	7.25e-07	3.38e-07	5.179.401
208257_x_at	7.26e-07	3.38e-07	-5.179.112
214692_s_at	7.31e-07	3.41e-07	5.177.711
211641_x_at	7.31e-07	3.41e-07	-517.752
216423_at	7.32e-07	3.41e-07	-5.177.267
205897_at	7.34e-07	3.42e-07	-5.176.884
221771_s_at	7.34e-07	3.42e-07	-5.176.671
208552_at	7.37e-07	3.43e-07	-5.176.013

207887_s_at	7.41e-07	3.46e-07	-5.174.725
210182_at	7.42e-07	3.46e-07	-5.174.517
214321_at	7.45e-07	3.48e-07	517.371
221959_at	7.47e-07	3.49e-07	5.173.141
211565_at	7.50e-07	3.50e-07	-5.172.256
221790_s_at	7.51e-07	3.50e-07	-5.172.072
206800_at	7.51e-07	3.51e-07	-5.171.932
204158_s_at	7.54e-07	3.52e-07	5.171.382
217524_x_at	7.56e-07	3.53e-07	-5.170.747
212699_at	7.56e-07	3.53e-07	5.170.621
211104_s_at	7.62e-07	3.56e-07	-5.169.201
207691_x_at	7.63e-07	3.56e-07	5.168.745
207612_at	7.66e-07	3.58e-07	-5.168.045
215755_at	7.66e-07	3.58e-07	-5.167.899
201287_s_at	7.67e-07	3.58e-07	-5.167.615
216955_at	7.72e-07	3.61e-07	-5.166.333
220499_at	7.73e-07	3.61e-07	-516.611
213548_s_at	7.73e-07	3.61e-07	5.166.057
202527_s_at	7.73e-07	3.61e-07	5.166.027
221350_at	7.75e-07	3.62e-07	-5.165.475
215514_at	7.76e-07	3.62e-07	-5.165.359
216177_at	7.76e-07	3.63e-07	5.165.271
211891_s_at	7.77e-07	3.63e-07	-5.165.051
204841_s_at	7.79e-07	3.64e-07	5.164.479
211163_s_at	7.79e-07	3.64e-07	-5.164.413
207151_at	7.84e-07	3.66e-07	-5.163.293
215556_at	7.85e-07	3.67e-07	-5.163.029
202501_at	7.85e-07	3.67e-07	-5.162.771
209031_at	7.89e-07	3.69e-07	5.161.895
219104_at	7.91e-07	3.70e-07	5.161.462
205465_x_at	7.95e-07	3.72e-07	-5.160.392
208718_at	7.97e-07	3.73e-07	5.159.844
201423_s_at	7.97e-07	3.73e-07	515.981
208075_s_at	7.97e-07	3.73e-07	-5.159.788
205124_at	7.99e-07	3.74e-07	-5.159.293
206597_at	7.99e-07	3.74e-07	-5.159.206
216858_x_at	8.00e-07	3.74e-07	-5.159.076
216149_at	8.00e-07	3.74e-07	-5.159.045
205088_at	8.00e-07	3.74e-07	5.158.907
215286_s_at	8.01e-07	3.75e-07	5.158.639
214776_x_at	8.02e-07	3.75e-07	-5.158.412
208244_at	8.06e-07	3.77e-07	-5.157.314
217191_x_at	8.06e-07	3.78e-07	5.157.269
208725_at	8.10e-07	3.79e-07	5.156.414

52731_at	8.11e-07	3.80e-07	5.156.092
221786_at	8.14e-07	3.81e-07	5.155.429
219467_at	8.15e-07	3.82e-07	5.155.151
207778_at	8.15e-07	3.82e-07	-5.155.098
220657_at	8.17e-07	3.83e-07	-5.154.657
219959_at	8.17e-07	3.83e-07	-5.154.459
203995_at	8.21e-07	3.85e-07	-5.153.617
221406_s_at	8.21e-07	3.85e-07	5.153.437
201785_at	8.28e-07	3.88e-07	515.173
207226_at	8.34e-07	3.91e-07	-5.150.361
205056_s_at	8.35e-07	3.91e-07	-5.150.178
216708_x_at	8.36e-07	3.92e-07	-5.149.762
202756_s_at	8.38e-07	3.93e-07	-5.149.449
219835_at	8.38e-07	3.93e-07	-5.149.389
211269_s_at	8.38e-07	3.93e-07	-51.493
214630_at	8.38e-07	3.93e-07	-5.149.208
217581_at	8.40e-07	3.94e-07	-5.148.797
216545_at	8.41e-07	3.94e-07	-5.148.587
206230_at	8.42e-07	3.95e-07	-5.148.292
217379_at	8.42e-07	3.95e-07	514.829
216492_at	8.45e-07	3.96e-07	-5.147.599
204439_at	8.46e-07	3.97e-07	-514.728
212533_at	8.48e-07	3.98e-07	5.146.854
203834_s_at	8.55e-07	4.01e-07	-514.515
219464_at	8.57e-07	4.02e-07	-5.144.672
218201_at	8.59e-07	4.03e-07	5.144.226
222288_at	8.59e-07	4.03e-07	5.144.165
215327_at	8.60e-07	4.04e-07	-5.143.908
221162_at	8.64e-07	4.06e-07	-5.142.897
211535_s_at	8.68e-07	4.08e-07	-5.142.026
207133_x_at	8.73e-07	4.10e-07	-514.088
215406_at	8.76e-07	4.12e-07	-5.140.092
203042_at	8.77e-07	4.12e-07	-5.140.024
200886_s_at	8.81e-07	4.14e-07	5.138.964
208000_at	8.90e-07	4.18e-07	-5.136.956
217525_at	8.96e-07	4.21e-07	-5.135.548
206889_at	8.98e-07	4.22e-07	-5.135.137
212047_s_at	9.01e-07	4.23e-07	-5.134.528
215889_at	9.02e-07	4.24e-07	-5.134.147
215586_at	9.04e-07	4.25e-07	-5.133.786
208273_at	9.04e-07	4.25e-07	-5.133.724
213029_at	9.09e-07	4.27e-07	-5.132.682
210723_x_at	9.09e-07	4.27e-07	-5.132.608
213623_at	9.09e-07	4.27e-07	5.132.596

210611_s_at	9.12e-07	4.29e-07	5.131.819
201880_at	9.15e-07	4.30e-07	5.131.178
201310_s_at	9.17e-07	4.31e-07	513.075
203006_at	9.18e-07	4.32e-07	5.130.549
209244_s_at	9.20e-07	4.33e-07	-5.130.095
213686_at	9.24e-07	4.35e-07	5.129.295
207288_at	9.29e-07	4.37e-07	-5.128.124
216414_at	9.34e-07	4.40e-07	-5.126.932
217566_s_at	9.38e-07	4.42e-07	-51.261
205583_s_at	9.41e-07	4.43e-07	-5.125.541
205420_at	9.47e-07	4.46e-07	5.124.222
206001_at	9.51e-07	4.47e-07	-5.123.443
212916_at	9.52e-07	4.48e-07	-5.123.189
219976_at	9.56e-07	4.50e-07	-5.122.324
221366_at	9.56e-07	4.50e-07	-5.122.216
208761_s_at	9.61e-07	4.53e-07	5.121.095
219190_s_at	9.62e-07	4.53e-07	-5.121.012
212209_at	9.63e-07	4.53e-07	5.120.819
211790_s_at	9.64e-07	4.54e-07	-5.120.549
222226_at	9.65e-07	4.55e-07	-512.032
204191_at	9.66e-07	4.55e-07	-5.120.117
204319_s_at	9.66e-07	4.55e-07	5.120.045
221105_at	9.70e-07	4.57e-07	-5.119.175
215426_at	9.71e-07	4.58e-07	-5.118.973
208183_at	9.80e-07	4.62e-07	-511.708
220320_at	9.81e-07	4.62e-07	-5.116.917
219398_at	9.90e-07	4.67e-07	5.115.067
211674_x_at	9.92e-07	4.68e-07	511.456
215320_at	9.95e-07	4.69e-07	-5.114.052
208779_x_at	1.00e-06	4.71e-07	5.113.064
201147_s_at	1.00e-06	4.71e-07	5.113.017
214727_at	1.00e-06	4.72e-07	5.112.807
203193_at	1.00e-06	4.74e-07	-5.111.968
216962_at	1.01e-06	4.75e-07	-5.111.404
208590_x_at	1.01e-06	4.76e-07	-5.111.153
202059_s_at	1.01e-06	4.77e-07	-5.110.854
212860_at	1.02e-06	4.80e-07	5.109.523
211105_s_at	1.02e-06	4.80e-07	-5.109.326
214869_x_at	1.02e-06	4.80e-07	5.109.256
210415_s_at	1.02e-06	4.81e-07	5.109.159
201269_s_at	1.02e-06	4.81e-07	-5.109.129
211021_s_at	1.02e-06	4.82e-07	-5.108.639
216491_x_at	1.02e-06	4.83e-07	-5.108.288
207595_s_at	1.02e-06	4.84e-07	-5.107.815

213098_at	1.03e-06	4.84e-07	510.762
209896_s_at	1.03e-06	4.85e-07	5.107.304
202523_s_at	1.03e-06	4.86e-07	-5.107.096
202484_s_at	1.03e-06	4.86e-07	5.106.937
213003_s_at	1.03e-06	4.87e-07	-510.635
220742_s_at	1.03e-06	4.88e-07	5.105.942
206193_s_at	1.04e-06	4.92e-07	-5.104.676
207615_s_at	1.04e-06	4.93e-07	-5.104.264
221991_at	1.04e-06	4.93e-07	-5.103.922
214932_at	1.05e-06	4.95e-07	-5.103.246
200660_at	1.05e-06	4.95e-07	5.103.213
209211_at	1.05e-06	4.97e-07	5.102.355
205236_x_at	1.05e-06	4.98e-07	-5.102.058
222347_at	1.06e-06	4.99e-07	5.101.613
208991_at	1.06e-06	4.99e-07	-5.101.477
208446_s_at	1.06e-06	5.01e-07	-5.100.818
204926_at	1.06e-06	5.02e-07	-510.043
207944_at	1.06e-06	5.03e-07	-5.100.061
207471_at	1.07e-06	5.04e-07	-5.099.606
203084_at	1.07e-06	5.06e-07	-5.098.766
216535_at	1.07e-06	5.07e-07	-5.098.308
207969_x_at	1.08e-06	5.11e-07	-5.096.835
209664_x_at	1.08e-06	5.12e-07	-5.096.543
207129_at	1.08e-06	5.13e-07	-5.095.951
208632_at	1.09e-06	5.16e-07	5.094.883
219887_at	1.09e-06	5.16e-07	-5.094.843
209074_s_at	1.09e-06	5.17e-07	-5.094.742
216013_at	1.10e-06	5.19e-07	-5.093.618
209224_s_at	1.10e-06	5.20e-07	5.093.444
217202_s_at	1.10e-06	5.21e-07	5.092.936
211432_s_at	1.10e-06	5.22e-07	5.092.764
201234_at	1.11e-06	5.24e-07	5.091.875
213219_at	1.11e-06	5.24e-07	-5.091.677
217342_x_at	1.11e-06	5.26e-07	-5.091.213
217361_at	1.11e-06	5.26e-07	-5.091.116
201769_at	1.12e-06	5.31e-07	-50.893
213672_at	1.13e-06	5.33e-07	5.088.304
219825_at	1.13e-06	5.35e-07	5.087.773
214407_x_at	1.13e-06	5.35e-07	-5.087.694
206203_at	1.13e-06	5.37e-07	-5.086.899
217515_s_at	1.14e-06	5.38e-07	-5.086.544
208059_at	1.14e-06	5.39e-07	-5.086.281
215365_at	1.14e-06	5.39e-07	-5.086.279
221340_at	1.14e-06	5.39e-07	-5.086.201

222363_at	1.14e-06	5.39e-07	-5.086.048
206826_at	1.14e-06	5.42e-07	-5.085.198
212588_at	1.15e-06	5.45e-07	-5.083.883
217570_x_at	1.15e-06	5.47e-07	-5.083.416
212908_at	1.16e-06	5.49e-07	-5.082.512
220142_at	1.16e-06	5.50e-07	-5.082.049
219879_s_at	1.16e-06	5.51e-07	-5.081.641
200852_x_at	1.16e-06	5.52e-07	5.081.398
212714_at	1.17e-06	5.53e-07	-5.080.914
205946_at	1.17e-06	5.56e-07	-5.079.887
204973_at	1.17e-06	5.57e-07	-5.079.752
216854_at	1.18e-06	5.58e-07	-5.079.205
207679_at	1.19e-06	5.63e-07	-5.077.581
211433_x_at	1.19e-06	5.63e-07	-5.077.374
202038_at	1.19e-06	5.67e-07	5.076.107
207959_s_at	1.20e-06	5.69e-07	-5.075.392
206874_s_at	1.20e-06	5.69e-07	5.075.338
212204_at	1.20e-06	5.69e-07	5.075.301
211809_x_at	1.20e-06	5.69e-07	-5.075.216
35254_at	1.20e-06	5.71e-07	5.074.527
AFFX-PheX-M_at	1.20e-06	5.72e-07	-5.074.153
218302_at	1.20e-06	5.73e-07	5.074.072
220689_at	1.21e-06	5.73e-07	-5.073.726
218693_at	1.21e-06	5.75e-07	5.073.241
210740_s_at	1.21e-06	5.76e-07	5.072.706
201768_s_at	1.21e-06	5.77e-07	-5.072.658
202235_at	1.21e-06	5.77e-07	-5.072.388
215941_at	1.21e-06	5.78e-07	-5.072.281
204582_s_at	1.22e-06	5.80e-07	-5.071.463
206388_at	1.23e-06	5.83e-07	-5.070.566
211661_x_at	1.23e-06	5.83e-07	-5.070.529
216531_at	1.23e-06	5.85e-07	-5.069.606
203206_at	1.23e-06	5.86e-07	5.069.535
211302_s_at	1.23e-06	5.87e-07	-5.068.918
215836_s_at	1.25e-06	5.93e-07	-5.067.125
214315_x_at	1.25e-06	5.93e-07	5.067.065
210879_s_at	1.25e-06	5.96e-07	5.065.953
210167_s_at	1.25e-06	5.96e-07	-5.065.861
217483_at	1.26e-06	5.98e-07	-5.065.427
220684_at	1.26e-06	5.99e-07	-506.511
202386_s_at	1.26e-06	5.99e-07	-5.065.085
206252_s_at	1.26e-06	5.99e-07	-5.065.079
217502_at	1.26e-06	6.00e-07	-5.064.673
206083_at	1.26e-06	6.02e-07	-5.064.123

222342_at	1.26e-06	6.02e-07	-5.063.941
206505_at	1.27e-06	6.03e-07	-5.063.682
206296_x_at	1.27e-06	6.03e-07	-5.063.473
AFFX-r2-Bs-dap-5_at	1.27e-06	6.05e-07	-5.063.107
213852_at	1.28e-06	6.08e-07	5.061.954
200745_s_at	1.28e-06	6.08e-07	506.188
206047_at	1.28e-06	6.08e-07	-5.061.844
215861_at	1.28e-06	6.11e-07	-5.060.976
217306_at	1.28e-06	6.12e-07	-5.060.786
208468_at	1.29e-06	6.14e-07	-5.060.143
215074_at	1.29e-06	6.16e-07	-5.059.427
210868_s_at	1.31e-06	6.23e-07	-5.057.192
211187_at	1.31e-06	6.25e-07	-5.056.534
210781_x_at	1.32e-06	6.28e-07	-5.055.591
221397_at	1.32e-06	6.28e-07	-5.055.288
222314_x_at	1.32e-06	6.31e-07	-5.054.472
202224_at	1.33e-06	6.32e-07	-5.054.176
217410_at	1.33e-06	6.32e-07	-5.054.064
204702_s_at	1.33e-06	6.33e-07	5.053.857
214839_at	1.33e-06	6.34e-07	-5.053.656
210934_at	1.33e-06	6.35e-07	-5.053.119
202444_s_at	1.33e-06	6.35e-07	-505.307
209785_s_at	1.33e-06	6.36e-07	5.052.989
206725_x_at	1.33e-06	6.37e-07	-5.052.596
204300_at	1.35e-06	6.44e-07	-5.050.346
202375_at	1.35e-06	6.45e-07	-5.050.139
214553_s_at	1.36e-06	6.49e-07	5.048.815
216711_s_at	1.36e-06	6.51e-07	-5.048.299
204429_s_at	1.36e-06	6.51e-07	5.048.017
211240_x_at	1.37e-06	6.52e-07	-5.047.707
211789_s_at	1.37e-06	6.53e-07	-504.746
213297_at	1.37e-06	6.54e-07	-5.047.198
202622_s_at	1.37e-06	6.54e-07	5.047.138
211772_x_at	1.38e-06	6.57e-07	-5.046.227
217153_at	1.38e-06	6.59e-07	-5.045.659
209643_s_at	1.38e-06	6.60e-07	-5.045.402
221119_at	1.38e-06	6.61e-07	-5.044.986
211237_s_at	1.39e-06	6.63e-07	5.044.585
220211_at	1.39e-06	6.63e-07	-5.044.448
221849_s_at	1.40e-06	6.68e-07	5.042.881
215060_at	1.41e-06	6.72e-07	-5.041.718
220121_at	1.41e-06	6.73e-07	5.041.589
205888_s_at	1.41e-06	6.73e-07	-5.041.364
216707_at	1.41e-06	6.75e-07	-5.040.839

213110_s_at	1.41e-06	6.76e-07	5.040.479
217535_at	1.41e-06	6.77e-07	-5.040.369
209858_x_at	1.42e-06	6.79e-07	5.039.535
217751_at	1.42e-06	6.80e-07	-5.039.325
204202_at	1.43e-06	6.82e-07	5.038.818
210835_s_at	1.43e-06	6.85e-07	-5.037.767
208058_s_at	1.43e-06	6.86e-07	-5.037.674
200081_s_at	1.44e-06	6.87e-07	5.037.372
212664_at	1.44e-06	6.89e-07	5.036.837
221613_s_at	1.44e-06	6.89e-07	5.036.654
221256_s_at	1.45e-06	6.93e-07	-5.035.472
206008_at	1.45e-06	6.95e-07	-5.035.021
218929_at	1.46e-06	6.98e-07	-5.033.974
210102_at	1.46e-06	7.01e-07	5.033.297
220996_s_at	1.47e-06	7.02e-07	-5.032.792
211149_at	1.47e-06	7.03e-07	-5.032.651
220267_at	1.47e-06	7.03e-07	-5.032.571
207453_s_at	1.48e-06	7.07e-07	-5.031.499
205278_at	1.49e-06	7.14e-07	5.029.461
206858_s_at	1.49e-06	7.15e-07	502.906
203167_at	1.50e-06	7.17e-07	5.028.651
215376_at	1.50e-06	7.17e-07	502.863
202245_at	1.50e-06	7.19e-07	5.028.173
220379_at	1.50e-06	7.19e-07	-5.028.014
207025_at	1.50e-06	7.21e-07	-5.027.493
213015_at	1.51e-06	7.25e-07	5.026.458
217034_at	1.51e-06	7.25e-07	-5.026.316
215061_at	1.52e-06	7.26e-07	-5.025.997
206905_s_at	1.52e-06	7.27e-07	-5.025.954
211178_s_at	1.52e-06	7.27e-07	-5.025.903
204214_s_at	1.52e-06	7.28e-07	5.025.572
217741_s_at	1.52e-06	7.28e-07	-5.025.522
218725_at	1.52e-06	7.30e-07	-5.024.894
206554_x_at	1.53e-06	7.33e-07	5.024.199
214380_at	1.53e-06	7.34e-07	-5.023.906
203841_x_at	1.54e-06	7.37e-07	-5.023.167
208519_x_at	1.54e-06	7.40e-07	-5.022.362
208013_s_at	1.54e-06	7.40e-07	-5.022.107
211626_x_at	1.54e-06	7.41e-07	-5.021.937
220412_x_at	1.55e-06	7.43e-07	-5.021.484
211209_x_at	1.55e-06	7.43e-07	-5.021.387
210401_at	1.55e-06	7.44e-07	-5.021.165
215828_at	1.55e-06	7.44e-07	-5.021.061
210564_x_at	1.56e-06	7.49e-07	-5.019.905

203875_at	1.58e-06	7.56e-07	5.017.804
208958_at	1.58e-06	7.60e-07	5.016.841
211686_s_at	1.58e-06	7.60e-07	5.016.695
213641_at	1.59e-06	7.62e-07	-501.619
206971_at	1.59e-06	7.63e-07	-5.016.054
208888_s_at	1.59e-06	7.64e-07	-5.015.632
210744_s_at	1.59e-06	7.65e-07	-501.542
215023_s_at	1.60e-06	7.67e-07	5.014.828
215290_at	1.60e-06	7.69e-07	-5.014.434
221323_at	1.61e-06	7.72e-07	-5.013.717
212603_at	1.61e-06	7.74e-07	-5.013.198
207248_at	1.62e-06	7.77e-07	-5.012.443
214586_at	1.62e-06	7.77e-07	5.012.392
207384_at	1.62e-06	7.77e-07	-5.012.306
202586_at	1.62e-06	7.79e-07	5.011.915
221016_s_at	1.62e-06	7.79e-07	5.011.805
212981_s_at	1.62e-06	7.80e-07	5.011.637
208256_at	1.63e-06	7.82e-07	-5.010.931
205791_x_at	1.64e-06	7.88e-07	500.942
208773_s_at	1.64e-06	7.89e-07	5.009.322
212624_s_at	1.65e-06	7.92e-07	5.008.549
202732_at	1.65e-06	7.94e-07	-5.007.873
208909_at	1.65e-06	7.95e-07	5.007.536
210393_at	1.66e-06	7.97e-07	5.007.226
211510_s_at	1.66e-06	7.97e-07	-5.007.209
220256_s_at	1.66e-06	7.97e-07	-5.007.159
206991_s_at	1.67e-06	8.02e-07	-5.005.856
213900_at	1.67e-06	8.04e-07	-5.005.483
34206_at	1.68e-06	8.07e-07	-5.004.609
203761_at	1.68e-06	8.08e-07	-5.004.456
214649_s_at	1.68e-06	8.08e-07	5.004.299
211835_at	1.68e-06	8.09e-07	-5.004.111
212570_at	1.69e-06	8.16e-07	-5.002.387
218531_at	1.70e-06	8.19e-07	-5.001.598
211168_s_at	1.71e-06	8.22e-07	5.000.874
217113_at	1.71e-06	8.22e-07	-5.000.785
206657_s_at	1.71e-06	8.24e-07	-5.000.481
206331_at	1.71e-06	8.24e-07	5.000.434
214975_s_at	1.72e-06	8.28e-07	4.999.421
218143_s_at	1.72e-06	8.28e-07	4.999.332
206217_at	1.72e-06	8.29e-07	-4.999.069
207873_x_at	1.72e-06	8.31e-07	-4.998.722
204084_s_at	1.72e-06	8.31e-07	-499.857
212070_at	1.73e-06	8.35e-07	4.997.567

220104_at	1.74e-06	8.38e-07	499.699
215151_at	1.74e-06	8.39e-07	-4.996.617
216862_s_at	1.76e-06	8.47e-07	-4.994.878
220819_at	1.76e-06	8.50e-07	-4.993.937
222030_at	1.76e-06	8.51e-07	-4.993.865
214477_at	1.77e-06	8.54e-07	-4.993.122
204348_s_at	1.77e-06	8.55e-07	-4.992.884
208655_at	1.78e-06	8.57e-07	-4.992.373
221372_s_at	1.78e-06	8.57e-07	-4.992.335
208526_at	1.78e-06	8.58e-07	-4.992.206
205938_at	1.79e-06	8.63e-07	4.991.033
205772_s_at	1.79e-06	8.64e-07	-4.990.631
210937_s_at	1.79e-06	8.65e-07	-4.990.505
208020_s_at	1.79e-06	8.65e-07	-4.990.447
216432_at	1.79e-06	8.65e-07	-4.990.422
220444_at	1.80e-06	8.68e-07	4.989.731
210110_x_at	1.80e-06	8.68e-07	498.969
215402_at	1.80e-06	8.69e-07	-4.989.573
215364_s_at	1.80e-06	8.69e-07	-4.989.565
208071_s_at	1.80e-06	8.72e-07	-49.889
217788_s_at	1.81e-06	8.75e-07	4.988.187
218934_s_at	1.81e-06	8.75e-07	-4.988.098
35179_at	1.82e-06	8.78e-07	4.987.423
220852_at	1.82e-06	8.80e-07	-4.987.037
217939_s_at	1.83e-06	8.82e-07	4.986.396
AFFX-DapX-5_at	1.83e-06	8.83e-07	-4.986.279
215495_s_at	1.83e-06	8.86e-07	-4.985.636
202833_s_at	1.83e-06	8.87e-07	-4.985.432
210560_at	1.85e-06	8.95e-07	-4.983.485
206551_x_at	1.86e-06	8.99e-07	-498.259
214659_x_at	1.86e-06	9.01e-07	4.982.215
218960_at	1.87e-06	9.03e-07	-4.981.774
220314_at	1.89e-06	9.14e-07	-4.979.154
221346_at	1.89e-06	9.14e-07	-4.979.145
212221_x_at	1.89e-06	9.15e-07	-4.978.958
213862_at	1.89e-06	9.16e-07	-497.889
220255_at	1.90e-06	9.18e-07	4.978.335
201324_at	1.91e-06	9.23e-07	4.977.238
208105_at	1.92e-06	9.27e-07	-4.976.301
204908_s_at	1.92e-06	9.28e-07	-4.976.134
216705_s_at	1.93e-06	9.32e-07	497.523
214147_at	1.93e-06	9.34e-07	-4.974.899
214249_at	1.94e-06	9.37e-07	-4.974.168
200719_at	1.95e-06	9.43e-07	4.972.828

207827_x_at	1.95e-06	9.43e-07	-4.972.822
215172_at	1.96e-06	9.47e-07	4.972.046
202879_s_at	1.96e-06	9.48e-07	-497.184
221947_at	1.96e-06	9.49e-07	-4.971.535
215511_at	1.97e-06	9.52e-07	-4.970.942
219552_at	1.97e-06	9.52e-07	-4.970.942
213834_at	1.99e-06	9.63e-07	-4.968.601
217937_s_at	2.00e-06	9.68e-07	-4.967.504
205462_s_at	2.01e-06	9.75e-07	4.965.941
207282_s_at	2.01e-06	9.76e-07	-4.965.805
221811_at	2.01e-06	9.76e-07	-4.965.785
222092_at	2.02e-06	9.77e-07	-4.965.558
208048_at	2.02e-06	9.79e-07	-4.965.119
209819_at	2.02e-06	9.80e-07	-4.965.055
50277_at	2.02e-06	9.80e-07	4.965.002
202416_at	2.02e-06	9.81e-07	4.964.701
215247_at	2.03e-06	9.85e-07	4.963.892
205131_x_at	2.03e-06	9.86e-07	-496.379
213842_x_at	2.03e-06	9.86e-07	4.963.775
210279_at	2.04e-06	9.89e-07	-4.963.055
215979_s_at	2.05e-06	9.93e-07	-4.962.333
214940_s_at	2.05e-06	9.95e-07	-4.961.778
204513_s_at	2.06e-06	9.98e-07	4.961.212
209007_s_at	2.07e-06	1.00e-06	-4.960.092
206079_at	2.07e-06	1.00e-06	4.960.057
212976_at	2.08e-06	1.01e-06	4.959.185
216482_x_at	2.08e-06	1.01e-06	-4.958.682
222045_s_at	2.09e-06	1.01e-06	4.958.255
209493_at	2.09e-06	1.01e-06	495.794
213827_at	2.10e-06	1.02e-06	4.957.126
208041_at	2.11e-06	1.02e-06	-4.956.159
201762_s_at	2.11e-06	1.02e-06	495.612
216704_at	2.11e-06	1.02e-06	-4.956.068
215857_at	2.11e-06	1.03e-06	-4.955.567
212303_x_at	2.11e-06	1.03e-06	4.955.436
205419_at	2.12e-06	1.03e-06	-4.954.784
220862_s_at	2.12e-06	1.03e-06	-4.954.548
207630_s_at	2.13e-06	1.03e-06	-4.954.017
221301_at	2.13e-06	1.03e-06	-4.953.872
213244_at	2.13e-06	1.04e-06	4.953.649
215193_x_at	2.14e-06	1.04e-06	-4.952.778
215555_at	2.15e-06	1.04e-06	-4.952.304
200091_s_at	2.15e-06	1.05e-06	4.951.447
208192_at	2.16e-06	1.05e-06	-4.951.137

216778_s_at	2.16e-06	1.05e-06	-4.951.118
210443_x_at	2.16e-06	1.05e-06	-4.950.602
219595_at	2.16e-06	1.05e-06	495.046
218113_at	2.17e-06	1.06e-06	494.939
218874_s_at	2.19e-06	1.06e-06	494.817
221404_at	2.20e-06	1.07e-06	-4.946.718
218601_at	2.21e-06	1.07e-06	-4.946.429
213919_at	2.21e-06	1.07e-06	-4.946.414
215119_at	2.21e-06	1.07e-06	-4.946.054
221280_s_at	2.21e-06	1.08e-06	4.945.927
216240_at	2.22e-06	1.08e-06	-4.945.467
221905_at	2.23e-06	1.09e-06	-4.943.748
219773_at	2.24e-06	1.09e-06	4.942.717
217025_s_at	2.25e-06	1.09e-06	4.942.609
219336_s_at	2.25e-06	1.09e-06	4.942.367
204674_at	2.25e-06	1.10e-06	-494.177
221013_s_at	2.26e-06	1.10e-06	-494.155
209961_s_at	2.27e-06	1.10e-06	-4.940.602
220072_at	2.27e-06	1.11e-06	494.026
203873_at	2.27e-06	1.11e-06	-4.940.236
212824_at	2.28e-06	1.11e-06	493.953
211637_x_at	2.28e-06	1.11e-06	-4.939.458
212383_at	2.28e-06	1.11e-06	4.939.424
213727_x_at	2.28e-06	1.11e-06	4.938.932
208916_at	2.29e-06	1.11e-06	4.938.754
204624_at	2.29e-06	1.11e-06	-4.938.561
216982_x_at	2.29e-06	1.12e-06	-4.937.915
208508_s_at	2.31e-06	1.13e-06	-493.654
213257_at	2.31e-06	1.13e-06	-4.936.451
AFFX-ThrX-M_at	2.32e-06	1.13e-06	-4.936.028
219370_at	2.32e-06	1.13e-06	-4.935.738
212480_at	2.36e-06	1.15e-06	4.932.092
215709_at	2.36e-06	1.15e-06	-4.931.674
205413_at	2.37e-06	1.15e-06	493.141
215415_s_at	2.37e-06	1.16e-06	-4.931.111
211258_s_at	2.38e-06	1.16e-06	-4.930.174
216800_at	2.38e-06	1.16e-06	-4.930.169
220024_s_at	2.38e-06	1.16e-06	-4.930.011
209752_at	2.38e-06	1.16e-06	4.929.918
201678_s_at	2.39e-06	1.16e-06	4.929.585
209574_s_at	2.39e-06	1.17e-06	4.929.326
207914_x_at	2.39e-06	1.17e-06	-492.917
217186_at	2.39e-06	1.17e-06	-4.929.065
214441_at	2.39e-06	1.17e-06	49.289

210123_s_at	2.40e-06	1.17e-06	-4.928.656
201627_s_at	2.40e-06	1.17e-06	-4.928.527
218362_s_at	2.43e-06	1.19e-06	4.925.794
206594_at	2.43e-06	1.19e-06	4.925.497
209029_at	2.45e-06	1.19e-06	4.924.373
214935_at	2.45e-06	1.20e-06	-4.924.035
211763_s_at	2.45e-06	1.20e-06	4.923.676
AFFX-PheX-3_at	2.46e-06	1.20e-06	-4.923.346
209060_x_at	2.46e-06	1.20e-06	4.922.724
212066_s_at	2.49e-06	1.22e-06	4.920.771
204676_at	2.49e-06	1.22e-06	4.920.527
209391_at	2.50e-06	1.22e-06	4.919.901
213912_at	2.50e-06	1.22e-06	4.919.535
221337_s_at	2.50e-06	1.22e-06	-4.919.295
214289_at	2.51e-06	1.23e-06	-491.868
215361_at	2.52e-06	1.23e-06	-4.918.345
219610_at	2.52e-06	1.23e-06	4.917.627
220336_s_at	2.52e-06	1.23e-06	-4.917.613
220625_s_at	2.53e-06	1.23e-06	-4.917.482
203770_s_at	2.53e-06	1.24e-06	-4.916.772
200939_s_at	2.54e-06	1.24e-06	-4.916.097
211808_s_at	2.55e-06	1.24e-06	-491.581
206821_x_at	2.55e-06	1.25e-06	4.915.732
206082_at	2.55e-06	1.25e-06	4.915.486
AFFX-LysX-M_at	2.55e-06	1.25e-06	-4.915.354
212213_x_at	2.55e-06	1.25e-06	4.915.181
211793_s_at	2.55e-06	1.25e-06	4.915.074
201110_s_at	2.57e-06	1.26e-06	-4.913.327
205776_at	2.57e-06	1.26e-06	-4.913.307
211546_x_at	2.58e-06	1.26e-06	-4.912.995
200816_s_at	2.58e-06	1.26e-06	-4.912.852
214650_x_at	2.58e-06	1.26e-06	-4.912.655
215623_x_at	2.58e-06	1.26e-06	4.912.647
221591_s_at	2.58e-06	1.27e-06	491.243
211023_at	2.59e-06	1.27e-06	4.912.057
202169_s_at	2.59e-06	1.27e-06	4.911.732
220624_s_at	2.60e-06	1.27e-06	-491.147
204647_at	2.60e-06	1.27e-06	4.911.322
203977_at	2.61e-06	1.28e-06	4.910.631
216406_at	2.61e-06	1.28e-06	-4.910.239
219628_at	2.61e-06	1.28e-06	-4.910.063
208246_x_at	2.62e-06	1.28e-06	-4.909.748
214018_at	2.64e-06	1.29e-06	-4.907.793
206530_at	2.67e-06	1.31e-06	-4.905.385

211097_s_at	2.67e-06	1.31e-06	-4.905.264
210946_at	2.68e-06	1.31e-06	4.904.759
208890_s_at	2.68e-06	1.31e-06	-4.904.684
211650_x_at	2.69e-06	1.32e-06	-4.904.127
201931_at	2.69e-06	1.32e-06	-4.904.113
212905_at	2.69e-06	1.32e-06	4.904.061
216377_x_at	2.70e-06	1.32e-06	-49.032
216851_at	2.70e-06	1.32e-06	-4.902.902
209892_at	2.70e-06	1.33e-06	4.902.769
220071_x_at	2.71e-06	1.33e-06	-4.902.575
206462_s_at	2.71e-06	1.33e-06	-4.902.461
202822_at	2.71e-06	1.33e-06	4.902.403
217543_s_at	2.71e-06	1.33e-06	-4.902.372
211181_x_at	2.72e-06	1.33e-06	-4.901.351
201064_s_at	2.72e-06	1.34e-06	4.901.183
211286_x_at	2.72e-06	1.34e-06	-4.901.128
206150_at	2.72e-06	1.34e-06	-4.901.102
214006_s_at	2.73e-06	1.34e-06	4.900.324
45749_at	2.74e-06	1.34e-06	-4.900.083
217700_at	2.74e-06	1.35e-06	-4.899.727
216846_at	2.75e-06	1.35e-06	-4.899.146
221962_s_at	2.76e-06	1.35e-06	4.898.472
214643_x_at	2.76e-06	1.36e-06	4.898.202
210152_at	2.76e-06	1.36e-06	-4.898.086
203865_s_at	2.76e-06	1.36e-06	-4.897.987
212875_s_at	2.76e-06	1.36e-06	-4.897.842
217236_x_at	2.77e-06	1.36e-06	-4.897.722
207132_x_at	2.77e-06	1.36e-06	4.897.419
220351_at	2.78e-06	1.36e-06	-4.896.897
207623_at	2.78e-06	1.37e-06	-4.896.465
207150_at	2.79e-06	1.37e-06	-4.895.855
215480_at	2.79e-06	1.37e-06	-4.895.592
204426_at	2.80e-06	1.38e-06	4.895
221181_at	2.82e-06	1.38e-06	-4.893.856
216077_s_at	2.82e-06	1.39e-06	-4.893.489
210813_s_at	2.86e-06	1.41e-06	4.890.434
216642_at	2.87e-06	1.41e-06	-4.890.158
216139_s_at	2.87e-06	1.41e-06	-4.889.895
211570_s_at	2.87e-06	1.41e-06	-4.889.755
201849_at	2.87e-06	1.41e-06	-4.889.706
210977_s_at	2.87e-06	1.41e-06	-4.889.561
210114_at	2.87e-06	1.41e-06	-4.889.491
222348_at	2.88e-06	1.42e-06	-4.888.987
AFFX-r2-Bs-phe-M_at	2.89e-06	1.42e-06	-4.888.246

214598_at	2.90e-06	1.43e-06	-4.887.635
215389_s_at	2.91e-06	1.43e-06	-4.886.887
AFFX-HUMISGF3A/M9	2.91e-06	1.43e-06	4.886.478
211735_x_at	2.91e-06	1.43e-06	-4.886.409
205011_at	2.93e-06	1.44e-06	-4.885.564
220818_s_at	2.93e-06	1.44e-06	-4.885.557
219285_s_at	2.94e-06	1.45e-06	-4.884.611
201609_x_at	2.95e-06	1.45e-06	4.883.991
205490_x_at	2.95e-06	1.45e-06	-4.883.703
221332_at	2.96e-06	1.46e-06	-4.883.414
221279_at	2.96e-06	1.46e-06	4.883.339
211310_at	2.96e-06	1.46e-06	-4.883.336
209245_s_at	2.99e-06	1.47e-06	-488.069
215850_s_at	3.00e-06	1.48e-06	-4.880.344
204813_at	3.01e-06	1.48e-06	-4.879.803
219781_s_at	3.01e-06	1.48e-06	-4.879.258
220207_at	3.02e-06	1.49e-06	-4.878.887
214467_at	3.02e-06	1.49e-06	-4.878.685
213624_at	3.02e-06	1.49e-06	-4.878.629
220944_at	3.03e-06	1.49e-06	-4.878.203
207862_at	3.10e-06	1.53e-06	-4.873.537
219024_at	3.10e-06	1.53e-06	4.873.453
219657_s_at	3.11e-06	1.53e-06	4.872.708
217660_at	3.12e-06	1.54e-06	-4.871.632
221427_s_at	3.15e-06	1.55e-06	-4.870.018
205714_s_at	3.15e-06	1.55e-06	-4.869.688
215472_at	3.16e-06	1.56e-06	-4.869.303
205709_s_at	3.16e-06	1.56e-06	4.868.954
217954_s_at	3.16e-06	1.56e-06	4.868.927
221876_at	3.17e-06	1.56e-06	-4.868.612
204009_s_at	3.17e-06	1.56e-06	4.868.415
219134_at	3.18e-06	1.57e-06	4.868.049
202509_s_at	3.18e-06	1.57e-06	-4.867.579
207569_at	3.18e-06	1.57e-06	-4.867.439
218372_at	3.19e-06	1.57e-06	4.867.291
205923_at	3.20e-06	1.58e-06	-4.866.267
208267_at	3.20e-06	1.58e-06	-4.866.257
202631_s_at	3.20e-06	1.58e-06	4.866.232
214367_at	3.20e-06	1.58e-06	-4.866.054
209225_x_at	3.21e-06	1.58e-06	4.865.881
220884_at	3.21e-06	1.59e-06	-4.865.583
209828_s_at	3.22e-06	1.59e-06	-4.864.723
212040_at	3.23e-06	1.60e-06	-4.864.323
221493_at	3.23e-06	1.60e-06	-4.864.314

209163_at	3.24e-06	1.60e-06	4.863.816
206311_s_at	3.24e-06	1.60e-06	486.376
202380_s_at	3.24e-06	1.60e-06	-486.369
220570_at	3.24e-06	1.60e-06	-4.863.498
205723_at	3.24e-06	1.60e-06	-4.863.321
203633_at	3.25e-06	1.61e-06	-486.303
214539_at	3.25e-06	1.61e-06	-4.862.792
216371_at	3.27e-06	1.62e-06	-4.861.722
213995_at	3.27e-06	1.62e-06	4.861.399
217732_s_at	3.27e-06	1.62e-06	4.861.266
201605_x_at	3.27e-06	1.62e-06	-4.861.206
219095_at	3.28e-06	1.62e-06	-4.860.664
208353_x_at	3.29e-06	1.63e-06	-4.860.332
211476_at	3.31e-06	1.63e-06	-4.859.229
206523_at	3.31e-06	1.64e-06	-4.859.056
210749_x_at	3.32e-06	1.64e-06	4.858.494
217654_at	3.32e-06	1.64e-06	-4.858.415
AFFX-r2-Ec-bioD-3_at	3.33e-06	1.65e-06	-4.857.909
206124_s_at	3.34e-06	1.65e-06	-4.857.264
210189_at	3.34e-06	1.65e-06	4.857.092
213603_s_at	3.34e-06	1.65e-06	-4.856.903
211136_s_at	3.34e-06	1.65e-06	485.679
212368_at	3.34e-06	1.65e-06	4.856.665
207500_at	3.34e-06	1.66e-06	-4.856.614
217488_x_at	3.35e-06	1.66e-06	-4.856.316
214400_at	3.35e-06	1.66e-06	-4.856.275
203067_at	3.36e-06	1.66e-06	4.855.655
219320_at	3.36e-06	1.67e-06	4.855.241
213160_at	3.42e-06	1.70e-06	-4.851.647
215257_at	3.43e-06	1.70e-06	-4.851.275
214447_at	3.45e-06	1.71e-06	-4.849.803
206969_at	3.46e-06	1.71e-06	-4.849.419
208384_s_at	3.46e-06	1.71e-06	4.849.243
209746_s_at	3.47e-06	1.72e-06	4.848.982
206004_at	3.47e-06	1.72e-06	4.848.612
215467_x_at	3.47e-06	1.72e-06	-4.848.594
217608_at	3.48e-06	1.72e-06	4.848.253
207247_s_at	3.50e-06	1.73e-06	4.846.853
207368_at	3.52e-06	1.74e-06	-4.845.598
213993_at	3.52e-06	1.75e-06	-4.845.353
221307_at	3.53e-06	1.75e-06	-4.844.877
208796_s_at	3.53e-06	1.75e-06	4.844.843
217804_s_at	3.54e-06	1.75e-06	4.844.585
206532_at	3.55e-06	1.76e-06	-4.843.952

207980_s_at	3.57e-06	1.77e-06	-4.842.825
205186_at	3.57e-06	1.77e-06	-4.842.481
208377_s_at	3.57e-06	1.77e-06	-4.842.327
201302_at	3.57e-06	1.77e-06	-4.842.261
221178_at	3.58e-06	1.78e-06	4.841.887
212600_s_at	3.58e-06	1.78e-06	-4.841.729
219942_at	3.58e-06	1.78e-06	-4.841.573
206977_at	3.58e-06	1.78e-06	-484.156
205537_s_at	3.59e-06	1.78e-06	-4.841.459
216222_s_at	3.59e-06	1.78e-06	-4.841.231
201708_s_at	3.60e-06	1.78e-06	-4.840.863
AFFX-r2-Bs-thr-M_s_a	3.60e-06	1.79e-06	-4.840.704
206413_s_at	3.60e-06	1.79e-06	-4.840.576
219126_at	3.60e-06	1.79e-06	-4.840.486
205810_s_at	3.60e-06	1.79e-06	-4.840.461
212329_at	3.60e-06	1.79e-06	-4.840.256
221709_s_at	3.61e-06	1.79e-06	-4.839.764
218762_at	3.61e-06	1.80e-06	-4.839.612
204400_at	3.62e-06	1.80e-06	-4.839.394
216766_at	3.62e-06	1.80e-06	-483.936
209566_at	3.63e-06	1.81e-06	-4.838.443
216193_at	3.65e-06	1.81e-06	-4.837.493
210100_s_at	3.66e-06	1.82e-06	-4.836.974
215265_at	3.67e-06	1.82e-06	-4.836.608
209009_at	3.67e-06	1.82e-06	-4.836.515
216938_x_at	3.67e-06	1.83e-06	-483.604
201415_at	3.69e-06	1.84e-06	483.506
218241_at	3.70e-06	1.84e-06	4.834.368
219093_at	3.72e-06	1.85e-06	-4.833.169
218116_at	3.73e-06	1.85e-06	-4.833.092
219811_at	3.73e-06	1.85e-06	-4.833.063
201981_at	3.73e-06	1.86e-06	-4.832.557
204708_at	3.74e-06	1.86e-06	-4.832.199
214132_at	3.75e-06	1.86e-06	4.831.692
207088_s_at	3.75e-06	1.87e-06	-483.146
209130_at	3.76e-06	1.87e-06	4.830.937
220252_x_at	3.77e-06	1.87e-06	-4.830.652
207556_s_at	3.77e-06	1.87e-06	483.059
221218_s_at	3.77e-06	1.88e-06	-4.830.345
220579_at	3.78e-06	1.88e-06	-4.829.836
219652_s_at	3.79e-06	1.89e-06	-4.829.401
210808_s_at	3.80e-06	1.89e-06	-4.828.816
219045_at	3.80e-06	1.89e-06	4.828.753
213466_at	3.80e-06	1.89e-06	-4.828.591

209340_at	3.81e-06	1.90e-06	-4.828.017
212338_at	3.84e-06	1.91e-06	4.826.506
78495_at	3.84e-06	1.91e-06	-4.826.505
208673_s_at	3.84e-06	1.91e-06	4.826.435
204660_at	3.85e-06	1.92e-06	-4.825.725
208748_s_at	3.86e-06	1.92e-06	4.825.492
207940_x_at	3.86e-06	1.92e-06	-4.825.364
211918_x_at	3.88e-06	1.93e-06	-4.824.409
202848_s_at	3.88e-06	1.93e-06	4.824.228
211754_s_at	3.88e-06	1.93e-06	4.823.997
207174_at	3.90e-06	1.94e-06	4.822.904
213049_at	3.90e-06	1.95e-06	4.822.852
212016_s_at	3.92e-06	1.96e-06	4.821.648
214680_at	3.93e-06	1.96e-06	-48.215
212284_x_at	3.94e-06	1.97e-06	-4.820.538
206126_at	3.95e-06	1.97e-06	-4.820.087
220748_s_at	3.97e-06	1.98e-06	481.931
206707_x_at	3.97e-06	1.98e-06	-4.819.115
212267_at	3.98e-06	1.98e-06	4.818.843
203132_at	3.98e-06	1.99e-06	4.818.567
205475_at	3.98e-06	1.99e-06	-4.818.453
211651_s_at	3.99e-06	1.99e-06	4.818.131
221102_s_at	4.00e-06	2.00e-06	-4.817.486
202524_s_at	4.00e-06	2.00e-06	-4.817.195
213776_at	4.01e-06	2.00e-06	481.686
214914_at	4.02e-06	2.01e-06	-481.645
201971_s_at	4.04e-06	2.02e-06	4.815.418
207739_s_at	4.05e-06	2.02e-06	4.814.772
214632_at	4.08e-06	2.04e-06	-4.813.346
209948_at	4.08e-06	2.04e-06	-4.813.123
222323_at	4.08e-06	2.04e-06	-4.812.961
218062_x_at	4.09e-06	2.04e-06	4.812.781
217953_at	4.10e-06	2.05e-06	4.812.138
216398_at	4.12e-06	2.06e-06	-481.085
216197_at	4.12e-06	2.06e-06	-481.082
202494_at	4.13e-06	2.06e-06	481.067
220846_s_at	4.13e-06	2.06e-06	-4.810.643
210078_s_at	4.20e-06	2.10e-06	-4.806.889
211073_x_at	4.20e-06	2.10e-06	4.806.755
221859_at	4.21e-06	2.10e-06	-4.806.578
215289_at	4.21e-06	2.10e-06	-4.806.512
220576_at	4.22e-06	2.11e-06	4.805.681
213875_x_at	4.23e-06	2.11e-06	4.805.475
207570_at	4.23e-06	2.12e-06	-4.805.184

216182_at	4.25e-06	2.12e-06	-4.804.404
201900_s_at	4.25e-06	2.12e-06	-4.804.279
215476_at	4.25e-06	2.13e-06	-4.804.096
221084_at	4.26e-06	2.13e-06	-4.803.998
213457_at	4.26e-06	2.13e-06	4.803.943
206635_at	4.28e-06	2.14e-06	-4.802.668
41577_at	4.29e-06	2.15e-06	-4.802.232
204975_at	4.30e-06	2.15e-06	-4.801.476
205912_at	4.31e-06	2.16e-06	-4.801.229
207476_at	4.32e-06	2.16e-06	-4.800.548
206218_at	4.39e-06	2.19e-06	4.797.532
212511_at	4.43e-06	2.22e-06	4.795.174
216994_s_at	4.49e-06	2.25e-06	-4.792.343
211082_x_at	4.50e-06	2.25e-06	-479.204
210614_at	4.51e-06	2.26e-06	-479.134
211189_x_at	4.52e-06	2.26e-06	-4.791.206
221469_at	4.52e-06	2.26e-06	-4.791.198
222034_at	4.52e-06	2.26e-06	4.791.078
213897_s_at	4.53e-06	2.27e-06	4.790.614
213479_at	4.53e-06	2.27e-06	479.051
209049_s_at	4.54e-06	2.27e-06	478.996
220763_at	4.57e-06	2.29e-06	-4.788.838
217454_at	4.57e-06	2.29e-06	-4.788.458
204751_x_at	4.58e-06	2.29e-06	4.788.056
220401_at	4.59e-06	2.30e-06	-478.766
218637_at	4.62e-06	2.32e-06	4.786.203
201949_x_at	4.66e-06	2.33e-06	4.784.531
212606_at	4.66e-06	2.33e-06	-478.445
220281_at	4.66e-06	2.34e-06	4.784.283
220874_at	4.66e-06	2.34e-06	-4.784.218
208591_s_at	4.67e-06	2.34e-06	-4.783.965
205958_x_at	4.67e-06	2.34e-06	-4.783.856
211915_s_at	4.67e-06	2.34e-06	-4.783.651
213283_s_at	4.69e-06	2.35e-06	4.782.989
201811_x_at	4.69e-06	2.35e-06	-4.782.885
221389_at	4.70e-06	2.36e-06	-4.782.585
206192_at	4.70e-06	2.36e-06	-4.782.534
AFFX-LysX-3_at	4.70e-06	2.36e-06	-4.782.328
213611_at	4.70e-06	2.36e-06	-4.782.229
201635_s_at	4.71e-06	2.36e-06	4.782.093
216155_at	4.73e-06	2.37e-06	-4.780.827
213595_s_at	4.75e-06	2.38e-06	4.780.022
210185_at	4.76e-06	2.39e-06	-4.779.503
207663_x_at	4.76e-06	2.39e-06	4.779.451

221098_x_at	4.77e-06	2.39e-06	-4.779.221
215440_s_at	4.79e-06	2.40e-06	4.778.324
214627_at	4.79e-06	2.40e-06	-4.778.199
205451_at	4.81e-06	2.41e-06	-477.749
211312_s_at	4.83e-06	2.42e-06	-4.776.619
216710_x_at	4.85e-06	2.43e-06	-4.775.695
217686_at	4.85e-06	2.43e-06	-4.775.593
215675_at	4.87e-06	2.45e-06	-477.447
205677_s_at	4.88e-06	2.45e-06	-4.773.948
207546_at	4.90e-06	2.46e-06	-4.773.086
208160_at	4.92e-06	2.47e-06	-4.772.476
213898_at	4.93e-06	2.48e-06	-4.772.003
214990_at	4.93e-06	2.48e-06	-4.771.842
217199_s_at	4.94e-06	2.48e-06	-4.771.393
210608_s_at	4.94e-06	2.48e-06	4.771.292
203733_at	4.95e-06	2.49e-06	-4.771.087
209513_s_at	4.95e-06	2.49e-06	-4.770.814
215216_at	4.98e-06	2.50e-06	-4.769.693
221221_s_at	4.99e-06	2.51e-06	-4.769.456
218633_x_at	4.99e-06	2.51e-06	-4.769.348
211190_x_at	4.99e-06	2.51e-06	-4.769.256
214092_x_at	4.99e-06	2.51e-06	476.901
210577_at	5.05e-06	2.54e-06	-4.766.547
207673_at	5.05e-06	2.54e-06	-4.766.423
201334_s_at	5.06e-06	2.55e-06	-4.766.141
208507_at	5.06e-06	2.55e-06	-4.766.063
215404_x_at	5.07e-06	2.55e-06	-4.765.669
201305_x_at	5.08e-06	2.56e-06	4.765.238
215864_at	5.09e-06	2.56e-06	-4.764.904
219096_at	5.12e-06	2.57e-06	-4.763.735
201461_s_at	5.12e-06	2.58e-06	4.763.522
206739_at	5.13e-06	2.58e-06	-4.763.014
215446_s_at	5.15e-06	2.59e-06	4.762.104
207289_at	5.17e-06	2.60e-06	-4.761.431
210917_at	5.19e-06	2.61e-06	-4.760.784
214926_at	5.19e-06	2.61e-06	-4.760.583
214021_x_at	5.20e-06	2.62e-06	4.760.303
222264_at	5.20e-06	2.62e-06	-4.760.244
215430_at	5.20e-06	2.62e-06	-4.760.093
217627_at	5.20e-06	2.62e-06	4.760.018
207954_at	5.20e-06	2.62e-06	-4.759.898
205852_at	5.20e-06	2.62e-06	-4.759.896
205065_at	5.27e-06	2.66e-06	-4.757.144
219169_s_at	5.27e-06	2.66e-06	-4.757.024

216713_at	5.28e-06	2.66e-06	4.756.748
201858_s_at	5.29e-06	2.67e-06	-4.756.161
211792_s_at	5.30e-06	2.67e-06	4.755.938
209984_at	5.31e-06	2.68e-06	4.755.511
219369_s_at	5.31e-06	2.68e-06	4.755.404
215694_at	5.32e-06	2.68e-06	4.755.153
215141_at	5.32e-06	2.68e-06	-4.754.977
205850_s_at	5.32e-06	2.68e-06	-4.754.885
215951_at	5.34e-06	2.69e-06	-4.754.307
208133_at	5.34e-06	2.70e-06	-4.754.018
211099_s_at	5.35e-06	2.70e-06	-4.753.815
220373_at	5.38e-06	2.71e-06	-4.752.601
220718_at	5.38e-06	2.71e-06	-4.752.514
219789_at	5.39e-06	2.72e-06	-4.752.283
201883_s_at	5.40e-06	2.72e-06	-4.751.783
204411_at	5.43e-06	2.74e-06	4.750.661
204172_at	5.43e-06	2.74e-06	-4.750.556
205605_at	5.49e-06	2.77e-06	-4.748.197
206868_at	5.49e-06	2.77e-06	-4.748.039
202405_at	5.52e-06	2.79e-06	4.746.829
221445_at	5.53e-06	2.79e-06	-4.746.615
203313_s_at	5.53e-06	2.79e-06	4.746.498
212285_s_at	5.53e-06	2.79e-06	4.746.421
211207_s_at	5.55e-06	2.80e-06	4.745.598
209506_s_at	5.56e-06	2.81e-06	-4.745.365
208616_s_at	5.57e-06	2.81e-06	4.744.956
200824_at	5.57e-06	2.81e-06	-4.744.867
219018_s_at	5.59e-06	2.82e-06	4.744.296
204177_s_at	5.60e-06	2.83e-06	-4.743.892
208404_x_at	5.64e-06	2.85e-06	-4.742.233
203877_at	5.65e-06	2.86e-06	-4.741.718
221122_at	5.65e-06	2.86e-06	-4.741.615
213628_at	5.67e-06	2.86e-06	4.741.037
217141_at	5.68e-06	2.87e-06	-4.740.586
204628_s_at	5.68e-06	2.87e-06	-4.740.458
202019_s_at	5.69e-06	2.88e-06	4.740.219
210546_x_at	5.70e-06	2.88e-06	4.739.783
219662_at	5.73e-06	2.90e-06	4.738.804
202046_s_at	5.74e-06	2.90e-06	-4.738.291
205795_at	5.74e-06	2.90e-06	473.822
206996_x_at	5.76e-06	2.91e-06	-4.737.562
200962_at	5.77e-06	2.92e-06	4.737.273
201150_s_at	5.77e-06	2.92e-06	-4.737.227
221219_s_at	5.78e-06	2.92e-06	-4.736.641

200600_at	5.80e-06	2.93e-06	4.735.902
213997_at	5.82e-06	2.94e-06	-4.735.296
214072_x_at	5.82e-06	2.94e-06	-4.735.219
214930_at	5.82e-06	2.95e-06	-4.735.156
222059_at	5.82e-06	2.95e-06	-4.735.007
209296_at	5.82e-06	2.95e-06	4.734.966
222295_x_at	5.83e-06	2.95e-06	-4.734.944
215090_x_at	5.83e-06	2.95e-06	4.734.757
201307_at	5.85e-06	2.96e-06	4.733.905
221953_s_at	5.86e-06	2.96e-06	-4.733.742
214785_at	5.90e-06	2.98e-06	-4.732.315
203027_s_at	5.91e-06	2.99e-06	4.731.702
215448_at	5.93e-06	3.01e-06	-4.730.848
213270_at	5.94e-06	3.01e-06	-4.730.699
206684_s_at	5.97e-06	3.02e-06	-4.729.516
208810_at	5.98e-06	3.03e-06	4.729.207
AFFX-HUMISGF3A/M9	5.98e-06	3.03e-06	4.729.127
219926_at	6.00e-06	3.04e-06	4.728.601
216953_s_at	6.00e-06	3.04e-06	-4.728.278
206583_at	6.02e-06	3.05e-06	4.727.534
214941_s_at	6.03e-06	3.06e-06	4.727.273
213107_at	6.07e-06	3.08e-06	4.725.795
205351_at	6.08e-06	3.08e-06	-4.725.471
220403_s_at	6.08e-06	3.08e-06	-4.725.391
206791_s_at	6.08e-06	3.08e-06	-4.725.381
205780_at	6.11e-06	3.10e-06	4.724.407
212552_at	6.12e-06	3.11e-06	4.723.869
219574_at	6.14e-06	3.11e-06	-4.723.334
211253_x_at	6.14e-06	3.12e-06	-472.318
208541_x_at	6.15e-06	3.12e-06	4.723.059
215194_at	6.16e-06	3.12e-06	-4.722.583
200839_s_at	6.19e-06	3.14e-06	-4.721.392
216127_at	6.22e-06	3.16e-06	-4.720.386
205853_at	6.23e-06	3.16e-06	-4.720.138
213371_at	6.23e-06	3.16e-06	-4.719.987
207148_x_at	6.24e-06	3.16e-06	-471.988
213081_at	6.24e-06	3.17e-06	4.719.663
215255_at	6.25e-06	3.17e-06	-471.947
204056_s_at	6.25e-06	3.17e-06	-4.719.312
203804_s_at	6.28e-06	3.19e-06	4.718.288
217032_at	6.29e-06	3.19e-06	-4.717.982
202804_at	6.30e-06	3.20e-06	4.717.425
208220_x_at	6.31e-06	3.20e-06	-4.717.338
201146_at	6.32e-06	3.21e-06	-4.716.679

212513_s_at	6.33e-06	3.21e-06	4.716.605
201155_s_at	6.35e-06	3.22e-06	-471.596
48117_at	6.35e-06	3.23e-06	-4.715.653
203402_at	6.37e-06	3.24e-06	-471.502
209768_s_at	6.38e-06	3.24e-06	-4.714.818
214927_at	6.39e-06	3.25e-06	-4.714.425
218369_s_at	6.42e-06	3.26e-06	-4.713.451
207143_at	6.43e-06	3.27e-06	-4.712.958
208068_x_at	6.46e-06	3.28e-06	-4.712.043
216239_at	6.46e-06	3.28e-06	4.711.991
219935_at	6.46e-06	3.29e-06	-4.711.805
203014_x_at	6.48e-06	3.29e-06	4.711.344
206695_x_at	6.49e-06	3.30e-06	4.710.765
208251_at	6.51e-06	3.31e-06	-4.710.256
207117_at	6.52e-06	3.31e-06	-4.709.994
211497_x_at	6.60e-06	3.36e-06	-4.707.136
212389_at	6.62e-06	3.37e-06	-4.706.625
212454_x_at	6.62e-06	3.37e-06	-4.706.531
220193_at	6.64e-06	3.38e-06	-470.576
218938_at	6.66e-06	3.39e-06	4.705.116
212259_s_at	6.71e-06	3.41e-06	4.703.694
212046_x_at	6.72e-06	3.42e-06	4.703.351
206078_at	6.72e-06	3.42e-06	-4.703.193
208272_at	6.73e-06	3.43e-06	-4.702.867
219742_at	6.73e-06	3.43e-06	4.702.806
203981_s_at	6.74e-06	3.43e-06	-4.702.668
210014_x_at	6.74e-06	3.43e-06	4.702.631
201421_s_at	6.74e-06	3.43e-06	4.702.497
45572_s_at	6.74e-06	3.43e-06	4.702.451
215354_s_at	6.77e-06	3.44e-06	4.701.701
204266_s_at	6.78e-06	3.45e-06	4.701.296
221595_at	6.78e-06	3.45e-06	4.701.286
205454_at	6.79e-06	3.46e-06	-4.700.763
215935_at	6.79e-06	3.46e-06	-4.700.725
220036_s_at	6.81e-06	3.47e-06	-4.700.073
222090_at	6.89e-06	3.51e-06	-4.697.765
221288_at	6.90e-06	3.51e-06	-469.738
220706_at	6.90e-06	3.52e-06	-4.697.288
206852_at	6.90e-06	3.52e-06	-4.697.158
210752_s_at	6.91e-06	3.52e-06	-4.696.893
211753_s_at	6.92e-06	3.53e-06	-4.696.688
208379_x_at	6.95e-06	3.54e-06	-469.578
215918_s_at	6.95e-06	3.54e-06	-4.695.594
212093_s_at	6.96e-06	3.55e-06	-4.695.333

219266_at	6.96e-06	3.55e-06	4.695.265
206278_at	6.98e-06	3.56e-06	-4.694.648
220705_s_at	7.01e-06	3.58e-06	-4.693.701
221640_s_at	7.02e-06	3.58e-06	4.693.422
214342_at	7.10e-06	3.62e-06	-4.691.136
210930_s_at	7.10e-06	3.62e-06	-4.691.086
216391_s_at	7.12e-06	3.63e-06	-4.690.302
221384_at	7.12e-06	3.63e-06	-4.690.265
206758_at	7.14e-06	3.64e-06	-4.689.837
220366_at	7.16e-06	3.65e-06	-468.906
204012_s_at	7.16e-06	3.65e-06	4.689.015
221610_s_at	7.16e-06	3.66e-06	-4.688.967
214079_at	7.17e-06	3.66e-06	-4.688.649
205925_s_at	7.18e-06	3.67e-06	4.688.295
200965_s_at	7.21e-06	3.68e-06	4.687.512
201878_at	7.22e-06	3.69e-06	4.687.181
211264_at	7.25e-06	3.70e-06	-4.686.246
210465_s_at	7.29e-06	3.72e-06	-4.685.132
201539_s_at	7.30e-06	3.73e-06	-468.469
212410_at	7.32e-06	3.74e-06	4.684.195
204215_at	7.36e-06	3.76e-06	468.304
206096_at	7.36e-06	3.76e-06	4.682.898
AFFX-r2-Bs-thr-5_s_at	7.36e-06	3.76e-06	-4.682.842
219938_s_at	7.39e-06	3.77e-06	468.212
211225_at	7.39e-06	3.78e-06	-4.681.948
221134_at	7.40e-06	3.78e-06	-4.681.766
201638_s_at	7.45e-06	3.81e-06	-4.680.319
205720_at	7.45e-06	3.81e-06	-4.680.318
214057_at	7.47e-06	3.82e-06	-4.679.694
211049_at	7.49e-06	3.83e-06	-4.679.032
200832_s_at	7.54e-06	3.85e-06	4.677.599
220010_at	7.54e-06	3.85e-06	4.677.571
203674_at	7.54e-06	3.86e-06	-467.747
205956_x_at	7.55e-06	3.86e-06	4.677.195
204878_s_at	7.57e-06	3.87e-06	-4.676.636
207190_at	7.65e-06	3.91e-06	-4.674.282
215596_s_at	7.72e-06	3.95e-06	4.672.515
215515_at	7.75e-06	3.96e-06	-4.671.515
221285_at	7.76e-06	3.97e-06	-4.671.307
208858_s_at	7.76e-06	3.97e-06	467.108
212452_x_at	7.77e-06	3.97e-06	-4.670.922
206266_at	7.82e-06	4.00e-06	-4.669.472
217701_x_at	7.83e-06	4.01e-06	-4.669.233
211241_at	7.84e-06	4.01e-06	4.668.922

212864_at	7.84e-06	4.01e-06	4.668.842
221216_s_at	7.85e-06	4.02e-06	4.668.494
209398_at	7.86e-06	4.02e-06	46.683
206402_s_at	7.95e-06	4.07e-06	-4.665.911
210237_at	7.97e-06	4.08e-06	-4.665.161
215890_at	7.98e-06	4.08e-06	-4.665.032
207393_at	7.98e-06	4.09e-06	-4.664.893
201445_at	8.02e-06	4.11e-06	4.663.932
203555_at	8.04e-06	4.12e-06	-4.663.251
206799_at	8.04e-06	4.12e-06	4.663.173
215845_x_at	8.09e-06	4.15e-06	-4.661.856
222228_s_at	8.10e-06	4.15e-06	4.661.572
210761_s_at	8.11e-06	4.15e-06	-4.661.431
221308_at	8.12e-06	4.16e-06	-466.122
210722_at	8.14e-06	4.17e-06	-4.660.584
212595_s_at	8.14e-06	4.17e-06	46.604
204625_s_at	8.23e-06	4.22e-06	-4.658.254
202279_at	8.23e-06	4.22e-06	4.658.126
208368_s_at	8.24e-06	4.22e-06	4.657.957
222067_x_at	8.25e-06	4.23e-06	4.657.477
220639_at	8.26e-06	4.23e-06	4.657.389
206795_at	8.26e-06	4.23e-06	-4.657.261
215035_at	8.28e-06	4.24e-06	-4.656.752
214673_s_at	8.30e-06	4.26e-06	-4.656.216
207902_at	8.30e-06	4.26e-06	-4.656.096
215963_x_at	8.31e-06	4.26e-06	4.655.948
221074_at	8.31e-06	4.26e-06	-4.655.798
221078_s_at	8.32e-06	4.27e-06	4.655.442
215825_at	8.33e-06	4.27e-06	-4.655.384
203821_at	8.34e-06	4.28e-06	-465.506
209631_s_at	8.34e-06	4.28e-06	4.654.893
209235_at	8.36e-06	4.29e-06	-4.654.572
209952_s_at	8.41e-06	4.32e-06	-4.653.161
201520_s_at	8.47e-06	4.35e-06	465.161
212408_at	8.49e-06	4.36e-06	4.650.939
204368_at	8.51e-06	4.37e-06	4.650.587
203200_s_at	8.56e-06	4.40e-06	4.649.223
218771_at	8.57e-06	4.40e-06	4.648.909
205993_s_at	8.60e-06	4.42e-06	-4.648.181
215205_x_at	8.60e-06	4.42e-06	-4.648.113
207767_s_at	8.61e-06	4.42e-06	-4.647.949
222328_x_at	8.61e-06	4.42e-06	-4.647.802
206948_at	8.62e-06	4.43e-06	-4.647.618
202221_s_at	8.64e-06	4.44e-06	4.647.146

217281_x_at	8.66e-06	4.45e-06	-4.646.474
213381_at	8.71e-06	4.47e-06	-4.645.423
209077_at	8.71e-06	4.48e-06	-4.645.275
211894_x_at	8.71e-06	4.48e-06	-4.645.199
204427_s_at	8.73e-06	4.49e-06	4.644.682
221390_s_at	8.75e-06	4.50e-06	-4.644.329
202661_at	8.75e-06	4.50e-06	-4.644.217
216587_s_at	8.76e-06	4.51e-06	-4.643.871
209373_at	8.78e-06	4.51e-06	4.643.545
213319_s_at	8.79e-06	4.52e-06	-4.643.176
217443_at	8.79e-06	4.52e-06	-4.643.151
48659_at	8.87e-06	4.56e-06	4.641.089
216699_s_at	8.88e-06	4.57e-06	-4.640.865
210772_at	8.96e-06	4.61e-06	-463.905
210339_s_at	8.97e-06	4.61e-06	-4.638.767
217382_at	8.97e-06	4.62e-06	-4.638.585
213876_x_at	8.98e-06	4.62e-06	-4.638.502
215519_x_at	8.99e-06	4.62e-06	4.638.255
214325_at	9.01e-06	4.63e-06	-4.637.778
221501_x_at	9.02e-06	4.64e-06	-4.637.455
220752_at	9.02e-06	4.64e-06	-4.637.367
202410_x_at	9.04e-06	4.65e-06	-4.636.855
216162_at	9.06e-06	4.66e-06	-4.636.428
218747_s_at	9.07e-06	4.67e-06	-4.636.116
215002_at	9.08e-06	4.67e-06	-4.635.935
210926_at	9.11e-06	4.69e-06	4.635.074
210276_s_at	9.13e-06	4.70e-06	4.634.646
206953_s_at	9.14e-06	4.71e-06	-4.634.309
37079_at	9.15e-06	4.71e-06	4.634.166
202784_s_at	9.16e-06	4.72e-06	-4.633.966
218521_s_at	9.17e-06	4.73e-06	4.633.506
202825_at	9.17e-06	4.73e-06	-4.633.502
221881_s_at	9.18e-06	4.73e-06	-4.633.402
216268_s_at	9.22e-06	4.75e-06	4.632.456
220628_s_at	9.23e-06	4.75e-06	-4.632.244
221150_at	9.23e-06	4.76e-06	-4.631.998
211787_s_at	9.27e-06	4.78e-06	4.631.244
205970_at	9.29e-06	4.79e-06	-4.630.755
208304_at	9.32e-06	4.80e-06	-4.629.965
213792_s_at	9.33e-06	4.81e-06	-4.629.787
208348_s_at	9.36e-06	4.83e-06	-4.628.928
207690_at	9.40e-06	4.85e-06	-4.628.094
217362_x_at	9.41e-06	4.85e-06	-4.627.822
214210_at	9.44e-06	4.87e-06	-4.626.977

220205_at	9.45e-06	4.87e-06	4.626.906
215422_at	9.51e-06	4.90e-06	-4.625.497
206304_at	9.51e-06	4.91e-06	-4.625.355
204508_s_at	9.55e-06	4.93e-06	462.451
220824_at	9.58e-06	4.94e-06	-4.623.687
211817_s_at	9.59e-06	4.95e-06	-4.623.541
34187_at	9.63e-06	4.97e-06	-462.272
215915_at	9.65e-06	4.98e-06	-4.622.231
221354_s_at	9.65e-06	4.98e-06	-4.622.181
219560_at	9.69e-06	5.00e-06	4.621.274
220359_s_at	9.71e-06	5.01e-06	-4.620.742
220529_at	9.74e-06	5.03e-06	-4.62
214652_at	9.74e-06	5.03e-06	-4.619.948
214521_at	9.76e-06	5.04e-06	-461.948
216327_s_at	9.77e-06	5.04e-06	-4.619.423
211820_x_at	9.84e-06	5.08e-06	-4.617.807
214978_s_at	9.89e-06	5.11e-06	4.616.592
219831_at	9.89e-06	5.11e-06	-4.616.556
211204_at	9.91e-06	5.12e-06	-4.616.136
209901_x_at	9.94e-06	5.13e-06	-461.547
219436_s_at	9.94e-06	5.14e-06	4.615.381
206838_at	1.00e-05	5.18e-06	461.359
220787_at	1.00e-05	5.19e-06	-4.613.294
214229_at	1.00e-05	5.19e-06	-4.613.225
210523_at	1.00e-05	5.19e-06	-4.613.026
220633_s_at	1.01e-05	5.22e-06	-4.612.066
217172_at	1.02e-05	5.26e-06	-4.610.182
210155_at	1.02e-05	5.27e-06	-4.609.971
203163_at	1.02e-05	5.27e-06	4.609.731
208492_at	1.02e-05	5.28e-06	-4.609.213
206700_s_at	1.02e-05	5.29e-06	-4.609.013
215239_x_at	1.03e-05	5.31e-06	460.807
215733_x_at	1.03e-05	5.33e-06	4.607.335
207210_at	1.03e-05	5.34e-06	-4.606.891
212669_at	1.03e-05	5.35e-06	4.606.573
211488_s_at	1.04e-05	5.38e-06	-4.605.126
202984_s_at	1.04e-05	5.39e-06	4.604.862
208548_at	1.05e-05	5.41e-06	-4.604.179
214936_at	1.05e-05	5.42e-06	-4.603.775
222185_at	1.05e-05	5.42e-06	-4.603.669
207359_at	1.05e-05	5.42e-06	-4.603.584
207734_at	1.05e-05	5.43e-06	-4.603.301
208594_x_at	1.05e-05	5.43e-06	-460.319
209487_at	1.05e-05	5.44e-06	4.603.037

215252_at	1.05e-05	5.44e-06	-4.602.849
202215_s_at	1.05e-05	5.46e-06	4.602.049
201831_s_at	1.06e-05	5.46e-06	-4.601.962
206721_at	1.06e-05	5.50e-06	-4.600.436
216646_at	1.06e-05	5.51e-06	-4.600.156
206195_x_at	1.06e-05	5.51e-06	-4.600.138
220664_at	1.06e-05	5.51e-06	-4.599.932
211844_s_at	1.07e-05	5.53e-06	-4.599.225
207849_at	1.07e-05	5.54e-06	-4.599.041
209965_s_at	1.07e-05	5.54e-06	4.598.885
204923_at	1.07e-05	5.55e-06	-4.598.429
210567_s_at	1.08e-05	5.58e-06	4.597.301
220119_at	1.08e-05	5.59e-06	-4.597.085
201526_at	1.08e-05	5.60e-06	4.596.552
216026_s_at	1.08e-05	5.60e-06	-4.596.429
218746_at	1.08e-05	5.61e-06	-4.596.308
204339_s_at	1.08e-05	5.61e-06	-4.596.023
220461_at	1.08e-05	5.62e-06	-4.595.892
215461_at	1.09e-05	5.67e-06	-4.593.974
210965_x_at	1.09e-05	5.67e-06	-4.593.844
219584_at	1.09e-05	5.68e-06	-459.364
214999_s_at	1.10e-05	5.68e-06	-4.593.293
1320_at	1.10e-05	5.70e-06	-4.592.779
215914_at	1.10e-05	5.73e-06	-4.591.682
210506_at	1.10e-05	5.73e-06	-4.591.603
210043_at	1.11e-05	5.75e-06	-4.590.848
201058_s_at	1.11e-05	5.77e-06	-4.589.913
218105_s_at	1.11e-05	5.77e-06	4.589.877
204922_at	1.11e-05	5.78e-06	4.589.667
216670_at	1.12e-05	5.79e-06	-4.589.285
209473_at	1.12e-05	5.80e-06	4.588.824
219536_s_at	1.12e-05	5.82e-06	-4.588.265
213011_s_at	1.13e-05	5.84e-06	4.587.268
208269_s_at	1.13e-05	5.87e-06	-4.586.182
214023_x_at	1.14e-05	5.90e-06	4.585.274
207990_x_at	1.15e-05	5.95e-06	-458.334
206582_s_at	1.15e-05	5.95e-06	-4.583.254
220719_at	1.16e-05	6.01e-06	-458.122
208112_x_at	1.16e-05	6.01e-06	-4.581.144
221437_s_at	1.17e-05	6.07e-06	4.579.051
218276_s_at	1.17e-05	6.08e-06	-4.578.708
201777_s_at	1.17e-05	6.09e-06	4.578.287
220411_x_at	1.18e-05	6.12e-06	-4.577.217
213744_at	1.19e-05	6.17e-06	-4.575.472

212176_at	1.19e-05	6.20e-06	-4.574.471
209547_s_at	1.20e-05	6.23e-06	4.573.397
212933_x_at	1.20e-05	6.23e-06	-4.573.209
222259_s_at	1.20e-05	6.24e-06	-4.572.773
220735_s_at	1.20e-05	6.25e-06	4.572.566
215490_at	1.20e-05	6.26e-06	-457.206
220162_s_at	1.21e-05	6.27e-06	-4.571.921
203425_s_at	1.21e-05	6.31e-06	-4.570.278
213579_s_at	1.22e-05	6.33e-06	4.569.901
215695_s_at	1.22e-05	6.34e-06	-4.569.546
202571_s_at	1.22e-05	6.37e-06	-4.568.328
202485_s_at	1.23e-05	6.37e-06	-4.568.219
214256_at	1.23e-05	6.38e-06	-4.567.976
203951_at	1.23e-05	6.39e-06	-4.567.699
215858_at	1.23e-05	6.40e-06	-4.567.322
205793_x_at	1.23e-05	6.41e-06	-4.566.956
204054_at	1.23e-05	6.42e-06	4.566.799
215584_at	1.25e-05	6.48e-06	-4.564.595
207941_s_at	1.25e-05	6.49e-06	4.564.382
201832_s_at	1.25e-05	6.50e-06	-4.563.796
219743_at	1.25e-05	6.51e-06	-4.563.677
216869_at	1.25e-05	6.52e-06	-4.563.148
206914_at	1.25e-05	6.53e-06	-4.563.035
216974_at	1.25e-05	6.53e-06	-4.563.018
211259_s_at	1.26e-05	6.55e-06	-4.562.344
216605_s_at	1.26e-05	6.55e-06	-456.228
220073_s_at	1.26e-05	6.59e-06	4.560.996
206631_at	1.27e-05	6.61e-06	-4.560.302
212593_s_at	1.27e-05	6.64e-06	4.559.402
49878_at	1.28e-05	6.66e-06	-4.558.607
220823_at	1.28e-05	6.67e-06	-4.558.193
203176_s_at	1.28e-05	6.69e-06	4.557.477
204167_at	1.29e-05	6.70e-06	-4.557.369
218855_at	1.29e-05	6.74e-06	4.555.911
216243_s_at	1.29e-05	6.74e-06	-4.555.844
207057_at	1.29e-05	6.74e-06	-4.555.838
212218_s_at	1.30e-05	6.76e-06	4.555.317
213594_x_at	1.30e-05	6.77e-06	4.554.962
214078_at	1.30e-05	6.78e-06	4.554.726
213006_at	1.30e-05	6.78e-06	-4.554.722
215677_s_at	1.30e-05	6.80e-06	-4.553.986
211461_at	1.31e-05	6.82e-06	-4.553.366
207701_at	1.31e-05	6.82e-06	-4.553.326
207274_at	1.31e-05	6.84e-06	-4.552.685

211493_x_at	1.31e-05	6.84e-06	4.552.667
215647_at	1.31e-05	6.84e-06	-4.552.626
221463_at	1.31e-05	6.85e-06	-4.552.486
220762_s_at	1.32e-05	6.87e-06	4.551.703
215491_at	1.32e-05	6.90e-06	-4.550.871
222268_x_at	1.33e-05	6.94e-06	-4.549.542
201781_s_at	1.34e-05	6.98e-06	4.548.153
222194_at	1.34e-05	7.01e-06	-4.547.457
218006_s_at	1.34e-05	7.01e-06	4.547.352
222294_s_at	1.35e-05	7.05e-06	-4.546.212
217681_at	1.36e-05	7.08e-06	-4.545.083
213155_at	1.36e-05	7.08e-06	4.545.032
217507_at	1.36e-05	7.11e-06	-4.544.284
216793_x_at	1.37e-05	7.13e-06	-4.543.612
206246_at	1.37e-05	7.14e-06	-4.543.313
210492_at	1.37e-05	7.15e-06	-4.543.005
205945_at	1.37e-05	7.15e-06	-4.542.896
210770_s_at	1.37e-05	7.15e-06	-4.542.875
204431_at	1.37e-05	7.16e-06	-4.542.677
213461_at	1.37e-05	7.17e-06	4.542.317
210006_at	1.37e-05	7.18e-06	4.542.072
205974_at	1.37e-05	7.18e-06	-4.541.962
210925_at	1.38e-05	7.21e-06	-4.541.155
216380_x_at	1.38e-05	7.21e-06	4.541.109
217705_at	1.39e-05	7.25e-06	-4.539.985
213512_at	1.39e-05	7.26e-06	-4.539.578
222223_s_at	1.39e-05	7.27e-06	-4.539.242
210120_s_at	1.40e-05	7.31e-06	-4.538.219
217506_at	1.40e-05	7.32e-06	-4.537.906
215502_at	1.40e-05	7.32e-06	-4.537.625
219141_s_at	1.40e-05	7.34e-06	4.537.178
215149_at	1.40e-05	7.34e-06	-4.537.149
211520_s_at	1.40e-05	7.35e-06	-4.536.918
217838_s_at	1.40e-05	7.35e-06	-4.536.912
212317_at	1.40e-05	7.35e-06	4.536.881
212759_s_at	1.41e-05	7.36e-06	-4.536.483
204219_s_at	1.42e-05	7.42e-06	4.534.872
219920_s_at	1.42e-05	7.42e-06	4.534.751
209555_s_at	1.42e-05	7.44e-06	4.534.282
207424_at	1.43e-05	7.48e-06	-4.533.139
204698_at	1.43e-05	7.48e-06	4.532.886
204007_at	1.43e-05	7.49e-06	-4.532.598
202225_at	1.43e-05	7.50e-06	-4.532.506
215527_at	1.43e-05	7.51e-06	-4.532.093

212430_at	1.43e-05	7.51e-06	4.532.089
211712_s_at	1.44e-05	7.53e-06	4.531.514
207324_s_at	1.44e-05	7.54e-06	-4.531.252
210118_s_at	1.44e-05	7.55e-06	-4.530.863
217890_s_at	1.45e-05	7.58e-06	-4.530.072
204710_s_at	1.45e-05	7.60e-06	45.296
219738_s_at	1.45e-05	7.60e-06	-4.529.589
201715_s_at	1.45e-05	7.60e-06	4.529.569
204600_at	1.45e-05	7.61e-06	-4.529.108
203601_s_at	1.46e-05	7.63e-06	-4.528.689
205636_at	1.46e-05	7.63e-06	-4.528.612
221850_x_at	1.46e-05	7.65e-06	-4.528.128
216700_at	1.46e-05	7.67e-06	-4.527.577
203215_s_at	1.47e-05	7.70e-06	4.526.583
213837_at	1.47e-05	7.71e-06	4.526.286
201542_at	1.47e-05	7.72e-06	4.526.109
211990_at	1.47e-05	7.72e-06	-4.526.085
215936_s_at	1.47e-05	7.73e-06	4.525.721
207590_s_at	1.47e-05	7.73e-06	4.525.632
207835_at	1.48e-05	7.78e-06	-4.524.406
216201_at	1.48e-05	7.78e-06	-4.524.285
201826_s_at	1.48e-05	7.79e-06	452.414
206312_at	1.49e-05	7.80e-06	452.378
219921_s_at	1.49e-05	7.80e-06	-452.376
206279_at	1.49e-05	7.81e-06	-4.523.397
200984_s_at	1.49e-05	7.81e-06	4.523.379
216485_s_at	1.50e-05	7.89e-06	-452.106
212778_at	1.51e-05	7.91e-06	4.520.755
212466_at	1.51e-05	7.91e-06	-4.520.666
214585_s_at	1.51e-05	7.93e-06	4.520.119
221717_at	1.51e-05	7.94e-06	-4.519.849
219367_s_at	1.51e-05	7.95e-06	-4.519.553
53991_at	1.52e-05	7.96e-06	-4.519.174
202358_s_at	1.52e-05	7.97e-06	-4.519.099
208995_s_at	1.52e-05	7.98e-06	451.866
211991_s_at	1.53e-05	8.02e-06	-4.517.599
207751_at	1.53e-05	8.02e-06	-4.517.508
216751_at	1.53e-05	8.05e-06	-4.516.739
218834_s_at	1.53e-05	8.06e-06	4.516.506
209162_s_at	1.53e-05	8.06e-06	4.516.445
208312_s_at	1.54e-05	8.08e-06	-4.515.948
201836_s_at	1.54e-05	8.08e-06	4.515.948
205439_at	1.54e-05	8.09e-06	-4.515.613
215703_at	1.55e-05	8.12e-06	-4.514.763

201355_s_at	1.55e-05	8.13e-06	-4.514.552
212198_s_at	1.55e-05	8.14e-06	4.514.287
219878_s_at	1.55e-05	8.16e-06	4.513.681
212397_at	1.56e-05	8.18e-06	4.513.268
212056_at	1.56e-05	8.18e-06	-4.513.251
204178_s_at	1.57e-05	8.23e-06	4.511.783
211411_at	1.57e-05	8.24e-06	-4.511.497
205948_at	1.57e-05	8.26e-06	-4.510.975
220408_x_at	1.57e-05	8.26e-06	4.510.929
201848_s_at	1.57e-05	8.28e-06	-4.510.495
49679_s_at	1.58e-05	8.29e-06	-4.510.213
215860_at	1.58e-05	8.30e-06	-4.509.891
209619_at	1.58e-05	8.34e-06	-4.509.008
203348_s_at	1.59e-05	8.34e-06	4.508.858
200083_at	1.59e-05	8.38e-06	450.774
205373_at	1.59e-05	8.38e-06	4.507.709
207597_at	1.60e-05	8.41e-06	-4.507.091
47105_at	1.60e-05	8.41e-06	-4.506.937
221674_s_at	1.60e-05	8.44e-06	-4.506.359
214337_at	1.60e-05	8.45e-06	450.608
210984_x_at	1.60e-05	8.45e-06	-4.505.986
220644_at	1.61e-05	8.45e-06	-4.505.931
211771_s_at	1.61e-05	8.50e-06	-4.504.603
206609_at	1.62e-05	8.52e-06	4.504.161
204626_s_at	1.62e-05	8.53e-06	-450.394
221269_s_at	1.62e-05	8.53e-06	4.503.919
206746_at	1.62e-05	8.54e-06	4.503.724
212161_at	1.63e-05	8.59e-06	-4.502.266
220857_at	1.63e-05	8.60e-06	-4.502.059
208385_at	1.63e-05	8.60e-06	-4.501.964
210822_at	1.64e-05	8.67e-06	4.500.402
215349_at	1.64e-05	8.67e-06	-4.500.314
205105_at	1.65e-05	8.67e-06	4.500.267
202278_s_at	1.65e-05	8.68e-06	4.500.056
38892_at	1.65e-05	8.68e-06	4.500.017
210532_s_at	1.65e-05	8.71e-06	4.499.337
214237_x_at	1.67e-05	8.80e-06	-4.496.851
207784_at	1.67e-05	8.82e-06	-4.496.412
209139_s_at	1.69e-05	8.91e-06	-4.494.132
208010_s_at	1.69e-05	8.92e-06	-449.385
215414_at	1.70e-05	8.95e-06	-4.493.302
203742_s_at	1.70e-05	8.98e-06	4.492.471
220614_s_at	1.71e-05	9.03e-06	-4.491.172
219688_at	1.72e-05	9.05e-06	4.490.665

210539_at	1.73e-05	9.14e-06	-4.488.522
217385_at	1.73e-05	9.15e-06	-4.488.202
206106_at	1.74e-05	9.16e-06	4.488.001
201120_s_at	1.74e-05	9.17e-06	-4.487.754
211694_at	1.74e-05	9.21e-06	-4.486.911
202623_at	1.75e-05	9.23e-06	448.631
202829_s_at	1.75e-05	9.25e-06	4.485.905
212719_at	1.75e-05	9.26e-06	-4.485.617
210517_s_at	1.76e-05	9.28e-06	4.485.258
217188_s_at	1.76e-05	9.30e-06	4.484.764
213925_at	1.76e-05	9.32e-06	-4.484.218
200711_s_at	1.77e-05	9.32e-06	4.484.116
200983_x_at	1.77e-05	9.34e-06	4.483.816
211159_s_at	1.77e-05	9.37e-06	4.483.109
212655_at	1.78e-05	9.39e-06	-4.482.619
202607_at	1.78e-05	9.42e-06	4.481.881
205367_at	1.79e-05	9.43e-06	-4.481.481
204433_s_at	1.79e-05	9.46e-06	4.480.981
217609_at	1.79e-05	9.47e-06	4.480.682
221292_at	1.80e-05	9.51e-06	-4.479.578
215204_at	1.80e-05	9.53e-06	-447.925
219684_at	1.80e-05	9.54e-06	4.479.072
214378_at	1.81e-05	9.55e-06	-4.478.663
211032_at	1.82e-05	9.60e-06	-4.477.603
208185_x_at	1.82e-05	9.61e-06	-4.477.435
206844_at	1.82e-05	9.61e-06	-4.477.242
206820_at	1.83e-05	9.69e-06	-4.475.589
216310_at	1.84e-05	9.70e-06	-4.475.184
217118_s_at	1.84e-05	9.72e-06	4.474.727
220600_at	1.84e-05	9.73e-06	-4.474.587
204899_s_at	1.84e-05	9.75e-06	4.474.192
203624_at	1.85e-05	9.77e-06	-4.473.598
208349_at	1.85e-05	9.78e-06	-4.473.401
213143_at	1.85e-05	9.80e-06	-4.473.027
208469_s_at	1.85e-05	9.80e-06	-4.472.893
216631_s_at	1.87e-05	9.89e-06	-4.470.929
213336_at	1.87e-05	9.91e-06	4.470.545
207618_s_at	1.87e-05	9.91e-06	4.470.388
204989_s_at	1.89e-05	9.98e-06	-4.468.917
217390_x_at	1.89e-05	1.00e-05	-4.467.983
37012_at	1.89e-05	1.00e-05	4.467.806
202664_at	1.90e-05	1.00e-05	-4.467.692
208918_s_at	1.90e-05	1.01e-05	-4.466.937
207246_at	1.90e-05	1.01e-05	-4.466.582

207692_s_at	1.91e-05	1.01e-05	-4.466.445
209213_at	1.91e-05	1.01e-05	-4.466.222
221327_s_at	1.91e-05	1.01e-05	-4.465.753
216916_s_at	1.92e-05	1.02e-05	-4.465.129
201078_at	1.93e-05	1.02e-05	4.464.036
208332_at	1.93e-05	1.02e-05	-4.463.945
210254_at	1.93e-05	1.02e-05	-4.463.832
209048_s_at	1.93e-05	1.02e-05	4.463.517
218713_at	1.93e-05	1.02e-05	4.463.135
207972_at	1.93e-05	1.03e-05	-4.462.935
208271_at	1.94e-05	1.03e-05	-4.462.623
204538_x_at	1.94e-05	1.03e-05	-4.462.119
219220_x_at	1.94e-05	1.03e-05	4.461.867
214509_at	1.95e-05	1.03e-05	-446.117
217888_s_at	1.95e-05	1.03e-05	4.461.053
217231_s_at	1.96e-05	1.04e-05	-4.459.663
206176_at	1.97e-05	1.05e-05	-4.458.328
212290_at	1.97e-05	1.05e-05	4.458.122
217925_s_at	1.98e-05	1.05e-05	4.457.753
202296_s_at	1.98e-05	1.05e-05	4.457.496
203526_s_at	1.98e-05	1.05e-05	4.456.979
204545_at	1.99e-05	1.05e-05	4.456.772
217450_at	2.00e-05	1.06e-05	-4.455.535
205255_x_at	2.00e-05	1.06e-05	-4.455.334
204199_at	2.00e-05	1.06e-05	4.455.043
213836_s_at	2.00e-05	1.06e-05	4.454.944
201626_at	2.00e-05	1.06e-05	-4.454.928
213514_s_at	2.00e-05	1.06e-05	-4.454.568
209005_at	2.02e-05	1.07e-05	4.452.631
204036_at	2.03e-05	1.08e-05	-4.451.882
206392_s_at	2.03e-05	1.08e-05	4.451.368
218345_at	2.03e-05	1.08e-05	-4.451.365
213468_at	2.03e-05	1.08e-05	4.451.133
206761_at	2.04e-05	1.08e-05	-4.451.045
201499_s_at	2.04e-05	1.08e-05	4.450.751
212427_at	2.05e-05	1.09e-05	4.449.922
205125_at	2.05e-05	1.09e-05	-4.449.329
201101_s_at	2.06e-05	1.09e-05	4.448.307
221432_s_at	2.07e-05	1.10e-05	-4.447.506
221233_s_at	2.07e-05	1.10e-05	-4.447.453
219977_at	2.07e-05	1.10e-05	-4.447.059
204369_at	2.07e-05	1.10e-05	4.447.001
205564_at	2.08e-05	1.10e-05	4.446.443
217040_x_at	2.08e-05	1.10e-05	-4.446.253

202284_s_at	2.08e-05	1.11e-05	-4.445.739
202921_s_at	2.08e-05	1.11e-05	-4.445.579
216185_at	2.09e-05	1.11e-05	-4.444.604
206297_at	2.09e-05	1.11e-05	-4.444.596
207260_at	2.09e-05	1.11e-05	-4.444.593
218967_s_at	2.09e-05	1.11e-05	-4.444.377
211643_x_at	2.09e-05	1.11e-05	-4.444.324
216416_at	2.10e-05	1.12e-05	-4.443.195
218313_s_at	2.11e-05	1.12e-05	4.442.693
221425_s_at	2.11e-05	1.12e-05	4.442.347
219005_at	2.11e-05	1.12e-05	-4.442.223
204334_at	2.11e-05	1.12e-05	-4.442.078
202600_s_at	2.12e-05	1.13e-05	-4.441.902
221019_s_at	2.12e-05	1.13e-05	4.441.734
212417_at	2.12e-05	1.13e-05	4.441.084
206294_at	2.12e-05	1.13e-05	-444.106
215617_at	2.13e-05	1.14e-05	-4.439.865
213213_at	2.14e-05	1.14e-05	4.438.992
207497_s_at	2.14e-05	1.14e-05	-4.438.892
203389_at	2.15e-05	1.14e-05	-4.438.689
213697_at	2.16e-05	1.15e-05	-4.436.744
214453_s_at	2.17e-05	1.16e-05	-4.435.877
210202_s_at	2.19e-05	1.16e-05	4.434.459
211603_s_at	2.19e-05	1.16e-05	4.434.447
217849_s_at	2.20e-05	1.17e-05	4.433.299
207280_at	2.22e-05	1.18e-05	-4.430.691
215368_at	2.23e-05	1.19e-05	-4.429.808
216473_x_at	2.23e-05	1.19e-05	-4.429.773
216828_at	2.23e-05	1.19e-05	-4.429.589
220827_at	2.23e-05	1.19e-05	-4.429.405
210422_x_at	2.25e-05	1.20e-05	-4.428.131
211069_s_at	2.25e-05	1.20e-05	4.427.448
205104_at	2.26e-05	1.20e-05	-4.427.186
216729_at	2.26e-05	1.20e-05	-4.427.028
207507_s_at	2.26e-05	1.21e-05	4.426.489
203038_at	2.26e-05	1.21e-05	4.426.451
205875_s_at	2.27e-05	1.21e-05	-4.425.914
216030_s_at	2.28e-05	1.21e-05	-4.424.929
215181_at	2.28e-05	1.22e-05	-442.445
201854_s_at	2.29e-05	1.22e-05	-4.423.979
217552_x_at	2.29e-05	1.22e-05	-4.423.643
209739_s_at	2.29e-05	1.22e-05	-4.423.503
203861_s_at	2.29e-05	1.22e-05	4.423.318
202654_x_at	2.30e-05	1.23e-05	4.422.695

220902_at	2.32e-05	1.24e-05	-4.420.225
207772_s_at	2.33e-05	1.24e-05	-4.419.582
210819_x_at	2.33e-05	1.24e-05	-4.419.563
203379_at	2.34e-05	1.25e-05	-4.418.327
205318_at	2.35e-05	1.25e-05	-4.417.849
219352_at	2.35e-05	1.25e-05	-4.417.826
209200_at	2.35e-05	1.26e-05	4.417.449
217240_at	2.36e-05	1.26e-05	-4.416.812
200086_s_at	2.37e-05	1.27e-05	-4.415.528
214560_at	2.37e-05	1.27e-05	-4.415.133
209042_s_at	2.38e-05	1.27e-05	-4.414.971
210832_x_at	2.38e-05	1.27e-05	-4.414.954
217476_at	2.38e-05	1.27e-05	-4.414.104
216262_s_at	2.38e-05	1.27e-05	44.141
208409_at	2.39e-05	1.28e-05	-4.413.863
219305_x_at	2.39e-05	1.28e-05	-4.413.788
217193_x_at	2.39e-05	1.28e-05	-4.413.106
208334_at	2.40e-05	1.28e-05	-4.412.571
212757_s_at	2.41e-05	1.29e-05	4.411.921
205438_at	2.41e-05	1.29e-05	-4.411.895
201490_s_at	2.41e-05	1.29e-05	-4.411.415
37254_at	2.41e-05	1.29e-05	441.135
216503_s_at	2.42e-05	1.29e-05	4.411.013
202092_s_at	2.42e-05	1.29e-05	4.411.009
201530_x_at	2.42e-05	1.29e-05	4.410.988
209357_at	2.42e-05	1.29e-05	-4.410.751
220917_s_at	2.43e-05	1.30e-05	4.410.043
217170_at	2.43e-05	1.30e-05	-4.409.871
217057_s_at	2.43e-05	1.30e-05	-4.409.646
200734_s_at	2.43e-05	1.30e-05	4.409.456
213505_s_at	2.43e-05	1.30e-05	4.409.448
217854_s_at	2.43e-05	1.30e-05	4.409.121
212039_x_at	2.44e-05	1.31e-05	4.408.526
201190_s_at	2.44e-05	1.31e-05	-4.408.131
207846_at	2.45e-05	1.31e-05	-4.407.959
205257_s_at	2.45e-05	1.31e-05	-4.407.873
203900_at	2.45e-05	1.31e-05	-4.407.453
203439_s_at	2.46e-05	1.31e-05	440.691
207708_at	2.46e-05	1.32e-05	-4.406.437
219898_at	2.47e-05	1.32e-05	-4.405.248
209764_at	2.48e-05	1.33e-05	-4.405.083
207397_s_at	2.49e-05	1.33e-05	-4.403.668
205237_at	2.50e-05	1.34e-05	-4.403.012
214207_s_at	2.50e-05	1.34e-05	-4.402.606

219930_at	2.51e-05	1.34e-05	-4.402.122
211380_s_at	2.51e-05	1.35e-05	-4.401.537
204338_s_at	2.53e-05	1.36e-05	-4.399.674
215763_at	2.55e-05	1.37e-05	-4.398.291
220992_s_at	2.55e-05	1.37e-05	4.398.191
216227_at	2.56e-05	1.37e-05	-4.397.022
213724_s_at	2.60e-05	1.39e-05	-4.394.072
202614_at	2.60e-05	1.39e-05	4.393.981
220720_x_at	2.60e-05	1.39e-05	-4.393.835
207920_x_at	2.60e-05	1.39e-05	4.393.554
207389_at	2.61e-05	1.40e-05	-4.393.065
206693_at	2.61e-05	1.40e-05	4.392.931
200774_at	2.61e-05	1.40e-05	4.392.754
203260_at	2.61e-05	1.40e-05	4.392.417
218671_s_at	2.61e-05	1.40e-05	-4.392.409
207231_at	2.61e-05	1.40e-05	4.392.409
215394_at	2.62e-05	1.41e-05	-4.391.618
212355_at	2.63e-05	1.41e-05	4.391.004
213541_s_at	2.64e-05	1.41e-05	-4.390.532
211518_s_at	2.64e-05	1.41e-05	439.047
217112_at	2.64e-05	1.42e-05	-4.390.019
207973_x_at	2.64e-05	1.42e-05	-438.995
213948_x_at	2.65e-05	1.42e-05	-4.389.246
207240_s_at	2.65e-05	1.42e-05	-4.389.172
217974_at	2.65e-05	1.42e-05	-4.389.078
213131_at	2.67e-05	1.43e-05	-4.387.736
209491_s_at	2.68e-05	1.44e-05	-4.386.756
217947_at	2.68e-05	1.44e-05	-4.386.751
218076_s_at	2.68e-05	1.44e-05	4.386.587
205602_x_at	2.68e-05	1.44e-05	-4.386.442
215059_at	2.68e-05	1.44e-05	-438.626
217866_at	2.69e-05	1.44e-05	-438.597
211233_x_at	2.69e-05	1.44e-05	-4.385.769
205180_s_at	2.69e-05	1.44e-05	-4.385.586
212975_at	2.70e-05	1.45e-05	-4.385.137
214552_s_at	2.70e-05	1.45e-05	-4.385.083
215962_at	2.70e-05	1.45e-05	-438.485
218565_at	2.70e-05	1.45e-05	4.384.564
211033_s_at	2.70e-05	1.45e-05	4.384.557
211182_x_at	2.71e-05	1.45e-05	-438.418
216516_at	2.71e-05	1.46e-05	-4.383.569
202183_s_at	2.73e-05	1.47e-05	-4.381.923
202854_at	2.74e-05	1.47e-05	438.095
214595_at	2.76e-05	1.48e-05	-4.379.813

202002_at	2.76e-05	1.48e-05	-4.379.512
210385_s_at	2.76e-05	1.48e-05	4.379.374
201932_at	2.76e-05	1.49e-05	4.379.317
220313_at	2.76e-05	1.49e-05	4.379.275
219751_at	2.77e-05	1.49e-05	-4.378.851
215085_x_at	2.77e-05	1.49e-05	-4.378.837
216181_at	2.80e-05	1.50e-05	-437.659
217209_at	2.80e-05	1.50e-05	-4.376.384
202669_s_at	2.82e-05	1.52e-05	4.374.651
212740_at	2.82e-05	1.52e-05	-4.374.483
200664_s_at	2.82e-05	1.52e-05	4.374.343
204562_at	2.84e-05	1.53e-05	-4.372.885
219423_x_at	2.84e-05	1.53e-05	4.372.738
205004_at	2.84e-05	1.53e-05	437.273
219505_at	2.85e-05	1.53e-05	-4.372.149
203665_at	2.85e-05	1.53e-05	-4.372.129
221309_at	2.85e-05	1.53e-05	-4.372.069
204795_at	2.85e-05	1.53e-05	4.371.923
219028_at	2.87e-05	1.54e-05	-4.370.704
202100_at	2.87e-05	1.54e-05	4.370.322
214417_s_at	2.87e-05	1.55e-05	-4.370.317
207890_s_at	2.88e-05	1.55e-05	-4.369.794
205464_at	2.90e-05	1.56e-05	-4.368.281
215665_at	2.93e-05	1.58e-05	-4.365.696
221865_at	2.93e-05	1.58e-05	4.365.615
219458_s_at	2.93e-05	1.58e-05	4.365.446
207199_at	2.93e-05	1.58e-05	4.365.275
206299_at	2.94e-05	1.58e-05	4.364.941
216076_at	2.94e-05	1.58e-05	-4.364.502
212326_at	2.95e-05	1.59e-05	-4.363.723
215118_s_at	2.96e-05	1.59e-05	-4.363.176
220394_at	2.97e-05	1.60e-05	-436.248
208488_s_at	2.97e-05	1.60e-05	-4.362.393
217351_at	2.97e-05	1.60e-05	-4.362.092
214364_at	2.98e-05	1.60e-05	-436.182
207634_at	2.98e-05	1.60e-05	-4.361.776
202698_x_at	2.98e-05	1.60e-05	-4.361.705
214301_s_at	2.98e-05	1.61e-05	-436.152
215329_s_at	3.00e-05	1.62e-05	-4.360.145
219492_at	3.00e-05	1.62e-05	-4.360.067
215237_at	3.00e-05	1.62e-05	-4.359.957
205586_x_at	3.00e-05	1.62e-05	-435.971
216988_s_at	3.00e-05	1.62e-05	4.359.413
208636_at	3.01e-05	1.63e-05	4.358.656

213308_at	3.02e-05	1.63e-05	4.358.519
213032_at	3.02e-05	1.63e-05	-4.358.407
209262_s_at	3.04e-05	1.64e-05	4.356.863
220217_x_at	3.05e-05	1.65e-05	-4.355.831
210333_at	3.05e-05	1.65e-05	-4.355.682
210702_s_at	3.06e-05	1.65e-05	-4.355.249
205018_s_at	3.06e-05	1.65e-05	-4.355.206
202883_s_at	3.06e-05	1.65e-05	-4.354.926
208225_at	3.07e-05	1.66e-05	-4.354.107
211502_s_at	3.08e-05	1.66e-05	4.353.812
219368_at	3.09e-05	1.67e-05	4.353.129
218858_at	3.09e-05	1.67e-05	4.352.816
201905_s_at	3.09e-05	1.67e-05	4.352.417
213960_at	3.10e-05	1.67e-05	-4.352.262
219173_at	3.10e-05	1.68e-05	-4.351.719
203816_at	3.11e-05	1.68e-05	4.351.219
201957_at	3.12e-05	1.68e-05	-4.350.605
218999_at	3.12e-05	1.68e-05	-4.350.547
201822_at	3.12e-05	1.69e-05	4.350.438
211351_at	3.12e-05	1.69e-05	-4.350.253
206109_at	3.13e-05	1.69e-05	4.349.934
202974_at	3.13e-05	1.69e-05	-4.349.745
218736_s_at	3.13e-05	1.69e-05	-4.349.654
214876_s_at	3.14e-05	1.69e-05	4.349.215
213527_s_at	3.14e-05	1.70e-05	-4.349.022
202684_s_at	3.14e-05	1.70e-05	4.348.881
221371_at	3.15e-05	1.70e-05	-4.347.978
209967_s_at	3.15e-05	1.70e-05	-4.347.851
217858_s_at	3.16e-05	1.71e-05	-4.347.367
209231_s_at	3.17e-05	1.71e-05	4.346.502
207711_at	3.18e-05	1.72e-05	4.346.156
213000_at	3.19e-05	1.73e-05	-4.345.103
207215_at	3.19e-05	1.73e-05	-4.344.919
214379_at	3.19e-05	1.73e-05	-4.344.747
220520_s_at	3.21e-05	1.73e-05	4.343.916
206742_at	3.21e-05	1.74e-05	4.343.322
213327_s_at	3.21e-05	1.74e-05	-4.343.283
201142_at	3.22e-05	1.74e-05	4.343.181
214222_at	3.22e-05	1.74e-05	-4.343.065
204394_at	3.22e-05	1.74e-05	-4.343.022
214157_at	3.22e-05	1.74e-05	4.342.661
217190_x_at	3.22e-05	1.74e-05	-434.261
208376_at	3.22e-05	1.75e-05	-4.342.501
213738_s_at	3.22e-05	1.75e-05	4.342.495

218770_s_at	3.22e-05	1.75e-05	4.342.434
207390_s_at	3.23e-05	1.75e-05	4.341.721
210787_s_at	3.24e-05	1.75e-05	-4.341.524
210288_at	3.25e-05	1.76e-05	-4.340.377
216760_at	3.26e-05	1.76e-05	-4.339.996
216439_at	3.26e-05	1.77e-05	-4.339.723
212012_at	3.27e-05	1.77e-05	-4.339.363
206766_at	3.27e-05	1.77e-05	-4.338.995
222078_at	3.27e-05	1.77e-05	-4.338.923
221376_at	3.27e-05	1.77e-05	-4.338.861
207373_at	3.29e-05	1.78e-05	-4.337.527
204336_s_at	3.29e-05	1.79e-05	4.337.318
204235_s_at	3.30e-05	1.79e-05	4.337.131
209192_x_at	3.31e-05	1.79e-05	4.336.368
201845_s_at	3.31e-05	1.79e-05	4.336.317
221683_s_at	3.31e-05	1.79e-05	4.336.231
214561_at	3.32e-05	1.80e-05	-4.335.774
218018_at	3.32e-05	1.80e-05	4.335.249
219929_s_at	3.33e-05	1.81e-05	-4.334.752
221011_s_at	3.34e-05	1.81e-05	-4.334.075
207004_at	3.35e-05	1.82e-05	-4.333.468
214067_at	3.36e-05	1.82e-05	-4.332.798
220228_at	3.36e-05	1.82e-05	-4.332.715
221329_at	3.37e-05	1.83e-05	-4.332.014
216273_at	3.37e-05	1.83e-05	-4.331.811
201108_s_at	3.37e-05	1.83e-05	-4.331.694
207962_at	3.38e-05	1.83e-05	-4.331.065
210790_s_at	3.39e-05	1.84e-05	4.330.269
207650_x_at	3.40e-05	1.85e-05	-4.329.517
208563_x_at	3.42e-05	1.85e-05	-4.328.674
221154_at	3.45e-05	1.87e-05	-4.326.639
211315_s_at	3.45e-05	1.87e-05	-432.631
217296_at	3.45e-05	1.87e-05	-4.326.259
214939_x_at	3.45e-05	1.88e-05	-4.325.982
208018_s_at	3.45e-05	1.88e-05	-4.325.977
212677_s_at	3.46e-05	1.88e-05	4.325.894
221081_s_at	3.46e-05	1.88e-05	-4.325.377
217856_at	3.47e-05	1.89e-05	4.324.743
217896_s_at	3.49e-05	1.90e-05	4.323.586
220610_s_at	3.50e-05	1.90e-05	4.323.022
207680_x_at	3.50e-05	1.90e-05	-432.271
219261_at	3.50e-05	1.90e-05	4.322.553
202363_at	3.51e-05	1.91e-05	432.224
222104_x_at	3.51e-05	1.91e-05	-432.219

220518_at	3.51e-05	1.91e-05	-4.322.184
214550_s_at	3.51e-05	1.91e-05	4.321.816
204991_s_at	3.51e-05	1.91e-05	-4.321.773
212840_at	3.52e-05	1.91e-05	4.321.201
216931_at	3.53e-05	1.92e-05	-4.320.849
202700_s_at	3.53e-05	1.92e-05	-4.320.551
200633_at	3.54e-05	1.92e-05	-4.320.333
216126_at	3.57e-05	1.94e-05	-4.318.141
218005_at	3.58e-05	1.95e-05	4.317.497
208907_s_at	3.58e-05	1.95e-05	431.714
208930_s_at	3.60e-05	1.96e-05	4.316.259
214487_s_at	3.60e-05	1.96e-05	4.316.217
204347_at	3.60e-05	1.96e-05	-4.315.907
204729_s_at	3.61e-05	1.96e-05	4.315.545
206476_s_at	3.62e-05	1.97e-05	-4.314.502
221593_s_at	3.63e-05	1.97e-05	4.314.179
202665_s_at	3.66e-05	1.99e-05	-4.312.246
204195_s_at	3.67e-05	2.00e-05	4.311.769
212760_at	3.67e-05	2.00e-05	-4.311.287
201055_s_at	3.68e-05	2.00e-05	4.311.011
216369_at	3.68e-05	2.00e-05	-4.310.951
219790_s_at	3.68e-05	2.00e-05	-4.310.746
219511_s_at	3.69e-05	2.01e-05	4.310.339
217618_x_at	3.70e-05	2.01e-05	430.974
208100_x_at	3.70e-05	2.02e-05	-4.309.467
209332_s_at	3.70e-05	2.02e-05	4.309.312
217295_at	3.71e-05	2.02e-05	-4.309.078
214385_s_at	3.72e-05	2.02e-05	-4.308.409
200868_s_at	3.73e-05	2.03e-05	4.307.773
211006_s_at	3.73e-05	2.03e-05	-4.307.666
220421_at	3.73e-05	2.03e-05	4.307.611
215685_s_at	3.73e-05	2.03e-05	-4.307.486
213055_at	3.73e-05	2.03e-05	-430.732
215924_at	3.73e-05	2.03e-05	-4.307.254
201824_at	3.74e-05	2.04e-05	4.307.147
211640_x_at	3.74e-05	2.04e-05	-4.306.858
206337_at	3.77e-05	2.05e-05	-4.305.241
214950_at	3.77e-05	2.06e-05	-4.304.682
212576_at	3.78e-05	2.06e-05	-4.304.266
211175_at	3.78e-05	2.06e-05	-4.304.099
220837_at	3.78e-05	2.06e-05	-4.304.092
221663_x_at	3.79e-05	2.07e-05	-4.303.743
213005_s_at	3.80e-05	2.07e-05	-4.302.865
202951_at	3.80e-05	2.07e-05	4.302.764

219228_at	3.81e-05	2.08e-05	430.214
211004_s_at	3.82e-05	2.09e-05	-4.301.498
220098_at	3.83e-05	2.09e-05	-4.301.416
208254_at	3.83e-05	2.09e-05	-430.101
216864_at	3.84e-05	2.10e-05	-4.300.347
220596_at	3.86e-05	2.11e-05	-4.299.061
221266_s_at	3.88e-05	2.12e-05	-4.298.305
202076_at	3.88e-05	2.12e-05	4.297.866
215747_s_at	3.89e-05	2.12e-05	-4.297.463
214219_x_at	3.90e-05	2.13e-05	-4.296.735
219748_at	3.91e-05	2.13e-05	-4.296.419
210158_at	3.91e-05	2.14e-05	-4.295.986
201705_at	3.92e-05	2.14e-05	4.295.677
213742_at	3.93e-05	2.15e-05	-429.474
219567_s_at	3.94e-05	2.15e-05	-4.294.323
204879_at	3.94e-05	2.15e-05	-4.294.109
220640_at	3.97e-05	2.17e-05	-4.292.877
203203_s_at	3.97e-05	2.17e-05	4.292.529
202080_s_at	3.98e-05	2.17e-05	4.291.886
200844_s_at	3.99e-05	2.18e-05	-4.291.512
218129_s_at	3.99e-05	2.18e-05	4.291.457
209876_at	4.00e-05	2.18e-05	4.291.044
209985_s_at	4.01e-05	2.19e-05	-4.290.437
221678_at	4.02e-05	2.20e-05	-4.289.497
207985_at	4.02e-05	2.20e-05	-4.289.369
207586_at	4.03e-05	2.20e-05	-4.289.049
215992_s_at	4.03e-05	2.20e-05	-4.289.005
202498_s_at	4.03e-05	2.20e-05	-4.288.853
210735_s_at	4.05e-05	2.21e-05	4.287.677
217151_at	4.05e-05	2.22e-05	-428.757
216727_at	4.06e-05	2.22e-05	-4.287.342
212347_x_at	4.06e-05	2.22e-05	4.287.331
217990_at	4.07e-05	2.23e-05	4.286.392
215334_at	4.07e-05	2.23e-05	-4.286.296
209107_x_at	4.08e-05	2.23e-05	4.286.173
205409_at	4.08e-05	2.23e-05	-42.857
202425_x_at	4.10e-05	2.24e-05	-4.284.932
204390_at	4.10e-05	2.24e-05	-4.284.589
208737_at	4.11e-05	2.25e-05	4.284.106
212668_at	4.16e-05	2.27e-05	-4.281.459
212641_at	4.17e-05	2.28e-05	4.280.619
221413_at	4.18e-05	2.29e-05	-4.280.297
205638_at	4.18e-05	2.29e-05	-4.279.936
216653_at	4.20e-05	2.30e-05	-4.279.235

201300_s_at	4.20e-05	2.30e-05	-4.279.168
221075_s_at	4.21e-05	2.30e-05	-4.278.633
206901_at	4.23e-05	2.31e-05	-4.277.581
209147_s_at	4.23e-05	2.32e-05	4.277.134
206489_s_at	4.24e-05	2.32e-05	-4.276.678
215564_at	4.25e-05	2.33e-05	-4.276.176
217985_s_at	4.27e-05	2.34e-05	4.275.035
58994_at	4.28e-05	2.35e-05	-4.274.377
210626_at	4.28e-05	2.35e-05	-4.274.309
220517_at	4.29e-05	2.35e-05	-4.273.982
214187_x_at	4.29e-05	2.35e-05	-4.273.716
2028_s_at	4.31e-05	2.36e-05	4.272.961
216926_s_at	4.31e-05	2.36e-05	-4.272.872
206012_at	4.31e-05	2.36e-05	-427.277
201162_at	4.32e-05	2.37e-05	-4.272.138
220220_at	4.33e-05	2.37e-05	-4.271.914
206034_at	4.33e-05	2.37e-05	-4.271.864
213062_at	4.34e-05	2.38e-05	4.271.066
217437_s_at	4.34e-05	2.38e-05	4.270.939
219677_at	4.34e-05	2.38e-05	4.270.809
200917_s_at	4.35e-05	2.39e-05	-4.270.353
202250_s_at	4.36e-05	2.39e-05	-426.977
210518_at	4.41e-05	2.42e-05	-4.267.479
207058_s_at	4.42e-05	2.42e-05	-4.266.967
203478_at	4.43e-05	2.43e-05	-4.266.279
203999_at	4.44e-05	2.43e-05	426.591
207519_at	4.44e-05	2.44e-05	-4.265.619
209166_s_at	4.45e-05	2.44e-05	4.265.224
219621_at	4.46e-05	2.45e-05	-4.264.371
215175_at	4.47e-05	2.45e-05	-4.264.162
214747_at	4.47e-05	2.45e-05	4.263.999
216771_at	4.47e-05	2.46e-05	-4.263.744
215696_s_at	4.48e-05	2.46e-05	-4.263.666
213111_at	4.48e-05	2.46e-05	4.263.608
205118_at	4.48e-05	2.46e-05	-4.263.494
219843_at	4.50e-05	2.47e-05	4.262.173
202222_s_at	4.51e-05	2.47e-05	-4.261.909
206255_at	4.51e-05	2.48e-05	-4.261.542
207555_s_at	4.54e-05	2.49e-05	-4.260.108
213633_at	4.55e-05	2.50e-05	-4.259.699
215278_at	4.56e-05	2.50e-05	-4.259.222
216453_at	4.59e-05	2.52e-05	-4.257.854
209145_s_at	4.59e-05	2.52e-05	4.257.663
209580_s_at	4.59e-05	2.52e-05	4.257.591

216024_at	4.60e-05	2.52e-05	-4.257.235
204316_at	4.60e-05	2.53e-05	-4.256.888
201123_s_at	4.63e-05	2.54e-05	-4.255.544
208567_s_at	4.63e-05	2.54e-05	-4.255.406
202780_at	4.65e-05	2.55e-05	4.254.575
208496_x_at	4.65e-05	2.56e-05	4.254.366
221724_s_at	4.66e-05	2.56e-05	-4.254.022
202150_s_at	4.72e-05	2.59e-05	4.250.929
205387_s_at	4.72e-05	2.60e-05	-4.250.801
220715_at	4.75e-05	2.61e-05	-4.249.588
210352_at	4.75e-05	2.61e-05	-4.249.501
220306_at	4.75e-05	2.61e-05	-424.922
220582_at	4.75e-05	2.61e-05	-4.249.169
216128_at	4.76e-05	2.62e-05	-424.902
211452_x_at	4.76e-05	2.62e-05	-4.248.698
221680_s_at	4.78e-05	2.63e-05	-4.247.776
219235_s_at	4.79e-05	2.64e-05	-4.247.189
201013_s_at	4.83e-05	2.66e-05	4.245.435
211377_x_at	4.85e-05	2.67e-05	-4.244.381
217601_at	4.85e-05	2.67e-05	-4.244.317
205996_s_at	4.85e-05	2.67e-05	-4.244.253
201022_s_at	4.85e-05	2.67e-05	4.244.208
204494_s_at	4.85e-05	2.67e-05	4.244.101
211254_x_at	4.86e-05	2.67e-05	-4.243.972
208461_at	4.86e-05	2.67e-05	-4.243.847
218096_at	4.87e-05	2.68e-05	4.243.148
205316_at	4.88e-05	2.69e-05	-4.242.696
202766_s_at	4.89e-05	2.69e-05	4.242.334
218508_at	4.89e-05	2.69e-05	4.242.132
211350_s_at	4.91e-05	2.70e-05	-4.241.404
213400_s_at	4.91e-05	2.70e-05	-4.241.353
216100_s_at	4.91e-05	2.70e-05	4.241.319
213497_at	4.91e-05	2.70e-05	-4.241.229
215531_s_at	4.95e-05	2.73e-05	-4.239.315
213961_s_at	4.95e-05	2.73e-05	-4.239.093
205900_at	4.97e-05	2.74e-05	-4.238.493
206565_x_at	4.97e-05	2.74e-05	4.238.303
205108_s_at	4.97e-05	2.74e-05	-4.238.139
219893_at	4.98e-05	2.74e-05	-4.237.775
218178_s_at	4.98e-05	2.75e-05	4.237.686
209954_x_at	4.99e-05	2.75e-05	-4.237.406
207600_at	4.99e-05	2.75e-05	-423.732
211263_s_at	5.03e-05	2.77e-05	-423.548
215966_x_at	5.03e-05	2.77e-05	-4.235.271

210935_s_at	5.03e-05	2.78e-05	4.235.145
207664_at	5.04e-05	2.78e-05	-4.235.065
216736_at	5.05e-05	2.78e-05	-4.234.472
204335_at	5.05e-05	2.79e-05	4.234.204
56197_at	5.06e-05	2.79e-05	-4.234.002
214248_s_at	5.07e-05	2.79e-05	-4.233.586
209494_s_at	5.07e-05	2.79e-05	4.233.572
40640_at	5.08e-05	2.80e-05	-4.232.901
215561_s_at	5.09e-05	2.81e-05	-4.232.512
220947_s_at	5.10e-05	2.81e-05	4.232.032
221594_at	5.10e-05	2.81e-05	-4.231.879
201600_at	5.13e-05	2.83e-05	-4.230.503
215313_x_at	5.14e-05	2.83e-05	423.022
207488_at	5.16e-05	2.85e-05	-4.229.038
217679_x_at	5.17e-05	2.85e-05	-4.228.868
203524_s_at	5.17e-05	2.85e-05	-4.228.682
207968_s_at	5.17e-05	2.86e-05	-4.228.449
209256_s_at	5.18e-05	2.86e-05	-4.228.184
221503_s_at	5.18e-05	2.86e-05	4.228.172
208089_s_at	5.19e-05	2.87e-05	4.227.651
216143_at	5.20e-05	2.87e-05	-4.227.323
204298_s_at	5.20e-05	2.87e-05	4.227.307
203574_at	5.20e-05	2.87e-05	-4.227.194
212037_at	5.20e-05	2.87e-05	4.226.998
211812_s_at	5.22e-05	2.88e-05	4.226.339
209726_at	5.23e-05	2.89e-05	-4.225.997
208449_s_at	5.23e-05	2.89e-05	-4.225.917
218264_at	5.25e-05	2.90e-05	4.224.966
208134_x_at	5.25e-05	2.90e-05	-4.224.693
221682_s_at	5.26e-05	2.91e-05	-4.224.257
210159_s_at	5.26e-05	2.91e-05	4.224.145
218733_at	5.27e-05	2.91e-05	4.223.961
201649_at	5.27e-05	2.91e-05	-4.223.843
202158_s_at	5.29e-05	2.92e-05	-4.223.022
212203_x_at	5.29e-05	2.93e-05	4.222.722
202050_s_at	5.30e-05	2.93e-05	4.222.578
222269_at	5.30e-05	2.93e-05	4.222.245
206660_at	5.31e-05	2.94e-05	-4.221.912
215908_at	5.32e-05	2.94e-05	-4.221.489
204910_s_at	5.33e-05	2.95e-05	-4.221.149
210312_s_at	5.37e-05	2.97e-05	4.219.461
211624_s_at	5.37e-05	2.97e-05	-421.908
204542_at	5.39e-05	2.98e-05	4.218.587
202065_s_at	5.39e-05	2.98e-05	4.218.378

203602_s_at	5.40e-05	2.99e-05	4.217.736
214362_at	5.41e-05	2.99e-05	-4.217.347
219758_at	5.41e-05	3.00e-05	4.217.279
204903_x_at	5.43e-05	3.00e-05	4.216.563
220709_at	5.44e-05	3.01e-05	-421.625
205838_at	5.44e-05	3.01e-05	-4.216.248
201617_x_at	5.46e-05	3.02e-05	-4.215.141
214682_at	5.46e-05	3.02e-05	-4.215.139
206689_x_at	5.47e-05	3.03e-05	4.214.836
214536_at	5.47e-05	3.03e-05	-4.214.759
212379_at	5.48e-05	3.03e-05	4.214.472
214544_s_at	5.48e-05	3.04e-05	4.214.199
203086_at	5.49e-05	3.04e-05	4.213.901
221379_at	5.49e-05	3.04e-05	-4.213.614
214059_at	5.50e-05	3.05e-05	-4.213.383
202696_at	5.50e-05	3.05e-05	4.213.359
219551_at	5.50e-05	3.05e-05	4.213.335
201839_s_at	5.50e-05	3.05e-05	4.213.284
203350_at	5.50e-05	3.05e-05	-4.213.108
218128_at	5.52e-05	3.06e-05	4.212.327
210363_s_at	5.52e-05	3.06e-05	-4.212.184
214524_at	5.54e-05	3.07e-05	-4.211.576
211000_s_at	5.55e-05	3.08e-05	-4.210.883
202987_at	5.56e-05	3.08e-05	-4.210.755
201069_at	5.56e-05	3.08e-05	-42.105
218764_at	5.57e-05	3.09e-05	-4.210.025
202855_s_at	5.58e-05	3.09e-05	4.209.633
208396_s_at	5.58e-05	3.10e-05	-4.209.526
207032_s_at	5.59e-05	3.10e-05	-4.209.468
203513_at	5.59e-05	3.10e-05	4.209.371
216220_s_at	5.60e-05	3.11e-05	-4.208.621
211463_at	5.61e-05	3.11e-05	-4.208.501
205953_at	5.61e-05	3.11e-05	4.208.451
216627_s_at	5.64e-05	3.13e-05	-4.207.177
204193_at	5.65e-05	3.13e-05	-4.206.752
219712_s_at	5.66e-05	3.14e-05	-4.206.327
203736_s_at	5.67e-05	3.15e-05	-4.205.592
AFFX-HSAC07/X0035	5.69e-05	3.16e-05	4.204.893
213913_s_at	5.72e-05	3.18e-05	4.203.577
218769_s_at	5.74e-05	3.19e-05	4.202.656
207089_at	5.75e-05	3.19e-05	-4.202.251
216528_at	5.76e-05	3.20e-05	-4.202.099
221101_at	5.77e-05	3.20e-05	-4.201.691
206822_s_at	5.77e-05	3.20e-05	4.201.487

205174_s_at	5.77e-05	3.21e-05	420.136
212700_x_at	5.79e-05	3.21e-05	-4.200.789
220068_at	5.80e-05	3.22e-05	-4.200.156
208162_s_at	5.83e-05	3.24e-05	-4.198.955
206289_at	5.83e-05	3.24e-05	-4.198.805
207861_at	5.84e-05	3.24e-05	-4.198.704
216533_at	5.85e-05	3.25e-05	-419.815
220611_at	5.85e-05	3.25e-05	-4.198.035
211657_at	5.87e-05	3.26e-05	-419.734
210876_at	5.87e-05	3.26e-05	4.197.247
208391_s_at	5.87e-05	3.26e-05	-4.197.141
222009_at	5.89e-05	3.28e-05	-4.196.295
205198_s_at	5.90e-05	3.28e-05	4.195.945
207876_s_at	5.92e-05	3.29e-05	4.195.381
213808_at	5.97e-05	3.32e-05	4.193.382
216695_s_at	5.98e-05	3.33e-05	-4.192.703
207709_at	5.99e-05	3.33e-05	4.192.412
52078_at	5.99e-05	3.33e-05	-4.192.284
1255_g_at	6.00e-05	3.34e-05	-4.191.916
222349_x_at	6.00e-05	3.34e-05	-41.919
203923_s_at	6.00e-05	3.34e-05	-4.191.778
215143_at	6.00e-05	3.34e-05	4.191.733
221914_at	6.01e-05	3.34e-05	-4.191.551
213406_at	6.01e-05	3.34e-05	-4.191.357
203710_at	6.02e-05	3.35e-05	4.191.042
205834_s_at	6.05e-05	3.37e-05	-4.189.708
217695_x_at	6.06e-05	3.37e-05	-4.189.486
211125_x_at	6.06e-05	3.37e-05	-4.189.424
211974_x_at	6.06e-05	3.37e-05	4.189.423
202084_s_at	6.09e-05	3.39e-05	4.188.144
211304_x_at	6.10e-05	3.40e-05	-4.187.655
201004_at	6.11e-05	3.40e-05	4.187.385
216464_x_at	6.12e-05	3.40e-05	-4.187.168
215534_at	6.12e-05	3.41e-05	-4.187.108
215474_at	6.12e-05	3.41e-05	4.187.084
49329_at	6.13e-05	3.41e-05	4.186.483
205220_at	6.15e-05	3.42e-05	418.587
220156_at	6.16e-05	3.43e-05	-4.185.494
202660_at	6.17e-05	3.44e-05	-4.184.958
220458_at	6.17e-05	3.44e-05	-4.184.929
212077_at	6.19e-05	3.45e-05	-4.184.037
214277_at	6.20e-05	3.46e-05	418.365
214532_x_at	6.21e-05	3.46e-05	-4.183.379
207514_s_at	6.21e-05	3.46e-05	-4.183.331

208474_at	6.21e-05	3.46e-05	-4.183.315
212472_at	6.22e-05	3.47e-05	-4.182.975
213550_s_at	6.23e-05	3.47e-05	4.182.494
214628_at	6.24e-05	3.48e-05	-4.182.108
205575_at	6.26e-05	3.49e-05	4.181.361
213020_at	6.27e-05	3.49e-05	-4.181.054
209065_at	6.27e-05	3.50e-05	418.079
214145_s_at	6.28e-05	3.50e-05	-4.180.667
209350_s_at	6.29e-05	3.50e-05	4.180.345
209407_s_at	6.29e-05	3.50e-05	4.180.325
212246_at	6.29e-05	3.51e-05	4.180.085
220192_x_at	6.30e-05	3.51e-05	-417.982
213568_at	6.34e-05	3.54e-05	4.178.181
206242_at	6.34e-05	3.54e-05	4.178.124
214994_at	6.35e-05	3.54e-05	-4.177.799
216307_at	6.36e-05	3.55e-05	-4.177.361
218040_at	6.39e-05	3.57e-05	4.176.171
208413_at	6.43e-05	3.59e-05	-4.174.729
31637_s_at	6.44e-05	3.59e-05	-4.174.562
208906_at	6.45e-05	3.60e-05	4.174.077
217160_at	6.46e-05	3.61e-05	-4.173.581
208340_at	6.50e-05	3.63e-05	-4.172.134
215186_at	6.51e-05	3.64e-05	-4.171.645
216682_s_at	6.53e-05	3.65e-05	-4.171.029
212162_at	6.53e-05	3.65e-05	4.170.978
214466_at	6.55e-05	3.66e-05	-4.170.267
212715_s_at	6.56e-05	3.66e-05	-4.169.779
218489_s_at	6.57e-05	3.67e-05	-416.966
220352_x_at	6.60e-05	3.69e-05	-4.168.421
204972_at	6.60e-05	3.69e-05	-416.825
207702_s_at	6.62e-05	3.70e-05	-4.167.786
210200_at	6.63e-05	3.70e-05	-4.167.317
214858_at	6.64e-05	3.71e-05	-4.166.896
204374_s_at	6.64e-05	3.71e-05	4.166.868
220804_s_at	6.65e-05	3.72e-05	-4.166.438
220743_at	6.69e-05	3.74e-05	-4.164.949
217555_at	6.71e-05	3.75e-05	-4.164.345
219895_at	6.72e-05	3.76e-05	4.163.896
204403_x_at	6.77e-05	3.78e-05	-4.162.141
210477_x_at	6.78e-05	3.79e-05	-4.161.974
215086_at	6.78e-05	3.79e-05	-4.161.863
218895_at	6.78e-05	3.79e-05	4.161.761
213940_s_at	6.80e-05	3.80e-05	4.161.135
204378_at	6.80e-05	3.80e-05	4.161.129

215540_at	6.84e-05	3.82e-05	-4.159.625
210816_s_at	6.85e-05	3.83e-05	-4.159.216
210015_s_at	6.87e-05	3.84e-05	4.158.553
221239_s_at	6.89e-05	3.86e-05	-4.157.709
219072_at	6.90e-05	3.86e-05	4.157.516
207587_at	6.90e-05	3.86e-05	-4.157.411
216040_x_at	6.90e-05	3.86e-05	-4.157.305
208311_at	6.91e-05	3.86e-05	-4.157.244
213249_at	6.91e-05	3.86e-05	4.157.218
220337_at	6.91e-05	3.87e-05	-4.157.071
214214_s_at	6.93e-05	3.88e-05	4.156.322
210967_x_at	6.94e-05	3.88e-05	-4.156.136
217690_at	6.94e-05	3.88e-05	-4.155.925
214992_s_at	6.95e-05	3.89e-05	4.155.521
217929_s_at	6.99e-05	3.91e-05	-4.154.315
205881_at	6.99e-05	3.91e-05	4.154.127
220266_s_at	7.01e-05	3.92e-05	-4.153.523
210624_s_at	7.01e-05	3.93e-05	-4.153.308
207042_at	7.02e-05	3.93e-05	-415.321
207865_s_at	7.03e-05	3.94e-05	-4.152.818
204656_at	7.04e-05	3.94e-05	-4.152.478
202942_at	7.04e-05	3.94e-05	-4.152.463
205460_at	7.09e-05	3.97e-05	4.150.839
216113_at	7.09e-05	3.97e-05	-4.150.751
36711_at	7.11e-05	3.98e-05	4.150.068
203055_s_at	7.11e-05	3.98e-05	-4.149.897
212880_at	7.11e-05	3.99e-05	-4.149.853
204279_at	7.12e-05	3.99e-05	414.964
206490_at	7.13e-05	3.99e-05	-4.149.369
204830_x_at	7.16e-05	4.01e-05	-4.148.294
210082_at	7.16e-05	4.02e-05	-4.148.061
216725_at	7.17e-05	4.02e-05	-4.147.917
216479_at	7.17e-05	4.02e-05	-4.147.737
213282_at	7.21e-05	4.04e-05	4.146.342
220995_at	7.22e-05	4.05e-05	-414.603
207205_at	7.23e-05	4.05e-05	-4.145.939
206158_s_at	7.23e-05	4.05e-05	-4.145.747
213935_at	7.25e-05	4.07e-05	-4.145.121
220291_at	7.27e-05	4.08e-05	-4.144.268
204723_at	7.30e-05	4.09e-05	-4.143.504
216357_at	7.30e-05	4.09e-05	-4.143.466
201827_at	7.31e-05	4.10e-05	4.142.897
216430_x_at	7.34e-05	4.12e-05	-4.142.147
202981_x_at	7.34e-05	4.12e-05	4.142.133

202267_at	7.35e-05	4.12e-05	-4.141.812
211635_x_at	7.36e-05	4.13e-05	-4.141.244
212844_at	7.37e-05	4.14e-05	-4.140.899
208278_s_at	7.39e-05	4.15e-05	-4.140.463
216374_at	7.39e-05	4.15e-05	-4.140.367
217054_at	7.43e-05	4.17e-05	-4.139.094
214971_s_at	7.44e-05	4.18e-05	-4.138.583
217569_x_at	7.45e-05	4.18e-05	-4.138.431
220387_s_at	7.46e-05	4.19e-05	-4.137.873
209869_at	7.50e-05	4.21e-05	-4.136.856
212298_at	7.50e-05	4.21e-05	413.674
208169_s_at	7.55e-05	4.24e-05	-4.135.159
201702_s_at	7.55e-05	4.24e-05	-4.134.916
219964_at	7.57e-05	4.25e-05	-4.134.501
205233_s_at	7.58e-05	4.26e-05	-4.133.943
54051_at	7.59e-05	4.26e-05	4.133.761
201219_at	7.60e-05	4.27e-05	-4.133.539
207904_s_at	7.60e-05	4.27e-05	41.335
213221_s_at	7.61e-05	4.27e-05	-4.133.163
201228_s_at	7.62e-05	4.28e-05	4.132.743
210335_at	7.63e-05	4.29e-05	-4.132.528
217038_at	7.63e-05	4.29e-05	-4.132.327
204169_at	7.66e-05	4.30e-05	4.131.485
216772_at	7.67e-05	4.31e-05	-4.130.939
209897_s_at	7.70e-05	4.33e-05	-4.130.232
217114_at	7.71e-05	4.33e-05	-4.129.877
210502_s_at	7.71e-05	4.34e-05	4.129.822
218396_at	7.71e-05	4.34e-05	412.981
220740_s_at	7.72e-05	4.34e-05	-4.129.502
61874_at	7.77e-05	4.37e-05	-4.127.865
210212_x_at	7.79e-05	4.38e-05	-4.127.185
208372_s_at	7.80e-05	4.39e-05	-412.706
217463_s_at	7.80e-05	4.39e-05	-4.126.986
207173_x_at	7.80e-05	4.39e-05	412.687
221971_x_at	7.80e-05	4.39e-05	-4.126.864
213218_at	7.81e-05	4.40e-05	4.126.542
206017_at	7.82e-05	4.40e-05	-412.626
202897_at	7.82e-05	4.40e-05	4.126.181
222352_at	7.82e-05	4.40e-05	-412.611
218197_s_at	7.83e-05	4.41e-05	4.125.982
216831_s_at	7.83e-05	4.41e-05	-4.125.795
209320_at	7.85e-05	4.42e-05	-4.125.219
220541_at	7.85e-05	4.42e-05	-4.125.201
207952_at	7.86e-05	4.43e-05	-4.124.887

215945_s_at	7.87e-05	4.43e-05	-4.124.591
206671_at	7.89e-05	4.44e-05	-4.123.945
200836_s_at	7.89e-05	4.44e-05	4.123.879
222060_at	7.90e-05	4.45e-05	4.123.537
205491_s_at	7.91e-05	4.45e-05	-412.338
203750_s_at	7.92e-05	4.46e-05	-4.122.933
205856_at	7.96e-05	4.49e-05	-412.157
203809_s_at	7.97e-05	4.49e-05	-4.121.297
213551_x_at	7.99e-05	4.50e-05	4.120.889
213138_at	8.01e-05	4.52e-05	-4.120.042
218822_s_at	8.04e-05	4.53e-05	4.119.376
206692_at	8.04e-05	4.53e-05	-4.119.267
210661_at	8.04e-05	4.53e-05	-4.119.189
220813_at	8.05e-05	4.54e-05	-4.118.893
213273_at	8.05e-05	4.54e-05	-4.118.881
202990_at	8.09e-05	4.56e-05	-4.117.688
208568_at	8.09e-05	4.56e-05	-4.117.502
206646_at	8.11e-05	4.57e-05	-4.117.102
214947_at	8.12e-05	4.58e-05	-411.659
208148_at	8.16e-05	4.60e-05	4.115.408
210180_s_at	8.20e-05	4.62e-05	4.114.479
211232_x_at	8.21e-05	4.63e-05	-4.113.994
222046_at	8.23e-05	4.64e-05	-4.113.429
221378_at	8.23e-05	4.64e-05	-4.113.364
206857_s_at	8.26e-05	4.66e-05	4.112.646
204523_at	8.26e-05	4.66e-05	4.112.616
210714_at	8.26e-05	4.66e-05	-4.112.574
211711_s_at	8.26e-05	4.66e-05	4.112.393
217892_s_at	8.26e-05	4.66e-05	4.112.391
221121_at	8.27e-05	4.67e-05	4.112.075
202550_s_at	8.34e-05	4.71e-05	4.110.161
213702_x_at	8.37e-05	4.72e-05	4.109.347
208903_at	8.38e-05	4.73e-05	-4.108.941
216673_at	8.40e-05	4.74e-05	-4.108.358
207092_at	8.41e-05	4.75e-05	-4.108.114
212339_at	8.41e-05	4.75e-05	4.108.006
216087_at	8.44e-05	4.76e-05	-4.107.221
205207_at	8.45e-05	4.77e-05	-4.106.889
214504_at	8.45e-05	4.77e-05	-4.106.713
207572_at	8.46e-05	4.78e-05	-4.106.606
214961_at	8.46e-05	4.78e-05	-4.106.345
201124_at	8.48e-05	4.79e-05	4.105.766
210773_s_at	8.49e-05	4.80e-05	-4.105.608
213777_s_at	8.55e-05	4.83e-05	-4.103.905

217587_at	8.55e-05	4.83e-05	-4.103.876
220246_at	8.56e-05	4.84e-05	-4.103.579
208261_x_at	8.57e-05	4.84e-05	-4.103.371
214558_at	8.58e-05	4.85e-05	-4.103.025
AFFX-DapX-M_at	8.58e-05	4.85e-05	-4.102.972
205271_s_at	8.58e-05	4.85e-05	-4.102.848
212932_at	8.58e-05	4.85e-05	4.102.835
202445_s_at	8.60e-05	4.86e-05	-4.102.313
209745_at	8.60e-05	4.86e-05	4.102.218
208967_s_at	8.61e-05	4.87e-05	4.101.906
221887_s_at	8.62e-05	4.88e-05	4.101.645
206839_at	8.62e-05	4.88e-05	-4.101.564
220901_at	8.63e-05	4.88e-05	-4.101.507
207895_at	8.66e-05	4.90e-05	4.100.675
211112_at	8.68e-05	4.91e-05	-4.099.845
201376_s_at	8.69e-05	4.92e-05	409.965
215871_at	8.70e-05	4.92e-05	-4.099.437
220225_at	8.70e-05	4.93e-05	-4.099.251
222302_at	8.71e-05	4.93e-05	-4.098.968
214231_s_at	8.72e-05	4.93e-05	-4.098.829
206142_at	8.73e-05	4.94e-05	4.098.493
216888_at	8.75e-05	4.95e-05	-4.097.934
215276_at	8.75e-05	4.96e-05	-4.097.759
221278_at	8.76e-05	4.96e-05	-409.766
216475_at	8.76e-05	4.96e-05	-4.097.575
220495_s_at	8.77e-05	4.96e-05	4.097.335
219802_at	8.82e-05	4.99e-05	4.095.906
220662_s_at	8.82e-05	4.99e-05	-40.959
217048_at	8.82e-05	5.00e-05	4.095.769
208234_x_at	8.83e-05	5.00e-05	-4.095.473
218437_s_at	8.85e-05	5.01e-05	-4.094.961
219089_s_at	8.86e-05	5.02e-05	-4.094.807
204498_s_at	8.86e-05	5.02e-05	-4.094.702
203980_at	8.87e-05	5.03e-05	409.431
207829_s_at	8.88e-05	5.03e-05	409.421
204892_x_at	8.88e-05	5.03e-05	409.415
215622_x_at	8.89e-05	5.04e-05	-4.093.926
212076_at	8.89e-05	5.04e-05	-4.093.903
215642_at	8.89e-05	5.04e-05	-4.093.704
219430_at	8.90e-05	5.04e-05	4.093.586
203255_at	8.93e-05	5.06e-05	4.092.696
221265_s_at	8.95e-05	5.07e-05	4.092.207
221539_at	8.95e-05	5.07e-05	4.092.156
209481_at	8.97e-05	5.08e-05	-4.091.588

203330_s_at	8.97e-05	5.09e-05	4.091.572
222118_at	8.97e-05	5.09e-05	4.091.473
213458_at	8.98e-05	5.09e-05	-4.091.265
217435_x_at	8.98e-05	5.09e-05	-4.091.175
213499_at	9.00e-05	5.10e-05	4.090.689
207021_at	9.00e-05	5.10e-05	-4.090.649
205509_at	9.00e-05	5.11e-05	-4.090.597
207539_s_at	9.03e-05	5.12e-05	-408.981
213642_at	9.04e-05	5.13e-05	4.089.575
206805_at	9.04e-05	5.13e-05	-4.089.501
212146_at	9.06e-05	5.14e-05	4.088.928
220866_at	9.11e-05	5.17e-05	-4.087.658
218007_s_at	9.11e-05	5.17e-05	-4.087.597
201360_at	9.13e-05	5.18e-05	-4.086.899
219388_at	9.15e-05	5.19e-05	-40.865
220806_x_at	9.15e-05	5.20e-05	-4.086.329
207095_at	9.18e-05	5.21e-05	-4.085.713
206559_x_at	9.19e-05	5.22e-05	408.538
221611_s_at	9.19e-05	5.22e-05	-4.085.283
210099_at	9.20e-05	5.23e-05	-4.084.994
208914_at	9.22e-05	5.24e-05	4.084.523
216773_at	9.23e-05	5.24e-05	-4.084.226
221352_at	9.25e-05	5.26e-05	-4.083.621
202477_s_at	9.25e-05	5.26e-05	4.083.607
211952_at	9.29e-05	5.28e-05	4.082.602
210841_s_at	9.29e-05	5.28e-05	-4.082.563
217227_x_at	9.34e-05	5.31e-05	-4.081.245
219734_at	9.36e-05	5.32e-05	-4.080.767
217648_at	9.37e-05	5.33e-05	-4.080.454
221949_at	9.38e-05	5.33e-05	-4.080.078
211086_x_at	9.40e-05	5.34e-05	-4.079.745
220177_s_at	9.40e-05	5.34e-05	4.079.577
203670_at	9.41e-05	5.35e-05	-4.079.284
215876_at	9.44e-05	5.36e-05	-4.078.672
220069_at	9.44e-05	5.37e-05	-4.078.429
213605_s_at	9.46e-05	5.38e-05	4.078.022
215613_at	9.46e-05	5.38e-05	-40.779
201741_x_at	9.49e-05	5.40e-05	-4.077.144
212414_s_at	9.53e-05	5.42e-05	4.076.303
215615_x_at	9.54e-05	5.43e-05	-4.075.885
215893_x_at	9.54e-05	5.43e-05	-4.075.816
219205_at	9.55e-05	5.43e-05	-4.075.659
205513_at	9.57e-05	5.45e-05	-4.074.968
202797_at	9.60e-05	5.46e-05	4.074.248

212812_at	9.60e-05	5.47e-05	4.074.206
208539_x_at	9.61e-05	5.47e-05	-407.391
209184_s_at	9.62e-05	5.48e-05	-4.073.673
201633_s_at	9.63e-05	5.48e-05	4.073.541
221543_s_at	9.64e-05	5.49e-05	-4.073.254
208381_s_at	9.66e-05	5.50e-05	-4.072.772
216747_at	9.66e-05	5.50e-05	-4.072.762
210893_at	9.71e-05	5.53e-05	-4.071.375
200838_at	9.74e-05	5.54e-05	-4.070.755
214599_at	9.76e-05	5.56e-05	-4.070.014
213644_at	9.80e-05	5.58e-05	4.069.171
220325_at	9.80e-05	5.58e-05	-4.069.025
205827_at	9.81e-05	5.59e-05	-4.068.927
201312_s_at	9.84e-05	5.61e-05	-4.068.082
AFFX-ThrX-3_at	9.86e-05	5.62e-05	-4.067.566
202814_s_at	9.87e-05	5.62e-05	-4.067.247
216608_at	9.89e-05	5.63e-05	-4.066.906
203510_at	9.89e-05	5.63e-05	4.066.902
222325_at	9.89e-05	5.63e-05	4.066.852
214171_s_at	9.89e-05	5.64e-05	-406.672
217314_at	9.92e-05	5.65e-05	-4.066.014
211059_s_at	9.94e-05	5.66e-05	-4.065.554
208087_s_at	9.95e-05	5.67e-05	-4.065.279
207712_at	9.96e-05	5.68e-05	4.064.857
212809_at	9.97e-05	5.69e-05	406.461
216064_s_at	1.00e-04	5.71e-05	406.378
208997_s_at	1.00e-04	5.72e-05	-4.063.369
214514_at	1.01e-04	5.74e-05	-406.252
208969_at	1.01e-04	5.76e-05	4.061.608
218150_at	1.01e-04	5.76e-05	406.137
221334_s_at	1.01e-04	5.77e-05	-406.127
205095_s_at	1.01e-04	5.77e-05	4.061.216
218540_at	1.01e-04	5.77e-05	4.061.158
210199_at	1.01e-04	5.77e-05	-4.061.027
215400_x_at	1.01e-04	5.77e-05	-4.060.929
203352_at	1.01e-04	5.78e-05	4.060.826
203485_at	1.02e-04	5.81e-05	4.059.375
203259_s_at	1.02e-04	5.81e-05	4.059.341
221107_at	1.02e-04	5.82e-05	-4.059.145
217782_s_at	1.02e-04	5.85e-05	4.057.895
215144_at	1.03e-04	5.87e-05	-4.056.921
205873_at	1.03e-04	5.89e-05	-4.056.125
209761_s_at	1.03e-04	5.90e-05	-4.055.697
208201_at	1.04e-04	5.93e-05	-4.054.271

210757_x_at	1.04e-04	5.96e-05	4.053.279
200974_at	1.05e-04	5.97e-05	4.052.734
216635_at	1.05e-04	6.01e-05	-4.051.059
211907_s_at	1.05e-04	6.02e-05	-4.050.641
213235_at	1.05e-04	6.02e-05	4.050.621
216433_s_at	1.06e-04	6.04e-05	-4.050.178
209097_s_at	1.06e-04	6.05e-05	-4.049.534
209886_s_at	1.06e-04	6.05e-05	-4.049.493
205676_at	1.06e-04	6.06e-05	4.049.254
218166_s_at	1.06e-04	6.06e-05	4.049.156
215529_x_at	1.06e-04	6.06e-05	-4.049.009
216796_s_at	1.07e-04	6.09e-05	-4.048.092
213358_at	1.07e-04	6.09e-05	4.047.923
204801_s_at	1.07e-04	6.09e-05	-4.047.917
218435_at	1.07e-04	6.10e-05	-4.047.739
218327_s_at	1.07e-04	6.10e-05	4.047.666
215161_at	1.07e-04	6.10e-05	-404.745
205706_s_at	1.07e-04	6.13e-05	4.046.403
218205_s_at	1.07e-04	6.13e-05	-4.046.356
219323_s_at	1.07e-04	6.14e-05	-4.045.884
215270_at	1.08e-04	6.18e-05	-4.044.545
208859_s_at	1.08e-04	6.18e-05	4.044.338
217895_at	1.08e-04	6.18e-05	4.044.235
211429_s_at	1.08e-04	6.19e-05	-4.044.154
219085_s_at	1.09e-04	6.21e-05	-4.043.253
222284_at	1.09e-04	6.21e-05	-4.043.234
214510_at	1.09e-04	6.22e-05	-4.042.874
220543_at	1.09e-04	6.22e-05	-4.042.784
220130_x_at	1.09e-04	6.22e-05	-4.042.672
201937_s_at	1.09e-04	6.23e-05	4.042.371
206704_at	1.09e-04	6.24e-05	-4.042.207
209032_s_at	1.09e-04	6.24e-05	4.042.046
208804_s_at	1.10e-04	6.28e-05	-4.040.403
201862_s_at	1.10e-04	6.29e-05	-4.040.078
206250_x_at	1.10e-04	6.32e-05	-4.039.062
201187_s_at	1.11e-04	6.34e-05	-4.038.219
212692_s_at	1.11e-04	6.35e-05	-4.037.821
635_s_at	1.11e-04	6.36e-05	4.037.577
205842_s_at	1.11e-04	6.37e-05	-4.037.046
203346_s_at	1.12e-04	6.42e-05	4.035.125
206986_at	1.12e-04	6.42e-05	-4.035.055
209867_s_at	1.12e-04	6.42e-05	-4.035.052
206563_s_at	1.13e-04	6.45e-05	-4.034.019
219943_s_at	1.13e-04	6.50e-05	-4.032.322

216343_at	1.13e-04	6.50e-05	-4.032.121
210457_x_at	1.13e-04	6.50e-05	-4.032.098
204978_at	1.13e-04	6.50e-05	-4.032.067
216510_x_at	1.14e-04	6.52e-05	-4.031.322
209428_s_at	1.14e-04	6.53e-05	4.031.209
201448_at	1.14e-04	6.56e-05	4.029.953
38707_r_at	1.15e-04	6.56e-05	-4.029.756
209364_at	1.15e-04	6.60e-05	4.028.346
201348_at	1.15e-04	6.62e-05	-40.277
207077_at	1.16e-04	6.62e-05	-4.027.556
209501_at	1.16e-04	6.63e-05	4.027.242
208226_x_at	1.16e-04	6.64e-05	-4.026.807
213289_at	1.16e-04	6.64e-05	4.026.774
221186_at	1.16e-04	6.66e-05	-4.026.259
218478_s_at	1.16e-04	6.66e-05	4.026.126
210687_at	1.16e-04	6.67e-05	-4.025.991
207893_at	1.16e-04	6.68e-05	-4.025.596
215961_at	1.17e-04	6.69e-05	-40.251
222222_s_at	1.17e-04	6.69e-05	4.024.992
212992_at	1.17e-04	6.70e-05	4.024.743
215524_x_at	1.17e-04	6.70e-05	-4.024.729
216011_at	1.17e-04	6.70e-05	-4.024.722
59433_at	1.17e-04	6.73e-05	-4.023.793
222135_at	1.17e-04	6.74e-05	-4.023.381
214418_at	1.18e-04	6.77e-05	-4.022.368
210969_at	1.18e-04	6.77e-05	-4.022.255
217967_s_at	1.19e-04	6.81e-05	4.020.966
220339_s_at	1.19e-04	6.82e-05	-4.020.401
203170_at	1.19e-04	6.83e-05	-4.019.942
202012_s_at	1.19e-04	6.84e-05	4.019.791
210921_at	1.19e-04	6.84e-05	-4.019.691
211030_s_at	1.19e-04	6.86e-05	-4.019.091
221544_s_at	1.19e-04	6.86e-05	-4.019.015
214136_at	1.20e-04	6.88e-05	-4.018.337
220687_at	1.20e-04	6.88e-05	-4.018.333
222178_s_at	1.20e-04	6.89e-05	-4.017.966
217934_x_at	1.20e-04	6.91e-05	-4.017.178
208463_at	1.20e-04	6.92e-05	-4.016.985
208060_at	1.20e-04	6.92e-05	-4.016.966
202417_at	1.20e-04	6.92e-05	4.016.728
209423_s_at	1.21e-04	6.93e-05	-401.637
201728_s_at	1.21e-04	6.93e-05	4.016.365
215190_at	1.21e-04	6.95e-05	-4.015.835
221342_at	1.21e-04	6.95e-05	-4.015.725

208344_x_at	1.21e-04	6.95e-05	-4.015.667
204073_s_at	1.21e-04	6.98e-05	4.014.596
211447_s_at	1.22e-04	6.99e-05	-4.014.465
208435_s_at	1.22e-04	7.00e-05	-4.014.187
210077_s_at	1.22e-04	7.01e-05	-4.013.874
212112_s_at	1.22e-04	7.02e-05	4.013.307
211453_s_at	1.22e-04	7.03e-05	-4.013.169
206048_at	1.23e-04	7.05e-05	-4.012.155
204504_s_at	1.23e-04	7.07e-05	4.011.582
215050_x_at	1.23e-04	7.08e-05	4.011.253
208044_s_at	1.23e-04	7.09e-05	4.010.961
214985_at	1.23e-04	7.09e-05	4.010.782
212258_s_at	1.23e-04	7.10e-05	-4.010.622
221043_at	1.23e-04	7.11e-05	-4.010.408
215601_at	1.24e-04	7.11e-05	-4.010.166
219992_at	1.24e-04	7.13e-05	-4.009.501
210196_s_at	1.24e-04	7.14e-05	-4.009.379
203498_at	1.24e-04	7.15e-05	-4.008.756
221632_s_at	1.24e-04	7.16e-05	-4.008.651
217250_s_at	1.25e-04	7.18e-05	-4.007.931
220141_at	1.25e-04	7.18e-05	-4.007.833
216355_at	1.25e-04	7.19e-05	-4.007.448
221969_at	1.25e-04	7.20e-05	-4.007.252
203985_at	1.25e-04	7.20e-05	4.007.148
212316_at	1.25e-04	7.20e-05	4.007.063
205139_s_at	1.25e-04	7.20e-05	-400.704
201914_s_at	1.25e-04	7.20e-05	4.007.017
206531_at	1.26e-04	7.25e-05	-4.005.563
203729_at	1.26e-04	7.26e-05	-4.005.158
208017_s_at	1.26e-04	7.27e-05	-4.004.786
210649_s_at	1.26e-04	7.28e-05	400.439
220125_at	1.28e-04	7.39e-05	-400.063
203412_at	1.29e-04	7.42e-05	3.999.839
221383_at	1.29e-04	7.42e-05	-3.999.734
210073_at	1.29e-04	7.43e-05	-3.999.409
220370_s_at	1.29e-04	7.45e-05	3.998.815
219899_x_at	1.29e-04	7.46e-05	-3.998.487
203365_s_at	1.29e-04	7.47e-05	-3.998.272
222383_s_at	1.30e-04	7.49e-05	-399.754
204684_at	1.30e-04	7.49e-05	-3.997.506
217245_at	1.30e-04	7.49e-05	-3.997.444
205030_at	1.30e-04	7.49e-05	-3.997.353
37226_at	1.30e-04	7.50e-05	3.997.144
214646_at	1.30e-04	7.51e-05	-3.996.964

210451_at	1.30e-04	7.51e-05	-3.996.795
220185_at	1.30e-04	7.52e-05	-3.996.679
220744_s_at	1.30e-04	7.53e-05	-3.996.329
217724_at	1.31e-04	7.53e-05	3.996.145
210305_at	1.31e-04	7.55e-05	-3.995.581
216853_x_at	1.31e-04	7.56e-05	-3.995.238
211399_at	1.31e-04	7.58e-05	-3.994.621
51226_at	1.31e-04	7.58e-05	-3.994.601
204936_at	1.31e-04	7.59e-05	-3.994.284
216174_at	1.32e-04	7.60e-05	-3.994.052
220484_at	1.32e-04	7.61e-05	3.993.662
205365_at	1.32e-04	7.62e-05	-3.993.257
221707_s_at	1.32e-04	7.63e-05	-3.993.108
221385_s_at	1.32e-04	7.64e-05	-3.992.788
217513_at	1.32e-04	7.65e-05	-3.992.465
220547_s_at	1.33e-04	7.66e-05	-3.991.871
217983_s_at	1.33e-04	7.66e-05	3.991.851
202932_at	1.33e-04	7.67e-05	3.991.689
215753_at	1.33e-04	7.69e-05	-3.990.987
206117_at	1.33e-04	7.70e-05	399.066
208055_s_at	1.33e-04	7.71e-05	3.990.275
210671_x_at	1.34e-04	7.76e-05	-3.988.687
219691_at	1.36e-04	7.85e-05	-3.985.841
220475_at	1.36e-04	7.86e-05	-3.985.662
218363_at	1.37e-04	7.94e-05	-3.983.306
208481_at	1.37e-04	7.94e-05	-3.983.209
201164_s_at	1.37e-04	7.94e-05	3.983.061
204417_at	1.38e-04	7.96e-05	3.982.617
203782_s_at	1.38e-04	7.97e-05	3.982.182
211107_s_at	1.38e-04	8.00e-05	-3.981.321
202699_s_at	1.38e-04	8.00e-05	-3.981.292
211192_s_at	1.38e-04	8.00e-05	-3.981.251
208412_s_at	1.39e-04	8.02e-05	-3.980.786
221957_at	1.39e-04	8.03e-05	3.980.367
216164_at	1.40e-04	8.08e-05	-3.978.925
207442_at	1.40e-04	8.08e-05	-3.978.837
211702_s_at	1.40e-04	8.12e-05	3.977.694
213533_at	1.41e-04	8.15e-05	-3.976.883
213220_at	1.41e-04	8.15e-05	3.976.856
200012_x_at	1.41e-04	8.19e-05	3.975.659
201163_s_at	1.42e-04	8.19e-05	-3.975.389
211201_at	1.42e-04	8.20e-05	-3.975.325
211604_x_at	1.42e-04	8.23e-05	-3.974.284
216983_s_at	1.42e-04	8.23e-05	-3.974.241

219670_at	1.43e-04	8.25e-05	-3.973.668
216857_at	1.43e-04	8.25e-05	-3.973.602
208222_at	1.43e-04	8.26e-05	-397.355
215591_at	1.43e-04	8.26e-05	3.973.299
214929_s_at	1.43e-04	8.27e-05	-3.973.038
201979_s_at	1.43e-04	8.29e-05	-3.972.642
221321_s_at	1.43e-04	8.29e-05	-3.972.477
215497_s_at	1.44e-04	8.33e-05	-3.971.235
208081_s_at	1.44e-04	8.37e-05	-3.970.296
212831_at	1.45e-04	8.37e-05	-3.970.043
214442_s_at	1.45e-04	8.39e-05	3.969.704
210042_s_at	1.45e-04	8.39e-05	-3.969.556
204064_at	1.46e-04	8.44e-05	3.968.082
221606_s_at	1.47e-04	8.49e-05	-3.966.521
38398_at	1.47e-04	8.50e-05	-3.966.257
212540_at	1.47e-04	8.51e-05	-3.966.084
216668_at	1.47e-04	8.52e-05	-3.965.864
209822_s_at	1.47e-04	8.52e-05	3.965.702
205185_at	1.47e-04	8.55e-05	3.964.919
212451_at	1.48e-04	8.57e-05	-3.964.249
216364_s_at	1.48e-04	8.61e-05	-3.963.296
217060_at	1.49e-04	8.63e-05	-3.962.694
209741_x_at	1.49e-04	8.64e-05	3.962.248
209722_s_at	1.49e-04	8.64e-05	-3.962.193
216744_at	1.49e-04	8.66e-05	-3.961.894
212157_at	1.49e-04	8.67e-05	3.961.481
208849_at	1.50e-04	8.68e-05	-3.961.079
202099_s_at	1.50e-04	8.71e-05	-3.960.418
213187_x_at	1.50e-04	8.71e-05	3.960.244
217921_at	1.50e-04	8.72e-05	3.960.154
211146_at	1.50e-04	8.73e-05	-3.959.701
215872_at	1.51e-04	8.77e-05	-3.958.611
219952_s_at	1.51e-04	8.79e-05	3.958.106
44040_at	1.52e-04	8.80e-05	3.957.804
222042_x_at	1.52e-04	8.80e-05	-3.957.692
216148_at	1.52e-04	8.81e-05	-3.957.485
212679_at	1.52e-04	8.84e-05	-3.956.547
218965_s_at	1.52e-04	8.85e-05	-3.956.525
210906_x_at	1.52e-04	8.85e-05	-3.956.434
206647_at	1.52e-04	8.85e-05	-3.956.289
204803_s_at	1.53e-04	8.87e-05	-3.955.955
208540_x_at	1.53e-04	8.88e-05	3.955.641
219723_x_at	1.53e-04	8.88e-05	-3.955.598
202115_s_at	1.53e-04	8.90e-05	3.955.131

215862_at	1.53e-04	8.90e-05	-3.955.037
210004_at	1.54e-04	8.94e-05	3.954.001
209589_s_at	1.54e-04	8.94e-05	3.953.855
217855_x_at	1.54e-04	8.95e-05	-3.953.667
218303_x_at	1.54e-04	8.96e-05	-3.953.222
207793_s_at	1.54e-04	8.97e-05	-3.953.104
203061_s_at	1.54e-04	8.98e-05	3.952.822
217461_x_at	1.55e-04	8.99e-05	3.952.589
219619_at	1.55e-04	9.01e-05	3.952.051
212583_at	1.55e-04	9.02e-05	3.951.569
209706_at	1.55e-04	9.03e-05	-3.951.462
210332_at	1.56e-04	9.04e-05	-3.951.098
207501_s_at	1.56e-04	9.04e-05	3.951.046
214739_at	1.56e-04	9.06e-05	3.950.636
209094_at	1.56e-04	9.07e-05	-3.950.307
209591_s_at	1.56e-04	9.09e-05	-3.949.654
205808_at	1.57e-04	9.10e-05	3.949.381
219150_s_at	1.57e-04	9.13e-05	-3.948.803
208221_s_at	1.57e-04	9.15e-05	-3.948.074
219402_s_at	1.58e-04	9.17e-05	3.947.526
217961_at	1.58e-04	9.20e-05	-3.946.834
216807_at	1.58e-04	9.20e-05	-3.946.764
210548_at	1.58e-04	9.22e-05	-3.946.343
219844_at	1.59e-04	9.22e-05	-3.946.121
204180_s_at	1.59e-04	9.23e-05	-3.946.028
216765_at	1.59e-04	9.27e-05	-3.945.016
206763_at	1.59e-04	9.28e-05	-3.944.711
212970_at	1.60e-04	9.29e-05	-394.439
206260_at	1.60e-04	9.30e-05	-3.944.131
211597_s_at	1.60e-04	9.31e-05	3.943.934
210993_s_at	1.60e-04	9.32e-05	-3.943.638
208769_at	1.60e-04	9.33e-05	3.943.263
208629_s_at	1.60e-04	9.34e-05	-3.943.092
213382_at	1.60e-04	9.34e-05	-3.943.039
206832_s_at	1.61e-04	9.35e-05	-3.942.667
213271_s_at	1.61e-04	9.36e-05	-3.942.605
222124_at	1.61e-04	9.39e-05	-3.941.748
212882_at	1.62e-04	9.42e-05	3.940.985
215130_s_at	1.62e-04	9.44e-05	-394.043
220286_at	1.62e-04	9.44e-05	-3.940.295
206975_at	1.63e-04	9.48e-05	-3.939.399
210940_s_at	1.63e-04	9.50e-05	-3.938.941
206444_at	1.63e-04	9.50e-05	-3.938.804
201591_s_at	1.63e-04	9.52e-05	-3.938.383

206804_at	1.64e-04	9.54e-05	-3.937.679
218447_at	1.64e-04	9.55e-05	-3.937.435
216663_s_at	1.65e-04	9.64e-05	-393.523
202831_at	1.66e-04	9.68e-05	3.934.276
219894_at	1.66e-04	9.70e-05	-393.357
215030_at	1.66e-04	9.70e-05	3.933.545
206598_at	1.67e-04	9.75e-05	-3.932.458
208597_at	1.67e-04	9.76e-05	-3.932.157
214461_at	1.67e-04	9.77e-05	-3.931.909
200813_s_at	1.67e-04	9.77e-05	-3.931.866
216465_at	1.68e-04	9.79e-05	-3.931.372
216724_at	1.68e-04	9.81e-05	-3.930.902
218863_s_at	1.69e-04	9.85e-05	-3.929.882
222123_s_at	1.69e-04	9.85e-05	-3.929.786
207936_x_at	1.70e-04	9.89e-05	-3.928.791
216838_at	1.70e-04	9.95e-05	-3.927.373
218957_s_at	1.72e-04	1.00e-04	3.925.741
220756_s_at	1.72e-04	1.00e-04	-3.925.587
216203_at	1.72e-04	1.00e-04	-3.925.419
213991_s_at	1.72e-04	1.00e-04	-3.925.223
221633_at	1.72e-04	1.00e-04	-3.924.847
215224_at	1.73e-04	1.01e-04	3.923.307
210795_s_at	1.73e-04	1.01e-04	-392.293
AFFX-r2-Bs-lys-3_at	1.74e-04	1.01e-04	-3.922.566
201911_s_at	1.74e-04	1.01e-04	392.256
206587_at	1.74e-04	1.02e-04	-3.922.304
205743_at	1.74e-04	1.02e-04	-3.921.665
221703_at	1.74e-04	1.02e-04	3.921.586
216125_s_at	1.74e-04	1.02e-04	3.921.415
215264_at	1.75e-04	1.02e-04	-3.921
204135_at	1.75e-04	1.02e-04	-3.920.076
214534_at	1.76e-04	1.03e-04	-3.919.588
221348_at	1.76e-04	1.03e-04	-3.919.493
205880_at	1.77e-04	1.03e-04	3.918.192
219217_at	1.77e-04	1.04e-04	3.917.326
32540_at	1.77e-04	1.04e-04	-3.917.019
206848_at	1.77e-04	1.04e-04	3.916.911
204742_s_at	1.78e-04	1.04e-04	3.916.146
222126_at	1.78e-04	1.04e-04	3.915.989
202428_x_at	1.78e-04	1.04e-04	3.915.858
220452_x_at	1.79e-04	1.04e-04	-3.915.183
208569_at	1.79e-04	1.05e-04	-3.915.035
221115_s_at	1.79e-04	1.05e-04	-3.915.022
217781_s_at	1.79e-04	1.05e-04	3.915.015

221460_at	1.79e-04	1.05e-04	-3.914.917
203144_s_at	1.81e-04	1.06e-04	-3.912.215
221277_s_at	1.81e-04	1.06e-04	-391.189
221764_at	1.81e-04	1.06e-04	3.911.554
205887_x_at	1.82e-04	1.06e-04	-391.088
204176_at	1.82e-04	1.06e-04	391.052
212638_s_at	1.83e-04	1.07e-04	3.909.671
220300_at	1.83e-04	1.07e-04	390.942
205963_s_at	1.83e-04	1.07e-04	-3.909.373
202764_at	1.83e-04	1.07e-04	3.909.232
207596_at	1.83e-04	1.07e-04	-390.883
212210_at	1.83e-04	1.07e-04	-3.908.559
217108_at	1.84e-04	1.08e-04	-3.908.014
204683_at	1.84e-04	1.08e-04	3.907.536
216512_s_at	1.84e-04	1.08e-04	-3.907.184
204005_s_at	1.84e-04	1.08e-04	3.907.182
204152_s_at	1.84e-04	1.08e-04	-3.907.156
203788_s_at	1.84e-04	1.08e-04	-3.906.997
202249_s_at	1.85e-04	1.08e-04	-3.906.515
209017_s_at	1.85e-04	1.08e-04	3.905.891
210034_s_at	1.85e-04	1.09e-04	3.905.587
204758_s_at	1.85e-04	1.09e-04	-3.905.414
209920_at	1.86e-04	1.09e-04	3.904.916
212484_at	1.86e-04	1.09e-04	3.904.629
205314_x_at	1.86e-04	1.09e-04	-3.904.596
206447_at	1.86e-04	1.09e-04	-3.904.089
210184_at	1.86e-04	1.09e-04	-3.903.888
204218_at	1.88e-04	1.10e-04	3.902.206
201474_s_at	1.89e-04	1.11e-04	-3.900.802
201891_s_at	1.89e-04	1.11e-04	-3.900.522
203384_s_at	1.89e-04	1.11e-04	3.900.486
210823_s_at	1.89e-04	1.11e-04	-3.900.222
215843_s_at	1.90e-04	1.11e-04	-3.899.272
217145_at	1.90e-04	1.11e-04	-3.898.939
220427_at	1.90e-04	1.12e-04	-3.898.775
218779_x_at	1.90e-04	1.12e-04	-3.898.599
206728_at	1.91e-04	1.12e-04	-3.898.336
207888_at	1.91e-04	1.12e-04	-3.898.052
202881_x_at	1.92e-04	1.12e-04	-389.672
215133_s_at	1.92e-04	1.13e-04	-3.896.552
207619_at	1.92e-04	1.13e-04	-3.895.747
210961_s_at	1.93e-04	1.13e-04	-3.895.063
208751_at	1.93e-04	1.14e-04	-3.894.362
205794_s_at	1.94e-04	1.14e-04	3.894.239

214716_at	1.94e-04	1.14e-04	-3.893.905
210885_s_at	1.94e-04	1.14e-04	-3.893.464
213909_at	1.95e-04	1.14e-04	-3.892.683
204529_s_at	1.95e-04	1.15e-04	3.892.099
202226_s_at	1.95e-04	1.15e-04	-389.178
213046_at	1.95e-04	1.15e-04	-3.891.736
220586_at	1.97e-04	1.16e-04	-3.889.793
217818_s_at	1.97e-04	1.16e-04	3.889.445
214884_at	1.97e-04	1.16e-04	-3.889.381
217536_x_at	1.98e-04	1.17e-04	-3.887.839
210475_at	1.99e-04	1.17e-04	-3.887.646
221946_at	1.99e-04	1.17e-04	-3.887.366
213026_at	1.99e-04	1.17e-04	3.887.356
205623_at	1.99e-04	1.17e-04	3.887.109
205574_x_at	1.99e-04	1.17e-04	-3.886.573
217055_x_at	2.00e-04	1.17e-04	-388.614
220811_at	2.00e-04	1.18e-04	-3.885.177
208277_at	2.01e-04	1.18e-04	-3.884.147
203891_s_at	2.01e-04	1.18e-04	3.883.932
213585_s_at	2.02e-04	1.19e-04	-388.348
220891_at	2.02e-04	1.19e-04	-3.883.003
206855_s_at	2.02e-04	1.19e-04	3.882.806
220733_at	2.04e-04	1.20e-04	-3.880.982
213368_x_at	2.05e-04	1.20e-04	3.879.965
217694_at	2.05e-04	1.20e-04	-3.879.653
202440_s_at	2.06e-04	1.21e-04	3.878.411
218180_s_at	2.06e-04	1.21e-04	-3.877.733
218099_at	2.07e-04	1.22e-04	3.876.904
213019_at	2.07e-04	1.22e-04	-3.876.407
221639_x_at	2.08e-04	1.23e-04	-387.522
215797_at	2.08e-04	1.23e-04	-38.752
217689_at	2.08e-04	1.23e-04	-3.875.142
213236_at	2.09e-04	1.23e-04	-3.874.503
201368_at	2.09e-04	1.23e-04	3.874.333
221264_s_at	2.10e-04	1.23e-04	3.873.357
208448_x_at	2.11e-04	1.24e-04	-3.872.072
212620_at	2.11e-04	1.24e-04	-3.871.846
222334_at	2.11e-04	1.24e-04	3.871.805
217638_at	2.12e-04	1.25e-04	-3.871.008
206456_at	2.12e-04	1.25e-04	-3.870.933
210384_at	2.12e-04	1.25e-04	-3.870.175
206460_at	2.13e-04	1.25e-04	-3.869.923
208204_s_at	2.13e-04	1.25e-04	-3.869.549
221157_s_at	2.13e-04	1.26e-04	-3.868.747

208149_x_at	2.14e-04	1.26e-04	3.868.655
214169_at	2.14e-04	1.26e-04	386.841
207444_at	2.14e-04	1.26e-04	-3.867.874
219136_s_at	2.15e-04	1.27e-04	-386.705
213547_at	2.15e-04	1.27e-04	-3.866.954
219213_at	2.15e-04	1.27e-04	-3.866.683
209477_at	2.16e-04	1.27e-04	3.866.019
201047_x_at	2.16e-04	1.27e-04	3.865.311
213620_s_at	2.17e-04	1.28e-04	3.864.766
210325_at	2.17e-04	1.28e-04	-386.476
221303_at	2.17e-04	1.28e-04	-3.864.632
203713_s_at	2.17e-04	1.28e-04	3.864.583
210132_at	2.17e-04	1.28e-04	3.863.981
203514_at	2.18e-04	1.28e-04	-3.863.548
211238_at	2.18e-04	1.29e-04	-3.863.189
211961_s_at	2.19e-04	1.29e-04	3.862.579
216160_at	2.19e-04	1.29e-04	-3.862.387
214868_at	2.19e-04	1.29e-04	-3.862.054
218359_at	2.19e-04	1.29e-04	3.861.896
214714_at	2.19e-04	1.29e-04	3.861.808
205982_x_at	2.19e-04	1.29e-04	-3.861.716
216107_at	2.20e-04	1.30e-04	-3.861.203
214206_at	2.20e-04	1.30e-04	-3.860.499
213556_at	2.20e-04	1.30e-04	-3.860.243
201975_at	2.21e-04	1.30e-04	3.859.905
206372_at	2.21e-04	1.30e-04	-385.942
222107_x_at	2.21e-04	1.30e-04	-3.859.348
218471_s_at	2.22e-04	1.31e-04	-3.858.851
221808_at	2.23e-04	1.32e-04	3.857.217
202055_at	2.23e-04	1.32e-04	385.703
40560_at	2.24e-04	1.32e-04	-3.856.465
214106_s_at	2.24e-04	1.32e-04	3.855.883
214517_at	2.24e-04	1.32e-04	-3.855.738
210953_at	2.25e-04	1.33e-04	-3.855.107
213284_at	2.25e-04	1.33e-04	-3.854.827
211642_at	2.25e-04	1.33e-04	-3.854.647
217909_s_at	2.26e-04	1.33e-04	385.387
211798_x_at	2.27e-04	1.34e-04	-3.853.071
AFFX-PheX-5_at	2.27e-04	1.34e-04	-3.852.405
219082_at	2.28e-04	1.35e-04	3.851.344
217357_at	2.28e-04	1.35e-04	-3.851.293
217908_s_at	2.28e-04	1.35e-04	3.851.264
212870_at	2.29e-04	1.35e-04	3.850.576
220769_s_at	2.29e-04	1.35e-04	-3.850.023

211631_x_at	2.29e-04	1.36e-04	-3.849.703
203018_s_at	2.29e-04	1.36e-04	3.849.662
221087_s_at	2.30e-04	1.36e-04	-384.851
201104_x_at	2.31e-04	1.37e-04	-3.847.683
218321_x_at	2.31e-04	1.37e-04	3.847.528
203707_at	2.32e-04	1.37e-04	3.847.237
204157_s_at	2.32e-04	1.37e-04	-3.847.153
207926_at	2.32e-04	1.37e-04	-3.846.999
207562_at	2.32e-04	1.37e-04	-3.846.962
214689_at	2.33e-04	1.37e-04	-3.846.102
213230_at	2.33e-04	1.38e-04	-3.845.973
204958_at	2.33e-04	1.38e-04	-3.845.945
218012_at	2.33e-04	1.38e-04	-3.845.743
201646_at	2.33e-04	1.38e-04	-3.845.572
212343_at	2.33e-04	1.38e-04	3.845.315
212598_at	2.34e-04	1.38e-04	-3.844.304
222353_at	2.35e-04	1.39e-04	-3.843.498
221408_x_at	2.35e-04	1.39e-04	-3.843.124
207068_at	2.35e-04	1.39e-04	3.842.896
218338_at	2.36e-04	1.40e-04	-3.842.334
210848_at	2.37e-04	1.40e-04	-3.841.228
208187_s_at	2.37e-04	1.40e-04	-3.840.624
206189_at	2.38e-04	1.41e-04	-3.839.989
204200_s_at	2.38e-04	1.41e-04	3.839.787
205699_at	2.38e-04	1.41e-04	-3.839.609
205362_s_at	2.38e-04	1.41e-04	3.839.561
214208_at	2.39e-04	1.41e-04	-3.839.108
209168_at	2.39e-04	1.41e-04	-3.838.876
217610_at	2.40e-04	1.42e-04	3.838.127
203303_at	2.40e-04	1.42e-04	3.837.767
202930_s_at	2.40e-04	1.42e-04	3.837.738
201265_at	2.40e-04	1.42e-04	-3.837.594
204242_s_at	2.40e-04	1.42e-04	-3.837.549
206194_at	2.40e-04	1.42e-04	-3.837.421
219244_s_at	2.40e-04	1.42e-04	-3.837.299
219310_at	2.40e-04	1.42e-04	-3.837.222
217668_at	2.41e-04	1.43e-04	-3.836.373
211559_s_at	2.41e-04	1.43e-04	3.836.245
209715_at	2.41e-04	1.43e-04	3.836.093
203099_s_at	2.42e-04	1.43e-04	3.835.925
207953_at	2.42e-04	1.43e-04	-3.835.588
217578_at	2.42e-04	1.43e-04	-3.835.345
217989_at	2.43e-04	1.44e-04	-3.834.548
216814_at	2.43e-04	1.44e-04	-3.834.428

221438_s_at	2.44e-04	1.45e-04	-3.833.292
210157_at	2.44e-04	1.45e-04	3.833.094
211445_x_at	2.44e-04	1.45e-04	3.833.016
220630_s_at	2.44e-04	1.45e-04	-3.832.912
160020_at	2.44e-04	1.45e-04	-3.832.795
37020_at	2.45e-04	1.45e-04	-3.832.535
208515_at	2.45e-04	1.45e-04	-3.832.203
207567_at	2.46e-04	1.46e-04	-3.831.292
204217_s_at	2.46e-04	1.46e-04	3.830.909
214625_s_at	2.46e-04	1.46e-04	-3.830.678
205241_at	2.47e-04	1.46e-04	3.830.366
222300_at	2.47e-04	1.46e-04	-383.028
220545_s_at	2.49e-04	1.48e-04	-3.827.843
208082_x_at	2.50e-04	1.48e-04	-3.827.264
202841_x_at	2.50e-04	1.48e-04	-3.827.156
216622_at	2.50e-04	1.48e-04	-3.827.041
208117_s_at	2.50e-04	1.49e-04	3.826.277
203718_at	2.51e-04	1.49e-04	-382.617
200727_s_at	2.51e-04	1.49e-04	3.825.817
221557_s_at	2.51e-04	1.49e-04	-3.825.317
219167_at	2.51e-04	1.49e-04	3.825.239
206519_x_at	2.52e-04	1.49e-04	-3.824.876
219317_at	2.52e-04	1.50e-04	3.824.475
221138_s_at	2.53e-04	1.50e-04	-3.823.823
204164_at	2.53e-04	1.50e-04	-3.823.155
216334_s_at	2.54e-04	1.51e-04	-3.822.328
210838_s_at	2.55e-04	1.51e-04	-3.821.931
215040_at	2.55e-04	1.52e-04	-3.821.419
216855_s_at	2.55e-04	1.52e-04	382.121
220608_s_at	2.55e-04	1.52e-04	-3.821.039
207415_at	2.57e-04	1.53e-04	-3.819.722
209361_s_at	2.58e-04	1.53e-04	3.818.858
216454_at	2.58e-04	1.53e-04	-3.818.373
213587_s_at	2.58e-04	1.53e-04	-3.818.357
201950_x_at	2.58e-04	1.53e-04	3.818.334
219468_s_at	2.58e-04	1.53e-04	3.818.111
205950_s_at	2.59e-04	1.54e-04	-3.817.536
207853_s_at	2.59e-04	1.54e-04	-3.817.341
207419_s_at	2.59e-04	1.54e-04	-3.817.316
217614_at	2.59e-04	1.54e-04	-3.817.033
205989_s_at	2.60e-04	1.54e-04	-3.816.735
207733_x_at	2.60e-04	1.54e-04	-3.816.703
216007_at	2.61e-04	1.55e-04	-3.815.393
206570_s_at	2.61e-04	1.55e-04	-3.815.234

205785_at	2.61e-04	1.55e-04	-3.815.067
212478_at	2.62e-04	1.56e-04	3.814.714
213508_at	2.62e-04	1.56e-04	3.814.632
202662_s_at	2.62e-04	1.56e-04	-381.451
212573_at	2.62e-04	1.56e-04	-3.814.009
211111_at	2.62e-04	1.56e-04	-3.814.003
219202_at	2.64e-04	1.57e-04	3.812.407
221722_x_at	2.64e-04	1.57e-04	-3.812.402
206368_at	2.65e-04	1.57e-04	-3.811.679
209099_x_at	2.65e-04	1.58e-04	3.811.228
207833_s_at	2.65e-04	1.58e-04	-3.810.997
212823_s_at	2.66e-04	1.58e-04	-3.810.341
203304_at	2.67e-04	1.59e-04	3.809.557
220757_s_at	2.67e-04	1.59e-04	-3.809.514
204764_at	2.67e-04	1.59e-04	3.809.514
205177_at	2.68e-04	1.59e-04	-3.808.357
203994_s_at	2.68e-04	1.59e-04	-3.808.335
204142_at	2.68e-04	1.60e-04	-3.807.827
211876_x_at	2.69e-04	1.60e-04	-3.807.666
203021_at	2.69e-04	1.60e-04	-3.807.415
206981_at	2.70e-04	1.61e-04	-3.806.531
211549_s_at	2.70e-04	1.61e-04	-3.806.005
208268_at	2.71e-04	1.62e-04	-3.805.123
201719_s_at	2.71e-04	1.62e-04	380.498
201653_at	2.72e-04	1.62e-04	3.804.339
209651_at	2.72e-04	1.62e-04	-3.804.168
217166_at	2.72e-04	1.62e-04	3.803.889
206003_at	2.72e-04	1.62e-04	3.803.874
206861_s_at	2.73e-04	1.63e-04	3.803.541
208992_s_at	2.73e-04	1.63e-04	-3.803.318
211305_x_at	2.73e-04	1.63e-04	-3.803.116
210482_x_at	2.73e-04	1.63e-04	3.802.897
213416_at	2.74e-04	1.63e-04	-3.802.206
216829_at	2.75e-04	1.64e-04	-3.801.488
210407_at	2.75e-04	1.64e-04	-3.801.297
202071_at	2.75e-04	1.64e-04	-3.801.169
215899_at	2.76e-04	1.64e-04	-3.800.466
211116_at	2.77e-04	1.65e-04	-3.799.785
221940_at	2.78e-04	1.66e-04	3.798.319
205375_at	2.79e-04	1.66e-04	-3.797.506
204964_s_at	2.79e-04	1.67e-04	-3.797.166
218825_at	2.80e-04	1.67e-04	-3.796.911
AFFX-r2-Bs-thr-3_s_at	2.80e-04	1.67e-04	-3.796.863
208988_at	2.80e-04	1.67e-04	3.796.849

214786_at	2.80e-04	1.67e-04	-3.796.811
200871_s_at	2.80e-04	1.67e-04	3.796.778
206603_at	2.80e-04	1.67e-04	-3.796.731
219486_at	2.80e-04	1.67e-04	-3.796.554
36019_at	2.80e-04	1.67e-04	3.796.455
205238_at	2.80e-04	1.67e-04	3.796.182
209905_at	2.81e-04	1.68e-04	-3.795.256
212957_s_at	2.81e-04	1.68e-04	379.513
210973_s_at	2.82e-04	1.68e-04	-3.794.637
207091_at	2.83e-04	1.69e-04	3.793.883
207272_at	2.83e-04	1.69e-04	-3.793.484
218195_at	2.84e-04	1.70e-04	3.792.745
207933_at	2.85e-04	1.70e-04	-3.792.075
217119_s_at	2.85e-04	1.70e-04	-3.791.378
215869_at	2.85e-04	1.70e-04	-3.791.309
215454_x_at	2.86e-04	1.71e-04	-3.791.009
202585_s_at	2.88e-04	1.72e-04	-3.789.159
209182_s_at	2.88e-04	1.72e-04	3.789.105
210359_at	2.88e-04	1.72e-04	-3.789.039
204380_s_at	2.88e-04	1.72e-04	-3.788.918
206812_at	2.88e-04	1.72e-04	-3.788.803
200779_at	2.89e-04	1.73e-04	3.788.019
211507_s_at	2.89e-04	1.73e-04	-3.787.957
207026_s_at	2.89e-04	1.73e-04	-3.787.588
217244_at	2.90e-04	1.73e-04	-3.787.049
208583_x_at	2.90e-04	1.73e-04	3.786.953
210547_x_at	2.90e-04	1.73e-04	3.786.829
219153_s_at	2.90e-04	1.73e-04	-3.786.828
203447_at	2.91e-04	1.74e-04	3.785.817
221091_at	2.91e-04	1.74e-04	-3.785.645
204452_s_at	2.92e-04	1.75e-04	-3.784.893
216043_x_at	2.93e-04	1.75e-04	-3.784.072
208006_at	2.93e-04	1.75e-04	-3.784.033
214178_s_at	2.93e-04	1.75e-04	-3.784.024
211916_s_at	2.93e-04	1.75e-04	3.783.915
220971_at	2.94e-04	1.76e-04	-3.783.304
214749_s_at	2.95e-04	1.76e-04	-3.782.767
202755_s_at	2.95e-04	1.77e-04	-3.782.264
212292_at	2.95e-04	1.77e-04	-3.782.176
215369_at	2.96e-04	1.77e-04	-3.781.254
209504_s_at	2.96e-04	1.77e-04	3.781.198
212107_s_at	2.96e-04	1.77e-04	378.117
202807_s_at	2.97e-04	1.78e-04	3.780.929
212175_s_at	2.97e-04	1.78e-04	-3.780.525

206251_s_at	2.98e-04	1.78e-04	-3.779.751
219403_s_at	2.99e-04	1.79e-04	3.779.063
208472_at	2.99e-04	1.79e-04	-3.778.456
214302_x_at	2.99e-04	1.79e-04	-3.778.431
210083_at	2.99e-04	1.79e-04	-3.778.339
213542_at	3.00e-04	1.80e-04	3.777.886
206843_at	3.01e-04	1.80e-04	-3.777.008
206474_at	3.02e-04	1.81e-04	3.776.142
211692_s_at	3.03e-04	1.82e-04	-377.506
215817_at	3.04e-04	1.82e-04	-3.774.489
219171_s_at	3.07e-04	1.84e-04	3.772.072
205182_s_at	3.07e-04	1.84e-04	3.771.364
213907_at	3.08e-04	1.84e-04	377.114
208479_at	3.08e-04	1.85e-04	-3.770.677
213817_at	3.09e-04	1.85e-04	-3.769.858
207061_at	3.10e-04	1.86e-04	-3.769.377
213407_at	3.10e-04	1.86e-04	3.769.235
AFFX-r2-Bs-phe-5_at	3.11e-04	1.86e-04	-376.822
219463_at	3.11e-04	1.87e-04	-3.768.163
1007_s_at	3.11e-04	1.87e-04	376.815
206569_at	3.12e-04	1.87e-04	-3.767.681
202140_s_at	3.12e-04	1.87e-04	-3.767.656
220745_at	3.12e-04	1.87e-04	-3.767.483
204535_s_at	3.13e-04	1.88e-04	-3.766.632
221295_at	3.14e-04	1.89e-04	-3.765.402
213835_x_at	3.15e-04	1.89e-04	3.764.596
210981_s_at	3.15e-04	1.89e-04	3.764.583
218661_at	3.16e-04	1.90e-04	376.381
215619_at	3.16e-04	1.90e-04	-3.763.575
207075_at	3.17e-04	1.90e-04	-3.763.037
207360_s_at	3.18e-04	1.91e-04	-3.761.996
215292_s_at	3.19e-04	1.91e-04	-3.761.555
206813_at	3.19e-04	1.92e-04	-3.761.196
210050_at	3.19e-04	1.92e-04	3.761.123
205681_at	3.20e-04	1.92e-04	376.098
216458_at	3.20e-04	1.92e-04	-3.760.206
209995_s_at	3.21e-04	1.92e-04	-3.760.053
211184_s_at	3.21e-04	1.93e-04	3.760.037
203785_s_at	3.21e-04	1.93e-04	3.759.588
210642_at	3.21e-04	1.93e-04	-3.759.503
219194_at	3.21e-04	1.93e-04	-3.759.336
201278_at	3.22e-04	1.94e-04	3.758.691
221672_s_at	3.22e-04	1.94e-04	-3.758.459
215651_at	3.23e-04	1.94e-04	-3.757.775

214468_at	3.23e-04	1.94e-04	-3.757.764
52837_at	3.24e-04	1.94e-04	-3.757.446
207845_s_at	3.27e-04	1.97e-04	3.754.724
206732_at	3.28e-04	1.97e-04	-3.754.085
222157_s_at	3.28e-04	1.97e-04	3.754
221822_at	3.29e-04	1.97e-04	-3.753.426
219924_s_at	3.29e-04	1.98e-04	-3.753.045
219309_at	3.30e-04	1.98e-04	-3.752.133
214091_s_at	3.32e-04	1.99e-04	-3.751.076
202253_s_at	3.32e-04	2.00e-04	3.750.376
207051_at	3.33e-04	2.00e-04	-3.750.022
207899_at	3.33e-04	2.00e-04	-3.749.943
208154_at	3.33e-04	2.00e-04	-374.965
206860_s_at	3.34e-04	2.01e-04	3.749.432
211176_s_at	3.34e-04	2.01e-04	-3.749.207
220163_s_at	3.35e-04	2.02e-04	-3.748.202
213747_at	3.36e-04	2.02e-04	-3.747.471
207966_s_at	3.37e-04	2.02e-04	-374.696
215326_at	3.37e-04	2.03e-04	-3.746.914
221422_s_at	3.38e-04	2.03e-04	-374.599
207825_s_at	3.38e-04	2.03e-04	3.745.976
216617_s_at	3.38e-04	2.04e-04	-3.745.498
219392_x_at	3.39e-04	2.04e-04	-3.745.109
207841_at	3.40e-04	2.05e-04	-3.744.297
202229_s_at	3.40e-04	2.05e-04	-374.401
216082_at	3.40e-04	2.05e-04	-374.396
215137_at	3.41e-04	2.05e-04	-3.743.244
215679_at	3.43e-04	2.06e-04	-3.742.312
219846_at	3.43e-04	2.07e-04	3.741.703
216366_x_at	3.43e-04	2.07e-04	-3.741.534
212065_s_at	3.44e-04	2.07e-04	3.741.241
209855_s_at	3.45e-04	2.07e-04	-3.740.713
204075_s_at	3.45e-04	2.08e-04	-3.740.356
207331_at	3.45e-04	2.08e-04	3.740.203
204987_at	3.48e-04	2.10e-04	-3.738.045
202388_at	3.48e-04	2.10e-04	-3.738.021
212834_at	3.48e-04	2.10e-04	3.738
206985_at	3.48e-04	2.10e-04	-3.737.695
216606_x_at	3.48e-04	2.10e-04	373.763
218430_s_at	3.49e-04	2.10e-04	3.737.215
209955_s_at	3.50e-04	2.11e-04	3.736.414
204174_at	3.51e-04	2.12e-04	-3.735.675
215769_at	3.51e-04	2.12e-04	-3.735.429
214391_x_at	3.53e-04	2.13e-04	-37.342

215562_at	3.53e-04	2.13e-04	-3.734.156
216740_at	3.53e-04	2.13e-04	-3.734.093
63305_at	3.53e-04	2.13e-04	-373.404
213782_s_at	3.54e-04	2.13e-04	-3.733.697
208320_at	3.54e-04	2.13e-04	-3.733.464
217380_s_at	3.55e-04	2.14e-04	-3.732.677
201288_at	3.55e-04	2.14e-04	-373.237
207137_at	3.55e-04	2.14e-04	-3.732.263
202070_s_at	3.56e-04	2.14e-04	373.209
200009_at	3.57e-04	2.15e-04	3.731.337
201902_s_at	3.58e-04	2.16e-04	3.730.625
91952_at	3.58e-04	2.16e-04	373.029
220849_at	3.59e-04	2.16e-04	-3.729.832
219739_at	3.60e-04	2.17e-04	-372.887
211589_at	3.60e-04	2.17e-04	-3.728.674
205576_at	3.61e-04	2.18e-04	-3.728.287
202281_at	3.61e-04	2.18e-04	3.728.046
214476_at	3.62e-04	2.18e-04	-37.276
213212_x_at	3.62e-04	2.18e-04	3.727.352
65585_at	3.62e-04	2.19e-04	372.712
207516_at	3.62e-04	2.19e-04	-3.726.917
202574_s_at	3.63e-04	2.19e-04	-3.726.658
205560_at	3.63e-04	2.19e-04	3.726.197
212999_x_at	3.63e-04	2.19e-04	-3.726.142
214334_x_at	3.65e-04	2.20e-04	3.725.096
215225_s_at	3.66e-04	2.21e-04	-3.724.281
207305_s_at	3.68e-04	2.22e-04	-372.256
36564_at	3.69e-04	2.23e-04	3.721.853
37892_at	3.70e-04	2.23e-04	3.721.694
206269_at	3.70e-04	2.23e-04	-3.721.642
221313_at	3.70e-04	2.23e-04	-3.721.339
210446_at	3.71e-04	2.24e-04	-3.720.483
217158_at	3.72e-04	2.25e-04	-371.961
209517_s_at	3.72e-04	2.25e-04	-3.719.598
220241_at	3.73e-04	2.26e-04	3.718.981
214101_s_at	3.74e-04	2.26e-04	3.718.698
205857_at	3.74e-04	2.26e-04	-3.718.229
208213_s_at	3.75e-04	2.26e-04	-3.717.943
202859_x_at	3.76e-04	2.27e-04	3.716.949
204246_s_at	3.77e-04	2.28e-04	3.716.623
215173_at	3.78e-04	2.28e-04	-3.715.827
208090_s_at	3.78e-04	2.29e-04	-3.715.285
205940_at	3.79e-04	2.29e-04	-371.506
211362_s_at	3.79e-04	2.29e-04	-3.715.004

210894_s_at	3.79e-04	2.29e-04	-3.714.798
206253_at	3.79e-04	2.29e-04	-3.714.461
205702_at	3.80e-04	2.30e-04	3.713.774
216880_at	3.82e-04	2.31e-04	-3.712.816
204653_at	3.83e-04	2.31e-04	3.712.283
221314_at	3.83e-04	2.31e-04	-3.712.265
207494_s_at	3.83e-04	2.32e-04	-3.711.964
216950_s_at	3.84e-04	2.32e-04	3.711.574
213338_at	3.84e-04	2.32e-04	-3.711.332
215045_at	3.84e-04	2.32e-04	-3.711.126
200900_s_at	3.86e-04	2.34e-04	3.709.747
214736_s_at	3.86e-04	2.34e-04	3.709.493
216692_at	3.88e-04	2.35e-04	-3.708.693
222014_x_at	3.88e-04	2.35e-04	-3.708.276
214606_at	3.90e-04	2.36e-04	-3.707.322
207646_s_at	3.90e-04	2.36e-04	-3.706.953
218183_at	3.92e-04	2.37e-04	3.705.992
222193_at	3.93e-04	2.38e-04	3.704.973
215909_x_at	3.94e-04	2.38e-04	-3.704.665
220710_at	3.94e-04	2.39e-04	3.704.185
216909_at	3.94e-04	2.39e-04	-3.704.166
207140_at	3.94e-04	2.39e-04	-3.703.999
213376_at	3.95e-04	2.39e-04	3.703.696
218634_at	3.97e-04	2.40e-04	-3.702.332
211870_s_at	3.99e-04	2.42e-04	-3.700.771
215036_at	3.99e-04	2.42e-04	-370.059
212487_at	4.01e-04	2.43e-04	3.699.339
209671_x_at	4.02e-04	2.43e-04	-3.699.013
201619_at	4.02e-04	2.44e-04	-3.698.854
208437_at	4.02e-04	2.44e-04	-3.698.813
217268_at	4.02e-04	2.44e-04	-3.698.579
206654_s_at	4.04e-04	2.45e-04	-3.697.782
212589_at	4.04e-04	2.45e-04	3.697.634
215762_at	4.04e-04	2.45e-04	-3.697.493
212181_s_at	4.05e-04	2.45e-04	369.705
210709_at	4.05e-04	2.46e-04	-3.696.701
222266_at	4.08e-04	2.47e-04	3.695.015
202378_s_at	4.09e-04	2.48e-04	3.694.137
202996_at	4.10e-04	2.48e-04	-3.693.759
203813_s_at	4.10e-04	2.48e-04	-3.693.717
210091_s_at	4.10e-04	2.49e-04	3.693.471
217870_s_at	4.13e-04	2.50e-04	-3.691.782
221386_at	4.13e-04	2.50e-04	-3.691.769
215273_s_at	4.13e-04	2.50e-04	3.691.744

216435_at	4.13e-04	2.50e-04	-3.691.625
204937_s_at	4.13e-04	2.51e-04	-3.691.455
200073_s_at	4.14e-04	2.51e-04	3.690.636
203589_s_at	4.14e-04	2.51e-04	3.690.532
202975_s_at	4.16e-04	2.52e-04	3.689.486
206899_at	4.16e-04	2.52e-04	-3.689.455
202997_s_at	4.17e-04	2.53e-04	-368.877
220204_s_at	4.17e-04	2.53e-04	-3.688.763
208182_x_at	4.19e-04	2.55e-04	-3.687.284
214962_s_at	4.20e-04	2.55e-04	3.687.038
217494_s_at	4.21e-04	2.56e-04	-3.686.284
205254_x_at	4.21e-04	2.56e-04	-3.686.127
214731_at	4.22e-04	2.56e-04	-3.685.838
32128_at	4.23e-04	2.57e-04	3.684.909
222026_at	4.23e-04	2.57e-04	3.684.779
211165_x_at	4.23e-04	2.57e-04	-3.684.746
212775_at	4.24e-04	2.58e-04	-3.684.281
203301_s_at	4.25e-04	2.58e-04	3.683.726
217442_at	4.26e-04	2.59e-04	-3.683.274
221439_at	4.26e-04	2.59e-04	-368.312
215998_at	4.27e-04	2.59e-04	-3.682.522
206044_s_at	4.27e-04	2.59e-04	3.682.343
220101_x_at	4.27e-04	2.60e-04	-3.682.226
219090_at	4.28e-04	2.60e-04	-368.153
219596_at	4.29e-04	2.60e-04	3.681.294
205461_at	4.29e-04	2.61e-04	-3.680.878
216329_at	4.30e-04	2.61e-04	-3.680.555
203926_x_at	4.32e-04	2.62e-04	-3.679.281
218347_at	4.32e-04	2.63e-04	3.679.098
214823_at	4.32e-04	2.63e-04	3.678.895
222361_at	4.33e-04	2.63e-04	3.678.487
204095_s_at	4.33e-04	2.63e-04	-3.678.423
216662_at	4.33e-04	2.64e-04	-3.678.214
208119_s_at	4.33e-04	2.64e-04	3.678.208
216906_at	4.37e-04	2.66e-04	-3.676.213
207011_s_at	4.37e-04	2.66e-04	3.675.858
203475_at	4.37e-04	2.66e-04	3.675.725
217102_at	4.38e-04	2.67e-04	-367.518
220820_at	4.40e-04	2.67e-04	-3.674.386
222329_x_at	4.40e-04	2.68e-04	-3.674.088
211287_x_at	4.41e-04	2.68e-04	-367.355
220279_at	4.41e-04	2.68e-04	-3.673.371
209360_s_at	4.41e-04	2.69e-04	3.673.259
219365_s_at	4.43e-04	2.70e-04	-3.672.254

216477_at	4.43e-04	2.70e-04	-3.672.134
213805_at	4.44e-04	2.70e-04	-3.671.671
216650_at	4.46e-04	2.71e-04	-3.670.396
216825_s_at	4.47e-04	2.72e-04	-3.670.047
211404_s_at	4.47e-04	2.72e-04	3.669.983
215742_at	4.48e-04	2.73e-04	-3.669.221
206614_at	4.52e-04	2.75e-04	-3.666.942
205796_at	4.53e-04	2.76e-04	3.666.259
211147_s_at	4.53e-04	2.76e-04	-3.666.189
218832_x_at	4.54e-04	2.76e-04	-3.665.775
206485_at	4.57e-04	2.78e-04	-3.663.807
208605_s_at	4.58e-04	2.79e-04	-3.663.442
204243_at	4.58e-04	2.79e-04	3.663.385
205000_at	4.58e-04	2.79e-04	-3.663.224
208239_at	4.59e-04	2.79e-04	-366.283
219705_at	4.59e-04	2.79e-04	-3.662.825
221166_at	4.59e-04	2.80e-04	-3.662.626
205274_at	4.60e-04	2.80e-04	-3.661.854
206379_at	4.60e-04	2.81e-04	-3.661.718
212924_s_at	4.62e-04	2.81e-04	366.092
209732_at	4.62e-04	2.82e-04	-3.660.821
216539_at	4.62e-04	2.82e-04	-3.660.635
215419_at	4.62e-04	2.82e-04	-366.054
206408_at	4.63e-04	2.82e-04	-3.660.351
220301_at	4.63e-04	2.82e-04	3.660.235
205665_at	4.65e-04	2.83e-04	-365.905
207178_s_at	4.67e-04	2.85e-04	3.657.874
216140_at	4.68e-04	2.85e-04	-3.657.298
212731_at	4.68e-04	2.86e-04	-3.656.959
219497_s_at	4.68e-04	2.86e-04	3.656.937
222111_at	4.69e-04	2.86e-04	365.668
217747_s_at	4.70e-04	2.86e-04	365.627
204337_at	4.70e-04	2.87e-04	-3.656.149
205391_x_at	4.71e-04	2.87e-04	-3.655.667
202009_at	4.71e-04	2.87e-04	3.655.619
214317_x_at	4.71e-04	2.87e-04	3.655.342
221144_at	4.71e-04	2.88e-04	-365.519
213181_s_at	4.72e-04	2.88e-04	-3.654.763
217422_s_at	4.72e-04	2.88e-04	-3.654.666
215128_at	4.75e-04	2.90e-04	3.653.297
207493_x_at	4.75e-04	2.90e-04	3.653.164
219845_at	4.75e-04	2.90e-04	-3.652.818
220969_s_at	4.77e-04	2.91e-04	3.652.101
206760_s_at	4.77e-04	2.91e-04	-3.652.038

202993_at	4.78e-04	2.92e-04	-3.651.507
202739_s_at	4.79e-04	2.92e-04	-3.650.972
217845_x_at	4.79e-04	2.93e-04	-3.650.626
208550_x_at	4.80e-04	2.93e-04	-3.650.278
221002_s_at	4.80e-04	2.93e-04	3.650.167
207875_at	4.81e-04	2.94e-04	-3.649.604
222289_at	4.82e-04	2.95e-04	-3.648.725
213731_s_at	4.83e-04	2.95e-04	-3.648.267
218704_at	4.83e-04	2.95e-04	364.822
211596_s_at	4.84e-04	2.95e-04	3.648.027
203199_s_at	4.85e-04	2.97e-04	3.647.041
203479_s_at	4.85e-04	2.97e-04	-3.646.994
203921_at	4.86e-04	2.97e-04	-3.646.931
206222_at	4.87e-04	2.98e-04	-364.612
215288_at	4.87e-04	2.98e-04	-3.645.883
220914_at	4.88e-04	2.98e-04	-3.645.521
204501_at	4.90e-04	3.00e-04	-3.644.377
204711_at	4.91e-04	3.00e-04	-3.644.009
216135_at	4.91e-04	3.00e-04	-3.643.591
214893_x_at	4.92e-04	3.01e-04	-3.643.398
222188_at	4.92e-04	3.01e-04	-3.643.151
209195_s_at	4.93e-04	3.01e-04	3.642.951
219603_s_at	4.93e-04	3.01e-04	3.642.914
203370_s_at	4.94e-04	3.02e-04	3.642.312
206849_at	4.97e-04	3.04e-04	-3.640.558
216313_at	4.97e-04	3.04e-04	-3.640.368
212950_at	4.97e-04	3.04e-04	3.640.362
201462_at	4.98e-04	3.05e-04	363.998
208584_at	4.98e-04	3.05e-04	-3.639.921
211242_x_at	4.98e-04	3.05e-04	-3.639.678
209316_s_at	4.99e-04	3.05e-04	3.639.263
221132_at	4.99e-04	3.05e-04	-3.639.234
202355_s_at	5.00e-04	3.06e-04	3.638.993
207376_at	5.00e-04	3.06e-04	-3.638.969
203315_at	5.00e-04	3.06e-04	3.638.842
207358_x_at	5.01e-04	3.06e-04	-3.638.387
204467_s_at	5.01e-04	3.06e-04	-3.638.346
216976_s_at	5.02e-04	3.07e-04	3.637.648
209802_at	5.02e-04	3.08e-04	-3.637.442
220703_at	5.03e-04	3.08e-04	3.637.084
207235_s_at	5.03e-04	3.08e-04	-3.636.801
215463_at	5.05e-04	3.09e-04	-3.635.868
217419_x_at	5.05e-04	3.09e-04	3.635.787
220958_at	5.06e-04	3.10e-04	-3.635.462

214491_at	5.06e-04	3.10e-04	-3.635.128
222345_at	5.07e-04	3.11e-04	-3.634.769
221599_at	5.08e-04	3.11e-04	3.634.537
208570_at	5.08e-04	3.11e-04	-3.634.296
209274_s_at	5.08e-04	3.11e-04	3.634.113
217062_at	5.09e-04	3.12e-04	-3.633.548
57082_at	5.10e-04	3.12e-04	-3.633.258
217311_at	5.10e-04	3.13e-04	-3.633.162
212795_at	5.10e-04	3.13e-04	3.633.061
37170_at	5.11e-04	3.13e-04	-3.632.831
221668_s_at	5.11e-04	3.13e-04	-3.632.411
214960_at	5.11e-04	3.13e-04	3.632.372
216514_at	5.15e-04	3.16e-04	-3.630.619
202096_s_at	5.17e-04	3.17e-04	3.629.401
212431_at	5.18e-04	3.17e-04	3.629.017
218261_at	5.18e-04	3.18e-04	3.628.909
214594_x_at	5.18e-04	3.18e-04	-3.628.752
208028_s_at	5.20e-04	3.19e-04	-3.628.023
208395_s_at	5.23e-04	3.21e-04	-3.626.174
211397_x_at	5.23e-04	3.21e-04	-3.626.091
214523_at	5.25e-04	3.22e-04	-3.625.121
221220_s_at	5.27e-04	3.23e-04	3.624.223
210618_at	5.28e-04	3.24e-04	-3.623.435
205822_s_at	5.29e-04	3.24e-04	3.623.195
220609_at	5.29e-04	3.25e-04	-3.622.862
215957_at	5.29e-04	3.25e-04	-3.622.831
206663_at	5.31e-04	3.26e-04	-3.622.086
207949_s_at	5.32e-04	3.26e-04	3.621.765
220938_s_at	5.34e-04	3.28e-04	362.028
212286_at	5.36e-04	3.29e-04	3.619.424
208499_s_at	5.36e-04	3.29e-04	3.619.216
205662_at	5.37e-04	3.30e-04	3.618.765
222138_s_at	5.38e-04	3.30e-04	3.618.344
217207_s_at	5.39e-04	3.31e-04	-3.618.085
220463_at	5.43e-04	3.33e-04	-3.616.009
205553_s_at	5.44e-04	3.34e-04	-3.615.643
217793_at	5.44e-04	3.34e-04	-3.615.551
219287_at	5.44e-04	3.34e-04	-3.615.315
214538_x_at	5.46e-04	3.36e-04	-3.614.151
204750_s_at	5.46e-04	3.36e-04	3.614.128
219084_at	5.47e-04	3.36e-04	3.613.617
208294_x_at	5.48e-04	3.36e-04	-3.613.539
206513_at	5.49e-04	3.38e-04	-3.612.685
205977_s_at	5.50e-04	3.38e-04	3.612.228

216735_x_at	5.53e-04	3.40e-04	-3.610.927
213309_at	5.54e-04	3.40e-04	-3.610.521
215325_x_at	5.56e-04	3.42e-04	-3.609.348
202018_s_at	5.57e-04	3.43e-04	-3.608.767
221001_at	5.58e-04	3.43e-04	-360.861
214432_at	5.58e-04	3.43e-04	-3.608.482
212232_at	5.63e-04	3.46e-04	3.606.112
222187_x_at	5.63e-04	3.46e-04	-36.058
219297_at	5.64e-04	3.47e-04	-3.605.635
221600_s_at	5.64e-04	3.47e-04	3.605.254
1438_at	5.65e-04	3.47e-04	-3.605.047
211884_s_at	5.65e-04	3.48e-04	-3.604.877
212206_s_at	5.66e-04	3.48e-04	3.604.474
221317_x_at	5.66e-04	3.48e-04	-3.604.196
214331_at	5.67e-04	3.49e-04	3.604.091
216094_at	5.70e-04	3.51e-04	-3.602.552
217175_at	5.70e-04	3.51e-04	-3.602.504
221852_at	5.70e-04	3.51e-04	-3.602.451
204044_at	5.71e-04	3.51e-04	-3.601.939
209041_s_at	5.72e-04	3.52e-04	-3.601.314
207040_s_at	5.73e-04	3.52e-04	360.117
205800_at	5.73e-04	3.53e-04	-3.600.968
208634_s_at	5.73e-04	3.53e-04	-3.600.833
215530_at	5.75e-04	3.54e-04	-3.600.172
217389_s_at	5.75e-04	3.54e-04	-3.599.768
207084_at	5.78e-04	3.56e-04	-3.598.688
219023_at	5.78e-04	3.56e-04	-3.598.516
203032_s_at	5.78e-04	3.56e-04	3.598.407
215661_at	5.78e-04	3.56e-04	-3.598.367
206439_at	5.79e-04	3.57e-04	-3.597.918
219689_at	5.80e-04	3.57e-04	3.597.597
213652_at	5.81e-04	3.58e-04	3.597.209
214199_at	5.81e-04	3.58e-04	-3.596.892
216129_at	5.82e-04	3.59e-04	-3.596.498
207461_at	5.84e-04	3.60e-04	-3.595.488
219585_at	5.86e-04	3.61e-04	-3.594.883
204982_at	5.86e-04	3.61e-04	-3.594.825
220347_at	5.87e-04	3.62e-04	-3.594.116
218422_s_at	5.88e-04	3.62e-04	3.593.865
216897_s_at	5.88e-04	3.62e-04	-3.593.584
214808_at	5.89e-04	3.63e-04	-3.593.359
207462_at	5.90e-04	3.64e-04	-3.592.756
202287_s_at	5.94e-04	3.66e-04	-3.591.052
215280_s_at	5.94e-04	3.66e-04	3.590.703

205855_at	5.95e-04	3.67e-04	-3.590.543
206484_s_at	5.96e-04	3.68e-04	-3.589.831
213403_at	5.96e-04	3.68e-04	3.589.786
209844_at	5.99e-04	3.69e-04	-3.588.702
218126_at	6.01e-04	3.71e-04	-3.587.703
201964_at	6.01e-04	3.71e-04	3.587.463
208124_s_at	6.02e-04	3.72e-04	-3.586.956
AFFX-r2-Bs-dap-M_at	6.03e-04	3.72e-04	-3.586.782
220537_at	6.03e-04	3.72e-04	-3.586.718
201099_at	6.03e-04	3.72e-04	3.586.603
217635_s_at	6.04e-04	3.73e-04	-3.586.062
212158_at	6.06e-04	3.74e-04	3.585.162
213272_s_at	6.07e-04	3.74e-04	-3.584.885
214081_at	6.07e-04	3.75e-04	3.584.842
206409_at	6.07e-04	3.75e-04	-3.584.739
214609_at	6.08e-04	3.75e-04	-3.584.216
AFFX-r2-Bs-lys-M_at	6.08e-04	3.75e-04	-3.584.148
207882_at	6.10e-04	3.77e-04	-3.583.232
209846_s_at	6.11e-04	3.77e-04	-3.583.025
202526_at	6.13e-04	3.78e-04	-3.582.109
215267_s_at	6.13e-04	3.78e-04	-3.582.077
215458_s_at	6.13e-04	3.79e-04	358.184
211070_x_at	6.14e-04	3.79e-04	3.581.723
215902_at	6.14e-04	3.79e-04	-3.581.656
216236_s_at	6.14e-04	3.79e-04	-3.581.573
204310_s_at	6.15e-04	3.80e-04	3.581.122
221900_at	6.17e-04	3.81e-04	-3.580.177
208123_at	6.18e-04	3.82e-04	-3.579.584
202805_s_at	6.19e-04	3.83e-04	3.579.109
215758_x_at	6.20e-04	3.83e-04	3.578.727
211180_x_at	6.21e-04	3.83e-04	-3.578.468
207741_x_at	6.22e-04	3.84e-04	-3.578.028
217087_at	6.25e-04	3.86e-04	-3.576.778
206562_s_at	6.25e-04	3.86e-04	3.576.716
204481_at	6.26e-04	3.87e-04	3.576.132
211550_at	6.26e-04	3.87e-04	-3.575.954
216006_at	6.33e-04	3.91e-04	-3.573.212
211905_s_at	6.34e-04	3.92e-04	-3.572.508
218656_s_at	6.35e-04	3.93e-04	-3.572.175
217387_at	6.35e-04	3.93e-04	-3.572.031
206588_at	6.36e-04	3.93e-04	-3.571.629
204285_s_at	6.39e-04	3.95e-04	3.570.449
217010_s_at	6.41e-04	3.96e-04	3.569.741
212205_at	6.43e-04	3.98e-04	3.568.731

212656_at	6.45e-04	3.99e-04	356.781
202988_s_at	6.48e-04	4.01e-04	-3.566.777
209190_s_at	6.48e-04	4.01e-04	-3.566.524
204564_at	6.49e-04	4.02e-04	3.565.946
210354_at	6.50e-04	4.02e-04	-3.565.796
36994_at	6.50e-04	4.02e-04	3.565.639
219653_at	6.51e-04	4.03e-04	3.565.055
220038_at	6.53e-04	4.04e-04	3.564.221
214619_at	6.54e-04	4.05e-04	-3.563.835
211821_x_at	6.57e-04	4.07e-04	-3.562.716
208237_x_at	6.58e-04	4.07e-04	-3.562.302
201169_s_at	6.60e-04	4.09e-04	-3.561.375
212755_at	6.60e-04	4.09e-04	-3.561.374
205106_at	6.60e-04	4.09e-04	-356.134
209203_s_at	6.60e-04	4.09e-04	3.561.174
215910_s_at	6.61e-04	4.09e-04	-3.560.999
212516_at	6.62e-04	4.10e-04	-3.560.628
211400_at	6.64e-04	4.11e-04	-3.559.727
219853_at	6.65e-04	4.12e-04	3.559.081
201793_x_at	6.66e-04	4.12e-04	3.559.032
213841_at	6.67e-04	4.13e-04	-3.558.243
210659_at	6.69e-04	4.15e-04	-3.557.474
220697_at	6.70e-04	4.15e-04	-3.557.121
212508_at	6.71e-04	4.16e-04	3.556.835
215646_s_at	6.72e-04	4.16e-04	3.556.372
210258_at	6.74e-04	4.18e-04	3.555.522
208263_at	6.74e-04	4.18e-04	-3.555.269
210843_s_at	6.74e-04	4.18e-04	-3.555.267
201294_s_at	6.75e-04	4.18e-04	-3.555.186
208104_s_at	6.75e-04	4.18e-04	3.554.995
216029_at	6.76e-04	4.19e-04	-3.554.502
216119_s_at	6.78e-04	4.20e-04	-3.553.808
205589_at	6.78e-04	4.21e-04	-3.553.612
210444_at	6.79e-04	4.21e-04	-3.553.493
220578_at	6.79e-04	4.21e-04	-3.553.162
208092_s_at	6.81e-04	4.22e-04	3.552.694
38157_at	6.81e-04	4.22e-04	3.552.591
218060_s_at	6.81e-04	4.22e-04	3.552.478
206658_at	6.81e-04	4.22e-04	-3.552.457
203539_s_at	6.82e-04	4.23e-04	-355.215
205864_at	6.82e-04	4.23e-04	-355.211
217891_at	6.83e-04	4.24e-04	-3.551.516
213147_at	6.83e-04	4.24e-04	3.551.405
221564_at	6.85e-04	4.25e-04	3.550.604

201332_s_at	6.86e-04	4.26e-04	-3.550.175
210227_at	6.88e-04	4.27e-04	-3.549.547
216358_at	6.88e-04	4.27e-04	-3.549.449
213357_at	6.89e-04	4.28e-04	3.549.075
204090_at	6.90e-04	4.28e-04	3.548.877
221338_at	6.90e-04	4.28e-04	-3.548.749
210807_s_at	6.91e-04	4.29e-04	-3.548.436
208560_at	6.91e-04	4.29e-04	-3.548.266
219121_s_at	6.91e-04	4.29e-04	3.548.063
202234_s_at	6.91e-04	4.29e-04	-3.548.022
201873_s_at	6.92e-04	4.30e-04	3.547.877
210107_at	6.92e-04	4.30e-04	-354.765
204136_at	6.94e-04	4.31e-04	3.547.134
204286_s_at	6.94e-04	4.31e-04	3.547.001
210607_at	6.94e-04	4.31e-04	-3.546.922
208008_at	6.95e-04	4.31e-04	-3.546.646
220272_at	6.95e-04	4.32e-04	-3.546.334
217176_s_at	6.96e-04	4.32e-04	3.546.222
216261_at	6.99e-04	4.34e-04	-3.544.844
213269_at	7.01e-04	4.36e-04	3.543.923
209936_at	7.04e-04	4.37e-04	-3.543.043
219922_s_at	7.05e-04	4.38e-04	-3.542.616
206526_at	7.05e-04	4.38e-04	3.542.347
210810_s_at	7.06e-04	4.39e-04	-3.542.072
220988_s_at	7.08e-04	4.40e-04	3.541.274
215407_s_at	7.10e-04	4.41e-04	3.540.496
210919_at	7.10e-04	4.41e-04	-3.540.423
200655_s_at	7.11e-04	4.42e-04	-3.540.064
215645_at	7.12e-04	4.43e-04	-3.539.678
206375_s_at	7.13e-04	4.43e-04	-3.539.397
220368_s_at	7.15e-04	4.45e-04	-3.538.405
214899_at	7.16e-04	4.45e-04	-3.538.164
204718_at	7.16e-04	4.45e-04	-3.537.969
217518_at	7.18e-04	4.46e-04	-3.537.519
204179_at	7.18e-04	4.47e-04	-3.537.188
213728_at	7.19e-04	4.47e-04	3.537.033
222265_at	7.21e-04	4.48e-04	-3.536.212
208643_s_at	7.21e-04	4.48e-04	3.536.163
214495_at	7.21e-04	4.49e-04	-3.535.953
208932_at	7.24e-04	4.50e-04	3.535.056
205651_x_at	7.24e-04	4.50e-04	-3.534.942
220361_at	7.26e-04	4.52e-04	3.534.226
220544_at	7.28e-04	4.53e-04	-3.533.341
221816_s_at	7.28e-04	4.53e-04	-3.533.219

208574_at	7.30e-04	4.54e-04	-3.532.721
220409_at	7.31e-04	4.55e-04	-3.532.102
217050_at	7.32e-04	4.56e-04	-353.191
206267_s_at	7.32e-04	4.56e-04	-3.531.875
206576_s_at	7.35e-04	4.58e-04	-3.530.578
218675_at	7.36e-04	4.58e-04	-3.530.441
216900_s_at	7.36e-04	4.58e-04	-3.530.224
211838_x_at	7.37e-04	4.59e-04	-3.530.047
221026_s_at	7.37e-04	4.59e-04	-3.530.032
208601_s_at	7.37e-04	4.59e-04	-3.529.916
211328_x_at	7.38e-04	4.60e-04	3.529.428
210964_s_at	7.38e-04	4.60e-04	-3.529.409
38043_at	7.42e-04	4.63e-04	3.527.774
220390_at	7.44e-04	4.63e-04	-352.728
216300_x_at	7.44e-04	4.64e-04	3.527.026
213172_at	7.44e-04	4.64e-04	3.527.007
217069_at	7.45e-04	4.64e-04	-3.526.817
214778_at	7.48e-04	4.66e-04	3.525.745
207313_x_at	7.48e-04	4.66e-04	-3.525.643
213120_at	7.48e-04	4.66e-04	-3.525.604
209223_at	7.48e-04	4.66e-04	-3.525.537
208341_x_at	7.50e-04	4.68e-04	-3.524.824
204583_x_at	7.50e-04	4.68e-04	-3.524.811
219107_at	7.54e-04	4.70e-04	-3.523.522
222062_at	7.55e-04	4.71e-04	3.523.069
217181_at	7.55e-04	4.71e-04	-3.523.047
214172_x_at	7.55e-04	4.71e-04	3.522.927
206050_s_at	7.56e-04	4.71e-04	3.522.691
204387_x_at	7.56e-04	4.71e-04	-3.522.644
214344_at	7.57e-04	4.72e-04	-3.521.983
203404_at	7.60e-04	4.74e-04	-3.521.214
214099_s_at	7.61e-04	4.75e-04	-3.520.701
216558_x_at	7.63e-04	4.76e-04	-3.519.883
211691_x_at	7.63e-04	4.76e-04	3.519.762
214287_s_at	7.68e-04	4.79e-04	-3.517.986
211402_x_at	7.69e-04	4.80e-04	-3.517.813
221232_s_at	7.70e-04	4.81e-04	-3.517.381
202317_s_at	7.71e-04	4.81e-04	3.517.066
207118_s_at	7.71e-04	4.81e-04	-3.517.048
212041_at	7.71e-04	4.81e-04	3.517.024
214720_x_at	7.72e-04	4.82e-04	3.516.761
214497_s_at	7.73e-04	4.82e-04	-351.625
219514_at	7.75e-04	4.84e-04	-3.515.623
207522_s_at	7.76e-04	4.84e-04	-3.515.246

200673_at	7.76e-04	4.84e-04	3.515.155
211330_s_at	7.76e-04	4.84e-04	3.515.141
214258_x_at	7.76e-04	4.85e-04	3.514.958
216219_at	7.79e-04	4.86e-04	-351.401
213857_s_at	7.80e-04	4.87e-04	3.513.513
204080_at	7.80e-04	4.88e-04	-3.513.433
203659_s_at	7.81e-04	4.88e-04	-3.513.239
AFFX-r2-P1-cre-5_at	7.83e-04	4.89e-04	-3.512.461
221577_x_at	7.84e-04	4.90e-04	3.512.198
218740_s_at	7.87e-04	4.92e-04	3.511.096
211831_s_at	7.88e-04	4.93e-04	-3.510.582
216689_x_at	7.89e-04	4.93e-04	-3.510.503
215516_at	7.89e-04	4.93e-04	-3.510.468
216637_at	7.91e-04	4.94e-04	-3.509.554
216993_s_at	7.92e-04	4.95e-04	-3.509.083
220909_at	7.93e-04	4.96e-04	-3.508.979
206720_at	7.93e-04	4.96e-04	-3.508.878
204376_at	7.93e-04	4.96e-04	-3.508.864
201341_at	7.93e-04	4.96e-04	-3.508.783
204909_at	7.94e-04	4.96e-04	-3.508.485
218383_at	7.95e-04	4.97e-04	3.508.151
202575_at	7.98e-04	4.99e-04	-3.507.111
219348_at	8.00e-04	5.00e-04	3.506.493
204482_at	8.01e-04	5.01e-04	-3.505.933
205299_s_at	8.03e-04	5.02e-04	3.505.195
203849_s_at	8.05e-04	5.03e-04	-3.504.748
220322_at	8.07e-04	5.05e-04	-3.504.038
208352_x_at	8.07e-04	5.05e-04	-3.503.792
206425_s_at	8.14e-04	5.10e-04	-3.501.337
216167_at	8.16e-04	5.11e-04	-3.500.629
217484_at	8.17e-04	5.11e-04	-3.500.405
37232_at	8.19e-04	5.13e-04	3.499.617
219868_s_at	8.19e-04	5.13e-04	-3.499.584
219955_at	8.20e-04	5.13e-04	-3.499.455
204713_s_at	8.20e-04	5.13e-04	-3.499.432
210269_s_at	8.20e-04	5.13e-04	-3.499.372
217674_at	8.21e-04	5.14e-04	-3.499.006
218254_s_at	8.22e-04	5.15e-04	-3.498.529
213258_at	8.23e-04	5.15e-04	-3.498.391
206537_at	8.24e-04	5.16e-04	-3.497.931
220732_at	8.25e-04	5.17e-04	-3.497.507
219144_at	8.26e-04	5.17e-04	-3.497.371
214060_at	8.26e-04	5.17e-04	3.497.282
205753_at	8.26e-04	5.17e-04	-3.497.149

212979_s_at	8.26e-04	5.18e-04	3.497.081
202056_at	8.27e-04	5.18e-04	3.496.742
40569_at	8.29e-04	5.19e-04	-3.496.223
201299_s_at	8.29e-04	5.20e-04	3.495.947
220963_s_at	8.31e-04	5.21e-04	-3.495.479
217263_x_at	8.32e-04	5.22e-04	-3.494.955
210419_at	8.34e-04	5.23e-04	-3.494.357
216378_at	8.35e-04	5.24e-04	-3.493.918
205823_at	8.35e-04	5.24e-04	349.388
207309_at	8.35e-04	5.24e-04	-3.493.869
205598_at	8.37e-04	5.25e-04	3.493.243
206842_at	8.38e-04	5.25e-04	-3.492.947
221443_x_at	8.38e-04	5.26e-04	-3.492.868
215180_at	8.40e-04	5.27e-04	-3.492.218
221169_s_at	8.40e-04	5.27e-04	-3.492.113
207395_at	8.41e-04	5.27e-04	-3.492.004
211444_at	8.41e-04	5.28e-04	3.491.766
203724_s_at	8.42e-04	5.28e-04	-3.491.545
217228_s_at	8.42e-04	5.28e-04	-349.151
207038_at	8.43e-04	5.28e-04	-349.134
215968_at	8.43e-04	5.29e-04	-3.491.165
216911_s_at	8.44e-04	5.29e-04	-3.490.957
211802_x_at	8.44e-04	5.29e-04	-3.490.829
216887_s_at	8.44e-04	5.30e-04	-3.490.731
210178_x_at	8.45e-04	5.30e-04	3.490.522
201039_s_at	8.45e-04	5.30e-04	3.490.475
212744_at	8.45e-04	5.31e-04	3.490.267
200880_at	8.46e-04	5.31e-04	3.490.179
211851_x_at	8.46e-04	5.31e-04	3.489.994
205428_s_at	8.47e-04	5.32e-04	-3.489.761
204746_s_at	8.47e-04	5.32e-04	-3.489.691
213546_at	8.48e-04	5.32e-04	3.489.409
210271_at	8.48e-04	5.32e-04	-3.489.329
216256_at	8.50e-04	5.34e-04	-3.488.649
209310_s_at	8.54e-04	5.36e-04	3.487.362
207715_at	8.54e-04	5.36e-04	-3.487.276
205001_s_at	8.56e-04	5.37e-04	-3.486.729
201393_s_at	8.57e-04	5.38e-04	348.626
214554_at	8.58e-04	5.39e-04	-3.486.097
215109_at	8.58e-04	5.39e-04	3.485.933
208422_at	8.59e-04	5.40e-04	-3.485.599
219850_s_at	8.60e-04	5.40e-04	3.485.433
215829_at	8.64e-04	5.43e-04	-3.483.991
219432_at	8.66e-04	5.44e-04	-3.483.312

222089_s_at	8.66e-04	5.44e-04	-348.319
209314_s_at	8.68e-04	5.45e-04	3.482.685
206443_at	8.70e-04	5.46e-04	-3.482.129
212134_at	8.70e-04	5.47e-04	-3.482.087
202637_s_at	8.74e-04	5.49e-04	3.480.719
216003_at	8.77e-04	5.51e-04	-3.479.635
213496_at	8.78e-04	5.52e-04	3.479.298
218821_at	8.78e-04	5.52e-04	3.479.229
214822_at	8.79e-04	5.53e-04	-3.479.057
213475_s_at	8.79e-04	5.53e-04	-3.479.053
221003_s_at	8.80e-04	5.53e-04	-3.478.818
207204_at	8.80e-04	5.53e-04	-3.478.785
215184_at	8.80e-04	5.54e-04	3.478.603
219694_at	8.80e-04	5.54e-04	347.858
216961_s_at	8.80e-04	5.54e-04	-3.478.495
207765_s_at	8.87e-04	5.58e-04	-3.476.321
218449_at	8.89e-04	5.59e-04	-3.475.765
206091_at	8.90e-04	5.60e-04	3.475.552
202938_x_at	8.91e-04	5.61e-04	3.475.106
221092_at	8.93e-04	5.62e-04	-3.474.342
210802_s_at	8.95e-04	5.63e-04	3.473.877
213045_at	8.97e-04	5.64e-04	-3.473.346
214276_at	9.02e-04	5.68e-04	-3.471.672
217233_at	9.03e-04	5.68e-04	-347.135
214303_x_at	9.03e-04	5.69e-04	-3.471.238
219840_s_at	9.05e-04	5.70e-04	-3.470.751
219625_s_at	9.05e-04	5.70e-04	-3.470.583
213873_at	9.07e-04	5.71e-04	-3.470.129
206465_at	9.12e-04	5.74e-04	-3.468.581
211826_s_at	9.12e-04	5.74e-04	-3.468.454
216359_at	9.14e-04	5.75e-04	-34.679
203683_s_at	9.14e-04	5.76e-04	346.787
209334_s_at	9.18e-04	5.78e-04	346.662
221886_at	9.19e-04	5.79e-04	-3.466.275
216470_x_at	9.22e-04	5.81e-04	3.465.326
204356_at	9.23e-04	5.81e-04	-3.465.021
213393_at	9.25e-04	5.83e-04	3.464.522
218475_at	9.27e-04	5.84e-04	3.463.937
218912_at	9.28e-04	5.85e-04	3.463.498
220398_at	9.30e-04	5.86e-04	-3.462.972
220880_at	9.30e-04	5.86e-04	-3.462.819
206391_at	9.32e-04	5.87e-04	3.462.365
201428_at	9.32e-04	5.88e-04	3.462.112
215814_at	9.33e-04	5.88e-04	-3.461.932

204680_s_at	9.35e-04	5.89e-04	-3.461.341
214266_s_at	9.35e-04	5.89e-04	-346.125
219052_at	9.38e-04	5.91e-04	3.460.326
202767_at	9.39e-04	5.92e-04	-3.460.063
201242_s_at	9.40e-04	5.93e-04	3.459.657
217285_at	9.41e-04	5.93e-04	-3.459.499
221117_at	9.50e-04	5.99e-04	-3.456.853
208208_at	9.50e-04	5.99e-04	-3.456.844
211898_s_at	9.50e-04	5.99e-04	-3.456.677
206811_at	9.54e-04	6.02e-04	-345.559
207155_at	9.57e-04	6.03e-04	-3.454.749
212071_s_at	9.57e-04	6.04e-04	3.454.666
211132_at	9.58e-04	6.04e-04	-3.454.311
215590_x_at	9.59e-04	6.05e-04	-3.454.001
206708_at	9.63e-04	6.08e-04	3.452.801
210300_at	9.68e-04	6.11e-04	-345.128
221906_at	9.70e-04	6.12e-04	3.450.763
207369_at	9.72e-04	6.13e-04	-3.450.239
202977_s_at	9.72e-04	6.14e-04	-3.450.119
215793_at	9.76e-04	6.16e-04	-344.913
213810_s_at	9.81e-04	6.19e-04	-3.447.742
208423_s_at	9.81e-04	6.19e-04	-3.447.529
217511_at	9.83e-04	6.21e-04	-3.446.986
213354_s_at	9.85e-04	6.22e-04	-344.647
214744_s_at	9.85e-04	6.22e-04	3.446.326
36030_at	9.86e-04	6.23e-04	3.445.974
211102_s_at	9.89e-04	6.25e-04	-3.445.158
215886_x_at	9.89e-04	6.25e-04	-3.445.118
218628_at	9.90e-04	6.26e-04	3.444.782
216422_at	9.91e-04	6.26e-04	344.471
216502_at	9.96e-04	6.29e-04	-3.443.213
210598_at	9.96e-04	6.29e-04	-3.443.198
202147_s_at	9.98e-04	6.30e-04	3.442.723
220821_at	9.99e-04	6.31e-04	-344.221
38340_at	9.99e-04	6.31e-04	3.442.183
222307_at	1.00e-03	6.34e-04	-3.440.848
207123_s_at	1.00e-03	6.35e-04	-3.440.738
215155_at	1.00e-03	6.35e-04	3.440.689
216413_at	1.01e-03	6.36e-04	-344.035
220592_at	1.01e-03	6.36e-04	-3.440.015
219152_at	1.01e-03	6.36e-04	3.440.002
201798_s_at	1.01e-03	6.41e-04	3.438.085
206678_at	1.01e-03	6.41e-04	-343.803
214698_at	1.01e-03	6.41e-04	343.784

207141_s_at	1.02e-03	6.42e-04	-3.437.504
222362_at	1.02e-03	6.43e-04	-3.437.188
212866_at	1.02e-03	6.43e-04	-3.436.985
213233_s_at	1.02e-03	6.45e-04	3.436.191
213932_x_at	1.02e-03	6.45e-04	34.361
206557_at	1.02e-03	6.46e-04	3.435.728
49049_at	1.02e-03	6.47e-04	-3.435.598
204475_at	1.02e-03	6.47e-04	3.435.414
214080_x_at	1.02e-03	6.48e-04	3.434.871
210500_at	1.03e-03	6.49e-04	-3.434.607
204536_s_at	1.03e-03	6.49e-04	-3.434.491
207327_at	1.03e-03	6.52e-04	-3.433.234
203948_s_at	1.03e-03	6.52e-04	-3.433.181
208502_s_at	1.03e-03	6.52e-04	3.433.161
220842_at	1.03e-03	6.53e-04	-3.432.685
36865_at	1.03e-03	6.54e-04	3.432.558
214010_s_at	1.03e-03	6.54e-04	3.432.347
200056_s_at	1.03e-03	6.55e-04	3.431.929
48808_at	1.04e-03	6.56e-04	3.431.512
210850_s_at	1.04e-03	6.57e-04	-3.430.979
201740_at	1.04e-03	6.57e-04	3.430.943
203631_s_at	1.04e-03	6.58e-04	-3.430.617
209907_s_at	1.04e-03	6.59e-04	3.430.312
204122_at	1.04e-03	6.62e-04	-3.429.117
203585_at	1.05e-03	6.63e-04	-342.858
203130_s_at	1.05e-03	6.64e-04	3.428.185
220081_x_at	1.05e-03	6.66e-04	3.427.564
210294_at	1.05e-03	6.66e-04	-3.427.488
208111_at	1.05e-03	6.67e-04	-3.426.902
200835_s_at	1.06e-03	6.69e-04	342.593
205563_at	1.06e-03	6.70e-04	-3.425.851
210685_s_at	1.06e-03	6.71e-04	3.425.472
211153_s_at	1.06e-03	6.71e-04	-3.425.304
204689_at	1.06e-03	6.72e-04	3.425.057
208062_s_at	1.06e-03	6.73e-04	-3.424.487
217098_s_at	1.06e-03	6.73e-04	-3.424.399
206111_at	1.06e-03	6.73e-04	-3.424.335
209602_s_at	1.06e-03	6.74e-04	-3.424.074
207167_at	1.06e-03	6.74e-04	-3.423.918
211467_s_at	1.07e-03	6.77e-04	-3.422.991
213954_at	1.07e-03	6.77e-04	3.422.668
221249_s_at	1.07e-03	6.77e-04	-3.422.659
202437_s_at	1.07e-03	6.79e-04	3.422.028
207222_at	1.07e-03	6.80e-04	-3.421.721

205792_at	1.07e-03	6.80e-04	-342.151
216292_at	1.07e-03	6.81e-04	-3.421.261
220191_at	1.07e-03	6.81e-04	-3.420.942
217121_at	1.08e-03	6.83e-04	-3.420.133
220485_s_at	1.08e-03	6.85e-04	-3.419.595
218902_at	1.08e-03	6.86e-04	-3.419.166
214987_at	1.08e-03	6.86e-04	-3.418.914
214223_at	1.09e-03	6.91e-04	-3.417.038
206281_at	1.09e-03	6.91e-04	-3.416.969
215882_at	1.09e-03	6.91e-04	-3.416.892
216218_s_at	1.09e-03	6.92e-04	3.416.728
205837_s_at	1.09e-03	6.92e-04	-3.416.612
209917_s_at	1.10e-03	6.95e-04	-3.415.327
210206_s_at	1.10e-03	6.96e-04	3.415.109
221882_s_at	1.10e-03	6.97e-04	-3.414.621
208429_x_at	1.10e-03	6.99e-04	-3.413.984
215612_at	1.10e-03	6.99e-04	-3.413.829
216394_x_at	1.10e-03	6.99e-04	-3.413.822
207524_at	1.10e-03	7.01e-04	341.298
214161_at	1.10e-03	7.01e-04	-3.412.949
222082_at	1.11e-03	7.07e-04	-3.410.794
218608_at	1.11e-03	7.07e-04	3.410.699
214691_x_at	1.11e-03	7.07e-04	3.410.654
202146_at	1.11e-03	7.07e-04	3.410.613
212312_at	1.11e-03	7.07e-04	341.051
209412_at	1.11e-03	7.08e-04	3.410.344
216860_s_at	1.11e-03	7.08e-04	3.410.314
206627_s_at	1.11e-03	7.08e-04	3.410.091
203504_s_at	1.12e-03	7.09e-04	-340.986
211558_s_at	1.12e-03	7.12e-04	3.408.864
210400_at	1.12e-03	7.12e-04	-3.408.627
207219_at	1.12e-03	7.13e-04	3.408.389
219460_s_at	1.12e-03	7.15e-04	3.407.554
219414_at	1.13e-03	7.17e-04	-3.406.661
206862_at	1.14e-03	7.23e-04	-3.404.415
207783_x_at	1.14e-03	7.25e-04	-3.403.623
205683_x_at	1.14e-03	7.26e-04	-3.403.328
217123_x_at	1.14e-03	7.26e-04	-3.403.219
219378_at	1.14e-03	7.27e-04	3.403.015
217498_at	1.14e-03	7.27e-04	-3.402.831
220672_at	1.15e-03	7.28e-04	3.402.348
207406_at	1.15e-03	7.29e-04	3.402.194
209708_at	1.15e-03	7.29e-04	-3.402.088
220315_at	1.15e-03	7.29e-04	-3.402.087

211877_s_at	1.15e-03	7.29e-04	-3.402.026
220654_at	1.15e-03	7.30e-04	-340.152
209953_s_at	1.15e-03	7.33e-04	3.400.664
214513_s_at	1.16e-03	7.35e-04	3.399.741
207308_at	1.16e-03	7.35e-04	-3.399.657
213242_x_at	1.16e-03	7.37e-04	-3.398.883
212990_at	1.16e-03	7.40e-04	3.397.788
211822_s_at	1.17e-03	7.43e-04	-3.396.914
200794_x_at	1.17e-03	7.45e-04	339.582
216341_s_at	1.17e-03	7.46e-04	-339.547
207906_at	1.17e-03	7.47e-04	-3.395.265
209389_x_at	1.17e-03	7.47e-04	339.526
215804_at	1.17e-03	7.48e-04	-3.394.847
211556_at	1.17e-03	7.48e-04	-3.394.827
215803_at	1.18e-03	7.49e-04	-3.394.508
220318_at	1.18e-03	7.53e-04	-339.285
217680_x_at	1.19e-03	7.56e-04	3.391.986
AFFX-r2-P1-cre-3_at	1.19e-03	7.56e-04	-3.391.978
215508_at	1.19e-03	7.56e-04	3.391.741
220810_at	1.19e-03	7.56e-04	-3.391.692
220527_at	1.19e-03	7.57e-04	-3.391.475
218127_at	1.19e-03	7.57e-04	3.391.388
208830_s_at	1.19e-03	7.60e-04	-3.390.273
217431_x_at	1.20e-03	7.62e-04	-3.389.665
209770_at	1.20e-03	7.62e-04	-3.389.574
200694_s_at	1.20e-03	7.62e-04	3.389.511
222176_at	1.20e-03	7.64e-04	-3.388.768
211266_s_at	1.20e-03	7.67e-04	-3.387.731
222027_at	1.20e-03	7.67e-04	3.387.655
202371_at	1.20e-03	7.68e-04	3.387.406
216065_at	1.21e-03	7.69e-04	-3.387.201
220492_s_at	1.21e-03	7.70e-04	-3.386.738
205137_x_at	1.21e-03	7.71e-04	3.386.416
217365_at	1.21e-03	7.71e-04	338.622
215509_s_at	1.21e-03	7.71e-04	3.386.178
213014_at	1.21e-03	7.73e-04	-3.385.448
202511_s_at	1.21e-03	7.75e-04	3.385.013
211230_s_at	1.22e-03	7.75e-04	-3.384.781
214338_at	1.22e-03	7.76e-04	-3.384.701
205033_s_at	1.22e-03	7.76e-04	-3.384.613
206568_at	1.22e-03	7.77e-04	-3.384.256
219583_s_at	1.22e-03	7.77e-04	-3.384.108
213450_s_at	1.22e-03	7.77e-04	-3.384
220955_x_at	1.22e-03	7.78e-04	3.383.828

210464_at	1.22e-03	7.79e-04	-3.383.587
206890_at	1.22e-03	7.79e-04	-3.383.263
210340_s_at	1.22e-03	7.82e-04	-3.382.472
205070_at	1.23e-03	7.83e-04	-338.199
202064_s_at	1.23e-03	7.84e-04	3.381.496
207689_at	1.23e-03	7.85e-04	-3.381.173
203044_at	1.23e-03	7.86e-04	3.381.066
208165_s_at	1.23e-03	7.88e-04	3.380.158
201882_x_at	1.24e-03	7.89e-04	-3.379.823
205288_at	1.24e-03	7.91e-04	3.379.156
203876_s_at	1.24e-03	7.92e-04	3.378.614
204039_at	1.24e-03	7.94e-04	3.378.231
220678_at	1.25e-03	7.95e-04	-3.377.557
213768_s_at	1.25e-03	7.97e-04	-3.377.057
220571_at	1.25e-03	8.00e-04	-3.375.903
221650_s_at	1.25e-03	8.00e-04	-3.375.808
218914_at	1.25e-03	8.01e-04	3.375.733
204053_x_at	1.26e-03	8.03e-04	3.375.057
203307_at	1.26e-03	8.04e-04	3.374.527
203982_s_at	1.26e-03	8.04e-04	-3.374.515
211154_at	1.26e-03	8.04e-04	-3.374.381
217554_at	1.26e-03	8.05e-04	-3.374.104
211008_s_at	1.26e-03	8.06e-04	-3.373.964
219833_s_at	1.26e-03	8.07e-04	3.373.534
206798_x_at	1.26e-03	8.07e-04	-3.373.428
200827_at	1.26e-03	8.07e-04	3.373.386
203421_at	1.26e-03	8.07e-04	3.373.323
220309_at	1.26e-03	8.08e-04	3.373.208
203450_at	1.27e-03	8.09e-04	3.372.631
218861_at	1.27e-03	8.09e-04	3.372.627
202608_s_at	1.27e-03	8.10e-04	-3.372.587
220759_at	1.27e-03	8.10e-04	-3.372.476
216331_at	1.27e-03	8.11e-04	337.214
215834_x_at	1.27e-03	8.11e-04	3.372.135
206529_x_at	1.27e-03	8.12e-04	-3.371.772
222331_at	1.27e-03	8.13e-04	-3.371.467
211607_x_at	1.27e-03	8.13e-04	-3.371.385
210439_at	1.27e-03	8.13e-04	-3.371.295
213100_at	1.27e-03	8.14e-04	-3.371.119
204616_at	1.27e-03	8.14e-04	337.109
208885_at	1.27e-03	8.14e-04	-3.371.055
214660_at	1.27e-03	8.14e-04	-337.096
219914_at	1.28e-03	8.16e-04	-3.370.192
220861_at	1.29e-03	8.23e-04	-3.368.015

218841_at	1.29e-03	8.23e-04	-3.367.817
218036_x_at	1.29e-03	8.25e-04	3.367.239
221742_at	1.29e-03	8.26e-04	-3.366.869
213767_at	1.29e-03	8.26e-04	-3.366.829
222053_at	1.29e-03	8.28e-04	-3.366.259
212874_at	1.29e-03	8.29e-04	-3.366.025
203505_at	1.29e-03	8.29e-04	-3.365.837
215933_s_at	1.30e-03	8.32e-04	3.365.018
205629_s_at	1.30e-03	8.32e-04	-3.364.919
220093_at	1.30e-03	8.32e-04	-3.364.735
217734_s_at	1.30e-03	8.32e-04	3.364.729
205910_s_at	1.30e-03	8.33e-04	3.364.499
59631_at	1.30e-03	8.34e-04	3.364.307
221638_s_at	1.30e-03	8.35e-04	-3.363.983
211878_at	1.31e-03	8.37e-04	-3.363.255
208260_at	1.31e-03	8.38e-04	-3.362.663
205280_at	1.31e-03	8.40e-04	3.362.237
210318_at	1.31e-03	8.41e-04	-336.176
216761_at	1.31e-03	8.42e-04	-3.361.581
216225_at	1.31e-03	8.42e-04	-3.361.533
214026_s_at	1.31e-03	8.42e-04	-3.361.502
211117_x_at	1.31e-03	8.42e-04	-3.361.379
211599_x_at	1.32e-03	8.44e-04	3.360.792
219532_at	1.32e-03	8.44e-04	3.360.719
210122_at	1.32e-03	8.45e-04	-3.360.431
41469_at	1.32e-03	8.45e-04	3.360.357
217492_s_at	1.32e-03	8.48e-04	3.359.353
214278_s_at	1.32e-03	8.49e-04	-3.359.152
210651_s_at	1.32e-03	8.50e-04	-3.358.847
206397_x_at	1.33e-03	8.50e-04	3.358.767
221252_s_at	1.33e-03	8.50e-04	-3.358.704
215765_at	1.33e-03	8.51e-04	-3.358.476
201718_s_at	1.33e-03	8.56e-04	3.356.884
210674_s_at	1.34e-03	8.57e-04	3.356.476
220360_at	1.34e-03	8.58e-04	-3.356.033
208531_at	1.34e-03	8.59e-04	-3.355.732
203690_at	1.34e-03	8.59e-04	3.355.686
204492_at	1.34e-03	8.61e-04	3.355.254
216885_s_at	1.34e-03	8.62e-04	-3.354.805
220195_at	1.34e-03	8.63e-04	3.354.643
206897_at	1.34e-03	8.63e-04	3.354.566
211649_x_at	1.35e-03	8.64e-04	-3.354.126
211659_at	1.35e-03	8.67e-04	-3.353.159
208259_x_at	1.35e-03	8.69e-04	-3.352.663

202791_s_at	1.35e-03	8.69e-04	3.352.577
220978_at	1.35e-03	8.70e-04	-3.352.285
207915_at	1.36e-03	8.71e-04	-3.351.864
215611_at	1.36e-03	8.73e-04	-3.351.318
204678_s_at	1.36e-03	8.75e-04	335.067
205488_at	1.37e-03	8.81e-04	-334.857
220100_at	1.38e-03	8.86e-04	3.346.961
208190_s_at	1.38e-03	8.86e-04	-3.346.954
204377_s_at	1.38e-03	8.88e-04	-3.346.369
214395_x_at	1.38e-03	8.89e-04	3.346.214
211300_s_at	1.38e-03	8.90e-04	3.345.856
221745_at	1.39e-03	8.92e-04	334.519
208516_at	1.39e-03	8.93e-04	-3.344.627
204951_at	1.39e-03	8.94e-04	-3.344.337
203940_s_at	1.40e-03	8.98e-04	3.343.055
203066_at	1.41e-03	9.03e-04	-3.341.476
220385_at	1.41e-03	9.04e-04	-3.341.415
217584_at	1.41e-03	9.06e-04	3.340.816
220501_at	1.41e-03	9.06e-04	-3.340.577
212313_at	1.41e-03	9.07e-04	-3.340.337
207747_s_at	1.41e-03	9.07e-04	-3.340.217
40284_at	1.41e-03	9.09e-04	-3.339.615
217282_at	1.41e-03	9.10e-04	-3.339.503
219406_at	1.42e-03	9.11e-04	-3.339.257
218849_s_at	1.42e-03	9.11e-04	3.339.043
208098_at	1.42e-03	9.12e-04	-3.338.909
216428_x_at	1.42e-03	9.12e-04	-3.338.785
206687_s_at	1.42e-03	9.12e-04	-3.338.664
221244_s_at	1.42e-03	9.13e-04	-333.843
215073_s_at	1.42e-03	9.16e-04	-3.337.701
214705_at	1.42e-03	9.17e-04	-3.337.241
89948_at	1.43e-03	9.18e-04	-3.337.044
207422_at	1.43e-03	9.20e-04	-333.643
207005_s_at	1.44e-03	9.26e-04	-3.334.578
221763_at	1.44e-03	9.29e-04	-3.333.595
208141_s_at	1.45e-03	9.31e-04	3.333.009
201450_s_at	1.45e-03	9.31e-04	3.332.959
204250_s_at	1.45e-03	9.31e-04	-3.332.865
204034_at	1.45e-03	9.31e-04	-3.332.859
206011_at	1.45e-03	9.33e-04	-3.332.162
218306_s_at	1.45e-03	9.36e-04	3.331.528
214383_x_at	1.45e-03	9.36e-04	333.144
220890_s_at	1.45e-03	9.36e-04	3.331.312
221030_s_at	1.45e-03	9.37e-04	-3.331.215

204959_at	1.45e-03	9.37e-04	-3.331.135
216585_at	1.46e-03	9.39e-04	-333.045
210374_x_at	1.47e-03	9.44e-04	-3.328.883
207128_s_at	1.47e-03	9.45e-04	3.328.704
202792_s_at	1.47e-03	9.45e-04	-3.328.703
201017_at	1.47e-03	9.47e-04	-332.816
211365_s_at	1.47e-03	9.48e-04	-332.766
201645_at	1.48e-03	9.53e-04	332.619
221289_at	1.48e-03	9.54e-04	-3.326.028
217406_at	1.48e-03	9.55e-04	-3.325.786
216368_s_at	1.48e-03	9.55e-04	-3.325.644
209658_at	1.48e-03	9.55e-04	3.325.582
202310_s_at	1.48e-03	9.55e-04	3.325.526
207757_at	1.48e-03	9.57e-04	-3.325.161
204396_s_at	1.48e-03	9.57e-04	-332.497
217216_x_at	1.48e-03	9.57e-04	3.324.921
218666_s_at	1.48e-03	9.57e-04	-3.324.904
205448_s_at	1.49e-03	9.59e-04	-3.324.587
216055_at	1.49e-03	9.60e-04	-3.324.232
201525_at	1.49e-03	9.61e-04	-3.323.854
214411_x_at	1.49e-03	9.61e-04	-3.323.854
203320_at	1.50e-03	9.67e-04	-3.322.175
222063_s_at	1.50e-03	9.71e-04	-3.320.939
217194_at	1.51e-03	9.73e-04	-3.320.432
221802_s_at	1.51e-03	9.76e-04	331.944
205246_at	1.51e-03	9.77e-04	3.319.006
220556_at	1.52e-03	9.78e-04	-3.318.729
220588_at	1.52e-03	9.79e-04	3.318.522
204881_s_at	1.52e-03	9.80e-04	3.318.141
213522_s_at	1.52e-03	9.81e-04	-3.317.886
215820_x_at	1.52e-03	9.81e-04	-3.317.824
202507_s_at	1.53e-03	9.85e-04	3.316.775
221159_at	1.53e-03	9.86e-04	-3.316.567
212559_at	1.53e-03	9.89e-04	-3.315.636
219256_s_at	1.54e-03	9.92e-04	-3.314.675
207206_s_at	1.54e-03	9.93e-04	-3.314.464
220741_s_at	1.54e-03	9.97e-04	-3.313.254
209095_at	1.55e-03	9.98e-04	3.313.002
203416_at	1.55e-03	1.00e-03	-3.312.562
216183_at	1.55e-03	1.00e-03	-3.312.301
216476_at	1.55e-03	1.00e-03	-3.312.255
212912_at	1.55e-03	1.00e-03	-3.311.984
219197_s_at	1.55e-03	1.00e-03	3.311.919
201213_at	1.56e-03	1.01e-03	3.310.912

211652_s_at	1.56e-03	1.01e-03	-331.065
216015_s_at	1.56e-03	1.01e-03	-3.310.635
AFFX-HUMISGF3A/M9	1.56e-03	1.01e-03	3.310.079
217347_at	1.56e-03	1.01e-03	3.309.874
208011_at	1.57e-03	1.01e-03	-3.308.207
217056_at	1.57e-03	1.02e-03	-3.307.978
214674_at	1.57e-03	1.02e-03	3.307.701
216034_at	1.58e-03	1.02e-03	-3.307.281
211902_x_at	1.58e-03	1.02e-03	-3.306.715
214036_at	1.58e-03	1.02e-03	-3.306.021
216808_at	1.58e-03	1.02e-03	-3.306.013
216008_s_at	1.58e-03	1.02e-03	3.305.895
215940_at	1.58e-03	1.02e-03	-3.305.695
217101_at	1.60e-03	1.03e-03	-3.302.752
212671_s_at	1.60e-03	1.04e-03	-3.302.109
207896_s_at	1.60e-03	1.04e-03	-3.301.852
201311_s_at	1.61e-03	1.04e-03	-3.301.288
220974_x_at	1.61e-03	1.04e-03	3.301.262
214968_at	1.61e-03	1.04e-03	-3.300.681
213740_s_at	1.61e-03	1.04e-03	-3.300.419
221997_s_at	1.61e-03	1.04e-03	3.300.253
203309_s_at	1.62e-03	1.05e-03	3.299.822
205157_s_at	1.62e-03	1.05e-03	-3.299.537
210040_at	1.62e-03	1.05e-03	-3.298.941
218206_x_at	1.62e-03	1.05e-03	3.298.536
221681_s_at	1.62e-03	1.05e-03	-329.815
216419_at	1.63e-03	1.05e-03	-3.297.891
210654_at	1.63e-03	1.05e-03	-3.297.676
210369_at	1.63e-03	1.05e-03	-3.297.177
205718_at	1.63e-03	1.06e-03	-3.296.399
201151_s_at	1.64e-03	1.06e-03	3.295.898
214232_at	1.64e-03	1.06e-03	-3.295.865
56829_at	1.64e-03	1.06e-03	3.295.582
215799_at	1.64e-03	1.06e-03	-3.295.503
200688_at	1.64e-03	1.06e-03	3.294.727
222370_x_at	1.65e-03	1.07e-03	-3.294.198
204255_s_at	1.65e-03	1.07e-03	3.294.036
219012_s_at	1.65e-03	1.07e-03	3.293.604
217523_at	1.65e-03	1.07e-03	-3.293.521
215174_at	1.65e-03	1.07e-03	-329.337
205884_at	1.66e-03	1.07e-03	-329.197
203700_s_at	1.66e-03	1.08e-03	3.291.571
211586_s_at	1.67e-03	1.08e-03	32.904
219919_s_at	1.67e-03	1.08e-03	3.290.224

211064_at	1.67e-03	1.08e-03	3.289.855
210994_x_at	1.67e-03	1.08e-03	3.289.441
201337_s_at	1.67e-03	1.08e-03	328.918
207045_at	1.67e-03	1.09e-03	3.288.848
215099_s_at	1.68e-03	1.09e-03	-3.287.996
201308_s_at	1.68e-03	1.09e-03	-3.287.854
206829_x_at	1.68e-03	1.09e-03	3.287.799
220050_at	1.68e-03	1.09e-03	-3.287.521
209833_at	1.68e-03	1.09e-03	-3.287.187
216685_s_at	1.69e-03	1.09e-03	-3.286.501
207787_at	1.69e-03	1.10e-03	-3.285.918
209945_s_at	1.69e-03	1.10e-03	3.285.303
212821_at	1.70e-03	1.10e-03	-3.285.079
214543_x_at	1.70e-03	1.10e-03	-3.283.921
206785_s_at	1.70e-03	1.10e-03	-3.283.857
214694_at	1.70e-03	1.11e-03	-3.283.594
207195_at	1.71e-03	1.11e-03	-3.283.058
209754_s_at	1.71e-03	1.11e-03	3.282.903
212982_at	1.71e-03	1.11e-03	3.282.533
216596_at	1.71e-03	1.11e-03	-3.282.524
221192_x_at	1.71e-03	1.11e-03	-3.281.737
213854_at	1.72e-03	1.11e-03	3.281.246
221085_at	1.72e-03	1.11e-03	3.281.176
210806_at	1.73e-03	1.12e-03	-3.278.996
219810_at	1.73e-03	1.12e-03	3.278.807
209650_s_at	1.73e-03	1.13e-03	3.278.386
220711_at	1.74e-03	1.13e-03	-3.277.891
205621_at	1.74e-03	1.13e-03	3.277.016
215041_s_at	1.75e-03	1.13e-03	-3.276.363
203540_at	1.75e-03	1.13e-03	-3.276.135
215478_at	1.75e-03	1.13e-03	-3.276.032
207506_at	1.75e-03	1.14e-03	-3.275.963
215271_at	1.75e-03	1.14e-03	-3.275.668
215243_s_at	1.75e-03	1.14e-03	-3.275.452
216714_at	1.76e-03	1.14e-03	-3.274.379
221624_at	1.76e-03	1.14e-03	-327.387
216325_x_at	1.76e-03	1.14e-03	-3.273.621
211560_s_at	1.76e-03	1.14e-03	-3.273.519
210260_s_at	1.76e-03	1.15e-03	3.273.392
204453_at	1.77e-03	1.15e-03	3.272.421
207505_at	1.77e-03	1.15e-03	-3.272.312
207044_at	1.77e-03	1.15e-03	-3.271.887
215359_x_at	1.77e-03	1.15e-03	-3.271.865
215938_s_at	1.78e-03	1.16e-03	3.270.941

219659_at	1.78e-03	1.16e-03	-327.001
205786_s_at	1.79e-03	1.16e-03	3.269.575
219419_at	1.79e-03	1.16e-03	326.923
215866_at	1.79e-03	1.16e-03	-3.268.875
210051_at	1.79e-03	1.16e-03	-3.268.818
220278_at	1.79e-03	1.16e-03	-3.268.789
217691_x_at	1.79e-03	1.16e-03	3.268.498
205975_s_at	1.80e-03	1.17e-03	3.267.818
205680_at	1.80e-03	1.17e-03	326.736
207310_s_at	1.80e-03	1.17e-03	-3.266.972
204810_s_at	1.81e-03	1.18e-03	-3.264.621
221369_at	1.82e-03	1.18e-03	-3.264.305
201544_x_at	1.82e-03	1.18e-03	3.264.091
202803_s_at	1.82e-03	1.18e-03	-3.263.968
204588_s_at	1.82e-03	1.18e-03	-3.263.757
209129_at	1.83e-03	1.19e-03	32.626
203685_at	1.83e-03	1.19e-03	-3.261.839
206877_at	1.84e-03	1.19e-03	-3.261.224
209067_s_at	1.84e-03	1.20e-03	3.260.723
222287_at	1.84e-03	1.20e-03	-3.260.692
219255_x_at	1.84e-03	1.20e-03	3.260.554
208280_at	1.85e-03	1.20e-03	-3.259.338
215317_at	1.85e-03	1.20e-03	-3.259.113
222152_at	1.85e-03	1.20e-03	-325.871
55616_at	1.85e-03	1.21e-03	-3.258.537
221297_at	1.85e-03	1.21e-03	-3.258.328
208772_at	1.85e-03	1.21e-03	3.258.026
209446_s_at	1.86e-03	1.21e-03	3.257.809
205997_at	1.86e-03	1.21e-03	-3.257.772
214738_s_at	1.86e-03	1.21e-03	-3.257.718
208307_at	1.86e-03	1.21e-03	3.257.676
216895_at	1.86e-03	1.21e-03	-3.257.438
206924_at	1.86e-03	1.21e-03	-3.257.398
215481_s_at	1.87e-03	1.22e-03	-3.256.141
210861_s_at	1.87e-03	1.22e-03	-3.256.104
208917_x_at	1.87e-03	1.22e-03	-3.256.058
213630_at	1.87e-03	1.22e-03	-3.256.027
219749_at	1.87e-03	1.22e-03	-3.255.914
219754_at	1.87e-03	1.22e-03	-3.255.649
208170_s_at	1.87e-03	1.22e-03	3.255.354
207529_at	1.87e-03	1.22e-03	-3.255.259
210148_at	1.87e-03	1.22e-03	32.548
90265_at	1.87e-03	1.22e-03	-3.254.723
220575_at	1.88e-03	1.22e-03	-325.395

215374_at	1.88e-03	1.23e-03	-3.253.779
213353_at	1.88e-03	1.23e-03	-3.253.165
208077_at	1.90e-03	1.24e-03	-3.249.903
212291_at	1.91e-03	1.24e-03	-324.925
203398_s_at	1.91e-03	1.25e-03	-3.248.624
214973_x_at	1.91e-03	1.25e-03	-3.248.303
209683_at	1.91e-03	1.25e-03	3.248.283
221152_at	1.92e-03	1.25e-03	-3.248.054
201933_at	1.92e-03	1.25e-03	324.762
206006_s_at	1.92e-03	1.25e-03	3.247.618
210322_x_at	1.92e-03	1.25e-03	-3.247.445
216415_at	1.92e-03	1.25e-03	-3.247.226
219647_at	1.92e-03	1.25e-03	-3.247.042
206105_at	1.92e-03	1.26e-03	-3.246.734
207207_at	1.93e-03	1.26e-03	-324.624
215986_at	1.93e-03	1.26e-03	-324.619
212421_at	1.93e-03	1.26e-03	3.246.016
216504_s_at	1.93e-03	1.26e-03	-3.245.206
204743_at	1.94e-03	1.26e-03	-3.244.714
211119_at	1.94e-03	1.27e-03	-3.244.459
200970_s_at	1.94e-03	1.27e-03	3.244.234
209270_at	1.94e-03	1.27e-03	3.243.514
218885_s_at	1.94e-03	1.27e-03	3.243.406
212721_at	1.95e-03	1.27e-03	3.242.921
219031_s_at	1.95e-03	1.27e-03	3.242.385
220188_at	1.95e-03	1.28e-03	-3.242.097
205952_at	1.96e-03	1.28e-03	-324.058
217702_at	1.96e-03	1.28e-03	-3.240.349
214013_s_at	1.97e-03	1.28e-03	-3.240.066
208173_at	1.97e-03	1.28e-03	-323.999
206521_s_at	1.97e-03	1.29e-03	-3.239.708
222272_x_at	1.97e-03	1.29e-03	-3.239.521
217723_x_at	1.97e-03	1.29e-03	-3.239.343
215853_at	1.97e-03	1.29e-03	-3.239.327
211910_at	1.97e-03	1.29e-03	-3.238.703
221684_s_at	1.99e-03	1.30e-03	-3.237.079
207014_at	1.99e-03	1.30e-03	3.237.077
AFFX-r2-Bs-lys-5_at	1.99e-03	1.30e-03	-3.235.754
202061_s_at	2.00e-03	1.31e-03	-3.234.792
210982_s_at	2.00e-03	1.31e-03	-3.234.563
217545_at	2.00e-03	1.31e-03	-32.344
219484_at	2.01e-03	1.32e-03	323.272
207957_s_at	2.02e-03	1.32e-03	-3.232.371
202627_s_at	2.02e-03	1.32e-03	-3.231.983

213061_s_at	2.02e-03	1.32e-03	3.231.762
203859_s_at	2.02e-03	1.32e-03	-3.231.513
209677_at	2.02e-03	1.32e-03	3.231.437
217830_s_at	2.02e-03	1.32e-03	3.231.144
220782_x_at	2.03e-03	1.32e-03	-3.230.997
218255_s_at	2.03e-03	1.33e-03	-3.230.947
208386_x_at	2.03e-03	1.33e-03	-3.230.266
206954_at	2.03e-03	1.33e-03	-3.229.532
212224_at	2.04e-03	1.33e-03	-322.939
206371_at	2.04e-03	1.33e-03	-3.228.875
221077_at	2.04e-03	1.34e-03	-3.228.481
213735_s_at	2.05e-03	1.34e-03	3.227.696
220033_at	2.05e-03	1.34e-03	3.227.035
201835_s_at	2.05e-03	1.34e-03	322.687
206442_at	2.05e-03	1.35e-03	-3.226.549
217023_x_at	2.06e-03	1.35e-03	-322.613
203092_at	2.06e-03	1.35e-03	3.225.944
210710_at	2.06e-03	1.35e-03	3.225.878
218476_at	2.06e-03	1.35e-03	3.225.261
AFFX-DapX-3_at	2.06e-03	1.35e-03	-3.225.016
211695_x_at	2.07e-03	1.35e-03	-3.224.714
217189_s_at	2.07e-03	1.36e-03	-3.224.271
220508_at	2.08e-03	1.36e-03	-3.223.239
207079_s_at	2.08e-03	1.36e-03	3.223.072
215928_at	2.08e-03	1.36e-03	-3.222.954
212278_x_at	2.08e-03	1.36e-03	-3.222.706
217772_s_at	2.08e-03	1.36e-03	3.222.532
218610_s_at	2.08e-03	1.36e-03	3.222.446
202568_s_at	2.08e-03	1.37e-03	3.222.057
203787_at	2.10e-03	1.37e-03	3.220.308
218940_at	2.10e-03	1.38e-03	3.219.595
201916_s_at	2.10e-03	1.38e-03	3.219.337
216405_at	2.10e-03	1.38e-03	-321.919
205010_at	2.11e-03	1.38e-03	3.218.636
209969_s_at	2.11e-03	1.38e-03	3.218.463
221368_at	2.11e-03	1.38e-03	-3.218.403
217776_at	2.11e-03	1.38e-03	3.218.363
204738_s_at	2.11e-03	1.38e-03	-3.218.215
210656_at	2.11e-03	1.39e-03	3.217.719
211024_s_at	2.11e-03	1.39e-03	-3.217.711
202208_s_at	2.11e-03	1.39e-03	-3.217.707
201181_at	2.12e-03	1.39e-03	3.217.221
204950_at	2.12e-03	1.39e-03	3.216.719
219764_at	2.12e-03	1.39e-03	-3.216.717

208525_s_at	2.13e-03	1.39e-03	-3.216.058
203476_at	2.13e-03	1.39e-03	-3.215.923
205390_s_at	2.13e-03	1.40e-03	-3.215.646
204292_x_at	2.13e-03	1.40e-03	-3.215.111
206307_s_at	2.13e-03	1.40e-03	-3.214.951
216550_x_at	2.14e-03	1.40e-03	-3.214.103
213819_s_at	2.14e-03	1.40e-03	-3.213.956
204692_at	2.15e-03	1.41e-03	-3.212.904
220299_at	2.15e-03	1.41e-03	-3.212.668
219849_at	2.15e-03	1.41e-03	-3.212.045
202478_at	2.15e-03	1.41e-03	3.212.006
219021_at	2.15e-03	1.41e-03	3.211.962
210528_at	2.16e-03	1.42e-03	-3.211.689
204069_at	2.16e-03	1.42e-03	3.211.003
209624_s_at	2.16e-03	1.42e-03	-3.210.979
216786_at	2.16e-03	1.42e-03	-3.210.919
204730_at	2.17e-03	1.42e-03	3.210.134
210956_at	2.17e-03	1.42e-03	-3.209.636
221226_s_at	2.18e-03	1.43e-03	-3.208.827
217448_s_at	2.18e-03	1.43e-03	3.208.737
211018_at	2.18e-03	1.43e-03	3.208.482
218892_at	2.18e-03	1.43e-03	-3.208.437
210356_x_at	2.18e-03	1.43e-03	-3.207.986
44146_at	2.18e-03	1.43e-03	3.207.902
212276_at	2.19e-03	1.44e-03	3.206.966
221667_s_at	2.19e-03	1.44e-03	320.647
208293_x_at	2.20e-03	1.45e-03	-3.205.098
207318_s_at	2.20e-03	1.45e-03	3.205.009
205206_at	2.21e-03	1.45e-03	-3.204.326
216257_at	2.22e-03	1.46e-03	-3.202.945
217628_at	2.23e-03	1.46e-03	-3.201.888
215311_at	2.24e-03	1.47e-03	-3.200.293
217418_x_at	2.24e-03	1.47e-03	-3.199.564
204925_at	2.24e-03	1.48e-03	3.199.295
202339_at	2.25e-03	1.48e-03	-319.919
214982_at	2.25e-03	1.48e-03	-319.902
213317_at	2.25e-03	1.48e-03	3.198.532
201602_s_at	2.25e-03	1.48e-03	3.198.324
213597_s_at	2.26e-03	1.48e-03	-3.197.775
216399_s_at	2.26e-03	1.48e-03	3.197.716
216659_at	2.26e-03	1.49e-03	-3.197.443
213872_at	2.26e-03	1.49e-03	3.197.016
208617_s_at	2.27e-03	1.49e-03	3.196.344
207342_at	2.27e-03	1.49e-03	-3.196.172

217438_at	2.27e-03	1.49e-03	-3.195.654
202887_s_at	2.27e-03	1.49e-03	3.195.525
203798_s_at	2.28e-03	1.50e-03	3.194.734
218427_at	2.28e-03	1.50e-03	3.194.508
208110_x_at	2.28e-03	1.50e-03	3.193.844
217760_at	2.29e-03	1.51e-03	3.193.431
215387_x_at	2.29e-03	1.51e-03	-3.192.486
206198_s_at	2.30e-03	1.51e-03	-3.192.255
214531_s_at	2.30e-03	1.51e-03	319.218
205964_at	2.30e-03	1.51e-03	3.192.036
207322_at	2.30e-03	1.52e-03	-3.191.383
51200_at	2.31e-03	1.52e-03	-3.190.623
216691_at	2.31e-03	1.52e-03	-3.190.011
206291_at	2.31e-03	1.52e-03	3.189.858
202949_s_at	2.32e-03	1.53e-03	-3.189.602
214883_at	2.32e-03	1.53e-03	-3.189.358
206131_at	2.32e-03	1.53e-03	-3.188.934
216075_at	2.32e-03	1.53e-03	-3.188.757
221198_at	2.33e-03	1.53e-03	-3.187.979
214849_at	2.33e-03	1.54e-03	3.187.438
219969_at	2.33e-03	1.54e-03	3.187.103
214116_at	2.34e-03	1.54e-03	-3.186.875
212907_at	2.34e-03	1.54e-03	-318.674
219813_at	2.35e-03	1.55e-03	-318.518
220977_x_at	2.35e-03	1.55e-03	-3.184.963
216940_x_at	2.36e-03	1.55e-03	-3.184.067
200953_s_at	2.36e-03	1.56e-03	3.183.259
208740_at	2.36e-03	1.56e-03	-3.183.121
220437_at	2.37e-03	1.56e-03	-3.183.026
207401_at	2.37e-03	1.56e-03	-3.182.949
209769_s_at	2.37e-03	1.56e-03	-3.182.254
206881_s_at	2.37e-03	1.56e-03	-3.182.201
206887_at	2.37e-03	1.56e-03	-3.182.045
211367_s_at	2.38e-03	1.57e-03	-3.181.601
205572_at	2.38e-03	1.57e-03	3.181.272
217470_at	2.38e-03	1.57e-03	-3.180.698
209050_s_at	2.39e-03	1.57e-03	318.012
201253_s_at	2.39e-03	1.58e-03	317.999
213167_s_at	2.39e-03	1.58e-03	-3.179.717
219589_s_at	2.39e-03	1.58e-03	-3.179.532
208545_x_at	2.40e-03	1.58e-03	-317.876
210627_s_at	2.40e-03	1.58e-03	3.178.565
201204_s_at	2.40e-03	1.58e-03	-3.178.351
207351_s_at	2.41e-03	1.59e-03	-3.177.471

213278_at	2.42e-03	1.60e-03	-3.176.217
220294_at	2.42e-03	1.60e-03	-317.603
220534_at	2.43e-03	1.60e-03	-3.175.201
211428_at	2.43e-03	1.60e-03	-3.175.108
210726_at	2.43e-03	1.60e-03	-3.174.587
202734_at	2.43e-03	1.61e-03	317.425
203595_s_at	2.43e-03	1.61e-03	3.174.115
210316_at	2.43e-03	1.61e-03	-3.174.052
216190_x_at	2.44e-03	1.61e-03	3.172.812
215513_at	2.45e-03	1.62e-03	-3.172.403
221743_at	2.45e-03	1.62e-03	-3.172.287
222181_at	2.45e-03	1.62e-03	-317.212
211958_at	2.45e-03	1.62e-03	-3.171.599
211901_s_at	2.45e-03	1.62e-03	-3.171.594
200969_at	2.46e-03	1.62e-03	-3.171.302
206370_at	2.46e-03	1.62e-03	-3.170.951
215232_at	2.46e-03	1.63e-03	-3.170.429
215598_at	2.47e-03	1.63e-03	-3.169.575
213042_s_at	2.47e-03	1.64e-03	-3.168.933
216935_at	2.48e-03	1.64e-03	-3.168.738
205944_s_at	2.48e-03	1.64e-03	3.168.093
219399_at	2.49e-03	1.65e-03	316.687
203949_at	2.50e-03	1.65e-03	-3.166.316
218701_at	2.50e-03	1.65e-03	3.166.261
208085_s_at	2.50e-03	1.65e-03	-3.166.027
208840_s_at	2.50e-03	1.66e-03	3.165.283
210724_at	2.50e-03	1.66e-03	-3.165.194
217015_at	2.51e-03	1.66e-03	-3.164.545
206467_x_at	2.51e-03	1.66e-03	-316.447
204777_s_at	2.51e-03	1.66e-03	-3.164.365
208532_x_at	2.51e-03	1.66e-03	-3.163.928
203295_s_at	2.52e-03	1.67e-03	-3.163.124
221472_at	2.52e-03	1.67e-03	316.273
205520_at	2.53e-03	1.67e-03	-316.262
204792_s_at	2.53e-03	1.67e-03	-3.161.786
205051_s_at	2.54e-03	1.68e-03	3.161.264
206917_at	2.54e-03	1.68e-03	3.160.704
203727_at	2.54e-03	1.68e-03	-3.160.237
205282_at	2.55e-03	1.69e-03	3.159.365
215751_at	2.55e-03	1.69e-03	-3.159.341
204213_at	2.55e-03	1.69e-03	-3.159.083
207490_at	2.56e-03	1.69e-03	3.158.358
217582_at	2.56e-03	1.70e-03	-3.157.969
218890_x_at	2.57e-03	1.70e-03	315.774

215877_at	2.57e-03	1.70e-03	-3.157.276
221608_at	2.58e-03	1.71e-03	-3.155.868
204479_at	2.59e-03	1.71e-03	-3.155.313
205179_s_at	2.59e-03	1.72e-03	-3.154.598
38069_at	2.59e-03	1.72e-03	3.154.483
206330_s_at	2.59e-03	1.72e-03	-3.154.342
206089_at	2.59e-03	1.72e-03	-3.154.157
216348_at	2.60e-03	1.72e-03	3.153.944
206850_at	2.60e-03	1.72e-03	-3.153.932
222166_at	2.60e-03	1.72e-03	-3.153.776
216288_at	2.60e-03	1.72e-03	-3.153.622
205698_s_at	2.60e-03	1.72e-03	-3.153.273
207831_x_at	2.60e-03	1.73e-03	3.152.889
220451_s_at	2.61e-03	1.73e-03	-3.152.851
218228_s_at	2.61e-03	1.73e-03	3.152.599
208894_at	2.61e-03	1.73e-03	-3.152.282
213783_at	2.61e-03	1.73e-03	-3.151.984
206694_at	2.61e-03	1.73e-03	-3.151.922
206498_at	2.62e-03	1.74e-03	3.150.923
206258_at	2.62e-03	1.74e-03	-3.150.915
214121_x_at	2.63e-03	1.75e-03	3.149.384
220194_at	2.64e-03	1.75e-03	-3.149.044
203647_s_at	2.64e-03	1.75e-03	-3.148.662
210084_x_at	2.64e-03	1.75e-03	-3.148.638
209587_at	2.64e-03	1.75e-03	3.148.616
206125_s_at	2.64e-03	1.75e-03	-3.148.116
208866_at	2.65e-03	1.75e-03	-3.148.004
217265_at	2.65e-03	1.76e-03	-3.147.406
207538_at	2.65e-03	1.76e-03	-314.732
207676_at	2.65e-03	1.76e-03	3.147.109
211096_at	2.65e-03	1.76e-03	-3.147.013
219176_at	2.66e-03	1.76e-03	-3.146.801
207930_at	2.66e-03	1.76e-03	-314.662
206334_at	2.66e-03	1.76e-03	-3.146.333
217626_at	2.66e-03	1.76e-03	-314.623
215639_at	2.66e-03	1.76e-03	-3.146.226
217430_x_at	2.67e-03	1.77e-03	-3.145.508
210414_at	2.67e-03	1.77e-03	-3.145.486
203197_s_at	2.67e-03	1.77e-03	-3.145.323
221951_at	2.67e-03	1.77e-03	3.144.791
34210_at	2.68e-03	1.78e-03	-3.143.885
220219_s_at	2.68e-03	1.78e-03	-3.143.396
207661_s_at	2.69e-03	1.78e-03	-3.143.207
203054_s_at	2.69e-03	1.78e-03	3.142.878

208214_at	2.69e-03	1.79e-03	-3.142.728
206768_at	2.69e-03	1.79e-03	-3.142.497
203796_s_at	2.69e-03	1.79e-03	3.142.212
208708_x_at	2.70e-03	1.80e-03	-3.141.005
215887_at	2.72e-03	1.80e-03	-3.139.588
210224_at	2.72e-03	1.81e-03	-3.139.416
213394_at	2.72e-03	1.81e-03	313.936
215697_at	2.72e-03	1.81e-03	-3.139.347
215305_at	2.72e-03	1.81e-03	-3.139.112
220531_at	2.72e-03	1.81e-03	-3.138.739
209359_x_at	2.74e-03	1.82e-03	-3.136.552
205558_at	2.74e-03	1.82e-03	-3.136.524
214389_at	2.75e-03	1.82e-03	-3.136.332
221898_at	2.75e-03	1.83e-03	-3.135.725
212602_at	2.76e-03	1.83e-03	3.135.094
221726_at	2.76e-03	1.83e-03	313.454
218808_at	2.76e-03	1.84e-03	-313.419
205196_s_at	2.77e-03	1.84e-03	3.134.042
206274_s_at	2.77e-03	1.84e-03	3.133.893
210529_s_at	2.77e-03	1.84e-03	313.387
216344_at	2.77e-03	1.84e-03	-3.133.575
206927_s_at	2.77e-03	1.84e-03	-3.133.006
204638_at	2.78e-03	1.85e-03	3.132.858
202256_at	2.78e-03	1.85e-03	3.132.631
221315_s_at	2.78e-03	1.85e-03	-3.131.804
203915_at	2.79e-03	1.85e-03	-3.131.517
215411_s_at	2.79e-03	1.85e-03	-3.131.391
212032_s_at	2.79e-03	1.85e-03	3.131.338
202646_s_at	2.79e-03	1.86e-03	3.131.259
217001_x_at	2.79e-03	1.86e-03	-3.131.044
219740_at	2.80e-03	1.86e-03	312.992
221320_at	2.81e-03	1.87e-03	-3.129.067
207992_s_at	2.82e-03	1.87e-03	3.128.348
207287_at	2.82e-03	1.88e-03	-3.127.881
220297_at	2.82e-03	1.88e-03	-3.127.542
215191_at	2.83e-03	1.88e-03	-3.126.969
211077_s_at	2.83e-03	1.88e-03	-3.126.877
209088_s_at	2.83e-03	1.88e-03	-3.126.847
219542_at	2.84e-03	1.89e-03	-312.547
217877_s_at	2.85e-03	1.90e-03	-3.124.554
201106_at	2.85e-03	1.90e-03	-3.124.524
201640_x_at	2.85e-03	1.90e-03	3.124.253
207316_at	2.85e-03	1.90e-03	-3.124.086
220825_s_at	2.86e-03	1.91e-03	-3.123.198

219669_at	2.86e-03	1.91e-03	-3.122.915
207803_s_at	2.87e-03	1.91e-03	-3.122.497
205497_at	2.87e-03	1.91e-03	-3.121.835
216079_at	2.88e-03	1.92e-03	-3.121.613
217684_at	2.88e-03	1.92e-03	-3.121.327
218376_s_at	2.89e-03	1.93e-03	3.119.729
209871_s_at	2.90e-03	1.93e-03	-3.118.995
206615_s_at	2.90e-03	1.93e-03	-3.118.578
210783_x_at	2.90e-03	1.94e-03	-3.118.508
208554_at	2.91e-03	1.94e-03	-3.118.142
216293_at	2.91e-03	1.94e-03	-3.117.654
220003_at	2.92e-03	1.94e-03	-3.117.322
221241_s_at	2.92e-03	1.95e-03	3.117.003
218928_s_at	2.92e-03	1.95e-03	3.116.952
213846_at	2.92e-03	1.95e-03	3.116.843
220713_at	2.92e-03	1.95e-03	-3.116.521
207234_at	2.92e-03	1.95e-03	-3.116.458
212604_at	2.93e-03	1.95e-03	-3.115.992
206534_at	2.93e-03	1.95e-03	-311.596
214862_x_at	2.93e-03	1.95e-03	3.115.657
200920_s_at	2.93e-03	1.96e-03	-3.115.381
221096_s_at	2.93e-03	1.96e-03	-3.115.275
202746_at	2.94e-03	1.96e-03	3.114.515
221076_at	2.94e-03	1.96e-03	-3.114.225
207139_at	2.94e-03	1.96e-03	-3.114.156
220686_s_at	2.94e-03	1.96e-03	-311.404
206817_x_at	2.96e-03	1.97e-03	-3.112.561
222384_at	2.96e-03	1.98e-03	-3.112.355
203266_s_at	2.97e-03	1.98e-03	-3.111.304
221000_s_at	2.97e-03	1.98e-03	3.111.211
221024_s_at	2.98e-03	1.99e-03	-311.037
210421_s_at	2.98e-03	1.99e-03	-3.110.138
201279_s_at	2.99e-03	2.00e-03	3.109.323
205818_at	2.99e-03	2.00e-03	-3.109.212
216643_at	2.99e-03	2.00e-03	-3.109.104
215810_x_at	2.99e-03	2.00e-03	-3.109.096
201538_s_at	3.00e-03	2.00e-03	3.108.576
204561_x_at	3.00e-03	2.00e-03	-3.107.939
209970_x_at	3.01e-03	2.01e-03	-3.107.464
203570_at	3.01e-03	2.01e-03	-3.107.094
201272_at	3.01e-03	2.01e-03	3.106.915
201057_s_at	3.01e-03	2.01e-03	3.106.687
221316_at	3.01e-03	2.01e-03	-3.106.636
221673_s_at	3.01e-03	2.01e-03	3.106.628

216819_at	3.02e-03	2.01e-03	-31.065
222278_at	3.02e-03	2.01e-03	-3.106.459
221801_x_at	3.02e-03	2.02e-03	-3.106.295
215459_at	3.02e-03	2.02e-03	-3.105.866
206769_at	3.02e-03	2.02e-03	-3.105.734
216106_at	3.03e-03	2.02e-03	-3.105.411
215013_s_at	3.03e-03	2.02e-03	-3.104.888
217009_at	3.03e-03	2.03e-03	-3.104.834
222146_s_at	3.04e-03	2.03e-03	-3.104.326
221530_s_at	3.04e-03	2.03e-03	3.103.987
203836_s_at	3.04e-03	2.03e-03	-3.103.985
212991_at	3.04e-03	2.03e-03	3.103.834
203240_at	3.04e-03	2.03e-03	310.368
207987_s_at	3.04e-03	2.03e-03	-3.103.644
215114_at	3.04e-03	2.03e-03	-3.103.379
210251_s_at	3.05e-03	2.04e-03	-3.102.806
221612_at	3.05e-03	2.04e-03	-3.102.383
212897_at	3.06e-03	2.04e-03	3.102.207
205939_at	3.06e-03	2.04e-03	-3.102.138
203523_at	3.06e-03	2.04e-03	-3.101.982
209498_at	3.06e-03	2.04e-03	-310.194
217624_at	3.06e-03	2.05e-03	-3.101.363
208927_at	3.07e-03	2.05e-03	3.100.945
216474_x_at	3.07e-03	2.05e-03	-3.100.452
206816_s_at	3.07e-03	2.06e-03	-3.100.174
203950_s_at	3.08e-03	2.06e-03	-309.981
206182_at	3.08e-03	2.06e-03	3.099.773
206270_at	3.09e-03	2.07e-03	-3.098.885
213636_at	3.09e-03	2.07e-03	-3.098.496
211517_s_at	3.11e-03	2.08e-03	-3.096.343
218790_s_at	3.11e-03	2.08e-03	-3.096.273
207495_at	3.11e-03	2.08e-03	3.096.185
AFFX-r2-Bs-dap-3_at	3.12e-03	2.09e-03	-309.596
209137_s_at	3.12e-03	2.09e-03	-3.095.311
212035_s_at	3.13e-03	2.10e-03	3.094.209
207377_at	3.13e-03	2.10e-03	-3.094.136
218215_s_at	3.13e-03	2.10e-03	3.094.047
32259_at	3.13e-03	2.10e-03	3.094.042
219761_at	3.14e-03	2.10e-03	-3.093.682
221767_x_at	3.14e-03	2.10e-03	3.093.553
216208_s_at	3.14e-03	2.10e-03	-3.093.225
215031_x_at	3.15e-03	2.11e-03	3.092.555
216783_at	3.16e-03	2.12e-03	-3.091.642
209700_x_at	3.16e-03	2.12e-03	-3.091.526

218727_at	3.16e-03	2.12e-03	3.091.274
222086_s_at	3.16e-03	2.12e-03	-3.090.979
218814_s_at	3.17e-03	2.12e-03	-3.090.475
215815_at	3.17e-03	2.12e-03	-309.041
220692_at	3.17e-03	2.12e-03	3.090.363
215338_s_at	3.17e-03	2.13e-03	-3.090.136
211084_x_at	3.17e-03	2.13e-03	3.089.981
220982_s_at	3.17e-03	2.13e-03	-3.089.908
222058_at	3.18e-03	2.13e-03	-3.089.432
205242_at	3.19e-03	2.13e-03	3.088.854
220271_x_at	3.19e-03	2.14e-03	-308.837
201309_x_at	3.20e-03	2.14e-03	3.087.715
217226_s_at	3.20e-03	2.15e-03	3.087.225
204401_at	3.21e-03	2.15e-03	-3.086.725
211751_at	3.21e-03	2.15e-03	-3.086.302
216675_at	3.21e-03	2.15e-03	3.086.085
216555_at	3.22e-03	2.16e-03	3.085.758
202884_s_at	3.22e-03	2.16e-03	-3.085.519
44065_at	3.22e-03	2.16e-03	3.085.052
214547_at	3.22e-03	2.16e-03	-3.084.944
208902_s_at	3.23e-03	2.17e-03	-3.084.573
206154_at	3.25e-03	2.18e-03	-3.082.711
202711_at	3.25e-03	2.18e-03	-3.082.419
208494_at	3.26e-03	2.19e-03	-3.081.584
211973_at	3.27e-03	2.19e-03	3.080.836
219383_at	3.27e-03	2.19e-03	3.080.832
218433_at	3.27e-03	2.19e-03	3.080.478
207454_at	3.27e-03	2.20e-03	-3.080.382
207579_at	3.27e-03	2.20e-03	-308.019
215010_s_at	3.28e-03	2.20e-03	-307.981
202905_x_at	3.28e-03	2.20e-03	3.079.627
206900_x_at	3.29e-03	2.21e-03	3.078.969
219932_at	3.29e-03	2.21e-03	-3.078.208
205631_at	3.29e-03	2.21e-03	3.078.086
207674_at	3.30e-03	2.21e-03	-3.077.694
206988_at	3.30e-03	2.22e-03	3.077.638
207212_at	3.30e-03	2.22e-03	-3.077.395
211257_x_at	3.31e-03	2.22e-03	-3.076.275
211151_x_at	3.31e-03	2.23e-03	-3.076.166
209870_s_at	3.32e-03	2.23e-03	-3.075.812
219770_at	3.32e-03	2.23e-03	307.567
203691_at	3.33e-03	2.24e-03	3.074.324
220539_at	3.33e-03	2.24e-03	-3.074.288
213780_at	3.34e-03	2.24e-03	-3.073.824

221915_s_at	3.34e-03	2.25e-03	3.073.493
221916_at	3.34e-03	2.25e-03	-3.073.432
221360_s_at	3.35e-03	2.25e-03	-3.072.811
210704_at	3.35e-03	2.25e-03	-3.072.645
205423_at	3.35e-03	2.25e-03	3.072.447
216684_s_at	3.35e-03	2.25e-03	-3.072.438
202274_at	3.36e-03	2.25e-03	3.072.209
215409_at	3.37e-03	2.27e-03	-3.070.547
209038_s_at	3.38e-03	2.27e-03	-3.070.225
212728_at	3.38e-03	2.27e-03	-307.013
205691_at	3.38e-03	2.27e-03	3.069.596
216367_at	3.38e-03	2.28e-03	-3.069.475
212495_at	3.38e-03	2.28e-03	-3.069.423
216586_at	3.39e-03	2.28e-03	-3.068.795
201596_x_at	3.39e-03	2.28e-03	3.068.446
210304_at	3.40e-03	2.29e-03	-3.067.632
221135_s_at	3.41e-03	2.29e-03	-3.067.422
219098_at	3.41e-03	2.29e-03	3.067.157
202827_s_at	3.41e-03	2.29e-03	-3.066.898
201223_s_at	3.41e-03	2.29e-03	3.066.896
220498_at	3.41e-03	2.30e-03	-3.066.757
208146_s_at	3.41e-03	2.30e-03	3.066.714
214610_at	3.41e-03	2.30e-03	-3.066.535
221466_at	3.42e-03	2.30e-03	-306.632
208401_s_at	3.42e-03	2.30e-03	-3.066.238
218455_at	3.42e-03	2.30e-03	3.065.752
213369_at	3.42e-03	2.30e-03	-3.065.628
215792_s_at	3.43e-03	2.31e-03	-3.065.278
205620_at	3.43e-03	2.31e-03	-3.065.037
212223_at	3.44e-03	2.32e-03	-3.064.112
209241_x_at	3.44e-03	2.32e-03	-3.064.063
219729_at	3.45e-03	2.32e-03	-3.062.933
204721_s_at	3.45e-03	2.32e-03	-3.062.895
212611_at	3.47e-03	2.33e-03	-3.061.585
219249_s_at	3.47e-03	2.34e-03	3.061.454
201053_s_at	3.47e-03	2.34e-03	3.060.997
221896_s_at	3.48e-03	2.34e-03	-3.060.706
218794_s_at	3.48e-03	2.34e-03	-3.060.561
208690_s_at	3.48e-03	2.34e-03	3.060.455
202028_s_at	3.48e-03	2.34e-03	3.060.374
204961_s_at	3.48e-03	2.34e-03	-3.060.251
216354_at	3.48e-03	2.35e-03	-3.060.217
202318_s_at	3.49e-03	2.35e-03	3.059.768
219477_s_at	3.49e-03	2.35e-03	3.059.243

210111_s_at	3.50e-03	2.36e-03	-3.058.843
213207_s_at	3.50e-03	2.36e-03	3.058.625
217138_x_at	3.50e-03	2.36e-03	-3.058.205
219967_at	3.51e-03	2.36e-03	-3.057.686
220970_s_at	3.51e-03	2.37e-03	-3.057.613
215574_at	3.51e-03	2.37e-03	-3.057.505
209057_x_at	3.51e-03	2.37e-03	-3.057.461
204187_at	3.52e-03	2.37e-03	-3.056.881
209749_s_at	3.53e-03	2.38e-03	-3.055.939
215739_s_at	3.53e-03	2.38e-03	305.588
214880_x_at	3.53e-03	2.38e-03	-3.055.736
205466_s_at	3.54e-03	2.39e-03	-3.054.833
205571_at	3.54e-03	2.39e-03	3.054.591
210392_x_at	3.54e-03	2.39e-03	-3.054.473
205916_at	3.55e-03	2.39e-03	-3.054.191
215567_at	3.56e-03	2.40e-03	-3.053.088
214631_at	3.56e-03	2.40e-03	3.053.007
208685_x_at	3.56e-03	2.40e-03	-3.052.919
202546_at	3.56e-03	2.40e-03	-3.052.659
216349_at	3.57e-03	2.41e-03	-3.051.953
206719_at	3.58e-03	2.42e-03	-3.050.993
214648_at	3.58e-03	2.42e-03	-3.050.936
201178_at	3.59e-03	2.42e-03	-305.071
204778_x_at	3.59e-03	2.42e-03	-3.050.429
208159_x_at	3.59e-03	2.42e-03	3.050.206
219520_s_at	3.60e-03	2.43e-03	3.049.692
209453_at	3.60e-03	2.43e-03	-3.049.167
208971_at	3.60e-03	2.43e-03	-3.048.941
222239_s_at	3.61e-03	2.44e-03	-3.048.403
220694_at	3.62e-03	2.44e-03	3.047.809
219435_at	3.62e-03	2.44e-03	-304.757
210972_x_at	3.62e-03	2.45e-03	-3.047.309
207134_x_at	3.63e-03	2.45e-03	-3.046.442
213622_at	3.64e-03	2.46e-03	3.045.559
203824_at	3.64e-03	2.46e-03	304.548
204954_s_at	3.64e-03	2.46e-03	-3.045.387
205130_at	3.64e-03	2.46e-03	-3.045.333
217887_s_at	3.64e-03	2.46e-03	3.045.325
206950_at	3.65e-03	2.46e-03	-3.045.097
201282_at	3.66e-03	2.47e-03	3.044.314
204572_s_at	3.66e-03	2.47e-03	-3.043.835
219155_at	3.66e-03	2.47e-03	3.043.751
AFFX-M27830_3_at	3.66e-03	2.48e-03	-3.043.604
217290_at	3.66e-03	2.48e-03	-3.043.526

212876_at	3.67e-03	2.48e-03	3.043.482
207073_at	3.67e-03	2.48e-03	-3.042.867
222255_at	3.67e-03	2.48e-03	-3.042.683
208786_s_at	3.68e-03	2.48e-03	3.042.562
209142_s_at	3.68e-03	2.49e-03	3.042.425
207135_at	3.69e-03	2.49e-03	-3.041.696
209983_s_at	3.69e-03	2.49e-03	-3.041.479
209183_s_at	3.69e-03	2.49e-03	3.041.392
221760_at	3.69e-03	2.50e-03	-3.041.137
214917_at	3.70e-03	2.50e-03	-3.040.767
205674_x_at	3.70e-03	2.50e-03	-3.040.716
220660_at	3.70e-03	2.50e-03	-3.040.376
213225_at	3.70e-03	2.50e-03	3.040.083
52255_s_at	3.70e-03	2.51e-03	3.039.957
208399_s_at	3.71e-03	2.51e-03	-3.039.504
210255_at	3.71e-03	2.51e-03	-3.039.471
208047_s_at	3.71e-03	2.51e-03	3.039.156
216960_s_at	3.72e-03	2.52e-03	3.038.426
219744_at	3.73e-03	2.52e-03	-3.038.074
219974_x_at	3.74e-03	2.53e-03	-3.037.078
208537_at	3.74e-03	2.53e-03	-3.036.812
206059_at	3.74e-03	2.53e-03	3.036.598
211690_at	3.74e-03	2.53e-03	-3.036.451
203007_x_at	3.75e-03	2.54e-03	3.036.013
207901_at	3.76e-03	2.54e-03	-3.035.132
216561_x_at	3.77e-03	2.55e-03	-3.034.412
202022_at	3.78e-03	2.56e-03	3.033.457
205435_s_at	3.78e-03	2.56e-03	-303.333
202458_at	3.78e-03	2.56e-03	3.033.303
206387_at	3.79e-03	2.57e-03	-3.032.533
213157_s_at	3.79e-03	2.57e-03	-3.032.196
206161_s_at	3.80e-03	2.57e-03	-3.032.065
219437_s_at	3.81e-03	2.58e-03	-3.031.268
217302_at	3.82e-03	2.59e-03	-3.029.896
216833_x_at	3.83e-03	2.59e-03	-3.029.278
221516_s_at	3.83e-03	2.59e-03	3.029.254
213534_s_at	3.83e-03	2.59e-03	3.029.242
206341_at	3.85e-03	2.61e-03	-3.027.786
32209_at	3.85e-03	2.61e-03	3.027.695
214718_at	3.86e-03	2.61e-03	3.027.029
205346_at	3.86e-03	2.62e-03	-302.665
217563_at	3.87e-03	2.62e-03	-3.026.242
215847_at	3.87e-03	2.62e-03	302.616
214104_at	3.87e-03	2.63e-03	3.025.498

216012_at	3.88e-03	2.63e-03	3.025.432
216730_at	3.88e-03	2.63e-03	-30.253
220985_s_at	3.88e-03	2.63e-03	3.025.072
200612_s_at	3.91e-03	2.65e-03	3.022.911
212434_at	3.91e-03	2.65e-03	-3.022.362
211196_at	3.92e-03	2.65e-03	-3.022.108
218203_at	3.93e-03	2.66e-03	3.021.244
209395_at	3.93e-03	2.67e-03	-302.075
218413_s_at	3.93e-03	2.67e-03	302.074
209185_s_at	3.93e-03	2.67e-03	-3.020.629
222293_at	3.94e-03	2.67e-03	-3.020.411
220766_at	3.94e-03	2.67e-03	-3.019.814
210976_s_at	3.95e-03	2.68e-03	3.019.578
33579_i_at	3.95e-03	2.68e-03	-3.019.459
216146_at	3.95e-03	2.68e-03	-3.019.459
202014_at	3.95e-03	2.68e-03	-3.019.062
216998_s_at	3.96e-03	2.68e-03	-3.018.749
211221_at	3.97e-03	2.69e-03	-3.017.572
220632_s_at	3.97e-03	2.69e-03	-301.757
213796_at	3.97e-03	2.70e-03	-3.017.435
204963_at	3.97e-03	2.70e-03	-3.017.179
218900_at	3.98e-03	2.70e-03	3.016.953
58367_s_at	3.98e-03	2.70e-03	-3.016.528
217004_s_at	3.99e-03	2.71e-03	-3.016.008
201889_at	3.99e-03	2.71e-03	3.015.842
208475_at	3.99e-03	2.71e-03	-3.015.819
212245_at	4.01e-03	2.72e-03	3.014.361
206037_at	4.02e-03	2.73e-03	3.013.763
202889_x_at	4.02e-03	2.73e-03	-3.013.637
218964_at	4.02e-03	2.73e-03	-3.013.419
203116_s_at	4.02e-03	2.73e-03	3.013.206
207052_at	4.02e-03	2.73e-03	3.013.107
210699_at	4.03e-03	2.73e-03	-3.012.919
210703_at	4.03e-03	2.74e-03	-3.012.822
205368_at	4.05e-03	2.75e-03	-3.010.825
217914_at	4.07e-03	2.76e-03	-3.009.667
218274_s_at	4.07e-03	2.76e-03	3.009.544
221399_at	4.08e-03	2.77e-03	-3.008.919
210190_at	4.08e-03	2.77e-03	-3.008.847
216443_at	4.08e-03	2.77e-03	-3.008.708
206137_at	4.08e-03	2.77e-03	-3.008.483
215532_x_at	4.09e-03	2.78e-03	3.008.108
206313_at	4.09e-03	2.78e-03	-3.007.801
213033_s_at	4.10e-03	2.78e-03	-3.007.407

221426_s_at	4.10e-03	2.79e-03	-3.006.783
218530_at	4.10e-03	2.79e-03	-3.006.672
209831_x_at	4.11e-03	2.79e-03	-3.006.395
221542_s_at	4.11e-03	2.79e-03	-3.006.388
207686_s_at	4.12e-03	2.80e-03	3.005.595
214156_at	4.12e-03	2.80e-03	-3.005.549
210676_x_at	4.14e-03	2.82e-03	-3.003.876
202609_at	4.16e-03	2.83e-03	3.002.749
211177_s_at	4.17e-03	2.83e-03	-3.002.013
214522_x_at	4.17e-03	2.83e-03	300.189
203777_s_at	4.17e-03	2.84e-03	3.001.615
218738_s_at	4.17e-03	2.84e-03	3.001.286
206676_at	4.18e-03	2.84e-03	-3.001.185
204948_s_at	4.18e-03	2.84e-03	-3.000.925
214746_s_at	4.18e-03	2.84e-03	3.000.765
221777_at	4.19e-03	2.85e-03	-3.000.248
209016_s_at	4.19e-03	2.85e-03	-3.000.145
215466_at	4.19e-03	2.85e-03	-2.999.767
219779_at	4.20e-03	2.86e-03	-2.999.431
207307_at	4.20e-03	2.86e-03	299.908
207485_x_at	4.20e-03	2.86e-03	-2.998.873
209778_at	4.21e-03	2.86e-03	2.998.685
205557_at	4.21e-03	2.86e-03	-2.998.637
216230_x_at	4.21e-03	2.87e-03	-2.998.448
215107_s_at	4.21e-03	2.87e-03	-2.998.146
217756_x_at	4.21e-03	2.87e-03	2.998.098
216877_at	4.21e-03	2.87e-03	-2.998.058
207335_x_at	4.22e-03	2.87e-03	2.997.819
56919_at	4.23e-03	2.88e-03	2.996.993
207186_s_at	4.23e-03	2.88e-03	2.996.747
219910_at	4.24e-03	2.89e-03	2.996.272
214924_s_at	4.24e-03	2.89e-03	2.996.253
209201_x_at	4.24e-03	2.89e-03	-2.996.226
213781_at	4.24e-03	2.89e-03	-2.995.901
209133_s_at	4.24e-03	2.89e-03	-2.995.803
221890_at	4.24e-03	2.89e-03	2.995.648
204308_s_at	4.24e-03	2.89e-03	2.995.547
212618_at	4.24e-03	2.89e-03	2.995.504
216703_at	4.24e-03	2.89e-03	-2.995.496
221339_at	4.25e-03	2.89e-03	-2.995.433
209390_at	4.25e-03	2.90e-03	299.486
205879_x_at	4.25e-03	2.90e-03	-2.994.785
205279_s_at	4.25e-03	2.90e-03	2.994.727
203679_at	4.25e-03	2.90e-03	-2.994.684

208128_x_at	4.25e-03	2.90e-03	-2.994.634
217329_x_at	4.25e-03	2.90e-03	2.994.616
211250_s_at	4.26e-03	2.90e-03	-2.994.534
217333_at	4.26e-03	2.90e-03	-2.994.297
214273_x_at	4.27e-03	2.91e-03	-2.993.556
222065_s_at	4.27e-03	2.91e-03	2.993.542
215285_s_at	4.28e-03	2.92e-03	2.993.039
207551_s_at	4.28e-03	2.92e-03	-2.992.616
207074_s_at	4.28e-03	2.92e-03	-2.992.414
207665_at	4.29e-03	2.92e-03	-2.992.127
215676_at	4.29e-03	2.93e-03	-2.991.728
205195_at	4.30e-03	2.93e-03	2.991.238
209872_s_at	4.30e-03	2.93e-03	-2.991.045
221950_at	4.30e-03	2.93e-03	-2.991.029
217652_at	4.30e-03	2.93e-03	-2.990.983
207065_at	4.31e-03	2.94e-03	-2.990.511
220175_s_at	4.31e-03	2.94e-03	2.990.229
204898_at	4.33e-03	2.96e-03	-2.988.657
204398_s_at	4.34e-03	2.96e-03	2.988.049
201430_s_at	4.35e-03	2.97e-03	-2.987.724
211654_x_at	4.35e-03	2.97e-03	-2.987.557
210067_at	4.35e-03	2.97e-03	-2.987.512
214851_at	4.36e-03	2.98e-03	-2.986.357
206525_at	4.37e-03	2.98e-03	-29.857
204552_at	4.37e-03	2.99e-03	2.985.522
211127_x_at	4.39e-03	3.00e-03	-2.984.301
215880_at	4.40e-03	3.00e-03	-2.983.926
214125_s_at	4.40e-03	3.00e-03	-298.384
221017_s_at	4.40e-03	3.01e-03	-2.983.532
211220_s_at	4.40e-03	3.01e-03	-2.983.398
221446_at	4.41e-03	3.01e-03	-2.983.002
215434_x_at	4.41e-03	3.01e-03	-2.982.671
203922_s_at	4.41e-03	3.02e-03	-2.982.537
208364_at	4.42e-03	3.02e-03	-2.982.276
201974_s_at	4.42e-03	3.02e-03	-2.982.043
221140_s_at	4.42e-03	3.02e-03	-2.981.932
211719_x_at	4.42e-03	3.02e-03	-2.981.921
217399_s_at	4.42e-03	3.02e-03	-2.981.906
221116_at	4.42e-03	3.02e-03	-2.981.722
208452_x_at	4.43e-03	3.03e-03	-2.981.449
218553_s_at	4.45e-03	3.04e-03	-2.979.773
221282_x_at	4.45e-03	3.04e-03	-297.968
203899_s_at	4.45e-03	3.05e-03	-2.979.454
213683_at	4.46e-03	3.05e-03	2.978.944

202839_s_at	4.47e-03	3.06e-03	297.841
214757_at	4.47e-03	3.06e-03	-2.978.078
207283_at	4.49e-03	3.07e-03	2.977.216
217931_at	4.49e-03	3.07e-03	-2.977.185
215976_at	4.49e-03	3.07e-03	-2.977.011
212450_at	4.49e-03	3.07e-03	-2.976.647
204321_at	4.51e-03	3.08e-03	297.577
221164_x_at	4.51e-03	3.09e-03	-2.975.087
AFFX-LysX-5_at	4.52e-03	3.09e-03	-2.974.598
208756_at	4.53e-03	3.10e-03	2.973.906
212829_at	4.53e-03	3.10e-03	2.973.751
216400_at	4.53e-03	3.10e-03	-2.973.651
220860_at	4.54e-03	3.10e-03	-2.973.588
221856_s_at	4.55e-03	3.11e-03	2.972.696
219092_s_at	4.55e-03	3.12e-03	-2.972.264
220285_at	4.57e-03	3.13e-03	2.971.203
221358_at	4.58e-03	3.13e-03	-2.970.509
214255_at	4.58e-03	3.13e-03	-2.970.432
213732_at	4.59e-03	3.14e-03	-296.991
213443_at	4.60e-03	3.15e-03	-2.969.309
216957_at	4.60e-03	3.15e-03	-2.969.213
210584_s_at	4.60e-03	3.15e-03	-2.969.032
218524_at	4.60e-03	3.15e-03	2.968.771
210449_x_at	4.60e-03	3.15e-03	2.968.576
209021_x_at	4.61e-03	3.16e-03	2.968.364
205934_at	4.61e-03	3.16e-03	2.968.248
202400_s_at	4.61e-03	3.16e-03	2.967.844
218629_at	4.62e-03	3.17e-03	2.967.304
212096_s_at	4.62e-03	3.17e-03	-2.967.165
209444_at	4.63e-03	3.17e-03	-2.967.013
208380_at	4.63e-03	3.17e-03	-2.966.555
210248_at	4.65e-03	3.18e-03	-2.965.551
208400_at	4.65e-03	3.19e-03	-2.965.328
219517_at	4.66e-03	3.19e-03	2.964.666
211584_s_at	4.67e-03	3.20e-03	-2.964.271
205640_at	4.67e-03	3.20e-03	2.963.714
215903_s_at	4.68e-03	3.21e-03	296.297
216016_at	4.69e-03	3.22e-03	-2.962.431
210617_at	4.72e-03	3.23e-03	-2.960.665
213060_s_at	4.72e-03	3.24e-03	-2.960.242
217201_at	4.73e-03	3.24e-03	-2.959.714
216753_at	4.73e-03	3.24e-03	-2.959.701
222242_s_at	4.73e-03	3.24e-03	-2.959.576
215827_x_at	4.74e-03	3.25e-03	-2.958.896

200904_at	4.76e-03	3.26e-03	-2.957.967
212698_s_at	4.76e-03	3.26e-03	2.957.965
215572_at	4.76e-03	3.26e-03	-2.957.792
217391_x_at	4.77e-03	3.27e-03	2.957.319
215401_at	4.78e-03	3.28e-03	-2.956.557
205021_s_at	4.78e-03	3.28e-03	-2.956.495
214635_at	4.78e-03	3.28e-03	-2.956.433
220303_at	4.78e-03	3.28e-03	-2.956.154
203838_s_at	4.79e-03	3.28e-03	-2.955.715
220406_at	4.79e-03	3.29e-03	-2.955.649
216296_at	4.80e-03	3.29e-03	-2.955.008
220875_at	4.81e-03	3.30e-03	-2.954.382
202912_at	4.83e-03	3.32e-03	2.952.619
215787_at	4.84e-03	3.32e-03	-2.952.259
218409_s_at	4.85e-03	3.33e-03	2.951.689
220563_s_at	4.85e-03	3.33e-03	-295.125
210593_at	4.87e-03	3.34e-03	-2.950.311
220680_at	4.87e-03	3.34e-03	-2.950.189
34260_at	4.91e-03	3.37e-03	-2.947.817
214520_at	4.91e-03	3.37e-03	-294.771
203898_at	4.91e-03	3.37e-03	2.947.337
215895_x_at	4.92e-03	3.37e-03	-2.947.186
220443_s_at	4.93e-03	3.39e-03	-2.946.001
202131_s_at	4.94e-03	3.39e-03	-2.945.671
215342_s_at	4.95e-03	3.40e-03	-29.452
211061_s_at	4.95e-03	3.40e-03	2.945.152
216080_s_at	4.95e-03	3.40e-03	2.945.145
217059_at	4.95e-03	3.40e-03	-2.945.035
203024_s_at	4.95e-03	3.40e-03	2.944.702
204971_at	4.96e-03	3.41e-03	2.944.102
209134_s_at	4.96e-03	3.41e-03	2.943.849
209232_s_at	4.97e-03	3.41e-03	2.943.774
206466_at	4.98e-03	3.42e-03	-2.942.668
209710_at	4.98e-03	3.42e-03	-2.942.599
210264_at	4.99e-03	3.43e-03	2.942.431
210662_at	4.99e-03	3.43e-03	-2.942.019
211949_s_at	4.99e-03	3.43e-03	294.195
202741_at	4.99e-03	3.43e-03	-2.941.884
208732_at	5.00e-03	3.43e-03	2.941.746
220567_at	5.00e-03	3.44e-03	-2.941.621
205136_s_at	5.02e-03	3.45e-03	294.033
211366_x_at	5.03e-03	3.45e-03	-2.939.815
212727_at	5.04e-03	3.47e-03	-2.938.783
215367_at	5.05e-03	3.47e-03	-2.938.413

216540_at	5.06e-03	3.48e-03	-2.937.324
206051_at	5.08e-03	3.49e-03	-2.936.563
202473_x_at	5.08e-03	3.49e-03	-2.936.554
216136_at	5.10e-03	3.51e-03	-2.935.167
211731_x_at	5.11e-03	3.52e-03	2.934.112
212409_s_at	5.12e-03	3.52e-03	2.933.707
213718_at	5.14e-03	3.53e-03	-2.932.672
210065_s_at	5.15e-03	3.54e-03	-2.931.954
208274_at	5.15e-03	3.54e-03	-2.931.664
205577_at	5.15e-03	3.54e-03	-293.166
214959_s_at	5.16e-03	3.55e-03	2.931
35626_at	5.19e-03	3.57e-03	2.929.351
219504_s_at	5.20e-03	3.58e-03	-292.857
203219_s_at	5.21e-03	3.58e-03	292.832
216067_at	5.21e-03	3.59e-03	-2.927.861
217155_at	5.22e-03	3.59e-03	-2.927.398
206389_s_at	5.22e-03	3.60e-03	-2.927.114
218169_at	5.23e-03	3.60e-03	2.926.834
202058_s_at	5.24e-03	3.61e-03	2.926.213
209900_s_at	5.25e-03	3.61e-03	-2.925.746
211426_x_at	5.26e-03	3.62e-03	-292.503
210459_at	5.26e-03	3.62e-03	-2.925.011
211272_s_at	5.26e-03	3.62e-03	-2.924.669
201436_at	5.26e-03	3.62e-03	2.924.657
201042_at	5.30e-03	3.65e-03	-2.922.505
210604_at	5.30e-03	3.65e-03	-2.922.478
210579_s_at	5.30e-03	3.65e-03	-292.223
214348_at	5.31e-03	3.65e-03	-2.922.004
213116_at	5.31e-03	3.66e-03	2.921.839
209986_at	5.31e-03	3.66e-03	-2.921.794
221204_s_at	5.32e-03	3.66e-03	-2.921.295
211085_s_at	5.32e-03	3.66e-03	-2.921.175
209730_at	5.32e-03	3.66e-03	-2.921.158
206960_at	5.32e-03	3.67e-03	-2.920.985
210693_at	5.33e-03	3.67e-03	-2.920.368
215839_at	5.33e-03	3.68e-03	-2.920.222
208513_at	5.34e-03	3.68e-03	-2.919.776
219563_at	5.34e-03	3.68e-03	-2.919.669
212344_at	5.34e-03	3.68e-03	2.919.476
219607_s_at	5.34e-03	3.68e-03	-2.919.466
216053_x_at	5.35e-03	3.69e-03	-2.919.074
216573_at	5.35e-03	3.69e-03	-2.918.969
212497_at	5.35e-03	3.69e-03	2.918.948
216039_at	5.35e-03	3.69e-03	2.918.898

205782_at	5.36e-03	3.70e-03	-2.918.477
212283_at	5.36e-03	3.70e-03	-2.918.448
206757_at	5.36e-03	3.70e-03	-2.918.184
215433_at	5.37e-03	3.70e-03	-2.917.897
207834_at	5.38e-03	3.71e-03	-2.917.267
220088_at	5.39e-03	3.71e-03	-2.916.796
207127_s_at	5.41e-03	3.73e-03	2.915.367
207870_at	5.41e-03	3.73e-03	-291.533
219321_at	5.42e-03	3.74e-03	2.914.572
214481_at	5.42e-03	3.74e-03	2.914.473
219472_at	5.43e-03	3.75e-03	-2.914.046
222313_at	5.44e-03	3.75e-03	-2.913.575
213618_at	5.44e-03	3.76e-03	-2.913.361
213775_x_at	5.44e-03	3.76e-03	-2.913.288
207412_x_at	5.44e-03	3.76e-03	-2.913.258
212743_at	5.45e-03	3.76e-03	-2.913.071
212557_at	5.45e-03	3.76e-03	2.913.009
216217_at	5.45e-03	3.76e-03	-2.912.581
216817_s_at	5.46e-03	3.77e-03	-291.193
220249_at	5.48e-03	3.78e-03	-2.911.036
216116_at	5.49e-03	3.79e-03	-2.910.234
213195_at	5.51e-03	3.81e-03	-2.909.015
211108_s_at	5.52e-03	3.81e-03	-2.908.694
215900_at	5.52e-03	3.81e-03	2.908.693
211353_at	5.52e-03	3.81e-03	-2.908.648
201210_at	5.52e-03	3.81e-03	2.908.571
203285_s_at	5.53e-03	3.82e-03	2.907.948
202933_s_at	5.55e-03	3.84e-03	2.906.512
203587_at	5.55e-03	3.84e-03	-2.906.481
214333_x_at	5.56e-03	3.84e-03	2.906.352
207868_at	5.57e-03	3.85e-03	-2.905.663
205214_at	5.57e-03	3.85e-03	2.905.329
214498_at	5.58e-03	3.86e-03	-2.904.985
203420_at	5.60e-03	3.87e-03	-290.378
210686_x_at	5.61e-03	3.87e-03	-2.903.425
207732_s_at	5.61e-03	3.88e-03	-2.903.279
218204_s_at	5.61e-03	3.88e-03	2.903.092
217246_s_at	5.61e-03	3.88e-03	-2.903.038
219634_at	5.62e-03	3.88e-03	2.902.792
209376_x_at	5.62e-03	3.89e-03	-2.902.313
207924_x_at	5.63e-03	3.89e-03	-2.901.805
215287_at	5.64e-03	3.90e-03	290.143
211516_at	5.66e-03	3.92e-03	-2.899.914
212324_s_at	5.67e-03	3.92e-03	-2.899.475

209396_s_at	5.68e-03	3.93e-03	-2.899.221
208356_x_at	5.69e-03	3.94e-03	-2.898.449
213040_s_at	5.71e-03	3.95e-03	-2.897.282
205783_at	5.72e-03	3.96e-03	-2.896.494
215081_at	5.74e-03	3.97e-03	-2.895.608
217847_s_at	5.75e-03	3.97e-03	-289.526
207449_s_at	5.75e-03	3.98e-03	-2.895.011
207166_at	5.78e-03	4.00e-03	-2.893.052
207982_at	5.79e-03	4.00e-03	-2.892.901
220471_s_at	5.79e-03	4.00e-03	-2.892.812
203833_s_at	5.79e-03	4.01e-03	-2.892.536
209854_s_at	5.79e-03	4.01e-03	-2.892.417
207921_x_at	5.80e-03	4.01e-03	-2.892.083
221783_at	5.80e-03	4.02e-03	2.891.783
205603_s_at	5.82e-03	4.03e-03	2.890.856
205156_s_at	5.82e-03	4.03e-03	289.066
219830_at	5.83e-03	4.04e-03	-2.890.419
214382_at	5.83e-03	4.04e-03	-2.890.377
200635_s_at	5.84e-03	4.05e-03	2.889.585
213985_s_at	5.85e-03	4.05e-03	-2.889.141
220236_at	5.85e-03	4.05e-03	-2.888.931
217000_at	5.87e-03	4.06e-03	-2.888.064
205613_at	5.88e-03	4.07e-03	-2.887.372
219800_s_at	5.88e-03	4.07e-03	-2.887.331
218340_s_at	5.88e-03	4.07e-03	2.887.302
55872_at	5.88e-03	4.08e-03	2.887.217
222003_s_at	5.89e-03	4.08e-03	-288.685
202151_s_at	5.90e-03	4.08e-03	2.886.505
219042_at	5.90e-03	4.09e-03	-2.886.338
202343_x_at	5.90e-03	4.09e-03	2.886.303
209620_s_at	5.90e-03	4.09e-03	-2.886.119
204404_at	5.90e-03	4.09e-03	2.885.942
217862_at	5.91e-03	4.09e-03	2.885.737
203871_at	5.93e-03	4.11e-03	2.884.386
214542_x_at	5.94e-03	4.11e-03	2.884.199
221565_s_at	5.95e-03	4.13e-03	-2.883.305
211576_s_at	5.96e-03	4.13e-03	2.883.117
206221_at	5.97e-03	4.14e-03	-2.882.301
214492_at	5.97e-03	4.14e-03	-2.882.153
206128_at	5.97e-03	4.14e-03	-2.882.075
207060_at	5.98e-03	4.14e-03	-2.881.856
203381_s_at	5.98e-03	4.15e-03	-288.163
203124_s_at	6.01e-03	4.17e-03	2.879.788
206448_at	6.03e-03	4.18e-03	-2.879.175

204206_at	6.05e-03	4.20e-03	-28.779
207838_x_at	6.06e-03	4.20e-03	2.877.553
211265_at	6.07e-03	4.21e-03	-2.876.634
210963_s_at	6.08e-03	4.22e-03	-2.876.303
204741_at	6.08e-03	4.22e-03	-2.876.044
211883_x_at	6.09e-03	4.23e-03	-2.875.543
205192_at	6.10e-03	4.23e-03	-2.875.265
213089_at	6.10e-03	4.23e-03	2.875.171
204414_at	6.10e-03	4.23e-03	-2.875.042
219036_at	6.11e-03	4.24e-03	-2.874.767
214499_s_at	6.11e-03	4.24e-03	2.874.503
215154_at	6.11e-03	4.24e-03	-2.874.373
202472_at	6.12e-03	4.25e-03	2.873.767
219614_s_at	6.13e-03	4.25e-03	-2.873.524
217995_at	6.13e-03	4.26e-03	287.317
221106_at	6.15e-03	4.27e-03	-2.872.532
213345_at	6.16e-03	4.27e-03	-2.871.952
209621_s_at	6.16e-03	4.28e-03	2.871.633
213299_at	6.17e-03	4.28e-03	-2.871.212
207064_s_at	6.17e-03	4.29e-03	-2.871.048
218537_at	6.18e-03	4.29e-03	2.870.572
216468_s_at	6.19e-03	4.30e-03	2.869.929
203174_s_at	6.19e-03	4.30e-03	-2.869.826
206780_at	6.20e-03	4.31e-03	-2.869.564
209866_s_at	6.20e-03	4.31e-03	-2.869.457
217697_at	6.21e-03	4.31e-03	-2.869.049
208317_at	6.22e-03	4.32e-03	-2.868.641
209747_at	6.22e-03	4.32e-03	-2.868.641
222169_x_at	6.23e-03	4.33e-03	-2.867.671
206494_s_at	6.25e-03	4.34e-03	-2.866.721
208528_x_at	6.27e-03	4.36e-03	-2.865.761
206225_at	6.27e-03	4.36e-03	-2.865.734
212455_at	6.28e-03	4.36e-03	-2.865.319
222213_x_at	6.28e-03	4.37e-03	-2.864.931
216645_at	6.29e-03	4.37e-03	-2.864.667
219871_at	6.30e-03	4.38e-03	-2.863.918
207267_s_at	6.33e-03	4.40e-03	2.862.697
214954_at	6.33e-03	4.40e-03	-2.862.407
209973_at	6.34e-03	4.41e-03	2.861.966
201367_s_at	6.36e-03	4.42e-03	-2.860.974
203489_at	6.37e-03	4.43e-03	2.860.572
210061_at	6.37e-03	4.43e-03	-2.860.338
202593_s_at	6.38e-03	4.43e-03	28.601
219490_s_at	6.38e-03	4.44e-03	2.859.811

216759_at	6.39e-03	4.45e-03	-2.859.265
205578_at	6.39e-03	4.45e-03	-285.906
207245_at	6.40e-03	4.45e-03	-2.858.764
206736_x_at	6.40e-03	4.45e-03	-2.858.684
211314_at	6.42e-03	4.47e-03	-2.857.721
219822_at	6.42e-03	4.47e-03	-285.764
213250_at	6.42e-03	4.47e-03	-2.857.621
217479_at	6.42e-03	4.47e-03	-285.748
216552_x_at	6.44e-03	4.48e-03	-2.856.623
215916_at	6.45e-03	4.49e-03	-2.856.347
221757_at	6.45e-03	4.49e-03	2.856.302
218742_at	6.45e-03	4.49e-03	-2.856.059
207016_s_at	6.46e-03	4.49e-03	2.855.783
64432_at	6.46e-03	4.50e-03	2.855.306
218290_at	6.48e-03	4.51e-03	2.854.298
218760_at	6.50e-03	4.52e-03	-2.853.604
216937_s_at	6.50e-03	4.53e-03	-2.853.401
208240_s_at	6.51e-03	4.53e-03	-285.296
219545_at	6.53e-03	4.55e-03	2.851.991
41553_at	6.53e-03	4.55e-03	-2.851.893
212025_s_at	6.53e-03	4.55e-03	2.851.811
35147_at	6.54e-03	4.55e-03	2.851.414
215929_at	6.57e-03	4.58e-03	-2.849.902
206617_s_at	6.57e-03	4.58e-03	284.962
218975_at	6.58e-03	4.58e-03	2.849.381
214684_at	6.58e-03	4.58e-03	284.933
210413_x_at	6.58e-03	4.59e-03	-2.849.201
203247_s_at	6.59e-03	4.59e-03	-284.865
207013_s_at	6.61e-03	4.61e-03	-2.847.812
220234_at	6.61e-03	4.61e-03	-2.847.553
212350_at	6.63e-03	4.62e-03	2.846.998
216215_s_at	6.64e-03	4.63e-03	2.846.401
210683_at	6.65e-03	4.63e-03	2.845.908
216165_at	6.65e-03	4.63e-03	2.845.893
200054_at	6.66e-03	4.64e-03	2.845.431
209943_at	6.66e-03	4.64e-03	2.845.395
209800_at	6.66e-03	4.64e-03	-2.845.359
206087_x_at	6.67e-03	4.65e-03	2.844.474
214511_x_at	6.68e-03	4.65e-03	2.844.395
213855_s_at	6.68e-03	4.65e-03	-2.844.371
206866_at	6.68e-03	4.66e-03	-2.844.051
205032_at	6.68e-03	4.66e-03	2.843.928
211436_at	6.68e-03	4.66e-03	-2.843.926
212321_at	6.68e-03	4.66e-03	2.843.885

207087_x_at	6.70e-03	4.67e-03	-284.325
217906_at	6.70e-03	4.68e-03	-2.842.842
201296_s_at	6.71e-03	4.68e-03	-2.842.398
207753_at	6.72e-03	4.69e-03	284.219
217728_at	6.72e-03	4.69e-03	284.203
209255_at	6.75e-03	4.71e-03	-2.840.782
220102_at	6.75e-03	4.71e-03	-2.840.438
206967_at	6.75e-03	4.71e-03	-2.840.361
212833_at	6.76e-03	4.71e-03	2.840.237
208299_at	6.76e-03	4.72e-03	-283.995
212024_x_at	6.79e-03	4.74e-03	283.868
220636_at	6.79e-03	4.74e-03	-2.838.289
208227_x_at	6.80e-03	4.75e-03	-2.837.773
210939_s_at	6.81e-03	4.75e-03	-2.837.536
210450_at	6.81e-03	4.76e-03	-283.737
206061_s_at	6.82e-03	4.76e-03	-2.836.766
216879_at	6.83e-03	4.77e-03	-2.836.625
204375_at	6.83e-03	4.77e-03	-2.836.478
215357_s_at	6.84e-03	4.77e-03	2.836.122
214546_s_at	6.84e-03	4.77e-03	-2.836.121
219333_s_at	6.85e-03	4.78e-03	2.835.686
216914_at	6.85e-03	4.79e-03	-2.835.321
212464_s_at	6.87e-03	4.80e-03	-2.834.378
206586_at	6.87e-03	4.80e-03	-2.834.265
206332_s_at	6.88e-03	4.81e-03	-283.378
221305_s_at	6.88e-03	4.81e-03	-2.833.705
221442_at	6.89e-03	4.81e-03	-2.833.509
201800_s_at	6.90e-03	4.82e-03	-2.833.059
214575_s_at	6.90e-03	4.82e-03	-2.832.975
204094_s_at	6.91e-03	4.83e-03	283.253
217550_at	6.93e-03	4.84e-03	2.831.674
207391_s_at	6.93e-03	4.84e-03	2.831.536
213159_at	6.94e-03	4.85e-03	283.096
208925_at	6.94e-03	4.85e-03	2.830.857
207565_s_at	6.96e-03	4.86e-03	2.830.175
220133_at	6.97e-03	4.87e-03	2.829.668
205228_at	6.97e-03	4.87e-03	-2.829.477
206328_at	6.98e-03	4.88e-03	-2.828.895
201861_s_at	6.99e-03	4.89e-03	-2.828.536
218958_at	7.00e-03	4.89e-03	-2.828.214
200680_x_at	7.00e-03	4.90e-03	2.827.942
208512_s_at	7.01e-03	4.90e-03	-2.827.708
204686_at	7.02e-03	4.91e-03	-2.827.141
215833_s_at	7.03e-03	4.92e-03	-2.826.409

207694_at	7.04e-03	4.92e-03	-2.826.121
200795_at	7.04e-03	4.92e-03	2.826.091
207533_at	7.06e-03	4.94e-03	-2.825.159
217345_at	7.07e-03	4.94e-03	-2.824.746
204761_at	7.08e-03	4.96e-03	-2.823.933
211025_x_at	7.10e-03	4.97e-03	2.823.084
211706_s_at	7.11e-03	4.98e-03	-2.822.481
214944_at	7.12e-03	4.98e-03	-2.822.312
205286_at	7.12e-03	4.99e-03	2.822.019
210600_s_at	7.12e-03	4.99e-03	-282.197
38766_at	7.13e-03	4.99e-03	-2.821.798
214938_x_at	7.13e-03	4.99e-03	2.821.686
216072_at	7.13e-03	4.99e-03	-2.821.648
221582_at	7.15e-03	5.00e-03	282.094
214458_at	7.15e-03	5.01e-03	2.820.676
221054_s_at	7.15e-03	5.01e-03	-2.820.552
216021_s_at	7.16e-03	5.01e-03	-2.820.229
65521_at	7.17e-03	5.02e-03	-2.819.764
209468_at	7.17e-03	5.02e-03	-2.819.573
213976_at	7.20e-03	5.04e-03	-2.818.162
219706_at	7.20e-03	5.05e-03	2.818.125
212392_s_at	7.22e-03	5.05e-03	-2.817.524
205437_at	7.22e-03	5.06e-03	2.817.407
217893_s_at	7.23e-03	5.07e-03	-2.816.749
213953_at	7.24e-03	5.07e-03	2.816.579
221690_s_at	7.24e-03	5.07e-03	2.816.311
219658_at	7.26e-03	5.09e-03	-2.815.416
215413_at	7.28e-03	5.10e-03	2.814.561
212314_at	7.28e-03	5.10e-03	2.814.483
202941_at	7.31e-03	5.12e-03	-2.813.215
218509_at	7.32e-03	5.13e-03	2.812.806
210274_at	7.33e-03	5.14e-03	2.812.127
215973_at	7.33e-03	5.14e-03	-2.811.996
209131_s_at	7.33e-03	5.14e-03	2.811.993
79005_at	7.33e-03	5.14e-03	2.811.979
213484_at	7.34e-03	5.15e-03	-281.148
219339_s_at	7.35e-03	5.15e-03	-281.126
221213_s_at	7.36e-03	5.16e-03	-2.810.776
215568_x_at	7.36e-03	5.16e-03	2.810.612
210495_x_at	7.38e-03	5.17e-03	-2.809.996
218120_s_at	7.39e-03	5.18e-03	-280.947
203037_s_at	7.39e-03	5.18e-03	-2.809.381
220399_at	7.39e-03	5.18e-03	2.809.295
219861_at	7.40e-03	5.19e-03	-2.808.971

221343_at	7.40e-03	5.19e-03	-2.808.894
209167_at	7.45e-03	5.23e-03	-2.806.654
201249_at	7.45e-03	5.23e-03	-2.806.393
210360_s_at	7.46e-03	5.24e-03	-2.805.892
218111_s_at	7.47e-03	5.24e-03	2.805.538
208521_at	7.53e-03	5.28e-03	-2.803.025
205703_at	7.55e-03	5.30e-03	-2.801.982
207526_s_at	7.56e-03	5.30e-03	-2.801.835
215097_at	7.56e-03	5.30e-03	-2.801.729
219100_at	7.58e-03	5.32e-03	2.800.675
210183_x_at	7.59e-03	5.33e-03	2.800.093
213673_x_at	7.61e-03	5.34e-03	-2.799.384
214952_at	7.61e-03	5.34e-03	-2.799.357
211663_x_at	7.62e-03	5.35e-03	-2.798.929
210424_s_at	7.62e-03	5.35e-03	2.798.769
220356_at	7.63e-03	5.36e-03	-279.828
204196_x_at	7.64e-03	5.36e-03	-2.798.046
211094_s_at	7.64e-03	5.37e-03	2.797.886
203839_s_at	7.64e-03	5.37e-03	-2.797.839
219026_s_at	7.64e-03	5.37e-03	2.797.736
222069_s_at	7.67e-03	5.39e-03	-2.796.471
200666_s_at	7.69e-03	5.40e-03	2.795.783
200008_s_at	7.69e-03	5.40e-03	2.795.757
202325_s_at	7.70e-03	5.41e-03	-2.795.164
210222_s_at	7.71e-03	5.42e-03	279.482
214977_at	7.72e-03	5.42e-03	-2.794.579
201757_at	7.72e-03	5.43e-03	2.794.321
201536_at	7.73e-03	5.43e-03	-2.793.928
201522_x_at	7.74e-03	5.44e-03	2.793.268
209461_x_at	7.75e-03	5.45e-03	279.307
206558_at	7.75e-03	5.45e-03	-2.792.973
217596_at	7.78e-03	5.47e-03	-2.791.626
215637_at	7.80e-03	5.48e-03	-2.790.799
218406_x_at	7.81e-03	5.49e-03	-2.790.438
220548_at	7.82e-03	5.49e-03	-2.790.149
204062_s_at	7.83e-03	5.50e-03	2.789.509
213349_at	7.84e-03	5.51e-03	2.788.969
205552_s_at	7.85e-03	5.52e-03	-278.849
205496_at	7.89e-03	5.54e-03	-2.787.126
212005_at	7.89e-03	5.55e-03	-2.787.031
206301_at	7.89e-03	5.55e-03	-2.786.766
203244_at	7.94e-03	5.59e-03	-2.784.692
219138_at	7.95e-03	5.59e-03	2.784.312
40524_at	7.95e-03	5.59e-03	-2.784.288

202139_at	7.96e-03	5.60e-03	2.784.043
201866_s_at	7.96e-03	5.60e-03	2.783.901
217128_s_at	7.99e-03	5.62e-03	-2.782.543
206201_s_at	7.99e-03	5.62e-03	2.782.429
221933_at	8.00e-03	5.63e-03	2.782.254
222327_x_at	8.00e-03	5.63e-03	-2.781.978
214049_x_at	8.01e-03	5.64e-03	2.781.601
220515_at	8.01e-03	5.64e-03	-278.152
214605_x_at	8.02e-03	5.64e-03	-2.781.408
212686_at	8.03e-03	5.65e-03	2.780.964
214821_at	8.03e-03	5.65e-03	-2.780.812
564_at	8.03e-03	5.65e-03	2.780.763
201394_s_at	8.03e-03	5.66e-03	-2.780.605
222208_s_at	8.05e-03	5.67e-03	-2.779.925
204858_s_at	8.05e-03	5.67e-03	2.779.788
215057_at	8.05e-03	5.67e-03	-2.779.719
220584_at	8.07e-03	5.68e-03	2.779.056
211148_s_at	8.07e-03	5.68e-03	277.897
220476_s_at	8.09e-03	5.70e-03	-2.778.143
201216_at	8.10e-03	5.71e-03	-2.777.697
216613_at	8.15e-03	5.74e-03	-277.562
32094_at	8.16e-03	5.75e-03	-2.775.342
211718_at	8.17e-03	5.75e-03	-2.775.025
60084_at	8.17e-03	5.75e-03	-2.774.946
208139_s_at	8.17e-03	5.76e-03	-277.482
40225_at	8.18e-03	5.76e-03	2.774.362
823_at	8.18e-03	5.76e-03	-277.427
221559_s_at	8.19e-03	5.77e-03	2.773.832
219482_at	8.21e-03	5.78e-03	2.773.263
207035_at	8.22e-03	5.79e-03	-2.772.717
221824_s_at	8.22e-03	5.79e-03	277.266
206624_at	8.26e-03	5.82e-03	-2.770.983
211040_x_at	8.28e-03	5.83e-03	2.770.268
222373_at	8.28e-03	5.84e-03	-27.702
91617_at	8.28e-03	5.84e-03	-2.770.168
220242_x_at	8.30e-03	5.85e-03	-2.769.454
215770_at	8.30e-03	5.85e-03	-2.769.217
213766_x_at	8.31e-03	5.86e-03	2.768.703
209300_s_at	8.32e-03	5.86e-03	2.768.564
53071_s_at	8.33e-03	5.87e-03	2.768.136
220327_at	8.34e-03	5.88e-03	-2.767.475
204049_s_at	8.34e-03	5.88e-03	-2.767.446
207082_at	8.35e-03	5.89e-03	2.767.025
218493_at	8.36e-03	5.89e-03	276.696

218877_s_at	8.36e-03	5.90e-03	-2.766.802
207193_at	8.37e-03	5.90e-03	-2.766.496
207425_s_at	8.39e-03	5.92e-03	2.765.583
203311_s_at	8.40e-03	5.92e-03	2.765.222
222017_x_at	8.43e-03	5.95e-03	-2.764.019
220173_at	8.43e-03	5.95e-03	2.763.763
219279_at	8.45e-03	5.96e-03	-2.763.098
207557_s_at	8.46e-03	5.97e-03	-2.762.641
202615_at	8.46e-03	5.97e-03	276.253
210001_s_at	8.47e-03	5.98e-03	2.762.027
203943_at	8.48e-03	5.99e-03	276.176
208862_s_at	8.50e-03	6.00e-03	-2.761.143
206904_at	8.50e-03	6.00e-03	-2.761.031
210484_s_at	8.52e-03	6.01e-03	-2.760.281
212888_at	8.53e-03	6.02e-03	-2.759.924
216580_at	8.53e-03	6.02e-03	2.759.776
203716_s_at	8.54e-03	6.03e-03	2.759.262
202522_at	8.55e-03	6.04e-03	2.758.991
201547_at	8.55e-03	6.04e-03	-2.758.954
210075_at	8.58e-03	6.06e-03	2.757.596
208375_at	8.59e-03	6.07e-03	-2.757.382
206591_at	8.60e-03	6.08e-03	-2.756.727
220051_at	8.62e-03	6.09e-03	-2.756.231
221977_at	8.62e-03	6.09e-03	-2.756.035
219535_at	8.63e-03	6.10e-03	-2.755.618
205101_at	8.63e-03	6.10e-03	-2.755.488
206276_at	8.64e-03	6.10e-03	-2.755.293
216672_s_at	8.65e-03	6.11e-03	-2.754.929
203808_at	8.67e-03	6.13e-03	-2.754.061
201403_s_at	8.68e-03	6.14e-03	2.753.569
214831_at	8.68e-03	6.14e-03	2.753.549
213986_s_at	8.71e-03	6.16e-03	-2.752.359
206650_at	8.75e-03	6.19e-03	-2.750.885
219766_at	8.75e-03	6.19e-03	-2.750.848
210342_s_at	8.75e-03	6.19e-03	-2.750.775
208350_at	8.76e-03	6.19e-03	-2.750.452
220918_at	8.76e-03	6.20e-03	-2.750.276
208279_s_at	8.78e-03	6.20e-03	-2.749.831
211667_x_at	8.78e-03	6.21e-03	-2.749.598
216719_s_at	8.79e-03	6.21e-03	-27.493
206357_at	8.80e-03	6.22e-03	2.748.985
217669_s_at	8.81e-03	6.23e-03	-2.748.394
214636_at	8.82e-03	6.24e-03	-2.747.943
221412_at	8.83e-03	6.24e-03	-2.747.722

210923_at	8.83e-03	6.25e-03	-2.747.533
216444_at	8.86e-03	6.27e-03	-2.746.521
201860_s_at	8.87e-03	6.27e-03	-2.746.101
210814_at	8.88e-03	6.28e-03	-2.745.846
218074_at	8.88e-03	6.28e-03	-2.745.703
213025_at	8.88e-03	6.29e-03	2.745.536
220768_s_at	8.90e-03	6.30e-03	2.744.824
219771_at	8.91e-03	6.31e-03	-2.744.424
207780_at	8.91e-03	6.31e-03	-2.744.346
202124_s_at	8.93e-03	6.32e-03	2.743.783
216339_s_at	8.93e-03	6.32e-03	-2.743.712
217283_at	8.93e-03	6.32e-03	-2.743.459
206103_at	8.98e-03	6.36e-03	2.741.641
51192_at	8.98e-03	6.36e-03	2.741.617
207963_at	9.00e-03	6.37e-03	-274.114
220707_s_at	9.04e-03	6.40e-03	2.739.433
217222_at	9.09e-03	6.44e-03	-2.737.485
205891_at	9.09e-03	6.44e-03	2.737.431
211141_s_at	9.11e-03	6.45e-03	2.736.644
208947_s_at	9.14e-03	6.47e-03	2.735.649
210181_s_at	9.15e-03	6.48e-03	-2.735.272
206336_at	9.16e-03	6.49e-03	-2.734.845
219834_at	9.18e-03	6.51e-03	-273.399
220712_at	9.19e-03	6.51e-03	-2.733.843
206968_s_at	9.20e-03	6.52e-03	2.733.269
201682_at	9.21e-03	6.52e-03	2.733.147
212928_at	9.22e-03	6.54e-03	-2.732.454
220701_at	9.23e-03	6.54e-03	-2.732.335
203912_s_at	9.25e-03	6.56e-03	273.142
206745_at	9.26e-03	6.56e-03	-273.104
209633_at	9.27e-03	6.57e-03	-2.730.764
215412_x_at	9.27e-03	6.57e-03	-2.730.659
220772_at	9.28e-03	6.58e-03	-2.730.212
218785_s_at	9.29e-03	6.58e-03	-2.730.045
211372_s_at	9.31e-03	6.60e-03	2.729.148
205533_s_at	9.33e-03	6.61e-03	-272.847
216822_x_at	9.33e-03	6.62e-03	2.728.389
217655_at	9.33e-03	6.62e-03	-2.728.251
217321_x_at	9.34e-03	6.62e-03	-2.728.144
207149_at	9.35e-03	6.63e-03	2.727.543
204282_s_at	9.36e-03	6.64e-03	2.727.319
214526_x_at	9.37e-03	6.64e-03	2.727.071
205994_at	9.37e-03	6.64e-03	-2.727.029
205745_x_at	9.38e-03	6.65e-03	2.726.576

211481_at	9.40e-03	6.67e-03	-2.725.897
213183_s_at	9.40e-03	6.67e-03	2.725.831
207249_s_at	9.42e-03	6.68e-03	2.725.153
210135_s_at	9.42e-03	6.68e-03	272.492
222081_at	9.42e-03	6.69e-03	-2.724.822
218148_at	9.43e-03	6.69e-03	-2.724.717
219307_at	9.44e-03	6.70e-03	-2.724.314
218813_s_at	9.44e-03	6.70e-03	2.724.112
209287_s_at	9.44e-03	6.70e-03	-2.723.993
211897_s_at	9.45e-03	6.70e-03	-2.723.951
217912_at	9.45e-03	6.71e-03	2.723.681
220407_s_at	9.45e-03	6.71e-03	2.723.662
213637_at	9.45e-03	6.71e-03	2.723.644
214820_at	9.46e-03	6.72e-03	2.723.274
208145_at	9.47e-03	6.72e-03	-2.722.991
218332_at	9.52e-03	6.76e-03	2.721.084
216070_at	9.54e-03	6.77e-03	-2.720.585
201364_s_at	9.57e-03	6.79e-03	-2.719.534
205092_x_at	9.58e-03	6.80e-03	2.719.042
211867_s_at	9.59e-03	6.81e-03	-271.855
205029_s_at	9.59e-03	6.81e-03	-2.718.449
217764_s_at	9.61e-03	6.83e-03	2.717.763
214324_at	9.62e-03	6.83e-03	2.717.679
212528_at	9.62e-03	6.83e-03	2.717.553
218823_s_at	9.63e-03	6.84e-03	2.717.033
219273_at	9.64e-03	6.85e-03	-2.716.887
216442_x_at	9.64e-03	6.85e-03	-2.716.691
204577_s_at	9.65e-03	6.85e-03	-2.716.459
207116_s_at	9.68e-03	6.88e-03	-2.715.327
220002_at	9.68e-03	6.88e-03	-2.715.176
205903_s_at	9.69e-03	6.89e-03	-2.714.912
219617_at	9.71e-03	6.90e-03	2.714.358
214782_at	9.71e-03	6.90e-03	2.714.224
219996_at	9.71e-03	6.90e-03	-2.714.116
220593_s_at	9.71e-03	6.90e-03	-2.714.082
218022_at	9.72e-03	6.91e-03	-2.713.827
205028_at	9.72e-03	6.91e-03	-2.713.564
219278_at	9.73e-03	6.92e-03	-2.713.493
217330_at	9.73e-03	6.92e-03	-2.713.433
220867_s_at	9.73e-03	6.92e-03	-2.713.288
202735_at	9.74e-03	6.92e-03	-2.713.052
215943_at	9.74e-03	6.93e-03	-2.713.031
205955_at	9.74e-03	6.93e-03	-2.712.945
205634_x_at	9.76e-03	6.95e-03	2.712.055

220008_at	9.78e-03	6.96e-03	-2.711.404
205334_at	9.80e-03	6.97e-03	2.710.908
209763_at	9.82e-03	6.99e-03	-2.709.943
210173_at	9.89e-03	7.03e-03	-2.707.808
220077_at	9.90e-03	7.04e-03	-2.707.455
219727_at	9.90e-03	7.04e-03	2.707.308
211919_s_at	9.91e-03	7.05e-03	-2.706.946
214671_s_at	9.93e-03	7.07e-03	-2.706.244
202976_s_at	9.94e-03	7.07e-03	270.593
211646_at	9.97e-03	7.09e-03	-2.704.878
203368_at	9.97e-03	7.09e-03	2.704.877
202118_s_at	9.98e-03	7.10e-03	2.704.388
210530_s_at	9.98e-03	7.11e-03	2.704.302
212697_at	9.99e-03	7.11e-03	2.704.052
221566_s_at	1.00e-02	7.12e-03	2.703.813
213398_s_at	1.00e-02	7.12e-03	-270.379
218781_at	1.00e-02	7.13e-03	2.703.398
208365_s_at	1.00e-02	7.14e-03	-2.702.878
210682_at	1.00e-02	7.16e-03	-2.702.006
216529_at	1.01e-02	7.18e-03	-2.700.816
213426_s_at	1.01e-02	7.20e-03	-2.700.078
218031_s_at	1.01e-02	7.20e-03	270.001
201677_at	1.01e-02	7.20e-03	-26.999
218403_at	1.01e-02	7.22e-03	-269.882
212609_s_at	1.01e-02	7.22e-03	-2.698.763
203592_s_at	1.01e-02	7.23e-03	-2.698.462
220174_at	1.02e-02	7.23e-03	2.698.359
204516_at	1.02e-02	7.24e-03	-2.698.228
219891_at	1.02e-02	7.24e-03	-2.697.994
215672_s_at	1.02e-02	7.24e-03	-2.697.972
212336_at	1.02e-02	7.24e-03	2.697.827
222286_at	1.02e-02	7.24e-03	26.978
216221_s_at	1.02e-02	7.25e-03	2.697.458
209072_at	1.02e-02	7.26e-03	-269.691
205928_at	1.02e-02	7.28e-03	2.696.041
215818_at	1.02e-02	7.30e-03	-269.518
220635_at	1.02e-02	7.30e-03	-2.695.114
216518_at	1.03e-02	7.34e-03	2.693.357
217279_x_at	1.03e-02	7.36e-03	-2.692.563
216097_at	1.03e-02	7.36e-03	-2.692.389
210525_x_at	1.04e-02	7.39e-03	-2.691.086
221149_at	1.04e-02	7.40e-03	-2.690.761
209929_s_at	1.04e-02	7.41e-03	2.690.084
217464_at	1.04e-02	7.41e-03	-2.689.978

221869_at	1.04e-02	7.42e-03	2.689.858
213824_at	1.04e-02	7.43e-03	-2.689.011
208586_s_at	1.04e-02	7.44e-03	2.688.942
220950_s_at	1.04e-02	7.44e-03	-2.688.796
218714_at	1.04e-02	7.44e-03	2.688.588
211383_s_at	1.05e-02	7.46e-03	2.688.009
207323_s_at	1.05e-02	7.46e-03	-2.687.819
217142_at	1.05e-02	7.49e-03	-2.686.401
218517_at	1.05e-02	7.50e-03	-2.686.186
202207_at	1.05e-02	7.51e-03	-2.685.627
214886_s_at	1.05e-02	7.53e-03	-2.684.888
201295_s_at	1.06e-02	7.54e-03	-2.684.457
44669_at	1.06e-02	7.56e-03	2.683.542
217327_at	1.06e-02	7.57e-03	-2.682.994
207136_at	1.06e-02	7.58e-03	-2.682.625
214384_s_at	1.06e-02	7.59e-03	-268.215
220011_at	1.06e-02	7.60e-03	2.681.743
207681_at	1.07e-02	7.61e-03	-26.813
221525_at	1.07e-02	7.61e-03	-2.681.276
202577_s_at	1.07e-02	7.61e-03	2.681.165
210471_s_at	1.07e-02	7.62e-03	-2.680.845
220465_at	1.07e-02	7.63e-03	2.680.234
212008_at	1.07e-02	7.64e-03	2.679.549
219704_at	1.07e-02	7.66e-03	-2.679.005
216680_s_at	1.07e-02	7.66e-03	-2.678.916
217254_s_at	1.07e-02	7.66e-03	-2.678.863
215087_at	1.07e-02	7.67e-03	-2.678.372
213254_at	1.07e-02	7.67e-03	-2.678.293
210569_s_at	1.07e-02	7.68e-03	-2.678.193
211522_s_at	1.08e-02	7.69e-03	-2.677.388
207548_at	1.08e-02	7.70e-03	-2.677.249
216098_s_at	1.08e-02	7.71e-03	-2.676.687
212842_x_at	1.08e-02	7.73e-03	-2.675.759
216774_at	1.08e-02	7.74e-03	-2.675.298
219795_at	1.09e-02	7.76e-03	2.674.577
213772_s_at	1.09e-02	7.76e-03	2.674.527
202816_s_at	1.09e-02	7.76e-03	267.448
211638_at	1.09e-02	7.76e-03	-2.674.343
218879_s_at	1.09e-02	7.77e-03	2.674.181
206943_at	1.09e-02	7.78e-03	2.673.536
205311_at	1.09e-02	7.80e-03	-2.672.886
210511_s_at	1.09e-02	7.80e-03	-267.256
218043_s_at	1.09e-02	7.81e-03	2.672.295
221828_s_at	1.09e-02	7.81e-03	2.672.054

214913_at	1.09e-02	7.82e-03	2.671.661
220838_at	1.09e-02	7.83e-03	2.671.425
216521_s_at	1.10e-02	7.85e-03	2.670.701
208069_x_at	1.10e-02	7.85e-03	-2.670.401
221040_at	1.10e-02	7.86e-03	-2.670.022
219291_at	1.10e-02	7.87e-03	2.669.687
205103_at	1.10e-02	7.88e-03	-2.669.271
210239_at	1.10e-02	7.89e-03	-2.668.746
202584_at	1.11e-02	7.92e-03	-2.667.395
215323_at	1.11e-02	7.92e-03	-2.667.306
222071_s_at	1.11e-02	7.93e-03	-2.667.021
214201_x_at	1.11e-02	7.94e-03	2.666.664
216654_at	1.11e-02	7.95e-03	-2.666.083
210718_s_at	1.11e-02	7.96e-03	2.665.783
221837_at	1.11e-02	7.96e-03	-2.665.778
207599_at	1.11e-02	7.96e-03	-2.665.687
222095_s_at	1.12e-02	7.99e-03	-2.664.494
207854_at	1.12e-02	8.02e-03	-266.305
206298_at	1.12e-02	8.03e-03	-26.629
202504_at	1.12e-02	8.05e-03	-2.662.023
203596_s_at	1.12e-02	8.06e-03	266.168
203577_at	1.13e-02	8.08e-03	2.660.838
203041_s_at	1.13e-02	8.09e-03	-2.660.396
206300_s_at	1.13e-02	8.09e-03	2.660.182
220128_s_at	1.13e-02	8.12e-03	-2.658.805
220019_s_at	1.13e-02	8.13e-03	-2.658.568
215959_at	1.13e-02	8.13e-03	-2.658.526
211632_at	1.14e-02	8.15e-03	-2.657.663
211634_x_at	1.14e-02	8.16e-03	-2.657.413
212269_s_at	1.14e-02	8.16e-03	2.657.173
216633_s_at	1.15e-02	8.21e-03	-2.655.061
221499_s_at	1.15e-02	8.22e-03	-2.654.714
220598_at	1.15e-02	8.23e-03	-2.654.462
217514_at	1.15e-02	8.23e-03	-2.654.421
207722_s_at	1.15e-02	8.27e-03	2.652.649
212095_s_at	1.16e-02	8.30e-03	-2.651.442
215982_s_at	1.16e-02	8.30e-03	2.651.319
213506_at	1.16e-02	8.31e-03	2.651.088
215841_at	1.16e-02	8.32e-03	-2.650.696
217656_at	1.16e-02	8.32e-03	-265.052
213128_s_at	1.16e-02	8.33e-03	2.650.295
204779_s_at	1.16e-02	8.34e-03	2.649.783
216081_at	1.16e-02	8.34e-03	-2.649.639
203848_at	1.17e-02	8.37e-03	2.648.662

219605_at	1.17e-02	8.39e-03	-264.781
217522_at	1.17e-02	8.39e-03	-2.647.594
221481_x_at	1.17e-02	8.41e-03	2.646.952
213489_at	1.17e-02	8.41e-03	-2.646.863
200646_s_at	1.17e-02	8.42e-03	-2.646.471
216462_at	1.17e-02	8.42e-03	2.646.468
205008_s_at	1.17e-02	8.43e-03	2.646.262
212018_s_at	1.18e-02	8.45e-03	2.645.467
220182_at	1.18e-02	8.45e-03	-2.645.183
214014_at	1.18e-02	8.46e-03	-2.644.779
211486_s_at	1.18e-02	8.47e-03	-2.644.624
202692_s_at	1.18e-02	8.47e-03	2.644.471
209571_at	1.18e-02	8.48e-03	2.644.175
205134_s_at	1.18e-02	8.50e-03	2.643.404
211593_s_at	1.18e-02	8.50e-03	2.643.364
203901_at	1.18e-02	8.50e-03	2.643.169
216764_at	1.19e-02	8.51e-03	-2.642.829
211504_x_at	1.19e-02	8.51e-03	264.277
207417_s_at	1.19e-02	8.52e-03	2.642.559
216186_at	1.19e-02	8.54e-03	-2.641.766
215906_at	1.19e-02	8.55e-03	-2.641.396
216717_at	1.19e-02	8.56e-03	-2.640.725
209098_s_at	1.19e-02	8.57e-03	2.640.367
211881_x_at	1.19e-02	8.57e-03	-2.640.263
210364_at	1.19e-02	8.57e-03	-264.026
203794_at	1.20e-02	8.59e-03	2.639.804
205403_at	1.20e-02	8.61e-03	263.869
220881_at	1.20e-02	8.62e-03	-2.638.292
204384_at	1.20e-02	8.64e-03	2.637.677
221160_s_at	1.20e-02	8.65e-03	-2.637.294
217077_s_at	1.21e-02	8.66e-03	-2.636.877
209575_at	1.21e-02	8.67e-03	2.636.449
204816_s_at	1.21e-02	8.69e-03	-2.635.618
208745_at	1.21e-02	8.70e-03	2.635.205
216156_at	1.21e-02	8.71e-03	-2.634.671
209728_at	1.21e-02	8.72e-03	-2.634.669
202917_s_at	1.21e-02	8.73e-03	-2.634.089
221423_s_at	1.22e-02	8.76e-03	2.632.715
212305_s_at	1.22e-02	8.78e-03	-2.632.182
208587_s_at	1.22e-02	8.79e-03	-2.631.604
214892_x_at	1.22e-02	8.80e-03	-2.631.236
215218_s_at	1.22e-02	8.81e-03	2.631.079
205708_s_at	1.23e-02	8.81e-03	-2.630.803
217603_at	1.23e-02	8.82e-03	-2.630.732

210368_at	1.23e-02	8.83e-03	-2.630.303
221126_at	1.23e-02	8.83e-03	-2.630.094
214082_at	1.23e-02	8.85e-03	-2.629.418
211359_s_at	1.24e-02	8.88e-03	-2.628.013
222320_at	1.24e-02	8.89e-03	2.627.821
217215_s_at	1.24e-02	8.91e-03	-2.627.134
216448_at	1.24e-02	8.92e-03	-2.626.584
207404_s_at	1.24e-02	8.93e-03	-2.626.212
221073_s_at	1.24e-02	8.94e-03	-262.596
211131_s_at	1.24e-02	8.94e-03	-2.625.785
216775_at	1.24e-02	8.95e-03	-2.625.555
206972_s_at	1.24e-02	8.95e-03	-2.625.523
208457_at	1.25e-02	8.98e-03	2.624.277
213966_at	1.25e-02	8.99e-03	-2.623.989
206959_s_at	1.25e-02	8.99e-03	2.623.847
200924_s_at	1.25e-02	8.99e-03	2.623.784
220500_s_at	1.25e-02	9.00e-03	2.623.539
212958_x_at	1.25e-02	9.00e-03	2.623.531
201037_at	1.25e-02	9.00e-03	2.623.445
209783_at	1.25e-02	9.01e-03	-2.623.273
213853_at	1.26e-02	9.05e-03	2.621.632
210742_at	1.26e-02	9.06e-03	-2.621.303
219546_at	1.26e-02	9.08e-03	-2.620.585
209109_s_at	1.26e-02	9.09e-03	2.620.241
210562_at	1.26e-02	9.10e-03	-2.619.902
221644_s_at	1.26e-02	9.11e-03	-2.619.303
213621_s_at	1.27e-02	9.11e-03	-261.922
210295_at	1.27e-02	9.13e-03	-2.618.533
209930_s_at	1.27e-02	9.15e-03	-2.617.672
215606_s_at	1.27e-02	9.15e-03	-2.617.671
222127_s_at	1.27e-02	9.15e-03	2.617.653
210847_x_at	1.27e-02	9.17e-03	261.726
210684_s_at	1.28e-02	9.19e-03	-2.616.138
219143_s_at	1.28e-02	9.20e-03	2.615.842
205590_at	1.28e-02	9.22e-03	-2.615.314
214811_at	1.28e-02	9.22e-03	-2.615.219
206209_s_at	1.28e-02	9.22e-03	-2.615.208
203708_at	1.28e-02	9.23e-03	-2.614.795
221720_s_at	1.28e-02	9.25e-03	-2.614.243
55583_at	1.28e-02	9.25e-03	2.614.241
203932_at	1.28e-02	9.25e-03	-2.614.171
222210_at	1.28e-02	9.25e-03	-2.613.949
222076_at	1.28e-02	9.26e-03	-2.613.643
208243_s_at	1.29e-02	9.27e-03	-2.613.257

216525_x_at	1.29e-02	9.28e-03	2.612.914
121_at	1.29e-02	9.29e-03	-2.612.487
211191_at	1.29e-02	9.30e-03	-2.612.295
204186_s_at	1.29e-02	9.30e-03	2.612.174
220895_at	1.29e-02	9.30e-03	-261.204
209315_at	1.29e-02	9.31e-03	-2.611.654
221330_at	1.29e-02	9.32e-03	-2.611.341
220702_at	1.29e-02	9.34e-03	-2.610.842
211526_s_at	1.29e-02	9.34e-03	-2.610.622
209199_s_at	1.30e-02	9.35e-03	2.610.408
206029_at	1.30e-02	9.35e-03	2.610.302
216618_at	1.30e-02	9.35e-03	-2.610.292
221344_at	1.30e-02	9.36e-03	-2.610.102
215939_at	1.30e-02	9.37e-03	-2.609.588
221231_s_at	1.30e-02	9.38e-03	-2.609.369
52169_at	1.30e-02	9.39e-03	2.609.027
207285_x_at	1.30e-02	9.42e-03	-2.607.813
207662_at	1.30e-02	9.42e-03	-260.771
214756_x_at	1.31e-02	9.47e-03	26.058
218348_s_at	1.31e-02	9.47e-03	2.605.754
212575_at	1.31e-02	9.49e-03	-2.604.991
208987_s_at	1.32e-02	9.53e-03	-2.603.723
207159_x_at	1.32e-02	9.53e-03	-2.603.618
208421_at	1.32e-02	9.54e-03	-2.603.486
213171_s_at	1.32e-02	9.54e-03	-260.329
216861_at	1.32e-02	9.54e-03	-2.603.232
216455_at	1.32e-02	9.54e-03	-2.603.169
221441_at	1.32e-02	9.55e-03	-2.602.795
206645_s_at	1.32e-02	9.57e-03	2.602.387
208565_at	1.33e-02	9.58e-03	-2.601.875
216568_x_at	1.33e-02	9.59e-03	2.601.673
58308_at	1.33e-02	9.59e-03	-2.601.507
214503_x_at	1.33e-02	9.59e-03	-2.601.401
218485_s_at	1.33e-02	9.61e-03	2.600.792
208807_s_at	1.33e-02	9.62e-03	2.600.269
213010_at	1.33e-02	9.63e-03	2.599.933
216196_at	1.34e-02	9.67e-03	-2.598.767
213537_at	1.34e-02	9.67e-03	-2.598.613
219385_at	1.34e-02	9.67e-03	2.598.452
210430_x_at	1.34e-02	9.68e-03	-2.598.404
220462_at	1.34e-02	9.69e-03	-2.597.766
215020_at	1.34e-02	9.70e-03	2.597.412
201628_s_at	1.35e-02	9.77e-03	2.595.083
206421_s_at	1.35e-02	9.78e-03	-2.594.772

205831_at	1.35e-02	9.80e-03	-2.594.028
211392_s_at	1.36e-02	9.81e-03	2.593.587
206446_s_at	1.36e-02	9.81e-03	-2.593.426
209148_at	1.36e-02	9.83e-03	-259.288
214139_at	1.36e-02	9.84e-03	-2.592.658
213606_s_at	1.36e-02	9.86e-03	2.591.678
205610_at	1.37e-02	9.91e-03	-2.590.117
65770_at	1.37e-02	9.92e-03	-2.589.626
221294_at	1.37e-02	9.94e-03	-2.589.045
207398_at	1.38e-02	9.96e-03	-2.588.275
210134_x_at	1.38e-02	9.97e-03	-2.587.991
221948_s_at	1.38e-02	1.00e-02	-2.586.689
204865_at	1.38e-02	1.00e-02	-2.586.647
210831_s_at	1.39e-02	1.00e-02	-2.585.491
212045_at	1.39e-02	1.01e-02	258.502
210498_at	1.39e-02	1.01e-02	-2.584.362
214587_at	1.39e-02	1.01e-02	-2.584.179
210064_s_at	1.39e-02	1.01e-02	-2.583.772
206020_at	1.39e-02	1.01e-02	-258.353
204320_at	1.40e-02	1.01e-02	258.301
204043_at	1.40e-02	1.01e-02	-2.582.981
221351_at	1.40e-02	1.01e-02	-2.582.729
213463_s_at	1.40e-02	1.01e-02	2.582.167
214090_at	1.40e-02	1.01e-02	-258.194
211824_x_at	1.40e-02	1.01e-02	-2.581.765
220440_at	1.40e-02	1.02e-02	-2.581.502
208411_x_at	1.40e-02	1.02e-02	-2.580.807
220013_at	1.41e-02	1.02e-02	-2.580.689
204148_s_at	1.41e-02	1.02e-02	2.580.601
219425_at	1.41e-02	1.02e-02	2.580.517
220129_at	1.41e-02	1.02e-02	2.580.238
222096_x_at	1.41e-02	1.02e-02	-2.579.347
208915_s_at	1.41e-02	1.02e-02	2.578.763
207984_s_at	1.41e-02	1.02e-02	-2.578.511
206473_at	1.41e-02	1.02e-02	-2.578.489
210055_at	1.41e-02	1.02e-02	-2.578.394
33778_at	1.42e-02	1.03e-02	2.577.669
205832_at	1.42e-02	1.03e-02	-2.576.843
216169_at	1.42e-02	1.03e-02	-2.576.663
217775_s_at	1.42e-02	1.03e-02	2.575.645
201006_at	1.42e-02	1.03e-02	2.575.483
206602_s_at	1.43e-02	1.03e-02	-2.575.053
211050_x_at	1.43e-02	1.04e-02	-2.574.385
204885_s_at	1.43e-02	1.04e-02	-257.415

205931_s_at	1.43e-02	1.04e-02	-2.573.973
220665_at	1.43e-02	1.04e-02	-2.572.951
216910_at	1.44e-02	1.04e-02	-2.572.523
221104_s_at	1.44e-02	1.05e-02	2.571.298
211401_s_at	1.44e-02	1.05e-02	-257.076
208430_s_at	1.44e-02	1.05e-02	2.570.674
216390_at	1.44e-02	1.05e-02	-2.570.555
205600_x_at	1.45e-02	1.05e-02	-2.570.198
201063_at	1.45e-02	1.05e-02	-2.570.015
216562_at	1.45e-02	1.05e-02	-2.569.773
216517_at	1.45e-02	1.05e-02	-2.569.746
207604_s_at	1.45e-02	1.05e-02	256.973
217966_s_at	1.45e-02	1.05e-02	2.569.291
219626_at	1.45e-02	1.05e-02	-2.568.406
219855_at	1.45e-02	1.05e-02	-2.568.401
200699_at	1.45e-02	1.05e-02	256.828
204386_s_at	1.45e-02	1.06e-02	-2.567.946
215580_at	1.45e-02	1.06e-02	-2.567.844
203393_at	1.45e-02	1.06e-02	-2.567.768
209885_at	1.46e-02	1.06e-02	2.567.656
214844_s_at	1.46e-02	1.06e-02	2.567.393
208534_s_at	1.46e-02	1.06e-02	-2.567.358
210480_s_at	1.46e-02	1.06e-02	2.567.227
219782_s_at	1.46e-02	1.06e-02	-2.566.768
207294_at	1.46e-02	1.06e-02	-2.566.015
207343_at	1.46e-02	1.06e-02	-2.565.257
217161_x_at	1.47e-02	1.06e-02	-2.564.921
214725_at	1.47e-02	1.06e-02	-2.564.794
215865_at	1.47e-02	1.07e-02	-2.564.359
207823_s_at	1.47e-02	1.07e-02	-2.564.227
208084_at	1.47e-02	1.07e-02	-2.563.281
219454_at	1.48e-02	1.07e-02	2.562.261
207530_s_at	1.48e-02	1.07e-02	-2.561.744
211561_x_at	1.48e-02	1.07e-02	2.561.614
217423_at	1.48e-02	1.08e-02	2.561.006
206157_at	1.48e-02	1.08e-02	2.560.329
209561_at	1.48e-02	1.08e-02	-2.560.215
208151_x_at	1.48e-02	1.08e-02	-2.560.212
213494_s_at	1.49e-02	1.08e-02	2.559.697
222227_at	1.49e-02	1.08e-02	-2.559.555
207994_s_at	1.49e-02	1.08e-02	-2.558.918
216226_at	1.50e-02	1.09e-02	-2.557.196
207271_x_at	1.50e-02	1.09e-02	-2.556.685
202336_s_at	1.50e-02	1.09e-02	2.556.003

220111_s_at	1.50e-02	1.09e-02	-2.555.968
215131_at	1.50e-02	1.09e-02	-2.555.909
215576_at	1.50e-02	1.09e-02	-2.555.559
205069_s_at	1.50e-02	1.09e-02	-2.555.319
210161_at	1.51e-02	1.10e-02	-2.554.515
214040_s_at	1.51e-02	1.10e-02	-2.554.025
217434_at	1.51e-02	1.10e-02	-2.553.701
215944_at	1.51e-02	1.10e-02	-2.553.654
212672_at	1.51e-02	1.10e-02	2.553.173
211020_at	1.51e-02	1.10e-02	-25.531
208993_s_at	1.51e-02	1.10e-02	2.553.012
200980_s_at	1.51e-02	1.10e-02	2.552.929
216054_x_at	1.52e-02	1.10e-02	-2.552.083
204323_x_at	1.52e-02	1.10e-02	-2.551.923
214215_s_at	1.52e-02	1.11e-02	-2.551.644
214009_at	1.52e-02	1.11e-02	-2.551.446
78047_s_at	1.52e-02	1.11e-02	-2.551.233
219958_at	1.52e-02	1.11e-02	2.551.157
222093_s_at	1.53e-02	1.11e-02	-2.549.713
214292_at	1.53e-02	1.12e-02	-2.548.569
216180_s_at	1.53e-02	1.12e-02	-2.548.364
211460_at	1.53e-02	1.12e-02	-2.548.155
212262_at	1.53e-02	1.12e-02	2.548.065
214298_x_at	1.53e-02	1.12e-02	2.547.962
208637_x_at	1.54e-02	1.12e-02	2.547.627
204604_at	1.54e-02	1.12e-02	2.547.575
204952_at	1.54e-02	1.12e-02	-2.547.188
34063_at	1.54e-02	1.12e-02	-2.547.135
213536_s_at	1.54e-02	1.12e-02	-2.546.863
204933_s_at	1.54e-02	1.12e-02	-2.546.234
210883_x_at	1.54e-02	1.12e-02	-2.545.876
205447_s_at	1.54e-02	1.12e-02	-2.545.639
216045_at	1.54e-02	1.12e-02	-2.545.589
222236_s_at	1.54e-02	1.13e-02	2.545.324
205766_at	1.55e-02	1.13e-02	-2.544.963
213972_at	1.55e-02	1.13e-02	-2.544.558
206865_at	1.55e-02	1.13e-02	-2.544.004
215800_at	1.56e-02	1.14e-02	2.542.251
207250_at	1.56e-02	1.14e-02	-2.542.132
220152_at	1.56e-02	1.14e-02	-2.542.092
217307_at	1.56e-02	1.14e-02	-2.542.054
221968_s_at	1.56e-02	1.14e-02	-2.542.022
215167_at	1.56e-02	1.14e-02	2.541.725
213660_s_at	1.56e-02	1.14e-02	2.541.265

220150_s_at	1.56e-02	1.14e-02	2.541.073
207642_at	1.56e-02	1.14e-02	-2.540.507
220178_at	1.56e-02	1.14e-02	254.039
206748_s_at	1.57e-02	1.15e-02	-2.539.084
220226_at	1.57e-02	1.15e-02	-2.539.079
206696_at	1.57e-02	1.15e-02	2.538.559
221894_at	1.57e-02	1.15e-02	2.538.449
214227_at	1.58e-02	1.15e-02	-2.537.744
207314_x_at	1.58e-02	1.15e-02	-2.537.574
205243_at	1.58e-02	1.15e-02	2.537.468
213067_at	1.58e-02	1.15e-02	-2.537.132
215258_at	1.58e-02	1.16e-02	-2.535.659
205217_at	1.59e-02	1.16e-02	253.493
203627_at	1.59e-02	1.16e-02	-2.534.786
207998_s_at	1.59e-02	1.16e-02	-2.534.749
213774_s_at	1.59e-02	1.16e-02	-2.534.183
202134_s_at	1.60e-02	1.17e-02	2.532.535
214577_at	1.60e-02	1.17e-02	2.532.382
204465_s_at	1.60e-02	1.17e-02	-2.531.851
216973_s_at	1.60e-02	1.17e-02	253.142
206731_at	1.60e-02	1.17e-02	-2.531.098
221453_at	1.61e-02	1.17e-02	-2.530.717
217214_s_at	1.61e-02	1.17e-02	-2.530.646
220829_s_at	1.61e-02	1.18e-02	-2.529.818
220562_at	1.61e-02	1.18e-02	-2.529.507
219799_s_at	1.61e-02	1.18e-02	-2.529.452
217300_at	1.61e-02	1.18e-02	-2.529.164
215248_at	1.62e-02	1.18e-02	-2.528.275
207031_at	1.62e-02	1.18e-02	2.528.113
200923_at	1.62e-02	1.18e-02	-2.527.386
218309_at	1.62e-02	1.18e-02	2.527.344
217371_s_at	1.62e-02	1.19e-02	-2.526.785
209367_at	1.62e-02	1.19e-02	2.526.718
215664_s_at	1.63e-02	1.19e-02	-252.614
219867_at	1.63e-02	1.19e-02	2.526.112
209982_s_at	1.63e-02	1.19e-02	-2.525.688
214964_at	1.63e-02	1.19e-02	2.525.536
209438_at	1.63e-02	1.19e-02	2.525.472
217485_x_at	1.63e-02	1.19e-02	2.525.431
212128_s_at	1.63e-02	1.19e-02	2.525.126
211656_x_at	1.63e-02	1.19e-02	-2.524.514
207951_at	1.63e-02	1.19e-02	-2.524.325
207196_s_at	1.63e-02	1.20e-02	2.523.918
218937_at	1.64e-02	1.20e-02	2.523.458

204048_s_at	1.64e-02	1.20e-02	-2.523.009
209702_at	1.64e-02	1.20e-02	-2.522.932
220797_at	1.64e-02	1.20e-02	-2.522.565
215028_at	1.64e-02	1.20e-02	-2.522.195
217860_at	1.65e-02	1.20e-02	-2.521.528
219417_s_at	1.65e-02	1.20e-02	-252.137
212404_s_at	1.65e-02	1.20e-02	2.521.017
215251_at	1.65e-02	1.21e-02	-2.520.965
216494_at	1.66e-02	1.21e-02	-2.519.075
206399_x_at	1.66e-02	1.21e-02	-251.901
217580_x_at	1.66e-02	1.21e-02	-2.518.454
204099_at	1.66e-02	1.21e-02	251.825
213339_at	1.66e-02	1.22e-02	-2.517.851
216664_at	1.66e-02	1.22e-02	-2.517.743
219499_at	1.66e-02	1.22e-02	2.517.336
202334_s_at	1.67e-02	1.22e-02	2.516.942
216544_at	1.67e-02	1.22e-02	-2.516.024
211823_s_at	1.67e-02	1.23e-02	-2.514.887
222322_at	1.68e-02	1.23e-02	2.514.683
221649_s_at	1.68e-02	1.23e-02	2.514.552
221713_s_at	1.68e-02	1.23e-02	-2.514.549
205005_s_at	1.68e-02	1.23e-02	2.514.501
220080_at	1.68e-02	1.23e-02	-2.514.325
221411_at	1.68e-02	1.23e-02	-25.142
215496_at	1.68e-02	1.23e-02	-2.513.574
220843_s_at	1.68e-02	1.23e-02	2.513.523
215260_s_at	1.68e-02	1.23e-02	2.513.196
204706_at	1.68e-02	1.23e-02	-2.513.037
215283_at	1.68e-02	1.23e-02	2.512.633
207018_s_at	1.69e-02	1.24e-02	-2.512.045
216599_x_at	1.69e-02	1.24e-02	-2.511.473
209991_x_at	1.69e-02	1.24e-02	-2.511.367
222379_at	1.69e-02	1.24e-02	-2.510.726
202894_at	1.69e-02	1.24e-02	2.510.516
206895_at	1.70e-02	1.24e-02	-2.510.033
216110_x_at	1.70e-02	1.25e-02	-2.509.265
207716_at	1.70e-02	1.25e-02	-2.509.218
210938_at	1.70e-02	1.25e-02	-2.508.936
220166_at	1.70e-02	1.25e-02	2.508.933
218589_at	1.70e-02	1.25e-02	-2.508.384
202017_at	1.70e-02	1.25e-02	-2.508.338
217710_x_at	1.70e-02	1.25e-02	-2.508.299
221415_s_at	1.70e-02	1.25e-02	-250.817
208500_x_at	1.70e-02	1.25e-02	-2.508.091

210515_at	1.70e-02	1.25e-02	-2.507.962
221989_at	1.71e-02	1.25e-02	2.507.541
219426_at	1.71e-02	1.25e-02	-2.506.983
217994_x_at	1.71e-02	1.25e-02	2.506.802
215471_s_at	1.71e-02	1.26e-02	-2.506.313
219533_at	1.71e-02	1.26e-02	-2.506.248
216090_x_at	1.72e-02	1.26e-02	-2.505.498
215324_at	1.72e-02	1.26e-02	-250.469
206282_at	1.72e-02	1.26e-02	-2.504.179
208553_at	1.72e-02	1.26e-02	-2.503.876
207487_at	1.73e-02	1.27e-02	-2.503.404
221575_at	1.73e-02	1.27e-02	2.503.084
207230_at	1.73e-02	1.27e-02	-2.502.806
208382_s_at	1.73e-02	1.27e-02	-2.502.439
205327_s_at	1.73e-02	1.27e-02	-2.502.424
207399_at	1.73e-02	1.27e-02	-2.502.225
201733_at	1.73e-02	1.27e-02	-2.501.979
217042_at	1.73e-02	1.27e-02	-2.501.769
208582_s_at	1.73e-02	1.27e-02	-2.501.337
210390_s_at	1.73e-02	1.27e-02	2.501.247
208283_at	1.74e-02	1.27e-02	2.501.129
216905_s_at	1.74e-02	1.27e-02	2.501.105
210029_at	1.74e-02	1.27e-02	2.501.038
221924_at	1.74e-02	1.27e-02	250.078
212413_at	1.74e-02	1.28e-02	2.500.672
209879_at	1.74e-02	1.28e-02	-2.500.665
39705_at	1.74e-02	1.28e-02	-2.500.435
207540_s_at	1.74e-02	1.28e-02	-2.499.574
221217_s_at	1.74e-02	1.28e-02	-249.915
209519_at	1.74e-02	1.28e-02	2.498.989
217597_x_at	1.75e-02	1.28e-02	-249.892
217604_at	1.75e-02	1.28e-02	-2.498.764
205199_at	1.75e-02	1.28e-02	2.498.591
218850_s_at	1.75e-02	1.29e-02	2.497.727
220022_at	1.75e-02	1.29e-02	-2.497.624
216742_at	1.75e-02	1.29e-02	-2.497.173
209807_s_at	1.76e-02	1.29e-02	-2.495.867
210432_s_at	1.76e-02	1.29e-02	-2.495.573
204230_s_at	1.76e-02	1.30e-02	-2.495.094
204245_s_at	1.77e-02	1.30e-02	-2.493.792
211701_s_at	1.77e-02	1.30e-02	-2.493.279
213685_at	1.77e-02	1.30e-02	-2.493.229
221936_x_at	1.77e-02	1.30e-02	-2.493.211
203528_at	1.77e-02	1.30e-02	-2.492.737

205512_s_at	1.77e-02	1.30e-02	-2.492.698
206081_at	1.78e-02	1.31e-02	2.492.305
209594_x_at	1.78e-02	1.31e-02	-2.492.252
210012_s_at	1.78e-02	1.31e-02	-2.491.382
221615_at	1.78e-02	1.31e-02	-2.491.351
204506_at	1.78e-02	1.31e-02	-2.491.212
206770_s_at	1.78e-02	1.31e-02	-2.491.096
213858_at	1.78e-02	1.31e-02	2.490.709
221189_s_at	1.79e-02	1.31e-02	2.490.252
204917_s_at	1.79e-02	1.32e-02	248.929
219164_s_at	1.79e-02	1.32e-02	2.489.125
210631_at	1.79e-02	1.32e-02	-2.489.102
217867_x_at	1.79e-02	1.32e-02	2.488.627
213456_at	1.79e-02	1.32e-02	2.488.457
202354_s_at	1.79e-02	1.32e-02	2.488.378
221721_s_at	1.79e-02	1.32e-02	-2.488.317
222112_at	1.80e-02	1.32e-02	-2.487.963
202725_at	1.80e-02	1.32e-02	-248.793
206824_at	1.80e-02	1.32e-02	-2.487.672
206524_at	1.80e-02	1.33e-02	-2.486.496
216541_x_at	1.80e-02	1.33e-02	-2.486.126
222084_s_at	1.81e-02	1.33e-02	-2.485.294
217475_s_at	1.81e-02	1.33e-02	2.485.279
221109_at	1.81e-02	1.33e-02	-2.485.217
220456_at	1.81e-02	1.33e-02	-2.485.008
216438_s_at	1.81e-02	1.34e-02	2.483.978
218033_s_at	1.82e-02	1.34e-02	2.483.676
209495_at	1.82e-02	1.34e-02	2.483.667
215382_x_at	1.82e-02	1.34e-02	-2.483.554
216995_x_at	1.82e-02	1.34e-02	-2.483.451
210007_s_at	1.82e-02	1.34e-02	2.482.791
219685_at	1.82e-02	1.34e-02	-2.482.644
208354_s_at	1.82e-02	1.34e-02	-2.481.842
213288_at	1.83e-02	1.34e-02	-2.481.572
206673_at	1.83e-02	1.35e-02	-2.480.945
202044_at	1.83e-02	1.35e-02	-2.480.851
209492_x_at	1.83e-02	1.35e-02	2.480.803
211778_s_at	1.83e-02	1.35e-02	-2.480.378
222022_at	1.83e-02	1.35e-02	-2.480.249
221021_s_at	1.83e-02	1.35e-02	2.480.157
221392_at	1.83e-02	1.35e-02	-2.480.077
209603_at	1.83e-02	1.35e-02	-2.479.917
204324_s_at	1.84e-02	1.36e-02	-2.478.702
206475_x_at	1.84e-02	1.36e-02	-2.478.564

217642_at	1.84e-02	1.36e-02	-2.478.522
32699_s_at	1.84e-02	1.36e-02	-247.844
213439_x_at	1.84e-02	1.36e-02	-2.478.056
205397_x_at	1.85e-02	1.36e-02	-2.477.156
38241_at	1.85e-02	1.36e-02	-2.476.827
212935_at	1.85e-02	1.36e-02	2.476.312
207161_at	1.85e-02	1.37e-02	-2.475.801
212628_at	1.85e-02	1.37e-02	2.475.657
211508_s_at	1.85e-02	1.37e-02	-2.475.638
215022_x_at	1.86e-02	1.37e-02	-2.475.339
204679_at	1.86e-02	1.37e-02	2.475.218
AFFX-HUMRGE/M100	1.86e-02	1.37e-02	2.474.902
204216_s_at	1.86e-02	1.37e-02	-2.473.847
210343_s_at	1.86e-02	1.37e-02	-2.473.814
218044_x_at	1.87e-02	1.38e-02	-24.731
204740_at	1.87e-02	1.38e-02	-2.472.517
213557_at	1.87e-02	1.38e-02	-2.472.432
213517_at	1.87e-02	1.38e-02	-2.472.191
219947_at	1.87e-02	1.38e-02	-2.472.191
209086_x_at	1.87e-02	1.38e-02	2.472.138
211525_s_at	1.88e-02	1.39e-02	-2.470.813
47560_at	1.88e-02	1.39e-02	2.470.803
210581_x_at	1.88e-02	1.39e-02	2.470.184
202617_s_at	1.88e-02	1.39e-02	2.470.138
213950_s_at	1.88e-02	1.39e-02	-2.469.288
216947_at	1.89e-02	1.40e-02	-2.467.918
203465_at	1.89e-02	1.40e-02	2.467.725
213558_at	1.89e-02	1.40e-02	246.768
205287_s_at	1.90e-02	1.40e-02	-2.466.928
204814_at	1.90e-02	1.40e-02	2.466.387
211198_s_at	1.90e-02	1.40e-02	-2.466.322
216027_at	1.90e-02	1.40e-02	-2.466.019
206909_at	1.90e-02	1.40e-02	-2.465.899
213430_at	1.90e-02	1.41e-02	-2.465.618
207910_at	1.90e-02	1.41e-02	-2.465.459
210361_s_at	1.91e-02	1.41e-02	-2.465.012
222167_at	1.91e-02	1.41e-02	-2.464.667
214166_at	1.91e-02	1.41e-02	-2.464.629
217259_at	1.91e-02	1.41e-02	-2.464.618
208653_s_at	1.91e-02	1.41e-02	2.464.336
204166_at	1.91e-02	1.41e-02	-2.464.003
214530_x_at	1.91e-02	1.41e-02	-2.463.713
37872_at	1.92e-02	1.42e-02	2.462.687
213619_at	1.92e-02	1.42e-02	-2.462.521

217538_at	1.92e-02	1.42e-02	-2.462.498
211834_s_at	1.92e-02	1.42e-02	-2.461.546
219326_s_at	1.92e-02	1.42e-02	2.461.534
201748_s_at	1.92e-02	1.42e-02	2.461.442
58916_at	1.92e-02	1.42e-02	2.461.166
202989_at	1.93e-02	1.42e-02	-2.460.965
203339_at	1.93e-02	1.42e-02	246.092
215954_s_at	1.93e-02	1.42e-02	-2.460.749
215777_at	1.93e-02	1.43e-02	-2.460.549
206433_s_at	1.93e-02	1.43e-02	-2.460.404
220513_at	1.94e-02	1.43e-02	2.459.026
211591_s_at	1.94e-02	1.43e-02	-2.458.912
217462_at	1.94e-02	1.43e-02	-2.458.796
209811_at	1.94e-02	1.43e-02	2.458.598
212498_at	1.94e-02	1.44e-02	2.457.931
209401_s_at	1.94e-02	1.44e-02	-2.457.886
217683_at	1.94e-02	1.44e-02	-2.457.871
220455_at	1.94e-02	1.44e-02	-2.457.827
205081_at	1.94e-02	1.44e-02	2.457.125
216436_at	1.94e-02	1.44e-02	-2.457.033
222189_at	1.95e-02	1.44e-02	-2.456.352
212362_at	1.95e-02	1.44e-02	2.456.335
215344_at	1.95e-02	1.44e-02	2.455.981
204087_s_at	1.95e-02	1.44e-02	2.455.824
222136_x_at	1.95e-02	1.45e-02	2.455.398
207479_at	1.96e-02	1.45e-02	-2.454.857
215831_at	1.96e-02	1.45e-02	-2.454.694
205149_s_at	1.96e-02	1.45e-02	-2.454.623
216567_at	1.96e-02	1.45e-02	-2.454.361
214406_s_at	1.96e-02	1.45e-02	-245.401
207328_at	1.96e-02	1.45e-02	-2.453.693
207976_at	1.96e-02	1.45e-02	-2.453.651
204876_at	1.96e-02	1.45e-02	-2.453.237
215277_at	1.96e-02	1.45e-02	-2.453.138
214891_at	1.97e-02	1.46e-02	-2.452.891
205534_at	1.97e-02	1.46e-02	-2.452.787
207671_s_at	1.97e-02	1.46e-02	-2.452.611
207223_s_at	1.97e-02	1.46e-02	2.452.441
216275_at	1.97e-02	1.46e-02	-2.452.438
216527_at	1.97e-02	1.46e-02	-2.452.141
213678_at	1.97e-02	1.46e-02	-2.452.049
211608_at	1.98e-02	1.46e-02	-2.450.616
203417_at	1.98e-02	1.46e-02	2.450.508
200094_s_at	1.98e-02	1.47e-02	-2.449.534

212080_at	1.98e-02	1.47e-02	-2.449.267
213280_at	1.98e-02	1.47e-02	2.449.213
202943_s_at	1.98e-02	1.47e-02	-2.449.006
218341_at	1.99e-02	1.47e-02	-2.448.813
212813_at	1.99e-02	1.47e-02	2.448.714
212750_at	1.99e-02	1.47e-02	-2.448.696
207085_x_at	1.99e-02	1.48e-02	-2.447.905
207158_at	1.99e-02	1.48e-02	-2.447.823
206989_s_at	1.99e-02	1.48e-02	-2.447.382
214008_at	2.00e-02	1.48e-02	2.446.706
216762_at	2.00e-02	1.48e-02	-2.445.752
216365_x_at	2.00e-02	1.49e-02	-2.445.152
214565_s_at	2.01e-02	1.49e-02	-2.443.903
215756_at	2.01e-02	1.49e-02	-2.443.708
220250_at	2.01e-02	1.49e-02	-2.443.416
206394_at	2.02e-02	1.50e-02	-2.441.757
203029_s_at	2.02e-02	1.50e-02	-2.441.752
211129_x_at	2.03e-02	1.50e-02	-2.441.116
217913_at	2.03e-02	1.50e-02	-2.441.109
206168_at	2.03e-02	1.50e-02	-2.440.801
216582_at	2.03e-02	1.50e-02	-2.440.768
201401_s_at	2.03e-02	1.51e-02	-2.440.522
217657_at	2.03e-02	1.51e-02	-2.440.266
203236_s_at	2.03e-02	1.51e-02	2.440.259
215830_at	2.03e-02	1.51e-02	-2.440.121
213326_at	2.03e-02	1.51e-02	2.439.612
215984_s_at	2.04e-02	1.52e-02	2.437.936
213849_s_at	2.04e-02	1.52e-02	2.437.617
222263_at	2.05e-02	1.52e-02	2.436.699
217551_at	2.05e-02	1.52e-02	2.436.564
212726_at	2.06e-02	1.53e-02	2.434.703
205921_s_at	2.06e-02	1.53e-02	-2.434.197
207568_at	2.07e-02	1.53e-02	-2.433.797
209859_at	2.07e-02	1.53e-02	2.433.705
216563_at	2.07e-02	1.53e-02	2.433.702
222007_s_at	2.07e-02	1.54e-02	-2.433.117
218658_s_at	2.07e-02	1.54e-02	2.432.805
222256_s_at	2.07e-02	1.54e-02	-2.432.491
217589_at	2.08e-02	1.54e-02	-2.431.887
215760_s_at	2.08e-02	1.54e-02	-2.431.159
32502_at	2.08e-02	1.55e-02	-2.430.867
207748_at	2.08e-02	1.55e-02	-2.430.377
203256_at	2.09e-02	1.55e-02	-2.429.789
206018_at	2.09e-02	1.55e-02	2.429.533

219529_at	2.09e-02	1.55e-02	2.429.158
206113_s_at	2.09e-02	1.55e-02	2.428.805
206522_at	2.10e-02	1.56e-02	2.428.098
210291_s_at	2.10e-02	1.56e-02	-2.428.048
215003_at	2.10e-02	1.56e-02	-2.427.762
218453_s_at	2.10e-02	1.56e-02	-2.427.164
217559_at	2.10e-02	1.56e-02	2.426.558
219875_s_at	2.10e-02	1.56e-02	-2.426.409
207455_at	2.11e-02	1.57e-02	-2.425.849
207647_at	2.11e-02	1.57e-02	-2.425.637
205770_at	2.11e-02	1.57e-02	2.425.394
218100_s_at	2.11e-02	1.57e-02	2.425.188
204722_at	2.11e-02	1.57e-02	-2.425.003
210805_x_at	2.11e-02	1.57e-02	-2.424.705
222087_at	2.12e-02	1.57e-02	2.424.243
206823_at	2.12e-02	1.57e-02	-2.424.081
215105_at	2.12e-02	1.58e-02	-2.423.839
203490_at	2.12e-02	1.58e-02	2.423.683
210403_s_at	2.12e-02	1.58e-02	-2.423.033
206765_at	2.12e-02	1.58e-02	2.422.919
213946_s_at	2.13e-02	1.58e-02	-2.422.135
201986_at	2.13e-02	1.59e-02	242.157
207918_s_at	2.14e-02	1.59e-02	2.420.778
222091_at	2.14e-02	1.59e-02	-2.420.604
215441_at	2.14e-02	1.59e-02	2.420.488
214865_at	2.14e-02	1.59e-02	-2.420.388
202605_at	2.14e-02	1.59e-02	-2.420.202
221283_at	2.14e-02	1.59e-02	-2.419.569
201030_x_at	2.14e-02	1.60e-02	-2.419.087
215878_at	2.14e-02	1.60e-02	-2.419.073
204440_at	2.15e-02	1.60e-02	-2.418.862
215638_at	2.15e-02	1.60e-02	-2.418.786
219601_s_at	2.15e-02	1.60e-02	-2.418.377
206573_at	2.15e-02	1.60e-02	-2.418.182
209488_s_at	2.15e-02	1.60e-02	2.417.562
221687_s_at	2.15e-02	1.60e-02	-241.739
219180_s_at	2.15e-02	1.60e-02	2.417.263
204357_s_at	2.16e-02	1.61e-02	2.416.582
212294_at	2.16e-02	1.61e-02	-2.416.542
213569_at	2.16e-02	1.61e-02	-241.603
215196_at	2.16e-02	1.61e-02	-2.415.997
217636_at	2.16e-02	1.61e-02	-2.415.814
221128_at	2.16e-02	1.61e-02	-2.415.697
205920_at	2.16e-02	1.61e-02	-2.415.642

205734_s_at	2.17e-02	1.62e-02	-2.413.556
215634_at	2.18e-02	1.63e-02	-2.412.294
206367_at	2.19e-02	1.63e-02	2.411.526
220637_at	2.19e-02	1.63e-02	2.410.787
203541_s_at	2.20e-02	1.64e-02	-2.409.798
215993_at	2.20e-02	1.64e-02	-2.409.761
221033_s_at	2.20e-02	1.64e-02	-2.409.705
208426_x_at	2.20e-02	1.64e-02	-2.409.577
204815_s_at	2.20e-02	1.64e-02	-2.409.558
202328_s_at	2.20e-02	1.64e-02	-2.409.154
215242_at	2.20e-02	1.64e-02	-2.408.518
222106_at	2.21e-02	1.65e-02	2.407.299
217537_x_at	2.21e-02	1.65e-02	-2.407.074
201194_at	2.21e-02	1.65e-02	-2.406.687
212103_at	2.21e-02	1.65e-02	2.406.582
214184_at	2.22e-02	1.65e-02	-2.406.361
213888_s_at	2.22e-02	1.65e-02	-2.405.832
211917_s_at	2.22e-02	1.65e-02	2.405.668
204082_at	2.23e-02	1.66e-02	2.404.363
201126_s_at	2.23e-02	1.66e-02	2.404.118
207028_at	2.23e-02	1.67e-02	-240.332
215021_s_at	2.23e-02	1.67e-02	2.403.169
215720_s_at	2.23e-02	1.67e-02	2.403.059
206545_at	2.24e-02	1.67e-02	2.402.759
217298_at	2.24e-02	1.67e-02	-2.402.555
220323_at	2.24e-02	1.67e-02	-240.249
213956_at	2.24e-02	1.67e-02	2.401.431
209474_s_at	2.25e-02	1.68e-02	240.081
201449_at	2.25e-02	1.68e-02	240.053
209062_x_at	2.25e-02	1.68e-02	2.400.005
210717_at	2.25e-02	1.68e-02	-2.399.887
216543_at	2.25e-02	1.68e-02	-2.399.597
212636_at	2.26e-02	1.68e-02	2.399.409
220025_at	2.26e-02	1.69e-02	-2.398.241
203807_x_at	2.27e-02	1.69e-02	-2.397.408
200903_s_at	2.27e-02	1.69e-02	2.397.267
205052_at	2.27e-02	1.69e-02	-2.397.236
215356_at	2.27e-02	1.69e-02	-2.397.125
213823_at	2.27e-02	1.70e-02	-2.396.523
220899_at	2.27e-02	1.70e-02	-2.396.367
214357_at	2.28e-02	1.70e-02	-2.395.051
220795_s_at	2.28e-02	1.71e-02	-2.394.439
213889_at	2.29e-02	1.71e-02	2.394.136
214911_s_at	2.29e-02	1.71e-02	-239.403

206577_at	2.29e-02	1.71e-02	-2.393.781
211039_at	2.29e-02	1.71e-02	-239.292
36566_at	2.30e-02	1.72e-02	2.392.106
209939_x_at	2.30e-02	1.72e-02	-2.391.321
209812_x_at	2.30e-02	1.72e-02	-239.125
208109_s_at	2.30e-02	1.72e-02	2.390.958
214591_at	2.31e-02	1.72e-02	-2.390.782
47550_at	2.31e-02	1.72e-02	239.046
212980_at	2.31e-02	1.73e-02	-2.389.896
203047_at	2.31e-02	1.73e-02	-2.389.688
203903_s_at	2.32e-02	1.73e-02	-2.388.883
219741_x_at	2.32e-02	1.73e-02	2.388.853
206830_at	2.32e-02	1.73e-02	-2.388.438
209611_s_at	2.32e-02	1.73e-02	2.388.325
202877_s_at	2.32e-02	1.73e-02	2.388.245
206127_at	2.32e-02	1.74e-02	-2.387.727
208080_at	2.32e-02	1.74e-02	-2.387.716
216234_s_at	2.33e-02	1.75e-02	-2.385.894
219151_s_at	2.33e-02	1.75e-02	2.385.864
220885_s_at	2.34e-02	1.75e-02	-2.384.921
219262_at	2.34e-02	1.75e-02	2.384.641
216651_s_at	2.34e-02	1.75e-02	-2.384.431
200762_at	2.34e-02	1.75e-02	-2.384.114
217212_s_at	2.34e-02	1.75e-02	-2.384.105
214332_s_at	2.35e-02	1.75e-02	-2.383.907
207706_at	2.35e-02	1.76e-02	2.383.798
211668_s_at	2.35e-02	1.76e-02	2.383.593
205663_at	2.35e-02	1.76e-02	-2.382.625
218527_at	2.36e-02	1.76e-02	2.382.024
220504_at	2.36e-02	1.76e-02	-2.381.951
214533_at	2.36e-02	1.77e-02	-2.381.228
206053_at	2.36e-02	1.77e-02	2.381.002
209489_at	2.36e-02	1.77e-02	-2.380.943
202610_s_at	2.37e-02	1.77e-02	238.066
215544_s_at	2.37e-02	1.77e-02	-2.380.565
218246_at	2.37e-02	1.77e-02	2.380.321
220259_at	2.37e-02	1.77e-02	-237.967
221405_at	2.37e-02	1.78e-02	-2.379.526
212254_s_at	2.38e-02	1.78e-02	-2.378.428
204464_s_at	2.39e-02	1.79e-02	2.377.332
209261_s_at	2.39e-02	1.79e-02	2.376.618
213963_s_at	2.39e-02	1.79e-02	-2.376.372
219719_at	2.39e-02	1.79e-02	2.375.828
208670_s_at	2.40e-02	1.80e-02	2.374.918

209457_at	2.40e-02	1.80e-02	-2.374.652
212463_at	2.40e-02	1.80e-02	2.374.639
221322_at	2.40e-02	1.80e-02	-2.374.414
219785_s_at	2.40e-02	1.80e-02	-237.431
216431_at	2.40e-02	1.80e-02	-2.374.296
203052_at	2.41e-02	1.80e-02	-2.374.034
210382_at	2.41e-02	1.80e-02	-2.373.966
220424_at	2.41e-02	1.81e-02	-2.373.335
219025_at	2.41e-02	1.81e-02	-2.373.316
221653_x_at	2.41e-02	1.81e-02	-2.373.261
220126_at	2.41e-02	1.81e-02	-2.372.774
216352_x_at	2.42e-02	1.81e-02	-2.372.319
203518_at	2.42e-02	1.81e-02	2.371.953
211866_x_at	2.42e-02	1.82e-02	-237.127
213564_x_at	2.43e-02	1.82e-02	-2.370.409
220561_at	2.43e-02	1.82e-02	-2.370.226
212360_at	2.43e-02	1.82e-02	-2.369.731
216445_at	2.44e-02	1.83e-02	-2.368.938
209661_at	2.44e-02	1.83e-02	-2.368.866
222350_at	2.44e-02	1.83e-02	-2.368.545
219073_s_at	2.44e-02	1.83e-02	2.368.232
221070_s_at	2.44e-02	1.83e-02	-2.368.222
204423_at	2.44e-02	1.83e-02	2.367.981
201789_at	2.44e-02	1.83e-02	-2.367.663
214925_s_at	2.45e-02	1.84e-02	-236.711
210079_x_at	2.45e-02	1.84e-02	-2.366.503
206517_at	2.45e-02	1.84e-02	-2.366.376
203591_s_at	2.45e-02	1.84e-02	-2.366.079
211199_s_at	2.45e-02	1.84e-02	-2.365.951
203537_at	2.45e-02	1.84e-02	2.365.886
208686_s_at	2.46e-02	1.84e-02	-2.365.695
210355_at	2.46e-02	1.84e-02	2.365.379
219547_at	2.46e-02	1.85e-02	2.364.561
222015_at	2.47e-02	1.85e-02	2.363.774
209721_s_at	2.47e-02	1.85e-02	2.363.254
210864_x_at	2.47e-02	1.85e-02	-2.363.234
221944_at	2.48e-02	1.86e-02	-2.362.471
216681_at	2.48e-02	1.86e-02	-2.362.242
218421_at	2.48e-02	1.86e-02	2.362.188
218606_at	2.48e-02	1.86e-02	-2.362.001
213217_at	2.48e-02	1.86e-02	-2.361.604
211752_s_at	2.49e-02	1.87e-02	-2.360.706
214644_at	2.49e-02	1.87e-02	-235.998
205317_s_at	2.49e-02	1.87e-02	-2.359.752

220653_at	2.49e-02	1.87e-02	2.359.653
202239_at	2.49e-02	1.87e-02	2.359.541
207333_at	2.50e-02	1.87e-02	-2.359.164
210833_at	2.50e-02	1.88e-02	-2.358.673
37996_s_at	2.50e-02	1.88e-02	-2.358.479
213477_x_at	2.50e-02	1.88e-02	2.358.372
201395_at	2.50e-02	1.88e-02	-2.357.802
210345_s_at	2.51e-02	1.88e-02	-2.357.732
207635_s_at	2.51e-02	1.88e-02	-2.357.581
207293_s_at	2.51e-02	1.89e-02	-2.357.073
206308_at	2.51e-02	1.89e-02	-2.356.827
214242_at	2.51e-02	1.89e-02	-2.356.739
214922_at	2.51e-02	1.89e-02	-2.356.657
216144_at	2.52e-02	1.89e-02	-2.355.479
206491_s_at	2.52e-02	1.89e-02	-2.355.109
210035_s_at	2.52e-02	1.90e-02	-2.354.825
201195_s_at	2.52e-02	1.90e-02	-2.354.719
202466_at	2.52e-02	1.90e-02	-2.354.541
218191_s_at	2.53e-02	1.90e-02	-235.414
213638_at	2.53e-02	1.90e-02	-2.353.926
220817_at	2.53e-02	1.90e-02	2.353.569
207552_at	2.53e-02	1.91e-02	2.353.078
214286_at	2.54e-02	1.91e-02	-2.352.863
222346_at	2.54e-02	1.91e-02	-2.352.599
221675_s_at	2.54e-02	1.91e-02	2.351.902
200605_s_at	2.55e-02	1.92e-02	2.351.053
210531_at	2.55e-02	1.92e-02	-2.350.547
214762_at	2.55e-02	1.92e-02	-2.349.912
214525_x_at	2.56e-02	1.92e-02	2.349.791
218953_s_at	2.56e-02	1.92e-02	2.349.434
216712_at	2.56e-02	1.93e-02	-2.348.921
216873_s_at	2.56e-02	1.93e-02	2.348.892
220374_at	2.56e-02	1.93e-02	-2.348.657
200945_s_at	2.57e-02	1.93e-02	2.348.016
222164_at	2.57e-02	1.93e-02	2.347.237
207770_x_at	2.58e-02	1.94e-02	-2.346.235
212629_s_at	2.58e-02	1.94e-02	-2.345.588
214165_s_at	2.58e-02	1.94e-02	-2.345.533
222173_s_at	2.59e-02	1.95e-02	-2.345.033
221456_at	2.59e-02	1.95e-02	-2.344.825
218655_s_at	2.59e-02	1.95e-02	-2.344.759
217224_at	2.59e-02	1.95e-02	-2.344.083
220389_at	2.59e-02	1.95e-02	-2.344.037
214850_at	2.59e-02	1.95e-02	2.343.794

218572_at	2.60e-02	1.96e-02	2.343.063
212468_at	2.60e-02	1.96e-02	-2.342.914
210349_at	2.61e-02	1.96e-02	-23.418
207996_s_at	2.61e-02	1.96e-02	234.165
209937_at	2.61e-02	1.96e-02	2.341.628
204804_at	2.61e-02	1.97e-02	2.341.244
212207_at	2.61e-02	1.97e-02	2.340.706
212442_s_at	2.62e-02	1.97e-02	234.022
220378_at	2.62e-02	1.97e-02	-2.339.839
211368_s_at	2.62e-02	1.97e-02	-2.339.725
203106_s_at	2.63e-02	1.98e-02	2.338.686
214905_at	2.63e-02	1.98e-02	-2.338.372
203945_at	2.63e-02	1.98e-02	-2.338.145
211095_at	2.63e-02	1.98e-02	-2.338.011
204102_s_at	2.63e-02	1.98e-02	-2.337.644
204272_at	2.63e-02	1.98e-02	2.337.613
221275_s_at	2.64e-02	1.99e-02	2.337.214
213210_at	2.64e-02	1.99e-02	-2.337.032
212904_at	2.64e-02	1.99e-02	-2.336.938
210817_s_at	2.64e-02	1.99e-02	-23.365
214761_at	2.65e-02	1.99e-02	2.335.811
201581_at	2.65e-02	1.99e-02	2.335.747
219609_at	2.65e-02	2.00e-02	-2.335.633
216375_s_at	2.66e-02	2.01e-02	2.333.245
206777_s_at	2.67e-02	2.01e-02	2.332.726
206164_at	2.67e-02	2.01e-02	-2.332.695
211903_s_at	2.67e-02	2.01e-02	-2.332.261
200656_s_at	2.67e-02	2.02e-02	233.156
209958_s_at	2.68e-02	2.02e-02	2.331.339
208223_s_at	2.69e-02	2.03e-02	-2.329.727
206847_s_at	2.69e-02	2.03e-02	-2.329.715
218880_at	2.69e-02	2.03e-02	2.329.071
208966_x_at	2.70e-02	2.03e-02	-2.328.316
208604_s_at	2.70e-02	2.04e-02	-2.328.058
207182_at	2.70e-02	2.04e-02	-2.327.877
205731_s_at	2.70e-02	2.04e-02	2.327.842
215913_s_at	2.70e-02	2.04e-02	2.327.276
215881_x_at	2.71e-02	2.05e-02	2.325.973
219573_at	2.72e-02	2.05e-02	232.557
217374_x_at	2.72e-02	2.05e-02	-2.325.292
222356_at	2.72e-02	2.05e-02	-232.521
206786_at	2.72e-02	2.05e-02	2.325.023
209170_s_at	2.73e-02	2.06e-02	-2.324.142
219939_s_at	2.73e-02	2.06e-02	232.366

221174_at	2.73e-02	2.06e-02	-2.323.444
204591_at	2.73e-02	2.06e-02	2.323.049
204735_at	2.74e-02	2.07e-02	2.322.459
212806_at	2.74e-02	2.07e-02	2.322.031
203769_s_at	2.74e-02	2.07e-02	-232.138
207381_at	2.75e-02	2.08e-02	-2.320.283
214213_x_at	2.76e-02	2.08e-02	-231.972
215740_at	2.76e-02	2.08e-02	-231.959
203559_s_at	2.76e-02	2.08e-02	-2.319
215500_at	2.76e-02	2.09e-02	-2.318.867
221626_at	2.76e-02	2.09e-02	2.318.434
214109_at	2.76e-02	2.09e-02	-2.318.413
219908_at	2.76e-02	2.09e-02	2.318.344
219836_at	2.77e-02	2.09e-02	2.317.936
202598_at	2.77e-02	2.09e-02	2.317.293
210760_x_at	2.77e-02	2.10e-02	-2.317.026
207558_s_at	2.77e-02	2.10e-02	2.316.915
218551_at	2.78e-02	2.10e-02	2.316.586
210647_x_at	2.78e-02	2.10e-02	2.316.557
207703_at	2.78e-02	2.10e-02	2.316.404
219444_at	2.78e-02	2.10e-02	2.315.526
211514_at	2.78e-02	2.10e-02	-2.315.503
211193_at	2.79e-02	2.11e-02	-2.315.286
205713_s_at	2.79e-02	2.11e-02	2.315.044
220223_at	2.79e-02	2.11e-02	231.484
203910_at	2.79e-02	2.11e-02	-2.313.884
204924_at	2.80e-02	2.11e-02	-2.313.691
219968_at	2.80e-02	2.12e-02	-2.313.116
209191_at	2.80e-02	2.12e-02	2.312.952
221093_at	2.80e-02	2.12e-02	-231.291
210069_at	2.80e-02	2.12e-02	2.312.895
207232_s_at	2.81e-02	2.12e-02	2.312.182
215790_at	2.81e-02	2.12e-02	-2.311.847
209941_at	2.82e-02	2.13e-02	2.309.981
221108_at	2.82e-02	2.14e-02	-230.979
200881_s_at	2.83e-02	2.14e-02	2.309.538
216551_x_at	2.83e-02	2.14e-02	-2.309.022
202924_s_at	2.83e-02	2.14e-02	-2.308.978
207346_at	2.83e-02	2.14e-02	-23.085
217349_s_at	2.83e-02	2.14e-02	2.308.411
216566_at	2.83e-02	2.14e-02	-2.308.315
216437_at	2.84e-02	2.15e-02	-2.308.011
201313_at	2.84e-02	2.15e-02	2.306.997
78330_at	2.85e-02	2.16e-02	-2.306.072

218647_s_at	2.85e-02	2.16e-02	2.305.473
208630_at	2.86e-02	2.17e-02	-2.304.411
212315_s_at	2.87e-02	2.17e-02	2.303.188
213178_s_at	2.87e-02	2.17e-02	-2.303.033
212752_at	2.88e-02	2.18e-02	-2.302.408
221414_s_at	2.88e-02	2.18e-02	-2.302.231
222040_at	2.88e-02	2.18e-02	-2.301.617
220159_at	2.89e-02	2.18e-02	-2.301.113
209816_at	2.89e-02	2.19e-02	-2.300.117
217547_x_at	2.90e-02	2.19e-02	2.299.631
220936_s_at	2.90e-02	2.19e-02	2.299.511
31846_at	2.90e-02	2.19e-02	-2.299.359
205432_at	2.90e-02	2.20e-02	-2.299.035
210912_x_at	2.92e-02	2.21e-02	-2.296.802
219366_at	2.92e-02	2.21e-02	2.296.262
217028_at	2.92e-02	2.21e-02	-2.295.997
215656_at	2.93e-02	2.22e-02	-2.295.073
214866_at	2.93e-02	2.22e-02	-2.294.954
222131_x_at	2.93e-02	2.22e-02	-2.294.798
216091_s_at	2.93e-02	2.22e-02	-2.294.789
204672_s_at	2.93e-02	2.22e-02	229.463
207099_s_at	2.93e-02	2.22e-02	2.294.452
219645_at	2.93e-02	2.22e-02	-2.294.347
201320_at	2.94e-02	2.22e-02	2.294.189
205153_s_at	2.94e-02	2.22e-02	-229.412
208138_at	2.94e-02	2.23e-02	-2.293.533
215565_at	2.94e-02	2.23e-02	-2.293.168
219699_at	2.95e-02	2.23e-02	-2.292.467
214993_at	2.95e-02	2.24e-02	2.291.826
217261_at	2.95e-02	2.24e-02	-2.291.513
219872_at	2.95e-02	2.24e-02	-2.291.486
207101_at	2.96e-02	2.24e-02	-2.291.321
213307_at	2.96e-02	2.24e-02	229.108
210367_s_at	2.96e-02	2.24e-02	2.290.819
210047_at	2.96e-02	2.24e-02	2.290.711
209767_s_at	2.96e-02	2.25e-02	2.290.591
206427_s_at	2.96e-02	2.25e-02	-229.021
212524_x_at	2.96e-02	2.25e-02	-229.011
201961_s_at	2.97e-02	2.25e-02	2.289.961
212884_x_at	2.97e-02	2.25e-02	-2.289.934
216918_s_at	2.97e-02	2.25e-02	-2.289.851
210341_at	2.97e-02	2.25e-02	-2.289.106
204842_x_at	2.97e-02	2.25e-02	-2.289.067
210193_at	2.97e-02	2.26e-02	-2.288.878

59705_at	2.98e-02	2.26e-02	2.288.451
211123_at	2.98e-02	2.26e-02	-2.288.037
220681_at	2.98e-02	2.26e-02	-2.287.967
218333_at	2.98e-02	2.26e-02	2.287.768
207710_at	2.99e-02	2.27e-02	-2.286.463
219319_at	2.99e-02	2.27e-02	-2.286.094
203070_at	2.99e-02	2.27e-02	-2.285.969
203933_at	3.00e-02	2.28e-02	-2.285.435
205418_at	3.01e-02	2.28e-02	-2.284.253
214540_at	3.01e-02	2.28e-02	2.284.032
202117_at	3.01e-02	2.28e-02	2.283.924
206783_at	3.02e-02	2.29e-02	-2.282.745
217621_at	3.02e-02	2.29e-02	2.282.727
201806_s_at	3.02e-02	2.29e-02	-2.282.423
216142_at	3.02e-02	2.29e-02	-2.282.166
208296_x_at	3.02e-02	2.30e-02	2.282.157
208137_x_at	3.02e-02	2.30e-02	-2.282.057
219690_at	3.02e-02	2.30e-02	-2.281.965
200954_at	3.03e-02	2.30e-02	2.280.494
204248_at	3.03e-02	2.31e-02	2.280.434
206156_at	3.04e-02	2.31e-02	-2.280.346
213115_at	3.04e-02	2.31e-02	2.280.082
220261_s_at	3.04e-02	2.31e-02	2.279.973
204791_at	3.04e-02	2.31e-02	-2.279.095
207923_x_at	3.05e-02	2.32e-02	-2.278.708
214854_at	3.05e-02	2.32e-02	-2.278.266
208669_s_at	3.06e-02	2.32e-02	2.277.702
212777_at	3.06e-02	2.32e-02	227.765
218451_at	3.06e-02	2.33e-02	2.277.149
205481_at	3.06e-02	2.33e-02	-2.277.085
204623_at	3.06e-02	2.33e-02	227.663
205567_at	3.07e-02	2.33e-02	2.276.213
212662_at	3.07e-02	2.33e-02	2.276.049
222261_at	3.07e-02	2.33e-02	-2.275.832
208338_at	3.07e-02	2.33e-02	-2.275.712
207749_s_at	3.07e-02	2.34e-02	-2.275.045
204210_s_at	3.08e-02	2.34e-02	2.274.754
214070_s_at	3.08e-02	2.34e-02	-2.274.693
202832_at	3.08e-02	2.34e-02	-2.274.643
206784_at	3.08e-02	2.34e-02	2.274.573
201045_s_at	3.08e-02	2.34e-02	-2.274.537
201208_s_at	3.08e-02	2.34e-02	-2.274.374
91703_at	3.08e-02	2.34e-02	2.274.232
214798_at	3.08e-02	2.34e-02	-2.273.977

205608_s_at	3.08e-02	2.35e-02	2.273.751
216117_at	3.08e-02	2.35e-02	-2.273.628
219070_s_at	3.09e-02	2.35e-02	2.273.262
217293_at	3.09e-02	2.35e-02	-2.272.803
216781_at	3.11e-02	2.36e-02	-2.270.651
202793_at	3.11e-02	2.37e-02	2.270.539
216790_at	3.11e-02	2.37e-02	-2.270.531
204278_s_at	3.11e-02	2.37e-02	2.270.043
220698_at	3.12e-02	2.37e-02	-2.269.517
210945_at	3.12e-02	2.37e-02	-2.269.065
210433_at	3.12e-02	2.37e-02	-2.269.064
209782_s_at	3.12e-02	2.38e-02	2.268.512
200866_s_at	3.13e-02	2.38e-02	2.267.902
212192_at	3.13e-02	2.38e-02	-2.267.515
214787_at	3.13e-02	2.38e-02	2.267.476
218058_at	3.13e-02	2.39e-02	2.267.248
200006_at	3.14e-02	2.39e-02	2.266.096
203837_at	3.14e-02	2.39e-02	-2.266.005
213713_s_at	3.15e-02	2.40e-02	2.265.475
206232_s_at	3.15e-02	2.40e-02	2.265.234
216850_at	3.15e-02	2.40e-02	2.265.121
212297_at	3.15e-02	2.40e-02	-2.265.022
216353_s_at	3.15e-02	2.40e-02	-2.264.902
205905_s_at	3.16e-02	2.40e-02	-2.264.271
214129_at	3.16e-02	2.40e-02	-2.264.267
209327_s_at	3.16e-02	2.41e-02	-2.263.856
215528_at	3.16e-02	2.41e-02	2.263.771
219201_s_at	3.16e-02	2.41e-02	2.263.224
632_at	3.16e-02	2.41e-02	2.263.207
203183_s_at	3.17e-02	2.41e-02	2.262.914
206136_at	3.17e-02	2.42e-02	226.181
211347_at	3.18e-02	2.42e-02	-2.261.534
214638_s_at	3.18e-02	2.42e-02	-226.112
207465_at	3.18e-02	2.43e-02	-2.260.432
201482_at	3.19e-02	2.43e-02	-2.259.318
210290_at	3.19e-02	2.43e-02	2.259.268
207872_s_at	3.20e-02	2.44e-02	-2.258.788
205653_at	3.20e-02	2.44e-02	-2.258.619
215819_s_at	3.21e-02	2.45e-02	2.257.503
206578_at	3.21e-02	2.45e-02	-2.257.375
215558_at	3.21e-02	2.45e-02	-2.257.329
218959_at	3.21e-02	2.45e-02	225.684
210516_at	3.23e-02	2.46e-02	-225.512
217211_at	3.23e-02	2.46e-02	2.254.678

209585_s_at	3.24e-02	2.47e-02	2.254.047
207364_at	3.24e-02	2.47e-02	-2.253.049
205581_s_at	3.24e-02	2.48e-02	-2.252.907
206423_at	3.25e-02	2.48e-02	-2.252.672
212211_at	3.25e-02	2.48e-02	2.252.528
212310_at	3.25e-02	2.48e-02	-2.252.501
208785_s_at	3.25e-02	2.48e-02	2.252.313
220991_s_at	3.25e-02	2.48e-02	-2.252.232
213614_x_at	3.25e-02	2.48e-02	2.251.639
216363_at	3.26e-02	2.49e-02	-225.108
217951_s_at	3.26e-02	2.49e-02	2.251.065
216255_s_at	3.26e-02	2.49e-02	-2.250.608
203886_s_at	3.27e-02	2.49e-02	-2.250.116
209908_s_at	3.27e-02	2.49e-02	-2.249.993
203134_at	3.27e-02	2.50e-02	-2.249.198
210794_s_at	3.28e-02	2.50e-02	2.248.772
221855_at	3.28e-02	2.50e-02	2.248.343
220384_at	3.28e-02	2.51e-02	-2.247.979
214484_s_at	3.28e-02	2.51e-02	2.247.972
206987_x_at	3.28e-02	2.51e-02	-2.247.748
202657_s_at	3.29e-02	2.51e-02	2.247.496
205632_s_at	3.29e-02	2.51e-02	2.247.006
206317_s_at	3.29e-02	2.51e-02	-224.683
200706_s_at	3.30e-02	2.52e-02	-2.246.363
216305_s_at	3.30e-02	2.52e-02	-2.246.354
206620_at	3.30e-02	2.52e-02	-2.246.324
206566_at	3.30e-02	2.52e-02	-2.246.162
219260_s_at	3.30e-02	2.52e-02	2.245.874
222247_at	3.30e-02	2.52e-02	-2.245.822
217594_at	3.30e-02	2.52e-02	2.245.585
203772_at	3.30e-02	2.52e-02	-2.245.178
214183_s_at	3.31e-02	2.53e-02	2.245.015
221225_at	3.31e-02	2.53e-02	-2.244.687
216896_at	3.31e-02	2.53e-02	-2.244.559
203237_s_at	3.31e-02	2.53e-02	-2.244.008
218702_at	3.32e-02	2.54e-02	2.242.901
203698_s_at	3.33e-02	2.54e-02	2.242.197
216322_at	3.33e-02	2.55e-02	2.241.979
220853_at	3.33e-02	2.55e-02	-2.241.846
220190_s_at	3.33e-02	2.55e-02	-224.144
210603_at	3.34e-02	2.55e-02	224.118
216565_x_at	3.34e-02	2.55e-02	2.240.544
216298_at	3.34e-02	2.56e-02	-2.240.452
214675_at	3.34e-02	2.56e-02	-2.240.348

217242_at	3.36e-02	2.57e-02	-2.238.719
213871_s_at	3.36e-02	2.57e-02	-2.237.971
40149_at	3.37e-02	2.57e-02	-223.762
203205_at	3.37e-02	2.57e-02	2.237.533
201919_at	3.37e-02	2.58e-02	-2.236.543
222156_x_at	3.38e-02	2.59e-02	-2.235.664
221333_at	3.39e-02	2.59e-02	-2.235.023
204911_s_at	3.39e-02	2.60e-02	-2.234.307
212386_at	3.40e-02	2.60e-02	-2.233.865
212159_x_at	3.40e-02	2.60e-02	2.233.511
212420_at	3.40e-02	2.60e-02	-2.233.014
217003_s_at	3.41e-02	2.61e-02	-2.232.492
210483_at	3.41e-02	2.61e-02	-223.178
209451_at	3.42e-02	2.62e-02	-2.231.368
207049_at	3.42e-02	2.62e-02	-22.309
207946_at	3.42e-02	2.62e-02	-2.230.886
210620_s_at	3.42e-02	2.62e-02	2.230.602
214661_s_at	3.42e-02	2.62e-02	2.230.553
215599_at	3.42e-02	2.62e-02	2.230.525
217310_s_at	3.42e-02	2.62e-02	-2.230.432
206740_x_at	3.43e-02	2.62e-02	-2.230.316
207467_x_at	3.43e-02	2.63e-02	2.229.269
217573_at	3.44e-02	2.63e-02	-2.228.958
215510_at	3.44e-02	2.63e-02	-2.228.944
205068_s_at	3.44e-02	2.63e-02	2.228.941
214569_at	3.44e-02	2.63e-02	-2.228.671
214996_at	3.46e-02	2.65e-02	2.226.707
213012_at	3.46e-02	2.65e-02	2.226.521
202867_s_at	3.47e-02	2.66e-02	2.225.434
200851_s_at	3.47e-02	2.66e-02	2.225.405
214781_at	3.47e-02	2.66e-02	-2.225.277
213365_at	3.47e-02	2.66e-02	2.224.753
207034_s_at	3.47e-02	2.66e-02	-2.224.491
211327_x_at	3.48e-02	2.67e-02	2.223.745
211222_s_at	3.49e-02	2.68e-02	-2.222.345
218685_s_at	3.50e-02	2.68e-02	2.221.857
207078_at	3.50e-02	2.68e-02	-2.221.764
205473_at	3.50e-02	2.68e-02	-2.221.053
215980_s_at	3.51e-02	2.69e-02	-2.220.481
221762_s_at	3.51e-02	2.69e-02	2.220.049
219127_at	3.51e-02	2.69e-02	2.219.859
220673_s_at	3.52e-02	2.70e-02	2.218.839
40687_at	3.53e-02	2.71e-02	-2.217.505
211698_at	3.53e-02	2.71e-02	2.217.493

220845_at	3.54e-02	2.71e-02	-2.217.143
218186_at	3.54e-02	2.71e-02	-2.216.976
211387_x_at	3.54e-02	2.71e-02	-2.216.949
209227_at	3.54e-02	2.71e-02	-2.216.811
220779_at	3.54e-02	2.72e-02	-2.216.517
214479_at	3.54e-02	2.72e-02	-2.216.296
204181_s_at	3.56e-02	2.73e-02	-2.214.911
215536_at	3.56e-02	2.73e-02	-2.214.357
215805_at	3.56e-02	2.73e-02	-2.214.045
216393_at	3.57e-02	2.74e-02	-2.213.431
218248_at	3.57e-02	2.74e-02	2.213.317
204533_at	3.57e-02	2.74e-02	2.213.093
219951_s_at	3.57e-02	2.74e-02	-221.305
216594_x_at	3.58e-02	2.75e-02	2.211.836
207451_at	3.58e-02	2.75e-02	-2.211.777
217923_at	3.58e-02	2.75e-02	2.211.501
210995_s_at	3.59e-02	2.75e-02	2.211.347
216933_x_at	3.59e-02	2.75e-02	-22.109
203723_at	3.59e-02	2.76e-02	-2.210.397
206309_at	3.60e-02	2.76e-02	-2.209.579
205641_s_at	3.61e-02	2.77e-02	-2.208.952
214355_x_at	3.61e-02	2.77e-02	-2.208.586
209926_at	3.61e-02	2.77e-02	-2.208.039
211993_at	3.62e-02	2.78e-02	2.207.588
203650_at	3.62e-02	2.78e-02	-2.207.055
222321_at	3.63e-02	2.78e-02	-2.206.794
208848_at	3.63e-02	2.79e-02	-2.206.262
204530_s_at	3.63e-02	2.79e-02	-2.206.027
205096_at	3.63e-02	2.79e-02	-2.205.919
209100_at	3.64e-02	2.79e-02	2.205.597
207602_at	3.64e-02	2.80e-02	-22.046
209324_s_at	3.65e-02	2.80e-02	-2.204.483
216093_at	3.65e-02	2.80e-02	-2.204.369
205443_at	3.65e-02	2.80e-02	2.204.294
207638_at	3.65e-02	2.80e-02	2.204.193
211130_x_at	3.65e-02	2.80e-02	-2.203.943
221454_at	3.65e-02	2.80e-02	-2.203.915
216166_at	3.67e-02	2.82e-02	-2.201.593
221049_s_at	3.67e-02	2.82e-02	-2.201.458
206062_at	3.67e-02	2.82e-02	-2.201.449
214410_at	3.67e-02	2.82e-02	-2.201.345
204724_s_at	3.67e-02	2.82e-02	-2.201.316
206717_at	3.67e-02	2.82e-02	-2.201.074
221420_at	3.68e-02	2.83e-02	-2.200.926

202206_at	3.68e-02	2.83e-02	-2.200.073
203471_s_at	3.68e-02	2.83e-02	-219.994
31861_at	3.69e-02	2.83e-02	-2.199.654
220124_at	3.69e-02	2.84e-02	2.199.419
219241_x_at	3.69e-02	2.84e-02	2.198.772
221361_at	3.69e-02	2.84e-02	-2.198.737
220414_at	3.70e-02	2.84e-02	-219.852
219409_at	3.70e-02	2.85e-02	2.197.981
34031_i_at	3.70e-02	2.85e-02	2.197.808
214216_s_at	3.71e-02	2.86e-02	-2.196.774
208874_x_at	3.71e-02	2.86e-02	-2.196.747
219767_s_at	3.72e-02	2.86e-02	2.196.232
207510_at	3.73e-02	2.87e-02	-2.194.982
202342_s_at	3.73e-02	2.87e-02	-2.194.743
211462_s_at	3.73e-02	2.87e-02	-2.194.426
208522_s_at	3.74e-02	2.87e-02	-2.194.048
205421_at	3.74e-02	2.88e-02	-2.193.894
220426_at	3.74e-02	2.88e-02	-2.193.558
217517_x_at	3.75e-02	2.88e-02	2.193.034
207858_s_at	3.75e-02	2.88e-02	-2.192.721
212654_at	3.75e-02	2.89e-02	2.192.293
214639_s_at	3.76e-02	2.89e-02	-2.191.479
213751_at	3.76e-02	2.89e-02	-2.191.377
221147_x_at	3.77e-02	2.90e-02	-219.028
203709_at	3.78e-02	2.91e-02	-2.189.739
216732_at	3.78e-02	2.91e-02	-2.189.718
214620_x_at	3.78e-02	2.91e-02	2.189.096
202640_s_at	3.78e-02	2.91e-02	2.189.003
35436_at	3.78e-02	2.91e-02	-2.188.787
207822_at	3.78e-02	2.91e-02	-2.188.783
208074_s_at	3.79e-02	2.92e-02	2.188.413
215988_s_at	3.79e-02	2.92e-02	-2.188.211
219571_s_at	3.79e-02	2.92e-02	2.187.879
207192_at	3.79e-02	2.92e-02	-2.187.863
214127_s_at	3.80e-02	2.93e-02	-2.186.905
206343_s_at	3.80e-02	2.93e-02	-2.186.864
201501_s_at	3.80e-02	2.93e-02	218.654
208136_s_at	3.81e-02	2.93e-02	-2.185.984
215417_at	3.81e-02	2.94e-02	-2.185.722
207456_at	3.82e-02	2.95e-02	-2.184.306
217037_at	3.83e-02	2.95e-02	-2.184.093
221103_s_at	3.83e-02	2.95e-02	2.184.091
208566_at	3.83e-02	2.95e-02	-2.183.451
210551_s_at	3.83e-02	2.95e-02	-2.183.158

212036_s_at	3.84e-02	2.96e-02	-21.828
207452_s_at	3.85e-02	2.96e-02	2.181.877
221932_s_at	3.85e-02	2.97e-02	2.181.688
217339_x_at	3.85e-02	2.97e-02	2.181.403
209595_at	3.86e-02	2.97e-02	-2.180.843
206407_s_at	3.86e-02	2.97e-02	-2.180.771
221880_s_at	3.86e-02	2.97e-02	2.180.533
214871_x_at	3.86e-02	2.98e-02	2.180.372
211716_x_at	3.86e-02	2.98e-02	2.180.233
207059_at	3.86e-02	2.98e-02	-2.179.789
206463_s_at	3.87e-02	2.98e-02	-2.179.645
210924_at	3.87e-02	2.99e-02	-2.179.049
211930_at	3.87e-02	2.99e-02	2.179
209110_s_at	3.87e-02	2.99e-02	2.178.938
201735_s_at	3.87e-02	2.99e-02	2.178.813
211169_s_at	3.88e-02	2.99e-02	-2.178.231
213659_at	3.88e-02	2.99e-02	-2.178.178
214755_at	3.88e-02	2.99e-02	2.178.163
219892_at	3.88e-02	3.00e-02	2.177.694
33767_at	3.89e-02	3.00e-02	2.177.298
207257_at	3.89e-02	3.00e-02	2.177.225
215704_at	3.89e-02	3.00e-02	-2.176.983
221353_at	3.90e-02	3.01e-02	-2.176.235
214529_at	3.90e-02	3.01e-02	-2.175.915
210242_x_at	3.90e-02	3.01e-02	2.175.678
203613_s_at	3.90e-02	3.01e-02	2.175.257
219966_x_at	3.91e-02	3.02e-02	2.174.403
200628_s_at	3.92e-02	3.03e-02	2.173.594
214244_s_at	3.92e-02	3.03e-02	2.173.408
220310_at	3.92e-02	3.03e-02	-2.173.224
214580_x_at	3.93e-02	3.03e-02	-2.172.931
208959_s_at	3.93e-02	3.03e-02	2.172.513
216930_at	3.94e-02	3.04e-02	2.171.654
218819_at	3.94e-02	3.04e-02	-217.124
202301_s_at	3.95e-02	3.05e-02	2.170.464
205966_at	3.95e-02	3.05e-02	-2.170.052
204522_at	3.97e-02	3.07e-02	2.168.155
220627_at	3.98e-02	3.07e-02	-2.167.189
211449_at	3.98e-02	3.08e-02	-2.166.921
211736_at	3.98e-02	3.08e-02	-216.683
204063_s_at	3.98e-02	3.08e-02	2.166.785
204499_at	3.99e-02	3.08e-02	-216.625
205742_at	4.00e-02	3.09e-02	-2.165.365
211904_x_at	4.00e-02	3.09e-02	-2.165.128

211653_x_at	4.00e-02	3.09e-02	216.512
204994_at	4.00e-02	3.09e-02	2.165.048
211155_s_at	4.00e-02	3.09e-02	2.164.744
203954_x_at	4.01e-02	3.10e-02	-2.164.427
220391_at	4.01e-02	3.10e-02	2.164.052
220528_at	4.01e-02	3.10e-02	-216.396
210858_x_at	4.01e-02	3.10e-02	-2.163.874
216592_at	4.01e-02	3.10e-02	-2.163.813
207464_at	4.01e-02	3.10e-02	-2.163.595
212490_at	4.02e-02	3.11e-02	2.163.108
207339_s_at	4.02e-02	3.11e-02	-2.162.968
216103_at	4.02e-02	3.11e-02	-2.162.947
203823_at	4.02e-02	3.11e-02	-2.162.851
204222_s_at	4.02e-02	3.11e-02	2.162.433
200602_at	4.02e-02	3.11e-02	-2.162.399
209234_at	4.03e-02	3.11e-02	2.162.131
213945_s_at	4.03e-02	3.12e-02	2.161.622
201384_s_at	4.03e-02	3.12e-02	-2.161.354
218519_at	4.04e-02	3.12e-02	2.161.151
210508_s_at	4.04e-02	3.12e-02	-2.160.988
221630_s_at	4.04e-02	3.13e-02	-2.160.653
215761_at	4.04e-02	3.13e-02	-2.160.217
204085_s_at	4.05e-02	3.13e-02	2.160.087
203363_s_at	4.05e-02	3.14e-02	2.159.239
213185_at	4.05e-02	3.14e-02	-2.159.232
216312_at	4.06e-02	3.14e-02	-2.158.937
205015_s_at	4.06e-02	3.14e-02	-2.158.156
206067_s_at	4.07e-02	3.15e-02	-2.157.537
204644_at	4.07e-02	3.15e-02	2.157.309
214253_s_at	4.08e-02	3.16e-02	-215.658
57588_at	4.08e-02	3.16e-02	-2.156.213
218899_s_at	4.09e-02	3.17e-02	-2.155.292
210705_s_at	4.09e-02	3.17e-02	-2.155.126
208336_s_at	4.09e-02	3.17e-02	2.155.119
202394_s_at	4.10e-02	3.17e-02	-2.154.419
218326_s_at	4.10e-02	3.17e-02	-2.154.328
210319_x_at	4.12e-02	3.19e-02	-2.152.388
210505_at	4.12e-02	3.19e-02	-2.152.015
212558_at	4.12e-02	3.19e-02	-2.152.004
221222_s_at	4.13e-02	3.20e-02	2.151.753
205247_at	4.13e-02	3.20e-02	-215.099
205555_s_at	4.14e-02	3.20e-02	-2.150.865
200931_s_at	4.15e-02	3.21e-02	2.149.706
205366_s_at	4.15e-02	3.22e-02	-2.149.176

208661_s_at	4.15e-02	3.22e-02	2.149.163
49051_g_at	4.16e-02	3.22e-02	-2.148.824
218878_s_at	4.16e-02	3.22e-02	-2.148.442
221817_at	4.16e-02	3.22e-02	214.807
206185_at	4.17e-02	3.23e-02	-2.146.927
204047_s_at	4.17e-02	3.23e-02	-2.146.813
201189_s_at	4.19e-02	3.25e-02	2.145.392
207100_s_at	4.20e-02	3.26e-02	2.144.199
219145_at	4.20e-02	3.26e-02	2.144.007
205904_at	4.21e-02	3.26e-02	-2.143.553
207067_s_at	4.21e-02	3.26e-02	-2.143.052
212582_at	4.22e-02	3.27e-02	2.142.522
205540_s_at	4.22e-02	3.27e-02	2.142.399
207094_at	4.22e-02	3.27e-02	-2.142.223
206622_at	4.22e-02	3.27e-02	-214.187
212026_s_at	4.23e-02	3.28e-02	2.141.474
216478_at	4.23e-02	3.28e-02	-214.121
200952_s_at	4.24e-02	3.29e-02	-2.140.362
219661_at	4.25e-02	3.29e-02	213.974
208406_s_at	4.25e-02	3.29e-02	-2.139.608
213655_at	4.25e-02	3.29e-02	-2.139.451
208431_s_at	4.25e-02	3.30e-02	-2.139.223
216924_s_at	4.25e-02	3.30e-02	-2.139.163
220679_s_at	4.25e-02	3.30e-02	-2.138.994
46947_at	4.26e-02	3.30e-02	2.138.416
205962_at	4.27e-02	3.31e-02	-2.136.913
204809_at	4.27e-02	3.32e-02	-2.136.861
211473_s_at	4.27e-02	3.32e-02	-2.136.838
215485_s_at	4.30e-02	3.33e-02	-213.476
202714_s_at	4.31e-02	3.34e-02	-213.375
201965_s_at	4.32e-02	3.35e-02	2.132.628
207672_at	4.32e-02	3.35e-02	-2.132.394
212732_at	4.32e-02	3.35e-02	2.132.049
220649_at	4.33e-02	3.36e-02	2.131.663
215550_at	4.33e-02	3.36e-02	-2.130.964
212105_s_at	4.34e-02	3.37e-02	2.130.365
212758_s_at	4.34e-02	3.37e-02	213.015
203150_at	4.35e-02	3.37e-02	-2.129.825
201993_x_at	4.35e-02	3.38e-02	-212.958
204074_s_at	4.35e-02	3.38e-02	-2.129.336
212707_s_at	4.35e-02	3.38e-02	-2.129.027
215343_at	4.37e-02	3.39e-02	-2.127.773
209512_at	4.37e-02	3.39e-02	-2.127.702
204061_at	4.37e-02	3.40e-02	2.127.176

211592_s_at	4.38e-02	3.40e-02	-2.126.739
220791_x_at	4.38e-02	3.40e-02	-2.126.679
220464_at	4.38e-02	3.41e-02	-2.125.964
203959_s_at	4.39e-02	3.41e-02	2.125.323
203681_at	4.39e-02	3.41e-02	-2.125.064
214841_at	4.40e-02	3.42e-02	-2.124.829
214065_s_at	4.40e-02	3.42e-02	-2.124.794
214141_x_at	4.40e-02	3.42e-02	2.124.106
215669_at	4.41e-02	3.42e-02	-2.123.947
209342_s_at	4.41e-02	3.43e-02	2.123.554
215348_at	4.42e-02	3.43e-02	-2.122.544
215101_s_at	4.43e-02	3.44e-02	2.121.504
215150_at	4.43e-02	3.45e-02	2.121.184
211779_x_at	4.44e-02	3.45e-02	2.121.063
206478_at	4.44e-02	3.45e-02	-2.120.684
218580_x_at	4.44e-02	3.45e-02	-212.066
209450_at	4.44e-02	3.45e-02	2.120.633
212208_at	4.44e-02	3.45e-02	2.120.104
202826_at	4.45e-02	3.46e-02	2.119.622
214576_at	4.45e-02	3.46e-02	-2.119.118
220799_at	4.46e-02	3.46e-02	-2.118.946
205127_at	4.47e-02	3.48e-02	-2.117.642
201446_s_at	4.47e-02	3.48e-02	2.117.404
207561_s_at	4.48e-02	3.48e-02	2.116.941
208142_at	4.48e-02	3.48e-02	-2.116.902
217169_at	4.48e-02	3.48e-02	-2.116.638
217103_at	4.48e-02	3.48e-02	-2.116.631
209524_at	4.48e-02	3.49e-02	211.652
217352_at	4.49e-02	3.49e-02	2.115.887
222141_at	4.49e-02	3.49e-02	-2.115.704
210141_s_at	4.49e-02	3.49e-02	2.115.558
220460_at	4.50e-02	3.50e-02	-2.115.083
209805_at	4.50e-02	3.50e-02	2.114.556
217381_s_at	4.50e-02	3.50e-02	-2.114.406
221341_s_at	4.50e-02	3.50e-02	-211.427
204974_at	4.51e-02	3.51e-02	-2.113.587
202597_at	4.52e-02	3.52e-02	2.112.953
203535_at	4.52e-02	3.52e-02	2.112.714
207535_s_at	4.52e-02	3.52e-02	-2.112.516
209997_x_at	4.52e-02	3.52e-02	-2.112.374
215102_at	4.53e-02	3.52e-02	2.111.934
211568_at	4.53e-02	3.53e-02	-2.111.752
208266_at	4.54e-02	3.53e-02	-2.111.293
216603_at	4.54e-02	3.53e-02	-2.111.115

203862_s_at	4.54e-02	3.53e-02	2.110.883
203810_at	4.54e-02	3.54e-02	-2.110.707
209758_s_at	4.55e-02	3.54e-02	-2.110.059
208733_at	4.55e-02	3.54e-02	2.110.051
209242_at	4.55e-02	3.54e-02	2.109.643
205868_s_at	4.55e-02	3.55e-02	-2.109.451
200670_at	4.56e-02	3.55e-02	2.109.254
209705_at	4.56e-02	3.55e-02	2.109.073
206790_s_at	4.56e-02	3.55e-02	-2.108.653
213806_at	4.56e-02	3.55e-02	-2.108.635
221298_s_at	4.57e-02	3.56e-02	-2.108.156
220415_at	4.58e-02	3.57e-02	-2.107.096
222243_s_at	4.58e-02	3.57e-02	2.106.935
221363_x_at	4.58e-02	3.57e-02	-2.106.821
211620_x_at	4.59e-02	3.58e-02	-2.105.662
215589_at	4.60e-02	3.58e-02	-210.535
213266_at	4.60e-02	3.58e-02	210.503
207261_at	4.60e-02	3.59e-02	-2.104.852
210116_at	4.61e-02	3.60e-02	-2.103.722
216843_x_at	4.61e-02	3.60e-02	2.103.703
AFFX-TrpnX-5_at	4.62e-02	3.60e-02	-2.102.896
208702_x_at	4.63e-02	3.61e-02	2.102.156
206778_at	4.63e-02	3.61e-02	-2.101.969
212749_s_at	4.64e-02	3.62e-02	2.101.435
218987_at	4.64e-02	3.62e-02	2.101.391
211093_at	4.64e-02	3.62e-02	-210.135
221659_s_at	4.64e-02	3.62e-02	-2.100.876
221281_at	4.64e-02	3.62e-02	-2.100.834
210552_s_at	4.65e-02	3.62e-02	-2.100.785
220381_at	4.65e-02	3.62e-02	-2.100.719
209073_s_at	4.65e-02	3.62e-02	-2.100.473
209601_at	4.65e-02	3.63e-02	-2.100.377
207745_at	4.66e-02	3.64e-02	-2.098.966
211464_x_at	4.67e-02	3.64e-02	-2.098.776
219264_s_at	4.68e-02	3.65e-02	2.097.871
219995_s_at	4.68e-02	3.65e-02	-209.749
208994_s_at	4.69e-02	3.66e-02	2.096.874
216690_at	4.69e-02	3.66e-02	-2.096.325
214966_at	4.70e-02	3.67e-02	-2.095.744
221818_at	4.71e-02	3.68e-02	2.094.447
217343_at	4.71e-02	3.68e-02	-2.094.388
221388_at	4.72e-02	3.68e-02	-2.094.135
202681_at	4.73e-02	3.69e-02	-2.093.087
38703_at	4.76e-02	3.72e-02	209.035

221047_s_at	4.76e-02	3.72e-02	2.090.011
207375_s_at	4.77e-02	3.72e-02	2.089.768
202255_s_at	4.77e-02	3.72e-02	-2.089.581
201353_s_at	4.77e-02	3.72e-02	2.089.516
211861_x_at	4.77e-02	3.72e-02	-2.089.494
209792_s_at	4.77e-02	3.72e-02	-2.089.413
217312_s_at	4.77e-02	3.73e-02	-2.089.136
213784_at	4.78e-02	3.73e-02	2.088.548
206681_x_at	4.78e-02	3.73e-02	2.088.492
216763_at	4.79e-02	3.74e-02	-2.087.982
217792_at	4.79e-02	3.74e-02	2.087.378
214571_at	4.80e-02	3.75e-02	-2.086.515
210734_x_at	4.80e-02	3.75e-02	-2.086.327
222341_x_at	4.81e-02	3.76e-02	-2.085.771
220980_s_at	4.81e-02	3.76e-02	2.085.389
202778_s_at	4.81e-02	3.76e-02	2.085.374
221206_at	4.82e-02	3.76e-02	-2.084.923
208784_s_at	4.82e-02	3.77e-02	2.084.454
202007_at	4.83e-02	3.77e-02	2.084.005
201215_at	4.83e-02	3.77e-02	2.084.003
208442_s_at	4.83e-02	3.77e-02	2.083.872
211815_s_at	4.84e-02	3.78e-02	-2.083.313
221967_at	4.85e-02	3.79e-02	-2.081.779
32099_at	4.86e-02	3.80e-02	208.118
221067_s_at	4.87e-02	3.81e-02	-2.080.406
216915_s_at	4.88e-02	3.81e-02	2.079.658
202940_at	4.88e-02	3.82e-02	-2.079.302
215117_at	4.88e-02	3.82e-02	-2.079.279
202136_at	4.88e-02	3.82e-02	2.079.267
215241_at	4.88e-02	3.82e-02	-2.079.201
217097_s_at	4.89e-02	3.82e-02	2.078.813
216216_at	4.89e-02	3.82e-02	-2.078.791
205378_s_at	4.89e-02	3.82e-02	-2.078.445
208129_x_at	4.89e-02	3.83e-02	-2.078.189
217210_at	4.90e-02	3.83e-02	-2.077.839
208900_s_at	4.90e-02	3.83e-02	2.077.829
211029_x_at	4.90e-02	3.83e-02	-2.077.721
221751_at	4.90e-02	3.83e-02	207.733
207448_at	4.92e-02	3.85e-02	-2.076.172
214390_s_at	4.92e-02	3.85e-02	-2.076.136
204902_s_at	4.92e-02	3.85e-02	-2.075.613
212652_s_at	4.93e-02	3.86e-02	2.074.918
217663_at	4.93e-02	3.86e-02	-2.074.589
211421_s_at	4.94e-02	3.86e-02	-2.074.305

220581_at	4.94e-02	3.86e-02	2.074.164
205886_at	4.94e-02	3.86e-02	2.074.117
202652_at	4.94e-02	3.87e-02	-2.073.901
220850_at	4.95e-02	3.87e-02	-2.073.358
215189_at	4.95e-02	3.87e-02	-2.073.337
220434_at	4.95e-02	3.87e-02	-2.073.184
213658_at	4.95e-02	3.88e-02	-2.072.881
214745_at	4.96e-02	3.88e-02	-2.072.266
208467_at	4.96e-02	3.88e-02	-2.072.091
211512_s_at	4.96e-02	3.88e-02	-2.071.978
37793_r_at	4.97e-02	3.89e-02	2.071.272
222153_at	4.97e-02	3.89e-02	-2.071.091
210672_s_at	4.99e-02	3.91e-02	-2.069.225
217540_at	5.00e-02	3.91e-02	2.069.008
220764_at	5.02e-02	3.93e-02	206.702
203567_s_at	5.03e-02	3.94e-02	-2.066.468
57703_at	5.03e-02	3.94e-02	2.066.353
208342_x_at	5.04e-02	3.94e-02	-2.065.552
204259_at	5.04e-02	3.95e-02	-2.065.477
209693_at	5.05e-02	3.96e-02	2.064.191
202383_at	5.05e-02	3.96e-02	-2.064.079
212767_at	5.07e-02	3.98e-02	2.062.374
220847_x_at	5.08e-02	3.98e-02	-2.061.804
212667_at	5.08e-02	3.98e-02	-2.061.617
218922_s_at	5.09e-02	3.99e-02	-2.060.691
212237_at	5.09e-02	3.99e-02	2.060.638
221789_x_at	5.09e-02	3.99e-02	-206.063
219223_at	5.10e-02	4.00e-02	-2.060.209
210440_s_at	5.10e-02	4.00e-02	-2.060.166
205527_s_at	5.10e-02	4.00e-02	-2.059.779
215969_at	5.11e-02	4.00e-02	-2.059.437
204864_s_at	5.11e-02	4.01e-02	-2.059.064
205517_at	5.11e-02	4.01e-02	2.058.994
212873_at	5.12e-02	4.01e-02	-2.058.283
222335_at	5.12e-02	4.02e-02	-2.057.963
220597_s_at	5.13e-02	4.02e-02	-2.057.796
208387_s_at	5.13e-02	4.02e-02	-2.057.738
206793_at	5.13e-02	4.02e-02	-2.057.331
217889_s_at	5.15e-02	4.04e-02	2.055.676
217397_at	5.15e-02	4.04e-02	-205.552
217156_at	5.16e-02	4.04e-02	-2.055.267
217325_at	5.16e-02	4.05e-02	-205.468
220048_at	5.16e-02	4.05e-02	-2.054.568
203616_at	5.17e-02	4.06e-02	2.053.971

219674_s_at	5.18e-02	4.06e-02	-2.053.541
212473_s_at	5.19e-02	4.07e-02	-2.052.253
219247_s_at	5.19e-02	4.07e-02	2.052.106
215236_s_at	5.22e-02	4.09e-02	205.019
39650_s_at	5.22e-02	4.10e-02	2.049.599
206553_at	5.23e-02	4.10e-02	-2.049.473
49111_at	5.23e-02	4.10e-02	2.049.405
203866_at	5.25e-02	4.12e-02	204.707
216553_x_at	5.26e-02	4.13e-02	-204.667
201193_at	5.27e-02	4.13e-02	-2.046.032
221554_at	5.27e-02	4.14e-02	2.045.476
214444_s_at	5.28e-02	4.14e-02	-204.527
215443_at	5.28e-02	4.14e-02	-2.045.016
213788_s_at	5.28e-02	4.15e-02	2.044.678
219578_s_at	5.29e-02	4.15e-02	-20.441
217350_at	5.29e-02	4.15e-02	-2.044.017
218641_at	5.30e-02	4.16e-02	-2.043.644
208857_s_at	5.30e-02	4.16e-02	2.043.548
216424_at	5.30e-02	4.16e-02	-2.043.485
204601_at	5.30e-02	4.16e-02	2.043.327
210914_at	5.30e-02	4.17e-02	-2.042.782
218307_at	5.31e-02	4.17e-02	-2.042.574
203118_at	5.31e-02	4.17e-02	-2.041.945
220872_at	5.32e-02	4.18e-02	-2.041.596
216388_s_at	5.33e-02	4.19e-02	-2.040.441
214818_at	5.34e-02	4.19e-02	-2.040.188
218815_s_at	5.34e-02	4.19e-02	2.040.159
219199_at	5.34e-02	4.20e-02	-2.039.786
203753_at	5.36e-02	4.21e-02	-2.038.399
208140_s_at	5.36e-02	4.21e-02	-2.038.339
211348_s_at	5.37e-02	4.22e-02	-2.037.371
215263_at	5.38e-02	4.23e-02	-203.689
211660_at	5.38e-02	4.23e-02	-2.036.758
203026_at	5.38e-02	4.23e-02	2.036.397
212805_at	5.38e-02	4.23e-02	-2.036.332
210109_at	5.38e-02	4.23e-02	2.036.266
221975_s_at	5.38e-02	4.23e-02	-2.036.241
217698_at	5.40e-02	4.25e-02	-2.034.556
209483_s_at	5.42e-02	4.26e-02	-2.033.718
216991_at	5.42e-02	4.26e-02	-2.033.377
208178_x_at	5.42e-02	4.26e-02	2.033.009
219001_s_at	5.42e-02	4.26e-02	2.032.962
37549_g_at	5.43e-02	4.27e-02	2.032.532
208577_at	5.43e-02	4.27e-02	2.032.358

203614_at	5.43e-02	4.27e-02	2.032.164
205642_at	5.44e-02	4.28e-02	2.031.317
214551_s_at	5.44e-02	4.28e-02	2.031.224
219792_at	5.45e-02	4.29e-02	2.030.949
203783_x_at	5.45e-02	4.29e-02	2.030.797
206151_x_at	5.46e-02	4.29e-02	-2.030.315
221457_s_at	5.47e-02	4.30e-02	-2.029.561
221032_s_at	5.47e-02	4.30e-02	-2.029.133
214637_at	5.47e-02	4.31e-02	-2.028.891
215319_at	5.48e-02	4.31e-02	-2.028.693
216792_at	5.48e-02	4.31e-02	-2.028.229
217146_at	5.48e-02	4.32e-02	-2.027.981
209030_s_at	5.50e-02	4.33e-02	-2.027.018
217606_at	5.50e-02	4.33e-02	2.026.595
203382_s_at	5.51e-02	4.33e-02	-2.026.186
217687_at	5.52e-02	4.35e-02	-2.025.039
200714_x_at	5.54e-02	4.36e-02	-2.023.347
206022_at	5.54e-02	4.36e-02	-2.023.201
212322_at	5.55e-02	4.37e-02	2.023.115
202005_at	5.55e-02	4.37e-02	-2.023.001
206625_at	5.55e-02	4.37e-02	2.022.679
213908_at	5.55e-02	4.37e-02	-2.022.649
43511_s_at	5.55e-02	4.37e-02	2.022.418
217278_x_at	5.55e-02	4.37e-02	-2.022.326
202090_s_at	5.56e-02	4.38e-02	-2.021.952
219783_at	5.59e-02	4.40e-02	-2.019.891
211925_s_at	5.59e-02	4.40e-02	2.019.688
211267_at	5.59e-02	4.40e-02	-2.019.662
214326_x_at	5.60e-02	4.41e-02	-2.018.531
37278_at	5.61e-02	4.42e-02	-2.018.086
216657_at	5.61e-02	4.42e-02	-2.017.695
208186_s_at	5.61e-02	4.42e-02	-2.017.685
206181_at	5.62e-02	4.42e-02	-2.017.461
54970_at	5.62e-02	4.43e-02	-2.017.056
217863_at	5.63e-02	4.44e-02	2.016.355
212879_x_at	5.64e-02	4.44e-02	2.015.536
202236_s_at	5.64e-02	4.45e-02	-2.015.507
221373_x_at	5.64e-02	4.45e-02	-2.015.465
214399_s_at	5.66e-02	4.46e-02	-2.014.197
201920_at	5.67e-02	4.47e-02	2.013.155
211087_x_at	5.67e-02	4.47e-02	2.013.103
217833_at	5.68e-02	4.47e-02	2.012.783
216890_at	5.68e-02	4.48e-02	-2.012.603
203257_s_at	5.68e-02	4.48e-02	2.012.521

209877_at	5.68e-02	4.48e-02	-2.012.065
217440_at	5.69e-02	4.49e-02	-2.011.488
217309_s_at	5.69e-02	4.49e-02	-201.136
204856_at	5.69e-02	4.49e-02	2.011.354
217709_at	5.69e-02	4.49e-02	-2.011.279
203767_s_at	5.71e-02	4.51e-02	-2.009.747
207575_at	5.72e-02	4.51e-02	2.009.433
205746_s_at	5.74e-02	4.53e-02	-2.007.897
218390_s_at	5.75e-02	4.54e-02	2.006.893
208302_at	5.76e-02	4.54e-02	-2.006.371
205379_at	5.76e-02	4.55e-02	2.005.881
208441_at	5.77e-02	4.55e-02	-2.005.205
213482_at	5.77e-02	4.56e-02	-2.005.102
219550_at	5.78e-02	4.56e-02	-2.004.675
220472_at	5.79e-02	4.57e-02	2.003.952
207878_at	5.80e-02	4.57e-02	-2.003.494
210675_s_at	5.80e-02	4.57e-02	-2.003.455
216946_at	5.80e-02	4.58e-02	-2.002.965
217644_s_at	5.81e-02	4.58e-02	-2.002.474
207534_at	5.81e-02	4.59e-02	2.002.346
210045_at	5.81e-02	4.59e-02	-2.002.067
216337_at	5.82e-02	4.59e-02	-2.001.818
220666_at	5.83e-02	4.60e-02	2.000.999
222267_at	5.83e-02	4.60e-02	-2.000.877
205947_s_at	5.85e-02	4.62e-02	-19.994
217577_at	5.85e-02	4.62e-02	-1.999.109
207371_at	5.85e-02	4.62e-02	-1.998.966
65133_i_at	5.86e-02	4.62e-02	-1.998.812
220362_at	5.86e-02	4.62e-02	-1.998.776
204281_at	5.86e-02	4.63e-02	-1.998.657
210535_at	5.86e-02	4.63e-02	-1.998.496
207484_s_at	5.86e-02	4.63e-02	1.998.045
205300_s_at	5.88e-02	4.65e-02	1.996.736
219422_at	5.89e-02	4.66e-02	-1.995.914
203374_s_at	5.90e-02	4.66e-02	-1.995.617
211937_at	5.90e-02	4.66e-02	1.995.048
207639_at	5.91e-02	4.67e-02	-1.994.431
206920_s_at	5.91e-02	4.67e-02	1.994.261
213885_at	5.93e-02	4.68e-02	1.993.492
219864_s_at	5.93e-02	4.69e-02	1.993.169
211800_s_at	5.95e-02	4.70e-02	1.991.689
221051_s_at	5.95e-02	4.70e-02	-1.991.437
212954_at	5.96e-02	4.71e-02	1.991.099
203231_s_at	5.96e-02	4.71e-02	-1.991.008

215698_at	5.96e-02	4.71e-02	199.096
212044_s_at	5.97e-02	4.72e-02	198.994
214770_at	5.98e-02	4.73e-02	-1.989.462
212027_at	5.98e-02	4.73e-02	-1.989.134
201323_at	5.99e-02	4.73e-02	1.988.974
215789_s_at	5.99e-02	4.73e-02	-1.988.846
206039_at	5.99e-02	4.74e-02	-1.988.347
205678_at	5.99e-02	4.74e-02	-1.988.237
217272_s_at	6.02e-02	4.76e-02	-1.986.619
215776_at	6.02e-02	4.76e-02	-1.986.596
221393_at	6.02e-02	4.76e-02	-1.986.411
209136_s_at	6.02e-02	4.76e-02	-1.986.394
201675_at	6.02e-02	4.76e-02	1.986.133
209684_at	6.02e-02	4.76e-02	-1.985.973
215018_at	6.03e-02	4.77e-02	-1.985.717
207002_s_at	6.03e-02	4.77e-02	1.985.349
206295_at	6.03e-02	4.77e-02	-1.985.201
220230_s_at	6.04e-02	4.77e-02	-1.985.083
207160_at	6.04e-02	4.78e-02	-198.476
214360_at	6.05e-02	4.79e-02	-1.984.034
207012_at	6.05e-02	4.79e-02	-1.984.013
217813_s_at	6.06e-02	4.79e-02	1.983.289
211564_s_at	6.06e-02	4.79e-02	1.983.272
208339_at	6.06e-02	4.80e-02	-1.983.091
203451_at	6.06e-02	4.80e-02	1.982.892
222257_s_at	6.06e-02	4.80e-02	-1.982.823
216440_at	6.07e-02	4.80e-02	-1.982.709
203069_at	6.07e-02	4.80e-02	-1.982.624
210486_at	6.07e-02	4.81e-02	-1.982.362
208321_s_at	6.08e-02	4.81e-02	-1.981.676
221056_x_at	6.09e-02	4.82e-02	1.981.136
222298_at	6.09e-02	4.82e-02	-1.980.946
215442_s_at	6.09e-02	4.82e-02	-1.980.786
206458_s_at	6.09e-02	4.82e-02	-1.980.686
207046_at	6.10e-02	4.83e-02	1.980.324
217171_at	6.11e-02	4.84e-02	-1.979.564
205814_at	6.12e-02	4.84e-02	1.978.976
214984_at	6.12e-02	4.85e-02	-1.978.736
208295_x_at	6.12e-02	4.85e-02	-1.978.394
204100_at	6.12e-02	4.85e-02	-1.978.259
218744_s_at	6.14e-02	4.86e-02	-1.977.402
215024_at	6.14e-02	4.86e-02	-1.977.334
203958_s_at	6.14e-02	4.86e-02	197.729
216078_at	6.14e-02	4.87e-02	-1.976.937

219636_s_at	6.17e-02	4.89e-02	-1.974.825
218826_at	6.18e-02	4.90e-02	-1.974.394
219949_at	6.18e-02	4.90e-02	1.974.068
217375_at	6.19e-02	4.90e-02	-1.973.634
205949_at	6.19e-02	4.91e-02	-197.323
217599_s_at	6.20e-02	4.92e-02	-1.972.632
219500_at	6.20e-02	4.92e-02	-1.972.501
215473_at	6.21e-02	4.92e-02	-1.972.022
204981_at	6.21e-02	4.92e-02	-1.971.908
216057_at	6.21e-02	4.92e-02	-19.719
210025_s_at	6.22e-02	4.93e-02	-1.971.243
221985_at	6.22e-02	4.93e-02	-1.970.934
217051_s_at	6.24e-02	4.95e-02	-1.969.911
209037_s_at	6.24e-02	4.95e-02	-1.969.851
214299_at	6.24e-02	4.95e-02	1.969.767
207486_x_at	6.24e-02	4.95e-02	-1.969.483
205166_at	6.25e-02	4.95e-02	-19.692
221120_at	6.25e-02	4.96e-02	-1.968.812
214995_s_at	6.25e-02	4.96e-02	-1.968.683
216427_at	6.26e-02	4.96e-02	-1.968.541
220876_at	6.26e-02	4.96e-02	-1.968.494
210192_at	6.26e-02	4.97e-02	-1.968.296
204296_at	6.26e-02	4.97e-02	-1.968.248
211459_at	6.27e-02	4.98e-02	-1.967.316
202295_s_at	6.28e-02	4.98e-02	1.967.124
210801_at	6.28e-02	4.98e-02	-1.966.664
207965_at	6.29e-02	4.99e-02	-1.966.253
213395_at	6.30e-02	5.00e-02	-1.965.563
213952_s_at	6.30e-02	5.00e-02	-1.965.555
209108_at	6.31e-02	5.01e-02	-1.964.492
220319_s_at	6.32e-02	5.02e-02	1.963.928
210635_s_at	6.33e-02	5.02e-02	-196.334
219721_at	6.33e-02	5.02e-02	-1.963.263
201470_at	6.34e-02	5.03e-02	-1.962.452
206564_at	6.34e-02	5.04e-02	-1.962.228
220932_at	6.34e-02	5.04e-02	-1.962.183
211887_x_at	6.35e-02	5.04e-02	-196.204
202083_s_at	6.35e-02	5.04e-02	1.961.845
207943_x_at	6.35e-02	5.04e-02	1.961.657
211197_s_at	6.37e-02	5.06e-02	-1.960.547
207355_at	6.38e-02	5.06e-02	1.959.776
211500_at	6.38e-02	5.06e-02	-1.959.775
202624_s_at	6.41e-02	5.09e-02	1.957.583
203108_at	6.41e-02	5.09e-02	-1.957.381

215066_at	6.41e-02	5.09e-02	-1.957.283
222061_at	6.42e-02	5.10e-02	-1.956.727
215796_at	6.42e-02	5.10e-02	-1.956.642
218202_x_at	6.42e-02	5.10e-02	1.956.516
59625_at	6.42e-02	5.10e-02	1.956.477
220873_at	6.43e-02	5.11e-02	-1.956.195
209554_at	6.43e-02	5.11e-02	-1.956.137
208121_s_at	6.43e-02	5.11e-02	1.955.935
216969_s_at	6.44e-02	5.12e-02	1.955.237
214473_x_at	6.45e-02	5.13e-02	1.954.377
208325_s_at	6.45e-02	5.13e-02	-1.954.359
206908_s_at	6.45e-02	5.13e-02	-1.954.308
218147_s_at	6.45e-02	5.13e-02	-1.954.307
213344_s_at	6.46e-02	5.13e-02	1.953.957
218525_s_at	6.49e-02	5.16e-02	1.952.061
217486_s_at	6.49e-02	5.16e-02	-1.951.484
214366_s_at	6.50e-02	5.16e-02	-1.951.311
205117_at	6.50e-02	5.17e-02	-195.103
203277_at	6.50e-02	5.17e-02	1.950.806
211704_s_at	6.51e-02	5.17e-02	1.950.609
64474_g_at	6.51e-02	5.18e-02	-1.950.216
203984_s_at	6.52e-02	5.18e-02	-1.949.823
220023_at	6.52e-02	5.18e-02	-1.949.694
217612_at	6.53e-02	5.19e-02	-1.948.822
221111_at	6.54e-02	5.20e-02	-1.947.995
220494_s_at	6.55e-02	5.21e-02	-1.947.777
202247_s_at	6.55e-02	5.21e-02	-1.947.671
213105_s_at	6.55e-02	5.21e-02	1.947.172
204609_at	6.56e-02	5.22e-02	-1.947.004
210317_s_at	6.56e-02	5.22e-02	1.946.722
215570_s_at	6.56e-02	5.22e-02	1.946.584
207629_s_at	6.57e-02	5.23e-02	1.946.261
210712_at	6.57e-02	5.23e-02	-1.946.199
210249_s_at	6.59e-02	5.25e-02	1.944.541
216683_at	6.60e-02	5.25e-02	-1.944.164
212925_at	6.60e-02	5.26e-02	-1.943.658
204941_s_at	6.61e-02	5.26e-02	-1.943.435
209808_x_at	6.61e-02	5.26e-02	-1.943.336
218566_s_at	6.61e-02	5.26e-02	1.943.246
216809_at	6.61e-02	5.26e-02	-1.942.984
200634_at	6.61e-02	5.27e-02	1.942.956
208070_s_at	6.62e-02	5.27e-02	1.942.246
220986_s_at	6.64e-02	5.28e-02	-1.941.422
219501_at	6.65e-02	5.30e-02	-1.940.462

220423_at	6.66e-02	5.30e-02	1.940.047
208076_at	6.66e-02	5.30e-02	-1.939.941
208252_s_at	6.66e-02	5.30e-02	-1.939.895
204916_at	6.66e-02	5.30e-02	193.979
206184_at	6.66e-02	5.30e-02	193.978
212111_at	6.66e-02	5.31e-02	1.939.478
212868_x_at	6.67e-02	5.31e-02	-1.938.855
208433_s_at	6.69e-02	5.33e-02	1.937.873
203632_s_at	6.71e-02	5.35e-02	1.936.129
206931_at	6.72e-02	5.35e-02	-1.935.822
205556_at	6.73e-02	5.36e-02	-1.934.971
202446_s_at	6.73e-02	5.36e-02	-193.485
211079_s_at	6.77e-02	5.39e-02	-1.932.443
218650_at	6.78e-02	5.40e-02	-1.931.716
213566_at	6.81e-02	5.42e-02	-1.930.049
211170_s_at	6.81e-02	5.43e-02	-1.929.766
211521_s_at	6.83e-02	5.44e-02	-1.928.717
205455_at	6.84e-02	5.45e-02	-1.927.818
203186_s_at	6.84e-02	5.45e-02	1.927.777
202986_at	6.85e-02	5.46e-02	1.927.394
217277_at	6.85e-02	5.46e-02	-1.926.786
202464_s_at	6.87e-02	5.48e-02	-1.925.559
207292_s_at	6.88e-02	5.48e-02	1.925.309
214012_at	6.88e-02	5.49e-02	-1.925.045
215362_at	6.89e-02	5.49e-02	-1.924.716
204956_at	6.90e-02	5.50e-02	1.923.907
216980_s_at	6.92e-02	5.52e-02	-1.922.603
210669_at	6.92e-02	5.52e-02	-1.922.223
204502_at	6.92e-02	5.52e-02	1.922.213
206880_at	6.94e-02	5.53e-02	-19.214
216370_s_at	6.94e-02	5.54e-02	1.920.994
214607_at	6.94e-02	5.54e-02	-1.920.866
218230_at	6.95e-02	5.54e-02	1.920.364
208157_at	6.96e-02	5.55e-02	-1.920.061
213899_at	6.96e-02	5.56e-02	1.919.487
221993_s_at	6.97e-02	5.56e-02	-1.919.107
210373_at	6.98e-02	5.57e-02	-1.918.694
218293_x_at	6.99e-02	5.58e-02	1.917.805
220832_at	6.99e-02	5.58e-02	-19.177
204014_at	7.00e-02	5.58e-02	191.739
200671_s_at	7.00e-02	5.58e-02	1.917.362
215522_at	7.02e-02	5.61e-02	1.915.608
208347_at	7.03e-02	5.61e-02	-1.915.141
204904_at	7.03e-02	5.62e-02	-1.914.828

208924_at	7.06e-02	5.64e-02	-1.912.967
213196_at	7.08e-02	5.65e-02	1.911.874
210743_s_at	7.08e-02	5.65e-02	-1.911.751
212789_at	7.09e-02	5.66e-02	1.911.549
221059_s_at	7.10e-02	5.67e-02	-1.910.398
207683_at	7.10e-02	5.67e-02	-1.910.396
207055_at	7.11e-02	5.68e-02	-1.910.014
208663_s_at	7.13e-02	5.69e-02	1.908.976
215879_at	7.13e-02	5.69e-02	-1.908.643
204939_s_at	7.14e-02	5.70e-02	-19.083
205688_at	7.14e-02	5.70e-02	-1.908.096
219513_s_at	7.14e-02	5.70e-02	-190.804
203751_x_at	7.14e-02	5.70e-02	-1.907.984
214834_at	7.14e-02	5.71e-02	1.907.699
52159_at	7.16e-02	5.72e-02	-1.906.882
205729_at	7.16e-02	5.72e-02	-1.906.831
206922_at	7.16e-02	5.72e-02	1.906.792
212830_at	7.16e-02	5.72e-02	-1.906.617
203441_s_at	7.17e-02	5.73e-02	1.905.814
217195_at	7.17e-02	5.73e-02	-1.905.774
204690_at	7.19e-02	5.74e-02	-1.904.903
205396_at	7.19e-02	5.75e-02	1.904.765
215822_x_at	7.19e-02	5.75e-02	-1.904.597
207610_s_at	7.20e-02	5.76e-02	-1.904.009
220413_at	7.20e-02	5.76e-02	-1.903.987
209013_x_at	7.20e-02	5.76e-02	1.903.781
214567_s_at	7.21e-02	5.76e-02	1.903.378
209221_s_at	7.21e-02	5.77e-02	1.903.257
201100_s_at	7.22e-02	5.78e-02	-1.902.479
210307_s_at	7.22e-02	5.78e-02	-1.902.429
211448_s_at	7.23e-02	5.78e-02	-190.197
203939_at	7.23e-02	5.78e-02	-190.179
209909_s_at	7.24e-02	5.79e-02	1.901.331
210476_s_at	7.25e-02	5.80e-02	-1.900.791
218242_s_at	7.27e-02	5.81e-02	1.899.658
207659_s_at	7.27e-02	5.81e-02	-189.952
208049_s_at	7.27e-02	5.82e-02	-1.899.133
219353_at	7.28e-02	5.82e-02	1.899.045
218050_at	7.28e-02	5.82e-02	1.898.702
209202_s_at	7.28e-02	5.83e-02	1.898.625
207306_at	7.29e-02	5.83e-02	-1.898.283
208408_at	7.31e-02	5.85e-02	-1.897.003
203858_s_at	7.32e-02	5.86e-02	1.896.395
45297_at	7.32e-02	5.86e-02	1.896.318

219651_at	7.33e-02	5.87e-02	-1.895.668
217533_x_at	7.34e-02	5.87e-02	-1.895.273
213516_at	7.34e-02	5.87e-02	-1.895.124
217591_at	7.34e-02	5.88e-02	-1.894.766
217671_at	7.35e-02	5.88e-02	-1.894.317
208200_at	7.35e-02	5.88e-02	-1.894.203
210068_s_at	7.35e-02	5.89e-02	-1.894.004
212952_at	7.36e-02	5.89e-02	-1.893.834
210943_s_at	7.36e-02	5.89e-02	-1.893.665
207553_at	7.37e-02	5.90e-02	-1.893.015
214519_s_at	7.37e-02	5.90e-02	-1.892.924
206841_at	7.37e-02	5.90e-02	-1.892.703
211630_s_at	7.39e-02	5.92e-02	-1.891.439
217883_at	7.40e-02	5.92e-02	-1.891.231
219276_x_at	7.40e-02	5.93e-02	-1.890.916
205315_s_at	7.40e-02	5.93e-02	1.890.755
202994_s_at	7.41e-02	5.93e-02	-1.890.581
217490_at	7.41e-02	5.94e-02	-1.890.346
217328_at	7.42e-02	5.94e-02	-1.889.772
207372_s_at	7.43e-02	5.95e-02	-1.889.302
202496_at	7.43e-02	5.96e-02	1.888.833
221143_at	7.44e-02	5.96e-02	188.874
221037_s_at	7.44e-02	5.96e-02	-1.888.661
220729_at	7.44e-02	5.96e-02	-1.888.264
209923_s_at	7.45e-02	5.97e-02	1.888.043
216572_at	7.46e-02	5.98e-02	-1.887.419
220298_s_at	7.48e-02	6.00e-02	-1.885.727
64900_at	7.50e-02	6.01e-02	-1.884.982
216944_s_at	7.50e-02	6.01e-02	188.457
210780_at	7.51e-02	6.02e-02	-1.884.277
210913_at	7.52e-02	6.03e-02	-1.883.728
207284_s_at	7.52e-02	6.03e-02	1.883.448
204691_x_at	7.52e-02	6.03e-02	1.883.378
206723_s_at	7.52e-02	6.03e-02	1.883.247
211869_at	7.54e-02	6.05e-02	-1.882.045
213930_at	7.54e-02	6.05e-02	1.882.034
215127_s_at	7.55e-02	6.05e-02	-1.881.862
216254_at	7.55e-02	6.05e-02	-1.881.778
214559_at	7.55e-02	6.06e-02	-1.881.426
221168_at	7.55e-02	6.06e-02	-1.881.338
220836_at	7.56e-02	6.06e-02	-1.880.964
217600_at	7.59e-02	6.09e-02	-1.879.058
220483_s_at	7.59e-02	6.09e-02	-1.878.948
208012_x_at	7.59e-02	6.09e-02	1.878.887

202663_at	7.60e-02	6.09e-02	-1.878.608
213962_s_at	7.60e-02	6.10e-02	-1.878.478
219459_at	7.60e-02	6.10e-02	187.827
222097_at	7.62e-02	6.11e-02	-1.877.441
209272_at	7.62e-02	6.11e-02	1.877.171
214969_at	7.62e-02	6.12e-02	1.876.884
203011_at	7.63e-02	6.12e-02	-1.876.731
204028_s_at	7.63e-02	6.12e-02	187.659
208703_s_at	7.64e-02	6.13e-02	-1.875.929
219987_at	7.66e-02	6.15e-02	-1.874.584
211788_s_at	7.67e-02	6.16e-02	-1.874.025
217029_at	7.67e-02	6.16e-02	-1.873.973
216788_at	7.68e-02	6.16e-02	-187.356
213840_s_at	7.69e-02	6.17e-02	-1.873.114
212781_at	7.69e-02	6.18e-02	-187.267
200643_at	7.70e-02	6.18e-02	1.872.398
216616_at	7.71e-02	6.19e-02	-1.871.825
209582_s_at	7.71e-02	6.19e-02	1.871.758
209159_s_at	7.71e-02	6.19e-02	-1.871.517
221299_at	7.72e-02	6.20e-02	-1.870.727
215538_at	7.73e-02	6.21e-02	-1.870.496
212387_at	7.73e-02	6.21e-02	-1.870.339
222371_at	7.73e-02	6.21e-02	-187.018
205350_at	7.74e-02	6.21e-02	-1.869.996
206764_x_at	7.74e-02	6.21e-02	1.869.933
215112_x_at	7.74e-02	6.22e-02	1.869.458
221255_s_at	7.75e-02	6.23e-02	-1.869.121
216487_at	7.75e-02	6.23e-02	-1.868.864
206224_at	7.76e-02	6.23e-02	1.868.736
205389_s_at	7.76e-02	6.23e-02	-1.868.637
216192_at	7.79e-02	6.26e-02	-186.686
207443_at	7.79e-02	6.26e-02	-1.866.598
214041_x_at	7.79e-02	6.26e-02	-1.866.394
219829_at	7.80e-02	6.27e-02	1.866.164
204111_at	7.82e-02	6.28e-02	-1.864.984
211019_s_at	7.83e-02	6.29e-02	1.864.388
210169_at	7.83e-02	6.29e-02	-1.864.361
213412_at	7.84e-02	6.30e-02	1.863.864
210545_at	7.84e-02	6.30e-02	-1.863.672
203669_s_at	7.84e-02	6.31e-02	1.863.385
222374_at	7.85e-02	6.31e-02	-1.863.279
202130_at	7.85e-02	6.31e-02	-1.863.012
216816_at	7.86e-02	6.32e-02	-1.862.228
219033_at	7.86e-02	6.32e-02	-1.862.129

217130_at	7.87e-02	6.33e-02	-1.861.976
208593_x_at	7.87e-02	6.33e-02	-1.861.822
216278_at	7.87e-02	6.33e-02	-1.861.527
209578_s_at	7.88e-02	6.34e-02	-1.861.191
218587_s_at	7.88e-02	6.34e-02	186.092
211393_at	7.89e-02	6.35e-02	-1.860.634
204677_at	7.89e-02	6.35e-02	-1.860.517
205817_at	7.89e-02	6.35e-02	-1.860.422
217572_at	7.89e-02	6.35e-02	-1.860.305
214711_at	7.89e-02	6.35e-02	-1.860.291
220993_s_at	7.89e-02	6.35e-02	-1.860.197
207759_s_at	7.90e-02	6.35e-02	-1.860.088
221129_at	7.90e-02	6.35e-02	-1.859.977
210574_s_at	7.92e-02	6.37e-02	1.858.885
215008_at	7.92e-02	6.38e-02	-1.858.438
216480_x_at	7.93e-02	6.38e-02	-1.858.287
218730_s_at	7.93e-02	6.38e-02	-1.858.178
220254_at	7.93e-02	6.38e-02	1.858.027
217180_at	7.94e-02	6.39e-02	-1.857.318
219469_at	7.97e-02	6.42e-02	-1.855.712
204737_s_at	7.97e-02	6.42e-02	-1.855.674
213580_at	7.98e-02	6.42e-02	-1.855.153
214981_at	7.98e-02	6.43e-02	-1.855.009
202175_at	7.99e-02	6.43e-02	-1.854.452
203962_s_at	7.99e-02	6.44e-02	1.854.249
220953_s_at	8.00e-02	6.44e-02	185.369
206605_at	8.01e-02	6.45e-02	-1.853.494
216105_x_at	8.03e-02	6.47e-02	-1.852.024
221022_s_at	8.04e-02	6.48e-02	-185.145
215065_at	8.04e-02	6.48e-02	-1.851.375
208970_s_at	8.04e-02	6.48e-02	-185.132
213232_at	8.04e-02	6.48e-02	-185.131
217039_x_at	8.04e-02	6.48e-02	-1.851.215
218034_at	8.05e-02	6.49e-02	1.850.685
216449_x_at	8.05e-02	6.49e-02	1.850.516
204611_s_at	8.06e-02	6.49e-02	-1.850.225
210429_at	8.06e-02	6.50e-02	-1.850.016
218754_at	8.07e-02	6.50e-02	1.849.777
208205_at	8.08e-02	6.51e-02	-1.848.795
216619_at	8.10e-02	6.53e-02	-1.847.915
221631_at	8.10e-02	6.53e-02	-1.847.633
209331_s_at	8.11e-02	6.54e-02	1.847.295
216056_at	8.13e-02	6.55e-02	-1.846.206
201375_s_at	8.13e-02	6.56e-02	-1.845.878

204681_s_at	8.14e-02	6.56e-02	-1.845.422
219003_s_at	8.16e-02	6.58e-02	1.844.537
202837_at	8.16e-02	6.58e-02	1.844.401
215037_s_at	8.16e-02	6.58e-02	184.434
211331_x_at	8.16e-02	6.58e-02	1.844.163
213807_x_at	8.17e-02	6.59e-02	1.843.533
209083_at	8.17e-02	6.59e-02	-1.843.455
32402_s_at	8.18e-02	6.60e-02	1.843.225
206636_at	8.18e-02	6.60e-02	1.842.857
202308_at	8.19e-02	6.60e-02	1.842.791
221137_at	8.19e-02	6.61e-02	-1.842.344
218029_at	8.19e-02	6.61e-02	-1.842.336
214029_at	8.21e-02	6.62e-02	-1.841.257
220392_at	8.21e-02	6.63e-02	-1.841.185
219837_s_at	8.22e-02	6.63e-02	-1.840.781
220438_at	8.24e-02	6.65e-02	-1.839.421
220283_at	8.26e-02	6.67e-02	1.838.463
205735_s_at	8.27e-02	6.67e-02	-1.838.003
214480_at	8.28e-02	6.68e-02	-1.837.495
220263_at	8.28e-02	6.68e-02	1.837.197
212998_x_at	8.29e-02	6.69e-02	-1.836.714
222202_at	8.30e-02	6.70e-02	-1.836.359
221367_at	8.31e-02	6.71e-02	-1.835.729
215517_at	8.31e-02	6.71e-02	-1.835.634
216990_at	8.32e-02	6.72e-02	-1.835.011
205510_s_at	8.32e-02	6.72e-02	-1.834.855
200033_at	8.33e-02	6.72e-02	1.834.691
201152_s_at	8.35e-02	6.74e-02	-1.833.545
212280_x_at	8.35e-02	6.74e-02	1.833.504
220911_s_at	8.36e-02	6.75e-02	1.832.953
219586_at	8.36e-02	6.75e-02	-1.832.829
207329_at	8.36e-02	6.75e-02	-1.832.786
211285_s_at	8.36e-02	6.75e-02	1.832.744
205595_at	8.37e-02	6.76e-02	-1.832.308
217542_at	8.37e-02	6.76e-02	-1.832.118
221374_at	8.37e-02	6.76e-02	-1.832.094
207434_s_at	8.37e-02	6.76e-02	-1.832.068
221861_at	8.38e-02	6.77e-02	1.831.704
217135_x_at	8.38e-02	6.77e-02	1.831.616
36920_at	8.40e-02	6.79e-02	1.830.399
207477_at	8.40e-02	6.79e-02	-1.830.096
213916_at	8.41e-02	6.80e-02	1.829.625
219354_at	8.41e-02	6.80e-02	-182.958
212940_at	8.42e-02	6.80e-02	-1.829.217

206726_at	8.43e-02	6.81e-02	-1.828.504
219786_at	8.43e-02	6.82e-02	1.828.417
211332_x_at	8.43e-02	6.82e-02	-1.828.293
204659_s_at	8.44e-02	6.82e-02	1.828.192
214408_s_at	8.45e-02	6.83e-02	-182.725
222217_s_at	8.47e-02	6.84e-02	1.826.554
220505_at	8.47e-02	6.85e-02	-1.826.182
201610_at	8.48e-02	6.85e-02	-1.825.833
209415_at	8.48e-02	6.86e-02	-1.825.739
205402_x_at	8.48e-02	6.86e-02	1.825.611
218404_at	8.49e-02	6.86e-02	-1.825.331
206718_at	8.49e-02	6.87e-02	-1.825.121
212028_at	8.50e-02	6.87e-02	182.464
202834_at	8.51e-02	6.88e-02	1.824.173
216260_at	8.52e-02	6.89e-02	-1.823.517
213389_at	8.52e-02	6.89e-02	-1.823.183
219575_s_at	8.52e-02	6.90e-02	-1.823.126
217984_at	8.53e-02	6.90e-02	1.822.964
212802_s_at	8.53e-02	6.90e-02	1.822.908
203456_at	8.53e-02	6.90e-02	1.822.575
201779_s_at	8.54e-02	6.91e-02	1.822.197
216376_x_at	8.55e-02	6.91e-02	-1.821.875
204287_at	8.56e-02	6.93e-02	-1.820.981
211816_x_at	8.57e-02	6.93e-02	-1.820.628
201012_at	8.57e-02	6.93e-02	1.820.531
208486_at	8.57e-02	6.94e-02	-1.820.242
218780_at	8.60e-02	6.96e-02	-1.819.044
209207_s_at	8.61e-02	6.97e-02	1.818.263
218494_s_at	8.61e-02	6.97e-02	-1.818.034
201411_s_at	8.63e-02	6.99e-02	181.715
203784_s_at	8.64e-02	7.00e-02	-1.816.421
206691_s_at	8.65e-02	7.01e-02	1.815.921
214089_at	8.66e-02	7.01e-02	-1.815.733
205511_at	8.66e-02	7.01e-02	-1.815.582
211014_s_at	8.66e-02	7.01e-02	-181.541
205358_at	8.67e-02	7.02e-02	1.814.782
217203_at	8.68e-02	7.03e-02	-1.814.454
213851_at	8.68e-02	7.03e-02	181.427
203643_at	8.69e-02	7.04e-02	1.813.928
205725_at	8.69e-02	7.04e-02	-1.813.624
211164_at	8.70e-02	7.05e-02	-1.813.115
209206_at	8.70e-02	7.05e-02	-18.131
208533_at	8.71e-02	7.06e-02	-1.812.561
221753_at	8.71e-02	7.06e-02	1.812.494

220552_at	8.73e-02	7.07e-02	-1.811.793
211571_s_at	8.75e-02	7.09e-02	1.810.751
205585_at	8.77e-02	7.10e-02	-1.809.567
220695_at	8.78e-02	7.11e-02	-1.809.116
205482_x_at	8.78e-02	7.11e-02	1.809.065
220943_s_at	8.79e-02	7.13e-02	1.808.133
220348_at	8.80e-02	7.13e-02	1.807.798
215080_s_at	8.81e-02	7.14e-02	-1.807.202
207229_at	8.82e-02	7.15e-02	-1.806.838
221312_at	8.82e-02	7.15e-02	-1.806.742
204140_at	8.82e-02	7.15e-02	1.806.697
217105_at	8.84e-02	7.16e-02	-1.805.812
211679_x_at	8.86e-02	7.18e-02	1.804.546
210881_s_at	8.87e-02	7.19e-02	-1.804.171
217629_at	8.88e-02	7.20e-02	-1.803.686
201369_s_at	8.88e-02	7.20e-02	-1.803.467
220468_at	8.90e-02	7.21e-02	1.802.576
206552_s_at	8.90e-02	7.21e-02	-1.802.521
37005_at	8.90e-02	7.22e-02	1.802.429
221462_x_at	8.90e-02	7.22e-02	-1.802.337
205779_at	8.90e-02	7.22e-02	1.802.243
213113_s_at	8.91e-02	7.23e-02	-1.801.692
201863_at	8.94e-02	7.25e-02	1.800.168
221018_s_at	8.96e-02	7.27e-02	-1.799.145
211371_at	8.96e-02	7.27e-02	-1.799.057
213891_s_at	8.98e-02	7.28e-02	-1.798.238
217085_at	8.98e-02	7.28e-02	-1.798.071
221972_s_at	8.98e-02	7.29e-02	-1.797.874
217886_at	8.99e-02	7.29e-02	-1.797.418
214541_s_at	9.00e-02	7.31e-02	179.665
209075_s_at	9.01e-02	7.31e-02	-1.796.169
220856_x_at	9.02e-02	7.32e-02	-1.796.001
206013_s_at	9.02e-02	7.32e-02	-1.795.871
211109_at	9.02e-02	7.32e-02	-1.795.561
220153_at	9.03e-02	7.33e-02	1.795.447
201089_at	9.04e-02	7.33e-02	1.794.941
204469_at	9.04e-02	7.34e-02	-1.794.489
204329_s_at	9.05e-02	7.34e-02	1.794.332
214396_s_at	9.05e-02	7.35e-02	-1.793.972
214679_x_at	9.06e-02	7.36e-02	1.793.503
59644_at	9.06e-02	7.36e-02	-1.793.415
201969_at	9.07e-02	7.36e-02	1.793.168
200883_at	9.07e-02	7.37e-02	-1.792.998
217630_at	9.09e-02	7.38e-02	1.792.059

209437_s_at	9.09e-02	7.38e-02	-1.791.813
213192_at	9.11e-02	7.40e-02	1.791.001
206352_s_at	9.12e-02	7.41e-02	179.037
217456_x_at	9.13e-02	7.42e-02	-178.962
209212_s_at	9.14e-02	7.42e-02	1.789.537
216022_at	9.15e-02	7.44e-02	-1.788.595
215856_at	9.17e-02	7.45e-02	1.787.646
203248_at	9.18e-02	7.46e-02	1.787.305
205209_at	9.18e-02	7.46e-02	1.787.007
209993_at	9.18e-02	7.46e-02	-1.786.926
214875_x_at	9.21e-02	7.48e-02	1.785.786
205045_at	9.21e-02	7.49e-02	-1.785.403
206638_at	9.22e-02	7.49e-02	-1.785.139
212739_s_at	9.23e-02	7.50e-02	-1.784.729
221066_at	9.24e-02	7.51e-02	-17.839
213850_s_at	9.26e-02	7.52e-02	-1.783.221
214228_x_at	9.26e-02	7.53e-02	178.305
204821_at	9.27e-02	7.53e-02	-1.782.558
218584_at	9.27e-02	7.54e-02	-17.822
207592_s_at	9.27e-02	7.54e-02	-1.782.194
202103_at	9.27e-02	7.54e-02	-1.782.179
208599_at	9.28e-02	7.55e-02	-1.781.704
212104_s_at	9.29e-02	7.55e-02	1.781.554
206595_at	9.29e-02	7.56e-02	-1.781.216
216213_at	9.29e-02	7.56e-02	-1.781.209
218090_s_at	9.31e-02	7.57e-02	1.780.453
219451_at	9.32e-02	7.58e-02	-1.779.711
220670_at	9.33e-02	7.59e-02	-1.779.344
212299_at	9.33e-02	7.59e-02	1.779.252
214267_s_at	9.35e-02	7.60e-02	-1.778.432
208503_s_at	9.35e-02	7.61e-02	1.778.165
201611_s_at	9.35e-02	7.61e-02	-1.778.121
217666_at	9.35e-02	7.61e-02	-1.777.935
206076_at	9.36e-02	7.61e-02	-1.777.793
220897_at	9.37e-02	7.63e-02	-1.776.885
211226_at	9.38e-02	7.63e-02	-1.776.743
222292_at	9.38e-02	7.63e-02	-1.776.423
218807_at	9.39e-02	7.64e-02	1.775.902
206501_x_at	9.42e-02	7.67e-02	-1.774.537
207897_at	9.44e-02	7.69e-02	-1.773.306
208576_s_at	9.45e-02	7.69e-02	-177.304
202486_at	9.45e-02	7.69e-02	1.772.966
222224_at	9.46e-02	7.70e-02	-1.772.439
207466_at	9.46e-02	7.70e-02	-1.772.222

218066_at	9.47e-02	7.71e-02	1.772.019
211865_s_at	9.47e-02	7.71e-02	-1.771.902
215738_at	9.48e-02	7.72e-02	-1.771.377
205570_at	9.49e-02	7.73e-02	1.770.795
203254_s_at	9.49e-02	7.73e-02	1.770.789
215306_at	9.49e-02	7.73e-02	-1.770.769
215455_at	9.49e-02	7.73e-02	-1.770.685
203863_at	9.51e-02	7.74e-02	1.769.956
201926_s_at	9.51e-02	7.74e-02	-1.769.888
217449_at	9.51e-02	7.74e-02	-1.769.832
209779_at	9.52e-02	7.75e-02	-1.769.199
207881_at	9.52e-02	7.75e-02	-1.769.173
219282_s_at	9.53e-02	7.76e-02	1.768.695
203308_x_at	9.53e-02	7.76e-02	1.768.596
209452_s_at	9.53e-02	7.77e-02	-1.768.537
206787_at	9.54e-02	7.77e-02	-1.768.284
214047_s_at	9.55e-02	7.78e-02	1.767.523
213799_s_at	9.56e-02	7.79e-02	-1.766.859
212549_at	9.58e-02	7.81e-02	-1.766.086
216718_at	9.58e-02	7.81e-02	-1.765.815
210513_s_at	9.59e-02	7.82e-02	-1.765.395
217439_at	9.60e-02	7.83e-02	-176.497
205444_at	9.62e-02	7.84e-02	-1.763.816
217428_s_at	9.63e-02	7.85e-02	-1.763.481
213591_at	9.63e-02	7.85e-02	-1.763.453
215335_at	9.64e-02	7.85e-02	-1.763.225
216674_at	9.66e-02	7.88e-02	-1.761.933
215859_at	9.67e-02	7.88e-02	-1.761.633
214802_at	9.67e-02	7.88e-02	-1.761.503
203387_s_at	9.67e-02	7.89e-02	-1.761.427
211807_x_at	9.70e-02	7.91e-02	-175.991
204422_s_at	9.71e-02	7.92e-02	175.934
208036_at	9.73e-02	7.93e-02	-1.758.624
208493_at	9.75e-02	7.95e-02	-1.757.622
222170_at	9.76e-02	7.96e-02	-1.757.102
208054_at	9.77e-02	7.97e-02	-1.756.657
204115_at	9.80e-02	7.99e-02	-17.552
212797_at	9.80e-02	7.99e-02	175.512
221209_s_at	9.81e-02	8.01e-02	-1.754.347
207717_s_at	9.81e-02	8.01e-02	1.754.288
210128_s_at	9.81e-02	8.01e-02	-175.427
214137_at	9.83e-02	8.02e-02	1.753.502
215229_at	9.84e-02	8.03e-02	1.753.169
213679_at	9.85e-02	8.04e-02	1.752.515

209126_x_at	9.85e-02	8.04e-02	-175.248
217634_at	9.85e-02	8.04e-02	-1.752.392
202285_s_at	9.88e-02	8.06e-02	-175.094
203419_at	9.88e-02	8.07e-02	1.750.833
206947_at	9.89e-02	8.07e-02	-1.750.526
214230_at	9.90e-02	8.08e-02	-175.005
201906_s_at	9.93e-02	8.10e-02	1.748.694
221359_at	9.93e-02	8.10e-02	-1.748.673
205050_s_at	9.93e-02	8.10e-02	1.748.645
216547_at	9.93e-02	8.11e-02	1.748.448
209558_s_at	9.93e-02	8.11e-02	1.748.293
201028_s_at	9.95e-02	8.12e-02	-174.755
212518_at	9.96e-02	8.13e-02	1.747.175
214704_at	9.96e-02	8.13e-02	-1.746.979
202362_at	9.96e-02	8.14e-02	1.746.732
210954_s_at	9.96e-02	8.14e-02	-1.746.662
201113_at	9.97e-02	8.14e-02	-1.746.502
206941_x_at	9.97e-02	8.14e-02	-1.746.385
218745_x_at	9.97e-02	8.14e-02	1.746.334
212029_s_at	9.98e-02	8.15e-02	-174.602
215503_at	1.00e-01	8.17e-02	-1.744.894
211307_s_at	1.00e-01	8.18e-02	-1.744.449
211666_x_at	1.00e-01	8.18e-02	1.744.177
213892_s_at	1.00e-01	8.21e-02	1.742.488
203129_s_at	1.01e-01	8.23e-02	1.741.545
221382_at	1.01e-01	8.23e-02	-1.741.521
221491_x_at	1.01e-01	8.23e-02	-1.741.485
203896_s_at	1.01e-01	8.23e-02	-1.741.439
206146_s_at	1.01e-01	8.24e-02	-1.740.948
205668_at	1.01e-01	8.26e-02	1.739.723
205128_x_at	1.01e-01	8.26e-02	-1.739.582
217682_at	1.01e-01	8.27e-02	-1.739.324
206487_at	1.01e-01	8.27e-02	-1.739.222
221609_s_at	1.01e-01	8.28e-02	-1.738.677
209686_at	1.02e-01	8.30e-02	-1.737.505
221094_s_at	1.02e-01	8.30e-02	1.737.239
218506_x_at	1.02e-01	8.35e-02	-1.734.826
205499_at	1.02e-01	8.35e-02	-1.734.619
206268_at	1.02e-01	8.36e-02	-1.734.125
201737_s_at	1.02e-01	8.36e-02	-1.734.028
220650_s_at	1.02e-01	8.37e-02	-1.733.647
210062_s_at	1.02e-01	8.37e-02	-1.733.523
213694_at	1.02e-01	8.37e-02	1.733.508
216648_s_at	1.03e-01	8.39e-02	-1.732.246

213928_s_at	1.03e-01	8.39e-02	-1.732.238
215683_at	1.03e-01	8.40e-02	-1.731.572
221268_s_at	1.03e-01	8.41e-02	-1.731.488
203998_s_at	1.03e-01	8.41e-02	173.134
220620_at	1.03e-01	8.42e-02	-1.730.901
220170_at	1.03e-01	8.42e-02	-1.730.413
215077_at	1.03e-01	8.43e-02	-1.730.344
204446_s_at	1.03e-01	8.43e-02	-1.730.241
207430_s_at	1.03e-01	8.47e-02	1.728.141
209823_x_at	1.04e-01	8.47e-02	-1.727.628
212506_at	1.04e-01	8.49e-02	1.726.717
215487_x_at	1.04e-01	8.49e-02	-1.726.675
220594_at	1.04e-01	8.49e-02	-1.726.583
218436_at	1.04e-01	8.51e-02	-1.725.566
203493_s_at	1.04e-01	8.52e-02	1.725.276
202863_at	1.04e-01	8.52e-02	-1.725.192
204802_at	1.04e-01	8.52e-02	-1.725.088
216101_at	1.04e-01	8.52e-02	-1.724.923
209288_s_at	1.04e-01	8.53e-02	-1.724.312
205524_s_at	1.04e-01	8.53e-02	-1.724.301
214376_at	1.04e-01	8.54e-02	-1.723.731
212819_at	1.04e-01	8.55e-02	1.723.495
213745_at	1.05e-01	8.56e-02	1.722.945
219474_at	1.05e-01	8.57e-02	1.722.329
207211_at	1.05e-01	8.58e-02	-1.721.924
209486_at	1.05e-01	8.58e-02	-1.721.547
203274_at	1.05e-01	8.59e-02	1.720.986
216279_at	1.05e-01	8.61e-02	-1.720.292
204756_at	1.05e-01	8.61e-02	1.719.955
204631_at	1.05e-01	8.61e-02	-1.719.946
202098_s_at	1.05e-01	8.61e-02	-1.719.914
212634_at	1.05e-01	8.62e-02	-1.719.843
207577_at	1.05e-01	8.62e-02	1.719.377
204614_at	1.05e-01	8.63e-02	-1.719.122
211515_s_at	1.05e-01	8.63e-02	1.719.014
201683_x_at	1.05e-01	8.64e-02	-1.718.609
209898_x_at	1.05e-01	8.64e-02	17.184
201382_at	1.06e-01	8.65e-02	-1.718.005
217842_at	1.06e-01	8.65e-02	-1.717.896
202230_s_at	1.06e-01	8.66e-02	1.717.517
202016_at	1.06e-01	8.66e-02	-1.717.411
206482_at	1.06e-01	8.69e-02	-1.715.995
222339_x_at	1.06e-01	8.70e-02	-1.714.963
220855_at	1.06e-01	8.73e-02	-1.713.697

208009_s_at	1.06e-01	8.73e-02	-1.713.647
208514_at	1.07e-01	8.74e-02	-1.713.302
212613_at	1.07e-01	8.74e-02	-1.712.889
209697_at	1.07e-01	8.74e-02	-1.712.845
212514_x_at	1.07e-01	8.75e-02	1.712.371
220749_at	1.07e-01	8.75e-02	-1.712.355
207181_s_at	1.07e-01	8.77e-02	1.711.651
222215_at	1.07e-01	8.77e-02	-1.711.399
222117_s_at	1.07e-01	8.78e-02	171.112
216827_at	1.07e-01	8.78e-02	-1.710.711
211529_x_at	1.07e-01	8.78e-02	1.710.691
219890_at	1.07e-01	8.79e-02	-1.710.569
203290_at	1.07e-01	8.80e-02	-1.709.547
217762_s_at	1.07e-01	8.81e-02	1.709.301
220049_s_at	1.07e-01	8.81e-02	-1.709.296
210884_s_at	1.08e-01	8.82e-02	-170.852
211533_at	1.08e-01	8.83e-02	-1.708.392
210828_s_at	1.08e-01	8.83e-02	1.708.157
220542_s_at	1.08e-01	8.83e-02	-1.708.153
218943_s_at	1.08e-01	8.83e-02	1.708.084
219097_x_at	1.08e-01	8.84e-02	1.707.702
202431_s_at	1.08e-01	8.87e-02	-170.594
216767_at	1.08e-01	8.87e-02	-170.582
216954_x_at	1.08e-01	8.87e-02	-1.705.771
211195_s_at	1.08e-01	8.88e-02	-1.705.622
208357_x_at	1.08e-01	8.89e-02	-1.705.197
205256_at	1.08e-01	8.89e-02	1.704.909
60794_f_at	1.08e-01	8.89e-02	1.704.783
202120_x_at	1.09e-01	8.91e-02	1.703.684
221455_s_at	1.09e-01	8.92e-02	-1.703.257
220244_at	1.09e-01	8.93e-02	-1.702.754
216868_s_at	1.09e-01	8.95e-02	-1.701.485
207254_at	1.09e-01	8.96e-02	-170.135
209701_at	1.09e-01	8.98e-02	1.700.289
215217_at	1.09e-01	8.99e-02	169.984
208248_x_at	1.09e-01	8.99e-02	-1.699.648
220777_at	1.10e-01	9.02e-02	-1.697.935
203687_at	1.10e-01	9.02e-02	-1.697.887
209639_s_at	1.10e-01	9.03e-02	-1.697.641
211140_s_at	1.10e-01	9.03e-02	-1.697.307
209765_at	1.10e-01	9.03e-02	-1.697.247
204524_at	1.10e-01	9.04e-02	-1.696.993
202040_s_at	1.10e-01	9.05e-02	1.696.446
203376_at	1.10e-01	9.05e-02	1.696.206

220105_at	1.10e-01	9.06e-02	-1.695.973
210046_s_at	1.10e-01	9.06e-02	-1.695.843
204843_s_at	1.10e-01	9.08e-02	1.694.946
40850_at	1.10e-01	9.08e-02	1.694.837
209699_x_at	1.10e-01	9.08e-02	1.694.645
203467_at	1.11e-01	9.09e-02	-1.694.361
217800_s_at	1.11e-01	9.09e-02	-1.694.292
219040_at	1.11e-01	9.09e-02	-1.694.076
221958_s_at	1.11e-01	9.10e-02	-1.693.742
215771_x_at	1.11e-01	9.11e-02	-1.693.246
218265_at	1.11e-01	9.12e-02	-1.692.777
207550_at	1.11e-01	9.12e-02	1.692.776
219590_x_at	1.11e-01	9.12e-02	1.692.666
202702_at	1.11e-01	9.12e-02	1.692.662
220623_s_at	1.11e-01	9.14e-02	-1.691.956
214988_s_at	1.11e-01	9.16e-02	1.690.799
218401_s_at	1.11e-01	9.17e-02	1.690.352
216671_x_at	1.11e-01	9.17e-02	-169.005
213914_s_at	1.12e-01	9.20e-02	1.688.474
216061_x_at	1.12e-01	9.23e-02	1.687.124
211299_s_at	1.12e-01	9.24e-02	-1.686.741
211306_s_at	1.13e-01	9.26e-02	-1.685.541
221686_s_at	1.13e-01	9.26e-02	-1.685.279
216488_s_at	1.13e-01	9.27e-02	1.685.003
207037_at	1.13e-01	9.27e-02	1.684.883
206891_at	1.13e-01	9.28e-02	-1.684.469
202885_s_at	1.13e-01	9.28e-02	-1.684.409
203004_s_at	1.13e-01	9.29e-02	1.684.116
211455_at	1.13e-01	9.29e-02	1.683.904
204485_s_at	1.13e-01	9.31e-02	-1.682.706
203895_at	1.13e-01	9.32e-02	1.682.386
220275_at	1.13e-01	9.32e-02	-1.682.324
201226_at	1.13e-01	9.32e-02	1.682.117
202802_at	1.14e-01	9.35e-02	1.680.962
212515_s_at	1.14e-01	9.36e-02	1.680.476
210670_at	1.14e-01	9.37e-02	-1.679.686
207130_at	1.14e-01	9.39e-02	-1.678.973
214983_at	1.14e-01	9.39e-02	-1.678.716
216538_at	1.14e-01	9.43e-02	-167.682
215505_s_at	1.15e-01	9.45e-02	-167.562
206752_s_at	1.15e-01	9.47e-02	-1.674.883
214329_x_at	1.15e-01	9.47e-02	-1.674.805
204913_s_at	1.15e-01	9.47e-02	1.674.548
205983_at	1.15e-01	9.49e-02	-1.673.517

206538_at	1.15e-01	9.49e-02	-1.673.415
209777_s_at	1.15e-01	9.50e-02	1.673.398
217099_s_at	1.15e-01	9.51e-02	-1.672.437
212274_at	1.15e-01	9.52e-02	1.672.346
208829_at	1.15e-01	9.52e-02	1.672.327
206435_at	1.15e-01	9.52e-02	167.218
212356_at	1.16e-01	9.53e-02	-1.671.729
219730_at	1.16e-01	9.53e-02	1.671.715
202578_s_at	1.16e-01	9.53e-02	-1.671.585
211875_x_at	1.16e-01	9.54e-02	-1.670.958
208026_at	1.16e-01	9.55e-02	-1.670.736
207583_at	1.16e-01	9.55e-02	-1.670.694
220247_at	1.16e-01	9.55e-02	-1.670.533
204051_s_at	1.16e-01	9.56e-02	1.670.154
219708_at	1.16e-01	9.57e-02	1.669.765
206228_at	1.16e-01	9.57e-02	-1.669.647
217548_at	1.16e-01	9.58e-02	1.668.994
216001_at	1.16e-01	9.59e-02	-1.668.703
220717_at	1.16e-01	9.59e-02	-1.668.632
216588_at	1.16e-01	9.59e-02	1.668.507
216303_s_at	1.16e-01	9.60e-02	-1.668.164
201778_s_at	1.17e-01	9.64e-02	1.666.312
220656_at	1.17e-01	9.64e-02	-1.666.293
218546_at	1.17e-01	9.65e-02	-1.665.821
218800_at	1.17e-01	9.65e-02	1.665.677
210240_s_at	1.17e-01	9.66e-02	-1.665.185
216777_at	1.17e-01	9.67e-02	-166.478
208425_s_at	1.17e-01	9.68e-02	-1.664.084
207816_at	1.17e-01	9.69e-02	-1.663.733
202587_s_at	1.17e-01	9.69e-02	1.663.672
211431_s_at	1.18e-01	9.76e-02	-1.660.159
210873_x_at	1.18e-01	9.76e-02	-1.660.064
213833_x_at	1.18e-01	9.76e-02	1.660.054
217163_at	1.18e-01	9.76e-02	-1.659.928
211326_x_at	1.18e-01	9.77e-02	-1.659.678
212651_at	1.18e-01	9.78e-02	1.659.311
205957_at	1.18e-01	9.78e-02	-1.659.171
216318_at	1.19e-01	9.80e-02	-1.658.181
205121_at	1.19e-01	9.80e-02	-1.658.078
207361_at	1.19e-01	9.82e-02	-165.704
202087_s_at	1.19e-01	9.83e-02	-1.656.817
206095_s_at	1.19e-01	9.83e-02	-1.656.794
203697_at	1.19e-01	9.83e-02	1.656.742
201222_s_at	1.19e-01	9.83e-02	1.656.666

202434_s_at	1.19e-01	9.83e-02	-1.656.443
214381_at	1.19e-01	9.84e-02	1.656.319
209144_s_at	1.19e-01	9.84e-02	165.613
202186_x_at	1.19e-01	9.84e-02	-1.656.086
202205_at	1.19e-01	9.84e-02	1.655.997
205323_s_at	1.19e-01	9.86e-02	1.654.995
206851_at	1.19e-01	9.87e-02	-1.654.742
211551_at	1.19e-01	9.87e-02	-1.654.715
217134_at	1.20e-01	9.88e-02	-1.654.382
207066_at	1.20e-01	9.88e-02	-1.654.368
201056_at	1.20e-01	9.89e-02	-1.653.632
222248_s_at	1.20e-01	9.95e-02	1.650.935
215058_at	1.20e-01	9.95e-02	1.650.587
219757_s_at	1.20e-01	9.96e-02	-1.650.449
204409_s_at	1.21e-01	9.97e-02	-1.649.854
200759_x_at	1.21e-01	9.97e-02	1.649.703
206191_at	1.21e-01	9.97e-02	-1.649.698
201316_at	1.21e-01	9.97e-02	-1.649.628
205941_s_at	1.21e-01	9.98e-02	-1.649.237
203458_at	1.21e-01	9.98e-02	-1.649.224
208019_at	1.21e-01	1.00e-01	1.647.474
218824_at	1.21e-01	1.00e-01	1.647.106
213173_at	1.21e-01	1.00e-01	1.646.695
203757_s_at	1.21e-01	1.00e-01	-1.646.619
210713_at	1.21e-01	1.00e-01	-1.646.219
219039_at	1.22e-01	1.01e-01	-1.645.802
212974_at	1.22e-01	1.01e-01	-1.645.491
204269_at	1.22e-01	1.01e-01	-164.536
220076_at	1.22e-01	1.01e-01	1.645.042
212000_at	1.22e-01	1.01e-01	1.644.827
220233_at	1.22e-01	1.01e-01	-1.644.616
203390_s_at	1.22e-01	1.01e-01	-1.643.907
218864_at	1.22e-01	1.01e-01	-1.643.129
203965_at	1.22e-01	1.01e-01	1.643.046
216018_at	1.22e-01	1.01e-01	-1.642.893
220196_at	1.22e-01	1.01e-01	-1.642.312
201767_s_at	1.23e-01	1.01e-01	164.154
205889_s_at	1.23e-01	1.01e-01	-1.641.373
218729_at	1.23e-01	1.01e-01	-1.641.155
219841_at	1.23e-01	1.02e-01	-1.640.639
201426_s_at	1.23e-01	1.02e-01	1.640.495
217432_s_at	1.23e-01	1.02e-01	-163.826
201052_s_at	1.23e-01	1.02e-01	1.638.121
211047_x_at	1.23e-01	1.02e-01	1.637.964

215214_at	1.23e-01	1.02e-01	-1.637.946
218067_s_at	1.24e-01	1.03e-01	1.635.282
214555_at	1.24e-01	1.03e-01	-1.635.191
203151_at	1.24e-01	1.03e-01	-1.634.934
215766_at	1.24e-01	1.03e-01	-1.634.562
218428_s_at	1.24e-01	1.03e-01	-1.634.483
208331_at	1.24e-01	1.03e-01	-1.634.017
216620_s_at	1.24e-01	1.03e-01	-1.633.594
215781_s_at	1.24e-01	1.03e-01	1.633.395
214247_s_at	1.25e-01	1.03e-01	-1.632.636
205626_s_at	1.25e-01	1.03e-01	1.632.548
222079_at	1.25e-01	1.03e-01	-163.231
204447_at	1.25e-01	1.03e-01	1.631.865
217460_at	1.25e-01	1.04e-01	-1.631.063
202493_x_at	1.25e-01	1.04e-01	-1.630.972
206503_x_at	1.25e-01	1.04e-01	1.630.806
220474_at	1.25e-01	1.04e-01	-1.630.686
219654_at	1.25e-01	1.04e-01	1.629.983
214304_x_at	1.25e-01	1.04e-01	-1.629.597
205544_s_at	1.26e-01	1.04e-01	-1.628.988
206129_s_at	1.26e-01	1.04e-01	1.628.738
203906_at	1.26e-01	1.04e-01	1.628.436
211319_at	1.26e-01	1.04e-01	-1.628.262
211670_x_at	1.26e-01	1.04e-01	162.825
210175_at	1.26e-01	1.04e-01	1.627.811
219109_at	1.26e-01	1.04e-01	1.627.743
201868_s_at	1.26e-01	1.04e-01	-1.627.676
218256_s_at	1.26e-01	1.04e-01	1.627.565
211829_s_at	1.26e-01	1.04e-01	-1.627.368
215885_at	1.26e-01	1.04e-01	-1.627.224
221262_s_at	1.26e-01	1.04e-01	-1.627.107
217316_at	1.26e-01	1.04e-01	-162.678
215015_at	1.26e-01	1.05e-01	-1.626.086
203085_s_at	1.26e-01	1.05e-01	1.626.075
221701_s_at	1.26e-01	1.05e-01	1.625.862
213665_at	1.26e-01	1.05e-01	1.625.402
201438_at	1.26e-01	1.05e-01	-1.625.142
206382_s_at	1.26e-01	1.05e-01	-1.624.894
201865_x_at	1.27e-01	1.05e-01	-1.624.337
207263_x_at	1.27e-01	1.05e-01	-1.624.273
221450_x_at	1.27e-01	1.05e-01	-1.623.629
217125_at	1.27e-01	1.05e-01	-1.622.129
211407_at	1.27e-01	1.06e-01	-1.621.714
213415_at	1.27e-01	1.06e-01	-1.621.427

216171_at	1.27e-01	1.06e-01	-1.621.397
204736_s_at	1.27e-01	1.06e-01	162.121
221127_s_at	1.27e-01	1.06e-01	1.620.929
220868_s_at	1.27e-01	1.06e-01	1.620.751
212665_at	1.27e-01	1.06e-01	1.620.596
210874_s_at	1.28e-01	1.06e-01	-1.619.989
213815_x_at	1.28e-01	1.06e-01	-1.619.748
219491_at	1.28e-01	1.06e-01	1.619.559
205344_at	1.28e-01	1.06e-01	-1.619.382
216447_at	1.28e-01	1.06e-01	-1.617.736
220176_at	1.28e-01	1.06e-01	-1.617.517
211863_x_at	1.28e-01	1.07e-01	-1.617.126
217271_at	1.28e-01	1.07e-01	-1.617.022
209946_at	1.28e-01	1.07e-01	-1.616.921
204555_s_at	1.28e-01	1.07e-01	-1.616.668
210487_at	1.29e-01	1.07e-01	-1.616.284
221183_at	1.29e-01	1.07e-01	-1.616.119
208004_at	1.29e-01	1.07e-01	-161.601
220887_at	1.29e-01	1.07e-01	1.615.232
218515_at	1.29e-01	1.07e-01	1.614.604
207682_s_at	1.29e-01	1.07e-01	-1.614.266
214158_s_at	1.29e-01	1.07e-01	-1.614.072
219618_at	1.29e-01	1.07e-01	-1.614.046
211419_s_at	1.29e-01	1.07e-01	-1.613.049
213139_at	1.30e-01	1.08e-01	1.611.806
206015_s_at	1.30e-01	1.08e-01	1.611.741
205148_s_at	1.30e-01	1.08e-01	1.611.156
203185_at	1.30e-01	1.08e-01	-1.610.993
209414_at	1.30e-01	1.08e-01	1.610.607
39313_at	1.30e-01	1.08e-01	1.609.469
220493_at	1.31e-01	1.09e-01	-1.608.125
218691_s_at	1.31e-01	1.09e-01	-1.607.662
203822_s_at	1.31e-01	1.09e-01	-1.607.362
218469_at	1.31e-01	1.09e-01	1.607.312
208005_at	1.31e-01	1.09e-01	-1.607.286
204862_s_at	1.31e-01	1.09e-01	1.607.277
216876_s_at	1.31e-01	1.09e-01	-1.607.026
202618_s_at	1.31e-01	1.09e-01	1.606.998
203578_s_at	1.31e-01	1.09e-01	1.605.597
204896_s_at	1.31e-01	1.09e-01	-160.512
216709_at	1.31e-01	1.09e-01	-1.604.773
220984_s_at	1.31e-01	1.09e-01	-160.474
217575_s_at	1.32e-01	1.10e-01	-160.351
211665_s_at	1.32e-01	1.10e-01	1.603.484

206321_at	1.32e-01	1.10e-01	-1.602.276
207481_at	1.32e-01	1.10e-01	-1.601.813
214028_x_at	1.32e-01	1.10e-01	-1.601.735
214588_s_at	1.32e-01	1.10e-01	160.079
204662_at	1.32e-01	1.10e-01	1.600.543
214807_at	1.33e-01	1.10e-01	-1.600.051
213814_s_at	1.33e-01	1.10e-01	-1.599.135
203457_at	1.33e-01	1.11e-01	1.598.893
213343_s_at	1.33e-01	1.11e-01	-1.598.607
210941_at	1.33e-01	1.11e-01	-1.598.103
209635_at	1.33e-01	1.11e-01	1.597.875
41397_at	1.33e-01	1.11e-01	1.597.714
214149_s_at	1.33e-01	1.11e-01	1.597.425
212673_at	1.33e-01	1.11e-01	1.597.118
220237_at	1.33e-01	1.11e-01	1.596.414
207764_s_at	1.33e-01	1.11e-01	-1.596.323
210037_s_at	1.34e-01	1.11e-01	-1.594.954
221276_s_at	1.34e-01	1.11e-01	-1.594.841
216386_at	1.34e-01	1.12e-01	-1.594.425
216084_at	1.34e-01	1.12e-01	-1.593.587
222184_at	1.34e-01	1.12e-01	-1.593.362
219471_at	1.35e-01	1.12e-01	-1.591.922
217544_at	1.35e-01	1.12e-01	1.591.918
208290_s_at	1.35e-01	1.12e-01	-1.591.701
206888_s_at	1.35e-01	1.12e-01	-1.591.621
210350_x_at	1.35e-01	1.12e-01	-159.029
211611_s_at	1.35e-01	1.13e-01	-1.590.121
201711_x_at	1.35e-01	1.13e-01	-1.589.669
219473_at	1.35e-01	1.13e-01	1.589.492
202565_s_at	1.35e-01	1.13e-01	1.589.108
217645_at	1.35e-01	1.13e-01	-1.588.683
215743_at	1.36e-01	1.13e-01	-1.587.678
214780_s_at	1.36e-01	1.13e-01	1.587.173
217248_s_at	1.36e-01	1.13e-01	-1.586.771
203934_at	1.36e-01	1.13e-01	-1.586.027
207478_at	1.36e-01	1.13e-01	-1.585.864
221851_at	1.36e-01	1.13e-01	1.585.772
204454_at	1.36e-01	1.14e-01	-1.585.527
218273_s_at	1.36e-01	1.14e-01	1.585.062
205604_at	1.36e-01	1.14e-01	158.504
219568_x_at	1.36e-01	1.14e-01	1.584.894
215078_at	1.36e-01	1.14e-01	-1.584.815
205689_at	1.37e-01	1.14e-01	1.584.384
217417_at	1.37e-01	1.14e-01	-1.583.782

216258_s_at	1.37e-01	1.14e-01	-158.318
200927_s_at	1.37e-01	1.14e-01	1.582.991
211818_s_at	1.37e-01	1.14e-01	-1.582.787
222332_at	1.37e-01	1.14e-01	-1.582.523
216615_s_at	1.37e-01	1.14e-01	-1.581.575
207048_at	1.37e-01	1.14e-01	-1.581.437
201280_s_at	1.38e-01	1.15e-01	1.579.689
211804_s_at	1.38e-01	1.15e-01	1.579.485
201511_at	1.38e-01	1.15e-01	1.579.448
214496_x_at	1.38e-01	1.15e-01	-1.578.982
220870_at	1.38e-01	1.15e-01	-1.578.791
203990_s_at	1.38e-01	1.15e-01	1.578.262
210602_s_at	1.38e-01	1.15e-01	-1.577.658
217136_at	1.38e-01	1.16e-01	-1.576.955
209321_s_at	1.38e-01	1.16e-01	-1.576.884
214051_at	1.38e-01	1.16e-01	-1.576.855
208460_at	1.39e-01	1.16e-01	-1.576.387
215670_s_at	1.39e-01	1.16e-01	-1.574.642
220979_s_at	1.39e-01	1.16e-01	157.438
216253_s_at	1.39e-01	1.16e-01	1.574.121
204132_s_at	1.39e-01	1.16e-01	-1.573.731
220739_s_at	1.39e-01	1.16e-01	-1.573.065
32069_at	1.40e-01	1.17e-01	1.570.521
214668_at	1.40e-01	1.17e-01	-1.570.453
208573_s_at	1.40e-01	1.17e-01	-1.570.005
205422_s_at	1.41e-01	1.17e-01	-1.569.176
39729_at	1.41e-01	1.18e-01	1.567.722
218837_s_at	1.41e-01	1.18e-01	1.567.498
210664_s_at	1.41e-01	1.18e-01	1.566.541
214105_at	1.41e-01	1.18e-01	-1.566.096
215990_s_at	1.42e-01	1.18e-01	-1.565.448
213531_s_at	1.42e-01	1.18e-01	1.565.421
215543_s_at	1.42e-01	1.19e-01	-1.563.784
221451_s_at	1.42e-01	1.19e-01	-1.563.087
214254_at	1.42e-01	1.19e-01	1.562.946
200747_s_at	1.42e-01	1.19e-01	-1.562.826
220115_s_at	1.42e-01	1.19e-01	1.562.625
207675_x_at	1.42e-01	1.19e-01	-1.562.143
219124_at	1.43e-01	1.19e-01	1.561.138
206432_at	1.43e-01	1.19e-01	-1.560.165
208789_at	1.43e-01	1.19e-01	-1.560.015
205007_s_at	1.43e-01	1.20e-01	1.559.166
213787_s_at	1.43e-01	1.20e-01	-1.558.368
212786_at	1.44e-01	1.20e-01	1.557.642

216426_at	1.44e-01	1.20e-01	-1.557.356
219154_at	1.44e-01	1.20e-01	1.556.664
53912_at	1.44e-01	1.20e-01	1.556.143
210636_at	1.44e-01	1.21e-01	-1.555.287
210448_s_at	1.44e-01	1.21e-01	-1.555.137
217675_at	1.44e-01	1.21e-01	-1.554.908
222134_at	1.45e-01	1.21e-01	-1.553.498
214471_x_at	1.45e-01	1.21e-01	-1.553.418
218337_at	1.45e-01	1.21e-01	-1.553.404
201498_at	1.45e-01	1.21e-01	1.553.298
200686_s_at	1.45e-01	1.21e-01	-1.553.184
201103_x_at	1.45e-01	1.21e-01	155.303
212054_x_at	1.45e-01	1.22e-01	1.551.365
222340_at	1.45e-01	1.22e-01	-1.550.926
218368_s_at	1.45e-01	1.22e-01	-1.550.683
210188_at	1.46e-01	1.22e-01	-1.550.182
221044_s_at	1.46e-01	1.22e-01	-15.499
201545_s_at	1.46e-01	1.22e-01	1.549.718
201799_s_at	1.46e-01	1.22e-01	-1.549.595
205144_at	1.46e-01	1.22e-01	-1.549.386
220265_at	1.46e-01	1.22e-01	1.549.381
218950_at	1.46e-01	1.22e-01	-1.549.199
214309_s_at	1.46e-01	1.22e-01	-1.549.056
215655_at	1.46e-01	1.22e-01	1.549.047
210455_at	1.46e-01	1.22e-01	-154.896
201452_at	1.46e-01	1.22e-01	-1.548.852
204553_x_at	1.46e-01	1.22e-01	1.548.583
221158_at	1.46e-01	1.22e-01	15.481
218791_s_at	1.46e-01	1.23e-01	1.546.941
220277_at	1.47e-01	1.23e-01	-1.546.652
219074_at	1.47e-01	1.23e-01	-1.546.609
216754_at	1.47e-01	1.23e-01	-1.545.583
208462_s_at	1.47e-01	1.23e-01	-1.545.322
212527_at	1.47e-01	1.23e-01	-1.544.759
215124_at	1.47e-01	1.23e-01	-1.544.552
207605_x_at	1.47e-01	1.23e-01	-1.543.674
209707_at	1.48e-01	1.24e-01	1.541.613
215340_at	1.48e-01	1.24e-01	-1.541.461
211274_at	1.48e-01	1.24e-01	-1.540.981
212488_at	1.48e-01	1.24e-01	-1.540.259
218786_at	1.48e-01	1.24e-01	1.539.865
216925_s_at	1.48e-01	1.24e-01	-1.539.601
204096_s_at	1.48e-01	1.24e-01	1.539.469
213411_at	1.49e-01	1.24e-01	1.539.272

215729_s_at	1.49e-01	1.25e-01	-1.538.948
205258_at	1.49e-01	1.25e-01	1.538.772
221978_at	1.49e-01	1.25e-01	1.537.897
219224_x_at	1.49e-01	1.25e-01	1.537.882
204573_at	1.49e-01	1.25e-01	-1.537.666
219187_at	1.49e-01	1.25e-01	-1.537.409
220883_at	1.49e-01	1.25e-01	-1.537.146
205914_s_at	1.49e-01	1.25e-01	-1.537.129
220580_at	1.49e-01	1.25e-01	1.536.867
203661_s_at	1.49e-01	1.25e-01	-1.536.738
219308_s_at	1.50e-01	1.25e-01	-1.535.605
208450_at	1.50e-01	1.25e-01	-1.535.156
206796_at	1.50e-01	1.26e-01	-1.534.655
203644_s_at	1.50e-01	1.26e-01	-1.534.642
56821_at	1.50e-01	1.26e-01	1.534.164
214825_at	1.50e-01	1.26e-01	-1.532.815
201720_s_at	1.50e-01	1.26e-01	153.272
204696_s_at	1.50e-01	1.26e-01	-1.532.688
206249_at	1.51e-01	1.26e-01	-1.531.175
210402_at	1.51e-01	1.27e-01	-1.530.668
219965_s_at	1.51e-01	1.27e-01	1.529.843
208558_at	1.51e-01	1.27e-01	-1.529.413
210698_at	1.52e-01	1.27e-01	-1.527.775
219254_at	1.52e-01	1.28e-01	1.526.402
219592_at	1.52e-01	1.28e-01	1.526.266
215898_at	1.52e-01	1.28e-01	-1.525.949
213765_at	1.52e-01	1.28e-01	-1.525.673
220270_at	1.52e-01	1.28e-01	1.525.169
203649_s_at	1.52e-01	1.28e-01	-1.525.142
221421_s_at	1.53e-01	1.28e-01	-1.524.641
220302_at	1.53e-01	1.28e-01	-1.524.616
204130_at	1.53e-01	1.28e-01	1.524.171
210220_at	1.53e-01	1.28e-01	-1.524.129
216321_s_at	1.53e-01	1.28e-01	1.524.061
219692_at	1.53e-01	1.28e-01	-152.367
211128_at	1.53e-01	1.28e-01	1.523.207
217613_at	1.53e-01	1.28e-01	-1.523.016
210882_s_at	1.53e-01	1.28e-01	1.522.906
220388_at	1.53e-01	1.29e-01	-1.522.817
219975_x_at	1.54e-01	1.29e-01	-1.520.098
220910_at	1.54e-01	1.29e-01	-1.520.008
214347_s_at	1.54e-01	1.29e-01	1.520.005
216104_at	1.54e-01	1.29e-01	-1.52
207009_at	1.54e-01	1.29e-01	-1.519.245

202350_s_at	1.54e-01	1.29e-01	-1.519.024
221251_x_at	1.54e-01	1.30e-01	-151.868
208427_s_at	1.54e-01	1.30e-01	-1.518.374
220959_s_at	1.54e-01	1.30e-01	-1.518.127
204261_s_at	1.55e-01	1.30e-01	-1.517.778
205655_at	1.55e-01	1.30e-01	1.517.011
211872_s_at	1.55e-01	1.30e-01	-1.516.943
216486_x_at	1.55e-01	1.30e-01	-1.516.543
36830_at	1.55e-01	1.30e-01	-1.516.155
217369_at	1.55e-01	1.30e-01	-1.515.897
210411_s_at	1.55e-01	1.30e-01	-1.515.375
210297_s_at	1.55e-01	1.30e-01	1.515.169
205514_at	1.55e-01	1.30e-01	1.515.032
210441_at	1.55e-01	1.31e-01	-1.514.755
210971_s_at	1.55e-01	1.31e-01	1.514.545
208158_s_at	1.56e-01	1.31e-01	-1.513.864
207913_at	1.56e-01	1.31e-01	-1.513.388
213771_at	1.56e-01	1.31e-01	1.513.042
207010_at	1.56e-01	1.31e-01	-1.512.952
214722_at	1.56e-01	1.31e-01	-1.512.797
214155_s_at	1.56e-01	1.31e-01	1.512.522
202928_s_at	1.56e-01	1.31e-01	1.512.245
219999_at	1.56e-01	1.31e-01	-1.511.428
215970_at	1.56e-01	1.31e-01	-1.510.998
219916_s_at	1.56e-01	1.32e-01	-1.510.747
202771_at	1.56e-01	1.32e-01	-1.510.654
217869_at	1.56e-01	1.32e-01	-1.510.602
206479_at	1.57e-01	1.32e-01	-1.510.524
209311_at	1.57e-01	1.32e-01	150.919
221302_at	1.57e-01	1.32e-01	-150.912
219900_s_at	1.57e-01	1.32e-01	1.508.776
213087_s_at	1.57e-01	1.32e-01	1.508.662
220422_at	1.57e-01	1.32e-01	-1.507.992
214953_s_at	1.57e-01	1.32e-01	-150.745
210358_x_at	1.57e-01	1.32e-01	-1.507.098
210855_at	1.58e-01	1.33e-01	-1.506.765
217124_at	1.58e-01	1.33e-01	-1.506.191
203161_s_at	1.58e-01	1.33e-01	-1.505.637
217332_at	1.58e-01	1.33e-01	-1.505.179
204749_at	1.58e-01	1.33e-01	-1.504.205
35846_at	1.59e-01	1.34e-01	-1.503.102
218876_at	1.59e-01	1.34e-01	-1.503.031
208459_s_at	1.59e-01	1.34e-01	1.502.651
202864_s_at	1.59e-01	1.34e-01	-1.502.463

210730_s_at	1.59e-01	1.34e-01	-1.502.437
210280_at	1.59e-01	1.34e-01	1.502.157
219989_s_at	1.59e-01	1.34e-01	-1.501.807
217355_at	1.59e-01	1.34e-01	-1.501.434
221324_at	1.59e-01	1.34e-01	-1.500.401
219643_at	1.60e-01	1.34e-01	1.499.364
209922_at	1.60e-01	1.35e-01	1.498.995
211573_x_at	1.60e-01	1.35e-01	-149.847
219702_at	1.60e-01	1.35e-01	1.498.414
220066_at	1.60e-01	1.35e-01	1.498.356
205546_s_at	1.60e-01	1.35e-01	-1.498.352
201780_s_at	1.60e-01	1.35e-01	1.498.337
204893_s_at	1.60e-01	1.35e-01	149.808
208275_x_at	1.60e-01	1.35e-01	-1.497.575
201909_at	1.60e-01	1.35e-01	-1.497.545
220082_at	1.60e-01	1.35e-01	-1.497.451
206346_at	1.60e-01	1.35e-01	1.497.209
222246_at	1.60e-01	1.35e-01	-1.497.138
219784_at	1.61e-01	1.35e-01	-149.573
202747_s_at	1.61e-01	1.36e-01	1.495.129
217076_s_at	1.61e-01	1.36e-01	-1.493.735
210905_x_at	1.61e-01	1.36e-01	-1.493.332
213346_at	1.61e-01	1.36e-01	-1.493.229
211675_s_at	1.62e-01	1.36e-01	-1.492.675
203916_at	1.62e-01	1.36e-01	1.492.176
212753_at	1.62e-01	1.37e-01	1.490.747
203463_s_at	1.62e-01	1.37e-01	-1.490.505
218153_at	1.62e-01	1.37e-01	1.490.493
203488_at	1.62e-01	1.37e-01	1.490.319
215786_at	1.62e-01	1.37e-01	-1.490.006
221274_s_at	1.62e-01	1.37e-01	1.489.769
221961_at	1.62e-01	1.37e-01	-1.489.716
208704_x_at	1.63e-01	1.37e-01	-1.489.493
213584_s_at	1.63e-01	1.37e-01	-1.489.384
206403_at	1.63e-01	1.37e-01	-1.488.115
221664_s_at	1.63e-01	1.37e-01	1.488.106
211873_s_at	1.63e-01	1.38e-01	-1.487.435
218792_s_at	1.63e-01	1.38e-01	1.487.051
203230_at	1.64e-01	1.38e-01	-1.486.093
206383_s_at	1.64e-01	1.38e-01	-1.485.973
220405_at	1.64e-01	1.38e-01	-148.552
213099_at	1.64e-01	1.38e-01	1.485.499
36888_at	1.64e-01	1.38e-01	-1.485.242
220522_at	1.64e-01	1.38e-01	-1.484.207

214760_at	1.65e-01	1.39e-01	1.482.931
213922_at	1.65e-01	1.39e-01	-1.481.335
206315_at	1.65e-01	1.39e-01	-1.481.138
218432_at	1.65e-01	1.40e-01	-1.480.139
219683_at	1.66e-01	1.40e-01	1.478.768
211923_s_at	1.66e-01	1.40e-01	-1.478.422
217853_at	1.66e-01	1.40e-01	1.478.198
204992_s_at	1.66e-01	1.40e-01	-1.477.477
204437_s_at	1.66e-01	1.40e-01	1.477.333
221202_at	1.66e-01	1.40e-01	1.477.262
202790_at	1.66e-01	1.40e-01	1.477.037
205737_at	1.66e-01	1.40e-01	1.476.776
213746_s_at	1.66e-01	1.40e-01	-1.476.627
215795_at	1.67e-01	1.41e-01	1.475.915
205637_s_at	1.67e-01	1.41e-01	1.475.875
214608_s_at	1.67e-01	1.41e-01	-1.474.728
207290_at	1.67e-01	1.41e-01	-1.473.997
210842_at	1.67e-01	1.41e-01	-1.473.681
210136_at	1.67e-01	1.41e-01	-1.473.489
210875_s_at	1.67e-01	1.41e-01	1.472.991
217844_at	1.68e-01	1.41e-01	-147.289
220353_at	1.68e-01	1.42e-01	1.472.248
211203_s_at	1.68e-01	1.42e-01	1.471.859
217699_at	1.68e-01	1.42e-01	-1.471.772
200940_s_at	1.68e-01	1.42e-01	-1.471.601
210655_s_at	1.68e-01	1.42e-01	1.471.451
55705_at	1.68e-01	1.42e-01	-1.471.139
219057_at	1.68e-01	1.42e-01	-1.469.966
214297_at	1.69e-01	1.42e-01	1.469.404
215927_at	1.69e-01	1.43e-01	1.468.976
215705_at	1.69e-01	1.43e-01	-1.468.472
220502_s_at	1.69e-01	1.43e-01	-1.467.152
200899_s_at	1.69e-01	1.43e-01	-1.466.987
201087_at	1.70e-01	1.43e-01	1.465.541
213538_at	1.70e-01	1.44e-01	-146.545
209724_s_at	1.70e-01	1.44e-01	-1.465.339
219885_at	1.70e-01	1.44e-01	146.444
207850_at	1.70e-01	1.44e-01	-1.464.146
218573_at	1.70e-01	1.44e-01	-1.463.863
205036_at	1.71e-01	1.44e-01	1.462.965
65086_at	1.71e-01	1.44e-01	1.462.754
204927_at	1.71e-01	1.44e-01	-1.462.694
211044_at	1.71e-01	1.44e-01	-1.462.349
208291_s_at	1.71e-01	1.44e-01	-1.462.212

216314_at	1.71e-01	1.44e-01	-1.462.179
213544_at	1.71e-01	1.44e-01	-1.461.988
201354_s_at	1.71e-01	1.45e-01	1.461.442
210309_at	1.71e-01	1.45e-01	-1.460.908
209157_at	1.71e-01	1.45e-01	1.460.588
203168_at	1.72e-01	1.45e-01	-145.884
218354_at	1.72e-01	1.46e-01	145.769
215715_at	1.72e-01	1.46e-01	1.456.969
216456_at	1.72e-01	1.46e-01	-145.658
220078_at	1.72e-01	1.46e-01	1.456.514
213379_at	1.72e-01	1.46e-01	-1.456.499
204539_s_at	1.73e-01	1.46e-01	-1.456.204
208542_x_at	1.73e-01	1.46e-01	1.455.921
211333_s_at	1.73e-01	1.47e-01	-1.454.023
213510_x_at	1.73e-01	1.47e-01	1.453.941
206656_s_at	1.73e-01	1.47e-01	-1.453.433
216137_s_at	1.73e-01	1.47e-01	-1.453.356
211338_at	1.73e-01	1.47e-01	1.453.318
60471_at	1.74e-01	1.47e-01	-1.451.758
202133_at	1.74e-01	1.47e-01	1.451.013
220535_at	1.74e-01	1.48e-01	-1.450.329
204344_s_at	1.74e-01	1.48e-01	1.450.171
213401_s_at	1.75e-01	1.48e-01	-1.449.916
209535_s_at	1.75e-01	1.48e-01	144.976
217066_s_at	1.75e-01	1.48e-01	-1.449.134
218110_at	1.75e-01	1.48e-01	1.448.657
214285_at	1.76e-01	1.49e-01	1.446.802
211324_s_at	1.76e-01	1.49e-01	-1.444.568
215922_at	1.76e-01	1.49e-01	-1.444.105
210916_s_at	1.77e-01	1.50e-01	-1.443.693
202398_at	1.77e-01	1.50e-01	144.352
220913_at	1.77e-01	1.50e-01	-1.443.387
209820_s_at	1.77e-01	1.50e-01	1.442.916
217731_s_at	1.77e-01	1.50e-01	-1.442.429
211766_s_at	1.77e-01	1.50e-01	-1.442.308
201168_x_at	1.77e-01	1.50e-01	144.203
202091_at	1.77e-01	1.50e-01	-1.441.899
205013_s_at	1.77e-01	1.50e-01	1.441.852
219239_s_at	1.77e-01	1.50e-01	-1.441.667
213224_s_at	1.77e-01	1.50e-01	-144.144
215156_at	1.77e-01	1.50e-01	144.078
212817_at	1.78e-01	1.51e-01	-1.438.594
219338_s_at	1.78e-01	1.51e-01	-1.437.663
203930_s_at	1.78e-01	1.51e-01	1.437.573

204946_s_at	1.78e-01	1.51e-01	143.756
217192_s_at	1.78e-01	1.51e-01	-1.437.513
208361_s_at	1.78e-01	1.51e-01	1.437.357
219983_at	1.79e-01	1.51e-01	-1.437.242
221996_s_at	1.79e-01	1.51e-01	-1.437.049
214445_at	1.79e-01	1.51e-01	-1.436.918
205673_s_at	1.79e-01	1.51e-01	-1.436.805
221642_at	1.79e-01	1.51e-01	-1.436.771
221257_x_at	1.79e-01	1.52e-01	143.645
205918_at	1.79e-01	1.52e-01	1.436.271
210991_s_at	1.79e-01	1.52e-01	-1.435.856
214085_x_at	1.79e-01	1.52e-01	-143.542
213666_at	1.79e-01	1.52e-01	143.518
201915_at	1.79e-01	1.52e-01	1.435.035
205980_s_at	1.79e-01	1.52e-01	1.434.439
216945_x_at	1.80e-01	1.52e-01	1.433.646
209737_at	1.80e-01	1.52e-01	-1.433.513
220615_s_at	1.80e-01	1.53e-01	1.432.077
220273_at	1.80e-01	1.53e-01	-143.174
212735_at	1.80e-01	1.53e-01	143.171
206196_s_at	1.80e-01	1.53e-01	1.431.309
216641_s_at	1.80e-01	1.53e-01	1.431.017
213539_at	1.81e-01	1.53e-01	-1.430.607
202532_s_at	1.81e-01	1.53e-01	-1.430.416
210866_s_at	1.81e-01	1.53e-01	-1.430.272
214488_at	1.81e-01	1.54e-01	-1.429.552
206715_at	1.81e-01	1.54e-01	1.428.703
207916_at	1.82e-01	1.54e-01	-1.426.951
206412_at	1.82e-01	1.54e-01	1.426.938
213921_at	1.82e-01	1.55e-01	-1.425.603
208806_at	1.82e-01	1.55e-01	1.425.428
204549_at	1.83e-01	1.55e-01	1.424.618
220276_at	1.83e-01	1.55e-01	-1.424.602
203045_at	1.83e-01	1.55e-01	1.423.091
203931_s_at	1.83e-01	1.55e-01	1.423.042
207797_s_at	1.83e-01	1.55e-01	-1.422.975
205145_s_at	1.83e-01	1.56e-01	-1.422.718
208255_s_at	1.83e-01	1.56e-01	1.422.176
209405_s_at	1.83e-01	1.56e-01	1.421.574
218833_at	1.84e-01	1.56e-01	-1.421.423
214135_at	1.84e-01	1.56e-01	1.421.254
204466_s_at	1.84e-01	1.56e-01	-1.421.207
208318_s_at	1.84e-01	1.56e-01	1.420.586
221355_at	1.84e-01	1.56e-01	-142.047

218625_at	1.84e-01	1.56e-01	141.992
204072_s_at	1.84e-01	1.56e-01	141.982
214310_s_at	1.84e-01	1.57e-01	141.888
204156_at	1.84e-01	1.57e-01	-1.418.739
207341_at	1.84e-01	1.57e-01	-1.418.589
205549_at	1.85e-01	1.57e-01	-1.418.227
220158_at	1.85e-01	1.57e-01	1.417.854
219229_at	1.85e-01	1.57e-01	1.417.634
213190_at	1.85e-01	1.57e-01	1.417.461
207221_at	1.85e-01	1.57e-01	-1.417.086
215291_at	1.85e-01	1.57e-01	1.416.239
219067_s_at	1.85e-01	1.58e-01	-1.415.779
220783_at	1.85e-01	1.58e-01	-1.415.436
203492_x_at	1.86e-01	1.58e-01	1.414.477
208181_at	1.86e-01	1.58e-01	-1.413.086
220127_s_at	1.86e-01	1.58e-01	1.412.901
205906_at	1.86e-01	1.58e-01	-1.412.766
206871_at	1.87e-01	1.59e-01	-1.411.909
205579_at	1.87e-01	1.59e-01	141.187
201340_s_at	1.87e-01	1.59e-01	-1.410.946
207120_at	1.87e-01	1.59e-01	1.410.239
217452_s_at	1.87e-01	1.59e-01	1.409.995
219086_at	1.87e-01	1.59e-01	-140.935
220430_at	1.88e-01	1.60e-01	-1.408.619
210792_x_at	1.88e-01	1.60e-01	140.795
220931_at	1.88e-01	1.60e-01	-1.407.905
204769_s_at	1.88e-01	1.60e-01	1.407.827
208072_s_at	1.88e-01	1.60e-01	1.406.264
206035_at	1.89e-01	1.60e-01	-140.608
205981_s_at	1.89e-01	1.61e-01	-1.405.251
215429_s_at	1.89e-01	1.61e-01	1.404.968
209490_s_at	1.89e-01	1.61e-01	-1.404.332
206894_at	1.89e-01	1.61e-01	-1.404.025
216420_at	1.89e-01	1.61e-01	-1.403.963
204640_s_at	1.89e-01	1.61e-01	1.403.655
220557_s_at	1.89e-01	1.61e-01	-1.403.652
205376_at	1.90e-01	1.61e-01	1.402.824
207598_x_at	1.90e-01	1.61e-01	-1.402.708
215295_at	1.90e-01	1.61e-01	-1.402.622
204328_at	1.90e-01	1.62e-01	1.401.752
214791_at	1.90e-01	1.62e-01	1.400.926
203619_s_at	1.90e-01	1.62e-01	-1.400.833
214856_at	1.90e-01	1.62e-01	-140.055
217044_s_at	1.90e-01	1.62e-01	-1.400.283

219668_at	1.90e-01	1.62e-01	-139.997
208624_s_at	1.91e-01	1.62e-01	1.399.863
206633_at	1.91e-01	1.63e-01	-1.398.548
217024_x_at	1.91e-01	1.63e-01	-1.398.483
117_at	1.91e-01	1.63e-01	-1.397.614
220882_at	1.91e-01	1.63e-01	-1.397.523
214330_at	1.92e-01	1.63e-01	-1.396.212
206664_at	1.92e-01	1.63e-01	1.395.707
210872_x_at	1.92e-01	1.64e-01	-1.394.267
201304_at	1.93e-01	1.64e-01	-1.393.188
204554_at	1.93e-01	1.64e-01	1.392.811
217063_x_at	1.93e-01	1.64e-01	-1.392.781
214833_at	1.93e-01	1.64e-01	-1.392.656
35148_at	1.93e-01	1.64e-01	1.392.656
207110_at	1.93e-01	1.64e-01	-1.392.583
208363_s_at	1.93e-01	1.64e-01	139.244
211841_s_at	1.93e-01	1.65e-01	1.391.649
200026_at	1.93e-01	1.65e-01	-1.391.537
204325_s_at	1.94e-01	1.65e-01	-1.390.291
201846_s_at	1.94e-01	1.65e-01	-1.389.971
211705_s_at	1.94e-01	1.65e-01	-1.389.719
205671_s_at	1.94e-01	1.65e-01	-1.389.659
211089_s_at	1.94e-01	1.65e-01	1.389.204
216231_s_at	1.94e-01	1.65e-01	-1.389.196
214867_at	1.94e-01	1.66e-01	-1.388.833
206655_s_at	1.94e-01	1.66e-01	1.388.523
34406_at	1.94e-01	1.66e-01	1.387.949
213059_at	1.94e-01	1.66e-01	-1.387.908
215166_at	1.94e-01	1.66e-01	-1.387.896
208989_s_at	1.95e-01	1.66e-01	-1.387.634
211805_s_at	1.95e-01	1.66e-01	-1.386.857
210996_s_at	1.95e-01	1.66e-01	1.386.452
218488_at	1.95e-01	1.66e-01	1.385.964
200905_x_at	1.95e-01	1.67e-01	-1.385.728
211606_at	1.95e-01	1.67e-01	1.385.413
220132_s_at	1.95e-01	1.67e-01	1.384.951
214997_at	1.96e-01	1.67e-01	1.383.978
215666_at	1.96e-01	1.67e-01	-1.383.883
210585_s_at	1.96e-01	1.67e-01	-1.383.687
202519_at	1.96e-01	1.67e-01	-1.383.441
210719_s_at	1.96e-01	1.67e-01	-1.383.224
208346_at	1.96e-01	1.67e-01	-138.297
221998_s_at	1.96e-01	1.67e-01	-1.382.889
202821_s_at	1.96e-01	1.67e-01	1.382.595

220869_at	1.96e-01	1.68e-01	-1.382.119
207410_s_at	1.96e-01	1.68e-01	-1.382.083
207319_s_at	1.97e-01	1.68e-01	1.381.514
219557_s_at	1.97e-01	1.68e-01	-1.380.995
210412_at	1.97e-01	1.68e-01	-1.380.751
201553_s_at	1.97e-01	1.68e-01	-13.805
219408_at	1.97e-01	1.68e-01	-1.380.389
32541_at	1.97e-01	1.68e-01	-1.380.084
219577_s_at	1.97e-01	1.68e-01	-1.379.682
213132_s_at	1.97e-01	1.69e-01	1.379.216
220907_at	1.97e-01	1.69e-01	-1.379.116
221546_at	1.98e-01	1.69e-01	-1.378.554
205147_x_at	1.98e-01	1.69e-01	-1.376.361
212762_s_at	1.98e-01	1.70e-01	-1.375.998
222355_at	1.98e-01	1.70e-01	-1.375.868
218227_at	1.99e-01	1.70e-01	-1.375.516
205836_s_at	1.99e-01	1.70e-01	1.375.478
210756_s_at	1.99e-01	1.70e-01	-1.374.473
221710_x_at	1.99e-01	1.70e-01	-1.374.336
213418_at	1.99e-01	1.70e-01	1.374.191
202842_s_at	1.99e-01	1.70e-01	1.373.917
210634_at	1.99e-01	1.70e-01	-1.373.231
213331_s_at	2.00e-01	1.71e-01	1.372.525
213763_at	2.00e-01	1.71e-01	-1.372.454
217412_at	2.00e-01	1.71e-01	-1.371.533
209988_s_at	2.00e-01	1.71e-01	-1.371.391
217400_at	2.00e-01	1.71e-01	1.370.583
213446_s_at	2.01e-01	1.71e-01	-1.369.642
201686_x_at	2.01e-01	1.72e-01	1.369.311
218583_s_at	2.01e-01	1.72e-01	1.369.088
222301_at	2.01e-01	1.72e-01	-1.369.077
205143_at	2.01e-01	1.72e-01	-1.368.889
218171_at	2.01e-01	1.72e-01	1.368.626
220928_s_at	2.01e-01	1.72e-01	-1.368.102
218986_s_at	2.01e-01	1.72e-01	-1.368.052
206912_at	2.01e-01	1.72e-01	-1.367.761
214031_s_at	2.01e-01	1.72e-01	-1.367.624
220264_s_at	2.02e-01	1.72e-01	-1.366.868
211580_s_at	2.02e-01	1.72e-01	-1.366.602
215721_at	2.02e-01	1.73e-01	-1.365.021
210246_s_at	2.02e-01	1.73e-01	-1.364.581
204576_s_at	2.02e-01	1.73e-01	136.456
202944_at	2.03e-01	1.73e-01	-1.363.889
218927_s_at	2.03e-01	1.73e-01	1.363.564

215730_at	2.03e-01	1.74e-01	-1.362.706
216317_x_at	2.03e-01	1.74e-01	-1.362.451
213934_s_at	2.04e-01	1.75e-01	1.359.818
213893_x_at	2.04e-01	1.75e-01	-135.975
205385_at	2.04e-01	1.75e-01	-1.359.541
209112_at	2.04e-01	1.75e-01	-1.359.536
207047_s_at	2.04e-01	1.75e-01	-1.358.911
202851_at	2.04e-01	1.75e-01	1.358.781
207729_at	2.05e-01	1.75e-01	-1.357.931
217229_at	2.05e-01	1.75e-01	-1.357.706
217068_at	2.05e-01	1.75e-01	-1.357.508
205411_at	2.05e-01	1.76e-01	-1.356.865
210456_at	2.05e-01	1.76e-01	-1.356.774
210503_at	2.05e-01	1.76e-01	-1.356.635
202727_s_at	2.05e-01	1.76e-01	1.355.651
207636_at	2.06e-01	1.76e-01	-1.355.412
209720_s_at	2.06e-01	1.76e-01	-1.355.242
211722_s_at	2.06e-01	1.76e-01	-1.355.062
210836_x_at	2.06e-01	1.76e-01	-1.354.613
211475_s_at	2.06e-01	1.76e-01	-135.458
205701_at	2.06e-01	1.76e-01	1.353.919
205882_x_at	2.06e-01	1.76e-01	1.353.841
222290_at	2.07e-01	1.77e-01	13.522
216785_at	2.07e-01	1.77e-01	-1.351.542
201673_s_at	2.07e-01	1.77e-01	1.351.027
222238_s_at	2.08e-01	1.78e-01	1.349.332
212236_x_at	2.08e-01	1.78e-01	-1.349.144
216966_at	2.08e-01	1.78e-01	-1.348.588
220447_at	2.08e-01	1.78e-01	-134.832
210247_at	2.08e-01	1.78e-01	-1.347.555
210435_at	2.08e-01	1.79e-01	-1.347.469
216815_at	2.08e-01	1.79e-01	-1.347.205
208564_at	2.09e-01	1.79e-01	-1.346.903
91826_at	2.09e-01	1.79e-01	-1.346.846
201317_s_at	2.09e-01	1.79e-01	1.346.727
208509_s_at	2.09e-01	1.79e-01	1.346.379
208929_x_at	2.09e-01	1.79e-01	1.346.096
219211_at	2.09e-01	1.79e-01	1.346.013
206782_s_at	2.09e-01	1.79e-01	-1.344.928
45687_at	2.09e-01	1.79e-01	1.344.468
38691_s_at	2.09e-01	1.80e-01	-1.344.372
205210_at	2.09e-01	1.80e-01	-1.344.166
203516_at	2.09e-01	1.80e-01	1.344.108
221805_at	2.09e-01	1.80e-01	1.344.021

215423_at	2.10e-01	1.80e-01	-1.343.727
209727_at	2.10e-01	1.80e-01	1.342.931
221036_s_at	2.10e-01	1.80e-01	1.342.685
218677_at	2.10e-01	1.80e-01	1.342.177
202901_x_at	2.11e-01	1.81e-01	-1.340.293
205767_at	2.11e-01	1.81e-01	1.339.371
78383_at	2.11e-01	1.81e-01	1.338.534
220523_at	2.11e-01	1.81e-01	-1.338.489
214284_s_at	2.12e-01	1.82e-01	-1.338.158
206032_at	2.12e-01	1.82e-01	-1.337.981
201225_s_at	2.12e-01	1.82e-01	1.337.614
208314_at	2.12e-01	1.82e-01	-1.336.578
211161_s_at	2.12e-01	1.82e-01	-1.336.461
212084_at	2.12e-01	1.82e-01	1.336.219
213153_at	2.12e-01	1.82e-01	-1.336.171
222258_s_at	2.12e-01	1.82e-01	1.336.036
206438_x_at	2.12e-01	1.82e-01	-1.335.671
213350_at	2.13e-01	1.83e-01	-1.335.171
215718_s_at	2.13e-01	1.83e-01	1.335.127
222115_x_at	2.13e-01	1.83e-01	-1.334.951
222229_x_at	2.13e-01	1.83e-01	-1.334.834
213348_at	2.13e-01	1.83e-01	-1.333.672
202079_s_at	2.14e-01	1.83e-01	-1.332.281
215371_at	2.14e-01	1.84e-01	-1.331.958
220417_s_at	2.14e-01	1.84e-01	-1.331.537
219554_at	2.15e-01	1.84e-01	-1.329.875
208443_x_at	2.15e-01	1.84e-01	1.329.785
209681_at	2.15e-01	1.84e-01	-1.329.677
206395_at	2.15e-01	1.84e-01	-1.329.396
215832_x_at	2.15e-01	1.84e-01	1.329.134
222377_at	2.15e-01	1.85e-01	-1.328.634
209676_at	2.15e-01	1.85e-01	-1.328.532
214087_s_at	2.15e-01	1.85e-01	1.328.506
209622_at	2.15e-01	1.85e-01	132.846
212814_at	2.15e-01	1.85e-01	-1.327.761
217084_at	2.15e-01	1.85e-01	-1.327.362
215153_at	2.16e-01	1.85e-01	-132.691
200000_s_at	2.16e-01	1.85e-01	-1.326.708
215302_at	2.16e-01	1.85e-01	-1.326.581
211037_s_at	2.16e-01	1.85e-01	-1.326.385
203036_s_at	2.16e-01	1.86e-01	-1.325.959
203586_s_at	2.16e-01	1.86e-01	1.325.892
206978_at	2.16e-01	1.86e-01	-13.256
220215_at	2.16e-01	1.86e-01	1.324.829

204627_s_at	2.16e-01	1.86e-01	-1.324.768
214204_at	2.16e-01	1.86e-01	-1.324.413
201829_at	2.17e-01	1.87e-01	1.322.726
215811_at	2.17e-01	1.87e-01	-1.322.513
205388_at	2.17e-01	1.87e-01	-1.321.861
214693_x_at	2.17e-01	1.87e-01	-1.321.836
203294_s_at	2.17e-01	1.87e-01	-1.321.742
219860_at	2.18e-01	1.88e-01	1.320.023
212895_s_at	2.18e-01	1.88e-01	-1.319.511
221871_s_at	2.18e-01	1.88e-01	-1.319.452
207986_x_at	2.18e-01	1.88e-01	-131.897
201739_at	2.19e-01	1.88e-01	-1.318.667
214942_at	2.19e-01	1.88e-01	-1.318.449
220136_s_at	2.19e-01	1.88e-01	-1.318.387
204932_at	2.19e-01	1.89e-01	-1.316.896
215643_at	2.19e-01	1.89e-01	-131.671
212462_at	2.19e-01	1.89e-01	1.316.695
207971_s_at	2.19e-01	1.89e-01	1.316.564
217712_at	2.19e-01	1.89e-01	-1.316.545
210362_x_at	2.19e-01	1.89e-01	13.161
215835_at	2.20e-01	1.89e-01	-1.315.784
214262_at	2.20e-01	1.89e-01	-1.315.577
218603_at	2.20e-01	1.89e-01	-1.315.371
210867_at	2.20e-01	1.89e-01	1.315.103
217083_at	2.20e-01	1.89e-01	-1.315.061
217592_at	2.20e-01	1.89e-01	-1.314.996
208603_s_at	2.20e-01	1.89e-01	-131.476
205109_s_at	2.20e-01	1.89e-01	-1.314.478
64899_at	2.20e-01	1.89e-01	-1.314.456
219227_at	2.20e-01	1.90e-01	-1.313.779
215625_at	2.21e-01	1.90e-01	-131.238
207891_s_at	2.21e-01	1.90e-01	-1.311.942
213467_at	2.21e-01	1.90e-01	131.186
206980_s_at	2.21e-01	1.90e-01	-1.311.258
212327_at	2.21e-01	1.91e-01	-1.311.008
219760_at	2.21e-01	1.91e-01	-1.310.866
214312_at	2.21e-01	1.91e-01	-1.310.516
222004_s_at	2.22e-01	1.91e-01	-1.310.217
202551_s_at	2.22e-01	1.91e-01	-1.308.848
207815_at	2.22e-01	1.92e-01	1.307.873
207836_s_at	2.23e-01	1.92e-01	-130.757
217075_x_at	2.23e-01	1.92e-01	-1.307.491
219133_at	2.23e-01	1.92e-01	1.307.341
35974_at	2.23e-01	1.92e-01	-1.306.231

210644_s_at	2.23e-01	1.92e-01	1.305.906
202871_at	2.23e-01	1.92e-01	-1.305.881
219627_at	2.23e-01	1.93e-01	1.305.082
204257_at	2.24e-01	1.93e-01	1.304.613
212001_at	2.24e-01	1.93e-01	1.304.282
209114_at	2.24e-01	1.93e-01	-130.334
206685_at	2.24e-01	1.93e-01	130.313
215420_at	2.24e-01	1.93e-01	-1.303.051
204253_s_at	2.24e-01	1.93e-01	-130.303
206722_s_at	2.24e-01	1.93e-01	1.302.984
203766_s_at	2.25e-01	1.94e-01	-1.301.905
220478_at	2.25e-01	1.94e-01	-1.301.699
213364_s_at	2.25e-01	1.94e-01	1.300.903
216660_at	2.26e-01	1.94e-01	-1.299.423
207912_s_at	2.26e-01	1.95e-01	1.299.107
200085_s_at	2.26e-01	1.95e-01	1.298.621
203039_s_at	2.26e-01	1.95e-01	-1.298.356
216934_at	2.26e-01	1.95e-01	-1.298.252
220785_at	2.26e-01	1.95e-01	1.298.142
220402_at	2.26e-01	1.95e-01	-1.297.374
215782_at	2.26e-01	1.95e-01	-1.297.227
209246_at	2.27e-01	1.95e-01	1.296.813
209989_at	2.27e-01	1.96e-01	-1.295.931
203575_at	2.27e-01	1.96e-01	-1.295.023
209887_at	2.27e-01	1.96e-01	-1.294.992
206825_at	2.27e-01	1.96e-01	-1.294.703
216554_s_at	2.27e-01	1.96e-01	-1.294.645
213609_s_at	2.28e-01	1.96e-01	-1.293.944
213797_at	2.28e-01	1.96e-01	-1.293.653
202057_at	2.28e-01	1.96e-01	-1.293.638
202966_at	2.28e-01	1.97e-01	-129.346
221185_s_at	2.28e-01	1.97e-01	1.293.364
215658_at	2.28e-01	1.97e-01	-129.323
216200_at	2.28e-01	1.97e-01	-1.293.205
220659_s_at	2.28e-01	1.97e-01	129.278
212382_at	2.28e-01	1.97e-01	-1.291.932
222066_at	2.28e-01	1.97e-01	-1.291.832
207321_s_at	2.29e-01	1.97e-01	-1.291.381
203961_at	2.29e-01	1.97e-01	129.097
217643_x_at	2.29e-01	1.98e-01	-1.290.517
208175_s_at	2.29e-01	1.98e-01	-1.290.137
205885_s_at	2.29e-01	1.98e-01	-1.289.224
208473_s_at	2.29e-01	1.98e-01	1.288.967
211769_x_at	2.29e-01	1.98e-01	1.288.893

209744_x_at	2.29e-01	1.98e-01	-1.288.793
210036_s_at	2.30e-01	1.98e-01	1.288.289
206808_at	2.30e-01	1.99e-01	1.287.254
212505_s_at	2.30e-01	1.99e-01	128.716
205618_at	2.30e-01	1.99e-01	-1.287.142
220930_s_at	2.30e-01	1.99e-01	-1.287.084
205672_at	2.30e-01	1.99e-01	1.286.851
206038_s_at	2.30e-01	1.99e-01	-1.286.805
211828_s_at	2.30e-01	1.99e-01	1.286.093
221889_at	2.31e-01	1.99e-01	1.285.813
214793_at	2.31e-01	1.99e-01	1.285.448
203249_at	2.31e-01	1.99e-01	-1.285.259
208153_s_at	2.31e-01	1.99e-01	-1.285.195
201621_at	2.31e-01	1.99e-01	1.284.934
207617_at	2.31e-01	2.00e-01	-1.284.534
208654_s_at	2.31e-01	2.00e-01	1.284.183
203789_s_at	2.32e-01	2.00e-01	1.283.166
40446_at	2.32e-01	2.00e-01	-1.282.939
221224_s_at	2.32e-01	2.00e-01	-1.282.865
211938_at	2.32e-01	2.00e-01	-1.282.725
214462_at	2.32e-01	2.00e-01	-1.282.666
209125_at	2.32e-01	2.01e-01	1.281.811
211697_x_at	2.32e-01	2.01e-01	-1.281.725
204819_at	2.32e-01	2.01e-01	-1.281.502
217139_at	2.32e-01	2.01e-01	-128.121
220457_at	2.32e-01	2.01e-01	-1.280.568
220937_s_at	2.33e-01	2.01e-01	1.280.237
201191_at	2.33e-01	2.02e-01	-127.917
218114_at	2.33e-01	2.02e-01	1.279.018
208416_s_at	2.33e-01	2.02e-01	-1.278.458
211103_at	2.34e-01	2.02e-01	-1.277.913
213561_at	2.34e-01	2.02e-01	-1.276.868
210563_x_at	2.34e-01	2.02e-01	-1.276.344
210021_s_at	2.34e-01	2.03e-01	1.276.139
216943_at	2.34e-01	2.03e-01	-1.275.913
208631_s_at	2.34e-01	2.03e-01	-1.275.886
205710_at	2.34e-01	2.03e-01	127.588
221551_x_at	2.34e-01	2.03e-01	1.275.801
205159_at	2.34e-01	2.03e-01	1.275.544
214482_at	2.35e-01	2.03e-01	1.275.143
208555_x_at	2.35e-01	2.03e-01	1.275.129
219608_s_at	2.35e-01	2.03e-01	1.273.904
206028_s_at	2.35e-01	2.04e-01	-1.273.179
211329_x_at	2.36e-01	2.04e-01	1.272.216

219613_s_at	2.36e-01	2.04e-01	1.271.675
214452_at	2.36e-01	2.04e-01	1.271.045
215047_at	2.36e-01	2.05e-01	-1.270.595
215019_x_at	2.37e-01	2.05e-01	-1.269.945
213831_at	2.37e-01	2.05e-01	-1.269.461
219206_x_at	2.37e-01	2.05e-01	-1.269.082
220774_at	2.37e-01	2.05e-01	-1.268.264
205686_s_at	2.37e-01	2.05e-01	-1.268.175
212007_at	2.37e-01	2.05e-01	1.268.044
215185_at	2.37e-01	2.06e-01	-1.267.766
206934_at	2.38e-01	2.06e-01	-1.267.428
206404_at	2.38e-01	2.06e-01	-1.265.313
202297_s_at	2.38e-01	2.06e-01	-1.265.176
217806_s_at	2.39e-01	2.07e-01	12.649
204042_at	2.39e-01	2.07e-01	-1.264.437
202160_at	2.39e-01	2.07e-01	1.264.437
210572_at	2.39e-01	2.07e-01	-1.264.056
203072_at	2.39e-01	2.07e-01	-12.628
217489_s_at	2.40e-01	2.08e-01	1.262.214
209462_at	2.40e-01	2.08e-01	1.261.693
215994_x_at	2.40e-01	2.08e-01	1.261.435
217633_at	2.40e-01	2.08e-01	1.261.378
200914_x_at	2.40e-01	2.08e-01	1.260.646
209847_at	2.40e-01	2.08e-01	1.259.851
211739_x_at	2.41e-01	2.08e-01	-1.259.692
220693_at	2.41e-01	2.09e-01	-1.259.403
220357_s_at	2.41e-01	2.09e-01	-125.931
216746_at	2.41e-01	2.09e-01	-1.259.128
214564_s_at	2.41e-01	2.09e-01	-1.257.354
202129_s_at	2.41e-01	2.09e-01	-1.257.272
208505_s_at	2.42e-01	2.10e-01	-125.675
206290_s_at	2.42e-01	2.10e-01	1.256.382
201655_s_at	2.42e-01	2.10e-01	1.256.145
211575_s_at	2.42e-01	2.10e-01	-1.255.847
221173_at	2.42e-01	2.10e-01	1.255.658
204468_s_at	2.42e-01	2.10e-01	-1.255.594
213657_s_at	2.42e-01	2.10e-01	-1.255.257
208551_at	2.42e-01	2.10e-01	-1.254.902
213583_x_at	2.42e-01	2.10e-01	1.254.894
200979_at	2.42e-01	2.10e-01	-1.254.817
220964_s_at	2.42e-01	2.10e-01	1.254.683
218414_s_at	2.43e-01	2.10e-01	1.254.122
208819_at	2.43e-01	2.11e-01	-1.253.977
206162_x_at	2.43e-01	2.11e-01	-125.339

213433_at	2.43e-01	2.11e-01	-1.253.177
214912_at	2.43e-01	2.11e-01	-125.268
217828_at	2.43e-01	2.11e-01	-1.252.662
213202_at	2.43e-01	2.11e-01	1.252.485
208333_at	2.43e-01	2.11e-01	-1.252.343
219009_at	2.43e-01	2.11e-01	-1.251.992
215399_s_at	2.44e-01	2.11e-01	-1.251.445
212101_at	2.44e-01	2.12e-01	1.251.222
202432_at	2.44e-01	2.12e-01	12.507
213004_at	2.44e-01	2.12e-01	1.250.679
203778_at	2.44e-01	2.12e-01	1.249.693
215960_at	2.45e-01	2.12e-01	-1.249.251
206879_s_at	2.45e-01	2.12e-01	-1.249.141
202387_at	2.45e-01	2.12e-01	-1.248.944
211034_s_at	2.45e-01	2.12e-01	-1.248.512
203375_s_at	2.45e-01	2.13e-01	-1.248.302
201559_s_at	2.46e-01	2.13e-01	1.246.047
211139_s_at	2.47e-01	2.14e-01	-1.244.115
201820_at	2.47e-01	2.14e-01	-1.243.707
212102_s_at	2.47e-01	2.14e-01	1.243.665
220350_at	2.47e-01	2.14e-01	1.243.556
217088_s_at	2.47e-01	2.14e-01	-1.243.367
213870_at	2.47e-01	2.14e-01	-1.243.227
219981_x_at	2.47e-01	2.15e-01	1.242.303
211601_at	2.47e-01	2.15e-01	-1.242.285
214159_at	2.47e-01	2.15e-01	-1.242.133
204101_at	2.48e-01	2.15e-01	-1.241.464
208184_s_at	2.48e-01	2.15e-01	124.125
214664_at	2.48e-01	2.15e-01	-1.240.743
219534_x_at	2.48e-01	2.16e-01	1.240.244
203325_s_at	2.48e-01	2.16e-01	-1.239.956
219331_s_at	2.48e-01	2.16e-01	-1.239.948
209577_at	2.48e-01	2.16e-01	1.239.657
218168_s_at	2.49e-01	2.16e-01	-1.238.527
205625_s_at	2.49e-01	2.16e-01	1.238.357
209775_x_at	2.49e-01	2.16e-01	-123.833
200704_at	2.49e-01	2.16e-01	1.238.199
210033_s_at	2.49e-01	2.16e-01	1.237.693
206729_at	2.49e-01	2.17e-01	-1.237.641
216812_at	2.49e-01	2.17e-01	-1.237.611
220685_at	2.49e-01	2.17e-01	123.738
208978_at	2.49e-01	2.17e-01	-1.236.623
220030_at	2.49e-01	2.17e-01	-1.236.596
208258_s_at	2.50e-01	2.17e-01	1.236.295

218916_at	2.50e-01	2.17e-01	1.236.251
203166_at	2.50e-01	2.17e-01	-123.523
211414_at	2.50e-01	2.17e-01	-1.235.066
217637_at	2.50e-01	2.18e-01	-1.234.589
209441_at	2.51e-01	2.18e-01	123.397
201904_s_at	2.51e-01	2.18e-01	1.233.652
202063_s_at	2.51e-01	2.18e-01	1.233.109
201083_s_at	2.51e-01	2.18e-01	123.246
202450_s_at	2.51e-01	2.19e-01	1.232.077
202552_s_at	2.51e-01	2.19e-01	-1.232.029
211619_s_at	2.51e-01	2.19e-01	-1.231.864
213612_x_at	2.51e-01	2.19e-01	1.231.798
206907_at	2.51e-01	2.19e-01	-123.179
215228_at	2.52e-01	2.19e-01	-1.230.747
209113_s_at	2.52e-01	2.19e-01	1.230.607
215391_at	2.52e-01	2.19e-01	-1.230.543
217650_x_at	2.52e-01	2.19e-01	1.229.646
214446_at	2.53e-01	2.20e-01	-1.228.327
217090_at	2.53e-01	2.20e-01	-122.797
218075_at	2.53e-01	2.20e-01	-1.227.853
210512_s_at	2.53e-01	2.20e-01	1.227.555
218716_x_at	2.53e-01	2.20e-01	-1.227.055
215964_at	2.53e-01	2.21e-01	-1.226.588
204630_s_at	2.54e-01	2.21e-01	1.225.551
209799_at	2.54e-01	2.21e-01	-1.225.386
203684_s_at	2.54e-01	2.21e-01	-1.225.115
209266_s_at	2.55e-01	2.22e-01	-1.223.896
219752_at	2.55e-01	2.22e-01	-1.223.306
202820_at	2.55e-01	2.22e-01	-1.223.232
210016_at	2.56e-01	2.23e-01	1.221.057
213231_at	2.56e-01	2.23e-01	-1.220.636
216154_at	2.56e-01	2.23e-01	1.220.233
220293_at	2.57e-01	2.24e-01	1.218.812
211673_s_at	2.57e-01	2.24e-01	121.862
208495_at	2.57e-01	2.24e-01	-1.218.612
221133_s_at	2.57e-01	2.24e-01	1.218.126
209068_at	2.57e-01	2.24e-01	1.217.919
209308_s_at	2.57e-01	2.24e-01	-1.217.316
211434_s_at	2.57e-01	2.24e-01	-1.217.179
203872_at	2.57e-01	2.24e-01	-1.216.952
219302_s_at	2.58e-01	2.24e-01	-1.216.464
214991_s_at	2.58e-01	2.25e-01	1.214.622
212171_x_at	2.58e-01	2.25e-01	-1.214.605
214352_s_at	2.58e-01	2.25e-01	1.214.493

207814_at	2.58e-01	2.25e-01	-1.214.442
210829_s_at	2.59e-01	2.26e-01	-121.283
204065_at	2.59e-01	2.26e-01	-1.212.667
213998_s_at	2.59e-01	2.26e-01	-1.212.295
217787_s_at	2.59e-01	2.26e-01	1.212.179
203828_s_at	2.60e-01	2.26e-01	-1.211.467
204110_at	2.60e-01	2.26e-01	1.211.466
204104_at	2.60e-01	2.26e-01	-1.211.286
201105_at	2.60e-01	2.26e-01	1.211.252
221170_at	2.60e-01	2.26e-01	-1.211.229
212792_at	2.60e-01	2.27e-01	1.210.817
212642_s_at	2.60e-01	2.27e-01	1.209.946
212663_at	2.60e-01	2.27e-01	120.957
214053_at	2.61e-01	2.27e-01	-1.209.151
215774_s_at	2.61e-01	2.27e-01	-1.209
206510_at	2.61e-01	2.27e-01	-1.208.909
219415_at	2.61e-01	2.27e-01	120.884
220728_at	2.62e-01	2.28e-01	1.206.814
211239_s_at	2.62e-01	2.28e-01	-1.206.585
210191_s_at	2.62e-01	2.28e-01	1.206.003
208199_s_at	2.62e-01	2.28e-01	-1.205.944
221986_s_at	2.62e-01	2.29e-01	-1.205.745
203003_at	2.62e-01	2.29e-01	-1.205.723
210218_s_at	2.62e-01	2.29e-01	-1.204.834
214923_at	2.62e-01	2.29e-01	-1.204.781
216283_s_at	2.62e-01	2.29e-01	-1.204.748
211289_x_at	2.63e-01	2.29e-01	-1.204.445
221331_x_at	2.63e-01	2.29e-01	-1.204.292
213996_at	2.63e-01	2.29e-01	1.204.267
219429_at	2.63e-01	2.29e-01	-1.203.974
206665_s_at	2.63e-01	2.29e-01	-1.203.832
211106_at	2.63e-01	2.29e-01	-1.203.687
217002_s_at	2.63e-01	2.30e-01	-1.203.098
211422_at	2.63e-01	2.30e-01	-120.241
207792_at	2.63e-01	2.30e-01	-1.202.348
221064_s_at	2.63e-01	2.30e-01	-1.202.256
205457_at	2.63e-01	2.30e-01	1.202.238
222205_x_at	2.64e-01	2.30e-01	-1.201.658
204984_at	2.64e-01	2.30e-01	-1.201.243
209400_at	2.64e-01	2.30e-01	-1.200.946
216865_at	2.64e-01	2.31e-01	-1.200.494
210989_at	2.64e-01	2.31e-01	1.200.227
201674_s_at	2.64e-01	2.31e-01	1.199.512
210160_at	2.65e-01	2.31e-01	-1.198.852

220830_at	2.65e-01	2.31e-01	-1.198.772
211506_s_at	2.65e-01	2.31e-01	1.198.548
206324_s_at	2.65e-01	2.31e-01	1.198.357
201759_at	2.65e-01	2.32e-01	1.198.133
202835_at	2.65e-01	2.32e-01	-1.198.074
214622_at	2.65e-01	2.32e-01	1.198.042
210353_s_at	2.65e-01	2.32e-01	1.197.973
219280_at	2.65e-01	2.32e-01	1.197.635
216686_at	2.65e-01	2.32e-01	-1.197.248
205330_at	2.65e-01	2.32e-01	1.197.011
217164_at	2.65e-01	2.32e-01	1.196.955
212701_at	2.66e-01	2.32e-01	-1.196.796
207029_at	2.66e-01	2.32e-01	-1.196.759
219434_at	2.66e-01	2.32e-01	1.196.731
219246_s_at	2.66e-01	2.32e-01	-1.196.472
40472_at	2.66e-01	2.32e-01	-1.196.256
211523_at	2.66e-01	2.32e-01	-1.196.069
201775_s_at	2.66e-01	2.32e-01	1.196.037
217955_at	2.66e-01	2.32e-01	119.592
215477_at	2.66e-01	2.32e-01	-1.195.879
64418_at	2.66e-01	2.32e-01	-119.586
200862_at	2.66e-01	2.32e-01	119.567
221576_at	2.66e-01	2.33e-01	-119.543
208844_at	2.66e-01	2.33e-01	1.195.395
212729_at	2.66e-01	2.33e-01	1.195.143
214863_at	2.66e-01	2.33e-01	-1.195.137
216893_s_at	2.66e-01	2.33e-01	-1.194.361
213866_at	2.66e-01	2.33e-01	-1.194.219
218380_at	2.67e-01	2.33e-01	-1.193.876
202497_x_at	2.67e-01	2.33e-01	-1.193.631
218969_at	2.67e-01	2.33e-01	-1.193.583
209339_at	2.67e-01	2.33e-01	1.193.399
204650_s_at	2.67e-01	2.33e-01	-1.193.172
206319_s_at	2.67e-01	2.34e-01	1.192.032
214732_at	2.68e-01	2.34e-01	1.191.505
207938_at	2.68e-01	2.34e-01	-1.191.081
208827_at	2.68e-01	2.34e-01	-11.909
206511_s_at	2.68e-01	2.34e-01	-119.061
215469_at	2.68e-01	2.35e-01	-1.190.344
216417_x_at	2.68e-01	2.35e-01	-119.001
201815_s_at	2.68e-01	2.35e-01	1.189.891
204707_s_at	2.68e-01	2.35e-01	-118.988
211914_x_at	2.68e-01	2.35e-01	-1.189.797
AFFX-TrpnX-3_at	2.68e-01	2.35e-01	-118.921

220470_at	2.69e-01	2.35e-01	-1.189.084
209987_s_at	2.69e-01	2.35e-01	-1.189.048
205262_at	2.69e-01	2.35e-01	118.899
216111_x_at	2.69e-01	2.35e-01	1.188.978
216324_at	2.69e-01	2.35e-01	-1.188.757
205057_s_at	2.69e-01	2.35e-01	118.833
214044_at	2.69e-01	2.36e-01	1.186.718
208407_s_at	2.69e-01	2.36e-01	118.668
216636_at	2.70e-01	2.36e-01	-1.186.048
219221_at	2.70e-01	2.36e-01	1.185.458
215056_at	2.70e-01	2.36e-01	1.185.447
200035_at	2.70e-01	2.37e-01	-1.184.666
217200_x_at	2.71e-01	2.37e-01	-1.184.129
220793_at	2.71e-01	2.37e-01	-1.184.046
215358_x_at	2.71e-01	2.37e-01	-1.183.152
206982_at	2.71e-01	2.38e-01	-1.182.523
202196_s_at	2.71e-01	2.38e-01	1.182.329
AFFX-hum_alu_at	2.71e-01	2.38e-01	-1.181.972
221473_x_at	2.71e-01	2.38e-01	1.181.915
216150_at	2.72e-01	2.38e-01	-1.181.516
219815_at	2.72e-01	2.38e-01	-1.180.909
218179_s_at	2.72e-01	2.38e-01	-1.180.787
207726_at	2.72e-01	2.38e-01	-1.180.695
208727_s_at	2.72e-01	2.38e-01	1.180.379
203464_s_at	2.72e-01	2.39e-01	-1.179.899
217625_x_at	2.72e-01	2.39e-01	-1.179.862
219909_at	2.73e-01	2.39e-01	1.179.284
210587_at	2.73e-01	2.39e-01	-1.178.857
218492_s_at	2.73e-01	2.39e-01	1.177.913
221467_at	2.73e-01	2.39e-01	-1.177.899
219611_s_at	2.73e-01	2.40e-01	1.177.523
210641_at	2.74e-01	2.40e-01	-1.176.888
212113_at	2.74e-01	2.40e-01	1.176.455
206951_at	2.74e-01	2.40e-01	-117.617
213791_at	2.75e-01	2.41e-01	1.174.273
213816_s_at	2.75e-01	2.41e-01	1.174.123
210970_s_at	2.75e-01	2.41e-01	117.398
204365_s_at	2.75e-01	2.41e-01	-1.173.682
203299_s_at	2.75e-01	2.41e-01	1.173.309
220055_at	2.75e-01	2.41e-01	-1.173.153
221585_at	2.75e-01	2.42e-01	117.264
207696_at	2.75e-01	2.42e-01	-1.172.572
216560_x_at	2.75e-01	2.42e-01	-1.172.408
200849_s_at	2.76e-01	2.42e-01	-1.171.238

201048_x_at	2.77e-01	2.43e-01	1.169.365
210895_s_at	2.77e-01	2.43e-01	1.169.352
204598_at	2.77e-01	2.43e-01	-1.169.178
219174_at	2.78e-01	2.44e-01	-1.167.602
215016_x_at	2.78e-01	2.44e-01	-1.166.392
218017_s_at	2.78e-01	2.44e-01	1.166.272
201495_x_at	2.79e-01	2.44e-01	-1.165.372
220086_at	2.79e-01	2.45e-01	-1.164.714
217030_at	2.79e-01	2.45e-01	-1.163.983
214275_at	2.80e-01	2.45e-01	-1.162.888
201752_s_at	2.80e-01	2.46e-01	-1.162.855
221069_s_at	2.80e-01	2.46e-01	-1.162.555
221291_at	2.80e-01	2.46e-01	-1.162.262
204636_at	2.80e-01	2.46e-01	1.162.091
222072_at	2.81e-01	2.47e-01	-1.160.292
212398_at	2.81e-01	2.47e-01	1.160.034
207036_x_at	2.81e-01	2.47e-01	-1.159.661
209608_s_at	2.81e-01	2.47e-01	1.159.523
217693_x_at	2.81e-01	2.47e-01	1.159.423
215518_at	2.81e-01	2.47e-01	-1.159.403
212030_at	2.82e-01	2.47e-01	-1.158.648
219447_s_at	2.82e-01	2.47e-01	-1.158.623
222305_at	2.82e-01	2.47e-01	-1.158.397
215594_at	2.82e-01	2.47e-01	-1.157.988
219599_at	2.82e-01	2.48e-01	-115.742
1773_at	2.82e-01	2.48e-01	1.157.086
212734_x_at	2.82e-01	2.48e-01	1.156.875
216967_at	2.82e-01	2.48e-01	-1.156.433
217206_at	2.82e-01	2.48e-01	-115.628
211485_s_at	2.83e-01	2.48e-01	-1.156.196
210194_at	2.83e-01	2.49e-01	-115.482
221243_s_at	2.83e-01	2.49e-01	1.154.626
216852_x_at	2.83e-01	2.49e-01	-115.444
207813_s_at	2.84e-01	2.49e-01	-1.153.311
207521_s_at	2.84e-01	2.49e-01	-1.153.308
209001_s_at	2.84e-01	2.49e-01	1.153.098
210775_x_at	2.84e-01	2.50e-01	-1.153.014
210697_at	2.84e-01	2.50e-01	1.152.884
209719_x_at	2.84e-01	2.50e-01	-1.152.738
212388_at	2.84e-01	2.50e-01	1.152.565
214130_s_at	2.84e-01	2.50e-01	1.152.075
210571_s_at	2.84e-01	2.50e-01	-1.151.969
203993_x_at	2.84e-01	2.50e-01	-1.151.844
203610_s_at	2.85e-01	2.50e-01	1.151.267

210315_at	2.85e-01	2.51e-01	-11.504
215728_s_at	2.85e-01	2.51e-01	1.150.172
201819_at	2.85e-01	2.51e-01	1.149.884
220450_at	2.85e-01	2.51e-01	11.493
219049_at	2.86e-01	2.51e-01	-1.149.162
203081_at	2.86e-01	2.51e-01	-1.149.112
210436_at	2.86e-01	2.51e-01	-1.148.749
221375_at	2.86e-01	2.52e-01	-1.148.094
216921_s_at	2.86e-01	2.52e-01	-1.147.475
210284_s_at	2.87e-01	2.52e-01	-114.646
215808_at	2.87e-01	2.52e-01	-11.463
207580_at	2.87e-01	2.53e-01	-1.145.774
221468_at	2.87e-01	2.53e-01	-114.561
202828_s_at	2.87e-01	2.53e-01	-114.553
221325_at	2.87e-01	2.53e-01	-1.145.468
204284_at	2.87e-01	2.53e-01	1.145.262
221310_at	2.88e-01	2.53e-01	-1.143.814
216987_at	2.88e-01	2.53e-01	-1.143.762
206215_at	2.88e-01	2.53e-01	-114.341
205580_s_at	2.88e-01	2.54e-01	-1.143.217
220197_at	2.88e-01	2.54e-01	-1.143.116
215009_s_at	2.88e-01	2.54e-01	1.143.057
203201_at	2.88e-01	2.54e-01	1.143.023
202399_s_at	2.88e-01	2.54e-01	-1.142.886
214034_at	2.88e-01	2.54e-01	-1.142.792
209273_s_at	2.88e-01	2.54e-01	1.142.788
212395_s_at	2.88e-01	2.54e-01	-1.142.752
204940_at	2.88e-01	2.54e-01	-1.142.544
202401_s_at	2.88e-01	2.54e-01	-1.142.258
217220_at	2.88e-01	2.54e-01	-1.142.212
221461_at	2.88e-01	2.54e-01	-1.142.019
220296_at	2.89e-01	2.54e-01	-1.141.288
220436_at	2.89e-01	2.55e-01	-1.140.343
208164_s_at	2.89e-01	2.55e-01	-1.140.077
217795_s_at	2.89e-01	2.55e-01	114.003
208373_s_at	2.89e-01	2.55e-01	-1.139.737
219344_at	2.90e-01	2.55e-01	-1.139.413
211051_s_at	2.90e-01	2.55e-01	1.139.317
220169_at	2.90e-01	2.55e-01	1.138.894
221999_at	2.90e-01	2.56e-01	-1.138.154
212849_at	2.90e-01	2.56e-01	1.138.142
210229_s_at	2.90e-01	2.56e-01	-1.138.039
201884_at	2.91e-01	2.56e-01	-1.137.016
81811_at	2.91e-01	2.56e-01	1.136.558

204897_at	2.91e-01	2.56e-01	-1.136.458
203083_at	2.91e-01	2.56e-01	1.136.287
220092_s_at	2.91e-01	2.57e-01	-1.135.573
206837_at	2.91e-01	2.57e-01	-1.135.548
212853_at	2.92e-01	2.57e-01	-1.134.968
204525_at	2.92e-01	2.57e-01	1.134.655
210164_at	2.92e-01	2.57e-01	-1.134.596
217436_x_at	2.92e-01	2.57e-01	-1.134.095
216042_at	2.92e-01	2.58e-01	-1.133.747
221300_at	2.92e-01	2.58e-01	1.133.177
202280_at	2.93e-01	2.58e-01	-1.132.296
220652_at	2.93e-01	2.59e-01	-1.131.039
206556_at	2.93e-01	2.59e-01	-1.130.965
207017_at	2.94e-01	2.59e-01	-1.130.085
37547_at	2.94e-01	2.59e-01	1.129.691
209078_s_at	2.94e-01	2.59e-01	-1.129.237
200930_s_at	2.94e-01	2.59e-01	-1.129.218
214797_s_at	2.94e-01	2.59e-01	-1.129.193
211934_x_at	2.94e-01	2.60e-01	-1.128.936
217012_at	2.94e-01	2.60e-01	-1.128.549
204032_at	2.94e-01	2.60e-01	1.128.357
221955_at	2.95e-01	2.60e-01	-1.128.258
202561_at	2.95e-01	2.60e-01	1.127.851
205532_s_at	2.95e-01	2.60e-01	1.126.853
221863_at	2.95e-01	2.60e-01	1.126.724
211513_s_at	2.95e-01	2.60e-01	-1.126.706
215135_at	2.95e-01	2.61e-01	-1.126.482
200985_s_at	2.95e-01	2.61e-01	112.634
222319_at	2.95e-01	2.61e-01	1.126.249
210425_x_at	2.96e-01	2.61e-01	1.124.901
211054_at	2.96e-01	2.61e-01	-1.124.616
219346_at	2.96e-01	2.61e-01	1.124.319
221395_at	2.96e-01	2.62e-01	-1.124.164
205828_at	2.97e-01	2.62e-01	-112.371
216281_at	2.97e-01	2.62e-01	1.123.404
201404_x_at	2.97e-01	2.62e-01	-1.122.533
202531_at	2.97e-01	2.62e-01	-1.122.078
205658_s_at	2.97e-01	2.63e-01	-1.121.759
218312_s_at	2.97e-01	2.63e-01	1.121.706
215896_at	2.98e-01	2.63e-01	-1.121.428
210715_s_at	2.98e-01	2.63e-01	-1.121.191
50376_at	2.98e-01	2.63e-01	-1.120.642
204496_at	2.98e-01	2.63e-01	11.206
218001_at	2.98e-01	2.63e-01	-1.120.216

221755_at	2.98e-01	2.63e-01	1.120.098
202787_s_at	2.98e-01	2.64e-01	-1.119.555
222221_x_at	2.98e-01	2.64e-01	-1.119.395
214597_at	2.99e-01	2.64e-01	-1.118.987
206108_s_at	2.99e-01	2.64e-01	-1.118.917
206543_at	2.99e-01	2.64e-01	-111.861
220724_at	2.99e-01	2.64e-01	-111.776
213209_at	2.99e-01	2.64e-01	-1.117.748
209931_s_at	2.99e-01	2.64e-01	1.117.664
202592_at	2.99e-01	2.64e-01	1.117.603
209551_at	2.99e-01	2.64e-01	1.117.442
200898_s_at	2.99e-01	2.64e-01	-1.117.409
206043_s_at	2.99e-01	2.65e-01	-1.116.989
208143_s_at	3.00e-01	2.65e-01	-1.116.175
220308_at	3.00e-01	2.65e-01	-1.115.542
214154_s_at	3.00e-01	2.65e-01	-1.115.024
204575_s_at	3.01e-01	2.66e-01	-1.114.529
210088_x_at	3.01e-01	2.66e-01	1.114.238
205646_s_at	3.01e-01	2.66e-01	-111.309
211749_s_at	3.01e-01	2.67e-01	1.112.496
217508_s_at	3.02e-01	2.67e-01	-1.112.321
201149_s_at	3.02e-01	2.67e-01	-1.111.524
207677_s_at	3.02e-01	2.67e-01	1.111.518
220330_s_at	3.02e-01	2.67e-01	-1.111.405
210688_s_at	3.02e-01	2.67e-01	-111.135
213145_at	3.02e-01	2.67e-01	1.111.108
210533_at	3.03e-01	2.68e-01	-1.109.743
221236_s_at	3.03e-01	2.68e-01	-1.109.048
205508_at	3.03e-01	2.68e-01	-1.109.032
220428_at	3.03e-01	2.68e-01	-110.809
205518_s_at	3.04e-01	2.69e-01	-1.107.813
220144_s_at	3.04e-01	2.69e-01	1.107.492
207632_at	3.04e-01	2.69e-01	-1.107.235
220001_at	3.04e-01	2.69e-01	-1.106.982
216005_at	3.04e-01	2.69e-01	-1.106.232
222172_at	3.05e-01	2.70e-01	-1.105.563
208797_s_at	3.05e-01	2.70e-01	-1.105.452
214843_s_at	3.05e-01	2.70e-01	-1.105.109
204221_x_at	3.05e-01	2.70e-01	1.104.932
204860_s_at	3.05e-01	2.70e-01	1.104.835
204487_s_at	3.05e-01	2.70e-01	-1.104.453
201029_s_at	3.05e-01	2.70e-01	-1.103.989
221695_s_at	3.06e-01	2.71e-01	-1.102.357
204004_at	3.06e-01	2.71e-01	-1.101.486

200669_s_at	3.07e-01	2.71e-01	1.101.239
210537_s_at	3.07e-01	2.72e-01	1.100.436
218411_s_at	3.07e-01	2.72e-01	-1.100.426
213096_at	3.07e-01	2.72e-01	-1.099.735
218925_s_at	3.07e-01	2.72e-01	-1.099.734
215654_at	3.08e-01	2.72e-01	-1.099.164
213251_at	3.08e-01	2.73e-01	1.098.411
220482_s_at	3.08e-01	2.73e-01	1.098.044
216211_at	3.08e-01	2.73e-01	1.097.816
202909_at	3.08e-01	2.73e-01	1.097.498
216466_at	3.08e-01	2.73e-01	-1.097.256
205647_at	3.08e-01	2.73e-01	1.097.237
215347_at	3.08e-01	2.73e-01	-1.097.127
209750_at	3.09e-01	2.73e-01	-1.096.845
208305_at	3.09e-01	2.73e-01	-109.651
206031_s_at	3.09e-01	2.73e-01	-1.096.505
203128_at	3.09e-01	2.73e-01	10.965
211188_at	3.09e-01	2.73e-01	-1.096.394
218234_at	3.09e-01	2.74e-01	-1.095.558
208871_at	3.09e-01	2.74e-01	-1.095.163
206318_at	3.09e-01	2.74e-01	-1.095.038
216265_x_at	3.10e-01	2.74e-01	-109.451
202920_at	3.10e-01	2.74e-01	-1.094.379
211680_at	3.10e-01	2.74e-01	-1.094.116
208483_x_at	3.10e-01	2.75e-01	-1.093.065
220559_at	3.11e-01	2.75e-01	-109.239
203280_at	3.11e-01	2.76e-01	109.163
212059_s_at	3.11e-01	2.76e-01	1.091.583
207528_s_at	3.11e-01	2.76e-01	1.091.505
31826_at	3.11e-01	2.76e-01	109.108
220634_at	3.12e-01	2.76e-01	1.090.238
204343_at	3.12e-01	2.76e-01	1.090.119
201564_s_at	3.12e-01	2.77e-01	1.089.196
222364_at	3.12e-01	2.77e-01	-1.089.041
205458_at	3.12e-01	2.77e-01	-1.089.039
201910_at	3.12e-01	2.77e-01	-1.088.758
221870_at	3.12e-01	2.77e-01	1.088.717
220004_at	3.12e-01	2.77e-01	1.088.551
210261_at	3.12e-01	2.77e-01	-108.827
212328_at	3.12e-01	2.77e-01	-1.088.226
206555_s_at	3.12e-01	2.77e-01	-1.088.173
213979_s_at	3.12e-01	2.77e-01	108.801
202639_s_at	3.13e-01	2.77e-01	1.087.871
209058_at	3.13e-01	2.77e-01	-108.777

220400_at	3.13e-01	2.77e-01	-1.087.491
220509_at	3.13e-01	2.77e-01	-1.087.331
216590_at	3.13e-01	2.78e-01	-1.086.762
209480_at	3.13e-01	2.78e-01	-1.086.601
216611_s_at	3.13e-01	2.78e-01	-1.086.552
218558_s_at	3.13e-01	2.78e-01	-1.086.386
205869_at	3.13e-01	2.78e-01	-1.086.364
207886_s_at	3.13e-01	2.78e-01	1.086.006
221477_s_at	3.13e-01	2.78e-01	1.085.765
204512_at	3.14e-01	2.78e-01	-1.085.521
201716_at	3.14e-01	2.79e-01	-108.463
218773_s_at	3.14e-01	2.79e-01	-108.462
215846_at	3.14e-01	2.79e-01	-1.084.145
220042_x_at	3.14e-01	2.79e-01	1.083.776
213877_x_at	3.15e-01	2.80e-01	-1.082.733
221248_s_at	3.15e-01	2.80e-01	-1.082.231
203000_at	3.15e-01	2.80e-01	-1.081.348
207225_at	3.16e-01	2.80e-01	-108.088
218811_at	3.16e-01	2.80e-01	-1.080.858
216115_at	3.16e-01	2.81e-01	-1.079.381
205744_at	3.17e-01	2.81e-01	-1.078.392
202742_s_at	3.17e-01	2.82e-01	1.077.666
222274_at	3.18e-01	2.82e-01	-107.704
219384_s_at	3.18e-01	2.82e-01	1.076.753
203546_at	3.18e-01	2.83e-01	-1.075.565
209353_s_at	3.18e-01	2.83e-01	-1.075.483
215431_at	3.18e-01	2.83e-01	1.075.417
217979_at	3.18e-01	2.83e-01	-1.075.013
203759_at	3.18e-01	2.83e-01	-1.075.003
218591_s_at	3.18e-01	2.83e-01	-1.074.936
206208_at	3.19e-01	2.83e-01	-1.074.885
220981_x_at	3.19e-01	2.83e-01	-10.746
215475_at	3.19e-01	2.83e-01	-1.074.148
217553_at	3.19e-01	2.84e-01	1.073.706
217465_at	3.19e-01	2.84e-01	-1.073.636
AFFX-TrpnX-M_at	3.19e-01	2.84e-01	-1.073.493
214030_at	3.19e-01	2.84e-01	1.073.438
202902_s_at	3.19e-01	2.84e-01	-1.073.319
219944_at	3.19e-01	2.84e-01	1.073.082
208835_s_at	3.20e-01	2.84e-01	107.223
206241_at	3.20e-01	2.84e-01	1.071.661
220015_at	3.20e-01	2.84e-01	-1.071.623
200659_s_at	3.20e-01	2.85e-01	-1.071.122
41160_at	3.21e-01	2.85e-01	-1.070.385

215296_at	3.21e-01	2.85e-01	-1.069.456
219660_s_at	3.21e-01	2.86e-01	-1.069.145
212939_at	3.21e-01	2.86e-01	1.068.994
212927_at	3.22e-01	2.86e-01	1.068.241
216669_at	3.22e-01	2.86e-01	-106.784
217031_at	3.22e-01	2.86e-01	-1.067.527
217457_s_at	3.22e-01	2.86e-01	1.067.123
206411_s_at	3.22e-01	2.87e-01	1.067.024
211076_x_at	3.22e-01	2.87e-01	1.066.827
47773_at	3.22e-01	2.87e-01	1.066.351
211282_x_at	3.23e-01	2.87e-01	-1.065.943
204637_at	3.23e-01	2.87e-01	1.065.404
204930_s_at	3.23e-01	2.88e-01	-1.064.526
204131_s_at	3.23e-01	2.88e-01	-1.064.406
201631_s_at	3.24e-01	2.88e-01	1.064.088
216130_at	3.24e-01	2.88e-01	-1.063.843
202402_s_at	3.24e-01	2.88e-01	-1.063.784
215210_s_at	3.24e-01	2.88e-01	-1.063.338
222324_at	3.24e-01	2.88e-01	-1.063.158
209824_s_at	3.24e-01	2.88e-01	1.062.884
204996_s_at	3.25e-01	2.89e-01	-1.061.535
214293_at	3.25e-01	2.89e-01	-1.060.903
216959_x_at	3.25e-01	2.89e-01	1.060.568
204268_at	3.25e-01	2.90e-01	1.060.406
203491_s_at	3.25e-01	2.90e-01	1.059.945
216968_at	3.26e-01	2.90e-01	1.059.158
211540_s_at	3.26e-01	2.90e-01	-1.059.081
219476_at	3.26e-01	2.90e-01	1.058.704
205329_s_at	3.27e-01	2.91e-01	105.747
202032_s_at	3.27e-01	2.91e-01	1.057.426
220798_x_at	3.27e-01	2.91e-01	-105.709
202528_at	3.27e-01	2.91e-01	1.056.836
210605_s_at	3.27e-01	2.91e-01	-1.056.711
221670_s_at	3.27e-01	2.91e-01	1.056.383
203634_s_at	3.27e-01	2.91e-01	105.624
215542_at	3.27e-01	2.92e-01	1.056.002
218466_at	3.27e-01	2.92e-01	1.055.894
203136_at	3.27e-01	2.92e-01	1.055.587
201325_s_at	3.28e-01	2.92e-01	-1.055.299
206502_s_at	3.28e-01	2.92e-01	-1.055.298
222351_at	3.28e-01	2.92e-01	-1.055.105
202547_s_at	3.28e-01	2.92e-01	1.055.014
216630_at	3.28e-01	2.92e-01	-1.054.868
201782_s_at	3.28e-01	2.92e-01	-1.054.603

206088_at	3.28e-01	2.92e-01	1.054.456
214170_x_at	3.28e-01	2.93e-01	-1.053.864
201081_s_at	3.28e-01	2.93e-01	-1.053.593
208281_x_at	3.29e-01	2.93e-01	1.052.524
216297_at	3.29e-01	2.93e-01	-1.052.406
209557_s_at	3.29e-01	2.93e-01	-105.231
210840_s_at	3.30e-01	2.94e-01	-1.051.217
212068_s_at	3.30e-01	2.94e-01	1.050.276
202479_s_at	3.30e-01	2.94e-01	1.050.073
201666_at	3.31e-01	2.95e-01	-1.048.963
219270_at	3.31e-01	2.95e-01	-1.048.715
209682_at	3.31e-01	2.95e-01	1.047.792
216610_at	3.31e-01	2.96e-01	-104.732
203293_s_at	3.32e-01	2.96e-01	-104.632
217585_at	3.32e-01	2.96e-01	1.046.005
219816_s_at	3.32e-01	2.96e-01	-1.045.862
205587_at	3.33e-01	2.97e-01	-1.044.834
219735_s_at	3.33e-01	2.97e-01	-1.044.743
220568_at	3.33e-01	2.97e-01	-1.044.048
206675_s_at	3.33e-01	2.97e-01	-1.043.931
205193_at	3.33e-01	2.97e-01	1.043.872
210287_s_at	3.33e-01	2.97e-01	-1.043.651
219503_s_at	3.33e-01	2.97e-01	-1.043.567
218592_s_at	3.33e-01	2.97e-01	-1.043.032
210458_s_at	3.34e-01	2.98e-01	104.277
202457_s_at	3.34e-01	2.98e-01	-1.042.615
201133_s_at	3.34e-01	2.98e-01	-1.042.364
219678_x_at	3.34e-01	2.98e-01	1.041.114
209717_at	3.34e-01	2.98e-01	-1.040.907
214563_at	3.35e-01	2.99e-01	-1.040.143
217415_at	3.35e-01	2.99e-01	1.039.924
206027_at	3.35e-01	2.99e-01	-1.039.527
216037_x_at	3.35e-01	2.99e-01	-1.039.506
214000_s_at	3.35e-01	2.99e-01	-1.038.782
219593_at	3.36e-01	3.00e-01	-1.038.126
219506_at	3.36e-01	3.00e-01	-1.038.068
205495_s_at	3.36e-01	3.00e-01	-1.037.848
222048_at	3.36e-01	3.00e-01	-1.037.625
204116_at	3.36e-01	3.00e-01	-1.036.872
217875_s_at	3.37e-01	3.01e-01	1.036.256
202454_s_at	3.38e-01	3.02e-01	1.034.297
204610_s_at	3.38e-01	3.02e-01	1.033.705
201692_at	3.38e-01	3.02e-01	1.033.215
218669_at	3.38e-01	3.02e-01	1.033.051

201167_x_at	3.39e-01	3.02e-01	1.032.573
203748_x_at	3.39e-01	3.02e-01	-103.241
201552_at	3.39e-01	3.03e-01	1.032.131
210281_s_at	3.39e-01	3.03e-01	-1.032.094
220014_at	3.39e-01	3.03e-01	103.197
213943_at	3.39e-01	3.03e-01	-1.031.362
212191_x_at	3.39e-01	3.03e-01	1.031.099
210711_at	3.39e-01	3.03e-01	-1.031.036
221444_at	3.39e-01	3.03e-01	-1.030.994
212369_at	3.39e-01	3.03e-01	-1.030.988
221214_s_at	3.39e-01	3.03e-01	-1.030.966
214459_x_at	3.40e-01	3.03e-01	1.030.391
203703_s_at	3.41e-01	3.04e-01	-1.028.311
215310_at	3.41e-01	3.04e-01	-1.028.153
214897_at	3.41e-01	3.05e-01	-1.027.741
204093_at	3.41e-01	3.05e-01	1.027.686
200668_s_at	3.41e-01	3.05e-01	-1.027.488
45714_at	3.41e-01	3.05e-01	10.273
211343_s_at	3.41e-01	3.05e-01	-1.026.847
203057_s_at	3.41e-01	3.05e-01	-1.026.768
214723_x_at	3.41e-01	3.05e-01	1.026.569
207147_at	3.41e-01	3.05e-01	102.649
207754_at	3.42e-01	3.05e-01	-1.026.137
217359_s_at	3.42e-01	3.06e-01	-1.024.334
205009_at	3.43e-01	3.07e-01	1.022.971
216845_x_at	3.43e-01	3.07e-01	1.022.941
201925_s_at	3.43e-01	3.07e-01	-1.022.909
207437_at	3.43e-01	3.07e-01	1.022.834
206149_at	3.43e-01	3.07e-01	-1.022.769
218970_s_at	3.43e-01	3.07e-01	-1.022.659
204797_s_at	3.44e-01	3.07e-01	-1.022.062
220214_at	3.44e-01	3.07e-01	-1.021.962
206504_at	3.44e-01	3.07e-01	-1.021.654
216534_at	3.44e-01	3.08e-01	-1.021.639
216286_at	3.44e-01	3.08e-01	1.021.483
210214_s_at	3.44e-01	3.08e-01	-1.021.447
209893_s_at	3.44e-01	3.08e-01	1.021.282
204071_s_at	3.44e-01	3.08e-01	-1.021.182
206585_at	3.45e-01	3.08e-01	-1.019.756
34726_at	3.45e-01	3.08e-01	1.019.709
202492_at	3.45e-01	3.09e-01	-1.019.423
202463_s_at	3.45e-01	3.09e-01	1.019.231
219604_s_at	3.45e-01	3.09e-01	101.885
201650_at	3.45e-01	3.09e-01	1.018.605

210727_at	3.46e-01	3.10e-01	1.016.764
210375_at	3.46e-01	3.10e-01	-1.016.346
217185_s_at	3.47e-01	3.10e-01	1.015.695
210812_at	3.47e-01	3.10e-01	-1.015.479
219722_s_at	3.47e-01	3.11e-01	101.452
206533_at	3.47e-01	3.11e-01	1.014.257
221319_at	3.47e-01	3.11e-01	-1.014.103
209205_s_at	3.48e-01	3.11e-01	-1.013.876
208194_s_at	3.48e-01	3.11e-01	-1.013.655
209216_at	3.48e-01	3.11e-01	-1.013.287
41657_at	3.48e-01	3.12e-01	1.012.497
206774_at	3.48e-01	3.12e-01	-1.012.075
220889_s_at	3.48e-01	3.12e-01	1.012.065
217267_s_at	3.49e-01	3.12e-01	-1.011.468
209588_at	3.49e-01	3.12e-01	-1.011.156
216301_at	3.49e-01	3.13e-01	-1.010.779
207103_at	3.49e-01	3.13e-01	-1.010.459
217247_at	3.50e-01	3.13e-01	-1.009.691
207183_at	3.50e-01	3.13e-01	-1.009.508
203599_s_at	3.50e-01	3.13e-01	-1.009.431
214464_at	3.50e-01	3.13e-01	-1.009.274
205679_x_at	3.50e-01	3.14e-01	-1.008.738
215355_at	3.50e-01	3.14e-01	-1.008.685
205244_s_at	3.50e-01	3.14e-01	100.867
205197_s_at	3.50e-01	3.14e-01	-1.008.608
206141_at	3.50e-01	3.14e-01	-1.008.467
AFFX-M27830_M_at	3.50e-01	3.14e-01	-1.008.169
215680_at	3.50e-01	3.14e-01	-1.008.166
218259_at	3.50e-01	3.14e-01	1.008.028
221597_s_at	3.51e-01	3.14e-01	-1.007.525
205093_at	3.51e-01	3.14e-01	1.007.334
34478_at	3.51e-01	3.14e-01	-1.007.061
211685_s_at	3.51e-01	3.15e-01	-1.006.506
206026_s_at	3.51e-01	3.15e-01	1.006.463
220503_at	3.51e-01	3.15e-01	-1.006.376
205493_s_at	3.51e-01	3.15e-01	-1.006.336
208623_s_at	3.51e-01	3.15e-01	-1.006.168
211618_s_at	3.51e-01	3.15e-01	1.005.616
210138_at	3.52e-01	3.15e-01	-1.005.415
210286_s_at	3.52e-01	3.15e-01	1.005.397
207208_at	3.52e-01	3.16e-01	-1.004.395
215749_s_at	3.53e-01	3.16e-01	1.003.461
207588_at	3.53e-01	3.16e-01	-1.003.316
207296_at	3.53e-01	3.17e-01	100.201

206046_at	3.53e-01	3.17e-01	1.001.747
220231_at	3.53e-01	3.17e-01	-1.001.694
215801_at	3.53e-01	3.17e-01	-1.001.576
200733_s_at	3.54e-01	3.18e-01	-1.000.666
207472_at	3.54e-01	3.18e-01	-100.057
204330_s_at	3.54e-01	3.18e-01	-100.055
204969_s_at	3.54e-01	3.18e-01	100.052
217602_at	3.54e-01	3.18e-01	-1.000.515
201115_at	3.54e-01	3.18e-01	1.000.514
211724_x_at	3.54e-01	3.18e-01	0.999967
217615_at	3.54e-01	3.18e-01	-0.999349
207935_s_at	3.55e-01	3.18e-01	-0.999306
218182_s_at	3.55e-01	3.18e-01	0.998863
201721_s_at	3.55e-01	3.18e-01	0.99867
202549_at	3.56e-01	3.19e-01	0.996815
219218_at	3.56e-01	3.20e-01	0.996546
221879_at	3.56e-01	3.20e-01	0.995443
204471_at	3.57e-01	3.20e-01	-0.995088
214188_at	3.57e-01	3.20e-01	0.9948
221938_x_at	3.57e-01	3.21e-01	-0.994537
210176_at	3.57e-01	3.21e-01	-0.994478
211528_x_at	3.57e-01	3.21e-01	0.993973
210130_s_at	3.57e-01	3.21e-01	0.993876
211562_s_at	3.57e-01	3.21e-01	0.993675
218084_x_at	3.57e-01	3.21e-01	-0.99357
206248_at	3.57e-01	3.21e-01	0.993448
218891_at	3.58e-01	3.21e-01	0.993122
221182_at	3.58e-01	3.21e-01	-0.992607
203815_at	3.58e-01	3.22e-01	-0.992255
217252_at	3.58e-01	3.22e-01	-0.992147
207743_at	3.58e-01	3.22e-01	-0.992085
209111_at	3.58e-01	3.22e-01	0.991943
216972_at	3.58e-01	3.22e-01	-0.991499
206712_at	3.58e-01	3.22e-01	0.991297
221394_at	3.58e-01	3.22e-01	-0.99127
222280_at	3.59e-01	3.22e-01	-0.990678
202849_x_at	3.59e-01	3.22e-01	0.99044
203461_at	3.59e-01	3.23e-01	-0.990406
213413_at	3.59e-01	3.23e-01	-0.990341
222085_at	3.59e-01	3.23e-01	-0.990026
214826_at	3.59e-01	3.23e-01	-0.989546
212534_at	3.60e-01	3.23e-01	0.988509
206365_at	3.60e-01	3.24e-01	-0.988322
216519_s_at	3.60e-01	3.24e-01	0.988259

205919_at	3.60e-01	3.24e-01	-0.987235
221418_s_at	3.61e-01	3.24e-01	-0.987111
205727_at	3.61e-01	3.24e-01	0.986789
204410_at	3.61e-01	3.24e-01	-0.986476
222333_at	3.61e-01	3.25e-01	-0.985612
214046_at	3.62e-01	3.26e-01	-0.984061
209590_at	3.62e-01	3.26e-01	-0.983916
207281_x_at	3.63e-01	3.26e-01	0.983162
202682_s_at	3.63e-01	3.26e-01	0.983139
AFFX-HUMRGE/M100	3.63e-01	3.26e-01	0.982944
215842_s_at	3.63e-01	3.26e-01	0.982646
214225_at	3.63e-01	3.26e-01	-0.982275
219993_at	3.63e-01	3.27e-01	0.981943
220669_at	3.63e-01	3.27e-01	-0.981801
221035_s_at	3.63e-01	3.27e-01	0.98144
207640_x_at	3.63e-01	3.27e-01	-0.981253
204942_s_at	3.63e-01	3.27e-01	-0.981204
211899_s_at	3.64e-01	3.27e-01	0.980728
213559_s_at	3.64e-01	3.27e-01	-0.980651
209169_at	3.64e-01	3.27e-01	-0.980467
214803_at	3.64e-01	3.27e-01	-0.980383
201284_s_at	3.64e-01	3.27e-01	0.980268
206326_at	3.64e-01	3.28e-01	-0.980181
219019_at	3.65e-01	3.28e-01	0.978815
205893_at	3.65e-01	3.28e-01	-0.978736
201647_s_at	3.65e-01	3.28e-01	-0.978688
204415_at	3.65e-01	3.28e-01	0.978458
205787_x_at	3.65e-01	3.29e-01	0.97808
221845_s_at	3.65e-01	3.29e-01	-0.977719
220551_at	3.65e-01	3.29e-01	-0.97763
204425_at	3.65e-01	3.29e-01	0.977257
201753_s_at	3.66e-01	3.29e-01	-0.976723
215547_at	3.66e-01	3.29e-01	-0.976485
202292_x_at	3.66e-01	3.29e-01	0.976283
212550_at	3.66e-01	3.30e-01	0.97527
215053_at	3.67e-01	3.30e-01	0.974474
213959_s_at	3.67e-01	3.31e-01	0.973904
200730_s_at	3.67e-01	3.31e-01	-0.973833
201185_at	3.67e-01	3.31e-01	0.973646
219780_at	3.68e-01	3.31e-01	-0.972975
202900_s_at	3.68e-01	3.32e-01	0.971966
219769_at	3.68e-01	3.32e-01	-0.971385
217495_x_at	3.69e-01	3.32e-01	-0.970903
220487_at	3.69e-01	3.32e-01	-0.970874

202367_at	3.69e-01	3.32e-01	0.970653
220591_s_at	3.69e-01	3.33e-01	0.97
203660_s_at	3.69e-01	3.33e-01	-0.969983
213650_at	3.70e-01	3.33e-01	-0.968965
209850_s_at	3.70e-01	3.33e-01	-0.968671
205434_s_at	3.70e-01	3.34e-01	0.968097
202656_s_at	3.70e-01	3.34e-01	0.968054
214115_at	3.70e-01	3.34e-01	-0.967633
203597_s_at	3.71e-01	3.34e-01	0.966716
215707_s_at	3.71e-01	3.35e-01	-0.96541
217528_at	3.72e-01	3.35e-01	-0.964913
202320_at	3.72e-01	3.35e-01	-0.964436
216360_x_at	3.72e-01	3.35e-01	-0.964404
205035_at	3.72e-01	3.36e-01	0.963496
217086_at	3.73e-01	3.36e-01	-0.963038
57739_at	3.73e-01	3.36e-01	0.962202
204851_s_at	3.73e-01	3.36e-01	-0.962163
213211_s_at	3.73e-01	3.37e-01	-0.961743
204001_at	3.74e-01	3.37e-01	0.960356
201034_at	3.74e-01	3.37e-01	0.960336
210121_at	3.74e-01	3.37e-01	0.960258
211791_s_at	3.74e-01	3.38e-01	-0.960089
214518_at	3.74e-01	3.38e-01	-0.959903
221665_s_at	3.74e-01	3.38e-01	-0.95958
211975_at	3.75e-01	3.38e-01	-0.958491
220145_at	3.75e-01	3.39e-01	-0.95809
221787_at	3.75e-01	3.39e-01	-0.957759
203445_s_at	3.76e-01	3.39e-01	0.957452
206859_s_at	3.76e-01	3.39e-01	0.956969
218840_s_at	3.76e-01	3.40e-01	0.95608
206560_s_at	3.76e-01	3.40e-01	-0.95595
206040_s_at	3.77e-01	3.40e-01	-0.955523
213615_at	3.77e-01	3.40e-01	-0.955457
218913_s_at	3.78e-01	3.41e-01	0.953501
204682_at	3.78e-01	3.41e-01	0.953394
205349_at	3.78e-01	3.41e-01	-0.953088
208600_s_at	3.78e-01	3.41e-01	0.953027
212079_s_at	3.78e-01	3.41e-01	0.953011
213764_s_at	3.78e-01	3.41e-01	-0.952829
217661_x_at	3.78e-01	3.41e-01	-0.952572
210575_at	3.78e-01	3.42e-01	0.952162
201338_x_at	3.78e-01	3.42e-01	-0.951815
215605_at	3.79e-01	3.42e-01	-0.95122
215437_x_at	3.79e-01	3.42e-01	-0.950874

215331_at	3.79e-01	3.42e-01	-0.950443
207502_at	3.79e-01	3.42e-01	0.950251
221270_s_at	3.79e-01	3.43e-01	-0.950135
210951_x_at	3.80e-01	3.43e-01	0.949306
212761_at	3.80e-01	3.43e-01	-0.949088
207176_s_at	3.80e-01	3.43e-01	-0.94902
212854_x_at	3.80e-01	3.43e-01	0.948543
206084_at	3.80e-01	3.44e-01	-0.948099
204840_s_at	3.80e-01	3.44e-01	0.94798
210265_x_at	3.80e-01	3.44e-01	-0.947975
217646_at	3.80e-01	3.44e-01	-0.94792
215279_at	3.80e-01	3.44e-01	0.947828
206546_at	3.81e-01	3.44e-01	-0.947575
215965_at	3.81e-01	3.44e-01	-0.94743
213182_x_at	3.81e-01	3.44e-01	0.947088
220040_x_at	3.81e-01	3.44e-01	-0.946728
205985_x_at	3.81e-01	3.44e-01	-0.946448
212647_at	3.81e-01	3.44e-01	0.946349
220919_s_at	3.81e-01	3.45e-01	0.946082
215079_at	3.82e-01	3.45e-01	-0.944903
210462_at	3.82e-01	3.46e-01	0.943928
203705_s_at	3.83e-01	3.46e-01	0.943321
212901_s_at	3.83e-01	3.46e-01	-0.943289
201923_at	3.83e-01	3.46e-01	-0.943251
217072_at	3.83e-01	3.46e-01	-0.943141
219475_at	3.83e-01	3.46e-01	0.943024
209868_s_at	3.83e-01	3.46e-01	0.942934
202443_x_at	3.84e-01	3.47e-01	-0.941798
212109_at	3.84e-01	3.47e-01	0.941643
218664_at	3.84e-01	3.47e-01	-0.941178
209409_at	3.84e-01	3.47e-01	-0.941098
217213_at	3.84e-01	3.48e-01	-0.94024
216134_at	3.85e-01	3.49e-01	-0.938459
205635_at	3.86e-01	3.49e-01	0.937941
214316_x_at	3.86e-01	3.49e-01	0.937888
210461_s_at	3.86e-01	3.49e-01	0.937204
216638_s_at	3.86e-01	3.49e-01	0.936722
210625_s_at	3.86e-01	3.50e-01	-0.936362
216999_at	3.87e-01	3.50e-01	-0.936084
204421_s_at	3.87e-01	3.50e-01	-0.935716
204232_at	3.87e-01	3.50e-01	-0.935678
200760_s_at	3.87e-01	3.50e-01	0.934982
206937_at	3.87e-01	3.50e-01	-0.934976
219946_x_at	3.87e-01	3.50e-01	-0.934602

210550_s_at	3.88e-01	3.51e-01	0.933813
206086_x_at	3.88e-01	3.51e-01	-0.933327
207277_at	3.89e-01	3.52e-01	-0.932343
207154_at	3.89e-01	3.52e-01	-0.93136
222179_at	3.89e-01	3.53e-01	-0.930618
209755_at	3.90e-01	3.53e-01	0.930112
217620_s_at	3.90e-01	3.53e-01	0.930105
219682_s_at	3.90e-01	3.53e-01	-0.929917
220690_s_at	3.90e-01	3.53e-01	-0.928958
216425_at	3.91e-01	3.54e-01	-0.928503
209325_s_at	3.91e-01	3.54e-01	-0.928425
207582_at	3.91e-01	3.54e-01	-0.928293
221778_at	3.91e-01	3.54e-01	0.928026
221689_s_at	3.91e-01	3.54e-01	0.927117
221937_at	3.91e-01	3.54e-01	-0.92689
201336_at	3.92e-01	3.55e-01	0.926461
44790_s_at	3.92e-01	3.55e-01	-0.926037
216489_at	3.92e-01	3.55e-01	0.925971
220606_s_at	3.92e-01	3.55e-01	0.925562
213031_s_at	3.93e-01	3.56e-01	0.924713
212263_at	3.93e-01	3.56e-01	0.924637
211544_s_at	3.93e-01	3.56e-01	0.924378
203388_at	3.93e-01	3.56e-01	-0.923719
205094_at	3.93e-01	3.56e-01	-0.923113
206540_at	3.94e-01	3.57e-01	0.922125
204035_at	3.94e-01	3.57e-01	0.922104
219183_s_at	3.94e-01	3.57e-01	-0.922016
209102_s_at	3.94e-01	3.57e-01	-0.921809
214138_at	3.94e-01	3.57e-01	0.921378
36004_at	3.94e-01	3.57e-01	0.921179
206516_at	3.95e-01	3.58e-01	0.920923
212489_at	3.95e-01	3.58e-01	0.920677
206077_at	3.95e-01	3.58e-01	0.920616
210018_x_at	3.95e-01	3.58e-01	-0.920528
207142_at	3.95e-01	3.58e-01	-0.920456
208841_s_at	3.95e-01	3.58e-01	0.920444
204443_at	3.95e-01	3.58e-01	-0.919416
221733_s_at	3.96e-01	3.59e-01	-0.918025
212784_at	3.96e-01	3.59e-01	0.917892
211625_s_at	3.96e-01	3.59e-01	0.917537
201283_s_at	3.96e-01	3.59e-01	0.917396
220721_at	3.97e-01	3.60e-01	-0.916391
220213_at	3.97e-01	3.60e-01	0.915436
215748_at	3.98e-01	3.61e-01	-0.915032

211227_s_at	3.98e-01	3.61e-01	-0.914788
216469_at	3.98e-01	3.61e-01	-0.914771
214562_at	3.98e-01	3.61e-01	-0.914123
216912_at	3.99e-01	3.62e-01	-0.912018
208434_at	3.99e-01	3.62e-01	-0.911745
203880_at	4.00e-01	3.63e-01	-0.911226
205132_at	4.00e-01	3.63e-01	-0.910472
209878_s_at	4.00e-01	3.63e-01	0.910271
207779_at	4.00e-01	3.63e-01	0.910207
211776_s_at	4.00e-01	3.63e-01	-0.909937
206885_x_at	4.01e-01	3.64e-01	-0.909175
214717_at	4.01e-01	3.64e-01	0.90889
215297_at	4.01e-01	3.64e-01	-0.90846
204060_s_at	4.01e-01	3.64e-01	-0.908072
207170_s_at	4.02e-01	3.64e-01	0.90791
213868_s_at	4.02e-01	3.65e-01	-0.907474
40562_at	4.02e-01	3.65e-01	0.906812
217178_at	4.02e-01	3.65e-01	-0.906536
214516_at	4.02e-01	3.65e-01	0.906161
213472_at	4.02e-01	3.65e-01	-0.906107
205861_at	4.03e-01	3.66e-01	-0.905419
216731_s_at	4.03e-01	3.66e-01	-0.905215
212106_at	4.04e-01	3.66e-01	0.904141
216000_at	4.04e-01	3.67e-01	-0.903655
221625_at	4.04e-01	3.67e-01	0.903541
220262_s_at	4.04e-01	3.67e-01	-0.903405
214095_at	4.04e-01	3.67e-01	-0.903208
221832_s_at	4.04e-01	3.67e-01	0.903194
213419_at	4.04e-01	3.67e-01	-0.902951
201085_s_at	4.04e-01	3.67e-01	-0.902322
207439_s_at	4.05e-01	3.68e-01	0.901864
221603_at	4.05e-01	3.68e-01	0.90164
213487_at	4.05e-01	3.68e-01	-0.90153
216315_x_at	4.05e-01	3.68e-01	0.901368
203383_s_at	4.05e-01	3.68e-01	0.901195
203902_at	4.05e-01	3.68e-01	-0.900884
211468_s_at	4.05e-01	3.68e-01	0.900396
203699_s_at	4.06e-01	3.69e-01	0.899786
207755_at	4.06e-01	3.69e-01	-0.899497
206619_at	4.06e-01	3.69e-01	0.899306
213455_at	4.06e-01	3.69e-01	0.899083
215249_at	4.06e-01	3.69e-01	-0.899057
219956_at	4.06e-01	3.69e-01	-0.898547
218093_s_at	4.07e-01	3.69e-01	0.898361

214195_at	4.07e-01	3.70e-01	-0.897922
220961_s_at	4.07e-01	3.70e-01	-0.897762
218173_s_at	4.08e-01	3.71e-01	0.895944
218630_at	4.08e-01	3.71e-01	0.895819
212768_s_at	4.08e-01	3.71e-01	0.895608
204020_at	4.08e-01	3.71e-01	-0.895416
204645_at	4.08e-01	3.71e-01	-0.89496
208065_at	4.09e-01	3.72e-01	-0.893884
213721_at	4.10e-01	3.72e-01	-0.892803
215648_at	4.10e-01	3.73e-01	0.892619
215635_at	4.10e-01	3.73e-01	-0.89154
218908_at	4.11e-01	3.73e-01	0.890992
216839_at	4.11e-01	3.73e-01	-0.890925
216595_at	4.11e-01	3.74e-01	-0.890671
210032_s_at	4.11e-01	3.74e-01	-0.890282
219629_at	4.11e-01	3.74e-01	-0.890171
214179_s_at	4.11e-01	3.74e-01	0.890009
208027_s_at	4.12e-01	3.75e-01	-0.887764
214245_at	4.12e-01	3.75e-01	0.88767
220338_at	4.13e-01	3.75e-01	0.887473
221519_at	4.13e-01	3.75e-01	0.887136
211527_x_at	4.13e-01	3.76e-01	-0.887032
219106_s_at	4.13e-01	3.76e-01	-0.886503
207928_s_at	4.13e-01	3.76e-01	-0.886411
219973_at	4.13e-01	3.76e-01	-0.886284
204990_s_at	4.13e-01	3.76e-01	-0.886142
209228_x_at	4.13e-01	3.76e-01	0.885825
208063_s_at	4.14e-01	3.76e-01	-0.885526
212425_at	4.14e-01	3.76e-01	-0.885292
200859_x_at	4.14e-01	3.77e-01	-0.885117
206864_s_at	4.14e-01	3.77e-01	-0.884726
210277_at	4.14e-01	3.77e-01	-0.884324
210491_at	4.14e-01	3.77e-01	-0.884308
208482_at	4.14e-01	3.77e-01	0.884012
215854_at	4.15e-01	3.78e-01	0.8833
209283_at	4.15e-01	3.78e-01	-0.882916
206400_at	4.15e-01	3.78e-01	0.882907
220135_s_at	4.15e-01	3.78e-01	0.882345
203741_s_at	4.15e-01	3.78e-01	-0.882073
213809_x_at	4.16e-01	3.78e-01	-0.881748
219941_at	4.16e-01	3.79e-01	-0.881404
217061_s_at	4.16e-01	3.79e-01	-0.880393
216820_at	4.16e-01	3.79e-01	-0.880238
219163_at	4.16e-01	3.79e-01	0.880232

209815_at	4.17e-01	3.80e-01	-0.87848
214740_at	4.18e-01	3.81e-01	0.877715
203140_at	4.18e-01	3.81e-01	-0.876733
201212_at	4.18e-01	3.81e-01	-0.87661
205239_at	4.19e-01	3.81e-01	-0.876323
221448_s_at	4.19e-01	3.82e-01	-0.875926
214713_at	4.19e-01	3.82e-01	0.87581
206773_at	4.19e-01	3.82e-01	0.875444
216810_at	4.19e-01	3.82e-01	-0.874606
208798_x_at	4.20e-01	3.83e-01	-0.87405
219461_at	4.20e-01	3.83e-01	0.873486
217527_s_at	4.20e-01	3.83e-01	0.873444
207989_at	4.20e-01	3.83e-01	-0.873324
212965_at	4.20e-01	3.83e-01	0.873237
202377_at	4.20e-01	3.83e-01	0.873173
213106_at	4.20e-01	3.83e-01	0.872798
213703_at	4.21e-01	3.84e-01	0.872113
217392_at	4.21e-01	3.84e-01	-0.871729
203028_s_at	4.21e-01	3.84e-01	-0.871671
206063_x_at	4.21e-01	3.84e-01	0.871605
213481_at	4.22e-01	3.84e-01	0.870675
200697_at	4.22e-01	3.85e-01	0.870156
220095_at	4.22e-01	3.85e-01	0.869868
204665_at	4.22e-01	3.85e-01	-0.869186
202866_at	4.22e-01	3.85e-01	-0.869182
221943_x_at	4.23e-01	3.86e-01	0.868488
216666_at	4.23e-01	3.86e-01	-0.868261
218555_at	4.23e-01	3.86e-01	0.867757
221803_s_at	4.23e-01	3.86e-01	-0.867688
221846_s_at	4.24e-01	3.87e-01	0.866689
219963_at	4.24e-01	3.87e-01	-0.866151
204784_s_at	4.24e-01	3.87e-01	0.86608
220771_at	4.24e-01	3.87e-01	0.86555
221349_at	4.24e-01	3.87e-01	-0.865474
204884_s_at	4.25e-01	3.87e-01	-0.865175
201747_s_at	4.25e-01	3.87e-01	0.865085
220021_at	4.25e-01	3.88e-01	0.864749
205408_at	4.25e-01	3.88e-01	0.864717
208245_at	4.25e-01	3.88e-01	-0.864526
221234_s_at	4.25e-01	3.88e-01	-0.864367
216706_x_at	4.26e-01	3.88e-01	-0.863381
202403_s_at	4.26e-01	3.88e-01	-0.863265
222013_x_at	4.26e-01	3.89e-01	0.862606
214614_at	4.27e-01	3.89e-01	0.861676

215116_s_at	4.27e-01	3.89e-01	-0.861582
216506_x_at	4.27e-01	3.90e-01	-0.861068
206163_at	4.27e-01	3.90e-01	-0.860977
203156_at	4.27e-01	3.90e-01	-0.860754
210918_at	4.27e-01	3.90e-01	-0.860624
208666_s_at	4.27e-01	3.90e-01	0.860338
215111_s_at	4.27e-01	3.90e-01	0.860316
219204_s_at	4.27e-01	3.90e-01	0.860073
217409_at	4.27e-01	3.90e-01	-0.859871
203152_at	4.27e-01	3.90e-01	-0.859735
217208_s_at	4.28e-01	3.91e-01	-0.859056
203966_s_at	4.29e-01	3.91e-01	0.857779
216802_at	4.29e-01	3.92e-01	-0.856854
201560_at	4.29e-01	3.92e-01	0.85632
213332_at	4.29e-01	3.92e-01	0.8563
206942_s_at	4.30e-01	3.93e-01	-0.855262
210804_x_at	4.30e-01	3.93e-01	0.854855
212033_at	4.30e-01	3.93e-01	-0.85463
215234_at	4.31e-01	3.93e-01	0.854263
221114_at	4.31e-01	3.94e-01	-0.853376
215212_at	4.31e-01	3.94e-01	-0.853304
215195_at	4.32e-01	3.95e-01	0.852143
209090_s_at	4.32e-01	3.95e-01	0.851946
201333_s_at	4.32e-01	3.95e-01	0.85172
219522_at	4.32e-01	3.95e-01	0.851377
203797_at	4.32e-01	3.95e-01	-0.851286
207432_at	4.32e-01	3.95e-01	-0.851111
203125_x_at	4.32e-01	3.95e-01	0.851008
212384_at	4.32e-01	3.95e-01	-0.850866
217420_s_at	4.33e-01	3.95e-01	-0.850608
214583_at	4.33e-01	3.96e-01	-0.850099
204915_s_at	4.33e-01	3.96e-01	0.849699
219558_at	4.34e-01	3.96e-01	0.848899
205965_at	4.34e-01	3.97e-01	-0.848315
205087_at	4.34e-01	3.97e-01	0.847943
207781_s_at	4.34e-01	3.97e-01	0.847638
205841_at	4.34e-01	3.97e-01	-0.847632
208212_s_at	4.34e-01	3.97e-01	-0.847451
207383_s_at	4.34e-01	3.97e-01	-0.847141
215282_at	4.35e-01	3.98e-01	-0.846239
201031_s_at	4.35e-01	3.98e-01	0.846183
209652_s_at	4.35e-01	3.98e-01	0.845416
215238_s_at	4.36e-01	3.98e-01	-0.845253
211633_x_at	4.36e-01	3.98e-01	-0.845159

214102_at	4.36e-01	3.99e-01	0.844665
210302_s_at	4.36e-01	3.99e-01	0.84437
210219_at	4.36e-01	3.99e-01	0.844126
209318_x_at	4.36e-01	3.99e-01	0.843852
206946_at	4.36e-01	3.99e-01	-0.84385
204853_at	4.37e-01	4.00e-01	0.84252
203917_at	4.38e-01	4.00e-01	-0.841574
211083_s_at	4.38e-01	4.01e-01	0.841236
205917_at	4.38e-01	4.01e-01	0.841145
214790_at	4.38e-01	4.01e-01	-0.840903
208487_at	4.38e-01	4.01e-01	-0.840719
220134_x_at	4.38e-01	4.01e-01	0.840623
213322_at	4.39e-01	4.02e-01	-0.83912
204789_at	4.39e-01	4.02e-01	-0.838986
200861_at	4.40e-01	4.03e-01	0.837664
210223_s_at	4.40e-01	4.03e-01	0.836712
212717_at	4.41e-01	4.04e-01	-0.836047
206984_s_at	4.41e-01	4.04e-01	-0.836036
213259_s_at	4.41e-01	4.04e-01	0.835715
214160_at	4.41e-01	4.04e-01	-0.835282
218250_s_at	4.41e-01	4.04e-01	0.834891
205275_at	4.41e-01	4.04e-01	-0.834797
219196_at	4.42e-01	4.04e-01	-0.834664
205483_s_at	4.42e-01	4.05e-01	0.834365
211160_x_at	4.42e-01	4.05e-01	0.834248
214976_at	4.42e-01	4.05e-01	-0.833704
219301_s_at	4.42e-01	4.05e-01	0.833657
208872_s_at	4.43e-01	4.06e-01	-0.832065
212889_x_at	4.43e-01	4.06e-01	-0.831998
216847_at	4.43e-01	4.06e-01	0.831734
221403_s_at	4.44e-01	4.06e-01	0.831043
203101_s_at	4.44e-01	4.07e-01	-0.830579
207758_at	4.44e-01	4.07e-01	-0.830081
220274_at	4.45e-01	4.08e-01	-0.828755
203925_at	4.45e-01	4.08e-01	0.828729
203978_at	4.45e-01	4.08e-01	0.828588
214189_s_at	4.45e-01	4.08e-01	-0.828466
206652_at	4.45e-01	4.08e-01	-0.828119
202648_at	4.46e-01	4.08e-01	0.827538
208371_s_at	4.46e-01	4.09e-01	0.826388
206369_s_at	4.46e-01	4.09e-01	-0.826262
40420_at	4.47e-01	4.10e-01	-0.824626
211081_s_at	4.47e-01	4.10e-01	0.824346
219091_s_at	4.48e-01	4.10e-01	-0.823972

200702_s_at	4.48e-01	4.11e-01	-0.823646
215764_x_at	4.48e-01	4.11e-01	0.823281
203220_s_at	4.49e-01	4.11e-01	0.822174
210150_s_at	4.49e-01	4.12e-01	-0.821971
214633_at	4.49e-01	4.12e-01	-0.820964
203399_x_at	4.49e-01	4.12e-01	0.820879
217065_at	4.49e-01	4.12e-01	-0.82087
211245_x_at	4.49e-01	4.12e-01	-0.820768
215489_x_at	4.50e-01	4.12e-01	0.820612
219566_at	4.50e-01	4.13e-01	-0.820185
212855_at	4.50e-01	4.13e-01	0.819701
212633_at	4.51e-01	4.14e-01	-0.818421
210282_at	4.51e-01	4.14e-01	0.818017
207585_s_at	4.51e-01	4.14e-01	0.817748
208358_s_at	4.51e-01	4.14e-01	0.817648
214094_at	4.51e-01	4.14e-01	0.817232
220898_at	4.52e-01	4.15e-01	-0.816655
221578_at	4.52e-01	4.15e-01	0.815705
218639_s_at	4.52e-01	4.15e-01	0.815595
218806_s_at	4.53e-01	4.15e-01	-0.81511
217109_at	4.53e-01	4.16e-01	-0.814452
202765_s_at	4.53e-01	4.16e-01	0.814083
203264_s_at	4.54e-01	4.16e-01	-0.813654
220896_at	4.54e-01	4.17e-01	0.813117
214322_at	4.54e-01	4.17e-01	-0.812942
216824_at	4.54e-01	4.17e-01	0.812705
218994_s_at	4.54e-01	4.17e-01	-0.811999
215486_at	4.55e-01	4.17e-01	-0.811884
205138_s_at	4.55e-01	4.18e-01	-0.811053
40148_at	4.55e-01	4.18e-01	0.810803
212182_at	4.55e-01	4.18e-01	-0.81072
206483_at	4.55e-01	4.18e-01	-0.810389
209743_s_at	4.57e-01	4.19e-01	-0.808565
222005_s_at	4.57e-01	4.19e-01	-0.808136
214134_at	4.57e-01	4.20e-01	-0.808
214200_s_at	4.57e-01	4.20e-01	-0.80756
220332_at	4.57e-01	4.20e-01	-0.807355
213736_at	4.59e-01	4.21e-01	-0.804796
204584_at	4.59e-01	4.22e-01	-0.803623
206915_at	4.60e-01	4.22e-01	0.803431
216421_at	4.60e-01	4.22e-01	0.803014
220760_x_at	4.60e-01	4.22e-01	-0.802901
214434_at	4.60e-01	4.22e-01	0.802896
209834_at	4.60e-01	4.23e-01	0.802755

202349_at	4.60e-01	4.23e-01	-0.80236
217651_at	4.60e-01	4.23e-01	0.802092
220216_at	4.60e-01	4.23e-01	-0.80209
208282_x_at	4.60e-01	4.23e-01	-0.801802
219466_s_at	4.60e-01	4.23e-01	-0.801697
211003_x_at	4.61e-01	4.23e-01	-0.801485
218753_at	4.61e-01	4.24e-01	0.800971
221029_s_at	4.61e-01	4.24e-01	-0.800469
205494_at	4.62e-01	4.24e-01	0.799505
203010_at	4.62e-01	4.25e-01	0.799019
221062_at	4.62e-01	4.25e-01	-0.798985
221430_s_at	4.62e-01	4.25e-01	0.798774
204870_s_at	4.63e-01	4.25e-01	-0.798123
204783_at	4.63e-01	4.25e-01	0.798097
207105_s_at	4.63e-01	4.26e-01	0.797494
217340_at	4.63e-01	4.26e-01	-0.797249
214451_at	4.63e-01	4.26e-01	-0.796922
210017_at	4.63e-01	4.26e-01	0.796897
205120_s_at	4.63e-01	4.26e-01	-0.796598
214190_x_at	4.64e-01	4.26e-01	0.795996
203223_at	4.64e-01	4.27e-01	-0.795283
211658_at	4.64e-01	4.27e-01	0.795044
211010_s_at	4.64e-01	4.27e-01	-0.794965
219236_at	4.64e-01	4.27e-01	-0.794798
213148_at	4.65e-01	4.27e-01	-0.794459
209999_x_at	4.65e-01	4.27e-01	-0.794236
211057_at	4.65e-01	4.28e-01	0.793618
206145_at	4.65e-01	4.28e-01	-0.793085
206339_at	4.65e-01	4.28e-01	-0.793065
202435_s_at	4.65e-01	4.28e-01	-0.792846
AFFX-HUMRGE/M100	4.66e-01	4.28e-01	0.792653
218083_at	4.66e-01	4.29e-01	-0.792399
206273_at	4.66e-01	4.29e-01	0.792289
218054_s_at	4.66e-01	4.29e-01	-0.792154
208575_at	4.66e-01	4.29e-01	-0.791456
218820_at	4.67e-01	4.30e-01	-0.790715
210947_s_at	4.67e-01	4.30e-01	-0.790301
212444_at	4.67e-01	4.30e-01	-0.790268
219667_s_at	4.67e-01	4.30e-01	-0.790026
208867_s_at	4.68e-01	4.31e-01	0.788952
203754_s_at	4.68e-01	4.31e-01	-0.788797
202534_x_at	4.68e-01	4.31e-01	0.788601
216035_x_at	4.68e-01	4.31e-01	-0.788162
209525_at	4.68e-01	4.31e-01	-0.788142

207209_at	4.68e-01	4.31e-01	-0.787892
221145_at	4.69e-01	4.31e-01	-0.787462
212465_at	4.69e-01	4.32e-01	0.787314
204307_at	4.69e-01	4.32e-01	-0.787295
211067_s_at	4.69e-01	4.32e-01	0.786173
209537_at	4.69e-01	4.32e-01	-0.786168
212123_at	4.70e-01	4.32e-01	0.785731
207789_s_at	4.70e-01	4.33e-01	-0.784795
205515_at	4.71e-01	4.34e-01	0.783425
206347_at	4.72e-01	4.35e-01	-0.782121
214878_at	4.72e-01	4.35e-01	0.78104
222277_at	4.73e-01	4.35e-01	0.780827
211446_at	4.73e-01	4.36e-01	0.780408
205523_at	4.73e-01	4.36e-01	-0.779712
216856_s_at	4.73e-01	4.36e-01	-0.779206
206420_at	4.74e-01	4.37e-01	-0.778767
218896_s_at	4.74e-01	4.37e-01	0.778691
210454_s_at	4.74e-01	4.37e-01	0.777876
208719_s_at	4.75e-01	4.37e-01	-0.7774
219950_s_at	4.75e-01	4.37e-01	-0.777195
208606_s_at	4.75e-01	4.38e-01	0.776982
215062_at	4.75e-01	4.38e-01	0.776795
213200_at	4.77e-01	4.39e-01	-0.773862
217529_at	4.78e-01	4.41e-01	-0.771644
221982_x_at	4.78e-01	4.41e-01	-0.771404
210729_at	4.78e-01	4.41e-01	-0.771041
214341_at	4.78e-01	4.41e-01	0.770993
219882_at	4.78e-01	4.41e-01	0.770957
206257_at	4.79e-01	4.42e-01	0.770339
201903_at	4.79e-01	4.42e-01	-0.769795
219616_at	4.80e-01	4.43e-01	0.768376
203628_at	4.80e-01	4.43e-01	-0.768047
202102_s_at	4.81e-01	4.43e-01	0.767377
220489_s_at	4.81e-01	4.44e-01	-0.766961
201813_s_at	4.81e-01	4.44e-01	-0.766425
214217_at	4.81e-01	4.44e-01	-0.766034
216818_s_at	4.82e-01	4.45e-01	0.765264
205154_at	4.82e-01	4.45e-01	-0.764637
218410_s_at	4.82e-01	4.45e-01	0.764388
207056_s_at	4.83e-01	4.45e-01	-0.763803
215767_at	4.83e-01	4.46e-01	0.763317
201227_s_at	4.84e-01	4.46e-01	0.762601
216049_at	4.84e-01	4.46e-01	0.762377
205501_at	4.84e-01	4.46e-01	-0.762265

220151_at	4.84e-01	4.47e-01	-0.761699
211782_at	4.84e-01	4.47e-01	-0.761101
220997_s_at	4.84e-01	4.47e-01	-0.761063
209776_s_at	4.84e-01	4.47e-01	0.760993
200672_x_at	4.84e-01	4.47e-01	-0.760848
208760_at	4.85e-01	4.47e-01	-0.760726
205266_at	4.85e-01	4.47e-01	0.760542
208094_s_at	4.85e-01	4.47e-01	0.760279
213756_s_at	4.85e-01	4.47e-01	0.760269
214111_at	4.85e-01	4.48e-01	0.760245
220603_s_at	4.85e-01	4.48e-01	-0.760169
219637_at	4.85e-01	4.48e-01	-0.760002
209419_at	4.85e-01	4.48e-01	-0.759757
205386_s_at	4.85e-01	4.48e-01	-0.758953
201568_at	4.86e-01	4.48e-01	-0.758603
210728_s_at	4.86e-01	4.49e-01	0.75831
208359_s_at	4.86e-01	4.49e-01	-0.758286
210395_x_at	4.87e-01	4.49e-01	0.757109
210365_at	4.87e-01	4.49e-01	-0.757012
210706_s_at	4.87e-01	4.50e-01	-0.756627
208622_s_at	4.88e-01	4.50e-01	-0.755572
219718_at	4.88e-01	4.51e-01	0.755019
206428_s_at	4.88e-01	4.51e-01	-0.754017
216801_at	4.89e-01	4.51e-01	-0.753732
215710_at	4.89e-01	4.52e-01	-0.753491
212346_s_at	4.89e-01	4.52e-01	0.753406
215011_at	4.89e-01	4.52e-01	-0.752776
216395_at	4.89e-01	4.52e-01	-0.752739
214128_at	4.89e-01	4.52e-01	-0.752702
203300_x_at	4.89e-01	4.52e-01	-0.752293
201551_s_at	4.90e-01	4.53e-01	0.751336
205816_at	4.90e-01	4.53e-01	-0.750963
213967_at	4.91e-01	4.53e-01	0.750506
202181_at	4.91e-01	4.53e-01	-0.750442
215436_at	4.91e-01	4.54e-01	0.749677
222174_at	4.91e-01	4.54e-01	-0.7492
200761_s_at	4.92e-01	4.55e-01	-0.748481
205223_at	4.92e-01	4.55e-01	-0.748285
208701_at	4.92e-01	4.55e-01	-0.74782
202045_s_at	4.92e-01	4.55e-01	0.747802
203005_at	4.92e-01	4.55e-01	-0.747537
219182_at	4.93e-01	4.55e-01	-0.74703
218548_x_at	4.93e-01	4.56e-01	-0.746614
207458_at	4.94e-01	4.56e-01	-0.745303

221518_s_at	4.94e-01	4.57e-01	-0.745158
206212_at	4.94e-01	4.57e-01	0.744054
217944_at	4.95e-01	4.57e-01	0.743852
202191_s_at	4.95e-01	4.58e-01	0.743548
211013_x_at	4.96e-01	4.59e-01	0.741299
205507_at	4.96e-01	4.59e-01	-0.741213
207214_at	4.96e-01	4.59e-01	-0.740931
207670_at	4.98e-01	4.60e-01	-0.738836
211547_s_at	4.98e-01	4.61e-01	-0.738561
217122_s_at	4.98e-01	4.61e-01	-0.73798
220572_at	4.98e-01	4.61e-01	0.737919
222070_at	4.99e-01	4.62e-01	0.736803
218268_at	5.00e-01	4.62e-01	-0.735899
200620_at	5.00e-01	4.62e-01	-0.735384
204774_at	5.00e-01	4.63e-01	-0.734951
209059_s_at	5.00e-01	4.63e-01	-0.734872
202707_at	5.00e-01	4.63e-01	-0.734791
216621_at	5.00e-01	4.63e-01	-0.734507
211403_x_at	5.01e-01	4.63e-01	0.734083
204379_s_at	5.01e-01	4.63e-01	-0.733955
209178_at	5.01e-01	4.64e-01	-0.733377
203988_s_at	5.02e-01	4.64e-01	0.732526
217276_x_at	5.02e-01	4.65e-01	0.731908
200758_s_at	5.02e-01	4.65e-01	0.731533
214958_s_at	5.03e-01	4.65e-01	0.730737
211810_s_at	5.03e-01	4.65e-01	0.730715
221793_at	5.03e-01	4.66e-01	-0.730274
205445_at	5.03e-01	4.66e-01	-0.730073
219359_at	5.03e-01	4.66e-01	-0.729778
213077_at	5.03e-01	4.66e-01	0.729426
211124_s_at	5.03e-01	4.66e-01	-0.729251
221840_at	5.03e-01	4.66e-01	0.729186
201877_s_at	5.04e-01	4.66e-01	-0.728958
202896_s_at	5.04e-01	4.67e-01	-0.728532
214528_s_at	5.04e-01	4.67e-01	-0.728454
213964_x_at	5.04e-01	4.67e-01	-0.727832
214185_at	5.04e-01	4.67e-01	-0.727487
204258_at	5.05e-01	4.67e-01	0.72722
214600_at	5.05e-01	4.68e-01	-0.727171
215381_at	5.05e-01	4.68e-01	-0.726571
208908_s_at	5.05e-01	4.68e-01	0.726509
203001_s_at	5.05e-01	4.68e-01	-0.726421
200793_s_at	5.06e-01	4.69e-01	0.725182
212272_at	5.06e-01	4.69e-01	0.724698

217631_at	5.08e-01	4.71e-01	0.721621
221267_s_at	5.08e-01	4.71e-01	0.721517
209399_at	5.09e-01	4.71e-01	0.721011
211388_s_at	5.09e-01	4.72e-01	-0.720258
203868_s_at	5.09e-01	4.72e-01	0.720184
220916_at	5.09e-01	4.72e-01	-0.719738
37590_g_at	5.09e-01	4.72e-01	-0.719565
219756_s_at	5.10e-01	4.72e-01	0.71916
221794_at	5.10e-01	4.73e-01	0.718257
200707_at	5.10e-01	4.73e-01	0.717749
202773_s_at	5.11e-01	4.73e-01	-0.717604
214062_x_at	5.11e-01	4.73e-01	-0.717539
204938_s_at	5.11e-01	4.74e-01	-0.716945
213680_at	5.11e-01	4.74e-01	-0.716837
219050_s_at	5.12e-01	4.74e-01	0.715926
219847_at	5.12e-01	4.75e-01	-0.715619
210165_at	5.12e-01	4.75e-01	-0.715105
215233_at	5.12e-01	4.75e-01	-0.714803
214353_at	5.13e-01	4.75e-01	-0.71417
222344_at	5.13e-01	4.76e-01	0.71324
206405_x_at	5.14e-01	4.76e-01	-0.7127
45526_g_at	5.14e-01	4.77e-01	0.7125
219391_at	5.14e-01	4.77e-01	-0.712088
201687_s_at	5.14e-01	4.77e-01	0.711586
205415_s_at	5.15e-01	4.78e-01	0.710612
206477_s_at	5.15e-01	4.78e-01	-0.709997
208045_at	5.16e-01	4.79e-01	-0.708516
215398_at	5.16e-01	4.79e-01	0.708141
213078_x_at	5.16e-01	4.79e-01	-0.70812
216124_at	5.17e-01	4.79e-01	-0.707771
221567_at	5.17e-01	4.79e-01	-0.707746
208202_s_at	5.17e-01	4.80e-01	0.707599
202008_s_at	5.17e-01	4.80e-01	-0.707394
210232_at	5.17e-01	4.80e-01	0.706847
205026_at	5.17e-01	4.80e-01	0.706729
200767_s_at	5.17e-01	4.80e-01	0.706682
202774_s_at	5.17e-01	4.80e-01	0.70636
201173_x_at	5.17e-01	4.80e-01	0.706338
204149_s_at	5.18e-01	4.81e-01	0.705895
215113_s_at	5.18e-01	4.81e-01	-0.705663
206025_s_at	5.18e-01	4.81e-01	-0.705583
215392_at	5.18e-01	4.81e-01	0.70551
203367_at	5.18e-01	4.81e-01	-0.70519
220181_x_at	5.18e-01	4.81e-01	-0.704852

205978_at	5.18e-01	4.81e-01	0.704727
214175_x_at	5.18e-01	4.81e-01	0.704578
207678_s_at	5.19e-01	4.82e-01	0.703703
209637_s_at	5.19e-01	4.82e-01	-0.703676
50221_at	5.20e-01	4.83e-01	0.702654
205295_at	5.20e-01	4.83e-01	-0.702358
214957_at	5.20e-01	4.83e-01	-0.70228
211118_x_at	5.20e-01	4.83e-01	-0.702261
207909_x_at	5.20e-01	4.83e-01	-0.702152
214397_at	5.20e-01	4.83e-01	0.701429
216632_at	5.20e-01	4.83e-01	-0.701306
219184_x_at	5.21e-01	4.84e-01	-0.701046
202353_s_at	5.21e-01	4.84e-01	-0.700688
216195_at	5.22e-01	4.85e-01	-0.699072
205543_at	5.22e-01	4.85e-01	-0.699054
215626_at	5.22e-01	4.85e-01	-0.69865
214351_x_at	5.22e-01	4.86e-01	0.698012
213063_at	5.22e-01	4.86e-01	-0.697951
203148_s_at	5.23e-01	4.86e-01	-0.697637
214108_at	5.23e-01	4.86e-01	-0.697286
204895_x_at	5.23e-01	4.86e-01	-0.697228
208667_s_at	5.23e-01	4.86e-01	-0.696939
203030_s_at	5.23e-01	4.86e-01	-0.696856
210846_x_at	5.24e-01	4.87e-01	-0.695915
216285_at	5.24e-01	4.87e-01	-0.695384
222080_s_at	5.24e-01	4.88e-01	-0.694657
210080_x_at	5.25e-01	4.89e-01	-0.693248
218620_s_at	5.25e-01	4.89e-01	-0.693142
222249_at	5.25e-01	4.89e-01	-0.693085
220184_at	5.25e-01	4.89e-01	-0.692946
212261_at	5.26e-01	4.89e-01	0.692368
207053_at	5.26e-01	4.89e-01	-0.692081
217744_s_at	5.26e-01	4.89e-01	0.691721
220168_at	5.26e-01	4.90e-01	0.691553
204663_at	5.26e-01	4.90e-01	-0.691443
204412_s_at	5.27e-01	4.90e-01	0.691227
205267_at	5.27e-01	4.90e-01	-0.690246
209559_at	5.27e-01	4.90e-01	-0.690236
207125_at	5.27e-01	4.91e-01	0.69
210205_at	5.28e-01	4.91e-01	-0.689204
214281_s_at	5.28e-01	4.91e-01	0.688984
221604_s_at	5.28e-01	4.92e-01	0.688366
220616_at	5.28e-01	4.92e-01	-0.688322
219465_at	5.29e-01	4.92e-01	0.687422

211616_s_at	5.29e-01	4.92e-01	0.687407
37802_r_at	5.29e-01	4.92e-01	0.686982
204760_s_at	5.29e-01	4.93e-01	-0.686631
221500_s_at	5.29e-01	4.93e-01	0.686359
215468_at	5.30e-01	4.93e-01	-0.685379
202952_s_at	5.30e-01	4.94e-01	-0.685144
202359_s_at	5.31e-01	4.94e-01	-0.68421
222240_s_at	5.31e-01	4.94e-01	0.684089
206147_x_at	5.31e-01	4.94e-01	0.683968
215650_at	5.31e-01	4.94e-01	0.683818
210948_s_at	5.32e-01	4.95e-01	0.68303
206072_at	5.32e-01	4.95e-01	-0.682755
221357_at	5.32e-01	4.95e-01	-0.682516
205202_at	5.32e-01	4.95e-01	0.682439
216020_at	5.33e-01	4.96e-01	0.681435
217217_at	5.33e-01	4.96e-01	-0.681292
210262_at	5.33e-01	4.96e-01	-0.681125
210147_at	5.33e-01	4.97e-01	-0.680309
214816_x_at	5.33e-01	4.97e-01	0.680255
221273_s_at	5.34e-01	4.97e-01	-0.679054
219948_x_at	5.34e-01	4.98e-01	0.67876
202965_s_at	5.34e-01	4.98e-01	0.678615
221623_at	5.35e-01	4.98e-01	0.677785
218364_at	5.35e-01	4.99e-01	-0.676947
208812_x_at	5.35e-01	4.99e-01	0.676863
218582_at	5.36e-01	4.99e-01	0.676594
217561_at	5.36e-01	5.00e-01	0.675793
218690_at	5.36e-01	5.00e-01	-0.675371
216490_x_at	5.36e-01	5.00e-01	-0.675248
210195_s_at	5.37e-01	5.00e-01	-0.674406
203527_s_at	5.37e-01	5.01e-01	-0.674247
214502_at	5.37e-01	5.01e-01	-0.673733
215460_x_at	5.37e-01	5.01e-01	-0.673636
207723_s_at	5.38e-01	5.01e-01	-0.673237
212318_at	5.38e-01	5.01e-01	-0.673054
221756_at	5.38e-01	5.02e-01	-0.672189
218793_s_at	5.38e-01	5.02e-01	-0.672062
211864_s_at	5.38e-01	5.02e-01	-0.671963
200810_s_at	5.39e-01	5.02e-01	-0.671576
205414_s_at	5.39e-01	5.03e-01	0.670752
217269_s_at	5.39e-01	5.03e-01	0.670378
207043_s_at	5.39e-01	5.03e-01	-0.670213
203972_s_at	5.41e-01	5.04e-01	-0.668117
203494_s_at	5.41e-01	5.04e-01	-0.668056

206021_at	5.41e-01	5.05e-01	-0.667727
211078_s_at	5.42e-01	5.05e-01	0.666619
207705_s_at	5.42e-01	5.05e-01	-0.666403
207958_at	5.42e-01	5.06e-01	-0.666369
200961_at	5.42e-01	5.06e-01	-0.666055
209663_s_at	5.42e-01	5.06e-01	0.666042
201879_at	5.42e-01	5.06e-01	0.665747
202210_x_at	5.43e-01	5.06e-01	-0.664889
205825_at	5.43e-01	5.07e-01	0.664762
221874_at	5.43e-01	5.07e-01	-0.664727
204491_at	5.43e-01	5.07e-01	-0.664403
217743_s_at	5.43e-01	5.07e-01	0.664257
220287_at	5.43e-01	5.07e-01	-0.663941
203890_s_at	5.44e-01	5.07e-01	0.663702
206714_at	5.44e-01	5.07e-01	-0.663309
209341_s_at	5.44e-01	5.08e-01	-0.662253
206333_at	5.45e-01	5.08e-01	-0.662152
220612_at	5.45e-01	5.09e-01	0.661377
211499_s_at	5.45e-01	5.09e-01	-0.660807
206644_at	5.45e-01	5.09e-01	-0.660696
203314_at	5.46e-01	5.10e-01	-0.659562
221785_at	5.47e-01	5.11e-01	0.657996
206669_at	5.47e-01	5.11e-01	0.657987
203444_s_at	5.47e-01	5.11e-01	-0.657573
212443_at	5.48e-01	5.12e-01	-0.656908
221907_at	5.48e-01	5.12e-01	0.656807
218063_s_at	5.48e-01	5.12e-01	-0.656527
201396_s_at	5.48e-01	5.12e-01	-0.656378
217111_at	5.49e-01	5.12e-01	0.655589
215571_at	5.49e-01	5.13e-01	0.655468
208250_s_at	5.49e-01	5.13e-01	-0.654482
202738_s_at	5.50e-01	5.14e-01	-0.653903
210650_s_at	5.50e-01	5.14e-01	0.653767
205469_s_at	5.50e-01	5.14e-01	0.653195
216270_at	5.51e-01	5.15e-01	-0.651966
207802_at	5.51e-01	5.15e-01	0.651493
217110_s_at	5.52e-01	5.15e-01	-0.651056
205151_s_at	5.52e-01	5.16e-01	-0.650302
220621_at	5.53e-01	5.16e-01	0.649567
210767_at	5.53e-01	5.17e-01	-0.649249
206100_at	5.54e-01	5.17e-01	0.647754
205775_at	5.54e-01	5.18e-01	-0.647561
213563_s_at	5.54e-01	5.18e-01	-0.647525
216508_x_at	5.55e-01	5.18e-01	-0.646541

211998_at	5.55e-01	5.19e-01	0.646105
206940_s_at	5.55e-01	5.19e-01	-0.646098
217273_at	5.55e-01	5.19e-01	-0.645786
208315_x_at	5.55e-01	5.19e-01	0.645461
206348_s_at	5.56e-01	5.19e-01	0.644736
212682_s_at	5.56e-01	5.20e-01	0.644607
217623_at	5.56e-01	5.20e-01	-0.644367
220546_at	5.56e-01	5.20e-01	0.644286
212377_s_at	5.57e-01	5.20e-01	0.643241
202499_s_at	5.57e-01	5.21e-01	-0.64241
217497_at	5.57e-01	5.21e-01	-0.64224
209835_x_at	5.57e-01	5.21e-01	0.642154
216287_at	5.57e-01	5.21e-01	-0.642035
210818_s_at	5.57e-01	5.21e-01	-0.641806
219655_at	5.58e-01	5.22e-01	0.641162
208865_at	5.58e-01	5.22e-01	-0.641145
203942_s_at	5.58e-01	5.22e-01	0.640956
200811_at	5.58e-01	5.22e-01	0.640593
208328_s_at	5.58e-01	5.22e-01	0.640526
207428_x_at	5.59e-01	5.23e-01	0.639521
205100_at	5.59e-01	5.23e-01	-0.639162
215948_x_at	5.60e-01	5.24e-01	0.638313
205764_at	5.60e-01	5.24e-01	-0.637646
209552_at	5.60e-01	5.24e-01	-0.637508
216985_s_at	5.60e-01	5.24e-01	0.637313
213856_at	5.61e-01	5.25e-01	0.636838
43544_at	5.61e-01	5.25e-01	-0.636412
222088_s_at	5.61e-01	5.25e-01	-0.636094
215851_at	5.61e-01	5.25e-01	-0.635568
222094_at	5.62e-01	5.26e-01	0.634704
209349_at	5.62e-01	5.26e-01	0.634555
206167_s_at	5.62e-01	5.26e-01	0.634546
209413_at	5.62e-01	5.26e-01	-0.634444
210778_s_at	5.63e-01	5.27e-01	0.633732
209913_x_at	5.63e-01	5.27e-01	0.633658
207496_at	5.63e-01	5.27e-01	-0.633513
216052_x_at	5.63e-01	5.27e-01	-0.633104
64408_s_at	5.63e-01	5.27e-01	0.632911
221705_s_at	5.63e-01	5.28e-01	0.632309
205450_at	5.63e-01	5.28e-01	-0.632175
201913_s_at	5.63e-01	5.28e-01	0.632084
214243_s_at	5.64e-01	5.28e-01	0.631014
207418_s_at	5.64e-01	5.29e-01	0.630556
218382_s_at	5.65e-01	5.29e-01	0.630479

222108_at	5.65e-01	5.29e-01	-0.630313
214537_at	5.65e-01	5.30e-01	-0.629055
222366_at	5.66e-01	5.30e-01	0.628953
201218_at	5.66e-01	5.30e-01	-0.628447
211799_x_at	5.66e-01	5.30e-01	-0.627881
220026_at	5.66e-01	5.30e-01	-0.627868
215757_at	5.67e-01	5.31e-01	0.627212
218397_at	5.67e-01	5.31e-01	-0.626661
204861_s_at	5.67e-01	5.31e-01	-0.626488
210904_s_at	5.68e-01	5.32e-01	-0.624858
218468_s_at	5.68e-01	5.32e-01	0.624823
204381_at	5.68e-01	5.32e-01	0.624711
217218_at	5.68e-01	5.33e-01	-0.624601
211700_s_at	5.68e-01	5.33e-01	-0.62438
205778_at	5.68e-01	5.33e-01	-0.624255
204436_at	5.69e-01	5.33e-01	0.623765
214667_s_at	5.70e-01	5.34e-01	0.622606
208714_at	5.70e-01	5.34e-01	0.62209
215309_at	5.70e-01	5.35e-01	0.621575
222304_x_at	5.71e-01	5.35e-01	0.621052
221512_at	5.71e-01	5.35e-01	0.620251
209198_s_at	5.71e-01	5.36e-01	-0.620083
222276_at	5.72e-01	5.36e-01	0.619329
205319_at	5.73e-01	5.37e-01	-0.618096
219381_at	5.73e-01	5.37e-01	-0.617113
211479_s_at	5.74e-01	5.38e-01	-0.616624
219851_at	5.74e-01	5.38e-01	0.61641
216721_at	5.74e-01	5.38e-01	-0.616087
201842_s_at	5.74e-01	5.38e-01	-0.616028
202958_at	5.74e-01	5.39e-01	-0.615391
208501_at	5.74e-01	5.39e-01	0.615057
202271_at	5.74e-01	5.39e-01	0.614997
218990_s_at	5.75e-01	5.39e-01	-0.614496
203187_at	5.75e-01	5.39e-01	-0.614341
215159_s_at	5.75e-01	5.40e-01	-0.613993
206199_at	5.75e-01	5.40e-01	0.613958
203413_at	5.75e-01	5.40e-01	-0.613653
215094_at	5.75e-01	5.40e-01	-0.613622
216277_at	5.76e-01	5.40e-01	0.613235
207107_at	5.76e-01	5.40e-01	-0.612808
216697_at	5.76e-01	5.40e-01	-0.612549
219114_at	5.76e-01	5.41e-01	-0.612191
217556_at	5.76e-01	5.41e-01	0.611905
210745_at	5.77e-01	5.41e-01	-0.611289

218577_at	5.77e-01	5.42e-01	-0.610617
211092_s_at	5.77e-01	5.42e-01	-0.610327
219316_s_at	5.78e-01	5.43e-01	0.609265
215395_x_at	5.78e-01	5.43e-01	-0.608925
208023_at	5.79e-01	5.43e-01	-0.608573
204796_at	5.79e-01	5.43e-01	-0.608505
206431_x_at	5.79e-01	5.43e-01	0.608503
215711_s_at	5.79e-01	5.43e-01	0.608448
207520_at	5.79e-01	5.44e-01	0.607766
205322_s_at	5.79e-01	5.44e-01	0.607725
218177_at	5.79e-01	5.44e-01	0.6074
201046_s_at	5.80e-01	5.44e-01	0.606802
205694_at	5.80e-01	5.44e-01	0.606785
221478_at	5.80e-01	5.44e-01	-0.606592
220257_x_at	5.80e-01	5.45e-01	-0.605892
205426_s_at	5.80e-01	5.45e-01	0.605494
203532_x_at	5.81e-01	5.46e-01	0.604749
219877_at	5.83e-01	5.47e-01	-0.601975
201706_s_at	5.83e-01	5.48e-01	0.60113
203512_at	5.83e-01	5.48e-01	0.600993
202482_x_at	5.84e-01	5.48e-01	-0.600836
208215_x_at	5.84e-01	5.48e-01	-0.600689
202348_s_at	5.84e-01	5.49e-01	-0.600417
213640_s_at	5.84e-01	5.49e-01	-0.599636
214346_at	5.86e-01	5.50e-01	0.597947
200921_s_at	5.86e-01	5.51e-01	-0.597439
214624_at	5.87e-01	5.51e-01	-0.596447
217372_at	5.87e-01	5.51e-01	-0.596378
217383_at	5.87e-01	5.51e-01	0.596237
221628_s_at	5.87e-01	5.51e-01	0.596164
217500_at	5.88e-01	5.53e-01	0.594224
219209_at	5.88e-01	5.53e-01	0.594213
206935_at	5.88e-01	5.53e-01	0.593698
214887_at	5.89e-01	5.53e-01	0.593295
211874_s_at	5.89e-01	5.53e-01	-0.593056
207326_at	5.89e-01	5.53e-01	-0.593
215713_at	5.89e-01	5.54e-01	-0.592655
213201_s_at	5.89e-01	5.54e-01	0.592331
208790_s_at	5.90e-01	5.54e-01	-0.591731
214037_s_at	5.90e-01	5.55e-01	-0.591457
206366_x_at	5.90e-01	5.55e-01	-0.590756
206190_at	5.90e-01	5.55e-01	-0.590599
213802_at	5.91e-01	5.55e-01	-0.589988
215981_at	5.91e-01	5.56e-01	-0.589961

205991_s_at	5.92e-01	5.57e-01	-0.588326
206794_at	5.92e-01	5.57e-01	-0.588119
218064_s_at	5.92e-01	5.57e-01	-0.58807
208053_at	5.92e-01	5.57e-01	-0.587393
219750_at	5.92e-01	5.57e-01	-0.587273
203706_s_at	5.93e-01	5.58e-01	0.586947
218223_s_at	5.93e-01	5.58e-01	0.586485
208035_at	5.93e-01	5.58e-01	-0.58624
211648_at	5.93e-01	5.58e-01	-0.586064
201301_s_at	5.93e-01	5.58e-01	0.585879
200848_at	5.93e-01	5.58e-01	0.585724
206876_at	5.94e-01	5.59e-01	-0.58534
211472_at	5.94e-01	5.59e-01	-0.58519
212947_at	5.95e-01	5.60e-01	-0.583626
212890_at	5.95e-01	5.60e-01	-0.583208
220619_at	5.95e-01	5.60e-01	0.582879
205487_s_at	5.96e-01	5.61e-01	-0.581702
218112_at	5.96e-01	5.61e-01	0.581135
203440_at	5.97e-01	5.62e-01	-0.580618
220404_at	5.97e-01	5.62e-01	-0.580422
215523_at	5.97e-01	5.62e-01	-0.580258
211091_s_at	5.97e-01	5.62e-01	-0.580097
48825_at	5.97e-01	5.62e-01	-0.5796
214824_at	5.99e-01	5.64e-01	-0.577671
209972_s_at	5.99e-01	5.64e-01	-0.577375
202745_at	5.99e-01	5.64e-01	-0.577273
209439_s_at	5.99e-01	5.64e-01	-0.576726
216548_x_at	5.99e-01	5.64e-01	0.576714
212951_at	6.00e-01	5.65e-01	-0.576361
210933_s_at	6.00e-01	5.65e-01	-0.576017
206596_s_at	6.00e-01	5.65e-01	0.575737
206611_at	6.00e-01	5.65e-01	-0.575625
221507_at	6.01e-01	5.66e-01	-0.574047
214251_s_at	6.02e-01	5.67e-01	-0.573222
218851_s_at	6.02e-01	5.68e-01	0.572157
214611_at	6.03e-01	5.68e-01	-0.571589
213216_at	6.03e-01	5.68e-01	-0.571177
204117_at	6.03e-01	5.68e-01	-0.570712
219711_at	6.03e-01	5.69e-01	-0.570674
201918_at	6.04e-01	5.69e-01	-0.569977
216723_at	6.04e-01	5.69e-01	-0.569754
210721_s_at	6.04e-01	5.70e-01	-0.569094
213722_at	6.05e-01	5.70e-01	0.568716
211273_s_at	6.05e-01	5.70e-01	-0.568696

207649_at	6.05e-01	5.71e-01	-0.567642
211967_at	6.05e-01	5.71e-01	-0.567526
206997_s_at	6.05e-01	5.71e-01	0.567349
204249_s_at	6.05e-01	5.71e-01	-0.567338
206497_at	6.06e-01	5.71e-01	-0.567099
213340_s_at	6.07e-01	5.72e-01	-0.565807
217318_x_at	6.07e-01	5.72e-01	-0.565102
206434_at	6.08e-01	5.73e-01	-0.564263
215575_at	6.08e-01	5.73e-01	-0.563735
202423_at	6.08e-01	5.73e-01	-0.563691
206836_at	6.09e-01	5.74e-01	-0.562822
219936_s_at	6.09e-01	5.74e-01	0.562721
214515_at	6.09e-01	5.75e-01	-0.561633
217562_at	6.09e-01	5.75e-01	0.561538
215219_at	6.09e-01	5.75e-01	-0.561512
216581_at	6.10e-01	5.75e-01	-0.561239
206005_s_at	6.10e-01	5.76e-01	0.560067
219207_at	6.11e-01	5.76e-01	-0.559628
41660_at	6.11e-01	5.77e-01	-0.558509
220469_at	6.11e-01	5.77e-01	-0.558467
209264_s_at	6.12e-01	5.78e-01	0.557306
210936_at	6.13e-01	5.78e-01	-0.556758
208309_s_at	6.13e-01	5.78e-01	0.556564
219759_at	6.13e-01	5.78e-01	0.556547
221409_at	6.13e-01	5.78e-01	0.556139
210203_at	6.14e-01	5.79e-01	-0.55488
219680_at	6.14e-01	5.79e-01	-0.554585
204393_s_at	6.14e-01	5.80e-01	-0.554258
214416_at	6.14e-01	5.80e-01	-0.554161
204901_at	6.14e-01	5.80e-01	0.553834
219361_s_at	6.15e-01	5.80e-01	-0.553632
203265_s_at	6.15e-01	5.81e-01	0.552633
220324_at	6.16e-01	5.81e-01	-0.552114
216326_s_at	6.16e-01	5.81e-01	-0.551733
201958_s_at	6.16e-01	5.82e-01	-0.550962
221592_at	6.16e-01	5.82e-01	-0.550824
214025_at	6.17e-01	5.82e-01	0.550737
217526_at	6.18e-01	5.83e-01	-0.548804
206999_at	6.18e-01	5.84e-01	-0.548165
206240_s_at	6.19e-01	5.84e-01	-0.547866
220103_s_at	6.19e-01	5.84e-01	-0.547531
208300_at	6.20e-01	5.85e-01	-0.546111
220540_at	6.20e-01	5.85e-01	-0.5461
214666_x_at	6.20e-01	5.85e-01	0.546017

222052_at	6.20e-01	5.86e-01	0.545435
222057_at	6.20e-01	5.86e-01	-0.54501
220573_at	6.20e-01	5.86e-01	-0.544945
209672_s_at	6.20e-01	5.86e-01	0.544876
218668_s_at	6.21e-01	5.86e-01	0.544566
222245_s_at	6.22e-01	5.88e-01	0.5428
217639_at	6.23e-01	5.88e-01	-0.541664
215549_x_at	6.23e-01	5.89e-01	-0.540682
219556_at	6.24e-01	5.89e-01	0.540409
221812_at	6.24e-01	5.90e-01	-0.539533
216752_at	6.24e-01	5.90e-01	-0.539337
33148_at	6.24e-01	5.90e-01	0.539314
206014_at	6.24e-01	5.90e-01	-0.539208
201447_at	6.24e-01	5.90e-01	-0.539175
214176_s_at	6.25e-01	5.91e-01	0.538481
208662_s_at	6.25e-01	5.91e-01	-0.538438
213363_at	6.25e-01	5.91e-01	0.538283
221813_at	6.25e-01	5.91e-01	0.537621
202429_s_at	6.26e-01	5.91e-01	0.537348
222008_at	6.26e-01	5.91e-01	0.537177
207932_at	6.26e-01	5.92e-01	-0.537003
218418_s_at	6.26e-01	5.92e-01	0.536818
212311_at	6.26e-01	5.92e-01	-0.5365
221431_s_at	6.26e-01	5.92e-01	-0.535852
208195_at	6.27e-01	5.92e-01	0.535647
205840_x_at	6.27e-01	5.93e-01	0.535225
206641_at	6.27e-01	5.93e-01	-0.534894
204714_s_at	6.27e-01	5.93e-01	0.534481
215750_at	6.28e-01	5.94e-01	-0.533117
201613_s_at	6.28e-01	5.94e-01	-0.532885
207774_at	6.30e-01	5.95e-01	-0.531341
209101_at	6.30e-01	5.96e-01	-0.531226
204340_at	6.30e-01	5.96e-01	0.5309
212003_at	6.30e-01	5.96e-01	-0.530796
217429_at	6.30e-01	5.96e-01	-0.530784
215991_s_at	6.31e-01	5.97e-01	-0.529703
201506_at	6.31e-01	5.97e-01	-0.529234
201867_s_at	6.31e-01	5.97e-01	-0.529183
220565_at	6.31e-01	5.97e-01	-0.529164
204586_at	6.31e-01	5.97e-01	-0.529154
205006_s_at	6.31e-01	5.97e-01	0.528961
212173_at	6.31e-01	5.97e-01	0.52848
220822_at	6.31e-01	5.98e-01	-0.528358
208335_s_at	6.32e-01	5.98e-01	-0.528014

202335_s_at	6.32e-01	5.98e-01	0.527985
203734_at	6.32e-01	5.98e-01	-0.527794
204489_s_at	6.32e-01	5.98e-01	0.527521
219334_s_at	6.32e-01	5.98e-01	0.527399
219686_at	6.32e-01	5.98e-01	-0.527364
207109_at	6.32e-01	5.98e-01	0.52713
205551_at	6.32e-01	5.99e-01	0.526854
205113_at	6.32e-01	5.99e-01	-0.526841
217260_x_at	6.33e-01	5.99e-01	-0.525884
209640_at	6.33e-01	6.00e-01	-0.525131
201143_s_at	6.34e-01	6.00e-01	0.524953
207258_at	6.34e-01	6.00e-01	-0.524123
213884_s_at	6.34e-01	6.01e-01	-0.524048
206604_at	6.35e-01	6.02e-01	-0.522582
205615_at	6.35e-01	6.02e-01	-0.522459
203031_s_at	6.35e-01	6.02e-01	0.522368
213495_s_at	6.36e-01	6.02e-01	0.52181
214361_s_at	6.36e-01	6.02e-01	-0.521545
204983_s_at	6.36e-01	6.03e-01	-0.52082
217205_at	6.37e-01	6.03e-01	0.520144
216787_at	6.37e-01	6.03e-01	-0.519986
207402_at	6.38e-01	6.04e-01	0.518801
216526_x_at	6.38e-01	6.04e-01	0.518675
216452_at	6.39e-01	6.05e-01	0.517558
201996_s_at	6.39e-01	6.05e-01	-0.517331
204599_s_at	6.39e-01	6.05e-01	0.517115
214686_at	6.39e-01	6.06e-01	-0.51648
35671_at	6.39e-01	6.06e-01	0.51631
215684_s_at	6.40e-01	6.06e-01	-0.515699
214640_at	6.41e-01	6.07e-01	0.514526
207000_s_at	6.41e-01	6.08e-01	-0.513407
207879_at	6.42e-01	6.08e-01	0.512871
214370_at	6.42e-01	6.09e-01	0.512455
206243_at	6.42e-01	6.09e-01	-0.512452
221537_at	6.43e-01	6.10e-01	-0.510712
214618_at	6.44e-01	6.10e-01	-0.509818
221048_x_at	6.44e-01	6.11e-01	0.50962
202411_at	6.44e-01	6.11e-01	-0.509115
208621_s_at	6.44e-01	6.11e-01	-0.509107
202488_s_at	6.45e-01	6.11e-01	-0.50874
218502_s_at	6.45e-01	6.12e-01	0.50803
218887_at	6.45e-01	6.12e-01	0.507661
220328_at	6.46e-01	6.13e-01	-0.50664
216823_at	6.48e-01	6.14e-01	0.504258

213586_at	6.48e-01	6.15e-01	-0.504
205976_at	6.48e-01	6.15e-01	0.503433
215752_at	6.49e-01	6.15e-01	0.503117
205399_at	6.49e-01	6.15e-01	-0.503048
214120_at	6.49e-01	6.15e-01	0.502818
201148_s_at	6.49e-01	6.16e-01	-0.50252
212352_s_at	6.49e-01	6.16e-01	0.502182
214305_s_at	6.49e-01	6.16e-01	-0.502161
220027_s_at	6.49e-01	6.16e-01	0.502103
222199_s_at	6.49e-01	6.16e-01	-0.501617
206322_at	6.49e-01	6.16e-01	-0.501494
220716_at	6.50e-01	6.17e-01	0.501105
207852_at	6.50e-01	6.17e-01	0.501067
205715_at	6.50e-01	6.17e-01	-0.500818
220326_s_at	6.50e-01	6.17e-01	-0.500165
219701_at	6.50e-01	6.17e-01	-0.500124
220334_at	6.51e-01	6.18e-01	0.499055
213719_s_at	6.51e-01	6.18e-01	0.498853
217053_x_at	6.52e-01	6.18e-01	-0.498361
217107_at	6.52e-01	6.19e-01	0.497929
213325_at	6.52e-01	6.19e-01	-0.497889
204399_s_at	6.52e-01	6.19e-01	0.497698
211065_x_at	6.52e-01	6.19e-01	-0.497142
219859_at	6.53e-01	6.20e-01	-0.496566
220200_s_at	6.53e-01	6.20e-01	-0.496417
222254_at	6.53e-01	6.20e-01	0.496355
203020_at	6.53e-01	6.20e-01	0.495725
206637_at	6.53e-01	6.21e-01	0.495441
209911_x_at	6.54e-01	6.21e-01	0.495269
209352_s_at	6.54e-01	6.21e-01	-0.494898
206288_at	6.54e-01	6.21e-01	-0.49477
222195_s_at	6.54e-01	6.22e-01	0.493991
220229_s_at	6.55e-01	6.22e-01	0.493236
216513_at	6.56e-01	6.23e-01	-0.491295
204364_s_at	6.58e-01	6.25e-01	-0.489277
219714_s_at	6.59e-01	6.26e-01	0.488073
206393_at	6.59e-01	6.26e-01	0.488014
206884_s_at	6.60e-01	6.27e-01	-0.48677
217505_at	6.60e-01	6.27e-01	0.486345
215724_at	6.60e-01	6.27e-01	-0.48604
217588_at	6.60e-01	6.27e-01	0.4856
220380_at	6.60e-01	6.28e-01	0.485562
35685_at	6.61e-01	6.28e-01	-0.485018
214398_s_at	6.61e-01	6.28e-01	-0.484714

208990_s_at	6.61e-01	6.28e-01	-0.484235
215351_at	6.63e-01	6.30e-01	-0.48215
209197_at	6.63e-01	6.30e-01	-0.481874
214412_at	6.63e-01	6.30e-01	-0.481393
209008_x_at	6.64e-01	6.31e-01	-0.481036
213901_x_at	6.64e-01	6.31e-01	0.480679
220345_at	6.64e-01	6.31e-01	0.480676
217455_s_at	6.64e-01	6.31e-01	-0.480563
218030_at	6.64e-01	6.31e-01	-0.480261
208323_s_at	6.64e-01	6.32e-01	-0.479902
206415_at	6.64e-01	6.32e-01	-0.479577
211167_s_at	6.65e-01	6.32e-01	-0.479201
207869_s_at	6.65e-01	6.33e-01	0.478409
213743_at	6.65e-01	6.33e-01	-0.478336
214904_at	6.65e-01	6.33e-01	-0.478315
207867_at	6.65e-01	6.33e-01	-0.478126
216917_s_at	6.65e-01	6.33e-01	-0.478077
215072_x_at	6.65e-01	6.33e-01	0.478049
213470_s_at	6.65e-01	6.33e-01	-0.477849
206426_at	6.66e-01	6.34e-01	-0.476952
208547_at	6.66e-01	6.34e-01	0.476423
211889_x_at	6.67e-01	6.35e-01	-0.475697
218456_at	6.67e-01	6.35e-01	0.475525
207279_s_at	6.67e-01	6.35e-01	-0.475419
221976_s_at	6.68e-01	6.35e-01	-0.474619
207656_s_at	6.68e-01	6.35e-01	-0.4745
212822_at	6.69e-01	6.36e-01	0.473293
217539_at	6.69e-01	6.36e-01	-0.472981
208046_at	6.69e-01	6.37e-01	-0.472245
216501_at	6.70e-01	6.37e-01	0.471586
217935_s_at	6.70e-01	6.38e-01	-0.471479
205505_at	6.70e-01	6.38e-01	0.471445
220519_s_at	6.70e-01	6.38e-01	-0.471046
201182_s_at	6.70e-01	6.38e-01	-0.470784
205624_at	6.70e-01	6.38e-01	-0.470768
76897_s_at	6.71e-01	6.39e-01	-0.470114
204490_s_at	6.71e-01	6.39e-01	0.46996
221345_at	6.72e-01	6.39e-01	-0.468862
216798_at	6.72e-01	6.40e-01	0.468111
214246_x_at	6.73e-01	6.40e-01	-0.467438
201700_at	6.73e-01	6.41e-01	0.46731
213789_at	6.73e-01	6.41e-01	0.466577
202082_s_at	6.73e-01	6.41e-01	0.466449
220848_x_at	6.73e-01	6.41e-01	-0.46637

216246_at	6.74e-01	6.41e-01	-0.465945
219896_at	6.74e-01	6.42e-01	0.464951
211671_s_at	6.74e-01	6.42e-01	-0.464828
220998_s_at	6.75e-01	6.43e-01	0.464183
207413_s_at	6.75e-01	6.43e-01	-0.464068
214436_at	6.76e-01	6.44e-01	0.462709
220803_at	6.76e-01	6.44e-01	-0.462504
211713_x_at	6.76e-01	6.44e-01	-0.462172
218972_at	6.76e-01	6.44e-01	-0.461921
222196_at	6.77e-01	6.45e-01	-0.461348
217721_at	6.77e-01	6.46e-01	0.460292
219828_at	6.78e-01	6.46e-01	-0.460031
214651_s_at	6.79e-01	6.47e-01	0.458802
204406_at	6.79e-01	6.47e-01	0.458751
65438_at	6.79e-01	6.47e-01	-0.458223
212069_s_at	6.80e-01	6.48e-01	0.456729
212926_at	6.81e-01	6.49e-01	-0.455807
210145_at	6.81e-01	6.49e-01	-0.454999
222365_at	6.82e-01	6.50e-01	0.454353
220784_s_at	6.82e-01	6.50e-01	0.454352
214655_at	6.82e-01	6.51e-01	0.453342
202392_s_at	6.83e-01	6.51e-01	-0.452949
209570_s_at	6.83e-01	6.51e-01	-0.452525
217126_at	6.83e-01	6.51e-01	-0.452078
220258_s_at	6.83e-01	6.52e-01	0.451822
214123_s_at	6.84e-01	6.52e-01	-0.451425
218798_at	6.84e-01	6.52e-01	0.451327
205294_at	6.84e-01	6.52e-01	-0.451081
203377_s_at	6.84e-01	6.52e-01	-0.451075
213938_at	6.84e-01	6.52e-01	-0.450796
222367_at	6.84e-01	6.52e-01	-0.450741
203051_at	6.84e-01	6.52e-01	-0.450677
215315_at	6.84e-01	6.53e-01	-0.450507
203844_at	6.84e-01	6.53e-01	0.450506
212548_s_at	6.84e-01	6.53e-01	-0.450091
201732_s_at	6.85e-01	6.53e-01	-0.449837
203323_at	6.85e-01	6.53e-01	-0.449767
214443_at	6.85e-01	6.53e-01	-0.44966
222308_x_at	6.85e-01	6.53e-01	0.449413
221410_x_at	6.85e-01	6.54e-01	0.448988
52975_at	6.85e-01	6.54e-01	0.448764
217607_x_at	6.85e-01	6.54e-01	-0.448467
214763_at	6.85e-01	6.54e-01	0.448404
221788_at	6.86e-01	6.54e-01	0.448076

202490_at	6.86e-01	6.55e-01	0.447653
218818_at	6.87e-01	6.55e-01	0.446658
214250_at	6.87e-01	6.56e-01	-0.445842
214068_at	6.87e-01	6.56e-01	-0.445579
204520_x_at	6.87e-01	6.56e-01	-0.445564
214956_at	6.88e-01	6.56e-01	0.445211
217132_at	6.88e-01	6.57e-01	-0.444568
206806_at	6.88e-01	6.57e-01	-0.444385
201648_at	6.88e-01	6.57e-01	-0.443943
207179_at	6.89e-01	6.58e-01	-0.443548
205374_at	6.89e-01	6.58e-01	0.443401
212031_at	6.89e-01	6.58e-01	-0.44312
207607_at	6.89e-01	6.58e-01	0.443111
221792_at	6.89e-01	6.58e-01	-0.442947
202895_s_at	6.89e-01	6.58e-01	-0.442842
220280_s_at	6.89e-01	6.58e-01	0.442678
213234_at	6.90e-01	6.59e-01	-0.44146
207362_at	6.90e-01	6.59e-01	-0.44107
217467_at	6.91e-01	6.60e-01	0.440475
213396_s_at	6.91e-01	6.60e-01	-0.440198
219160_s_at	6.92e-01	6.61e-01	-0.43822
209239_at	6.93e-01	6.62e-01	-0.437923
205748_s_at	6.93e-01	6.62e-01	-0.436942
216874_at	6.93e-01	6.62e-01	-0.436859
212985_at	6.94e-01	6.63e-01	-0.436264
212458_at	6.94e-01	6.63e-01	0.436009
205954_at	6.94e-01	6.63e-01	0.435695
206607_at	6.94e-01	6.63e-01	0.435659
213651_at	6.94e-01	6.64e-01	-0.435169
209046_s_at	6.95e-01	6.64e-01	0.434208
214401_at	6.96e-01	6.65e-01	-0.433402
208313_s_at	6.96e-01	6.65e-01	0.433259
203204_s_at	6.96e-01	6.66e-01	0.432456
215301_at	6.96e-01	6.66e-01	0.43234
221688_s_at	6.96e-01	6.66e-01	-0.432279
1729_at	6.97e-01	6.66e-01	-0.431879
205123_s_at	6.97e-01	6.66e-01	-0.4317
210834_s_at	6.97e-01	6.66e-01	-0.431366
204021_s_at	6.98e-01	6.67e-01	0.430482
212470_at	6.98e-01	6.67e-01	0.429957
200850_s_at	6.98e-01	6.68e-01	-0.42974
221910_at	6.99e-01	6.68e-01	0.429039
201917_s_at	6.99e-01	6.69e-01	-0.428411
210278_s_at	6.99e-01	6.69e-01	-0.428317

206818_s_at	6.99e-01	6.69e-01	0.428092
206097_at	7.00e-01	6.69e-01	0.427621
217363_x_at	7.00e-01	6.69e-01	-0.427599
207266_x_at	7.00e-01	6.69e-01	-0.427219
206277_at	7.00e-01	6.70e-01	0.426787
202995_s_at	7.01e-01	6.71e-01	0.425161
217568_at	7.02e-01	6.71e-01	0.42494
215692_s_at	7.02e-01	6.71e-01	0.424901
216089_at	7.02e-01	6.71e-01	0.424776
221553_at	7.02e-01	6.71e-01	0.424546
213992_at	7.02e-01	6.72e-01	0.423855
212900_at	7.02e-01	6.72e-01	-0.423677
211741_x_at	7.03e-01	6.72e-01	0.423197
217969_at	7.03e-01	6.73e-01	-0.422843
213887_s_at	7.03e-01	6.73e-01	-0.422008
207152_at	7.04e-01	6.73e-01	-0.421816
205407_at	7.04e-01	6.74e-01	-0.421179
214828_s_at	7.04e-01	6.74e-01	0.421114
204309_at	7.04e-01	6.74e-01	0.420609
216556_x_at	7.05e-01	6.75e-01	-0.420215
215736_at	7.05e-01	6.75e-01	-0.419813
209532_at	7.05e-01	6.75e-01	0.419782
208428_at	7.05e-01	6.75e-01	-0.419665
209249_s_at	7.06e-01	6.76e-01	-0.418612
212617_at	7.06e-01	6.76e-01	-0.417832
214604_at	7.07e-01	6.77e-01	-0.416925
66053_at	7.07e-01	6.77e-01	-0.416733
214852_x_at	7.07e-01	6.77e-01	-0.416687
207445_s_at	7.07e-01	6.77e-01	0.416571
222074_at	7.07e-01	6.77e-01	-0.41638
211602_s_at	7.07e-01	6.78e-01	0.416096
204079_at	7.08e-01	6.78e-01	-0.415619
222234_s_at	7.08e-01	6.78e-01	0.415112
211911_x_at	7.09e-01	6.79e-01	0.414372
214001_x_at	7.09e-01	6.79e-01	-0.414187
201129_at	7.09e-01	6.79e-01	-0.413955
214616_at	7.09e-01	6.79e-01	0.413756
201050_at	7.10e-01	6.80e-01	-0.413059
201236_s_at	7.10e-01	6.80e-01	-0.412797
213057_at	7.10e-01	6.80e-01	0.41273
203306_s_at	7.10e-01	6.80e-01	-0.412138
222354_at	7.10e-01	6.81e-01	-0.412034
220700_at	7.11e-01	6.81e-01	-0.411023
217427_s_at	7.12e-01	6.82e-01	-0.409454

220738_s_at	7.12e-01	6.83e-01	-0.409158
220222_at	7.13e-01	6.83e-01	-0.408753
201160_s_at	7.13e-01	6.83e-01	0.408068
213260_at	7.14e-01	6.85e-01	0.40639
220028_at	7.15e-01	6.85e-01	-0.405671
202430_s_at	7.15e-01	6.85e-01	-0.405415
202077_at	7.15e-01	6.86e-01	0.405173
201188_s_at	7.15e-01	6.86e-01	0.40503
218301_at	7.16e-01	6.86e-01	0.404469
211363_s_at	7.16e-01	6.86e-01	-0.404394
213944_x_at	7.16e-01	6.86e-01	0.404392
206750_at	7.16e-01	6.86e-01	-0.404346
213701_at	7.16e-01	6.86e-01	0.404272
205736_at	7.17e-01	6.88e-01	0.402417
220087_at	7.17e-01	6.88e-01	-0.402415
219638_at	7.17e-01	6.88e-01	0.4022
213109_at	7.17e-01	6.88e-01	-0.402148
204280_at	7.18e-01	6.88e-01	0.401433
209906_at	7.18e-01	6.89e-01	0.401127
214645_at	7.18e-01	6.89e-01	-0.401095
217759_at	7.18e-01	6.89e-01	0.401091
203430_at	7.18e-01	6.89e-01	0.40059
219927_at	7.18e-01	6.89e-01	0.400169
217284_x_at	7.19e-01	6.89e-01	-0.39994
203626_s_at	7.19e-01	6.90e-01	-0.399774
201869_s_at	7.19e-01	6.90e-01	-0.399662
222142_at	7.19e-01	6.90e-01	0.399423
206779_s_at	7.19e-01	6.90e-01	-0.399413
213053_at	7.19e-01	6.90e-01	-0.399008
71933_at	7.19e-01	6.90e-01	-0.39866
216964_at	7.19e-01	6.90e-01	-0.398571
210010_s_at	7.19e-01	6.90e-01	-0.398565
203717_at	7.19e-01	6.91e-01	0.398383
221465_at	7.19e-01	6.91e-01	-0.398373
205347_s_at	7.20e-01	6.91e-01	0.398102
200908_s_at	7.22e-01	6.93e-01	0.394938
214945_at	7.22e-01	6.93e-01	0.394461
216299_s_at	7.22e-01	6.93e-01	-0.39446
201391_at	7.23e-01	6.94e-01	-0.394117
204236_at	7.23e-01	6.94e-01	-0.393453
202291_s_at	7.23e-01	6.94e-01	-0.393424
214626_s_at	7.24e-01	6.95e-01	-0.391991
201335_s_at	7.25e-01	6.96e-01	-0.391183
218035_s_at	7.25e-01	6.96e-01	0.391037

201844_s_at	7.25e-01	6.96e-01	0.390734
215370_at	7.25e-01	6.96e-01	-0.390613
219523_s_at	7.25e-01	6.96e-01	0.390455
222161_at	7.25e-01	6.96e-01	0.39042
207236_at	7.25e-01	6.97e-01	-0.389953
221326_s_at	7.26e-01	6.97e-01	0.389119
210853_at	7.27e-01	6.98e-01	0.388402
222376_at	7.27e-01	6.98e-01	-0.388085
204424_s_at	7.27e-01	6.98e-01	-0.388042
205968_at	7.27e-01	6.98e-01	0.387921
207334_s_at	7.27e-01	6.98e-01	0.387682
217230_at	7.27e-01	6.98e-01	0.387597
211320_s_at	7.27e-01	6.99e-01	0.387398
222144_at	7.27e-01	6.99e-01	-0.387223
220564_at	7.28e-01	7.00e-01	-0.386191
216009_at	7.29e-01	7.01e-01	0.384758
222125_s_at	7.29e-01	7.01e-01	-0.384381
221228_s_at	7.30e-01	7.02e-01	-0.383235
209416_s_at	7.31e-01	7.02e-01	-0.382687
215213_at	7.31e-01	7.02e-01	0.382558
202254_at	7.31e-01	7.02e-01	0.382498
40255_at	7.31e-01	7.03e-01	0.381597
44617_at	7.32e-01	7.04e-01	0.380468
218446_s_at	7.32e-01	7.04e-01	-0.380435
211605_s_at	7.32e-01	7.04e-01	0.380153
201997_s_at	7.32e-01	7.04e-01	-0.379958
211055_s_at	7.33e-01	7.04e-01	-0.379546
217477_at	7.33e-01	7.05e-01	-0.379494
37831_at	7.33e-01	7.05e-01	0.379472
206452_x_at	7.33e-01	7.05e-01	-0.379465
221046_s_at	7.33e-01	7.05e-01	-0.378952
201015_s_at	7.34e-01	7.06e-01	0.377371
204503_at	7.34e-01	7.06e-01	-0.377371
207194_s_at	7.34e-01	7.06e-01	-0.377283
222318_at	7.35e-01	7.07e-01	0.375748
202694_at	7.36e-01	7.08e-01	0.374395
203232_s_at	7.37e-01	7.09e-01	0.373736
220269_at	7.37e-01	7.09e-01	-0.373736
221875_x_at	7.38e-01	7.10e-01	0.372343
220466_at	7.38e-01	7.10e-01	0.372266
213114_at	7.38e-01	7.10e-01	-0.372123
216068_at	7.38e-01	7.10e-01	-0.372036
214409_at	7.38e-01	7.10e-01	-0.371776
207509_s_at	7.39e-01	7.11e-01	0.370889

208144_s_at	7.39e-01	7.11e-01	-0.370481
206377_at	7.40e-01	7.12e-01	-0.369839
214024_s_at	7.40e-01	7.12e-01	-0.369263
216497_at	7.40e-01	7.12e-01	-0.369127
208116_s_at	7.40e-01	7.12e-01	0.369077
204918_s_at	7.40e-01	7.12e-01	0.368767
209004_s_at	7.40e-01	7.13e-01	0.36862
213698_at	7.41e-01	7.13e-01	0.368161
215396_at	7.41e-01	7.13e-01	-0.368112
204806_x_at	7.41e-01	7.13e-01	0.368002
217807_s_at	7.41e-01	7.13e-01	-0.367413
200041_s_at	7.42e-01	7.14e-01	0.366658
205929_at	7.42e-01	7.14e-01	-0.366493
213639_s_at	7.42e-01	7.14e-01	0.366315
221153_s_at	7.42e-01	7.14e-01	-0.366137
217262_s_at	7.43e-01	7.15e-01	-0.365358
222068_s_at	7.43e-01	7.15e-01	0.365288
206994_at	7.43e-01	7.15e-01	0.364968
213741_s_at	7.43e-01	7.16e-01	0.364536
201366_at	7.43e-01	7.16e-01	0.364322
218945_at	7.43e-01	7.16e-01	0.364306
221765_at	7.43e-01	7.16e-01	0.364051
200791_s_at	7.43e-01	7.16e-01	0.364012
220355_s_at	7.44e-01	7.16e-01	0.36344
214593_at	7.44e-01	7.17e-01	-0.362798
210070_s_at	7.44e-01	7.17e-01	0.362776
201200_at	7.45e-01	7.17e-01	-0.362166
221945_at	7.45e-01	7.18e-01	0.361372
211913_s_at	7.46e-01	7.19e-01	0.360536
219498_s_at	7.46e-01	7.19e-01	-0.360502
207172_s_at	7.46e-01	7.19e-01	0.36039
205597_at	7.46e-01	7.19e-01	-0.360358
219139_s_at	7.46e-01	7.19e-01	-0.35991
214257_s_at	7.46e-01	7.19e-01	-0.359539
210383_at	7.46e-01	7.19e-01	0.359393
209938_at	7.47e-01	7.20e-01	0.358712
210630_s_at	7.47e-01	7.21e-01	0.358009
203989_x_at	7.48e-01	7.21e-01	-0.357895
219122_s_at	7.48e-01	7.21e-01	0.357616
201373_at	7.48e-01	7.21e-01	0.357568
215145_s_at	7.48e-01	7.21e-01	-0.357188
212152_x_at	7.49e-01	7.22e-01	-0.356306
201796_s_at	7.49e-01	7.22e-01	0.355995
214557_at	7.49e-01	7.22e-01	-0.355636

220667_at	7.49e-01	7.23e-01	-0.355218
210690_at	7.50e-01	7.23e-01	-0.354422
204114_at	7.50e-01	7.23e-01	-0.354392
214754_at	7.50e-01	7.23e-01	-0.354306
211842_s_at	7.50e-01	7.24e-01	-0.35388
213432_at	7.50e-01	7.24e-01	0.353801
209790_s_at	7.51e-01	7.24e-01	-0.353088
207725_at	7.51e-01	7.24e-01	-0.353047
208216_at	7.51e-01	7.24e-01	-0.352786
208506_at	7.51e-01	7.25e-01	0.352469
204526_s_at	7.52e-01	7.25e-01	0.351441
212289_at	7.52e-01	7.26e-01	0.351195
221843_s_at	7.53e-01	7.27e-01	-0.349511
202876_s_at	7.54e-01	7.27e-01	-0.349246
209766_at	7.54e-01	7.28e-01	-0.348628
209034_at	7.55e-01	7.28e-01	-0.347839
213987_s_at	7.55e-01	7.28e-01	0.347823
214252_s_at	7.55e-01	7.28e-01	-0.347409
221697_at	7.55e-01	7.29e-01	0.347152
219804_at	7.55e-01	7.29e-01	0.347009
205833_s_at	7.55e-01	7.29e-01	-0.346764
205022_s_at	7.56e-01	7.29e-01	-0.346201
213388_at	7.56e-01	7.29e-01	-0.34611
209950_s_at	7.56e-01	7.29e-01	-0.346044
217980_s_at	7.56e-01	7.29e-01	0.346026
211120_x_at	7.56e-01	7.30e-01	-0.345962
217704_x_at	7.56e-01	7.30e-01	-0.345895
210198_s_at	7.56e-01	7.30e-01	-0.345156
206518_s_at	7.56e-01	7.30e-01	0.344884
204560_at	7.57e-01	7.31e-01	-0.344503
209526_s_at	7.57e-01	7.31e-01	-0.344143
218366_x_at	7.57e-01	7.31e-01	0.344037
201679_at	7.57e-01	7.31e-01	-0.343886
204185_x_at	7.57e-01	7.31e-01	0.343728
207162_s_at	7.57e-01	7.31e-01	-0.343629
200919_at	7.58e-01	7.32e-01	-0.343167
220550_at	7.58e-01	7.32e-01	0.34305
218939_at	7.59e-01	7.33e-01	-0.341155
217308_at	7.59e-01	7.34e-01	-0.340589
206686_at	7.60e-01	7.34e-01	0.340321
212288_at	7.60e-01	7.34e-01	-0.340311
219300_s_at	7.60e-01	7.34e-01	-0.339469
200679_x_at	7.60e-01	7.35e-01	0.339121
206639_x_at	7.61e-01	7.35e-01	0.33895

214919_s_at	7.61e-01	7.35e-01	0.338545
205064_at	7.61e-01	7.35e-01	0.33849
216970_at	7.62e-01	7.37e-01	-0.336401
219457_s_at	7.62e-01	7.37e-01	-0.336321
209039_x_at	7.63e-01	7.37e-01	-0.335664
206601_s_at	7.63e-01	7.37e-01	-0.33543
219709_x_at	7.64e-01	7.38e-01	0.334698
214928_at	7.64e-01	7.38e-01	0.334679
212482_at	7.64e-01	7.38e-01	0.334367
204953_at	7.65e-01	7.39e-01	-0.333289
208615_s_at	7.66e-01	7.40e-01	-0.331833
217287_s_at	7.66e-01	7.40e-01	-0.331674
204184_s_at	7.66e-01	7.40e-01	-0.331645
217541_x_at	7.67e-01	7.41e-01	0.330515
217531_at	7.67e-01	7.41e-01	-0.33013
218212_s_at	7.67e-01	7.42e-01	0.329633
208108_s_at	7.68e-01	7.42e-01	0.328995
213863_s_at	7.68e-01	7.43e-01	0.32858
221194_s_at	7.68e-01	7.43e-01	0.328195
207447_s_at	7.69e-01	7.43e-01	-0.327638
220442_at	7.69e-01	7.43e-01	-0.327428
213779_at	7.69e-01	7.44e-01	-0.327414
211585_at	7.69e-01	7.44e-01	0.327259
217876_at	7.69e-01	7.44e-01	0.326753
201383_s_at	7.70e-01	7.44e-01	0.326219
216971_s_at	7.70e-01	7.45e-01	0.325444
205503_at	7.70e-01	7.45e-01	-0.325423
218139_s_at	7.70e-01	7.45e-01	0.325385
207925_at	7.70e-01	7.45e-01	-0.325005
215627_at	7.70e-01	7.45e-01	-0.324824
212166_at	7.71e-01	7.46e-01	-0.324356
218216_x_at	7.71e-01	7.46e-01	-0.323994
219697_at	7.71e-01	7.46e-01	-0.323554
207742_s_at	7.72e-01	7.47e-01	-0.323016
204687_at	7.72e-01	7.47e-01	-0.322532
220940_at	7.75e-01	7.50e-01	-0.319125
207859_s_at	7.75e-01	7.50e-01	0.31881
206886_x_at	7.75e-01	7.51e-01	-0.318063
208536_s_at	7.76e-01	7.51e-01	-0.317635
219656_at	7.76e-01	7.51e-01	-0.317448
212572_at	7.76e-01	7.51e-01	-0.316973
206418_at	7.77e-01	7.52e-01	0.31658
214730_s_at	7.77e-01	7.52e-01	0.31562
214066_x_at	7.77e-01	7.52e-01	0.315609

221208_s_at	7.77e-01	7.53e-01	0.315433
213392_at	7.78e-01	7.53e-01	-0.314986
202273_at	7.78e-01	7.53e-01	0.314806
203226_s_at	7.78e-01	7.53e-01	0.314628
220268_at	7.78e-01	7.53e-01	0.314625
207020_at	7.78e-01	7.53e-01	-0.314262
211524_at	7.79e-01	7.54e-01	-0.313521
213333_at	7.79e-01	7.54e-01	0.31293
219250_s_at	7.79e-01	7.55e-01	-0.31265
202132_at	7.80e-01	7.55e-01	-0.311959
203361_s_at	7.80e-01	7.56e-01	0.311291
202817_s_at	7.81e-01	7.56e-01	-0.310469
207299_s_at	7.81e-01	7.57e-01	-0.309946
203296_s_at	7.82e-01	7.57e-01	0.30929
217503_at	7.82e-01	7.58e-01	-0.308226
211879_x_at	7.83e-01	7.58e-01	0.307866
207347_at	7.83e-01	7.58e-01	-0.307771
207768_at	7.83e-01	7.59e-01	0.307246
215745_at	7.83e-01	7.59e-01	-0.30712
202744_at	7.83e-01	7.59e-01	-0.307062
214448_x_at	7.83e-01	7.59e-01	-0.306906
209874_x_at	7.84e-01	7.60e-01	0.305838
216951_at	7.84e-01	7.60e-01	-0.305659
200637_s_at	7.84e-01	7.60e-01	0.305564
212078_s_at	7.86e-01	7.61e-01	-0.303738
210774_s_at	7.86e-01	7.62e-01	0.303197
215070_x_at	7.86e-01	7.62e-01	-0.303069
214813_at	7.86e-01	7.62e-01	-0.302597
215272_at	7.87e-01	7.63e-01	-0.301251
216347_s_at	7.87e-01	7.63e-01	-0.301202
207197_at	7.88e-01	7.64e-01	-0.300887
222219_s_at	7.88e-01	7.64e-01	0.300507
208038_at	7.88e-01	7.64e-01	0.300478
220549_at	7.88e-01	7.64e-01	-0.300467
218739_at	7.88e-01	7.64e-01	0.300211
212183_at	7.88e-01	7.64e-01	0.299938
210096_at	7.89e-01	7.65e-01	-0.299279
204855_at	7.89e-01	7.65e-01	-0.298671
46665_at	7.89e-01	7.66e-01	-0.298253
219483_s_at	7.89e-01	7.66e-01	0.298182
210263_at	7.90e-01	7.66e-01	-0.297852
207276_at	7.90e-01	7.66e-01	-0.297325
204207_s_at	7.91e-01	7.67e-01	0.296608
212580_at	7.91e-01	7.67e-01	-0.296454

220957_at	7.91e-01	7.67e-01	0.295884
215457_at	7.91e-01	7.68e-01	-0.29555
206440_at	7.92e-01	7.68e-01	0.294731
217093_at	7.92e-01	7.68e-01	-0.29469
203340_s_at	7.92e-01	7.69e-01	-0.294349
211849_s_at	7.92e-01	7.69e-01	0.294032
212418_at	7.93e-01	7.69e-01	0.293408
205309_at	7.95e-01	7.71e-01	0.290821
206236_at	7.96e-01	7.73e-01	-0.289163
218657_at	7.96e-01	7.73e-01	0.28868
216927_at	7.97e-01	7.73e-01	0.288232
209140_x_at	7.97e-01	7.73e-01	0.288091
209472_at	7.98e-01	7.74e-01	-0.286908
211376_s_at	7.98e-01	7.74e-01	-0.286899
203329_at	7.98e-01	7.75e-01	0.286546
207241_at	7.99e-01	7.75e-01	-0.285587
205751_at	7.99e-01	7.75e-01	-0.285541
212911_at	7.99e-01	7.76e-01	-0.285283
222175_s_at	7.99e-01	7.76e-01	0.285261
216904_at	7.99e-01	7.76e-01	0.284336
210997_at	7.99e-01	7.76e-01	-0.284293
212014_x_at	7.99e-01	7.76e-01	0.284268
205805_s_at	8.00e-01	7.77e-01	0.284034
211480_s_at	8.01e-01	7.78e-01	-0.282282
203261_at	8.02e-01	7.79e-01	-0.281375
215091_s_at	8.02e-01	7.79e-01	-0.281361
220900_at	8.02e-01	7.79e-01	-0.281305
203324_s_at	8.02e-01	7.79e-01	0.280887
219945_at	8.03e-01	7.80e-01	-0.279898
204979_s_at	8.03e-01	7.80e-01	0.279845
212851_at	8.03e-01	7.80e-01	-0.279739
210000_s_at	8.03e-01	7.80e-01	-0.279393
217441_at	8.03e-01	7.80e-01	-0.279183
214191_at	8.03e-01	7.80e-01	0.278923
208405_s_at	8.03e-01	7.80e-01	0.278914
204546_at	8.03e-01	7.81e-01	0.278532
208998_at	8.04e-01	7.81e-01	-0.277604
201851_at	8.05e-01	7.82e-01	0.276738
216693_x_at	8.05e-01	7.83e-01	-0.27581
216737_at	8.06e-01	7.83e-01	-0.275249
221131_at	8.07e-01	7.84e-01	-0.274045
215339_at	8.07e-01	7.84e-01	-0.273864
218416_s_at	8.07e-01	7.85e-01	-0.273152
217910_x_at	8.09e-01	7.86e-01	0.271572

64371_at	8.09e-01	7.86e-01	-0.271118
219823_at	8.10e-01	7.87e-01	-0.270299
203811_s_at	8.10e-01	7.88e-01	-0.269066
202329_at	8.11e-01	7.89e-01	-0.268392
218694_at	8.11e-01	7.89e-01	-0.26778
214772_at	8.12e-01	7.89e-01	-0.267378
213676_at	8.12e-01	7.89e-01	-0.267285
220146_at	8.12e-01	7.89e-01	-0.267181
31837_at	8.12e-01	7.90e-01	-0.266992
208050_s_at	8.13e-01	7.90e-01	0.266004
209151_x_at	8.13e-01	7.91e-01	0.265635
218231_at	8.13e-01	7.91e-01	-0.264964
210256_s_at	8.14e-01	7.92e-01	0.264261
215521_at	8.14e-01	7.92e-01	0.264146
219411_at	8.14e-01	7.92e-01	-0.263871
204869_at	8.14e-01	7.92e-01	0.263795
220488_s_at	8.15e-01	7.93e-01	-0.262894
35160_at	8.15e-01	7.93e-01	0.262649
207704_s_at	8.15e-01	7.93e-01	-0.2626
202599_s_at	8.15e-01	7.93e-01	0.262528
209217_s_at	8.15e-01	7.93e-01	0.262453
207811_at	8.15e-01	7.93e-01	0.262419
221490_at	8.15e-01	7.93e-01	-0.262127
206579_at	8.15e-01	7.93e-01	-0.262038
208504_x_at	8.16e-01	7.94e-01	-0.261395
206342_x_at	8.16e-01	7.94e-01	0.261291
202840_at	8.16e-01	7.95e-01	0.260617
206186_at	8.17e-01	7.95e-01	-0.259758
219962_at	8.17e-01	7.95e-01	-0.259678
204697_s_at	8.17e-01	7.95e-01	-0.259406
215802_at	8.17e-01	7.96e-01	-0.259032
220915_s_at	8.18e-01	7.96e-01	0.258696
212325_at	8.18e-01	7.96e-01	0.258432
214874_at	8.19e-01	7.97e-01	0.257313
221061_at	8.19e-01	7.97e-01	-0.257289
213635_s_at	8.19e-01	7.97e-01	-0.257257
222198_at	8.20e-01	7.98e-01	-0.255926
207777_s_at	8.20e-01	7.98e-01	-0.255582
216170_at	8.20e-01	7.99e-01	0.255376
212565_at	8.21e-01	7.99e-01	0.254211
59437_at	8.21e-01	7.99e-01	-0.254162
206148_at	8.21e-01	8.00e-01	0.253634
203678_at	8.22e-01	8.01e-01	-0.252237
216577_at	8.24e-01	8.02e-01	-0.250328

205867_at	8.24e-01	8.03e-01	-0.249448
217632_at	8.24e-01	8.03e-01	-0.249444
207979_s_at	8.25e-01	8.03e-01	0.249268
204205_at	8.25e-01	8.03e-01	-0.249003
211738_x_at	8.25e-01	8.04e-01	-0.248418
219245_s_at	8.25e-01	8.04e-01	-0.248402
219984_s_at	8.25e-01	8.04e-01	-0.248001
215512_at	8.25e-01	8.04e-01	-0.247919
208464_at	8.26e-01	8.05e-01	-0.24727
221784_at	8.27e-01	8.06e-01	0.245913
204747_at	8.27e-01	8.06e-01	-0.24571
219866_at	8.27e-01	8.06e-01	0.245683
209996_x_at	8.28e-01	8.07e-01	0.244772
202086_at	8.29e-01	8.08e-01	0.242541
219736_at	8.31e-01	8.10e-01	-0.240894
1487_at	8.31e-01	8.10e-01	0.240412
209117_at	8.31e-01	8.10e-01	0.239981
209883_at	8.31e-01	8.11e-01	-0.239883
209951_s_at	8.31e-01	8.11e-01	-0.239719
217338_at	8.32e-01	8.11e-01	0.239494
212586_at	8.32e-01	8.11e-01	0.239449
213161_at	8.32e-01	8.11e-01	-0.239049
221643_s_at	8.32e-01	8.11e-01	-0.238869
221417_x_at	8.32e-01	8.11e-01	-0.238785
204734_at	8.33e-01	8.12e-01	-0.238004
219606_at	8.33e-01	8.12e-01	0.237787
204712_at	8.33e-01	8.12e-01	0.237483
216894_x_at	8.33e-01	8.12e-01	0.237382
217595_at	8.33e-01	8.13e-01	-0.236867
210445_at	8.35e-01	8.14e-01	-0.235352
202659_at	8.35e-01	8.14e-01	-0.235268
204877_s_at	8.35e-01	8.14e-01	-0.234796
213903_s_at	8.36e-01	8.16e-01	-0.233376
207054_at	8.36e-01	8.16e-01	-0.232737
37117_at	8.37e-01	8.16e-01	0.232582
210803_at	8.37e-01	8.17e-01	-0.232026
212808_at	8.37e-01	8.17e-01	-0.23168
204596_s_at	8.38e-01	8.17e-01	-0.23093
214045_at	8.38e-01	8.18e-01	-0.230617
219587_at	8.38e-01	8.18e-01	0.230414
214752_x_at	8.38e-01	8.18e-01	-0.230349
210089_s_at	8.38e-01	8.18e-01	0.23033
208572_at	8.38e-01	8.18e-01	-0.230212
202674_s_at	8.38e-01	8.18e-01	0.229733

219876_s_at	8.39e-01	8.19e-01	0.229551
211213_at	8.39e-01	8.19e-01	0.229539
203955_at	8.39e-01	8.19e-01	0.229482
47571_at	8.39e-01	8.19e-01	0.229289
203752_s_at	8.39e-01	8.19e-01	-0.22865
37953_s_at	8.39e-01	8.19e-01	0.228528
216835_s_at	8.39e-01	8.19e-01	0.228365
45653_at	8.40e-01	8.20e-01	-0.227454
214795_at	8.40e-01	8.21e-01	0.226846
206536_s_at	8.41e-01	8.21e-01	-0.226578
204980_at	8.41e-01	8.21e-01	-0.226155
205561_at	8.41e-01	8.21e-01	0.226109
207631_at	8.41e-01	8.21e-01	-0.226098
201203_s_at	8.41e-01	8.22e-01	-0.225663
219172_at	8.41e-01	8.22e-01	-0.225523
213845_at	8.42e-01	8.22e-01	-0.224553
201392_s_at	8.43e-01	8.23e-01	-0.223383
212477_at	8.43e-01	8.23e-01	0.223328
217368_at	8.43e-01	8.24e-01	-0.222776
211622_s_at	8.43e-01	8.24e-01	0.222588
212971_at	8.44e-01	8.24e-01	0.222141
212906_at	8.44e-01	8.25e-01	0.221293
208729_x_at	8.44e-01	8.25e-01	0.221149
212052_s_at	8.45e-01	8.25e-01	-0.220621
208345_s_at	8.45e-01	8.26e-01	-0.220081
220167_s_at	8.45e-01	8.26e-01	0.220011
213714_at	8.45e-01	8.26e-01	-0.219962
220079_s_at	8.46e-01	8.27e-01	0.21876
216549_s_at	8.46e-01	8.27e-01	-0.218342
202135_s_at	8.46e-01	8.27e-01	0.218252
217425_at	8.46e-01	8.27e-01	-0.218165
202616_s_at	8.46e-01	8.27e-01	-0.218105
218980_at	8.46e-01	8.27e-01	0.218068
202101_s_at	8.47e-01	8.28e-01	-0.217335
220946_s_at	8.47e-01	8.28e-01	0.216851
206528_at	8.48e-01	8.29e-01	-0.215946
216418_at	8.48e-01	8.29e-01	0.215882
212004_at	8.48e-01	8.29e-01	0.215842
206179_s_at	8.48e-01	8.30e-01	0.21546
215955_x_at	8.49e-01	8.30e-01	0.21483
202548_s_at	8.49e-01	8.30e-01	-0.214734
212818_s_at	8.49e-01	8.30e-01	-0.214362
207145_at	8.49e-01	8.30e-01	-0.214274
201206_s_at	8.49e-01	8.30e-01	-0.214268

221919_at	8.50e-01	8.31e-01	0.213602
205684_s_at	8.50e-01	8.31e-01	-0.213371
208316_s_at	8.50e-01	8.31e-01	0.213108
221545_x_at	8.50e-01	8.31e-01	0.213095
217780_at	8.50e-01	8.31e-01	-0.212971
214827_at	8.50e-01	8.32e-01	0.212628
210252_s_at	8.50e-01	8.32e-01	-0.212611
209204_at	8.51e-01	8.32e-01	0.212061
204211_x_at	8.51e-01	8.32e-01	0.211997
212525_s_at	8.51e-01	8.32e-01	-0.211807
215312_at	8.51e-01	8.32e-01	0.211803
203704_s_at	8.51e-01	8.32e-01	0.211706
209788_s_at	8.51e-01	8.33e-01	0.210636
215974_at	8.52e-01	8.34e-01	-0.209781
219886_at	8.52e-01	8.34e-01	-0.209383
60815_at	8.53e-01	8.35e-01	-0.208917
205467_at	8.54e-01	8.36e-01	-0.207562
40489_at	8.55e-01	8.37e-01	-0.206395
201315_x_at	8.55e-01	8.37e-01	0.205829
211530_x_at	8.55e-01	8.37e-01	-0.205711
204592_at	8.55e-01	8.37e-01	0.205695
211276_at	8.56e-01	8.38e-01	0.204779
46270_at	8.56e-01	8.38e-01	0.204111
216871_at	8.56e-01	8.39e-01	-0.203837
219068_x_at	8.57e-01	8.39e-01	0.203637
220058_at	8.57e-01	8.39e-01	-0.203457
217474_at	8.57e-01	8.39e-01	0.203076
206171_at	8.58e-01	8.40e-01	-0.202102
216002_at	8.58e-01	8.40e-01	0.20192
208168_s_at	8.58e-01	8.40e-01	-0.201744
207337_at	8.58e-01	8.40e-01	0.201657
217317_s_at	8.58e-01	8.40e-01	-0.201521
210437_at	8.58e-01	8.40e-01	0.201519
208595_s_at	8.59e-01	8.41e-01	-0.200775
216004_s_at	8.59e-01	8.41e-01	-0.200692
211676_s_at	8.59e-01	8.41e-01	0.200518
211341_at	8.59e-01	8.41e-01	0.200491
210736_x_at	8.59e-01	8.41e-01	0.20046
204260_at	8.59e-01	8.41e-01	0.200416
214729_at	8.59e-01	8.42e-01	0.200083
205705_at	8.59e-01	8.42e-01	0.20006
222110_at	8.59e-01	8.42e-01	-0.199471
217007_s_at	8.59e-01	8.42e-01	0.199326
58780_s_at	8.59e-01	8.42e-01	-0.199281

204945_at	8.59e-01	8.42e-01	-0.199265
211361_s_at	8.59e-01	8.42e-01	-0.198942
217993_s_at	8.60e-01	8.43e-01	-0.198385
220516_at	8.60e-01	8.43e-01	0.197724
205979_at	8.61e-01	8.44e-01	0.197412
207594_s_at	8.61e-01	8.44e-01	-0.197138
219907_at	8.61e-01	8.44e-01	-0.196548
222051_s_at	8.61e-01	8.44e-01	-0.19645
219624_at	8.62e-01	8.45e-01	0.195574
219214_s_at	8.62e-01	8.45e-01	-0.19534
203033_x_at	8.62e-01	8.45e-01	-0.195201
209873_s_at	8.62e-01	8.45e-01	0.195108
219034_at	8.62e-01	8.46e-01	0.194977
214815_at	8.62e-01	8.46e-01	-0.194665
209010_s_at	8.63e-01	8.46e-01	0.194362
44563_at	8.63e-01	8.46e-01	-0.193989
205023_at	8.64e-01	8.47e-01	-0.193015
211011_at	8.64e-01	8.48e-01	-0.192051
204635_at	8.65e-01	8.48e-01	-0.1916
216536_at	8.66e-01	8.50e-01	0.189696
206422_at	8.68e-01	8.51e-01	0.187926
221903_s_at	8.68e-01	8.51e-01	-0.187652
207884_at	8.68e-01	8.52e-01	0.187057
215581_s_at	8.68e-01	8.52e-01	0.186888
218317_x_at	8.68e-01	8.52e-01	-0.186576
216263_s_at	8.68e-01	8.52e-01	-0.186488
209598_at	8.69e-01	8.52e-01	-0.186135
220340_at	8.70e-01	8.54e-01	-0.184251
204015_s_at	8.70e-01	8.54e-01	-0.183924
221800_s_at	8.70e-01	8.54e-01	-0.183779
213267_at	8.71e-01	8.55e-01	0.18296
205927_s_at	8.72e-01	8.56e-01	0.182007
215164_at	8.72e-01	8.56e-01	0.181589
209863_s_at	8.72e-01	8.56e-01	0.181543
210845_s_at	8.73e-01	8.57e-01	-0.180418
205692_s_at	8.74e-01	8.58e-01	-0.179621
204770_at	8.74e-01	8.58e-01	0.179239
207720_at	8.74e-01	8.58e-01	0.17909
206902_s_at	8.74e-01	8.58e-01	0.178654
220321_s_at	8.75e-01	8.59e-01	0.177723
215744_at	8.76e-01	8.60e-01	-0.176954
204002_s_at	8.76e-01	8.60e-01	0.17668
209862_s_at	8.76e-01	8.60e-01	0.176354
221365_at	8.77e-01	8.61e-01	0.175545

209378_s_at	8.77e-01	8.61e-01	0.175271
206306_at	8.77e-01	8.61e-01	-0.174877
221927_s_at	8.77e-01	8.62e-01	0.174244
205630_at	8.77e-01	8.62e-01	-0.174111
217799_x_at	8.77e-01	8.62e-01	-0.174084
213383_at	8.78e-01	8.62e-01	-0.173432
217874_at	8.80e-01	8.64e-01	-0.171429
204209_at	8.80e-01	8.64e-01	0.171153
207022_s_at	8.80e-01	8.64e-01	0.170906
211783_s_at	8.80e-01	8.64e-01	0.170855
206670_s_at	8.80e-01	8.64e-01	0.170836
214209_s_at	8.80e-01	8.65e-01	0.170228
209193_at	8.80e-01	8.65e-01	-0.170012
222380_s_at	8.81e-01	8.65e-01	0.169492
221038_at	8.81e-01	8.66e-01	0.16935
218707_at	8.81e-01	8.66e-01	-0.16905
209427_at	8.81e-01	8.66e-01	0.168909
215816_at	8.81e-01	8.66e-01	-0.168633
210151_s_at	8.82e-01	8.66e-01	0.168327
206351_s_at	8.82e-01	8.67e-01	0.167373
211412_at	8.82e-01	8.67e-01	-0.167008
206135_at	8.83e-01	8.68e-01	0.166762
215169_at	8.83e-01	8.68e-01	-0.166737
209791_at	8.83e-01	8.68e-01	-0.166582
211391_s_at	8.83e-01	8.68e-01	0.166341
219865_at	8.83e-01	8.68e-01	0.165751
216797_at	8.83e-01	8.68e-01	0.165724
201086_x_at	8.83e-01	8.69e-01	-0.165614
219064_at	8.83e-01	8.69e-01	0.165565
212607_at	8.83e-01	8.69e-01	-0.165447
206023_at	8.84e-01	8.69e-01	-0.165067
202436_s_at	8.84e-01	8.69e-01	0.164734
206398_s_at	8.84e-01	8.70e-01	-0.163908
219582_at	8.85e-01	8.70e-01	0.16376
208115_x_at	8.86e-01	8.71e-01	0.162302
206921_at	8.86e-01	8.71e-01	0.162062
203826_s_at	8.86e-01	8.72e-01	-0.161603
221004_s_at	8.86e-01	8.72e-01	0.161576
206429_at	8.86e-01	8.72e-01	-0.161434
208233_at	8.87e-01	8.72e-01	-0.160912
206314_at	8.87e-01	8.72e-01	0.160794
220329_s_at	8.87e-01	8.73e-01	-0.160303
204967_at	8.87e-01	8.73e-01	-0.160249
216108_at	8.87e-01	8.73e-01	-0.159971

210474_s_at	8.87e-01	8.73e-01	-0.159854
221759_at	8.88e-01	8.74e-01	-0.159149
220754_at	8.88e-01	8.74e-01	0.158371
219911_s_at	8.89e-01	8.75e-01	0.157785
220805_at	8.89e-01	8.75e-01	0.15763
203386_at	8.90e-01	8.76e-01	0.156752
219665_at	8.90e-01	8.76e-01	-0.156213
205377_s_at	8.90e-01	8.76e-01	-0.155965
37950_at	8.90e-01	8.76e-01	0.155609
200951_s_at	8.90e-01	8.76e-01	-0.155544
217129_at	8.91e-01	8.77e-01	0.155038
205459_s_at	8.91e-01	8.78e-01	0.154185
207543_s_at	8.91e-01	8.78e-01	-0.154181
221506_s_at	8.92e-01	8.78e-01	-0.153641
207385_at	8.92e-01	8.78e-01	-0.153154
217810_x_at	8.92e-01	8.79e-01	0.152797
201431_s_at	8.92e-01	8.79e-01	-0.152633
205738_s_at	8.93e-01	8.79e-01	0.152015
211554_s_at	8.93e-01	8.80e-01	0.151614
204345_at	8.93e-01	8.80e-01	-0.151493
217331_at	8.93e-01	8.80e-01	0.15145
222032_s_at	8.93e-01	8.80e-01	-0.151176
206202_at	8.94e-01	8.81e-01	0.150376
212887_at	8.95e-01	8.81e-01	-0.14923
216922_x_at	8.95e-01	8.82e-01	0.148928
212401_s_at	8.95e-01	8.82e-01	-0.148861
213286_at	8.95e-01	8.82e-01	-0.148816
220154_at	8.95e-01	8.82e-01	0.148192
207302_at	8.96e-01	8.83e-01	0.146978
215717_s_at	8.97e-01	8.83e-01	-0.146677
216461_at	8.97e-01	8.83e-01	0.146626
207848_at	8.97e-01	8.84e-01	-0.146344
220375_s_at	8.97e-01	8.84e-01	0.146341
204685_s_at	8.98e-01	8.85e-01	0.145248
214343_s_at	8.98e-01	8.85e-01	-0.145041
216133_at	8.98e-01	8.85e-01	-0.144991
207317_s_at	8.98e-01	8.85e-01	0.14495
202566_s_at	8.98e-01	8.85e-01	0.144949
212479_s_at	8.98e-01	8.85e-01	0.144893
213667_at	8.98e-01	8.85e-01	0.144585
37145_at	8.99e-01	8.86e-01	0.143019
219806_s_at	8.99e-01	8.87e-01	-0.142651
210139_s_at	9.00e-01	8.87e-01	-0.142086
211012_s_at	9.00e-01	8.87e-01	0.141885

221728_x_at	9.00e-01	8.88e-01	-0.141482
216698_x_at	9.00e-01	8.88e-01	-0.14133
217447_at	9.00e-01	8.88e-01	-0.141029
201158_at	9.01e-01	8.89e-01	0.140279
209485_s_at	9.01e-01	8.89e-01	0.140255
214117_s_at	9.02e-01	8.89e-01	-0.139148
215281_x_at	9.02e-01	8.89e-01	0.13906
215152_at	9.02e-01	8.90e-01	-0.138892
205337_at	9.02e-01	8.90e-01	0.138831
213423_x_at	9.03e-01	8.90e-01	-0.13786
215888_at	9.03e-01	8.91e-01	-0.137075
214218_s_at	9.04e-01	8.91e-01	-0.136622
202650_s_at	9.04e-01	8.92e-01	0.136242
218278_at	9.04e-01	8.92e-01	0.135926
210066_s_at	9.05e-01	8.93e-01	0.135087
218651_s_at	9.05e-01	8.93e-01	0.134552
215250_at	9.06e-01	8.94e-01	0.132917
213725_x_at	9.07e-01	8.95e-01	-0.132672
202075_s_at	9.07e-01	8.95e-01	0.132638
207003_at	9.07e-01	8.95e-01	-0.132278
209351_at	9.07e-01	8.95e-01	-0.131883
205829_at	9.07e-01	8.95e-01	-0.13146
220363_s_at	9.07e-01	8.96e-01	-0.131351
215274_at	9.09e-01	8.97e-01	0.129743
206104_at	9.09e-01	8.97e-01	0.129423
205835_s_at	9.09e-01	8.98e-01	-0.128858
214623_at	9.09e-01	8.98e-01	-0.128851
220655_at	9.09e-01	8.98e-01	0.128695
213707_s_at	9.09e-01	8.98e-01	-0.128627
220526_s_at	9.10e-01	8.98e-01	0.128012
205588_s_at	9.11e-01	8.99e-01	-0.126974
206376_at	9.11e-01	8.99e-01	0.126545
207624_s_at	9.11e-01	8.99e-01	-0.126383
209794_at	9.11e-01	9.00e-01	0.125799
216109_at	9.12e-01	9.00e-01	-0.125381
213790_at	9.12e-01	9.01e-01	0.125046
216522_at	9.12e-01	9.01e-01	-0.124811
212189_s_at	9.12e-01	9.01e-01	0.124639
202182_at	9.12e-01	9.01e-01	0.124495
222283_at	9.12e-01	9.01e-01	0.124241
201843_s_at	9.13e-01	9.02e-01	-0.123768
210514_x_at	9.13e-01	9.02e-01	0.123484
220903_at	9.13e-01	9.02e-01	-0.123248
218759_at	9.13e-01	9.02e-01	0.123081

214392_at	9.13e-01	9.02e-01	0.122983
218686_s_at	9.15e-01	9.04e-01	0.120749
216361_s_at	9.15e-01	9.04e-01	-0.12019
211811_s_at	9.16e-01	9.06e-01	0.118777
209410_s_at	9.17e-01	9.06e-01	-0.118441
221071_at	9.17e-01	9.06e-01	-0.118368
217334_at	9.17e-01	9.06e-01	0.117853
207931_s_at	9.17e-01	9.06e-01	-0.117564
212043_at	9.17e-01	9.07e-01	0.11717
202910_s_at	9.18e-01	9.07e-01	-0.116395
216096_s_at	9.19e-01	9.08e-01	-0.115247
203123_s_at	9.19e-01	9.09e-01	0.114765
220333_at	9.19e-01	9.09e-01	0.114646
218379_at	9.20e-01	9.09e-01	-0.113905
210347_s_at	9.20e-01	9.09e-01	-0.113783
37652_at	9.20e-01	9.10e-01	0.11366
220481_at	9.21e-01	9.10e-01	-0.112686
219537_x_at	9.22e-01	9.11e-01	0.111586
203793_x_at	9.22e-01	9.11e-01	-0.111298
201019_s_at	9.22e-01	9.12e-01	-0.11116
206114_at	9.22e-01	9.12e-01	0.111007
217659_at	9.22e-01	9.12e-01	0.11057
202242_at	9.22e-01	9.12e-01	0.110354
204052_s_at	9.23e-01	9.13e-01	0.109261
204463_s_at	9.23e-01	9.13e-01	0.109235
217605_at	9.23e-01	9.13e-01	0.108946
211781_x_at	9.23e-01	9.13e-01	0.108853
214483_s_at	9.24e-01	9.14e-01	0.108658
211880_x_at	9.24e-01	9.14e-01	0.107603
205809_s_at	9.25e-01	9.15e-01	-0.106982
41113_at	9.25e-01	9.15e-01	-0.10666
214311_at	9.25e-01	9.16e-01	-0.106035
221862_at	9.26e-01	9.16e-01	-0.105609
219101_x_at	9.26e-01	9.16e-01	-0.104944
64438_at	9.27e-01	9.17e-01	0.104404
210133_at	9.27e-01	9.17e-01	0.104114
221702_s_at	9.27e-01	9.18e-01	0.103434
222343_at	9.28e-01	9.18e-01	-0.103126
210999_s_at	9.28e-01	9.18e-01	-0.102987
206254_at	9.28e-01	9.18e-01	-0.102794
214240_at	9.28e-01	9.18e-01	0.102713
204666_s_at	9.28e-01	9.19e-01	-0.10218
217204_at	9.28e-01	9.19e-01	-0.101952
201161_s_at	9.28e-01	9.19e-01	-0.101709

204470_at	9.29e-01	9.19e-01	0.101449
215390_at	9.29e-01	9.19e-01	-0.101308
210423_s_at	9.29e-01	9.19e-01	-0.101213
219094_at	9.29e-01	9.20e-01	-0.100954
212681_at	9.30e-01	9.21e-01	0.099351
204548_at	9.30e-01	9.21e-01	-0.098803
210557_x_at	9.31e-01	9.21e-01	-0.0986
203227_s_at	9.31e-01	9.22e-01	0.098193
208770_s_at	9.32e-01	9.23e-01	-0.097212
213384_x_at	9.32e-01	9.23e-01	-0.097129
207750_at	9.32e-01	9.23e-01	-0.097096
206710_s_at	9.32e-01	9.23e-01	0.096673
216141_at	9.32e-01	9.23e-01	0.09661
217234_s_at	9.32e-01	9.23e-01	0.096155
215667_x_at	9.33e-01	9.24e-01	0.095699
216989_at	9.33e-01	9.24e-01	0.095423
215188_at	9.33e-01	9.25e-01	-0.09473
221090_s_at	9.33e-01	9.25e-01	0.094708
218951_s_at	9.34e-01	9.25e-01	-0.093998
204138_s_at	9.34e-01	9.25e-01	0.093877
215240_at	9.34e-01	9.26e-01	0.093094
206500_s_at	9.34e-01	9.26e-01	-0.092963
216408_at	9.36e-01	9.28e-01	-0.090793
204805_s_at	9.36e-01	9.28e-01	0.090528
204076_at	9.36e-01	9.28e-01	0.090427
221227_x_at	9.37e-01	9.29e-01	-0.089423
212385_at	9.38e-01	9.30e-01	0.08819
211545_at	9.38e-01	9.30e-01	-0.08806
212063_at	9.38e-01	9.30e-01	0.087905
217426_at	9.39e-01	9.31e-01	0.08647
207798_s_at	9.39e-01	9.31e-01	0.086299
221716_s_at	9.40e-01	9.32e-01	0.085662
201070_x_at	9.40e-01	9.32e-01	-0.085555
221847_at	9.40e-01	9.32e-01	0.085372
207847_s_at	9.40e-01	9.32e-01	-0.084796
202062_s_at	9.41e-01	9.33e-01	0.084562
209928_s_at	9.41e-01	9.33e-01	-0.084152
201102_s_at	9.41e-01	9.33e-01	-0.08389
211323_s_at	9.41e-01	9.33e-01	-0.08363
216410_at	9.41e-01	9.33e-01	0.083508
200650_s_at	9.42e-01	9.34e-01	-0.082659
216014_s_at	9.42e-01	9.34e-01	0.082478
204270_at	9.42e-01	9.34e-01	-0.082377
204515_at	9.42e-01	9.34e-01	0.082349

201005_at	9.43e-01	9.36e-01	0.080737
203864_s_at	9.44e-01	9.36e-01	-0.080299
204145_at	9.45e-01	9.37e-01	0.078507
205619_s_at	9.46e-01	9.38e-01	-0.077205
220506_at	9.46e-01	9.39e-01	0.077018
209514_s_at	9.46e-01	9.39e-01	0.076674
205926_at	9.46e-01	9.39e-01	0.0765
204275_at	9.47e-01	9.40e-01	-0.075568
220067_at	9.47e-01	9.40e-01	0.07531
212776_s_at	9.48e-01	9.41e-01	0.073842
211613_s_at	9.49e-01	9.42e-01	0.072844
203071_at	9.50e-01	9.43e-01	-0.071963
51158_at	9.50e-01	9.43e-01	0.071666
202390_s_at	9.50e-01	9.43e-01	0.0715
219400_at	9.50e-01	9.43e-01	-0.071282
200777_s_at	9.50e-01	9.43e-01	-0.071028
214259_s_at	9.51e-01	9.44e-01	-0.070733
210741_at	9.51e-01	9.44e-01	-0.070311
207518_at	9.51e-01	9.44e-01	-0.070176
205599_at	9.51e-01	9.44e-01	0.069896
202438_x_at	9.52e-01	9.45e-01	0.069388
205902_at	9.52e-01	9.46e-01	-0.068252
203953_s_at	9.53e-01	9.46e-01	-0.068059
217451_at	9.53e-01	9.46e-01	-0.067227
211279_at	9.53e-01	9.47e-01	-0.067019
202812_at	9.53e-01	9.47e-01	-0.066911
222025_s_at	9.54e-01	9.47e-01	0.066263
212794_s_at	9.54e-01	9.47e-01	0.066215
214759_at	9.54e-01	9.47e-01	0.065907
216722_at	9.54e-01	9.48e-01	0.065315
219747_at	9.55e-01	9.48e-01	0.06499
201654_s_at	9.55e-01	9.49e-01	0.064268
215033_at	9.55e-01	9.49e-01	-0.064078
221304_at	9.56e-01	9.49e-01	-0.063618
218897_at	9.56e-01	9.49e-01	0.063582
202439_s_at	9.56e-01	9.49e-01	-0.063568
201159_s_at	9.56e-01	9.50e-01	-0.063051
204914_s_at	9.56e-01	9.50e-01	0.062705
221398_at	9.56e-01	9.50e-01	0.062477
206416_at	9.57e-01	9.51e-01	-0.061512
216073_at	9.57e-01	9.51e-01	-0.06137
201153_s_at	9.57e-01	9.51e-01	-0.061304
206159_at	9.57e-01	9.51e-01	-0.060906
206993_at	9.57e-01	9.52e-01	-0.06082

216923_at	9.58e-01	9.52e-01	-0.060102
210765_at	9.58e-01	9.52e-01	0.060008
211655_at	9.59e-01	9.54e-01	-0.058234
209328_x_at	9.60e-01	9.54e-01	0.057922
203202_at	9.60e-01	9.54e-01	-0.057867
201207_at	9.60e-01	9.54e-01	-0.05783
216302_at	9.60e-01	9.54e-01	-0.057434
200776_s_at	9.61e-01	9.55e-01	0.056158
211496_s_at	9.61e-01	9.55e-01	-0.056084
217094_s_at	9.61e-01	9.56e-01	-0.05583
218279_s_at	9.61e-01	9.56e-01	0.055455
218571_s_at	9.62e-01	9.56e-01	0.054979
204153_s_at	9.62e-01	9.56e-01	-0.054601
213708_s_at	9.63e-01	9.58e-01	-0.053269
209051_s_at	9.63e-01	9.58e-01	-0.053053
215303_at	9.63e-01	9.58e-01	0.052477
210616_s_at	9.64e-01	9.59e-01	-0.052048
217922_at	9.64e-01	9.59e-01	0.051275
206512_at	9.65e-01	9.59e-01	0.05086
212963_at	9.65e-01	9.60e-01	-0.049853
207653_at	9.66e-01	9.61e-01	-0.049431
204633_s_at	9.66e-01	9.61e-01	0.049138
212002_at	9.67e-01	9.62e-01	-0.04752
210127_at	9.68e-01	9.63e-01	0.046637
211364_at	9.68e-01	9.63e-01	0.046545
220063_at	9.68e-01	9.63e-01	-0.046286
205870_at	9.69e-01	9.64e-01	-0.045294
206165_s_at	9.69e-01	9.64e-01	-0.045202
205485_at	9.69e-01	9.64e-01	-0.045081
210707_x_at	9.70e-01	9.66e-01	-0.043243
215657_at	9.70e-01	9.66e-01	-0.042973
215494_at	9.70e-01	9.66e-01	0.042932
209550_at	9.70e-01	9.66e-01	-0.04293
219113_x_at	9.70e-01	9.66e-01	0.042827
215577_at	9.71e-01	9.66e-01	-0.042504
219382_at	9.71e-01	9.67e-01	-0.041598
213471_at	9.71e-01	9.67e-01	0.041487
206801_at	9.72e-01	9.68e-01	0.040597
204557_s_at	9.72e-01	9.68e-01	0.04022
217091_at	9.72e-01	9.68e-01	0.039879
222038_s_at	9.73e-01	9.68e-01	-0.039575
210171_s_at	9.73e-01	9.69e-01	-0.039117
205360_at	9.74e-01	9.69e-01	0.038362
207775_at	9.74e-01	9.70e-01	0.038252

34449_at	9.74e-01	9.70e-01	0.038149
215082_at	9.74e-01	9.70e-01	-0.037584
202512_s_at	9.74e-01	9.70e-01	-0.037134
220663_at	9.74e-01	9.70e-01	0.037064
40359_at	9.74e-01	9.71e-01	-0.036912
204151_x_at	9.75e-01	9.71e-01	0.035994
213117_at	9.75e-01	9.71e-01	-0.035862
216511_s_at	9.75e-01	9.72e-01	-0.035741
217472_at	9.76e-01	9.72e-01	0.035018
219856_at	9.76e-01	9.72e-01	-0.03462
208033_s_at	9.76e-01	9.72e-01	0.034524
216382_s_at	9.76e-01	9.73e-01	-0.034137
209040_s_at	9.77e-01	9.73e-01	-0.033767
210692_s_at	9.77e-01	9.73e-01	-0.033546
216351_x_at	9.77e-01	9.74e-01	0.032716
221207_s_at	9.78e-01	9.74e-01	-0.032359
216948_at	9.78e-01	9.74e-01	0.032103
220007_at	9.78e-01	9.74e-01	0.032004
AFFX-M27830_5_at	9.78e-01	9.75e-01	0.031349
220510_at	9.78e-01	9.75e-01	-0.031037
216677_at	9.78e-01	9.75e-01	-0.031035
215813_s_at	9.79e-01	9.75e-01	0.030839
221089_at	9.79e-01	9.75e-01	0.030741
221806_s_at	9.79e-01	9.76e-01	0.030338
204497_at	9.79e-01	9.76e-01	-0.030206
211906_s_at	9.79e-01	9.76e-01	0.030205
220683_at	9.79e-01	9.76e-01	-0.030168
207080_s_at	9.79e-01	9.77e-01	-0.029329
216205_s_at	9.80e-01	9.77e-01	-0.029143
201211_s_at	9.81e-01	9.78e-01	0.027488
205150_s_at	9.81e-01	9.78e-01	0.027167
210837_s_at	9.81e-01	9.79e-01	-0.026719
207356_at	9.81e-01	9.79e-01	-0.02666
48580_at	9.81e-01	9.79e-01	-0.026573
219075_at	9.82e-01	9.79e-01	0.026184
205171_at	9.82e-01	9.79e-01	0.02603
218304_s_at	9.82e-01	9.80e-01	-0.025568
220410_s_at	9.82e-01	9.80e-01	-0.025336
207269_at	9.83e-01	9.80e-01	-0.024549
214601_at	9.84e-01	9.81e-01	0.023391
218225_at	9.85e-01	9.82e-01	0.022307
215259_s_at	9.85e-01	9.82e-01	-0.02211
207917_at	9.85e-01	9.83e-01	0.021213
207818_s_at	9.85e-01	9.83e-01	-0.021087

221854_at	9.85e-01	9.83e-01	-0.020947
207286_at	9.86e-01	9.83e-01	-0.020755
203892_at	9.86e-01	9.84e-01	-0.020456
204175_at	9.86e-01	9.84e-01	0.020411
201372_s_at	9.86e-01	9.84e-01	-0.020322
215465_at	9.86e-01	9.84e-01	-0.020256
207559_s_at	9.87e-01	9.85e-01	0.019076
213693_s_at	9.87e-01	9.85e-01	0.018283
213417_at	9.87e-01	9.86e-01	-0.018128
202192_s_at	9.87e-01	9.86e-01	0.017886
209848_s_at	9.88e-01	9.86e-01	-0.017712
204935_at	9.88e-01	9.86e-01	-0.017548
219328_at	9.88e-01	9.86e-01	0.01724
214726_x_at	9.89e-01	9.87e-01	-0.016238
207295_at	9.89e-01	9.88e-01	0.015649
220638_s_at	9.89e-01	9.88e-01	0.015578
222381_at	9.89e-01	9.88e-01	-0.015506
214393_at	9.89e-01	9.88e-01	0.015047
203507_at	9.90e-01	9.89e-01	0.014248
217590_s_at	9.90e-01	9.89e-01	-0.013544
206112_at	9.91e-01	9.89e-01	-0.013363
212394_at	9.91e-01	9.90e-01	0.012295
218015_s_at	9.92e-01	9.91e-01	0.011752
222114_x_at	9.93e-01	9.91e-01	0.010758
208625_s_at	9.93e-01	9.92e-01	-0.010421
206992_s_at	9.93e-01	9.92e-01	-0.010347
214365_at	9.93e-01	9.92e-01	-0.010303
215723_s_at	9.93e-01	9.92e-01	0.01013
220064_at	9.93e-01	9.92e-01	-0.009904
210824_at	9.93e-01	9.92e-01	0.009675
203792_x_at	9.94e-01	9.93e-01	0.00818
216450_x_at	9.94e-01	9.94e-01	0.008141
205747_at	9.95e-01	9.94e-01	-0.007662
210586_x_at	9.95e-01	9.94e-01	0.007651
212703_at	9.95e-01	9.94e-01	0.007024
217763_s_at	9.95e-01	9.95e-01	-0.006759
217478_s_at	9.96e-01	9.95e-01	0.006115
219412_at	9.96e-01	9.95e-01	-0.005768
203791_at	9.96e-01	9.96e-01	0.005626
204332_s_at	9.96e-01	9.96e-01	-0.004938
203635_at	9.97e-01	9.97e-01	0.004212
206661_at	9.98e-01	9.98e-01	0.002998
203533_s_at	9.99e-01	9.98e-01	-0.001988
219115_s_at	9.99e-01	9.99e-01	-0.001252

219906_at	9.99e-01	9.99e-01	-0.00113
215337_at	9.99e-01	9.99e-01	-0.000829
220189_s_at	9.99e-01	9.99e-01	0.000714
219445_at	9.99e-01	9.99e-01	-0.000708
204106_at	1.00	1.00	0.000281

B	logFC		Gene.symb	Gene.title
	362.879.878	-23.778.707	ECM1	extracellular matrix protein 1
	34.578.494	-28.528.569	VIPR1	vasoactive intestinal peptide receptor 1
	343.494.483	-34.560.507	CXCL14	C-X-C motif chemokine ligand 14
	313.993.392	-19.174.259	STAB2	stabilin 2
	301.706.257	-33.611.909	FCN3	ficolin 3
	29.453.942	-42.427.123	CYP1A2	cytochrome P450 family 1 subfamily A member
	287.810.129	-30.160.896	LCAT	lecithin-cholesterol acyltransferase
	268.301.497	25.248.093	PTTG1	pituitary tumor-transforming 1
	264.875.855	-26.596.619	FCN2	ficolin 2
	264.723.498	-22.517.329	CLEC1B	C-type lectin domain family 1 member B
	26.355.623	-17.095.248	PTH1R	parathyroid hormone 1 receptor
	263.023.759	-28.138.157	ESR1	estrogen receptor 1
	262.258.101	24.761.852	KPNA2	karyopherin subunit alpha 2
	258.013.415	-51.937.146	CYP1A2	cytochrome P450 family 1 subfamily A member
	257.228.371	-21.290.636	CLEC4M	C-type lectin domain family 4 member M
	256.380.143	-38.117.662	NAT2	N-acetyltransferase 2
	252.236.575	15.696.213	CCT3	chaperonin containing TCP1 subunit 3
	251.788.701	30.396.575	ASPM	abnormal spindle microtubule assembly
	251.312.705	23.251.662	KPNA2	karyopherin subunit alpha 2
	243.714.309	22.191.296	RACGAP1	Rac GTPase activating protein 1
	242.644.147	-35.383.684	APOF	apolipoprotein F
	241.395.063	-3.740.732	FOS	Fos proto-oncogene, AP-1 transcription factor s
	241.167.121	25.289.544	CAP2	CAP, adenylate cyclase-associated protein, 2 (y
	238.293.013	25.271.482	CCNB1	cyclin B1
	237.593.004	24.569.889	PRC1	protein regulator of cytokinesis 1
	236.285.167	31.064.036	RRM2	ribonucleotide reductase regulatory subunit M2
	23.568.967	-27.328.441	LPA	lipoprotein(a)
	233.429.016	15.662.906	SNRPE	small nuclear ribonucleoprotein polypeptide E
	23.243.889	-34.279.337	CYP39A1	cytochrome P450 family 39 subfamily A membe
	230.915.863	-34.378.083	CYP2B6	cytochrome P450 family 2 subfamily B member
	22.879.457	-25.976.698	SRD5A2	steroid 5 alpha-reductase 2
	226.753.443	-54.455.838	HAMP	hepcidin antimicrobial peptide
	226.711.436	23.915.396	CENPF	centromere protein F
	225.381.144	24.395.974	NUSAP1	nucleolar and spindle associated protein 1
	224.305.191	-27.635.205	EGR1	early growth response 1
	223.753.791	-39.817.543	MT1F	metallothionein 1F
	222.251.368	28.818.546	RRM2	ribonucleotide reductase regulatory subunit M2
	22.209.572	-18.450.706	KDM8	lysine demethylase 8
	221.883.432	-29.406.049	DNASE1L3	deoxyribonuclease 1 like 3
	220.844.675	24.259.378	CDKN3	cyclin dependent kinase inhibitor 3
	219.547.035	27.911.067	KIAA0101	KIAA0101
	218.925.047	12.987.958	GMPS	guanine monophosphate synthase
	218.777.149	30.471.649	TOP2A	topoisomerase (DNA) II alpha

218.204.452		12.236.904	PSMD4	proteasome 26S subunit, non-ATPase 4
217.938.872		21.971.025	RFC4	replication factor C subunit 4
217.747.315		16.027.544	ACLY	ATP citrate lyase
216.896.448		27.190.528	TOP2A	topoisomerase (DNA) II alpha
215.832.382		24.739.191	CDK1	cyclin dependent kinase 1
215.500.456		-22.896.354	CYP2C9	cytochrome P450 family 2 subfamily C member
214.988.595		19.986.327	CNIH4	cornichon family AMPA receptor auxiliary protei
214.814.352		-24.412.541	GSTZ1	glutathione S-transferase zeta 1
213.588.386		-14.482.859	CFP	complement factor properdin
213.430.564		17.048.095	SNRPD1	small nuclear ribonucleoprotein D1 polypeptide
213.346.608		15.104.567	NME1	NME/NM23 nucleoside diphosphate kinase 1
213.026.823		-31.494.372	CXCL12	C-X-C motif chemokine ligand 12
212.873.405		-3.410.099		
212.228.443		-1.459.558	RDH5	retinol dehydrogenase 5
212.172.115		15.998.052	SAE1	SUMO1 activating enzyme subunit 1
211.452.163		18.497.507	ILF2	interleukin enhancer binding factor 2
210.308.275		-25.601.846	SRPX	sushi repeat containing protein, X-linked
210.139.558		24.708.027	ZWINT	ZW10 interacting kinetochore protein
210.027.469		-24.127.972	CD5L	CD5 molecule like
209.595.852	0.7811014		PPIA	peptidylprolyl isomerase A
209.249.094		-39.660.539	ADH1C///A	alcohol dehydrogenase 1C (class I), gamma po
208.515.584		22.745.921	AURKA	aurora kinase A
208.469.113		23.760.279	CKS2	CDC28 protein kinase regulatory subunit 2
208.242.131		-242.088	CXCL12	C-X-C motif chemokine ligand 12
208.196.023		-35.014.466	MT1F	metallothionein 1F
207.085.072	0.7918552		PPIA	peptidylprolyl isomerase A
206.513.855		1.246.077	MRPL9	mitochondrial ribosomal protein L9
206.285.513		23.747.657	GINS1	GINS complex subunit 1
205.985.985		-22.909.416	CDHR2	cadherin related family member 2
205.557.264		-22.980.544	ZGPAT	zinc finger CCCH-type and G-patch domain cor
205.172.985		-20.042.677	CLEC4M	C-type lectin domain family 4 member M
204.963.228		-24.082.645	ZGPAT	zinc finger CCCH-type and G-patch domain cor
204.378.456		-19.595.113	ADRA1A	adrenoceptor alpha 1A
203.294.149		16.912.478	SUCO	SUN domain containing ossification factor
202.701.525		-26.145.673	DCN	decorin
201.895.934		22.268.759	GMNN	geminin, DNA replication inhibitor
201.401.476		-35.734.196	GLYAT	glycine-N-acyltransferase
201.360.577		11.795.008	NUP205	nucleoporin 205
201.146.057		-20.435.279	IGFALS	insulin like growth factor binding protein acid la
199.952.461		-18.285.264	PER1	period circadian clock 1
199.248.811		-28.748.556	EGR1	early growth response 1
1.989.665		11.290.443	PARP1	poly(ADP-ribose) polymerase 1
198.446.478		-19.773.008	FAM149A	family with sequence similarity 149 member A
198.218.895		11.750.803	PSMD4	proteasome 26S subunit, non-ATPase 4

198.061.677	0.8062268		PPIA	peptidylprolyl isomerase A
197.998.687	0.8051826		PPIA	peptidylprolyl isomerase A
197.777.393		-19.398.886	N4BP2L1	NEDD4 binding protein 2 like 1
197.315.059		-41.172.573	MT1M	metallothionein 1M
196.356.435		15.225.495	TUBG1	tubulin gamma 1
19.630.695	0.786746		PPIA	peptidylprolyl isomerase A
195.375.286		17.848.055	FEN1	flap structure-specific endonuclease 1
1.953.359		15.947.393	SAC3D1	SAC3 domain containing 1
194.990.959		14.153.675	BOLA2B///I	bolA family member 2B///bolA family member 2
194.849.364		11.946.682	DAP3	death associated protein 3
193.930.465		13.704.504	NELFE	negative elongation factor complex member E
193.781.159		-10.734.235	TBXA2R	thromboxane A2 receptor
193.256.288		-1.334.364	PER1	period circadian clock 1
193.212.623		-22.482.056	F11	coagulation factor XI
191.849.018		48.149.283	GPC3	glypican 3
191.635.886		-15.865.134	MAN1C1	mannosidase alpha class 1C member 1
191.256.414		-24.917.137	KMO	kynurenine 3-monooxygenase (kynurenine 3-hy
191.233.301		-34.848.571	MT1G	metallothionein 1G
191.078.239		16.835.997	H2AFX	H2A histone family member X
190.941.328		14.495.798	SF3B4	splicing factor 3b subunit 4
190.817.213		20.510.312	CAP2	CAP, adenylate cyclase-associated protein, 2 (y
190.741.493		-14.805.859	CYP2C9	cytochrome P450 family 2 subfamily C member
189.938.448		22.728.242	DTL	denticleless E3 ubiquitin protein ligase homolo
189.640.215		15.033.938	HEATR1	HEAT repeat containing 1
189.458.124		16.514.614	TP53BP2	tumor protein p53 binding protein 2
189.069.981		-27.430.002	CYP2A13//,	cytochrome P450 family 2 subfamily A member
188.936.703		-15.949.649	NDRG2	NDRG family member 2
188.337.879		-12.977.109	N4BP2L1	NEDD4 binding protein 2 like 1
188.226.787		12.862.101	HEATR1	HEAT repeat containing 1
188.215.763		1.349.009	TUBA1B	tubulin alpha 1b
188.156.018		19.011.083	SMC4	structural maintenance of chromosomes 4
187.874.246		13.412.178	TUBA1B	tubulin alpha 1b
187.797.238		-1.253.682	ACADS	acyl-CoA dehydrogenase, C-2 to C-3 short chai
187.781.166		-25.238.635	CYP4A11	cytochrome P450 family 4 subfamily A member
186.804.304	0.9188203		ZNF207	zinc finger protein 207
185.946.003		19.022.809	SMC4	structural maintenance of chromosomes 4
185.826.628		1.337.988	TUBA1B	tubulin alpha 1b
185.610.182		-19.633.044	GCDH	glutaryl-CoA dehydrogenase
185.498.003		13.154.984	CPSF6	cleavage and polyadenylation specific factor 6
185.247.354		19.463.142	ENAH	enabled homolog (Drosophila)
185.080.488		-16.480.376	FYN	FYN proto-oncogene, Src family tyrosine kinase
184.418.736		-31.877.969	MT1X	metallothionein 1X
184.403.172		1.490.718	UBAP2L	ubiquitin associated protein 2 like
184.308.553		20.617.808	ECT2	epithelial cell transforming 2

184.225.699		-13.105.818	PAMR1	peptidase domain containing associated with m
183.759.336		-2.061.869	RCL1	RNA terminal phosphate cyclase like 1
183.647.523		-30.449.763	MT1X	metallothionein 1X
183.586.332	0.9180015		PSMB4	proteasome subunit beta 4
18.334.227		-15.619.808	SYNE1	spectrin repeat containing nuclear envelope pro
183.255.407		15.300.736	CCT6A	chaperonin containing TCP1 subunit 6A
183.224.307		-22.740.288	CRHBP	corticotropin releasing hormone binding protein
183.221.123		-30.369.844	HGFAC	HGF activator
183.220.523		1.373.946	PEA15	phosphoprotein enriched in astrocytes 15
183.217.512		20.870.718	MCM6	minichromosome maintenance complex comp
183.035.848		14.559.409	NUP37	nucleoporin 37
182.752.974		-17.733.536	CD1D	CD1d molecule
182.553.715		13.096.309	TUBA1B	tubulin alpha 1b
182.447.056		12.356.585	PSMD4	proteasome 26S subunit, non-ATPase 4
181.605.495		12.373.196	VPS72	vacuolar protein sorting 72 homolog
181.480.059		17.703.519	UBE2C///P/	ubiquitin conjugating enzyme E2 C///p21 (RAC1
180.750.874		14.970.435	RRP15	ribosomal RNA processing 15 homolog
180.680.106		-22.251.262		
180.344.253		-34.260.356	GLS2	glutaminase 2
180.256.011		11.067.293	SNRPD2	small nuclear ribonucleoprotein D2 polypeptide
180.249.938		-30.873.774	MT1H	metallothionein 1H
180.206.264		-32.834.728	C7	complement component 7
180.032.064		-35.900.407	CYP2B7P	cytochrome P450 family 2 subfamily B member
17.951.391		-19.406.775	ASS1	argininosuccinate synthase 1
17.937.239		14.495.963	SNRPB	small nuclear ribonucleoprotein polypeptides B
179.194.174		-13.940.705	CHST4	carbohydrate sulfotransferase 4
179.075.453		28.922.436	SQLE	squalene epoxidase
179.069.759		-13.922.649	N4BP2L1	NEDD4 binding protein 2 like 1
178.805.936		-22.039.843	GPD1	glycerol-3-phosphate dehydrogenase 1
178.300.675		19.586.359	HMMR	hyaluronan mediated motility receptor
178.087.507		158.045	CKS1B	CDC28 protein kinase regulatory subunit 1B
177.913.556		-28.462.194	MT1M	metallothionein 1M
177.608.601		-22.784.395	ACSM3	acyl-CoA synthetase medium-chain family mem
17.692.855		1.696.732	KIF20A	kinesin family member 20A
176.917.391		-34.073.749	GYS2	glycogen synthase 2
176.862.334		-25.412.918		
176.463.714		13.470.059	SSR2	signal sequence receptor subunit 2
176.355.383		12.751.083	IRAK1	interleukin 1 receptor associated kinase 1
17.614.041		-26.084.214	MT1HL1	metallothionein 1H-like 1
175.677.433		-1.290.033	SMAD6	SMAD family member 6
175.626.653		-35.072.483	CYP2A6	cytochrome P450 family 2 subfamily A member
175.545.302		-20.047.838	BDH2	3-hydroxybutyrate dehydrogenase, type 2
17.538.598		32.985.348	ACSL4	acyl-CoA synthetase long-chain family member
175.365.182		11.815.054	EPRS	glutamyl-prolyl-tRNA synthetase

175.246.849	14.394.931	UCK2	uridine-cytidine kinase 2
175.184.547	-24.901.255	MT2A	metallothionein 2A
175.173.883	-19.540.923	STEAP3	STEAP3 metalloreductase
174.907.883	-29.736.167	LOC65416	similar to cytochrome P450, family 4, subfamily
174.811.689	-15.762.486	GCGR	glucagon receptor
174.686.827	14.919.363	TBCE	tubulin folding cofactor E
174.500.198	-28.311.959	CYP3A4	cytochrome P450 family 3 subfamily A member
174.167.294	12.668.803	RBM34	RNA binding motif protein 34
174.123.458	-24.886.241	KLKB1	kallikrein B1
17.378.136	13.052.665	TUBA1B	tubulin alpha 1b
173.746.374	-33.088.527	DCN	decorin
173.660.004	-18.136.968	CPEB3	cytoplasmic polyadenylation element binding p
173.630.823	-30.733.747	GBA3	glucosylceramidase beta 3 (gene/pseudogene)
173.444.423	-30.688.017	DCN	decorin
172.905.977	-38.066.381	CYP2A6	cytochrome P450 family 2 subfamily A member
172.849.068	17.325.323	HN1	hematological and neurological expressed 1
172.641.609	17.692.648	EZH2	enhancer of zeste 2 polycomb repressive comp
172.625.298	-27.472.481	ID1	inhibitor of DNA binding 1, HLH protein
172.553.764	-16.114.997	CD4	CD4 molecule
172.373.058	-17.609.461	GCDH	glutaryl-CoA dehydrogenase
172.193.749	17.971.316	NEK2	NIMA related kinase 2
172.065.393	12.881.131	GMFB	glia maturation factor beta
171.395.376	1.422.217	CBX1	chromobox 1
17.084.758	13.917.493	NPM1	nucleophosmin
170.719.602	15.913.096	MCM3	minichromosome maintenance complex comp
17.071.564	11.769.274	MRPL9	mitochondrial ribosomal protein L9
16.949.131	-11.694.044		
169.140.628	14.767.185	PRKDC	protein kinase, DNA-activated, catalytic polype
169.071.781	12.814.807	TUBA1C	tubulin alpha 1c
169.036.907	-12.888.787	C1QTNF1	C1q and tumor necrosis factor related protein 1
168.993.593	16.901.301	MELK	maternal embryonic leucine zipper kinase
168.733.588	-31.825.283	MT1E	metallothionein 1E
168.720.866	-34.877.662	HAO2	hydroxyacid oxidase 2
168.343.701	12.178.721	VPS45	vacuolar protein sorting 45 homolog
168.062.053	-19.987.131	MARCO	macrophage receptor with collagenous structu
167.929.925	12.629.857	PIGC	phosphatidylinositol glycan anchor biosynthesi
167.909.259	-27.212.759	KCNN2	potassium calcium-activated channel subfamil
167.669.244	11.248.767	CSE1L	chromosome segregation 1 like
167.656.741	10.611.566	CKAP5	cytoskeleton associated protein 5
167.051.381	14.683.989	RFX5	regulatory factor X5
16.649.656	-40.644.673	CYP2A6	cytochrome P450 family 2 subfamily A member
166.306.212	10.281.101	PSMB4	proteasome subunit beta 4
166.268.783	0.9753459	NME1-NME	NME1-NME2 readthrough///NME/NM23 nucleo
166.176.747	-394.863	CYP2A6	cytochrome P450 family 2 subfamily A member

166.053.613	12.405.224	SUB1	SUB1 homolog, transcriptional regulator
165.940.229	10.804.052	POLR3C	RNA polymerase III subunit C
165.796.978	17.782.826	BIRC5	baculoviral IAP repeat containing 5
165.558.814	14.983.945	MEIS3///HS	Meis homeobox 3///heat shock protein family B
165.348.945	-32.009.159	DCN	decorin
165.281.078	-18.697.643	CCL15///C	C-C motif chemokine ligand 15///C-C motif che
165.194.918	-10.639.547	ADAMTSL2	ADAMTS like 2
165.129.416	-18.156.427	GCKR	glucokinase (hexokinase 4) regulator
165.061.805	13.009.371	NUP107	nucleoporin 107
165.035.504	-22.364.003	FAM13A	family with sequence similarity 13 member A
164.897.837	-24.862.969	ANXA10	annexin A10
16.487.301	10.109.637	LOC65282	26S protease regulatory subunit 6B-like///prote
164.797.598	15.132.252	PRIM1	primase (DNA) subunit 1
164.545.561	13.997.076	PSMD10	proteasome 26S subunit, non-ATPase 10
164.083.427	14.604.739	ITGA6	integrin subunit alpha 6
164.068.479	-12.393.722	STARD5	StAR related lipid transfer domain containing 5
163.779.563	1.277.834	TUBA1C	tubulin alpha 1c
163.676.845	12.063.057	SNRPB	small nuclear ribonucleoprotein polypeptides E
163.575.224	11.341.966	HSPA4	heat shock protein family A (Hsp70) member 4
163.378.905	1.390.104	ISG20L2	interferon stimulated exonuclease gene 20 like
163.356.185	-18.383.211	SORL1	sortilin related receptor 1
163.132.725	19.268.251	CDC20	cell division cycle 20
162.986.712	11.273.731	ACBD3	acyl-CoA binding domain containing 3
162.789.971	-2.152.338		
162.587.735	-18.260.224	GRAMD1C	GRAM domain containing 1C
162.458.137	-24.583.508	FBP1	fructose-bisphosphatase 1
162.447.304	11.148.948	CCT5	chaperonin containing TCP1 subunit 5
162.447.008	11.009.247	UBE2Q1	ubiquitin conjugating enzyme E2 Q1
162.192.264	13.498.619	NUP133	nucleoporin 133
162.050.436	0.7500189	PRICKLE4	prickle planar cell polarity protein 4
161.769.461	-14.922.804	DPT	dermatopontin
161.761.535	-2.759.941	IGF1	insulin like growth factor 1
161.597.199	0.7718859	RPN2	ribophorin II
161.535.067	-28.688.504	RDH16	retinol dehydrogenase 16 (all-trans)
161.447.205	1.884.353	NCAPG	non-SMC condensin I complex subunit G
161.421.795	17.351.767	BUB1B	BUB1 mitotic checkpoint serine/threonine kina
161.400.807	0.9327896	DYNLL1	dynein light chain LC8-type 1
161.336.838	11.606.153	PSMD14	proteasome 26S subunit, non-ATPase 14
161.304.496	-20.617.134	MAN1C1	mannosidase alpha class 1C member 1
161.301.672	-19.134.836	CIDEB	cell death-inducing DFFA-like effector b
161.110.416	-18.952.657	RCAN1	regulator of calcineurin 1
16.109.696	10.366.825	BTRC///TPF	beta-transducin repeat containing E3 ubiquitin
161.022.307	-40.838.041	SLC22A1	solute carrier family 22 member 1
160.977.223	14.935.459	CKAP2	cytoskeleton associated protein 2

160.931.297		12.036.983	CSE1L	chromosome segregation 1 like
160.768.573		-16.512.416	ECHDC2	enoyl-CoA hydratase domain containing 2
160.519.838		21.905.189	PLCB1	phospholipase C beta 1
16.042.467		-17.628.216	ASPA	aspartoacylase
160.140.344	0.8919285		SNRPC	small nuclear ribonucleoprotein polypeptide C
160.080.034		17.769.157	AKR1C3	aldo-keto reductase family 1, member C3
15.968.008		-17.535.486	ST3GAL6	ST3 beta-galactoside alpha-2,3-sialyltransferas
159.606.787		11.652.277	HSP90AB1	heat shock protein 90 alpha family class B mem
15.950.638		-16.986.568	OLFML3	olfactomedin like 3
159.268.944		-1.669.556	HGF	hepatocyte growth factor
159.251.089		-25.352.268	FAM13A	family with sequence similarity 13 member A
158.941.449		10.331.681	CCT6A	chaperonin containing TCP1 subunit 6A
158.940.331		14.084.078	CACYBP	calcyclin binding protein
158.921.595	0.7941803		RPN2	ribophorin II
158.540.425		17.229.995	LPGAT1	lysophosphatidylglycerol acyltransferase 1
158.463.709		11.978.585	OLA1	Obg-like ATPase 1
158.205.872		16.014.285	SLC12A4	solute carrier family 12 member 4
15.818.655		-2.994.982	IGF1	insulin like growth factor 1
158.097.808		16.551.453	CDK1	cyclin dependent kinase 1
157.869.696		13.019.664	GOLPH3L	golgi phosphoprotein 3 like
157.824.281		-21.020.539	RND3	Rho family GTPase 3
157.660.307		-1.523.617	ACAA1	acetyl-CoA acyltransferase 1
157.588.319		-17.180.493	ST3GAL6	ST3 beta-galactoside alpha-2,3-sialyltransferas
157.453.266		11.619.903	LOC73105!	similar to misato///misato 1, mitochondrial dist
157.335.789		13.379.397	UTP18	UTP18, small subunit processome component
156.576.395		27.700.676	ROBO1	roundabout guidance receptor 1
156.494.085	0.8487466		CANX	calnexin
156.489.996		10.715.111	DTYMK	deoxythymidylate kinase
156.412.439		-1.579.119	ACAA1	acetyl-CoA acyltransferase 1
156.367.287	0.9573554		CANT1	calcium activated nucleotidase 1
156.344.421		-14.389.704	FAM13A	family with sequence similarity 13 member A
156.235.119	0.9985387		NVL	nuclear VCP-like
156.047.862		1.270.463	DBF4	DBF4 zinc finger
156.034.971		-29.338.538	CYP4A11	cytochrome P450 family 4 subfamily A member
156.008.379		20.022.974	CENPU	centromere protein U
155.956.115	0.6847505		RPL30	ribosomal protein L30
155.955.348	-0.827193		NGFR	nerve growth factor receptor
155.734.177		14.703.867	H2AFZ	H2A histone family member Z
15.540.871		13.777.517	GBAP1///GI	glucosylceramidase beta pseudogene 1///gluco
155.352.148		-12.108.537	SLC6A13	solute carrier family 6 member 13
155.078.009		10.770.321	SCAMP3	secretory carrier membrane protein 3
15.505.359		-2.407.899	TAT	tyrosine aminotransferase
155.003.539		16.867.548	TPX2	TPX2, microtubule nucleation factor
15.485.498		11.371.365	ADSL	adenylosuccinate lyase

154.708.739		10.298.677	SCNM1	sodium channel modifier 1
1.545.556		-24.598.203	TAT	tyrosine aminotransferase
15.443.348		-17.241.361	ID2B///ID2	inhibitor of DNA binding 2B, HLH protein (pseuc
154.369.197		109.178	EPRS	glutamyl-prolyl-tRNA synthetase
154.329.834		10.537.932	H2AFV	H2A histone family member V
154.312.967	0.9583715		CTNNA1	catenin alpha 1
154.310.482		-16.530.971	FAM149A	family with sequence similarity 149 member A
154.049.556		-22.835.397	FAM134B	family with sequence similarity 134 member B
153.918.261		12.093.959	PSME3	proteasome activator subunit 3
153.653.715		-11.899.047	LPAL2	lipoprotein(a) like 2, pseudogene
153.651.117		16.914.358	AURKA	aurora kinase A
153.390.133		12.447.055	RRM1	ribonucleotide reductase catalytic subunit M1
153.359.823		16.984.746	ATAD2	ATPase family, AAA domain containing 2
153.202.812		-22.156.839	MFAP3L	microfibrillar associated protein 3 like
15.316.228		15.959.858	PBK	PDZ binding kinase
153.158.652		16.634.466	NEU1	neuraminidase 1 (lysosomal sialidase)
153.150.614		-19.380.466		
152.970.405		-13.434.565	CBR4	carbonyl reductase 4
152.912.994		-19.414.241	FOLH1B	folate hydrolase 1B
152.891.319		12.818.159	MSH2	mutS homolog 2
15.267.776	0.894012		RHEB	Ras homolog enriched in brain
152.655.897	0.8657168		XRCC6	X-ray repair cross complementing 6
152.480.484		-25.385.437	C8A	complement component 8 alpha subunit
152.246.878		14.592.629	PFDN4	prefoldin subunit 4
152.000.136		14.302.924	KIF4A	kinesin family member 4A
15.190.743		23.052.112	MDK	midkine (neurite growth-promoting factor 2)
151.839.445		-20.676.904	PROZ	protein Z, vitamin K dependent plasma glycopro
151.541.539		-22.544.026	C8orf4	chromosome 8 open reading frame 4
151.540.879		10.605.756	GNPAT	glyceronephosphate O-acyltransferase
151.526.973		12.184.136	TCEB1	transcription elongation factor B subunit 1
151.371.335		-22.898.977	CYP2C19	cytochrome P450 family 2 subfamily C member
151.152.523		12.453.028	STMN1	stathmin 1
151.014.213		10.898.459	DUSP12	dual specificity phosphatase 12
150.932.389		13.919.657	DESI2	desumoylating isopeptidase 2
150.761.116		14.922.982	GMFB	glia maturation factor beta
150.759.237		12.220.699	INTS8	integrator complex subunit 8
150.432.335		16.479.274	MCM2	minichromosome maintenance complex comp
150.381.892		12.771.172	MAPK9	mitogen-activated protein kinase 9
150.357.326		-18.386.677	CYP2B6	cytochrome P450 family 2 subfamily B member
150.248.425		10.740.279	RABIF	RAB interacting factor
150.246.086	0.9625375		SNX27	sorting nexin family member 27
150.167.993		11.144.279	MAPK1	mitogen-activated protein kinase 1
149.984.736		16.881.095	TTK	TTK protein kinase
149.870.909		-18.108.269	ETFDH	electron transfer flavoprotein dehydrogenase

149.773.461		-18.238.112	C1R	complement C1r subcomponent
149.764.146	0.8026828		SNRPG	small nuclear ribonucleoprotein polypeptide G
149.353.471		-34.091.037	AKR1D1	aldo-keto reductase family 1, member D1
149.343.624		-19.713.536	IGFBP3	insulin like growth factor binding protein 3
149.290.346	0.8284156		MEA1	male-enhanced antigen 1
148.989.498	-0.9409175		DBH	dopamine beta-hydroxylase
148.872.104		10.997.209	METTL5	methyltransferase like 5
148.578.864		12.445.628	EEF1E1	eukaryotic translation elongation factor 1 epsilo
148.365.696		13.828.105	DDX39A	DEAD-box helicase 39A
14.811.752		-14.714.482	ZFP36	ZFP36 ring finger protein
148.081.784		-16.812.536	RNF125	ring finger protein 125
148.070.479	0.9473502		GTPBP4	GTP binding protein 4
147.868.719		-12.963.056	NPY1R	neuropeptide Y receptor Y1
147.747.984		11.639.625	NOL7	nucleolar protein 7
147.710.963		-14.977.336	ALDH2	aldehyde dehydrogenase 2 family (mitochondri
147.636.696		-18.518.345	MAT1A	methionine adenosyltransferase 1A
147.538.414		13.248.529	RBM12	RNA binding motif protein 12
146.824.366		12.705.554	SLBP	stem-loop binding protein
146.696.326		13.231.452		
146.539.057		-16.818.172	ACACB	acetyl-CoA carboxylase beta
146.514.541		-11.862.932	AR	androgen receptor
146.389.842		-17.002.726	ETFDH	electron transfer flavoprotein dehydrogenase
146.360.824		10.966.919	NUP155	nucleoporin 155
145.987.009		-1.272.168	FAHD2A	fumarylacetoacetate hydrolase domain contair
145.940.883		-18.382.842	FAM134B	family with sequence similarity 134 member B
145.912.142		-15.779.261	SLC23A2	solute carrier family 23 member 2
145.870.921		-10.166.724	SPATA6L	spermatogenesis associated 6 like
145.841.973		-18.440.519	ACACB	acetyl-CoA carboxylase beta
145.809.154		11.819.378	FOXM1	forkhead box M1
14.570.723		10.932.352	GARS	glycyl-tRNA synthetase
145.625.556		-15.478.887	HAAO	3-hydroxyanthranilate 3,4-dioxygenase
145.514.396		11.695.821	LYRM4	LYR motif containing 4
145.386.281		12.745.307	CSE1L	chromosome segregation 1 like
145.269.521		-14.909.817	CSAD	cysteine sulfinic acid decarboxylase
145.117.569		14.119.944	CCNB2	cyclin B2
145.104.303		-12.526.379	PDLIM5	PDZ and LIM domain 5
144.965.341		13.536.648	MAPRE1	microtubule associated protein RP/EB family m
14.491.691		-15.391.595	C1RL	complement C1r subcomponent like
144.909.923		-16.498.599	GCH1	GTP cyclohydrolase 1
144.897.942	-0.919718		PDE2A	phosphodiesterase 2A
144.808.706		13.077.909	CACYBP	calcyclin binding protein
144.530.965		-27.745.838	GLYAT	glycine-N-acyltransferase
144.110.396		-14.640.651	SIGIRR	single Ig and TIR domain containing
144.021.877	0.9501308		NSF	N-ethylmaleimide sensitive factor, vesicle fusir

143.792.748	0.9365285		TMEM147	transmembrane protein 147
143.754.826		-26.168.947	SLC27A5	solute carrier family 27 member 5
143.719.232	-0.7321666		LILRB5	leukocyte immunoglobulin like receptor B5
143.603.574		13.898.164	RFC3	replication factor C subunit 3
143.582.508		10.540.291	COG2	component of oligomeric golgi complex 2
143.436.302	0.902603		SUB1	SUB1 homolog, transcriptional regulator
143.387.269		-22.650.377	MASP2	mannan binding lectin serine peptidase 2
14.335.232		12.584.239	PUS7	pseudouridylate synthase 7 (putative)
143.237.767		-23.709.465	AKR7A3	aldo-keto reductase family 7 member A3
143.205.681	0.7024747		CFL1	cofilin 1
143.123.809		-16.065.601	GABARAPL:	GABA type A receptor associated protein like 3	
143.077.927		-10.551.608	GGT5	gamma-glutamyltransferase 5
142.791.825		12.793.063	CDK4	cyclin dependent kinase 4
142.665.368		-37.475.597	C9	complement component 9
142.652.817		-15.393.348	SIGIRR	single Ig and TIR domain containing
142.566.848		-20.103	LY6E	lymphocyte antigen 6 complex, locus E
142.440.065		11.712.775	NOL7	nucleolar protein 7
142.218.084	-0.5842374		ANKRD55	ankyrin repeat domain 55
142.203.565		11.785.398	DIEXF	digestive organ expansion factor homolog (zebr
142.135.671		-21.321.566	OGDHL	oxoglutarate dehydrogenase-like
142.042.893		12.028.671	TTC13	tetratricopeptide repeat domain 13
1.420.344		12.095.646	TMEM106C	transmembrane protein 106C
142.017.275		-10.585.602	KAZN	kazrin, periplakin interacting protein
14.193.015		-154.096	CDC37L1	cell division cycle 37 like 1
141.791.893		11.918.995	STIP1	stress induced phosphoprotein 1
141.760.103		1.752.106	HMGB2	high mobility group box 2
141.710.808		12.099.114	HNRNPA3/	heterogeneous nuclear ribonucleoprotein A3///
14.164.393		-23.015.007	IGFBP3	insulin like growth factor binding protein 3
141.554.288		-29.404.357	ABCA8	ATP binding cassette subfamily A member 8
141.467.627		23.164.477	LAPTM4B	lysosomal protein transmembrane 4 beta
141.396.167		16.570.981	CDKN2C	cyclin dependent kinase inhibitor 2C
141.296.862		-27.091.007	GHR	growth hormone receptor
141.274.061		-23.174.981	KMO	kynurenine 3-monooxygenase (kynurenine 3-hy
141.119.016		-22.471.555	KMO	kynurenine 3-monooxygenase (kynurenine 3-hy
140.698.109		12.366.534	VBP1	VHL binding protein 1
140.621.521	0.9065769		KIF18B	kinesin family member 18B
140.542.175		-24.210.939	NR1I2	nuclear receptor subfamily 1 group I member 2
140.473.315		106.138	SMARCC1	SWI/SNF related, matrix associated, actin depe
140.384.923		-19.305.821	ACACB	acetyl-CoA carboxylase beta
14.026.178	0.9264761		TMEM267	transmembrane protein 267
140.252.864		-15.732.135	MTHFD1	methylenetetrahydrofolate dehydrogenase, cyc
139.834.851	0.9419059		H2AFY	H2A histone family member Y
139.813.475		-27.138.644	CXCL2	C-X-C motif chemokine ligand 2
139.780.987		19.888.814	TYMS	thymidylate synthetase

139.777.369		14.559.376	MCM5	minichromosome maintenance complex comp
139.687.163	0.9480098		DROSHA	drosha ribonuclease III
139.681.266		14.611.809	FDPS	farnesyl diphosphate synthase
139.628.335		-14.086.087	GABARAPL	GABA type A receptor associated protein like 1
139.443.571		11.542.589	UBAP2L	ubiquitin associated protein 2 like
139.415.969		19.726.999	NUSAP1	nucleolar and spindle associated protein 1
139.394.525	0.5449908		RPL38	ribosomal protein L38
13.934.854	0.9476709		SPDL1	spindle apparatus coiled-coil protein 1
139.278.638		11.010.546	UBA2	ubiquitin like modifier activating enzyme 2
139.147.515		14.110.539	H2AFZ	H2A histone family member Z
138.979.934		-10.957.012	LHX2	LIM homeobox 2
13.881.017		1.387.714	HSPA4	heat shock protein family A (Hsp70) member 4
138.773.545		10.325.898	CDC23	cell division cycle 23
138.727.038		18.054.359	ANXA2	annexin A2
138.571.359		20.835.769	LAPTM4B	lysosomal protein transmembrane 4 beta
138.570.079	0.9998237		WIPF2	WAS/WASL interacting protein family member 2
13.855.218		-21.972.864	IL1RAP	interleukin 1 receptor accessory protein
138.472.738		11.234.971	PRMT3	protein arginine methyltransferase 3
138.358.169	0.7848027		RPSAP19///	ribosomal protein SA pseudogene 19///ribosom
138.325.604		13.284.894	CSTB	cystatin B
138.306.342		11.284.336	LMNA	lamin A/C
138.222.672	0.5824118		SHFM1	split hand/foot malformation (ectrodactyly) typ
138.108.043		15.243.376	PLVAP	plasmalemma vesicle associated protein
138.074.086		1.800.134	ANXA2	annexin A2
137.928.599		18.012.421	ANXA2	annexin A2
13.774.613	0.9326269		MRPS14	mitochondrial ribosomal protein S14
137.712.438		10.446.516	FAM208A	family with sequence similarity 208 member A
137.596.402	0.7633904		HNRNPU	heterogeneous nuclear ribonucleoprotein U
13.754.023		-19.848.914	ACSM3	acyl-CoA synthetase medium-chain family mem
137.509.606		168.471	LAMC1	laminin subunit gamma 1
137.429.619		-12.349.659	IVD	isovaleryl-CoA dehydrogenase
137.385.126	0.6857196		DRG1	developmentally regulated GTP binding protein
137.367.992		12.058.628	INTS7	integrator complex subunit 7
137.242.514		-12.479.544	NAAA	N-acylethanolamine acid amidase
137.202.373	-0.886881		GNAO1	G protein subunit alpha o1
137.192.438	0.9338531		SRRD	SRR1 domain containing
137.114.988		11.190.612	TXNDC9	thioredoxin domain containing 9
137.110.257		-21.173.168	CYP3A43	cytochrome P450 family 3 subfamily A member
137.027.718		-12.561.049	ALDH6A1	aldehyde dehydrogenase 6 family member A1
13.701.011		12.089.406	AGFG1	ArfGAP with FG repeats 1
136.926.768	0.9981766		UFC1	ubiquitin-fold modifier conjugating enzyme 1
136.709.762		21.152.011	FABP5P2///	fatty acid binding protein 5 pseudogene 2///fatt
136.685.088		13.844.388	RCN2	reticulocalbin 2
136.647.283		-12.279.651	SKAP1	src kinase associated phosphoprotein 1

136.586.838	13.246.628	NDC80	NDC80, kinetochore complex component
136.520.304	12.740.902	NPC1	NPC intracellular cholesterol transporter 1
136.422.351	12.391.577	LBR	lamin B receptor
136.418.136	12.505.568	DNAJC9	DnaJ heat shock protein family (Hsp40) membe
136.329.411	-1.302.371	EPB41L4B	erythrocyte membrane protein band 4.1 like 4B
136.299.009	12.754.915	ACLY	ATP citrate lyase
136.027.484	-18.823.276		
135.953.824	13.065.199	POLR2K	RNA polymerase II subunit K
135.838.041	12.751.926	MCM4	minichromosome maintenance complex comp
135.709.759	11.819.677	RPS6KC1	ribosomal protein S6 kinase C1
135.687.183	-11.660.501	IER2	immediate early response 2
135.658.255	11.849.268	GTPBP4	GTP binding protein 4
135.243.509	-17.702.742	PLAC8	placenta specific 8
135.192.566	0.7272472	DYNLRB1	dynein light chain roadblock-type 1
135.178.704	1.285.357	XPOT	exportin for tRNA
135.163.817	-2.019.369	PBLD	phenazine biosynthesis like protein domain cor
135.154.524	13.723.808	RAD51AP1	RAD51 associated protein 1
135.075.746	-0.6238069	NTF3	neurotrophin 3
135.025.323	-11.868.051	IVD	isovaleryl-CoA dehydrogenase
134.941.731	126.007	CACYBP	calcyclin binding protein
134.863.334	-31.570.889	CYP3A4	cytochrome P450 family 3 subfamily A member
134.771.133	-24.796.471	FOSB	FosB proto-oncogene, AP-1 transcription factor
134.703.868	-14.222.583	NAT1	N-acetyltransferase 1
134.604.628	-10.466.073	TNS2	tensin 2
13.455.302	-18.038.886	ALDH6A1	aldehyde dehydrogenase 6 family member A1
134.548.854	0.9430902	EIF2D	eukaryotic translation initiation factor 2D
134.228.682	-12.833.743	SLC35D1	solute carrier family 35 member D1
134.221.966	15.236.926	MAD2L1	MAD2 mitotic arrest deficient-like 1 (yeast)
13.418.354	13.673.537	RAN	RAN, member RAS oncogene family
134.086.588	-22.180.327	AKR7A3	aldo-keto reductase family 7 member A3
134.019.644	11.541.129	HMGN4	high mobility group nucleosomal binding domai
133.860.907	0.9407954	WHSC1	Wolf-Hirschhorn syndrome candidate 1
133.820.288	-11.136.589	PRKAG2	protein kinase AMP-activated non-catalytic sub
1.337.842	-22.390.095	ALDH6A1	aldehyde dehydrogenase 6 family member A1
133.733.757	0.9827405	CALU	calumenin
133.731.823	1.236.583	USP1	ubiquitin specific peptidase 1
133.703.475	10.105.906	RFC1	replication factor C subunit 1
133.677.671	-20.577.954	FOLH1	folate hydrolase (prostate-specific membrane a
133.636.782	0.9120325	LOC65397:	similar to chromobox homolog 3///chromobox 3
133.478.478	17.348.947	TXNRD1	thioredoxin reductase 1
133.452.632	13.538.756	SSR1	signal sequence receptor subunit 1
133.351.263	13.764.511	ITGB3BP	integrin subunit beta 3 binding protein
133.337.205	13.531.185	CKAP4	cytoskeleton associated protein 4
133.320.183	11.551.224	LSM4	LSM4 homolog, U6 small nuclear RNA and mRN

133.133.264		1.196.808	FANCI	Fanconi anemia complementation group I
133.040.879		-1.276.333	HMGCL	3-hydroxymethyl-3-methylglutaryl-CoA lyase
132.878.175	-0.9337091		KBTBD11	kelch repeat and BTB domain containing 11
132.877.862		-12.224.591	KIAA0922	KIAA0922
132.689.561		15.626.523	PSPH	phosphoserine phosphatase
132.621.771	0.8566177		FAM189B	family with sequence similarity 189 member B
132.530.897		-16.300.645	GNE	glucosamine (UDP-N-acetyl)-2-epimerase/N-a
132.508.157		-14.864.366	CYP3A43	cytochrome P450 family 3 subfamily A member
132.405.146		-33.571.101	CYP3A4	cytochrome P450 family 3 subfamily A member
13.236.016		10.711.473	WHSC1	Wolf-Hirschhorn syndrome candidate 1
132.346.627		-22.334.307	FTCD	formimidoyltransferase cyclodeaminase
132.323.659		11.056.036	C5orf22	chromosome 5 open reading frame 22
132.245.111	0.8323282		TMEM258	transmembrane protein 258
132.150.748		-35.265.806	SLCO1B3	solute carrier organic anion transporter family r
132.126.058		10.274.665	CANT1	calcium activated nucleotidase 1
132.107.493		11.701.819	TXNDC9	thioredoxin domain containing 9
13.201.224		11.593.411	FEN1	flap structure-specific endonuclease 1
131.975.888		-20.139.795	FOLH1	folate hydrolase (prostate-specific membrane a
131.932.314	0.9591446		CCT4	chaperonin containing TCP1 subunit 4
131.801.061	0.9133681		POP4	POP4 homolog, ribonuclease P/MRP subunit
131.781.811	0.8222348		SAP130	Sin3A associated protein 130
131.781.618		-146.622	GRHPR	glyoxylate and hydroxypyruvate reductase
131.655.983		-11.828.018	BCKDHB	branched chain keto acid dehydrogenase E1 su
131.632.478	0.9185695		MFSD5	major facilitator superfamily domain containing
131.480.717		-18.925.717	IGF1	insulin like growth factor 1
131.444.002		-16.641.713	ITIH4	inter-alpha-trypsin inhibitor heavy chain family
131.332.953		14.740.022	DLG5	discs large MAGUK scaffold protein 5
131.230.106	-0.7836037		NAAA	N-acylethanolamine acid amidase
131.126.165		-14.271	NMRK1	nicotinamide riboside kinase 1
131.125.307		-14.039.711	LYVE1	lymphatic vessel endothelial hyaluronan recept
131.063.765		13.849.791	PCNA	proliferating cell nuclear antigen
1.310.121		-28.982.006	CYP2C9	cytochrome P450 family 2 subfamily C member
130.609.695	0.8302282		PSMD1	proteasome 26S subunit, non-ATPase 1
130.591.314		11.025.112	MCM7	minichromosome maintenance complex comp
130.569.546		-27.420.921	CYP2C9	cytochrome P450 family 2 subfamily C member
130.261.303	0.9015527		PIK3CB	phosphatidylinositol-4,5-bisphosphate 3-kinas
130.055.137	0.8385691		ELOVL1	ELOVL fatty acid elongase 1
130.035.879		13.117.554	ACTL6A	actin like 6A
130.031.616		-22.561.723	SLC4A4	solute carrier family 4 member 4
129.958.455	0.8719626		PTGES3	prostaglandin E synthase 3
129.900.503		-15.516.222	GADD45A	growth arrest and DNA damage inducible alpha
129.849.596		31.297.681	CCL20	C-C motif chemokine ligand 20
129.475.325		-11.945.306	SARDH	sarcosine dehydrogenase
129.465.163	0.9554148		SEC61G	Sec61 translocon gamma subunit

129.440.055		12.410.217	CLIC1	chloride intracellular channel 1
129.383.749	0.9684559		NCAPG2	non-SMC condensin II complex subunit G2
129.368.219	0.9057942		DNAJB6	DnaJ heat shock protein family (Hsp40) membe
129.225.047		-29.110.411	AFM	afamin
129.175.674		-10.548.754	GPM6A	glycoprotein M6A
129.174.671		13.158.643	DLGAP5	DLG associated protein 5
128.931.573	0.8408054		DYNLRB1	dynein light chain roadblock-type 1
128.873.143		10.226.143	DNAJC2	DnaJ heat shock protein family (Hsp40) membe
128.766.829		11.363.089	RAD21	RAD21 cohesin complex component
128.679.463		12.370.589	TARBP1	TAR (HIV-1) RNA binding protein 1
128.595.736		12.140.303	KIAA0196	KIAA0196
128.484.562		-24.760.394	BBOX1	gamma-butyrobetaine hydroxylase 1
128.481.758	0.7139435		CDC27	cell division cycle 27
128.308.492		-14.818.877	CETP	cholesteryl ester transfer protein
128.299.032	0.9918933		RNASEH2A	ribonuclease H2 subunit A
128.293.427	0.8401349		SMAD2	SMAD family member 2
128.262.986		12.926.371	CCNA2	cyclin A2
128.256.764		-25.612.098	CTH	cystathionine gamma-lyase
128.201.463		11.823.842	MPZL1	myelin protein zero like 1
128.118.299	0.7231331		PSMA6	proteasome subunit alpha 6
127.956.427		11.709.728	FANCI	Fanconi anemia complementation group I
127.893.338	0.7432744		METTL13	methyltransferase like 13
127.879.245		16.902.703	SMYD3	SET and MYND domain containing 3
127.874.832		-17.376.296	CYP3A43	cytochrome P450 family 3 subfamily A member
127.741.446		12.089.245	TACC3	transforming acidic coiled-coil containing prote
12.773.699		10.738.431	STAM	signal transducing adaptor molecule
127.721.025		-2.076.526	CYP26A1	cytochrome P450 family 26 subfamily A membe
127.498.796		10.888.832	ANP32E	acidic nuclear phosphoprotein 32 family memb
127.396.815		21.343.458	LAPTM4B	lysosomal protein transmembrane 4 beta
127.343.147		11.843.273	NCAPG	non-SMC condensin I complex subunit G
127.255.045		10.869.968	H2AFY///TL	H2A histone family member Y///toll like recepto
127.102.821	0.8634382		NUP62	nucleoporin 62
127.077.404		137.672	NRAS	neuroblastoma RAS viral oncogene homolog
126.907.755		11.217.129	TRIM37	tripartite motif containing 37
126.872.849	0.9238994		TXNL4A	thioredoxin like 4A
126.869.737		1.083.299	CAMSAP2	calmodulin regulated spectrin associated prote
126.831.229	0.8986523		PDCD5	programmed cell death 5
126.626.313		13.949.948	HLTF	helicase like transcription factor
12.642.053	0.6234047		RPS10	ribosomal protein S10
126.204.224		12.222.572	HMGN4	high mobility group nucleosomal binding domai
126.204.002		10.639.589	FBL	fibrillarin
126.134.429		-16.581.595	ACADL	acyl-CoA dehydrogenase, long chain
126.072.236		11.312.873	TAF9	TATA-box binding protein associated factor 9
12.599.757		-14.864.928	LONP2	lon peptidase 2, peroxisomal

125.982.425	-0.7780377		TBC1D2B	TBC1 domain family member 2B
125.911.825		2.024.963	COL4A1	collagen type IV alpha 1 chain
125.905.097		1.145.152	DKC1	dyskerin pseudouridine synthase 1
125.900.179		-19.365.374	EPHX2	epoxide hydrolase 2
125.859.971		-19.407.932	ADH6	alcohol dehydrogenase 6 (class V)
125.809.704	0.9966417		TCEB1	transcription elongation factor B subunit 1
125.799.266		11.136.899	ATIC	5-aminoimidazole-4-carboxamide ribonucleoti
125.779.667		-12.337.042	HDAC6	histone deacetylase 6
125.771.178		12.598.435	POLE2	DNA polymerase epsilon 2, accessory subunit
125.681.545	-0.6982059		MACF1	microtubule-actin crosslinking factor 1
125.676.855		-14.697.313	SLC35D1	solute carrier family 35 member D1
125.671.525		10.653.608	HSPA14	heat shock protein family A (Hsp70) member 14
125.640.449		13.392.616	LOC73104	similar to Ubiquitin-conjugating enzyme E2S (U
125.509.546		11.607.641	PRCC	papillary renal cell carcinoma (translocation-as
125.465.613		-26.386.505	PDGFRA	platelet derived growth factor receptor alpha
125.440.886	0.8716503		NOL11	nucleolar protein 11
125.289.888		1.048.681	WDR12	WD repeat domain 12
125.218.558	0.6104426		PSME3	proteasome activator subunit 3
125.092.573		-17.520.511	AR	androgen receptor
125.024.662	0.9629519		BUB3	BUB3, mitotic checkpoint protein
124.940.182	0.9902152		IARS	isoleucyl-tRNA synthetase
124.933.889		10.703.811	HJURP	Holliday junction recognition protein
124.885.739		1.011.058	RMI1	RecQ mediated genome instability 1
124.824.121		-12.612.476	GPD1	glycerol-3-phosphate dehydrogenase 1
124.543.786		-32.079.484	CYP2C8	cytochrome P450 family 2 subfamily C member
124.410.079		1.654.446	TKT	transketolase
124.318.325		-28.073.564	CYP2C9///(	cytochrome P450 family 2 subfamily C member
124.288.875		12.362.962	SLC38A6	solute carrier family 38 member 6
124.276.428	0.9215197		GGPS1	geranylgeranyl diphosphate synthase 1
124.143.544		-10.242.091	ECHS1	enoyl-CoA hydratase, short chain 1
124.110.541	0.7996759		DDX10	DEAD-box helicase 10
124.049.529		-29.128.687	CYP2C9	cytochrome P450 family 2 subfamily C member
123.982.863		10.645.728	LRRC59	leucine rich repeat containing 59
123.859.692	0.8127769		ATP6V1E1	ATPase H+ transporting V1 subunit E1
123.815.116	0.7491933		RPS10	ribosomal protein S10
123.749.846		11.230.744	GGPS1	geranylgeranyl diphosphate synthase 1
123.725.801	0.5661239		HNRNPC	heterogeneous nuclear ribonucleoprotein C (C1
123.646.867		13.584.791	MID1IP1	MID1 interacting protein 1
123.549.332		-30.071.967	GNMT	glycine N-methyltransferase
123.485.058	0.7612998		PWP1	PWP1 homolog, endonuclein
123.435.712	0.7305841		RPS10	ribosomal protein S10
123.359.497		13.919.989	ANXA2P2	annexin A2 pseudogene 2
123.337.359	0.7422508		RBM42	RNA binding motif protein 42
123.151.265		11.908.271	ANP32E	acidic nuclear phosphoprotein 32 family memb

12.314.967		-21.645.232	ACSM5	acyl-CoA synthetase medium-chain family mer
123.135.305	0.9030386		USP3	ubiquitin specific peptidase 3
123.121.458		11.463.847	ADSL	adenylosuccinate lyase
12.302.941		10.059.629	RPN2	ribophorin II
122.799.702		10.182.962	YWHAZ	tyrosine 3-monooxygenase/tryptophan 5-mono
122.767.019	0.8639939		HMGN1	high mobility group nucleosome binding domai
122.691.934		11.317.387	LANCL1	LanC like 1
122.662.553	0.7888202		RPS21	ribosomal protein S21
122.644.467		-17.154.771	DHRS1	dehydrogenase/reductase 1
122.607.783		-24.459.528	C6	complement component 6
122.371.905	0.9680466		HMGN1	high mobility group nucleosome binding domai
122.322.648		-12.046.633	FYN	FYN proto-oncogene, Src family tyrosine kinase
122.276.822	0.6867943		TRMT112	tRNA methyltransferase 11-2 homolog (S. cerev
122.195.177	0.9332054		CEP350	centrosomal protein 350
122.177.119		-10.181.486	KLHL2	kelch like family member 2
122.167.386		-13.096.515	GOT2	glutamic-oxaloacetic transaminase 2
12.216.698		-29.049.497	ALDOB	aldolase, fructose-bisphosphate B
122.145.619		-20.633.786	PLGLA	plasminogen-like A (pseudogene)
122.128.279		10.896.307	TPRKB	TP53RK binding protein
122.001.327	0.9767702		SRPK1	SRSF protein kinase 1
121.944.379		10.848.738	POLR2K	RNA polymerase II subunit K
12.178.053		-14.254.559	FAM149A	family with sequence similarity 149 member A
121.691.463		10.613.835	ORMDL2	ORMDL sphingolipid biosynthesis regulator 2
121.570.908	0.8874869		SETDB1	SET domain bifurcated 1
121.564.797	-0.6327672		FARP2	FERM, ARH/RhoGEF and pleckstrin domain prot
12.155.913		-18.795.219	CD14	CD14 molecule
121.426.666		-24.673.344	ACSM5	acyl-CoA synthetase medium-chain family mer
121.402.042	0.9319689		UBE2E1	ubiquitin conjugating enzyme E2 E1
121.393.795	0.5777106		RPL27	ribosomal protein L27
121.262.679		1.209.568	CENPA	centromere protein A
121.248.171	0.6872349		NONO	non-POU domain containing, octamer-binding
121.107.171		10.410.448	ITGB1BP1	integrin subunit beta 1 binding protein 1
121.032.356	0.9819492		GORASP2	golgi reassembly stacking protein 2
121.027.668	0.9515545		METTL13	methyltransferase like 13
120.994.699		10.551.706	KIAA0907	KIAA0907
120.974.973		11.258.178	WDYHV1	WDYHV motif containing 1
120.937.053	0.7861669		H3F3AP6//,	H3 histone, family 3A, pseudogene 6///H3 histo
120.752.615		-18.720.192	DUSP1	dual specificity phosphatase 1
120.614.891		-14.835.965	GRHPR	glyoxylate and hydroxypyruvate reductase
120.521.616	0.9724452		KIF14	kinesin family member 14
120.498.118		12.154.263	SLC1A4	solute carrier family 1 member 4
120.333.127		12.509.837	LAMC1	laminin subunit gamma 1
120.254.829		14.179.998	TUFT1	tuftelin 1
120.237.724		11.832.808	TMPO	thymopoietin

120.233.153	0.9358494		ZNF143	zinc finger protein 143
120.195.156		-10.033.796	TTC38	tetratricopeptide repeat domain 38
120.103.756	0.9964668		MARK4	microtubule affinity regulating kinase 4
120.102.423	0.8198315		POLR2G	RNA polymerase II subunit G
120.082.453		15.321.636	LRRC1	leucine rich repeat containing 1
120.013.641	0.8180036		SSR1	signal sequence receptor subunit 1
119.670.528	0.9704155		STT3A	STT3A, catalytic subunit of the oligosaccharyltra
119.641.468		14.562.363	CCNE2	cyclin E2
119.632.275		1.099.429	SMARCA4	SWI/SNF related, matrix associated, actin depe
119.598.678	0.7676084		CDC73	cell division cycle 73
11.958.198	0.8302364		NDRG3	NDRG family member 3
119.522.596	0.843414		CLTA	clathrin light chain A
119.504.514		-21.029.115	HBA2	hemoglobin subunit alpha 2
119.482.036		11.479.682	AGO2	argonaute 2, RISC catalytic component
119.445.517		14.113.856	PODXL	podocalyxin like
119.404.573		-10.786.677	GPM6A	glycoprotein M6A
119.322.525		12.429.295	MCM7	minichromosome maintenance complex comp
119.289.169		-22.718.014	ALDH8A1	aldehyde dehydrogenase 8 family member A1
11.925.141	0.9619088		TAF2	TATA-box binding protein associated factor 2
119.118.231		-11.347.754	ABHD6	abhydrolase domain containing 6
119.075.799		42.624.462	SPINK1	serine peptidase inhibitor, Kazal type 1
119.070.876	0.9988029		ZNF706	zinc finger protein 706
118.904.954	0.5377517		HNRNPC	heterogeneous nuclear ribonucleoprotein C (C1
118.887.553	0.5684856		DCTN2	dynactin subunit 2
118.865.318		-26.850.216	CYP2C9	cytochrome P450 family 2 subfamily C member
118.685.501		-23.196.554	FXYD1	FXYD domain containing ion transport regulator
118.678.816	0.7186041		ATP5J2	ATP synthase, H+ transporting, mitochondrial F
11.859.273	0.6328713		GEMIN6	gem nuclear organelle associated protein 6
118.170.449	0.8071043		BUB3	BUB3, mitotic checkpoint protein
118.170.124	0.9726891		PPP4R1	protein phosphatase 4 regulatory subunit 1
118.123.972		-10.491.687	NR3C2	nuclear receptor subfamily 3 group C member 2
118.093.316		-14.677.668	EXPH5	exophilin 5
118.058.009	-0.9069434		NR1I2	nuclear receptor subfamily 1 group I member 2
118.042.231	0.9723235		ACLY	ATP citrate lyase
118.016.312		1.112.291	GBAP1///GI	glucosylceramidase beta pseudogene 1///gluco
117.940.333		-30.783.799	ADH1C///A	alcohol dehydrogenase 1C (class I), gamma po
117.932.713	0.6218938		SUMO2	small ubiquitin-like modifier 2
117.915.504	0.8040214		MED20	mediator complex subunit 20
117.746.505		-13.113.115	PLPP3	phospholipid phosphatase 3
117.745.101		12.089.816	HAT1	histone acetyltransferase 1
117.671.787		15.919.296	RRAGD	Ras related GTP binding D
11.764.565	0.8570212		TRIP4	thyroid hormone receptor interactor 4
117.572.691	0.8213958		DENND5A	DENN domain containing 5A
11.749.347		-1.923.084	AASS	aminoadipate-semialdehyde synthase

117.343.386	0.6889949		NCOA6	nuclear receptor coactivator 6
117.322.638		-17.064.615	CYFIP2	cytoplasmic FMR1 interacting protein 2
117.243.605		-13.089.912	CYP3A43	cytochrome P450 family 3 subfamily A member
117.230.541	0.8977338		COPS6	COP9 signalosome subunit 6
117.222.195	-0.8769778		GPHN	gephyrin
11.715.364	0.9925924		RPP40	ribonuclease P/MRP subunit p40
117.078.042	-0.9237302		TPMT	thiopurine S-methyltransferase
117.075.169		11.186.046	UBR5	ubiquitin protein ligase E3 component n-recogr
117.067.016	-0.7092846		INPP5B	inositol polyphosphate-5-phosphatase B
116.983.877	0.8770679		SNU13	SNU13 homolog, small nuclear ribonucleoprote
116.815.151		-12.582.987	RAB17	RAB17, member RAS oncogene family
116.732.181		-21.043.818	HBA2///HB/	hemoglobin subunit alpha 2///hemoglobin subu
116.722.856		-14.413.531	GRHPR	glyoxylate and hydroxypyruvate reductase
116.703.743		-21.617.418	ENO3	enolase 3
116.664.074	0.7085589		H3F3AP4///	H3 histone, family 3A, pseudogene 4///H3 histo
116.635.961		10.317.702	FAM50A	family with sequence similarity 50 member A
116.458.474		14.703.856	TFRC	transferrin receptor
116.383.699		-21.403.925	ADH6	alcohol dehydrogenase 6 (class V)
116.380.496		-31.545.909	IGHA2///IG	immunoglobulin heavy constant alpha 2 (A2m r
11.634.951		-12.595.892	CA5A	carbonic anhydrase 5A
116.326.286		10.517.423	VRK1	vaccinia related kinase 1
116.308.243	0.5733785		NACA	nascent polypeptide-associated complex alpha
116.250.793	0.9553742		FLAD1	flavin adenine dinucleotide synthetase 1
116.230.611		1.088.977	NECAB3	N-terminal EF-hand calcium binding protein 3
116.185.724	0.8626897		RPL36AP37	ribosomal protein L36a pseudogene 37///riboso
116.046.841	0.6316826		ZFR	zinc finger RNA binding protein
116.011.019	-0.927358		FANCC	Fanconi anemia complementation group C
116.002.557		-1.376.537	CLU	clusterin
115.989.417		-15.668.986	BGN	biglycan
115.946.808		19.684.101	SQLE	squalene epoxidase
115.917.578		-11.583.465	SLC28A1	solute carrier family 28 member 1
115.807.711	0.8391772		DIEXF	digestive organ expansion factor homolog (zebr
115.796.767	0.9203231		GM2A	GM2 ganglioside activator
115.776.992		-20.780.155	HBA2///HB/	hemoglobin subunit alpha 2///hemoglobin subu
115.770.454		-12.917.182	CAPN3	calpain 3
115.762.733		-28.718.016	BCHE	butyrylcholinesterase
115.757.229	0.9465497		DNAAF5	dynein axonemal assembly factor 5
115.751.234		10.623.716	HMMR	hyaluronan mediated motility receptor
115.726.563		11.687.358	TMEM38B	transmembrane protein 38B
115.722.377		-29.311.583	PCK1	phosphoenolpyruvate carboxykinase 1
115.694.858		13.477.481	AZIN1	antizyme inhibitor 1
115.674.673	0.7970785		MTX1	metaxin 1
11.566.053	-0.9134684		TRPV1	transient receptor potential cation channel sub
115.617.036		10.231.278	NEK7	NIMA related kinase 7

115.570.868	0.9737223		SOCS5	suppressor of cytokine signaling 5
115.504.536	0.8984496		PRRC2C	proline rich coiled-coil 2C
115.382.881		-10.967.784	TNXB	tenascin XB
115.373.177	0.7147417		RBX1	ring-box 1
115.308.962		12.927.748	UCHL5	ubiquitin C-terminal hydrolase L5
115.044.835		-2.013.082	AZGP1P1//,	alpha-2-glycoprotein 1, zinc-binding pseudoger
115.043.613		-1.275.654	CAPN3	calpain 3
115.022.975	0.8783469		DARS2	aspartyl-tRNA synthetase 2, mitochondrial
114.957.723	0.702823		UBE2N	ubiquitin conjugating enzyme E2 N
114.645.702	0.9005895		TUBB4B	tubulin beta 4B class IVb
114.642.405	0.8461906		TMCO1	transmembrane and coiled-coil domains 1
114.604.088		-13.509.996	SULT1A2	sulfotransferase family 1A member 2
114.511.866	0.9787496		PTK2	protein tyrosine kinase 2
114.452.907		11.524.996	SLC1A4	solute carrier family 1 member 4
114.420.682		11.634.281	KNTC1	kinetochore associated 1
114.409.627	0.9313445		NXT1	nuclear transport factor 2 like export factor 1
114.385.033		12.301.903	GOLT1B	golgi transport 1B
114.381.198		12.299.941	PEA15	phosphoprotein enriched in astrocytes 15
114.348.601		-13.394.933	SLC16A2	solute carrier family 16 member 2
114.322.204		-12.751.184	CYP4F12	cytochrome P450 family 4 subfamily F member
114.234.541		-15.786.861	RCAN1	regulator of calcineurin 1
114.232.719	0.7750642		ATF2	activating transcription factor 2
114.155.169	0.7648183		YWHAQ	tyrosine 3-monooxygenase/tryptophan 5-mono
114.107.484		-13.291.493	CDC14B	cell division cycle 14B
114.097.453	0.9344289		UFD1L	ubiquitin fusion degradation 1 like (yeast)
11.404.281		-17.782.811	SEC14L2	SEC14 like lipid binding 2
11.400.205	0.525012		RPL23A	ribosomal protein L23a
11.397.169		12.076.773	BUB3	BUB3, mitotic checkpoint protein
113.960.051	0.8233953		EHMT2	euchromatic histone lysine methyltransferase 2
113.946.421	0.4832047		RPL23A	ribosomal protein L23a
113.905.297	0.9829438		PDIA6	protein disulfide isomerase family A member 6
113.818.116		-10.737.556	CHRD	chordin
113.813.519		-1.433.441	MSRA	methionine sulfoxide reductase A
113.809.635	0.988163		KIF2A	kinesin family member 2A
113.776.973	0.8173387		RPP30	ribonuclease P/MRP subunit p30
113.659.776	0.9634744		PSMD2	proteasome 26S subunit, non-ATPase 2
113.537.323	0.8140046		H3F3AP6//,	H3 histone, family 3A, pseudogene 6///H3 histc
113.517.396		37.837.773	PEG10	paternally expressed 10
113.513.012		-1.641.533	CYP2J2	cytochrome P450 family 2 subfamily J member
113.507.981	0.8030187		RUVBL2	RuvB like AAA ATPase 2
113.506.602		-13.847.978	RHOB	ras homolog family member B
113.413.578	0.7305523		PSMB5	proteasome subunit beta 5
113.367.052		-10.456.082	TNXB	tenascin XB
113.351.025		-22.031.971	HBA2///HB,	hemoglobin subunit alpha 2///hemoglobin subu

113.333.534	0.9431959		SPATS2	spermatogenesis associated serine rich 2
113.325.868		-21.590.014	HBA1	hemoglobin subunit alpha 1
113.319.759	0.9443118		FOXK2	forkhead box K2
113.290.286		-18.736.317	DCXR	dicarbonyl and L-xylulose reductase
113.204.191		-21.414.909	HBA2///HB/	hemoglobin subunit alpha 2///hemoglobin subu
113.085.017		-1.059.301	ACADVL	acyl-CoA dehydrogenase, very long chain
113.002.529		-1.059.931	CAPN3	calpain 3
112.980.397		-13.633.564	SULT1A2	sulfotransferase family 1A member 2
11.296.499	0.7462229		XPO1	exportin 1
112.958.628	0.8731733		PPOX	protoporphyrinogen oxidase
112.943.804		-17.194.132	TSHZ1///BG	teashirt zinc finger homeobox 1///biglycan
112.910.183		1.054.471	CCT2	chaperonin containing TCP1 subunit 2
112.807.741	-0.8146804		PDLIM5	PDZ and LIM domain 5
112.796.604		10.374.719	PDIA6	protein disulfide isomerase family A member 6
112.712.769	0.6650719		CASC3	cancer susceptibility candidate 3
112.646.344	0.9719208		B4GALT3	beta-1,4-galactosyltransferase 3
112.634.186		11.882.081	KLHL23	kelch like family member 23
112.631.018	0.9927869		HMGN4	high mobility group nucleosomal binding doma
112.618.913	0.6089244		CLTC	clathrin heavy chain
112.595.383	0.7971833		RBBP7	RB binding protein 7, chromatin remodeling fac
112.576.814		10.084.929	YWHAB	tyrosine 3-monooxygenase/tryptophan 5-mono
112.576.015	0.6965227		EIF5B	eukaryotic translation initiation factor 5B
112.534.856		11.412.068	GM2A	GM2 ganglioside activator
112.501.172	0.7433028		COG5	component of oligomeric golgi complex 5
112.498.762		-18.132.747		
112.493.836		142.318	CPD	carboxypeptidase D
112.404.863	0.9115881		MFAP1	microfibrillar associated protein 1
112.343.292	-0.6732057			
112.144.354	0.9376182		RNF8	ring finger protein 8
112.031.712	0.4783552		RPS20	ribosomal protein S20
111.977.553		-19.043.153	ALDH6A1	aldehyde dehydrogenase 6 family member A1
111.941.195	0.9794065		FNBP1L	formin binding protein 1 like
111.868.369	0.9340317		SLC35A2	solute carrier family 35 member A2
111.822.776	0.7662263			
11.181.302	-0.9569475		EHD3	EH domain containing 3
111.804.142		11.502.315	RCN2	reticulocalbin 2
111.718.812	0.5271286		RPL23A	ribosomal protein L23a
111.704.975		16.750.008	PLXNC1	plexin C1
111.606.285	0.7249212		USP39	ubiquitin specific peptidase 39
11.158.604	0.9329236		TUBB4B	tubulin beta 4B class IVb
111.571.457		23.551.996	SULT1C2	sulfotransferase family 1C member 2
111.507.337	0.9860116		DNAJC9	DnaJ heat shock protein family (Hsp40) membe
111.459.911		-1.545.259	LONP2	lon peptidase 2, peroxisomal
111.442.237		12.549.727	BOP1	block of proliferation 1

111.434.656		10.460.571	TBC1D16	TBC1 domain family member 16
111.433.758	0.7615275		RAB11A	RAB11A, member RAS oncogene family
11.135.067	0.6303909		DHX9	DEAH-box helicase 9
111.252.021		11.645.581	TOPBP1	topoisomerase (DNA) II binding protein 1
111.247.838	0.9263635		TBC1D31	TBC1 domain family member 31
111.247.455	0.8984305		SSBP1	single stranded DNA binding protein 1
111.107.748	-0.834408		ADAMTS2	ADAM metallopeptidase with thrombospondin t
111.029.626		-12.537.278	CPT2	carnitine palmitoyltransferase 2
111.026.634	0.9568754		GTSE1	G2 and S-phase expressed 1
110.979.084	0.7766558		NCKAP1	NCK associated protein 1
11.091.447		-15.754.183	IGF1	insulin like growth factor 1
110.848.201	0.9064152		RFXANK	regulatory factor X associated ankyrin containir
110.779.497		11.043.619	NOP56	NOP56 ribonucleoprotein
110.776.088		-19.793.189	COLEC11	collectin subfamily member 11
110.731.666	0.693676		LARP1	La ribonucleoprotein domain family member 1
110.707.488		11.931.682	RRS1	ribosome biogenesis regulator homolog
110.702.842	0.8452975		TIMELESS	timeless circadian clock
110.681.063	0.8847137		NUP85	nucleoporin 85
110.679.271		11.750.122	NEDD4L	neural precursor cell expressed, developmenta
110.677.308		-19.454.013	ITIH4	inter-alpha-trypsin inhibitor heavy chain family
110.670.871	0.9306289		GPSM2	G-protein signaling modulator 2
110.659.985	0.71361		ABCF2	ATP binding cassette subfamily F member 2
110.655.243		-14.451.278	ALDH1B1	aldehyde dehydrogenase 1 family member B1
110.603.066	0.5779448		LOC72882!	small ubiquitin-like modifier 2///SUMO2 pseud
110.569.169	0.9927602		MPZL1	myelin protein zero like 1
110.563.865		-16.927.331	ABAT	4-aminobutyrate aminotransferase
110.525.379		12.413.225	MRPL13	mitochondrial ribosomal protein L13
110.515.059	0.7390152		PSMC2	proteasome 26S subunit, ATPase 2
110.495.685	0.898969		TMEM184B	transmembrane protein 184B
110.465.977		11.265.067	C8orf33	chromosome 8 open reading frame 33
110.434.381		1.140.594	HS2ST1	heparan sulfate 2-O-sulfotransferase 1
110.423.995		-19.433.852	SLC46A3	solute carrier family 46 member 3
110.387.124	0.5644682		BRIX1	BRX1, biogenesis of ribosomes
110.309.285		10.090.262	HNRNPR	heterogeneous nuclear ribonucleoprotein R
110.292.325		10.785.436	LAGE3	L antigen family member 3
110.179.969	0.5357472		RPLP1	ribosomal protein lateral stalk subunit P1
110.018.701		10.056.634	DKC1	dyskerin pseudouridine synthase 1
109.962.302	0.836021		R3HDM1	R3H domain containing 1
109.953.368		-10.948.738	ACBD4	acyl-CoA binding domain containing 4
109.928.837	0.9554751		MANF	mesencephalic astrocyte derived neurotrophic
109.912.167	-0.5758138		GNA14	G protein subunit alpha 14
109.777.027		13.151.206	ATP6V1C1	ATPase H+ transporting V1 subunit C1
109.771.619	0.7730499		BPGM	bisphosphoglycerate mutase
109.752.468	-0.9863308		SAT1	spermidine/spermine N1-acetyltransferase 1

109.691.315	0.9631457		TSNAX	translin associated factor X
109.689.604		10.057.314	TMCO1	transmembrane and coiled-coil domains 1
109.636.675		-17.798.377	ABAT	4-aminobutyrate aminotransferase
109.636.109	0.7061023		DNAJC13	DnaJ heat shock protein family (Hsp40) membe
109.556.974	-0.7831506		SEC31B	SEC31 homolog B, COPII coat complex compor
109.546.726	0.8381018		PFDN2	prefoldin subunit 2
109.534.699	0.878916		CENPM	centromere protein M
109.494.752		12.919.439	PRKDC	protein kinase, DNA-activated, catalytic polype
109.472.711	0.8862159		GORASP2	golgi reassembly stacking protein 2
109.458.495	-0.8815474		FXN	frataxin
109.392.424	0.891868		PDSS1	prenyl (decaprenyl) diphosphate synthase, sub
109.316.844		13.018.465	BTG3	BTG anti-proliferation factor 3
109.283.731		11.499.445	BTG3	BTG anti-proliferation factor 3
109.269.229	0.829783		CCDC86	coiled-coil domain containing 86
109.262.802	0.9242141		DNM1L	dynamin 1 like
109.252.965	0.8414895		LOC65397	similar to chromobox homolog 3///chromobox 3
109.215.475		10.429.727	RNF187	ring finger protein 187
109.214.774	-0.4222643		ADRA1A	adrenoceptor alpha 1A
109.205.762		10.917.333	MRPS17	mitochondrial ribosomal protein S17
10.917.328	0.725372		GPKOW	G-patch domain and KOW motifs
109.128.726	0.9254229		DENR	density regulated re-initiation and release facto
10.907.231		-14.249.533	SULT1A1	sulfotransferase family 1A member 1
109.045.664	0.9007082		HNRNPU	heterogeneous nuclear ribonucleoprotein U
109.042.703	0.9947662		UPF3B	UPF3 regulator of nonsense transcripts homolo
108.907.512	0.7972205		TSN	translin
108.899.552	0.7602232		PSME3	proteasome activator subunit 3
108.864.886	-0.8116618		RBL2	RB transcriptional corepressor like 2
108.837.544		-10.204.894	TNXB	tenascin XB
10.883.369	-0.9137974		MLYCD	malonyl-CoA decarboxylase
108.761.052	0.7399717		SNRPF	small nuclear ribonucleoprotein polypeptide F
108.760.817		-12.890.556	FAHD2A	fumarylacetoacetate hydrolase domain contair
108.716.585		10.207.732	FAM21A	family with sequence similarity 21 member A
10.866.329	0.8468424		UBE2Z	ubiquitin conjugating enzyme E2 Z
108.630.312		-14.497.471	GADD45B	growth arrest and DNA damage inducible beta
108.578.566	-0.8694977		COLEC10	collectin subfamily member 10
108.497.551	-0.7786514		ALLC	allantoicase
108.455.977		11.121.578	KIF2C	kinesin family member 2C
108.352.158	0.5708543		RPL23A	ribosomal protein L23a
108.309.668	0.9052893		SEPHS1	selenophosphate synthetase 1
108.292.165	0.7826199		HNRNPA3/	heterogeneous nuclear ribonucleoprotein A3///
1.082.489		-21.061.162	PLGLB1///F	plasminogen-like B1///plasminogen-like B2
108.216.807	0.8635191		EED	embryonic ectoderm development
108.207.139	0.9787222		PAFAH1B3	platelet activating factor acetylhydrolase 1b ca
10.819.968		10.061.433	CHD1L	chromodomain helicase DNA binding protein 1

10.817.018	0.9789516		TUBB3	tubulin beta 3 class III
108.056.074		11.061.252	TRIP13	thyroid hormone receptor interactor 13
10.797.679	-0.7026448		PDXDC1	pyridoxal dependent decarboxylase domain co
107.949.426	0.9650294		ATR	ATR serine/threonine kinase
107.917.567		-11.082.972	CPT2	carnitine palmitoyltransferase 2
107.855.715		-14.795.931	SULT1A1	sulfotransferase family 1A member 1
107.816.809	0.8379488		MCM5	minichromosome maintenance complex comp
107.778.544		10.788.631	HSPA13	heat shock protein family A (Hsp70) member 1
107.750.501		-10.431.934	MPC1	mitochondrial pyruvate carrier 1
107.727.785	0.7002905		CSNK2B	casein kinase 2 beta
107.694.632	0.7506609		ARF1	ADP ribosylation factor 1
107.673.443		12.513.737	MKI67	marker of proliferation Ki-67
107.668.294		11.457.408	CDK1	cyclin dependent kinase 1
107.613.221		1.245.423	CPD	carboxypeptidase D
107.591.995	0.8507639		CHCHD3	coiled-coil-helix-coiled-coil-helix domain conta
107.557.568	0.8623389		UBE2D2	ubiquitin conjugating enzyme E2 D2
107.525.672	0.8951559		RAD51C	RAD51 paralog C
107.491.761		23.837.321	UBD	ubiquitin D
107.403.881	0.9936021		EIF2S2	eukaryotic translation initiation factor 2 subuni
107.403.526		10.751.866	RHEB	Ras homolog enriched in brain
107.394.423		-30.476.989	SLC10A1	solute carrier family 10 member 1
107.256.971		10.013.273	SUZ12	SUZ12 polycomb repressive complex 2 subunit
107.224.918	0.9195324		CCZ1	CCZ1 homolog, vacuolar protein trafficking and
10.721.106		-100.874	FAXDC2	fatty acid hydroxylase domain containing 2
107.207.463		-19.277.131	CYR61	cysteine rich angiogenic inducer 61
107.130.024		-10.092.249	EIF5	eukaryotic translation initiation factor 5
107.104.723	0.7835673		FAM21D///I	family with sequence similarity 21, member D//
107.100.445	0.5123268		SND1	staphylococcal nuclease and tudor domain co
107.070.583		11.893.355	ARMC1	armadillo repeat containing 1
107.058.749	0.8166423		C7orf49	chromosome 7 open reading frame 49
107.052.191	-0.8778641		ITSN1	intersectin 1
106.971.534		12.005.042	ATP6V1C1	ATPase H+ transporting V1 subunit C1
106.874.902	0.7090534		SAP30L	SAP30 like
106.833.297		11.509.748	YWHAH	tyrosine 3-monooxygenase/tryptophan 5-mono
106.819.421		-15.843.114	XDH	xanthine dehydrogenase
10.680.838		-15.493.209	GADD45B	growth arrest and DNA damage inducible beta
106.743.274		-20.204.253	SERPINA4	serpin family A member 4
106.652.788		-13.872.004	FCGR2B	Fc fragment of IgG receptor IIb
106.649.945	0.8725486		SMG5	SMG5, nonsense mediated mRNA decay factor
10.663.695	-0.6040811		HAND2-AS	HAND2 antisense RNA 1 (head to head)
106.630.481		-14.752.082	EDNRB	endothelin receptor type B
106.628.102	0.5854713		NACA	nascent polypeptide-associated complex alph
106.627.165		11.751.554	RBM3	RNA binding motif (RNP1, RRM) protein 3
10.659.454		11.231.831	PDCD2	programmed cell death 2

106.564.226	0.8687674		ENSA	endosulfine alpha
106.546.007		11.782.502	SSR1	signal sequence receptor subunit 1
106.514.244		10.775.007	E2F3	E2F transcription factor 3
106.499.143	0.7348868		OXLD1	oxidoreductase like domain containing 1
106.476.012	-0.8348004		SVEP1	sushi, von Willebrand factor type A, EGF and pe
106.475.422	0.9678665		MSH6	mutS homolog 6
106.465.092		12.043.639	TPGS2	tubulin polyglutamylase complex subunit 2
106.425.434	0.8054678		HMGXB4	HMG-box containing 4
106.404.501	0.8683161		KIN	Kin17 DNA and RNA binding protein
106.394.571		10.948.704	CTSA	cathepsin A
10.637.881	0.6669832		NPM1	nucleophosmin
106.314.383		-14.221.896	ACAA2	acetyl-CoA acyltransferase 2
106.310.137		-18.204.318	CYP4F2	cytochrome P450 family 4 subfamily F member
106.303.004	0.7588617		CTNNA1	catenin alpha 1
106.265.542	0.6273981		RPL7	ribosomal protein L7
106.222.058	0.9790082		PRKCI	protein kinase C iota
106.211.384		-12.809.995	ACADL	acyl-CoA dehydrogenase, long chain
10.617.232	0.9162269		MPZL1	myelin protein zero like 1
106.102.941	0.757179		PARP2	poly(ADP-ribose) polymerase 2
106.066.097	0.6455618		CLTA	clathrin light chain A
106.060.685		-19.003.198	ACSL1	acyl-CoA synthetase long-chain family member
10.587.275	0.7649447		BLZF1	basic leucine zipper nuclear factor 1
105.848.253		-12.298.246	MACROD1	MACRO domain containing 1
105.741.401		10.373.177	SPAG5	sperm associated antigen 5
105.614.889	0.9299314		SMARCA4	SWI/SNF related, matrix associated, actin depe
105.580.594	0.9235087		FIBP	FGF1 intracellular binding protein
105.505.524		-14.164.654	CFI	complement factor I
105.443.017	0.6311619		CALM2	calmodulin 2
105.422.162	-0.6086275		MOGAT2	monoacylglycerol O-acyltransferase 2
105.406.307		-10.070.488	IRF8	interferon regulatory factor 8
105.396.463	0.7226746		ATP6V1F	ATPase H+ transporting V1 subunit F
105.389.622		-11.175.523	ABHD6	abhydrolase domain containing 6
105.386.883		12.029.208	YWHAZ	tyrosine 3-monooxygenase/tryptophan 5-mono
105.328.198		1.060.222	DR1	down-regulator of transcription 1
105.248.422	-0.7576699		MPPED1	metallophosphoesterase domain containing 1
10.523.646		-26.819.842	ADH1B	alcohol dehydrogenase 1B (class I), beta polype
105.229.194	0.7498576		SSRP1	structure specific recognition protein 1
105.151.009	0.9159104		UBE2A	ubiquitin conjugating enzyme E2 A
105.146.662		14.538.896	CPD	carboxypeptidase D
105.140.692		11.012.869	CLN3	CLN3, battenin
105.054.376		-11.255.211	SLC6A16	solute carrier family 6 member 16
105.016.437		11.872.124	MOB4	MOB family member 4, phocein
104.950.451		-20.186.998	CYR61	cysteine rich angiogenic inducer 61
104.894.994	0.5850588		HNRNPA1P	heterogeneous nuclear ribonucleoprotein A1 ps

104.877.745	0.8938545		SYPL1	synaptophysin like 1
104.842.547	0.9806741		BZW2	basic leucine zipper and W2 domains 2
104.831.752	0.6543697		DRAP1	DR1 associated protein 1
104.774.712		10.223.132	CDC123	cell division cycle 123
104.566.553	0.8473197		EIF4A3	eukaryotic translation initiation factor 4A3
104.527.567	0.747736		TIMM9	translocase of inner mitochondrial membrane 9
104.469.291	0.7020114		RUFY1	RUN and FYVE domain containing 1
104.452.621		-26.616.879	F9	coagulation factor IX
10.445.184		10.726.455	CDK7	cyclin dependent kinase 7
10.444.486		-14.495.489	FAXDC2	fatty acid hydroxylase domain containing 2
104.360.806		17.259.008	COL15A1	collagen type XV alpha 1 chain
104.304.964	0.940863		SRM	spermidine synthase
10.417.763	0.8719168		NHP2	NHP2 ribonucleoprotein
104.095.777		-22.311.778	CTH	cystathionine gamma-lyase
104.065.563		10.308.478	PNO1	partner of NOB1 homolog
104.061.474		-11.884.359	CAT	catalase
104.041.837		-1.141.306	MUT	methylmalonyl-CoA mutase
104.004.188		-10.600.074	STAB1	stabilin 1
103.886.757	-0.6320218		FYN	FYN proto-oncogene, Src family tyrosine kinase
103.883.763	0.7896363		CENPF	centromere protein F
10.375.703	0.7961096		PABPC1	poly(A) binding protein cytoplasmic 1
103.743.884	0.6795944		CCDC93	coiled-coil domain containing 93
103.586.861		11.022.591	SDF2L1	stromal cell derived factor 2 like 1
103.533.532	0.874812		CREB3L2	cAMP responsive element binding protein 3 like
10.348.023	0.858324		RPAP3	RNA polymerase II associated protein 3
10.346.101		11.446.676	POGK	pogo transposable element with KRAB domain
103.428.843		-12.908.343		
103.375.285	0.7947761		PIGT	phosphatidylinositol glycan anchor biosynthesi
103.304.283		-14.231.919	ALAS1	5'-aminolevulinate synthase 1
103.296.783	0.916852		PUF60	poly(U) binding splicing factor 60
103.232.761	-0.924284		SPSB3	splA/ryanodine receptor domain and SOCS box
103.189.675	0.6278795		SNF8	SNF8, ESCRT-II complex subunit
103.092.454	0.5811349		RPS20	ribosomal protein S20
103.075.186	0.9769924		ANKRD27	ankyrin repeat domain 27
103.055.879		12.470.017	AZIN1	antizyme inhibitor 1
103.029.548		2.230.858	SULT1C2	sulfotransferase family 1C member 2
10.294.837		15.833.148	TKT	transketolase
102.931.087	0.7931057		COIL	coilin
10.288.527	0.9617172		NARF	nuclear prelamin A recognition factor
102.872.192	0.9037059		PDCD10	programmed cell death 10
102.854.526	0.9957167		BUB1	BUB1 mitotic checkpoint serine/threonine kina
102.827.358	0.5107512		FTH1P5	ferritin heavy chain 1 pseudogene 5
102.774.054	0.7453349		CNOT6	CCR4-NOT transcription complex subunit 6
102.755.518		12.664	GYG1	glycogenin 1

102.750.271	0.8300346		FAM20B	family with sequence similarity 20 member B
102.662.607		1.403.462	P4HA2	prolyl 4-hydroxylase subunit alpha 2
102.630.168		-15.215.283	PCK2	phosphoenolpyruvate carboxykinase 2, mitocho
102.616.384		-15.154.487	ALDH6A1	aldehyde dehydrogenase 6 family member A1
102.591.923	0.8964261		TPD52L2	tumor protein D52 like 2
10.254.738	0.7651222		TUBB3	tubulin beta 3 class III
102.537.849		-11.556.782	PON1	paraoxonase 1
102.498.214	0.6672353		CUTA	cutA divalent cation tolerance homolog
102.482.694		-14.679.223	ACADSB	acyl-CoA dehydrogenase, short/branched chair
102.461.152		-14.595.592	PEMT	phosphatidylethanolamine N-methyltransferas
102.440.405	0.9332463		NEMP1	nuclear envelope integral membrane protein 1
102.356.516	0.9469399		FAM49B	family with sequence similarity 49 member B
102.282.241	0.9400295		MTERF3	mitochondrial transcription termination factor 3
102.268.143	0.9148184		MRPS16	mitochondrial ribosomal protein S16
102.181.006	0.5804796		DDX1	DEAD/H-box helicase 1
102.171.476	-0.7596556		SIGLEC1	sialic acid binding Ig like lectin 1
102.152.196		-18.116.924	AZGP1	alpha-2-glycoprotein 1, zinc-binding
10.214.984	0.7567233		METTL18	methyltransferase like 18
102.133.554	0.7980906		MIS18A	MIS18 kinetochore protein A
102.121.613	0.8051921		UBR5	ubiquitin protein ligase E3 component n-recogr
102.095.061		36.601.641	AKR1B10	aldo-keto reductase family 1 member B10
102.049.103	0.4122357		RPL37	ribosomal protein L37
102.016.889		-25.557.872	ADH1B	alcohol dehydrogenase 1B (class I), beta polype
101.957.471		-13.669.315	MST1L	macrophage stimulating 1 like
101.892.074	-0.9348591		GPT	glutamic--pyruvic transaminase
101.831.028		12.524.462	MOSPD1	motile sperm domain containing 1
101.771.386		-16.498.017	CYP4F2///C	cytochrome P450 family 4 subfamily F member
10.174.719		-14.654.214	RGN	regucalcin
101.675.583		11.064.142	ILF3	interleukin enhancer binding factor 3
101.660.538	0.5049312		RPS7	ribosomal protein S7
101.637.015		-12.847.556	SRD5A1	steroid 5 alpha-reductase 1
10.162.044	0.7387605		EIF3B	eukaryotic translation initiation factor 3 subunit
101.614.761	0.9143178		SMARCE1	SWI/SNF related, matrix associated, actin depe
101.611.831	0.7873553		SRP19	signal recognition particle 19
101.597.561		10.106.808	PRPF4B	pre-mRNA processing factor 4B
101.470.081	0.6908424		CCT8	chaperonin containing TCP1 subunit 8
101.347.716		10.156.641	RBCK1	RANBP2-type and C3HC4-type zinc finger conta
101.285.917	0.7983522		ZNF318	zinc finger protein 318
101.214.005		12.734.958	SQLE	squalene epoxidase
101.198.473		11.178.794	MRPL42	mitochondrial ribosomal protein L42
101.123.873	0.7985283		FKBP1A	FK506 binding protein 1A
101.098.522		10.178.958	TOMM20	translocase of outer mitochondrial membrane 2
101.035.927	-0.5465123		CD160	CD160 molecule
100.983.539	0.7572801		FLAD1	flavin adenine dinucleotide synthetase 1

100.979.101	0.747658		E2F6	E2F transcription factor 6
100.928.914		-10.575.977	LINC-PINT	long intergenic non-protein coding RNA, p53 inc
100.858.028	0.7245429		TOMM40	translocase of outer mitochondrial membrane 4
100.839.231	0.8093298		SRPK2	SRSF protein kinase 2
100.829.103	0.6112522		TARDBP	TAR DNA binding protein
100.810.004		-13.699.051	ADGRA3	adhesion G protein-coupled receptor A3
100.729.405	0.5733559		NBAS	neuroblastoma amplified sequence
100.660.163	0.6737798		ARL8B	ADP ribosylation factor like GTPase 8B
100.645.177	0.809068		GTF2IP1///(	general transcription factor IIi pseudogene 1///g
100.605.843		13.759.539	GNS	glucosamine (N-acetyl)-6-sulfatase
100.561.304	0.69038		MAPKAPK5	mitogen-activated protein kinase-activated pro
100.519.521		-21.780.308	ADH1A	alcohol dehydrogenase 1A (class I), alpha polyp
100.506.514	-0.7509417		SPSB3	splA/ryanodine receptor domain and SOCS box
100.313.274	0.8361451		TMEM185B	transmembrane protein 185B
100.257.137		-12.090.865	IGFBP4	insulin like growth factor binding protein 4
100.175.295		-14.662.743	CDA	cytidine deaminase
100.151.241	0.4917006		RPL19	ribosomal protein L19
10.011.942	0.9332664		SHC1	SHC adaptor protein 1
100.008.173	0.9019842		GLRX2	glutaredoxin 2
10.000.067	0.8051902		TMED9	transmembrane p24 trafficking protein 9
99.941.203		-14.193.471	CDC14B	cell division cycle 14B
9.992.603	-0.9327435		ZNF330	zinc finger protein 330
99.923.307	0.7079147		SPC25	SPC25, NDC80 kinetochore complex compone
99.861.123		-11.882.798	STAB1	stabilin 1
99.824.244	-0.5402474		SHPK	sedoheptulokinase
99.810.409	0.7605351		C20orf24	chromosome 20 open reading frame 24
99.785.489	-0.8243311		SLC4A4	solute carrier family 4 member 4
99.758.046	0.7735347		CLTA	clathrin light chain A
9.974.756		10.082.161	SEC23B	Sec23 homolog B, coat complex II component
99.725.095	0.8881065		PSMD1	proteasome 26S subunit, non-ATPase 1
99.560.389		10.406.923	PAIP1	poly(A) binding protein interacting protein 1
99.525.627		10.861.816	DNMT1	DNA (cytosine-5-)-methyltransferase 1
99.415.087	0.7976471		TRRAP	transformation/transcription domain associate
99.288.485	0.8362362		TROVE2	TROVE domain family member 2
99.264.159	0.7032742		DOLK	dolichol kinase
99.233.881	0.9951403		NEK4	NIMA related kinase 4
99.226.092	0.8374713		VARS	valyl-tRNA synthetase
99.187.487	0.796001		NIPA2	non imprinted in Prader-Willi/Angelman syndro
9.915.042		-31.644.997	HPD	4-hydroxyphenylpyruvate dioxygenase
99.130.421	0.8197672		NCBP2	nuclear cap binding protein subunit 2
99.044.448		12.744.492	CKLF	chemokine like factor
98.853.197	0.7218275		NIF3L1	NGG1 interacting factor 3 like 1
98.775.722	0.810683		EIF3B	eukaryotic translation initiation factor 3 subuni
9.870.168		-25.381.468	SPP2	secreted phosphoprotein 2

98.609.583	0.7685935		ZWILCH	zwilch kinetochore protein
98.529.691		10.500.478	UXS1	UDP-glucuronate decarboxylase 1
98.411.678		-23.111.071	SAA4	serum amyloid A4, constitutive
98.405.032	-0.7613471		RHEB	Ras homolog enriched in brain
98.386.488	0.8472164		LSM8	LSM8 homolog, U6 small nuclear RNA associat
98.376.461	-0.5736535		TCF21	transcription factor 21
98.363.645	-0.9324178		C19orf66	chromosome 19 open reading frame 66
98.340.181	0.9571208		MFF	mitochondrial fission factor
98.257.308		12.182.084	ADNP	activity dependent neuroprotector homeobox
98.241.079	0.5738051		SNRPD1	small nuclear ribonucleoprotein D1 polypeptid
98.182.427	0.8928903		PAXIP1	PAX interacting protein 1
98.153.742	0.7816966		LARP4B	La ribonucleoprotein domain family member 4
981.195	0.9221723		ZNF207	zinc finger protein 207
98.091.821	0.8022985		GGCT	gamma-glutamylcyclotransferase
97.992.898	0.6431636		EIF5B	eukaryotic translation initiation factor 5B
97.939.817		10.076.165	MAP4K4	mitogen-activated protein kinase kinase kinase
97.917.818	0.6812247		PSME3	proteasome activator subunit 3
97.912.874	-0.9958771		SLC23A2	solute carrier family 23 member 2
97.874.814		10.035.954	CLN3	CLN3, battenin
97.740.472	0.8473787		RPL8	ribosomal protein L8
97.708.791	0.87413		ABCC10	ATP binding cassette subfamily C member 10
97.676.457	-0.5959058		KLHL36	kelch like family member 36
97.650.724	0.8149968		BCAP31	B-cell receptor-associated protein 31
97.640.071	0.7181423		RALY	RALY heterogeneous nuclear ribonucleoprotein
97.632.249	0.5347893		STARD7	StAR related lipid transfer domain containing 7
97.609.146		-10.956.661	LOC72789	similar to phosphodiesterase 4D interacting pro
97.592.894	0.8821028		TBCB	tubulin folding cofactor B
9.752.537		-14.894.288	SATB1	SATB homeobox 1
97.455.685		-15.864.301	ACSL1	acyl-CoA synthetase long-chain family membe
97.408.558	0.8760401		WDR26	WD repeat domain 26
97.358.594	0.7766933		RNASEH1	ribonuclease H1
97.331.903	0.8794031		MRS2	MRS2, magnesium transporter
97.324.497	-0.9776175		MGMT	O-6-methylguanine-DNA methyltransferase
97.287.515	0.7493879		EIF2S1	eukaryotic translation initiation factor 2 subuni
97.266.538	-0.9849346		FAAH	fatty acid amide hydrolase
97.254.613	-0.4844995		GAGE2C///	G antigen 2C///G antigen 1
97.223.055		-10.677.353	ID2B///ID2	inhibitor of DNA binding 2B, HLH protein (pseu
97.217.206	0.9559627		ARPC5	actin related protein 2/3 complex subunit 5
97.181.017	0.8487515		EIF3H	eukaryotic translation initiation factor 3 subuni
97.142.407	0.5742282		YWHAB	tyrosine 3-monooxygenase/tryptophan 5-mono
97.091.928		-15.135.643	PRODH2	proline dehydrogenase 2
97.079.112	0.9371323		SEC23B	Sec23 homolog B, coat complex II component
9.704.517	0.9455332		NME7	NME/NM23 family member 7
96.975.035		-13.231.679	EXPH5	exophilin 5

96.966.196	0.7191433		CDC25C	cell division cycle 25C
96.966.168	0.9836784		FTH1	ferritin heavy chain 1
96.931.503		14.115.003	YWHAZ	tyrosine 3-monooxygenase/tryptophan 5-mono
96.922.099	0.7691476		ZNF623	zinc finger protein 623
96.859.371	0.8153104		TBCB	tubulin folding cofactor B
96.854.874		-13.585.029	DPT	dermatopontin
96.853.336	0.8442		USP14	ubiquitin specific peptidase 14
96.842.513	0.5681562		GPN1	GPN-loop GTPase 1
968.305	0.878581		STX6	syntaxin 6
9.679.872	0.9446432		WSB2	WD repeat and SOCS box containing 2
96.674.224		-14.332.869	SRD5A1	steroid 5 alpha-reductase 1
96.653.686		-18.633.569	C8B	complement component 8, beta polypeptide
96.636.331	-0.6595312		SUN2	Sad1 and UNC84 domain containing 2
96.555.648	0.8098005		EIF3D	eukaryotic translation initiation factor 3 subunit
96.547.445	0.877424		RAB22A	RAB22A, member RAS oncogene family
96.456.007	0.7342937		UTP6	UTP6, small subunit processome component
96.442.625	-0.6943639		NFX1	nuclear transcription factor, X-box binding 1
96.428.957	-0.8658515		C21orf33	chromosome 21 open reading frame 33
96.423.902	0.9514052		KIF11	kinesin family member 11
96.382.118	0.7859375		KLHL7	kelch like family member 7
96.369.329		-15.778.965	TRIM22	tripartite motif containing 22
96.352.962	0.660386		LOC64815	similar to ataxia telangiectasia and Rad3 relate
96.324.254	0.7918202		IPO9	importin 9
96.256.989	-0.5638609		PLA2G5	phospholipase A2 group V
96.239.647	0.8749568		AP3B1	adaptor related protein complex 3 beta 1 subur
96.233.778	0.6596063		KHDRBS1	KH RNA binding domain containing, signal trans
96.228.094	0.9223852		RECQL	RecQ like helicase
96.208.563	0.738639		GTPBP2	GTP binding protein 2
96.078.088		-31.110.917	SDS	serine dehydratase
96.035.174		10.209.622	RALA	RALA Ras like proto-oncogene A
95.962.715		-1.321.657	GABARAPL	GABA type A receptor associated protein like 1
95.886.717	-0.8967185		PXDC1	PX domain containing 1
95.880.609	0.8065607		SLC26A2	solute carrier family 26 member 2
95.863.396		-11.866.515	SLCO2B1	solute carrier organic anion transporter family r
95.851.392		-22.511.253	TDO2	tryptophan 2,3-dioxygenase
95.753.118		-18.866.266	SOCS2	suppressor of cytokine signaling 2
95.733.565	0.7483482		LRRC42	leucine rich repeat containing 42
95.703.001	0.8244784		PTRH2	peptidyl-tRNA hydrolase 2
95.695.523	0.7898629		JADE3	jade family PHD finger 3
95.692.582		-17.528.594	HPX	hemopexin
95.595.045	-0.7649124		FEZ1	fasciculation and elongation protein zeta 1
95.585.413	0.5651501		UQCRB	ubiquinol-cytochrome c reductase binding prot
95.544.805	0.971086		TUBB	tubulin beta class I
95.529.223	0.9529278		DCK	deoxycytidine kinase

95.526.885		-24.282.082	BHMT	betaine--homocysteine S-methyltransferase
95.517.949	0.5717914		COX6B1	cytochrome c oxidase subunit 6B1
95.497.873	0.8141331		EIF3B	eukaryotic translation initiation factor 3 subunit
95.326.551	-0.7017049		RAMP3	receptor (G protein-coupled) activity modifying
95.325.756	0.7051246		BCOR	BCL6 corepressor
95.313.658	-0.774772		AQP7	aquaporin 7
95.270.006	-0.7140733			
95.248.723	0.6778256		BANF1	barrier to autointegration factor 1
95.229.722		-12.203.985	PRELP	proline and arginine rich end leucine rich repea
95.215.528	0.9745926		GNPDA1	glucosamine-6-phosphate deaminase 1
95.149.932		-27.073.779	NNMT	nicotinamide N-methyltransferase
95.147.806		-14.099.579	SLC6A12	solute carrier family 6 member 12
95.138.992	0.6568209		RTFDC1	replication termination factor 2 domain contain
95.067.916	-0.6969272		SPG7	SPG7, paraplegin matrix AAA peptidase subunit
95.062.677	0.9529536		CETN2	centrin 2
95.044.836	-0.9041266		MFAP4	microfibrillar associated protein 4
9.501.573	0.8207966		HEXB	hexosaminidase subunit beta
95.004.759	0.7894376		TBCB	tubulin folding cofactor B
94.984.944	0.7848757		ELAVL1	ELAV like RNA binding protein 1
94.925.856	0.5991054		SRP14	signal recognition particle 14
94.840.775	-0.4202258		ADRA1A	adrenoceptor alpha 1A
94.750.509		-15.496.863	HPX	hemopexin
94.736.202		10.083.651	EMC2	ER membrane protein complex subunit 2
94.687.713		-11.326.188	ID2	inhibitor of DNA binding 2, HLH protein
94.667.205	0.5945718		RPLP0	ribosomal protein lateral stalk subunit P0
94.631.375	0.8480567		HDAC2	histone deacetylase 2
94.622.341	0.6628964		HNRNPC	heterogeneous nuclear ribonucleoprotein C (C1
94.589.016		10.913.146	SCRIB	scribbled planar cell polarity protein
94.573.485	0.7911021		BUD31	BUD31 homolog
94.564.844	-0.5377636		LRRN3	leucine rich repeat neuronal 3
94.503.459	0.8955354		FAM21D///I	family with sequence similarity 21, member D//
94.470.973		-14.453.286	ABHD2	abhydrolase domain containing 2
94.396.563	-0.7675212		FAXDC2	fatty acid hydroxylase domain containing 2
94.391.837	0.7428342		UMPS	uridine monophosphate synthetase
94.342.251	0.9395352		CD2AP	CD2 associated protein
94.327.069	-0.9150066		MASP1	mannan binding lectin serine peptidase 1
94.204.664		-21.082.888	JCHAIN	joining chain of multimeric IgA and IgM
941.613	0.6151958		UCHL5	ubiquitin C-terminal hydrolase L5
94.151.755	-0.3832419		MBL1P	mannose binding lectin 1, pseudogene
94.125.927	-0.7079261		SAT1	spermidine/spermine N1-acetyltransferase 1
94.054.859		-10.209.377	MIR22HG	MIR22 host gene
93.951.391	0.750623		YEATS2	YEATS domain containing 2
93.950.447	0.7012581		NF2	neurofibromin 2
93.948.249	-0.9584072		SIRT5	sirtuin 5

93.941.835	-0.7936358		ANK3	ankyrin 3, node of Ranvier (ankyrin G)
93.932.069		12.777.529	SERPINB1	serpin family B member 1
93.864.274		-12.803.807	PHGDH	phosphoglycerate dehydrogenase
93.800.621	-0.6709996		SPTBN2	spectrin beta, non-erythrocytic 2
93.781.094	-0.6575714		SORBS3	sorbin and SH3 domain containing 3
93.690.803		12.399.538	TFRC	transferrin receptor
93.682.049	0.7191294		STIL	SCL/TAL1 interrupting locus
93.669.686	0.692588		RPS10P4///	ribosomal protein S10 pseudogene 4///ribosom
93.669.177		-10.149.927	ZCCHC24	zinc finger CCHC-type containing 24
93.662.772		10.177.585	SYNCRIP	synaptotagmin binding cytoplasmic RNA intera
93.655.091	0.7188108		C17orf75	chromosome 17 open reading frame 75
93.608.581	0.9194511		RUSC1	RUN and SH3 domain containing 1
93.595.531	0.9260502		GLA	galactosidase alpha
9.351.624		10.994.397	CDC7	cell division cycle 7
93.464.655		-12.598.518	MST1L	macrophage stimulating 1 like
93.441.085		-10.766.407	HADH	hydroxyacyl-CoA dehydrogenase
93.431.271		19.128.272	FADS1	fatty acid desaturase 1
93.426.634	0.888614		SMC2	structural maintenance of chromosomes 2
93.393.045	0.9181994		VPS13B	vacuolar protein sorting 13 homolog B
93.361.236	0.7598301		RPIA	ribose 5-phosphate isomerase A
93.360.243	0.7149554		TMCO1	transmembrane and coiled-coil domains 1
93.319.668	0.8110923		LOC10537	uncharacterized LOC105373045///sterol regula
93.269.837		10.086.052	DEK	DEK proto-oncogene
93.233.179		-29.290.339	HSD11B1	hydroxysteroid 11-beta dehydrogenase 1
93.219.553	0.7131373		RFC2	replication factor C subunit 2
93.173.098	-0.5148611		GLI3	GLI family zinc finger 3
93.161.153		10.896.382	PLEKHF2	pleckstrin homology and FYVE domain containi
9.314.549	0.6350042		PSMB3	proteasome subunit beta 3
93.047.694	0.8786209		TRIM24	tripartite motif containing 24
93.013.447		-13.469.885	SERPINF2	serpin family F member 2
9.298.969	0.8772792		LAMP2	lysosomal associated membrane protein 2
92.980.879	0.7610876		UBAP2L	ubiquitin associated protein 2 like
92.927.341		-13.517.394	PLIN1	perilipin 1
9.291.416	0.6089799		C14orf166	chromosome 14 open reading frame 166
92.911.447	-0.8969983		SCP2	sterol carrier protein 2
92.894.289	0.8936311		ATP6AP1	ATPase H+ transporting accessory protein 1
9.287.845	-0.9508521		SEC24B	SEC24 homolog B, COPII coat complex compor
92.839.005		1.067.473	C6orf48	chromosome 6 open reading frame 48
92.838.007	0.7799031		MKI67	marker of proliferation Ki-67
92.775.355	0.6390094		SNRPB2	small nuclear ribonucleoprotein polypeptide B2
92.760.033		-19.401.863	HBB	hemoglobin subunit beta
92.728.772	0.884134		SLC1A4	solute carrier family 1 member 4
92.584.217		-13.616.276	PTGIS	prostaglandin I2 (prostacyclin) synthase
92.553.982	0.9634602		SMAD2	SMAD family member 2

92.549.022	0.6051807		AURKB	aurora kinase B
92.528.135		-11.860.348	SHBG	sex hormone binding globulin
92.523.858	0.8360245		SP3	Sp3 transcription factor
92.417.511		-14.219.404	MPDZ	multiple PDZ domain crumbs cell polarity comp
92.377.184	-0.7554068		SFRP5	secreted frizzled related protein 5
92.309.897	0.8641189		HGS	hepatocyte growth factor-regulated tyrosine kin
9.230.693	0.8415409		FANCG	Fanconi anemia complementation group G
92.293.055	0.5069195		RPS16	ribosomal protein S16
92.234.811	-0.4757915		TBXA2R	thromboxane A2 receptor
92.224.177	0.8680284		CCZ1B///C	CCZ1 homolog B, vacuolar protein trafficking ar
92.215.484		-1.113.876	PGM1	phosphoglucomutase 1
92.141.241	0.8381586		COMMD4	COMM domain containing 4
92.134.977	-0.6165678		AZGP1P1	alpha-2-glycoprotein 1, zinc-binding pseudoger
92.130.414		10.274.571	ACACA	acetyl-CoA carboxylase alpha
92.116.855	0.6802684		DCTPP1	dCTP pyrophosphatase 1
9.210.871	-0.7766182		CYP2A13	cytochrome P450 family 2 subfamily A member
92.102.099	-0.5506831		CFLAR	CASP8 and FADD like apoptosis regulator
92.085.836	-0.438001		LHX2	LIM homeobox 2
92.080.808	0.6824265		TAF6	TATA-box binding protein associated factor 6
92.034.535	0.7978501		CDC27	cell division cycle 27
91.986.554	0.8996902		PHB	prohibitin
91.944.637		11.666.049	PDIA6	protein disulfide isomerase family A member 6
91.934.361	0.8018861		NSL1	NSL1, MIS12 kinetochore complex component
91.931.817	0.8635276		TPGS2	tubulin polyglutamylase complex subunit 2
91.920.907	0.8883547		SMARCA4	SWI/SNF related, matrix associated, actin depe
91.889.706	0.5883373		RPLP0	ribosomal protein lateral stalk subunit P0
91.885.883	0.9559961		CDC6	cell division cycle 6
91.865.398	0.7653489		IFT52	intraflagellar transport 52
91.856.494	0.6347592		ALMS1	ALMS1, centrosome and basal body associated
91.845.547		-12.998.066	SLCO2B1	solute carrier organic anion transporter family r
91.828.293		-15.672.608	TKFC	triokinase and FMN cyclase
91.827.057	0.5849946		YTHDF1	YTH N6-methyladenosine RNA binding protein [illegible]
91.815.823	0.8489112		ZFP64	ZFP64 zinc finger protein
91.752.851	0.6148144		FLOT1	flotillin 1
91.747.829	0.7666945		DYNC1H1	dynein cytoplasmic 1 heavy chain 1
9.173.558	0.7138785		ENSA	endosulfine alpha
91.718.638	0.8264342		UBE2A	ubiquitin conjugating enzyme E2 A
91.716.174		-11.765.083	SERPING1	serpin family G member 1
91.708.032	0.6293855		NDUFS6	NADH:ubiquinone oxidoreductase subunit S6
91.638.111		-16.271.127	PCOLCE	procollagen C-endopeptidase enhancer
91.617.879	-0.6634504		TRANK1	tetratricopeptide repeat and ankyrin repeat con
91.605.719		10.283.144	TRIM24	tripartite motif containing 24
91.586.542		-19.831.393	HBB	hemoglobin subunit beta
9.158.303		10.646.047	DESI2	desumoylating isopeptidase 2

9.156.575	0.7507778		TIPIN	TIMELESS interacting protein
91.541.971		14.236.886	TP53I3	tumor protein p53 inducible protein 3
91.540.547		-15.457.506	EHHADH	enoyl-CoA, hydratase/3-hydroxyacyl CoA dehyc
91.484.886		-15.182.853	PSD3	pleckstrin and Sec7 domain containing 3
91.377.466	0.9607866		CHMP3	charged multivesicular body protein 3
9.135.523	0.5479566		MED24	mediator complex subunit 24
91.316.581	0.7636552		TPGS2	tubulin polyglutamylase complex subunit 2
91.248.599	0.8595817		OIP5	Opa interacting protein 5
91.202.688	0.5029303		RAC1	ras-related C3 botulinum toxin substrate 1 (rho
91.182.446	0.7405755		LSM14A	LSM14A, mRNA processing body assembly fact
91.149.865	-0.3495688			
91.135.839	0.8370406		CAPZA2	capping actin protein of muscle Z-line alpha sul
91.062.644	0.9861754		TRAPPC4	trafficking protein particle complex 4
91.036.771		12.688.804	ITGA6	integrin subunit alpha 6
91.014.919		10.033.476	SMC3	structural maintenance of chromosomes 3
9.100.882	-0.9198735		PINK1	PTEN induced putative kinase 1
90.971.828	0.6114612		HSP90AA1	heat shock protein 90 alpha family class A mem
90.941.644	-0.8359374		NCOR1P1/,	nuclear receptor corepressor 1 pseudogene 1//
90.923.964	0.5879156		RPLP0	ribosomal protein lateral stalk subunit P0
90.876.809		10.357.951	HSPH1	heat shock protein family H (Hsp110) member
90.873.436	0.7281158		LASP1	LIM and SH3 protein 1
90.869.893	-0.7341388		PIGV///GFR	phosphatidylinositol glycan anchor biosynthesi
90.820.696	0.6429102		COX6C	cytochrome c oxidase subunit 6C
90.818.514		10.622.634	PRELID3B	PRELI domain containing 3B
90.742.673		16.250.408	CLGN	calmegin
9.069.485		10.539.131	HELLS	helicase, lymphoid-specific
90.678.824		-11.609.745	ZC3H13	zinc finger CCCH-type containing 13
90.598.985		-14.229.161	MS4A6A	membrane spanning 4-domains A6A
90.596.673		10.237.992	PARP12	poly(ADP-ribose) polymerase family member 1
90.569.296	0.8053143		ESM1	endothelial cell specific molecule 1
90.559.136	0.7294189		UGGT1	UDP-glucose glycoprotein glucosyltransferase
90.559.035	0.7265806		GTF3C3	general transcription factor IIIC subunit 3
90.541.783	0.8259574		TCERG1	transcription elongation regulator 1
90.511.712	0.9045633		TUBB	tubulin beta class I
90.509.323		-15.823.809	CBS	cystathionine-beta-synthase
90.485.527		-2.525.539	ETNPPL	ethanolamine-phosphate phospho-lyase
90.432.326	0.6862127		PRPF6	pre-mRNA processing factor 6
90.397.105	0.6193517		E2F3	E2F transcription factor 3
90.337.328		-11.408.605	MST1	macrophage stimulating 1
90.251.394	0.5688328		UBL4A	ubiquitin like 4A
90.203.748	0.8556159		SAMM50	SAMM50 sorting and assembly machinery com
90.193.532	-0.8404808		CPED1	cadherin like and PC-esterase domain containi
90.158.813		10.336.146	HSPA13	heat shock protein family A (Hsp70) member 1
90.150.129	0.9368751		DONSON	downstream neighbor of SON

9.006.964	0.8040082		TMEM97	transmembrane protein 97
90.055.449	0.8407216		SNRPA1	small nuclear ribonucleoprotein polypeptide A'
9.002.097	0.7664425		DAD1	defender against cell death 1
89.973.042		-24.921.008	CFHR4	complement factor H related 4
89.920.467		-1.343.222	AGL	amylo-alpha-1, 6-glucosidase, 4-alpha-glucanc
89.914.965	0.6430505		MAGED2	MAGE family member D2
89.877.905	-0.5618444		PRELP	proline and arginine rich end leucine rich repea
89.862.301	0.8848348		U2SURP	U2 snRNP associated SURP domain containing
89.849.728		-16.595.608	ANK3	ankyrin 3, node of Ranvier (ankyrin G)
89.846.866	-0.9986882		C19orf66	chromosome 19 open reading frame 66
89.810.919	0.9276354		PPP2R5A	protein phosphatase 2 regulatory subunit B'alpl
89.724.105		12.261.202	CDKN2A	cyclin dependent kinase inhibitor 2A
89.711.037	0.8163984		RRP1B	ribosomal RNA processing 1B
89.710.161	0.8622719		PIGC	phosphatidylinositol glycan anchor biosynthesi
8.970.885		10.038.163	ADSS	adenylosuccinate synthase
89.678.066	0.8375575		GPAA1	glycosylphosphatidylinositol anchor attachmer
89.649.679	0.9954789		SWAP70	SWAP switching B-cell complex 70kDa subunit
89.608.086	0.8997573		STK24	serine/threonine kinase 24
89.564.177	0.6927294		SFPQ	splicing factor proline and glutamine rich
89.422.516	0.9728008		PRUNE1	prune exopolyphosphatase
89.419.999	0.3659415		RPL37A///P	ribosomal protein L37a///polo like kinase 1
89.418.487	0.6551377		MORC2	MORC family CW-type zinc finger 2
89.372.711		11.416.579	NAP1L1	nucleosome assembly protein 1 like 1
8.936.329	0.608612		EIF4G2	eukaryotic translation initiation factor 4 gamma
89.320.092		-13.654.256	MXRA5	matrix remodeling associated 5
89.296.979	0.7032165		CDCA3	cell division cycle associated 3
8.929.392		-16.933.946	PON3	paraoxonase 3
89.236.746	0.7286458		TEX10	testis expressed 10
89.218.338		-21.249.759	MBL2	mannose binding lectin 2
89.172.203		-14.709.365	ENPEP	glutamyl aminopeptidase
89.088.911		12.444.171	CANX	calnexin
89.077.963		-13.100.827	PIK3R1	phosphoinositide-3-kinase regulatory subunit 1
89.037.779	0.7594157		SSR1	signal sequence receptor subunit 1
89.023.876	0.6094669		RPLP0	ribosomal protein lateral stalk subunit P0
89.010.196	0.6663853		TTC27	tetratricopeptide repeat domain 27
89.000.189	0.7558874		SUPT16H	SPT16 homolog, facilitates chromatin remodeli
88.992.757		10.944.255	ALDOA	aldolase, fructose-bisphosphate A
8.899.179	0.6244491		C11orf57	chromosome 11 open reading frame 57
88.983.427	0.8441762		CSNK2A1	casein kinase 2 alpha 1
88.875.612	0.918518		BARD1	BRCA1 associated RING domain 1
88.867.412	0.7158537		PLOD3	procollagen-lysine,2-oxoglutarate 5-dioxygenas
88.845.651	0.6659834		SLC39A1	solute carrier family 39 member 1
88.822.825	-0.4478197		BAIAP2	BAI1 associated protein 2
887.501	0.8703338		RPS5P8///F	ribosomal protein S5 pseudogene 8///ribosoma

88.694.233	0.8731103		RFC1	replication factor C subunit 1
88.648.774	0.9721625		NPM1	nucleophosmin
88.598.908		-17.017.115	RNASE4///ʻ	ribonuclease A family member 4///angiogenin
88.578.863	0.7764807		HAX1	HCLS1 associated protein X-1
88.568.355	0.818287		EHD4	EH domain containing 4
88.560.361	0.7741003		UQCRH	ubiquinol-cytochrome c reductase hinge protei
88.524.935	-0.6994908		NDEL1	nudE neurodevelopment protein 1 like 1
88.476.227		10.223.099	TBCE	tubulin folding cofactor E
88.472.786	-0.9437218		SUOX	sulfite oxidase
88.421.574	0.7020157		DUS4L	dihydrouridine synthase 4 like
88.401.135	-0.8083564		FXYD6	FXYD domain containing ion transport regulato
88.378.323	0.7866706		PPP1CC	protein phosphatase 1 catalytic subunit gamm
88.354.291	0.6999213		NUPL2	nucleoporin like 2
88.334.333	0.6497674		FUBP1	far upstream element binding protein 1
88.333.435	0.7724885		TK1	thymidine kinase 1
88.310.605	0.6187395		ARPP19	cAMP regulated phosphoprotein 19
88.258.406		-21.155.953	HBB	hemoglobin subunit beta
8.823.786	0.9534101		GNL3	G protein nucleolar 3
88.204.273	0.7411924		BMI1	BMI1 proto-oncogene, polycomb ring finger
88.153.931		10.052.996	MYO5C	myosin VC
88.148.766		-17.234.664	ADIRF	adipogenesis regulatory factor
88.128.536	-0.9392227		PAIP2B	poly(A) binding protein interacting protein 2B
88.114.795		12.310.799	RAP2A	RAP2A, member of RAS oncogene family
88.078.314		19.136.338	IGF2BP3	insulin like growth factor 2 mRNA binding protei
88.073.151	0.8120934		NAE1	NEDD8 activating enzyme E1 subunit 1
88.050.076	0.7717491		DUT	deoxyuridine triphosphatase
88.041.634		-14.786.233	RIDA	reactive intermediate imine deaminase A homo
88.013.371		11.943.981	ASPH	aspartate beta-hydroxylase
88.010.728		10.474.013	ALDOA	aldolase, fructose-bisphosphate A
87.965.922	-0.7007736		ARMC6	armadillo repeat containing 6
87.961.919	0.7109466		TGS1	trimethylguanosine synthase 1
87.945.337	-0.3968741		CBFA2T3	CBFA2/RUNX1 translocation partner 3
87.919.221	0.535323		RIC8A	RIC8 guanine nucleotide exchange factor A
87.908.607	0.7638811		COPS8	COP9 signalosome subunit 8
87.881	0.7256938		ACTR3	ARP3 actin related protein 3 homolog
8.786.102		11.098.408	GYG1	glycogenin 1
87.814.516	0.8794791		RAB22A	RAB22A, member RAS oncogene family
87.787.613		-11.402.089	FAH	fumarylacetoacetate hydrolase
87.785.719	0.6712194		NCL	nucleolin
87.739.413		-14.771.369	ECM2	extracellular matrix protein 2
87.712.157	0.9781823		NUAK1	NUAK family kinase 1
8.769.803	0.8636081		TXN	thioredoxin
87.679.857	0.5467719		UQCRB	ubiquinol-cytochrome c reductase binding prot
87.673.359	0.9699828		UBAP2	ubiquitin associated protein 2

87.644.589	0.9069303		TROVE2	TROVE domain family member 2
87.613.777		-12.960.728	ASL	argininosuccinate lyase
87.602.656	0.7385926		TULP4	tubby like protein 4
87.572.659	-0.9509815		MASP1	mannan binding lectin serine peptidase 1
87.484.112		22.855.771	PEG10	paternally expressed 10
87.440.576	-0.8390269		DPT	dermatopontin
87.435.791	0.7608183		NANS	N-acetylneuraminate synthase
87.411.218	0.6357421		SLC26A6	solute carrier family 26 member 6
87.373.866	0.9596498		NPC2	NPC intracellular cholesterol transporter 2
87.321.644		10.509.415	RAB29	RAB29, member RAS oncogene family
87.310.133		11.332.411	SERPINB1	serpin family B member 1
87.305.422	-0.8026609		FCGRT	Fc fragment of IgG receptor and transporter
87.228.254		14.654.372	ADAM9	ADAM metallopeptidase domain 9
87.166.503		-11.530.975	MST1	macrophage stimulating 1
87.141.036	0.6592189		HNRNPA1	heterogeneous nuclear ribonucleoprotein A1
87.125.091		10.786.624	RPS21	ribosomal protein S21
87.077.429	0.8449409		TCP1	t-complex 1
87.063.877		10.376.815	HMGA1	high mobility group AT-hook 1
87.061.625		12.674.138	HEY1	hes related family bHLH transcription factor wit
87.057.093		-16.770.846	UGT2B28	UDP glucuronosyltransferase family 2 member
87.041.857		-1.222.927	SULT1A4///	sulfotransferase family 1A member 4///sulfotra
87.016.754		-12.743.106	HAGH	hydroxyacylglutathione hydrolase
86.954.336	0.7372374		UBAP2	ubiquitin associated protein 2
86.948.909	0.6831282		ALG3	ALG3, alpha-1,3- mannosyltransferase
86.944.505		12.338.101	LPCAT1	lysophosphatidylcholine acyltransferase 1
86.869.449	0.7462251		VPS26A	VPS26, retromer complex component A
86.822.767	0.7115745		MARS	methionyl-tRNA synthetase
86.777.923	0.7593801		UBE2D1	ubiquitin conjugating enzyme E2 D1
86.755.848	0.8689654		WFS1	wolframin ER transmembrane glycoprotein
86.751.049	0.6538435		RPL17	ribosomal protein L17
86.742.113	0.8413784		YY1AP1	YY1 associated protein 1
86.699.213		-24.486.442	NNMT	nicotinamide N-methyltransferase
86.689.495	0.7359572		SMG8	SMG8, nonsense mediated mRNA decay factor
86.668.077	-0.8762158		LYVE1	lymphatic vessel endothelial hyaluronan recept
86.577.675	0.925336		AVL9	AVL9 cell migration associated
86.568.923	0.9007957		CRNKL1	crooked neck pre-mRNA splicing factor 1
86.537.753	0.7200545		SF3A1	splicing factor 3a subunit 1
86.532.081	0.6733649		HNRNPA3/	heterogeneous nuclear ribonucleoprotein A3///
86.454.451	0.9012358		GPAA1	glycosylphosphatidylinositol anchor attachmer
8.640.545	0.8692967		SSB	Sjogren syndrome antigen B
86.402.028	0.528131		RPS27	ribosomal protein S27
86.373.732	0.657844		SHQ1	SHQ1, H/ACA ribonucleoprotein assembly fact
86.371.402	0.5800641		ANGEL2	angel homolog 2
86.340.789		-12.965.026	MPDZ	multiple PDZ domain crumbs cell polarity comp

86.304.627	-0.9288432		PTMS	parathymosin
86.285.232	0.6863832		NELFB	negative elongation factor complex member B
86.253.829		11.498.511	KDM5B	lysine demethylase 5B
86.226.535		-18.689.609	HAO1	hydroxyacid oxidase 1
86.091.089		-31.251.121	CYP2E1	cytochrome P450 family 2 subfamily E member
85.943.299	0.66937		EIF2B2	eukaryotic translation initiation factor 2B subur
85.923.505	0.8660698		SORT1	sortilin 1
85.921.842		10.140.105	IMPDH2	inosine monophosphate dehydrogenase 2
85.911.023	0.5929805		SMG5	SMG5, nonsense mediated mRNA decay factor
85.870.566	0.8991719		ARID4B	AT-rich interaction domain 4B
85.853.224	-0.7391839		SLC25A37	solute carrier family 25 member 37
85.841.339	0.8991695		TMEM183A	transmembrane protein 183A
85.802.636	0.598078		HSF2	heat shock transcription factor 2
85.797.198		-18.111.447	DNAJC12	DnaJ heat shock protein family (Hsp40) membe
85.735.163		-14.096.517	PFKFB1	6-phosphofructo-2-kinase/fructose-2,6-biphos
85.720.556		-14.329.498	CPN2	carboxypeptidase N subunit 2
85.657.889	0.6736995		DDOST	dolichyl-diphosphooligosaccharide--protein gly
85.655.745	0.7313271		MCM4	minichromosome maintenance complex comp
85.642.591	0.5720336		RPS18	ribosomal protein S18
85.576.662	-0.6767515		BMP1	bone morphogenetic protein 1
85.512.759	0.7288641		RNF4	ring finger protein 4
85.508.805		-20.680.683	CYP2B7P	cytochrome P450 family 2 subfamily B member
85.479.291		13.401.723	RAD21	RAD21 cohesin complex component
852.905	0.731606		ZMPSTE24	zinc metallopeptidase STE24
85.275.001	0.9418252		ARPC1A	actin related protein 2/3 complex subunit 1A
85.262.307	-0.5424123		RASGRP2	RAS guanyl releasing protein 2
85.173.992		-10.952.219	NR4A1	nuclear receptor subfamily 4 group A member 1
85.171.098	0.6500466		STAMBP	STAM binding protein
85.165.618	0.7530955		CS	citrate synthase
85.144.668		11.335.667	LGALS8	galectin 8
850.888	0.8494703		RTCA	RNA 3'-terminal phosphate cyclase
85.017.243	0.6694947		ADAR	adenosine deaminase, RNA specific
84.993.307		-14.131.959	GADD45B	growth arrest and DNA damage inducible beta
84.897.441		10.081.762	SQSTM1	sequestosome 1
84.884.552	0.844773		UTP14A	UTP14A small subunit processome component
84.818.647	0.7428815		MAPK6	mitogen-activated protein kinase 6
84.735.346		-15.525.913	ABCA6	ATP binding cassette subfamily A member 6
84.698.845	0.9531171		ELOVL5	ELOVL fatty acid elongase 5
84.697.986	0.6859411		E2F8	E2F transcription factor 8
84.676.034	0.734879		FBXW11	F-box and WD repeat domain containing 11
84.658.862	0.9516817		CAPRIN1	cell cycle associated protein 1
84.609.061	0.8332926		COPA	coatomer protein complex subunit alpha
8.458.868	0.6504535		RPL17	ribosomal protein L17
84.543.381	0.4814153		PARPBP	PARP1 binding protein

84.401.473	0.5620459		NKIRAS2	NFKB inhibitor interacting Ras like 2
84.399.964	0.6453771		PES1	pescadillo ribosomal biogenesis factor 1
84.399.887	0.7011017		TRMT12	tRNA methyltransferase 12 homolog
8.438.523		-10.582.466	ZBTB20	zinc finger and BTB domain containing 20
84.312.042	0.8772205		ALG6	ALG6, alpha-1,3-glucosyltransferase
84.308.051	-0.8536994		KCNMA1	potassium calcium-activated channel subfamil
84.262.817	0.7135145		NAA10	N(alpha)-acetyltransferase 10, NatA catalytic s
84.258.116		-11.523.667	NSUN6	NOP2/Sun RNA methyltransferase family meml
84.238.303		10.234.096	MAP4K4	mitogen-activated protein kinase kinase kinase
84.228.423	0.8600699		OSBPL3	oxysterol binding protein like 3
84.205.311	0.7988288		INTS7	integrator complex subunit 7
84.195.685	0.9448865		TUBB	tubulin beta class I
8.418.007	0.7696939		SEC23B	Sec23 homolog B, coat complex II component
8.417.597	-0.4949687		DMD	dystrophin
84.165.649		-10.231.195	MAGI1	membrane associated guanylate kinase, WW a
84.151.551	0.669629		RALBP1	ralA binding protein 1
84.130.997	0.7622522		DDX41	DEAD-box helicase 41
84.095.209		-18.251.593	NR1I3	nuclear receptor subfamily 1 group I member 3
84.062.664	0.5231053		ADCK2	aarF domain containing kinase 2
84.051.688		10.881.514	SYNJ2	synaptojanin 2
84.001.701	0.5558646		NIPA2	non imprinted in Prader-Willi/Angelman syndro
83.996.834		-10.555.482	CD302	CD302 molecule
8.399.369	-0.6443538		LONRF1	LON peptidase N-terminal domain and ring fing
83.980.961		-1.009.476	SCP2	sterol carrier protein 2
83.943.392	0.84589		COPS5	COP9 signalosome subunit 5
83.900.042	-0.7497193		MAP2K3	mitogen-activated protein kinase kinase 3
83.896.575		10.110.726	IDI1	isopentenyl-diphosphate delta isomerase 1
83.890.907		-11.926.803	CD81	CD81 molecule
83.872.091	0.7249014		URB2	URB2 ribosome biogenesis 2 homolog (S. cerev
83.854.571	-0.9885865		FAS	Fas cell surface death receptor
8.376.488		-20.760.156	APOC4	apolipoprotein C4
83.714.782	0.7470753		FAM21D///I	family with sequence similarity 21, member D/
83.709.747	0.5789391		RAC1	ras-related C3 botulinum toxin substrate 1 (rho
83.701.101	0.7703119		CSNK1D	casein kinase 1 delta
83.617.732		-17.615.481	ALB	albumin
83.607.779	0.9143971		RPL39L	ribosomal protein L39 like
83.583.141	0.8173029		GPAA1	glycosylphosphatidylinositol anchor attachmer
83.560.705	0.6941086		SPCS2	signal peptidase complex subunit 2
83.544.274		-10.719.429	TRIB1	tribbles pseudokinase 1
83.458.515		24.221.932	SFN	stratifin
83.409.793		-14.750.989	CYP3A5	cytochrome P450 family 3 subfamily A member
83.394.216	0.6433407		AP3M2	adaptor related protein complex 3 mu 2 subuni
8.334.646		10.695.161	IDI1	isopentenyl-diphosphate delta isomerase 1
83.313.135	0.7920869		CDC6	cell division cycle 6

83.290.182	0.5854583		POP7	POP7 homolog, ribonuclease P/MRP subunit
83.280.927	-0.9015025		METTL7A	methyltransferase like 7A
83.274.311	0.7494169		FAM134A	family with sequence similarity 134 member A
83.227.167	0.8765263		HTATSF1	HIV-1 Tat specific factor 1
83.222.875	0.8450309		TRIM28	tripartite motif containing 28
83.157.079	0.7651923		CTNNA1	catenin alpha 1
83.154.779	0.4641962		NUP205	nucleoporin 205
83.141.408	-0.7145567		LTBP4	latent transforming growth factor beta binding p
83.112.816	0.5364464		TLK2	tousled like kinase 2
83.079.959		23.978.114		
83.063.517	0.6506244		HSPA4	heat shock protein family A (Hsp70) member 4
82.966.495		10.766.756	IGSF3	immunoglobulin superfamily member 3
82.914.996	-0.7078357		B3GAT1	beta-1,3-glucuronyltransferase 1
82.865.871	0.604846		CSTF2	cleavage stimulation factor subunit 2
82.800.988	-0.8386199		HP	haptoglobin
82.761.543	0.755404		SRP9	signal recognition particle 9
82.682.713	0.5844003		RPL23	ribosomal protein L23
82.642.292	0.7048826		NARS	asparaginyl-tRNA synthetase
82.629.155	0.5833996		TROAP	trophinin associated protein
82.618.332	0.6349285		RPS7P11//,	ribosomal protein S7 pseudogene 11///ribosom
8.261.244		-10.675.319	PDXDC2P	pyridoxal dependent decarboxylase domain co
82.598.188	0.699969		CCT7	chaperonin containing TCP1 subunit 7
82.559.679	0.5526668		ERH	enhancer of rudimentary homolog (Drosophila)
82.516.625		-31.172.293	CYP2E1	cytochrome P450 family 2 subfamily E member
82.487.669	0.6339468		RPL36AP7/	ribosomal protein L36a pseudogene 7///ribosor
82.445.818		-10.929.337	SLC38A2	solute carrier family 38 member 2
82.390.068		-10.243.422	SORBS2	sorbin and SH3 domain containing 2
82.387.528		-11.187.739	SLC38A2	solute carrier family 38 member 2
82.331.122	0.9270218		PPP2R3C	protein phosphatase 2 regulatory subunit B''gar
82.324.382	0.5924418		YEATS4	YEATS domain containing 4
82.254.012	0.9684148		IER5	immediate early response 5
8.224.168	-0.6871891		RBPMS	RNA binding protein with multiple splicing
82.223.573	0.7600954		GNL2	G protein nucleolar 2
82.218.296		1.013.328	CHD7	chromodomain helicase DNA binding protein 7
82.215.126	0.8669087		ACTR3	ARP3 actin related protein 3 homolog
8.220.563	0.6571115		ARHGEF11	Rho guanine nucleotide exchange factor 11
82.178.541	0.6867351		HIKESHI	Hikeshi, heat shock protein nuclear import fact
82.151.823	0.8760668		FAM208B	family with sequence similarity 208 member B
82.144.517	0.7730003		PDIA6	protein disulfide isomerase family A member 6
82.133.977	0.6354837		RAB1A	RAB1A, member RAS oncogene family
82.127.329	0.7782605		PPM1G	protein phosphatase, Mg2+/Mn2+ dependent 1
82.108.389	0.7480916		ICE1	interactor of little elongation complex ELL subu
82.097.582	0.9384357		ARPP19	cAMP regulated phosphoprotein 19
82.061.805	0.7516691		LSM7	LSM7 homolog, U6 small nuclear RNA and mRN

82.016.909	0.8091788		IPO7	importin 7
81.971.455	0.5614428		NAT10	N-acetyltransferase 10
81.957.458	0.6185982		STX6	syntaxin 6
81.938.959	0.8260804		SSB	Sjogren syndrome antigen B
81.934.816		-13.362.557	CTSO	cathepsin O
81.915.656		-10.776.973	QDPR	quinoid dihydropteridine reductase
81.910.422		-18.083.774	SLC22A7	solute carrier family 22 member 7
81.896.677	0.478297		RPS11	ribosomal protein S11
81.895.208		-10.719.825	FOXO1	forkhead box O1
8.185.757	0.7185372		THOC7	THO complex 7
8.184.454	-0.4369503		LIFR	leukemia inhibitory factor receptor alpha
81.833.119		-10.692.996	SLC16A4	solute carrier family 16 member 4
81.822.438	0.941599		CCNA2	cyclin A2
81.802.058	0.7601636			
81.771.008	0.7980408		SLC9A6	solute carrier family 9 member A6
81.731.926	0.7973858		VPS54	VPS54, GARP complex subunit
81.726.809		10.467.421	KDM5B	lysine demethylase 5B
81.693.456	-0.7690443		SLC4A4	solute carrier family 4 member 4
81.672.246		10.744.153	CNPY2	canopy FGF signaling regulator 2
81.667.165		-1.374.887	SORBS2	sorbin and SH3 domain containing 2
81.661.497	0.9264259		TDG	thymine DNA glycosylase
8.159.318	0.8397036		ARPC3	actin related protein 2/3 complex subunit 3
81.592.444		-11.487.575	JUNB	JunB proto-oncogene, AP-1 transcription factor
81.577.727	0.6338436		ADAM10	ADAM metallopeptidase domain 10
81.559.141	0.7985242		APIP	APAF1 interacting protein
81.552.243		10.593.099	BTBD3	BTB domain containing 3
81.550.372	0.8106687		PPP3CB	protein phosphatase 3 catalytic subunit beta
81.506.889	0.7073373		ECD	ecdysoneless cell cycle regulator
81.447.872		-15.126.078	LEPR	leptin receptor
81.441.253	0.6492945		NOL12///TF	nucleolar protein 12///TRIO and F-actin binding
8.142.659	0.4756892		RPL27AP6/	ribosomal protein L27a pseudogene 6///ribosor
81.389.785	0.652488		FANCF	Fanconi anemia complementation group F
81.373.169	0.9090916		PLXND1	plexin D1
81.295.788		-15.939.655	HGD	homogentisate 1,2-dioxygenase
81.276.985	0.8971072		PIGF	phosphatidylinositol glycan anchor biosynthesi
81.269.531	0.7280704		ALG8	ALG8, alpha-1,3-glucosyltransferase
81.238.481	0.6810231		DDX18	DEAD-box helicase 18
81.189.561	0.5187773		RINT1	RAD50 interactor 1
8.118.909	-0.9816562		USO1	USO1 vesicle transport factor
81.147.193	0.7017105		SET	SET nuclear proto-oncogene
81.092.056		10.172.432	RBBP8	RB binding protein 8, endonuclease
8.108.014	0.9702764		ZNF217	zinc finger protein 217
81.054.536	0.7240887		NUP62	nucleoporin 62
81.045.816	0.8423978		CAMSAP2	calmodulin regulated spectrin associated prote

8.101.168	0.7054531		TSG101	tumor susceptibility 101
80.971.512	0.5640444		MGAT4A	mannosyl (alpha-1,3-)-glycoprotein beta-1,4-N
80.958.354	0.6569216		TNPO1	transportin 1
8.092.629	0.483741		RPS27A	ribosomal protein S27a
80.914.152	0.6110641		SNRPD3	small nuclear ribonucleoprotein D3 polypeptide
8.084.084	0.636281		RPL17	ribosomal protein L17
8.082.015	0.7480222		MKI67	marker of proliferation Ki-67
80.800.653		12.227.851	S100A10	S100 calcium binding protein A10
80.780.131		-11.576.099	AIM1	absent in melanoma 1
80.759.442		-15.283.134	TMEM47	transmembrane protein 47
80.690.114	0.8202588		CEP55	centrosomal protein 55
80.623.644	0.536697		LARP4B	La ribonucleoprotein domain family member 4B
80.613.788	0.822349		SEPT8	septin 8
80.605.386	-0.8559827		LOC65299	uncharacterized LOC652993///pleckstrin homo
80.551.014	-0.5281923		MYOM2	myomesin 2
80.527.671	-0.6565385		SLC25A37	solute carrier family 25 member 37
80.497.533		12.316.406	CKAP4	cytoskeleton associated protein 4
8.048.496	0.5689886		CNP	2',3'-cyclic nucleotide 3' phosphodiesterase
80.480.759	0.9898558		MPC2	mitochondrial pyruvate carrier 2
80.403.063		11.238.108	DNAJC6	DnaJ heat shock protein family (Hsp40) membe
80.317.334		-12.115.377	LIME1	Lck interacting transmembrane adaptor 1
80.267.897	0.6939589		TMCO1	transmembrane and coiled-coil domains 1
80.267.713	0.7444998		PAK2	p21 (RAC1) activated kinase 2
80.261.079	0.8896853		MEX3C	mex-3 RNA binding family member C
80.236.159	0.7870927		PSMA7	proteasome subunit alpha 7
8.020.394	0.6701268		CDYL	chromodomain Y-like
80.200.697	-0.5145243		ACACB	acetyl-CoA carboxylase beta
801.607	0.9910616		ASF1A	anti-silencing function 1A histone chaperone
80.143.977	0.9412709		TMEM251	transmembrane protein 251
80.094.121	0.6054764		BYSL	bystin like
80.066.937		-15.188.308	SLC25A15	solute carrier family 25 member 15
80.025.984	0.6291051		RAD1	RAD1 checkpoint DNA exonuclease
79.988.668		10.016.968	MTR	5-methyltetrahydrofolate-homocysteine methy
79.951.148	0.7912126		AHSA1	activator of Hsp90 ATPase activity 1
79.915.229		11.154.911	BLVRA	biliverdin reductase A
79.907.571	0.6114565		POLA1	DNA polymerase alpha 1, catalytic subunit
79.893.172	0.7978903		SEH1L	SEH1 like nucleoporin
79.874.792		15.520.145	ASPH	aspartate beta-hydroxylase
79.840.797	-0.6400269		FOXP1	forkhead box P1
79.799.155	0.8282679		SLC33A1	solute carrier family 33 member 1
79.695.228		-11.524.227	COMT	catechol-O-methyltransferase
79.684.906	0.5015108		H3F3AP6//	H3 histone, family 3A, pseudogene 6///H3 histo
79.681.971	0.8813136		TOMM20	translocase of outer mitochondrial membrane
79.643.812	0.7059589		NUP153	nucleoporin 153

79.618.019	0.6599713		DSN1	DSN1 homolog, MIS12 kinetochore complex co
79.569.202		1.766.009	IGF2BP3	insulin like growth factor 2 mRNA binding protei
79.547.291	0.5677222		TUBA3C	tubulin alpha 3c
79.530.735	0.7700373		RNF34	ring finger protein 34
79.507.585	0.6381134		RPL7	ribosomal protein L7
79.491.932	0.7051737		DDX27	DEAD-box helicase 27
79.465.902		-23.774.965	LECT2	leukocyte cell derived chemotaxin 2
79.446.441		-20.464.378	FETUB	fetuin B
79.444.218	-0.7357716		SAT1	spermidine/spermine N1-acetyltransferase 1
7.943.267		-15.797.774	SULT1E1	sulfotransferase family 1E member 1
79.432.073	0.5327953		RPLP2	ribosomal protein lateral stalk subunit P2
79.416.077	0.8170163		MAGED2	MAGE family member D2
79.410.449	-0.8688027		DCAF11	DDB1 and CUL4 associated factor 11
79.356.179		-11.638.581	ETS2	ETS proto-oncogene 2, transcription factor
79.354.068		-18.053.344	CYP2C18	cytochrome P450 family 2 subfamily C member
79.288.946	-0.9474124		CTBS	chitobiase
7.927.571	0.6766818		TRAM1	translocation associated membrane protein 1
79.267.095	0.5634518		PTBP1	polypyrimidine tract binding protein 1
7.924.673		-10.021.963	SULT1A4///	sulfotransferase family 1A member 4///sulfotra
79.235.812	0.615151		PSMA1	proteasome subunit alpha 1
79.181.788	0.6934411		MORC2	MORC family CW-type zinc finger 2
79.166.628		10.249.542	COG5	component of oligomeric golgi complex 5
79.165.099	0.6654802		SLC36A1	solute carrier family 36 member 1
79.124.896	0.600917		MED7	mediator complex subunit 7
79.119.933	0.8139119		TMEM189-U	TMEM189-UBE2V1 readthrough///ubiquitin con
79.114.791	0.6781651		SRPRB	SRP receptor beta subunit
79.059.453	0.8570187		MTX1	metaxin 1
79.017.006	-0.8555666		C14orf159	chromosome 14 open reading frame 159
79.011.374	0.8001168		TUG1	taurine up-regulated 1 (non-protein coding)
79.010.733	0.7760322		ATP2A2	ATPase sarcoplasmic/endoplasmic reticulum C
78.993.762	0.6740063		ARL1	ADP ribosylation factor like GTPase 1
78.977.252	0.582314		PTCD1	pentatricopeptide repeat domain 1
78.886.386	0.5847491		QARS	glutaminyl-tRNA synthetase
78.858.377	0.507769		EFNA4	ephrin A4
78.841.788		-10.579.996	C1S	complement component 1, s subcomponent
78.809.339		10.870.696	ACP1	acid phosphatase 1, soluble
78.775.872	0.5971982		PSMA1	proteasome subunit alpha 1
787.587	-0.4515847		PLCB2	phospholipase C beta 2
78.719.315		-11.498.143	BCKDHB	branched chain keto acid dehydrogenase E1 su
78.707.814		-10.794.436	DUSP6	dual specificity phosphatase 6
7.868.944	-0.6054814		NXF1	nuclear RNA export factor 1
78.658.007	0.7352482		NUP43	nucleoporin 43
78.647.442	0.9241122		RPRD1A	regulation of nuclear pre-mRNA domain contai
78.619.254	0.8271377		EXOSC4	exosome component 4

78.610.486	-0.9793552		CROCCP2/	ciliary rootlet coiled-coil, rootletin pseudogene
78.601.173		-15.111.791	BDH1	3-hydroxybutyrate dehydrogenase, type 1
78.585.504	0.8656154		FAM216A	family with sequence similarity 216 member A
78.573.466	0.6266573		SNW1	SNW domain containing 1
78.526.188		12.377.238	SRI	sorcin
78.522.228	0.6025721		PCGF1	polycomb group ring finger 1
78.506.831	0.7379637		POP5	POP5 homolog, ribonuclease P/MRP subunit
78.474.201	0.5584457		PTBP1	polypyrimidine tract binding protein 1
78.411.137	0.7700159		C1orf27	chromosome 1 open reading frame 27
78.308.008	0.5014746		LOC64328	similar to prothymosin alpha///prothymosin, al
78.190.265	0.6234834		CDK16	cyclin dependent kinase 16
78.188.506		-11.601.867	EPB41L4B	erythrocyte membrane protein band 4.1 like 4B
78.186.392	0.7211348		DPAGT1	dolichyl-phosphate N-acetylglucosaminephos
78.138.805		-1.015.078	PLSCR4	phospholipid scramblase 4
78.132.966	0.7920944		IARS2	isoleucyl-tRNA synthetase 2, mitochondrial
78.096.717		16.834.927	IGF2BP2	insulin like growth factor 2 mRNA binding protei
7.802.312	0.9851037		DLAT	dihydrolipoamide S-acetyltransferase
78.008.232	0.7052968		DYNC1LI1	dynein cytoplasmic 1 light intermediate chain 1
78.005.896	0.6429101		MFN1	mitofusin 1
77.993.097	-0.9346097		SMARCA2	SWI/SNF related, matrix associated, actin depe
77.980.809	-0.4985292		DDX19A///I	DEAD-box helicase 19A///DEAD-box helicase 1
779.376		-12.186.146	DMD	dystrophin
77.903.052	0.7987741		MPV17	MPV17, mitochondrial inner membrane protein
77.870.894	0.8014234		NCBP2	nuclear cap binding protein subunit 2
77.852.202	0.6309753		GTF2E1	general transcription factor IIE subunit 1
77.825.786	0.6431415		VDAC2	voltage dependent anion channel 2
77.808.393		-11.390.823	COMT	catechol-O-methyltransferase
77.779.315	0.8618651		SUMO2	small ubiquitin-like modifier 2
77.718.964	0.6502854		DPP3	dipeptidyl peptidase 3
77.668.746	0.652084		WIPF2	WAS/WASL interacting protein family member
77.667.259	-0.9286205		FRMD4B	FERM domain containing 4B
77.644.715		-18.753.067	IGHM	immunoglobulin heavy constant mu
77.639.772	0.6586045		PSMC6	proteasome 26S subunit, ATPase 6
77.563.402	0.7304721		GNS	glucosamine (N-acetyl)-6-sulfatase
77.562.073	0.5946727		EFCAB11	EF-hand calcium binding domain 11
77.537.137	0.7915976		SLC35E3	solute carrier family 35 member E3
77.459.451	0.6815198		MARK4	microtubule affinity regulating kinase 4
77.421.653		-15.067.534	CYP2C18	cytochrome P450 family 2 subfamily C membe
77.369.661	-0.59977		PLA2G5	phospholipase A2 group V
77.338.958	-0.4868383			
77.235.416	0.5469687		AAGAB	alpha- and gamma-adaptin binding protein
77.225.192	0.5721748		EFL1	elongation factor like GTPase 1
77.224.883	-0.6741583		SCARB2	scavenger receptor class B member 2
77.198.079	0.5917016		SPAST	spastin

77.147.519		1.031.224	SUB1	SUB1 homolog, transcriptional regulator
77.138.397	0.5347488		TMEM160	transmembrane protein 160
77.126.711		-14.169.797	CAT	catalase
77.060.877	-0.7005616		MYO1F	myosin IF
77.030.755		-10.866.257	COBLL1	cordon-bleu WH2 repeat protein like 1
77.019.911	0.6115655		TRMT61B	tRNA methyltransferase 61B
77.012.956	0.7984567		PAK2	p21 (RAC1) activated kinase 2
7.697.978	0.5047137		EIF3K	eukaryotic translation initiation factor 3 subunit
76.967.972	-0.9096899		PCDH9	protocadherin 9
76.855.268	0.5030808		TUBA3D	tubulin alpha 3d
76.836.588	-0.5589333		DMD	dystrophin
76.794.351		-21.680.945	AQP9	aquaporin 9
76.766.888	0.5695482		GOSR2	golgi SNAP receptor complex member 2
76.758.697	0.7319131		MPP6	membrane palmitoylated protein 6
76.744.086		-18.058.054	PZP	PZP, alpha-2-macroglobulin like
767.287		-12.624.442	CDC14B	cell division cycle 14B
76.727.801	0.8765574		STK24	serine/threonine kinase 24
76.722.856		-16.147.956	SHMT1	serine hydroxymethyltransferase 1
76.722.333	0.7026507		DYNC1I2P1	dynein cytoplasmic 1 intermediate chain 2 pseu
76.721.278	0.5760535		COPZ1	coatomer protein complex subunit zeta 1
76.696.571	0.6139761		GTF3C2	general transcription factor IIIC subunit 2
76.654.021		-18.897.569	AGXT	alanine-glyoxylate aminotransferase
76.653.384	0.659664		FAM134A	family with sequence similarity 134 member A
76.543.304	0.7453707		LRPPRC	leucine rich pentatricopeptide repeat containin
76.483.145	0.9250064		NAP1L1	nucleosome assembly protein 1 like 1
76.456.494		-17.575.456	CA2	carbonic anhydrase 2
76.382.859	0.7555796		CKLF	chemokine like factor
76.382.736	0.6357011		JTB	jumping translocation breakpoint
76.299.014	0.6795519		ABCF1	ATP binding cassette subfamily F member 1
76.274.888	0.5415983		DHX15	DEAH-box helicase 15
76.274.061	0.510566		VAPA	VAMP associated protein A
76.250.577		-11.996.256	RBL2	RB transcriptional corepressor like 2
76.203.665	0.700617		PDCD6	programmed cell death 6
76.151.264	-0.9316651		AVPI1	arginine vasopressin induced 1
76.143.866	0.6483062		MRPL24	mitochondrial ribosomal protein L24
76.053.826	0.5620224		NDUFA1	NADH:ubiquinone oxidoreductase subunit A1
76.042.992	-0.5353989		TSPAN9	tetraspanin 9
76.042.773	0.5706016		BRAP	BRCA1 associated protein
75.985.237	0.9350195		PAIP1	poly(A) binding protein interacting protein 1
75.978.853	-0.9022984		FNDC4	fibronectin type III domain containing 4
75.968.768	0.5205471		RPP21	ribonuclease P/MRP subunit p21
75.909.797	-0.528132		HEXIM1	hexamethylene bisacetamide inducible 1
75.907.978	0.8549833		PEX11B	peroxisomal biogenesis factor 11 beta
75.888.389		-1.336.602	TSC22D3	TSC22 domain family member 3

75.801.161	0.8500233		MARS	methionyl-tRNA synthetase
7.573.253	0.5342397		JTB	jumping translocation breakpoint
75.688.962	0.6130362		TATDN2	TatD DNase domain containing 2
75.659.743	-0.4481492		SGMS1	sphingomyelin synthase 1
75.648.993		11.005.097	ACSL3	acyl-CoA synthetase long-chain family member
75.648.518		-10.474.108	PROC	protein C, inactivator of coagulation factors Va
75.647.904		-10.781.223	MUT	methylmalonyl-CoA mutase
75.642.419		-10.126.649	DHTKD1	dehydrogenase E1 and transketolase domain c
75.627.226	0.7847842		HSP90AA1	heat shock protein 90 alpha family class A mem
75.598.822	0.8417908		FAM20B	family with sequence similarity 20 member B
75.590.641	-0.6538609			
75.580.678	0.7305454		RSU1	Ras suppressor protein 1
7.555.113	-0.4664243		TNXB///TN>	tenascin XB///tenascin XA (pseudogene)
75.550.564		-16.428.384	SLC22A7	solute carrier family 22 member 7
75.508.671	-0.3080407		HTR6	5-hydroxytryptamine receptor 6
75.495.961	0.5078768		NCAPH2	non-SMC condensin II complex subunit H2
75.475.911	-0.6904659		WFDC1	WAP four-disulfide core domain 1
75.466.778	-0.6310178		HMBOX1	homeobox containing 1
7.536.946	-0.766867		RANBP10	RAN binding protein 10
75.361.028	-0.99845		FOXO1	forkhead box O1
75.345.302	0.7621696		LSM2	LSM2 homolog, U6 small nuclear RNA and mRN
75.293.031	0.5088387		EIF3K	eukaryotic translation initiation factor 3 subuni
75.249.481		-1.592.099	MTTP	microsomal triglyceride transfer protein
75.142.709	-0.8679075		EPHA2	EPH receptor A2
75.101.074	0.8101481		DIEXF	digestive organ expansion factor homolog (zebr
75.093.567		-10.690.194	MYLK	myosin light chain kinase
75.042.689	0.6149039		SOCS5	suppressor of cytokine signaling 5
74.966.693	-0.9762646		GSDMB	gasdermin B
7.496.203		-11.787.205	ENTPD5	ectonucleoside triphosphate diphosphohydrola
74.886.515	-0.782079		ZCCHC24	zinc finger CCHC-type containing 24
74.862.307		-10.589.701	SLC37A4	solute carrier family 37 member 4
74.808.712	-0.5380322		ABHD18	abhydrolase domain containing 18
7.478.269	0.8666929		FASTKD3	FAST kinase domains 3
74.781.129	-0.8546757		BGN	biglycan
74.768.563	0.7128002		AHCTF1	AT-hook containing transcription factor 1
74.765.767	-0.5583486		PRB2///PRE	proline rich protein BstNI subfamily 2///proline
74.759.396		10.339.525	GBP2	guanylate binding protein 2
74.744.671	0.7319004		EIF3E	eukaryotic translation initiation factor 3 subuni
74.741.424		10.938.506	ODC1	ornithine decarboxylase 1
74.726.679	0.9772592		ALDH18A1	aldehyde dehydrogenase 18 family member A1
74.724.021	0.3456378		ANKRD40	ankyrin repeat domain 40
74.720.176	0.5790233		AGK	acylglycerol kinase
7.469.258	-0.8741921		LHPP	phospholysine phosphohistidine inorganic pyro
74.632.246	0.5522496		NEDD8	neural precursor cell expressed, developmenta

74.628.122	0.3819573		TMA7	translation machinery associated 7 homolog
7.462.402	0.7384886		TOP2B	topoisomerase (DNA) II beta
74.616.581		12.672.391	GSTA4	glutathione S-transferase alpha 4
74.546.242		12.123.692	FKBP11	FK506 binding protein 11
74.539.454	0.5288899		HSPD1	heat shock protein family D (Hsp60) member 1
74.478.165	0.6666329		RAE1	ribonucleic acid export 1
74.474.021		-12.812.736	CYP4F3	cytochrome P450 family 4 subfamily F member
74.431.941	0.6311545		UBP1	upstream binding protein 1 (LBP-1a)
74.373.645	0.7113165		SKP1	S-phase kinase-associated protein 1
74.362.974	-0.5088263		SCARF1	scavenger receptor class F member 1
7.433.808	0.4422487		MED27	mediator complex subunit 27
74.311.814	0.6162806		PUS1	pseudouridylate synthase 1
74.282.591	0.6626051		PARP2	poly(ADP-ribose) polymerase 2
74.252.102	0.540804		BORA	bora, aurora kinase A activator
74.233.311		-10.470.693	DHODH	dihydroorotate dehydrogenase (quinone)
74.224.988	0.5453675		JTB	jumping translocation breakpoint
74.218.441		12.295.116	BAG2	BCL2 associated athanogene 2
74.086.912	0.5884832		NABP2	nucleic acid binding protein 2
74.063.901	0.7544957		DPY19L4	dpy-19 like 4 (C. elegans)
7.403.435	0.7364928		ISG20L2	interferon stimulated exonuclease gene 20 like
74.018.793	0.8425457		ABI1	abl interactor 1
73.932.853	0.630653			
73.927.223	0.5798898		FLOT1	flotillin 1
73.895.773	0.4188254		C20orf27	chromosome 20 open reading frame 27
73.863.748	0.6585593		CDCA8	cell division cycle associated 8
73.838.854	0.8022637		THOC2	THO complex 2
73.760.836	0.9553868		PPARG	peroxisome proliferator activated receptor gam
73.758.933	0.8100105		EGLN1	egl-9 family hypoxia inducible factor 1
7.375.226	-0.8620368		DAPK1	death associated protein kinase 1
73.743.594	0.6918054		LIG1	DNA ligase 1
73.739.862		-13.406.612	FBLN5	fibulin 5
73.736.408	-0.6324923		CAMK2B	calcium/calmodulin dependent protein kinase
73.732.417	0.7976233		YME1L1	YME1 like 1 ATPase
7.373.201	0.5044592		CDK16	cyclin dependent kinase 16
73.701.514	0.7539119		PSMD12	proteasome 26S subunit, non-ATPase 12
73.688.496	0.6370589		SRSF10	serine and arginine rich splicing factor 10
73.668.797	0.7737771		DEGS1	delta 4-desaturase, sphingolipid 1
73.654.715	0.6496246		ELOVL1	ELOVL fatty acid elongase 1
73.623.746	0.5853306		ALMS1	ALMS1, centrosome and basal body associated
73.619.807	0.7266586		PABPC3	poly(A) binding protein cytoplasmic 3
73.592.817	0.4738716		CHAF1A	chromatin assembly factor 1 subunit A
73.590.949	0.4428374		RPL7P52	ribosomal protein L7 pseudogene 52
73.586.913	0.7906194		KDM3A	lysine demethylase 3A
73.564.224		-13.030.488	SRD5A1	steroid 5 alpha-reductase 1

73.553.764	0.5606116		HNRNPUL1	heterogeneous nuclear ribonucleoprotein U like
73.506.676	0.619651		RNF220	ring finger protein 220
73.492.938	0.6559239		RNF115	ring finger protein 115
73.452.513	0.665342		PARP2	poly(ADP-ribose) polymerase 2
73.439.226		10.485.646	ENPP4	ectonucleotide pyrophosphatase/phosphodies
73.379.831		13.041.559	SULT1C2	sulfotransferase family 1C member 2
73.371.398		-10.706.524	ANK3	ankyrin 3, node of Ranvier (ankyrin G)
73.345.414	0.7948785		ENOPH1	enolase-phosphatase 1
73.331.532	0.9434876		SMAGP	small cell adhesion glycoprotein
73.321.039	0.733847		SUMO3	small ubiquitin-like modifier 3
73.310.937	-0.5445188		NPIPB6///N	nuclear pore complex interacting protein family
73.274.542	0.4181001		HCFC1	host cell factor C1
73.254.237	-0.3484048		CD244	CD244 molecule
73.197.679	0.8710446		LOC73183	similar to family with sequence similarity 45, m
73.189.885	0.7030795		CASK	calcium/calmodulin dependent serine protein k
73.186.157	0.748067		TROVE2	TROVE domain family member 2
73.174.794		12.546.195	RRAGD	Ras related GTP binding D
73.127.486	0.5261426		CHCHD2	coiled-coil-helix-coiled-coil-helix domain conta
73.126.714	0.9453681		ESF1	ESF1 nucleolar pre-rRNA processing protein ho
73.083.214		15.205.989	FADS1	fatty acid desaturase 1
73.077.789	0.5493074		MNAT1	MNAT1, CDK activating kinase assembly factor
73.075.502		-12.424.675	ABHD2	abhydrolase domain containing 2
73.064.823		-15.524.357	MRC1	mannose receptor, C type 1
73.047.388	0.6995085		SKIV2L2	Ski2 like RNA helicase 2
72.979.319	0.6646707		AGBL5	ATP/GTP binding protein like 5
72.945.873		-12.341.084	PSD3	pleckstrin and Sec7 domain containing 3
7.291.749	0.7500399		SOX13	SRY-box 13
72.916.941	0.868128		LIMK2	LIM domain kinase 2
72.857.586	0.6647175		METTL3	methyltransferase like 3
72.848.252	-0.8456763		ABAT	4-aminobutyrate aminotransferase
72.845.344	0.7397782		UMPS	uridine monophosphate synthetase
7.284.434	0.6176892		PPP1CB	protein phosphatase 1 catalytic subunit beta
72.824.326	0.9469585		RIPK2	receptor interacting serine/threonine kinase 2
72.812.686	0.426791		RABIF	RAB interacting factor
72.805.821		-11.869.519	ADAMTSL3	ADAMTS like 3
7.275.685	0.7783962		CUL4B	cullin 4B
72.691.874	0.5083274		PTBP1	polypyrimidine tract binding protein 1
72.685.088	0.9792317		ME2	malic enzyme 2
72.590.969	0.4860108		C10orf88	chromosome 10 open reading frame 88
72.590.218		-17.371.356	SLC17A2	solute carrier family 17 member 2
72.535.034	0.8187227		MTDH	metadherin
72.523.419	0.6497715			
7.251.568		11.984.591	TMED3	transmembrane p24 trafficking protein 3
7.247.857	0.6888278		ARPC2	actin related protein 2/3 complex subunit 2

7.246.386	0.6600536		HNRNPAB	heterogeneous nuclear ribonucleoprotein A/B
72.444.451		-13.253.596	AASS	aminoadipate-semialdehyde synthase
72.428.012	0.5497341		YME1L1	YME1 like 1 ATPase
72.382.269	0.5866567		COMMD4	COMM domain containing 4
72.341.945	-0.4829303		KDM6B	lysine demethylase 6B
72.319.039		10.983.217	AIDA	axin interactor, dorsalization associated
723.093	0.7443367		LOC65260	polyadenylate-binding protein 1-like///poly(A) b
72.303.627	0.6021474		DIMT1	DIM1 dimethyladenosine transferase 1 homolo
72.254.336	0.8359512		SMYD2	SET and MYND domain containing 2
72.253.647	-0.8335013		SFXN1	sideroflexin 1
72.240.363	-0.9223493			
72.235.603		10.453.803	TMEM97	transmembrane protein 97
72.227.726	0.5946008		COPB2	coatomer protein complex subunit beta 2
72.206.178		-14.742.038	LIPC	lipase C, hepatic type
72.204.912	0.7913545		CD58	CD58 molecule
72.183.814	0.8020565		BUB3	BUB3, mitotic checkpoint protein
72.113.224	-0.8848413		KLRK1	killer cell lectin like receptor K1
72.095.767	0.590964		CNPY2	canopy FGF signaling regulator 2
72.088.289	0.4547863		QTRT2	queuine tRNA-ribosyltransferase accessory sub
72.087.895	0.4839387		PARG	poly(ADP-ribose) glycohydrolase
72.074.665	0.581777		KXD1	KxDL motif containing 1
72.061.155	0.72104		PSMD11	proteasome 26S subunit, non-ATPase 11
72.054.295	0.925029		LOXL2	lysyl oxidase like 2
72.042.378	0.5023483		POLR3C	RNA polymerase III subunit C
72.029.154	-0.5880785		NAB2	NGFI-A binding protein 2
7.198.918	0.5205425		PRPF31	pre-mRNA processing factor 31
71.962.922	-0.4424992			
7.194.509	0.5699		POLR2D	RNA polymerase II subunit D
71.938.718		10.479.768	ABCC4	ATP binding cassette subfamily C member 4
71.937.714	-0.6002655		IL17RC	interleukin 17 receptor C
71.924.281		1.300.951	HSP90AB1	heat shock protein 90 alpha family class B mem
71.912.881	0.9425581		WASF1	WAS protein family member 1
71.896.689	0.5200239		PPP1R11	protein phosphatase 1 regulatory inhibitor subu
7.187.413	0.6229115		CDCA4	cell division cycle associated 4
71.860.367	-0.6695953		ITSN1	intersectin 1
71.838.044	0.6791632		KPNB1	karyopherin subunit beta 1
7.180.309	0.5653535		EXOSC7	exosome component 7
7.178.487	0.7017693		CSNK1E	casein kinase 1 epsilon
71.744.593	-0.5952369		ARHGEF26	Rho guanine nucleotide exchange factor 26
71.733.183	0.5831059		HARS2	histidyl-tRNA synthetase 2, mitochondrial
71.732.119	0.6065807		POLD1	DAN polymerase delta 1, catalytic subunit
71.710.193	0.4960427		TAF5L	TATA-box binding protein associated factor 5 lik
71.709.853		-12.861.075	MYO10	myosin X
717.006	0.5997428		EIF6	eukaryotic translation initiation factor 6

71.679.585	0.5404028		TAF10	TATA-box binding protein associated factor 10
71.637.142	-0.8461308		PIGV///GFR	phosphatidylinositol glycan anchor biosynthesi
71.624.183	0.5664564		SLC4A1AP	solute carrier family 4 member 1 adaptor protei
71.621.996	0.9505529		BTRC///TPF	beta-transducin repeat containing E3 ubiquitin
71.615.164	0.6574231		SWAP70	SWAP switching B-cell complex 70kDa subunit
71.614.395	0.5347535		CDC45	cell division cycle 45
71.605.852	0.765832		SMARCA4	SWI/SNF related, matrix associated, actin depe
71.585.526	0.5774454		LBHD1	LBH domain containing 1
7.156.824	-0.5537938		METTL7A	methyltransferase like 7A
71.553.619		-15.229.769	PIPOX	pipecolic acid and sarcosine oxidase
71.497.543		10.773.743	CAPG	capping actin protein, gelsolin like
71.494.902	0.7476415		FBXO5	F-box protein 5
71.478.482	0.9449904		BLVRA	biliverdin reductase A
71.453.818	-0.9808609		SUCLG2	succinate-CoA ligase GDP-forming beta subuni
71.422.276	0.6918215		NGRN	neugrin, neurite outgrowth associated
71.402.207	-0.8420024			
71.384.154	0.8330806		RFC5	replication factor C subunit 5
71.365.157	-0.839308		MT1G	metallothionein 1G
71.331.186	0.7299846		KIDINS220	kinase D-interacting substrate 220kDa
71.316.832	-0.6566739		BCKDHA	branched chain keto acid dehydrogenase E1, al
71.248.652	0.9864846		DDOST	dolichyl-diphosphooligosaccharide--protein gly
71.220.286	0.540669		ASF1B	anti-silencing function 1B histone chaperone
7.121.177	0.513239		TTF2	transcription termination factor 2
71.208.251	0.5958695		CASK	calcium/calmodulin dependent serine protein k
71.189.906	0.6578989		JARID2	jumonji and AT-rich interaction domain contain
71.177.552	0.5766806		METTL13	methyltransferase like 13
71.149.533	0.6723997		KIF23	kinesin family member 23
71.117.511	0.8966674		CEP350	centrosomal protein 350
71.109.575	0.9237739		GNB1	G protein subunit beta 1
71.045.655	0.7574967		AGAP1	ArfGAP with GTPase domain, ankyrin repeat and
70.972.674	-0.6705054		RCN3	reticulocalbin 3
70.962.075		-10.347.404	ADI1	acireductone dioxygenase 1
7.092.956	0.6576612		CDC25A	cell division cycle 25A
7.092.941	-0.8147921		CD300A	CD300a molecule
7.091.035	0.3932573		RPL32	ribosomal protein L32
70.889.638	0.5568764		ZBED8	zinc finger BED-type containing 8
70.889.282	-0.4793005		MYO9A	myosin IXA
70.856.808		20.275.305	SOX4	SRY-box 4
70.825.054	-0.708181		HIGD2B///F	HIG1 hypoxia inducible domain family member
70.818.822	0.6880026		SNW1	SNW domain containing 1
70.801.955	0.5351737		KBTBD4	kelch repeat and BTB domain containing 4
70.753.111	-0.6190957		TMEM53	transmembrane protein 53
7.074.351	0.6824163		NDUFAF5//	NADH:ubiquinone oxidoreductase complex ass
70.695.821	0.5364307		CD34	CD34 molecule

70.688.669	0.780748		BLM	Bloom syndrome RecQ like helicase
70.671.996	-0.8175114		SIGLEC1	sialic acid binding Ig like lectin 1
70.622.068		1.309.461	PIR	pirin
70.611.919	-0.3142919		LRAT	lecithin retinol acyltransferase (phosphatidylch
70.591.381	0.7163184		YWHAQ	tyrosine 3-monooxygenase/tryptophan 5-mono
70.578.651		-19.269.453	HAL	histidine ammonia-lyase
70.577.858	0.6449952		VPS35	VPS35 retromer complex component
70.573.459	0.4708684		C7orf25	chromosome 7 open reading frame 25
70.555.355	0.7862656		NCSTN///IK	nicastrin///inhibitor of kappa light polypeptide g
70.554.138	0.8759575		DLAT	dihydrolipoamide S-acetyltransferase
70.462.724	0.443571		SHQ1	SHQ1, H/ACA ribonucleoprotein assembly fact
70.462.667		-14.963.807	HGD	homogentisate 1,2-dioxygenase
70.459.742	0.8005221		SMC1A	structural maintenance of chromosomes 1A
70.453.691		-14.360.197	ACSL5	acyl-CoA synthetase long-chain family member
70.437.367	-0.7874622		MAF	MAF bZIP transcription factor
70.419.324	0.6263051		MRGBP	MRG/MORF4L binding protein
70.417.445	0.5940704		NMT1	N-myristoyltransferase 1
70.360.529	0.7187684		NUP93	nucleoporin 93
7.029.415	-0.4564079		PRB2///PRE	proline rich protein BstNI subfamily 2///proline
70.285.092		10.184.447	STK17A	serine/threonine kinase 17a
70.278.033	0.5831139		STAG1	stromal antigen 1
7.027.707		13.181.487		
7.027.138		12.006.961	COL4A1	collagen type IV alpha 1 chain
70.234.653	0.5127016		BIRC5	baculoviral IAP repeat containing 5
70.213.988		-25.709.573	CYP2E1	cytochrome P450 family 2 subfamily E member
70.202.114	-0.8325579		ERLIN1	ER lipid raft associated 1
7.018.025	-0.8614105		CYFIP2	cytoplasmic FMR1 interacting protein 2
70.176.685	0.5989756		DPF2	double PHD fingers 2
70.157.176	0.8334248		CALU	calumenin
70.143.105	-0.4586885		P3H2	prolyl 3-hydroxylase 2
70.137.479	0.7872022		GNAI3	G protein subunit alpha i3
70.086.001		-15.040.044	ABCG2	ATP binding cassette subfamily G member 2 (Ju
70.077.843	0.9319717		PPP1R2	protein phosphatase 1 regulatory inhibitor subu
70.051.225	-0.568671		KRT81	keratin 81
70.005.115	-0.7442174		RAD54L2	RAD54-like 2 (S. cerevisiae)
69.986.125	0.6109631		NENF	neudesin neurotrophic factor
69.985.348	0.5870637		FTH1	ferritin heavy chain 1
69.957.441	0.5393024		PAK2	p21 (RAC1) activated kinase 2
6.995.103	-0.5246478		SIGLEC7	sialic acid binding Ig like lectin 7
69.939.311	-0.9083455		MAF	MAF bZIP transcription factor
69.914.988	0.5374276		TAX1BP1	Tax1 binding protein 1
69.897.761		17.189.388	SFN	stratifin
69.875.929	0.6569209		AP3S1	adaptor related protein complex 3 sigma 1 subu
69.874.975	0.5159671		TSN	translin

6.986.106	0.5703796		GPR107	G protein-coupled receptor 107
69.839.839	0.4622528		ZBED4	zinc finger BED-type containing 4
69.826.879	0.7846803		TACC2	transforming acidic coiled-coil containing prote
69.818.768	0.7223436		NFYA	nuclear transcription factor Y subunit alpha
69.778.187	-0.9253033		PRKAG2	protein kinase AMP-activated non-catalytic sub
69.768.586	0.5459812		CIAPIN1	cytokine induced apoptosis inhibitor 1
69.750.107	-0.9475624		EIF4A1	eukaryotic translation initiation factor 4A1
69.687.679	-0.9345766		SUCLG2	succinate-CoA ligase GDP-forming beta subuni
69.664.381	0.9762585		ZNF468	zinc finger protein 468
69.635.971	0.5881191		DDX49	DEAD-box helicase 49
69.628.196	-0.5693262		COX6A2	cytochrome c oxidase subunit 6A2
69.619.529	0.7716663		IPO7	importin 7
69.613.261	0.6994443		MTIF2	mitochondrial translational initiation factor 2
6.961.233	-0.7079525		MYD88	myeloid differentiation primary response 88
69.528.468	0.4289916		RAD51C	RAD51 paralog C
69.516.771	0.7455889		TSN	translin
69.467.614	0.6526157		FXR1	FMR1 autosomal homolog 1
69.446.152	0.5416116		MRPL17	mitochondrial ribosomal protein L17
69.440.257		12.653.329	NDRG1	N-myc downstream regulated 1
69.411.195	-0.9306687		SUCLG2	succinate-CoA ligase GDP-forming beta subuni
69.381.319	0.5763444		CAPZA1	capping actin protein of muscle Z-line alpha sul
69.362.078	0.83441		SMAD2	SMAD family member 2
69.299.193		-10.406.598	FMO2	flavin containing monooxygenase 2
69.275.835	0.7672574		VEZF1	vascular endothelial zinc finger 1
69.224.079		-12.138.611	SLC1A2	solute carrier family 1 member 2
69.209.924	0.666078		GTF2IRD1	GTF2I repeat domain containing 1
69.206.066	0.789103		NUP160	nucleoporin 160
69.197.972	0.9880986		MRPS12	mitochondrial ribosomal protein S12
69.196.216	-0.4816371		RECK	reversion inducing cysteine rich protein with ka
69.191.722	-0.6082455		RUFY3	RUN and FYVE domain containing 3
69.189.924	0.7444395		SPATA5L1	spermatogenesis associated 5 like 1
69.176.173	0.6313539		ANO10	anoctamin 10
69.173.705		14.222.349	PKM	pyruvate kinase, muscle
69.162.592		-10.883.722	PRG4	proteoglycan 4
69.154.954	0.9415152		SMG1P5///	SMG1P5, nonsense mediated mRNA decay ass
69.071.344	0.4988872		HNRNPA1P	heterogeneous nuclear ribonucleoprotein A1 ps
69.066.673	0.5930339		BOLA1	bolA family member 1
69.060.854		-14.562.135	MASP2	mannan binding lectin serine peptidase 2
69.020.892	-0.6360055		CHP1	calcineurin like EF-hand protein 1
68.986.744	0.8857016		DLAT	dihydrolipoamide S-acetyltransferase
68.980.124	-0.6129844		REPS1	RALBP1 associated Eps domain containing 1
68.926.011	0.5848828		RPL28	ribosomal protein L28
68.921.939		-18.814.278	AOX1	aldehyde oxidase 1
68.890.369	0.8281221		SOAT1	sterol O-acyltransferase 1

68.851.708		-12.074.181	PLPP3	phospholipid phosphatase 3
68.840.013	0.7297238		COX11	COX11, cytochrome c oxidase copper chaperor
68.800.198		14.548.123	COCH	cochlin
68.799.465	0.534123		MICALL1	MICAL like 1
68.787.232	0.6588267		STAG1	stromal antigen 1
68.713.802	0.3773798		RPS2	ribosomal protein S2
68.696.649	0.8020427		ATP6V1H	ATPase H+ transporting V1 subunit H
68.689.571	0.4832673		PARL	presenilin associated rhomboid like
68.660.571	0.7599382		MRS2	MRS2, magnesium transporter
68.650.926	0.6235563		TSSC1	tumor suppressing subtransferable candidate 1
68.647.284	0.7986326		AMMECR1	Alport syndrome, mental retardation, midface h
68.639.045		-22.118.982	ALDOB	aldolase, fructose-bisphosphate B
68.626.004	0.6408934		ZNF544	zinc finger protein 544
6.854.148	0.9806414		SATB2	SATB homeobox 2
68.538.054		-1.716.754	PLG///LPA	plasminogen///lipoprotein(a)
68.523.507	0.5320784		EXOC2	exocyst complex component 2
68.456.678	0.8291057		B4GALT5	beta-1,4-galactosyltransferase 5
68.455.641	0.4141214		STMN1	stathmin 1
68.362.633	0.6848081		VRK2	vaccinia related kinase 2
68.353.761	0.7799709		PUDP	pseudouridine 5'-phosphatase
68.340.264	-0.8608038		MAP2K3	mitogen-activated protein kinase kinase 3
68.321.758	0.803037		GTF2IP1///(	general transcription factor IIi pseudogene 1///g
68.316.521	0.6267811		MRPS10	mitochondrial ribosomal protein S10
68.302.503	0.8884022		CD58	CD58 molecule
68.258.716	-0.9723933		GAS1	growth arrest specific 1
68.255.412	-0.9427923		GSDMB	gasdermin B
68.227.017		-15.331.721	SLCO1B1	solute carrier organic anion transporter family r
68.165.542	0.6495508		RECQL	RecQ like helicase
68.126.917	0.7256443		KIF5B	kinesin family member 5B
681.204		-18.699.859	ALDOB	aldolase, fructose-bisphosphate B
68.050.382	-0.7802865		MICU1	mitochondrial calcium uptake 1
68.049.566	0.7386746		ANKLE2	ankyrin repeat and LEM domain containing 2
68.045.811	-0.6921448		ALDOB	aldolase, fructose-bisphosphate B
68.028.272		-17.924.498	ACSM2B///.	acyl-CoA synthetase medium-chain family mer
68.017.206	0.7667295		FOCAD	focadhesin
68.016.995		-11.317.498	CCL21	C-C motif chemokine ligand 21
68.009.113	-0.8889074		KLF2	Kruppel like factor 2
68.007.852	0.6962837		PPM1D	protein phosphatase, Mg2+/Mn2+ dependent 1
68.004.231	0.6453184		LSM3	LSM3 homolog, U6 small nuclear RNA and mRN
67.986.093	0.5187396		SMC2	structural maintenance of chromosomes 2
67.944.491	0.8338594		WAPL	WAPL cohesin release factor
67.900.139	0.7274675		MRTO4	MRT4 homolog, ribosome maturation factor
67.879.907	-0.9613555		A2M	alpha-2-macroglobulin
67.828.603	0.5965865		RALGAPB	Ral GTPase activating protein non-catalytic beta

67.820.696	0.6911338		SMARCC1	SWI/SNF related, matrix associated, actin depe
67.748.046	0.5224453		MTCH1	mitochondrial carrier 1
67.744.355		1.015.111	GLRX3	glutaredoxin 3
6.772.258	0.3993703		RARS	arginyl-tRNA synthetase
67.709.935	0.6956973		CENPE	centromere protein E
67.707.865	0.5047975		C1orf109	chromosome 1 open reading frame 109
67.644.521	-0.7133473		N4BP2L2	NEDD4 binding protein 2 like 2
67.632.458		12.593.455	PDGFA	platelet derived growth factor subunit A
67.613.132	-0.5374932		CFLAR	CASP8 and FADD like apoptosis regulator
67.593.869	0.7402433		MCTS1	MCTS1, re-initiation and release factor
67.583.573	0.5328749		TOMM34	translocase of outer mitochondrial membrane 3
67.557.985	0.7685925		PGK1	phosphoglycerate kinase 1
67.507.263	0.6887128		MDC1	mediator of DNA damage checkpoint 1
6.749.226	0.8145394		PTPN12	protein tyrosine phosphatase, non-receptor typ
67.486.417	-0.5192306		PRSS53	protease, serine 53
6.747.381	0.6248967		AATF	apoptosis antagonizing transcription factor
67.450.854	0.5537586		C14orf169	chromosome 14 open reading frame 169
67.445.142		-13.898.009	DAO	D-amino acid oxidase
67.424.379	0.6394616		SNRPA	small nuclear ribonucleoprotein polypeptide A
67.401.529	-0.8806146		DHRS4	dehydrogenase/reductase 4
67.368.799		-17.866.825	CCL19	C-C motif chemokine ligand 19
6.735.925	-0.5623627			
67.321.082	-0.5541573			
67.313.555	0.6028692		MRPL11	mitochondrial ribosomal protein L11
67.282.176		-17.219.713	SLC38A4	solute carrier family 38 member 4
67.272.254	0.6312688		TCAF2P1///	TRPM8 channel associated factor 2 pseudogen
6.724.843	0.3854176		HNRNPA1P	heterogeneous nuclear ribonucleoprotein A1 ps
67.247.496	0.6333249		CUL2	cullin 2
67.178.499	0.391249		RPS23	ribosomal protein S23
67.174.682	0.9617535		PEX2	peroxisomal biogenesis factor 2
67.166.376	0.7267505		RANBP1	RAN binding protein 1
6.715.702	0.677667			
67.134.221	0.5246483		RPL15	ribosomal protein L15
67.078.945	0.6058385		PPME1	protein phosphatase methylesterase 1
67.076.433	0.7686892		RRAGC	Ras related GTP binding C
67.075.795		-15.407.039	PLG	plasminogen
67.046.125	0.6808665		GAPDH	glyceraldehyde-3-phosphate dehydrogenase
66.978.519	-0.4613329		PRB2///PRE	proline rich protein BstNI subfamily 2///proline
66.975.072	0.5963216		WDR45B	WD repeat domain 45B
66.943.541	-0.4682734		GFOD2	glucose-fructose oxidoreductase domain conta
66.918.275	0.9399085		NXT2	nuclear transport factor 2 like export factor 2
66.873.407	0.7184128		TCEA1	transcription elongation factor A1
66.855.244		11.995.262	SPON2	spondin 2
66.815.888	0.6987981		MED28	mediator complex subunit 28

66.799.561	0.587184		RAD1	RAD1 checkpoint DNA exonuclease
66.778.137	0.7820022		SMAP1	small ArfGAP 1
66.737.266	0.5688162		TAX1BP1	Tax1 binding protein 1
66.721.109	0.5146964		TRIM33	tripartite motif containing 33
6.668.012		-144.651	SLC17A3	solute carrier family 17 member 3
66.675.988	-0.3812242		THSD4	thrombospondin type 1 domain containing 4
66.674.875		-10.204.212	ASGR1	asialoglycoprotein receptor 1
66.642.124		-20.045.669	SLC1A1	solute carrier family 1 member 1
66.597.125	0.908716		PGK1	phosphoglycerate kinase 1
66.579.079	-0.3985545		TBXA2R	thromboxane A2 receptor
66.573.735		-10.188.005	SOX5	SRY-box 5
66.546.361	-0.7458464		PTPRO	protein tyrosine phosphatase, receptor type O
66.526.712	0.5599238		SRSF9	serine and arginine rich splicing factor 9
6.648.807	0.7424262		MTFR1	mitochondrial fission regulator 1
66.478.698	0.6361125		PSMA1	proteasome subunit alpha 1
6.645.827	-0.4877223		CRAT	carnitine O-acetyltransferase
66.451.963	-0.5784454		CDK3	cyclin dependent kinase 3
66.399.437	-0.9440005		PDLIM5	PDZ and LIM domain 5
66.373.324	-0.7767241		ALDH1B1	aldehyde dehydrogenase 1 family member B1
6.633.327		-16.853.058	TTR	transthyretin
66.218.083	-0.9989661		ADK	adenosine kinase
66.198.417	0.4881059		IP6K1	inositol hexakisphosphate kinase 1
6.618.181	0.5159416		YKT6	YKT6 v-SNARE homolog (S. cerevisiae)
66.133.665	-0.57714		BSDC1	BSD domain containing 1
66.128.025	0.6095748		DEDD	death effector domain containing
66.092.488	0.7191865		IL17RB///H	interleukin 17 receptor B///heat shock protein f
66.063.917		-14.245.197	ADCY1	adenylate cyclase 1
66.057.505	0.5350494		EIF2B4	eukaryotic translation initiation factor 2B subur
66.033.474		11.624.409	G6PD	glucose-6-phosphate dehydrogenase
66.001.351	-0.56624		DIRAS3	DIRAS family GTPase 3
65.972.376	-0.6584879		POLR1E	RNA polymerase I subunit E
65.946.406	0.894734		CDC25B	cell division cycle 25B
65.934.544	0.5646392		RFC2	replication factor C subunit 2
65.921.749	0.7481614		AACS	acetoacetyl-CoA synthetase
65.912.441		-11.924.742	TPD52L1	tumor protein D52-like 1
65.894.745		-16.281.998	ALDOB	aldolase, fructose-bisphosphate B
65.854.346	0.6019679		VPS37C	VPS37C, ESCRT-I subunit
65.852.873	0.893972		CAPZA2	capping actin protein of muscle Z-line alpha sul
65.848.152	-0.6894743		FERMT2	fermitin family member 2
65.835.248	-0.438241		TREH	trehalase
6.581.992		12.353.803	ATP1B3	ATPase Na+/K+ transporting subunit beta 3
6.579.615	0.7566547		ANGEL2	angel homolog 2
65.766.377	-0.9547578		ENG	endoglin
65.760.486	-0.5655385		KLF6	Kruppel like factor 6

65.747.505	0.4577272		SRSF2	serine and arginine rich splicing factor 2
65.741.993	0.8932631		EPRS	glutamyl-prolyl-tRNA synthetase
65.725.736	0.5394614		APEX1	apurinic/apyrimidinic endodeoxyribonuclease 1
65.699.989	0.637192		ADCK2	aarF domain containing kinase 2
65.675.069	0.7703601		DR1	down-regulator of transcription 1
65.665.362	0.6013116		GTSE1	G2 and S-phase expressed 1
6.565.467	0.4385012		POLR3F	RNA polymerase III subunit F
65.646.446	0.7566355		UBXN2B	UBX domain protein 2B
65.627.813	0.6339821		PNKP	polynucleotide kinase 3'-phosphatase
65.612.765	0.6759459		NPVF///KIF	neuropeptide VF precursor///kinesin family mer
6.560.989	0.4573133		QRICH1	glutamine rich 1
65.568.959	0.5797031		RAB3GAP2	RAB3 GTPase activating non-catalytic protein su
65.559.508	0.9607341		TAGLN2	transgelin 2
65.509.824	0.6396895		AQR	aquarius intron-binding spliceosomal factor
65.489.539	0.7036337		NDC1	NDC1 transmembrane nucleoporin
65.474.647		-10.199.633	RORA	RAR related orphan receptor A
65.466.069	-0.6597254		MAP2K3	mitogen-activated protein kinase kinase 3
65.457.535	0.6984959		GTF2H2C_2	GTF2H2 family member C, copy 2///GTF2H2 far
65.453.606	0.6546957		CUL4B	cullin 4B
65.444.265	-0.8106016		PEBP1	phosphatidylethanolamine binding protein 1
65.428.256		10.973.549	PNMA1	paraneoplastic Ma antigen 1
65.415.804	0.5020182		TEFM	transcription elongation factor, mitochondrial
65.378.651	0.6083057		THAP12	THAP domain containing 12
65.360.564	0.4892863		IK	IK cytokine, down-regulator of HLA II
65.359.928		-10.740.141	GK	glycerol kinase
65.343.362		-12.971.684	ITIH1	inter-alpha-trypsin inhibitor heavy chain 1
65.323.775	-0.4661346		FAT4	FAT atypical cadherin 4
65.317.728	0.6190689		RIOK2	RIO kinase 2
65.267.003	0.7693768		AGPAT1	1-acylglycerol-3-phosphate O-acyltransferase 1
65.244.968	0.8126325		ARL1	ADP ribosylation factor like GTPase 1
65.227.864	0.5413073		SLC25A3	solute carrier family 25 member 3
65.226.785	0.5753607		SMG7	SMG7, nonsense mediated mRNA decay factor
65.225.519	0.5259445		GTF3C2	general transcription factor IIIC subunit 2
65.224.589		10.703.682	NT5DC2	5'-nucleotidase domain containing 2
65.206.078	0.5891156		TPR	translocated promoter region, nuclear basket p
65.197.975		-13.089.995	HGD	homogentisate 1,2-dioxygenase
65.187.357	0.6580275		NPTN	neuroplastin
65.166.568	0.6573412		IDE	insulin degrading enzyme
65.151.092		-127.881	CP	ceruloplasmin (ferroxidase)
65.142.322	-0.6113677		CCDC25	coiled-coil domain containing 25
65.135.732	0.7582755		GNAZ	G protein subunit alpha z
65.135.498	-0.8515824		CDH19	cadherin 19
65.129.367	0.5093149		SEPT9	septin 9
65.086.959	0.6007558		RFX5	regulatory factor X5

65.074.401	0.772868		HYOU1	hypoxia up-regulated 1
65.069.631	0.8260475		MTMR2	myotubularin related protein 2
65.059.784	-0.7250287		LYRM1	LYR motif containing 1
65.004.136	0.6629054		SET	SET nuclear proto-oncogene
64.988.055	0.4379738		BLZF1	basic leucine zipper nuclear factor 1
64.986.375	0.7011697		PPP2CA	protein phosphatase 2 catalytic subunit alpha
64.964.646	-0.9466175		AXL	AXL receptor tyrosine kinase
6.492.064		-15.544.745	HLF	HLF, PAR bZIP transcription factor
64.900.134		-13.182.318	CYP3A5	cytochrome P450 family 3 subfamily A member
6.489.137	0.6285723		RPS12	ribosomal protein S12
6.488.219	0.8696695		HEY1	hes related family bHLH transcription factor wit
64.880.051		16.902.567	FADS1	fatty acid desaturase 1
64.857.416		-13.364.378	MME	membrane metallo-endopeptidase
64.853.346	-0.6527709		FTO	fat mass and obesity associated
64.833.308	0.5791511		TFIP11	tuftelin interacting protein 11
6.481.385		-12.126.163	RUNDC3B	RUN domain containing 3B
64.771.472	0.6173741		HSP90AA1	heat shock protein 90 alpha family class A mem
64.764.604	0.7782876		DR1	down-regulator of transcription 1
64.761.224	0.4495631		RIF1	replication timing regulatory factor 1
64.744.876		-13.339.226	PSD3	pleckstrin and Sec7 domain containing 3
64.723.947	0.5344328		PPM1F	protein phosphatase, Mg2+/Mn2+ dependent 1
64.694.722	0.590389		GORASP2	golgi reassembly stacking protein 2
64.691.411	0.5889071		PSRC1	proline and serine rich coiled-coil 1
64.682.581	0.5337745		SERBP1	SERPINE1 mRNA binding protein 1
64.650.707	0.6017108		MOB4	MOB family member 4, phocein
64.646.452	0.44643		CENPQ	centromere protein Q
64.635.864	0.6872098		OSBPL3	oxysterol binding protein like 3
64.629.506	0.6708779		MRM2	mitochondrial rRNA methyltransferase 2
64.611.439	-0.7605569		CCS	copper chaperone for superoxide dismutase
64.544.862	0.8432315		ACTR6	ARP6 actin-related protein 6 homolog
64.531.785	0.7919211		RAP2B	RAP2B, member of RAS oncogene family
64.529.376	0.4729982		RFC3	replication factor C subunit 3
64.519.628	0.5948257		SLIRP	SRA stem-loop interacting RNA binding protein
64.505.375	0.633072		SRPK2	SRSF protein kinase 2
64.502.582	0.6003222		SLC50A1	solute carrier family 50 member 1
64.488.683	-0.7946141		CTBS	chitobiase
64.477.507	0.6466279		SEMA3F	semaphorin 3F
64.460.858	-0.3539468		ALDOB	aldolase, fructose-bisphosphate B
64.438.037	0.6265673		RPS16	ribosomal protein S16
64.412.023	0.5114781		PGS1	phosphatidylglycerophosphate synthase 1
6.439.733	0.5791556		SLC4A2	solute carrier family 4 member 2
64.391.477	0.3507354		ZKSCAN5	zinc finger with KRAB and SCAN domains 5
64.222.416	-0.4977687		MXRA7///T(	matrix remodeling associated 7///transcription
6.420.176	-0.8621075		STOM	stomatin

64.184.485	0.705027		YIPF3	Yip1 domain family member 3
64.170.898		-10.439.341	ACY1	aminoacylase 1
64.166.645		11.126.058	STK39	serine/threonine kinase 39
64.008.997	0.6670388		BECN1	beclin 1
64.007.355	-0.7071482		RANBP10	RAN binding protein 10
64.001.694	0.7288252		C9orf16	chromosome 9 open reading frame 16
63.943.909		-11.780.457	ABCC6	ATP binding cassette subfamily C member 6
63.942.798	0.836207		NAP1L1	nucleosome assembly protein 1 like 1
63.941.762	0.4862402		MORF4L2	mortality factor 4 like 2
63.932.539		-12.274.473	RAB26	RAB26, member RAS oncogene family
63.931.399	0.9868858		ASRGL1	asparaginase like 1
63.927.859	0.440044		MAPK9	mitogen-activated protein kinase 9
6.390.622		-17.710.257	HRG	histidine rich glycoprotein
63.903.538	0.4417311		SSNA1	SS nuclear autoantigen 1
63.874.906		-18.918.725	PON1	paraoxonase 1
63.826.133	0.6885856		KPNA4	karyopherin subunit alpha 4
63.774.442	-0.4433135		ARHGAP10	Rho GTPase activating protein 10
63.760.501	0.420052		JMJD4	jumonji domain containing 4
63.747.543	0.7193628		TRAPPC2B/	trafficking protein particle complex 2B///traffick
63.731.074	-0.6722563		ATMIN	ATM interactor
63.707.636	0.5685846		TRIM32	tripartite motif containing 32
63.700.449	0.563208		SNRNP27	small nuclear ribonucleoprotein U4/U6.U5 sub
63.678.951	-0.5561576		NCOR1P1/	nuclear receptor corepressor 1 pseudogene 1//
63.676.253		-13.399.097	DUSP1	dual specificity phosphatase 1
63.659.058	0.5752459		PKN1	protein kinase N1
63.649.081		16.496.041	SGCE	sarcoglycan epsilon
63.638.486		-10.800.543	SOCS2	suppressor of cytokine signaling 2
63.590.722		10.514.333	CCT2	chaperonin containing TCP1 subunit 2
63.579.779	-0.682798		AQP3	aquaporin 3 (Gill blood group)
63.532.228	0.7280316		HILPDA	hypoxia inducible lipid droplet associated
63.525.017	-0.8241006		SLC39A14	solute carrier family 39 member 14
63.515.405	0.6790896		NSDHL	NAD(P) dependent steroid dehydrogenase-like
63.469.091	0.7641826		SMS	spermine synthase
63.437.229	0.5724156		SLC35B1	solute carrier family 35 member B1
63.432.272	-0.8186836		PDLIM5	PDZ and LIM domain 5
63.396.443	0.7146487		AGPS	alkylglycerone phosphate synthase
63.369.006	0.6535726		ENTPD6	ectonucleoside triphosphate diphosphohydrola
63.362.916	-0.6044727		CLUH	clustered mitochondria homolog
63.356.361	0.7978214		UBE2N	ubiquitin conjugating enzyme E2 N
63.344.296	0.5784341		DVL2	dishevelled segment polarity protein 2
63.337.453	0.8620199		PTPRG	protein tyrosine phosphatase, receptor type G
6.333.566	0.4672153		MAGOHB	mago homolog B, exon junction complex core c
63.276.968	-0.53926		APOC1	apolipoprotein C1
63.275.894	0.5990038		FTSJ3	FtsJ homolog 3

63.272.019		-1.078.199	PXMP2	peroxisomal membrane protein 2
63.195.452	0.5664774		EIF3K	eukaryotic translation initiation factor 3 subuni
63.193.071	0.7906633		KDM5B	lysine demethylase 5B
63.190.796	0.6130729		ZNF318	zinc finger protein 318
63.184.658	0.5303124		GAPDH	glyceraldehyde-3-phosphate dehydrogenase
63.169.195	0.6908496		DSCC1	DNA replication and sister chromatid cohesion
6.316.709	0.4997417		CHEK1	checkpoint kinase 1
63.154.288		-15.192.707	ASPN	asporin
63.121.685	-0.5850131		CFLAR	CASP8 and FADD like apoptosis regulator
63.095.005		-13.068.536	ACOX2	acyl-CoA oxidase 2
63.092.483	0.4573069		DDX52	DEAD-box helicase 52
63.090.819	0.8074255		RBM8A	RNA binding motif protein 8A
63.081.996		-17.764.317	TMEM45A	transmembrane protein 45A
63.012.473	0.7862531		MAPRE1	microtubule associated protein RP/EB family m
63.006.599	0.6805715		SPTAN1	spectrin alpha, non-erythrocytic 1
62.983.962	-0.9155689		ZCCHC6	zinc finger CCHC-type containing 6
62.967.468		-1.873.502	AOX1	aldehyde oxidase 1
62.965.423	-0.5389346		SPAG7	sperm associated antigen 7
6.293.827	0.704321		PPP1R2	protein phosphatase 1 regulatory inhibitor subu
62.938.013	0.7368829		ME2	malic enzyme 2
6.293.404	-0.4551321		PSD4	pleckstrin and Sec7 domain containing 4
62.931.531	0.8696175		PPT1	palmitoyl-protein thioesterase 1
62.894.297	0.9181324		SLC39A6	solute carrier family 39 member 6
628.865	0.6980972		U2SURP	U2 snRNP associated SURP domain containing
62.882.661	0.597823		FN3KRP	fructosamine 3 kinase related protein
62.877.091	0.4982561		SLC52A2	solute carrier family 52 member 2
62.873.104	0.2922095		RPL39	ribosomal protein L39
62.794.356	0.5728423		VPS16	VPS16, CORVET/HOPS core subunit
62.749.489	-0.2792233		ID2B	inhibitor of DNA binding 2B, HLH protein (pseu
62.739.705		11.691.379	SERPINI1	serpin family I member 1
62.733.781	0.4342093		TMEM115	transmembrane protein 115
62.728.658	0.8113442		C9orf16	chromosome 9 open reading frame 16
62.721.122	0.879736		IVNS1ABP	influenza virus NS1A binding protein
62.640.598	-0.5125239		EI24	EI24, autophagy associated transmembrane pr
62.619.737	0.6856546		ADAM10	ADAM metallopeptidase domain 10
62.610.757	0.697108		PDS5B	PDS5 cohesin associated factor B
62.595.723	0.7553145		OPTN	optineurin
62.592.898	0.8909985		GFPT1	glutamine--fructose-6-phosphate transaminas
62.578.248	0.4600235		RPL13A	ribosomal protein L13a
62.562.561	0.5643549		UIMC1	ubiquitin interaction motif containing 1
62.525.988	0.4757572		RPL13A	ribosomal protein L13a
62.523.196	0.440348		ATP5E	ATP synthase, H+ transporting, mitochondrial F
62.494.526	0.7706013		UBE2E3	ubiquitin conjugating enzyme E2 E3
62.492.932	0.8558294		MYL6B	myosin light chain 6B

62.398.817	-0.8789253		PIK3C2G	phosphatidylinositol-4-phosphate 3-kinase cat
62.395.188	0.5679311		MAP4	microtubule associated protein 4
62.391.626	0.9521564		FKBP11	FK506 binding protein 11
62.386.999	-0.5529583		METAP2	methionyl aminopeptidase 2
62.366.286	0.5344904		ARPC2	actin related protein 2/3 complex subunit 2
62.347.857	0.4323961		MCRS1	microspherule protein 1
62.285.345	-0.3940655		CYLD	CYLD lysine 63 deubiquitinase
62.261.382	0.7904049		ARPC2	actin related protein 2/3 complex subunit 2
6.225.604	0.3973155		DDB1	damage specific DNA binding protein 1
62.216.907	0.6456486		GINS2	GINS complex subunit 2
62.216.709		-19.898.575	HPR	haptoglobin-related protein
62.152.023	-0.8266639		GAS6	growth arrest specific 6
62.151.846	-0.351756		KCNJ15	potassium voltage-gated channel subfamily J m
62.134.697		26.274.348	S100P	S100 calcium binding protein P
62.133.682	0.8944868			
62.111.422	-0.4952052		PHF2	PHD finger protein 2
62.093.847	0.700296		MTDH	metadherin
62.093.236	0.869144		TTLL4	tubulin tyrosine ligase like 4
62.076.721	0.7012445		CNPY2	canopy FGF signaling regulator 2
62.073.255	0.8607172		COPE	coatomer protein complex subunit epsilon
62.003.337	0.5178511		ANAPC1	anaphase promoting complex subunit 1
61.979.415		-18.101.358	AGXT	alanine-glyoxylate aminotransferase
61.956.177	-0.5538032		EMILIN1	elastin microfibril interfacer 1
61.954.437	0.5514408		EIF2B4	eukaryotic translation initiation factor 2B subur
61.940.198	0.5591639		GOSR2	golgi SNAP receptor complex member 2
6.191.881	0.7049334		RPRD2	regulation of nuclear pre-mRNA domain contai
61.897.699	0.6163006		ABCF2	ATP binding cassette subfamily F member 2
61.861.525	-0.5701143		EDEM1	ER degradation enhancing alpha-mannosidase
61.859.145	-0.5309199		IL17RC	interleukin 17 receptor C
61.844.818	-0.4807615		LILRB3///LI	leukocyte immunoglobulin like receptor B3///le
61.843.567	-0.6568299		CAMK2B	calcium/calmodulin dependent protein kinase
61.828.004	-0.7785613		MRC2	mannose receptor C type 2
61.766.422	0.7260437		CDKN2A	cyclin dependent kinase inhibitor 2A
61.748.285	0.545522		AGFG1	ArfGAP with FG repeats 1
61.745.622		-11.263.429	CRYL1	crystallin lambda 1
61.739.757	0.5115057		RPL13A	ribosomal protein L13a
61.723.212	0.4918038		PSMA7	proteasome subunit alpha 7
61.705.782	0.491628		TIMM17B	translocase of inner mitochondrial membrane 1
61.681.312	0.492712		COIL	coilin
6.164.914	-0.2837278		SMPD3	sphingomyelin phosphodiesterase 3
61.630.748	0.6590716		ZNF146	zinc finger protein 146
61.610.602	0.5854382		ZNF672	zinc finger protein 672
61.552.367	0.3121077		ARFGEF2//.	ADP ribosylation factor guanine nucleotide excl
61.551.321	-0.8712833		EGR2	early growth response 2

61.536.713	-0.3125486		DACH1	dachshund family transcription factor 1
61.531.205	0.6727449		RCOR1	REST corepressor 1
6.148.837	0.5906512		ORC3	origin recognition complex subunit 3
61.453.609	0.6664183		THOC5	THO complex 5
61.453.104	-0.8143596		SLCO2B1	solute carrier organic anion transporter family r
6.144.908	0.6325206		GTF2A2	general transcription factor IIA subunit 2
61.421.055	-0.9606774		CLDN10	claudin 10
61.403.418	0.6254945		GAPDH	glyceraldehyde-3-phosphate dehydrogenase
61.386.319	-0.8141936		ZFP36L1	ZFP36 ring finger protein like 1
61.361.757	0.9942878		LSM4	LSM4 homolog, U6 small nuclear RNA and mRN
61.349.654		-14.095.755	SLC27A2	solute carrier family 27 member 2
61.315.975	-0.6357749		ATG2A	autophagy related 2A
61.310.935	0.6223341		ARMC8	armadillo repeat containing 8
61.269.237		11.742.928	GJA1	gap junction protein alpha 1
61.266.156	0.6889175		EXOSC4	exosome component 4
61.236.302	0.7542351		CYB5R1	cytochrome b5 reductase 1
6.122.674	-0.4796763			
612.196	0.6262256		PJA1	praja ring finger ubiquitin ligase 1
61.203.077	0.8692645		TACC2	transforming acidic coiled-coil containing prote
61.199.977		-17.488.215	ABCB4	ATP binding cassette subfamily B member 4
61.172.592	0.5514411		CSNK1D	casein kinase 1 delta
61.168.505	-0.9581		CLEC3B	C-type lectin domain family 3 member B
61.143.751	0.646204		PHF20	PHD finger protein 20
61.122.702	0.6024614		C17orf62	chromosome 17 open reading frame 62
61.111.671		-1.128.709	AQP3	aquaporin 3 (Gill blood group)
61.105.728	0.7589624		RAB29	RAB29, member RAS oncogene family
61.091.061	-0.9360571		ACAT1	acetyl-CoA acetyltransferase 1
61.090.389	0.7242434		PPA1	pyrophosphatase (inorganic) 1
6.106.304	0.6480753		PON2	paraoxonase 2
61.054.614	0.515446		TCF3	transcription factor 3
61.014.185	0.7699825		PHIP	pleckstrin homology domain interacting protein
61.012.653		-12.570.559	FMO4	flavin containing monooxygenase 4
60.972.051	0.6735187		TAF9	TATA-box binding protein associated factor 9
60.925.474		-11.227.836	PDGFC	platelet derived growth factor C
60.916.718	0.6241785		SNRPA1	small nuclear ribonucleoprotein polypeptide A'
60.911.368	0.5736232		HMGN2	high mobility group nucleosomal binding domai
6.090.094	0.7029805		DR1	down-regulator of transcription 1
60.891.091	-0.3819613		PDE8B	phosphodiesterase 8B
60.796.183	0.6073063		EIF4ENIF1	eukaryotic translation initiation factor 4E nucle
60.747.162	-0.7141593		FERMT2	fermitin family member 2
60.719.837	-0.9358873		RNASE4	ribonuclease A family member 4
60.696.817		-1.123.761	SORD	sorbitol dehydrogenase
60.693.064	0.5446573		SRSF1	serine and arginine rich splicing factor 1
60.683.291	0.8072904		COMMD8	COMM domain containing 8

60.679.001	-0.8262387		ZBTB20	zinc finger and BTB domain containing 20
6.066.939	0.9510813		THY1	Thy-1 cell surface antigen
60.649.353	0.5753721		ZNF107	zinc finger protein 107
60.640.152	-0.3245093		NTRK2	neurotrophic receptor tyrosine kinase 2
60.633.899	0.6208863		PSMD8	proteasome 26S subunit, non-ATPase 8
60.615.303	0.5187158		CHTOP	chromatin target of PRMT1
60.607.164	0.6610581		SPTAN1	spectrin alpha, non-erythrocytic 1
60.602.246	-0.4141988		NXF3	nuclear RNA export factor 3
6.057.674	0.5654227		RNF7	ring finger protein 7
60.570.558	-0.6358856		PDK2	pyruvate dehydrogenase kinase 2
60.561.882	0.6879616		NFU1	NFU1 iron-sulfur cluster scaffold
6.055.679	0.6507984			
60.523.868	0.7134385		HAUS3	HAUS augmin like complex subunit 3
60.516.106		-10.260.319	GPLD1	glycosylphosphatidylinositol specific phosphol
6.048.951	0.6549751		AMD1	adenosylmethionine decarboxylase 1
60.485.411		-14.126.684	CES2	carboxylesterase 2
60.462.291	0.5250606		SNRNP200	small nuclear ribonucleoprotein U5 subunit 20(
60.435.255	0.5276459		SOCS5	suppressor of cytokine signaling 5
6.041.645	-0.4036085		DTX2P1-UF	DTX2P1-UPK3BP1-PMS2P11 readthrough, trans
60.413.392	-0.6177405		MUC6	mucin 6, oligomeric mucus/gel-forming
6.036.328	0.4642029		CD3EAP	CD3e molecule associated protein
60.356.816		13.164.155	PLPPR1	phospholipid phosphatase related 1
60.355.907	-0.667336		LIMS2	LIM zinc finger domain containing 2
60.303.403	-0.5253152		UBE2D3	ubiquitin conjugating enzyme E2 D3
60.234.558	0.8716815		TMEM70	transmembrane protein 70
6.015.686	0.4628809		PIAS3	protein inhibitor of activated STAT 3
60.137.378	0.8775575		CTR9	CTR9 homolog, Paf1/RNA polymerase II comple
6.012.314	-0.7294834		TBC1D2B	TBC1 domain family member 2B
60.092.939	0.5943976		NENF	neudesin neurotrophic factor
60.055.855	0.5642636		PI4KB	phosphatidylinositol 4-kinase beta
60.051.749	-0.9537553		SLC39A8	solute carrier family 39 member 8
60.047.126	0.4616594		DPH2	DPH2 homolog
60.040.703		11.828.809	RGS5	regulator of G-protein signaling 5
60.009.481	-0.5700823		CFB	complement factor B
60.000.579		-10.447.789	RORA	RAR related orphan receptor A
59.980.317	0.4332569		SRSF3	serine and arginine rich splicing factor 3
59.956.573	0.3457662		FTSJ1	FtsJ RNA methyltransferase homolog 1 (E. coli)
5.995.422	0.9896385		FNDC3B	fibronectin type III domain containing 3B
59.954.076	-0.5508975		HBG2///HB	hemoglobin subunit gamma 2///hemoglobin su
59.931.023	-0.5234		NR4A1	nuclear receptor subfamily 4 group A member 1
59.896.274	0.5080556		DDX49	DEAD-box helicase 49
59.861.669	0.5003513		NSUN5	NOP2/Sun RNA methyltransferase family meml
59.857.373	0.629207		SRP72	signal recognition particle 72
59.849.033		13.706.627	MARCKS	myristoylated alanine rich protein kinase C sub

59.843.772		-17.567.007	ALDH1L1	aldehyde dehydrogenase 1 family member L1
59.830.696		-1.584.697	CUX2	cut like homeobox 2
59.819.999	0.806533		P2RX4	purinergic receptor P2X 4
59.811.967	0.4829975		RRP12	ribosomal RNA processing 12 homolog
59.810.886	-0.8625901		NCOR1	nuclear receptor corepressor 1
59.771.606	0.6648853		IL17RB	interleukin 17 receptor B
59.764.983	-0.6092294		SRRM2	serine/arginine repetitive matrix 2
59.667.963	-0.353764		POU6F1	POU class 6 homeobox 1
59.609.101	-0.9254727		SLC25A20	solute carrier family 25 member 20
59.593.882	0.7767226		ZNF189	zinc finger protein 189
59.587.007	-0.8430925		ADK	adenosine kinase
59.568.306		-18.461.116	FMO3	flavin containing monooxygenase 3
59.566.015	-0.5556336		HEMK1	HemK methyltransferase family member 1
59.559.592	-0.60904		ALDH9A1	aldehyde dehydrogenase 9 family member A1
59.553.279		-12.500.392	KLF9	Kruppel like factor 9
59.522.917	-0.6047738		MAP2K1	mitogen-activated protein kinase kinase 1
59.494.515	0.5575312		TCF3	transcription factor 3
59.471.953	0.4477237		SLC41A3	solute carrier family 41 member 3
594.481	0.5824283		MRE11A	MRE11 homolog A, double strand break repair r
59.432.094	-0.8074155		AGPAT2	1-acylglycerol-3-phosphate O-acyltransferase
59.414.376	-0.5271595		RAB33B	RAB33B, member RAS oncogene family
59.412.844	0.5193063		RPE	ribulose-5-phosphate-3-epimerase
59.408.878	-0.6358105		KDM6B	lysine demethylase 6B
59.393.173		13.716.515	TCEAL9	transcription elongation factor A like 9
59.390.465	0.5403875		AGPAT1	1-acylglycerol-3-phosphate O-acyltransferase
59.389.923		-11.243.594	KHK	ketohexokinase
59.358.006	0.4839355		CCHCR1	coiled-coil alpha-helical rod protein 1
59.355.152	-0.9468869		ASGR2	asialoglycoprotein receptor 2
59.326.614	0.5322891		KLHL12	kelch like family member 12
59.309.467		11.210.075	SYPL1	synaptophysin like 1
59.295.072	0.3214946		C7orf25	chromosome 7 open reading frame 25
59.289.979		-14.755.563	HP	haptoglobin
5.927.821	0.5322157		RUVBL1	RuvB like AAA ATPase 1
59.256.701	0.921373		CEBPG	CCAAT/enhancer binding protein gamma
59.239.875	0.5482447		PPP1R7	protein phosphatase 1 regulatory subunit 7
5.923.146	0.6056232		FADD	Fas associated via death domain
59.206.426	0.4215035		KIF2C	kinesin family member 2C
59.199.129	0.4526248		SERP1	stress-associated endoplasmic reticulum prote
59.178.851	-0.5808108		CDK10	cyclin dependent kinase 10
59.159.421	0.7529485		PRRC2C	proline rich coiled-coil 2C
5.912.748	0.6545925		EXOSC4	exosome component 4
59.110.222	0.5658122		ACTG1	actin gamma 1
59.082.114	0.9153247		NAP1L1	nucleosome assembly protein 1 like 1
59.069.519	0.7713626		SRP72	signal recognition particle 72

59.032.025	0.600792		UBE2K	ubiquitin conjugating enzyme E2 K
59.031.426	0.4482828		SIPA1L3	signal induced proliferation associated 1 like 3
59.030.662		-10.972.835	DUSP6	dual specificity phosphatase 6
59.025.025	0.5374421		PIAS4	protein inhibitor of activated STAT 4
59.020.084	-0.9347773		GRB14	growth factor receptor bound protein 14
59.008.332	-0.6569381		KIAA1109	KIAA1109
59.002.597	-0.4326701		MYL6	myosin light chain 6
59.000.208	0.5477372		HRAS	HRas proto-oncogene, GTPase
58.976.176	-0.7931021		MUC3A	mucin 3A, cell surface associated
58.975.157	0.5622464		MSH5	mutS homolog 5
58.970.188	0.5949969		CD58	CD58 molecule
58.968.799	-0.3862641		SLC25A37	solute carrier family 25 member 37
58.949.518	0.7789531		ENO1	enolase 1
58.914.376	0.442007		LMNB2	lamin B2
58.913.954	0.3844116		CEP131	centrosomal protein 131
58.911.152	0.6129379		FKBP9	FK506 binding protein 9
58.894.014	0.7604415		NAP1L1	nucleosome assembly protein 1 like 1
58.888.355	0.5630347		DGCR2	DiGeorge syndrome critical region gene 2
58.885.026	0.6066926		TRAPPC4	trafficking protein particle complex 4
58.882.111	-0.6080945		PLA2G12A	phospholipase A2 group XIIA
58.855.948	0.4504695		ARHGAP19	Rho GTPase activating protein 19
5.885.163	-0.9599085		HPN	hepsin
58.828.399	0.8270403		SLC17A5	solute carrier family 17 member 5
58.810.815	0.3801984		CAMSAP1	calmodulin regulated spectrin associated prote
58.809.221	0.8530489		ACBD3	acyl-CoA binding domain containing 3
58.807.017	0.5114448		PRKAB1	protein kinase AMP-activated non-catalytic sub
58.805.516		-10.350.346	HYAL1	hyaluronoglucosaminidase 1
5.876.989		-12.169.849	EFHD1	EF-hand domain family member D1
58.769.142	0.6992038		VAT1	vesicle amine transport 1
58.763.982	0.5296588		MKI67	marker of proliferation Ki-67
58.747.698	0.4778314		GNA12	G protein subunit alpha 12
58.747.502	0.5593761		PSMA4	proteasome subunit alpha 4
58.745.983	0.8011038		LOC73042!	e3 ubiquitin-protein ligase UBR5-like///ubiquiti
58.731.031		10.877.163	MMD	monocyte to macrophage differentiation associ
58.717.563	-0.484336		ESR1	estrogen receptor 1
58.665.258	0.5450315		UTP11	UTP11, small subunit processome component
58.662.916	0.479963		JMJD6	arginine demethylase and lysine hydroxylase
58.660.376		10.305.867	SMC3	structural maintenance of chromosomes 3
58.644.589	-0.4662208		RCN3	reticulocalbin 3
58.618.666	-0.82824		PLCG2	phospholipase C gamma 2
5.861.761	0.6279981		FTSJ1	FtsJ RNA methyltransferase homolog 1 (E. coli)
58.609.697	0.5080002		SSSCA1	Sjogren syndrome/scleroderma autoantigen 1
58.592.561		-13.060.999	CD163	CD163 molecule
58.573.242		11.597.993	RGS5	regulator of G-protein signaling 5

58.572.641	-0.9552806	SRRM2	serine/arginine repetitive matrix 2
58.571.151	0.5278765	MAP3K7	mitogen-activated protein kinase kinase kinase
58.533.816	0.6438268	NOLC1	nucleolar and coiled-body phosphoprotein 1
58.524.751	0.4927438	GABPB1	GA binding protein transcription factor beta sub
58.524.591	0.4613914	GNG5	G protein subunit gamma 5
58.494.731	0.6397601	LAMTOR2	late endosomal/lysosomal adaptor, MAPK and l
5.846.835	-0.8514918	PROSC	proline synthetase cotranscribed homolog (bac
58.467.472	0.4643717	TAF1A	TATA-box binding protein associated factor, RN
58.464.444	0.5022282	MRPL3	mitochondrial ribosomal protein L3
58.434.525	0.5816238	CLASP2	cytoplasmic linker associated protein 2
58.426.524	0.5297061	TMEM189-U	TMEM189-UBE2V1 readthrough///ubiquitin con
58.377.566	-0.8130676	AMT	aminomethyltransferase
58.374.221	0.7471571	DHX15	DEAH-box helicase 15
58.361.478	0.7106538	AIMP1	aminoacyl tRNA synthetase complex interacting
58.337.779	0.4959375	IMMT	inner membrane mitochondrial protein
58.299.515	0.5407013	RPL23AP7	ribosomal protein L23a pseudogene 7
58.283.838	0.8088703	RBBP4	RB binding protein 4, chromatin remodeling fac
58.273.294	-0.8532242	APOL1	apolipoprotein L1
58.244.751	0.9405394	ASCC3	activating signal cointegrator 1 complex subuni
5.823.665	0.7216242	PAK2	p21 (RAC1) activated kinase 2
5.822.902	0.4692037	NDUFB3	NADH:ubiquinone oxidoreductase subunit B3
58.217.899	-0.8415208	PPL	periplakin
58.209.246	0.5136172	LETM1	leucine zipper and EF-hand containing transme
58.199.331	0.7118053	SSRP1	structure specific recognition protein 1
58.199.007	0.4430755	TFG	TRK-fused gene
58.182.258	0.6449919	UPF2	UPF2 regulator of nonsense transcripts homolo
5.817.697	0.5233421	GEMIN2	gem nuclear organelle associated protein 2
58.134.604	0.5960104	HDAC1	histone deacetylase 1
58.126.732	0.5701634	MAPKAPK2	mitogen-activated protein kinase-activated pro
58.108.929	0.83781	NPAS2	neuronal PAS domain protein 2
58.092.766	0.6781852	ABI1	abl interactor 1
58.084.748	0.4413379		
58.063.699	0.7294001	EMG1	EMG1, N1-specific pseudouridine methyltransf
58.052.005	-0.3102051	SPAG8	sperm associated antigen 8
58.027.409	0.6360172	DLST	dihydrolipoamide S-succinyltransferase
58.015.405	0.3466994	CUL2	cullin 2
57.989.901	-0.5256554	ANKS1A	ankyrin repeat and sterile alpha motif domain c
5.798.627	0.4978401	BAZ1B	bromodomain adjacent to zinc finger domain 1I
57.974.016	-0.8991963	PROS1	protein S (alpha)
57.969.524	-0.502241	TMBIM6	transmembrane BAX inhibitor motif containing
57.967.711	-0.3567409	CEMIP	cell migration inducing hyaluronan binding prot
5.796.388	0.4825526	MPHOSPH9	M-phase phosphoprotein 9
57.945.016	-0.5809401	LOC72788	hypothetical LOC727882///matrix remodeling a
57.897.883	0.5106335	ZNF200	zinc finger protein 200

57.897.614		10.617.775	HOMER1	homer scaffolding protein 1
57.873.486	-0.5315615		LILRB2	leukocyte immunoglobulin like receptor B2
57.833.532	0.9125102		ATP2B1	ATPase plasma membrane Ca2+ transporting 1
57.816.141	0.6398967		CORO1B	coronin 1B
57.815.835	0.7008293		DCP2	decapping mRNA 2
57.794.922	0.5896022		GAPDH	glyceraldehyde-3-phosphate dehydrogenase
57.792.039		-10.955.862	SERPINA10	serpin family A member 10
57.757.361	0.5896711		REPIN1	replication initiator 1
57.741.163	0.5103923		MED8	mediator complex subunit 8
577.177	0.6327897		ENY2	ENY2, transcription and export complex 2 subu
57.716.224		-16.051.201	ANO1	anoctamin 1
5.770.885	-0.8368872		ACADM	acyl-CoA dehydrogenase, C-4 to C-12 straight c
5.770.475	0.677339		CHEK2	checkpoint kinase 2
57.691.513	-0.4761216		BSDC1	BSD domain containing 1
57.639.783	-0.7405404		NIT2	nitrilase family member 2
57.639.338	0.5794158		USP21	ubiquitin specific peptidase 21
57.632.418		12.928.383	C1orf106	chromosome 1 open reading frame 106
57.615.344	0.6001244		FAIM	Fas apoptotic inhibitory molecule
5.759.637	-0.8031971		ALPL	alkaline phosphatase, liver/bone/kidney
57.595.338	0.7669372		ZYX	zyxin
57.582.958	0.4403723		ZNF322P1	zinc finger protein 322 pseudogene 1
57.572.961	0.5881978		NPAS2	neuronal PAS domain protein 2
57.561.417		-11.257.599	EDNRB	endothelin receptor type B
57.521.801	0.7256437		TWF1	twinfilin actin binding protein 1
57.516.663	0.507273		ANP32A	acidic nuclear phosphoprotein 32 family memb
5.750.837	0.7727486		TM9SF1	transmembrane 9 superfamily member 1
57.475.637	0.9389415		TMEM97	transmembrane protein 97
57.448.577	0.6517561		IRAK1BP1	interleukin 1 receptor associated kinase 1 bindi
57.444.823	0.4658798		HTRA2	HtrA serine peptidase 2
57.414.824	0.73693		ACTR3	ARP3 actin related protein 3 homolog
57.410.586	0.6610198		HSPA5	heat shock protein family A (Hsp70) member 5
57.386.644		-10.464.914	PLA2G16	phospholipase A2 group XVI
57.382.358	0.5426331		MAP4	microtubule associated protein 4
57.371.188	0.5031945		POLG2	DNA polymerase gamma 2, accessory subunit
57.358.121		-18.630.523	HSD17B6	hydroxysteroid 17-beta dehydrogenase 6
57.348.593	0.5970652		PPAT	phosphoribosyl pyrophosphate amidotransfera
57.344.037	0.6905075		CYCS	cytochrome c, somatic
57.343.006	0.6839019		SNRPA1	small nuclear ribonucleoprotein polypeptide A'
57.328.524	-0.5181725		RPH3AL	rabphilin 3A-like (without C2 domains)
57.311.698	0.73991		BAZ1A	bromodomain adjacent to zinc finger domain 1/
57.282.563	0.4971224		MATR3	matrin 3
57.271.441	-0.3419887		LILRA2	leukocyte immunoglobulin like receptor A2
5.726.567	0.7372475		GRN	granulin
57.251.918		-12.523.811	TF	transferrin

57.211.153	0.6557363		PRPF3	pre-mRNA processing factor 3
5.719.651	0.6544987		SYNGR2	synaptogyrin 2
57.186.904	0.5758027		TMBIM1	transmembrane BAX inhibitor motif containing
57.173.302	0.7388155		STXBP6	syntaxin binding protein 6
57.169.367		-13.705.794	HLF	HLF, PAR bZIP transcription factor
57.127.667	0.6052094		LCMT1	leucine carboxyl methyltransferase 1
57.091.926		-1.014.203	COL6A1	collagen type VI alpha 1 chain
57.072.257	0.5518715		LTA4H	leukotriene A4 hydrolase
57.062.155		10.047.037	TES	testin LIM domain protein
57.046.736	0.7677388		NAP1L1	nucleosome assembly protein 1 like 1
57.033.411	0.5080357		RBMX	RNA binding motif protein, X-linked
57.027.589	-0.6188845		ZFPM2	zinc finger protein, FOG family member 2
57.002.532	0.4249651		RPL31	ribosomal protein L31
5.699.825	-0.7054683		P2RY13	purinergic receptor P2Y13
5.696.161	0.5403426		MED12	mediator complex subunit 12
56.949.265	0.6414098		KBTBD2	kelch repeat and BTB domain containing 2
56.906.716	0.4571689		HNRNPA2E	heterogeneous nuclear ribonucleoprotein A2/B
5.689.918	0.7504805		SAP30	Sin3A associated protein 30
56.892.405	0.6130102		MRPL48	mitochondrial ribosomal protein L48
56.879.412	0.6912271		HSP90AA1	heat shock protein 90 alpha family class A mem
56.869.001	0.4236604		ERAL1	Era like 12S mitochondrial rRNA chaperone 1
56.867.616	0.6703806		ATG3	autophagy related 3
56.851.749	0.5657976		CGRRF1	cell growth regulator with ring finger domain 1
56.822.722	0.4032934		IRGQ	immunity related GTPase Q
56.822.069	-0.7401812		TMEM47	transmembrane protein 47
56.804.466	0.5023401		RNF113A	ring finger protein 113A
56.754.541	0.4909374		APAF1	apoptotic peptidase activating factor 1
5.674.344	0.5638611		CD2BP2	CD2 cytoplasmic tail binding protein 2
56.736.009		13.746.205	TRIM16L///	tripartite motif containing 16-like///tripartite mo
56.732.562	0.6355985		KDM3B	lysine demethylase 3B
56.692.557	-0.9048332		ALAD	aminolevulinate dehydratase
56.676.569	0.4351267		RAD51	RAD51 recombinase
56.659.684	0.7058873		ARFGAP3	ADP ribosylation factor GTPase activating prote
56.658.604	0.5329742		GOLGA3	golgin A3
56.652.709	0.524729		PAPOLA	poly(A) polymerase alpha
56.625.197	0.5106548		PRKD2	protein kinase D2
56.600.047	-0.9012407		SLC17A1	solute carrier family 17 member 1
56.594.458	0.4540654		CLP1	cleavage and polyadenylation factor I subunit 1
56.594.035	0.7088928		CD46	CD46 molecule
56.592.575	-0.76981		ART4	ADP-ribosyltransferase 4 (Dombrock blood grou
5.657.398	0.6013465		WDR61	WD repeat domain 61
56.538.818		11.870.649	PLP2	proteolipid protein 2
56.533.083		-11.492.825	CDHR5	cadherin related family member 5
56.528.673	0.7167222		CLNS1A	chloride nucleotide-sensitive channel 1A

56.521.124	0.4949961		SRSF2	serine and arginine rich splicing factor 2
56.520.332	0.5436638		RPS19	ribosomal protein S19
56.513.103	0.6503725		RABGGTB	Rab geranylgeranyltransferase beta subunit
56.508.351		-11.412.235	VSIG4	V-set and immunoglobulin domain containing 4
56.460.686	-0.2822694		RARG	retinoic acid receptor gamma
56.394.449	0.6532505		GGCX	gamma-glutamyl carboxylase
56.382.712	0.5630546		H2AFY	H2A histone family member Y
56.381.301		12.127.271	TPD52	tumor protein D52
563.809	0.4548852		USP24	ubiquitin specific peptidase 24
56.362.777	0.4875094		PPIH	peptidylprolyl isomerase H
56.353.904	-0.6551476		C11orf24	chromosome 11 open reading frame 24
5.634.896	0.7456587		PRMT5	protein arginine methyltransferase 5
56.300.904	0.4433688		SPATA5L1	spermatogenesis associated 5 like 1
56.298.414	0.574567		NCAPD2	non-SMC condensin I complex subunit D2
56.282.717		-15.017.687	CES2	carboxylesterase 2
56.272.581	0.6187186		TTC37	tetratricopeptide repeat domain 37
56.264.459	0.7823548		GRN	granulin
56.260.597	0.4655594		SRSF1	serine and arginine rich splicing factor 1
56.257.864	0.8962878		POLE3	DNA polymerase epsilon 3, accessory subunit
56.231.248		-14.921.251	RBP1	retinol binding protein 1
56.171.634	0.7689157		CALU	calumenin
56.155.866	0.7711985		TMEM50A	transmembrane protein 50A
56.147.588	0.5280109		FAF1	Fas associated factor 1
5.614.715	0.8028983		PIK3C2B	phosphatidylinositol-4-phosphate 3-kinase cat
56.143.888	0.6038662		PRKAR2B//,	protein kinase cAMP-dependent type II regulato
56.090.698	0.7580029		PHTF2	putative homeodomain transcription factor 2
56.045.843	-0.3203964		LTBP4	latent transforming growth factor beta binding p
5.604.437	0.6730988		GART	phosphoribosylglycinamide formyltransferase,
56.040.086	0.5841338		ACTG1	actin gamma 1
560.339	0.5752375		LPL	lipoprotein lipase
56.017.867	0.3954447		RPL9	ribosomal protein L9
56.014.442	-0.5835572		AQP3	aquaporin 3 (Gill blood group)
56.002.075	0.6448602		TMEM164	transmembrane protein 164
55.994.117	0.6252752		CRKL	CRK like proto-oncogene, adaptor protein
55.985.147	0.6934046		TAF5	TATA-box binding protein associated factor 5
55.969.373	-0.5411125		KIAA1217	KIAA1217
5.596.311	0.5381078		CCDC91	coiled-coil domain containing 91
55.962.467	0.5722163		SOX12	SRY-box 12
55.917.121		-14.302.247	SLC27A2	solute carrier family 27 member 2
55.902.289	0.5921897		LMNA	lamin A/C
55.870.431	0.5336118		PRPF40A	pre-mRNA processing factor 40 homolog A
55.869.526	0.4479463		BRAP	BRCA1 associated protein
55.869.414	-0.9934596		PIK3R1	phosphoinositide-3-kinase regulatory subunit 1
5.585.848	0.418704		UBA52	ubiquitin A-52 residue ribosomal protein fusion

55.840.677	-0.6948001		HEBP1	heme binding protein 1
55.838.475	0.5794454		ADA	adenosine deaminase
55.815.738	0.6929605		GRN	granulin
55.790.871		-18.407.927	FMO3	flavin containing monooxygenase 3
5.575.895	0.7634771		STIP1	stress induced phosphoprotein 1
55.749.669	0.6584252		CCNK	cyclin K
5.570.286	-0.6992039		NCOR1	nuclear receptor corepressor 1
55.685.635	-0.4240136		EPM2A	epilepsy, progressive myoclonus type 2A, Lafor
55.671.985	-0.881972		ID3	inhibitor of DNA binding 3, HLH protein
55.666.574	0.4495688		CDK5RAP1	CDK5 regulatory subunit associated protein 1
55.657.853	0.6289487		MICB	MHC class I polypeptide-related sequence B
55.655.915	0.8125686		SET	SET nuclear proto-oncogene
55.646.229	0.6372761		PSEN1	presenilin 1
55.642.696	0.4577197		RNMT	RNA guanine-7 methyltransferase
55.603.142	-0.5183325		KCNJ15	potassium voltage-gated channel subfamily J m
55.599.465	0.5453403		ACTN4	actinin alpha 4
55.564.281	-0.5022102		CACNG4	calcium voltage-gated channel auxiliary subuni
55.545.953	0.4870112		TRIP12	thyroid hormone receptor interactor 12
55.528.256	0.599753		DHX29	DEAH-box helicase 29
55.475.897	-0.807903		PINK1	PTEN induced putative kinase 1
55.472.773	0.4888024		RPS2	ribosomal protein S2
55.472.393	0.5360288		C1orf216	chromosome 1 open reading frame 216
55.465.323	0.3729658		XPO4	exportin 4
55.436.225	-0.4876701		SRSF5	serine and arginine rich splicing factor 5
55.432.943	-0.2602226		NDST3	N-deacetylase and N-sulfotransferase 3
55.406.005		-15.426.182	HPR///HP	haptoglobin-related protein///haptoglobin
55.397.968	0.4391365		COMMD3	COMM domain containing 3
5.538.802		-17.933.395	OAT	ornithine aminotransferase
55.381.031	0.6052798		CSNK2A1	casein kinase 2 alpha 1
55.377.678		13.079.234	ASNS	asparagine synthetase (glutamine-hydrolyzing)
5.535.544	0.6491168		BCO2///CD	beta-carotene oxygenase 2///cyclin dependent
55.316.457		-12.942.577	NDRG2	NDRG family member 2
55.301.755	0.743603		LOC65388	actin-related protein 2/3 complex subunit 1B-li
5.528.861	0.5807919		TMEM39A	transmembrane protein 39A
55.287.852	0.8239198		TCFL5	transcription factor like 5
55.275.931	-0.9795494		RAB26	RAB26, member RAS oncogene family
55.234.316		-1.353.963	HSD17B2	hydroxysteroid 17-beta dehydrogenase 2
55.234.067	0.7393798		YWHAQ	tyrosine 3-monooxygenase/tryptophan 5-mono
55.230.374	0.8948946		GLS	glutaminase
55.217.713	0.6386821		TIPRL	TOR signaling pathway regulator
55.215.747	0.8102389		BSG	basigin (Ok blood group)
55.206.627	-0.8203485			
55.176.133	0.6145557		PIGF	phosphatidylinositol glycan anchor biosynthesi
55.159.192	0.7087366		SMC1B///R	structural maintenance of chromosomes 1B///r

55.125.725	-0.2983657			
55.108.679	0.8148888		MSH6	mutS homolog 6
55.104.724		10.310.418	SPARC	secreted protein acidic and cysteine rich
55.090.115	-0.2259432		DTX3	deltex E3 ubiquitin ligase 3
55.088.126	-0.6544774		CTSB	cathepsin B
55.062.923	-0.4471721		ID4	inhibitor of DNA binding 4, HLH protein
55.062.462	0.5555874		FAM168B	family with sequence similarity 168 member B
55.060.579		-12.095.715	LIPG	lipase G, endothelial type
55.058.304	-0.8305608		PCTP	phosphatidylcholine transfer protein
55.025.307	0.5584079		TBL2	transducin (beta)-like 2
55.019.029	0.5429135		SFPQ	splicing factor proline and glutamine rich
55.004.592		-16.369.811	UGT2B15	UDP glucuronosyltransferase family 2 member
54.947.874	0.5367967		SNX3	sorting nexin 3
54.947.218	0.7597847		STOML2	stomatin like 2
54.936.624	-0.735417		ALDH4A1	aldehyde dehydrogenase 4 family member A1
54.936.547	-0.6165272		KCNK5	potassium two pore domain channel subfamily
54.899.409	-0.6621574		EPOR	erythropoietin receptor
54.874.401	0.7863823		TLE1	transducin like enhancer of split 1
54.864.168		11.183.327	GLS	glutaminase
54.860.275	-0.2657254			
54.789.411	0.5708034		DNAJC25-G	DNAJC25-GNG10 readthrough///G protein subu
54.774.656	0.5677396		TCF3	transcription factor 3
54.769.862	-0.4467037		ECE1	endothelin converting enzyme 1
5.475.988	0.6100451		TCF3	transcription factor 3
54.753.245		-21.476.311	CPS1	carbamoyl-phosphate synthase 1
54.743.602	0.6856482		UBR7	ubiquitin protein ligase E3 component n-recogr
54.739.963	-0.293679			
54.737.685	0.5305998		JRKL	JRK-like
54.715.855	0.4534002		MRPL43///S	mitochondrial ribosomal protein L43///SWI/SNI
54.715.767	0.5039855		SF3B5	splicing factor 3b subunit 5
54.687.467	0.3616888		SRPK2	SRSF protein kinase 2
54.655.065	0.4838609		NDUFA6	NADH:ubiquinone oxidoreductase subunit A6
54.641.944	-0.7828932		SAMD4A	sterile alpha motif domain containing 4A
54.523.961	-0.6107278		THSD4	thrombospondin type 1 domain containing 4
54.510.776	0.5296543		SPATA2	spermatogenesis associated 2
54.507.345	0.5219082		UBE3B	ubiquitin protein ligase E3B
54.502.802	0.7011175		RAB11A	RAB11A, member RAS oncogene family
54.485.908	0.5942253		EIF3A	eukaryotic translation initiation factor 3 subunit
5.448.068	-0.3096639		SGCD	sarcoglycan delta
54.474.092	0.5408399		SEPT9	septin 9
54.459.505	0.6786035		CASP8AP2	caspase 8 associated protein 2
5.441.295	0.5319847		ANXA11	annexin A11
54.402.003	0.5856909		TAX1BP1	Tax1 binding protein 1
54.393.575		-1.506.008	DPYS	dihydropyrimidinase

54.393.511	0.494622		BRMS1	breast cancer metastasis suppressor 1
54.390.731	0.5044363		SAP30BP	SAP30 binding protein
54.350.521	-0.8240321		AMOTL2	angiomotin like 2
5.433.038	0.2668745		RPL41	ribosomal protein L41
54.327.888		-12.252.275	F12	coagulation factor XII
54.305.005	0.6226691		PFDN6	prefoldin subunit 6
54.302.663	0.6796263		RPA2	replication protein A2
54.298.085	0.7552848		TMEM41B	transmembrane protein 41B
54.292.712	-0.3867725		AK2	adenylate kinase 2
54.282.716	0.7264474		COPS8	COP9 signalosome subunit 8
54.257.539		14.053.644	PHLDA2	pleckstrin homology like domain family A meml
54.206.161		11.178.144	SLC7A1	solute carrier family 7 member 1
54.196.742	0.5251273		FASTKD5	FAST kinase domains 5
54.153.094	-0.4992933			
54.139.548	-0.3836757		RCE1	Ras converting CAAX endopeptidase 1
54.086.853	-0.6764729		PQLC1	PQ loop repeat containing 1
54.073.033		-17.746.048	SULT2A1	sulfotransferase family 2A member 1
54.062.492	0.559264		PA2G4///DI	proliferation-associated 2G4///dihydrolipoamic
54.062.342	-0.2914642			
5.406.232	0.4334549		DHX8	DEAH-box helicase 8
54.060.266	-0.6924109		CRAT	carnitine O-acetyltransferase
54.035.775	0.5395575		PSMD13	proteasome 26S subunit, non-ATPase 13
54.035.342		-10.534.684	PCSK6	proprotein convertase subtilisin/kexin type 6
54.032.942	-0.5452828		COQ4	coenzyme Q4
54.019.988	0.4567549		SEC13	SEC13 homolog, nuclear pore and COPII coat c
54.009.879	-0.4615589		LILRB3///LI	leukocyte immunoglobulin like receptor B3///le
53.987.508	0.6322916		UBE2J1	ubiquitin conjugating enzyme E2 J1
53.983.071	0.604108		SPCS1	signal peptidase complex subunit 1
53.954.301	0.5155737		EIF2AK1	eukaryotic translation initiation factor 2 alpha k
53.952.892	-0.8003447		ESRP2	epithelial splicing regulatory protein 2
53.938.009	-0.6052516		PDXDC1	pyridoxal dependent decarboxylase domain cor
53.934.086	-0.9681891		PAPSS2	3'-phosphoadenosine 5'-phosphosulfate syntha
53.873.575	-0.9576291		CYP27A1	cytochrome P450 family 27 subfamily A membe
5.385.945		-17.497.151	UGT2B17//.	UDP glucuronosyltransferase family 2 member
53.842.523	0.4794061		STK25	serine/threonine kinase 25
53.803.729	-0.386051		SNRK	SNF related kinase
53.788.143	0.5742899		PAIP1	poly(A) binding protein interacting protein 1
53.782.222		-21.770.568	IGKV2-24//.	immunoglobulin kappa variable 2-24///immunc
5.373.018	-0.3131434		MAG///CD2	myelin associated glycoprotein///CD22 molecu
53.678.334	-0.5608943		RNF123	ring finger protein 123
53.653.759	0.4663986		SETD2	SET domain containing 2
53.646.633	-0.5282106		PEBP1	phosphatidylethanolamine binding protein 1
53.636.466	0.7275284			
5.363.639	0.5964945		MTX2	metaxin 2

53.623.542		-21.744.536	IGKV2-24//.	immunoglobulin kappa variable 2-24///immunc
53.597.029	0.6975283		PACSIN2	protein kinase C and casein kinase substrate in
53.590.309	-0.9582567			
5.358.772	-0.3596805		TMPRSS2	transmembrane protease, serine 2
53.565.837	0.5747465		TNIP2	TNFAIP3 interacting protein 2
53.495.699	0.3190467		PRIM2	primase (DNA) subunit 2
5.348.455	-0.5099351		LRRN3	leucine rich repeat neuronal 3
5.345.166	0.5931082		MATR3	matrin 3
53.444.033	0.5601689		GNAI3	G protein subunit alpha i3
53.422.385	0.6593903		KIAA0100	KIAA0100
53.410.287	0.7556097		EIF3M	eukaryotic translation initiation factor 3 subunit
53.376.386		-19.595.304	CPS1	carbamoyl-phosphate synthase 1
53.368.101		-12.812.966	NEAT1	nuclear paraspeckle assembly transcript 1 (nor
5.334.547		-13.510.258	MME	membrane metallo-endopeptidase
53.338.904	0.521319		TCOF1	treacle ribosome biogenesis factor 1
53.335.299	-0.5961782		RBP4	retinol binding protein 4
533.219	0.6738082		QKI	QKI, KH domain containing RNA binding
53.307.222	0.7295966		CHTOP	chromatin target of PRMT1
53.305.363	-0.8540569		MARC2	mitochondrial amidoxime reducing component
53.289.121	0.8792691		SMURF2	SMAD specific E3 ubiquitin protein ligase 2
53.270.044	0.6577518		RALBP1	ralA binding protein 1
53.267.386	0.4708733		PTBP1	polypyrimidine tract binding protein 1
53.253.762	0.6278837		MARCH6	membrane associated ring-CH-type finger 6
53.251.577	-0.383782		LRCH4	leucine rich repeats and calponin homology do
53.245.989	-0.8117941		SLC5A3	solute carrier family 5 member 3
53.245	0.7157355		IPO7	importin 7
53.235.318	0.5904149		INHBC	inhibin beta C subunit
5.323.245	0.5049807		SFT2D2	SFT2 domain containing 2
53.231.376		-10.646.239	ALDH4A1	aldehyde dehydrogenase 4 family member A1
53.190.584	0.5460654		TIGAR	TP53 induced glycolysis regulatory phosphatas
53.175.302	0.7075211		ASAP1	ArfGAP with SH3 domain, ankyrin repeat and PH
5.313.968	-0.8964884		SMARCA2	SWI/SNF related, matrix associated, actin depe
53.131.061	0.5751112		ACTG1	actin gamma 1
53.127.292	0.5095567		RBM4	RNA binding motif protein 4
5.310.493	0.6138112		SMNDC1	survival motor neuron domain containing 1
53.092.042		-18.170.049	HSD17B6	hydroxysteroid 17-beta dehydrogenase 6
53.075.292	0.43015		TRIM27	tripartite motif containing 27
53.074.766	0.6025232		PDS5A	PDS5 cohesin associated factor A
53.071.031	-0.7286598		PRRG4	proline rich and Gla domain 4
53.061.439	0.5524828		ACTG1	actin gamma 1
53.045.043	0.2961273		BCL9	B-cell CLL/lymphoma 9
53.041.942	-0.897479		TMPRSS6	transmembrane protease, serine 6
53.025.149	0.5893369		SEPHS1	selenophosphate synthetase 1
53.008.385	0.5089024		NOP10	NOP10 ribonucleoprotein

52.991.278		-11.314.031	FGA	fibrinogen alpha chain
52.967.618	0.685234		VOPP1	vesicular, overexpressed in cancer, prosurvival
52.921.778		-10.154.315	ALDH5A1	aldehyde dehydrogenase 5 family member A1
52.918.563		10.327.973	TUBB2A	tubulin beta 2A class IIa
52.894.905	0.6051942		RAD50	RAD50 double strand break repair protein
52.884.379	-0.973491		FOLH1	folate hydrolase (prostate-specific membrane a
52.865.669	0.3831467		DGCR14	DiGeorge syndrome critical region gene 14
52.856.481		-1.272.739	CD163	CD163 molecule
52.856.089	0.6574641		CAND1	cullin associated and neddylation dissociated 1
52.842.811	0.5480662		UBE2D2	ubiquitin conjugating enzyme E2 D2
52.822.226	0.5863274		LIN37	lin-37 DREAM MuvB core complex component
52.821.887	0.649028		VDAC1	voltage dependent anion channel 1
52.809.448	0.6111317		PWP1	PWP1 homolog, endonuclein
52.773.496	0.6511906		ILF3	interleukin enhancer binding factor 3
52.767	0.7188378		SEC23IP	SEC23 interacting protein
52.765.312	0.5444801		TTI1	TELO2 interacting protein 1
52.750.222	0.6340876		SLC33A1	solute carrier family 33 member 1
52.717.923	0.6169527		RCBTB1	RCC1 and BTB domain containing protein 1
52.689.523	-0.6564248		PDLIM2	PDZ and LIM domain 2
52.673.997	0.5499078		ATRAID	all-trans retinoic acid induced differentiation fa
5.264.399	0.581072		PPP2R1A	protein phosphatase 2 scaffold subunit Aalpha
52.643.248		-10.993.456	TSKU	tsukushi, small leucine rich proteoglycan
52.642.898	0.9309693		FZD6	frizzled class receptor 6
52.635.669		-10.553.842	SELENBP1	selenium binding protein 1
52.628.163		-16.573.103	OTC	ornithine carbamoyltransferase
52.627.363	0.486621		KDM1A	lysine demethylase 1A
52.622.145	0.7617167		IVNS1ABP	influenza virus NS1A binding protein
52.616.229	0.620797		MMP11	matrix metallopeptidase 11
52.614.391	0.5746288		SLC1A3	solute carrier family 1 member 3
52.611.729	0.8200691		MCAM	melanoma cell adhesion molecule
52.600.614	0.6750876		NAA35	N(alpha)-acetyltransferase 35, NatC auxiliary s
52.575.392		-15.549.926	CYP1A1	cytochrome P450 family 1 subfamily A member
52.569.984	0.4866411		ZFYVE26	zinc finger FYVE-type containing 26
52.567.731	0.6220451		PMF1	polyamine-modulated factor 1
5.256.712	0.7817183		LARP1	La ribonucleoprotein domain family member 1
52.558.985	0.7311004		ZNHIT3	zinc finger HIT-type containing 3
52.533.377	-0.6402075		SPG7	SPG7, paraplegin matrix AAA peptidase subunit
52.511.202	0.6664636		RB1CC1	RB1 inducible coiled-coil 1
52.497.434	0.334327		KIF18A	kinesin family member 18A
52.474.754	0.4696738		LAMTOR5	late endosomal/lysosomal adaptor, MAPK and
52.450.491	0.811507		SKP1	S-phase kinase-associated protein 1
52.370.615	0.3838809		TIMM10B	translocase of inner mitochondrial membrane 1
52.365.204	0.8360681		ACSL3	acyl-CoA synthetase long-chain family member
52.335.441	0.4631051		ASNSD1	asparagine synthetase domain containing 1

52.327.169	0.6782534		BMPR1A	bone morphogenetic protein receptor type 1A
52.315.707	0.676834		UNG	uracil DNA glycosylase
52.300.052	0.5932777		GFPT1	glutamine--fructose-6-phosphate transaminas
52.295.757	0.653434		CYC1	cytochrome c1
52.275.684	0.5292695			
52.201.317	0.5211514		RPS19	ribosomal protein S19
52.185.334	-0.7274743		HADH	hydroxyacyl-CoA dehydrogenase
52.181.872		-12.753.612	ZBTB16	zinc finger and BTB domain containing 16
52.148.493	0.5589062		KPNB1	karyopherin subunit beta 1
52.143.283	-0.4759774		FBXW11	F-box and WD repeat domain containing 11
52.124.188	0.4900962		MRPS18A	mitochondrial ribosomal protein S18A
52.119.096		-14.103.419	PDK4	pyruvate dehydrogenase kinase 4
52.112.497	-0.4573299		KDM6B	lysine demethylase 6B
52.070.212	-0.5530063		KIAA0894	KIAA0894 protein
52.020.281	0.6450412		ZC3H15	zinc finger CCCH-type containing 15
52.016.428	0.4873057		NIPBL	NIPBL, cohesin loading factor
51.941.633	0.5289278		MRPL18	mitochondrial ribosomal protein L18
51.927.636		-10.593.428	NR0B2	nuclear receptor subfamily 0 group B member
5.189.016	0.5790984		ACTG1	actin gamma 1
51.876.734	0.5945756		KRT10	keratin 10
51.850.723	-0.6006908		BAIAP2	BAI1 associated protein 2
51.836.869		21.709.953	NQO1	NAD(P)H quinone dehydrogenase 1
5.181.926	-0.8688638		PHLDA1	pleckstrin homology like domain family A mem
51.810.872		10.289.816	FAM60A	family with sequence similarity 60 member A
51.805.319	-0.9851627		GADD45G	growth arrest and DNA damage inducible gam
51.800.123	0.4891349		ATP6AP2	ATPase H+ transporting accessory protein 2
51.765.125	0.4304333		SEC61B	Sec61 translocon beta subunit
51.747.454	-0.5647315		WBP1L	WW domain binding protein 1-like
51.739.648	0.5468485		OSER1	oxidative stress responsive serine rich 1
51.708.224	0.4629023		EIF3M	eukaryotic translation initiation factor 3 subuni
5.170.397	0.6454509		SLC29A1	solute carrier family 29 member 1 (Augustine b
51.701.485	0.7665324		IPO7	importin 7
51.691.295	-0.9744296		DUSP6	dual specificity phosphatase 6
51.676.187	0.6258685		ARFGEF1	ADP ribosylation factor guanine nucleotide exc
51.675.811	0.4395824		DAXX	death domain associated protein
51.664.463	-0.6419071		HP	haptoglobin
51.664.006		-13.567.661	HABP2	hyaluronan binding protein 2
51.645.711	-0.5212917		FOXF1	forkhead box F1
51.638.496	-0.9896277		ABLIM3	actin binding LIM protein family member 3
51.636.072	0.7083716		CHMP2B	charged multivesicular body protein 2B
51.585.793	0.4412775		IQCB1	IQ motif containing B1
51.570.238	-0.8785925		PLPP3	phospholipid phosphatase 3
51.557.326	0.9435743		APOLD1	apolipoprotein L domain containing 1
51.535.579	0.4507054		ZSCAN16	zinc finger and SCAN domain containing 16

51.528.869		12.703.398	VNN2	vanin 2
51.517.757	0.6203182		CDK5	cyclin dependent kinase 5
51.497.669	0.7668516		TMF1	TATA element modulatory factor 1
51.493.573	-0.9730004		GOT1	glutamic-oxaloacetic transaminase 1
51.483.536	-0.7822257		HNRNPD	heterogeneous nuclear ribonucleoprotein D
5.147.408	0.3840828		LSM14A	LSM14A, mRNA processing body assembly fact
51.451.214	0.7181616		CCNE1	cyclin E1
51.433.036	-0.2588628		EPHB1	EPH receptor B1
51.428.852	0.4994002		COPB1	coatomer protein complex subunit beta 1
51.397.623	0.4775321		KDM3B	lysine demethylase 3B
51.369.761	0.5814299		SREBF2	sterol regulatory element binding transcription
51.365.395	0.5102456		RTCB	RNA 2',3'-cyclic phosphate and 5'-OH ligase
51.320.288	0.5420415		PPP1R12A	protein phosphatase 1 regulatory subunit 12A
51.315.627	0.4587185		ATXN10	ataxin 10
51.278.867	0.6098823		GCN1	GCN1, eIF2 alpha kinase activator homolog
51.216.636	0.6229108		VPS28	VPS28, ESCRT-I subunit
5.113.796		-1.235.652	ANGPTL4	angiopoietin like 4
51.115.716		10.795.267	TPD52	tumor protein D52
51.098.694	0.662319		WDR61	WD repeat domain 61
51.087.304	0.5956889		HSPE1	heat shock protein family E (Hsp10) member 1
51.068.814	0.4394785		COA4	cytochrome c oxidase assembly factor 4 homol
51.066.007	0.6157763		CHMP2B	charged multivesicular body protein 2B
51.050.043	0.6866714		MED17	mediator complex subunit 17
51.033.643	0.3081628		MED22	mediator complex subunit 22
51.000.829	0.4921494		TMEM248	transmembrane protein 248
5.098.001	0.6699543		SAR1A	secretion associated Ras related GTPase 1A
50.948.404	-0.7521941		CLN8	ceroid-lipofuscinosis, neuronal 8
50.943.637	0.5625858		ACTG1	actin gamma 1
50.937.051	0.5143724		DYRK2	dual specificity tyrosine phosphorylation regula
50.881.913	0.7126469		TTC39A	tetratricopeptide repeat domain 39A
50.806.956	0.4652908		IQCB1	IQ motif containing B1
50.794.698	0.4396405		PTBP1	polypyrimidine tract binding protein 1
50.792.855	-0.3795528		IL18R1	interleukin 18 receptor 1
50.780.381	0.8236114		PAPSS1	3'-phosphoadenosine 5'-phosphosulfate synth
50.770.209	0.6024172		SUPT4H1	SPT4 homolog, DSIF elongation factor subunit
5.075.341	0.4838032		TSEN34	tRNA splicing endonuclease subunit 34
5.072.461	-0.8444076		KANK1///A(	KN motif and ankyrin repeat domains 1///aconi
50.716.684	0.588476		IDE	insulin degrading enzyme
5.070.614	0.3957775		RBMX2	RNA binding motif protein, X-linked 2
50.705.302	0.8630213		PAPOLA	poly(A) polymerase alpha
50.677.326	0.5522657		ACTR10	actin-related protein 10 homolog
50.674.483	-0.3151635		RUNX1T1	RUNX1 translocation partner 1
50.671.063	0.5629477		NPLOC4	NPL4 homolog, ubiquitin recognition factor
50.666.139		10.682.806	CPD	carboxypeptidase D

50.642.304	0.6273432		RAD54B	RAD54 homolog B (S. cerevisiae)
50.632.562		-13.432.446	CCL16	C-C motif chemokine ligand 16
50.630.689	-0.2988519			
50.627.024	0.5564338		NF1	neurofibromin 1
50.625.769	-0.5551329		RHEB	Ras homolog enriched in brain
50.621.044		13.096.969	SOX4	SRY-box 4
50.599.884	0.6512516		NEDD4L	neural precursor cell expressed, developmenta
50.595.191	0.4244133		UBE2D1	ubiquitin conjugating enzyme E2 D1
50.563.143		-15.392.224	HLA-C	major histocompatibility complex, class I, C
50.556.006	0.5981198		HNRNPA3	heterogeneous nuclear ribonucleoprotein A3
50.529.558		-11.457.787	SLC47A1	solute carrier family 47 member 1
50.521.563	0.55235		CRIPT	CXXC repeat containing interactor of PDZ3 dom
50.504.328	0.4003109		C12orf4	chromosome 12 open reading frame 4
50.502.948	0.5624156		ITGB1	integrin subunit beta 1
5.049.865	-0.4511737		SLC16A3	solute carrier family 16 member 3
50.493.879	0.6210041		DZIP3	DAZ interacting zinc finger protein 3
50.482.749	0.8133285		MTFR1	mitochondrial fission regulator 1
50.477.235	0.3965719		MRPL58	mitochondrial ribosomal protein L58
50.466.334		-1.577.677	UPB1	beta-ureidopropionase 1
50.442.109		11.118.153	TM4SF1	transmembrane 4 L six family member 1
50.405.056	0.4954773		PDE6D	phosphodiesterase 6D
50.374.519	0.5114504		PSMB2	proteasome subunit beta 2
50.370.033		13.853.564	NEB	nebulin
50.356.706		12.054.829	KDELR3	KDEL endoplasmic reticulum protein retention
50.338.443	0.5860473		DYRK2	dual specificity tyrosine phosphorylation regula
50.331.311	0.5497299		SHCBP1	SHC binding and spindle associated 1
50.311.057	0.6573615		KIAA0368	KIAA0368
5.030.372		-10.946.188	ID4	inhibitor of DNA binding 4, HLH protein
50.301.836	-0.7706691		COL18A1	collagen type XVIII alpha 1 chain
50.273.358	-0.4486097		LOC10050	uncharacterized LOC100507424
50.255.101	0.5622629		WBSCR22	Williams-Beuren syndrome chromosome regior
50.186.823	0.5434391		RPP38	ribonuclease P/MRP subunit p38
50.178.257	0.4895743		RAE1	ribonucleic acid export 1
50.176.251	0.4888166		ZNF410	zinc finger protein 410
50.168.446	0.514394		HDAC4	histone deacetylase 4
50.149.304	-0.3709394		IL18RAP	interleukin 18 receptor accessory protein
50.144.071	0.6831018		PTK2	protein tyrosine kinase 2
50.140.977	-0.8492704		COQ9	coenzyme Q9
50.131.307	0.3674111		TDP1	tyrosyl-DNA phosphodiesterase 1
50.070.444	0.4481484		RPS4X	ribosomal protein S4, X-linked
50.052.183	0.5287794		CAP1	adenylate cyclase associated protein 1
50.036.273	0.5236174		LSM14A	LSM14A, mRNA processing body assembly fact
50.015.194	-0.4248195		CAMK2B	calcium/calmodulin dependent protein kinase
49.993.466		16.899.026		

49.983.068		-16.393.009	HRG	histidine rich glycoprotein
49.981.364	0.3852809		PPCDC	phosphopantothenoylcysteine decarboxylase
49.895.317	-0.5881216		NEIL1	nei like DNA glycosylase 1
49.880.615	0.8019442		PRKCI	protein kinase C iota
49.878.531		-12.293.915	KCNJ8	potassium voltage-gated channel subfamily J m
49.877.113	0.423382		TRNAU1AP	tRNA selenocysteine 1 associated protein 1
49.871.971	0.4001111		ZNF184	zinc finger protein 184
49.863.235	0.4434207		MRPL49	mitochondrial ribosomal protein L49
49.856.023	0.3723462		AP5Z1	adaptor related protein complex 5 zeta 1 subun
49.840.149		15.045.597	ENPP2	ectonucleotide pyrophosphatase/phosphodies
49.816.433	0.4503726		DGUOK	deoxyguanosine kinase
49.811.592	0.6635238		TMEM126B	transmembrane protein 126B
49.808.296	-0.2876207		RNASEL	ribonuclease L
49.795.699	0.4667985		NUDT1	nudix hydrolase 1
49.793.307	0.6822268		EDEM3	ER degradation enhancing alpha-mannosidase
4.977.834	0.578213		IFT81	intraflagellar transport 81
49.765.504	0.8282969		LOC64785	occludin pseudogene
49.756.425	0.7212013		NCOA2	nuclear receptor coactivator 2
4.973.767	0.5866569		SYNCRIP	synaptotagmin binding cytoplasmic RNA intera
49.725.482	-0.4512172		IFT46	intraflagellar transport 46
49.717.108	-0.4376133		KLF12	Kruppel like factor 12
49.690.415	-0.894106		F8	coagulation factor VIII
49.680.718	-0.506522		HECTD3	HECT domain E3 ubiquitin protein ligase 3
49.679.847	0.8024309		KDELR2	KDEL endoplasmic reticulum protein retention
49.663.988	0.5076816		WAC	WW domain containing adaptor with coiled-coi
49.654.016	0.3914287		RPS17	ribosomal protein S17
4.962.148	0.5077586		DDX27	DEAD-box helicase 27
49.608.049		-12.133.693	GREM2	gremlin 2, DAN family BMP antagonist
49.592.758	0.6234527		KIF5B	kinesin family member 5B
49.559.268	0.508724		CHEK1	checkpoint kinase 1
49.526.985		12.665.294	TOX3	TOX high mobility group box family member 3
49.523.486	0.4062455		GART	phosphoribosylglycinamide formyltransferase,
49.512.578	0.6801221		RPLP0P6///	ribosomal protein lateral stalk subunit P0 pseu
49.504.125	-0.489656		CTIF	cap binding complex dependent translation init
49.474.525	0.4918482		MORF4L1	mortality factor 4 like 1
49.472.698	0.6520854		UBE2M	ubiquitin conjugating enzyme E2 M
49.456.871	-0.5260299		SIRT3	sirtuin 3
49.440.169	0.657159		CHUK	conserved helix-loop-helix ubiquitous kinase
49.432.454	0.5442296		APC	APC, WNT signaling pathway regulator
4.941.831	0.6349984		ARHGAP12	Rho GTPase activating protein 12
494.149	-0.4567736		HDAC6	histone deacetylase 6
49.408.673	-0.3835152		LILRB3///LI	leukocyte immunoglobulin like receptor B3///le
49.356.404	-0.8851135		POR	cytochrome p450 oxidoreductase
49.332.589	-0.4121416		TK2	thymidine kinase 2, mitochondrial

49.332.427	-0.4713685		WAS	Wiskott-Aldrich syndrome
49.303.092		11.546.736	COL4A2	collagen type IV alpha 2 chain
49.296.201	-0.7779963		PTN	pleiotrophin
49.289.381	0.6977337		ATP1B1	ATPase Na+/K+ transporting subunit beta 1
49.272.719	0.8041311		USP1	ubiquitin specific peptidase 1
49.264.366	0.5303969		GOSR2	golgi SNAP receptor complex member 2
49.207.717	0.489809		CEP83	centrosomal protein 83
49.191.472	0.5708782		COPS2	COP9 signalosome subunit 2
4.918.562	-0.6091905		PPFIBP2	PPFIA binding protein 2
49.183.992		-12.953.813	APOA1	apolipoprotein A1
49.175.138	0.4478765		TXNL1	thioredoxin like 1
49.152.485	0.838959		ATP1A1	ATPase Na+/K+ transporting subunit alpha 1
49.150.293	0.4621904		SEC11A	SEC11 homolog A, signal peptidase complex su
49.126.891	0.6625535		ANP32A	acidic nuclear phosphoprotein 32 family memb
4.911.264	0.4627797		SF3B2	splicing factor 3b subunit 2
49.078.334	-0.2430267		LPAL2	lipoprotein(a) like 2, pseudogene
49.076.006	0.5903341		MOB1A	MOB kinase activator 1A
49.066.849	0.5122724		ITPA	inosine triphosphatase
49.017.218	0.4044668		THUMPD2	THUMP domain containing 2
48.995.919	0.3898138		SUMO4	small ubiquitin-like modifier 4
48.980.953	0.4040431		AAR2	AAR2 splicing factor homolog
48.980.605	0.5269209		ZMYM1	zinc finger MYM-type containing 1
4.896.529	0.4480411		ATP5SL	ATP5S like
48.963.123	0.6008229		POLD3	DNA polymerase delta 3, accessory subunit
48.957.063	0.654812		RBM8A	RNA binding motif protein 8A
48.895.694	0.4337542		DDX23	DEAD-box helicase 23
48.829.636	-0.9784708		PAPSS2	3'-phosphoadenosine 5'-phosphosulfate synth
48.818.621	-0.5193737		ARHGAP25	Rho GTPase activating protein 25
48.810.412	0.8226045		SRP72	signal recognition particle 72
48.800.407	0.4194969		TRA2B	transformer 2 beta homolog (Drosophila)
48.789.594	0.3150211		SPIDR	scaffolding protein involved in DNA repair
4.878.284	-0.3504417			
48.769.372	-0.329327		GNAO1	G protein subunit alpha o1
48.755.818	0.6289011		ZYX	zyxin
48.748.386	0.4366549		IDE	insulin degrading enzyme
48.739.194	0.8805828		TPD52	tumor protein D52
48.737.746	0.6499217		ICK	intestinal cell (MAK-like) kinase
48.727.444	0.6389886		TMEM165	transmembrane protein 165
48.710.463	-0.3349971		SFRP1	secreted frizzled related protein 1
4.869.205	0.4361794		ABCC10	ATP binding cassette subfamily C member 10
48.679.151	-0.5368299		ZFAND6	zinc finger AN1-type containing 6
48.675.802	0.5721213		TBCCD1	TBCC domain containing 1
48.670.205	0.618399		TARS	threonyl-tRNA synthetase
48.665.802	-0.6268971		KLRB1	killer cell lectin like receptor B1

48.641.674	0.3501478		PDS5A	PDS5 cohesin associated factor A
48.638.437	0.7521129		COMMD10	COMM domain containing 10
48.616.202	0.7958492		OSTM1	osteopetrosis associated transmembrane prot
48.592.915	-0.7014217		ABHD2	abhydrolase domain containing 2
4.856.647		-13.281.645	APOA1	apolipoprotein A1
4.855.802	0.6770237		HIPK1	homeodomain interacting protein kinase 1
48.524.825	-0.3194332		RBMS3	RNA binding motif single stranded interacting p
48.519.249		-10.622.547	CRISPLD2	cysteine rich secretory protein LCCL domain cc
48.515.521	0.6788477		EPPK1	epiplakin 1
48.494.613	0.5708951		CCDC47	coiled-coil domain containing 47
48.492.419	0.7021559		VAMP5	vesicle associated membrane protein 5
48.491.596	0.3723333		CHAF1B	chromatin assembly factor 1 subunit B
48.486.317	-0.9556921		MARC2	mitochondrial amidoxime reducing component
4.848.453		-19.267.855	IGLV2-14//	immunoglobulin lambda variable 2-14///immur
48.461.816		-12.336.515	PPARGC1A	PPARG coactivator 1 alpha
48.430.774	0.8810943		DERL1	derlin 1
48.414.421	-0.3336513			
48.410.824	0.7892171		RBBP4	RB binding protein 4, chromatin remodeling fac
48.400.204	0.6224825		UNC119B	unc-119 lipid binding chaperone B
48.383.274	0.4835264		TBC1D12	TBC1 domain family member 12
48.371.576	0.5184927		NIPBL	NIPBL, cohesin loading factor
48.333.693		13.703.549	ENPP2	ectonucleotide pyrophosphatase/phosphodies
4.832.749	0.5691936		KRT10	keratin 10
4.829.057	-0.6836847		METTL7A	methyltransferase like 7A
48.286.869		-10.114.836	NR4A2	nuclear receptor subfamily 4 group A member 2
48.257.582	0.4909463		APPL1	adaptor protein, phosphotyrosine interacting w
48.247.605	-0.3661811		HMX1	H6 family homeobox 1
48.185.506	-0.7689896		ASB13	ankyrin repeat and SOCS box containing 13
48.180.855	0.5245424		TAF11	TATA-box binding protein associated factor 11
48.166.669	0.5926062		EIF3J	eukaryotic translation initiation factor 3 subuni
4.816.494	0.6982419		LGALS8	galectin 8
48.152.827	0.4586906		TTLL5	tubulin tyrosine ligase like 5
48.149.504	-0.8627269		ACOX1	acyl-CoA oxidase 1
48.116.015	0.6932567		MKKS	McKusick-Kaufman syndrome
48.111.632	0.6333036		NDUFA8	NADH:ubiquinone oxidoreductase subunit A8
48.102.085	0.4395049		YIPF6	Yip1 domain family member 6
48.095.774		-10.184.066	CFTR	cystic fibrosis transmembrane conductance re
48.090.965		-14.630.429	G6PC	glucose-6-phosphatase catalytic subunit
4.805.324	0.4732751		URI1	URI1, prefoldin like chaperone
48.036.015	-0.2665589			
48.025.132		-13.730.081	SULT2A1	sulfotransferase family 2A member 1
48.020.441	-0.4545782		SRSF4	serine and arginine rich splicing factor 4
48.002.241		-16.367.734	CYP2D6	cytochrome P450 family 2 subfamily D membe
47.996.041	-0.6137355		MGST2	microsomal glutathione S-transferase 2

47.986.493	0.4592257		CDV3	CDV3 homolog
47.980.148	0.9665199		RGS5	regulator of G-protein signaling 5
47.978.882	-0.7493038		HIBCH	3-hydroxyisobutyryl-CoA hydrolase
47.978.144	0.6889626		PCDH17	protocadherin 17
47.963.138	0.5399382		ATP6V0E1	ATPase H+ transporting V0 subunit e1
47.951.885	-0.3956869		MCC	mutated in colorectal cancers
47.945.603		13.295.327	LGALS3	lectin, galactoside binding soluble 3
47.822.559	0.485581		EXO1	exonuclease 1
47.800.119	-0.3840922		COMT///AR	catechol-O-methyltransferase///armadillo repe
47.786.193	0.5524068		MATR3	matrin 3
47.772.361	0.4288023		GPN2	GPN-loop GTPase 2
47.771.745	-0.4977343		CEPT1	choline/ethanolamine phosphotransferase 1
47.769.262	0.5119185		POLDIP3	DNA polymerase delta interacting protein 3
47.750.041	0.5139162		GPATCH1	G-patch domain containing 1
47.749.439		-12.498.861	ANGPTL3	angiopoietin like 3
47.733.294	0.6852659		TMEM50B	transmembrane protein 50B
47.728.122	0.5378409		MAP3K9	mitogen-activated protein kinase kinase kinase
47.720.922	-0.7986416		GUCY1A3	guanylate cyclase 1 soluble subunit alpha
47.705.487	0.6091516		OTUB1	OTU deubiquitinase, ubiquitin aldehyde binding
47.702.096	0.6286169		UBE2N	ubiquitin conjugating enzyme E2 N
47.673.194	0.383518		CHTOP	chromatin target of PRMT1
47.634.252	0.5450987		NSFL1C	NSFL1 cofactor
47.620.639	0.6548989		STAU2	staufen double-stranded RNA binding protein 2
47.609.472	-0.5940706		TEK	TEK receptor tyrosine kinase
47.603.967	0.5961973		LRPPRC	leucine rich pentatricopeptide repeat containin
47.576.606	0.5339674		FAM134A	family with sequence similarity 134 member A
47.576.427	-0.2198434		BMPER	BMP binding endothelial regulator
47.576.303	0.4116169		LOC64328	similar to prothymosin alpha///prothymosin, al
4.756.822	0.4518482		SLC39A7	solute carrier family 39 member 7
47.556.425	0.5675372		PRUNE1	prune exopolyphosphatase
47.549.841	0.5608847		ETF1	eukaryotic translation termination factor 1
47.535.697		-10.121.452	BHMT2	betaine--homocysteine S-methyltransferase 2
4.753.036	0.4737352		FAM222B	family with sequence similarity 222 member B
47.515.845	0.6594572		SMARCA4	SWI/SNF related, matrix associated, actin depe
47.479.974	-0.3173275			
47.473.103	0.5302467		HPS1	HPS1, biogenesis of lysosomal organelles comp
4.747.255	0.8945128		STK26	serine/threonine protein kinase 26
47.461.866		-11.382.132	NPC1L1	NPC1 like intracellular cholesterol transporter
4.745.851		-11.825.083	IFITM1	interferon induced transmembrane protein 1
47.452.238	-0.3110255		PDPK1	3-phosphoinositide dependent protein kinase 1
47.440.149		11.511.956	GABRE	gamma-aminobutyric acid type A receptor epsil
4.742.223	-0.7065581		STAT4	signal transducer and activator of transcription
47.391.673	0.4129834		NCAPH	non-SMC condensin I complex subunit H
47.387.992	0.692518		LMNB1	lamin B1

47.361.137	-0.2850516		SIRT3	sirtuin 3
4.727.803	0.5404771		RC3H2	ring finger and CCCH-type domains 2
4.727.376	-0.8022126		PHYH	phytanoyl-CoA 2-hydroxylase
47.273.113	0.6120382		LINC00111	long intergenic non-protein coding RNA 111///ir
47.253.568	0.651396		RABGGTB	Rab geranylgeranyltransferase beta subunit
47.246.213	-0.7215097		GZMK	granzyme K
47.231.476	0.4469462		CD63	CD63 molecule
472.245	0.7595111		GNB5	G protein subunit beta 5
4.720.801	0.2254531		TROVE2	TROVE domain family member 2
47.202.489	0.4267886		GINS4	GINS complex subunit 4
47.195.329	0.6794981		FRAT2	frequently rearranged in advanced T-cell lymph
47.167.814	0.5155057		AP3D1	adaptor related protein complex 3 delta 1 subur
4.716.279	0.5168471		NFKBIE	NFKB inhibitor epsilon
47.140.425	-0.6287049		RBKS	ribokinase
47.134.579	0.6846357		SYNJ2	synaptojanin 2
47.121.372	0.4064891		SUGP1	SURP and G-patch domain containing 1
47.121.176	0.4578079		NSUN5	NOP2/Sun RNA methyltransferase family meml
47.121.101	-0.6449694		ZFP36L1	ZFP36 ring finger protein like 1
47.119.609	0.3340935		TFPT	TCF3 fusion partner
47.112.969	0.4258942		COMMD9	COMM domain containing 9
47.057.889	0.9895045		DUSP9	dual specificity phosphatase 9
47.056.946	-0.2515567		CAMK2B	calcium/calmodulin dependent protein kinase
47.056.411	0.3472903		XRCC1	X-ray repair cross complementing 1
47.021.717	0.5718787		VGLL4	vestigial like family member 4
46.988.935	-0.5468311		C11orf24	chromosome 11 open reading frame 24
46.982.278	0.3755037		REXO4	REX4 homolog, 3'-5' exonuclease
4.694.804	0.5800907		GID8	GID complex subunit 8 homolog
46.944.353	0.511839		POLR2H	RNA polymerase II subunit H
4.694.291	-0.720514		FGB	fibrinogen beta chain
46.936.452		26.212.413	AFP	alpha fetoprotein
4.690.657	0.4110595		RMND5B	required for meiotic nuclear division 5 homolog
46.894.018	0.522004		MED12	mediator complex subunit 12
46.893.336	0.4052712		WDR70	WD repeat domain 70
46.871.302	0.5627084		TALDO1	transaldolase 1
46.846.668	-0.6337964		SNED1	sushi, nidogen and EGF like domains 1
46.842.125	0.415917		DHX57	DEAH-box helicase 57
46.835.682	-0.6048706		LMF1	lipase maturation factor 1
46.793.635		-12.742.773	TFR2	transferrin receptor 2
46.752.557	-0.2365635		DKFZp566I	hypothetical LOC54744
46.746.083		19.612.836	NQO1	NAD(P)H quinone dehydrogenase 1
4.673.905	-0.3518866		EPOR	erythropoietin receptor
46.723.464	-0.6001553		PEX14	peroxisomal biogenesis factor 14
46.712.336	0.6091102		RRM1	ribonucleotide reductase catalytic subunit M1
46.705.124	0.4370268		DARS	aspartyl-tRNA synthetase

46.664.301	-0.33547		CDRT1	CMT1A duplicated region transcript 1
46.656.511	0.5908778		LRRC8D	leucine rich repeat containing 8 family member
46.639.494	-0.5882154		LAMA2	laminin subunit alpha 2
46.635.678	0.5224972		TMEM9B	TMEM9 domain family member B
46.633.108	-0.8019425		COL18A1	collagen type XVIII alpha 1 chain
46.612.369	0.4895804		MTMR1	myotubularin related protein 1
46.605.769	0.5952311		TDRD7	tudor domain containing 7
46.592.024	-0.6697412		CLDN14	claudin 14
4.655.968		11.325.865	YWHAZ	tyrosine 3-monooxygenase/tryptophan 5-mono
46.516.971	0.7438429		MINA	MYC induced nuclear antigen
46.509.188	-0.3081063		CD44	CD44 molecule (Indian blood group)
46.491.659	0.4541829		GPATCH2	G-patch domain containing 2
46.473.919	0.5373666		DDX27	DEAD-box helicase 27
464.586	-0.8163671		PC	pyruvate carboxylase
46.420.102	0.4932182		PYGB	phosphorylase, glycogen; brain
46.419.412	0.3998404		RFWD3	ring finger and WD repeat domain 3
46.414.745	-0.6228675		N4BP2L2	NEDD4 binding protein 2 like 2
46.405.845	-0.7026969		ZNF395	zinc finger protein 395
46.402.716	0.5435581		RBM28	RNA binding motif protein 28
46.391.973	0.8281295		WWP1	WW domain containing E3 ubiquitin protein liga
46.380.242	-0.9976225		ORM2///OF	orosomucoid 2///orosomucoid 1
46.378.136	0.5211652		STX10	syntaxin 10
46.354.296	0.5662174		MRPS30	mitochondrial ribosomal protein S30
46.353.912	0.4139094		LOC73189(	similar to ribosomal protein S6 kinase, polypep
46.347.612	0.447767		KPNA4	karyopherin subunit alpha 4
46.342.426	-0.8395552		GAMT	guanidinoacetate N-methyltransferase
4.633.737	-0.9360416		PIK3R1	phosphoinositide-3-kinase regulatory subunit 1
46.335.086	0.3670761		LOC73186:	similar to zinc finger protein 282///zinc finger pr
46.315.427	-0.4442686		BIN2	bridging integrator 2
46.311.739	0.5386063		TAF12	TATA-box binding protein associated factor 12
46.279.647	-0.5070733		RREB1	ras responsive element binding protein 1
46.262.278	0.8327678		DBN1	drebrin 1
46.243.067	-0.7216654		DGCR6L///I	DiGeorge syndrome critical region gene 6-like//
46.237.427		-20.657.956	IGL	immunoglobulin lambda locus
46.232.932	0.4957084		CDK8	cyclin dependent kinase 8
46.231.808		-11.591.872	CES2	carboxylesterase 2
46.203.584	0.6848144		SHC1	SHC adaptor protein 1
4.620.192	0.8048185		PTP4A3	protein tyrosine phosphatase type IVA, member
46.198.755	0.3753833		MOSPD2	motile sperm domain containing 2
4.618.244	0.7678493		CORO1C	coronin 1C
46.178.124	0.5496228		TIMM17A	translocase of inner mitochondrial membrane 1
46.176.541		-17.575.289	VNN1	vanin 1
46.169.257		-13.334.793	CDHR5	cadherin related family member 5
46.096.629	0.7776765		UGDH	UDP-glucose 6-dehydrogenase

46.090.709	0.3748918		MYL12B	myosin light chain 12B
46.055.489	0.5735567		NUTF2	nuclear transport factor 2
46.054.937	0.8414154		PTP4A3	protein tyrosine phosphatase type IVA, member
4.604.037	-0.5276116		SRSF5	serine and arginine rich splicing factor 5
46.019.237	-0.8068856		SLC17A9	solute carrier family 17 member 9
46.003.252	0.6704912		LRIF1	ligand dependent nuclear receptor interacting f
45.999.166	0.5500461		ZBED5	zinc finger BED-type containing 5
45.999.004	0.5325261		PPP1R8	protein phosphatase 1 regulatory subunit 8
45.993.231		14.752.025		
45.985.149	0.5800139		GTF2H1	general transcription factor IIH subunit 1
45.967.019	0.6315446		ATP5G1	ATP synthase, H+ transporting, mitochondrial F
45.948.376	0.4452817		KATNB1	katanin regulatory subunit B1
45.942.274	0.6797655		TAF1D	TATA-box binding protein associated factor, RN
4.593.862	0.3678983		DHX30	DEAH-box helicase 30
45.907.283	-0.8406339		HCLS1	hematopoietic cell-specific Lyn substrate 1
45.903.558	0.591117		ZNF518A	zinc finger protein 518A
45.898.863		-22.790.819	IGLJ3///IGL	immunoglobulin lambda joining 3///immunoglo
45.897.753	0.6456601		CETN3	centrin 3
45.848.743	0.4766086		LARP4B	La ribonucleoprotein domain family member 4E
45.828.612		-1.080.279	ADGRG6	adhesion G protein-coupled receptor G6
45.826.575	0.4129492		KCMF1	potassium channel modulatory factor 1
45.797.886	0.4832098		CTNNB1	catenin beta 1
45.795.775	-0.5722052		DAAM2	dishevelled associated activator of morphogen
45.777.188	0.6297178		LOC72884	similar to putative DNA dependent ATPase and
45.758.634	-0.2727018		GDF2	growth differentiation factor 2
45.754.791		-15.654.449	IGLJ3///IGL	immunoglobulin lambda joining 3///immunoglo
45.750.927	0.7854856		IFI30	IFI30, lysosomal thiol reductase
45.736.487	-0.7174745		RETSAT	retinol saturase
45.727.263	0.4115677		COPS6	COP9 signalosome subunit 6
45.710.552	0.5820251		MIEF1	mitochondrial elongation factor 1
4.569.398	-0.2935176		CA6	carbonic anhydrase 6
45.682.302	0.5649613		CEP170	centrosomal protein 170
45.665.772	0.4731232		HTATIP2	HIV-1 Tat interactive protein 2
45.662.652	0.725559		GMCL1	germ cell-less, spermatogenesis associated 1
4.565.294	0.5504464		WNK1	WNK lysine deficient protein kinase 1
45.626.519	0.8592353		PLOD2	procollagen-lysine,2-oxoglutarate 5-dioxygena
45.582.559	0.5513595		CAND1	cullin associated and neddylation dissociated 1
45.563.911	0.4346304		NLE1	notchless homolog 1
45.548.152		16.862.074	COL1A2	collagen type I alpha 2 chain
45.545.236	0.6168497		PTDSS1	phosphatidylserine synthase 1
45.535.395	-0.6356253		EI24	EI24, autophagy associated transmembrane pr
45.516.606	0.8388988		TAGLN2	transgelin 2
45.509.108	0.3239933		RPS13	ribosomal protein S13
45.487.121	0.7023403		MRPL15	mitochondrial ribosomal protein L15

4.547.529	0.5835273		LIMS1	LIM zinc finger domain containing 1
45.442.142	-0.4219151		LRP6	LDL receptor related protein 6
45.425.556	0.5736177		TSTA3	tissue specific transplantation antigen P35B
45.390.759	0.3931151		RRP7A	ribosomal RNA processing 7 homolog A
45.370.545	0.6517453		HNRNPA0	heterogeneous nuclear ribonucleoprotein A0
45.353.975	0.577701		ZNF7	zinc finger protein 7
4.534.736	0.4013743		RPS17	ribosomal protein S17
45.339.361	0.2942017		SEC22A	SEC22 homolog A, vesicle trafficking protein
45.313.351	0.4792078		ATP2C1	ATPase secretory pathway Ca2+ transporting 1
45.287.221	0.9782244		TAX1BP3	Tax1 binding protein 3
45.283.975	0.4539722		ATG13	autophagy related 13
45.276.242	0.6400982		SEC11A	SEC11 homolog A, signal peptidase complex su
45.256.835	-0.7760176		KIAA0485	uncharacterized LOC57235
45.251.126	0.894898		EPS8L3	EPS8 like 3
45.241.169	-0.5150558		SARAF	store-operated calcium entry associated regula
45.237.864	0.6945509		ACSL3	acyl-CoA synthetase long-chain family member
45.229.319	0.5442394		G3BP1	G3BP stress granule assembly factor 1
45.219.131	0.5969202		SLC35D2	solute carrier family 35 member D2
45.214.261	0.7128145		MAP4K3	mitogen-activated protein kinase kinase kinase
45.211.134	-0.3113129		LILRA2	leukocyte immunoglobulin like receptor A2
45.208.088	-0.5122741		SAMD4A	sterile alpha motif domain containing 4A
45.205.333	-0.3190665		CD82	CD82 molecule
45.194.447	-0.5326253		SMARCA2	SWI/SNF related, matrix associated, actin depe
45.181.998	-0.3598		TMEM212	transmembrane protein 212
45.132.522		-1.126.008	SORD2P///!	sorbitol dehydrogenase 2, pseudogene///sorbit
45.105.015	0.9308248		TRAM1	translocation associated membrane protein 1
45.101.245	0.642767		CASD1	CAS1 domain containing 1
45.080.355		-24.740.135	IGHV4-31//	immunoglobulin heavy variable 4-31///immuno
45.052.136	0.432151		SLC22A5	solute carrier family 22 member 5
45.041.718	0.5774473		DKC1	dyskerin pseudouridine synthase 1
45.032.584	-0.4843937		SDHA	succinate dehydrogenase complex flavoprotein
45.029.293	0.583962		UBE3C	ubiquitin protein ligase E3C
44.998.799	0.3941888		GLMN	glomulin, FKBP associated protein
44.994.739	-0.7243723		MAOB	monoamine oxidase B
44.975.258		-11.852.034	ABCG5	ATP binding cassette subfamily G member 5
44.969.724	0.4462827		TLK2	tousled like kinase 2
44.966.968	0.7236912		SF3A2	splicing factor 3a subunit 2
44.962.984	-0.9159251		XAF1	XIAP associated factor 1
44.949.095		12.115.464	TOX3	TOX high mobility group box family member 3
44.940.892		-18.106	HPGD	hydroxyprostaglandin dehydrogenase 15-(NAD)
44.927.747	-0.921599		PAH	phenylalanine hydroxylase
44.924.481	0.5300764		ZNF271P	zinc finger protein 271, pseudogene
44.865.376	0.6172137		DUT	deoxyuridine triphosphatase
44.860.088	0.3482042		OXLD1	oxidoreductase like domain containing 1

44.848.906	-0.6814761		CHST7	carbohydrate sulfotransferase 7
44.845.738	0.4434487		KRT10	keratin 10
44.817.449	0.4858656		RAD1	RAD1 checkpoint DNA exonuclease
44.814.874	0.5124674		PDCD6IP	programmed cell death 6 interacting protein
44.810.626	0.6394149		SAMM50	SAMM50 sorting and assembly machinery com
44.788.687	0.5465252		PSMD6	proteasome 26S subunit, non-ATPase 6
44.779.838	-0.860783		LINC01314	long intergenic non-protein coding RNA 1314
44.779.604	0.5609391		ATP2A2	ATPase sarcoplasmic/endoplasmic reticulum
4.477.724	0.5691208		PRPF18	pre-mRNA processing factor 18
44.766.004	-0.3366108		SLC13A4	solute carrier family 13 member 4
44.738.851	0.4854984		LOC81691	exonuclease NEF-sp
44.737.775	0.7686388		CEP170	centrosomal protein 170
44.733.989	-0.7509362		KLF11	Kruppel like factor 11
44.730.299	0.3183486		PPIP5K1	diphosphoinositol pentakisphosphate kinase 1
44.722.994	0.4510623		ACTR1A	ARP1 actin-related protein 1 homolog A, centra
44.700.325	0.3445415		HPS4	HPS4, biogenesis of lysosomal organelles com
4.469.289	0.9481083		HMGB3	high mobility group box 3
44.688.433	0.6668673		TLK1	tousled like kinase 1
44.688.046	0.417422		BIRC5	baculoviral IAP repeat containing 5
44.655.043	-0.4885324		RER1	retention in endoplasmic reticulum sorting rece
44.639.999	0.4366758		GMPPA	GDP-mannose pyrophosphorylase A
44.632.237	-0.4180647		PPIG	peptidylprolyl isomerase G
44.626.901	0.383253		USP46	ubiquitin specific peptidase 46
44.618.982		1.002.678	HIC2	hypermethylated in cancer 2
4.461.445	0.5108802		FAM193A	family with sequence similarity 193 member A
44.599.104	0.382603		RPS17	ribosomal protein S17
44.587.217		10.403.737	MMP9	matrix metallopeptidase 9
44.566.618	0.4668593		SART1	squamous cell carcinoma antigen recognized b
44.537.392	0.5836329		LSM12	LSM12 homolog
44.535.923	-0.6480361		ASMTL	acetylserotonin O-methyltransferase-like
44.531.369	-0.6034972		RXRA	retinoid X receptor alpha
44.513.177	-0.3641775		PQBP1	polyglutamine binding protein 1
44.503.361	0.3843964		TNIP2	TNFAIP3 interacting protein 2
44.500.391	-0.6025069		CCDC28A	coiled-coil domain containing 28A
4.449.754	-0.478188		ITSN1	intersectin 1
44.488.369	0.8311371		XK	X-linked Kx blood group
4.448.529	0.6467428		RHOA	ras homolog family member A
44.481.116	0.5678791		PQBP1	polyglutamine binding protein 1
44.424.399	-0.2153685		FOXH1	forkhead box H1
44.415.961	0.3766131		MPZL1	myelin protein zero like 1
44.415.021		-12.172.166	TFR2	transferrin receptor 2
44.396.378	-0.6106411		STAG3L1	stromal antigen 3-like 1 (pseudogene)
44.391.443	0.4226753		UBL5	ubiquitin like 5
44.373.503	0.5978317		TM9SF4	transmembrane 9 superfamily member 4

4.436.986		-13.974.323	IGKV1-5///I	immunoglobulin kappa variable 1-5///immunog
44.368.456	0.4570653		SLC52A2	solute carrier family 52 member 2
44.360.472	0.6158375		GBAS	glioblastoma amplified sequence
44.358.166	0.3923434		PSMC5	proteasome 26S subunit, ATPase 5
44.356.366		205.298	SPP1	secreted phosphoprotein 1
44.346.418	0.5874258		SRP72	signal recognition particle 72
4.434.472	0.7781929		PRRC2C	proline rich coiled-coil 2C
44.343.655	0.4885579		PRDM4	PR/SET domain 4
44.325.548	-0.2618476		LILRA1	leukocyte immunoglobulin like receptor A1
44.281.881	0.357297			
44.273.294	0.6388466		RPA1	replication protein A1
44.272.305	0.8202164		ARF4	ADP ribosylation factor 4
44.271.869	0.3883354		STAM2	signal transducing adaptor molecule 2
44.250.178	-0.994824		RNASE4	ribonuclease A family member 4
44.231.184	0.6756474		STC1	stanniocalcin 1
44.214.366	-0.5679039		CAT	catalase
44.208.591	0.4962092		MAP2K2	mitogen-activated protein kinase kinase 2
4.420.111	-0.6581739		PTS	6-pyruvoyltetrahydropterin synthase
44.193.398	0.8906577		TMEM14B//	transmembrane protein 14B///NADH:ubiquinor
44.180.089	-0.7015964		MYO1B	myosin IB
44.147.994	0.6001335		PPP2R5E	protein phosphatase 2 regulatory subunit B'eps
44.143.764	0.5829317		DDX50	DEAD-box helicase 50
44.116.581	0.5957108		TRAK2	trafficking kinesin protein 2
44.099.956	0.5837779		TRIM27	tripartite motif containing 27
44.090.894	0.3279373		LIG3	DNA ligase 3
44.040.508	0.5437555		WASF2	WAS protein family member 2
44.026.707	0.4584627		C21orf59	chromosome 21 open reading frame 59
44.010.987	0.3039346		ENOX2	ecto-NOX disulfide-thiol exchanger 2
43.989.447	0.6874588		FKBP1A	FK506 binding protein 1A
43.983.638	-0.5320577		OPHN1	oligophrenin 1
43.981.732	0.4194657		SEC23IP	SEC23 interacting protein
43.954.286	-0.7910159		GLUD1	glutamate dehydrogenase 1
43.953.196	0.5601199		HNRNPR	heterogeneous nuclear ribonucleoprotein R
43.926.355	0.4089863		ATP5L	ATP synthase, H+ transporting, mitochondrial F
43.925.557	-0.2905373		SEC24D	SEC24 homolog D, COPII coat complex compor
43.924.025	0.5048807		CDK5RAP2	CDK5 regulatory subunit associated protein 2
43.916.549	-0.4431703		EIF5A	eukaryotic translation initiation factor 5A
43.892.979	0.547547		ENPP4	ectonucleotide pyrophosphatase/phosphodies
43.870.225	-0.8635462		SORBS1	sorbin and SH3 domain containing 1
43.857.794	0.5479539		RPL13AP5/	ribosomal protein L13a pseudogene 5///ribosor
43.843.772	0.494184		LOC64413:	similar to basic-leucine zipper transcription fac
43.843.752	-0.4328863		ZBTB48	zinc finger and BTB domain containing 48
43.834.425	0.7247719		KIFAP3	kinesin associated protein 3
43.831.029	-0.2721242		TSPAN32	tetraspanin 32

43.825.956	-14.655.745	LEPR	leptin receptor
43.814.903	-0.6744218	RBKS	ribokinase
43.811.464	-0.7972595	LTBP1	latent transforming growth factor beta binding p
43.807.969	0.6120239	PPFIA1	PTPRF interacting protein alpha 1
43.789.627	0.6504457	TMEM168	transmembrane protein 168
43.752.656	0.466189	ZBTB11	zinc finger and BTB domain containing 11
4.374.674	-0.6236598	CFLAR	CASP8 and FADD like apoptosis regulator
43.729.126	0.444206	NELFA	negative elongation factor complex member A
43.717.307	0.650784	SLC30A5	solute carrier family 30 member 5
43.706.009	0.7087313		
43.704.325	0.4737736	UBE2L3	ubiquitin conjugating enzyme E2 L3
43.643.769	0.404632	ATP5L	ATP synthase, H+ transporting, mitochondrial F
43.637.026	-0.5682317	AOC3	amine oxidase, copper containing 3
43.636.033	0.4333262	RNF219	ring finger protein 219
43.607.815	0.5976622	PIK3R3	phosphoinositide-3-kinase regulatory subunit 3
43.597.152	-0.2669269	CYP2C8	cytochrome P450 family 2 subfamily C member
43.587.757	-0.4333797	USF2	upstream transcription factor 2, c-fos interactin
43.577.859	0.6720344	AMD1	adenosylmethionine decarboxylase 1
43.577.675	0.7787982	DEGS1	delta 4-desaturase, sphingolipid 1
43.574.814	0.4797909	EIF1	eukaryotic translation initiation factor 1
43.564.584	-0.3842232	GADD45B	growth arrest and DNA damage inducible beta
43.557.107	0.532189	PRPF4	pre-mRNA processing factor 4
43.555.356	0.45075	POU2F1	POU class 2 homeobox 1
43.547.193	-0.2646386	PDLIM5	PDZ and LIM domain 5
43.534.761	-0.7649339	C14orf105	chromosome 14 open reading frame 105
43.513.962	-0.6080477	FZD4	frizzled class receptor 4
4.351.209	0.5746186	SNRNP40	small nuclear ribonucleoprotein U5 subunit 40
43.490.699	0.5429405	RPL5	ribosomal protein L5
43.479.122	-0.4969573	TARP///TRG	TCR gamma alternate reading frame protein///T
43.475.048	0.4940305	IVNS1ABP	influenza virus NS1A binding protein
43.444.726	0.6562348	MTDH	metadherin
43.440.663	-0.8696013	PDLIM5	PDZ and LIM domain 5
43.436.873	0.4535375	ZNF764	zinc finger protein 764
43.432.384	0.4432989	CEP72	centrosomal protein 72
4.343.133	0.7936049	APOBEC3B	apolipoprotein B mRNA editing enzyme catalyti
43.429.243	-0.5157525	SFRP1	secreted frizzled related protein 1
43.407.982	-0.6077149	IL13RA1	interleukin 13 receptor subunit alpha 1
43.405.415	0.5037199	CEP68	centrosomal protein 68
43.399.646	0.8434523	IKBKAP	inhibitor of kappa light polypeptide gene enhan
43.382.661	-0.3855395	NRG1	neuregulin 1
43.372.169	0.5297991	MRPS27	mitochondrial ribosomal protein S27
43.350.625	-0.2478286	NAB2	NGFI-A binding protein 2
43.345.801	-0.8845613	PNPLA3	patatin like phospholipase domain containing 3
43.337.397	-0.5342169	AKAP13	A-kinase anchoring protein 13

43.328.664	0.6537912		TOMM70	translocase of outer mitochondrial membrane [illegible]
43.302.511	0.5538213		VEZF1	vascular endothelial zinc finger 1
43.297.432	-0.5728282		NEK1	NIMA related kinase 1
43.286.783	0.5985336		TAF4	TATA-box binding protein associated factor 4
43.257.415	0.6027214		IMPAD1	inositol monophosphatase domain containing [illegible]
43.254.198	0.713582		PLXNA1	plexin A1
43.246.036	-0.7032645		COPZ2	coatomer protein complex subunit zeta 2
43.206.601	-0.4101889		PSD4	pleckstrin and Sec7 domain containing 4
43.115.856	-0.5138216		COL6A1	collagen type VI alpha 1 chain
43.112.902	0.4419077		RECQL	RecQ like helicase
43.094.919	0.4412043		MED14	mediator complex subunit 14
43.077.597	0.3904867		RPL6	ribosomal protein L6
43.076.784	0.3329153		PLK1	polo like kinase 1
43.076.537	-0.6951859		PNP	purine nucleoside phosphorylase
43.060.798	0.5002662		EIF3A	eukaryotic translation initiation factor 3 subuni[illegible]
43.053.998		10.498.062	TOX3	TOX high mobility group box family member 3
43.047.767	0.7876945		ARHGEF3	Rho guanine nucleotide exchange factor 3
43.045.824	0.5225666		IPO4	importin 4
4.303.367	0.5675997		HMGXB4	HMG-box containing 4
43.019.685	-0.3997163		GATA3	GATA binding protein 3
43.014.481	0.545002		SCAMP1	secretory carrier membrane protein 1
43.008.849	0.5394183		PPIB	peptidylprolyl isomerase B
42.992.889	0.5744633		CAPRIN1	cell cycle associated protein 1
42.973.161	0.4283873		DEF8	differentially expressed in FDCP 8 homolog
42.956.018	-0.4184045		TRD///TRA	T cell receptor delta locus///T-cell receptor alph[illegible]
42.946.609	0.582913		DENND4B	DENN domain containing 4B
429.296		13.765.959	RFK///LYZ	riboflavin kinase///lysozyme
42.928.957	0.5357183		HCCS	holocytochrome c synthase
42.924.689		12.392.316	TM4SF1	transmembrane 4 L six family member 1
42.912.386	0.6270676		EIF3A	eukaryotic translation initiation factor 3 subuni[illegible]
42.891.632		1.054.123	SQSTM1	sequestosome 1
42.891.627	-0.2883428		PDE7B	phosphodiesterase 7B
42.881.146	0.4310419		SEPT7	septin 7
42.880.741	0.4756418		ZNF787	zinc finger protein 787
42.856.582	-0.4753946		SDHAP1	succinate dehydrogenase complex flavoprotei[illegible]
42.819.878	0.3314579		TAF1B	TATA-box binding protein associated factor, RN[illegible]
42.818.908	0.4065899		KAT7	lysine acetyltransferase 7
42.816.796	-0.287157			
42.813.804	0.6085236		AGGF1	angiogenic factor with G-patch and FHA domai[illegible]
42.812.549	0.658474		ROCK2	Rho associated coiled-coil containing protein k[illegible]
428.083	-0.5829296			
42.805.673	0.9331272		DHCR7	7-dehydrocholesterol reductase
42.797.545	-0.4693202		LURAP1L	leucine rich adaptor protein 1 like
42.779.764	-0.7586907		LPIN2	lipin 2

42.774.356	-0.5237304		ZBED1	zinc finger BED-type containing 1
4.275.552	0.4219289		MRM2	mitochondrial rRNA methyltransferase 2
42.726.819	0.3863234		SMCHD1	structural maintenance of chromosomes flexib
42.696.301	0.7839665		STX3	syntaxin 3
42.694.361	-0.3247784		FCMR	Fc fragment of IgM receptor
42.686.508	0.6460417		E2F5	E2F transcription factor 5
42.682.883		-10.428.771	TXNIP	thioredoxin interacting protein
42.681.092	0.4716458		CGREF1	cell growth regulator with EF-hand domain 1
42.678.636	-0.9773644		SLC22A7	solute carrier family 22 member 7
42.637.722	0.654339		UBE2V2	ubiquitin conjugating enzyme E2 V2
42.626.325	0.3473614		NAP1L4	nucleosome assembly protein 1 like 4
42.591.889	-0.4666421			
42.573.048	0.4018957		DEDD	death effector domain containing
42.567.511	-0.4064582		CYP2D6	cytochrome P450 family 2 subfamily D member
42.553.693	0.7157336		ATP9A	ATPase phospholipid transporting 9A (putative)
42.539.764	0.3015546		METTL4	methyltransferase like 4
42.523.565	-0.8401551		EPHA3	EPH receptor A3
42.503.239	0.4593519		MROH7///T	maestro heat like repeat family member 7///tet
42.501.522	-0.4604407		SPON1	spondin 1
42.488.521	-0.4135855		MBTPS1	membrane bound transcription factor peptidas
4.248.649	0.54903		ASUN	asunder, spermatogenesis regulator
42.477.184	-0.4750607		AGPAT2	1-acylglycerol-3-phosphate O-acyltransferase 2
42.460.494	0.5940893		MAPK1IP1L	mitogen-activated protein kinase 1 interacting p
42.448.192	0.9644134		HMGCR	3-hydroxy-3-methylglutaryl-CoA reductase
42.445.711	0.5259883		NGDN	neuroguidin
42.435.237	-0.6447265		PHLDA1	pleckstrin homology like domain family A meml
42.424.509	0.5018182		RPL14P1///	ribosomal protein L14 pseudogene 1///ribosom
42.369.666	0.299059		NPAS2	neuronal PAS domain protein 2
4.236.241	0.5102639		ADNP2	ADNP homeobox 2
42.339.179	-0.3094048		TG	thyroglobulin
42.334.799	0.4697757		EIF2B1	eukaryotic translation initiation factor 2B subur
42.333.044	-0.6739308		NR1H4	nuclear receptor subfamily 1 group H member 4
42.332.703	0.4365464		RPL4	ribosomal protein L4
42.291.616	0.5995904		TMED5	transmembrane p24 trafficking protein 5
42.285.267	-0.3635882		ST3GAL1	ST3 beta-galactoside alpha-2,3-sialyltransferas
42.276.028	-0.7106815		TARP///TRG	TCR gamma alternate reading frame protein///T
42.261.087	0.4117572		EXOC7	exocyst complex component 7
42.251.678	0.4682909		ZNF606	zinc finger protein 606
42.216.666	0.456986		MYDGF	myeloid derived growth factor
42.216.221	-0.269744		EPB42	erythrocyte membrane protein band 4.2
42.209.072	0.4313957		ROCK1	Rho associated coiled-coil containing protein k
42.199.311	0.4193081		KCTD7	potassium channel tetramerization domain cor
42.187.681	-0.6126198		HERPUD1	homocysteine inducible ER protein with ubiquit
42.175.834	-0.4531413		CLEC10A	C-type lectin domain family 10 member A

4.216.039	0.4628404		RAD17	RAD17 checkpoint clamp loader component
42.156.627	0.3879769		TTC1	tetratricopeptide repeat domain 1
42.150.428	0.4337597		PI4KB	phosphatidylinositol 4-kinase beta
42.133.946	0.5839174		ARF4	ADP ribosylation factor 4
42.119.967	0.6369783		DMXL2	Dmx like 2
42.119.272	0.5014162		STRN4	striatin 4
42.110.753	0.3635732		PFDN1	prefoldin subunit 1
42.107.187	0.4258678		HSP90B1	heat shock protein 90 beta family member 1
42.088.249	0.4463882		DLEU2L	deleted in lymphocytic leukemia 2-like
42.072.387	-0.7658882		IL7R	interleukin 7 receptor
42.066.652	0.8495244		TRAF5	TNF receptor associated factor 5
4.205.635	0.5412833		COPS3	COP9 signalosome subunit 3
4.205.154	-0.3086959		CNGB3	cyclic nucleotide gated channel beta 3
42.048.643	0.8924764		CD93	CD93 molecule
42.036.722	0.453367		CEBPZ	CCAAT/enhancer binding protein zeta
42.033.134	-0.372611		CAMTA2	calmodulin binding transcription activator 2
42.014.633	0.4624882		DEPDC1P1	DEP domain containing 1 pseudogene 1///DEP
42.012.334	0.4437136		NKIRAS2	NFKB inhibitor interacting Ras like 2
42.003.136	0.445649		RPL4	ribosomal protein L4
42.000.984	0.5272225		TUBB2B///T	tubulin beta 2B class IIb///tubulin beta 2A class
41.994.356	0.6659483		ASNA1	arsA arsenite transporter, ATP-binding, homolo
41.990.801	0.8017491		TMEM14A	transmembrane protein 14A
41.978.615	0.4649383		RHOQ	ras homolog family member Q
41.973.029	-0.7902418		UGP2	UDP-glucose pyrophosphorylase 2
41.967.422	-0.3354018		PLA2G3	phospholipase A2 group III
41.959.092	-0.5218578		ABCC9	ATP binding cassette subfamily C member 9
41.957.887	0.3436173		YKT6	YKT6 v-SNARE homolog (S. cerevisiae)
41.949.659		13.014.139	VIL1	villin 1
41.932.818		-10.932.915	KCNJ8	potassium voltage-gated channel subfamily J m
41.920.163		-1.687.932	HPGD	hydroxyprostaglandin dehydrogenase 15-(NAD
41.901.104	0.6467253		GLB1	galactosidase beta 1
41.888.939	0.4924249		COX5A	cytochrome c oxidase subunit 5A
41.887.161	-0.7238685		GPHN	gephyrin
41.842.632	0.4371762		PTBP3	polypyrimidine tract binding protein 3
41.840.115	0.6399316		FUBP1	far upstream element binding protein 1
4.181.762	0.5715509		NSDHL	NAD(P) dependent steroid dehydrogenase-like
41.808.608	-0.2491937		EPOR	erythropoietin receptor
41.788.231	0.3544672		SDF2	stromal cell derived factor 2
41.782.879	-0.3388903		IFT74	intraflagellar transport 74
41.750.464	-0.3806539		PLSCR3	phospholipid scramblase 3
41.748.699	0.6302243		CENPN	centromere protein N
41.709.226	-0.8887301		IL1RAP	interleukin 1 receptor accessory protein
41.691.827	0.4679458		TMED9	transmembrane p24 trafficking protein 9
41.647.672	0.3994292		RPS24	ribosomal protein S24

41.644.413	-0.7315818		ICAM3	intercellular adhesion molecule 3
41.628.926	0.4790294		MRPS35	mitochondrial ribosomal protein S35
41.626.585	0.6020238		PCID2	PCI domain containing 2
41.609.562	-0.260433		RARB	retinoic acid receptor beta
41.576.977	0.3933143		CBLL1	Cbl proto-oncogene like 1
41.576.935	0.4588611		PRKRIP1	PRKR interacting protein 1 (IL11 inducible)
41.570.789	0.4237531		NOSIP	nitric oxide synthase interacting protein
41.561.469	0.7580654		MCAM	melanoma cell adhesion molecule
41.552.967	-0.6302043		SLC31A2	solute carrier family 31 member 2
41.541.135	0.4669275		IRGQ	immunity related GTPase Q
4.151.667	0.6344913		C2CD5	C2 calcium dependent domain containing 5
41.516.029	0.3789298		METTL1	methyltransferase like 1
41.504.053	-0.5448227		LOC73044	hypothetical protein LOC730444///FKSG49
41.502.485		-13.560.179	ARG1	arginase 1
41.502.121	0.4752413		TMEM41B	transmembrane protein 41B
41.484.343	-0.686244		RUFY3	RUN and FYVE domain containing 3
41.483.467		-15.217.745	LEPR	leptin receptor
41.472.282	-0.7574992		SDC3	syndecan 3
41.469.401	-0.532638		MCCC2	methylcrotonoyl-CoA carboxylase 2
41.456.891	0.3516985		MYL6	myosin light chain 6
41.437.168	0.3583675		KIF20B	kinesin family member 20B
41.433.893	0.6510945		ASF1A	anti-silencing function 1A histone chaperone
41.420.985	0.4365648		ACD	adrenocortical dysplasia homolog
41.408.635	-0.4955622		EPOR	erythropoietin receptor
41.405.957	-0.3401727		PLA2G15	phospholipase A2 group XV
41.404.339	-0.5687753		PTN	pleiotrophin
41.396.932	0.5479973		TRMT1	tRNA methyltransferase 1
41.395.801	0.7214557		RNF6	ring finger protein 6
41.367.267	0.5267752		RCOR3	REST corepressor 3
41.353.567	0.5032069		LSM5	LSM5 homolog, U6 small nuclear RNA and mRN
41.343.477	0.5995196		ENSA	endosulfine alpha
41.337.719	0.5382988		SSR3	signal sequence receptor subunit 3
41.337.212		11.101.246	TFPI	tissue factor pathway inhibitor
41.336.854	0.6349235		SPCS2P4//	signal peptidase complex subunit 2 homolog (S
41.334.971	0.9124315		SOX4	SRY-box 4
41.321.251	-0.4322723		GPSM3	G-protein signaling modulator 3
41.319.141	0.8553765		TNFSF4	tumor necrosis factor superfamily member 4
41.312.796	0.5152907		ADAM17	ADAM metallopeptidase domain 17
41.303.443		-10.313.463	APOC3	apolipoprotein C3
41.282.901	0.4676636		WDR74	WD repeat domain 74
41.251.305	-0.5606774		PEBP1	phosphatidylethanolamine binding protein 1
41.237.039	0.6482871		BAG3	BCL2 associated athanogene 3
41.210.073	-0.3331753		NFATC3	nuclear factor of activated T-cells 3
41.187.444	0.6587815		BID	BH3 interacting domain death agonist

41.182.164	0.3416148		HTRA2	HtrA serine peptidase 2
41.176	0.8635091		ARID3A	AT-rich interaction domain 3A
41.175.217	-0.4792086		TMEM57///,	transmembrane protein 57///adducin 3
41.174.931	0.4099599		PCNP	PEST proteolytic signal containing nuclear prote
41.164.724	-0.6871406		AKAP9	A-kinase anchoring protein 9
41.140.771	0.4162535		ATP5H	ATP synthase, H+ transporting, mitochondrial F
411.367	-0.6762775		PROSC	proline synthetase cotranscribed homolog (bac
41.128.726	0.5268396		ATP2C1	ATPase secretory pathway Ca2+ transporting 1
41.124.824	0.3107508		SEMA4F	ssemaphorin 4F
41.120.944	-0.386521		DHX58	DEXH-box helicase 58
41.116.961	0.5286758		U2AF2	U2 small nuclear RNA auxiliary factor 2
41.111.848	0.6544241		SLC39A6	solute carrier family 39 member 6
41.089.555	-0.4985966			
41.087.851	0.5151963		MRPL33	mitochondrial ribosomal protein L33
41.078.311	0.4899959		UBA3	ubiquitin like modifier activating enzyme 3
41.053.624		-15.521.266	LEPR	leptin receptor
41.051.237	0.496398		NCK1	NCK adaptor protein 1
41.049.569	0.2879927		GINS3	GINS complex subunit 3
41.039.209	0.3626597		DCAF7	DDB1 and CUL4 associated factor 7
41.027.232	0.5542275		LMNA	lamin A/C
41.025.755	0.3469992		SMARCAL1	SWI/SNF related, matrix associated, actin depe
41.024.652	-0.3862777		USF2	upstream transcription factor 2, c-fos interactir
41.014.755		-10.145.465	AUTS2	autism susceptibility candidate 2
40.995.111	-0.7529496		PCBD1	pterin-4 alpha-carbinolamine dehydratase 1
40.988.664	0.3531448		VPS33B	VPS33B, late endosome and lysosome associat
40.963.393	-0.7197864		ZBTB20	zinc finger and BTB domain containing 20
40.960.052	0.8001097		PON2	paraoxonase 2
409.453	0.6584786		HTATIP2	HIV-1 Tat interactive protein 2
40.932.302	0.6179789		SCFD1	sec1 family domain containing 1
40.924.883	-0.26808		SIGLEC7	sialic acid binding Ig like lectin 7
40.893.091		-10.614.051	COL6A2	collagen type VI alpha 2 chain
40.870.128	0.3870944		RAB28	RAB28, member RAS oncogene family
40.809.751	-0.4312626		MRC2	mannose receptor C type 2
40.776.726	-0.5097229		SEMA5A	semaphorin 5A
40.744.534	0.4238197		PSMD13	proteasome 26S subunit, non-ATPase 13
40.735.351	-0.4976904		SOD1	superoxide dismutase 1, soluble
40.734.017	-0.3197242		SYTL2	synaptotagmin like 2
40.718.581	-0.7106268		RBP4	retinol binding protein 4
40.695.677		-11.699.601	HMGCS2	3-hydroxy-3-methylglutaryl-CoA synthase 2
40.693.589	-0.7279926		DNMT3L	DNA methyltransferase 3 like
4.066.992	0.4256412		RFC5	replication factor C subunit 5
40.658.785	-0.6488898		CSF1R	colony stimulating factor 1 receptor
40.643.133	-0.6514152		DLC1	DLC1 Rho GTPase activating protein
40.638.451	0.4557546		GCN1	GCN1, eIF2 alpha kinase activator homolog

40.623.961	0.2928621		NRF1	nuclear respiratory factor 1
40.618.102	0.7505264		PRRC2C	proline rich coiled-coil 2C
40.617.366	-0.3931439		PLK3	polo like kinase 3
40.612.928		-11.045.679	CYP3A5	cytochrome P450 family 3 subfamily A member
40.602.938	0.4423711		SMCHD1	structural maintenance of chromosomes flexib
40.600.247	0.4260101		USP46	ubiquitin specific peptidase 46
40.570.308	-0.7721566		KCNJ16	potassium voltage-gated channel subfamily J m
40.553.349	0.3949559		SPA17	sperm autoantigenic protein 17
4.053.446	0.4659114		RPL35	ribosomal protein L35
40.527.266	0.2781028		HEATR6	HEAT repeat containing 6
40.527.064	0.5806681		FPGT	fucose-1-phosphate guanylyltransferase
40.522.095		12.992.767	PSPH	phosphoserine phosphatase
40.521.702	0.5780985		H1F0	H1 histone family member 0
40.521.446	-0.6662689		IL4R	interleukin 4 receptor
40.498.317	0.4062941		SART3	squamous cell carcinoma antigen recognized b
40.497.045	-0.4070634		SARDH	sarcosine dehydrogenase
40.489.784	0.5339012		GALNT1	polypeptide N-acetylgalactosaminyltransferase
40.484.862	0.5310854		BRCA1	BRCA1, DNA repair associated
40.476.087	-0.3784074		SLC9A3R2	SLC9A3 regulator 2
40.463.653	0.4676155		RPL18	ribosomal protein L18
40.445.663	0.3461924		DDX23	DEAD-box helicase 23
4.041.168		-12.925.788	BAAT	bile acid-CoA:amino acid N-acyltransferase
40.410.167	0.5343365		LMAN2	lectin, mannose binding 2
40.398.843		10.932.282	TM4SF1	transmembrane 4 L six family member 1
40.390.231		-10.161.672	CPN1	carboxypeptidase N subunit 1
40.386.136	0.5757339		IFNGR2	interferon gamma receptor 2 (interferon gamma
40.380.133	0.5503572		CAND1	cullin associated and neddylation dissociated 1
40.375.915	0.4420272		RPL7A	ribosomal protein L7a
40.374.207	0.4384505		PRPF19	pre-mRNA processing factor 19
40.358.908	-0.7768109		GABRP	gamma-aminobutyric acid type A receptor pi su
40.353.368	0.47214		TBCC	tubulin folding cofactor C
40.350.102	0.6052177		ATP6V0E1	ATPase H+ transporting V0 subunit e1
40.349.836	0.5545426		DTNA	dystrobrevin alpha
40.307.381	-0.3686221		ITFG2	integrin alpha FG-GAP repeat containing 2
40.296.201	-0.2871534		PTGIR	prostaglandin I2 (prostacyclin) receptor (IP)
40.295.955	-0.3217164		GRIK5	glutamate ionotropic receptor kainate type sub
40.284.158	0.2974393		NPM1P22	nucleophosmin 1 pseudogene 22
4.023.536	0.4680044		TIAL1	TIA1 cytotoxic granule-associated RNA binding
40.229.489	0.9292502		ABCC6P2	ATP binding cassette subfamily C member 6 ps
40.221.521	0.3590628		KIF16B	kinesin family member 16B
4.021.835	0.5120816		NRBP1	nuclear receptor binding protein 1
40.213.037	0.4878347		RPS2	ribosomal protein S2
40.191.607		-14.589.779	APCS	amyloid P component, serum
4.018.623	-0.3241273		SLC7A8	solute carrier family 7 member 8

40.179.713	-0.8193109		FHL1	four and a half LIM domains 1
4.017.766	0.4463582		TTC33	tetratricopeptide repeat domain 33
40.176.796	-0.7235453		PTPRD	protein tyrosine phosphatase, receptor type D
40.173.572	0.7492284		KDELC1	KDEL motif containing 1
40.163.077	0.3675643		GABPB1	GA binding protein transcription factor beta sub
40.132.168	0.6362923		RAB3GAP2	RAB3 GTPase activating non-catalytic protein s
4.012.982	0.9479674		CYP51A1	cytochrome P450 family 51 subfamily A membe
40.088.591	0.5211263		CSRNP2	cysteine and serine rich nuclear protein 2
40.082.695	0.4773773		EIF1	eukaryotic translation initiation factor 1
40.077.784	-0.5644292		SMAD7	SMAD family member 7
40.064.345	0.3935301		PAPOLG	poly(A) polymerase gamma
40.041.673		-11.444.988	GPLD1	glycosylphosphatidylinositol specific phosphol
40.035.461		-13.924.574	ATF5	activating transcription factor 5
40.016.453	0.5199794		SMCHD1	structural maintenance of chromosomes flexib
40.001.252	0.7994465		TTC37	tetratricopeptide repeat domain 37
39.959.142	0.3927151		RRP7A	ribosomal RNA processing 7 homolog A
39.957.711	-0.5689759		FPR1	formyl peptide receptor 1
39.955.627	0.6312828		DDAH2	dimethylarginine dimethylaminohydrolase 2
39.953.394	0.6279424		ILF3	interleukin enhancer binding factor 3
39.909.861	0.4410349		LARP1	La ribonucleoprotein domain family member 1
39.859.233	0.3705716		UBE2D2	ubiquitin conjugating enzyme E2 D2
39.841.508	0.7058067		TUG1	taurine up-regulated 1 (non-protein coding)
39.840.606	0.4635558		PIP4K2C	phosphatidylinositol-5-phosphate 4-kinase typ
39.829.422	0.4693336		NUDT2	nudix hydrolase 2
39.822.975	0.667229		GAPDH	glyceraldehyde-3-phosphate dehydrogenase
39.817.659	0.354999		DEDD	death effector domain containing
39.815.386	-0.5492457		PAN2	PAN2 poly(A) specific ribonuclease subunit
39.812.702	-0.698821		GAS6	growth arrest specific 6
39.809.273	0.5368024		MAP3K7	mitogen-activated protein kinase kinase kinase
39.805.733	-0.5274608		BACE1	beta-secretase 1
39.792.644	0.4577665		TMX2	thioredoxin related transmembrane protein 2
39.790.044	0.4450769		AREL1	apoptosis resistant E3 ubiquitin protein ligase 1
39.773.982	0.4728955		SNHG17	small nucleolar RNA host gene 17
3.976.413	-0.5356466		AMFR	autocrine motility factor receptor
39.762.103	-0.6612012		SLC2A9	solute carrier family 2 member 9
39.759.676	0.3483593		PDE6D	phosphodiesterase 6D
39.739.709	0.6500248		NSMAF	neutral sphingomyelinase activation associate
39.734.433	0.5577925		ZNF329	zinc finger protein 329
39.688.918	0.5565855		ANAPC5	anaphase promoting complex subunit 5
396.886	-0.559391		TARP///TRG	TCR gamma alternate reading frame protein///T
39.672.219	0.9679806		CA12	carbonic anhydrase 12
39.662.725	0.5867787		MOB1A	MOB kinase activator 1A
39.653.037	0.2438476		RNASEH2B	ribonuclease H2 subunit B
39.638.896	0.3195342		CCHCR1	coiled-coil alpha-helical rod protein 1

39.629.356	-0.3969615		PEX14	peroxisomal biogenesis factor 14
39.585.558	0.4285387			
39.582.601	0.3499097		ZFYVE26	zinc finger FYVE-type containing 26
39.572.968	0.7330609		SYS1-DBNI	SYS1-DBNDD2 readthrough (NMD candidate)//
39.568.074	-0.3682029		ESD	esterase D
39.559.917	-0.3215933		CEACAM1	carcinoembryonic antigen related cell adhesior
39.527.551	0.3590083		RPS4X	ribosomal protein S4, X-linked
39.510.639	0.3667259		MTMR2	myotubularin related protein 2
3.947.402	0.409273		FAM131A	family with sequence similarity 131 member A
39.449.201	0.5370189		TRMT5	tRNA methyltransferase 5
39.419.039	0.5050242		DNTTIP2	deoxynucleotidyltransferase terminal interactir
39.412.741		-10.331.593	KLF9	Kruppel like factor 9
39.411.093	-0.5507125		SLC19A3	solute carrier family 19 member 3
3.941.083	0.3634892		CYHR1	cysteine and histidine rich 1
39.381.548	0.4015746		DNMT3B	DNA methyltransferase 3 beta
39.365.934	0.5000442		TRAF3	TNF receptor associated factor 3
39.357.434	0.8783357		ITGAV	integrin subunit alpha V
39.357.333	-0.4610688		DGCR5	DiGeorge syndrome critical region gene 5 (non-
39.334.251	0.4530948		LDAH	lipid droplet associated hydrolase
39.319.535	0.5265153		ORC5	origin recognition complex subunit 5
39.299.818	-0.7103437		LOC73010	similar to Glycine cleavage system H protein, m
39.296.623		15.511.523	LCN2	lipocalin 2
39.275.053	0.3413432		ATP6V0E1	ATPase H+ transporting V0 subunit e1
39.268.707	0.5728757		ABCC5	ATP binding cassette subfamily C member 5
39.266.406	0.4889202		C10orf2	chromosome 10 open reading frame 2
39.259.388	0.6018984		MRPL22	mitochondrial ribosomal protein L22
39.218.779	0.4904965		PSEN2	presenilin 2
39.198.556	-0.2537228		S100A12	S100 calcium binding protein A12
39.191.142	0.2213712		WDHD1	WD repeat and HMG-box DNA binding protein 1
39.191.036		10.781.339	COL5A2	collagen type V alpha 2 chain
39.184.137	0.4596055		MTMR1	myotubularin related protein 1
39.182.924	0.447261		GCOM1///F	GRINL1A complex locus 1///RNA polymerase II
3.916.096	0.2479938		CENPA	centromere protein A
39.158.987	0.4919572		ERO1A	endoplasmic reticulum oxidoreductase 1 alpha
39.154.361	0.4527145		MZT2B	mitotic spindle organizing protein 2B
39.138.905	-0.4617391		EIF5	eukaryotic translation initiation factor 5
39.097.119	0.5842374		ZNF432	zinc finger protein 432
39.027.664	0.3498182		OLFML2A	olfactomedin like 2A
39.006.447	0.421827		ATP6V1A	ATPase H+ transporting V1 subunit A
38.992.681	0.5910513		NBN	nibrin
38.987.877	-0.3246599			
38.978.985	0.5576508		COPB1	coatomer protein complex subunit beta 1
38.967.797	0.4061455		MAP4	microtubule associated protein 4
38.955.974	-0.9680376		CYP4F11	cytochrome P450 family 4 subfamily F member

3.894.314	0.6483382	SMARCC1	SWI/SNF related, matrix associated, actin depe
38.918.319	0.4769868	FARSA	phenylalanyl-tRNA synthetase alpha subunit
38.911.243	-0.4579927	CTAGE5	CTAGE family member 5, ER export factor
38.884.472	0.4147068	ZNF813	zinc finger protein 813
3.888.073	0.341304	CHAF1A	chromatin assembly factor 1 subunit A
38.860.081	0.4614702	RBBP5	RB binding protein 5, histone lysine methyltrans
38.855.717	0.3614829	SEPT8	septin 8
38.848.093	0.5751284	MED21	mediator complex subunit 21
38.827.398	0.4978569	EIF5B	eukaryotic translation initiation factor 5B
38.818.226	0.4540798	ZBTB24	zinc finger and BTB domain containing 24
38.813.012	-0.7429285	ETS2	ETS proto-oncogene 2, transcription factor
38.799.151	0.4881484	MAPK1IP1L	mitogen-activated protein kinase 1 interacting p
38.763.433	0.5588326	PAIP1	poly(A) binding protein interacting protein 1
38.760.938	-0.5303905	MLF2	myeloid leukemia factor 2
38.748.576	0.3548208	PDS5A	PDS5 cohesin associated factor A
38.739.033	0.3340005	PLK4	polo like kinase 4
38.735.828	0.5621497	DCLRE1C	DNA cross-link repair 1C
38.714.846	0.6601855	CXorf57	chromosome X open reading frame 57
38.712.582	0.5712744	KIZ	kizuna centrosomal protein
38.711.389	0.5607845	ACTR2	ARP2 actin related protein 2 homolog
38.698.769	0.3928618	RPAP1	RNA polymerase II associated protein 1
38.694.371	-0.3017457	KCNJ15	potassium voltage-gated channel subfamily J m
38.692.316	0.6732491	MAGED1	MAGE family member D1
3.868.383	0.3995064	TMEM183A	transmembrane protein 183A
3.867.258	0.7960479	TUBA4A	tubulin alpha 4a
3.866.835	0.3372122	VPS33B	VPS33B, late endosome and lysosome associat
38.661.246	0.9648726	NEDD9	neural precursor cell expressed, developmenta
38.641.215	-0.2686813	LCMT2	leucine carboxyl methyltransferase 2
38.630.683	0.7129087	MORC4	MORC family CW-type zinc finger 4
38.606.801	-0.6026358	CALM1	calmodulin 1
38.577.936	0.5710826	PLXNA2	plexin A2
38.577.108	0.4492924	SART3	squamous cell carcinoma antigen recognized b
38.577.095	-0.5424001	GFOD1	glucose-fructose oxidoreductase domain conta
38.563.327	0.6020814	FUBP1	far upstream element binding protein 1
3.855.598	-0.6317106	FUT6	fucosyltransferase 6
38.534.873	0.46577	BABAM1	BRISC and BRCA1 A complex member 1
38.531.291	0.363238	MCM4	minichromosome maintenance complex comp
38.519.924	0.3546015	MICALL1	MICAL like 1
38.519.088	-0.4153398	PCSK6	proprotein convertase subtilisin/kexin type 6
38.518.285	0.5106567	MAD2L1BP	MAD2L1 binding protein
38.516.998	0.4770373	GNB1	G protein subunit beta 1
38.504.835	0.4668273	RAB15	RAB15, member RAS oncogene family
38.496.171	0.3903508	ATG12	autophagy related 12
38.493.438	-0.2628155	SORBS1	sorbin and SH3 domain containing 1

38.479.211	0.557478		ORC6	origin recognition complex subunit 6
38.478.705	-0.248044		AFF1	AF4/FMR2 family member 1
3.846.929		-14.917.362	LEPR	leptin receptor
38.456.459	0.9194543		FAT1	FAT atypical cadherin 1
38.431.927	0.5337839		ZDHHC6	zinc finger DHHC-type containing 6
38.426.057	0.6003375		HSPH1	heat shock protein family H (Hsp110) member
38.420.185	-0.6282087		C9orf3	chromosome 9 open reading frame 3
38.416.834	0.593796		PAIP1	poly(A) binding protein interacting protein 1
3.840.943	-0.287018		KLRF1	killer cell lectin like receptor F1
38.379.697	0.4066338		AARSD1	alanyl-tRNA synthetase domain containing 1
38.371.139		-14.254.275	PHLDA1	pleckstrin homology like domain family A meml
3.836.019	-0.3564582		IL1B	interleukin 1 beta
38.353.903	0.5869318		ACAP2	ArfGAP with coiled-coil, ankyrin repeat and PH
38.312.688	0.5238361		XPNPEP1	X-prolyl aminopeptidase 1
38.311.805	-0.4822071		SRPRA	SRP receptor alpha subunit
38.287.699	-0.5724151		DBT	dihydrolipoamide branched chain transacylase
38.259.295	0.407293		EFCAB2	EF-hand calcium binding domain 2
38.244.507	0.3932544		GON4L	gon-4 like
38.241.122	-0.9979833		LOC72896	hypothetical protein LOC728965
38.228.913	-0.3210114		WDTC1	WD and tetratricopeptide repeats 1
38.221.739	0.401202		RPS15	ribosomal protein S15
38.214		17.882.202	DKK1	dickkopf WNT signaling pathway inhibitor 1
38.203.538	0.4862003		SNX3	sorting nexin 3
3.820.128	0.4824054		KATNA1	katanin catalytic subunit A1
38.195.539	0.6454512		FAM127A	family with sequence similarity 127 member A
38.179.685	-0.5614581		SULT1B1	sulfotransferase family 1B member 1
38.157.939	0.9168238		PGK1	phosphoglycerate kinase 1
38.151.471	0.3766939		FAM114A2	family with sequence similarity 114 member A2
38.145.649	0.5184002		U2SURP	U2 snRNP associated SURP domain containing
38.141.128	0.343297			
38.140.578	-0.2462963		LILRA4	leukocyte immunoglobulin like receptor A4
38.130.643	0.4715318		EIF1	eukaryotic translation initiation factor 1
38.120.909	0.5272154		NONO	non-POU domain containing, octamer-binding
38.117.595	0.4102002		COX8A	cytochrome c oxidase subunit 8A
3.811.578	0.6288637		C11orf58	chromosome 11 open reading frame 58
38.105.233	0.6359665		TERF1	telomeric repeat binding factor 1
38.083.719	0.3823021		WDR47	WD repeat domain 47
38.077.256		-12.444.711	ABCB4///AI	ATP binding cassette subfamily B member 4///
3.806.122	-0.6058428		LINC-PINT	long intergenic non-protein coding RNA, p53 in
38.056.278	0.5340236		EEF1B2	eukaryotic translation elongation factor 1 beta
38.055.971	-0.9005096		CPB2	carboxypeptidase B2
38.046.123	-0.5622711		HSD17B10	hydroxysteroid 17-beta dehydrogenase 10
3.804.484		11.448.843	SLC6A8	solute carrier family 6 member 8
38.032.163	0.4806164		INPP5F	inositol polyphosphate-5-phosphatase F

38.024.034	0.7869968		TMSB10	thymosin beta 10
37.985.102	0.2965577		ARHGEF11	Rho guanine nucleotide exchange factor 11
37.974.493	0.8709742		TFDP1	transcription factor Dp-1
37.964.256	0.2790188		PTTG3P	pituitary tumor-transforming 3, pseudogene
37.954.255	-0.6277919		PILRB	paired immunoglobin-like type 2 receptor beta
37.951.684	0.5821147		KHSRP	KH-type splicing regulatory protein
37.950.362	0.6983634		GLUD2	glutamate dehydrogenase 2
37.936.919		12.115.836	MMP12	matrix metallopeptidase 12
37.923.584	0.4997226		SP2	Sp2 transcription factor
37.919.624	-0.5384797		MXI1	MAX interactor 1, dimerization protein
37.900.795	0.3090769		VPS11	VPS11, CORVET/HOPS core subunit
37.890.736	-0.722571		CYB5A	cytochrome b5 type A
37.863.515	0.5473948		RPL26L1	ribosomal protein L26 like 1
37.857.427	0.8002967		MAPK13	mitogen-activated protein kinase 13
37.855.642	0.5979992		DDA1	DET1 and DDB1 associated 1
37.844.762	0.4199596		SUPT4H1	SPT4 homolog, DSIF elongation factor subunit
37.823.724	-0.7428568		ZBTB20	zinc finger and BTB domain containing 20
37.808.024	0.3013623		KPNB1	karyopherin subunit beta 1
3.780.546	0.6197958		THEM6	thioesterase superfamily member 6
37.795.655		-12.139.885	CES1	carboxylesterase 1
37.791.333	0.5309653		CAB39	calcium binding protein 39
37.780.203	-0.5066464		NR2F2	nuclear receptor subfamily 2 group F member 2
37.768.483	0.5346319		PALB2	partner and localizer of BRCA2
37.753.418	-0.4820331		PTPRD	protein tyrosine phosphatase, receptor type D
37.738.562	0.4843451		MPHOSPH6	M-phase phosphoprotein 6
37.737.967	0.5315584		MGA	MGA, MAX dimerization protein
37.732.931	-0.3339489		ADGRE1	adhesion G protein-coupled receptor E1
37.725.911	-0.2247348		FABP3P2	fatty acid binding protein 3 pseudogene 2
37.722.647	-0.9334583		COBLL1	cordon-bleu WH2 repeat protein like 1
37.717.832	-0.4434362		SEPP1	selenoprotein P, plasma, 1
37.716.344		-1.215.526	SLC2A2	solute carrier family 2 member 2
37.705.356	-0.7362255			
37.699.415	0.4270107		ARIH2	ariadne RBR E3 ubiquitin protein ligase 2
37.698.689	0.5088829		SUMO3	small ubiquitin-like modifier 3
37.666.469	0.5805837		NECTIN2	nectin cell adhesion molecule 2
37.662.724	0.3741343		ACTR5	ARP5 actin-related protein 5 homolog
37.612.319	-0.4119116		SORBS3	sorbin and SH3 domain containing 3
37.604.208	0.4824018		CLK2	CDC like kinase 2
3.759.385	0.423904		MYBL2	MYB proto-oncogene like 2
37.590.393	-0.2498694		SIGLEC7	sialic acid binding Ig like lectin 7
37.581.947	-0.5741722		TNFRSF10B	TNF receptor superfamily member 10b
37.575.996	0.4057938		IDH3B	isocitrate dehydrogenase 3 (NAD(+)) beta
37.575.804	0.4489163		HIST1H2BG	histone cluster 1, H2bg
37.556.126	-0.6313251		SLC16A10	solute carrier family 16 member 10

37.553.603	0.349221		RPS29	ribosomal protein S29
37.552.354	0.9608617		MARCKS	myristoylated alanine rich protein kinase C sub
37.551.097	0.4677681			
3.755.061		10.566.404	ELOVL2	ELOVL fatty acid elongase 2
37.545.016	-0.229739		SLIT1	slit guidance ligand 1
3.753.387	0.4392443		OPA1	OPA1, mitochondrial dynamin like GTPase
37.521.713		-10.262.927	FGB	fibrinogen beta chain
37.513.671		-10.151.707	KYNU	kynureninase
37.507.858		15.757.201		
37.497.741	0.2658425		YARS2	tyrosyl-tRNA synthetase 2
37.470.733	0.3589884		CYB561D2	cytochrome b561 family member D2
37.453.664	-0.5047263		RCBTB2	RCC1 and BTB domain containing protein 2
37.451.492	-0.2989873		CASP10	caspase 10
37.444.993	-0.6659114		ALDH5A1	aldehyde dehydrogenase 5 family member A1
37.434.349	0.5682362		ZNF692	zinc finger protein 692
37.375.546	-0.5140676		LRP1	LDL receptor related protein 1
37.371.198	0.4356461		COX7A2L	cytochrome c oxidase subunit 7A2 like
37.369.691	0.4208849		UNC50	unc-50 inner nuclear membrane RNA binding p
37.367.955	0.3642746		RCC1L	RCC1 like
37.335.788	0.7522596		ZSCAN31	zinc finger and SCAN domain containing 31
37.303.972	0.4089849		ZNF292	zinc finger protein 292
37.278.401	-0.4408005		USP15	ubiquitin specific peptidase 15
37.271.914	0.3987154		BLVRA	biliverdin reductase A
37.260.937	0.5817134		KPNB1	karyopherin subunit beta 1
37.259.801	0.4418059		FAM53C	family with sequence similarity 53 member C
37.237.293	0.3537811		WDCP	WD repeat and coiled coil containing
37.232.537	-0.5282634		CTAGE5	CTAGE family member 5, ER export factor
3.722.964	0.5106312		PLGRKT	plasminogen receptor with a C-terminal lysine
37.227.878	0.386488			
37.227.449	0.5143159		VAMP4	vesicle associated membrane protein 4
37.226.548	-0.2976171		CASP10	caspase 10
37.218.678	-0.8175639		MAOA	monoamine oxidase A
37.197.945	0.4613757		MMS19	MMS19 homolog, cytosolic iron-sulfur assembl
37.197.058	0.3430875		NAA40	N(alpha)-acetyltransferase 40, NatD catalytic s
37.177.809	-0.8207074		HADH	hydroxyacyl-CoA dehydrogenase
37.168.672	0.4907184		NOP2	NOP2 nucleolar protein
37.166.541	0.519117		FKTN	fukutin
37.164.029	-0.2356179		KCND3	potassium voltage-gated channel subfamily D r
37.159.734	-0.3848382		RAB2A	RAB2A, member RAS oncogene family
37.146.814	-0.4428731		USF2	upstream transcription factor 2, c-fos interactir
37.143.341	0.3432264		GEMIN2	gem nuclear organelle associated protein 2
37.126.611	0.5333769		MEX3D	mex-3 RNA binding family member D
37.111.229	-0.9195765		ETNK2	ethanolamine kinase 2
37.086.765	0.4880472		SLC25A5	solute carrier family 25 member 5

37.081.249	-0.6025352		ARHGAP15	Rho GTPase activating protein 15
37.079.148	0.5714031		LOC73142	similar to SMAD specific E3 ubiquitin protein lig
37.062.591	0.5594476		LRP10	LDL receptor related protein 10
37.060.772	0.3523673		BMPR1A	bone morphogenetic protein receptor type 1A
37.056.518	-0.377926		PARVA	parvin alpha
37.053.775	-0.1900652		PVALB	parvalbumin
37.041.951	0.5587701		STAG2	stromal antigen 2
37.032.034	-0.3342682		TNP2	transition protein 2
37.027.439	0.3887883		VSIG10	V-set and immunoglobulin domain containing 1
37.018.102	0.5326859		ATP2B1	ATPase plasma membrane Ca2+ transporting 1
37.010.988	-0.7770422		PALM2-AKA	PALM2-AKAP2 readthrough///A-kinase anchorir
37.009.899	-0.6958507		NFIC	nuclear factor I C
36.988.028	-0.6375223		DDB2	damage specific DNA binding protein 2
3.697.561	0.8503972		MARCKSL1	MARCKS like 1
36.958.863	0.5002279		SDHC	succinate dehydrogenase complex subunit C
36.953.907		-10.415.965	MGLL	monoglyceride lipase
36.939.437	0.4092856		EXOSC9	exosome component 9
36.934.843	-0.3286712		TSPOAP1	TSPO associated protein 1
36.932.303	0.5900906		TMCO3	transmembrane and coiled-coil domains 3
36.930.801	-0.4593998		FKSG49	FKSG49
36.928.691	0.6017399			
36.904.282	-0.5428226		GSTA3	glutathione S-transferase alpha 3
36.889.065	0.4585553			
36.882.781	-0.6425216		MYO1B	myosin IB
36.876.982		10.465.456	CA12	carbonic anhydrase 12
36.873.778	0.5841246		GNPTAB	N-acetylglucosamine-1-phosphate transferase
36.862.687		11.170.478	MEP1A	meprin A subunit alpha
36.847.666	-0.5518432		TARP///TRG	TCR gamma alternate reading frame protein///T
36.834.589	-0.6242899		SPG20	spastic paraplegia 20 (Troyer syndrome)
36.829.314	0.5924303		ATF1	activating transcription factor 1
36.828.495	0.3061048		ZSCAN12	zinc finger and SCAN domain containing 12
36.828.458	0.2875185		XYLT2	xylosyltransferase 2
36.820.882	-0.7020714		FLRT2	fibronectin leucine rich transmembrane proteir
36.818.738	0.4595205		CAPN7	calpain 7
36.799.897	0.5782378		DPM1	dolichyl-phosphate mannosyltransferase subur
36.786.804	0.4999483		RBM48	RNA binding motif protein 48
36.783.016	0.4509148		NSA2	NSA2, ribosome biogenesis homolog
36.771.104	0.7587589		SPHAR	S-phase response (cyclin related)
36.760.256	-0.3694846		IL16	interleukin 16
36.756.483	0.9129485		HMGCR	3-hydroxy-3-methylglutaryl-CoA reductase
36.744.121	0.4815557		COPG1	coatomer protein complex subunit gamma 1
36.735.292	-0.2587664			
36.717.943	0.5490657		APITD1	apoptosis-inducing, TAF9-like domain 1
36.712.833		15.545.446		

36.709.201	0.4165586	KDM6A	lysine demethylase 6A
36.702.584	0.387363	RPL10	ribosomal protein L10
36.696.358	-0.2906413		
36.691.117	-0.3201617	ZC2HC1C	zinc finger C2HC-type containing 1C
36.686.888	-0.3716703		
36.682.434	0.5088506	GNAS	GNAS complex locus
36.664.036	0.4820696	RNF44	ring finger protein 44
36.635.967	0.3890476	NOC4L	nucleolar complex associated 4 homolog
36.632.311	-0.3762755	ARHGAP25	Rho GTPase activating protein 25
36.629.314	-0.6018288	DECR1	2,4-dienoyl-CoA reductase 1, mitochondrial
36.608.766	0.4064385	RNF216	ring finger protein 216
36.584.039	0.4566452	GDI1	GDP dissociation inhibitor 1
36.562.161	0.3649371	THADA	THADA, armadillo repeat containing
36.558.334	0.4955753	EMC8	ER membrane protein complex subunit 8
36.546.879	0.3342288	PGAP1	post-GPI attachment to proteins 1
36.542.634	0.7041666	RMDN1	regulator of microtubule dynamics 1
36.536.313	0.5306207	PCBP1	poly(rC) binding protein 1
36.503.061	0.449416	MOSPD2	motile sperm domain containing 2
36.475.791	0.4899794	WDR43	WD repeat domain 43
36.472.882	0.375221	PPME1	protein phosphatase methylesterase 1
36.465.698	-0.9713126	AGTR1	angiotensin II receptor type 1
36.458.422	0.4999503	PFAS	phosphoribosylformylglycinamidine synthase
36.457.924	-0.3947	SH3BP2	SH3 domain binding protein 2
36.444.238	-0.2142052	STATH	statherin
36.436.527	0.5659572	SPATS2L	spermatogenesis associated serine rich 2 like
36.433.763	0.4303381	CASP3	caspase 3
36.433.069	0.5729736	PTMA	prothymosin, alpha
36.420.564	0.4487338	HNRNPA1	heterogeneous nuclear ribonucleoprotein A1
36.389.973	0.5856488	AIMP2	aminoacyl tRNA synthetase complex interactin
36.383.476	0.442652	TRIO	trio Rho guanine nucleotide exchange factor
36.374.574	0.4160181	ASXL1	additional sex combs like 1, transcriptional reg
363.724	0.8981906	CA12	carbonic anhydrase 12
36.345.819	0.3384406	RAB5A	RAB5A, member RAS oncogene family
36.343.989	-0.7455755	FGA	fibrinogen alpha chain
36.316.887	-0.2167297	ABCC3	ATP binding cassette subfamily C member 3
36.310.171	0.7132302	TOR3A	torsin family 3 member A
36.305.711	0.5481574	AGA	aspartylglucosaminidase
36.280.702	-0.6154301	DCAF11	DDB1 and CUL4 associated factor 11
36.273.164	0.523578	TCF12	transcription factor 12
36.261.845	0.460016	TSR1	TSR1, ribosome maturation factor
36.261.582	-0.2131722	SNAPC5	small nuclear RNA activating complex polypept
36.257.953	-0.2495738	SELPLG	selectin P ligand
36.255.031	0.4976211	TERF1	telomeric repeat binding factor 1
36.243.187	0.6615508	ERV3-2	endogenous retrovirus group 3 member 2

36.231.897	0.4349106		KIFC1	kinesin family member C1
36.222.969	-0.6814367		NFIC	nuclear factor I C
36.216.514	-0.5585589		LAMC3	laminin subunit gamma 3
36.215.183	0.5631824		SLC35D2	solute carrier family 35 member D2
36.213.612	0.3173775		RBM12	RNA binding motif protein 12
36.203.962	0.5137324		MEMO1	mediator of cell motility 1
36.203.321	-0.5202561		CFLAR	CASP8 and FADD like apoptosis regulator
36.197.633	-0.2566813		IZUMO4	IZUMO family member 4
36.193.454	0.3827118		NAA50	N(alpha)-acetyltransferase 50, NatE catalytic s
36.185.359	0.6340139		THY1	Thy-1 cell surface antigen
36.168.292	0.5906304		SET	SET nuclear proto-oncogene
3.616.699	0.3112151		ENOX2	ecto-NOX disulfide-thiol exchanger 2
36.163.202	0.5496959		RNF144A	ring finger protein 144A
36.160.043	-0.6984936		LDLR	low density lipoprotein receptor
36.156.527	-0.7316496		LOC72922	liprin-beta-1-like///PPFIA binding protein 1
36.150.525	0.643011		ERMP1	endoplasmic reticulum metallopeptidase 1
36.146.694	0.3019259		DNAL4	dynein axonemal light chain 4
36.142.814	0.4521475		SHOC2	SHOC2, leucine rich repeat scaffold protein
36.137.915	-0.3121676		TMEM92	transmembrane protein 92
36.122.499	0.4157609		EXOSC5	exosome component 5
3.611.059	0.4602941		MTPAP	mitochondrial poly(A) polymerase
36.108.431	0.3420824		NDRG3	NDRG family member 3
36.088.141	0.4251369		GADD45GII	GADD45G interacting protein 1
36.071.876		-15.584.553	GSTA1	glutathione S-transferase alpha 1
36.037.314	-0.6401637		DIP2C	disco interacting protein 2 homolog C
36.035.883	0.3332399		KPTN	kaptin, actin binding protein
36.024.648	-0.5368141		LAMA2	laminin subunit alpha 2
36.015.396	0.426386		VPS8	VPS8, CORVET complex subunit
36.011.504	0.7463631		SERPINH1	serpin family H member 1
36.009.583	-0.4575747		SELP	selectin P
35.998.783	0.3633065		PIGH	phosphatidylinositol glycan anchor biosynthesi
35.997.596	-0.2700939		ADD2	adducin 2
35.977.104	-0.2328635		POM121L9	POM121 transmembrane nucleoporin like 9, ps
35.971.061	-0.6887027		A1CF	APOBEC1 complementation factor
35.969.309	0.4917382		KIAA1462	KIAA1462
35.954.544	0.4507641		HPF1	histone PARylation factor 1
35.951.142	-0.3668402		PTPN13	protein tyrosine phosphatase, non-receptor typ
35.940.657	0.4021567		UBE2J1	ubiquitin conjugating enzyme E2 J1
35.938.325	-0.5043691		CDK9	cyclin dependent kinase 9
35.937.172	0.4104155		DIMT1	DIM1 dimethyladenosine transferase 1 homolo
35.916.797	0.3966776		CMTR1	cap methyltransferase 1
35.914.676	-0.2598866		ATP8A2	ATPase phospholipid transporting 8A2
35.902.553	0.4270158		FAM149B1	family with sequence similarity 149 member B1
35.895.823	0.6616484		TIMM17A	translocase of inner mitochondrial membrane [illegible]

35.892.449	0.5766966		LAMTOR5	late endosomal/lysosomal adaptor, MAPK and I
35.878.324	0.4103831		RAB11FIP2	RAB11 family interacting protein 2
35.868.134	0.3461219		PDCL	phosducin like
35.866.343	-0.3471715		KRAS	KRAS proto-oncogene, GTPase
35.857.175	-0.4152893		PPFIBP1	PPFIA binding protein 1
35.849.366	-0.4071828		PNPLA2	patatin like phospholipase domain containing 2
35.838.623		10.241.985	MUC13	mucin 13, cell surface associated
35.836.096	0.52362		FXR1	FMR1 autosomal homolog 1
35.815.998	0.5506272		HGH1	HGH1 homolog
3.579.831	0.3944067		NDUFAF3	NADH:ubiquinone oxidoreductase complex ass
35.797.494	0.5308883		NOL8	nucleolar protein 8
35.790.984	0.8303762		FADS2	fatty acid desaturase 2
35.765.048	0.6208814		PRMT1	protein arginine methyltransferase 1
35.729.881	0.3494233		NFYA	nuclear transcription factor Y subunit alpha
35.718.816	0.3763976		CHD4	chromodomain helicase DNA binding protein 4
35.706.219	-0.8539014		CDH1	cadherin 1
35.702.974	0.5039545		PLAA	phospholipase A2 activating protein
35.696.586	0.5433099		ETF1	eukaryotic translation termination factor 1
35.696	0.4686647		HUWE1	HECT, UBA and WWE domain containing 1, E3 u
35.694.859	0.5410243		NOP16	NOP16 nucleolar protein
35.682.926	0.3304558		GLRX3P2	glutaredoxin 3 pseudogene 2
35.665.451	0.3226314		RPL5	ribosomal protein L5
35.659.142	0.5890665		CDC42SE1	CDC42 small effector 1
35.639.946	0.6035704		ITGA5	integrin subunit alpha 5
35.611.743		-10.876.026	AMACR	alpha-methylacyl-CoA racemase
35.607.354	0.4227584		TCP1	t-complex 1
35.594.223	0.5232921		DNAJB6	DnaJ heat shock protein family (Hsp40) membe
35.591.172	0.4454012		COA1	cytochrome c oxidase assembly factor 1 homol
35.586.178	-0.4530713		TRAPPC10	trafficking protein particle complex 10
35.580.423	0.4287727		MZT2B	mitotic spindle organizing protein 2B
35.575.154	-0.4659043		DHRS12	dehydrogenase/reductase 12
35.569.309	-0.3272839		LILRB1	leukocyte immunoglobulin like receptor B1
35.551.513	0.6556892		SRSF3	serine and arginine rich splicing factor 3
35.547.982	0.4462058		PXMP4	peroxisomal membrane protein 4
35.540.787	-0.5221666		IL2RB	interleukin 2 receptor subunit beta
35.526.775	0.5100554		PARVB	parvin beta
35.521.979	0.3693325		TMED7	transmembrane p24 trafficking protein 7
35.516.755	0.5120522		AIMP2	aminoacyl tRNA synthetase complex interactin
35.511.883	-0.7211395		SMARCA2	SWI/SNF related, matrix associated, actin depe
35.510.786		10.685.457	MAGED4B	MAGE family member D4B
35.507.609	-0.3111235		CDADC1	cytidine and dCMP deaminase domain containi
35.502.557	0.4887471		RPL35A	ribosomal protein L35a
35.501.842	-0.4510441		RREB1	ras responsive element binding protein 1
35.491.332	-0.79314		FERMT2	fermitin family member 2

3.548.302	-0.2304572		SLC22A2	solute carrier family 22 member 2
3.547.672		-13.798.418	AKR1C4	aldo-keto reductase family 1, member C4
35.470.231	0.5903552		BCR	BCR, RhoGEF and GTPase activating protein
35.469.898	0.6686075		NR5A2	nuclear receptor subfamily 5 group A member 2
35.468.564	0.5955127		LYRM2	LYR motif containing 2
3.546.278	0.4745905		RFK	riboflavin kinase
35.462.039	-0.5088196		MYO18A///	myosin XVIIIA///TGFB1-induced anti-apoptotic f
35.458.141	0.4430029		GPATCH8	G-patch domain containing 8
35.451.777	-0.7668525		DIP2C	disco interacting protein 2 homolog C
35.438.959	-0.5261673		GOLGA4	golgin A4
35.434.201	0.641633		THY1	Thy-1 cell surface antigen
35.427.863	-0.5763008		AKAP13	A-kinase anchoring protein 13
35.422.994	0.5944131		DRAM1	DNA damage regulated autophagy modulator 1
3.540.436	0.4594486		LSG1	large 60S subunit nuclear export GTPase 1
35.390.294	0.3888596		ATAD2B	ATPase family, AAA domain containing 2B
35.378.361	0.7669218		TPM4	tropomyosin 4
3.536.301	0.4914663		ZNF267	zinc finger protein 267
35.345.688	-0.4534763		PAPSS2	3'-phosphoadenosine 5'-phosphosulfate synth
35.345.491	0.7851498		THBS4	thrombospondin 4
35.327.399		-10.881.498	NR4A2	nuclear receptor subfamily 4 group A member 2
35.315.155	-0.5683797		ELN	elastin
35.311.941	0.3643329		ZSCAN9	zinc finger and SCAN domain containing 9
35.307.046	-0.3260607		CD33	CD33 molecule
3.530.501	0.4572513		ABT1	activator of basal transcription 1
35.282.405	0.3816836		CAMSAP1	calmodulin regulated spectrin associated prote
35.274.142	-0.2687261		GRIN1	glutamate ionotropic receptor NMDA type subu
35.271.925	0.311389		EIF5B	eukaryotic translation initiation factor 5B
35.263.414	0.4711815		ANP32B	acidic nuclear phosphoprotein 32 family memb
35.260.665	0.3823252		LOC15077	sphingomyelin phosphodiesterase 4, neutral m
3.524.997	-0.50076		ATXN7	ataxin 7
35.248.931	-0.3068518		HGF	hepatocyte growth factor
35.244.806	0.9518375		PALLD	palladin, cytoskeletal associated protein
35.227.773	0.4115991		TOX4	TOX high mobility group box family member 4
35.225.004	0.7835983		TRIB3	tribbles pseudokinase 3
35.210.684	-0.5867325		IGHM	immunoglobulin heavy constant mu
35.206.289	0.4496281		HPS4	HPS4, biogenesis of lysosomal organelles comp
35.203.606	-0.7049204		DSE	dermatan sulfate epimerase
35.188.198	0.3166001		HS2ST1	heparan sulfate 2-O-sulfotransferase 1
35.175.292	-0.5033781		SLA	Src-like-adaptor
35.173.723		-13.239.669	PPP1R1A	protein phosphatase 1 regulatory inhibitor subu
35.167.977	-0.3019858		LAMA4	laminin subunit alpha 4
35.163.428	0.8187757		SSUH2	ssu-2 homolog (C. elegans)
3.515.515	0.6903211		STC1	stanniocalcin 1
35.145.534		-15.311.537	ATF5	activating transcription factor 5

35.141.713	-0.4745405		GABBR1	gamma-aminobutyric acid type B receptor subu
35.139.109	0.4301893		PDCD11	programmed cell death 11
35.137.725	-0.2560316		HAND2	heart and neural crest derivatives expressed 2
35.134.162	-0.7019969		DLC1	DLC1 Rho GTPase activating protein
351.331	0.4636695		HIST1H3H	histone cluster 1, H3h
35.129.872	-0.5675177		AKAP9	A-kinase anchoring protein 9
35.106.912	0.7099671		IFI35	interferon induced protein 35
35.096.867	-0.2373895		NGF	nerve growth factor
35.092.535	-0.3998977		RNF24	ring finger protein 24
35.085.149		11.520.446		
35.066.937		-14.907.907	HAL	histidine ammonia-lyase
35.052.805	0.3788722		PYCRL	pyrroline-5-carboxylate reductase-like
35.036.022	-0.8316017		PLIN2	perilipin 2
35.030.882	0.5663083		JMJD6	arginine demethylase and lysine hydroxylase
35.026.798	-0.3786427		WNT10B	Wnt family member 10B
35.015.168	0.5112798		RPL10A	ribosomal protein L10a
35.010.406	0.3846537		PRDM10	PR/SET domain 10
35.006.371	0.3874078		CYTH2	cytohesin 2
35.005.954	0.4563162		SNX3	sorting nexin 3
35.002.401	0.7741649		LRRC31	leucine rich repeat containing 31
35.002.073	0.5897365		RHOC	ras homolog family member C
34.940.132	-0.6442652		NAXD	NAD(P)HX dehydratase
34.916.569	0.4313575		NIPBL	NIPBL, cohesin loading factor
34.908.175	-0.3025671		SIDT2	SID1 transmembrane family member 2
34.905.093	-0.323049			
3.487.393	-0.675943		ACYP2	acylphosphatase 2
3.487.182	-0.2765814		SHMT1	serine hydroxymethyltransferase 1
34.864.441	0.5517056		PDIA3	protein disulfide isomerase family A member 3
34.857.557	-0.5137742		HLX	H2.0 like homeobox
34.854.007	0.4941141		CSNK1A1	casein kinase 1 alpha 1
3.484.696	0.4166477		MED7	mediator complex subunit 7
34.807.969	-0.3024605		PSG4	pregnancy specific beta-1-glycoprotein 4
34.806.299	-0.1931496		ASAH1	N-acylsphingosine amidohydrolase 1
34.780.618	0.5357324		EXOSC8	exosome component 8
34.775.328	-0.5035145		KLC1	kinesin light chain 1
34.764.619	0.8006912		SFN	stratifin
34.761.383	0.6143245		LAMA4	laminin subunit alpha 4
34.759.828	-0.2420342		MAPK8IP1	mitogen-activated protein kinase 8 interacting p
34.732.343	0.5537776		FKBP1A	FK506 binding protein 1A
3.472.567	0.601358		ABHD3	abhydrolase domain containing 3
34.717.204	-0.6689152		TMEM100	transmembrane protein 100
34.708.945	0.5952624		MYBL1	MYB proto-oncogene like 1
34.702.487	0.5881855		SRSF9	serine and arginine rich splicing factor 9
34.660.079	-0.7915971		ENPP1	ectonucleotide pyrophosphatase/phosphodies

34.655.201	-0.2977734		CTSW	cathepsin W
34.636.707	-0.3249795		MYL10	myosin light chain 10
34.629.226	-0.9144282		HLF	HLF, PAR bZIP transcription factor
3.460.777	0.407957		ACYP1	acylphosphatase 1
34.599.888	-0.2706875		PLEKHA2	pleckstrin homology domain containing A2
34.599.054	0.4626255		MTF2	metal response element binding transcription f
34.592.418	0.3104826		DGCR14	DiGeorge syndrome critical region gene 14
34.591.924	0.2258385		TAOK3	TAO kinase 3
34.569.084	-0.3139476		SUPT20H//.	SPT20 homolog, SAGA complex component///d
34.550.995	-0.263613		RNFT1	ring finger protein, transmembrane 1
34.541.689	-0.7037641		LDB2	LIM domain binding 2
34.540.707	0.4914827		HLA-F-AS1	HLA-F antisense RNA 1
34.540.326	0.3588086		SRGAP2	SLIT-ROBO Rho GTPase activating protein 2
34.527.537	0.4544294		HSPA8	heat shock protein family A (Hsp70) member 8
3.452.377	0.517514		C19orf53	chromosome 19 open reading frame 53
34.522.135	0.6449111		GPX7	glutathione peroxidase 7
34.521.936	-0.8697197		ST3GAL1	ST3 beta-galactoside alpha-2,3-sialyltransferas
34.505.698	-0.2634071		ZC3H7B	zinc finger CCCH-type containing 7B
34.481.512	-0.2798993			
34.469.889	-0.8176351		CNGA1	cyclic nucleotide gated channel alpha 1
34.455.879	-0.8112382		ORM1	orosomucoid 1
34.438.811	0.5491761		CAPNS1	calpain small subunit 1
34.437.858	0.4076328		DALRD3	DALR anticodon binding domain containing 3
34.423.267	0.3564603		HSPBP1	HSPA (Hsp70) binding protein 1
34.419.009	0.3827611		MKRN2	makorin ring finger protein 2
34.406.532	0.4404484		C12orf10	chromosome 12 open reading frame 10
3.439.383	-0.3130702		LOC55338	uncharacterized LOC55338
34.380.888	-0.5039361		CRY2	cryptochrome circadian clock 2
34.379.976	-0.4566485		AFF1	AF4/FMR2 family member 1
34.374.574	0.5248061		PPP1R14B	protein phosphatase 1 regulatory inhibitor subu
34.373.658	0.1902797		NRF1	nuclear respiratory factor 1
34.365.669	-0.5370713		CHAD	chondroadherin
34.335.821	0.2936169		PRDM4	PR/SET domain 4
34.329.311	0.434139		LINC00869	long intergenic non-protein coding RNA 869
34.315.006	-0.226513		TAL1	TAL bHLH transcription factor 1, erythroid differ
34.298.381	0.3001734		RPEL1///RF	ribulose-5-phosphate-3-epimerase like 1///ribu
34.297.234	-0.4540548		SMIM14	small integral membrane protein 14
34.290.386	0.3745462		LSM5	LSM5 homolog, U6 small nuclear RNA and mRN
3.428.667	0.7236085		DNPH1	2'-deoxynucleoside 5'-phosphate N-hydrolase 1
34.278.079		-11.104.085	SIK1	salt inducible kinase 1
34.263.028	-0.2446985		KCNE2	potassium voltage-gated channel subfamily E r
34.249.472	0.4625165		DNAJC8	DnaJ heat shock protein family (Hsp40) membe
34.229.193	0.4830813		TUG1	taurine up-regulated 1 (non-protein coding)
34.223.626	0.4047404		HHAT	hedgehog acyltransferase

34.188.375	0.5442246	MECOM	MDS1 and EVI1 complex locus
3.418.592	0.5380487	GPN3	GPN-loop GTPase 3
34.179.481	-0.3274406	LILRB1	leukocyte immunoglobulin like receptor B1
34.178.106	-0.3857889	RSL1D1	ribosomal L1 domain containing 1
34.168.536	0.4662122	LOC72931(	POM121 membrane glycoprotein pseudogene/
34.158.612	-0.3778052	ROCK1	Rho associated coiled-coil containing protein k
34.158.599	-0.6813778	ANXA8L1//	annexin A8-like 1///annexin A8
3.415.459	0.3966013	MKRN1	makorin ring finger protein 1
34.154.314	0.7175921	PELI1	pellino E3 ubiquitin protein ligase 1
34.113.379	-0.2353889	ZSCAN32	zinc finger and SCAN domain containing 32
34.109.191	0.3625863	FAM175B	family with sequence similarity 175 member B
34.097.709	-0.4999312	CNDP2	CNDP dipeptidase 2 (metallopeptidase M20 fa
34.087.214	-0.1978498	ERC2-IT1	ERC2 intronic transcript 1
34.057.602	0.6128716	SYNCRIP	synaptotagmin binding cytoplasmic RNA intera
34.053.005	0.3503684	RBM34	RNA binding motif protein 34
34.042.059	-0.5483224	PCSK6	proprotein convertase subtilisin/kexin type 6
34.019.354	-0.3397544	ELK1	ELK1, ETS transcription factor
33.997.773	0.5434194	CTTN	cortactin
33.987.716	0.6500804	POLR1D	RNA polymerase I subunit D
33.982.677	-0.2998945	NPR1	natriuretic peptide receptor 1
33.978.038	0.4306876	CNOT4	CCR4-NOT transcription complex subunit 4
33.976.369	-0.2925385	NF2	neurofibromin 2
33.971.337	-0.5359386	TMEM204	transmembrane protein 204
33.968.166	0.3546355	ATF2	activating transcription factor 2
3.395.557	0.4730478	CDK2AP1	cyclin dependent kinase 2 associated protein 1
33.951.886	0.4588125	GTF2H1	general transcription factor IIH subunit 1
33.948.104	0.3148185	ACTB	actin beta
33.943.289	0.3854804	TFCP2	transcription factor CP2
33.918.034	0.2630083	AVL9	AVL9 cell migration associated
33.916.248	0.5666559	PTMA	prothymosin, alpha
33.885.155	-0.5881768	ZNF395	zinc finger protein 395
33.884.084	-0.388884	PTPRB	protein tyrosine phosphatase, receptor type B
33.871.739	0.3871219	PQBP1	polyglutamine binding protein 1
33.871.619	0.5143985	G3BP1	G3BP stress granule assembly factor 1
33.865.618	0.5208388	LRRC20	leucine rich repeat containing 20
33.860.459	0.663039	EFNB2	ephrin B2
33.854.022	0.6899539	CYP51A1	cytochrome P450 family 51 subfamily A membe
33.852.747	0.5562635	PDXK	pyridoxal (pyridoxine, vitamin B6) kinase
3.384.657	0.4368386	FAM208A	family with sequence similarity 208 member A
3.383.401	0.4489871	CCDC59	coiled-coil domain containing 59
33.830.101	0.5586828	BID	BH3 interacting domain death agonist
33.825.584	0.5156566	CDV3	CDV3 homolog
33.824.259	-0.7518985	PER3	period circadian clock 3
33.802.912	0.7433522	TPM1	tropomyosin 1 (alpha)

33.792.756	0.5758824	RPA1	replication protein A1
33.771.184	0.4775637	RACK1	receptor for activated C kinase 1
33.763.381	0.4137402	CUL4B	cullin 4B
33.741.302	0.4913982	GATAD1	GATA zinc finger domain containing 1
33.731.343	0.4042195	NOMO2	NODAL modulator 2
33.727.647	-0.4228338	LUC7L	LUC7 like
33.716.639	0.4183557	FASTK	Fas activated serine/threonine kinase
33.714.097	0.3045152	KLC2	kinesin light chain 2
33.709.246	-0.3189609	INO80D	INO80 complex subunit D
33.699.095	-0.5011699	WDR59	WD repeat domain 59
33.684.956	0.3615656	HTT	huntingtin
33.680.413	0.7971806	PDZK1	PDZ domain containing 1
33.679.409	-0.2370757	IST1	IST1, ESCRT-III associated factor
33.674.171	0.5715574	TMEM33	transmembrane protein 33
33.655.116	0.5902536	ACTG1	actin gamma 1
33.654.179	0.4865533	MDH2	malate dehydrogenase 2
33.653.321	0.3814878	OGG1	8-oxoguanine DNA glycosylase
33.625.917	-0.6900296	CYB5A	cytochrome b5 type A
33.624.955	-0.3052417	WNT2	Wnt family member 2
33.624.741	-0.4517958	NTHL1	nth like DNA glycosylase 1
33.622.046	-0.2205736	WSCD2///P	WSC domain containing 2///protein kinase cAM
33.614.697	0.4356999	EPS15L1	epidermal growth factor receptor pathway subs
33.610.008	0.4139209	SNRNP27	small nuclear ribonucleoprotein U4/U6.U5 sub
33.608.875	0.5170675	CERS2	ceramide synthase 2
33.602.121	0.2541441	POLR2D	RNA polymerase II subunit D
33.588.896	-0.1981919	AKAP13	A-kinase anchoring protein 13
33.583.049	0.4785236	STK3	serine/threonine kinase 3
33.572.199	0.469398	SNTB1	syntrophin beta 1
33.569.472	0.5971731	TDP2	tyrosyl-DNA phosphodiesterase 2
33.569.279	-0.472736	SEC62	SEC62 homolog, preprotein translocation facto
33.530.326	0.3236415	DBR1	debranching RNA lariats 1
33.525.592	0.6946811	GAL3ST1	galactose-3-O-sulfotransferase 1
33.509.017	0.630556	SEPT6	septin 6
33.487.771	0.5819823	GPD1L	glycerol-3-phosphate dehydrogenase 1-like
33.481.075	-0.3194416	AHDC1	AT-hook DNA binding motif containing 1
3.347.715	0.3125747	ACACA	acetyl-CoA carboxylase alpha
3.345.005	0.3715979	RIPK2	receptor interacting serine/threonine kinase 2
33.408.628	-0.3063441	LINC01482	long intergenic non-protein coding RNA 1482
3.340.588	0.7068694	LEF1	lymphoid enhancer binding factor 1
3.340.201	-0.3151405	PARP3	poly(ADP-ribose) polymerase family member 3
33.375.005	-0.2166128	INPP5B	inositol polyphosphate-5-phosphatase B
33.370.691	0.7280965	EFNA1	ephrin A1
3.336.861	0.4665227	RAB15	RAB15, member RAS oncogene family
33.350.764	0.5391333	LOC64328	similar to prothymosin alpha

33.345.862	0.7108814		CAPN2	calpain 2
33.344.092	0.4471501		ELAVL1	ELAV like RNA binding protein 1
33.328.988	0.3916706		PCGF1	polycomb group ring finger 1
33.324.174	-0.7138344		ENPEP	glutamyl aminopeptidase
33.319.568	0.3410381		NAA35	N(alpha)-acetyltransferase 35, NatC auxiliary s
33.315.851	-0.4664042		SCAF4	SR-related CTD associated factor 4
33.313.306	0.4397152		COPA	coatomer protein complex subunit alpha
3.329.567	0.3814747		SNX11	sorting nexin 11
33.294.946	0.3795031		FBXL18	F-box and leucine rich repeat protein 18
33.288.146	0.4802988		EIF5B	eukaryotic translation initiation factor 5B
33.274.371	0.5890605		NBN	nibrin
33.270.666	-0.5958256		MSRB1	methionine sulfoxide reductase B1
33.264.797	0.5109688		CDC5L	cell division cycle 5 like
33.260.119	0.4446491		ACP1	acid phosphatase 1, soluble
33.254.852	-0.4263547		ZNF358	zinc finger protein 358
33.254.702	-0.3025643		LAT2	linker for activation of T-cells family member 2
33.247.806	-0.4035871		VAMP2	vesicle associated membrane protein 2
33.246.613	-0.7514618		FAS	Fas cell surface death receptor
33.241.325	-0.5812399		FHL1	four and a half LIM domains 1
33.233.768	-0.8706168		CP	ceruloplasmin (ferroxidase)
3.322.061	0.2572903		TMA16	translation machinery associated 16 homolog
33.196.701	-0.4044414		FHL1	four and a half LIM domains 1
33.188.102	-0.3119551		ERN2	endoplasmic reticulum to nucleus signaling 2
33.182.055	0.3887798		CPNE9///Pl	copine family member 9///phospholipase C gar
33.180.817	0.2986212		ACTB	actin beta
3.317.401	0.5923644		LOC72884!	similar to putative DNA dependent ATPase and
33.167.344	0.8129501		NUCB2	nucleobindin 2
3.316.703	-0.669311		PARP6	poly(ADP-ribose) polymerase family member 6
33.151.122	0.3595549		SMARCD1	SWI/SNF related, matrix associated, actin depe
33.142.035	0.3915116		MYL12B///N	myosin light chain 12B///myosin light chain 12A
33.138.818	0.4185384		BBS10	Bardet-Biedl syndrome 10
33.129.455	0.3324229		XRCC4	X-ray repair cross complementing 4
33.117.112	-0.496756		FUT6	fucosyltransferase 6
33.093.773	-0.5464279		RWDD2B	RWD domain containing 2B
33.090.713	0.3466224		TBRG4	transforming growth factor beta regulator 4
33.084.664	-0.4494352		TREX1	three prime repair exonuclease 1
3.308.148	0.5713103		HACD2	3-hydroxyacyl-CoA dehydratase 2
33.073.398	-0.2327147		SFTPC	surfactant protein C
33.065.886	0.377108		CNPPD1	cyclin Pas1/PHO80 domain containing 1
33.032.961		-11.599.854		
33.017.311	0.4741342		MAGOH	mago homolog, exon junction complex core cor
32.999.311	-0.3427987		COX4I1	cytochrome c oxidase subunit 4I1
32.998.825		-11.452.338	CDO1	cysteine dioxygenase type 1
32.998.402	0.4296979		DYNC2LI1	dynein cytoplasmic 2 light intermediate chain 1

32.991.422	0.532083		ATP6V0B	ATPase H+ transporting V0 subunit b
32.983.589	-0.7349209		F2	coagulation factor II, thrombin
32.977.973	0.3684062		STOML1	stomatin like 1
32.966.261	0.4862736		NCOA2	nuclear receptor coactivator 2
32.944.991	-0.7276111		ALDH7A1	aldehyde dehydrogenase 7 family member A1
32.941.193	0.4001153		WDR55	WD repeat domain 55
32.917.584	0.5998088		ACP6	acid phosphatase 6, lysophosphatidic
32.895.274	0.340913		TUSC2	tumor suppressor candidate 2
32.889.438	0.5743109		TAX1BP3	Tax1 binding protein 3
3.288.924	-0.2960172		TMF1	TATA element modulatory factor 1
32.889.109	0.4549589		MFN1///SY	mitofusin 1///synaptotagmin 7
32.888.523	0.5117694		TBPL1	TATA-box binding protein like 1
32.887.604	-0.3743507		INF2	inverted formin, FH2 and WH2 domain containi
32.879.118	0.6784635		NUDT4P1//	nudix hydrolase 4 pseudogene 1///nudix hydrol
3.287.627	0.5183326		EIF4G3	eukaryotic translation initiation factor 4 gamma
32.859.244	-0.3635878		VPS9D1	VPS9 domain containing 1
32.852.015	0.5891221		NONO	non-POU domain containing, octamer-binding
32.837.662	0.5467923		ALPK3	alpha kinase 3
32.823.304	0.6063665		ZHX2	zinc fingers and homeoboxes 2
32.798.576	0.3953172		NPRL2	NPR2-like, GATOR1 complex subunit
32.794.556	-0.3679564		CX3CR1	C-X3-C motif chemokine receptor 1
32.787.295	-0.3396034		DNAH3	dynein axonemal heavy chain 3
32.768.204	-0.2072538		FGD2	FYVE, RhoGEF and PH domain containing 2
32.756.889	0.5217438		ADIPOR1	adiponectin receptor 1
32.750.031	0.5074044		DIDO1	death inducer-obliterator 1
3.274.835	0.5097581		TOMM22	translocase of outer mitochondrial membrane
32.738.474	-0.6705514		HERC5	HECT and RLD domain containing E3 ubiquitin
32.733.925	0.6601359		HNRNPC	heterogeneous nuclear ribonucleoprotein C (C
32.731.065	-0.7512568		IL10RA	interleukin 10 receptor subunit alpha
32.727.302	0.4887376		PUM2	pumilio RNA binding family member 2
32.715.166	-0.5066643		RXRA	retinoid X receptor alpha
32.692.716	0.4697637		DDX46	DEAD-box helicase 46
32.689.199		-11.355.983	CPS1-IT1	CPS1 intronic transcript 1
32.685.687	0.3908427		GTSE1	G2 and S-phase expressed 1
32.680.817	0.6133283		GPI	glucose-6-phosphate isomerase
32.665.761	-0.254069		TAPT1	transmembrane anterior posterior transformati
32.647.463	-0.2908341		LILRB3///LI	leukocyte immunoglobulin like receptor B3///le
32.645.964	0.4566567		SSX2IP	SSX family member 2 interacting protein
32.641.263		-11.996.264	UGT1A6	UDP glucuronosyltransferase family 1 member
32.637.864	0.3098458		C10orf76	chromosome 10 open reading frame 76
32.635.093	-0.2309137		BMP5	bone morphogenetic protein 5
32.632.778	-0.3030875			
32.585.368	0.4101141		MED21	mediator complex subunit 21
3.257.367	0.5498584		HSPA8	heat shock protein family A (Hsp70) member 8

3.256.552	0.3565311		TP53BP1	tumor protein p53 binding protein 1
32.560.265	-0.2972739			
3.255.633	0.6526269		NETO2	neuropilin and tolloid like 2
32.543.397	0.4076799		WARS2	tryptophanyl tRNA synthetase 2, mitochondrial
32.526.002	-0.2445692		HERC3	HECT and RLD domain containing E3 ubiquitin p
32.520.166	0.334876		SMAD3	SMAD family member 3
32.515.633	0.4176943		DIMT1	DIM1 dimethyladenosine transferase 1 homolo
32.491.603	0.5176227		FAM136A	family with sequence similarity 136 member A
32.476.936	-0.2492945		ZNF276	zinc finger protein 276
32.470.439	0.4420143		DUSP11	dual specificity phosphatase 11
32.458.626		-11.558.717	CYP3A7	cytochrome P450 family 3 subfamily A member
32.456.045	0.2941765		ZNF473	zinc finger protein 473
32.444.544	0.56048		DDAH2	dimethylarginine dimethylaminohydrolase 2
32.438.645	0.7398494		HSPD1	heat shock protein family D (Hsp60) member 1
32.437.013	-0.5436229		LOC72782!	hypothetical LOC727825///ceramide-1-phosph
32.414.395	0.4369977		PRPF40A	pre-mRNA processing factor 40 homolog A
32.406.906	0.407677		INTS9	integrator complex subunit 9
32.402.585	-0.6967095		ERBB2	erb-b2 receptor tyrosine kinase 2
32.399.383		-11.676.158	TACSTD2	tumor-associated calcium signal transducer 2
32.389.118	0.4492288		SMARCA5	SWI/SNF related, matrix associated, actin depe
32.356.813	0.3713355		RPL14	ribosomal protein L14
32.353.317	-0.6478107		IQGAP2	IQ motif containing GTPase activating protein 2
32.343.771	-0.6386041		SRSF7	serine and arginine rich splicing factor 7
32.322.775	0.3940109		DNAJC8	DnaJ heat shock protein family (Hsp40) membe
32.319.222	0.531999		MYCBP	MYC binding protein
32.318.362		-10.340.826	PHLDA1	pleckstrin homology like domain family A meml
32.316.811	-0.6421569		CFLAR	CASP8 and FADD like apoptosis regulator
32.315.341	0.6273757		TNPO1	transportin 1
32.311.365	0.2808796		FAU	FAU, ubiquitin like and ribosomal protein S30 fu
32.280.093	-0.5663928		IL33	interleukin 33
3.226.831	0.4571459		TRMT13	tRNA methyltransferase 13 homolog
32.267.008	0.4019698		PRKD2	protein kinase D2
32.241.232	-0.2006753		KLRD1	killer cell lectin like receptor D1
32.233.512	-0.280911		SOX10	SRY-box 10
32.228.167	0.3346427		UBXN1	UBX domain protein 1
32.215.525	0.3601614		ADRM1	adhesion regulating molecule 1
32.208.764	-0.6660317		CLU	clusterin
3.220.303	0.5983498		NUAK2///AI	NUAK family kinase 2///A-kinase interacting pro
32.192.375	0.4478712			
3.216.227	0.5860816		TMEM106B	transmembrane protein 106B
32.160.112	-0.4676942		FCGR2C	Fc fragment of IgG receptor IIc (gene/pseudoge
32.138.979	0.6961745		GNB5	G protein subunit beta 5
32.138.413	-0.8133537		CISH	cytokine inducible SH2 containing protein
32.127.696	-0.2268868		AMPD1	adenosine monophosphate deaminase 1

32.114.054	-0.6982342		PBX1	PBX homeobox 1
32.110.005	0.4839157		TRMT1	tRNA methyltransferase 1
32.103.086	0.3253357		CEP76	centrosomal protein 76
32.092.238	0.509762		SDCBP	syndecan binding protein
32.078.718	0.3354508		AKIP1	A-kinase interacting protein 1
32.078.116	-0.25729		CEND1	cell cycle exit and neuronal differentiation 1
32.070.954	0.320619		MOB1A	MOB kinase activator 1A
32.070.058	0.4915183		GPR89A///(	G protein-coupled receptor 89A///G protein-cou
32.068.678	-0.40672		TRIM66	tripartite motif containing 66
32.059.802	0.4775164		RPS8	ribosomal protein S8
32.020.427	-0.4707721		LST1	leukocyte specific transcript 1
32.013.559	-0.6959944		SS18L1	SS18L1, nBAF chromatin remodeling complex s
32.006.396	0.378352		GGNBP2	gametogenetin binding protein 2
31.986.617	0.5208007		UBE2I	ubiquitin conjugating enzyme E2 I
31.985.434	-0.2696747		KCNJ5	potassium voltage-gated channel subfamily J m
31.985.118	0.7251109		FDFT1	farnesyl-diphosphate farnesyltransferase 1
31.984.252	0.6527535		CPQ	carboxypeptidase Q
31.970.821	0.3435097		EZH2	enhancer of zeste 2 polycomb repressive comp
31.967.225	0.2900991		VANGL1	VANGL planar cell polarity protein 1
3.196.286	-0.266219		LOC72981!	uncharacterized LOC729815
31.952.164		-11.831.508	UGT1A4	UDP glucuronosyltransferase family 1 member
31.951.246	0.4450303		ORC4	origin recognition complex subunit 4
31.918.092	0.4231305		RBPJ	recombination signal binding protein for immur
31.912.202	-0.3822385			
31.908.481	-0.2397211		ADRA1A	adrenoceptor alpha 1A
31.896.529	0.555113		DSP	desmoplakin
31.891.347		-16.568.337	LUM	lumican
31.876.515	-0.7933508		TXNIP	thioredoxin interacting protein
31.870.287	-0.2344772		MTMR7	myotubularin related protein 7
31.858.663		-11.163.103	PRODH	proline dehydrogenase 1
31.853.935		-12.720.028	UGT1A3///U	UDP glucuronosyltransferase family 1 member
31.840.588	-0.267682		AXL	AXL receptor tyrosine kinase
31.825.384	-0.4566131		LOC65262(	similar to Leukocyte immunoglobulin-like recep
31.816.048	0.3894166		CCNG2	cyclin G2
31.801.849	-0.4600526		NPDC1	neural proliferation, differentiation and control
31.779	0.5438902		DDX18	DEAD-box helicase 18
31.763.082	-0.2283551		DACH1	dachshund family transcription factor 1
31.754.746	0.4684317		GLS	glutaminase
31.754.438	0.5081532		NOC3L	NOC3 like DNA replication regulator
31.748.991	0.7157975		ASAP2	ArfGAP with SH3 domain, ankyrin repeat and PH
3.172.234	0.3944772		IPO9	importin 9
31.714.799	0.3996033		MED1	mediator complex subunit 1
3.170.448	-0.307012		AUTS2	autism susceptibility candidate 2
31.686.827	0.4911342		AMZ2	archaelysin family metallopeptidase 2

31.660.728	-0.33977		EZR	ezrin
31.644.536	0.3516393		TEAD3	TEA domain transcription factor 3
31.643.715	-0.4413264		SLC7A8	solute carrier family 7 member 8
31.641.712	0.2068004		PRIM2	primase (DNA) subunit 2
31.635.674	0.329873		CDK12	cyclin dependent kinase 12
31.621.932	0.9186304		TPM1	tropomyosin 1 (alpha)
3.161.862	0.3630908		LOC72999	eukaryotic translation elongation factor 1 gamn
31.617.313	0.2623842		GEMIN2	gem nuclear organelle associated protein 2
31.601.713	0.4134197		TMPO	thymopoietin
31.592.571	0.3809743		LOC72999	eukaryotic translation elongation factor 1 gamn
31.586.392	-0.4993011		NR2F2	nuclear receptor subfamily 2 group F member 2
31.576.258	0.3873416		CCDC22	coiled-coil domain containing 22
31.573.141	-0.5678306		CHP1	calcineurin like EF-hand protein 1
31.572.671	0.3964052		ANKLE2	ankyrin repeat and LEM domain containing 2
31.568.133	0.623599		PRKAR1A	protein kinase cAMP-dependent type I regulator
31.558.813	0.4047091		NAT9	N-acetyltransferase 9 (putative)
31.541.683	0.5160138		GPR89A///	G protein-coupled receptor 89A///G protein-cou
31.541.471	0.5134514		USP14	ubiquitin specific peptidase 14
31.532.827	-0.2291892		RAP2A	RAP2A, member of RAS oncogene family
31.522.714	0.4244435		UBE2L3	ubiquitin conjugating enzyme E2 L3
31.517.888	0.4551715		MAP4K5	mitogen-activated protein kinase kinase kinase
31.512.807	0.5302504		ZMIZ1	zinc finger MIZ-type containing 1
31.510.405	0.481791		TRPC1	transient receptor potential cation channel sub
31.508.018	0.4004761		ARID1A	AT-rich interaction domain 1A
31.507.385	0.680879		YAP1	Yes associated protein 1
31.506.073	0.5851865		ESPL1	extra spindle pole bodies like 1, separase
31.504.595	-0.9593928		AEBP1	AE binding protein 1
31.503.212	0.51937		ACOT7	acyl-CoA thioesterase 7
31.498.091	-0.2878807		MAPRE3	microtubule associated protein RP/EB family m
3.149.524	-0.6186349		ALDH3A2	aldehyde dehydrogenase 3 family member A2
31.482.818	0.3252358		PPP1CB	protein phosphatase 1 catalytic subunit beta
31.474.536	0.4960978		PRDX1	peroxiredoxin 1
31.428.813	0.3118173		MYNN	myoneurin
31.424.656		-11.816.725	UGT1A4	UDP glucuronosyltransferase family 1 member
31.422.837	0.2701918		TRAF2	TNF receptor associated factor 2
31.422.256	0.6295569		ZNF532	zinc finger protein 532
31.418.419	0.447306		BACH1	BTB domain and CNC homolog 1
31.415.032	-0.6080791		AKAP13	A-kinase anchoring protein 13
31.414.375	0.3655443		OSER1	oxidative stress responsive serine rich 1
31.407.354	-0.2265072		NAV1	neuron navigator 1
31.406.601	-0.6851237		CSRP1	cysteine and glycine rich protein 1
31.406.582	-0.9905233		ADAMTS1	ADAM metallopeptidase with thrombospondin t
31.393.414	0.6456152		MINA	MYC induced nuclear antigen
31.387.374	-0.5540302		CHD9	chromodomain helicase DNA binding protein 9

31.371.471	0.574173		SACS	sacsin molecular chaperone
31.371.419	0.3800388		RPL29	ribosomal protein L29
31.371.106	-0.4866368		MTHFD2L	methylenetetrahydrofolate dehydrogenase (NA
31.364.006	0.3607871		GPR107	G protein-coupled receptor 107
31.361.021	0.2884438		CCHCR1	coiled-coil alpha-helical rod protein 1
31.357.899	0.5337188		MRPS7	mitochondrial ribosomal protein S7
31.356.037	0.3333845		POLR2F	RNA polymerase II subunit F
31.349.311	0.5284248		CSGALNAC	chondroitin sulfate N-acetylgalactosaminyltran
31.326.667	-0.2748897		KAT6A	lysine acetyltransferase 6A
31.321.563	0.7451256		MID1	midline 1
31.296.341	0.5963361		CSNK2A1	casein kinase 2 alpha 1
31.296.148	-0.2602088		HNF1B	HNF1 homeobox B
31.269.781		-14.076.289	HLA-C	major histocompatibility complex, class I, C
3.123.059	-0.4257389		BCO2///DG	beta-carotene oxygenase 2///diacylglycerol kin
31.225.638	0.5325449		CPNE3	copine 3
31.217.931	0.3788256		TRRAP	transformation/transcription domain associate
31.213.934	0.4265076		PREPL	prolyl endopeptidase-like
31.213.007	0.4747808		COQ10B	coenzyme Q10B
31.200.748	-0.3086985		PRB3	proline rich protein BstNI subfamily 3
31.190.961	-0.4505315		SLC31A1	solute carrier family 31 member 1
31.184.572	0.4480439		DHX30	DEAH-box helicase 30
31.182.867	-0.2188641			
31.159.869	0.3787369		C2CD3	C2 calcium dependent domain containing 3
31.154.503	0.4138312		POLR2B	RNA polymerase II subunit B
31.143.131	-0.501376		MUC3B	mucin 3B, cell surface associated
31.133.237	0.5363059		ATP13A1	ATPase 13A1
31.120.739	0.3552451		RDH14	retinol dehydrogenase 14 (all-trans/9-cis/11-ci
31.111.789	0.4951849		MRPS11	mitochondrial ribosomal protein S11
31.100.509	-0.2683551		FAM134B	family with sequence similarity 134 member B
31.081.408	-0.8304811		EXT1	exostosin glycosyltransferase 1
31.079.841	0.5135394		ATP6V1D	ATPase H+ transporting V1 subunit D
310.569	-0.3096887		AOAH	acyloxyacyl hydrolase
31.053.078	0.5741917		SLC7A11	solute carrier family 7 member 11
31.030.059	0.3657825		GAPVD1	GTPase activating protein and VPS9 domains 1
31.014.602	0.4587762		RTN3	reticulon 3
30.996.899	0.3318896		ATG7	autophagy related 7
30.992.401	0.3644346		BRE	brain and reproductive organ-expressed (TNFR
30.978.931	0.3710322		PTPRA	protein tyrosine phosphatase, receptor type A
30.969.451	-0.2451567		CYP2E1	cytochrome P450 family 2 subfamily E member
30.965.325	0.3708603		CDC27	cell division cycle 27
30.962.592	0.7270768		RAB4A	RAB4A, member RAS oncogene family
30.953.556	-0.3572106		ARVCF	armadillo repeat gene deleted in velocardiofaci
30.948.778	0.3765163		UBE3B	ubiquitin protein ligase E3B
3.094.264	0.3850885		RECQL4	RecQ like helicase 4

30.936.301	0.4263784		MARCH7	membrane associated ring-CH-type finger 7
30.933.789	0.5927305		GSPT1	G1 to S phase transition 1
30.926.277	-0.3476579		N4BP1	NEDD4 binding protein 1
30.924.086	-0.9871142		NR4A2	nuclear receptor subfamily 4 group A member 2
30.910.844	-0.2111365		SCN7A	sodium voltage-gated channel alpha subunit 7
30.899.674	0.4773076		PTMAP8///l	prothymosin, alpha pseudogene 8///prothymos
30.850.744	-0.5514388		LOC65387	similar to Complement C3 precursor
30.849.036	0.3499736		ERCC1	ERCC excision repair 1, endonuclease non-cata
30.846.927	0.440297		ARL3	ADP ribosylation factor like GTPase 3
30.838.538	-0.1908208		ORAI2	ORAI calcium release-activated calcium modul
30.825.966	0.4792221			
30.820.479	0.4800135		NELFCD	negative elongation factor complex member C/
30.783.008	-0.8083972		LOC73092	similar to Pancreatic alpha-amylase precursor
30.781.823	0.3210529		AIM1L	absent in melanoma 1-like
307.721	-0.444716		APOC1	apolipoprotein C1
30.767.617	0.325656		TUBG2	tubulin gamma 2
30.764.623	0.3052976		C1orf35	chromosome 1 open reading frame 35
30.762.373	0.4650746		HIST1H2AG	histone cluster 1, H2ag
30.761.843	-0.7118847		HSD17B8	hydroxysteroid 17-beta dehydrogenase 8
30.758.038	0.3836931		WDR77	WD repeat domain 77
30.735.337	-0.4044414		CFDP1	craniofacial development protein 1
30.735.213	-0.7174129		MYH11	myosin heavy chain 11
30.730.819	0.3243279		PKNOX1	PBX/knotted 1 homeobox 1
30.724.266	0.3030166		PDCL	phosducin like
30.682.141	-0.2475977			
3.067.675	-0.4283726		LST1	leukocyte specific transcript 1
30.674.796	0.3943173		C6orf62	chromosome 6 open reading frame 62
30.671.157	0.2691584		CCDC93	coiled-coil domain containing 93
30.663.095	-0.4030143			
30.662.993	0.5713497		SLC25A16	solute carrier family 25 member 16
30.647.422	0.3031737		RRP1	ribosomal RNA processing 1
30.639.528	-0.3887293		LST1	leukocyte specific transcript 1
30.630.956	0.5481967		GATAD2A	GATA zinc finger domain containing 2A
3.062.376	0.4866941			
30.611.231		10.842.028	ENO1	enolase 1
30.595.343	0.359489		GALNT10	polypeptide N-acetylgalactosaminyltransferase
30.580.891	-0.3818486		IL1B	interleukin 1 beta
30.577.968	0.4701124		EIF4G3	eukaryotic translation initiation factor 4 gamma
30.559.725	0.6175573		PRPF4B	pre-mRNA processing factor 4B
30.550.875	-0.4806983		LRP1	LDL receptor related protein 1
30.548.914	0.4353817		CPSF1	cleavage and polyadenylation specific factor 1
30.539.916	-0.3995024		SGSM2	small G protein signaling modulator 2
30.537.094	0.495248		ADAM10	ADAM metallopeptidase domain 10
30.529.967	-0.4980082		RORC	RAR related orphan receptor C

30.529.202	-0.4125144		SETBP1	SET binding protein 1
30.511.296	-0.2587026		MMP19	matrix metallopeptidase 19
30.500.231	0.4440525		SLC7A6	solute carrier family 7 member 6
30.493.856	-0.6173879		COL14A1	collagen type XIV alpha 1 chain
30.493.424	-0.7039332		MLXIPL	MLX interacting protein like
30.490.164	-0.4299396		WWC2	WW and C2 domain containing 2
30.490.158	-0.5018064		CYP7B1	cytochrome P450 family 7 subfamily B member
3.048.844	-0.7569952			
30.474.076	0.7905221		MIF	macrophage migration inhibitory factor (glycosy
30.473.149	0.3900948		RNASEH1	ribonuclease H1
30.467.514	-0.7915345		ARSE	arylsulfatase E (chondrodysplasia punctata 1)
30.459.399	-0.2750314		LILRA2	leukocyte immunoglobulin like receptor A2
30.459.306	-0.3040986		ZER1	zyg-11 related cell cycle regulator
30.454.586	0.5474282		CALM3	calmodulin 3
30.442.534	0.3922226		HMBS	hydroxymethylbilane synthase
30.427.295	0.365128		RAB5B	RAB5B, member RAS oncogene family
30.424.884	0.3488309		RBMX2	RNA binding motif protein, X-linked 2
30.415.441	0.6163856		RPS6KA3	ribosomal protein S6 kinase A3
30.398.057	-0.2295236		ZNF440	zinc finger protein 440
30.396.441	0.4272416		H3F3B	H3 histone, family 3B
30.390.261	-0.2748931		JUN	Jun proto-oncogene, AP-1 transcription factor s
3.036.293	0.7632122		CLDN15	claudin 15
30.351.036	0.5122852		DNAJC10	DnaJ heat shock protein family (Hsp40) membe
3.033.374	0.4492111		CALM3	calmodulin 3
30.324.658	0.3645266		GAB2	GRB2 associated binding protein 2
30.319.486	0.5501273		KHDRBS3	KH RNA binding domain containing, signal trans
30.310.886	-0.2285472		PYHIN1	pyrin and HIN domain family member 1
3.028.122	0.5729102		RAB40B	RAB40B, member RAS oncogene family
30.280.121	0.4712153		BCAP29	B-cell receptor-associated protein 29
30.278.844	0.2891819		ZMAT5	zinc finger matrin-type 5
3.026.541	0.4438928		TULP3	tubby like protein 3
30.262.606	0.459558		EFHD2	EF-hand domain family member D2
30.262.526	0.4683858		MRPS14	mitochondrial ribosomal protein S14
30.249.093	-0.7345153		CYB5A	cytochrome b5 type A
30.245.065	-0.2717758		SNED1	sushi, nidogen and EGF like domains 1
3.024.286	-0.4897664		CFD	complement factor D
30.234.796		-11.363.604	IGFBP1	insulin like growth factor binding protein 1
30.232.202	0.5214845		RAB21	RAB21, member RAS oncogene family
30.215.539	0.4158697		ZCCHC10	zinc finger CCHC-type containing 10
30.206.597	0.4867695		ZNF148	zinc finger protein 148
30.185.025	0.5910239		SUZ12P1///	SUZ12 polycomb repressive complex 2 subunit
30.184.004	0.5803948		RPA3	replication protein A3
30.182.806	0.529083		GTF2H2B	general transcription factor IIH subunit 2B (pse
30.166.341	0.5047417		CRY1	cryptochrome circadian clock 1

30.159.324	0.5121169	HNRNPH2	heterogeneous nuclear ribonucleoprotein H2 (H
30.148.003	0.5372856	PHF21A	PHD finger protein 21A
30.133.781	0.5241733	URI1	URI1, prefoldin like chaperone
30.130.281	0.381794	CUL3	cullin 3
30.128.845	-0.5347907	CLK4	CDC like kinase 4
30.127.232	0.4581437	ALG9	ALG9, alpha-1,2-mannosyltransferase
30.125.815	0.4302131	C11orf58	chromosome 11 open reading frame 58
30.123.539	0.4848582	NDUFA6	NADH:ubiquinone oxidoreductase subunit A6
30.114.453	-0.2583707	POLD2	DAN polymerase delta 2, accessory subunit
30.112.957	0.4609904	MLH1	mutL homolog 1
30.105.639	0.3580943	EIF3L	eukaryotic translation initiation factor 3 subunit
30.104.984	-0.3580592	DAPK1	death associated protein kinase 1
3.007.672	0.3444347	INTS1	integrator complex subunit 1
30.074.128	-0.2208603	GPR3	G protein-coupled receptor 3
30.074.034	0.7077332	PIEZO2	piezo type mechanosensitive ion channel comp
30.058.483	0.2896966	UNC45A	unc-45 myosin chaperone A
30.056.837	0.6178565	EDEM3	ER degradation enhancing alpha-mannosidase
3.005.172	0.5420173	DHX40	DEAH-box helicase 40
30.047.015	-0.2376123	FLT3	fms related tyrosine kinase 3
30.034.429	0.3718134	WASH3P///	WAS protein family homolog 3 pseudogene///ca
30.023.185	-0.3884628	MBD4	methyl-CpG binding domain 4, DNA glycosylase
3.001.618	0.2865785	KLHL7	kelch like family member 7
29.984.452	0.4269141	ATP5G2	ATP synthase, H+ transporting, mitochondrial F
29.976.487	0.3286217	EXOG	endo/exonuclease (5'-3'), endonuclease G-like
29.972.814	0.3033143	PAQR4	progestin and adipoQ receptor family member 4
2.996.253	0.583568	SLC6A8	solute carrier family 6 member 8
29.961.851	0.4881966	CHMP2B	charged multivesicular body protein 2B
29.958.891	0.7676015	OPN3	opsin 3
29.957.438	0.5763825	HIST1H2BH	histone cluster 1, H2bh
29.954.612	-0.8262389	ANPEP	alanyl aminopeptidase, membrane
29.954.314	-0.6276078	SPRY2	sprouty RTK signaling antagonist 2
29.953.836	0.2700437	TRABD	TraB domain containing
29.953.134	0.733829	SOX4	SRY-box 4
299.367	0.4520953	PSMA3	proteasome subunit alpha 3
29.932.288	0.4236111	CHAF1A	chromatin assembly factor 1 subunit A
2.993.123	0.2999384	RNF41	ring finger protein 41
29.930.809	-0.1795268	CR1	complement component 3b/4b receptor 1 (Kno
29.928.237	-0.2472511	ALOX5	arachidonate 5-lipoxygenase
29.918.851	0.5647456	PPARD	peroxisome proliferator activated receptor delta
29.912.409	0.3798895	TJAP1	tight junction associated protein 1
29.900.779	-0.4325716	ZNF862	zinc finger protein 862
29.896.844	-0.2588552	COL14A1	collagen type XIV alpha 1 chain
29.879.277	0.4763209	SEC61A1	Sec61 translocon alpha 1 subunit
29.877.765	-0.6924121	ALDH1L1	aldehyde dehydrogenase 1 family member L1

29.875.766	0.4148623		ZDHHC13	zinc finger DHHC-type containing 13
29.852.299	-0.2481244		NR6A1	nuclear receptor subfamily 6 group A member 1
2.985.028	0.4216472		ATP2C1	ATPase secretory pathway Ca2+ transporting 1
2.984.122	-0.3457801		REC8	REC8 meiotic recombination protein
29.836.807	0.631361		MAPK13	mitogen-activated protein kinase 13
29.835.104	0.3634408		ATP5C1	ATP synthase, H+ transporting, mitochondrial F
29.829.936		-10.398.294	SH3YL1	SH3 and SYLF domain containing 1
29.812.738	0.3894716		YBX1	Y-box binding protein 1
29.789.062	0.322317		LOC65325(	similar to RAB, member of RAS oncogene family
29.767.626	-0.2063227		CACNG5	calcium voltage-gated channel auxiliary subuni
29.751.983	0.3533803		DHX16	DEAH-box helicase 16
29.747.065	0.4465697		MYCBP	MYC binding protein
29.731.489	0.4783315		DPP8	dipeptidyl peptidase 8
29.704.504	0.4298107		PRPF39///T	pre-mRNA processing factor 39///TATA-box bin(
29.702.214	-0.9088551		C1QA	complement C1q A chain
2.969.876	0.6356554		GNAI1	G protein subunit alpha i1
29.691.035	0.2751978		RC3H2	ring finger and CCCH-type domains 2
29.687.419	-0.7726713		LPIN2	lipin 2
29.686.762	-0.2078325			
29.678.037	0.4081512		PDCD11	programmed cell death 11
29.671.351	0.2519176		GINS3	GINS complex subunit 3
2.966.942	-0.4682716		PRR5	proline rich 5
29.663.025	0.4388878		VCP	valosin containing protein
2.966.287	0.4746388		RBBP8///G/	RB binding protein 8, endonuclease///galactoki
29.645.719	0.2833443		ACTB	actin beta
29.642.936	0.5204606		TMCC1	transmembrane and coiled-coil domain family
29.642.098		-1.013.164	C4BPA	complement component 4 binding protein alph
29.629.642	0.4389583		ARFGEF1	ADP ribosylation factor guanine nucleotide excl
29.627.408	0.3240729		MCM10	minichromosome maintenance 10 replication i
29.621.265	0.4198243		PBXIP1	PBX homeobox interacting protein 1
29.604.975	0.3640278		TRAPPC9	trafficking protein particle complex 9
29.574.531	0.4911219		CHFR	checkpoint with forkhead and ring finger domai
29.557.941	0.4519328		C1orf112	chromosome 1 open reading frame 112
29.556.433	0.3383397		NDUFB4	NADH:ubiquinone oxidoreductase subunit B4
29.543.493	0.4700226		DLGAP4	DLG associated protein 4
29.542.283	-0.6187234		VWA8	von Willebrand factor A domain containing 8
29.538.074	-0.5560787		RNF130	ring finger protein 130
29.532.893	0.2436759		FEM1B	fem-1 homolog B
295.315	0.3518492		SF3B1	splicing factor 3b subunit 1
29.523.881	0.2744229		SETD4	SET domain containing 4
29.523.825	0.4505916		CREB1	cAMP responsive element binding protein 1
29.511.694	-0.3260804		CLN8	ceroid-lipofuscinosis, neuronal 8
2.951.135	0.3454112		SBF1	SET binding factor 1
29.500.893	0.7248199		SPTSSA	serine palmitoyltransferase small subunit A

29.490.397	-0.3382993		HAB1	B1 for mucin
29.478.306	0.247224		ZNF142	zinc finger protein 142
29.468.517	0.364828		KCNMB3	potassium calcium-activated channel subfamil
29.451.674	-0.2675703		PRB4	proline rich protein BstNI subfamily 4
29.451.144	0.6313583		TFB2M	transcription factor B2, mitochondrial
29.391.969	-0.7803317		ABCB11	ATP binding cassette subfamily B member 11
2.938.192	0.3877085		POLR1C	RNA polymerase I subunit C
29.380.107	-0.3184071		CDKN2A	cyclin dependent kinase inhibitor 2A
29.360.625	0.3142987		NLK	nemo like kinase
29.351.113	-0.3044526		NFATC3	nuclear factor of activated T-cells 3
29.349.975	0.9262107		PALLD	palladin, cytoskeletal associated protein
29.347.156	0.3466371		TASP1	taspase 1
29.318.505	-0.5486918		MYH11	myosin heavy chain 11
29.304.665	0.2741844		RRP9	ribosomal RNA processing 9, small subunit (SS
29.290.886	-0.2531918		CEACAM3	carcinoembryonic antigen related cell adhesior
29.284.522	-0.2105239			
29.278.881	0.4462253		UBE2L3	ubiquitin conjugating enzyme E2 L3
29.273.349	-0.6300693		FN1	fibronectin 1
29.271.577	-0.2169835		AVIL	advillin
2.926.497	0.4771064		SEPHS1	selenophosphate synthetase 1
29.264.913	0.3073756		C20orf27	chromosome 20 open reading frame 27
29.257.589	-0.2373558			
29.257.186	0.8027676		SLC29A1	solute carrier family 29 member 1 (Augustine bl
2.924.943		10.577.991	COL5A2	collagen type V alpha 2 chain
29.242.322	0.7717573		MNS1	meiosis specific nuclear structural 1
29.236.126	0.3311922		ANAPC5	anaphase promoting complex subunit 5
29.215.605	-0.4692405		SHMT2	serine hydroxymethyltransferase 2
29.213.721	-0.3221025			
29.208.178	0.2904618		DDX42	DEAD-box helicase 42
29.204.059	0.5466704		METAP2	methionyl aminopeptidase 2
29.196.938		13.235.744	MAGEA6	MAGE family member A6
29.193.714	0.3926007		BTF3	basic transcription factor 3
29.192.388	-0.3067224		HIBADH	3-hydroxyisobutyrate dehydrogenase
29.191.268	0.7263185		CD46	CD46 molecule
2.918.112	-0.4308821		LST1	leukocyte specific transcript 1
29.179.765	-0.5322786		GSAP	gamma-secretase activating protein
29.160.715	-0.2563088			
2.912.491	-0.5573838		AQP1	aquaporin 1 (Colton blood group)
29.120.175	0.4485657		SET	SET nuclear proto-oncogene
29.118.924	0.8104907		ELF3	E74 like ETS transcription factor 3
29.117.267	-0.2164474		FUT3	fucosyltransferase 3 (Lewis blood group)
29.111.058	-0.3174657		COL6A2	collagen type VI alpha 2 chain
29.106.286	-0.2958731		DRP2	dystrophin related protein 2
29.099.276	0.5936545		ATP6AP2	ATPase H+ transporting accessory protein 2

29.090.444	0.514263		ZNF165	zinc finger protein 165
29.087.053		14.591.243	MAGEA3	MAGE family member A3
29.059.804	-0.3158422		RRN3P1	RRN3 homolog, RNA polymerase I transcription
2.905.323	0.46406		NF1	neurofibromin 1
29.051.425	0.3072081		RPS14P3//,	ribosomal protein S14 pseudogene 3///ribosom
29.043.552	-0.8230753		CCL5	C-C motif chemokine ligand 5
29.042.308	0.4485578		EXOSC10	exosome component 10
29.038.282	0.2983647		ZNF200	zinc finger protein 200
2.903.043	-0.2565273		STAP1	signal transducing adaptor family member 1
29.016.453	0.8252294		ELOVL2	ELOVL fatty acid elongase 2
29.008.612	-0.6082899		AMBP	alpha-1-microglobulin/bikunin precursor
29.007.827	0.4019712		ELP4	elongator acetyltransferase complex subunit 4
29.002.873	0.4610309		ACP1	acid phosphatase 1, soluble
28.993.302	-0.5805137			
28.989.494	0.8125519		BMP2	bone morphogenetic protein 2
289.886	-0.7390479		PECR	peroxisomal trans-2-enoyl-CoA reductase
28.978.995	0.4798765		TRA2B	transformer 2 beta homolog (Drosophila)
2.895.808	0.3557098			
28.954.842	0.4823606		DUT	deoxyuridine triphosphatase
28.947.811	-0.4429182		SPATA2L	spermatogenesis associated 2 like
28.945.773	-0.5229661		FZD1	frizzled class receptor 1
2.894.019	-0.2732022		GABARAPL:	GABA type A receptor associated protein like 3
28.935.968	0.3204758		MAP1S	microtubule associated protein 1S
28.929.133	0.4070097		POLR1C	RNA polymerase I subunit C
28.905.703	-0.4062336		CCPG1	cell cycle progression 1
28.903.165	-0.8889651		IFITM1	interferon induced transmembrane protein 1
2.889.949	0.425179		ACOT9	acyl-CoA thioesterase 9
28.898.452	-0.6251807		THBD	thrombomodulin
28.896.655	0.3658152		YME1L1	YME1 like 1 ATPase
28.893.437	0.3644487		DSTYK	dual serine/threonine and tyrosine protein kina
28.892.631	0.9743385		SCD	stearoyl-CoA desaturase
28.877.661	0.5886349		SART3	squamous cell carcinoma antigen recognized b
28.871.767	0.5146594		DDAH2	dimethylarginine dimethylaminohydrolase 2
28.870.187	-0.4233596		DBT	dihydrolipoamide branched chain transacylase
28.867.926	-0.420193		GALNS	galactosamine (N-acetyl)-6-sulfatase
28.862.333	0.3834838		CIT	citron rho-interacting serine/threonine kinase
28.859.191	0.3589637		SAYSD1	SAYSVFN motif domain containing 1
28.857.179	0.3369174		RBM39	RNA binding motif protein 39
28.856.145	0.7189172		DFNA5	DFNA5, deafness associated tumor suppressor
28.855.899	0.4523915		ARL2	ADP ribosylation factor like GTPase 2
2.885.479	-0.2249002		RAB5A	RAB5A, member RAS oncogene family
28.834.936	0.5531017		TM2D1	TM2 domain containing 1
28.826.347	0.3431671		SENP5	SUMO1/sentrin specific peptidase 5
28.825.259	-0.6621995		JUN	Jun proto-oncogene, AP-1 transcription factor s

28.814.512	0.3571218		PSMC3IP	PSMC3 interacting protein
28.810.501	0.5557851		RIT1	Ras like without CAAX 1
288.079	-0.7005357		CCL4	C-C motif chemokine ligand 4
28.788.252	0.3376408		MAML1	mastermind like transcriptional coactivator 1
28.770.057		-10.731.519	ECHDC3	enoyl-CoA hydratase domain containing 3
28.765.727	-0.2808103		DSCAM	DS cell adhesion molecule
28.749.491	0.7241999		FDFT1	farnesyl-diphosphate farnesyltransferase 1
28.748.851	0.9298251		SLC39A4	solute carrier family 39 member 4
28.740.688	0.5243356		LHFPL2	lipoma HMGIC fusion partner-like 2
2.872.583	0.4158549		TRIM52	tripartite motif containing 52
28.724.921	0.3397819		MAPK1	mitogen-activated protein kinase 1
2.869.566	0.3483465		C2CD3	C2 calcium dependent domain containing 3
28.682.105	0.5115878		MAP4K5	mitogen-activated protein kinase kinase kinase
28.680.061	0.2899584		MAP4	microtubule associated protein 4
28.679.259	-0.3114313		EYA2	EYA transcriptional coactivator and phosphatas
2.866.485	0.3664129		CELSR3	cadherin EGF LAG seven-pass G-type receptor 3
28.659.941	0.2582766		LOC38816:	LOC388161///golgin A2 pseudogene 7///golgin
28.655.108	-0.8105479		CH25H	cholesterol 25-hydroxylase
28.642.992	0.4736016		MAT2A	methionine adenosyltransferase 2A
28.628.153	0.872568		RGCC	regulator of cell cycle
28.609.401	0.5096551		CYB5R4	cytochrome b5 reductase 4
28.607.208	0.4880693		MCAM	melanoma cell adhesion molecule
28.600.558	0.4309925		FLT1	fms related tyrosine kinase 1
28.593.746	0.3744297		TRIM27	tripartite motif containing 27
28.593.414	0.3604955		ALG12	ALG12, alpha-1,6-mannosyltransferase
28.579.987	-0.5834448		ISOC1	isochorismatase domain containing 1
28.578.712	0.6467741		UPF3A	UPF3 regulator of nonsense transcripts homolo
28.557.164	-0.5491589		CLU	clusterin
2.855.106	0.6651688		CANX	calnexin
2.855.071	0.3596898		ITPKA	inositol-trisphosphate 3-kinase A
28.549.787	-0.248434			
28.547.267	0.3457713		HAUS6	HAUS augmin like complex subunit 6
28.545.995	0.4082347		PIN4	peptidylprolyl cis/trans isomerase, NIMA-intera
28.533.666	-0.6669815		TGDS	TDP-glucose 4,6-dehydratase
28.530.863	-0.4722433		ABCC6	ATP binding cassette subfamily C member 6
28.529.164	0.5267745		AP2M1	adaptor related protein complex 2 mu 1 subunit
28.506.119	0.5234747		MLEC	malectin
28.505.377	-0.6394197		LOC73010:	similar to Glycine cleavage system H protein, m
28.503.276	-0.2943298		PTGER3	prostaglandin E receptor 3
28.500.272	0.4888681		PSMG1	proteasome assembly chaperone 1
28.491.709	-0.2338084		TPT1	tumor protein, translationally-controlled 1
28.487.749	-0.935046		F7	coagulation factor VII
28.486.403	0.7427141		CD46	CD46 molecule
28.479.456	0.5172123		ZC3H15	zinc finger CCCH-type containing 15

28.476.665	0.4365684		CRLF3	cytokine receptor like factor 3
28.470.791	0.3891522		NCLN	nicalin
2.846.539	-0.3077283			
28.451.416	0.3216631		NUP188	nucleoporin 188
28.439.917	0.2802327		TMUB2	transmembrane and ubiquitin like domain cont
28.424.911	0.3977331		EWSR1	EWS RNA binding protein 1
28.423.945	-0.5668878		LOC72883(	similar to C-C motif chemokine 3-like 1 precurs
28.420.634	0.3493302		PLXNC1	plexin C1
28.419.347	0.5267458		BCAT2	branched chain amino acid transaminase 2
28.416.003	0.4053658		ACTR1A	ARP1 actin-related protein 1 homolog A, centra
28.409.028	0.6039735		MORF4///M	mortality factor 4 (pseudogene)///mortality fact
28.396.338	0.3780546		TFCP2	transcription factor CP2
28.387.723	0.4437748		EIF1B	eukaryotic translation initiation factor 1B
28.364.989	0.2155274		PI4K2A	phosphatidylinositol 4-kinase type 2 alpha
28.356.888	0.4355515		ANKRD49	ankyrin repeat domain 49
28.355.233	0.4197774		SRI	sorcin
28.347.845	-0.3201587		PTN	pleiotrophin
28.337.815	-0.2812472		TK2	thymidine kinase 2, mitochondrial
28.333.175	0.3390012		ATP5L	ATP synthase, H+ transporting, mitochondrial F
28.327.641		10.218.321	SOBP	sine oculis binding protein homolog
28.321.521	0.3132234		SNX16	sorting nexin 16
28.319.742	0.4472816		DYNLT1	dynein light chain Tctex-type 1
28.317.045	-0.5243712		GLRX	glutaredoxin
28.300.415	-0.2060583		CCL23	C-C motif chemokine ligand 23
28.298.812	0.3841935		NMB	neuromedin B
2.829.744	-0.4433323		ITSN1	intersectin 1
28.296.532	0.3875496		SF3A3	splicing factor 3a subunit 3
28.280.795	-0.2333842		HS3ST3A1	heparan sulfate-glucosamine 3-sulfotransferas
28.276.185	-0.7229297		CFB	complement factor B
28.251.976	0.3049714		MTOR	mechanistic target of rapamycin
28.247.507	-0.2486006		GRIP2	glutamate receptor interacting protein 2
28.245.599	0.6513313		SH3GLB1	SH3 domain containing GRB2 like endophilin B
2.823.166	-0.3598753		LST1	leukocyte specific transcript 1
28.220.255	0.3589113		LOC72999(	eukaryotic translation elongation factor 1 gamn
28.204.655	0.5940189		TBL1XR1	transducin (beta)-like 1 X-linked receptor 1
28.199.433		-11.315.655	SLC23A2	solute carrier family 23 member 2
28.190.711	0.4007032		RNF24	ring finger protein 24
28.168.112	-0.4037688		LAMA2	laminin subunit alpha 2
28.150.318	-0.4044193		STAG3	stromal antigen 3
281.437	-0.3927959		PRG2	proteoglycan 2, pro eosinophil major basic prot
28.139.892	0.550869		PPIB	peptidylprolyl isomerase B
28.126.207	0.6057776		RSRC1	arginine and serine rich coiled-coil 1
28.125.226	0.4474018		RAF1	Raf-1 proto-oncogene, serine/threonine kinase
28.111.599	-0.4979575		MMACHC	methylmalonic aciduria (cobalamin deficiency)

28.109.428	-0.1820518		AMOT	angiomotin
28.107.105	0.3699743		INTS5	integrator complex subunit 5
28.105.913	0.4615204		POGZ	pogo transposable element with ZNF domain
28.102.927	0.6935624		GREB1	growth regulation by estrogen in breast cancer 1
28.101.997	-0.6063875		PCCA	propionyl-CoA carboxylase alpha subunit
28.101.107	-0.2716451		TAF9B	TATA-box binding protein associated factor 9b
28.098.823	0.4512788		EDRF1	erythroid differentiation regulatory factor 1
28.095.564	0.3461426			
28.087.638	-0.2315228		CFAP70	cilia and flagella associated protein 70
28.086.162	-0.3460499		ABO	ABO blood group (transferase A, alpha 1-3-N-ac
28.078.777	-0.36004		CD1C	CD1c molecule
28.052.383	0.3048925		DHX35	DEAH-box helicase 35
28.050.913	0.3250407		LOC81691	exonuclease NEF-sp
2.805.026	0.3772431		C12orf49	chromosome 12 open reading frame 49
28.049.454	0.3602665		B9D1	B9 domain containing 1
28.043.536	-0.8318534		GLUD1	glutamate dehydrogenase 1
28.029.633	0.3892452		LGALSL	galectin like
28.023.295	0.4458923		RPS4XP7///	ribosomal protein S4X pseudogene 7///ribosom
28.018.089	0.4807182		FBXO28	F-box protein 28
28.003.922	-0.2548355		ST3GAL6	ST3 beta-galactoside alpha-2,3-sialyltransferas
28.003.027	-0.2191169		SCAND2P	SCAN domain containing 2 pseudogene
28.001.485		1.014.134	ME1	malic enzyme 1
27.995.788	-0.2740587		ACAP1	ArfGAP with coiled-coil, ankyrin repeat and PH
27.992.551	-0.2603904		NUP88	nucleoporin 88
27.977.439	-0.212226		PRDM5	PR/SET domain 5
27.974.131	-0.3125047		MVK	mevalonate kinase
27.973.221	0.5373885		ERGIC2	ERGIC and golgi 2
27.972.172	0.2481475		LOC72802	chromosome X open reading frame 56 pseudog
27.971.907	0.533199		ALCAM	activated leukocyte cell adhesion molecule
27.965.166	0.3979937		SNU13	SNU13 homolog, small nuclear ribonucleoprote
27.961.069	0.4290165		NOMO3///A	NODAL modulator 3///apolipoprotein O
27.960.601	-0.5425739		ORAI3	ORAI calcium release-activated calcium modul
27.944.224	-0.3350656		RAB3IL1	RAB3A interacting protein like 1
27.936.784	-0.5685654		TTPAL///AR	alpha tocopherol transfer protein like///ADP-rib
27.930.748	0.4688166		ARIH1	ariadne RBR E3 ubiquitin protein ligase 1
2.792.985	-0.4754099		CASP4	caspase 4
27.928.855	-0.4009948		CAMK2B	calcium/calmodulin dependent protein kinase
27.924.874	-0.3954888		PBX1	PBX homeobox 1
27.916.648	0.3850382		PWP2	PWP2 periodic tryptophan protein homolog (yea
27.914.741	-0.526343		MXRA7///TC	matrix remodeling associated 7///transcription
2.791.086	0.5398108		DUSP3	dual specificity phosphatase 3
27.902.385	0.2810356		ZDHHC3	zinc finger DHHC-type containing 3
27.901.934	0.3007034		CENPI	centromere protein I
27.882.764	-0.6864025		ACOT1///AC	acyl-CoA thioesterase 1///acyl-CoA thioesteras

27.863.478	-0.5947814		TNFRSF1B	TNF receptor superfamily member 1B
27.854.927	0.3934194		RNGTT	RNA guanylyltransferase and 5'-phosphatase
27.849.809	0.3120699		WIPF2	WAS/WASL interacting protein family member 2
27.849.152	0.3935771		FKBP3	FK506 binding protein 3
27.845.134	-0.3165942		SYT2	synaptotagmin 2
27.843.306	0.4915734		SRP72	signal recognition particle 72
2.783.873	-0.6728452		ZKSCAN1	zinc finger with KRAB and SCAN domains 1
27.833.959		10.120.831	SOX9	SRY-box 9
27.832.809	-0.2497147		SPRR1A	small proline rich protein 1A
27.827.228	-0.1786069		IGHD	immunoglobulin heavy constant delta
2.782.195	0.43946		RNPS1	RNA binding protein with serine rich domain 1
27.815.823	-0.4208249		PSPC1	paraspeckle component 1
27.815.117	0.3847778		CSTF1	cleavage stimulation factor subunit 1
27.802.378	0.8640156		HK2	hexokinase 2
27.789.706	-0.4465016		HBG2///HB	hemoglobin subunit gamma 2///hemoglobin su
27.768.896	0.4291854		SNX13	sorting nexin 13
27.762.804	0.2917666		TMOD3	tropomodulin 3
27.756.878	0.427843		GALNT10	polypeptide N-acetylgalactosaminyltransferase
2.775.426	0.4849785		NOMO3///N	NODAL modulator 3///NODAL modulator 2///NO
27.748.455	0.3338675		DUSP7	dual specificity phosphatase 7
27.739.347	0.5538622		SLC35G2	solute carrier family 35 member G2
27.734.434	0.3831727		P3H1	prolyl 3-hydroxylase 1
2.772.637	0.3055015		CTPS2	CTP synthase 2
27.684.766	0.3600121		FAF2	Fas associated factor family member 2
27.683.305	-0.7084301		C11orf71	chromosome 11 open reading frame 71
27.669.519	-0.5345475		PPFIBP1	PPFIA binding protein 1
27.666.238	0.4438142		GGA3	golgi associated, gamma adaptin ear containing
27.653.155	-0.5490922		SHC2	SHC adaptor protein 2
27.642.187	0.373433		CEP350	centrosomal protein 350
27.620.205	-0.250221		KHSRP	KH-type splicing regulatory protein
2.761.045	0.8834486		SNX7	sorting nexin 7
27.566.244	-0.4384034		SEC62	SEC62 homolog, preprotein translocation facto
27.562.054	0.489946		NCOA3	nuclear receptor coactivator 3
27.561.859	0.7107891		TMEM135	transmembrane protein 135
27.561.828	0.4177316		ZNF45	zinc finger protein 45
27.558.379	-0.2484153			
27.556.363	-0.520819		PPP2R2A	protein phosphatase 2 regulatory subunit Balph
27.555.481	-0.551674		NUDT6	nudix hydrolase 6
27.551.397	-0.354897		SPON1	spondin 1
27.543.257	0.5641189		RASA1	RAS p21 protein activator 1
27.519.371	0.500748		PAPOLA	poly(A) polymerase alpha
27.507.813	-0.4947783		SELE	selectin E
27.505.862	0.2243645		ZNF473	zinc finger protein 473
27.505.652	-0.2702842		RARG	retinoic acid receptor gamma

27.500.994	-0.5247553		CARHSP1	calcium regulated heat stable protein 1
27.492.285	-0.2510433		TIMM8A	translocase of inner mitochondrial membrane 8
2.749.182	-0.6018814		CD69	CD69 molecule
27.490.139	0.3878199		PDCL3P5//	phosducin-like 3 pseudogene 5///phosducin-lik
27.485.254	0.8709657		BIRC3	baculoviral IAP repeat containing 3
27.468.898	0.5261914		DCTN1	dynactin subunit 1
27.459.231	0.4205065		NAGPA	N-acetylglucosamine-1-phosphodiester alpha-
27.442.597	0.3931838		HCCS	holocytochrome c synthase
27.433.112	0.2440099		CCHCR1	coiled-coil alpha-helical rod protein 1
27.428.609	0.3581769		PSMD3	proteasome 26S subunit, non-ATPase 3
2.740.761	0.4241726		PLXND1	plexin D1
27.387.216	-0.2336184		AKAP7	A-kinase anchoring protein 7
27.375.936		-11.666.153	G0S2	G0/G1 switch 2
27.370.692	0.3302838		RWDD2A	RWD domain containing 2A
27.367.521	0.6669861		LGALS8	galectin 8
27.363.175	-0.2628816		C7orf69	chromosome 7 open reading frame 69
27.361.319	0.348296		RABGEF1	RAB guanine nucleotide exchange factor 1
27.361.184	-0.4176886		PDE8A	phosphodiesterase 8A
27.325.395	0.4005477		H3F3B	H3 histone, family 3B
27.315.788	0.483264		MIR21///VM	microRNA 21///vacuole membrane protein 1
27.304.045	0.3516118		CHD1L	chromodomain helicase DNA binding protein 1
2.729.349	0.5899539		KCTD3	potassium channel tetramerization domain cor
27.293.331	0.3661968		NUP98	nucleoporin 98
27.292.856	0.7956986		ARF1	ADP ribosylation factor 1
27.290.036	0.5014986		WTAP	Wilms tumor 1 associated protein
27.259.429	-0.2267897		BMP7	bone morphogenetic protein 7
27.247.709	0.3884035		CTSV	cathepsin V
2.724.667		-1.095.076	SERPINC1	serpin family C member 1
27.236.066	0.2651431		TSSC4	tumor suppressing subtransferable candidate 4
27.223.026	0.450233		DNPH1	2'-deoxynucleoside 5'-phosphate N-hydrolase 1
27.221.108	-0.4441308		NADK	NAD kinase
27.220.601	0.326092		PSMG2	proteasome assembly chaperone 2
27.216.269	0.4291897		GOLPH3	golgi phosphoprotein 3
27.213.902	-0.2237158		LCT	lactase
27.205.656	0.3823922		NXT2	nuclear transport factor 2 like export factor 2
27.193.701	0.2701635		ZNF180	zinc finger protein 180
27.192.962	-0.250811		NRGN	neurogranin
27.184.999	0.4027577		PI4K2A	phosphatidylinositol 4-kinase type 2 alpha
27.184.967	-0.5774503		TRAM2	translocation associated membrane protein 2
27.174.309	-0.3413253		PTK2B	protein tyrosine kinase 2 beta
27.170.851	0.3619409		ERCC3	ERCC excision repair 3, TFIIH core complex heli
27.163.357	-0.4623127		SMPD1	sphingomyelin phosphodiesterase 1
27.163.167	0.4359507		FAM98A	family with sequence similarity 98 member A
27.162.566	0.6166745		SMAD5	SMAD family member 5

27.155.425	-0.5889009		ACOT13	acyl-CoA thioesterase 13
2.714.428	-0.582998		SDHB	succinate dehydrogenase complex iron sulfur s
27.142.595	-0.2157915		PGA4///PG/	pepsinogen 4, group I (pepsinogen A)///pepsinc
27.142.522	0.3966953		BTN2A2	butyrophilin subfamily 2 member A2
27.140.331	-0.3210935		PAOX	polyamine oxidase (exo-N4-amino)
27.132.043		-10.549.258	GJB6///NTN	gap junction protein beta 6///netrin 3///immunc
27.114.715	0.4648208		WBP11	WW domain binding protein 11
2.711.405	0.3488808		CXorf40B//	chromosome X open reading frame 40B///chror
27.101.544	-0.2942577		LAMC2	laminin subunit gamma 2
27.098.615	0.4776181		TRIM31	tripartite motif containing 31
27.094.578	-0.2288051		CELF2	CUGBP, Elav-like family member 2
27.090.485	-0.2844114		NOX1	NADPH oxidase 1
27.085.602	-0.5027426		MREG	melanoregulin
27.057.368	0.4275479		WDR46	WD repeat domain 46
27.043.138	-0.4279842		TNFRSF1A	TNF receptor superfamily member 1A
27.021.889	-0.2672732		MYO1E	myosin IE
27.020.538	0.4189086		YTHDF3	YTH N6-methyladenosine RNA binding protein 3
27.018.175	0.2598837		WDR76	WD repeat domain 76
26.989.682	-0.2202301		GRM7	glutamate metabotropic receptor 7
26.978.819	-0.1699243		COL8A2	collagen type VIII alpha 2 chain
26.978.246	0.5037928		ZFAND1	zinc finger AN1-type containing 1
26.976.492	-0.4494229		FUCA1	fucosidase, alpha-L- 1, tissue
26.973.398	0.3438489		DCLRE1A	DNA cross-link repair 1A
26.970.833	-0.2461799		TRPV6	transient receptor potential cation channel sub
26.969.001	-0.7055094		ABCB11	ATP binding cassette subfamily B member 11
26.967.641	0.2305779		VILL	villin like
26.953.027	0.3060254		RPL24	ribosomal protein L24
26.952.989	-0.2441344		LOC10192!	uncharacterized LOC101929073
26.951.657	-0.2681684		ARVCF	armadillo repeat gene deleted in velocardiofaci
26.950.098	0.3522544		UBTF	upstream binding transcription factor, RNA poly
26.945.415	0.4006398		TMEM5	transmembrane protein 5
26.943.564	0.3244943		CUL7	cullin 7
2.693.672	-0.5713125		ID4	inhibitor of DNA binding 4, HLH protein
26.931.267	0.2432929		RNF2	ring finger protein 2
2.692.305	-0.4694611		ADGRA2	adhesion G protein-coupled receptor A2
26.918.692	-0.2260875		IQSEC2	IQ motif and Sec7 domain 2
26.902.049	0.5701744		SLC6A8	solute carrier family 6 member 8
26.895.665	-0.2293907		FUT6	fucosyltransferase 6
26.892.772	-0.1955586		MELTF	melanotransferrin
26.892.439	-0.6200375			
26.889.721	0.4123959		SS18L2	SS18 like 2
26.881.831	-0.2441088		PRKCH	protein kinase C eta
26.867.609	0.2795594		RPSAP44	ribosomal protein SA pseudogene 44
26.866.385	-0.2885478		MEP1B	meprin A subunit beta

26.850.937	-0.1948141		IVD	isovaleryl-CoA dehydrogenase
26.848.912	0.6176822		HEG1	heart development protein with EGF like domai
26.846.883	-0.4099273		EIF5A	eukaryotic translation initiation factor 5A
26.833.019	0.5156307		LRP12	LDL receptor related protein 12
26.826.978	0.8627267		NUPR1	nuclear protein 1, transcriptional regulator
26.823.689	0.2885699		SBNO1	strawberry notch homolog 1
26.799.563	-0.1804157			
26.783.493		10.299.691	TDGF1P3//.	teratocarcinoma-derived growth factor 1 pseud
26.779.342	-0.37625		N4BP2L2	NEDD4 binding protein 2 like 2
26.778.234	-0.1572996		RNF6	ring finger protein 6
26.775.196	0.5831974		HIF1A	hypoxia inducible factor 1 alpha subunit
26.751.426	-0.489113		REEP5	receptor accessory protein 5
26.734.914	0.2874825		BTN2A1	butyrophilin subfamily 2 member A1
26.730.818	0.2658202		RBBP8///G.	RB binding protein 8, endonuclease///galactoki
26.729.379	0.360383		ATP5C1	ATP synthase, H+ transporting, mitochondrial F
26.726.093	-0.2017871		CWH43	cell wall biogenesis 43 C-terminal homolog
26.722.734	-0.5914495		MAT2A	methionine adenosyltransferase 2A
26.715.523	0.3636329		RANGAP1	Ran GTPase activating protein 1
2.671.463	0.3162528		DCAF7	DDB1 and CUL4 associated factor 7
26.692.661	-0.3272449		STARD7	StAR related lipid transfer domain containing 7
26.692.529	-0.2385964		TRPV4	transient receptor potential cation channel sub
26.685.683	0.5509014		RAI14	retinoic acid induced 14
26.680.828	0.3901799		PRKAR2A	protein kinase cAMP-dependent type II regulato
26.676.277	0.6366318		RB1CC1	RB1 inducible coiled-coil 1
2.667.185	-0.519191		MTHFS	5,10-methenyltetrahydrofolate synthetase (5-fo
2.665.859	0.52353		BRD7	bromodomain containing 7
26.649.425	0.4560968		DIEXF	digestive organ expansion factor homolog (zebr
26.647.443	0.3977656		SLC25A44	solute carrier family 25 member 44
26.629.002	0.4335308		PIGN	phosphatidylinositol glycan anchor biosynthesi
26.624.465	0.6317935		KDELR3	KDEL endoplasmic reticulum protein retention
26.623.591	-0.1996581		NRF1	nuclear respiratory factor 1
26.606.621	0.3699285		UXT	ubiquitously expressed prefoldin like chaperon
26.606.613	-0.1919432		RNF6	ring finger protein 6
26.606.128	0.2186197		ARHGAP19	Rho GTPase activating protein 19
26.595.726	0.366893		RPL4	ribosomal protein L4
26.593.409	-0.3428443		SLC35D1	solute carrier family 35 member D1
26.582.826	-0.7960338		AGTR1	angiotensin II receptor type 1
26.581.699	-0.9134383		CCL2	C-C motif chemokine ligand 2
26.566.745		11.869.142	GOLM1	golgi membrane protein 1
2.656.506	0.3186513		WDR5B	WD repeat domain 5B
26.549.895	-0.3039574		SIX3	SIX homeobox 3
26.542.034	-0.713077		SC5D	sterol-C5-desaturase
26.541.358	-0.2193523		SIGLEC6	sialic acid binding Ig like lectin 6
26.538.233	0.3519512		ATP5C1	ATP synthase, H+ transporting, mitochondrial F

26.531.604	0.5988062		BMP2	bone morphogenetic protein 2
26.521.598	0.5193014		CARM1	coactivator associated arginine methyltransfera
26.518.903	0.4538166		TJP1	tight junction protein 1
26.476	-0.7951744		TGFBR3	transforming growth factor beta receptor 3
26.458.156	0.3751235		MAPT	microtubule associated protein tau
26.453.128	0.820747		NQO1	NAD(P)H quinone dehydrogenase 1
26.450.451		-11.218.142	SERPINE1	serpin family E member 1
26.434.899	0.4245156		ANAPC5	anaphase promoting complex subunit 5
26.425.648	-0.6630384		ORM2///OF	orosomucoid 2///orosomucoid 1
264.206	0.4029069		PPWD1	peptidylprolyl isomerase domain and WD repea
26.415.924	-0.2344582		FUT6	fucosyltransferase 6
26.413.376	-0.5860155		TST	thiosulfate sulfurtransferase
26.412.568		-20.589.954	INS-IGF2///	INS-IGF2 readthrough///insulin like growth fact
26.393.335	-0.4252806		KIAA0040	KIAA0040
26.383.765	-0.3059154		GLT8D2	glycosyltransferase 8 domain containing 2
26.374.977	0.4033872		ZFC3H1	zinc finger C3H1-type containing
26.372.001	-0.2991069		ZNF330	zinc finger protein 330
26.368.695	0.36597		MED8	mediator complex subunit 8
26.367.852	0.2477997		UBC	ubiquitin C
26.328.459	-0.5600864		GCHFR	GTP cyclohydrolase I feedback regulator
26.310.789	0.4405166		NOLC1	nucleolar and coiled-body phosphoprotein 1
26.281.362	0.3232653		ARMC8	armadillo repeat containing 8
26.278.878	0.4006424		RBM15	RNA binding motif protein 15
26.272.293	-0.2271486		IGHG1	immunoglobulin heavy constant gamma 1 (G1r
26.260.288	-0.3026828		KAT8	lysine acetyltransferase 8
26.258.402	0.2912625		COPS2	COP9 signalosome subunit 2
26.258.027	0.4739149		C1GALT1C	C1GALT1 specific chaperone 1
26.247.091	0.7823729		PLOD2	procollagen-lysine,2-oxoglutarate 5-dioxygenas
26.233.536	-0.5324507		GSAP	gamma-secretase activating protein
26.233.334	0.6218152		HTATIP2	HIV-1 Tat interactive protein 2
26.225.571	0.5414854		GNS	glucosamine (N-acetyl)-6-sulfatase
2.622.434	0.4042835			
26.224.162	0.2495109		SLC36A2///	solute carrier family 36 member 2///acyl-CoA sy
26.213.829	0.3764191		BAG6	BCL2 associated athanogene 6
26.191.507	-0.2037819		CACNA1I	calcium voltage-gated channel subunit alpha1
26.174.503	0.4723157		TPP1	tripeptidyl peptidase 1
26.171.908	0.2850408		DNA2	DNA replication helicase/nuclease 2
26.170.942	-0.2732164		APP	amyloid beta precursor protein
26.166.331	0.5431476		PPAT	phosphoribosyl pyrophosphate amidotransfera
26.165.117	0.9282884		EEF1A2	eukaryotic translation elongation factor 1 alpha
26.162.816	0.3392741		ZNF529	zinc finger protein 529
26.156.564	-0.9329554		AMACR	alpha-methylacyl-CoA racemase
2.615.391	0.3832351		POT1	protection of telomeres 1
26.141.845	-0.3482956		DDR2	discoidin domain receptor tyrosine kinase 2

2.613.677	0.357828		WDR1	WD repeat domain 1
26.119.783	0.5100293		CALU	calumenin
26.119.656	0.4117766		FNTA	farnesyltransferase, CAAX box, alpha
26.107.498	-0.3207941		IGHG1///IG	immunoglobulin heavy constant gamma 1 (G1r
2.608.755	0.584881		RHOQ	ras homolog family member Q
26.076.184	0.4578933		SLF2	SMC5-SMC6 complex localization factor 2
26.074.506	-0.3671941		SEC14L4	SEC14 like lipid binding 4
26.064.924	0.3472455		TM9SF3	transmembrane 9 superfamily member 3
26.061.674	-0.4102794		AVIL	advillin
26.054.726	0.5049085		EFR3A	EFR3 homolog A
26.052.716	0.3797737		SEPT2	septin 2
26.035.181	-0.5420017		APOL6	apolipoprotein L6
26.034.854	0.5791187		SYNGR1	synaptogyrin 1
26.031.704	0.4295836		EDEM2	ER degradation enhancing alpha-mannosidase
26.027.918	0.5424001			
25.997.835	-0.2207892		DPEP2	dipeptidase 2
25.993.413	-0.3770888		AKT1	AKT serine/threonine kinase 1
25.991.576	-0.6174333		FAM46A	family with sequence similarity 46 member A
25.977.781	-0.2958939		LOC55338	uncharacterized LOC55338
25.974.762	0.3932919		MAPK1IP1L	mitogen-activated protein kinase 1 interacting
25.968.774	-0.3754023		MDM2	MDM2 proto-oncogene
25.960.955		10.833.156	ME1	malic enzyme 1
2.595.895	0.3497425		PPP1R12A	protein phosphatase 1 regulatory subunit 12A
25.952.986	0.6542791		MBNL3	muscleblind like splicing regulator 3
25.941.347	0.5008584		CSNK1A1	casein kinase 1 alpha 1
25.935.325	-0.241882		ADRA1B	adrenoceptor alpha 1B
25.933.571	0.519898		WRB	tryptophan rich basic protein
25.931.739	0.5574955		STX7	syntaxin 7
25.929.486	0.3826659		MAPK14	mitogen-activated protein kinase 14
25.925.476	0.3386882		CSPP1	centrosome and spindle pole associated protei
25.922.679	-0.357817		FYB	FYN binding protein
25.918.564	0.4154689		PI4KB	phosphatidylinositol 4-kinase beta
25.911.507	0.4496835		GNAS///FG	GNAS complex locus///fibrinogen beta chain
25.907.636	0.4478997		RPL29	ribosomal protein L29
25.903.614	0.508111		COL21A1	collagen type XXI alpha 1 chain
25.860.643	0.3450519		MTF2	metal response element binding transcription f
2.585.839	0.5962495		RHOQ	ras homolog family member Q
25.854.623	0.6019978		MARCKS	myristoylated alanine rich protein kinase C sub
2.584.862	0.3466733		WBP4	WW domain binding protein 4
25.843.226	-0.7825961		SCG5	secretogranin V
25.831.593	0.3295894		TMF1	TATA element modulatory factor 1
25.829.632	0.3646498		CUL7	cullin 7
25.827.855	0.4070477		FEM1B	fem-1 homolog B
2.580.731	0.7899667		HMGCS1	3-hydroxy-3-methylglutaryl-CoA synthase 1

25.791.131	0.4425166		CPSF1	cleavage and polyadenylation specific factor 1
25.791.035	0.405802		HEATR3	HEAT repeat containing 3
25.782.923	0.5241625		SF3A2	splicing factor 3a subunit 2
25.780.999	-0.4512731		TRIM15	tripartite motif containing 15
25.780.816	0.3764288		IP6K2	inositol hexakisphosphate kinase 2
25.780.189	-0.9172207		SYBU	syntabulin
25.769.079	-0.2068672		ALDOAP2	aldolase, fructose-bisphosphate A pseudogene
25.758.268	0.6815828		HIST2H2BE	histone cluster 2, H2be
25.730.075	-0.2762641		HSPB6	heat shock protein family B (small) member 6
25.730.002	-0.2164485		SLC9A2	solute carrier family 9 member A2
25.719.698		-13.925.473	SAA1	serum amyloid A1
25.704.915	0.4584692		CXorf40B	chromosome X open reading frame 40B
25.686.926	-0.1871084		KSR1	kinase suppressor of ras 1
25.683.182	-0.5188096		RIOK3	RIO kinase 3
2.568.174	0.5016603		GALNT1	polypeptide N-acetylgalactosaminyltransferase
25.670.045	-0.1749128		INSL4	insulin like 4
25.668.847	0.3179468		MYL12BP1/	myosin light chain 12B pseudogene 1///myosin
25.664.196	0.4787505		RASA1	RAS p21 protein activator 1
25.661.886	0.3174349		CEP290	centrosomal protein 290
2.566.066	-0.4366878		LRP5L	LDL receptor related protein 5 like
25.648.585	0.4629022		FBXO9	F-box protein 9
25.647.291	0.4441749		OCRL	OCRL, inositol polyphosphate-5-phosphatase
25.644.205	0.4719692		SET	SET nuclear proto-oncogene
2.563.902	0.3737441		NDUFS2	NADH:ubiquinone oxidoreductase core subunit
25.627.757	0.4104906		BAG6	BCL2 associated athanogene 6
25.614.897	-0.1755863		SCAND2P	SCAN domain containing 2 pseudogene
25.610.573	0.4585495		CYB5B	cytochrome b5 type B
25.608.072	0.4633207		ASXL2	additional sex combs like 2, transcriptional regu
25.598.889	0.4552582		NUDT15	nudix hydrolase 15
25.583.471	-0.2916813		ENGASE	endo-beta-N-acetylglucosaminidase
2.557.984	0.2782482		CLN6	ceroid-lipofuscinosis, neuronal 6, late infantile,
25.578.273	0.5962034		HSPA2	heat shock protein family A (Hsp70) member 2
25.577.918	0.5763484		NCOA3	nuclear receptor coactivator 3
25.560.056	-0.1818964		IRF4	interferon regulatory factor 4
25.553	0.5347714		ARL4A	ADP ribosylation factor like GTPase 4A
25.551.948	0.8594792		SMC3	structural maintenance of chromosomes 3
25.546.785	0.3386569		TIMM8B	translocase of inner mitochondrial membrane 8
25.542.695	-0.1801401		KIF1A	kinesin family member 1A
25.511.246	-0.2007283		IL23A	interleukin 23 subunit alpha
25.510.633	-0.2066065		ATP10B	ATPase phospholipid transporting 10B (putative
25.508.632	0.2893486		KLC1	kinesin light chain 1
2.550.621	-0.4019284		SCD5	stearoyl-CoA desaturase 5
25.506.181	0.3734239		ATP2C1	ATPase secretory pathway Ca2+ transporting 1
25.482.198	-0.4813856		PPIF	peptidylprolyl isomerase F

2.547.818	0.4170251	PIK3C2A	phosphatidylinositol-4-phosphate 3-kinase cat
25.475.496	0.4540535	BCL10	B-cell CLL/lymphoma 10
2.546.014	-0.6130548	RGL1	ral guanine nucleotide dissociation stimulator l
25.428.653	-0.2331735	ALOX12P2	arachidonate 12-lipoxygenase pseudogene 2
25.422.592	-0.3552727	NOL4	nucleolar protein 4
25.417.742	-0.3009333	IL11	interleukin 11
25.408.337	0.3252046	RAB13	RAB13, member RAS oncogene family
25.403.449	-0.2599952	NOX5	NADPH oxidase, EF-hand calcium binding doma
25.400.644	0.1974258	CINP	cyclin dependent kinase 2 interacting protein
25.382.304	-0.3736311	THBD	thrombomodulin
25.379.084	-0.5372195	AHI1	Abelson helper integration site 1
25.371.543	0.3346054	TAOK2	TAO kinase 2
25.349.164	0.4457417	NOP16	NOP16 nucleolar protein
25.345.662	-0.5123208	STOM	stomatin
25.338.223	-0.4014795	SLC7A8	solute carrier family 7 member 8
25.330.365	0.3916317	XRCC5	X-ray repair cross complementing 5
2.532.134	0.3047083	SF3A1	splicing factor 3a subunit 1
25.314.918	-0.9076702	LIPE	lipase E, hormone sensitive type
25.314.784	0.3570654	CREBZF	CREB/ATF bZIP transcription factor
25.310.744	-0.2215644	PHLDB1	pleckstrin homology like domain family B meml
25.301.969	-0.2433991	PPP4R3A	protein phosphatase 4 regulatory subunit 3A
25.293.727	0.4140865	MTMR6	myotubularin related protein 6
25.286.848	-0.1723286	CHRM5	cholinergic receptor muscarinic 5
25.286.358	0.3570863	FBXO46	F-box protein 46
25.286.259	0.3723207	RBM22	RNA binding motif protein 22
25.285.798	-0.3324653	CACNA1H	calcium voltage-gated channel subunit alpha1
25.285.646	-0.2518341	SC5D	sterol-C5-desaturase
25.278.415	0.5173003	UBE2J1	ubiquitin conjugating enzyme E2 J1
25.271.842	-0.2552718	CD163	CD163 molecule
25.266.038	0.3645131	FASTKD2	FAST kinase domains 2
25.255.525	0.8546174	SCD	stearoyl-CoA desaturase
25.253.946	-0.5939244	HES1	hes family bHLH transcription factor 1
25.253.333	-0.8647385	IGKV1D-13	immunoglobulin kappa variable 1D-13
25.251.122	0.66577	ATP2B4	ATPase plasma membrane Ca2+ transporting 4
25.249.638	0.5021346	TSTA3	tissue specific transplantation antigen P35B
25.241.386	0.5271173	TSPAN3	tetraspanin 3
25.236.906	-0.2691972	EIF1	eukaryotic translation initiation factor 1
25.220.371	0.4158496	PARD3	par-3 family cell polarity regulator
2.521.654	-0.187444	APAF1	apoptotic peptidase activating factor 1
25.210.725	-0.2538678	CEACAM21	carcinoembryonic antigen related cell adhesior
2.519.972	0.5400542	PKP4	plakophilin 4
2.519.471	0.2589713	ZNF219	zinc finger protein 219
2.517.443	0.3697948	VEZF1	vascular endothelial zinc finger 1
25.169.678	0.3840847	U2AF1	U2 small nuclear RNA auxiliary factor 1

25.167.622	-0.2504994	ZCWPW1	zinc finger CW-type and PWWP domain contain
25.159.591	0.2651849	HEATR6	HEAT repeat containing 6
25.151.821	-0.4629377	WWOX	WW domain containing oxidoreductase
25.137.164	-0.6101593	MAD1L1	MAD1 mitotic arrest deficient like 1
25.136.756	0.2640673	KNOP1	lysine rich nucleolar protein 1
25.124.525	0.3042532	MPZL2	myelin protein zero like 2
25.122.679	0.3186778	CEP76	centrosomal protein 76
25.118.036	0.4828557	TXN	thioredoxin
25.103.917	0.3475777	POLR2I///R	RNA polymerase II subunit I///ribosomal protein
25.098.602	-0.3385985	GZMH	granzyme H
25.079.717	0.4172414	CREB3	cAMP responsive element binding protein 3
25.079.428	-0.7980383	EGFR	epidermal growth factor receptor
25.069.679	0.2272193	TBC1D19	TBC1 domain family member 19
25.068.054	0.5066974	SLC6A10PE	solute carrier family 6 member 8 pseudogene//.
25.065.554	0.2049303	SPAST	spastin
25.059.227	-0.4826482	APLNR	apelin receptor
2.503.707	-0.4097792	TRIM15	tripartite motif containing 15
2.503.209	0.4027006	FBXW2	F-box and WD repeat domain containing 2
25.026.567	0.3242557	PPM1F	protein phosphatase, Mg2+/Mn2+ dependent 1
25.025.896	-0.5058043	POLH	DNA polymerase eta
25.014.577	0.4406677	ARF6	ADP ribosylation factor 6
24.999.624	-0.5849543	IL6ST	interleukin 6 signal transducer
24.999.215	0.4655436	TRIM68	tripartite motif containing 68
24.994.276	-0.6262144	RAPGEF2	Rap guanine nucleotide exchange factor 2
24.989.458	0.2483488	GCOM2///C	GRINL1B complex locus 2 (pseudogene)///GRIN
24.986.775	-0.219394		
24.972.596	-0.2866038	C21orf91	chromosome 21 open reading frame 91
24.971.178	-0.3961605	MTMR9	myotubularin related protein 9
24.964.629	0.3914122	ARIH2	ariadne RBR E3 ubiquitin protein ligase 2
24.960.009	0.34252	ZW10	zw10 kinetochore protein
24.955.833	0.4314125	DNAAF2	dynein axonemal assembly factor 2
24.950.838	0.4120978	RFK	riboflavin kinase
24.946.596	0.4483969	SFI1	SFI1 centrin binding protein
24.945.119	0.2800414	HDGF	hepatoma-derived growth factor
24.929.332	0.4427449	ZNHIT6	zinc finger HIT-type containing 6
24.926.829	-0.3903208	GPR171	G protein-coupled receptor 171
24.926.086	-0.7033137	SEPT4	septin 4
24.922.649	0.2586695	POLA2	DNA polymerase alpha 2, accessory subunit
24.918.093	-0.1926267	MAP7D3	MAP7 domain containing 3
24.915.708	0.4090384	POT1	protection of telomeres 1
24.915.303	0.3890016	BAG6	BCL2 associated athanogene 6
24.904.988	0.3675528	PAFAH1B1	platelet activating factor acetylhydrolase 1b reg
24.902.561	0.3806217	QRSL1	glutaminyl-tRNA synthase (glutamine-hydrolyzi
24.898.529	-0.2044356		

24.893.391	0.3379041		EXT2	exostosin glycosyltransferase 2
24.887.866	-0.2267567		CYLC1	cylicin 1
24.884.261	-0.2660829		PAK5	p21 (RAC1) activated kinase 5
24.857.712	0.3275522		SF3B1	splicing factor 3b subunit 1
24.832.956	0.4115985		CEP192	centrosomal protein 192
24.831.835	0.6246104		ACTR2	ARP2 actin related protein 2 homolog
24.826.591	-0.2916218		TRAF3IP3	TRAF3 interacting protein 3
24.826.556		-10.787.659	FGFR2	fibroblast growth factor receptor 2
24.785.421	0.342372		ZC3H11A	zinc finger CCCH-type containing 11A
24.777.976	-0.2088937		SEMA6D	semaphorin 6D
24.777.968	0.5101684		ACTG1	actin gamma 1
24.777.131	-0.8296643		SERPINA5	serpin family A member 5
24.768.003	0.4797934		DCTN4	dynactin subunit 4
24.756.732	0.4993871		PAPOLA	poly(A) polymerase alpha
24.756.051	0.4146738		ZZZ3	zinc finger ZZ-type containing 3
24.750.054	0.2558459		SBF1	SET binding factor 1
24.734.017	0.2793463		CSPG5	chondroitin sulfate proteoglycan 5
24.729.088	-0.1986794		PRKAR1B	protein kinase cAMP-dependent type I regulator
24.727.152	-0.7576825		GATM	glycine amidinotransferase
24.726.417	-0.3802551		XPNPEP3	X-prolyl aminopeptidase 3
2.472.508	-0.2012435		CCKBR	cholecystokinin B receptor
24.716.953	0.4068004		PAGR1	PAXIP1 associated glutamate rich protein 1
24.708.031	0.3883452		EMC7	ER membrane protein complex subunit 7
2.469.704	-0.1886857		VIPR2	vasoactive intestinal peptide receptor 2
24.696.494	-0.1934229		CEACAM7	carcinoembryonic antigen related cell adhesion
24.692.673	-0.6849646		CTSB	cathepsin B
24.683.247	0.3621354		BECN1	beclin 1
24.680.257	-0.2452696		SYDE1	synapse defective Rho GTPase homolog 1
24.678.141	-0.5196142		LCP2	lymphocyte cytosolic protein 2
24.677.782	-0.5859061		ZCWPW2//.	zinc finger CW-type and PWWP domain contain
24.662.764	-0.4050936		MRPL57	mitochondrial ribosomal protein L57
24.643.391	0.458629		ERBIN	erbb2 interacting protein
24.638.096	0.3876849		GIPC1	GIPC PDZ domain containing family member 1
24.637.534	0.4818474		PREB	prolactin regulatory element binding
24.630.654	-0.381504		SH3PXD2A	SH3 and PX domains 2A
24.621.654	0.3226337		CSNK1G2	casein kinase 1 gamma 2
24.616.519	-0.8210896		GBP1	guanylate binding protein 1
24.615.865	0.2102971		SIRT4	sirtuin 4
24.613.901	-0.1422351		MFAP3L	microfibrillar associated protein 3 like
24.599.396	0.5288058		GUK1	guanylate kinase 1
24.591.129	0.4128578		NOL7	nucleolar protein 7
24.584.613	0.3884563		DYNC2LI1	dynein cytoplasmic 2 light intermediate chain 1
24.579.312	0.550503		APH1A	aph-1 homolog A, gamma-secretase subunit
24.575.864	0.4389935		UBFD1	ubiquitin family domain containing 1

24.570.522	0.2801259		RBM15B	RNA binding motif protein 15B
24.568.043	-0.1746741		SGCD	sarcoglycan delta
24.531.354	0.4558744		CCNG2	cyclin G2
24.529.239	0.3684679		HDAC5	histone deacetylase 5
24.527.464	0.2361436		NEK2	NIMA related kinase 2
2.452.547	-0.2517423		PTGES	prostaglandin E synthase
24.525.049	0.8694746		SULF1	sulfatase 1
24.515.202	-0.2196805			
24.513.765	0.2896302		WIPI2	WD repeat domain, phosphoinositide interactir
24.507.388	0.3696367		PKM	pyruvate kinase, muscle
2.450.558	0.3903018		CLTB	clathrin light chain B
24.478.971	0.2817364		PHF20L1	PHD finger protein 20-like 1
24.474.601	0.2723324		THOC6	THO complex 6
2.446.971		10.471.918	PCOLCE2	procollagen C-endopeptidase enhancer 2
24.465.419	0.385533		PSMA5	proteasome subunit alpha 5
24.438.575	0.3052758		MUS81	MUS81 structure-specific endonuclease subun
24.433.058	0.6282901		SRSF1	serine and arginine rich splicing factor 1
24.427.472	0.2735975		CELSR2	cadherin EGF LAG seven-pass G-type receptor :
24.418.883	0.2437566		RHBDD3	rhomboid domain containing 3
24.417.916	0.4656352		TPP1	tripeptidyl peptidase 1
24.416.417	-0.2370965		ZAP70	zeta chain of T cell receptor associated protein
2.441.139	-0.7306818		CCL5	C-C motif chemokine ligand 5
24.408.043	0.4904221		AP2B1	adaptor related protein complex 2 beta 1 subur
24.402.376	0.4646431		MANSC1	MANSC domain containing 1
24.387.952	0.6318801		RNF14	ring finger protein 14
24.382.053	0.4661434		PPP1R7	protein phosphatase 1 regulatory subunit 7
24.370.964	0.392012		NSFL1C	NSFL1 cofactor
24.357.003	0.3564619		RHOT1	ras homolog family member T1
24.352.415	-0.4075897		DIXDC1	DIX domain containing 1
24.351.607	0.4444913		EMC1	ER membrane protein complex subunit 1
24.350.439	-0.2148762		ZNF44	zinc finger protein 44
24.320.421	0.3407099		ZNF3	zinc finger protein 3
24.316.223	0.4065335		TRAPPC13	trafficking protein particle complex 13
2.431.212	0.3799642		MPHOSPH9	M-phase phosphoprotein 9
24.304.907	-0.2245183		SOX5	SRY-box 5
24.302.023	-0.2137239		SCD5	stearoyl-CoA desaturase 5
24.279.442	-0.2038897		PDE11A	phosphodiesterase 11A
24.271.433	-0.2276277		KCNH6	potassium voltage-gated channel subfamily H r
2.426.895	-0.2443616		HNRNPM	heterogeneous nuclear ribonucleoprotein M
24.268.279	0.4799235		CUL4B	cullin 4B
2.426.378		-12.086.971	UGT1A3///U	UDP glucuronosyltransferase family 1 member
24.263.468	-0.3333392			
24.258.662	-0.6038821		SDC1	syndecan 1
24.251.066	0.4113498		UTP14A///U	UTP14A small subunit processome component

24.248.172	0.4868296	HSPA9	heat shock protein family A (Hsp70) member 9
24.248.135	0.4889031	NR5A2	nuclear receptor subfamily 5 group A member 2
24.244.349	-0.219016	POU6F1	POU class 6 homeobox 1
2.424.193	0.5669226	PPIC	peptidylprolyl isomerase C
24.238.719	-0.2243212	VPS13D	vacuolar protein sorting 13 homolog D
24.234.634	0.2239387	TRIM45	tripartite motif containing 45
24.234.066	-0.2331204	KIR2DL5A	killer cell immunoglobulin like receptor, two Ig c
24.229.988	0.4566316	KLC1	kinesin light chain 1
24.217.675	0.7672052	LAMA3	laminin subunit alpha 3
24.196.337	0.5852998	PAICS	phosphoribosylaminoimidazole carboxylase; pl
24.192.889	-0.4669895	PTGS2	prostaglandin-endoperoxide synthase 2
24.191.907	0.2911954	B4GALT7	beta-1,4-galactosyltransferase 7
2.418.863	0.6912029	FGF13	fibroblast growth factor 13
24.184.047	-0.4300545	CDK10	cyclin dependent kinase 10
24.179.662	0.3912289	BTF3	basic transcription factor 3
24.177.672	-0.4637104	FBXO21	F-box protein 21
24.176.453	-0.2663488	PPBP	pro-platelet basic protein
24.168.266	0.285382	WDR82	WD repeat domain 82
24.162.824	-0.278023	DOPEY1	dopey family member 1
24.150.228	-0.306732	PELI2	pellino E3 ubiquitin protein ligase family memb
24.142.206	-0.3017602	AOC4P	amine oxidase, copper containing 4, pseudoger
24.123.504	-0.6063095	MBNL2	muscleblind like splicing regulator 2
24.096.234	0.4922346	RSL24D1	ribosomal L24 domain containing 1
24.093.638	0.4970842	NDUFS4	NADH:ubiquinone oxidoreductase subunit S4
24.084.224	-0.2506538	CRYGD	crystallin gamma D
24.082.315	0.3962564	SLC2A6	solute carrier family 2 member 6
2.407.171	-0.9342623	AQP1	aquaporin 1 (Colton blood group)
24.070.991	0.3549994	CIZ1	CDKN1A interacting zinc finger protein 1
24.070.548	0.2493309	ERCC6L	ERCC excision repair 6 like, spindle assembly c
24.063.009	-0.3347445	DMP1	dentin matrix acidic phosphoprotein 1
24.057.778	0.5419534	LOC72821(	hypothetical LOC728210
2.404.853	0.3750673	CHPF2	chondroitin polymerizing factor 2
24.042.748	0.6862016	PRRC1	proline rich coiled-coil 1
24.036.849	-0.3931561	DNAJC22	DnaJ heat shock protein family (Hsp40) membe
24.024.883	0.3177902	UBAP2L	ubiquitin associated protein 2 like
24.024.857	-0.4612726	THYN1	thymocyte nuclear protein 1
24.016.126	0.3769703	LIMK2	LIM domain kinase 2
2.401.561	0.3511838	TMEM109	transmembrane protein 109
24.008.841	-0.396239	MAGI1	membrane associated guanylate kinase, WW a
24.008.401	-0.2039597	FLRT2	fibronectin leucine rich transmembrane proteir
23.997.322	-0.775539	TSPAN12	tetraspanin 12
23.994.206	0.6121203	ANXA9	annexin A9
23.979.551	0.263621	PLK4	polo like kinase 4
23.972.291	0.4231564	RCC1	regulator of chromosome condensation 1

23.968.124	0.5594175		RAB4A	RAB4A, member RAS oncogene family
23.949.132	-0.2964768		SEC24D	SEC24 homolog D, COPII coat complex compor
23.946.701	0.4474767		BCAS2	breast carcinoma amplified sequence 2
23.922.748	0.2824209		NOL6	nucleolar protein 6
2.392.211	-0.2189386		NEBL	nebulette
23.919.178	0.4487041		METTL9	methyltransferase like 9
23.913	-0.6793938		FBXO21	F-box protein 21
2.390.994	0.3557603		TBC1D5	TBC1 domain family member 5
23.906.156	-0.2759077		SPI1	Spi-1 proto-oncogene
23.895.233	-0.1915009		MYO1E	myosin IE
23.881.573	-0.1868303		NAG18	NAG18 mRNA
23.873.913	-0.5808056		EDNRB	endothelin receptor type B
23.870.375	0.3433901		EOGT	EGF domain specific O-linked N-acetylglucosar
23.859.563	0.2513906		CSTF1	cleavage stimulation factor subunit 1
23.831.903		11.125.461	SSX1	SSX family member 1
23.822.431	-0.4468776		RUFY2	RUN and FYVE domain containing 2
23.813.864	0.3828211		PITRM1	pitrilysin metallopeptidase 1
23.810.053	-0.5632716		CLMN	calmin
23.798.334	-0.2917058		FXR2	FMR1 autosomal homolog 2
2.379.741	0.3676078		TXNDC5	thioredoxin domain containing 5
23.794.113	0.4190304		DUSP22	dual specificity phosphatase 22
2.379.337	-0.3208417		LRRFIP1	LRR binding FLII interacting protein 1
2.379.108	-0.1982593		FOXG1	forkhead box G1
23.785.102	-0.5475796		TFPI2	tissue factor pathway inhibitor 2
23.775.187	0.3891874		MARCH3	membrane associated ring-CH-type finger 3
23.763.167	0.4217412		GNAS///FG	GNAS complex locus///fibrinogen beta chain
23.755.082	-0.2428812		AP4S1	adaptor related protein complex 4 sigma 1 subu
2.374.682	0.2453432		MAPKAPK5-	MAPKAPK5 antisense RNA 1
23.736.183	0.3215269		GLRX3	glutaredoxin 3
2.373.172	0.3808214		FEM1B	fem-1 homolog B
23.730.734	0.3684842		DLEU2L///D	deleted in lymphocytic leukemia 2-like///delete
23.709.202	0.3683342		STRAP	serine/threonine kinase receptor associated pr
23.700.396	-0.821474		PCYOX1	prenylcysteine oxidase 1
23.696.818	-0.7687999		SERPINA3	serpin family A member 3
23.696.287	-0.2469014		LY9	lymphocyte antigen 9
23.689.254	-0.7023914		SLC37A4	solute carrier family 37 member 4
23.687.216	-0.2070506		CTNNA3	catenin alpha 3
23.684.528	0.3264092			
2.368.386	0.4246813		INTS3	integrator complex subunit 3
23.672.869	0.3951436		MFSD10	major facilitator superfamily domain containing
23.660.394	-0.3144694		MITF	melanogenesis associated transcription factor
23.658.395	-0.4806071		CALD1	caldesmon 1
23.652.794	0.4840918		PPIC	peptidylprolyl isomerase C
23.648.818	0.3790026		POLR2L	RNA polymerase II subunit L

23.644.336	-0.1941064			
23.636.169	-0.5141198		TPD52L1	tumor protein D52-like 1
23.633.934	0.4611823		TCP1	t-complex 1
2.361.482	0.2629003		RPS28P7//	ribosomal protein S28 pseudogene 7///ribosom
2.361.441	0.2375596		KBTBD4	kelch repeat and BTB domain containing 4
23.611.145	0.3881259		ERI3	ERI1 exoribonuclease family member 3
23.610.039	0.3124292		LOC73097	similar to ribosomal protein S10///ribosomal pr
23.602.662	-0.2169794		OSBPL7	oxysterol binding protein like 7
23.595.863	-0.5235561		DERA	deoxyribose-phosphate aldolase
23.591.029	-0.4955502		ADIPOR2	adiponectin receptor 2
23.589.952	0.4449557		GNAS	GNAS complex locus
23.585.632	0.6341331		MAP1B	microtubule associated protein 1B
2.357.931	0.4734599		TRAPPC3	trafficking protein particle complex 3
2.357.613	-0.7695564		KLF6	Kruppel like factor 6
23.575.617	0.4142945		HNRNPUL1	heterogeneous nuclear ribonucleoprotein U like
23.574.938	0.3119604		TREM2	triggering receptor expressed on myeloid cells
23.573.119	0.4779771		ALCAM	activated leukocyte cell adhesion molecule
23.572.328	0.5345047		THOC2	THO complex 2
23.568.906	-0.4876748		THBS1	thrombospondin 1
23.560.739	0.4194325		S100PBP	S100P binding protein
2.355.156	0.5748538		SKAP2	src kinase associated phosphoprotein 2
23.544.165	0.3590358		UVRAG	UV radiation resistance associated
23.543.977	-0.1954925		MAPRE3	microtubule associated protein RP/EB family m
23.539.476	-0.1721115		PTGDR	prostaglandin D2 receptor
23.527.865		-10.145.986	FST	follistatin
23.523.617	-0.1651554		EPM2A	epilepsy, progressive myoclonus type 2A, Lafor
23.523.588	0.5375319		DOCK9	dedicator of cytokinesis 9
2.352.253	-0.1963436		CACNG1	calcium voltage-gated channel auxiliary subuni
23.518.364	0.7400951		UPK3A	uroplakin 3A
23.502.834	-0.2896594		ATP1B2	ATPase Na+/K+ transporting subunit beta 2
23.499.841	-0.3068118		ARHGEF17	Rho guanine nucleotide exchange factor 17
23.468.688	-0.602987		HPS5	HPS5, biogenesis of lysosomal organelles comp
23.463.554	-0.4546301		BCKDK	branched chain ketoacid dehydrogenase kinase
23.463.128	0.4650623		TM9SF1	transmembrane 9 superfamily member 1
23.460.698	0.5635391		ABCE1	ATP binding cassette subfamily E member 1
23.448.546	-0.4443582		ARHGEF10	Rho guanine nucleotide exchange factor 10 like
23.419.053	0.3652481		FLOT2	flotillin 2
23.413.365		-12.878.078	HPGD	hydroxyprostaglandin dehydrogenase 15-(NAD)
23.397.396	-0.3358431		CPZ	carboxypeptidase Z
2.339.412	0.2840765		NFYA	nuclear transcription factor Y subunit alpha
23.392.368	0.2939406		DCTN1	dynactin subunit 1
23.392.058	0.3978097		CHMP2A	charged multivesicular body protein 2A
23.387.834	0.5064107		LEPROTL1	leptin receptor overlapping transcript-like 1
23.371.338	-0.3333361		TACR1	tachykinin receptor 1

23.370.382	0.5382595		TMED7	transmembrane p24 trafficking protein 7
23.370.154	0.4268926		GNAS///FG	GNAS complex locus///fibrinogen beta chain
23.363.605	0.4583417		RPF1	ribosome production factor 1 homolog
2.335.466	-0.2939326		KCNMA1	potassium calcium-activated channel subfamil
23.347.537	-0.231699		DNAJA4	DnaJ heat shock protein family (Hsp40) membe
2.333.666	0.3849232		LRPAP1	LDL receptor related protein associated protein
23.327.102	-0.1987422		CD226	CD226 molecule
23.323.686	-0.2310581			
23.319.672	-0.2415311		NFATC1	nuclear factor of activated T-cells 1
23.307.094	-0.8500287		IGKV1OR2-	immunoglobulin kappa variable 1/OR2-108 (no
2.330.543	0.305191		RAB8B	RAB8B, member RAS oncogene family
23.302.166	-0.2383986		ADGRD2	adhesion G protein-coupled receptor D2
23.300.668	-0.2418168		SIT1	signaling threshold regulating transmembrane a
2.329.699	0.3181311		ZNF16	zinc finger protein 16
23.293.179	0.3332383		PIP4K2B	phosphatidylinositol-5-phosphate 4-kinase typ
23.288.169	0.2992968		METTL3	methyltransferase like 3
2.327.473	-0.3059048		MCM3AP	minichromosome maintenance complex comp
23.271.376	0.7506495		HIST2H2AA	histone cluster 2, H2aa4///histone cluster 2, H2
23.270.475	-0.2297787		EVC	EvC ciliary complex subunit 1
23.258.799	-0.6428404		PLXNB1	plexin B1
23.251.068	0.2676234		CIZ1	CDKN1A interacting zinc finger protein 1
23.221.028	-0.2016279		HR44	Hr44 antigen
23.215.438	0.5109367		ITGAE	integrin subunit alpha E
23.214.025	0.2049928		DUS4L	dihydrouridine synthase 4 like
23.203.905	-0.2087693			
23.203.416	-0.2027484		RBPJL	recombination signal binding protein for immur
23.201.027	0.5731201		MYDGF	myeloid derived growth factor
2.319.571	-0.2093988		RCAN1	regulator of calcineurin 1
23.187.322	-0.6073229		BLVRB	biliverdin reductase B
23.185.708	-0.3045238		VPS13D	vacuolar protein sorting 13 homolog D
23.181.595	0.3016003		POLQ	DNA polymerase theta
23.170.888	-0.1589423		RS1	retinoschisin 1
23.162.294	0.2782931		AGK	acylglycerol kinase
23.158.116	-0.2136336		MYO15A	myosin XVA
23.147.017	0.5204255		STC2	stanniocalcin 2
23.146.302	0.4230341		COPB2	coatomer protein complex subunit beta 2
23.145.122		-10.141.428	FABP1	fatty acid binding protein 1
23.142.639	-0.5678694		KYNU	kynureninase
23.133.232	-0.201537			
23.129.183	0.7002604		ADGRG2	adhesion G protein-coupled receptor G2
23.114.911	0.3633079		BAG5	BCL2 associated athanogene 5
23.098.554	-0.2055353		FLI1	Fli-1 proto-oncogene, ETS transcription factor
23.098.278	-0.3469706		LLGL1	LLGL1, scribble cell polarity complex compone
23.096.803	0.2675529		NUP160	nucleoporin 160

23.086.517	0.4956482		USP11	ubiquitin specific peptidase 11
23.085.885	-0.9212308		FOLR1	folate receptor 1
23.084.801	-0.3962323			
23.082.606	0.3499461		ZNF551	zinc finger protein 551
23.074.511	0.2171452			
23.073.119	-0.1772178		SLC5A7	solute carrier family 5 member 7
23.065.318	-0.1999388		LRRC19	leucine rich repeat containing 19
23.054.713	0.3150138		HKDC1	hexokinase domain containing 1
23.048.917	-0.3169992		NFASC	neurofascin
23.047.255	0.3154025		CIZ1	CDKN1A interacting zinc finger protein 1
23.045.532	0.3313284		CCNE2	cyclin E2
23.043.288	-0.2191129		HCRP1	hepatocellular carcinoma-related HCRP1
23.040.964		-11.001.517	GSTM2	glutathione S-transferase mu 2 (muscle)
23.033.302	-0.3275328		OAZ2	ornithine decarboxylase antizyme 2
23.027.949	0.3154438			
23.023.767	0.7027075		ARG2	arginase 2
23.019.142	0.6927489		IMPA2	inositol monophosphatase 2
23.018.657	-0.5517715		UBL3	ubiquitin like 3
22.993.594	-0.2952266		SFTPB	surfactant protein B
22.989.574	-0.6984928		TRBC1///TF	T cell receptor beta constant 1///T cell receptor
22.989.218	-0.7459324		PBX1	PBX homeobox 1
22.972.967	-0.248523		PBRM1	polybromo 1
22.957.725	-0.8877828		EHBP1	EH domain binding protein 1
22.956.578	0.4613874		PEX19	peroxisomal biogenesis factor 19
22.950.258	-0.2142409			
22.946.876	0.3268309		OSGEPL1	O-sialoglycoprotein endopeptidase-like 1
22.939.858	-0.1627404		N4BP2L2	NEDD4 binding protein 2 like 2
22.939.749	-0.2437429			
22.938.707	0.2691607		WRAP73	WD repeat containing, antisense to TP73
22.931.499	0.2754817		COX15	COX15, cytochrome c oxidase assembly homol
22.928.067	-0.2519451		FEZ1	fasciculation and elongation protein zeta 1
22.919.177	-0.2157347		PTGER3	prostaglandin E receptor 3
22.916.328	0.3690379		PLAG1	PLAG1 zinc finger
22.899.717	0.4190803		UBXN7	UBX domain protein 7
22.885.641	-0.3315138		TAF1C	TATA-box binding protein associated factor, RN
2.288.254	0.3464289		THAP1	THAP domain containing 1
22.876.067	-0.2347768		ABCC8	ATP binding cassette subfamily C member 8
22.872.328	0.3042013		ADD1	adducin 1
22.867.797	-0.3156029		A4GALT	alpha 1,4-galactosyltransferase
22.862.147	-0.216989		ACTL6B	actin like 6B
22.861.611	0.525413			
22.857.319	-0.3227663		TESK2	testis-specific kinase 2
22.850.519	0.3956088		SMYD2	SET and MYND domain containing 2
22.849.113	0.2474482		RBM19	RNA binding motif protein 19

2.283.146	0.4406805	ERGIC3	ERGIC and golgi 3
22.821.775	0.525623	ESPL1	extra spindle pole bodies like 1, separase
22.819.626	-0.4222661	MCCC1	methylcrotonoyl-CoA carboxylase 1
2.281.446	-0.2868224	SNRNP70	small nuclear ribonucleoprotein U1 subunit 70
22.813.775	0.3505244	RABGAP1	RAB GTPase activating protein 1
22.809.865	-0.3152614	CD79A	CD79a molecule
22.791.731	0.446426	MAP3K7	mitogen-activated protein kinase kinase kinase
2.278.004	0.7347388	MAGEA1	MAGE family member A1
22.773.926	-0.375853	TERF2IP	TERF2 interacting protein
22.764.849	-0.5163968	ENOSF1	enolase superfamily member 1
22.761.062	-0.8905283	GEM	GTP binding protein overexpressed in skeletal n
2.275.239	0.3411452	KANSL2	KAT8 regulatory NSL complex subunit 2
22.750.263	0.3526092	CLCC1	chloride channel CLIC like 1
22.735.643	-0.1822896	PCNX4	pecanex homolog 4 (Drosophila)
22.721.245	0.3685381	CLIP2	CAP-Gly domain containing linker protein 2
22.715.007	-0.5137864	ENDOG	endonuclease G
22.709.819	0.4911742	DOCK4	dedicator of cytokinesis 4
22.697.051	0.6936013	UGDH///PG	UDP-glucose 6-dehydrogenase///phosphogluc
22.694.009	-0.5398583	RIPK4	receptor interacting serine/threonine kinase 4
22.681.338	-0.3678826	ARL6IP1	ADP ribosylation factor like GTPase 6 interactin
22.649.925	0.4405977	KIF1BP	KIF1 binding protein
22.649.191	0.4198725	EMC3	ER membrane protein complex subunit 3
22.648.544	0.4196261	SUPV3L1	Suv3 like RNA helicase
22.620.701	-0.2185762	GRK2	G protein-coupled receptor kinase 2
22.618.295	0.3116925	FAM134A	family with sequence similarity 134 member A
2.261.293	0.1956633	AVL9	AVL9 cell migration associated
22.608.815	-0.9213162	TXNIP	thioredoxin interacting protein
22.596.616	-0.3546064	LINC00574	long intergenic non-protein coding RNA 574
22.592.111	-0.2122566	CD86	CD86 molecule
22.586.255	0.4197687	ARF6	ADP ribosylation factor 6
22.584.481	0.3853934	TAOK3	TAO kinase 3
22.584.106	-0.5066653	RAB14	RAB14, member RAS oncogene family
22.555.792	0.2499709	HMGXB3	HMG-box containing 3
22.554.886	0.5532688	STXBP1	syntaxin binding protein 1
22.554.796	-0.1869987	NOX1	NADPH oxidase 1
2.255.393	-0.2043177	DNAJC16	DnaJ heat shock protein family (Hsp40) membe
22.553.116	0.4221184	CHD4	chromodomain helicase DNA binding protein 4
22.549.069	-0.3945766	ARHGAP32	Rho GTPase activating protein 32
2.254.845	-0.3439014	NDUFB8	NADH:ubiquinone oxidoreductase subunit B8
22.527.187	-0.1935777	SEZ6L	seizure related 6 homolog like
2.249.416	-0.1857818	SLC15A1	solute carrier family 15 member 1
22.456.109	0.6348825	SYNM	synemin
22.451.435	-0.2067997	MIA2	melanoma inhibitory activity 2
22.431.163	0.595087	PECAM1	platelet and endothelial cell adhesion molecule

22.425.977	-0.2918068	MAP4K1	mitogen-activated protein kinase kinase kinase
22.424.157	0.3811092	PRKAG1	protein kinase AMP-activated non-catalytic sub
22.423.102	-0.2384588		
22.416.817	0.8902491	GPNMB	glycoprotein nmb
22.401.828	-0.2812413	UTY	ubiquitously transcribed tetratricopeptide repe
22.394.821	-0.3894916	TPMT	thiopurine S-methyltransferase
22.389.347	-0.3930843	ARID4A	AT-rich interaction domain 4A
2.238.894	0.489783	PREPL	prolyl endopeptidase-like
22.382.392	0.4583793	ASAH1	N-acylsphingosine amidohydrolase 1
22.379.133	0.5592754	CDK19	cyclin dependent kinase 19
22.378.383	0.4413865	ARNTL2	aryl hydrocarbon receptor nuclear translocator
22.378.352	0.2259356	BAK1	BCL2 antagonist/killer 1
22.352.118	-0.2158005	TP63	tumor protein p63
2.234.546	-0.1858419	RHO	rhodopsin
22.336.423	-0.3019296	DNAJB12	DnaJ heat shock protein family (Hsp40) membe
22.336.087	-0.2115787	TRA	T-cell receptor alpha locus
22.335.155	-0.6564197	DDT	D-dopachrome tautomerase
223.343	-0.2139265	BCL11B	B-cell CLL/lymphoma 11B
22.328.317	0.323647	SOS1	SOS Ras/Rac guanine nucleotide exchange fact
22.320.555	-0.2208272	COLQ	collagen like tail subunit of asymmetric acetylc
22.315.799	-0.1874856	PWWP2A	PWWP domain containing 2A
22.309.314	-0.5287188	RNF128	ring finger protein 128, E3 ubiquitin protein ligas
22.307.702	0.5066059	CDS2	CDP-diacylglycerol synthase 2
22.305.344	0.3941618	TRIM33	tripartite motif containing 33
22.290.542	-0.2842112	TTLL5	tubulin tyrosine ligase like 5
22.288.151	-0.25256	GFRA1	GDNF family receptor alpha 1
22.282.204	-0.5398485	FAS	Fas cell surface death receptor
22.281.622	-0.2729973	WNT11	Wnt family member 11
2.227.014	0.3931823	KHDRBS1	KH RNA binding domain containing, signal trans
22.259.901	-0.4083081	TSPYL5	TSPY like 5
2.225.785	-0.1763104	DDN	dendrin
22.257.064	0.5770352	MAGEA5	MAGE family member A5
2.225.506	-0.2474638	GSTM5	glutathione S-transferase mu 5
22.250.613	0.2876472	MCM3AP-A	MCM3AP antisense RNA 1
22.244.631	0.3927508	TMEM131	transmembrane protein 131
22.243.863	-0.5699157	PALM2-AKA	PALM2-AKAP2 readthrough///A-kinase anchorir
22.210.599	0.2677522	ZNF227	zinc finger protein 227
22.205.792	0.3368499	NDUFA7	NADH:ubiquinone oxidoreductase subunit A7
22.205.221	0.32548	HYPK	huntingtin interacting protein K
22.187.221	-0.2139521	SRRM2	serine/arginine repetitive matrix 2
22.179.868	-0.1956753	SMCP	sperm mitochondria associated cysteine rich p
221.797	0.3895358	G6PC3	glucose-6-phosphatase catalytic subunit 3
22.179.562	0.2836766	TMEM11	transmembrane protein 11
22.179.327	0.4190274	GLO1	glyoxalase I

22.177.552	0.7361388	LOC73010:	uncharacterized LOC730101
22.177.167	0.8587011	SOD2	superoxide dismutase 2, mitochondrial
22.169.066	0.3344715	GOSR2	golgi SNAP receptor complex member 2
2.215.707	-0.5814193	MPDU1	mannose-P-dolichol utilization defect 1
22.148.737	-0.4694408	ADRB2	adrenoceptor beta 2
22.146.136	0.3494716	MAZ	MYC associated zinc finger protein
2.214.082	-0.3161439	TK2	thymidine kinase 2, mitochondrial
22.137.041	0.3558368	MAPKAP1	mitogen-activated protein kinase associated pr
22.122.604	-0.7160867	TIAM1	T-cell lymphoma invasion and metastasis 1
22.122.291	0.3294736	BTN2A1	butyrophilin subfamily 2 member A1
22.119.926	0.3343412	TRAPPC2	trafficking protein particle complex 2
22.109.475	-0.2238816	TCF3	transcription factor 3
22.106.868	-0.5668718	MYH10	myosin, heavy chain 10, non-muscle
22.100.511	0.2926268	MED12	mediator complex subunit 12
22.097.353	0.5587798	ALDH1A3	aldehyde dehydrogenase 1 family member A3
22.078.193	-0.2482125	IDS	iduronate 2-sulfatase
2.206.509	0.2472564	SMARCA5	SWI/SNF related, matrix associated, actin depe
22.044.895	-0.3138658	HCG2P7	HLA complex group 2 pseudogene 7
22.041.655	-0.4860919	PTPN3	protein tyrosine phosphatase, non-receptor typ
220.413	0.4045589	NDUFS8	NADH:ubiquinone oxidoreductase core subunit
2.203.824	0.3098971	AGTPBP1	ATP/GTP binding protein 1
22.031.023	0.4617332	LRRC8B	leucine rich repeat containing 8 family member
22.022.928	-0.3326276	IFNAR2	interferon alpha and beta receptor subunit 2
22.014.465	0.4264575	FARS2	phenylalanyl-tRNA synthetase 2, mitochondrial
22.009.362	0.4215192	TOR1B	torsin family 1 member B
21.984.147	-0.2003356	FXR2	FMR1 autosomal homolog 2
21.979.549	0.3822932	SMARCB1	SWI/SNF related, matrix associated, actin depe
21.978.595	-0.3078409	TNFRSF10E	TNF receptor superfamily member 10b
2.196.375	-0.3589236	AP3S2	adaptor related protein complex 3 sigma 2 subu
21.957.884	-0.5194892	TJP2	tight junction protein 2
21.956.422	0.3089269	STAU1	staufen double-stranded RNA binding protein 1
21.952.907	-0.3753827	EPAS1	endothelial PAS domain protein 1
21.945.841	0.307337	GPSM2	G-protein signaling modulator 2
21.931.402	0.4306373	P3H4	prolyl 3-hydroxylase family member 4 (non-enzy
21.927.513	0.6269624	PRRC2C	proline rich coiled-coil 2C
21.923.485	0.4978707	FERMT1	fermitin family member 1
21.919.696	0.8704469	ICAM1	intercellular adhesion molecule 1
21.916.826	-0.1858712	ENPP3	ectonucleotide pyrophosphatase/phosphodies
21.913.878	0.3784935	CBFA2T2	CBFA2/RUNX1 translocation partner 2
21.911.442	0.6008265	MPZL2	myelin protein zero like 2
21.907.913	-0.1607849	HAND1	heart and neural crest derivatives expressed 1
21.905.456	-0.8107815	SERPINA6	serpin family A member 6
21.895.223	-0.3703149	MYO7A	myosin VIIA
21.885.575	0.4005281	YY1	YY1 transcription factor

21.881.226	0.4066667	CAP1	adenylate cyclase associated protein 1
21.874.115	-0.2366056	FCN2	ficolin 2
21.869.421	0.1909529	FBXO11	F-box protein 11
21.867.732	-0.2238159	LPAR1	lysophosphatidic acid receptor 1
2.186.139	-0.6463998	C4B///C4A	complement component 4B (Chido blood grou
21.860.381	-0.1718515	LOC28424	uncharacterized LOC284244
21.855.222	-0.2380929	RNF122	ring finger protein 122
21.852.362	-0.2690794	RABEP2	rabaptin, RAB GTPase binding effector protein 2
2.184.829	0.4279249	DARS	aspartyl-tRNA synthetase
21.845.921	0.4184111		
21.844.155	0.2191873	N6AMT1	N-6 adenine-specific DNA methyltransferase 1
21.843.515	-0.1867673	ALG1L2	ALG1, chitobiosyldiphosphodolichol beta-man
21.818.635	0.312958	ZNF131	zinc finger protein 131
21.810.402	-0.1626842	AKT3	AKT serine/threonine kinase 3
21.807.758	0.2522652	URM1	ubiquitin related modifier 1
21.807.279	-0.3239446	CD247	CD247 molecule
21.798.615	0.496002	SPTLC1	serine palmitoyltransferase long chain base su
21.788.194	0.4516589	FEZ2	fasciculation and elongation protein zeta 2
21.782.748	0.3485616	C5orf30	chromosome 5 open reading frame 30
21.779.238	0.3900421	HS1BP3	HCLS1 binding protein 3
21.772.543	0.4393649	RAB7A	RAB7A, member RAS oncogene family
21.769.783	0.3110221	QRSL1	glutaminyl-tRNA synthase (glutamine-hydrolyz
2.176.183	0.5605228	TNFRSF21	TNF receptor superfamily member 21
217.576	-0.216608	LOC73009	hypothetical protein LOC730096
21.754.801	0.47098	DPM3	dolichyl-phosphate mannosyltransferase subu
21.754.374	0.5063708	RAN	RAN, member RAS oncogene family
2.174.731	-0.2012188	LPAR3	lysophosphatidic acid receptor 3
21.736.286	0.3906289	GSDMD	gasdermin D
21.728.613	0.2955031	IER3IP1	immediate early response 3 interacting protein
21.725.112	-0.2897387	PDE6G	phosphodiesterase 6G
21.714.403	0.5134215	MEAF6	MYST/Esa1 associated factor 6
21.711.877	0.3100738	RCE1	Ras converting CAAX endopeptidase 1
21.710.657	0.4749027	SH3GLB1	SH3 domain containing GRB2 like endophilin B
21.708.861	0.4446506	PIGG	phosphatidylinositol glycan anchor biosynthesi
2.170.435	-0.1555103	SYNE1	spectrin repeat containing nuclear envelope pr
21.688.196	0.4483093	TFAM	transcription factor A, mitochondrial
21.687.344	0.3961344	ARMCX5	armadillo repeat containing, X-linked 5
21.682.757	-0.7867223	AADAC	arylacetamide deacetylase
21.682.304	-0.5261032	NFKBIA	NFKB inhibitor alpha
21.680.185	0.651894	TMEM97	transmembrane protein 97
21.677.461	-0.2076566	SP100	SP100 nuclear antigen
21.664.854	-0.4999866	PGRMC2	progesterone receptor membrane component 2
21.662.795	-0.2501614	INPP5K	inositol polyphosphate-5-phosphatase K
21.662.604	-0.6085081	GCAT	glycine C-acetyltransferase

21.661.621	-0.4886847	GIMAP4	GTPase, IMAP family member 4
21.660.947	0.4602321	LOC73074	similar to macrophage erythroblast attacher///r
21.653.799	0.3370725	PSEN2	presenilin 2
21.646.575	-0.2181465	GALNT14	polypeptide N-acetylgalactosaminyltransferase
21.638.832	0.333743	MEPCE	methylphosphate capping enzyme
21.635.578	-0.2070979	SRPK3	SRSF protein kinase 3
21.628.898	0.2675684		
21.617.059	0.5050776	PTBP2	polypyrimidine tract binding protein 2
21.615.164	-0.4920073	F3	coagulation factor III, tissue factor
21.606.802	-0.2121339	PDE4B	phosphodiesterase 4B
21.595.908	0.4544675	ITGB5	integrin subunit beta 5
21.595.182	0.3459912	HINT1	histidine triad nucleotide binding protein 1
21.594.395	-0.2020713	FAM162A	family with sequence similarity 162 member A
21.591.403	0.4104088	KPNA3	karyopherin subunit alpha 3
21.572.593	0.2933458	SLF2	SMC5-SMC6 complex localization factor 2
21.571.728	-0.4190475	RPL35A	ribosomal protein L35a
21.564.273	-0.32468	SDPR	serum deprivation response
21.559.692	0.316507	RPN1	ribophorin I
21.554.133	0.3426036	YIPF6	Yip1 domain family member 6
21.547.173	-0.4011858	SPHK2	sphingosine kinase 2
21.544.745	0.9467935	MAGEA12	MAGE family member A12
21.533.579	-0.2118678	NNAT	neuronatin
21.520.997	0.4464967	EEF1D	eukaryotic translation elongation factor 1 delta
21.514.369	-0.8034927	NAT8	N-acetyltransferase 8 (putative)
21.513.958	-0.4789657	MRPL34	mitochondrial ribosomal protein L34
21.502.638	-0.1779238	SULT2B1	sulfotransferase family 2B member 1
21.497.121	0.4381455	MLH3	mutL homolog 3
2.149.212	-0.4361101	PEX11A	peroxisomal biogenesis factor 11 alpha
21.484.216	-0.2477681	PRKCQ	protein kinase C theta
21.482.658	0.7117222	RAB1A	RAB1A, member RAS oncogene family
21.476.622	0.3267656	CELSR3	cadherin EGF LAG seven-pass G-type receptor 3
21.470.509	-0.2679215	HOXB1	homeobox B1
21.467.416	-0.3412946	MUC5AC	mucin 5AC, oligomeric mucus/gel-forming
21.460.523	-0.5877787	KDM4B	lysine demethylase 4B
21.458.163	0.3227319	EIF4H	eukaryotic translation initiation factor 4H
21.450.207	0.3626347	LEMD3	LEM domain containing 3
21.444.842	-0.2460247	LAT	linker for activation of T-cells
21.429.174	-0.2310542	MICAL2	microtubule associated monooxygenase, calpo
21.427.856	0.3129365	GMEB2	glucocorticoid modulatory element binding pro
21.427.064	-0.2107511	PAX5	paired box 5
21.420.872	0.2604352	SLC25A51	solute carrier family 25 member 51
21.417.927	0.8643607	SLC38A1	solute carrier family 38 member 1
21.405.967	0.2631091	CDKAL1	CDK5 regulatory subunit associated protein 1 li
21.376.869	0.2469155	BAP1	BRCA1 associated protein 1

21.370.778	0.3807057	NCK1	NCK adaptor protein 1
21.360.257	0.4201417	PDCD2	programmed cell death 2
21.359.774	-0.4390484	IGFBP6	insulin like growth factor binding protein 6
21.343.424	-0.1796006	BMP5	bone morphogenetic protein 5
21.343.144	0.2584278		
21.342.446	-0.1501322	AKIRIN2-AS	AKIRIN2 antisense RNA 1
21.331.789	0.320249	DNAJB2	DnaJ heat shock protein family (Hsp40) membe
2.132.916	-0.503487	PIK3CD	phosphatidylinositol-4,5-bisphosphate 3-kinas
21.323.054	0.3542007	NDUFA4	NDUFA4, mitochondrial complex associated
21.312.176	0.3809151	PDGFA	platelet derived growth factor subunit A
21.304.804	0.3160598	CCDC51	coiled-coil domain containing 51
21.304.564	0.7190501	GGH	gamma-glutamyl hydrolase
2.129.734	-0.209099	BTK	Bruton tyrosine kinase
21.294.883	0.2896603	SNX2	sorting nexin 2
21.284.582	-0.2932126	PRDM2	PR/SET domain 2
2.128.346	0.408138	RNF139	ring finger protein 139
21.276.402	0.3869767	ZNF516	zinc finger protein 516
21.273.824	0.3554588	HNRNPM	heterogeneous nuclear ribonucleoprotein M
21.270.267	-0.1767874	EMILIN2	elastin microfibril interfacer 2
21.264.792	0.4070142	SHARPIN	SHANK associated RH domain interactor
2.126.191	0.8338704	TWF1	twinfilin actin binding protein 1
21.261.588	0.3223216	RPS2P53///	ribosomal protein S2 pseudogene 53///ribosom
21.245.836	-0.153472	MAP3K4	mitogen-activated protein kinase kinase kinase
21.239.807	0.3875426	HARS	histidyl-tRNA synthetase
21.238.889	0.2845416	RNFT2	ring finger protein, transmembrane 2
21.238.859	0.3490602	GAS2L1	growth arrest specific 2 like 1
212.332	0.5153621	SSX2IP	SSX family member 2 interacting protein
21.229.735	0.5298231	VDAC3///IT	voltage dependent anion channel 3///inositol 1,
21.227.275	-0.1739906	TRIM10	tripartite motif containing 10
21.224.472	-0.5523651	EGFR	epidermal growth factor receptor
21.221.342	0.3315108	EEF1DP1///	eukaryotic translation elongation factor 1 delta
21.196.593	0.6030578	TSPAN3	tetraspanin 3
21.189.715	-0.1905689	TRMT61A	tRNA methyltransferase 61A
21.181.854	-0.2638184	APBB1IP	amyloid beta precursor protein binding family B
21.180.696	-0.146414	WNT2B	Wnt family member 2B
21.176.449	0.3539577	TCF20	transcription factor 20
21.159.536	-0.2213637	CADM3	cell adhesion molecule 3
21.154.704	-0.2309177	TCP11L1	t-complex 11 like 1
21.151.188	-0.7579527	HLA-C	major histocompatibility complex, class I, C
21.147.103	-0.4993062	CCDC69	coiled-coil domain containing 69
21.145.972	0.3737147	ATP9B	ATPase phospholipid transporting 9B (putative)
21.144.639	0.2730143	OAZ1	ornithine decarboxylase antizyme 1
21.140.786	-0.1683284	IGHD	immunoglobulin heavy constant delta
21.139.453	0.6568076	ABCG1	ATP binding cassette subfamily G member 1

21.097.861	-0.2377277	GFRA2	GDNF family receptor alpha 2
21.075.412	0.5902677	FGFR4	fibroblast growth factor receptor 4
21.068.835	0.2242767	LRRC14	leucine rich repeat containing 14
21.057.892	-0.5382948	CREBL2	cAMP responsive element binding protein like 2
21.049.371	0.2685468	LOC65387	small subunit processome component 20 hom
2.104.476	0.5059278	TPP1	tripeptidyl peptidase 1
21.043.119	-0.2116764	SMAD1	SMAD family member 1
21.040.328	0.2994275	NFYC	nuclear transcription factor Y subunit gamma
210.182	-0.3141842	EDN1	endothelin 1
21.002.093	0.4204067	CXorf40A	chromosome X open reading frame 40A
2.099.938	-0.1670475	MED25	mediator complex subunit 25
2.098.668	-0.2855353	SIRT5	sirtuin 5
20.986.449	-0.4246446		
20.968.847	0.439707	MED23	mediator complex subunit 23
20.964.442	-0.2532498	SLC34A2	solute carrier family 34 member 2
20.960.963	-0.2942227	AMBP	alpha-1-microglobulin/bikunin precursor
20.957.731	0.1883508	SMIM8	small integral membrane protein 8
20.956.849	0.4724339	ARFGEF1	ADP ribosylation factor guanine nucleotide exc
20.956.279	0.615983	CAV1	caveolin 1
20.954.486	-0.1661586	CYP2U1	cytochrome P450 family 2 subfamily U membe
20.949.013	0.4684148	KPNB1	karyopherin subunit beta 1
20.945.623	-0.2709922	TINAGL1	tubulointerstitial nephritis antigen like 1
20.936.704	0.3673812	SDAD1	SDA1 domain containing 1
20.931.851	-0.2119352	RSU1	Ras suppressor protein 1
20.926.603	-0.1498929	CD40LG	CD40 ligand
20.924.403	-0.2806526	DMWD	dystrophia myotonica, WD repeat containing
20.902.328	0.2652852	SUV39H1	suppressor of variegation 3-9 homolog 1
2.088.923	-0.1914015	ITFG2	integrin alpha FG-GAP repeat containing 2
20.879.247	0.3922909	RANGAP1	Ran GTPase activating protein 1
20.872.283	-0.1544477	DLGAP1	DLG associated protein 1
20.862.429	-0.2069689	SH2B1	SH2B adaptor protein 1
20.862.016	-0.2157391	ARHGAP28	Rho GTPase activating protein 28
20.847.815	0.2905993	TSR2	TSR2, ribosome maturation factor
20.836.793	0.2901815	VAMP4	vesicle associated membrane protein 4
20.836.131	-0.535832	PXDN	peroxidasin
20.824.868	-0.3163771	SGCD	sarcoglycan delta
20.808.921	0.3235985	ZFX	zinc finger protein, X-linked
20.804.547	-0.8550806	AHSG	alpha 2-HS glycoprotein
20.802.043	0.3995985	UTRN	utrophin
20.791.997	0.3922986	BTBD1	BTB domain containing 1
20.782.936	-0.3236995	SH3BP2	SH3 domain binding protein 2
20.778.972	0.5080875	TMX1	thioredoxin related transmembrane protein 1
20.771.525	0.3544343	CNOT3	CCR4-NOT transcription complex subunit 3
20.751.621	-0.1792388	IRAK3	interleukin 1 receptor associated kinase 3

20.734.528	0.4385079		NRP1	neuropilin 1
20.734.374	-0.2128429		CASS4	Cas scaffolding protein family member 4
20.720.203	0.3206182		CCDC6	coiled-coil domain containing 6
20.707.903	0.7664257		CPE	carboxypeptidase E
2.070.403	-0.3253988		PAFAH2	platelet activating factor acetylhydrolase 2
20.687.747	-0.4695241		ANGPTL2	angiopoietin like 2
20.682.762	0.3728721		IL17RB///YI	interleukin 17 receptor B///Yip1 domain family r
20.679.536	-0.2521531		FUZ	fuzzy planar cell polarity protein
20.677.211	0.2036175		CCDC93	coiled-coil domain containing 93
20.674.076	0.6004137		PRAME	preferentially expressed antigen in melanoma
20.648.541	0.5299012		GAS2L1	growth arrest specific 2 like 1
20.641.982	0.3682373		TTC23	tetratricopeptide repeat domain 23
2.064.098	-0.264723		TFPI2	tissue factor pathway inhibitor 2
20.635.699	-0.2104813		PAX2	paired box 2
20.614.705	-0.1630958		BCO2///CD	beta-carotene oxygenase 2///cyclin dependent
20.608.912	-0.4891604		FNDC3A	fibronectin type III domain containing 3A
20.608.639	-0.1441244		ACVR1B	activin A receptor type 1B
2.060.676	0.3307898		RBM10	RNA binding motif protein 10
2.060.265	-0.2675311		GFOD2	glucose-fructose oxidoreductase domain conta
20.601.642	-0.2741424		HK3	hexokinase 3
20.597.125	-0.1684562			
20.590.867	-0.5124214		KIAA1551	KIAA1551
20.575.238	-0.1736594		IVD	isovaleryl-CoA dehydrogenase
20.565.516	0.3595324		VPS41	VPS41, HOPS complex subunit
20.561.408	0.3524581		BAX	BCL2 associated X, apoptosis regulator
20.558.797	-0.1978124		FUT6	fucosyltransferase 6
20.555.024	0.2941521		PRR13	proline rich 13
20.547.508		10.200.608	LGR5	leucine rich repeat containing G protein-couple
20.542.486	0.6020097		SLC2A5	solute carrier family 2 member 5
20.541.027	0.4527659		JOSD1	Josephin domain containing 1
20.540.449	-0.1783101		THAP3	THAP domain containing 3
205.403	0.3052476		CLCN4	chloride voltage-gated channel 4
20.539.162	0.3063034		NUP214	nucleoporin 214
20.522.111	-0.1958343		TBX6	T-box 6
2.051.835	0.2956019		CBX4	chromobox 4
20.492.241	-0.4338634		FAM193B	family with sequence similarity 193 member B
20.481.865	-0.4143125		MYO7A	myosin VIIA
204.529	0.2679356		IFNAR2	interferon alpha and beta receptor subunit 2
20.452.798	0.2723087		HAUS5	HAUS augmin like complex subunit 5
20.452.294	-0.1894064		C14orf1	chromosome 14 open reading frame 1
20.447.243	0.3856345		PIGZ	phosphatidylinositol glycan anchor biosynthesi
20.441.246	-0.4394379		CTSF	cathepsin F
2.043.658	-0.1479581		TXK	TXK tyrosine kinase
20.436.161	0.4641505		TLE1	transducin like enhancer of split 1

20.435.461	-0.7437612	C8G	complement component 8, gamma polypeptide
20.432.385	0.3285285	RANBP9	RAN binding protein 9
20.432.156	0.2965191	BRF2	BRF2, RNA polymerase III transcription initiatio
20.420.516	0.4489588	BLMH	bleomycin hydrolase
20.415.719	0.4944313	UNC13B	unc-13 homolog B
20.407.944	0.2603491	SMARCC1	SWI/SNF related, matrix associated, actin depe
20.403.212	0.5764629	SUSD4	sushi domain containing 4
20.395.747	-0.2165513	LOC64253:	uncharacterized LOC642533///hypothetical pro
20.394.189	0.2776632	PLXNA3	plexin A3
2.037.878	-0.3860075	PCBP2	poly(rC) binding protein 2
20.374.903	0.3422397	LPL	lipoprotein lipase
20.373.371	0.7061194	LOC73240:	similar to butyrate-induced transcript 1///3-hyd
20.370.824	-0.3093439	CENPC	centromere protein C
20.363.988	0.2540262	B3GNTL1	UDP-GlcNAc:betaGal beta-1,3-N-acetylglucosa
20.352.778	-0.222773	FRMD4B	FERM domain containing 4B
20.333.907	0.3590764	NSUN5P1	NOP2/Sun RNA methyltransferase family meml
20.333.501	0.3544541	UQCR10	ubiquinol-cytochrome c reductase, complex III
20.330.185	-0.4141688	ABCC9	ATP binding cassette subfamily C member 9
20.326.142	-0.4047656	CCNL1	cyclin L1
20.325.037	0.3236221	CALR	calreticulin
20.318.684	0.3836871	FUS	FUS RNA binding protein
20.317.711	-0.2716534	ARSD	arylsulfatase D
20.314.254	-0.3394477	RBBP6	RB binding protein 6, ubiquitin ligase
2.030.312	-0.5486943	PEPD	peptidase D
20.301.733	-0.3626029	VAMP2	vesicle associated membrane protein 2
20.300.318	0.4542147	NDUFS8	NADH:ubiquinone oxidoreductase core subunit
20.295.082	0.5362832	SPHK1	sphingosine kinase 1
20.293.284	-0.206796	TYR	tyrosinase
20.288.431	0.2456685	MFN1///SY	mitofusin 1///synaptotagmin 7
20.282.625	0.2691948	TTC4	tetratricopeptide repeat domain 4
20.279.641	-0.1833201	CSF2	colony stimulating factor 2
202.731	-0.2020428	TSPAN4	tetraspanin 4
20.266.358	0.1853724	FANCA	Fanconi anemia complementation group A
20.266.289	0.4051243	ARF3	ADP ribosylation factor 3
20.265.449	-0.5468921	NFIB	nuclear factor I B
20.254.341	0.2274814	SMG9	SMG9, nonsense mediated mRNA decay factor
20.245.985	-0.2349675	LINC00939	long intergenic non-protein coding RNA 939
20.240.239	0.2721938	RHOT1	ras homolog family member T1
20.238.427	0.3347455	ERCC1	ERCC excision repair 1, endonuclease non-cata
20.237.327	-0.2235139	KANK3	KN motif and ankyrin repeat domains 3
20.232.672	0.2315796	SLC12A9	solute carrier family 12 member 9
20.215.346	-0.3768712	NPIPB6///N	nuclear pore complex interacting protein family
20.210.409	0.4420016	PCDHGC3	protocadherin gamma subfamily C, 3
20.197.503	0.3529141	STX7	syntaxin 7

20.196.166	0.3683122	FAM175B	family with sequence similarity 175 member B
20.192.972	-0.219993	OMG	oligodendrocyte myelin glycoprotein
20.176.456	-0.1921718	CD6	CD6 molecule
20.174.251	0.3322397	CNOT2	CCR4-NOT transcription complex subunit 2
20.172.748	-0.1754826	SFTPB	surfactant protein B
20.170.208	-0.2273022	RAB22A	RAB22A, member RAS oncogene family
20.167.519	-0.2456824	LOC57399	uncharacterized gastric protein ZA52P
20.164.886	0.4971014	CLSTN1	calsyntenin 1
20.163.872	-0.4333623	BLCAP	bladder cancer associated protein
20.162.658	-0.1877944	AAK1	AP2 associated kinase 1
20.159.202	0.785229	MLLT11	myeloid/lymphoid or mixed-lineage leukemia; t
20.152.216	0.3584724	NIPBL	NIPBL, cohesin loading factor
20.141.101	0.3057526	RPL15P7///	ribosomal protein L15 pseudogene 7///ribosom
20.140.708	-0.3994182	SIDT2	SID1 transmembrane family member 2
20.138.308	-0.2921865	PIP	prolactin induced protein
20.135.302	0.2706317	NPAT	nuclear protein, coactivator of histone transcrip
20.131.268	0.6222056	SNTB1	syntrophin beta 1
20.116.572	-0.2565435	MORN1	MORN repeat containing 1
20.110.541	-0.2191507	MRE11A	MRE11 homolog A, double strand break repair r
20.105.952	0.4933301	IDH3A	isocitrate dehydrogenase 3 (NAD(+)) alpha
20.105.404	-0.5877294	C4B///C4A	complement component 4B (Chido blood group
20.104.071	-0.6353176	BLNK	B-cell linker
20.097.238	0.5015156	GLCE	glucuronic acid epimerase
20.095.036	0.2935871	JAG2	jagged 2
20.081.811	-0.2843902	CXCR2	C-X-C motif chemokine receptor 2
20.079.584	-0.5525244	CDHR5	cadherin related family member 5
20.060.473	-0.1752119	KLF1	Kruppel like factor 1
20.060.281	-0.2409772	SIRT3	sirtuin 3
20.052.431	0.5486073	CTNNAL1	catenin alpha like 1
20.038.409	0.5313988	WWC1	WW and C2 domain containing 1
20.025.469	0.3943077	H3F3B	H3 histone, family 3B
20.018.536	-0.4717806	GK	glycerol kinase
20.017.284	-0.3738243	CLPP	caseinolytic mitochondrial matrix peptidase pro
20.013.835	0.4281129	GNB5	G protein subunit beta 5
20.010.583	0.444606	STAG2	stromal antigen 2
20.009.602	-0.197646	TCL1A	T-cell leukemia/lymphoma 1A
20.009.578	-0.1872873	IL37	interleukin 37
20.008.401	-0.4239709	COL4A3	collagen type IV alpha 3 chain
20.005.577	0.2429706	ZMYM4	zinc finger MYM-type containing 4
20.000.568	-0.3195375		
20.000.075	0.579943	RAB11FIP1	RAB11 family interacting protein 1
19.996.229	-0.2496857	PPP2R2B	protein phosphatase 2 regulatory subunit Bbeta
19.991.967	0.3537057	TBCA	tubulin folding cofactor A
19.989.162	0.3175075	COX7A2	cytochrome c oxidase subunit 7A2

19.989.147	-0.13516	TMPRSS11I	transmembrane protease, serine 11E
19.986.897	0.3191922	BTN2A1	butyrophilin subfamily 2 member A1
19.975.926	-0.212864	NCR2	natural cytotoxicity triggering receptor 2
19.970.079	0.3555414	RPS14	ribosomal protein S14
1.996.961	-0.4372102	C16orf45	chromosome 16 open reading frame 45
19.952.896	0.1785588	ARL15	ADP ribosylation factor like GTPase 15
19.944.448	0.3850719	XPNPEP1	X-prolyl aminopeptidase 1
19.937.921	0.3229485	HIST1H2BI	histone cluster 1, H2bi
19.936.332	0.2400674	SUPT3H	SPT3 homolog, SAGA and STAGA complex comp
19.926.386	-0.6102459	SERPINF1	serpin family F member 1
19.923.505	-0.6005857	TBC1D9	TBC1 domain family member 9
19.922.903	-0.2230378	HNRNPD	heterogeneous nuclear ribonucleoprotein D
19.920.142	-0.7918938	GGT1	gamma-glutamyltransferase 1
19.912.891	0.3563954	EIF3J	eukaryotic translation initiation factor 3 subunit
19.910.336	-0.5817414	MAOA	monoamine oxidase A
19.910.236	-0.458386	NFASC	neurofascin
19.905.623	-0.2531796	LCK	LCK proto-oncogene, Src family tyrosine kinase
19.903.502	0.4387976	PDCD4	programmed cell death 4 (neoplastic transform
19.903.204	-0.2618323	SLC45A2	solute carrier family 45 member 2
19.902.022	0.3779472	NUP58	nucleoporin 58
19.899.698	-0.4066115	USP34	ubiquitin specific peptidase 34
19.895.336	0.3039679	C12orf43	chromosome 12 open reading frame 43
19.894.134	0.4451594	NES	nestin
19.885.965	-0.1855944	FCHO1	FCH domain only 1
19.883.084	0.6299608	TPM1	tropomyosin 1 (alpha)
19.871.776	0.3257175	ZNF419	zinc finger protein 419
19.851.443	-0.1607379	NIPAL3	NIPA like domain containing 3
19.846.536	0.3644417	FHIT	fragile histidine triad
19.840.012	-0.539876	CEBPD	CCAAT/enhancer binding protein delta
1.983.879	0.2578619	CREB1	cAMP responsive element binding protein 1
19.831.289	-0.3150265	GPR153	G protein-coupled receptor 153
19.824.703	-0.170481	LOC73156!	similar to fibronectin type III and SPRY domain c
19.812.004	-0.4366268	ISOC2	isochorismatase domain containing 2
1.981.076	0.3167571	CEP63	centrosomal protein 63
19.796.545	-0.2177278	FUT7	fucosyltransferase 7
19.795.632	-0.7091347	ABCC3	ATP binding cassette subfamily C member 3
19.793.635	0.2424418	MXD3	MAX dimerization protein 3
19.789.824	0.5498608	HOXA10	homeobox A10
19.788.782	0.2166421	DNMT3A	DNA methyltransferase 3 alpha
19.786.786	-0.7566628	PPP2R1B	protein phosphatase 2 scaffold subunit Abeta
19.784.416	-0.2204531	CHST8	carbohydrate sulfotransferase 8
19.782.102	-0.1647233	CTIF	cap binding complex dependent translation init
19.778.933	0.4828304	LSM5	LSM5 homolog, U6 small nuclear RNA and mRN
19.769.347	-0.2057671	MYCNOS	MYCN opposite strand

1.976.479	0.2568616	ZNF696	zinc finger protein 696
19.762.771	-0.1963937	OMD	osteomodulin
19.751.729	0.4602309	ZBTB10	zinc finger and BTB domain containing 10
19.747.818	-0.2051522	AKAP8L	A-kinase anchoring protein 8 like
19.747.259	0.4615315	NDUFAF4	NADH:ubiquinone oxidoreductase complex ass
19.745.198	-0.2357798	C11orf16	chromosome 11 open reading frame 16
19.736.459	0.444641	PI4KA	phosphatidylinositol 4-kinase alpha
19.734.543	-0.1882202	PDE8A	phosphodiesterase 8A
19.718.614	0.2312714	DAXX	death domain associated protein
19.716.918	-0.193236	PDX1	pancreatic and duodenal homeobox 1
19.716.737	0.2776634	SCYL3	SCY1 like pseudokinase 3
19.710.319	0.7789195	HIST2H2AA	histone cluster 2, H2aa4///histone cluster 2, H2
19.709.506	0.3905302	SNN	stannin
19.706.857	-0.4792087	ATF7IP2	activating transcription factor 7 interacting prot
19.701.614	-0.1883959	KAZN	kazrin, periplakin interacting protein
19.696.085	0.4125049	SRSF2	serine and arginine rich splicing factor 2
19.691.917	-0.2608495	RUNX1T1	RUNX1 translocation partner 1
19.674.652	-0.2975847	KIAA0141	KIAA0141
19.674.208	-0.2149939	ATP8B4	ATPase phospholipid transporting 8B4 (putative
19.666.895	0.3422182	TRIM33	tripartite motif containing 33
19.662.868	0.2869615	STAU1	staufen double-stranded RNA binding protein 1
19.657.192	-0.8038264	AGXT	alanine-glyoxylate aminotransferase
19.646.949	0.3819781	STX4	syntaxin 4
19.643.344	-0.1710487	SIGLEC5	sialic acid binding Ig like lectin 5
19.636.968	-0.3052722	TBX5	T-box 5
19.632.313	0.2972951	APEX2	apurinic/apyrimidinic endodeoxyribonuclease 2
19.630.932	-0.7086934	GSPT2	G1 to S phase transition 2
19.619.938	-0.2603557	PTPN18	protein tyrosine phosphatase, non-receptor typ
19.614.514	-0.2545825	HTR4	5-hydroxytryptamine receptor 4
19.611.962	0.4781754	ZNF239	zinc finger protein 239
19.611.274	0.4245835	PMS1	PMS1 homolog 1, mismatch repair system com
19.606.654	-0.5142127	MAP7	microtubule associated protein 7
19.599.765	0.2151255	DDX54	DEAD-box helicase 54
19.587.913	-0.8612902	JUN	Jun proto-oncogene, AP-1 transcription factor s
19.583.325	0.2495489	TLK1	tousled like kinase 1
1.958.169	-0.5233289	ENOSF1	enolase superfamily member 1
19.577.553	0.5921324	VDAC1	voltage dependent anion channel 1
19.546.826	-0.4535117	NUP210	nucleoporin 210
19.540.765	-0.1850352	LOC55338	uncharacterized LOC55338
1.953.973	0.3201186	RPL12	ribosomal protein L12
1.953.838	0.3260796	SCARA3	scavenger receptor class A member 3
19.537.614	-0.3234856	MYO1C	myosin IC
19.537.318	-0.3019413	FCMR	Fc fragment of IgM receptor
19.533.208	-0.1622327		

19.530.481	0.3834367		ARHGEF18	Rho/Rac guanine nucleotide exchange factor 18
19.529.936	0.4457939		RABGGTB	Rab geranylgeranyltransferase beta subunit
19.527.671	-0.3568103		MUM1	melanoma associated antigen (mutated) 1
19.527.219	0.3029953		GOSR1	golgi SNAP receptor complex member 1
19.507.455	-0.3472574		IRF2	interferon regulatory factor 2
19.487.897	-0.4486014		ZC3H12A	zinc finger CCCH-type containing 12A
19.481.643	-0.1760367		GIF	gastric intrinsic factor
19.480.381	-0.2028241		TNPO2	transportin 2
19.470.626	-0.2567413		EFNB3	ephrin B3
19.468.853	0.2545027		NEIL3	nei like DNA glycosylase 3
19.461.875	-0.514413		SOWAHC	sosondowah ankyrin repeat domain family men
19.458.874		-11.472.267		
19.458.227	0.3842543		SSX2IP	SSX family member 2 interacting protein
19.450.118	-0.4155399		GLOD4	glyoxalase domain containing 4
19.440.771	-0.1817135		PDSS2	prenyl (decaprenyl) diphosphate synthase, sub
19.427.001	0.5262836		TES	testin LIM domain protein
19.419.953	-0.1625984		TNMD	tenomodulin
19.415.814	-0.1844232			
1.941.331	0.3670549		RASGRP3	RAS guanyl releasing protein 3
19.411.976	0.4630582		ATP6V1D	ATPase H+ transporting V1 subunit D
19.410.518	-0.1990444		SLC5A1	solute carrier family 5 member 1
19.399.078	0.4015402		PKP4	plakophilin 4
19.384.504	0.2034971		STK17A	serine/threonine kinase 17a
19.376.414	-0.2456061		PKN2	protein kinase N2
1.936.907	-0.6366602		HLA-C	major histocompatibility complex, class I, C
19.360.254	-0.3653292		FAM162A	family with sequence similarity 162 member A
19.351.667	0.3582073		RAB3B	RAB3B, member RAS oncogene family
19.349.191	-0.1598873		APBA1	amyloid beta precursor protein binding family A
1.932.281	0.4634511		SNX3	sorting nexin 3
1.932.019	-0.223821		KCNN1	potassium calcium-activated channel subfamil
19.311.737	0.4150519		DOPEY2	dopey family member 2
19.309.763	-0.4176576		ZEB2	zinc finger E-box binding homeobox 2
1.930.866	-0.232551		GTPBP1	GTP binding protein 1
19.305.771	-0.2045729		SLC34A1	solute carrier family 34 member 1
19.297.887	0.3995735		RPL17P16/	ribosomal protein L17 pseudogene 16///ribosor
19.288.237	-0.387397		LPXN	leupaxin
19.278.362	0.3846758		PMVK	phosphomevalonate kinase
19.277.107	0.5752757		MYCN	v-myc avian myelocytomatosis viral oncogene r
19.276.454	0.2227637		PIAS2	protein inhibitor of activated STAT 2
19.274.635	-0.2896543		JMJD7-PLA2	JMJD7-PLA2G4B readthrough
1.926.798	0.4140452		ATP11B	ATPase phospholipid transporting 11B (putative
19.265.885	-0.3070897		ANKRD10	ankyrin repeat domain 10
19.242.055	0.4411843		PREPL	prolyl endopeptidase-like
19.240.718	-0.2121835		EXPH5	exophilin 5

19.225.761	-0.3991141		TAB2	TGF-beta activated kinase 1/MAP3K7 binding pr
19.223.198	0.3602135		HNRNPL	heterogeneous nuclear ribonucleoprotein L
19.208.871	-0.1946436		TMEM257	transmembrane protein 257
19.204.377	0.3456446		TSR1	TSR1, ribosome maturation factor
19.195.876	-0.2894931		SIRT2	sirtuin 2
1.919.311	0.6579894		LOC65434	lymphocyte-specific protein 1 pseudogene
19.189.212	-0.1896366		CAMK2A	calcium/calmodulin dependent protein kinase
19.186.992	0.3715906		SLC25A44	solute carrier family 25 member 44
19.172.372	0.6390913		PLA2G7	phospholipase A2 group VII
19.168.301	-0.1603414		HBQ1	hemoglobin subunit theta 1
19.159.639		19.094.974	DLK1	delta like non-canonical Notch ligand 1
19.155.723	-0.2736412		PVRIG	poliovirus receptor related immunoglobulin dor
19.154.245	-0.2285864		CPNE7	copine 7
19.152.426	-0.2317899		IGHA1	immunoglobulin heavy constant alpha 1
19.150.527	0.4046678		BRD8	bromodomain containing 8
19.136.345	0.1437353		DCAF17	DDB1 and CUL4 associated factor 17
19.131.472	-0.2011225		ZNF214	zinc finger protein 214
19.129.694	-0.6765018		ABCC3	ATP binding cassette subfamily C member 3
19.120.094	0.369463		OSGIN2	oxidative stress induced growth inhibitor family
19.108.489	0.4089298		GLS	glutaminase
19.106.609	0.4028058		EFCAB14	EF-hand calcium binding domain 14
19.097.271	-0.1691488		TCEB3B	transcription elongation factor B subunit 3B
19.095.723	-0.5113912		PKD2	polycystin 2, transient receptor potential cation
1.909.155	-0.196982		DNAH2	dynein axonemal heavy chain 2
19.087.711	0.3884094		ATG101	autophagy related 101
19.081.058	-0.5925587		ABHD2	abhydrolase domain containing 2
19.077.238	-0.6075005		DICER1	dicer 1, ribonuclease III
19.075.051	0.3702589		ATP5F1	ATP synthase, H+ transporting, mitochondrial F
19.063.186	0.3934357		SMN2///SM	survival of motor neuron 2, centromeric///surviv
19.055.835	-0.1587641		TRIM29	tripartite motif containing 29
19.050.417	-0.1610367		MYL4	myosin light chain 4
19.050.049	-0.1711909		TNFRSF9	TNF receptor superfamily member 9
19.043.227	0.3996508		PRPSAP1	phosphoribosyl pyrophosphate synthetase asso
19.041.723	-0.4381507		OXT	oxytocin/neurophysin I prepropeptide
19.032.154	-0.4468751		CCPG1	cell cycle progression 1
19.030.795	-0.2333538		TGM5	transglutaminase 5
19.018.703	-0.3798542		IFT88	intraflagellar transport 88
19.013.013	-0.2296943		GVINP1	GTPase, very large interferon inducible pseudog
19.008.918	0.3926299		ARFIP2	ADP ribosylation factor interacting protein 2
19.007.791	-0.2376755		ADRB3	adrenoceptor beta 3
19.001.518	-0.3534388		GPLD1	glycosylphosphatidylinositol specific phosphol
18.999.638	0.2966018		FANCA	Fanconi anemia complementation group A
1.899.478	-0.2089867		PDE4D	phosphodiesterase 4D
18.980.861	-0.2123554		RHBDD3	rhomboid domain containing 3

18.975.925	0.3552076		MRPS33	mitochondrial ribosomal protein S33
18.973.703	0.3037622		DUSP8	dual specificity phosphatase 8
18.960.962	0.3326458		USP16	ubiquitin specific peptidase 16
18.959.918	-0.4130983		EGR3	early growth response 3
18.956.084	0.2664248		FARSA	phenylalanyl-tRNA synthetase alpha subunit
18.953.153	0.6393482		BEX3	brain expressed X-linked 3
18.938.168	-0.2308277		OXNAD1	oxidoreductase NAD binding domain containing
18.933.421	0.2490503		B4GALT7	beta-1,4-galactosyltransferase 7
1.891.947	-0.1700666		MTHFR	methylenetetrahydrofolate reductase (NAD(P)H
1.891.722	-0.6164642		HOMER2	homer scaffolding protein 2
18.913.677	0.7358278		VCAN	versican
18.912.816	0.4032645		MRPS18A	mitochondrial ribosomal protein S18A
18.908.561	0.3110313		ITGB1BP1	integrin subunit beta 1 binding protein 1
18.905.372	0.5688604		MCTP1	multiple C2 and transmembrane domain conta
18.904.969	-0.1910466		MT4	metallothionein 4
18.903.221	-0.2417425		SARDH	sarcosine dehydrogenase
18.902.563	-0.1986952		GRK5	G protein-coupled receptor kinase 5
18.894.241	-0.2724337		LST1	leukocyte specific transcript 1
18.893.401	-0.4494678		HSD17B4	hydroxysteroid 17-beta dehydrogenase 4
18.887.936	0.3254401		AKAP8	A-kinase anchoring protein 8
18.884.981	-0.1544973		TFAP2B	transcription factor AP-2 beta
18.871.831	-0.1722473		GRID2	glutamate ionotropic receptor delta type subun
18.870.556	0.218919		C9orf16	chromosome 9 open reading frame 16
18.868.649	-0.4030287		LRP6	LDL receptor related protein 6
18.868.354	0.7384328		GSTM3	glutathione S-transferase mu 3
18.865.746	0.3227297		NSUN5P2	NOP2/Sun RNA methyltransferase family meml
18.857.996	-0.5791444		FGG	fibrinogen gamma chain
18.856.297	-0.4137924		NFATC3	nuclear factor of activated T-cells 3
18.854.378	0.3858897		DDX18	DEAD-box helicase 18
18.849.519	0.25547		HIST1H2BG	histone cluster 1, H2bg
18.843.306	-0.1875564		RGS12	regulator of G-protein signaling 12
18.838.249	-0.1975031		CLDN17	claudin 17
18.827.102	0.8281788		FGF21	fibroblast growth factor 21
18.825.882	0.3821367		CPNE1	copine 1
18.811.976	0.4029233		EIF4E	eukaryotic translation initiation factor 4E
18.809.256		11.713.021	COL2A1	collagen type II alpha 1 chain
18.806.436	-0.5453359		PCCB	propionyl-CoA carboxylase beta subunit
18.805.761	-0.486639		EFEMP2	EGF containing fibulin like extracellular matrix p
18.802.309	-0.2577626		TSNAXIP1	translin associated factor X interacting protein
18.801.432	-0.3029904		SNX9	sorting nexin 9
18.793.983	0.5059777		HIST1H2BE	histone cluster 1, H2be
18.786.813	0.4495372			
18.784.747	-0.2118507		SPANXA2//.	SPANX family member A2///SPANX family mem
18.783.521	0.3067228		ZFP30	ZFP30 zinc finger protein

1.878.293	0.307753	EXOSC2	exosome component 2
18.782.726	0.4070991	POLR3K	RNA polymerase III subunit K
18.780.779	-0.2929209	CD84	CD84 molecule
18.777.825	0.3676007	EXOC3	exocyst complex component 3
18.775.674	0.5891231	HIC2	hypermethylated in cancer 2
18.766.412	0.1968393	RPL22	ribosomal protein L22
18.753.591	0.2788753	ORC5	origin recognition complex subunit 5
18.752.691	0.3282603	SOX13	SRY-box 13
18.751.919	0.2577102	ZNF337	zinc finger protein 337
18.748.591	0.3532636	KTN1	kinectin 1
1.874.586	-0.2520932	GRK2	G protein-coupled receptor kinase 2
18.745.011	-0.2557268	CMC1	C-X9-C motif containing 1
18.743.756	0.3746844	RWDD1	RWD domain containing 1
18.741.194	-0.1897488	CASKIN2	CASK interacting protein 2
18.732.565	0.3805443	PLIN3	perilipin 3
18.725.633	-0.3498997	MKNK1	MAP kinase interacting serine/threonine kinase
18.724.478	0.3170661	TBC1D13	TBC1 domain family member 13
18.721.363	0.3183243	HAUS7	HAUS augmin like complex subunit 7
18.712.652	-0.3569064	SAP18	Sin3A associated protein 18
18.710.942	0.2895437	EGLN2	egl-9 family hypoxia inducible factor 2
1.871.081	-0.1712232	FGFR1	fibroblast growth factor receptor 1
18.710.457	0.2657744	SPTLC2	serine palmitoyltransferase long chain base sul
1.869.711	0.4158882	ACVR1	activin A receptor type 1
18.695.871	0.6282834	IGSF1	immunoglobulin superfamily member 1
18.683.614	0.5687608	MTMR11	myotubularin related protein 11
1.867.297	-0.1524585		
18.667.542	0.3639339	GOLGA7	golgin A7
18.663.905	0.3253889	DIS3	DIS3 homolog, exosome endoribonuclease and
18.663.669	-0.2283219	OSBPL9	oxysterol binding protein like 9
18.659.796	-0.3914996	NAGLU	N-acetyl-alpha-glucosaminidase
18.640.149	-0.1810366	CEACAM3	carcinoembryonic antigen related cell adhesior
18.639.608	-0.4013847	ABCD3	ATP binding cassette subfamily D member 3
18.636.864	-0.1524366	ZZEF1	zinc finger ZZ-type and EF-hand domain contair
1.863.319	0.2827709	RPS3A	ribosomal protein S3A
1.862.338	-0.1879951	HPSE2	heparanase 2 (inactive)
18.622.151	0.3818164	SLC7A11	solute carrier family 7 member 11
18.616.727	-0.4904361		
18.613.898	0.3811073	PLXND1	plexin D1
18.610.726	-0.8697539	ATF3	activating transcription factor 3
18.609.167	-0.2197778	MYOZ1	myozenin 1
18.605.825	0.8436037	SSX2	SSX family member 2
18.605.394	-0.1719469	NRXN1	neurexin 1
18.585.393	-0.1794443	TESPA1	thymocyte expressed, positive selection associ
1.858.398	0.4154084	RNF114	ring finger protein 114

18.569.654	-0.5251175	SALL1	spalt like transcription factor 1
18.555.376	-0.2313761	KIAA1644	KIAA1644
18.553.412	0.4581698	FMR1	fragile X mental retardation 1
18.541.869	0.3314221	UBE2L3	ubiquitin conjugating enzyme E2 L3
18.538.862	0.2849943	TSR3	TSR3, acp transferase ribosome maturation fac
18.533.916	0.3536346	BMS1	BMS1, ribosome biogenesis factor
18.530.057	0.375403	CD200	CD200 molecule
1.852.977	0.3540287	TRAM1	translocation associated membrane protein 1
1.852.466	-0.1784504	IL21R	interleukin 21 receptor
18.523.542	-0.4838601	IRF7	interferon regulatory factor 7
18.519.996	-0.2480551	LDLRAD4	low density lipoprotein receptor class A domair
18.517.148	-0.2943503	PARVA	parvin alpha
185.104	-0.3449133	TRAM2	translocation associated membrane protein 2
18.504.456	-0.1744043	BCL11B///T	B-cell CLL/lymphoma 11B///T cell receptor delt
18.503.505	-0.4024394	DCHS1	dachsous cadherin-related 1
18.501.653	-0.5285243	VTN	vitronectin
18.500.081	0.4054459	POLR3E	RNA polymerase III subunit E
18.488.324	0.3281319	SNX24	sorting nexin 24
18.483.706	0.2889152	NELFA	negative elongation factor complex member A
18.479.484	-0.391331	ERCC5	ERCC excision repair 5, endonuclease
18.473.035	-0.2189717	SPDEF	SAM pointed domain containing ETS transcripti
18.469.508	0.3153811	SLC48A1	solute carrier family 48 member 1
18.468.658	0.4088292	APPL2	adaptor protein, phosphotyrosine interacting w
18.467.451	0.4286426	SLC25A6	solute carrier family 25 member 6
18.466.125	-0.1933678		
18.458.402	-0.2974099	CALD1	caldesmon 1
18.453.515	0.2868613	WIZ	widely interspaced zinc finger motifs
18.450.768	0.3747373	OPA1	OPA1, mitochondrial dynamin like GTPase
18.449.498	0.2437376	RPL18AP5/	ribosomal protein L18a pseudogene 5///ribosor
18.442.788	-0.7865391	GATM	glycine amidinotransferase
18.432.863	-0.2422802	TOM1L2	target of myb1 like 2 membrane trafficking prot
18.432.472	-0.2857057	NME5	NME/NM23 family member 5
18.428.288	-0.1981552		
18.428.004	0.3738306	NOTCH3	notch 3
18.424.118	0.6215469	MCAM	melanoma cell adhesion molecule
18.418.137	-0.1729638	PML	promyelocytic leukemia
18.412.555	0.3391797	TGIF2	TGFB induced factor homeobox 2
18.399.562	0.2474433	TOX4	TOX high mobility group box family member 4
18.395.895	0.5183703	SCCPDH	saccharopine dehydrogenase (putative)
18.384.739	0.3806809	BTAF1	B-TFIID TATA-box binding protein associated fac
18.382.214	-0.1453363	CELF2	CUGBP, Elav-like family member 2
18.376.425	-0.1905994	ADTRP	androgen dependent TFPI regulating protein
18.368.105	-0.2877227	HTR1B	5-hydroxytryptamine receptor 1B
18.358.189	0.3084	HINT1	histidine triad nucleotide binding protein 1

18.357.849	-0.8505349	PAH	phenylalanine hydroxylase
18.357.382	-0.6191028	DPYD	dihydropyrimidine dehydrogenase
18.354.488	0.2956692	LINC00094	long intergenic non-protein coding RNA 94
18.343.828	0.5825034	STAT1	signal transducer and activator of transcription
18.339.844	-0.1962003	PRKCQ	protein kinase C theta
18.330.265	-0.4247765	TNS1	tensin 1
18.322.813	0.5845105	TAP1	transporter 1, ATP binding cassette subfamily B
18.316.094	-0.3303777	USPL1	ubiquitin specific peptidase like 1
18.312.829	-0.2800789	DPH1	diphthamide biosynthesis 1
18.305.787	-0.2216654	PCYT1B///C	phosphate cytidylyltransferase 1, choline, beta
18.304.911	0.297971	BGLAP	bone gamma-carboxyglutamate protein
18.299.869	-0.1852745	MADCAM1	mucosal vascular addressin cell adhesion mole
18.290.093	-0.4008125		
18.289.603	0.3018967	MPHOSPH1	M-phase phosphoprotein 10
18.288.321	-0.1949844	DPP6	dipeptidyl peptidase like 6
18.287.993	-0.1944586	BCAN	brevican
18.286.672	-0.2777225	FAM188A	family with sequence similarity 188 member A
18.278.907	-0.4570003		
1.827.474	0.3767067	UCKL1	uridine-cytidine kinase 1 like 1
18.268.725	0.2387508	SNHG20	small nucleolar RNA host gene 20
18.266.023	0.2660522	PPP6R1	protein phosphatase 6 regulatory subunit 1
18.258.068	-0.2117182	SLC16A5	solute carrier family 16 member 5
18.255.053	-0.1869628		
18.248.613	0.2334932	GEMIN2	gem nuclear organelle associated protein 2
18.244.015	-0.1682051	KCNQ4	potassium voltage-gated channel subfamily Q r
18.243.905	-0.4742798	DBT	dihydrolipoamide branched chain transacylase
18.243.036	-0.2031325		
18.238.078	-0.3010292	DUSP2	dual specificity phosphatase 2
1.823.618	-0.2800841		
18.232.596	0.2024516	FTL	ferritin light chain
18.227.232	0.2548076	PPP2R5D	protein phosphatase 2 regulatory subunit B'delt
18.213.191	-0.2259585	SLC35A2	solute carrier family 35 member A2
18.197.936	0.4018514	MDM1	Mdm1 nuclear protein
18.193.631	-0.1841412	RASL11B	RAS like family 11 member B
18.181.024	-0.2284714	CDH6	cadherin 6
18.176.443	-0.166097	NOCT	nocturnin
18.174.198	-0.1763916	HRH1	histamine receptor H1
18.167.464	-0.4276319	MDN1	midasin AAA ATPase 1
18.166.964	0.4918552	CPQ	carboxypeptidase Q
18.159.209	0.3290952	UBR2	ubiquitin protein ligase E3 component n-recogr
18.154.644	-0.1943346	PMS2P4	PMS1 homolog 2, mismatch repair system com
18.151.593	0.2675795	SKA1	spindle and kinetochore associated complex su
18.139.734	0.5277219	RRAS2	related RAS viral (r-ras) oncogene homolog 2
18.139.164	-0.5276977	CALD1	caldesmon 1

18.126.957	0.541946	COL4A2	collagen type IV alpha 2 chain
18.125.328	0.4364374	RAB2A	RAB2A, member RAS oncogene family
18.118.571	0.5431154	ZBTB18	zinc finger and BTB domain containing 18
18.115.148	-0.2103255	KANSL1L	KAT8 regulatory NSL complex subunit 1 like
18.111.348	-0.6041577	LDLR	low density lipoprotein receptor
18.099.689	0.2910967	GGA2	golgi associated, gamma adaptin ear containing
18.095.594	-0.6194983	CRYM	crystallin mu
18.092.555	0.3048215	SKAP2	src kinase associated phosphoprotein 2
18.090.278	0.3198134	SNUPN	snurportin 1
18.085.331	0.4420236	LMCD1	LIM and cysteine rich domains 1
18.082.307	0.5498813	PSIP1	PC4 and SFRS1 interacting protein 1
18.076.653	0.2835498	SUPT5H	SPT5 homolog, DSIF elongation factor subunit
18.074.332	-0.2563836	CALCOCO1	calcium binding and coiled-coil domain 1
18.069.738	0.3918286	TRPC1	transient receptor potential cation channel sub
18.067.324	-0.2092328	PLK4	polo like kinase 4
18.025.206	-0.5668927	CCNB1IP1	cyclin B1 interacting protein 1
18.023.775	0.3759777	RPL36	ribosomal protein L36
18.022.957	0.5994176	SSX2IP	SSX family member 2 interacting protein
18.022.436	-0.1877214		
18.019.239	-0.1818819	DGCR14	DiGeorge syndrome critical region gene 14
18.015.534	0.3437542	RAPGEF6	Rap guanine nucleotide exchange factor 6
18.010.896	0.6841172	MTHFD2	methylenetetrahydrofolate dehydrogenase (NA
17.996.789	-0.1557715	ITGB3	integrin subunit beta 3
1.798.933	-0.2730731	OTUD4	OTU deubiquitinase 4
17.974.859	0.3738838	NT5C2	5'-nucleotidase, cytosolic II
17.973.832	0.3710308	FIG4	FIG4 phosphoinositide 5-phosphatase
17.973.552	0.3425462	CRTAP	cartilage associated protein
17.971.235	0.5085046	DDX21	DEAD-box helicase 21
17.969.945	-0.1614577	SH2D1A	SH2 domain containing 1A
17.963.903	-0.5726291	GRAMD4	GRAM domain containing 4
17.962.759	0.6101253	ARID5B	AT-rich interaction domain 5B
17.954.045	0.380592	LINC00094	long intergenic non-protein coding RNA 94
17.950.155	0.3925849	NUP50	nucleoporin 50
17.949.848	-0.1315943	CD8B	CD8b molecule
17.940.636	0.2224227	CPSF6	cleavage and polyadenylation specific factor 6
1.794.063	-0.6026703	ANXA6	annexin A6
1.793.661	-0.2058279		
17.928.384	-0.1529288	IL13	interleukin 13
17.926.556	-0.432128	PDE3B	phosphodiesterase 3B
17.906.129	-0.4044761	PDE4DIP	phosphodiesterase 4D interacting protein
17.906.076	-0.3033494	TMEM163	transmembrane protein 163
1.790.149	0.3368245	MBOAT7	membrane bound O-acyltransferase domain co
17.890.131	0.4082278	SEP15	15 kDa selenoprotein
17.885.055	-0.8829954		

178.846	0.3011209	BCL7A	BCL tumor suppressor 7A
17.876.082	-0.3327234		
17.875.524	-0.2147828	PPP2CB	protein phosphatase 2 catalytic subunit beta
17.871.623	-0.1726267	LOC10013:	uncharacterized LOC100131510
1.787.112	0.2241121	PRPF31	pre-mRNA processing factor 31
17.869.256	0.2260603	ZKSCAN5	zinc finger with KRAB and SCAN domains 5
17.861.451	-0.1880174	KLRD1	killer cell lectin like receptor D1
17.858.781	-0.6061377	ACOX1	acyl-CoA oxidase 1
1.785.738	0.7207946	GAPDH	glyceraldehyde-3-phosphate dehydrogenase
17.856.686	0.2202598	TRMU	tRNA 5-methylaminomethyl-2-thiouridylate me
17.847.541	0.3156162	PIGA	phosphatidylinositol glycan anchor biosynthesi
17.839.496	-0.2429059	FXR2	FMR1 autosomal homolog 2
17.834.194	-0.1705673	ZNF362	zinc finger protein 362
1.783.172	-0.177896	DCAF4	DDB1 and CUL4 associated factor 4
17.824.181	0.3862846	BICD2	BICD cargo adaptor 2
17.817.056	-0.180178	TRIM10	tripartite motif containing 10
17.815.383	0.3388438	RBM4B	RNA binding motif protein 4B
17.814.596	0.3384754	OSBPL2	oxysterol binding protein like 2
17.813.182	-0.2109514	HR	hair growth associated
17.809.099	-0.9749094	SERPINA7	serpin family A member 7
17.805.644	0.3810912	C1GALT1	core 1 synthase, glycoprotein-N-acetylgalactos
17.795.431	0.3121252	IGBP1	immunoglobulin (CD79A) binding protein 1
1.779.526	0.2934179	UBA1	ubiquitin like modifier activating enzyme 1
17.791.373	0.3649544	HFE	hemochromatosis
17.782.103	0.4069288	EGLN3	egl-9 family hypoxia inducible factor 3
17.781.998	0.368408	YTHDF2	YTH N6-methyladenosine RNA binding protein 2
17.771.999	0.3410638	SMARCE1	SWI/SNF related, matrix associated, actin depe
17.764.514	0.4322817	HIST1H4C	histone cluster 1, H4c
17.763.944	0.3883058	CSGALNAC	chondroitin sulfate N-acetylgalactosaminyltran
17.753.772	0.4072561	RABGAP1L	RAB GTPase activating protein 1 like
17.745.563	0.267906	UBN1	ubinuclein 1
1.772.232	0.5221119	PECAM1	platelet and endothelial cell adhesion molecule
17.717.764	0.3426232	OGG1	8-oxoguanine DNA glycosylase
17.717.359	-0.1984935	TNFSF12-TI	TNFSF12-TNFSF13 readthrough///tumor necros
17.710.281	0.3460476	ATP5B	ATP synthase, H+ transporting, mitochondrial F
17.701.818	0.2660015	CLASP2	cytoplasmic linker associated protein 2
17.700.134	-0.1998338	FSHB	follicle stimulating hormone beta subunit
17.699.518	-0.3263365	MEF2A	myocyte enhancer factor 2A
17.692.165	-0.2099335		
17.691.278	-0.1391956	CHRNA10	cholinergic receptor nicotinic alpha 10 subunit
17.690.406	-0.4027869	TP53TG1	TP53 target 1 (non-protein coding)
17.686.912	0.4136923	RALGAPA1	Ral GTPase activating protein catalytic alpha su
17.667.816	0.255113	SRBD1	S1 RNA binding domain 1
17.663.528	-0.4754844	HNRNPL	heterogeneous nuclear ribonucleoprotein L

17.662.453	-0.2163608		SIGLEC8	sialic acid binding Ig like lectin 8
17.658.545	-0.2376694			
17.650.544	-0.241077		GRPR	gastrin releasing peptide receptor
17.647.116	0.4016526		PPFIA1	PTPRF interacting protein alpha 1
17.644.275	-0.193943		PDLIM5	PDZ and LIM domain 5
17.639.961	0.2067745		WDHD1	WD repeat and HMG-box DNA binding protein 1
17.623.421	0.5488991		DVL3	dishevelled segment polarity protein 3
17.623.114	0.3406664		SSH1	slingshot protein phosphatase 1
17.616.102	0.4826616		BCAM	basal cell adhesion molecule (Lutheran blood g
17.613.671	-0.7697021		BASP1	brain abundant membrane attached signal prot
17.609.782	-0.2085188		EFCC1	EF-hand and coiled-coil domain containing 1
17.604.011	-0.1700638			
17.589.226		11.158.006	VCAN	versican
17.587.772	0.5379362		EXT1	exostosin glycosyltransferase 1
1.758.623	-0.2021437		HOXB3	homeobox B3
17.584.446	-0.2753221		KCNS1	potassium voltage-gated channel modifier subf
17.581.401	-0.5278775		ABHD6	abhydrolase domain containing 6
17.561.542	0.423199		MPPE1	metallophosphoesterase 1
17.557.747	-0.1583835		RPS6KA6	ribosomal protein S6 kinase A6
17.553.868	-0.4210222		GYPC	glycophorin C (Gerbich blood group)
17.551.051	0.2451439		ATP6V1C1	ATPase H+ transporting V1 subunit C1
17.550.869	-0.2557277		PADI1	peptidyl arginine deiminase 1
17.550.459	-0.2393408		TUB	tubby bipartite transcription factor
17.547.119	0.1653526		CDT1	chromatin licensing and DNA replication factor
1.753.716	-0.5921984		ALDH7A1	aldehyde dehydrogenase 7 family member A1
17.531.218	-0.4719287		MYCL	v-myc avian myelocytomatosis viral oncogene l
17.525.739	-0.1689373		PPFIA2	PTPRF interacting protein alpha 2
17.518.712	-0.1893174		PHYHIP	phytanoyl-CoA 2-hydroxylase interacting protei
17.518.411	0.3152775		PDS5A	PDS5 cohesin associated factor A
17.512.287	0.55263		STAT1	signal transducer and activator of transcription
17.506.203	-0.7797014		ALB	albumin
1.750.142	0.3355297		RAB11FIP2	RAB11 family interacting protein 2
17.499.397	-0.5187678		TOB1	transducer of ERBB2, 1
17.499.028	0.4128199		MCAM	melanoma cell adhesion molecule
17.482.059	-0.1812582		GIMAP5	GTPase, IMAP family member 5
17.477.139	-0.1833552		AKAP4	A-kinase anchoring protein 4
17.471.486	-0.1687038		RPGRIP1	retinitis pigmentosa GTPase regulator interactir
17.469.433	-0.7079453		GGT1	gamma-glutamyltransferase 1
17.464.999	-0.7358177		AMACR	alpha-methylacyl-CoA racemase
17.437.061	0.3489715		REST	RE1 silencing transcription factor
17.435.974	0.4509634		PDIA4	protein disulfide isomerase family A member 4
17.426.627	-0.1914507		C1orf21	chromosome 1 open reading frame 21
17.419.505	-0.2088041		PSKH1	protein serine kinase H1
17.411.414	-0.1478159		RARB	retinoic acid receptor beta

17.397.415	-0.1602233	PGM5	phosphoglucomutase 5
1.739.414	0.3328802	ASXL1	additional sex combs like 1, transcriptional regu
17.384.365	-0.3200514	PDZRN3	PDZ domain containing ring finger 3
1.738.361	-0.5660413	CELF2	CUGBP, Elav-like family member 2
17.383.037	0.3307081	NEMF	nuclear export mediator factor
1.737.039	0.3715034	DAZAP1	DAZ associated protein 1
17.366.898	-0.436923	IL1R1	interleukin 1 receptor type 1
17.362.066	-0.2678767	TNFRSF10E	TNF receptor superfamily member 10b
17.361.717	-0.4019721	GOLGA8EP	golgin A8 family member E, pseudogene///golgi
17.361.504	-0.1596062	ECRP///RN	ribonuclease A family member 2 pseudogene//
17.353.468	-0.1583954	TLR6	toll like receptor 6
17.352.872	-0.2501732	GNG7	G protein subunit gamma 7
17.334.802	-0.4374827	LINC01558	long intergenic non-protein coding RNA 1558
17.321.419	0.2154654	ZNF112	zinc finger protein 112
17.321.009	-0.4503755	CLEC7A	C-type lectin domain family 7 member A
17.320.161	0.6313238	SLC16A3	solute carrier family 16 member 3
17.317.773	0.7056966	HIST1H2BK	histone cluster 1, H2bk
17.314.958	0.4103999	C1orf174	chromosome 1 open reading frame 174
17.296.495	-0.2416886	DDR1-AS1	DDR1 antisense RNA 1 (head to head)
17.283.691	0.413483	PI4KAP1///I	phosphatidylinositol 4-kinase alpha pseudogen
17.267.664	0.3027967	STX18	syntaxin 18
17.252.637	-0.1807928	MYOZ3	myozenin 3
17.238.412	0.3883394	RAB2A	RAB2A, member RAS oncogene family
17.232.184	-0.1812659	CHRND	cholinergic receptor nicotinic delta subunit
17.229.859	0.4386775	OGT	O-linked N-acetylglucosamine (GlcNAc) transfe
17.220.205	0.4479185	WBP11	WW domain binding protein 11
17.212.147	0.5971356	FRRS1L	ferric chelate reductase 1 like
17.211.084	-0.215482	MGEA5	meningioma expressed antigen 5 (hyaluronidas
17.208.299	-0.1564018	ANKRD53	ankyrin repeat domain 53
17.200.615	0.4005499	CBX5	chromobox 5
17.199.658	0.1611803	GTSE1	G2 and S-phase expressed 1
17.199.109	-0.194754	AKAP5	A-kinase anchoring protein 5
17.196.207	0.3160456	BRCC3	BRCA1/BRCA2-containing complex subunit 3
17.186.909	0.7695412	NRCAM	neuronal cell adhesion molecule
17.176.201	0.2871426	SLC48A1	solute carrier family 48 member 1
17.173.942	-0.4724314	LRRC32	leucine rich repeat containing 32
17.166.805	-0.7456213	SF1	splicing factor 1
1.715.862	0.3446997	NPEPPS	aminopeptidase puromycin sensitive
17.157.607	-0.1563617	CLC	Charcot-Leyden crystal galectin
17.157.085	0.1829275	LOC73002	eukaryotic translation initiation factor 3 subuni
17.152.746	-0.3461366	SPON1	spondin 1
17.136.308	-0.1925227	MVB12B	multivesicular body subunit 12B
17.130.652	0.3821722	FLNB	filamin B
17.129.231	0.3561096	RPS3AP5//	ribosomal protein S3A pseudogene 5///ribosom

17.102.494	0.4105259	KDELR2	KDEL endoplasmic reticulum protein retention
17.100.285	-0.4391458	PGRMC1	progesterone receptor membrane component 1
17.091.336	-0.3181586	THEMIS2	thymocyte selection associated family member
17.090.946	0.4359842	ZC3H3	zinc finger CCCH-type containing 3
17.086.465	-0.2332921	ZMYM6	zinc finger MYM-type containing 6
17.083.938	0.6686038	MEIS2	Meis homeobox 2
17.079.119	-0.1536352	S100A5	S100 calcium binding protein A5
17.077.968	-0.1552746	AKAP13	A-kinase anchoring protein 13
17.075.498	-0.1442648	ZNF81	zinc finger protein 81
17.065.477	0.4362785	HSPA1A	heat shock protein family A (Hsp70) member 1A
17.064.368	0.54935	FERMT1	fermitin family member 1
17.058.096	-0.5191537	TNS1	tensin 1
17.048.097	-0.1864295	SNAI1	snail family transcriptional repressor 1
17.035.112	-0.1765877	TEKT2	tektin 2
17.034.158	-0.2411267	WBSCR23	Williams-Beuren syndrome chromosome region
1.702.435	-0.1935284	IGHG1	immunoglobulin heavy constant gamma 1 (G1m
170.174	-0.3223934	TNFSF12-TI	TNFSF12-TNFSF13 readthrough///tumor necros
17.014.363	-0.2937716	CDC42EP3	CDC42 effector protein 3
17.012.343	-0.5014278	BPHL	biphenyl hydrolase like
17.011.378	-0.1212083	LRTM1	leucine rich repeats and transmembrane doma
17.009.353	0.310067	DDX56	DEAD-box helicase 56
17.005.504	-0.1783949	SOS2	SOS Ras/Rho guanine nucleotide exchange fac
16.997.313	0.3895094	PPRC1	peroxisome proliferator-activated receptor gam
16.997.211	0.2928424	NPM3	nucleophosmin/nucleoplasmin 3
16.996.608	0.2635388	GTF2F1	general transcription factor IIF subunit 1
16.993.137	0.3615936	ANKHD1	ankyrin repeat and KH domain containing 1
16.992.587	0.2615475	BCR	BCR, RhoGEF and GTPase activating protein
16.989.396	-0.3484147	PLAUR	plasminogen activator, urokinase receptor
16.976.527	0.461311	TPTE///PCN	transmembrane phosphatase with tensin homo
16.972.318	0.5109242	CABYR	calcium binding tyrosine phosphorylation regul
16.969.409	-0.5306071	FCGR3B///I	Fc fragment of IgG receptor IIIb///Fc fragment o
16.966.697	-0.2086247	DYRK1A	dual specificity tyrosine phosphorylation regula
16.961.187	0.2604931	AGGF1	angiogenic factor with G-patch and FHA domai
16.959.983	0.3254274	PLAGL2	PLAG1 like zinc finger 2
16.959.855	0.1864511	LOC10334	uncharacterized LOC103344931
16.952.319	0.2909702	ZNF137P	zinc finger protein 137, pseudogene
16.940.524	-0.1502251	SYDE1	synapse defective Rho GTPase homolog 1
16.935.759	-0.1549052	DCC	DCC netrin 1 receptor
16.927.849	-0.1468754	TMEM220	transmembrane protein 220
16.927.523	-0.4595848	PCBP2	poly(rC) binding protein 2
16.927.267	0.2280897	FKBP14	FK506 binding protein 14
16.923.826	0.3159709	SERINC3	serine incorporator 3
16.917.651	-0.2231428	PTOV1-AS2	PTOV1 antisense RNA 2
16.917.461	0.3676946	PSME4	proteasome activator subunit 4

16.912.952	-0.4368134		GCAT	glycine C-acetyltransferase
1.690.909	0.2386065		NOP56	NOP56 ribonucleoprotein
16.902.244	-0.2601375			
16.898.169	-0.1617612		PPP2R3A	protein phosphatase 2 regulatory subunit B''alp
16.889.609	-0.2325719		LIMD2	LIM domain containing 2
16.884.971	0.2343253		CEP63	centrosomal protein 63
16.863.327	-0.1554128		NEU3	neuraminidase 3
16.858.076	-0.2832803		SRSF8	serine and arginine rich splicing factor 8
16.853.616	0.4262822		SCAF8	SR-related CTD associated factor 8
16.841.244	-0.1873495		ADRA2B	adrenoceptor alpha 2B
16.826.683	-0.1906199		APC2	APC2, WNT signaling pathway regulator
16.825.598	-0.1621653		NTNG1	netrin G1
16.822.938	-0.2354022		TAL1	TAL bHLH transcription factor 1, erythroid differ
16.819.614	0.2557282		EWSR1	EWS RNA binding protein 1
16.805.497		-12.940.805	DEFB1	defensin beta 1
16.803.975	0.4944358		PKP4	plakophilin 4
16.803.251	0.4086112		SPAG4	sperm associated antigen 4
16.798.354	-0.3593628		RPS6KA2	ribosomal protein S6 kinase A2
16.791.714	0.2877597		MED1	mediator complex subunit 1
16.785.481	-0.3852932		STAT6	signal transducer and activator of transcription
16.784.944	0.3261389		TBK1	TANK binding kinase 1
16.782.886	0.4434864		TIMM13	translocase of inner mitochondrial membrane [illegible]
1.678.063	-0.3730327		METRN	meteorin, glial cell differentiation regulator
1.676.927	-0.4584235		LCP2	lymphocyte cytosolic protein 2
16.763.475	0.5350715		PRSS3	protease, serine 3
16.761.065	0.3075967		FBXL6	F-box and leucine rich repeat protein 6
16.750.917	-0.1735878		NR4A1	nuclear receptor subfamily 4 group A member 1
16.750.705	0.3042832		PIK3C3	phosphatidylinositol 3-kinase catalytic subunit
16.732.704	0.2094934		VPS33A	VPS33A, CORVET/HOPS core subunit
167.246	0.3950831		PARVB	parvin beta
16.722.193	-0.1956902			
16.721.402	-0.4235058		CREBL2	cAMP responsive element binding protein like 2
16.712.117	0.2155188		NCAPH2	non-SMC condensin II complex subunit H2
1.671.193	-0.4273471		ISLR	immunoglobulin superfamily containing leucine
16.706.571	-0.426078		PRCP	prolylcarboxypeptidase
16.705.506	0.4511826		UPF3A	UPF3 regulator of nonsense transcripts homolo
16.701.975	0.4643204		H2AFY2	H2A histone family member Y2
16.699.791	-0.2910263		LMF1	lipase maturation factor 1
16.689.786	-0.1638007		JAK3	Janus kinase 3
16.688.969	0.1507282		COPS8	COP9 signalosome subunit 8
16.685.023	-0.4664339		AIF1	allograft inflammatory factor 1
16.681.141	0.2201999		PROSER1	proline and serine rich 1
16.676.314	-0.4166096		TTC31	tetratricopeptide repeat domain 31
16.669.121	0.2251734		WHSC1	Wolf-Hirschhorn syndrome candidate 1

16.668.679	0.3581497	DHRS11	dehydrogenase/reductase 11
16.665.812	0.2984225	NFRKB	nuclear factor related to kappaB binding protei
16.664.038	-0.2448558	UNC13A	unc-13 homolog A
16.650.577	-0.19426	ADCY6	adenylate cyclase 6
16.644.171	-0.743578	GGT1	gamma-glutamyltransferase 1
16.642.549	0.4140263	UBE2K	ubiquitin conjugating enzyme E2 K
16.640.914	0.2160224	FKBP1A	FK506 binding protein 1A
16.639.602	0.3297332		
16.639.213	-0.1565345	RAD23BP1/	RAD23 homolog B, nucleotide excision repair p
16.632.804	-0.1298593	KRTAP9-9	keratin associated protein 9-9
16.630.184	0.3766713	CDC16	cell division cycle 16
16.627.756	0.1957498	EIF1	eukaryotic translation initiation factor 1
16.624.237	-0.1818199	SLC26A1	solute carrier family 26 member 1
16.616.207	-0.4026335	DNMBP	dynamin binding protein
16.611.837	-0.1683904	CASR	calcium sensing receptor
16.592.306	-0.4581625	INPP1	inositol polyphosphate-1-phosphatase
165.887	0.3294191	CUL1	cullin 1
1.658.713	-0.4143423	ASMTL	acetylserotonin O-methyltransferase-like
16.562.996	-0.2045366	CC2D1A	coiled-coil and C2 domain containing 1A
16.560.539	0.155089	HNRNPA1P	heterogeneous nuclear ribonucleoprotein A1 ps
16.559.767	-0.261829	COL6A1	collagen type VI alpha 1 chain
16.558.619	0.3166882	HUS1	HUS1 checkpoint clamp component
16.554.019	0.4220766	WDR1	WD repeat domain 1
16.547.483	-0.1292299	CFTR	cystic fibrosis transmembrane conductance re
16.542.894	0.2664339	MAP2K2	mitogen-activated protein kinase kinase 2
16.542.304	0.4331277	CERS6	ceramide synthase 6
16.542.007	-0.3709472	ADAP2	ArfGAP with dual PH domains 2
16.530.456	0.8982312	SOX9	SRY-box 9
16.528.554	-0.2040458		
16.523.996	0.6209869	VWF	von Willebrand factor
1.652.064	0.2597209	RGP1	RGP1 homolog, RAB6A GEF complex partner 1
16.519.875	-0.230621	SOX10	SRY-box 10
1.651.833	-0.1607861	TAPT1	transmembrane anterior posterior transformati
16.514.821	0.4690526	OPTN	optineurin
16.513.729	-0.2056185	KDM4B	lysine demethylase 4B
16.512.903	0.3667447	KIAA0368	KIAA0368
16.504.435	0.3170682	LUC7L3	LUC7 like 3 pre-mRNA splicing factor
16.501.165	-0.1865321	DCBLD2	discoidin, CUB and LCCL domain containing 2
1.649.508	0.2957126	WWP2	WW domain containing E3 ubiquitin protein liga
16.491.232	-0.2354548	SARDH	sarcosine dehydrogenase
16.490.951	0.2846802	CNOT8	CCR4-NOT transcription complex subunit 8
16.490.024	-0.1410262	DPYSL4	dihydropyrimidinase like 4
16.489.342	-0.1908553	ST3GAL1	ST3 beta-galactoside alpha-2,3-sialyltransferas
16.485.861	-0.169462	LOC10272	putative V-set and immunoglobulin domain-cor

16.485.338	0.3267336	RNF111	ring finger protein 111
16.485.176	0.3997101	ZDHHC3	zinc finger DHHC-type containing 3
16.482.455	-0.6355972	GAS2	growth arrest specific 2
1.647.679	-0.1714533	RIN3	Ras and Rab interactor 3
16.476.671	0.3772782	UHRF1BP1	UHRF1 binding protein 1 like
16.461.345	-0.152656	KAT6B	lysine acetyltransferase 6B
16.452.688	-0.2678237	GLP1R	glucagon like peptide 1 receptor
1.645.072	-0.1707211	MTHFR	methylenetetrahydrofolate reductase (NAD(P)H
16.441.813	-0.5541145	AHSG	alpha 2-HS glycoprotein
16.437.683	-0.6697169	IGLL3P	immunoglobulin lambda like polypeptide 3, pse
16.429.727	0.3725752	LANCL2	LanC like 2
16.418.474	-0.1936586	SIPA1L3	signal induced proliferation associated 1 like 3
16.416.314	-0.1495128	TRAF3IP3	TRAF3 interacting protein 3
16.413.859	-0.1627465	AD7C-NTP	neuronal thread protein AD7c-NTP
16.412.911	-0.2085893	AK1	adenylate kinase 1
16.403.414	-0.3480597	MASP1	mannan binding lectin serine peptidase 1
1.638.984	-0.1348334	PDE10A	phosphodiesterase 10A
16.386.114	0.764907	TESC	tescalcin
16.384.584	-0.8348237	TF	transferrin
16.382.531	0.2742369	CCNF	cyclin F
16.381.497	0.1596346	ITGB1	integrin subunit beta 1
16.379.957	0.2218827	CORO1B	coronin 1B
16.361.264	0.3978594	CASP8	caspase 8
16.361.253	-0.2075079		
16.358.442	0.5869969	EML4	echinoderm microtubule associated protein lik
1.635.284	0.1417486	LOC73079	similar to peptidylprolyl isomerase A isoform 1/
1.634.259	-0.1429218		
16.333.439	0.2367148	RTF1	RTF1 homolog, Paf1/RNA polymerase II comple
16.333.375	0.2420882	ZNF764	zinc finger protein 764
16.332.022	-0.2481212	TOMM40	translocase of outer mitochondrial membrane
1.632.626	-0.1841021	LARP7	La ribonucleoprotein domain family member 7
16.326.122	0.3076005	FGD6	FYVE, RhoGEF and PH domain containing 6
1.632.052	-0.5447316	GC	GC, vitamin D binding protein
16.316.556	-0.1562488	FAM134B	family with sequence similarity 134 member B
1.630.891	-0.2984632	HOXB2	homeobox B2
16.302.862	0.3757565	LOC44043	aminopeptidase puromycin sensitive pseudoge
16.302.274	-0.6605259	SLC38A3	solute carrier family 38 member 3
1.629.055	0.3778109	DCUN1D2	defective in cullin neddylation 1 domain contai
16.289.563	0.2825234		
16.286.077	0.2972806	PGAP2	post-GPI attachment to proteins 2
16.282.573	0.3733347	REXO2	RNA exonuclease 2
16.281.631	-0.1743752		
16.275.446	-0.2594778	PRR5	proline rich 5
16.273.793	-0.1434544		

1.626.637	-0.2468648	WDR59	WD repeat domain 59
16.262.998	-0.1428083	ADAM12	ADAM metallopeptidase domain 12
16.261.862	-0.1355831	SYK	spleen associated tyrosine kinase
16.255.108	-0.1646862	ARHGEF15	Rho guanine nucleotide exchange factor 15
16.251.553	-0.5172529	KHK	ketohexokinase
16.248.643	-0.2294207	MS4A5	membrane spanning 4-domains A5
16.245.896	0.2768289	BICD1	BICD cargo adaptor 1
16.245.847	-0.1406083	CPNE6	copine 6
16.236.718	0.7157182	ACKR3	atypical chemokine receptor 3
16.235.159	0.376052	RPL28	ribosomal protein L28
16.231.478	-0.6756501	GLDC	glycine decarboxylase
16.221.744	-0.1675595	KCNQ1	potassium voltage-gated channel subfamily Q r
16.221.393	-0.1393156	XRCC6	X-ray repair cross complementing 6
1.621.849	0.5878653	DHRS7	dehydrogenase/reductase 7
16.218.372	0.2618152	PPIL2	peptidylprolyl isomerase like 2
16.215.282	0.1864924	RNF24	ring finger protein 24
16.210.103	-0.2079945	IL1RAPL2	interleukin 1 receptor accessory protein like 2
16.193.982	0.3335826	MRPS18B	mitochondrial ribosomal protein S18B
16.189.087	-0.1480873	TRA	T-cell receptor alpha locus
16.187.222	0.3220675	DRG2	developmentally regulated GTP binding protein
16.186.735	-0.2569027	YTHDC1	YTH domain containing 1
16.182.742	-0.6959956	CFH	complement factor H
16.182.451	0.2308274	TBC1D22A	TBC1 domain family member 22A
16.180.767	0.4453101	GLUL	glutamate-ammonia ligase
1.616.246	0.5882877	TMX1	thioredoxin related transmembrane protein 1
16.160.987	-0.1933435	ZNF407	zinc finger protein 407
1.615.151	-0.1535416	CCL17	C-C motif chemokine ligand 17
16.144.769	-0.2548279	ERMAP	erythroblast membrane associated protein (Sci
16.144.415	0.5510851	HSPA1B///I	heat shock protein family A (Hsp70) member 1E
16.138.309	0.3243541	ELMO2	engulfment and cell motility 2
16.136.944	-0.5505651	SLC5A3	solute carrier family 5 member 3
16.136.848	-0.2175331	KLRD1	killer cell lectin like receptor D1
1.613.555	0.205343	PAK1	p21 (RAC1) activated kinase 1
16.135.043	0.3922086	FOXA1	forkhead box A1
16.132.484	-0.2647498	CD6	CD6 molecule
16.125.417	-0.1296931	ELN	elastin
16.124.243	0.2543266	CDC42	cell division cycle 42
16.118.562	0.4061533	LYN	LYN proto-oncogene, Src family tyrosine kinase
16.117.891	0.2823064	RP2	retinitis pigmentosa 2 (X-linked recessive)
16.115.686	-0.223686	C5orf45	chromosome 5 open reading frame 45
16.112.135	0.2367321	MTMR14	myotubularin related protein 14
16.111.958	0.3302087	HOXD4	homeobox D4
16.110.479	0.2194312	GM2A	GM2 ganglioside activator
16.101.159	0.4280536	ATOX1	antioxidant 1 copper chaperone

16.100.577	0.2696221	SMYD5	SMYD family member 5
1.608.895	-0.4956687	GCLC	glutamate-cysteine ligase catalytic subunit
16.073.486	0.2482398	XPO6	exportin 6
16.063.076	0.3106651	POMP	proteasome maturation protein
16.060.125	-0.1833193	SLC5A2	solute carrier family 5 member 2
16.056.675	0.3516803	MIEF1	mitochondrial elongation factor 1
16.056.473	0.5019242	NCF2	neutrophil cytosolic factor 2
16.054.398	0.4918361	SYNCRIP	synaptotagmin binding cytoplasmic RNA intera
16.039.563	0.257316	TMEFF1	transmembrane protein with EGF like and two f
16.035.207	-0.1439205	PTK2B	protein tyrosine kinase 2 beta
16.027.935	0.2299914	SGSH	N-sulfoglucosamine sulfohydrolase
16.023.343	0.3204204	TARBP2	TARBP2, RISC loading complex RNA binding sul
16.016.892	-0.1910673	OSBPL10	oxysterol binding protein like 10
16.008.639	0.3160841	HNRNPL	heterogeneous nuclear ribonucleoprotein L
16.003.526	-0.2030144	BAZ1B	bromodomain adjacent to zinc finger domain 1
15.998.245	-0.3726508	ZZEF1	zinc finger ZZ-type and EF-hand domain contai
1.599.049	-0.2971745	KLK11	kallikrein related peptidase 11
15.986.238	0.3099888	PSEN1	presenilin 1
15.984.895	-0.1189265	SIGLEC8	sialic acid binding Ig like lectin 8
15.974.578	-0.1655202	POU6F2	POU class 6 homeobox 2
15.973.902	-0.1658055	KIR3DL2///	killer cell immunoglobulin like receptor, three I
15.964.973	-0.3708615	SCAF4	SR-related CTD associated factor 4
15.953.815	-0.1944195	LZTS1	leucine zipper tumor suppressor 1
15.946.182	-0.3496559	RHNO1	RAD9-HUS1-RAD1 interacting nuclear orphan 1
15.943.133	0.3351642	PRRC2A	proline rich coiled-coil 2A
15.942.512	0.4554565	EIF1AX	eukaryotic translation initiation factor 1A, X-link
15.930.405	-0.1808885	SCNN1A	sodium channel epithelial 1 alpha subunit
15.927.131	0.198557	TMEM8B	transmembrane protein 8B
15.923.246	-0.6190353	EVI2B	ecotropic viral integration site 2B
15.917.027	-0.4645709	CREM	cAMP responsive element modulator
15.901.672	-0.745981	BST2	bone marrow stromal cell antigen 2
15.897.636	0.2428589		
15.887.937	0.8952732	SSX4B///SS	SSX family member 4B///SSX family member 4
15.879.793	-0.1941848	PRSS1	protease, serine 1
15.877.879	0.2913086	EIF4A2	eukaryotic translation initiation factor 4A2
15.869.358	-0.3334365	NCAM1	neural cell adhesion molecule 1
15.869.092	-0.2006716	ACSL6	acyl-CoA synthetase long-chain family member
15.862.607	-0.4450301	PDIA5	protein disulfide isomerase family A member 5
15.858.597	0.2191197	RNPS1	RNA binding protein with serine rich domain 1
15.857.655	0.182733	ASAP1	ArfGAP with SH3 domain, ankyrin repeat and P
15.852.724	0.5223681	ELK3	ELK3, ETS transcription factor
15.848.773	0.5063132	SEPT2	septin 2
15.839.903	0.1652794	C9orf40	chromosome 9 open reading frame 40
15.839.027	-0.3814618	GPRC5C	G protein-coupled receptor class C group 5 me

1.583.733	0.514869	PLEKHB2	pleckstrin homology domain containing B2
15.836.423	-0.3569351	IL1RN	interleukin 1 receptor antagonist
15.830.412	0.3346214	TBCD	tubulin folding cofactor D
15.825.858	0.2189845	ITGB5	integrin subunit beta 5
15.825.001	0.2593902	BCL7B	BCL tumor suppressor 7B
15.824.906	0.4342895	NMI	N-myc and STAT interactor
15.820.267	-0.1209584		
15.819.549	0.4749104	SNX2	sorting nexin 2
1.581.869	-0.3745827	TCEB3	transcription elongation factor B subunit 3
15.817.451	0.4181623	TM2D1	TM2 domain containing 1
15.808.925	-0.1889278	UNC5B	unc-5 netrin receptor B
15.798.371	-0.1655294	TEX13B	testis expressed 13B
15.798.339	-0.1313618	CACNA1G	calcium voltage-gated channel subunit alpha1
15.798.126	-0.3139713	SURF1	surfeit 1
15.794.133	0.3360457	RAB1A	RAB1A, member RAS oncogene family
15.792.566	-0.2197687	PRMT2	protein arginine methyltransferase 2
15.792.339	0.3414016	MAP7D1	MAP7 domain containing 1
15.779.198	-0.1520685	GGA2	golgi associated, gamma adaptin ear containin
15.772.494	0.5075426	RPL18AP3/	ribosomal protein L18a pseudogene 3///ribosor
15.771.515	-0.6555783	KLF6	Kruppel like factor 6
15.769.181	0.2221974	MCMBP	minichromosome maintenance complex bindir
15.764.613	-0.1733261		
15.762.491	-0.1786636	MRPS11	mitochondrial ribosomal protein S11
15.757.427	0.3935811	CDC42EP4	CDC42 effector protein 4
15.756.368	-0.1901777	GRM4	glutamate metabotropic receptor 4
15.755.699	-0.2183229	NPR1	natriuretic peptide receptor 1
15.753.319	-0.2480342	ZNF10	zinc finger protein 10
15.751.041	0.8202304	SSX2	SSX family member 2
15.747.681	-0.2824281	DZIP1	DAZ interacting zinc finger protein 1
15.741.198	0.6896691	HIST1H2AC	histone cluster 1, H2ac
15.734.551	-0.2033515	LOC44193	similar to signal-regulatory protein alpha
15.731.712	0.4660684	SEC24A	SEC24 homolog A, COPII coat complex compor
15.731.445	-0.3800195	CHI3L1	chitinase 3 like 1
15.718.137	0.297723	GNAS	GNAS complex locus
1.571.709	-0.2939526	R3HCC1	R3H domain and coiled-coil containing 1
15.715.681	0.3610049	RHOQ	ras homolog family member Q
15.706.704	-0.5889857	RARRES3	retinoic acid receptor responder 3
15.699.798	-0.3396293	TLR3	toll like receptor 3
15.695.287	-0.1177767	DEC1	deleted in esophageal cancer 1
15.684.328	0.3780487	NUP50	nucleoporin 50
15.682.881	-0.3628352	MZB1	marginal zone B and B1 cell specific protein
15.681.497	0.4282966	GNG4	G protein subunit gamma 4
15.676.999	0.2376743	TINF2	TERF1 interacting nuclear factor 2
15.676.572	-0.3369157	TMBIM6	transmembrane BAX inhibitor motif containing

15.675.544	0.195624		POLQ	DNA polymerase theta
15.672.561	0.5036214			
15.667.634	0.3993156		PCGF2	polycomb group ring finger 2
15.664.852	0.4395995		DLG1	discs large MAGUK scaffold protein 1
15.661.954	0.898564		STEAP1	six transmembrane epithelial antigen of the pro
15.659.185	0.351232		MED6	mediator complex subunit 6
15.654.636	-0.3521004		THEMIS2	thymocyte selection associated family member
15.651.929	-0.1775886		FAM20B	family with sequence similarity 20 member B
1.565.021	-0.1875821		EPOR	erythropoietin receptor
15.649.762	0.339189		RHOG	ras homolog family member G
15.648.408	0.3004169		RALGAPB	Ral GTPase activating protein non-catalytic beta
15.647.664	0.3203241		PRKCD	protein kinase C delta
15.641.126	-0.1635893		CSDC2	cold shock domain containing C2
15.639.728	-0.1781594		GFRA4	GDNF family receptor alpha 4
15.636.212	-0.2969667		TNFSF12-TI	TNFSF12-TNFSF13 readthrough///tumor necros
15.632.366	0.1586405		UBE2NL	ubiquitin conjugating enzyme E2 N like (gene/ps
1.561.692	0.9280111		SCD	stearoyl-CoA desaturase
15.612.204	0.369624		PGLS	6-phosphogluconolactonase
15.597.932	0.4623381		TMCC1	transmembrane and coiled-coil domain family
15.596.066	-0.1347855		ACVR1B	activin A receptor type 1B
15.587.196	-0.5853615		KAT2B	lysine acetyltransferase 2B
15.583.755	0.2729063		B3GAT3	beta-1,3-glucuronyltransferase 3
15.579.809	-0.2196743		ABCA1	ATP binding cassette subfamily A member 1
15.567.951	0.6861066			
15.555.532		12.193.573	REG3A	regenerating family member 3 alpha
15.552.544	-0.1870847		AMN	amnion associated transmembrane protein
15.539.855	-0.2456958		ST7L	suppression of tumorigenicity 7 like
15.527.954	-0.4310927		CFAP20	cilia and flagella associated protein 20
15.512.569	0.2036834		EIF5A2	eukaryotic translation initiation factor 5A2
15.501.526	0.5362411		ETV5	ETS variant 5
15.498.715	-0.1933689		KDM4B	lysine demethylase 4B
15.495.244	0.3106939		TTC28	tetratricopeptide repeat domain 28
15.492.949	0.4522294		FNTA	farnesyltransferase, CAAX box, alpha
15.488.743	-0.1902837		ADM2	adrenomedullin 2
15.483.605	0.339414		SLC9A3R1	SLC9A3 regulator 1
15.479.696	0.350898		MSL1	male specific lethal 1 homolog
15.474.788	-0.1437447		HPCAL4	hippocalcin like 4
1.547.082		-11.085.199	DIO1	deiodinase, iodothyronine type I
15.465.718	0.3138548		CCSER2	coiled-coil serine rich protein 2
15.464.189	-0.2661928			
1.546.316	-0.1914963		TSPO2	translocator protein 2
15.451.857	-0.6512745		GK	glycerol kinase
15.449.056	-0.1972073		RPL10	ribosomal protein L10
15.446.495	0.3701991		CACNA1D	calcium voltage-gated channel subunit alpha1

15.442.543	0.2524511	RNF31	ring finger protein 31
15.442.082	-0.4571102	HNMT	histamine N-methyltransferase
15.440.527	-0.3291625	TOLLIP	toll interacting protein
15.438.317	0.2359668	TMEM70	transmembrane protein 70
15.432.617	-0.1478507	CYP2A7P1	cytochrome P450 family 2 subfamily A member
15.428.619	0.3578625	HERC2	HECT and RLD domain containing E3 ubiquitin
15.426.119	-0.3399755	EGFL8	EGF like domain multiple 8
15.424.293	0.2326586	FLJ14082	hypothetical protein FLJ14082
15.413.752	0.1874191	CDK16	cyclin dependent kinase 16
15.409.143	0.2758614	SLC10A3	solute carrier family 10 member 3
15.408.713	-0.5339147	C21orf33	chromosome 21 open reading frame 33
1.540.729	0.2852728	PPP1R9A	protein phosphatase 1 regulatory subunit 9A
15.406.878	-0.1499973	THRA	thyroid hormone receptor, alpha
1.540.086	-0.1249588	ZNF835	zinc finger protein 835
15.395.829	-0.1680663	CHRNA4	cholinergic receptor nicotinic alpha 4 subunit
15.395.287	-0.2319829		
15.388.181	-0.413866	ASMTL	acetylserotonin O-methyltransferase-like
15.385.373	0.2818689	RPL12	ribosomal protein L12
15.380.808	-0.1308668	COL4A3	collagen type IV alpha 3 chain
15.375.877	0.4367578	PCM1	pericentriolar material 1
15.359.855	0.3394516	CHD8	chromodomain helicase DNA binding protein 8
15.352.616	-0.7047061	MLPH	melanophilin
15.348.883	-0.163104		
15.346.767	-0.2034192	GGA1	golgi associated, gamma adaptin ear containin
15.339.254	-0.5420251	LGSN	lengsin, lens protein with glutamine synthetase
15.336.056	-0.2830828	NFAT5	nuclear factor of activated T-cells 5
1.533.072	0.3550349	RECQL	RecQ like helicase
15.325.208	0.4052993	SMAD3	SMAD family member 3
15.313.845	-0.2703848	TFR2	transferrin receptor 2
15.303.435	0.2523538	PPIL2	peptidylprolyl isomerase like 2
15.288.148	-0.2576119	FOSL1	FOS like 1, AP-1 transcription factor subunit
15.280.575	-0.1653802	CC2D1A	coiled-coil and C2 domain containing 1A
15.279.964	-0.5253663		
15.273.145	0.4508402	OGT	O-linked N-acetylglucosamine (GlcNAc) transfe
15.267.286	0.2708405	OTUB1	OTU deubiquitinase, ubiquitin aldehyde binding
15.267.075	-0.1340528	RAB3D	RAB3D, member RAS oncogene family
15.254.462	-0.1712877	PKP1	plakophilin 1
15.253.439	0.2153254	TLE3	transducin like enhancer of split 3
15.248.028	0.4253645	NBN	nibrin
15.246.351	-0.3727638	PDE3B	phosphodiesterase 3B
15.245.873	-0.1739558	PATJ	PATJ, crumbs cell polarity complex component
15.244.379	-0.2115253	SP110	SP110 nuclear body protein
15.243.978	-0.3332995	MACF1	microtubule-actin crosslinking factor 1
15.231.855	-0.1924324	UBR4	ubiquitin protein ligase E3 component n-recogr

15.230.256	0.2939059	ATXN7L3B	ataxin 7 like 3B
15.229.575	0.3733466	TCTN3	tectonic family member 3
15.221.757	0.4789876	WAC	WW domain containing adaptor with coiled-coi
15.218.709	0.3866772	CDC5L	cell division cycle 5 like
15.209.793	-0.1606416	IPCEF1	interaction protein for cytohesin exchange facto
15.208.029	0.2831523	WWC1	WW and C2 domain containing 1
15.195.005	-0.5331429	NFIB	nuclear factor I B
15.177.612	-0.2779832	SOX5	SRY-box 5
15.176.079	0.2939316	UBIAD1	UbiA prenyltransferase domain containing 1
15.164.949	-0.1776779	KIR2DS3	killer cell immunoglobulin like receptor, two Ig d
1.514.761	-0.42924	MAN2B2	mannosidase alpha class 2B member 2
15.145.543	-0.2307715	SCNN1A	sodium channel epithelial 1 alpha subunit
15.142.823	-0.948459	SLC3A1	solute carrier family 3 member 1
15.142.578	0.3734829	AIMP1	aminoacyl tRNA synthetase complex interacting
15.141.614	-0.1654037	KIAA1660	KIAA1660 protein
15.133.312	-0.3579622	OCEL1	occludin/ELL domain containing 1
15.132.457	-0.1524394	LMTK2	lemur tyrosine kinase 2
15.125.741	-0.5734458	DNAJC22	DnaJ heat shock protein family (Hsp40) membe
1.512.479	-0.2476802	CALM1	calmodulin 1
15.123.687	0.3393252	BRD9	bromodomain containing 9
15.122.427	-0.1794717	PROP1	PROP paired-like homeobox 1
15.120.919	-0.1420819		
15.119.421	0.2685071	UBE2O	ubiquitin conjugating enzyme E2 O
15.119.117	-0.371411	CYLD	CYLD lysine 63 deubiquitinase
15.117.153	0.3318865	RASA4	RAS p21 protein activator 4
15.113.937	-0.1605302	HHLA2	HERV-H LTR-associating 2
15.108.646	-0.14462	LOC73000(	similar to Succinate dehydrogenase [ubiquinon
15.108.576	-0.3806872	TANK	TRAF family member associated NFKB activator
1.510.588	-0.1468099	ITGB6	integrin subunit beta 6
15.104.848	0.2839308	RNF20	ring finger protein 20
15.104.196	-0.1426286	POLDIP3	DNA polymerase delta interacting protein 3
15.103.773	-0.1384245	DYNC1LI2	dynein cytoplasmic 1 light intermediate chain 2
15.101.911	0.2772495	GSPT1	G1 to S phase transition 1
15.100.948	-0.4186379	GDF7	growth differentiation factor 7
15.094.261	-0.1409855	SOCS3	suppressor of cytokine signaling 3
15.082.301	-0.7563174	CCND1	cyclin D1
15.076.984	0.3325272	ARPC5L	actin related protein 2/3 complex subunit 5 like
15.076.294	-0.1445535	LOC72894(	hypothetical LOC728946///keratin associated p
15.072.981	-0.1989891		
15.066.321	0.3024156	MTMR3	myotubularin related protein 3
15.065.319	-0.1748223	DPF3	double PHD fingers 3
15.056.675	-0.3842102	PDE3B	phosphodiesterase 3B
15.052.606	0.6388428	C3orf14	chromosome 3 open reading frame 14
15.051.189	-0.1053733		

15.043.435	0.4774923		GALNT1	polypeptide N-acetylgalactosaminyltransferase
15.040.345	0.3852598		POLR2J	RNA polymerase II subunit J
15.038.091	-0.6862047		IGL	immunoglobulin lambda locus
15.035.342	-0.2200094		CALD1	caldesmon 1
15.031.724	-0.1749207		STMN3	stathmin 3
15.029.705		10.063.436	PDZK1IP1	PDZK1 interacting protein 1
15.026.821	-0.2277299		ACAP1	ArfGAP with coiled-coil, ankyrin repeat and PH d
15.025.653	-0.179199		DNAJC28	DnaJ heat shock protein family (Hsp40) membe
15.022.383	-0.1570786		ELAC1	elaC ribonuclease Z 1
15.014.567	-0.2381285		N4BP1	NEDD4 binding protein 1
15.012.054	-0.3662219			
15.009.192	-0.1525975		TULP2	tubby like protein 2
15.008.925	0.2464287		CENPB	centromere protein B
1.500.706	0.2634824		URB1	URB1 ribosome biogenesis 1 homolog (S. cerev
14.998.664	-0.1834566		LSAMP	limbic system-associated membrane protein
14.998.161	-0.1641083		RIMS1	regulating synaptic membrane exocytosis 1
14.995.882	-0.1352411		LDB3	LIM domain binding 3
14.994.871	0.1748282			
14.989.727	-0.3091859		CCDC92	coiled-coil domain containing 92
14.989.392	-0.1995778		WDTC1	WD and tetratricopeptide repeats 1
14.989.384	-0.3519256		HBG2///HB	hemoglobin subunit gamma 2///hemoglobin su
14.988.209	0.21752		CWF19L1	CWF19-like 1, cell cycle control (S. pombe)
14.986.161	0.3328511		BAX	BCL2 associated X, apoptosis regulator
14.985.203	-0.3897023		FAS	Fas cell surface death receptor
14.980.043	0.4852707		PLEKHA5	pleckstrin homology domain containing A5
14.977.676	0.2886977		BTF3	basic transcription factor 3
1.497.741	0.3221899		EIF3F	eukaryotic translation initiation factor 3 subunit
1.497.703	0.2850168		DIABLO	diablo IAP-binding mitochondrial protein
14.973.529	0.5134041		CARD10	caspase recruitment domain family member 10
1.497.172	-0.1774361			
14.971.052	-0.2150029		ARHGAP33	Rho GTPase activating protein 33
14.968.461	-0.1413992			
14.967.085	0.2831983		TUBGCP4	tubulin gamma complex associated protein 4
14.960.052	-0.3733932		CLK1	CDC like kinase 1
14.954.253	0.2771196		ZNF85	zinc finger protein 85
14.952.854	-0.2137827			
14.948.817	-0.2323928		CUBN	cubilin
14.948.263	0.8940911		NAV3	neuron navigator 3
14.941.471	0.381726		B4GALT4	beta-1,4-galactosyltransferase 4
14.931.825	-0.3001655		TSC22D3	TSC22 domain family member 3
14.931.514	-0.2500683		NTRK2	neurotrophic receptor tyrosine kinase 2
14.930.133	-0.6167693		ABCC2	ATP binding cassette subfamily C member 2
14.929.452	-0.7203711		HLA-C	major histocompatibility complex, class I, C
1.492.739	-0.2200812		LINC01140	long intergenic non-protein coding RNA 1140

14.925.555	-0.5709798	SYNE2	spectrin repeat containing nuclear envelope pro
14.914.977	0.4028554	ZFR	zinc finger RNA binding protein
14.912.856	0.6117334	CKB	creatine kinase B
14.911.942	-0.1665383	PTGDR	prostaglandin D2 receptor
14.907.645	0.1893175	RNF216	ring finger protein 216
14.900.108	-0.2182675	OR1D2	olfactory receptor family 1 subfamily D membe
14.898.752	0.2890571	SRRT	serrate, RNA effector molecule
14.889.583	0.2544951	PUM1	pumilio RNA binding family member 1
14.888.111	-0.3335324	CCDC90B	coiled-coil domain containing 90B
14.879.641	-0.3756733	KIF13B	kinesin family member 13B
14.879.279	0.2948161	VDAC3	voltage dependent anion channel 3
14.874.937	0.4240595	PTGFR	prostaglandin F receptor
14.873.753	0.7436031	COL2A1	collagen type II alpha 1 chain
14.864.497	0.2089127	B4GALT6	beta-1,4-galactosyltransferase 6
14.851.354	0.3250136	UBQLN2	ubiquilin 2
1.484.899	-0.1803975	INSR	insulin receptor
14.847.738	-0.1520778	PDE4B	phosphodiesterase 4B
1.484.676	0.3000231	PATZ1	POZ/BTB and AT hook containing zinc finger 1
14.844.287	0.2975645	NDUFA13	NADH:ubiquinone oxidoreductase subunit A13
14.842.485	0.2789776	NUDCD3	NudC domain containing 3
14.842.265	0.3226373	SNAPC5	small nuclear RNA activating complex polypept
14.837.754	-0.3140511	DYSF	dysferlin
14.834.001	-0.301259	UBQLN4	ubiquilin 4
14.825.811	0.3939363	SEC23IP	SEC23 interacting protein
14.824.795	-0.2137797	SEMA6A	semaphorin 6A
14.822.114	0.3885477	DYNC1I1	dynein cytoplasmic 1 intermediate chain 1
14.819.489	0.3520397	LSM12	LSM12 homolog
14.810.557	0.4316326	ZNF277	zinc finger protein 277
14.809.859	0.4181895	KPNB1	karyopherin subunit beta 1
14.802.515	-0.1689896	PRDM9	PR/SET domain 9
14.801.768	0.4184447	DDIT3	DNA damage inducible transcript 3
1.480.172	0.3221719	RANBP2	RAN binding protein 2
14.797.169	0.4004074	IDUA	iduronidase, alpha-L-
1.479.494	-0.1768113	IGHM///IGH	immunoglobulin heavy constant mu///immunog
14.789.095	-0.2902784	SIK3	SIK family kinase 3
14.780.525	-0.6317082	GLRX	glutaredoxin
14.777.774	0.2977672	RBM10	RNA binding motif protein 10
14.773.454	-0.1486386	RSPH6A	radial spoke head 6 homolog A
14.772.435	0.5266297	SSX3	SSX family member 3
14.762.898	-0.1551583	SLCO3A1	solute carrier organic anion transporter family r
14.762.671	-0.4501366	KDM4B	lysine demethylase 4B
14.753.959	-0.5638764	CHN2	chimerin 2
14.736.983	-0.3261922	VAMP2	vesicle associated membrane protein 2
1.473.672	0.2074415	ZNF576	zinc finger protein 576

14.736.292	0.4208543		KDELR1	KDEL endoplasmic reticulum protein retention
14.733.516	0.1505967		CYP2R1	cytochrome P450 family 2 subfamily R member
1.473.325	-0.1993836		TOP3A	topoisomerase (DNA) III alpha
14.729.402	-0.2000841		GYPB	glycophorin B (MNS blood group)
14.726.707	0.2087689		DVL3	dishevelled segment polarity protein 3
14.722.053	0.3602543		TFDP2	transcription factor Dp-2
14.719.314	-0.2341811		TNFSF12-TI	TNFSF12-TNFSF13 readthrough///tumor necros
1.471.313	-0.2900987		FBXW7	F-box and WD repeat domain containing 7
14.711.137	-0.1943671		SMPD1	sphingomyelin phosphodiesterase 1
14.707.216	0.3053908		HN1L	hematological and neurological expressed 1 lik
1.470.601	-0.4457683		TF	transferrin
14.700.173	-0.1207221		ADARB2	adenosine deaminase, RNA specific B2 (inactiv
14.699.894	0.2584032		BPTF	bromodomain PHD finger transcription factor
14.692.436	-0.6965254		ALDH3A2	aldehyde dehydrogenase 3 family member A2
14.691.167	-0.5118318		H6PD	hexose-6-phosphate dehydrogenase/glucose 1
14.685.098	-0.1456528		MS4A12	membrane spanning 4-domains A12
14.665.771	-0.5958426		GSN	gelsolin
14.648.654	-0.2742767		KIAA0754	KIAA0754
14.646.322	-0.1532521		LOC73032	similar to postmeiotic segregation increased 2-
14.638.422	0.1700527		ZNF45	zinc finger protein 45
14.637.358	0.439073		LGALS8	galectin 8
14.636.181	0.2035014		TULP3	tubby like protein 3
14.630.191	-0.8462311		F13B	coagulation factor XIII B chain
1.462.234	0.2209854		ELK4	ELK4, ETS transcription factor
14.612.449	0.7667163		HSPA1B	heat shock protein family A (Hsp70) member 1E
14.596.114	0.2799254		ZKSCAN3	zinc finger with KRAB and SCAN domains 3
14.575.688	0.3217493		NCBP1	nuclear cap binding protein subunit 1
14.574.147	-0.2034564		KIR2DL1	killer cell immunoglobulin like receptor, two Ig (
14.571.946		-13.089.023	SAA2///SAA	serum amyloid A2///serum amyloid A1
14.566.571	0.2456636		NUDT3	nudix hydrolase 3
14.556.031	0.2235643			
14.545.313	0.300613		TRA2A	transformer 2 alpha homolog
14.536.571	0.2524522		PPP4R3B	protein phosphatase 4 regulatory subunit 3B
14.527.673	-0.4044546		ALB	albumin
14.527.388	-0.2236063		SRCAP	Snf2-related CREBBP activator protein
1.452.605	-0.4299668		PCDHGA1/	protocadherin gamma subfamily A, 1///protoca
14.520.351	-0.1687758		KIR2DS5	killer cell immunoglobulin like receptor, two Ig (
14.520.317	0.2448748		RBM12B	RNA binding motif protein 12B
14.517.804	0.3528047		CIAPIN1	cytokine induced apoptosis inhibitor 1
14.517.071	0.4027211		SUN1	Sad1 and UNC84 domain containing 1
14.513.496	0.2704342		APTX	aprataxin
1.450.759	-0.3404513		PNPLA4	patatin like phospholipase domain containing 4
14.506.872	-0.2361209		SFTPB	surfactant protein B
14.490.626	0.2042405		PDS5A	PDS5 cohesin associated factor A

14.488.238	-0.3872881	ARHGEF6	Rac/Cdc42 guanine nucleotide exchange facto
14.486.223	0.4807512	THSD7A	thrombospondin type 1 domain containing 7A
14.480.906	0.2104492	RNFT2	ring finger protein, transmembrane 2
14.471.844	-0.1567081	KIR3DL1	killer cell immunoglobulin like receptor, three Ig
14.466.979	-0.4024678	FOLR2	folate receptor beta
14.455.889	0.1981232	SGSM3	small G protein signaling modulator 3
14.455.761	-0.414106	TF	transferrin
14.446.591	-0.2326948		
14.440.061	-0.1840132	PTPRN2	protein tyrosine phosphatase, receptor type N2
14.435.633	-0.1705107	L1CAM	L1 cell adhesion molecule
14.433.857	0.2703192	C7orf26	chromosome 7 open reading frame 26
14.429.991	0.2749694	CAMSAP2	calmodulin regulated spectrin associated prote
14.420.951	-0.2058018	LAT	linker for activation of T-cells
14.416.947	0.4385756	GCA	grancalcin
14.402.348	0.3858167	CREBZF	CREB/ATF bZIP transcription factor
14.396.344	-0.2042198	KRT83	keratin 83
14.390.794	-0.1888563	DNM1	dynamin 1
14.383.416	-0.245177	CACNB2	calcium voltage-gated channel auxiliary subuni
14.377.395	-0.2055973	CHRNA3	cholinergic receptor nicotinic alpha 3 subunit
14.369.953	-0.2162747	BRAP	BRCA1 associated protein
14.357.708	-0.219456	CIAO1	cytosolic iron-sulfur assembly component 1
14.353.887	-0.293966	PCF11	PCF11 cleavage and polyadenylation factor sub
14.350.963	-0.194358	GPSM3	G-protein signaling modulator 3
14.335.289	0.2458053		
14.334.567	-0.1580784	UMOD	uromodulin
14.332.149	0.3193395	LOC64412	hypothetical LOC644124///sirtuin 7
14.332.006	0.2086336	DNM3	dynamin 3
14.326.732	-0.2662004	PDLIM5	PDZ and LIM domain 5
14.325.857	-0.2869334	RUSC2	RUN and SH3 domain containing 2
14.322.875	-0.2714838	PPP4R3A	protein phosphatase 4 regulatory subunit 3A
14.317.785	-0.1641582	ADHFE1	alcohol dehydrogenase, iron containing 1
14.310.742	0.2596293	SFMBT1	Scm-like with four mbt domains 1
1.430.762	-0.1741354	B3GNT4	UDP-GlcNAc:betaGal beta-1,3-N-acetylglucosa
14.305.401	0.2652967	ZNF131	zinc finger protein 131
1.430.142	-0.4828005	BMS1P20	BMS1, ribosome biogenesis factor pseudogene
14.300.866	0.3269687	ADO	2-aminoethanethiol dioxygenase
14.299.334	-0.4704268	PLK2	polo like kinase 2
14.298.054	0.2314508	TMEM80	transmembrane protein 80
14.295.011	-0.1314256	IL1RN	interleukin 1 receptor antagonist
14.287.513	0.3027522	C4orf46///T	chromosome 4 open reading frame 46///translo
14.282.755	-0.207286	SYNPO2	synaptopodin 2
14.271.251	-0.6819439	PTPRC	protein tyrosine phosphatase, receptor type C
14.264.578	-0.5684384	DUSP10	dual specificity phosphatase 10
14.263.978	0.2660783	PSMD11	proteasome 26S subunit, non-ATPase 11

14.261.633	-0.1699456	ERVH-6	endogenous retrovirus group H member 6
14.260.763	-0.1688266	AMHR2	anti-Mullerian hormone receptor type 2
14.256.143	-0.3814618	UBE2D2	ubiquitin conjugating enzyme E2 D2
1.424.753	-0.4708821	C4orf19	chromosome 4 open reading frame 19
1.424.573	-0.1889254	SFTPA2///S	surfactant protein A2///surfactant protein A2B
14.241.978	0.1696525	STARD3	StAR related lipid transfer domain containing 3
14.238.887	0.378278	PCMT1	protein-L-isoaspartate (D-aspartate) O-methylt
14.231.994	0.5047867	FZD5	frizzled class receptor 5
14.227.682	-0.2295685	EPHB1	EPH receptor B1
14.222.849	0.3895202	CUX1	cut like homeobox 1
14.216.469	0.3068173		
14.213.864	-0.1797343	GP9	glycoprotein IX platelet
14.213.265	-0.141914	DEF6	DEF6, guanine nucleotide exchange factor
14.209.575	-0.3623616	PRF1	perforin 1
14.208.657	0.4129998	CUL4A	cullin 4A
14.207.012	-0.3427391	CALM1	calmodulin 1
14.199.721	-0.4240048	TRIM14	tripartite motif containing 14
14.199.601	-0.2063141	GNAO1	G protein subunit alpha o1
14.190.212	0.2248798	CREB1	cAMP responsive element binding protein 1
1.417.887	-0.2045232	STK38	serine/threonine kinase 38
14.171.437	-0.4227787	APOH	apolipoprotein H
14.168.465	0.4013798	YWHAB	tyrosine 3-monooxygenase/tryptophan 5-mono
14.167.054	-0.2133918	CD79B	CD79b molecule
14.166.447	-0.1461046	TLE4	transducin like enhancer of split 4
14.166.272	-0.1623766	CD180	CD180 molecule
14.160.269	0.3405827	CHCHD7	coiled-coil-helix-coiled-coil-helix domain conta
14.158.154	-0.1720654		
14.157.137	-0.5300715	ITFG1	integrin alpha FG-GAP repeat containing 1
14.156.697	-0.1308205	SULT1A4	sulfotransferase family 1A member 4
14.154.557	0.4717698	MVP	major vault protein
14.153.602	-0.5351985	IL13RA1	interleukin 13 receptor subunit alpha 1
14.151.022	0.4184005	LAMTOR3	late endosomal/lysosomal adaptor, MAPK and I
14.138.147	-0.1501471	SIGLEC6	sialic acid binding Ig like lectin 6
14.126.556	0.2811071	PLXNC1	plexin C1
14.124.911	0.3579302	KARS	lysyl-tRNA synthetase
14.123.251	0.270267	RTF1	RTF1 homolog, Paf1/RNA polymerase II comple
14.119.407	0.3437021	SKAP2	src kinase associated phosphoprotein 2
14.113.488	0.7698815	TNFRSF21	TNF receptor superfamily member 21
14.112.428	-0.2026919	LAG3	lymphocyte activating 3
14.108.636	-0.5822501	GATA6	GATA binding protein 6
14.107.572	-0.3031479		
14.107.223	-0.2262771	UBE3A	ubiquitin protein ligase E3A
14.079.132	-0.3019406	RRN3	RRN3 homolog, RNA polymerase I transcription
14.074.869	0.3080717	PFKFB2	6-phosphofructo-2-kinase/fructose-2,6-biphos

14.073.228	-0.1769349	RIF1	replication timing regulatory factor 1
14.070.644	-0.4823862		
14.054.297	0.4741495	LIPA	lipase A, lysosomal acid type
14.047.642	-0.131008	ADAM20	ADAM metallopeptidase domain 20
14.045.529	0.3493047	EIF3CL///EI	eukaryotic translation initiation factor 3 subunit
14.044.694	0.2959006	EIF4E2	eukaryotic translation initiation factor 4E family
14.033.098	0.3265485	YIPF6	Yip1 domain family member 6
14.032.333	-0.169103	PSG3	pregnancy specific beta-1-glycoprotein 3
14.030.478	0.4189786	RNPEP	arginyl aminopeptidase
14.022.862	-0.2356682		
14.011.245	-0.196638		
13.989.614	-0.1532592	SMR3A	submaxillary gland androgen regulated protein
13.987.862	-0.6674715	GGT1	gamma-glutamyltransferase 1
13.985.066	0.2783914	CTCF	CCCTC-binding factor
13.977.048	0.2283981	ZNF34	zinc finger protein 34
13.972.348	-0.1639462	KCNH4	potassium voltage-gated channel subfamily H r
13.972.044	-0.1883772	CD6	CD6 molecule
1.396.908	0.3891075	HSPA9	heat shock protein family A (Hsp70) member 9
13.964.134	-0.3404323	TMEM254	transmembrane protein 254
13.964.126	0.3270835	PANK2	pantothenate kinase 2
13.963.047	0.2778105	RPS26P25/	ribosomal protein S26 pseudogene 25///ribosor
13.962.758	-0.1338268	ABCG4	ATP binding cassette subfamily G member 4
13.955.086	0.4108535	PELO	pelota homolog (Drosophila)
13.948.986	-0.1571989	SPDEF	SAM pointed domain containing ETS transcripti
13.945.916	-0.1617561	ZNF443	zinc finger protein 443
13.945.217	-0.1774728	IKZF1	IKAROS family zinc finger 1
13.935.094	-0.1761254	KLK13	kallikrein related peptidase 13
1.392.064	-0.1640814	ITGA8	integrin subunit alpha 8
1.391.279	-0.2653119	RHOH	ras homolog family member H
13.909.577	-0.1721476	ABCB6	ATP binding cassette subfamily B member 6 (La
1.389.885	-0.1748076	IL1RN	interleukin 1 receptor antagonist
13.891.917	-0.6867016		
13.890.972	-0.1254548	RUNX1	runt related transcription factor 1
13.890.769	0.2684692	TEX261	testis expressed 261
13.880.995	0.3057426	GGA2	golgi associated, gamma adaptin ear containing
13.878.095	-0.2483306	SEPT4	septin 4
13.877.108	-0.1700393	ADD2	adducin 2
13.876.183	-0.1455763	CNTN2	contactin 2
13.862.183	-0.7913535		
13.861.067	0.7640492	SSX4B///SS	SSX family member 4B///SSX family member 4
13.855.291	0.1793642	HBZ	hemoglobin subunit zeta
13.853.558	0.249812	EIF2AK1	eukaryotic translation initiation factor 2 alpha k
13.852.628	0.2029157	SLC7A6	solute carrier family 7 member 6
13.851.582	0.3364691	PPP2R5C	protein phosphatase 2 regulatory subunit B'gan

13.850.888	0.2571475	TBC1D13	TBC1 domain family member 13
13.845.224	0.6807656	SLC26A3	solute carrier family 26 member 3
13.842.659	0.4755116	SOAT2	sterol O-acyltransferase 2
13.837.426	-0.2938255	ABCA2	ATP binding cassette subfamily A member 2
13.832.752	-0.1155919	MYH8	myosin heavy chain 8
13.829.895	0.2017033	EXOC5	exocyst complex component 5
13.826.429	-0.323298		
1.382.581	0.2851583	PDHB	pyruvate dehydrogenase (lipoamide) beta
1.382.236	0.3916877	TJP1	tight junction protein 1
13.817.332	-0.2984285	DCTD	dCMP deaminase
13.813.553	-0.1509595	CXCR5	C-X-C motif chemokine receptor 5
13.799.236	0.6190233	SNAP25	synaptosome associated protein 25
13.799.141	-0.1981746	TRIM25	tripartite motif containing 25
13.794.623	-0.2833274	AHNAK	AHNAK nucleoprotein
13.793.739	0.1535904	ZNF668	zinc finger protein 668
13.791.761	0.2410794	SENP6	SUMO1/sentrin specific peptidase 6
13.788.718	-0.336006	PDE4C	phosphodiesterase 4C
13.779.531	-0.3674343	KLC1	kinesin light chain 1
13.777.436	0.2653152	ABCG1	ATP binding cassette subfamily G member 1
13.775.785	-0.320136	PTPRCAP	protein tyrosine phosphatase, receptor type C a
1.377.395	0.3451083	ZNF14	zinc finger protein 14
13.771.471	-0.2680569	KLF12	Kruppel like factor 12
1.375.746	0.6822877	ACSM1	acyl-CoA synthetase medium-chain family mer
13.756.246	-0.7497719	PSAT1	phosphoserine aminotransferase 1
13.754.953	-0.2107701	SGCA	sarcoglycan alpha
13.749.831	-0.4334951	FRA10AC1/	fragile site, folic acid type, rare, fra(10)(q23.3) c
13.749.199	0.1786696	LIG3	DNA ligase 3
13.745.206	-0.8064146	IGLV2-14	immunoglobulin lambda variable 2-14
13.740.376	0.7115137	CPE	carboxypeptidase E
13.733.613	-0.3258577	RBM6	RNA binding motif protein 6
137.289	-0.5679948	BHLHE40	basic helix-loop-helix family member e40
13.726.306	-0.1490646	PNOC	prepronociceptin
13.726.177	0.3709719	SMPX	small muscle protein, X-linked
13.707.659	0.6284215	HSPA8	heat shock protein family A (Hsp70) member 8
13.705.822	0.4261155	NDUFA4L2	NDUFA4, mitochondrial complex associated lik
13.703.498	0.2904033	GRK3	G protein-coupled receptor kinase 3
13.697.916	0.3564697	MFSD1	major facilitator superfamily domain containing
13.697.097	-0.1898701	MBNL2	muscleblind like splicing regulator 2
13.692.443	0.224292	CAMSAP1	calmodulin regulated spectrin associated prote
13.691.006	0.2335695	ATP6V0A2	ATPase H+ transporting V0 subunit a2
13.687.291	-0.1605138	LRRK1	leucine rich repeat kinase 1
13.683.051	-0.254377		
13.678.707	0.1735877	MFSD6	major facilitator superfamily domain containing
13.675.866	-0.2088876	DLC1	DLC1 Rho GTPase activating protein

13.674.812	0.4312957	CBX6	chromobox 6
1.366.984	-0.1244529	KCNJ14	potassium voltage-gated channel subfamily J m
13.658.342	-0.4952076	GPER1	G protein-coupled estrogen receptor 1
13.654.219	-0.1652872	TRPC7	transient receptor potential cation channel sub
13.653.459	-0.207043	MYBPC3	myosin binding protein C, cardiac
13.649.744	0.2274603	E2F1	E2F transcription factor 1
13.648.361	0.2640684	ZNF202	zinc finger protein 202
13.646.136	-0.1752913	GRIA3	glutamate ionotropic receptor AMPA type subu
13.641.559	0.1569107	ZNF571	zinc finger protein 571
13.640.404	0.2529884	EMC9	ER membrane protein complex subunit 9
13.628.559	0.4421526	TP53	tumor protein p53
13.626.673	0.3414753	SAPCD1///I	suppressor APC domain containing 1///mutS ho
136.238	-0.2812301	GCNT2	glucosaminyl (N-acetyl) transferase 2, I-branch
13.616.633	-0.1398442	ITGA2B	integrin subunit alpha 2b
13.612.758	-0.1900699	PLAC4	placenta specific 4
13.612.396	-0.356768	RRN3P1///I	RRN3 homolog, RNA polymerase I transcription
13.601.429	-0.1975032	SLIT3	slit guidance ligand 3
13.593.353	-0.2465285	PBX1	PBX homeobox 1
13.584.978	-0.3560352	VPS37B	VPS37B, ESCRT-I subunit
13.581.791	-0.1336654	ANP32C	acidic nuclear phosphoprotein 32 family memb
13.577.828	-0.2168268	LOC64797	similar to Chymotrypsinogen B precursor///chy
13.551.672	-0.1883278	DIO2	deiodinase, iodothyronine, type II
13.541.926	-0.174511	SMAD6	SMAD family member 6
13.539.164	0.2647274	PIAS1	protein inhibitor of activated STAT 1
13.537.128	0.2546452	PTDSS2	phosphatidylserine synthase 2
13.534.537	-0.1613316		
13.534.335	-0.1968983		
13.521.805	-0.1470482	FGFBP1	fibroblast growth factor binding protein 1
13.519.018	0.3526779	FBXO9	F-box protein 9
1.351.696	0.4845531	SGMS1	sphingomyelin synthase 1
13.512.345	-0.2324724		
13.512.262	0.3316962	C6orf62	chromosome 6 open reading frame 62
13.498.509	-0.1609125	ELOVL5	ELOVL fatty acid elongase 5
13.496.196	-0.1476721	PDE6A	phosphodiesterase 6A
1.348.713	-0.7084843	KNG1	kininogen 1
13.481.561	0.2661768	GIGYF2	GRB10 interacting GYF protein 2
13.478.996	-0.1620395	PTPRB	protein tyrosine phosphatase, receptor type B
1.347.724	0.307524	COLGALT1	collagen beta(1-O)galactosyltransferase 1
13.476.074	-0.2194008	CDX1	caudal type homeobox 1
13.472.416	0.3446056	LOC72790	hypothetical LOC727901///von Willebrand fact
13.469.101	-0.3654184	HES1	hes family bHLH transcription factor 1
13.448.975	-0.2636559	DMTN	dematin actin binding protein
13.442.218	-0.2024887	NCR2	natural cytotoxicity triggering receptor 2
13.440.766	0.2860716	TIMM10	translocase of inner mitochondrial membrane 1

13.434.725	-0.1679293	RIC3	RIC3 acetylcholine receptor chaperone
13.432.255	0.3022754	MAPKAPK3	mitogen-activated protein kinase-activated pro
13.426.692	-0.1546696	PAX3	paired box 3
13.426.166	-0.4145783	SERINC1	serine incorporator 1
13.423.154	-0.3021593	PRDX6	peroxiredoxin 6
13.415.483	-0.2327067	PF4	platelet factor 4
13.400.697	-0.1416392	SH3TC1	SH3 domain and tetratricopeptide repeats 1
13.400.327	0.3417012	SEC24C	SEC24 homolog C, COPII coat complex compor
13.399.965	-0.158013	MEFV	Mediterranean fever
13.392.034	-0.1436063	NCR3	natural cytotoxicity triggering receptor 3
13.389.299	-0.1719768	SPG20	spastic paraplegia 20 (Troyer syndrome)
13.386.561	0.1884637	TMEM104	transmembrane protein 104
13.380.138	0.477564	SDHC	succinate dehydrogenase complex subunit C
13.373.366	0.1767606	POP1	POP1 homolog, ribonuclease P/MRP subunit
13.370.087	-0.2656167	ADCY10	adenylate cyclase 10, soluble
13.369.463	-0.2490653	SNW1	SNW domain containing 1
1.336.563	-0.2030617	CACNA1A	calcium voltage-gated channel subunit alpha1 /
13.357.664	-0.2244616	IGHD	immunoglobulin heavy constant delta
13.356.592	-0.133306	FLJ11827	hypothetical protein FLJ11827
13.353.615	-0.2108033	ATF7IP	activating transcription factor 7 interacting prot
13.352.944	-0.3341396	CACNB4	calcium voltage-gated channel auxiliary subuni
1.335.147	-0.3863219	POLR1B	RNA polymerase I subunit B
13.348.769	-0.1889386	TRIM3	tripartite motif containing 3
13.339.894	-0.1837587	PDZD7	PDZ domain containing 7
13.330.652	0.2686887	MED8	mediator complex subunit 8
13.330.262	-0.1589533	UCP3	uncoupling protein 3
13.325.958	0.7227541	ZNF83	zinc finger protein 83
13.323.993	-0.134998	ATF7IP	activating transcription factor 7 interacting prot
13.323.706	0.2546977	TRIO	trio Rho guanine nucleotide exchange factor
13.311.627	0.6383422	CD47	CD47 molecule
13.303.378	-0.1864469	KRT8P7///K	keratin 8 pseudogene 7///keratin 8 pseudogene
13.302.738	-0.243237	NFATC1	nuclear factor of activated T-cells 1
13.294.446	-0.2517998	KAT8	lysine acetyltransferase 8
13.293.767	0.2884483	DLGAP4	DLG associated protein 4
1.328.876	-0.1752288	PRO0478	PRO0478 protein
1.328.516	-0.1625534		
132.743	0.3046706	FRYL	FRY like transcription coactivator
13.273.723	0.2850582	GOLGA4	golgin A4
13.272.285	-0.4214395	PEX11A	peroxisomal biogenesis factor 11 alpha
13.272.282	-0.3219457	TNFRSF14	TNF receptor superfamily member 14
13.260.915	0.2679413	SFI1	SFI1 centrin binding protein
13.257.498	-0.3595732	ITSN1	intersectin 1
132.572	-0.4509863	NFAT5	nuclear factor of activated T-cells 5
13.257.159	-0.201549	TLR4	toll like receptor 4

13.252.907	0.1849927	MRPS12	mitochondrial ribosomal protein S12
13.250.304	-0.1660144		
13.233.382	0.297927	PSME4	proteasome activator subunit 4
13.231.437	-0.3783785	TSR1	TSR1, ribosome maturation factor
13.229.316	-0.2073733	EDN3	endothelin 3
1.321.367	0.1898443		
13.210.639	-0.4334558	AIF1	allograft inflammatory factor 1
13.208.485	0.7242825	GLUL	glutamate-ammonia ligase
13.189.926	0.3485297	MDH1	malate dehydrogenase 1
13.165.262	-0.2180166	SLC8B1	solute carrier family 8 member B1
13.160.438	0.3119406	ZNF195	zinc finger protein 195
13.159.034	-0.3868099	PTP4A1	protein tyrosine phosphatase type IVA, membe
13.155.644	0.2504604	MAPT	microtubule associated protein tau
13.155.248	0.3149149	PIN4	peptidylprolyl cis/trans isomerase, NIMA-intera
1.315.294	0.313783	ARL1	ADP ribosylation factor like GTPase 1
13.146.864	-0.1624526	RAPGEF1	Rap guanine nucleotide exchange factor 1
131.463	-0.1482498	FBN2	fibrillin 2
13.139.306	-0.6961318	ZDHHC11	zinc finger DHHC-type containing 11
13.138.191	0.2333531	RPS3A	ribosomal protein S3A
13.135.566	-0.1377466	NECTIN1	nectin cell adhesion molecule 1
13.134.887	0.4142737	BET1	Bet1 golgi vesicular membrane trafficking prote
13.133.085	0.3842166	TAF1D	TATA-box binding protein associated factor, RN
1.311.412	-0.2630058		
13.111.937	0.4579084	MXRA7	matrix remodeling associated 7
13.109.863	0.3007736	TPD52	tumor protein D52
13.102.628	0.3303382	ERP44	endoplasmic reticulum protein 44
13.099.773	-0.3604502	MUC3B	mucin 3B, cell surface associated
130.952	0.2156856	HABP4	hyaluronan binding protein 4
1.308.465	0.3223321	CDK17	cyclin dependent kinase 17
13.081.484	0.2032897	L3MBTL1	l(3)mbt-like 1 (Drosophila)
13.079.189	0.2462446	MAPT	microtubule associated protein tau
13.067.308	0.2684253	CUL9	cullin 9
13.066.222	-0.1655052	ALPPL2	alkaline phosphatase, placental like 2
13.065.067	-0.3503463	SFPQ	splicing factor proline and glutamine rich
13.060.741	-0.1590127		
13.056.489	-0.4954451	GBE1	glucan (1,4-alpha-), branching enzyme 1
13.055.121	-0.3430438	GPRASP1	G protein-coupled receptor associated sorting
13.049.864	-0.1793687	IAPP	islet amyloid polypeptide
13.033.695	0.3187258	HINT1	histidine triad nucleotide binding protein 1
13.031.192	-0.2884127	ROBO2	roundabout guidance receptor 2
13.030.601	-0.1898672	AKAP11	A-kinase anchoring protein 11
13.028.722	-0.1734894	PRSS22	protease, serine 22
13.026.232	-0.7514458	SLC6A1	solute carrier family 6 member 1
13.025.628	0.4685058	FMR1	fragile X mental retardation 1

13.025.472	-0.4930965	DCTD	dCMP deaminase
1.301.395	0.3593667	HIST1H2BF	histone cluster 1, H2bf
13.011.829	0.2923231	BCLAF1	BCL2 associated transcription factor 1
13.009.421	-0.1625103	KCNH6	potassium voltage-gated channel subfamily H r
13.008.814	-0.5876373	GGT2///GG	gamma-glutamyltransferase 2///gamma-glutan
13.004.833	-0.3380523	COBLL1	cordon-bleu WH2 repeat protein like 1
13.002.808	-0.1734105	RAB14	RAB14, member RAS oncogene family
12.998.817	-0.2039404	NUDT7	nudix hydrolase 7
12.979.433	0.3248888	HSBP1	heat shock factor binding protein 1
12.979.022	-0.1680462	HN1L	hematological and neurological expressed 1 lik
12.975.269	-0.1731608	UBASH3A//	ubiquitin associated and SH3 domain containir
12.973.459	0.3046741	HIGD2A	HIG1 hypoxia inducible domain family member
12.972.385	-0.1609768	NPAS1	neuronal PAS domain protein 1
12.967.054	0.2107811		
12.963.848	-0.2864281	REL	REL proto-oncogene, NF-kB subunit
12.961.338	-0.4652607	SCNN1A	sodium channel epithelial 1 alpha subunit
12.959.061	-0.1620889	CALML3	calmodulin like 3
12.957.844	-0.2003123	BAIAP3	BAI1 associated protein 3
12.950.235	0.2938285	CSTF3	cleavage stimulation factor subunit 3
129.484	0.3510361	ZCCHC11	zinc finger CCHC-type containing 11
12.947.466	-0.6346014	TMEM30B	transmembrane protein 30B
12.945.463	0.5268815	RAP1GAP	RAP1 GTPase activating protein
12.941.702	-0.1660734	DRD2	dopamine receptor D2
12.937.096	-0.1519298	OMD	osteomodulin
12.932.857	-0.1205772	PADI4	peptidyl arginine deiminase 4
12.932.727	0.2198534	MTERF1	mitochondrial transcription termination factor :
12.930.951	0.5702036	DHCR7	7-dehydrocholesterol reductase
1.292.846	-0.2055839	SMAD9	SMAD family member 9
12.916.945	-0.1705411	RERE	arginine-glutamic acid dipeptide repeats
12.911.738	-0.1359486	GPR31	G protein-coupled receptor 31
12.908.197	0.2838623	MAGT1	magnesium transporter 1
12.902.079	-0.1882008	AIM1L	absent in melanoma 1-like
12.899.359	0.2924507	SETD2	SET domain containing 2
12.897.015	0.3038442	RPL37A	ribosomal protein L37a
12.895.988	0.2086209	REEP4	receptor accessory protein 4
12.893.759	0.1908842	POMZP3	POM121 and ZP3 fusion
12.891.493	0.3265677	IMP4	IMP4 homolog, U3 small nucleolar ribonucleop
12.887.744	-0.4096225	CCNI	cyclin I
12.876.669	-0.2177746	FLJ14327	hypothetical protein FLJ14327
12.868.317	0.464792	ATP2B1	ATPase plasma membrane Ca2+ transporting 1
12.851.319	-0.1598582		
12.846.275	-0.1871843	APBB2	amyloid beta precursor protein binding family B
12.841.817	0.2867763	RSRC2	arginine and serine rich coiled-coil 2
12.839.997	0.3058184	CIAO1	cytosolic iron-sulfur assembly component 1

12.837.834	-0.4606248		RAB20	RAB20, member RAS oncogene family
12.837.538		10.075.353		
12.830.857	0.2885614		RPL12	ribosomal protein L12
12.826.122	0.2250055		UBE2J1	ubiquitin conjugating enzyme E2 J1
12.818.354	0.2351113		FAM168A	family with sequence similarity 168 member A
12.813.656	-0.4904758		AK2	adenylate kinase 2
12.810.539	-0.2514224		DENND1C	DENN domain containing 1C
12.805.684	-0.271647			
12.802.143	0.2636003		PPP6C	protein phosphatase 6 catalytic subunit
12.800.966	-0.1613349		GCNT2	glucosaminyl (N-acetyl) transferase 2, I-branch
1.279.447	-0.2070982		USP2	ubiquitin specific peptidase 2
12.793.457	0.3967202		RARA	retinoic acid receptor alpha
12.792.121	-0.1821252		PHKG1	phosphorylase kinase catalytic subunit gamma
12.782.744	-0.3647101		PDE8A	phosphodiesterase 8A
12.781.989	0.1572664		APBA3	amyloid beta precursor protein binding family A
12.779.204	0.3404957		RNF38	ring finger protein 38
12.772.122	0.352827		PRPF6	pre-mRNA processing factor 6
1.276.753	-0.1439414		OR10H3	olfactory receptor family 10 subfamily H memb
12.759.693	-0.1449672		LOC44079	proline dehydrogenase 1 pseudogene
12.757.921	-0.245727		PAOX	polyamine oxidase (exo-N4-amino)
12.749.649	-0.6450189		LDLR	low density lipoprotein receptor
12.749.568	-0.4888497		KLF10	Kruppel like factor 10
12.747.686	-0.4565868		SHMT2	serine hydroxymethyltransferase 2
12.744.752	-0.1440862		ESR1	estrogen receptor 1
1.272.962	-0.216597		GRM8	glutamate metabotropic receptor 8
12.729.415	0.3023652		CCNJ	cyclin J
12.729.114	-0.2946445		BACH2	BTB domain and CNC homolog 2
12.727.279	0.245333		CPSF4	cleavage and polyadenylation specific factor 4
12.727.007	-0.1290083		ALPP	alkaline phosphatase, placental
12.723.908	0.2963632		USE1	unconventional SNARE in the ER 1
12.717.244	0.2994326		DDX18///W	DEAD-box helicase 18///Wnt family member 8E
12.712.844	-0.166718		MAP1A	microtubule associated protein 1A
1.271.187	-0.3399638		FLJ12151	hypothetical protein FLJ12151
12.707.475	-0.1668256		PI4K2A	phosphatidylinositol 4-kinase type 2 alpha
12.705.568	0.6048385		TPM2	tropomyosin 2 (beta)
1.270.327	-0.1709586			
12.702.885	-0.1934191		NCKIPSD	NCK interacting protein with SH3 domain
12.696.881	0.3649372		ZNF302	zinc finger protein 302
12.693.648	0.2771328		AUP1	ancient ubiquitous protein 1
12.686.242	0.3135864		RNF114	ring finger protein 114
12.684.247	-0.1541066		ARHGEF10	Rho guanine nucleotide exchange factor 10
126.814	0.2196158		METTL2A///	methyltransferase like 2A///methyltransferase
12.679.881	-0.2132543		TMEM92-AS	TMEM92 antisense RNA 1
12.676.446	0.5702982		GABBR2	gamma-aminobutyric acid type B receptor subu

12.665.598	-0.1347584		
12.665.146	0.3325077	YARS	tyrosyl-tRNA synthetase
12.663.081	-0.2025833	SLC16A5	solute carrier family 16 member 5
12.661.121	-0.1730208	HOXB5	homeobox B5
12.656.477	-0.1312247	PSMD5	proteasome 26S subunit, non-ATPase 5
12.655.844	-0.3527229	TMEM259	transmembrane protein 259
12.651.818	0.4542555	EIF4E	eukaryotic translation initiation factor 4E
12.651.166	-0.1729424	IBSP	integrin binding sialoprotein
12.647.047	-0.3045589	IGHV4-31//	immunoglobulin heavy variable 4-31///immuno
1.264.365	-0.3678609		
12.641.737	-0.1727213	EML2	echinoderm microtubule associated protein lik
12.640.855	-0.7345435	IFIT1	interferon induced protein with tetratricopeptid
12.640.568	-0.152892	FSTL4	follistatin like 4
12.639.719	0.2729753	CD320	CD320 molecule
12.625.223	-0.1985242	IGHM///IGH	immunoglobulin heavy constant mu///immunog
12.624.916	-0.1916375	SLC11A1	solute carrier family 11 member 1
12.618.395	0.3518109	NCOA3	nuclear receptor coactivator 3
12.604.043	0.3692427	DNAJC10	DnaJ heat shock protein family (Hsp40) membe
12.603.624	0.5741423	ATRN	attractin
12.600.835	0.2870816	NCKIPSD	NCK interacting protein with SH3 domain
1.259.918	-0.6558405	NQO2	NAD(P)H quinone dehydrogenase 2
12.597.631	0.3900636	TLR5	toll like receptor 5
12.594.513	-0.1280568	TCL6	T-cell leukemia/lymphoma 6 (non-protein codir
12.594.315	0.2816679	TRAPPC12	trafficking protein particle complex 12
12.588.542	-0.3484529	DSG1	desmoglein 1
12.586.117	-0.1524755	OSBPL7	oxysterol binding protein like 7
12.579.863	-0.427319	GIMAP5	GTPase, IMAP family member 5
12.578.216	-0.1228059	MYL1	myosin light chain 1
12.574.605	0.6307829	CTSC	cathepsin C
12.574.205	-0.2587846		
12.571.753	0.1847882	PPP3R1	protein phosphatase 3 regulatory subunit B, alp
12.570.265	0.1920076	GALNT10	polypeptide N-acetylgalactosaminyltransferase
12.568.723	-0.1617352	ZNF518A	zinc finger protein 518A
12.536.504	-0.1597629	MYO3A	myosin IIIA
12.536.397	-0.3799307	VKORC1	vitamin K epoxide reductase complex subunit 1
12.535.594	0.1711101	FDPSP5	farnesyl diphosphate synthase pseudogene 5
12.530.901	0.2993597	TRIOBP	TRIO and F-actin binding protein
12.530.316	-0.4073287		
12.528.055	-0.447486	DAAM1	dishevelled associated activator of morphogen
12.526.613	-0.3653144	PGRMC2	progesterone receptor membrane component 2
12.524.491	-0.4403005	SEMA5A	semaphorin 5A
12.519.065	-0.3346085	SRRM1	serine and arginine repetitive matrix 1
1.251.842	0.3073924	TICAM1	toll like receptor adaptor molecule 1
1.251.332	-0.2376948		

12.511.797	0.4401881	BBX	BBX, HMG-box containing
12.504.726	0.257757	C19orf24	chromosome 19 open reading frame 24
12.504.146	-0.3794135	PAPD4	poly(A) RNA polymerase D4, non-canonical
12.492.511	-0.3597	DNAJB14	DnaJ heat shock protein family (Hsp40) membe
12.492.299	-0.3873423	FURIN	furin, paired basic amino acid cleaving enzyme
12.490.788	-0.6117293	MCL1	BCL2 family apoptosis regulator
12.488.725	-0.2951553	WDR45	WD repeat domain 45
12.488.378	-0.2525255	ERICH1	glutamate rich 1
12.487.097	0.4203553	RANBP9	RAN binding protein 9
12.486.784	0.2967695	TTC9	tetratricopeptide repeat domain 9
12.484.959	-0.1686679	WNT16	Wnt family member 16
12.484.943	-0.2619902	TMEM63A	transmembrane protein 63A
12.482.836	-0.606096	NAT8B///NA	N-acetyltransferase 8B (putative, gene/pseudo
12.482.673	-0.1446937	KRT32	keratin 32
12.481.727	-0.2575069	MPG	N-methylpurine DNA glycosylase
1.247.775	-0.2589931	GNAI2	G protein subunit alpha i2
12.472.387	0.3882412	SDHAF3	succinate dehydrogenase complex assembly fa
12.472.189	-0.1684946	IL1RL1	interleukin 1 receptor like 1
12.468.506	-0.1411789	SLC30A6	solute carrier family 30 member 6
12.463.563	0.3021429	PCNX4	pecanex homolog 4 (Drosophila)
12.456.963	0.4055554	ZNF814	zinc finger protein 814
12.452.953	-0.2822066	PPARA	peroxisome proliferator activated receptor alph
1.244.662	0.3300097	HIST1H2AE	histone cluster 1, H2ae
12.432.243	-0.1925919	IZUMO4	IZUMO family member 4
12.428.548	0.283921	SCYL3	SCY1 like pseudokinase 3
12.418.187	-0.3785537	NKG7	natural killer cell granule protein 7
12.417.612	-0.1811871	LUZP1	leucine zipper protein 1
12.417.395	0.1344793	SLC35F5	solute carrier family 35 member F5
12.413.854	0.4385292	CTNND2	catenin delta 2
12.411.445	-0.2471336	PNPLA2	patatin like phospholipase domain containing 2
12.409.753	-0.2139016	ADAMTS2	ADAM metallopeptidase with thrombospondin t
1.240.955	0.7317579	HMGA2	high mobility group AT-hook 2
12.409.465	0.2846596	MAP3K7	mitogen-activated protein kinase kinase kinase
12.408.702	0.1940578	ATP11A	ATPase phospholipid transporting 11A
12.399.426	0.2187428	GUF1	GUF1 homolog, GTPase
12.396.741	0.2584043	FAM168A	family with sequence similarity 168 member A
12.396.188	-0.2978222	PITPNA	phosphatidylinositol transfer protein alpha
12.390.636	-0.2366275	ARSF	arylsulfatase F
12.389.734	-0.8991102	GSTM1	glutathione S-transferase mu 1
12.388.542	-0.2162924	BTG2	BTG anti-proliferation factor 2
12.385.937	-0.1567182	AKAP3	A-kinase anchoring protein 3
12.383.574	-0.3032787	ZSWIM8	zinc finger SWIM-type containing 8
12.378.665	-0.3566969	ZNHIT1	zinc finger HIT-type containing 1
12.378.618	-0.167164	CD209	CD209 molecule

12.368.383	0.3068527	PIGB	phosphatidylinositol glycan anchor biosynthesi
12.366.276	-0.1679479	MTRF1L	mitochondrial translational release factor 1 like
1.236.598	-0.4418867	CTPS1	CTP synthase 1
12.363.653	-0.3307787	IL13RA1	interleukin 13 receptor subunit alpha 1
12.361.816	-0.3148307	CCR1	C-C motif chemokine receptor 1
12.361.129	-0.1866278	GPR15	G protein-coupled receptor 15
12.349.627	-0.3352864	TLE2	transducin like enhancer of split 2
12.348.466	0.5629287	SLC30A10	solute carrier family 30 member 10
12.347.991	-0.1375513	HOXC13	homeobox C13
12.347.958	0.3066932	HSPA4	heat shock protein family A (Hsp70) member 4
12.341.414	-0.4247357	ZFAND5	zinc finger AN1-type containing 5
1.234.083	0.5531674	TPM4	tropomyosin 4
12.337.745	-0.1446612	PSG9	pregnancy specific beta-1-glycoprotein 9
12.322.324	0.2462431	OLFML2B	olfactomedin like 2B
12.321.851	-0.1605216		
12.300.623	0.4079088	MPPE1	metallophosphoesterase 1
12.297.336	-0.8635716	GSTM1	glutathione S-transferase mu 1
12.296.917	0.3384581	SP110	SP110 nuclear body protein
12.296.714	0.2174114	RPL22	ribosomal protein L22
12.294.645	0.588976	PALLD	palladin, cytoskeletal associated protein
12.293.545	-0.1969609	CEP164	centrosomal protein 164
1.229.265	0.3359634	PTPRF	protein tyrosine phosphatase, receptor type F
12.290.298	0.2931296	CAMK1	calcium/calmodulin dependent protein kinase
12.289.337	-0.5998913	NXN	nucleoredoxin
12.278.001	0.4099694	LYN	LYN proto-oncogene, Src family tyrosine kinase
12.276.174	-0.1883499	METTL5	methyltransferase like 5
12.274.745	0.289801	PRRC2A	proline rich coiled-coil 2A
12.273.478	-0.1530617	MYOT	myotilin
1.226.883	-0.1118632	HBBP1	hemoglobin subunit beta pseudogene 1
12.267.124	-0.1404565		
12.266.174	-0.1687242	ALK	anaplastic lymphoma receptor tyrosine kinase
12.261.972	-0.1606661	AFDN	afadin, adherens junction formation factor
12.260.978	-0.3403801	SLC25A10	solute carrier family 25 member 10
1.225.877	-0.2761343	MACF1	microtubule-actin crosslinking factor 1
12.258.736	0.3220392	NCOR2	nuclear receptor corepressor 2
12.255.876	-0.1055731		
12.252.359	-0.4120959	CAMKK2	calcium/calmodulin dependent protein kinase
12.249.294	0.3050727	PSMD9	proteasome 26S subunit, non-ATPase 9
1.224.195	0.282467	DET1	de-etiolated homolog 1 (Arabidopsis)
12.240.898	-0.1387805		
12.234.672	-0.1690988	CMKLR1	chemerin chemokine-like receptor 1
12.232.191	0.154596	TDRKH	tudor and KH domain containing
12.230.658	-0.1876766	RAPGEF2	Rap guanine nucleotide exchange factor 2
12.225.061	-0.1763526	CHD5	chromodomain helicase DNA binding protein 5

12.212.566	0.406584	ARHGEF2	Rho/Rac guanine nucleotide exchange factor 2
12.209.544	-0.3508863	RTN4	reticulon 4
12.205.035	-0.4304706	CCDC68	coiled-coil domain containing 68
12.196.442	0.2290897	HSPB11	heat shock protein family B (small) member 11
12.194.585	0.3594776	PDXK	pyridoxal (pyridoxine, vitamin B6) kinase
12.193.435	0.6008384	EIF2S3	eukaryotic translation initiation factor 2 subunit
12.190.523	0.4769955	PSIP1	PC4 and SFRS1 interacting protein 1
12.188.534	0.4200243	PPP6C	protein phosphatase 6 catalytic subunit
12.187.375	-0.1448563	CASP10	caspase 10
12.184.084	0.2513538	PLCE1	phospholipase C epsilon 1
1.218.369	0.3443679	IL22RA1	interleukin 22 receptor subunit alpha 1
12.183.637	0.3319542	RUBCN	RUN and cysteine rich domain containing becli
12.183.201	0.3705716	PPP1CA	protein phosphatase 1 catalytic subunit alpha
12.175.565	-0.1733884	HYPM	huntingtin interacting protein M
1.217.479	0.3803174	NUP98	nucleoporin 98
1.217.243	0.4191451	LOC64250	succinate dehydrogenase complex subunit C p
12.171.863	-0.1561484	SPAM1	sperm adhesion molecule 1
12.157.474	-0.141511	PRR4	proline rich 4 (lacrimal)
12.155.137	0.4914073	TGFBR2	transforming growth factor beta receptor 2
12.148.196	0.3291384	YIF1A	Yip1 interacting factor homolog A, membrane tr
1.214.369	-0.1197738		
12.139.125	0.294834	SSX2IP	SSX family member 2 interacting protein
12.135.743	0.2087717	SURF2	surfeit 2
12.135.093	0.3270186	TMEM87A	transmembrane protein 87A
12.132.058	-0.1494445	KMT2A	lysine methyltransferase 2A
12.129.885	-0.1319906	BNC1	basonuclin 1
12.125.863	-0.1558791	SMR3B	submaxillary gland androgen regulated protein
12.121.556	-0.1886558		
12.117.859	-0.6061905	NR2F1	nuclear receptor subfamily 2 group F member 1
12.108.697	0.345863	SPAG1	sperm associated antigen 1
1.210.759	-0.1800172	KCNQ1	potassium voltage-gated channel subfamily Q r
12.100.998	0.6449826	CXCL5	C-X-C motif chemokine ligand 5
12.095.841	0.3099386	RAD17	RAD17 checkpoint clamp loader component
12.085.711	-0.1449003	PER2	period circadian clock 2
12.080.911	-0.2075019	IGHM	immunoglobulin heavy constant mu
12.076.288	0.3634453	CDC42EP1	CDC42 effector protein 1
12.065.182	-0.1228425	RXRB	retinoid X receptor beta
12.054.126	0.3363177	PHF3	PHD finger protein 3
12.049.899	-0.5896873	HLA-DPB1	major histocompatibility complex, class II, DP b
12.049.623	0.1987666	ZBTB18	zinc finger and BTB domain containing 18
12.049.243	0.2612007	SUPT6H	SPT6 homolog, histone chaperone
12.048.358	-0.576793	NNT	nicotinamide nucleotide transhydrogenase
12.048.232	-0.2992828	SIK3	SIK family kinase 3
12.038.269	0.2682335	ELMO2	engulfment and cell motility 2

12.032.625	0.6230136	PTH2R	parathyroid hormone 2 receptor
12.031.234	-0.1306282	PHF7	PHD finger protein 7
12.028.527	-0.1201858	OR10H2	olfactory receptor family 10 subfamily H memb
12.005.513	-0.2046432	PILRA	paired immunoglobin like type 2 receptor alpha
12.001.182	0.4924257	PRSS3	protease, serine 3
11.999.825	0.2664068	PBX2	PBX homeobox 2
11.994.394	-0.4338228	RTN4	reticulon 4
11.994.018	-0.5657021		
1.198.901	-0.2460217	ACAD10	acyl-CoA dehydrogenase family member 10
11.981.222	-0.259361	CAMKK2	calcium/calmodulin dependent protein kinase
11.976.939	-0.6266337	FGL1	fibrinogen like 1
11.973.924	0.2832566	DHDDS	dehydrodolichyl diphosphate synthase subunit
11.973.921	-0.1600331	TPT1P8	tumor protein, translationally-controlled 1 pseu
11.971.157	-0.1500746	CD6	CD6 molecule
11.964.622	-0.4789812	TMOD1	tropomodulin 1
11.963.674	0.3105386	ZMYM4	zinc finger MYM-type containing 4
1.195.952	0.3394537	CLTB	clathrin light chain B
11.953.432	-0.1608345	MARCH1	membrane associated ring-CH-type finger 1
11.943.163	0.3213632	SERBP1	SERPINE1 mRNA binding protein 1
11.933.282	-0.1362801	SQSTM1	sequestosome 1
11.921.731	0.1622674	LRRC14	leucine rich repeat containing 14
11.920.549	-0.1011079	PDZRN4	PDZ domain containing ring finger 4
11.919.432	0.25563	SMOX	spermine oxidase
11.917.311	0.2530465	TCF3	transcription factor 3
11.910.363	-0.149071		
11.909.553	0.2280564	NAT8B	N-acetyltransferase 8B (putative, gene/pseudo
11.907.321	-0.2028736	ADAMTS13	ADAM metallopeptidase with thrombospondin t
11.902.264	-0.3145952	REPS2	RALBP1 associated Eps domain containing 2
11.902.094	0.3309281	GRB2	growth factor receptor bound protein 2
11.886.827	-0.2169494	SFRP1	secreted frizzled related protein 1
11.886.663	0.2025382	COPB2	coatomer protein complex subunit beta 2
11.883.541	-0.2256278	VAV2	vav guanine nucleotide exchange factor 2
11.878.512	-0.1994417	KCNK10	potassium two pore domain channel subfamily
11.872.239	0.2999343	KLHL21	kelch like family member 21
11.868.019	-0.1908748	C11orf21	chromosome 11 open reading frame 21
11.867.231	0.374747	STAU1	staufen double-stranded RNA binding protein 1
11.865.467	-0.2194484	ABR	active BCR-related
11.862.308	-0.4253688	CREBL2	cAMP responsive element binding protein like 2
1.186.053	0.248732	SUPT6H	SPT6 homolog, histone chaperone
11.852.167	0.3260836	NSUN5P1	NOP2/Sun RNA methyltransferase family meml
11.845.087	-0.1471525	PTPN12	protein tyrosine phosphatase, non-receptor typ
11.843.757	0.4816927	PCSK1N	proprotein convertase subtilisin/kexin type 1 inl
11.834.831	-0.2633949	MLLT10	myeloid/lymphoid or mixed-lineage leukemia; t
11.805.111	-0.1876658	VSX1	visual system homeobox 1

11.792.039	-0.1495663	CRNN	cornulin
117.876	0.3759597	ADAMDEC1	ADAM like decysin 1
11.785.414	0.2837649	CNOT1	CCR4-NOT transcription complex subunit 1
11.775.941	0.2149424	SF3A1	splicing factor 3a subunit 1
11.775.488	-0.4085511	SDHD	succinate dehydrogenase complex subunit D
11.771.337	0.2347503	LOC64461	uncharacterized LOC644617
11.768.422	0.3549041	SLK	STE20 like kinase
11.756.361	0.2709	LOC64284	DEAD/H (Asp-Glu-Ala-Asp/His) box polypeptide
11.755.565	-0.252436	FPGS	folylpolyglutamate synthase
11.752.139	0.126211	ZBTB6	zinc finger and BTB domain containing 6
11.751.354	-0.1525888	C7orf26	chromosome 7 open reading frame 26
1.174.762	-0.5640047	MARC1	mitochondrial amidoxime reducing component
11.741.098	0.4151329	PRKCA	protein kinase C alpha
11.726.435	-0.5415591	JUN	Jun proto-oncogene, AP-1 transcription factor s
11.725.972	0.3330119	CUL5	cullin 5
11.719.499	-0.1493614	TP53TG5	TP53 target 5
11.715.993	0.3376411	EIF2AK3	eukaryotic translation initiation factor 2 alpha k
11.714.831	-0.126828	KRT31	keratin 31
11.710.616	-0.3296863	TP53TG1	TP53 target 1 (non-protein coding)
11.702.323	-0.1613556	TP53I11	tumor protein p53 inducible protein 11
1.170.028	-0.2160746	ELAVL3	ELAV like neuron-specific RNA binding protein 3
11.697.759	0.3613026	DAP	death associated protein
11.697.404	-0.1425162	DDR1-AS1	DDR1 antisense RNA 1 (head to head)
11.690.115	-0.1510452		
11.687.939	-0.3415941	RTN4	reticulon 4
1.168.119	-0.2218675	YAF2	YY1 associated factor 2
11.677.506	0.1842378	NEMP1	nuclear envelope integral membrane protein 1
11.676.517	-0.6216823		
116.644	-0.1541589	LOC10029	uncharacterized LOC100294145
11.663.536	-0.2205833	ADGRA2	adhesion G protein-coupled receptor A2
116.618	-0.3019581	LTBP1	latent transforming growth factor beta binding p
11.661.283	-0.145644	KCNK12	potassium two pore domain channel subfamily
11.659.132	-0.2243409	ZBTB44	zinc finger and BTB domain containing 44
11.657.479	-0.1552513	APOBEC2	apolipoprotein B mRNA editing enzyme catalyti
1.165.724	0.4241726	MAGEA2B/	MAGE family member A2B///MAGE family mem
11.653.926	-0.204669	AATK	apoptosis associated tyrosine kinase
11.651.722	-0.193058	TTLL3	tubulin tyrosine ligase like 3
11.649.498	-0.23169	ZC3H7B	zinc finger CCCH-type containing 7B
11.647.573	-0.3085651	PLD1	phospholipase D1
11.645.251	-0.3886937	IGHV4-31//	immunoglobulin heavy variable 4-31///immuno
11.642.351	0.4022434	OGT	O-linked N-acetylglucosamine (GlcNAc) transfe
11.641.377	-0.1339833	BTN2A3P	butyrophilin subfamily 2 member A3, pseudoge
11.639.697	0.3163419	RAD9A	RAD9 checkpoint clamp component A
11.639.384	0.2380612	KCTD2	potassium channel tetramerization domain cor

11.639.103	-0.1850501		
11.639.076	0.4415336	LGALS8	galectin 8
11.637.763	0.1922742	SERF1B///S	small EDRK-rich factor 1B///small EDRK-rich fa
11.637.192	-0.1229289	CDH4	cadherin 4
11.636.788	0.4986707	IPO5	importin 5
11.633.082	0.2943484	ATF6	activating transcription factor 6
1.162.878	-0.3331292	GK3P///GK	glycerol kinase 3 pseudogene///glycerol kinase
11.626.605	-0.1662948	FLI1	Fli-1 proto-oncogene, ETS transcription factor
11.624.518	-0.1615557	NEUROD6	neuronal differentiation 6
11.612.549	-0.248803	ANK3	ankyrin 3, node of Ranvier (ankyrin G)
11.612.365	0.2950866	MAP3K7CL	MAP3K7 C-terminal like
1.160.837	-0.3815172	MPRIP	myosin phosphatase Rho interacting protein
1.160.539	0.7321008	PEG3	paternally expressed 3
1.160.498	-0.1575349	INPP5D	inositol polyphosphate-5-phosphatase D
11.602.448	-0.1565912	THEG	theg spermatid protein
11.600.088	0.3097344	RANGRF	RAN guanine nucleotide release factor
11.598.694	0.2550255	HNRNPK	heterogeneous nuclear ribonucleoprotein K
11.596.975	-0.1488671		
11.595.731	0.3742418	CAPN7	calpain 7
11.589.129	0.3600527	PDCD4	programmed cell death 4 (neoplastic transform
11.583.256	-0.2552435	PLXNA2	plexin A2
11.571.067	-0.170614		
11.569.225	0.2492028	PAF1	PAF1 homolog, Paf1/RNA polymerase II comple
11.567.252	-0.136359	YIPF4	Yip1 domain family member 4
11.563.709	0.1362074	DHX29	DEAH-box helicase 29
11.560.987	-0.2773807	SASH1	SAM and SH3 domain containing 1
11.554.208	-0.1521247	PIGQ	phosphatidylinositol glycan anchor biosynthesi
11.553.825	0.386003	LAMP3	lysosomal associated membrane protein 3
11.550.342	-0.3349969		
11.540.483	-0.1506255	IL10	interleukin 10
115.402	-0.1505428	CTSZ	cathepsin Z
11.539.601	-0.1708874	SLC26A10	solute carrier family 26 member 10
11.539.259	0.3475646	DHFR	dihydrofolate reductase
11.539.029	-0.1475939	SLC9A7	solute carrier family 9 member A7
11.535.787	0.3071858	KIAA0232	KIAA0232
11.534.176	-0.3665885	RSL1D1	ribosomal L1 domain containing 1
11.533.135	-0.2065572	C2orf83	chromosome 2 open reading frame 83
11.531.199	-0.1486102	DKFZP434A	uncharacterized LOC26102
1.152.806	-0.1501959	PLCB2	phospholipase C beta 2
11.512.108	-0.1890617	DUX1	double homeobox 1
11.510.255	-0.1844676	PPIL2	peptidylprolyl isomerase like 2
11.509.208	-0.1233195	ADPRH	ADP-ribosylarginine hydrolase
11.506.685	0.9743371	KRT23	keratin 23
11.503.703	-0.1492008	PIK3R5	phosphoinositide-3-kinase regulatory subunit 5

11.501.522	0.2778564	CYP20A1	cytochrome P450 family 20 subfamily A membe
11.497.286	-0.2870938	RRBP1	ribosome binding protein 1
11.497.065	0.240662	WDR91	WD repeat domain 91
11.489.283	-0.1923798	PTCRA	pre T-cell antigen receptor alpha
114.865	-0.4079376	ECI1	enoyl-CoA delta isomerase 1
11.482.431	-0.2931001		
11.471.749	0.2160369	COX11	COX11, cytochrome c oxidase copper chaperor
11.471.267	-0.2021412	BAIAP2	BAI1 associated protein 2
11.467.783	0.5094584	CAV1	caveolin 1
11.464.302	0.2643281	JARID2	jumonji and AT-rich interaction domain contain
11.454.968	-0.217718	FYB	FYN binding protein
11.451.142	0.3589025		
11.450.839	-0.2489966	SNAPC4	small nuclear RNA activating complex polypept
11.447.383	0.4204492	GCNT3	glucosaminyl (N-acetyl) transferase 3, mucin ty
11.445.946	0.3778532	HOXA5	homeobox A5
11.438.403	-0.3052748	FCHSD2	FCH and double SH3 domains 2
11.429.623	-0.2152498	TEX13A	testis expressed 13A
11.427.256	-0.4171477	PNPO	pyridoxamine 5'-phosphate oxidase
11.424.498	0.2842191	CAMTA1	calmodulin binding transcription activator 1
11.423.734	-0.1437694	KIAA1654	KIAA1654 protein
11.419.316	-0.1127167	PITPNM3	PITPNM family member 3
11.417.324	-0.2794814	CCPG1	cell cycle progression 1
11.407.781	0.2593622	NAPG	NSF attachment protein gamma
11.404.625	-0.3487247	UBXN8	UBX domain protein 8
11.404.112	-0.2306766	SCAPER	S-phase cyclin A associated protein in the ER
11.401.588	0.2100387	TCOF1	treacle ribosome biogenesis factor 1
11.399.605	0.2565647	SLC29A2	solute carrier family 29 member 2
11.395.702	0.1934941	COX7C	cytochrome c oxidase subunit 7C
11.394.422	-0.1580356		
11.394.238	-0.1274629	GAP43	growth associated protein 43
11.393.526	-0.5542972	NR4A3	nuclear receptor subfamily 4 group A member 3
1.138.874	-0.3823511	RPL5	ribosomal protein L5
11.387.907	-0.1441813	EPHA3	EPH receptor A3
11.387.906	-0.7696161	RGS1	regulator of G-protein signaling 1
11.371.938	0.1562768	GART	phosphoribosylglycinamide formyltransferase,
11.368.313	-0.1401702	MYL2	myosin light chain 2
11.364.337	0.3958834	LYN	LYN proto-oncogene, Src family tyrosine kinase
11.361.472	-0.2653088	SMOX	spermine oxidase
11.361.165	-0.1754899	HAB1	B1 for mucin
11.361.131	0.2330392	ABHD4	abhydrolase domain containing 4
11.360.722	-0.1500687	PRO1848	PRO1848 protein
11.360.442	0.3241206	PARD3	par-3 family cell polarity regulator
11.359.459	-0.3109445	SLAMF7	SLAM family member 7
1.135.404	-0.3949313	ZC3H14	zinc finger CCCH-type containing 14

11.346.872	-0.6078091		
11.346.254	0.2835931	TTLL12	tubulin tyrosine ligase like 12
11.345.389	-0.3258355	MEIS3P1	Meis homeobox 3 pseudogene 1
11.335.846	0.3236514	EIF3CL///EI	eukaryotic translation initiation factor 3 subunit
11.335.844	-0.2526807	TRA2A	transformer 2 alpha homolog
11.335.125	0.3734983	DKFZP564C	DKFZP564C152 protein
11.333.774	0.3481531	SERBP1	SERPINE1 mRNA binding protein 1
11.331.659	0.4771368	PLPP2	phospholipid phosphatase 2
11.325.982	0.2677974	PEX1	peroxisomal biogenesis factor 1
11.320.606	-0.3484458	FDX1	ferredoxin 1
1.132.004	0.6007901	SOD2	superoxide dismutase 2, mitochondrial
11.315.071	0.2383654	ITPKC	inositol-trisphosphate 3-kinase C
11.313.229	-0.1419155	COL1A1	collagen type I alpha 1 chain
1.130.942	0.2231795	THAP11	THAP domain containing 11
11.307.883	0.2812173	LDAH	lipid droplet associated hydrolase
11.306.463	0.6752761	SETP4///SE	SET pseudogene 4///SET pseudogene 14///SET
11.305.212	0.3032108	GTF2B	general transcription factor IIB
11.301.802	-0.1990582		
1.129.797	0.2047402	WHRN	whirlin
11.286.946	0.2414182	TFE3	transcription factor binding to IGHM enhancer 3
11.284.224	-0.286062	LINC01314	long intergenic non-protein coding RNA 1314
11.281.896	-0.1582467	ASCL3	achaete-scute family bHLH transcription factor
11.268.473	0.3326086	RANBP2	RAN binding protein 2
11.267.176	-0.3034228	GIMAP5	GTPase, IMAP family member 5
11.267.169	0.217316	TLE3	transducin like enhancer of split 3
11.265.977	-0.1776868	CORO2B	coronin 2B
11.262.856	-0.1423713	HIST1H1A	histone cluster 1, H1a
11.260.126	-0.209544	LRCH4	leucine rich repeats and calponin homology do
11.252.606	-0.1966341	COL6A1	collagen type VI alpha 1 chain
11.249.127	0.456641	YBX1	Y-box binding protein 1
11.248.886	0.2396334	TUSC2	tumor suppressor candidate 2
11.242.981	-0.1047171	AMELX	amelogenin, X-linked
11.239.825	0.2356571	RASSF1	Ras association domain family member 1
11.238.829	-0.6869249	MAGEA4///	MAGE family member A4///interleukin 6 signal t
11.238.436	-0.2929082	UBR4	ubiquitin protein ligase E3 component n-recogr
11.233.024	0.2510466	TIMM44	translocase of inner mitochondrial membrane 4
11.230.258	-0.1018109	STOM	stomatin
1.122.941	0.3782788	ETNK1	ethanolamine kinase 1
11.227.578	-0.1666916	LINC00483	long intergenic non-protein coding RNA 483
1.122.647	-0.106199	BMP8A	bone morphogenetic protein 8a
11.226.276	0.3242397	WDR48	WD repeat domain 48
11.224.803	0.2569256	JAG2	jagged 2
11.215.371	-0.2379715	TAF1C	TATA-box binding protein associated factor, RN
11.204.235	-0.9921299	IGFBP2	insulin like growth factor binding protein 2

11.197.526	-0.5249521	KYNU	kynureninase
11.196.672	-0.2498507	S1PR1	sphingosine-1-phosphate receptor 1
11.195.822	-0.3205993	C16orf62	chromosome 16 open reading frame 62
11.195.079	-0.1299903	PEX5L	peroxisomal biogenesis factor 5 like
11.192.608	-0.4463183	MSMO1	methylsterol monooxygenase 1
11.192.046	-0.3161731		
11.191.278	-0.5953451	GBP1	guanylate binding protein 1
11.184.725	0.4017472	OSBPL8	oxysterol binding protein like 8
1.118.217	-0.1389533	NR2E3	nuclear receptor subfamily 2 group E member 3
11.172.948	0.3077247	RABEP2	rabaptin, RAB GTPase binding effector protein 2
11.171.027	-0.1919921	ANKDD1A	ankyrin repeat and death domain containing 1A
11.170.844	-0.132716		
11.168.294	0.2600827	SFI1	SFI1 centrin binding protein
11.162.133	-0.1278276	PRKG1	protein kinase, cGMP-dependent, type I
11.161.778	-0.1482153	NECTIN1	nectin cell adhesion molecule 1
11.151.626	-0.1312969	TNR	tenascin R
11.148.241	-0.1189204		
11.147.735	-0.5985793	ELOVL6	ELOVL fatty acid elongase 6
11.146.762	-0.2298077	ARHGAP32	Rho GTPase activating protein 32
11.146.156	0.2524568	TMED2	transmembrane p24 trafficking protein 2
11.138.259	-0.2224628	TBC1D9	TBC1 domain family member 9
11.135.815	0.3982323	SLC12A8	solute carrier family 12 member 8
11.133.391	0.2320395	COX6A1	cytochrome c oxidase subunit 6A1
11.133.146	0.1710021	DLG5	discs large MAGUK scaffold protein 5
11.115.627	-0.1745913	VPS35	VPS35 retromer complex component
11.110.377	-0.1815707	DOK2	docking protein 2
11.108.395	-0.1271705	NCR3	natural cytotoxicity triggering receptor 3
11.108.325	0.2728381	ZNF410	zinc finger protein 410
11.108.109	0.335279	PRPS1	phosphoribosyl pyrophosphate synthetase 1
11.105.609	-0.1436424	LOC64215(	similar to Cationic trypsin III precursor (Pretryps
11.095.372	-0.1633248	VAV1	vav guanine nucleotide exchange factor 1
11.093.866	-0.3459064	CBX7	chromobox 7
11.084.129	-0.1714052	SOCS1	suppressor of cytokine signaling 1
11.083.857	-0.2660281	HDAC9	histone deacetylase 9
1.108.127	-0.1670258	ARID1A	AT-rich interaction domain 1A
11.081.268	-0.1354511	NCAM2	neural cell adhesion molecule 2
11.079.121	-0.1649279	PTCRA	pre T-cell antigen receptor alpha
1.107.865	-0.519566	PTPRC	protein tyrosine phosphatase, receptor type C
11.077.912	0.3453438	NUCKS1	nuclear casein kinase and cyclin dependent kir
11.077.479	0.2487222	ZNF354A	zinc finger protein 354A
11.074.408	-0.3192451	DIAPH2	diaphanous related formin 2
11.072.655	-0.5223351		
1.107.016	-0.3296447	PEX3	peroxisomal biogenesis factor 3
11.069.778	0.2598043	DLG1	discs large MAGUK scaffold protein 1

11.068.288	-0.1587256	CHAT	choline O-acetyltransferase
11.063.507	-0.1569234		
11.062.834	0.8766348	VCAN	versican
11.062.252	-0.1311536	RPL19P9	ribosomal protein L19 pseudogene 9
11.060.405	-0.5122713	CD8A	CD8a molecule
11.050.348	-0.147829	SMG7-AS1	SMG7 antisense RNA 1
110.465	0.3054243	UBE4B	ubiquitination factor E4B
11.040.416	0.6294123	RARRES1	retinoic acid receptor responder 1
11.040.285	-0.1804458	PRH2///PRI	proline rich protein HaeIII subfamily 2///proline
1.103.568	0.3017753	PIH1D1	PIH1 domain containing 1
11.034.011	-0.2570577	ZBTB43	zinc finger and BTB domain containing 43
1.103.384	-0.1231886	PNMA2	paraneoplastic Ma antigen 2
11.033.009	0.4704975	ATP2B4	ATPase plasma membrane Ca2+ transporting 4
11.032.706	-0.1449622	GIMAP5	GTPase, IMAP family member 5
11.029.187	-0.1970235	SERINC2	serine incorporator 2
11.028.394	0.9159084	AGR2	anterior gradient 2, protein disulphide isomeras
1.101.698	0.2326596	NRDC	nardilysin convertase
11.013.594	-0.1286317	ADAMTS8	ADAM metallopeptidase with thrombospondin t
11.009.674	0.4842394	SCPEP1	serine carboxypeptidase 1
11.004.843	0.40934	FKBP4	FK506 binding protein 4
11.004.568	0.2591614	SUMO1	small ubiquitin-like modifier 1
11.003.856	0.3738429	TNPO1	transportin 1
11.003.553	-0.1592548	OLIG2	oligodendrocyte lineage transcription factor 2
11.003.295	-0.2647798	CRBN	cereblon
1.100.281	-0.1657806	GCNT4	glucosaminyl (N-acetyl) transferase 4, core 2
11.002.775	0.3053694	PTPN2	protein tyrosine phosphatase, non-receptor typ
1.099.605	0.3465511	NPEPPS	aminopeptidase puromycin sensitive
10.993.625	-0.1537977	DOC2B	double C2 domain beta
10.990.424	0.2342532	ERCC8	ERCC excision repair 8, CSA ubiquitin ligase co
10.988.615	0.2200166	CNPPD1	cyclin Pas1/PHO80 domain containing 1
10.987.557	0.22537	BOP1	block of proliferation 1
10.970.916	-0.457896	TRA2A	transformer 2 alpha homolog
10.969.572	-0.3396797	LPP	LIM domain containing preferred translocation
10.968.226	0.2953502	CISD1	CDGSH iron sulfur domain 1
10.960.938	-0.1209951	PKD1	polycystin 1, transient receptor potential chann
10.956.123	-0.1704196		
10.955.455	-0.2463356	PLEK	pleckstrin
10.954.982	0.1708936	VAMP4	vesicle associated membrane protein 4
10.953.621	-0.1509949	KCNJ13	potassium voltage-gated channel subfamily J m
10.951.153	-0.1335807	SH2D1A	SH2 domain containing 1A
1.095.043	0.2350681	STAM2	signal transducing adaptor molecule 2
1.093.796	-0.1809893	PKD1	polycystin 1, transient receptor potential chann
10.935.027	-0.1742307	CPNE6	copine 6
10.931.101	-0.3476371	NADK	NAD kinase

10.929.226	0.3553816	PRKD3	protein kinase D3
10.927.736	-0.1432182		
10.923.316	-0.2643079	NR4A3	nuclear receptor subfamily 4 group A member 3
10.921.858	0.1820328	PIP5K1A	phosphatidylinositol-4-phosphate 5-kinase typ
10.919.599	0.4367712	PSMC3	proteasome 26S subunit, ATPase 3
1.091.671	0.4241721	ELF3	E74 like ETS transcription factor 3
1.091.148	-0.1720623		
1.091.039	0.2133697	TM2D1	TM2 domain containing 1
10.905.382	0.382172	CCL8	C-C motif chemokine ligand 8
10.901.611	0.2615646	PPP6R3	protein phosphatase 6 regulatory subunit 3
10.900.057	-0.1866195	ADARB1	adenosine deaminase, RNA specific B1
10.899.712	0.2776571	UBE3A	ubiquitin protein ligase E3A
10.898.472	0.1997905	BHLHB9	basic helix-loop-helix domain containing, class
10.897.584	0.3568919	SNX6	sorting nexin 6
10.891.765	0.2440948	COA7	cytochrome c oxidase assembly factor 7 (putati
10.882.845	-0.1716921	CD70	CD70 molecule
10.877.951	-0.1888435	NTRK3	neurotrophic receptor tyrosine kinase 3
10.876.803	0.5007892	ANP32A	acidic nuclear phosphoprotein 32 family memb
10.874.117	0.2469977	GTPBP1	GTP binding protein 1
10.871.522	-0.1349714	GDF3	growth differentiation factor 3
10.868.612	-0.2932794	ZHX3	zinc fingers and homeoboxes 3
10.867.951	-0.2812761	MAN2C1	mannosidase alpha class 2C member 1
10.867.721	-0.1157606	IL9	interleukin 9
1.086.736	0.2730702	FBXO34	F-box protein 34
10.863.841	0.3803325	MAT2A	methionine adenosyltransferase 2A
10.862.702	-0.1373358	MGAT5	mannosyl (alpha-1,6-)-glycoprotein beta-1,6-N
10.860.367	-0.1651188	DNAH3	dynein axonemal heavy chain 3
10.856.478	-0.2399712		
10.855.393	-0.46323	COX7A1	cytochrome c oxidase subunit 7A1
10.855.283	-0.1851048	KIR2DS1	killer cell immunoglobulin like receptor, two Ig d
10.853.066	-0.2631774	MDM2	MDM2 proto-oncogene
1.084.884	-0.5266062	IL13RA2	interleukin 13 receptor subunit alpha 2
10.846.395	-0.3902388	ANXA3	annexin A3
10.842.553	0.3687198	NAA15	N(alpha)-acetyltransferase 15, NatA auxiliary su
10.839.368	-0.2668408	CYLD	CYLD lysine 63 deubiquitinase
10.838.001	0.2982107	ANXA7	annexin A7
10.833.812	-0.135148	GSN-AS1	GSN antisense RNA 1
10.825.969	-0.1157194	CBX2	chromobox 2
10.823.274	-0.1914263	GNAS	GNAS complex locus
10.822.327	-0.1426316	RASGRF1	Ras protein specific guanine nucleotide releasi
10.822.205	-0.3210588	ENGASE	endo-beta-N-acetylglucosaminidase
10.819.429	0.4007785	CHMP5	charged multivesicular body protein 5
1.081.901	0.2102384	EP400	E1A binding protein p400
10.816.039	-0.1673041	NEUROG1	neurogenin 1

10.812.656	0.1957028	NME6	NME/NM23 nucleoside diphosphate kinase 6
10.807.413	-0.4015173	TRIM2	tripartite motif containing 2
10.807.305	-0.1531056	LRTOMT	leucine rich transmembrane and O-methyltrans
10.803.417	-0.2190104	CILP	cartilage intermediate layer protein
10.797.438	0.3801545	PRRC2C	proline rich coiled-coil 2C
10.791.882	0.1883464	ZFAND3	zinc finger AN1-type containing 3
10.791.041	0.4079954	ETNK1	ethanolamine kinase 1
1.078.957	-0.1380433	EPB41L4A	erythrocyte membrane protein band 4.1 like 4A
1.078.354	-0.1285436		
1.077.824	-0.1257259	TSSK2	testis specific serine kinase 2
10.778.172	-0.1977124	BCL3	B-cell CLL/lymphoma 3
10.776.157	-0.1315577		
10.774.487	-0.1612895	GPR137	G protein-coupled receptor 137
10.769.677	-0.15725	LTK	leukocyte receptor tyrosine kinase
10.769.182	0.2508555	FASTK	Fas activated serine/threonine kinase
10.766.054	0.330654	PSMB1	proteasome subunit beta 1
10.765.689	-0.1787963	PAPPA	pappalysin 1
10.762.632	-0.3073218	SH3BP5	SH3 domain binding protein 5
10.759.297	-0.2622526	CXCL8	C-X-C motif chemokine ligand 8
10.755.289	-0.1512121	SFTPB	surfactant protein B
10.750.986	-0.2033255	C2CD2L	C2CD2 like
10.748.867	-0.1186103	CPM	carboxypeptidase M
10.743.892	-0.5820846	PLEK2	pleckstrin 2
10.728.794	-0.3004749	MYO1C	myosin IC
10.727.514	0.3025551	EP400	E1A binding protein p400
10.723.658	0.3791833	PKIA	protein kinase (cAMP-dependent, catalytic) inh
10.722.047	-0.3414456	N4BP2L2	NEDD4 binding protein 2 like 2
10.719.498	-0.4243751	BTN3A2///E	butyrophilin subfamily 3 member A2///butyroph
10.719.414	-0.111423	MBNL1	muscleblind like splicing regulator 1
10.717.182	-0.2420814	EML3	echinoderm microtubule associated protein lik
10.714.373	0.3912157	BIN1	bridging integrator 1
10.712.827	0.3625799	MAGOH	mago homolog, exon junction complex core co
10.712.794	0.3589447	KIAA0895	KIAA0895
10.712.433	-0.1843793	TCP10L2///	t-complex 10-like 2///t-complex 10
10.703.499	0.1757701	RAD54L	RAD54-like (S. cerevisiae)
10.692.928	-0.1904589	TTC38	tetratricopeptide repeat domain 38
10.690.559	0.3669367	SECTM1	secreted and transmembrane 1
1.069.025	-0.175197	GPRIN2	G protein regulated inducer of neurite outgrowt
10.689.711	-0.1304862	RFPL1	ret finger protein like 1
10.688.804	0.3015885	BFAR	bifunctional apoptosis regulator
10.688.594	0.1881754	BRE	brain and reproductive organ-expressed (TNFR
10.687.052	0.1783701	MAPK7	mitogen-activated protein kinase 7
10.687.033	-0.2859373	CD3E	CD3e molecule
10.686.296	0.3025301	ANXA11	annexin A11

10.685.893	-0.1923906	FGF12	fibroblast growth factor 12
10.685.591	-0.1179055	ASTN2	astrotactin 2
10.683.254	0.1556137	WDHD1	WD repeat and HMG-box DNA binding protein 1
10.682.661	-0.5274856		
10.681.536	-0.2183919	GABRR2	gamma-aminobutyric acid type A receptor rho2
10.680.607	-0.1063891		
10.679.439	0.3105596	ZNF232	zinc finger protein 232
10.675.823	0.2859889	KTN1	kinectin 1
10.667.124	0.3167245	DYRK1A	dual specificity tyrosine phosphorylation regula
10.664.722	0.5589476	SNURF///SI	SNRPN upstream reading frame///small nuclea
10.655.684	-0.2554664	ZBTB7A	zinc finger and BTB domain containing 7A
10.655.485	0.2817195	BICD2	BICD cargo adaptor 2
10.655.032	0.3298377	CCNC	cyclin C
10.647.631	-0.175423	PRRG2	proline rich and Gla domain 2
10.646.329	0.209144	ZNF222	zinc finger protein 222
1.064.621	-0.1160199	POU6F1	POU class 6 homeobox 1
10.631.282	-0.1234241	CD300C	CD300c molecule
10.626.648	-0.1921371	C1orf159	chromosome 1 open reading frame 159
10.624.446	-0.121233	KIR3DL3	killer cell immunoglobulin like receptor, three Ig
10.623.034	-0.7193828	THBS1	thrombospondin 1
10.609.572	-0.1995438	TNK1	tyrosine kinase non receptor 1
10.607.871	-0.3591993	TBC1D3H//	TBC1 domain family member 3H///TBC1 domai
10.605.809	-0.1554932	CDKL1	cyclin dependent kinase like 1
1.060.137	-0.195008	ESRRG	estrogen related receptor gamma
10.601.008	-0.4037367	PRSS8	protease, serine 8
10.600.494	-0.1173241	TNF	tumor necrosis factor
10.597.598	0.4299988	PSMC2	proteasome 26S subunit, ATPase 2
10.597.412	-0.1555686	LTK	leukocyte receptor tyrosine kinase
10.593.944	-0.1471231	ZC3H13	zinc finger CCCH-type containing 13
1.059.255	0.6112983	LOC64250:	succinate dehydrogenase complex subunit C p
10.584.376	0.3188924	LARS2	leucyl-tRNA synthetase 2, mitochondrial
10.578.266	0.3064343	MKRN1	makorin ring finger protein 1
1.056.766	-0.3621244		
1.056.633	0.4400329	NINJ2	ninjurin 2
10.564.539	-0.1490022	SEPT11	septin 11
10.560.936	0.2537083	RNF19B	ring finger protein 19B
10.547.332	-0.1483383		
10.546.618	-0.2023017	VPS39	VPS39, HOPS complex subunit
10.546.041	-0.2399509	MAP2K7	mitogen-activated protein kinase kinase 7
10.543.732	-0.1489647	NKX2-1	NK2 homeobox 1
10.543.583	0.2092416	RPL22	ribosomal protein L22
10.530.854	-0.4041639	PCDHGA1/	protocadherin gamma subfamily A, 1///protoca
10.530.475	0.3000438	APPBP2	amyloid beta precursor protein binding protein
10.522.839	-0.2141553	PPP2R2D	protein phosphatase 2 regulatory subunit Bdelt

10.515.064	-0.3613764	MCL1	BCL2 family apoptosis regulator
10.514.569	-0.1484782	SCGB2A2	secretoglobin family 2A member 2
10.513.547	0.30931	PARD3	par-3 family cell polarity regulator
1.051.327	0.2384779	TOMM70	translocase of outer mitochondrial membrane 7
10.510.141	-0.1799213	TJP1	tight junction protein 1
10.495.336	-0.4276289	INHBC	inhibin beta C subunit
10.493.618	-0.4603764	RAB27A	RAB27A, member RAS oncogene family
10.493.176	0.2101057	GCOM1///F	GRINL1A complex locus 1///RNA polymerase II
10.489.471	-0.2333485	TUBGCP2	tubulin gamma complex associated protein 2
10.469.013	0.5077231	GMDS	GDP-mannose 4,6-dehydratase
10.467.211	-0.1596057	ALG8	ALG8, alpha-1,3-glucosyltransferase
10.464.195	0.3889666	SLC25A32	solute carrier family 25 member 32
10.461.121	0.2566182	PTPN11	protein tyrosine phosphatase, non-receptor typ
10.455.696	0.2605356	CNOT8	CCR4-NOT transcription complex subunit 8
10.450.957	0.1936688	GRWD1	glutamate rich WD repeat containing 1
1.043.536	-0.1717922	SLC1A6	solute carrier family 1 member 6
10.426.471	-0.141529	IL21	interleukin 21
10.424.621	-0.2977707	PRKACA	protein kinase cAMP-activated catalytic subuni
10.424.445	-0.402508	FGL2	fibrinogen like 2
10.418.925	-0.166477	INE1	inactivation escape 1 (non-protein coding)
10.410.505	0.4172307	IPO5	importin 5
10.408.285	-0.1595496	LTC4S	leukotriene C4 synthase
10.407.946	-0.1474115	IGHA1	immunoglobulin heavy constant alpha 1
10.407.733	-0.2472779	ATL2	atlastin GTPase 2
10.407.011	0.3083357	CTBP1	C-terminal binding protein 1
10.405.821	-0.2052247	TTC38	tetratricopeptide repeat domain 38
10.405.322	0.2273836	MYO9B	myosin IXB
10.400.155	-0.1392563	NCAM1	neural cell adhesion molecule 1
10.399.678	0.3998157	PCSK5	proprotein convertase subtilisin/kexin type 5
10.397.943	0.1520865	TJAP1	tight junction associated protein 1
10.392.927	-0.1373099	SBNO1	strawberry notch homolog 1
10.391.245	-0.1843448	OSBPL10	oxysterol binding protein like 10
1.038.849	-0.3098113	CST7	cystatin F
10.381.579	0.2842217	WTAP	Wilms tumor 1 associated protein
10.381.372	-0.145252	TPT1	tumor protein, translationally-controlled 1
10.380.703	0.1666091	SERBP1	SERPINE1 mRNA binding protein 1
10.372.407	-0.1481647	ADAM22	ADAM metallopeptidase domain 22
10.369.659	0.1817594	PUS7L	pseudouridylate synthase 7 like
10.360.234	0.326687	JADE2	jade family PHD finger 2
10.359.874	0.3044866	YPEL5	yippee like 5
10.357.725	-0.1594565		
10.356.985	-0.2125951	LINC00652	long intergenic non-protein coding RNA 652
10.351.055	0.2343223	PPIL2	peptidylprolyl isomerase like 2
1.035.062	-0.1660351	PTPRD	protein tyrosine phosphatase, receptor type D

10.349.728	0.3428708	LYPLA1	lysophospholipase I
10.348.047	0.4827783	TNFAIP3	TNF alpha induced protein 3
10.345.544	-0.1617996	PCDH11Y	protocadherin 11 Y-linked
10.341.293	-0.298427	KDSR	3-ketodihydrosphingosine reductase
10.338.418	-0.2166666	CDK10	cyclin dependent kinase 10
10.335.204	-0.2893859	MACF1	microtubule-actin crosslinking factor 1
10.324.607	-0.1402248	FETUB	fetuin B
1.032.182	0.4638813	SEZ6L2	seizure related 6 homolog like 2
1.031.208	0.2763998	MAZ	MYC associated zinc finger protein
10.297.869	-0.3615185	SLC35E1	solute carrier family 35 member E1
10.295.083	0.1948219	SMPD2	sphingomyelin phosphodiesterase 2
10.293.112	-0.2191664	C1orf50	chromosome 1 open reading frame 50
10.292.884	0.3647031	UBE2B	ubiquitin conjugating enzyme E2 B
1.029.273	0.2259772	XAGE3///PS	X antigen family member 3///proteasome subu
10.291.111	0.193154	XRCC4	X-ray repair cross complementing 4
10.269.212	0.3231882	UBE2V1P2/	ubiquitin conjugating enzyme E2 V1 pseudogen
10.264.196	-0.1859847	SCAPER	S-phase cyclin A associated protein in the ER
10.260.211	-0.1403933	HHCM	Mahlavu hepatocellular carcinoma
10.259.954	-0.134046	FCGR2C	Fc fragment of IgG receptor IIc (gene/pseudoge
10.259.741	-0.208198	ADAMTS8	ADAM metallopeptidase with thrombospondin
10.259.187	0.2262027	RPS15A	ribosomal protein S15a
10.256.057	-0.1884145	CDH18	cadherin 18
10.254.224	0.2613392	TMEM214	transmembrane protein 214
10.254.062	-0.16368		
10.252.569	-0.3629416	PGF	placental growth factor
10.249.426	-0.2434296	PRDM2	PR/SET domain 2
10.245.642	0.3432919	BIN1	bridging integrator 1
1.024.331	-0.1381638	CD1B	CD1b molecule
10.242.251	-0.1135471		
10.239.322	0.365386	ATXN10	ataxin 10
10.236.983	-0.2183779	ZBTB7A	zinc finger and BTB domain containing 7A
10.236.579	-0.1435949	IDUA	iduronidase, alpha-L-
10.236.156	-0.1534321	VPS53	VPS53, GARP complex subunit
10.227.548	-0.2624735	HADHB	hydroxyacyl-CoA dehydrogenase/3-ketoacyl-C
10.219.646	-0.3460158	TMPRSS2	transmembrane protease, serine 2
10.215.599	-0.1713954	CNGB1	cyclic nucleotide gated channel beta 1
10.210.653	-0.5040039	C5	complement component 5
10.207.988	0.2815837	TRPM4	transient receptor potential cation channel sub
10.206.853	-0.1723245		
10.203.196	-0.1818196	SPANXB1	SPANX family member B1
10.202.524	-0.3867791	DECR2	2,4-dienoyl-CoA reductase 2, peroxisomal
10.197.499	0.2451207	CENPBD1P	CENPB DNA-binding domains containing 1 pse
10.192.512	0.5178619	HIST1H2BK	histone cluster 1, H2bk
10.186.664	-0.2329048	ZNF747	zinc finger protein 747

10.186.086	-0.1704615	ATOH1	atonal bHLH transcription factor 1
10.180.889	0.2605484	PGAP2	post-GPI attachment to proteins 2
10.178.692	-0.2173284	INPP5K	inositol polyphosphate-5-phosphatase K
10.172.038	0.3365346	FRAT1	frequently rearranged in advanced T-cell lymph
10.170.003	-0.2785553	ARAF	A-Raf proto-oncogene, serine/threonine kinase
10.163.114	0.1877686	CDH13	cadherin 13
10.160.271	-0.1651409	S1PR4	sphingosine-1-phosphate receptor 4
10.159.967	-0.3192248	EVI5	ecotropic viral integration site 5
10.157.958	0.1864716	TUBD1	tubulin delta 1
10.147.857	0.2616167	COX7B	cytochrome c oxidase subunit 7B
10.146.584	0.3365651	SLC4A7	solute carrier family 4 member 7
10.145.612	0.194211	MOB3B	MOB kinase activator 3B
10.144.935	0.3049115	LRRC37A2	leucine rich repeat containing 37 member A2
10.139.692	-0.4006412	GIMAP6	GTPase, IMAP family member 6
10.133.834	-0.2162545	PTPN22	protein tyrosine phosphatase, non-receptor typ
10.132.324	-0.1323475	CA12	carbonic anhydrase 12
10.130.151	-0.1504896	FGF6	fibroblast growth factor 6
10.128.129	-0.1711829	GJB4	gap junction protein beta 4
10.127.245	-0.5388759	ITPR2	inositol 1,4,5-trisphosphate receptor type 2
10.124.086	-0.1386783	RGR	retinal G protein coupled receptor
10.122.632	-0.1137075	TRIO	trio Rho guanine nucleotide exchange factor
1.011.917	-0.2228663	KRT4	keratin 4
1.011.873	0.2310945	XPO6	exportin 6
10.118.218	0.2058484	DRG2	developmentally regulated GTP binding protein
10.117.131	-0.1895366	NCS1	neuronal calcium sensor 1
10.106.495	0.1641299	HIST1H3D	histone cluster 1, H3d
1.010.135	-0.0892677	COG5	component of oligomeric golgi complex 5
10.099.469	-0.1998443	IKZF1	IKAROS family zinc finger 1
10.096.555	-0.2959074	LCK	LCK proto-oncogene, Src family tyrosine kinase
10.095.808	-0.1287141	SAMD4A	sterile alpha motif domain containing 4A
10.092.281	0.2645947	MBD1	methyl-CpG binding domain protein 1
10.083.827	-0.2374476	THBS1	thrombospondin 1
10.080.258	0.4419245	BTG3	BTG anti-proliferation factor 3
10.077.572	-0.1479347	NKX3-1	NK3 homeobox 1
10.068.621	-0.1619661	RBBP9	RB binding protein 9, serine hydrolase
10.066.854	-0.3553111	SESN1	sestrin 1
10.065.718	-0.2968645	CELF2	CUGBP, Elav-like family member 2
10.064.136	0.4054147	OAZ1	ornithine decarboxylase antizyme 1
10.062.849	-0.1557021	MUC2	mucin 2, oligomeric mucus/gel-forming
10.059.197	0.3467023	MCUB	mitochondrial calcium uniporter dominant neg
10.055.288	-0.1912314	ZER1	zyg-11 related cell cycle regulator
10.054.833	0.2946125	MAP3K4	mitogen-activated protein kinase kinase kinase
10.054.149	-0.2026688	LRRC40	leucine rich repeat containing 40
10.049.718	-0.1622302	TBX6	T-box 6

10.046.164	-0.204631	ENTPD4	ectonucleoside triphosphate diphosphohydrola
1.004.424	-0.1887337	NRG1	neuregulin 1
10.042.676	-0.1714135	ESR1	estrogen receptor 1
10.035.805	-0.1001296	PCDH11Y//	protocadherin 11 Y-linked///protocadherin 11 X
10.034.382	-0.3113258	NIPSNAP1	nipsnap homolog 1 (C. elegans)
10.021.102	-0.234553	NMT2	N-myristoyltransferase 2
10.015.401	-0.1398295	HMP19	HMP19 protein
10.012.455	0.2647568	OGT	O-linked N-acetylglucosamine (GlcNAc) transfe
10.012.214	0.2077068	MTF2	metal response element binding transcription f
10.012.012	-0.2158487	CPLX3	complexin 3
10.011.486	0.1913565	HINFP	histone H4 transcription factor
10.007.761	0.2715048	RIC8B	RIC8 guanine nucleotide exchange factor B
1.000.193	0.4341227	MRPS28	mitochondrial ribosomal protein S28
9.998.347	-0.2042144	LTBP4	latent transforming growth factor beta binding p
9.997.095	-0.2052488	SPG21	spastic paraplegia 21 (autosomal recessive, Ma
9.996.117	-0.2156659	PPP2R5C	protein phosphatase 2 regulatory subunit B'gan
9.995.285	-0.4722852	MAFB	MAF bZIP transcription factor B
9.995.092	-0.1393941	TPSD1	tryptase delta 1
9.992.803	-0.1596555	CD28	CD28 molecule
9.985.075	-0.1152176	KRT9	keratin 9
9.978.077	-0.1346571	EPB41L4A-	EPB41L4A antisense RNA 2 (head to head)
9.977.417	0.1860027	NHEJ1	non-homologous end joining factor 1
9.972.126	-0.1273546	ZNF423	zinc finger protein 423
9.968.785	0.2302379	SRPK1	SRSF protein kinase 1
9.967.998	0.5290139	SULF1	sulfatase 1
9.965.191	0.1569658	SETDB1	SET domain bifurcated 1
9.964.858	0.2796695	PYCR1	pyrroline-5-carboxylate reductase 1
9.962.646	0.2720708	OXA1L	OXA1L, mitochondrial inner membrane protein
9.960.458	0.2618273	YLPM1	YLP motif containing 1
9.958.022	-0.125582	HLA-DOA	major histocompatibility complex, class II, DO a
9.957.922	0.2608521	HNRNPH3	heterogeneous nuclear ribonucleoprotein H3
9.955.651	0.2805769	YY1	YY1 transcription factor
9.955.467	-0.1857408	ZCCHC14	zinc finger CCHC-type containing 14
9.943.785	-0.1250019	TP63	tumor protein p63
9.943.489	0.2294609	HGH1	HGH1 homolog
9.941.219	-0.1086891	ST8SIA4	ST8 alpha-N-acetyl-neuraminide alpha-2,8-sial
9.936.033	-0.3056965	ECH1	enoyl-CoA hydratase 1
9.927.336	-0.1747483	NTRK3	neurotrophic receptor tyrosine kinase 3
9.926.221	-0.3631893	HNF4A	hepatocyte nuclear factor 4 alpha
9.925.713	-0.1566407	SCN10A	sodium voltage-gated channel alpha subunit 10
9.922.517	0.2347617	DLG1	discs large MAGUK scaffold protein 1
9.918.898	0.2007545	RBM19	RNA binding motif protein 19
9.916.352	-0.2361713	TNS1	tensin 1
9.914.044	0.2165963	BBS4	Bardet-Biedl syndrome 4

9.908.478	0.1107166	ZCCHC4	zinc finger CCHC-type containing 4
9.902.323	-0.2024036	MYLPF	myosin light chain, phosphorylatable, fast skele
989.381	0.273267	HTATSF1	HIV-1 Tat specific factor 1
9.893.683	-0.1589765	NEUROD4	neuronal differentiation 4
988.462	-0.1451873	FCER2	Fc fragment of IgE receptor II
9.879.247	-0.2305556	SLC2A8	solute carrier family 2 member 8
9.863.526	-0.4750979	CALD1	caldesmon 1
9.861.871	0.1586299	AP3D1	adaptor related protein complex 3 delta 1 subu
9.824.099	-0.1406946	SEMA4G	semaphorin 4G
9.821.531	0.186079	ATP5SL	ATP5S like
9.821.225	-0.1740076	IFT122	intraflagellar transport 122
9.818.931	0.2856053	TRIOBP	TRIO and F-actin binding protein
9.817.106	-0.1455377	AKAP8L	A-kinase anchoring protein 8 like
9.813.629	0.2755555	SMARCC2	SWI/SNF related, matrix associated, actin depe
980.856	-0.5867131	NAMPT	nicotinamide phosphoribosyltransferase
9.806.517	0.2618112	UGGT2	UDP-glucose glycoprotein glucosyltransferase
9.804.125	-0.3728662	LOC64839	similar to ubiquitin B///ubiquitin B
9.802.388	-0.1729529	IGFBP5	insulin like growth factor binding protein 5
9.798.874	-0.5588489	MLIP	muscular LMNA-interacting protein
979.827	-0.4923873	GK	glycerol kinase
9.796.136	-0.183268	TNFRSF13E	TNF receptor superfamily member 13B
9.794.961	0.2191131	EXOSC7	exosome component 7
9.793.655	0.3183139	SNX27	sorting nexin family member 27
979.142	0.5107538	PGBD5	piggyBac transposable element derived 5
9.778.415	-0.1980403	STS	steroid sulfatase (microsomal), isozyme S
9.776.702	-0.1646545	OR1F1	olfactory receptor family 1 subfamily F member
9.770.278	-0.1497026	SCNN1D	sodium channel epithelial 1 delta subunit
9.768.742	-0.1840569	PRPH	peripherin
9.767.593	-0.2213398	EML3	echinoderm microtubule associated protein lik
9.766.618	-0.1917631	EPN1	epsin 1
976.422	-0.5814455	CFHR2	complement factor H related 2
9.751.799	-0.125098	SLC16A5	solute carrier family 16 member 5
9.748.506	0.3718971	RHOBTB3	Rho related BTB domain containing 3
9.744.633	0.2069177	APBA3	amyloid beta precursor protein binding family A
9.740.281	-0.1405347	SOX15	SRY-box 15
9.733.087	0.1267633	MED20	mediator complex subunit 20
973.155	-0.1443081	SLC12A4	solute carrier family 12 member 4
9.725.909	-0.1022073	ICA1	islet cell autoantigen 1
971.891	-0.5357365	PROM1	prominin 1
9.717.347	-0.3045268		
9.717.292	-0.1106652	SPINK2	serine peptidase inhibitor, Kazal type 2
9.716.855	-0.1147269	CORO2B	coronin 2B
9.716.342	-0.411969	NPIPB11///	nuclear pore complex interacting protein family
9.716.178	0.3565035	AHNAK	AHNAK nucleoprotein

9.706.628	0.3356745	HNRNPD	heterogeneous nuclear ribonucleoprotein D
9.698.384	0.3724749	TAF7	TATA-box binding protein associated factor 7
9.696.724	0.1852112	MIER2	MIER family member 2
9.694.699	-0.1124674	CGGBP1	CGG triplet repeat binding protein 1
9.692.463	-0.3572099	SLC35A3	solute carrier family 35 member A3
9.689.111	0.4021904	CTNND2	catenin delta 2
968.882	0.5537929	MID1	midline 1
9.688.174	0.1712251	MYB	MYB proto-oncogene, transcription factor
9.680.341	0.3028064	SARS	seryl-tRNA synthetase
9.676.423	-0.1345343	FAM153A	family with sequence similarity 153 member A
9.675.288	-0.1496128	ACR	acrosin
9.674.795	-0.1620702	LOC645226	similar to melanoma antigen family C, 3 isoforn
9.673.819	-0.3007337	SNRNP70	small nuclear ribonucleoprotein U1 subunit 70
9.671.864	-0.2218331	DAPP1	dual adaptor of phosphotyrosine and 3-phosph
9.667.261	0.1918417	ZNF710	zinc finger protein 710
9.660.267	-0.1219727		
965.699	0.1905897	TDRKH	tudor and KH domain containing
9.652.749	0.5440186	SMAD5	SMAD family member 5
9.651.634	0.2604616	IDH3B	isocitrate dehydrogenase 3 (NAD(+)) beta
9.647.112	0.1909865	ALG13	ALG13, UDP-N-acetylglucosaminyltransferase
9.640.189	-0.1604888	ATP12A	ATPase H+/K+ transporting non-gastric alpha2 s
9.636.086	-0.1256803	CHRNA4	cholinergic receptor nicotinic alpha 4 subunit
963.544	-0.3004532	LIN7C	lin-7 homolog C, crumbs cell polarity complex c
9.632.251	0.1957906	ZNF142	zinc finger protein 142
9.629.848	-0.1541151	ELAC2	elaC ribonuclease Z 2
9.623.386	-0.1561522	CAMP	cathelicidin antimicrobial peptide
9.619.679	0.2570761	ZNF32	zinc finger protein 32
9.619.255	0.3407879	NACA3P///I	NACA family member 3 pseudogene///nascent
9.615.724	-0.1951715	PTPN7	protein tyrosine phosphatase, non-receptor typ
961.449	-0.230358	LIG4	DNA ligase 4
9.612.063	0.4041241	LEPROTL1	leptin receptor overlapping transcript-like 1
9.608.025	0.3469964	TCEAL1	transcription elongation factor A like 1
960.537	-0.1124441	TPT1	tumor protein, translationally-controlled 1
9.589.246	0.3984235	TMEM5	transmembrane protein 5
9.583.945	0.3618839	DDHD2	DDHD domain containing 2
9.581.147	-0.1364393	TIMM17A	translocase of inner mitochondrial membrane 1
9.579.295	0.2946069	EIF3G	eukaryotic translation initiation factor 3 subunit
9.577.869	-0.3553862	LAMB2	laminin subunit beta 2
9.574.498	0.2699715	NUP210	nucleoporin 210
956.975	0.1452694	ZNF8	zinc finger protein 8
9.568.982	0.423083	ARPC4	actin related protein 2/3 complex subunit 4
9.565.352	-0.2206549	SPHK2	sphingosine kinase 2
956.296	-0.2742482	IGFBP7	insulin like growth factor binding protein 7
9.561.433	-0.1117595		

9.549.036	0.3940788	KDM6A	lysine demethylase 6A
9.547.676	0.4922375	FBLN1	fibulin 1
9.547.147	-0.1771089	DSC3	desmocollin 3
9.541.878	-0.1090401	CD300A	CD300a molecule
9.541.699	-0.359696	CDH1	cadherin 1
9.541.043	-0.2105651	POU5F1P4,	POU class 5 homeobox 1 pseudogene 4///POU
9.532.864	0.4562588	MYO6	myosin VI
9.530.973	0.2165408	RAB3GAP1	RAB3 GTPase activating protein catalytic subun
9.527.448	0.289666	NDUFB5	NADH:ubiquinone oxidoreductase subunit B5
9.526.556	-0.6888631	INSIG1	insulin induced gene 1
952.277	-0.3152548	GHITM	growth hormone inducible transmembrane prot
9.518.178	-0.1093072	PRO1621	PRO1621 protein
9.515.542	0.2892457	RAD17	RAD17 checkpoint clamp loader component
9.512.668	0.6258118	GULP1	GULP, engulfment adaptor PTB domain contain
9.510.335	-0.2584666	C2orf42	chromosome 2 open reading frame 42
9.502.339	0.2592267	SLC25A14	solute carrier family 25 member 14
9.501.142	0.2495881	SF3B3	splicing factor 3b subunit 3
9.497.567	-0.2385731	XPC	XPC complex subunit, DNA damage recognitior
9.494.403	0.3903971	SLAMF8	SLAM family member 8
9.494.192	0.2469789	CYB5R3	cytochrome b5 reductase 3
9.491.397	-0.3737799	TTC19	tetratricopeptide repeat domain 19
9.491.116	-0.1692145	ZER1	zyg-11 related cell cycle regulator
9.489.877	0.1874627	ZKSCAN4	zinc finger with KRAB and SCAN domains 4
9.484.386	-0.3395043	ALDH1A3	aldehyde dehydrogenase 1 family member A3
9.480.406	0.4300212	TNFAIP3	TNF alpha induced protein 3
947.619	-0.1540749	MZF1	myeloid zinc finger 1
9.465.738	0.3515611	EIF3CL///EI	eukaryotic translation initiation factor 3 subunit
9.463.156	0.3203183	PRPS1	phosphoribosyl pyrophosphate synthetase 1
946.181	0.1614693	KCTD2	potassium channel tetramerization domain cor
9.460.408	-0.4006886	GMFG	glia maturation factor gamma
9.459.963	-0.1052966	C3orf36	chromosome 3 open reading frame 36
9.459.794	0.3145253	TMEM161A	transmembrane protein 161A
9.453.609	-0.1412695	POM121L9	POM121 transmembrane nucleoporin like 9, ps
9.452.677	-0.1187452	LOC73118:	similar to beta-1,4-mannosyltransferase///aspa
9.450.873	-0.1692763	KANK2	KN motif and ankyrin repeat domains 2
9.450.855	-0.3770846	PROSC	proline synthetase cotranscribed homolog (bac
9.450.194	-0.1650018	CYP4F8	cytochrome P450 family 4 subfamily F member
9.448.184	0.2649589	UBXN4	UBX domain protein 4
9.430.025	0.348239	MGAT2	mannosyl (alpha-1,6-)-glycoprotein beta-1,2-N
9.429.559	-0.1296319	NPY5R	neuropeptide Y receptor Y5
9.411.824	-0.73771	TNFSF14	tumor necrosis factor superfamily member 14
9.410.019	-0.1689266	BCAP29	B-cell receptor-associated protein 29
9.409.907	-0.4878859	RARRES2	retinoic acid receptor responder 2
9.407.559	0.1906009	ACTR3B	ARP3 actin related protein 3 homolog B

9.402.902	-0.1375548	APC2	APC2, WNT signaling pathway regulator
940.252	0.2300295	TBP	TATA-box binding protein
939.798	0.318389	TMED10	transmembrane p24 trafficking protein 10
9.396.912	-0.1553628	PKNOX2	PBX/knotted 1 homeobox 2
9.366.569	0.3281318	ELF4	E74 like ETS transcription factor 4
9.358.812	-0.1434482	TSPY1	testis specific protein, Y-linked 1
9.356.997	-0.1747551	CNOT4	CCR4-NOT transcription complex subunit 4
9.354.041	-0.1126454	PIWIL2	piwi like RNA-mediated gene silencing 2
935.321	0.2334848	STAU1	staufen double-stranded RNA binding protein 1
9.330.616	0.4966872	GPR137B	G protein-coupled receptor 137B
9.329.525	-0.1424299		
9.328.872	0.4849351	SLC25A24	solute carrier family 25 member 24
9.324.589	-0.1544724	NPTXR	neuronal pentraxin receptor
932.315	-0.1375685	HECW1	HECT, C2 and WW domain containing E3 ubiqu
9.316.627	0.2390646	ZFYVE16	zinc finger FYVE-type containing 16
9.314.716	-0.187441	FHL1	four and a half LIM domains 1
9.314.274	-0.161886	PTOV1-AS2	PTOV1 antisense RNA 2
9.308.844	-0.1411062	NECTIN1	nectin cell adhesion molecule 1
930.777	-0.3219281	NUCB1	nucleobindin 1
9.307.136	0.2870248	TRIT1	tRNA isopentenyltransferase 1
9.303.498	-0.1121641	KRT2	keratin 2
9.302.213	-0.1507776	PPARA	peroxisome proliferator activated receptor alph
9.299.369	-0.1355033	RBM///RBM	similar to RNA binding motif protein, Y chromos
9.297.403	-0.2561267		
9.293.248	0.2522508	RAP1B	RAP1B, member of RAS oncogene family
9.293.021	0.293256	WDR41	WD repeat domain 41
9.291.569	-0.1466273	CRX	cone-rod homeobox
9.288.905	-0.1730939	NXPE4	neurexophilin and PC-esterase domain family r
928.187	-0.1515803	LY6G6C	lymphocyte antigen 6 complex, locus G6C
927.714	0.2938239	MED26	mediator complex subunit 26
9.276.612	-0.2094544	HBD	hemoglobin subunit delta
9.274.927	-0.1290567	SPAG11B	sperm associated antigen 11B
9.269.953	-0.1533715	MMP17	matrix metallopeptidase 17
9.268.606	-0.1576426		
9.263.934	0.4064134	CBX6	chromobox 6
9.263.216	-0.2992132	CD40	CD40 molecule
925.743	0.1725865	SLC39A9	solute carrier family 39 member 9
9.251.662	-0.3666159	BNIP3L	BCL2 interacting protein 3 like
9.250.782	0.3370973	CHMP5	charged multivesicular body protein 5
9.250.504	0.1982947		
9.244.265	-0.2113551	RARB	retinoic acid receptor beta
9.240.147	0.2389146	LOC72931(	POM121 membrane glycoprotein pseudogene/
9.235.816	-0.2663125	AES	amino-terminal enhancer of split
9.235.278	-0.1946196	LY6G6D	lymphocyte antigen 6 complex, locus G6D

9.234.903	-0.1577285	GRIA3	glutamate ionotropic receptor AMPA type subur
9.233.808	0.2385856	COA3	cytochrome c oxidase assembly factor 3
9.233.325	0.2649028	ARCN1	archain 1
9.231.261	-0.1246176	GLI2	GLI family zinc finger 2
9.226.928	-0.5698742	TCEA2	transcription elongation factor A2
9.224.326	-0.1731737		
9.219.641	-0.115602		
9.214.679	-0.1409913	TRIM62	tripartite motif containing 62
9.213.912	-0.1521214	CCR2	C-C motif chemokine receptor 2
9.212.096	0.4836138	PYCARD	PYD and CARD domain containing
9.209.179	-0.2006158	PRDM14	PR/SET domain 14
9.208.132	0.3030764	TPTE///PCN	transmembrane phosphatase with tensin homo
9.200.552	-0.2888368	CLIP3	CAP-Gly domain containing linker protein 3
9.182.375	0.4348544	WARS	tryptophanyl-tRNA synthetase
9.175.807	0.2969686	CKMT1A///(	creatine kinase, mitochondrial 1A///creatine kir
9.166.505	-0.1681196	HCG26	HLA complex group 26 (non-protein coding)
9.162.612	0.2099004	GPALPP1	GPALPP motifs containing 1
9.158.417	-0.3284878	PPP2CA	protein phosphatase 2 catalytic subunit alpha
9.141.674	-0.1362351	KCNB2	potassium voltage-gated channel subfamily B r
9.141.061	-0.1306845	P2RX2	purinergic receptor P2X 2
9.138.801	-0.1788694	ZSWIM8	zinc finger SWIM-type containing 8
9.137.485	0.3312289	HIST1H4J//	histone cluster 1, H4j///histone cluster 1, H4k
9.134.937	0.1307975	LOC39099	ribosomal protein L10 pseudogene
9.129.836	0.6047323	FMO1	flavin containing monooxygenase 1
9.125.464	0.292562	CSNK2A3//	casein kinase 2 alpha 3///casein kinase 2 alpha
9.124.664	0.1190266	FGFR2	fibroblast growth factor receptor 2
911.188	0.2789779	FECH	ferrochelatase
9.109.163	-0.1303677	SCN2A	sodium voltage-gated channel alpha subunit 2
9.108.058	-0.1815812	CRMP1	collapsin response mediator protein 1
910.736	-0.1673249	SPDEF	SAM pointed domain containing ETS transcripti
9.101.712	-0.2554131	SEMA6A	semaphorin 6A
9.100.755	-0.2181345	POLR2C	RNA polymerase II subunit C
9.094.647	-0.1408881	PSG6	pregnancy specific beta-1-glycoprotein 6
9.090.815	0.2608402	AGO1	argonaute 1, RISC catalytic component
9.090.637	-0.3175341	OASL	2'-5'-oligoadenylate synthetase like
9.090.558	-0.1549151	RPH3A	rabphilin 3A
9.086.902	0.462741	NR5A2	nuclear receptor subfamily 5 group A member 2
908.012	0.3445564	WDR3	WD repeat domain 3
9.072.739	-0.2797305	GLT8D1	glycosyltransferase 8 domain containing 1
9.064.049	-0.1689507	LYL1	LYL1, basic helix-loop-helix family member
9.063.985	-0.2356729	CLEC11A	C-type lectin domain family 11 member A
9.062.616	-0.2583965	STARD13	StAR related lipid transfer domain containing 13
9.059.879	-0.2969561	THOP1	thimet oligopeptidase 1
9.049.957	-0.1634134	SP3P	Sp3 transcription factor pseudogene

9.045.573	0.1699752	PLEKHA8//,	pleckstrin homology domain containing A8///pl
9.044.386	0.2088475	PAK4	p21 (RAC1) activated kinase 4
904.319	0.286814	PLAU	plasminogen activator, urokinase
9.042.403	-0.1560543	GPR27	G protein-coupled receptor 27
9.037.758	0.4025673	CHKA	choline kinase alpha
9.034.749	-0.2388842	TEX11	testis expressed 11
9.030.844	-0.1387773	LDLR	low density lipoprotein receptor
9.029.403	-0.3144667	UBL3	ubiquitin like 3
9.027.397	-0.139289	TAS2R14	taste 2 receptor member 14
9.025.632	-0.1625412	ZNF440	zinc finger protein 440
9.024.876	-0.1515219	VRTN	vertebrae development associated
9.019.703	0.4177948	PDCD4	programmed cell death 4 (neoplastic transform
901.409	0.4810555	SELT	selenoprotein T
9.009.452	0.2290855	MED13	mediator complex subunit 13
8.997.485	0.2722015	RYK	receptor-like tyrosine kinase
8.994.964	0.2805936	RPL22	ribosomal protein L22
8.994.827	-0.3250373	HMGN3	high mobility group nucleosomal binding domai
8.994.358	0.2993208	RPL13A	ribosomal protein L13a
8.991.993	-0.3134617	RAI2	retinoic acid induced 2
8.989.673	-0.3648667	CRYZ	crystallin zeta
8.980.656	0.555924	CXCL11	C-X-C motif chemokine ligand 11
8.975.593	0.242487	KCTD5	potassium channel tetramerization domain cor
8.974.128	-0.1102612	MYCN	v-myc avian myelocytomatosis viral oncogene r
8.973.319	0.2476408	SFI1	SFI1 centrin binding protein
8.972.943	0.607791	PGC	progastricsin
8.972.165	-0.3344931	GGTLC1	gamma-glutamyltransferase light chain 1
8.969.431	0.3902144	B4GALT5	beta-1,4-galactosyltransferase 5
8.966.747	0.2625332	FAM206A	family with sequence similarity 206 member A
8.964.229	-0.2024107	MAGI1	membrane associated guanylate kinase, WW a
8.963.561	-0.2939792	ROGDI	rogdi homolog
8.959.965	0.2852892	VCP	valosin containing protein
8.956.424	-0.1968623	MERTK	MER proto-oncogene, tyrosine kinase
8.954.673	0.4007356	CXCL11	C-X-C motif chemokine ligand 11
8.952.826	-0.4691135	CTBP2	C-terminal binding protein 2
8.952.333	-0.156311	SYDE1	synapse defective Rho GTPase homolog 1
8.950.741	-0.1421075	KIAA0485	uncharacterized LOC57235
8.948.732	-0.5071899	HLA-DRB1	major histocompatibility complex, class II, DR b
8.948.206	0.2255952	MYO5A	myosin VA
8.947.752	-0.1662266	NPAS1	neuronal PAS domain protein 1
8.947.536	0.7580572	ZIC1	Zic family member 1
8.939.432	0.5194256	ANXA5	annexin A5
8.938.664	0.2306119	TAF1B	TATA-box binding protein associated factor, RN.
893.686	0.2217131	SUPT20H	SPT20 homolog, SAGA complex component
8.935.794	-0.1491884	TBKBP1	TBK1 binding protein 1

8.931.679	-0.1552645	ADAM11	ADAM metallopeptidase domain 11
893.044	-0.2231723	C16orf58	chromosome 16 open reading frame 58
8.928.674	-0.3328581	ZNF451	zinc finger protein 451
8.928.122	-0.5656276	IL1RN	interleukin 1 receptor antagonist
8.913.807	0.1737177	COQ7	coenzyme Q7, hydroxylase
8.913.094	-0.1588195	MAS1	MAS1 proto-oncogene, G protein-coupled recep
8.912.477	-0.2473559	NRG1	neuregulin 1
8.912.307	0.2953224	ANKRD6	ankyrin repeat domain 6
8.908.831	0.2309876	ST20	suppressor of tumorigenicity 20
89.079	-0.3658316	GAREM1	GRB2 associated regulator of MAPK1 subtype 1
8.906.243	-0.2201146	FBXO40	F-box protein 40
8.902.465	-0.1320482		
8.894.174	-0.1703467	KDM4C	lysine demethylase 4C
8.892.611	-0.1890763	MBTD1	mbt domain containing 1
8.887.352	0.2127393	MUTYH	mutY DNA glycosylase
8.882.953	-0.21039	HMOX2	heme oxygenase 2
8.882.051	0.2958876	FAM13B	family with sequence similarity 13 member B
8.880.023	-0.1921682	RPL18	ribosomal protein L18
8.878.657	-0.1158719	HTR1F	5-hydroxytryptamine receptor 1F
8.877.713	-0.2432382	MGC12488	uncharacterized protein MGC12488
886.577	-0.1205991	NDUFAF5	NADH:ubiquinone oxidoreductase complex ass
8.864.461	-0.1280622	SH3TC2	SH3 domain and tetratricopeptide repeats 2
8.863.239	-0.2508986	GPR153	G protein-coupled receptor 153
8.859.717	-0.3449396	GALNT3	polypeptide N-acetylgalactosaminyltransferase
8.854.874	0.218683	POFUT1	protein O-fucosyltransferase 1
8.852.111	-0.1266191	AGER	advanced glycosylation end-product specific re
884.831	-0.1451822	BMP8A	bone morphogenetic protein 8a
8.847.869	-0.541009	TNFSF10	tumor necrosis factor superfamily member 10
8.843.964	-0.5474158	SERPINB9	serpin family B member 9
8.843.956	-0.3528515	SYNPO	synaptopodin
8.837.252	0.380489	UBE2G1	ubiquitin conjugating enzyme E2 G1
8.830.783	0.2106637	UEVLD	UEV and lactate/malate dehyrogenase domains
8.819.027	-0.1214493		
8.814.709	0.4051535	AP1M2	adaptor related protein complex 1 mu 2 subunit
881.161	0.1515919	POLR3G	RNA polymerase III subunit G
8.808.707	-0.1542826	NRXN1	neurexin 1
8.808.582	0.21796	GTF2I	general transcription factor IIi
8.806.345	0.1936866	CELSR2	cadherin EGF LAG seven-pass G-type receptor 2
8.805.125	-0.1497226	TCL6	T-cell leukemia/lymphoma 6 (non-protein codir
8.796.921	-0.1969904	GPR137	G protein-coupled receptor 137
8.794.947	0.5021312	WNT5A	Wnt family member 5A
879.214	-0.1908064	MAPK8IP3	mitogen-activated protein kinase 8 interacting p
8.788.233	-0.1585987	TEK	TEK receptor tyrosine kinase
8.787.221	-0.2339525	TNNT3	troponin T3, fast skeletal type

8.786.971	-0.1798711	TRERF1	transcriptional regulating factor 1
8.786.634	-0.1662952	PHLDB1	pleckstrin homology like domain family B meml
8.777.418	-0.2239649	GAS8	growth arrest specific 8
8.764.318	0.234301	CYTH1	cytohesin 1
8.762.621	-0.2568635	FGR	FGR proto-oncogene, Src family tyrosine kinase
8.761.372	-0.2598294		
8.761.253	0.2725244	CTBP1	C-terminal binding protein 1
8.759.265	-0.1433838	RIN3	Ras and Rab interactor 3
8.757.355	0.2852296	ZNF124	zinc finger protein 124
8.755.968	-0.1801006	NCOR2	nuclear receptor corepressor 2
8.754.182	0.2287565	AP3B1	adaptor related protein complex 3 beta 1 subur
8.750.741	0.2375413	MLST8	MTOR associated protein, LST8 homolog
8.748.198	-0.1431409	HIST1H4I	histone cluster 1, H4i
8.739.176	-0.1684293	LOC73090!	uncharacterized LOC730909
8.739.057	-0.2621455	CFAP69	cilia and flagella associated protein 69
8.735.932	0.1377484	SUPT7L	SPT7-like STAGA complex gamma subunit
8.718.109	-0.2788496	ATP5D	ATP synthase, H+ transporting, mitochondrial F
8.716.028	-0.1711948	CAPN2	calpain 2
8.713.689	-0.3060381	ALG13	ALG13, UDP-N-acetylglucosaminyltransferase
8.712.361	-0.1172304	DKFZP434(	DKFZP434C153 protein
8.702.651	0.2057745	NUP98	nucleoporin 98
869.491	0.3352948	GTF2E2	general transcription factor IIE subunit 2
8.692.207	-0.1165975	TMEM121	transmembrane protein 121
8.691.592	0.2065589	CHMP6	charged multivesicular body protein 6
8.690.652	-0.1230757	IKZF1	IKAROS family zinc finger 1
8.688.907	0.1996504	PTPN1	protein tyrosine phosphatase, non-receptor typ
8.678.778	-0.2599881	SOCS3	suppressor of cytokine signaling 3
8.672.575	-0.2103461	HSPB2	heat shock protein family B (small) member 2
8.671.661	-0.1999658	MEGF6	multiple EGF like domains 6
8.669.007	0.2936212	SMA5	glucuronidase beta pseudogene
8.668.962	-0.1376888	COL4A3	collagen type IV alpha 3 chain
8.666.311	-0.1053876	NOP14	NOP14 nucleolar protein
8.663.415	-0.4226714	PCDHGA1/	protocadherin gamma subfamily A, 1///protoca
8.660.481	-0.3708932	HDGFRP2	hepatoma-derived growth factor-related protein
8.653.549	-0.3840145	HNMT	histamine N-methyltransferase
8.653.424	0.1666087	MFSD13A	major facilitator superfamily domain containing
8.652.551	-0.1857085	LPAR1	lysophosphatidic acid receptor 1
8.647.483	-0.1456991	WAS	Wiskott-Aldrich syndrome
8.646.917	-0.3681379	PRKAR2B	protein kinase cAMP-dependent type II regulato
8.646.647	-0.2410064	APOBEC3G	apolipoprotein B mRNA editing enzyme catalyti
8.645.176	-0.1316716	HOXA2	homeobox A2
8.643.929	-0.1395505	IGFBP5	insulin like growth factor binding protein 5
8.633.492	-0.2549815	HYI	hydroxypyruvate isomerase (putative)
8.633.439	0.2299816	THADA	THADA, armadillo repeat containing

8.629.481	0.4590004	SCGN	secretagogin, EF-hand calcium binding protein
8.627.017	-0.1582943	CXCL8	C-X-C motif chemokine ligand 8
8.626.835	-0.1359297	DYRK1B	dual specificity tyrosine phosphorylation regula
8.626.359	0.2390852	NDUFA3	NADH:ubiquinone oxidoreductase subunit A3
8.625.831	-0.1383167	EML3	echinoderm microtubule associated protein lik
8.617.417	-0.1734107	TBXAS1	thromboxane A synthase 1
8.616.888	0.1872635	WDR48	WD repeat domain 48
8.611.428	-0.1352673	KIR3DL1	killer cell immunoglobulin like receptor, three Ig
8.608.617	0.243261	ABCD1	ATP binding cassette subfamily D member 1
8.603.102	-0.1236191	DICER1	dicer 1, ribonuclease III
8.602.302	0.2669388	TERF2	telomeric repeat binding factor 2
8.602.144	0.3460799	NACC2	NACC family member 2
8.599.408	0.2327312	VIPAS39	VPS33B interacting protein, apical-basolateral
8.597.136	0.4079568	FBXW2	F-box and WD repeat domain containing 2
8.596.878	0.1894921	ZCCHC4	zinc finger CCHC-type containing 4
8.589.945	0.261553	RGS14	regulator of G-protein signaling 14
8.581.938	0.3246286	GRINA	glutamate ionotropic receptor NMDA type subu
858.188	0.1458782	MDM1	Mdm1 nuclear protein
8.579.501	0.6438372	UCHL1	ubiquitin C-terminal hydrolase L1
857.845	0.3746032	HEXA	hexosaminidase subunit alpha
8.578.441	-0.1363528	PDYN	prodynorphin
857.766	0.7330271	BICC1	BicC family RNA binding protein 1
8.571.717	-0.1387761	GGTLC1	gamma-glutamyltransferase light chain 1
85.641	-0.4353014	DUSP10	dual specificity phosphatase 10
8.561.202	-0.1879371	BTBD9	BTB domain containing 9
8.560.055	-0.1584178	TSC2	tuberous sclerosis 2
8.559.558	-0.2167103	MMP16	matrix metallopeptidase 16
8.556.473	-0.2643622	FKBP2	FK506 binding protein 2
8.551.528	-0.4384837	RSRP1	arginine and serine rich protein 1
8.548.256	-0.1334728	TRHR	thyrotropin releasing hormone receptor
8.542.638	0.1903974	PHF20	PHD finger protein 20
8.538.023	-0.3396288	PRKCZ	protein kinase C zeta
8.531.637	0.3339153	FUS	FUS RNA binding protein
8.523.529	0.2289058	CYFIP1	cytoplasmic FMR1 interacting protein 1
8.518.389	0.167822	SPTLC2	serine palmitoyltransferase long chain base sul
8.516.511	-0.1861002	RGS11	regulator of G-protein signaling 11
8.507.389	-0.1686863	ARC	activity regulated cytoskeleton associated prote
850.601	0.2238221	MAGEF1	MAGE family member F1
8.505.387	-0.1548492	OR7A5	olfactory receptor family 7 subfamily A member
8.505.306	-0.1448245	NPPA	natriuretic peptide A
8.503.326	0.2429748	ACOT8	acyl-CoA thioesterase 8
8.497	-0.1522836	H6PD	hexose-6-phosphate dehydrogenase/glucose 1
8.496.656	-0.1006366	CHRNA3	cholinergic receptor nicotinic alpha 3 subunit
8.490.163	0.1943545	RAB35	RAB35, member RAS oncogene family

8.488.466	-0.1237448	LY9	lymphocyte antigen 9
848.675	-0.1205924		
8.485.491	0.3895225	TMX4	thioredoxin related transmembrane protein 4
8.482.105	0.4870539	SMARCA1	SWI/SNF related, matrix associated, actin depe
8.477.874	0.3215693	BIN1	bridging integrator 1
8.477.082	0.2249206	DDRGK1	DDRGK domain containing 1
8.476.155	-0.1631331	GZMM	granzyme M
8.474.851	0.3091164	NASP	nuclear autoantigenic sperm protein
8.474.525	-0.1588087	CTBP2	C-terminal binding protein 2
8.471.843	-0.2788601	NFIB	nuclear factor I B
8.467.432	0.285363	TRIM8	tripartite motif containing 8
8.465.144	-0.3696732	GSE1	Gse1 coiled-coil protein
8.463.662	0.200088	UTRN	utrophin
8.450.967	0.2253885	DHX34	DEAH-box helicase 34
844.999	-0.2413085	AGBL5///GI	ATP/GTP binding protein like 5///glutamate ionc
8.442.608	0.2392973	BCAM	basal cell adhesion molecule (Lutheran blood g
8.440.255	-0.1449566	KCNMB2	potassium calcium-activated channel subfamil
8.437.746	0.2553098	MRFAP1L1	Morf4 family associated protein 1 like 1
8.434.873	-0.2798521	CEBPB	CCAAT/enhancer binding protein beta
8.425.599	-0.1472942	FASLG	Fas ligand
8.423.464	-0.1884799	TAF9B	TATA-box binding protein associated factor 9b
842.207	0.3785367		
8.421.766	0.1591645	BRF2	BRF2, RNA polymerase III transcription initiatio
8.420.822	-0.272764	MZF1	myeloid zinc finger 1
8.411.535	-0.1603558	CFTR	cystic fibrosis transmembrane conductance re
8.409.122	-0.2913812	DYNC1LI2	dynein cytoplasmic 1 light intermediate chain 2
8.407.072	0.1679722	ATXN3	ataxin 3
8.404.351	-0.2455761	UBA7	ubiquitin like modifier activating enzyme 7
8.400.216	0.2933208	LIMS1	LIM zinc finger domain containing 1
8.399.843	0.2832176	ARHGEF5	Rho guanine nucleotide exchange factor 5
8.398.287	-0.9027114	PTGDS	prostaglandin D2 synthase
8.393.668	-0.1289396	GPATCH2L	G-patch domain containing 2 like
8.390.362	0.2807098	RPL29P5	ribosomal protein L29 pseudogene 5
8.388.783	-0.147993		
8.386.045	-0.4436424		
8.372.851	0.4019984	AASDHPPT	aminoadipate-semialdehyde dehydrogenase-p
8.367.699	0.2065666	MPZL1	myelin protein zero like 1
8.366.194	-0.1802964	SLC4A4	solute carrier family 4 member 4
8.359.423	-0.2709136	VTCN1	V-set domain containing T cell activation inhibit
8.351.937	0.3697495	PECAM1	platelet and endothelial cell adhesion molecule
8.351.604	0.3720692	IPO5	importin 5
8.351.078	-0.2118852	SH3BP2	SH3 domain binding protein 2
834.867	0.2841813	MAP3K4	mitogen-activated protein kinase kinase kinase
8.346.428	-0.1288027	DCT	dopachrome tautomerase

8.340.714	0.2349496	SMU1	DNA replication regulator and spliceosomal fac
8.339.821	-0.3317221	SSFA2	sperm specific antigen 2
8.335.635	-0.1215359	ADIPOQ	adiponectin, C1Q and collagen domain contain
8.330.062	-0.1453476	MAST2	microtubule associated serine/threonine kinas
832.673	-0.3575358	CLCN3	chloride voltage-gated channel 3
8.326.677	-0.1613286		
8.324.018	0.2738107	PSMB7	proteasome subunit beta 7
8.321.235	-0.124776	PBOV1	prostate and breast cancer overexpressed 1
8.318.848	-0.362973	STBD1	starch binding domain 1
8.318.453	-0.1803894	NCR1	natural cytotoxicity triggering receptor 1
8.317.144	-0.1729302	NLGN3	neuroligin 3
8.316.903	-0.1456476	ODF1	outer dense fiber of sperm tails 1
8.311.462	-0.1563533	SLC22A14	solute carrier family 22 member 14
8.310.595	-0.1740618	CA7	carbonic anhydrase 7
8.307.125	0.2276624	FAM57A	family with sequence similarity 57 member A
8.306.625	0.2724928	IDH3G	isocitrate dehydrogenase 3 (NAD(+)) gamma
8.306.291	-0.1323609	KDM4B	lysine demethylase 4B
8.303.769	-0.1954019	STEAP4	STEAP4 metalloreductase
8.302.179	0.2775141	GALNT11	polypeptide N-acetylgalactosaminyltransferase
8.296.889	-0.1667441	UBXN1	UBX domain protein 1
8.295.049	0.4204297	WIPI1	WD repeat domain, phosphoinositide interactir
8.294.553	-0.2776772	NACC1	nucleus accumbens associated 1
8.292.446	-0.1225459	MED14	mediator complex subunit 14
8.290.289	-0.1220481		
8.288.748	-0.1361824	NKAIN1	Na+/K+ transporting ATPase interacting 1
8.282.102	-0.1461596	IGHG1///IG	immunoglobulin heavy constant gamma 1 (G1r
827.633	0.1528522	OTUB1	OTU deubiquitinase, ubiquitin aldehyde binding
8.276.131	-0.1145668	ZMYM5	zinc finger MYM-type containing 5
8.275.742	-0.1068865	SLC4A7	solute carrier family 4 member 7
826.843	-0.1997544	MVK	mevalonate kinase
8.265.111	0.3379963	MAP3K8	mitogen-activated protein kinase kinase kinase
8.261.538	-0.1583197	AKAP6	A-kinase anchoring protein 6
825.706	0.1758899	ORC1	origin recognition complex subunit 1
8.255.292	-0.3108689	NDUFAF1	NADH:ubiquinone oxidoreductase complex ass
8.250.788	-0.2912486	LARP7	La ribonucleoprotein domain family member 7
8.241.845	-0.2136982	MYH11	myosin heavy chain 11
8.239.904	0.3788978	F13A1	coagulation factor XIII A chain
8.239.314	-0.4289073	LAP3	leucine aminopeptidase 3
8.234.386	-0.1491053	HTR5A	5-hydroxytryptamine receptor 5A
8.232.914	-0.6231656	FMO5	flavin containing monooxygenase 5
8.232.432	-0.1902235		
8.232.171	0.3720894	ATRN	attractin
8.229.677	0.2509911	SRC	SRC proto-oncogene, non-receptor tyrosine kin
822.933	-0.1771714	ATG16L1	autophagy related 16 like 1

8.221.913	-0.1710043	L2HGDH	L-2-hydroxyglutarate dehydrogenase
8.219.725	-0.1487986	ZNF155	zinc finger protein 155
8.216.369	0.7314235	SERPINE2	serpin family E member 2
8.215.249	-0.1175892	PSG6	pregnancy specific beta-1-glycoprotein 6
821.291	-0.3514399	CCPG1	cell cycle progression 1
8.207.493	-0.1502705	RFX2	regulatory factor X2
8.205.647	-0.2076572		
820.201	0.5203249	SDC2	syndecan 2
8.200.725	-0.1605547	RNASET2	ribonuclease T2
8.196.626	-0.1167783	PLSCR2	phospholipid scramblase 2
8.193.867	-0.1853435	KCNA5	potassium voltage-gated channel subfamily A n
8.193.317	-0.1420581	CDH15	cadherin 15
8.190.934	-0.3800239	GRAMD3	GRAM domain containing 3
818.932	-0.1336472	LGI1	leucine rich glioma inactivated 1
8.187.663	-0.6145231	APOM	apolipoprotein M
818.564	0.433219	LAD1	ladinin 1
8.181.633	-0.1293725	FASN	fatty acid synthase
8.181.186	-0.1678357	SPRY4	sprouty RTK signaling antagonist 4
8.179.817	0.1592487	ZNF592	zinc finger protein 592
8.178.539	-0.1611661	RUNX1T1	RUNX1 translocation partner 1
8.176.909	0.1684744	MKL1	megakaryoblastic leukemia (translocation) 1
8.176.152	0.2712797	AFDN-AS1	AFDN antisense RNA 1 (head to head)
8.173.059	-0.2850891	ING1	inhibitor of growth family member 1
8.169.996	-0.1525569	KSR1	kinase suppressor of ras 1
8.168.113	-0.1314581	PAX8	paired box 8
8.159.381	-0.4044138	AKAP7	A-kinase anchoring protein 7
8.156.322	-0.1427557	LMAN1L	lectin, mannose binding 1 like
8.154.992	-0.198732	DTX2	deltex E3 ubiquitin ligase 2
8.154.319	0.260511	PSPC1	paraspeckle component 1
8.149.931	-0.3786367	NKTR	natural killer cell triggering receptor
8.148.263	-0.6961225	TAGLN	transgelin
8.139.577	-0.1969783	PLEKHA5	pleckstrin homology domain containing A5
8.133.307	-0.5488	HLA-DRB1	major histocompatibility complex, class II, DR b
8.132.951	-0.1903545		
8.127.906	-0.1059648	KCNJ13	potassium voltage-gated channel subfamily J m
8.127.148	0.2893792	SLC2A1	solute carrier family 2 member 1
8.124.071	0.3147156	SCAMP1	secretory carrier membrane protein 1
8.119.946	0.3130525	CNOT8	CCR4-NOT transcription complex subunit 8
8.118.722	-0.1799695	DISC1	disrupted in schizophrenia 1
8.113.206	-0.2399056	WDR60	WD repeat domain 60
8.108.371	-0.2036976	POM121L7	POM121 transmembrane nucleoporin like 7///p
8.103.549	0.2279062	CCDC88A	coiled-coil domain containing 88A
8.094.988	0.3606681	HIST1H4J//	histone cluster 1, H4j///histone cluster 1, H4k
8.092.341	0.4442929	THSD7A	thrombospondin type 1 domain containing 7A

8.088.092	-0.5653425	IGFBP5	insulin like growth factor binding protein 5
8.086.089	-0.1328028	PKD2L2	polycystin 2 like 2, transient receptor potential [illegible]
8.084.918	0.2982238	AGFG1	ArfGAP with FG repeats 1
8.084.655	0.2660698	CAPN1	calpain 1
8.083.861	0.3651891	CBFB	core-binding factor beta subunit
8.083.497	-0.1331712	IRF5	interferon regulatory factor 5
8.077.982	-0.1304475	LOC22007[illegible]	dedicator of cytokinesis 1 pseudogene
8.072.299	0.2535185	DENND1A	DENN domain containing 1A
8.068.705	-0.1436574	CYP2D6	cytochrome P450 family 2 subfamily D membe[illegible]
8.066.354	-0.4074975	CYTIP	cytohesin 1 interacting protein
8.064.283	0.3709012	SLC25A6	solute carrier family 25 member 6
8.062.887	-0.1142381	PTGIS	prostaglandin I2 (prostacyclin) synthase
8.061.384	-0.1297089	NRG1	neuregulin 1
8.059.106	-0.3954852	PER2	period circadian clock 2
805.478	-0.1483156	PART1	prostate androgen-regulated transcript 1 (non-[illegible]
8.047.669	0.2590424	AARS	alanyl-tRNA synthetase
8.045.251	-0.1619938		
8.041.663	-0.1248281	HMGN5	high mobility group nucleosome binding domai[illegible]
8.038.109	-0.1210155	ACRV1	acrosomal vesicle protein 1
8.031.246	-0.1323676	ENG	endoglin
8.022.462	0.2766543	TTF1	transcription termination factor 1
8.017.327	-0.1435602	TRAT1	T cell receptor associated transmembrane ada[illegible]
8.016.983	-0.6292685	GLUD2	glutamate dehydrogenase 2
8.015.607	-0.1314187	MTSS1L	metastasis suppressor 1 like
8.014.201	0.1783176	PRUNE1	prune exopolyphosphatase
8.013.357	-0.1508503	NIPAL3	NIPA like domain containing 3
8.006.432	0.2810247	C6orf106	chromosome 6 open reading frame 106
7.995.331	-0.1444889	EMP1	epithelial membrane protein 1
7.994.996	-0.1513076	FGF5	fibroblast growth factor 5
7.991.058	0.3586725	RAB5C	RAB5C, member RAS oncogene family
7.987.632	-0.1217883	FGF5	fibroblast growth factor 5
7.987.298	-0.3271794	KIAA0141	KIAA0141
7.984.743	0.234104	METTL21B	methyltransferase like 21B
7.981.893	-0.1331084		
7.981.494	-0.138606	IFNA17	interferon alpha 17
7.979.292	0.212078	ZC3H4	zinc finger CCCH-type containing 4
7.978.579	0.2013688	RELB	RELB proto-oncogene, NF-kB subunit
7.976.979	-0.1771946	TOR4A	torsin family 4 member A
7.971.327	-0.4567351	CTBP2	C-terminal binding protein 2
7.967.328	-0.1310359	ZNF235	zinc finger protein 235
7.967.093	-0.2310881	ELK1	ELK1, ETS transcription factor
7.965.878	0.1935303	ABI2	abl-interactor 2
7.965.854	-0.2020888	CTDSP2	CTD small phosphatase 2
795.586	-0.2797837	NCKAP1L	NCK associated protein 1 like

7.954.438	0.1393807	GRK6	G protein-coupled receptor kinase 6
7.953.244	0.2000181	PLCE1	phospholipase C epsilon 1
7.951.812	-0.1609997	CRYGC	crystallin gamma C
7.948.337	-0.1424428	E2F4	E2F transcription factor 4
7.947.783	-0.2443456	ROM1	retinal outer segment membrane protein 1
7.945.641	0.2267106	COPS7B	COP9 signalosome subunit 7B
7.941.823	0.5828804	DSG2	desmoglein 2
7.941.029	0.4273395	DNAJB9	DnaJ heat shock protein family (Hsp40) membe
7.940.891	-0.2168073	GABARAP	GABA type A receptor-associated protein
7.936.966	-0.1532805		
7.936.875	0.3067593	MED4	mediator complex subunit 4
7.934.796	-0.2981511	SLC7A2	solute carrier family 7 member 2
7.932.727	0.3302999	NUDT21	nudix hydrolase 21
7.927.822	-0.2491773	CALM1	calmodulin 1
7.927.312	0.2988393	ABL1	ABL proto-oncogene 1, non-receptor tyrosine ki
7.924.751	-0.367311	COBL	cordon-bleu WH2 repeat protein
7.922.164	-0.1791911	CCL27	C-C motif chemokine ligand 27
7.921.245	-0.1507947	TRIM29	tripartite motif containing 29
7.918.329	-0.1358206	FTH1	ferritin heavy chain 1
7.918.163	0.2922831		
7.917.304	0.2208725	CDYL	chromodomain Y-like
7.912.425	-0.1844776	EDIL3	EGF like repeats and discoidin domains 3
7.908.865	0.3292278	CADPS2	calcium dependent secretion activator 2
7.907.744	-0.1226531	AGER	advanced glycosylation end-product specific re
7.904.105	0.2419499	TTC28	tetratricopeptide repeat domain 28
7.902.336	0.3659493	PTPN11	protein tyrosine phosphatase, non-receptor typ
790.079	-0.3419427	PLLP	plasmolipin
7.897.402	-0.3361548		
7.895.433	-0.6171881	APOM	apolipoprotein M
7.895.099	0.2295772	CNOT2	CCR4-NOT transcription complex subunit 2
7.894.897	-0.3136426	DHRS3	dehydrogenase/reductase 3
7.881.961	0.1425871	EIF5B	eukaryotic translation initiation factor 5B
78.804	0.2167864	PFDN5	prefoldin subunit 5
7.871.405	-0.3100014	GIPC2	GIPC PDZ domain containing family member 2
7.868.101	-0.1215106	MTMR3	myotubularin related protein 3
7.866.067	-0.3013195	FRMD4A	FERM domain containing 4A
7.865.046	-0.1639768	NPFFR1	neuropeptide FF receptor 1
785.393	-0.2428847	ULK1	unc-51 like autophagy activating kinase 1
7.853.567	-0.3964776	TNFAIP2	TNF alpha induced protein 2
7.850.044	0.1545037	ABI2	abl-interactor 2
7.846.719	-0.2054995	LOC38990(	zinc finger protein 839 pseudogene
7.846.382	-0.1618035	ANKHD1	ankyrin repeat and KH domain containing 1
7.844.374	-0.1652591	AHI1	Abelson helper integration site 1
7.842.894	-0.1237429	MYH1	myosin heavy chain 1

7.840.236	0.2836274	FIP1L1	factor interacting with PAPOLA and CPSF1
7.833.253	-0.3594856	SLC25A13	solute carrier family 25 member 13
7.828.707	0.2660712	MARK3	microtubule affinity regulating kinase 3
7.828.587	-0.1283863	GPR135	G protein-coupled receptor 135
7.828.179	-0.1108103	GPR68	G protein-coupled receptor 68
7.825.401	0.3411858	FCGR2A	Fc fragment of IgG receptor IIa
7.818.576	-0.2477217	RUNX3	runt related transcription factor 3
7.813.198	0.2557072	LRP12	LDL receptor related protein 12
7.812.601	0.3794483	ERO1B	endoplasmic reticulum oxidoreductase 1 beta
7.812.502	-0.7742575	COL3A1	collagen type III alpha 1 chain
7.799.804	-0.4167985	MAOA	monoamine oxidase A
7.797.337	0.3342049	PGLS	6-phosphogluconolactonase
7.795.835	-0.1136436	VPS45	vacuolar protein sorting 45 homolog
7.795.434	-0.6281459	FGFR2	fibroblast growth factor receptor 2
7.794.841	-0.48406	NAV2	neuron navigator 2
779.473	-0.1590542	GUSBP11	glucuronidase, beta pseudogene 11
778.754	0.2595612	NFYC	nuclear transcription factor Y subunit gamma
7.787.122	0.2649488	NFYC	nuclear transcription factor Y subunit gamma
7.781.512	0.2289034	MYH9	myosin, heavy chain 9, non-muscle
7.774.966	-0.1424524	DOHH	deoxyhypusine hydroxylase/monooxygenase
7.770.293	-0.2563422	LRRC17	leucine rich repeat containing 17
7.767.451	-0.166599	NGLY1	N-glycanase 1
7.767.029	0.2152152	CORO2A	coronin 2A
776.449	-0.3005507	IVD	isovaleryl-CoA dehydrogenase
7.760.438	-0.1419609	PCLO	piccolo presynaptic cytomatrix protein
7.758.166	-0.1346793	CXCR6	C-X-C motif chemokine receptor 6
7.758.102	-0.1442015	FGFR2	fibroblast growth factor receptor 2
7.755.667	0.1663026	TOP1	topoisomerase (DNA) I
7.751.656	0.2629595	SRP54	signal recognition particle 54
7.740.439	-0.225313	SRSF8	serine and arginine rich splicing factor 8
7.737.924	0.4867616	TUBA1A	tubulin alpha 1a
7.737.422	0.4459149	DDR1	discoidin domain receptor tyrosine kinase 1
7.736.706	-0.4706342	OASL	2'-5'-oligoadenylate synthetase like
7.734.942	-0.1927712	ZNF385D	zinc finger protein 385D
7.733.089	-0.1430244	CACNA2D1	calcium voltage-gated channel auxiliary subuni
7.726.799	-0.3927854	NPIPB11///	nuclear pore complex interacting protein family
7.722.307	-0.1549275	BMX	BMX non-receptor tyrosine kinase
7.717.508	-0.2257321	HGF	hepatocyte growth factor
7.717.319	-0.1726412	NUMB	NUMB, endocytic adaptor protein
7.716.831	-0.15872	AGAP2	ArfGAP with GTPase domain, ankyrin repeat anc
7.716.205	-0.1397289	ADAM3A	ADAM metallopeptidase domain 3A (pseudoger
7.713.224	-0.1285437	COL13A1	collagen type XIII alpha 1 chain
770.728	-0.1973462	ITGA2B	integrin subunit alpha 2b
7.707.015	0.3200072	POLR2J2	RNA polymerase II subunit J2

7.705.263	-0.1178432	CARD14	caspase recruitment domain family member 14
770.496	0.3482996	CDK2AP2	cyclin dependent kinase 2 associated protein 2
7.704.337	0.0885432	KLHL5	kelch like family member 5
7.701.023	0.2644943	YIPF2	Yip1 domain family member 2
7.692.153	0.4619148	C1QBP	complement C1q binding protein
7.687.795	-0.2746838	MYCBP2	MYC binding protein 2, E3 ubiquitin protein ligas
7.685.738	-0.1916506	SEMA6B	semaphorin 6B
7.681.448	-0.1467029	RGS6	regulator of G-protein signaling 6
767.823	-0.1371125		
7.677.026	-0.1270971	NPHP1	nephrocystin 1
7.677.009	0.3196311	PDIA4	protein disulfide isomerase family A member 4
7.672.734	-0.1949932	GOLGA8G	golgin A8 family member G
766.694	-0.1455127		
7.664.337	0.2962381	APPBP2	amyloid beta precursor protein binding protein
7.661.684	-0.1082517	KIAA1456	KIAA1456
7.661.572	-0.2515993	SYNJ2BP	synaptojanin 2 binding protein
7.661.016	-0.3519019	GUCY1B3	guanylate cyclase 1 soluble subunit beta
7.660.261	-0.1096911	MED31	mediator complex subunit 31
7.654.363	0.2396177	RBM10	RNA binding motif protein 10
7.652.466	-0.1623375	CYP46A1	cytochrome P450 family 46 subfamily A membe
7.644.709	0.330418	CD151	CD151 molecule (Raph blood group)
7.641.352	-0.1087849	AAK1	AP2 associated kinase 1
7.641.224	0.3133282	CRTC3	CREB regulated transcription coactivator 3
7.637.819	0.3180698		
763.592	-0.336344	PPP1R10	protein phosphatase 1 regulatory subunit 10
7.632.004	0.17767	DPH5	diphthamide biosynthesis 5
7.631.064	0.2130062	RAB4B	RAB4B, member RAS oncogene family
7.626.125	-0.1089102	CCDC33	coiled-coil domain containing 33
7.624.634	0.361758	RASSF4	Ras association domain family member 4
7.622.993	-0.1284534	KCNC1	potassium voltage-gated channel subfamily C r
7.621.843	0.2585836	HSPA9	heat shock protein family A (Hsp70) member 9
7.620.721	0.1369291	GM2A	GM2 ganglioside activator
7.611.441	0.2564452	EIF4E2	eukaryotic translation initiation factor 4E family
7.605.292	-0.1266549	TNFSF8	tumor necrosis factor superfamily member 8
7.604.521	-0.1189434	RFPL2	ret finger protein like 2
7.587.971	0.3280491	TRAPPC6A	trafficking protein particle complex 6A
7.587.291	0.2460552	SFSWAP	splicing factor SWAP homolog
7.576.649	0.3864671	PGM3	phosphoglucomutase 3
7.570.745	-0.1540902	TLE4	transducin like enhancer of split 4
7.570.039	0.340814	C14orf1	chromosome 14 open reading frame 1
7.569.285	-0.2692911	UBA7	ubiquitin like modifier activating enzyme 7
7.568.859	0.201189	MAP2K5	mitogen-activated protein kinase kinase 5
7.568.149	-0.2300529	TNFSF11	tumor necrosis factor superfamily member 11
7.564.454	-0.2420952	SPN	sialophorin

7.564.127	0.3503227	USP25	ubiquitin specific peptidase 25
7.563.857	-0.1974372	ZSCAN18	zinc finger and SCAN domain containing 18
7.562.702	-0.1361434	PLCH1	phospholipase C eta 1
7.562.257	0.3070615	MEN1	menin 1
7.560.066	0.2131373	ASAP3	ArfGAP with SH3 domain, ankyrin repeat and PH
7.559.804	-0.2074904	ZRSR2	zinc finger CCCH-type, RNA binding motif and s
7.557.376	-0.3315657	SPATA20	spermatogenesis associated 20
7.556.239	0.2811833	ZNF148	zinc finger protein 148
7.550.502	-0.1181409	IL22	interleukin 22
7.549.384	0.8432582	GAGE2A///(	G antigen 2A///G antigen 12H///G antigen 12E//
7.542.249	0.4046822	EFR3A	EFR3 homolog A
7.537.994	-0.2098635	ESRRG	estrogen related receptor gamma
7.534.862	-0.1355296		
7.533.918	0.4182438	C2orf54	chromosome 2 open reading frame 54
7.533.123	-0.3160379	FAM102A	family with sequence similarity 102 member A
7.529.349	0.2106802	PIN1	peptidylprolyl cis/trans isomerase, NIMA-intera
752.818	-0.5242959	TNFSF10	tumor necrosis factor superfamily member 10
752.601	-0.1751368	CXCL8	C-X-C motif chemokine ligand 8
7.524.941	-0.1724714	ALDH1A2	aldehyde dehydrogenase 1 family member A2
7.524.584	-0.1294483	PDLIM7	PDZ and LIM domain 7
7.521.165	0.1746252	EIF4E2	eukaryotic translation initiation factor 4E family
7.518.176	-0.1578758	ART1	ADP-ribosyltransferase 1
7.510.174	-0.1941647	C21orf2	chromosome 21 open reading frame 2
7.505.773	-0.2820344	INPP5D	inositol polyphosphate-5-phosphatase D
7.499.899	-0.1107521	N4BP2L1	NEDD4 binding protein 2 like 1
7.496.549	-0.1433789	GRM2	glutamate metabotropic receptor 2
7.495.772	-0.1810661	MMP13	matrix metallopeptidase 13
7.493.811	0.1539544	TRMO	tRNA methyltransferase O
7.489.318	0.4488532	HSP90B1	heat shock protein 90 beta family member 1
7.487.062	-0.225431	TRIM10	tripartite motif containing 10
7.483.805	-0.2704915	STAT2	signal transducer and activator of transcription
7.480.177	0.290238	ZC2HC1A	zinc finger C2HC-type containing 1A
747.158	0.1586784	MED27	mediator complex subunit 27
7.470.584	-0.1945848	ECE1	endothelin converting enzyme 1
7.469.585	-0.1490069	KLK6	kallikrein related peptidase 6
746.898	0.2702656	PAQR3	progestin and adipoQ receptor family member 3
7.458.676	0.1836405	PPFIA1	PTPRF interacting protein alpha 1
7.457.296	0.3640329		
7.453.195	-0.1338364	PDLIM4	PDZ and LIM domain 4
7.452.595	-0.2105676	KCND3	potassium voltage-gated channel subfamily D r
7.448.414	-0.1948107	PQLC3	PQ loop repeat containing 3
7.445.504	0.2552811	LSM1	LSM1 homolog, mRNA degradation associated
7.443.588	-0.2760537	BAZ2B	bromodomain adjacent to zinc finger domain 2I
7.438.484	-0.2057999	MIPEP	mitochondrial intermediate peptidase

74.358	0.1769498	TLE3	transducin like enhancer of split 3
7.431.091	-0.2231155	LOC44079:	proline dehydrogenase 1 pseudogene
7.428.076	0.3276286	ATP10D	ATPase phospholipid transporting 10D (putative
7.425.421	-0.1653086	PKNOX2	PBX/knotted 1 homeobox 2
7.420.835	-0.1375103	MAGEB4	MAGE family member B4
7.420.126	-0.137942	HLA-DQB1	major histocompatibility complex, class II, DQ b
7.416.773	0.3941198	CCL18	C-C motif chemokine ligand 18
7.416.392	-0.5978267		
7.415.116	-0.1199921	B3GNTL1	UDP-GlcNAc:betaGal beta-1,3-N-acetylglucosa
7.415.019	0.1728982	ZNF174	zinc finger protein 174
7.410.806	0.3840931	TNPO1	transportin 1
7.408.475	-0.124366	TECTA	tectorin alpha
7.402.766	-0.1807743	IVD	isovaleryl-CoA dehydrogenase
7.399.952	-0.1182638	PAX8	paired box 8
7.397.615	-0.1032852	CTNNA1	catenin alpha 1
7.395.311	-0.1417116	SPN	sialophorin
7.394.755	-0.4632535	PDGFD	platelet derived growth factor D
7.387.468	0.2265662	CALCRL	calcitonin receptor like receptor
7.387.384	-0.2873503	IL6ST	interleukin 6 signal transducer
738.701	-0.1834876	MMRN1	multimerin 1
7.386.511	0.2114011	ILKAP	ILK associated serine/threonine phosphatase
738.418	-0.3413384	MYCBP2	MYC binding protein 2, E3 ubiquitin protein ligas
7.383.384	0.2630745	PUM3	pumilio RNA binding family member 3
7.381.583	-0.133307	SH3BP2	SH3 domain binding protein 2
737.708	-0.1119601	HTR7P1	5-hydroxytryptamine receptor 7 pseudogene 1
7.372.492	-0.1525733		
7.371.319	0.3323587	CEMIP	cell migration inducing hyaluronan binding prot
7.363.884	-0.1950689	LARS2	leucyl-tRNA synthetase 2, mitochondrial
7.363.599	-0.1326485	OR2H2	olfactory receptor family 2 subfamily H membe
7.362.812	-0.1795031	OLFM1	olfactomedin 1
7.356.129	-0.1392722	ICAM5	intercellular adhesion molecule 5
7.354.421	-0.1209941	PILRA	paired immunoglobin like type 2 receptor alpha
735.205	0.3901395	RRAS2	related RAS viral (r-ras) oncogene homolog 2
7.351.694	-0.1417919	CCKAR	cholecystokinin A receptor
734.939	0.2507142	UBE4B	ubiquitination factor E4B
7.348.158	0.2171049	GLTP	glycolipid transfer protein
7.345.487	0.1502926	SCRN3	secernin 3
7.345.091	-0.1132863	CCDC70	coiled-coil domain containing 70
7.343.798	-0.1598349	CDKL5	cyclin dependent kinase like 5
7.343.659	-0.3803723	TRAC	T-cell receptor alpha constant
7.343.399	-0.1576946	NTM	neurotrimin
7.331.869	0.2335778	GNAL	G protein subunit alpha L
7.331.023	-0.1372604	AFF2	AF4/FMR2 family member 2
7.327.979	-0.1148732		

7.317.996	0.2774484	TTC3	tetratricopeptide repeat domain 3
7.310.123	-0.1303511	OSBP2	oxysterol binding protein 2
7.307.672	-0.1443467	RASA3	RAS p21 protein activator 3
730.705	0.2429136	RPL11	ribosomal protein L11
7.304.397	-0.1275324	ITPK1	inositol-tetrakisphosphate 1-kinase
730.062	0.1717547	BRE	brain and reproductive organ-expressed (TNFRS
7.300.547	-0.2494777	HSBP1	heat shock factor binding protein 1
7.300.336	-0.3345792	TRIM38	tripartite motif containing 38
7.297.951	-0.134478	BAIAP3	BAI1 associated protein 3
729.607	-0.1320993	EXTL1	exostosin like glycosyltransferase 1
7.292.255	-0.2960443	LUC7L2	LUC7 like 2, pre-mRNA splicing factor
7.290.839	-0.2695709	SLC25A11	solute carrier family 25 member 11
7.284.974	0.1222603	CINP	cyclin dependent kinase 2 interacting protein
7.282.499	0.3171684	ANKMY2	ankyrin repeat and MYND domain containing 2
7.281.865	0.8254742	GAGE7	G antigen 7
7.278.133	-0.3472664	SLC39A8	solute carrier family 39 member 8
7.276.159	0.2047044	BAD	BCL2 associated agonist of cell death
7.272.421	0.2527498	FAM172A	family with sequence similarity 172 member A
7.272.205	-0.1684422	PDLIM7	PDZ and LIM domain 7
7.270.982	-0.1343826	NSG1	neuron specific gene family member 1
7.270.272	-0.1400881		
7.268.529	-0.1648804	YES1	YES proto-oncogene 1, Src family tyrosine kinas
726.621	-0.4433063	RND1	Rho family GTPase 1
7.264.083	-0.1085732	FAM83E	family with sequence similarity 83 member E
7.262.872	0.4207533	LOC64273:	hypothetical LOC642732
7.260.282	-0.1367242	GFI1	growth factor independent 1 transcriptional rep
7.253.913	-0.1483401	LHX6	LIM homeobox 6
7.253.541	-0.1622919	C16orf70	chromosome 16 open reading frame 70
7.252.328	-0.2884613	SFPQ	splicing factor proline and glutamine rich
7.239.857	-0.1296217	SV2C	synaptic vesicle glycoprotein 2C
7.237.279	0.2949937	DST	dystonin
7.235.585	0.2247659	C10orf95	chromosome 10 open reading frame 95
7.235.404	0.1960686	CYHR1	cysteine and histidine rich 1
723.242	0.3668444	DCAF16	DDB1 and CUL4 associated factor 16
7.231.067	0.3081679	OFD1	OFD1, centriole and centriolar satellite protein
7.219.658	-0.2484384	ST3GAL5	ST3 beta-galactoside alpha-2,3-sialyltransferas
7.219.093	-0.1600457	RTEL1	regulator of telomere elongation helicase 1
721.642	0.375568	PTPN2	protein tyrosine phosphatase, non-receptor typ
7.212.696	-0.1777174	CPVL	carboxypeptidase, vitellogenic like
7.212.593	0.3322321	FRY	FRY microtubule binding protein
7.212.477	-0.1520305	ADARB1	adenosine deaminase, RNA specific B1
7.210.685	-0.189806	KCNQ1DN	KCNQ1 downstream neighbor (non-protein cod
7.209.996	-0.1162917	TAS2R7	taste 2 receptor member 7
7.207.587	-0.1418917	PLXNB1	plexin B1

7.205.188	-0.1495977	RPS6KA4	ribosomal protein S6 kinase A4
7.204.956	-0.1224655	BMP6	bone morphogenetic protein 6
7.200.395	0.3009086	B4GAT1	beta-1,4-glucuronyltransferase 1
7.199.412	-0.1343308	MOG	myelin oligodendrocyte glycoprotein
7.198.662	-0.1157867	SNX13	sorting nexin 13
7.195.585	-0.115318	IKBKB	inhibitor of kappa light polypeptide gene enhan
7.193.512	0.3484679	PRPS2	phosphoribosyl pyrophosphate synthetase 2
7.189.933	-0.2867563	DST	dystonin
7.185.748	-0.1330646	TULP1	tubby like protein 1
7.182.919	-0.2641775	RUNX3	runt related transcription factor 3
7.181.978	0.3847624	DSTN	destrin, actin depolymerizing factor
7.178.692	0.2674503	SNX17	sorting nexin 17
7.176.711	-0.089888	PCDH11X	protocadherin 11 X-linked
7.167.744	0.2007241	PWP1	PWP1 homolog, endonuclein
7.165.997	-0.3794896	TMEM176B	transmembrane protein 176B
7.162.316	0.1706146	SLC25A14	solute carrier family 25 member 14
7.158.187	-0.1532878	RAB36	RAB36, member RAS oncogene family
7.157.328	0.3822877	PPDPF	pancreatic progenitor cell differentiation and pr
7.157.105	-0.1716964	IL21R	interleukin 21 receptor
7.150.503	-0.1803635	CCDC106	coiled-coil domain containing 106
71.483	0.1916986	NUS1P1///I	NUS1 dehydrodolichyl diphosphate synthase su
7.144.025	-0.2752429	FMOD	fibromodulin
7.142.828	0.1977278	TSEN2	tRNA splicing endonuclease subunit 2
7.142.466	-0.107219	HMX1	H6 family homeobox 1
7.138.295	-0.1212376	NEURL1	neuralized E3 ubiquitin protein ligase 1
7.136.457	-0.3268225	ST7	suppression of tumorigenicity 7
7.133.409	0.1312958	LOC64675(	hypothetical LOC646756///HPS1, biogenesis o
7.122.155	-0.1215093	KIR3DL2	killer cell immunoglobulin like receptor, three Ig
7.119.172	-0.1162324	ARL4C	ADP ribosylation factor like GTPase 4C
7.115.068	-0.1402397	MAP3K19	mitogen-activated protein kinase kinase kinase
7.111.907	-0.1795164	LINC01260	long intergenic non-protein coding RNA 1260
7.107.469	0.2809101	HSPBAP1	HSPB1 associated protein 1
7.105.785	0.2547607	FKBP4	FK506 binding protein 4
7.101.643	-0.1066958	LOC64409	similar to ovo-like 2 isoform A
7.094.827	-0.173443	CSF1	colony stimulating factor 1
7.094.497	0.2328004	JRK	Jrk helix-turn-helix protein
7.073.716	-0.1408933		
7.073.572	0.1993359	NCDN	neurochondrin
7.073.041	-0.4127276	CD48	CD48 molecule
7.069.807	0.2527306	UBC	ubiquitin C
7.068.267	0.2199047	PDCD6IPP2	PDCD6IP pseudogene 2
7.062.324	-0.1042859	TTTY2	testis-specific transcript, Y-linked 2 (non-protei
7.061.439	-0.3335379	SSBP3	single stranded DNA binding protein 3
7.059.249	-0.1447564	C6orf15	chromosome 6 open reading frame 15

7.059.011	-0.1267124	KCNJ9	potassium voltage-gated channel subfamily J m
7.050.016	-0.0969397	ATP6AP2	ATPase H+ transporting accessory protein 2
7.048.747	-0.1350318	NPAS3	neuronal PAS domain protein 3
7.045.467	0.311127	CDC27	cell division cycle 27
7.045.418	0.2194088	SMIM8	small integral membrane protein 8
7.038.121	-0.3278121	MPRIP	myosin phosphatase Rho interacting protein
7.032.318	-0.1587544	TFEB	transcription factor EB
702.973	-0.3257757	ACOX3	acyl-CoA oxidase 3, pristanoyl
7.028.055	0.2206217	TTC3	tetratricopeptide repeat domain 3
7.024.887	-0.3254476	IL11RA	interleukin 11 receptor subunit alpha
7.021.436	-0.3000577	DHX32	DEAH-box helicase 32 (putative)
7.020.612	-0.11367	CITED1	Cbp/p300 interacting transactivator with Glu/As
7.019.351	-0.2600883	CHD9	chromodomain helicase DNA binding protein 9
7.014.988	-0.09561	MED14	mediator complex subunit 14
701.476	-0.5003085	RFTN1	raftlin, lipid raft linker 1
7.013.119	0.193669	RABGGTA	Rab geranylgeranyltransferase alpha subunit
7.011.264	0.2821747	FBXO11	F-box protein 11
7.009.285	-0.1354927	WIPI2	WD repeat domain, phosphoinositide interactir
7.009.177	0.2744297	ATG5	autophagy related 5
7.001.276	-0.1695391	GJA8	gap junction protein alpha 8
6.999.163	-0.2097158	WWOX	WW domain containing oxidoreductase
6.995.824	-0.1537131	FABP2	fatty acid binding protein 2
6.991.268	0.1919932	NDUFB11	NADH:ubiquinone oxidoreductase subunit B11
6.990.389	-0.1140753	FRMPD4	FERM and PDZ domain containing 4
699.001	0.4619242	MLEC	malectin
6.989.024	-0.1752536	OVCA2///D	ovarian tumor suppressor candidate 2///diphth
6.988.985	0.2325359	ZMYND11	zinc finger MYND-type containing 11
6.987.214	0.1940511	ARHGEF9	Cdc42 guanine nucleotide exchange factor 9
6.985.711	-0.1266273	CCDC30	coiled-coil domain containing 30
6.985.372	0.4788563		
6.982.513	0.1595448	GTF3C4	general transcription factor IIIC subunit 4
6.979.763	-0.1508282	EFNA5	ephrin A5
6.977.704	-0.1777348	GABRB2	gamma-aminobutyric acid type A receptor beta
6.975.359	-0.2589483	SGSM2	small G protein signaling modulator 2
697.087	0.2664668	PUM1	pumilio RNA binding family member 1
6.958.281	-0.1741328	GAB1	GRB2 associated binding protein 1
6.955.987	-0.1251266	SLC17A7	solute carrier family 17 member 7
6.955.845	0.3082938	LRP4	LDL receptor related protein 4
6.955.579	0.3279682	STAG2	stromal antigen 2
695.036	-0.3841279	ITPR2	inositol 1,4,5-trisphosphate receptor type 2
6.949.995	-0.1430362	CADM3	cell adhesion molecule 3
6.947.389	0.3269597	TXLNA	taxilin alpha
6.932.614	-0.1622496	NTRK3	neurotrophic receptor tyrosine kinase 3
6.932.272	-0.2342612	ALMS1	ALMS1, centrosome and basal body associated

6.932.025	-0.1408333	DOHH	deoxyhypusine hydroxylase/monooxygenase
6.931.414	-0.1103188	PCDH7	protocadherin 7
6.927.479	-0.1632719	SOGA1	suppressor of glucose, autophagy associated 1
6.927.022	-0.0973892	KCNH6	potassium voltage-gated channel subfamily H r
692.356	0.2481263	IRF3	interferon regulatory factor 3
6.923.398	-0.1437603	MAG///CD2	myelin associated glycoprotein///CD22 molecu
692.276	0.4860863	IPW	imprinted in Prader-Willi syndrome (non-protei
6.921.858	-0.1917148	APOBEC3C	apolipoprotein B mRNA editing enzyme catalyti
6.921.349	-0.140136	SLC34A1	solute carrier family 34 member 1
6.915.992	-0.1144947	GTF2IRD2	GTF2I repeat domain containing 2
6.911.962	-0.1415339	PCDH7	protocadherin 7
6.910.113	0.2140652	LOC72890(	similar to prolyl 4-hydroxylase beta-subunit///p
6.908.251	-0.1330056		
6.905.316	-0.2214041	PLEKHA4	pleckstrin homology domain containing A4
69.043	0.2952027	RAB2A	RAB2A, member RAS oncogene family
6.902.449	-0.1527505		
6.902.294	0.3066573	UPP1	uridine phosphorylase 1
6.900.107	-0.2798238	PRDX2	peroxiredoxin 2
689.705	-0.3624743	PTP4A1	protein tyrosine phosphatase type IVA, membe
6.895.942	0.2464002	TCEB3	transcription elongation factor B subunit 3
6.891.971	0.3154327	HIST1H2BC	histone cluster 1, H2bc///histone cluster 1, H2
6.891.786	-0.4956502	C4BPB	complement component 4 binding protein beta
6.891.752	0.3147924	ANKRD28	ankyrin repeat domain 28
6.891.367	-0.1341077	BCAP29	B-cell receptor-associated protein 29
6.887.917	-0.5874563	HLA-DRB1	major histocompatibility complex, class II, DR b
6.886.632	0.1979678	NCOA1	nuclear receptor coactivator 1
6.880.324	0.2266639	INPPL1	inositol polyphosphate phosphatase like 1
6.879.772	-0.4192025	ECI2	enoyl-CoA delta isomerase 2
6.876.135	0.212806	ARNT	aryl hydrocarbon receptor nuclear translocator
6.875.887	-0.1452642	CCKAR	cholecystokinin A receptor
6.875.385	-0.1862946	IRS4	insulin receptor substrate 4
6.874.691	-0.1341479	HOXA6	homeobox A6
6.870.776	0.1889315	C2orf68	chromosome 2 open reading frame 68
6.870.275	-0.09745		
6.866.714	-0.1457245	ST8SIA3	ST8 alpha-N-acetyl-neuraminide alpha-2,8-sial
6.853.941	-0.1661084	MAB21L2	mab-21 like 2
6.850.975	-0.3142469	LY86	lymphocyte antigen 86
6.849.888	0.404877	CTBP1	C-terminal binding protein 1
6.844.493	0.2089526	SUPT7L	SPT7-like STAGA complex gamma subunit
6.844.089	0.3534385	PCMTD2	protein-L-isoaspartate (D-aspartate) O-methylt
6.842.942	-0.1804033	CC2D1A	coiled-coil and C2 domain containing 1A
683.979	-0.1164609	EPHA5	EPH receptor A5
6.838.618	-0.1893068	SYDE1	synapse defective Rho GTPase homolog 1
6.838.259	-0.2808663	ZKSCAN1	zinc finger with KRAB and SCAN domains 1

683.631	-0.3139672	NIT1	nitrilase 1
6.835.985	-0.1884712	IL17RA	interleukin 17 receptor A
6.835.764	0.2062197	PSIP1	PC4 and SFRS1 interacting protein 1
6.834.879	0.2400202	NECAP2	NECAP endocytosis associated 2
6.834.716	-0.1491417	RTEL1	regulator of telomere elongation helicase 1
6.828.457	0.2639954	DAG1	dystroglycan 1
6.824.465	-0.1106973		
6.824.387	-0.1200836	CACNA1E	calcium voltage-gated channel subunit alpha1
6.816.767	-0.1261344	PLA2G2F	phospholipase A2 group IIF
6.810.694	-0.1995134	RIN1	Ras and Rab interactor 1
6.803.456	-0.3487013	FOXA2	forkhead box A2
6.801.512	-0.1741616	KCNG1	potassium voltage-gated channel modifier subf
6.796.886	0.3690681		
6.794.715	0.2960837	TTC3	tetratricopeptide repeat domain 3
6.794.647	-0.1318679	ZBTB32	zinc finger and BTB domain containing 32
6.792.407	-0.1316367	ALX4	ALX homeobox 4
6.788.424	-0.1687254	RFTN1	raftlin, lipid raft linker 1
6.788.248	-0.1221527	SHPRH	SNF2 histone linker PHD RING helicase
6.788.228	0.2443891	KARS	lysyl-tRNA synthetase
6.785.847	-0.1157522	ZNF614	zinc finger protein 614
6.785.566	-0.1264192	C6orf10	chromosome 6 open reading frame 10
6.784.034	-0.1323659	GJD2	gap junction protein delta 2
6.783.183	-0.1474894	OR51E2	olfactory receptor family 51 subfamily E membe
6.781.992	0.2661168	UBA5	ubiquitin like modifier activating enzyme 5
6.781.678	-0.179552	GOLIM4	golgi integral membrane protein 4
6.769.575	-0.121676	ACRV1	acrosomal vesicle protein 1
6.762.109	0.320216	CLIP1	CAP-Gly domain containing linker protein 1
675.938	-0.4328358	THNSL2	threonine synthase like 2
6.758.016	-0.1415597	GABRQ	gamma-aminobutyric acid type A receptor theta
6.757.005	0.3421487	ARHGAP5	Rho GTPase activating protein 5
6.755.303	0.2873406	RAB6C///R/	RAB6C, member RAS oncogene family///RAB6A
6.733.775	-0.1944035	TFAP2A	transcription factor AP-2 alpha
6.733.437	-0.1409303	LOC10028!	immunoglobin superfamily member 21 pseudo
6.726.811	-0.4626988	TRBC1///TF	T cell receptor beta constant 1///T cell receptor
6.725.779	-0.1584388	ZNF688	zinc finger protein 688
6.723.736	-0.5036059	SRGN	serglycin
6.723.101	0.3406182	ZNF652	zinc finger protein 652
6.718.616	-0.1300282	RAX	retina and anterior neural fold homeobox
6.718.146	-0.13108	ZFP36L1	ZFP36 ring finger protein like 1
6.717.499	0.290951	LSG1	large 60S subunit nuclear export GTPase 1
6.713.892	-0.3074206	SHB	SH2 domain containing adaptor protein B
6.704.044	0.2765963	TFDP1	transcription factor Dp-1
6.703.912	0.2815461	ACVR1B	activin A receptor type 1B
6.698.731	0.178092	ASB6	ankyrin repeat and SOCS box containing 6

669.818	-0.2070778	PCDH1	protocadherin 1
6.697.249	-0.2061545	TELO2	telomere maintenance 2
6.691.858	-0.6401415	FAM171A1	family with sequence similarity 171 member A1
6.691.529	0.2727986	PANX1	pannexin 1
6.687.788	0.5614588	TMEM230	transmembrane protein 230
6.685.582	-0.1253596	GRIN1	glutamate ionotropic receptor NMDA type subu
6.685.268	-0.134306	EFR3B	EFR3 homolog B
6.685.182	-0.1680425	CACNA2D2	calcium voltage-gated channel auxiliary subuni
6.683.131	0.2778473	PPP1CB	protein phosphatase 1 catalytic subunit beta
6.682.119	-0.2364275	DIAPH1	diaphanous related formin 1
6.681.574	0.2740505	STXBP3	syntaxin binding protein 3
6.680.658	-0.3375316	TMED5	transmembrane p24 trafficking protein 5
6.679.246	0.1541409	KLHL22	kelch like family member 22
6.678.893	0.5957071	LY96	lymphocyte antigen 96
6.678.565	0.260471	PIGO	phosphatidylinositol glycan anchor biosynthesi
6.673.877	0.3790818	ISG20	interferon stimulated exonuclease gene 20
667.247	-0.1223056	HIST1H2BL	histone cluster 1, H2bl
6.669.764	0.1789263	RPL3	ribosomal protein L3
6.666.246	-0.2665787	MCL1	BCL2 family apoptosis regulator
6.665.381	0.3828502	MAGEC2	MAGE family member C2
6.665.167	0.216006	SRRT	serrate, RNA effector molecule
666.233	-0.1436047	ZHX3	zinc fingers and homeoboxes 3
666.216	0.2933213	TRHDE	thyrotropin releasing hormone degrading enzym
6.655.861	0.18166	COX7C	cytochrome c oxidase subunit 7C
6.654.906	0.5637102	CSRP2	cysteine and glycine rich protein 2
6.654.321	0.2318828	PAK4	p21 (RAC1) activated kinase 4
665.047	0.1284021	AP4M1	adaptor related protein complex 4 mu 1 subunit
6.646.505	0.2399779	FEM1C	fem-1 homolog C
6.639.451	0.1931446	TEAD4	TEA domain transcription factor 4
6.638.268	-0.3196408	RAPGEF2	Rap guanine nucleotide exchange factor 2
663.455	0.2097087	TMEM62	transmembrane protein 62
6.627.059	-0.1844885	ATP2B4	ATPase plasma membrane Ca2+ transporting 4
6.626.961	-0.1626552	SPDEF	SAM pointed domain containing ETS transcripti
6.626.572	-0.2896843	ZCCHC2	zinc finger CCHC-type containing 2
6.623.194	-0.3815781	SLC1A1	solute carrier family 1 member 1
6.622.993	-0.1730852	CRTC1	CREB regulated transcription coactivator 1
6.622.992	0.2249809	COPS8	COP9 signalosome subunit 8
6.622.922	-0.3184421	EIF5	eukaryotic translation initiation factor 5
6.620.565	-0.2113113	NR4A3	nuclear receptor subfamily 4 group A member 3
6.619.365	-0.1194838	PHF24	PHD finger protein 24
6.618.998	-0.1150674		
6.618.742	-0.3580918		
661.227	0.6138207	DPP4	dipeptidyl peptidase 4
6.605.377	-0.1944957	PLD1	phospholipase D1

6.603.738	-0.1915822	FAM65B	family with sequence similarity 65 member B
6.602.616	0.2807091	ATP5G3	ATP synthase, H+ transporting, mitochondrial F
6.601.827	0.1534755	KIAA1107	KIAA1107
6.598.607	0.3545284	ETV1	ETS variant 1
659.621	0.2578646	CTTN	cortactin
6.595.156	-0.1425615	UNC45A	unc-45 myosin chaperone A
6.592.248	-0.1285936	MUSK	muscle associated receptor tyrosine kinase
658.469	-0.0991801	P2RY10	purinergic receptor P2Y10
6.580.224	-0.1337397	RALGAPA1	Ral GTPase activating protein catalytic alpha su
6.578.787	0.1824585	PTGDR2	prostaglandin D2 receptor 2
6.578.209	0.1917764	ARPC4	actin related protein 2/3 complex subunit 4
6.575.442	0.3562271	ABCB6	ATP binding cassette subfamily B member 6 (La
657.446	-0.1212112	MAF	MAF bZIP transcription factor
657.437	0.3118781	INTS12	integrator complex subunit 12
6.571.905	-0.1044166	N4BP3	NEDD4 binding protein 3
6.571.766	0.1540612	RPL18P11	ribosomal protein L18 pseudogene 11
656.913	-0.1731116	CXCR6	C-X-C motif chemokine receptor 6
6.568.562	-0.3197812	PPP1R15A	protein phosphatase 1 regulatory subunit 15A
6.552.123	-0.2803498	TLE4	transducin like enhancer of split 4
6.551.492	0.1828324	SCAMP1	secretory carrier membrane protein 1
654.436	-0.2861655	FBXW12	F-box and WD repeat domain containing 12
654.272	-0.1315766	NRP1	neuropilin 1
6.540.976	0.6007069	LAMB1	laminin subunit beta 1
6.528.087	-0.2309358	R3HDM2	R3H domain containing 2
652.777	-0.26468	MON2	MON2 homolog, regulator of endosome-to-Golg
6.525.463	-0.3995276	GCLC	glutamate-cysteine ligase catalytic subunit
6.523.885	0.306259	ANGPT1	angiopoietin 1
6.510.091	0.1264586	AFAP1	actin filament associated protein 1
6.509.472	0.3385715	ASAH1	N-acylsphingosine amidohydrolase 1
6.509.468	0.3226823	PPIP5K2	diphosphoinositol pentakisphosphate kinase 2
6.504.839	-0.3346658		
6.503.011	-0.1733011	GNL3L	G protein nucleolar 3 like
6.499.643	0.2314681	NUFIP1	NUFIP1, FMR1 interacting protein 1
6.490.105	-0.1219018	OTUD7B	OTU deubiquitinase 7B
6.490.016	-0.121057	IL17A	interleukin 17A
6.489.563	-0.5287399	DCPS	decapping enzyme, scavenger
6.488.571	0.2517281	NCOA1	nuclear receptor coactivator 1
6.484.575	0.3120711	WNK1	WNK lysine deficient protein kinase 1
6.479.446	-0.1170913	IFNW1	interferon omega 1
6.477.101	-0.1985773	FCER1A	Fc fragment of IgE receptor Ia
6.472.448	-0.3705276	ST6GAL1	ST6 beta-galactoside alpha-2,6-sialyltransferas
6.471.025	-0.3200447	PNISR	PNN interacting serine and arginine rich proteir
647.039	-0.1327553	PRM1	protamine 1
6.466.246	-0.6132678	C1QB	complement C1q B chain

6.465.195	0.2613889	ARNT	aryl hydrocarbon receptor nuclear translocator
6.458.913	0.1933897	KDELR3	KDEL endoplasmic reticulum protein retention
6.454.012	-0.2126461	IKZF1	IKAROS family zinc finger 1
6.451.615	-0.1399684	GRSF1	G-rich RNA sequence binding factor 1
6.449.663	-0.2322202	CIB1	calcium and integrin binding 1
6.448.526	-0.2336121	RASGRP2	RAS guanyl releasing protein 2
6.448.247	-0.1279989	PQLC2	PQ loop repeat containing 2
6.447.952	-0.1492734	ESR1	estrogen receptor 1
6.442.197	-0.2573177	SMG1	SMG1, nonsense mediated mRNA decay assoc
6.430.458	-0.2764246	DCTD	dCMP deaminase
6.427.268	0.175627	ATP6V0E1	ATPase H+ transporting V0 subunit e1
642.278	-0.6975947	COL3A1	collagen type III alpha 1 chain
6.420.515	-0.2614764	STX2	syntaxin 2
6.420.441	-0.1408688		
6.416.267	-0.0995273	ANP32AP1/	acidic nuclear phosphoprotein 32 family memb
641.249	-0.1116166	IFNA4	interferon alpha 4
641.179	-0.2394353	ADH5	alcohol dehydrogenase 5 (class III), chi polypep
6.408.451	-0.132969	CYB5A	cytochrome b5 type A
6.407.839	0.3092176	FASTKD1	FAST kinase domains 1
6.406.376	-0.4078522	CCR1	C-C motif chemokine receptor 1
6.404.768	-0.2790903	OSBPL9	oxysterol binding protein like 9
6.403.599	-0.1099077	DLX4	distal-less homeobox 4
6.403.563	0.1518481	LOC64546(	insulin like growth factor 2 mRNA binding protei
6.400.457	-0.1447203	UNC13B	unc-13 homolog B
6.398.668	-0.1364063	MFSD7	major facilitator superfamily domain containing
6.397.794	-0.1599558	ASIC2	acid sensing ion channel subunit 2
6.393.211	0.2700287	USP13	ubiquitin specific peptidase 13 (isopeptidase T-
638.852	0.2072475	CHPF2	chondroitin polymerizing factor 2
6.387.449	-0.1711094	EPS8L1	EPS8 like 1
638.483	-0.1441448	CRLF2	cytokine receptor-like factor 2
6.384.296	0.1990039	MSX1	msh homeobox 1
6.380.711	0.2122631	PDGFRL	platelet derived growth factor receptor like
6.378.599	0.2169506	TPI1	triosephosphate isomerase 1
6.376.164	-0.3418384	NUDT9	nudix hydrolase 9
6.375.645	-0.3519975	HIBCH	3-hydroxyisobutyryl-CoA hydrolase
6.366.697	0.2327742	RNF40	ring finger protein 40
6.365.185	0.2650239	CCDC28B	coiled-coil domain containing 28B
6.356.762	-0.2391302	ZNF354A	zinc finger protein 354A
6.355.053	-0.0958945	MLF1	myeloid leukemia factor 1
6.354.921	-0.0966757	CHD3	chromodomain helicase DNA binding protein 3
6.343.954	-0.6813082	FGFR2	fibroblast growth factor receptor 2
6.331.611	0.2119533	RELA	RELA proto-oncogene, NF-kB subunit
6.329.302	-0.8634515	CFHR5	complement factor H related 5
6.329.261	-0.1287498	DNAJC4	DnaJ heat shock protein family (Hsp40) membe

6.328.724	-0.1833494	KDSR	3-ketodihydrosphingosine reductase
6.323.043	0.2572441	FRY	FRY microtubule binding protein
6.320.637	-0.4400621	CALM1	calmodulin 1
6.316.493	-0.1348081	ADRA1D	adrenoceptor alpha 1D
631.384	-0.1237506	GFRA2	GDNF family receptor alpha 2
6.312.678	0.3441582	PDE9A	phosphodiesterase 9A
6.309.251	0.1981539	EIF2B5	eukaryotic translation initiation factor 2B subur
6.302.669	0.2679163	PCBP2	poly(rC) binding protein 2
6.299.915	-0.1851527	TPT1	tumor protein, translationally-controlled 1
6.299.194	-0.149228	PTCRA	pre T-cell antigen receptor alpha
629.481	0.2423145	SAP18	Sin3A associated protein 18
628.885	-0.1618218		
6.286.571	0.1757228	OAZ3	ornithine decarboxylase antizyme 3
6.277.997	-0.1370728	CACNG3	calcium voltage-gated channel auxiliary subuni
6.271.838	-0.1005068		
6.271.804	0.2446962	UNC119	unc-119 lipid binding chaperone
6.270.992	-0.2983955	FYB	FYN binding protein
6.267.412	0.4059427	AP3D1	adaptor related protein complex 3 delta 1 subur
6.267.213	-0.1237228	EPS8L1	EPS8 like 1
6.267.023	-0.1206299	MIP	major intrinsic protein of lens fiber
6.263.033	-0.1486261	DKKL1	dickkopf like acrosomal protein 1
6.262.128	-0.1241971	CDH8	cadherin 8
6.262.114	-0.1035364	HRK	harakiri, BCL2 interacting protein
625.759	-0.1518484	TNXB	tenascin XB
6.257.275	-0.1288097	LINC01565	long intergenic non-protein coding RNA 1565
6.256.801	0.1488647	ZSCAN5A	zinc finger and SCAN domain containing 5A
6.256.696	0.2261102	PARN	poly(A)-specific ribonuclease
6.254.795	-0.4546117	GUSBP11	glucuronidase, beta pseudogene 11
6.253.473	-0.1470183		
6.251.279	0.2332652	KIAA0391	KIAA0391
6.250.259	-0.2863344	PRKCB	protein kinase C beta
6.249.102	-0.1601131	REEP2	receptor accessory protein 2
6.244.937	-0.1949415	LAT2	linker for activation of T-cells family member 2
6.241.981	-0.2649652	GUCY1B3	guanylate cyclase 1 soluble subunit beta
6.235.278	-0.278197	EHBP1	EH domain binding protein 1
6.235.081	-0.2276099		
6.233.982	-0.1536962	NPRL3	NPR3 like, GATOR1 complex subunit
6.232.046	0.1358716	HIF1AN	hypoxia inducible factor 1 alpha subunit inhibit
6.231.224	-0.3902501	CD52	CD52 molecule
6.227.484	0.2136519	MAN1A2	mannosidase alpha class 1A member 2
6.225.849	0.2101757	GAR1	GAR1 ribonucleoprotein
622.417	0.7641767	GAGE2A///(	G antigen 2A///G antigen 12H///G antigen 12E//
6.222.242	0.2270973	ROCK1	Rho associated coiled-coil containing protein k
6.221.295	-0.5367738	IGLL5///IGL	immunoglobulin lambda like polypeptide 5///im

6.211.472	-0.1485775		
6.210.687	-0.1375037	FUT3	fucosyltransferase 3 (Lewis blood group)
6.208.727	-0.257721	RFNG	RFNG O-fucosylpeptide 3-beta-N-acetylglucosa
6.202.827	0.3404902	GPX1	glutathione peroxidase 1
6.202.649	0.2059461	MAN1B1	mannosidase alpha class 1B member 1
619.991	0.1321395	TTLL1	tubulin tyrosine ligase like 1
6.198.544	0.2652908	FLNB	filamin B
6.187.744	-0.3638363	SCHIP1	schwannomin interacting protein 1
6.185.982	-0.2130049	ITK	IL2 inducible T-cell kinase
6.182.312	-0.2196703	CD37	CD37 molecule
6.179.319	-0.2885871	ATP2A3	ATPase sarcoplasmic/endoplasmic reticulum C
6.178.851	0.2105888	GLTSCR1L	GLTSCR1 like
6.176.634	0.1883458	HYAL3	hyaluronoglucosaminidase 3
6.176.482	-0.1413088	NEUROG2	neurogenin 2
6.169.028	0.3484776	CYB561	cytochrome b561
6.167.857	0.1761036	C6orf47	chromosome 6 open reading frame 47
6.166.807	0.346309	FRG1JP	FSHD region gene 1 family member J, pseudoge
616.629	0.2570458	PLXDC1	plexin domain containing 1
6.165.183	-0.3782609	ZNF160	zinc finger protein 160
6.158.796	-0.3541855	IRF9	interferon regulatory factor 9
6.155.747	0.2032177	CUEDC2	CUE domain containing 2
6.155.429	0.2446573	RPL15	ribosomal protein L15
615.382	0.2664691	PNRC2	proline rich nuclear receptor coactivator 2
6.151.917	0.2741257	FEZ2	fasciculation and elongation protein zeta 2
6.148.578	-0.3079565	IL15	interleukin 15
6.148.312	-0.1091037	CLCA2	chloride channel accessory 2
6.142.827	0.1316949	CDK5R1	cyclin dependent kinase 5 regulatory subunit 1
6.141.365	0.2470553	SLC22A4	solute carrier family 22 member 4
6.140.038	-0.1503275	CRYBB3	crystallin beta B3
6.137.434	-0.1179702		
6.137.085	-0.1637399	SEMA4A	semaphorin 4A
6.136.349	0.5732868	PRKAB2	protein kinase AMP-activated non-catalytic sub
6.136.315	0.4137615	CALR	calreticulin
6.130.172	-0.1230516	SNTB2	syntrophin beta 2
6.125.334	-0.1116232	CCNA1	cyclin A1
6.124.239	0.2220422	RNF10	ring finger protein 10
6.117.284	-0.1488499	NTRK3	neurotrophic receptor tyrosine kinase 3
6.115.341	-0.1049893	SRPX2	sushi repeat containing protein, X-linked 2
6.114.711	-0.1541215	FOSL2	FOS like 2, AP-1 transcription factor subunit
6.114.067	-0.125101	STYK1	serine/threonine/tyrosine kinase 1
6.107.792	-0.2598231	AKTIP	AKT interacting protein
6.107.126	-0.1226453	S100G	S100 calcium binding protein G
6.103.792	-0.4183053	KCTD12	potassium channel tetramerization domain cor
6.102.072	0.1860003	STOML1	stomatin like 1

6.097.404	0.2549553	ADNP2	ADNP homeobox 2
6.095.887	-0.2777336	RBBP6	RB binding protein 6, ubiquitin ligase
6.091.878	0.3456421	VGLL4	vestigial like family member 4
6.090.952	-0.1039771	SHISA6	shisa family member 6
6.087.476	-0.1178178	GRIK1	glutamate ionotropic receptor kainate type sub
6.071.898	-0.1224463		
6.069.782	0.2086601	WRN	Werner syndrome RecQ like helicase
6.068.838	0.2338442	HIP1	huntingtin interacting protein 1
6.067.726	-0.1220911	HTR7	5-hydroxytryptamine receptor 7
6.066.978	-0.38622	ALDH3A2	aldehyde dehydrogenase 3 family member A2
6.066.224	-0.2043825	HBEGF	heparin binding EGF like growth factor
6.065.305	-0.1190138	CTRL	chymotrypsin like
6.059.581	-0.1172898		
6.058.502	0.1780497	ELL3	elongation factor for RNA polymerase II 3
6.051.497	0.4565375	NET1	neuroepithelial cell transforming 1
604.625	0.2622762	HMG20A	high mobility group 20A
604.169	-0.1639937	CD40	CD40 molecule
603.888	-0.0971367	FAIM2	Fas apoptotic inhibitory molecule 2
6.024.984	0.4649655	CSRP2	cysteine and glycine rich protein 2
6.024.936	-0.2049046	FAM49B	family with sequence similarity 49 member B
6.023.017	0.1786793	CNR1	cannabinoid receptor 1
6.017.698	0.2100953	LOC15506(	AI894139 pseudogene
6.015.767	0.8167126	POSTN	periostin
6.015.217	0.2324462	M6PR	mannose-6-phosphate receptor, cation depenc
6.010.176	0.1409908	CBX8	chromobox 8
6.005.653	-0.5873094	TRBC1///TF	T cell receptor beta constant 1///T cell receptor
6.004.783	-0.1167691	KIAA1659	KIAA1659 protein
6.004.045	-0.1219277	LILRA5	leukocyte immunoglobulin like receptor A5
6.003.618	-0.103354		
6.003.201	-0.4609608	FSTL1	follistatin like 1
6.001.437	0.1555309	C15orf39	chromosome 15 open reading frame 39
6.000.676	-0.1632479	GALNT8	polypeptide N-acetylgalactosaminyltransferase
5.998.622	-0.1556901	ABO	ABO blood group (transferase A, alpha 1-3-N-ac
5.994.288	-0.1299193	AQP2	aquaporin 2
5.993.272	-0.1891702		
5.989.456	-0.1076449	RREB1	ras responsive element binding protein 1
5.988.552	-0.1637651	LOC10537:	uncharacterized LOC105377443
5.980.251	-0.1527154	BATF3	basic leucine zipper ATF-like transcription facto
5.977.493	0.2192567	TSPAN5	tetraspanin 5
5.970.634	-0.6031119	AMFR	autocrine motility factor receptor
5.969.798	-0.1108734	RGS12///GI	regulator of G-protein signaling 12///glutamate
5.967.934	0.2926169	IQSEC1	IQ motif and Sec7 domain 1
5.964.616	0.3673671	PDAP1	PDGFA associated protein 1
5.964.535	-0.1686914		

5.963.643	-0.5332088	ITIH3	inter-alpha-trypsin inhibitor heavy chain 3
5.962.704	-0.1268296	RREB1	ras responsive element binding protein 1
5.960.258	-0.1329643	FLJ22596	hypothetical protein FLJ22596
5.955.068	-0.2975324	CFHR1///C	complement factor H related 1///complement f
5.953.738	-0.1163008	PPEF1	protein phosphatase with EF-hand domain 1
5.952.346	0.2423886	ATP5O	ATP synthase, H+ transporting, mitochondrial F
5.952.035	-0.2923647	GRPEL1	GrpE like 1, mitochondrial
5.951.094	0.75479	GAGE12J///	G antigen 12J///G antigen 12G///G antigen 12I//
5.948.721	-0.3843203	WSB1	WD repeat and SOCS box containing 1
5.946.088	-0.1212536	DPEP3	dipeptidase 3
5.944.453	-0.155688	LOXL2	lysyl oxidase like 2
5.940.416	-0.1235753	ATP2B3	ATPase plasma membrane Ca2+ transporting 3
5.939.303	-0.1529171		
5.938.053	0.21089	LRRC61	leucine rich repeat containing 61
5.936.218	-0.1175381	HLA-DRB6	major histocompatibility complex, class II, DR b
5.933.137	-0.1337209	SPEG	SPEG complex locus
5.932.754	0.3405457	TNNC1	troponin C1, slow skeletal and cardiac type
593.106	-0.4476589	NECAB2	N-terminal EF-hand calcium binding protein 2
5.927.962	-0.1474621	ARVCF	armadillo repeat gene deleted in velocardiofaci
592.709	0.1637693	HIST1H4H	histone cluster 1, H4h
5.926.537	0.4019673	ACTB	actin beta
5.922.915	-0.1791392		
5.922.161	-0.164453	MAX	MYC associated factor X
5.918.883	0.2199146	GBF1	golgi brefeldin A resistant guanine nucleotide e
5.918.625	0.2068133	LOC72819	similar to transcription associated factor TAFII3
5.916.063	-0.126149	CDC27	cell division cycle 27
5.915.371	-0.1402999	IDS	iduronate 2-sulfatase
5.910.975	0.29424	PARVB	parvin beta
5.903.319	-0.1813831	PARD6A	par-6 family cell polarity regulator alpha
5.901.524	0.2935448	RBCK1	RANBP2-type and C3HC4-type zinc finger conta
589.916	0.208144	B3GALNT1	beta-1,3-N-acetylgalactosaminyltransferase 1
589.701	-0.1484553	SPN	sialophorin
5.896.334	-0.3579646	SPRY4	sprouty RTK signaling antagonist 4
5.892.927	-0.2051913	RASGRP2	RAS guanyl releasing protein 2
5.892.911	-0.1441371		
5.891.539	-0.3461573	MBD4	methyl-CpG binding domain 4, DNA glycosylase
5.887.931	0.113276	CCNF	cyclin F
5.885.357	-0.427291	GPLD1	glycosylphosphatidylinositol specific phosphol
5.885.259	-0.3666939	KLHDC10	kelch domain containing 10
5.884.148	-0.2970513	EFEMP2	EGF containing fibulin like extracellular matrix p
5.882.603	-0.1070401	RPL23AP47	ribosomal protein L23a pseudogene 47
5.882.149	-0.3750532	FN1	fibronectin 1
5.881.888	-0.1446777	LOC65067	similar to aconitase 2, mitochondrial///similar t
5.879.666	-0.1080818	TGM4	transglutaminase 4

5.879.108	-0.3549501	ACSF2	acyl-CoA synthetase family member 2
5.876.712	-0.1712589	PYY2	peptide YY, 2 (pseudogene)
5.874.456	0.1975348	FASTK	Fas activated serine/threonine kinase
5.871.616	-0.106107	CSF1	colony stimulating factor 1
5.868.584	-0.1650114	ATXN8OS	ATXN8 opposite strand (non-protein coding)
586.444	-0.175304	CACNB3	calcium voltage-gated channel auxiliary subuni
586.221	-0.2222791	PDLIM3	PDZ and LIM domain 3
5.859.181	-0.1291539	CTNNA1	catenin alpha 1
5.856.977	-0.2340961	NPIPA2///N	nuclear pore complex interacting protein family
5.850.832	0.2181611	RPS6KB1	ribosomal protein S6 kinase B1
5.850.195	0.3807411	WNT5A	Wnt family member 5A
5.848.595	-0.2970619	TSPAN4	tetraspanin 4
5.847.409	-0.1333266		
5.846.006	0.2354152	NDUFC2	NADH:ubiquinone oxidoreductase subunit C2
5.845.101	-0.1489487	PRDM12	PR/SET domain 12
5.838.028	-0.1257997	MMP16	matrix metallopeptidase 16
5.835.326	-0.2568972	RRBP1	ribosome binding protein 1
5.828.371	0.4213218	RHOQ	ras homolog family member Q
5.824.086	-0.1574269	PRKACG	protein kinase cAMP-activated catalytic subuni
5.821.974	0.2409907	SERPINB6	serpin family B member 6
5.817.352	0.401567	SMARCA1	SWI/SNF related, matrix associated, actin depe
5.814.601	-0.1340968	CD1E	CD1e molecule
5.807.433	-0.1097862	NRIP2	nuclear receptor interacting protein 2
5.803.677	-0.1362105		
5.800.613	0.2150283	ADO	2-aminoethanethiol dioxygenase
5.796.233	-0.16639	SSTR4	somatostatin receptor 4
5.791.475	-0.1983197	SRSF7	serine and arginine rich splicing factor 7
5.790.738	-0.1670195	ROBO4	roundabout guidance receptor 4
5.790.486	-0.3457753	TGFA	transforming growth factor alpha
5.790.202	0.2308234	RAB5C	RAB5C, member RAS oncogene family
5.785.195	0.24918	MAN1B1	mannosidase alpha class 1B member 1
5.785.076	-0.1358642	EBI3	Epstein-Barr virus induced 3
5.783.318	-0.1605754	TNFRSF9	TNF receptor superfamily member 9
578.288	-0.2160246	ESD	esterase D
5.774.189	0.2458415	KIAA0355	KIAA0355
5.773.817	-0.6088794	KLF4	Kruppel like factor 4
5.772.729	-0.2404041	MRM3	mitochondrial rRNA methyltransferase 3
5.770.967	-0.1579673	NPEPL1	aminopeptidase-like 1
5.767.268	0.2918696	GPC5	glypican 5
5.767.146	-0.1250971	RHO	rhodopsin
5.761.309	0.1991704	HNRNPD	heterogeneous nuclear ribonucleoprotein D
5.757.394	-0.1061652	GEMIN8	gem nuclear organelle associated protein 8
5.756.763	-0.3121309	MICALL2	MICAL like 2
5.755.609	-0.1408849	FXYD7	FXYD domain containing ion transport regulator

5.755.582	-0.3046266	NR1H3	nuclear receptor subfamily 1 group H member 3
5.753.921	-0.1778339	COL26A1	collagen type XXVI alpha 1 chain
5.749.785	-0.1529182	PDZRN3	PDZ domain containing ring finger 3
5.736.885	-0.1602974	COL4A4	collagen type IV alpha 4 chain
5.733.395	0.2265953	HSF1	heat shock transcription factor 1
5.732.121	0.2444361	RTN2	reticulon 2
573.157	-0.200384	ITGA9	integrin subunit alpha 9
5.729.767	-0.1680489	PVT1	Pvt1 oncogene (non-protein coding)
5.728.556	-0.3716642	TOB2	transducer of ERBB2, 2
5.728.367	0.4644184	COL1A1	collagen type I alpha 1 chain
5.728.103	-0.2895898	USP15	ubiquitin specific peptidase 15
5.728.076	0.1991294	C11orf68	chromosome 11 open reading frame 68
5.727.313	0.2442117	PSME1	proteasome activator subunit 1
5.726.539	-0.2870822	DACT1	dishevelled binding antagonist of beta catenin 1
5.722.267	-0.123769	NRG1	neuregulin 1
5.720.385	0.3239531	EIF1AX	eukaryotic translation initiation factor 1A, X-link
5.719.145	0.3723936	TACC1	transforming acidic coiled-coil containing prote
5.711.917	-0.1137783	ZNF280B	zinc finger protein 280B
5.706.531	0.2042262	ANAPC5	anaphase promoting complex subunit 5
5.701.944	0.2665259	LYPD1	LY6/PLAUR domain containing 1
5.699.742	0.1946873		
5.699.034	-0.1127095	RAB5A	RAB5A, member RAS oncogene family
5.696.933	0.3028168	DUOX1	dual oxidase 1
5.689.746	0.1902676	HDGF	hepatoma-derived growth factor
5.688.491	0.2145922	PIBF1	progesterone immunomodulatory binding facto
5.687.967	-0.1573579	NLRP1	NLR family pyrin domain containing 1
568.122	0.137779	NFKB2	nuclear factor kappa B subunit 2
5.676.617	0.170164	TFE3	transcription factor binding to IGHM enhancer 3
5.675.195	0.3682407	OAS3	2'-5'-oligoadenylate synthetase 3
5.673.121	0.2682817	HNRNPK	heterogeneous nuclear ribonucleoprotein K
5.667.178	-0.1221827	EDNRA	endothelin receptor type A
5.657.211	-0.2161492	FCGR2C	Fc fragment of IgG receptor IIc (gene/pseudoge
5.656.816	-0.1495032	BTF3P11	basic transcription factor 3 pseudogene 11
5.652.995	0.1374767	GNAL	G protein subunit alpha L
5.647.318	-0.1294676	GAS1	growth arrest specific 1
5.645.262	-0.1517829	KIR2DL3	killer cell immunoglobulin like receptor, two Ig d
5.644.742	-0.1202484	ROPN1B	rhophilin associated tail protein 1B
5.639.034	0.2155293	ZC3HAV1	zinc finger CCCH-type containing, antiviral 1
5.636.069	0.2613996	MPHOSPH9	M-phase phosphoprotein 9
5.633.708	0.1966545	TRIM23	tripartite motif containing 23
56.321	-0.1454755	DCAF15	DDB1 and CUL4 associated factor 15
5.631.254	-0.1938024	PLCH2	phospholipase C eta 2
563.025	0.2523961	MAP3K11	mitogen-activated protein kinase kinase kinase
5.628.735	0.1454353	USP39	ubiquitin specific peptidase 39

5.625.977	0.1399189	SLC25A40	solute carrier family 25 member 40
5.623.222	0.1667437	TTF1	transcription termination factor 1
5.617.071	-0.3371885	ZC3H7B	zinc finger CCCH-type containing 7B
5.615.907	0.2804646	VDR	vitamin D (1,25- dihydroxyvitamin D3) receptor
5.614.302	-0.1132623	RAB33A	RAB33A, member RAS oncogene family
5.613.327	-0.1110057	SEC14L3	SEC14 like lipid binding 3
5.611.326	-0.1324327	HCG9	HLA complex group 9 (non-protein coding)
5.611.302	0.3268036	PRPF4B	pre-mRNA processing factor 4B
5.608.096	-0.2284914	MZF1	myeloid zinc finger 1
560.616	0.2735384	FAM179B	family with sequence similarity 179 member B
5.606.132	-0.1122936	TRGV7	T cell receptor gamma variable 7 (pseudogene)
5.598.761	-0.1006943	ALDH1A3	aldehyde dehydrogenase 1 family member A3
559.144	-0.0956503		
5.590.575	-0.1149656	CREB3L1	cAMP responsive element binding protein 3 like
5.588.234	0.2583678	PSME4	proteasome activator subunit 4
5.586.623	0.2590258	ATG14	autophagy related 14
558.461	0.2169625	RPS6	ribosomal protein S6
5.584.171	-0.8353472	PTGDS	prostaglandin D2 synthase
5.583.764	0.2698463	PTHLH	parathyroid hormone like hormone
557.938	-0.3062745	SELL	selectin L
5.576.219	0.3907944	ATP2B2	ATPase plasma membrane Ca2+ transporting 2
5.572.424	-0.3604101	MTMR4	myotubularin related protein 4
5.570.099	0.1948488	PEX6	peroxisomal biogenesis factor 6
5.567.728	-0.3267113	MTMR4	myotubularin related protein 4
5.554.319	-0.1268819	NOP14-AS1	NOP14 antisense RNA 1
5.553.167	0.2071393	LYPLA2	lysophospholipase II
5.553.131	0.2215272	RRP8	ribosomal RNA processing 8, methyltransferase
5.552.726	0.2839781	BRD3	bromodomain containing 3
5.552.614	-0.3024462	PNISR	PNN interacting serine and arginine rich protein
5.546.123	0.4974714	PLBD1	phospholipase B domain containing 1
5.545.834	-0.326518	FAM69A	family with sequence similarity 69 member A
5.545.768	-0.1700423	TNFSF12	tumor necrosis factor superfamily member 12
5.544.477	-0.1230316	CEACAM5	carcinoembryonic antigen related cell adhesion
5.541.525	0.2045426	EXOSC2	exosome component 2
5.532.175	-0.2911622	ACAD8	acyl-CoA dehydrogenase family member 8
5.531.641	-0.5432361	IFI16	interferon gamma inducible protein 16
5.527.835	-0.1687794	ST6GAL1	ST6 beta-galactoside alpha-2,6-sialyltransferas
5.526.357	-0.3612771	TCEB3	transcription elongation factor B subunit 3
5.522.496	-0.3407696	DOK4	docking protein 4
552.111	-0.1707054	LRCH4	leucine rich repeats and calponin homology dor
5.515.463	-0.1991069	EHD2	EH domain containing 2
5.504.968	-0.1515188	KCNMA1	potassium calcium-activated channel subfamil
5.501.129	-0.117753	EVI5	ecotropic viral integration site 5
5.500.213	-0.1553983	VAC14	Vac14, PIKFYVE complex component

5.497.806	-0.2918048	MYO15B	myosin XVB
5.496.787	0.2287795	CUL3	cullin 3
5.495.542	-0.3250964	MCL1	BCL2 family apoptosis regulator
5.492.013	-0.1190157	TAAR5	trace amine associated receptor 5
549.153	0.2726873	CDC16	cell division cycle 16
5.481.206	-0.1182445	UCP3	uncoupling protein 3
5.480.672	-0.1051454	MLN	motilin
5.478.402	-0.1603462	SCLY	selenocysteine lyase
5.477.477	-0.1526631	MUC5AC	mucin 5AC, oligomeric mucus/gel-forming
5.472.333	-0.1510785	MAP3K10	mitogen-activated protein kinase kinase kinase
5.464.998	0.1548353	SLC25A16	solute carrier family 25 member 16
5.464.983	-0.1196806	INSL3	insulin like 3
5.461.918	-0.1210208	SLC24A1	solute carrier family 24 member 1
5.461.781	-0.3407965	HNF1B	HNF1 homeobox B
5.459.337	-0.1292916	NF2	neurofibromin 2
5.458.243	-0.1311018	POLR2C	RNA polymerase II subunit C
5.456.229	-0.3205754	NR2F2	nuclear receptor subfamily 2 group F member 2
5.453.833	0.2316586	NDUFB2	NADH:ubiquinone oxidoreductase subunit B2
5.453.287	-0.1786792	EPAS1	endothelial PAS domain protein 1
5.450.184	-0.1464526	FAHD2P1//	fumarylacetoacetate hydrolase domain contair
5.449.409	-0.1314682	ATRX	ATRX, chromatin remodeler
5.441.388	0.2155198		
5.440.178	-0.1003762	SLC4A5	solute carrier family 4 member 5
5.437.739	0.1897936	SIAH1	siah E3 ubiquitin protein ligase 1
5.437.113	-0.1067498	DCX	doublecortin
5.434.547	-0.1125742	HAB1	B1 for mucin
5.432.142	-0.3853736	FLJ21272	hypothetical protein FLJ21272
5.432.104	-0.4087917	OAS1	2'-5'-oligoadenylate synthetase 1
5.427.755	-0.1485867	FAM107A	family with sequence similarity 107 member A
5.425.975	-0.0910761	SNPH	syntaphilin
5.425.403	0.3300371	MCUR1	mitochondrial calcium uniporter regulator 1
5.425.179	0.276936	PRKAR1A	protein kinase cAMP-dependent type I regulato
5.421.736	-0.1231991	FGF5	fibroblast growth factor 5
5.420.544	0.1763882	DSTNP2	destrin, actin depolymerizing factor pseudogen
5.420.103	0.2340888	SPCS3	signal peptidase complex subunit 3
5.419.915	0.3194117	COPS4	COP9 signalosome subunit 4
5.419.015	0.5682066	CD36	CD36 molecule
5.417.681	-0.1410377	P3H3	prolyl 3-hydroxylase 3
5.416.515	0.2489066	TMEM223	transmembrane protein 223
5.408.913	-0.1359895	HTR4	5-hydroxytryptamine receptor 4
5.407.999	0.3248985	CTSD	cathepsin D
5.401.245	-0.155569	ZC3H7B	zinc finger CCCH-type containing 7B
5.400.133	-0.1636147	ADGRB2	adhesion G protein-coupled receptor B2
5.395.935	-0.1804615	POLG	DNA polymerase gamma, catalytic subunit

5.395.713	-0.1096855	CAMK2A	calcium/calmodulin dependent protein kinase
5.395.175	-0.1270515	ASTN1	astrotactin 1
5.394.541	0.1017731	CDKAL1	CDK5 regulatory subunit associated protein 1 li
5.393.635	-0.5181071	NAMPT	nicotinamide phosphoribosyltransferase
5.389.007	-0.1629971	DGCR11	DiGeorge syndrome critical region gene 11 (nor
5.386.068	-0.1663759	CBFB	core-binding factor beta subunit
5.379.101	0.1968817	SPG21	spastic paraplegia 21 (autosomal recessive, Ma
537.685	-0.1048453	NCR1	natural cytotoxicity triggering receptor 1
5.373.447	0.3507629	TMC5	transmembrane channel like 5
5.369.836	-0.1245937	TACC1	transforming acidic coiled-coil containing prote
5.368.129	-0.2780726	SRSF11	serine and arginine rich splicing factor 11
5.366.498	-0.16823	APBB2	amyloid beta precursor protein binding family B
5.366.428	-0.0900664	CALML3	calmodulin like 3
5.364.419	-0.1170898		
5.363.017	-0.1376007	PRKCA	protein kinase C alpha
5.361.596	-0.1607294	MR1	major histocompatibility complex, class I-relate
5.359.723	-0.1048165		
5.359.357	0.2586189	C18orf8	chromosome 18 open reading frame 8
5.356.828	-0.1933008	AHSP	alpha hemoglobin stabilizing protein
5.351.523	-0.1631353		
5.349.067	-0.1121211	PDE1C	phosphodiesterase 1C
5.346.875	-0.153716	DOK4	docking protein 4
5.343.221	-0.2328321	LONRF3	LON peptidase N-terminal domain and ring fing
5.337.603	0.1914182	CCR6	C-C motif chemokine receptor 6
5.336.809	-0.1623775	PLCB1	phospholipase C beta 1
5.336.115	-0.1383911	ASH1L	ASH1 like histone lysine methyltransferase
5.336.109	-0.1740243	CHRNB1	cholinergic receptor nicotinic beta 1 subunit
5.334.253	0.2698874	NUP50	nucleoporin 50
5.330.387	0.3930038	FXYD3	FXYD domain containing ion transport regulator
5.327.237	-0.1823273	DENND2A	DENN domain containing 2A
5.326.577	0.2168979	CLTB	clathrin light chain B
5.325.979	-0.1301895	SOCS7	suppressor of cytokine signaling 7
5.320.981	-0.161397	PNMA3	paraneoplastic Ma antigen 3
5.319.542	0.2760389	PTTG1IP	pituitary tumor-transforming 1 interacting prote
531.919	-0.109432	BMP10	bone morphogenetic protein 10
531.814	-0.0989705	MGC13053	uncharacterized MGC13053
5.316.816	-0.1111299		
5.315.567	-0.1411761	NUP214	nucleoporin 214
5.314.919	0.6687104	CYP17A1	cytochrome P450 family 17 subfamily A membe
5.313.632	0.2761586	HNRNPA3	heterogeneous nuclear ribonucleoprotein A3
5.310.036	0.1653941	PKMYT1	protein kinase, membrane associated tyrosine/
5.307.983	-0.1183324	KIAA1109	KIAA1109
5.302.096	-0.1464595	HNF4A	hepatocyte nuclear factor 4 alpha
5.298.221	0.170728	CLASP2	cytoplasmic linker associated protein 2

5.297.487	-0.1270703	TTC22	tetratricopeptide repeat domain 22
5.297.035	0.4039741	PLS1	plastin 1
5.296.589	0.1475261	CEP152	centrosomal protein 152
5.294.859	0.1489653	AGPAT4	1-acylglycerol-3-phosphate O-acyltransferase 4
5.291.858	-0.1167576	KCNJ4	potassium voltage-gated channel subfamily J m
5.291.462	0.4696387	NPL	N-acetylneuraminate pyruvate lyase
5.291.315	0.2352396	SENP2	SUMO1/sentrin/SMT3 specific peptidase 2
529.121	-0.1086988	PYGO1	pygopus family PHD finger 1
5.290.699	-0.175316	CD1E	CD1e molecule
5.289.465	0.1452904	TFIP11	tuftelin interacting protein 11
5.288.474	-0.1063088	EFS	embryonal Fyn-associated substrate
5.287.501	-0.2096704	CD72	CD72 molecule
5.283.612	-0.1566248	PLEKHS1	pleckstrin homology domain containing S1
5.279.503	-0.1424886	LDLR	low density lipoprotein receptor
5.272.582	0.198746	CELF1	CUGBP, Elav-like family member 1
5.272.499	-0.123302	PSD	pleckstrin and Sec7 domain containing
5.268.952	-0.1227051	PRRG3	proline rich and Gla domain 3
5.265.217	0.2694739	MARCKS	myristoylated alanine rich protein kinase C sub
5.262.573	-0.1340923	GK	glycerol kinase
5.261.151	0.2380532	NUDT4P1//	nudix hydrolase 4 pseudogene 1///nudix hydrol
5.255.512	-0.5429993	CCND1	cyclin D1
5.254.088	-0.0998935	IFNA21	interferon alpha 21
5.252.924	-0.2768993	TSC2	tuberous sclerosis 2
5.242.287	-0.115967	KCNA3	potassium voltage-gated channel subfamily A m
5.236.402	0.1439273	NDUFB6	NADH:ubiquinone oxidoreductase subunit B6
5.235.881	0.2816846	EBAG9	estrogen receptor binding site associated, antig
5.230.494	-0.1198057	SRC	SRC proto-oncogene, non-receptor tyrosine kin
5.223.763	0.2089611	SYF2	SYF2 pre-mRNA splicing factor
5.216.429	-0.1478401	DOK1	docking protein 1
5.216.059	-0.1366562	VENTXP1	VENT homeobox pseudogene 1
5.215.859	0.2071421	RNF103	ring finger protein 103
5.215.259	-0.3275787	POLR2C	RNA polymerase II subunit C
521.206	-0.1461171	NEURL1	neuralized E3 ubiquitin protein ligase 1
5.210.569	-0.1144911	RPS2P45	ribosomal protein S2 pseudogene 45
5.206.817	-0.15565	CALHM2	calcium homeostasis modulator 2
5.206.709	-0.1544794	FEZF2	FEZ family zinc finger 2
5.203.836	0.177138	SMG1P1///:	SMG1P1, nonsense mediated mRNA decay ass
5.202.423	-0.1797773	PSG1	pregnancy specific beta-1-glycoprotein 1
5.195.576	0.1824801	JRK	Jrk helix-turn-helix protein
5.194.644	-0.2543977	C14orf99	uncharacterized LOC317730
5.193.406	-0.1138227	MED26	mediator complex subunit 26
5.191.536	-0.1328182	NFATC4	nuclear factor of activated T-cells 4
5.190.494	-0.3219299	MPHOSPH8	M-phase phosphoprotein 8
5.187.282	-0.1413205	GRIK4	glutamate ionotropic receptor kainate type sub

5.180.989	-0.120774	CALCR	calcitonin receptor
5.179.974	-0.1170562	CORT	cortistatin
5.176.033	0.1935146	NOV	nephroblastoma overexpressed
5.173.254	0.2251038	FAM110B	family with sequence similarity 110 member B
5.168.931	-0.1795276	SH3GL3	SH3 domain containing GRB2 like 3, endophilin
5.168.031	-0.162551	LDLRAP1	low density lipoprotein receptor adaptor protein
5.167.348	-0.1279433	MTHFR	methylenetetrahydrofolate reductase (NAD(P)H
5.164.665	0.3441712	TCIRG1	T-cell immune regulator 1, ATPase H+ transport
5.161.564	-0.1034653		
516.095	0.2207212	SCAMP5	secretory carrier membrane protein 5
515.402	-0.1497758	MYO7A	myosin VIIA
5.151.794	0.1633701	ENTPD1	ectonucleoside triphosphate diphosphohydrola
5.148.376	-0.1200566	WNT8B	Wnt family member 8B
5.147.663	-0.1171935	C5orf45	chromosome 5 open reading frame 45
5.146.281	-0.3898475	SDC1	syndecan 1
5.140.026	-0.106661	TAF1	TATA-box binding protein associated factor 1
5.138.938	-0.1282505	FNDC8	fibronectin type III domain containing 8
5.138.682	0.2874508	CDV3	CDV3 homolog
5.138.535	0.367516	SMAD4	SMAD family member 4
513.584	-0.1629478	HOXC8	homeobox C8
5.135.277	-0.1620997		
5.134.848	0.1799893	LOC39113	similar to hCG2041276
5.133.774	-0.1207526	ARHGEF4	Rho guanine nucleotide exchange factor 4
5.130.986	0.1244521	EEA1	early endosome antigen 1
5.130.662	-0.102598	TNFRSF10C	TNF receptor superfamily member 10c
5.125.203	-0.1011737	ADCYAP1R	ADCYAP receptor type I
5.123.914	-0.1201708	RSRP1	arginine and serine rich protein 1
5.122.658	-0.3656908	MAPRE2	microtubule associated protein RP/EB family m
5.118.386	0.4482118	CADM1	cell adhesion molecule 1
5.116.277	0.1965685	RNF141	ring finger protein 141
5.111.065	-0.0991107	HS3ST1	heparan sulfate-glucosamine 3-sulfotransferas
5.108.393	0.1717437	DDX17	DEAD-box helicase 17
5.108.226	0.3342924	CUL4A	cullin 4A
510.812	-0.1228905	CCL7	C-C motif chemokine ligand 7
5.105.711	-0.1295401	BORCS8-M	BORCS8-MEF2B readthrough
5.105.287	-0.1134439	NRL	neural retina leucine zipper
5.104.651	-0.3634147		
5.104.502	-0.1181128	LRRC37BP	leucine rich repeat containing 37B pseudogene
5.103.827	0.3584615	MAMLD1	mastermind like domain containing 1
5.102.521	0.1256759	PHTF2	putative homeodomain transcription factor 2
5.101.417	-0.2475077	XYLB	xylulokinase
5.096.071	-0.1171902	BMP3	bone morphogenetic protein 3
5.095.849	0.1301201		
5.091.689	0.2207318	EIF2S2	eukaryotic translation initiation factor 2 subunit

509.012	0.1578984	AMBRA1	autophagy and beclin 1 regulator 1
5.086.891	0.3481559	C6orf120	chromosome 6 open reading frame 120
5.085.536	0.1757076	GIN1///DSC	gypsy retrotransposon integrase 1///desmoglei
5.085.282	-0.1358269	REG1CP	regenerating family member 1 gamma, pseudo
5.083.133	-0.1072007	KLHL11	kelch like family member 11
5.082.172	-0.4902129	MOCOS///F	molybdenum cofactor sulfurase///parathyroid
5.078.069	-0.148107	C21orf2	chromosome 21 open reading frame 2
5.077.193	0.1115057	SAPCD1///I	suppressor APC domain containing 1///mutS h
5.068.889	0.4105463	RNASE1	ribonuclease A family member 1, pancreatic
5.062.229	-0.1528891	HIST1H2BN	histone cluster 1, H2bn
5.061.341	-0.1451635	GPR162	G protein-coupled receptor 162
5.059.314	-0.1147468	IGL	immunoglobulin lambda locus
5.057.795	-0.2907786	GPC1	glypican 1
50.575	-0.1114724	PRDM8	PR/SET domain 8
5.057.069	-0.1506001	IL2RA	interleukin 2 receptor subunit alpha
5.056.623	-0.1417139	CYP11B2	cytochrome P450 family 11 subfamily B memb
5.054.621	-0.1068229	PKM	pyruvate kinase, muscle
5.053.602	-0.121033	GOT2P1	glutamic-oxaloacetic transaminase 2 pseudog
5.052.169	-0.1443944	LHX1	LIM homeobox 1
5.052.159	0.2351952	RPL10P2	ribosomal protein L10 pseudogene 2
5.048.795	-0.1209797	KIR3DX1	killer cell immunoglobulin like receptor, three I
5.047.247	-0.521556	IFI44L	interferon induced protein 44 like
5.045.178	0.3848253	WEE1	WEE1 G2 checkpoint kinase
5.036.895	-0.2033283	TGOLN2	trans-golgi network protein 2
5.034.569	-0.3370931	CA14	carbonic anhydrase 14
5.032.404	0.2120061	NDUFB2	NADH:ubiquinone oxidoreductase subunit B2
5.032.108	0.3695234		
5.030.859	-0.1252342		
5.025.948	-0.1591875	HHLA1	HERV-H LTR-associating 1
5.021.716	-0.2959043	FGFR1	fibroblast growth factor receptor 1
5.016.149	-0.2082372	ALPK1	alpha kinase 1
5.012.323	-0.1036942	TRIO	trio Rho guanine nucleotide exchange factor
5.011.995	-0.3532387	LAMP2	lysosomal associated membrane protein 2
5.006.845	0.2342828	LOC64357(	phosphoglycerate mutase 1 (brain) pseudogen
4.997.101	-0.1268973	GML	glycosylphosphatidylinositol anchored molecu
4.990.267	-0.3435986	OLFML1	olfactomedin like 1
4.988.275	-0.1315983	PDIA2	protein disulfide isomerase family A member 2
4.985.319	-0.1787646	RNF167	ring finger protein 167
4.983.471	-0.1391449	SKIL	SKI-like proto-oncogene
4.981.722	-0.1508912	PPP3CB	protein phosphatase 3 catalytic subunit beta
4.981.422	-0.0759639	ZNF695	zinc finger protein 695
4.976.368	-0.3577325	NFIB	nuclear factor I B
4.976.006	-0.1018383	MGC4771	hypothetical LOC84754
497.595	0.1112499	KIF3A	kinesin family member 3A

4.972.181	0.1970219	DTNA	dystrobrevin alpha
4.969.076	0.2631852	ARIH1	ariadne RBR E3 ubiquitin protein ligase 1
4.967.001	0.5365675	NREP	neuronal regeneration related protein
4.966.026	0.242526	INPP5A	inositol polyphosphate-5-phosphatase A
4.963.825	-0.2297813	KIF1C	kinesin family member 1C
4.959.945	0.3008369	VPS13A	vacuolar protein sorting 13 homolog A
4.954.272	-0.1046181	ASMTL-AS1	ASMTL antisense RNA 1
4.948.499	-0.1055171		
4.944.467	-0.0998606	TGM4	transglutaminase 4
4.941.761	-0.3283922	ALG13	ALG13, UDP-N-acetylglucosaminyltransferase
4.935.373	0.200044	PEX7	peroxisomal biogenesis factor 7
49.316	-0.1641056	NPY	neuropeptide Y
493.037	-0.2516815	LOC73106(	similar to PHD finger protein 8
4.926.184	-0.2942193	HOOK1	hook microtubule tethering protein 1
4.925.658	-0.1230959	NKX6-1	NK6 homeobox 1
4.920.233	0.2531625	SUMO1	small ubiquitin-like modifier 1
4.919.833	-0.1478871	AGO4	argonaute 4, RISC catalytic component
4.918.899	0.2026126	MED13L	mediator complex subunit 13 like
4.917.594	-0.1152078	KMT2D	lysine methyltransferase 2D
4.916.485	-0.1206829	SAA3P	serum amyloid A3 pseudogene
4.915.503	-0.1747756	IFNAR1	interferon alpha and beta receptor subunit 1
4.915.154	0.3922356	RGS10	regulator of G-protein signaling 10
4.910.942	-0.1059775		
4.909.967	-0.112059	ZCCHC14	zinc finger CCHC-type containing 14
4.900.809	-0.0933818	TACR3	tachykinin receptor 3
490.002	-0.1135604	DOK3	docking protein 3
4.891.074	0.1755896	CIDEC	cell death inducing DFFA like effector c
4.888.623	0.4347102	CTAG1A///(	cancer/testis antigen 1A///cancer/testis antiger
4.886.169	-0.1472336	SPATA31C1	SPATA31 subfamily C member 1
4.881.393	0.3463966	DDR1	discoidin domain receptor tyrosine kinase 1
4.881.164	0.3976494	TIMP3	TIMP metallopeptidase inhibitor 3
4.880.152	0.1429046	BRCA2	BRCA2, DNA repair associated
4.876.095	-0.1364886	ESRRA	estrogen related receptor alpha
4.873.371	-0.1654234	OR5T2///RI	olfactory receptor family 5 subfamily T member
4.872.158	-0.1133099	GJA3	gap junction protein alpha 3
4.870.717	-0.2095801	KPNA1	karyopherin subunit alpha 1
4.864.288	0.1996373	ZDHHC18	zinc finger DHHC-type containing 18
4.863.334	-0.2171389	NFATC1	nuclear factor of activated T-cells 1
4.862.997	0.2206986	GAPVD1	GTPase activating protein and VPS9 domains 1
4.862.526	0.1440467	ODF2	outer dense fiber of sperm tails 2
4.862.383	-0.1337501	NUDCD3	NudC domain containing 3
4.860.016	-0.1244251	RGS14	regulator of G-protein signaling 14
485.832	-0.4791157	IGHM	immunoglobulin heavy constant mu
4.856.037	-0.2048732	BMP1	bone morphogenetic protein 1

4.855.095	0.1420427	CNOT9	CCR4-NOT transcription complex subunit 9
4.853.572	0.3555001	PTPN11	protein tyrosine phosphatase, non-receptor typ
4.852.566	-0.1325797	SPOCK2	sparc/osteonectin, cwcv and kazal-like domain
4.851.798	0.2539825	MBD2	methyl-CpG binding domain protein 2
4.848.964	-0.1484345	CEBPD	CCAAT/enhancer binding protein delta
4.846.994	0.2031485	NGLY1	N-glycanase 1
4.840.885	-0.0955749	CDSN	corneodesmosin
4.838.899	-0.1301784	GAS8-AS1	GAS8 antisense RNA 1
4.837.247	-0.1784446	NXPH3	neurexophilin 3
4.833.988	-0.1023884	KIDINS220	kinase D-interacting substrate 220kDa
4.833.828	0.5241198	S100A11	S100 calcium binding protein A11
4.829.686	0.4288981	KLF5	Kruppel like factor 5
4.828.256	-0.3156907	SOD3	superoxide dismutase 3, extracellular
4.826.107	0.2348565	LOC64445(	uncharacterized LOC644450
4.825.454	-0.3579552	STAT3	signal transducer and activator of transcription
4.822.275	-0.1084859	ZFYVE9	zinc finger FYVE-type containing 9
4.820.403	-0.1472869	INHBA	inhibin beta A subunit
4.818.624	-0.1056831	OCM2	oncomodulin 2
4.816.431	-0.0836002	RARS2	arginyl-tRNA synthetase 2, mitochondrial
4.812.378	-0.1569272	TGFB1	transforming growth factor beta 1
4.810.172	-0.1559664	CADM3	cell adhesion molecule 3
480.307	-0.1184608	ACRV1	acrosomal vesicle protein 1
4.801.667	-0.0920608	NFATC1	nuclear factor of activated T-cells 1
4.798.814	-0.1473033	CA5B	carbonic anhydrase 5B
4.793.668	0.2443996	RNF10	ring finger protein 10
4.793.474	-0.1303895	CCDC87	coiled-coil domain containing 87
4.792.987	-0.226574	FAM107A	family with sequence similarity 107 member A
4.787.573	-0.0996791	ZXDB	zinc finger, X-linked, duplicated B
4.786.735	0.2181105	NDUFA2	NADH:ubiquinone oxidoreductase subunit A2
4.784.287	0.5629371	GLUL	glutamate-ammonia ligase
4.783.462	0.2153151	TYRO3	TYRO3 protein tyrosine kinase
4.779.181	0.2515852	ILK	integrin linked kinase
4.778.227	-0.1515441	ADCY2	adenylate cyclase 2
477.599	-0.1175919	LOC55338	uncharacterized LOC55338
4.775.526	-0.1644802	PRKAR1AP:	protein kinase cAMP-dependent type I regulator
4.766.781	-0.2103817	CLINT1	clathrin interactor 1
4.761.989	0.1645686	MARS	methionyl-tRNA synthetase
4.759.433	0.3167261	CYP26B1	cytochrome P450 family 26 subfamily B membe
4.759.055	-0.1247128	GYPB	glycophorin B (MNS blood group)
4.755.228	-0.1091899	RCVRN	recoverin
4.753.524	-0.0996096	CACNA1S	calcium voltage-gated channel subunit alpha1
4.752.257	-0.1039866	CCR8	C-C motif chemokine receptor 8
4.752.247	-0.1255301	CACNB2	calcium voltage-gated channel auxiliary subuni
4.751.872	-0.0862067	CDX4	caudal type homeobox 4

4.751.137	-0.2026153		
4.747.049	-0.1067083	PMP2	peripheral myelin protein 2
4.740.723	-0.5266358	PTPRC	protein tyrosine phosphatase, receptor type C
4.738.478	-0.1047516		
4.734.135	-0.2429931	DNAJC16	DnaJ heat shock protein family (Hsp40) membe
4.731.905	-0.1134142	HAPLN2	hyaluronan and proteoglycan link protein 2
4.729.948	-0.1325653	C17orf53	chromosome 17 open reading frame 53
4.728.778	0.2641566	GNB2	G protein subunit beta 2
4.726.453	-0.3013878	LARP4	La ribonucleoprotein domain family member 4
4.721.518	-0.1671561	VIPR2	vasoactive intestinal peptide receptor 2
472.087	-0.3502614	GJB1	gap junction protein beta 1
4.718.239	-0.1127272	GDF11	growth differentiation factor 11
4.710.439	-0.1300181	PAX3	paired box 3
4.709.443	-0.1642248	FAM214B	family with sequence similarity 214 member B
4.703.361	0.2031185	UBE4A	ubiquitination factor E4A
4.699.929	-0.1066654	DNAH9	dynein axonemal heavy chain 9
4.699.666	0.3257191		
4.699.492	0.2519218	TMEM87A	transmembrane protein 87A
4.699.082	-0.1175641	COL13A1	collagen type XIII alpha 1 chain
4.695.775	0.1762532	TRAFD1	TRAF-type zinc finger domain containing 1
4.693.978	-0.3529739		
4.693.592	0.3100362	PSENEN	presenilin enhancer gamma-secretase subunit
4.691.932	-0.1722597	NODAL	nodal growth differentiation factor
4.689.602	0.1997746	TSPAN15	tetraspanin 15
4.687.034	0.2777332	ITPK1	inositol-tetrakisphosphate 1-kinase
4.686.802	-0.3034994	CLINT1	clathrin interactor 1
4.685.508	-0.351943	SLC16A1	solute carrier family 16 member 1
4.684.995	-0.1080149		
4.681.071	-0.1264759	KLK3	kallikrein related peptidase 3
4.676.767	-0.1374878	PDE3A	phosphodiesterase 3A
4.676.591	-0.1722778	PTAFR	platelet activating factor receptor
467.216	-0.1327149	YY2	YY2 transcription factor
4.671.819	0.1695428	FAM53B	family with sequence similarity 53 member B
4.668.864	-0.1850902	PDE4B	phosphodiesterase 4B
4.660.266	-0.3147292	PCDHGA1/	protocadherin gamma subfamily A, 1///protoca
4.659.976	0.380538	CALR	calreticulin
4.654.648	0.2142568	RAB11FIP5	RAB11 family interacting protein 5
4.654.206	-0.1324065	TEF	TEF, PAR bZIP transcription factor
4.652.127	-0.1300182	FOLH1	folate hydrolase (prostate-specific membrane a
4.650.608	-0.146053	TBX21	T-box 21
4.650.486	-0.2566326	KIAA0430	KIAA0430
4.650.456	-0.1679321	AVPR1A	arginine vasopressin receptor 1A
4.648.513	-0.2780912	IFIT2	interferon induced protein with tetratricopeptid
4.645.878	-0.1036838	ADGRB1	adhesion G protein-coupled receptor B1

4.645.004	-0.1565572	ADAMTS6	ADAM metallopeptidase with thrombospondin t
4.643.762	-0.5141196	UGT2B4	UDP glucuronosyltransferase family 2 member
4.642.763	-0.1789575	MAP4K1	mitogen-activated protein kinase kinase kinase
464.101	-0.7625961		
4.635.486	0.3068833	RBM8A	RNA binding motif protein 8A
4.635.133	0.3358197	GNB1	G protein subunit beta 1
463.496	-0.1836495	GNB3	G protein subunit beta 3
46.308	-0.1392853	FLJ14031	hypothetical protein FLJ14031
4.629.891	-0.0829288	LOC64574!	zinc finger protein 131 pseudogene
4.626.811	-0.126572	SOX21	SRY-box 21
4.623.383	-0.2211676	MYO1B	myosin IB
4.612.685	-0.3934363	ELOVL6	ELOVL fatty acid elongase 6
4.609.537	-0.1236567		
4.605.027	-0.1221527	GRIN1	glutamate ionotropic receptor NMDA type subu
4.603.573	-0.1144601	TAS2R10	taste 2 receptor member 10
459.967	-0.1589986	GAN///ITPR	gigaxonin///inositol 1,4,5-trisphosphate recept
4.598.255	-0.3190923	CRK	CRK proto-oncogene, adaptor protein
4.597.718	-0.1635849	AGRN	agrin
4.596.731	0.1789003	NFE2L3	nuclear factor, erythroid 2 like 3
4.595.767	-0.1688138	LINC00599	long intergenic non-protein coding RNA 599
4.593.201	-0.1297888	BLK	BLK proto-oncogene, Src family tyrosine kinase
4.592.968	-0.4746111	ERLIN1	ER lipid raft associated 1
4.592.581	0.4520763	PLA2G4C	phospholipase A2 group IVC
4.590.698	-0.186496	BMP1	bone morphogenetic protein 1
457.994	-0.2103451	GATB	glutamyl-tRNA amidotransferase subunit B
4.578.953	-0.3543453	SEC24D	SEC24 homolog D, COPII coat complex compo
4.572.624	0.177253	ARPP19	cAMP regulated phosphoprotein 19
4.570.161	-0.1437195	TAF1	TATA-box binding protein associated factor 1
4.568.811	0.2666707	SLC2A5	solute carrier family 2 member 5
456.733	-0.2311775	CTNND1	catenin delta 1
456.615	-0.1013132	MLXIP	MLX interacting protein
4.564.897	-0.1694605	RMND5B	required for meiotic nuclear division 5 homolog
4.564.611	0.2099763	ATXN2	ataxin 2
4.560.262	-0.099748	CHRNA3	cholinergic receptor nicotinic alpha 3 subunit
4.557.547	-0.1300408	ARHGAP1	Rho GTPase activating protein 1
4.556.318	-0.1689109	PLD2	phospholipase D2
4.554.331	-0.1196465	ARHGEF38	Rho guanine nucleotide exchange factor 38
455.242	0.3098351	FGFR4	fibroblast growth factor receptor 4
4.551.766	-0.0966014	FLJ13224	uncharacterized LOC79857
4.544.281	0.171853	DCAF15	DDB1 and CUL4 associated factor 15
4.538.733	-0.120707	LOC10192	uncharacterized LOC101927610
4.538.114	0.1050346	LINS1	lines homolog 1
4.537.042	-0.169551	MYT1L///JA	myelin transcription factor 1 like///janus kinase
4.534.536	-0.1254654	PCDH9	protocadherin 9

453.282	0.4893012	COL4A5	collagen type IV alpha 5 chain
4.532.293	-0.1010745	FAM49B	family with sequence similarity 49 member B
4.528.316	0.2741249	MPPE1	metallophosphoesterase 1
4.527.313	-0.2852015	GSTK1	glutathione S-transferase kappa 1
4.524.896	0.2085137	IQCE	IQ motif containing E
451.988	-0.4387982	CTBP2	C-terminal binding protein 2
4.519.436	-0.1262145	MGAT3	mannosyl (beta-1,4-)-glycoprotein beta-1,4-N-a
4.517.995	0.2661416	RPS6	ribosomal protein S6
4.515.446	0.3122642	TUBB4A	tubulin beta 4A class IVa
4.514.574	0.2195221	ZFAND6	zinc finger AN1-type containing 6
4.508.936	-0.3634276	HDHD3	haloacid dehalogenase like hydrolase domain c
4.506.785	-0.1136503	TGM1	transglutaminase 1
4.501.796	-0.2154004	CDKN2AIP	CDKN2A interacting protein
449.857	0.2411992	VWA5A	von Willebrand factor A domain containing 5A
4.496.163	-0.136328	SHCBP1L	SHC binding and spindle associated 1 like
4.495.492	-0.1463383	UTY	ubiquitously transcribed tetratricopeptide repe
449.511	-0.1097928	KRT24	keratin 24
4.490.005	-0.1205006	DNAJB5	DnaJ heat shock protein family (Hsp40) membe
4.480.294	0.2091734	GAD1	glutamate decarboxylase 1
4.478.387	0.339283	HOXC6	homeobox C6
4.476.438	0.3688375	TIMP2	TIMP metallopeptidase inhibitor 2
4.476.337	0.169991		
4.474.163	0.3901035	LSS	lanosterol synthase (2,3-oxidosqualene-lanost
4.473.404	-0.110083	FSCN3	fascin actin-bundling protein 3
4.470.925	-0.1264951	GJC2	gap junction protein gamma 2
4.465.996	0.3322227	BBX	BBX, HMG-box containing
4.465.319	-0.1288862	HLA-C	major histocompatibility complex, class I, C
4.463.802	-0.1228409	METTL10	methyltransferase like 10
4.463.596	-0.1073704	MATN1	matrilin 1, cartilage matrix protein
4.463.357	-0.1824803	PSTPIP1	proline-serine-threonine phosphatase interacti
446.178	0.3305362	RAB32	RAB32, member RAS oncogene family
4.461.544	-0.4522221	ZFAND5	zinc finger AN1-type containing 5
4.458.552	-0.2222953	SLC25A22	solute carrier family 25 member 22
4.455.248	0.2330359	SETMAR	SET domain and mariner transposase fusion ge
4.453.853	-0.1031105	PRPF31	pre-mRNA processing factor 31
4.450.337	-0.1024597	MAPRE3	microtubule associated protein RP/EB family m
4.446.508	-0.1451495	GNRH2	gonadotropin releasing hormone 2
4.445.297	-0.1062047	ACRV1	acrosomal vesicle protein 1
4.444.489	-0.1414763	ERG	ERG, ETS transcription factor
4.442.333	-0.1017668	KCNK7	potassium two pore domain channel subfamily
4.441.869	-0.0942302	SH2D1A	SH2 domain containing 1A
4.440.816	-0.1425992	P2RX1	purinergic receptor P2X 1
444.032	-0.1986667	ZFHX3	zinc finger homeobox 3
4.434.825	-0.2889647	CFLAR	CASP8 and FADD like apoptosis regulator

4.424.839	0.347247	SMARCA1	SWI/SNF related, matrix associated, actin depe
4.420.263	0.1679247	ERP44	endoplasmic reticulum protein 44
4.419.566	0.2085306	MAK16	MAK16 homolog
4.417.167	-0.1643879	ZNF500	zinc finger protein 500
4.416.523	-0.0969394	GPR161	G protein-coupled receptor 161
4.414.515	-0.1242413	NCOR2	nuclear receptor corepressor 2
4.413.508	-0.1201975	IL5RA	interleukin 5 receptor subunit alpha
4.410.696	0.2333317	PEX1	peroxisomal biogenesis factor 1
4.408.825	-0.0902945	LINC00461	long intergenic non-protein coding RNA 461
4.405.419	-0.120418	ULBP1	UL16 binding protein 1
4.402.954	-0.2894642	MRPS31	mitochondrial ribosomal protein S31
4.399.369	-0.1164034	KCNA4	potassium voltage-gated channel subfamily A m
4.399.128	0.2351874	GPR37	G protein-coupled receptor 37
4.398.718	-0.101774	PGLYRP1	peptidoglycan recognition protein 1
4.396.863	0.1253474	POLR2L	RNA polymerase II subunit L
4.396.343	0.334294	TCF7L1	transcription factor 7 like 1
4.395.543	0.3480888	TCAF1	TRPM8 channel associated factor 1
4.392.195	-0.119477	EFNA2	ephrin A2
438.502	0.1605306	ZNF230	zinc finger protein 230
4.384.556	0.278253	ANKHD1-EI	ANKHD1-EIF4EBP3 readthrough///ankyrin repea
4.380.888	0.2356712	CHN1	chimerin 1
437.768	-0.259854	PKIG	protein kinase (cAMP-dependent, catalytic) inh
4.376.083	0.2484929	UQCRFS1	ubiquinol-cytochrome c reductase, Rieske iron-
4.374.613	0.3020515	LGR5	leucine rich repeat containing G protein-couple
437.453	-0.1239269	CRHR2	corticotropin releasing hormone receptor 2
4.374.293	-0.1253211	OXCT2	3-oxoacid CoA-transferase 2
4.368.113	-0.2388792	CCR5	C-C motif chemokine receptor 5 (gene/pseudog
4.366.347	-0.1348439	FAM189A2	family with sequence similarity 189 member A2
4.362.201	-0.216274	ARAP1	ArfGAP with RhoGAP domain, ankyrin repeat an
4.361.477	-0.3282467	SLA	Src-like-adaptor
4.360.733	0.1746904	MTMR2	myotubularin related protein 2
435.984	-0.1080036	RHNO1///IC	RAD9-HUS1-RAD1 interacting nuclear orphan 1
4.351.666	-0.1842878	ENDOD1	endonuclease domain containing 1
4.347.926	-0.2456636	TMEM134	transmembrane protein 134
4.344.498	0.234061	UPF1	UPF1, RNA helicase and ATPase
4.344.077	-0.0921701	AMACR	alpha-methylacyl-CoA racemase
4.342.638	-0.1629779	MYOD1	myogenic differentiation 1
4.342.412	0.246282	CALCRL	calcitonin receptor like receptor
4.337.613	0.1450815	MTMR1	myotubularin related protein 1
4.337.195	0.2199974	SCAMP2	secretory carrier membrane protein 2
4.335.948	-0.1644947	EDA	ectodysplasin A
4.334.305	-0.1591726	SEZ6L	seizure related 6 homolog like
4.333.581	-0.3767007	CLN5	ceroid-lipofuscinosis, neuronal 5
4.328.832	0.3617938	ADGRG1	adhesion G protein-coupled receptor G1

4.326.103	0.1426398	ZC3HAV1	zinc finger CCCH-type containing, antiviral 1
4.324.336	-0.1252794	DOCK10	dedicator of cytokinesis 10
4.316.103	-0.2922724	MTCP1	mature T-cell proliferation 1
4.311.649	-0.1145075	FRMD1	FERM domain containing 1
4.311.308	-0.2395858	SIVA1	SIVA1 apoptosis inducing factor
4.307.793	-0.1187346	MLLT1	MLLT1, super elongation complex subunit
4.306.665	-0.4436544	AK4	adenylate kinase 4
4.304.248	-0.3095678	CCNI	cyclin I
4.304.069	-0.1194435	P2RX2	purinergic receptor P2X 2
4.303.458	-0.1339832	OR2F1	olfactory receptor family 2 subfamily F member
4.297.909	0.1263659	PPM1E	protein phosphatase, Mg2+/Mn2+ dependent 1
4.296.011	-0.1048266	AKAP7	A-kinase anchoring protein 7
4.295.414	-0.1444002	PDX1	pancreatic and duodenal homeobox 1
4.295.139	-0.1191931	CACNA1C	calcium voltage-gated channel subunit alpha1
4.295.022	-0.1171605	SLC28A2	solute carrier family 28 member 2
4.291.754	0.1194689	ZNF557	zinc finger protein 557
4.291.558	0.1797572	HNRNPH3	heterogeneous nuclear ribonucleoprotein H3
4.291.006	-0.1435032	APPBP2	amyloid beta precursor protein binding protein
4.290.966	-0.1738917	SZT2	seizure threshold 2 homolog (mouse)
4.287.821	-0.1449927	LAIR1	leukocyte associated immunoglobulin like rece
4.284.451	0.3268587	GALNT2	polypeptide N-acetylgalactosaminyltransferase
4.284.033	-0.1532251	HSPB7	heat shock protein family B (small) member 7
428.084	0.1341414	B3GAT3	beta-1,3-glucuronyltransferase 3
4.279.017	-0.1236411	FOXN3-AS2	FOXN3 antisense RNA 2
4.275.984	0.2018799	AFTPH	aftiphilin
4.275.434	-0.6794648		
4.272.394	-0.1868413	SAMD4A	sterile alpha motif domain containing 4A
4.271.429	-0.2007375	SERPINA1	serpin family A member 1
4.262.233	-0.1126294	GBX2	gastrulation brain homeobox 2
4.258.006	-0.2713976	KLHL24	kelch like family member 24
4.256.236	0.2895649	YLPM1	YLP motif containing 1
4.254.154	-0.1927555	TMPRSS4	transmembrane protease, serine 4
4.241.785	-0.1139197		
4.241.742	-0.1208933	OR10J1	olfactory receptor family 10 subfamily J membe
4.240.859	-0.2977183	IDS	iduronate 2-sulfatase
4.240.538	-0.109638	TSPAN4	tetraspanin 4
4.237.916	0.1600178	FANCE	Fanconi anemia complementation group E
4.232.743	0.5352719	EMP1	epithelial membrane protein 1
4.228.323	-0.1530529	GIPR	gastric inhibitory polypeptide receptor
4.227.532	-0.3501726	BCL3	B-cell CLL/lymphoma 3
422.327	0.1429538	ADA	adenosine deaminase
4.221.709	-0.1758624	MROH7	maestro heat like repeat family member 7
421.826	-0.105693	TRIM2	tripartite motif containing 2
4.211.941	0.2572721	SKP1	S-phase kinase-associated protein 1

4.211.913	-0.1555649	SNCA	synuclein alpha
4.208.257	0.2166691	PTPN20	protein tyrosine phosphatase, non-receptor typ
4.207.283	-0.1350302	CYTH1	cytohesin 1
4.205.848	-0.1340762	IL17RC	interleukin 17 receptor C
4.203.054	-0.1365955	TCF20	transcription factor 20
4.203.053	-0.1153783	SVEP1	sushi, von Willebrand factor type A, EGF and pe
4.192.025	-0.1337056	IQSEC3	IQ motif and Sec7 domain 3
4.186.857	-0.1582608	HDAC7	histone deacetylase 7
4.179.495	0.2419035	HPCAL1	hippocalcin like 1
4.178.852	-0.1615787	MYOG	myogenin (myogenic factor 4)
4.178.762	-0.1872842	PGAP3	post-GPI attachment to proteins 3
417.769	-0.0993294	PTPN21	protein tyrosine phosphatase, non-receptor typ
4.175.624	-0.1221102	TACR1	tachykinin receptor 1
4.175.321	-0.1602945	HABP4	hyaluronan binding protein 4
4.175.073	0.2531661	GGA1	golgi associated, gamma adaptin ear containin
4.173.658	0.2719938	DNAJC7	DnaJ heat shock protein family (Hsp40) membe
4.169.851	0.234839	LOC44089	two pore channel 3 pseudogene
4.169.369	-0.1466617	CLEC11A	C-type lectin domain family 11 member A
41.693	0.2016126	NSUN5P2	NOP2/Sun RNA methyltransferase family meml
4.165.908	-0.1795706	GPR18	G protein-coupled receptor 18
4.162.513	-0.1384606	SLC7A1	solute carrier family 7 member 1
41.599	-0.1377947	SMG6	SMG6, nonsense mediated mRNA decay factor
4.157.237	0.2999111	ELMO1	engulfment and cell motility 1
4.151.969	-0.2514809	RSRP1	arginine and serine rich protein 1
4.151.803	0.1108621	CHML	CHM like, Rab escort protein 2
4.147.702	0.1032417	LRRC8B	leucine rich repeat containing 8 family member
4.145.338	-0.0935619	ZNF79	zinc finger protein 79
4.143.328	0.0984091	PCIF1	PDX1 C-terminal inhibiting factor 1
4.141.849	0.1974354	PDZD2	PDZ domain containing 2
413.802	0.1727362	ARHGAP33	Rho GTPase activating protein 33
4.133.476	-0.1005209	GRK1	G protein-coupled receptor kinase 1
4.133.292	0.241871	PSME2	proteasome activator subunit 2
4.133.047	-0.0960964	TBCA	tubulin folding cofactor A
4.130.692	-0.1113397	NCLN	nicalin
4.130.077	0.2718447	KHSRP	KH-type splicing regulatory protein
4.127.013	-0.2891406	GPR183	G protein-coupled receptor 183
4.125.903	-0.1118706	USP22	ubiquitin specific peptidase 22
4.123.408	-0.3205746	CREM	cAMP responsive element modulator
412.273	-0.1286985	VWA7	von Willebrand factor A domain containing 7
4.121.682	0.2471575	SCAMP4	secretory carrier membrane protein 4
411.759	-0.5412711	HLA-DRB3/	major histocompatibility complex, class II, DR b
411.536	-0.1676011	RSRP1	arginine and serine rich protein 1
4.111.337	0.1984134	RPS25	ribosomal protein S25
4.109.883	-0.1204886		

4.109.793	-0.0876576	CYLC1	cylicin 1
410.737	-0.1733638	OGFR	opioid growth factor receptor
4.106.702	0.2620472	ZNF26	zinc finger protein 26
4.101.676	0.3260402	TMEM2	transmembrane protein 2
4.095.954	0.1805449	ATAT1	alpha tubulin acetyltransferase 1
4.089.139	-0.1133874	IL36A	interleukin 36, alpha
4.087.782	-0.2003233	URGCP	upregulator of cell proliferation
4.087.711	-0.1278829	DNAJC4	DnaJ heat shock protein family (Hsp40) membe
408.602	-0.1809959	MYO16	myosin XVI
4.085.425	0.1181305	PARD3	par-3 family cell polarity regulator
4.083.269	-0.1232654	PVT1	Pvt1 oncogene (non-protein coding)
4.075.207	-0.1760032	CYLD	CYLD lysine 63 deubiquitinase
4.070.372	0.190907	NOX4	NADPH oxidase 4
4.069.865	0.172149	DBN1	drebrin 1
4.068.729	0.2339244	ASCC1	activating signal cointegrator 1 complex subuni
4.065.929	-0.1787894	LRMP	lymphoid restricted membrane protein
4.064.901	-0.1751497	APOL2	apolipoprotein L2
4.060.455	-0.1429926	HGF	hepatocyte growth factor
4.058.854	0.3431678	CSPP1	centrosome and spindle pole associated protei
4.058.742	-0.1424132	SMARCA1	SWI/SNF related, matrix associated, actin depe
4.055.434	0.2507128	FUBP3	far upstream element binding protein 3
4.055.096	-0.4158615		
4.054.937	0.2270135	ATP6V0A1	ATPase H+ transporting V0 subunit a1
4.052.631	0.2634245	MPPE1	metallophosphoesterase 1
4.051.793	0.1997816	SLC1A5	solute carrier family 1 member 5
4.050.889	-0.3739269	ATP7B	ATPase copper transporting beta
4.047.866	-0.1323806	ACTL6B	actin like 6B
4.041.422	-0.0945953	OR2J2	olfactory receptor family 2 subfamily J member
4.041.005	-0.1337602	SARM1	sterile alpha and TIR motif containing 1
4.039.026	-0.4250428		
4.037.667	-0.1162537	RPRM	reprimo, TP53 dependent G2 arrest mediator ca
40.206	0.2186687	SPECC1L	sperm antigen with calponin homology and coil
4.018.641	-0.1275209	PRIM2	primase (DNA) subunit 2
4.017.409	0.1811344	MPPED2	metallophosphoesterase domain containing 2
4.016.008	-0.0986087	LYST	lysosomal trafficking regulator
4.011.626	-0.0947685	TGFA	transforming growth factor alpha
4.011.603	-0.1223063		
4.010.862	-0.1391288	PRX	periaxin
4.010.427	0.5674179	REG1A	regenerating family member 1 alpha
4.008.869	0.1716718	HMCES	5-hydroxymethylcytosine (hmC) binding, ES cel
4.007.659	0.2416928	LDLRAD4	low density lipoprotein receptor class A domair
4.006.928	-0.1268131	EVX1	even-skipped homeobox 1
4.006.439	-0.1249728	ZNF259P1	zinc finger protein 259 pseudogene 1
4.005.667	0.1007276	STX6	syntaxin 6

4.004.526	-0.1494713	CHRFAM7A	CHRNA7 (exons 5-10) and FAM7A (exons A-E) fu
4.003.921	-0.5859566	INSIG1	insulin induced gene 1
3.991.141	0.1917434	DIS3	DIS3 homolog, exosome endoribonuclease and
3.989.755	0.0952696	PASK	PAS domain containing serine/threonine kinase
39.845	0.2185375	COPS7A	COP9 signalosome subunit 7A
3.982.923	-0.1116794	IL4I1///NUF	interleukin 4 induced 1///nucleoporin 62
3.981.243	0.3380959	UBE2B	ubiquitin conjugating enzyme E2 B
3.979.703	-0.3394642		
3.976.795	0.3159924	NCOA3	nuclear receptor coactivator 3
3.967.674	0.190592	USP34	ubiquitin specific peptidase 34
3.966.532	0.1692545	TMEM186	transmembrane protein 186
3.963.609	0.2492742	DPM2	dolichyl-phosphate mannosyltransferase subur
3.961.904	0.182317	TBC1D30	TBC1 domain family member 30
396.078	-0.1310492	ADAM29	ADAM metallopeptidase domain 29
395.791	-0.1805616	PSMB1	proteasome subunit beta 1
3.956.344	-0.1391827	DOCK5	dedicator of cytokinesis 5
3.952.996	0.2243023	ARHGEF28	Rho guanine nucleotide exchange factor 28
395.293	-0.1315076	GP6	glycoprotein VI platelet
3.952.317	-0.1025863	ELF5	E74 like ETS transcription factor 5
3.949.006	-0.1522493	STS	steroid sulfatase (microsomal), isozyme S
3.945.852	-0.1215647	RERE	arginine-glutamic acid dipeptide repeats
3.944.514	-0.1219888	CREBBP	CREB binding protein
394.415	0.1374455	AGFG2	ArfGAP with FG repeats 2
3.943.004	0.3592797	HCP5	HLA complex P5 (non-protein coding)
3.942.389	-0.2367445		
3.941.579	0.2767848	OPA1	OPA1, mitochondrial dynamin like GTPase
3.941.082	0.1611337	ABI2	abl-interactor 2
3.932.933	-0.7460013	THBS1	thrombospondin 1
3.932.838	-0.4699459	FMO5	flavin containing monooxygenase 5
3.931.384	-0.1408261	SNCA	synuclein alpha
3.930.719	-0.2699002	PAFAH1B1	platelet activating factor acetylhydrolase 1b reg
3.929.796	-0.1276617	MOG	myelin oligodendrocyte glycoprotein
3.929.759	0.1586951	SMC4	structural maintenance of chromosomes 4
392.875	0.1618955	FAM64A	family with sequence similarity 64 member A
392.701	0.2223444	PDHB	pyruvate dehydrogenase (lipoamide) beta
3.925.492	0.293038	AASDHPPT	aminoadipate-semialdehyde dehydrogenase-p
3.924.271	-0.1137544	ELF5	E74 like ETS transcription factor 5
3.923.581	0.2216033	HOMER3	homer scaffolding protein 3
3.920.359	0.1709133	TAZ	tafazzin
3.918.536	-0.1047199		
3.917.714	-0.3740566	ZMAT3	zinc finger matrin-type 3
3.916.244	-0.2908291		
3.907.137	-0.1181936	GRIP1	glutamate receptor interacting protein 1
3.895.916	-0.1051972	RAB30	RAB30, member RAS oncogene family

3.895.354	-0.1409957	PBX2	PBX homeobox 2
3.893.001	0.3839757	PLPP1	phospholipid phosphatase 1
3.892.655	-0.2577849	PLXNB2	plexin B2
3.890.059	-0.2844092	CXCL8	C-X-C motif chemokine ligand 8
3.889.995	-0.3081088	ETFA	electron transfer flavoprotein alpha subunit
3.889.752	0.2616317	CSTF2T	cleavage stimulation factor subunit 2, tau varia
3.885.745	-0.1240281	ALPPL2	alkaline phosphatase, placental like 2
3.884.357	-0.1407546	IGL	immunoglobulin lambda locus
3.883.737	0.272777	FUT4	fucosyltransferase 4
3.882.835	-0.266267	HAUS2	HAUS augmin like complex subunit 2
3.882.302	-0.1042269	NTRK3	neurotrophic receptor tyrosine kinase 3
3.882.033	0.2798865	LPP	LIM domain containing preferred translocation
3.881.891	-0.2170169	MBTPS1	membrane bound transcription factor peptidas
3.877.139	-0.1541994	RUNX1	runt related transcription factor 1
3.876.358	0.2471734	PABPC4	poly(A) binding protein cytoplasmic 4
3.876.103	-0.1283038	CSF2RA	colony stimulating factor 2 receptor alpha subu
3.875.978	-0.1979283	CD27	CD27 molecule
3.872.358	0.2549923	GGCX	gamma-glutamyl carboxylase
3.871.237	-0.1732177	FAM65A	family with sequence similarity 65 member A
3.869.584	-0.1445485	CNPY4	canopy FGF signaling regulator 4
386.688	-0.100183	IGL	immunoglobulin lambda locus
3.863.746	0.2312015	UBE2H	ubiquitin conjugating enzyme E2 H
3.862.488	0.1530506	BIN1	bridging integrator 1
3.861.949	-0.1430407	LILRB4	leukocyte immunoglobulin like receptor B4
386.149	-0.2266779	ADARB1	adenosine deaminase, RNA specific B1
3.860.815	-0.2565427	C2CD2	C2 calcium dependent domain containing 2
3.860.254	-0.1656417	IGHG1	immunoglobulin heavy constant gamma 1 (G1r
3.858.846	0.1820413	PFDN5	prefoldin subunit 5
3.856.417	-0.1220907	ACKR4	atypical chemokine receptor 4
3.854.412	-0.1284501	ABCF2	ATP binding cassette subfamily F member 2
3.851.576	-0.1463037	SLC18A3	solute carrier family 18 member A3
3.850.352	-0.0998653	KIAA0509	uncharacterized LOC57242
3.847.602	0.4734688	TMED2	transmembrane p24 trafficking protein 2
3.842.283	-0.1069875	LOC65179:	hypothetical protein LOC651791///uncharacter
3.840.576	-0.1344561	L3MBTL1	l(3)mbt-like 1 (Drosophila)
3.826.385	0.1445819	XRCC4	X-ray repair cross complementing 4
3.825.105	-0.1222608	SEC14L1	SEC14 like lipid binding 1
3.823.881	-0.0960244	MAPK8IP3	mitogen-activated protein kinase 8 interacting p
3.823.232	-0.0913202	RAPSN	receptor associated protein of the synapse
3.823.003	-0.2879601	BNIP3	BCL2 interacting protein 3
3.822.333	-0.1917788	HSF4	heat shock transcription factor 4
3.822.006	-0.2114455	INVS	inversin
3.819.667	-0.2073734	GALNT2	polypeptide N-acetylgalactosaminyltransferase
3.816.224	-0.3789823		

381.339	-0.079608	CLDN8	claudin 8
3.809.915	-0.1313247	TNNT2	troponin T2, cardiac type
3.808.021	0.4120897	STAT1	signal transducer and activator of transcription
3.807.697	-0.1058171	SFTPC	surfactant protein C
3.803.777	-0.2543461	VWA5A	von Willebrand factor A domain containing 5A
3.803.742	-0.1427647	TRPC4	transient receptor potential cation channel sub
3.799.355	-0.1331463	NIN	ninein
3.796.477	0.2693048	ICMT	isoprenylcysteine carboxyl methyltransferase
3.795.142	-0.1411639	GJB3	gap junction protein beta 3
3.793.805	-0.1165919	BMP15	bone morphogenetic protein 15
3.793.456	0.0941923	GDAP1	ganglioside induced differentiation associated
379.344	-0.0858599	EZH1	enhancer of zeste 1 polycomb repressive comp
3.781.172	-0.1255729	KIF1C	kinesin family member 1C
3.779.566	-0.1532343	NDUFA5	NADH:ubiquinone oxidoreductase subunit A5
3.777.058	-0.208847	MAPK10	mitogen-activated protein kinase 10
3.774.531	-0.1359419	ZNF771	zinc finger protein 771
3.772.815	-0.0989849		
3.771.876	-0.1963773	GPR65	G protein-coupled receptor 65
3.771.617	-0.3338803	SMPDL3A	sphingomyelin phosphodiesterase acid like 3A
3.769.646	-0.1663082	PGLYRP4	peptidoglycan recognition protein 4
3.748.032	-0.1027539	UPK2	uroplakin 2
374.764	0.2529036	PLEKHA1	pleckstrin homology domain containing A1
3.744.194	0.1920367	KLF3	Kruppel like factor 3
3.739.213	-0.1643562	MYH14	myosin, heavy chain 14, non-muscle
3.731.743	-0.3311472	LOC72787	hypothetical LOC727877///cyclin L2
3.730.218	-0.1065933	ZMYND10	zinc finger MYND-type containing 10
3.728.434	-0.1220937	PACRG	PARK2 coregulated
3.726.822	0.2483346	CDS1	CDP-diacylglycerol synthase 1
3.726.694	0.2695046	PHF3	PHD finger protein 3
3.725.239	-0.1128965		
372.433	0.2768153	KRAS	KRAS proto-oncogene, GTPase
3.722.634	0.3196066	ADGRL4	adhesion G protein-coupled receptor L4
3.720.461	-0.207651	TNFAIP2	TNF alpha induced protein 2
3.719.815	-0.0947865	ROS1	ROS proto-oncogene 1, receptor tyrosine kinas
3.719.129	0.150713	MED9	mediator complex subunit 9
3.714.395	-0.7697994	RELN	reelin
3.714.347	-0.1271857	TRPV5	transient receptor potential cation channel sub
3.714.232	0.1839735	APPBP2	amyloid beta precursor protein binding protein
3.713.409	-0.1048356	RASGRP2	RAS guanyl releasing protein 2
3.712.611	0.2666583	TNPO1	transportin 1
3.711.231	-0.1119787		
3.707.254	-0.1232217	IL16	interleukin 16
3.705.405	-0.30204	TGOLN2	trans-golgi network protein 2
3.705.364	-0.2851197	TSPYL1	TSPY like 1

3.703.062	0.3405297	CYB561	cytochrome b561
3.702.804	0.2350469	PLA2G1B	phospholipase A2 group IB
3.702.479	-0.2327271	NKTR	natural killer cell triggering receptor
3.701.592	-0.1425895	RETN	resistin
3.700.778	-0.1258118	CNTFR	ciliary neurotrophic factor receptor
3.699.433	-0.3439149	CPT1A	carnitine palmitoyltransferase 1A
3.698.331	-0.1132918	SERPINB10	serpin family B member 10
369.339	-0.1406576		
3.691.896	0.2356517	ATP5S	ATP synthase, H+ transporting, mitochondrial F
3.691.282	0.2351724	ITM2B	integral membrane protein 2B
3.691.006	-0.225102	CNN2	calponin 2
3.688.501	-0.1731765	JMJD7-PLA2	JMJD7-PLA2G4B readthrough
3.686.968	-0.0948943	ANK1	ankyrin 1
3.681.874	-0.1295192	MYOZ2	myozenin 2
3.681.073	-0.1148267	CYTH3	cytohesin 3
3.678.478	0.3802664	DDR1	discoidin domain receptor tyrosine kinase 1
3.678.114	-0.1248646	CFLAR	CASP8 and FADD like apoptosis regulator
3.675.777	-0.3098197		
3.672.803	-0.1346669	LLGL1	LLGL1, scribble cell polarity complex compone
3.672.005	0.1271002	HSPA1L	heat shock protein family A (Hsp70) member 1
3.671.134	-0.3584429	RAC2	ras-related C3 botulinum toxin substrate 2 (rho
3.670.614	0.3764628	CLPTM1	CLPTM1, transmembrane protein
3.670.034	0.3221092	ZNF292	zinc finger protein 292
3.669.802	-0.0983191	CASP5	caspase 5
3.668.425	-0.090488	PMS2P3	PMS1 homolog 2, mismatch repair system com
3.668.235	-0.0830843	INSL3	insulin like 3
3.665.374	0.251239	PDHX	pyruvate dehydrogenase complex component X
3.663.463	0.1485393	MYO19	myosin XIX
3.646.884	-0.2411761	DOCK2	dedicator of cytokinesis 2
364.517	-0.1027857	ERGIC3	ERGIC and golgi 3
3.638.385	-0.1193414	ETS1	ETS proto-oncogene 1, transcription factor
3.636.613	-0.094707	KRT34	keratin 34
3.635.801	0.1707043	MID2	midline 2
3.634.601	0.1587722	COQ7	coenzyme Q7, hydroxylase
3.632.893	0.2623145	TGM3	transglutaminase 3
3.632.812	-0.1834261	LOC64707(	nascent polypeptide-associated complex subu
363.124	0.1557102	SREK1IP1	SREK1 interacting protein 1
3.624.788	0.1163912	ZFY///ZFX	zinc finger protein, Y-linked///zinc finger protein
3.619.009	-0.1331858	HTR1D	5-hydroxytryptamine receptor 1D
3.617.878	-0.1514264	SPON1	spondin 1
3.615.687	-0.1142159	KCNIP1	potassium voltage-gated channel interacting pr
3.615.529	0.3010333	CCNG1	cyclin G1
3.614.341	0.2196545	ILF3	interleukin enhancer binding factor 3
3.611.428	-0.1022133	SMARCB1	SWI/SNF related, matrix associated, actin depe

360.624	-0.3630726	CITED2	Cbp/p300 interacting transactivator with Glu/A:
3.604.654	-0.1729877	DNALI1	dynein axonemal light intermediate chain 1
3.603.944	-0.1294711	CACNA1F	calcium voltage-gated channel subunit alpha1
3.603.642	-0.3754612	ANXA4	annexin A4
360.192	0.103647	BAIAP2L2	BAI1 associated protein 2 like 2
3.601.194	-0.2552107	UQCRC2	ubiquinol-cytochrome c reductase core protein
3.600.478	-0.1092073	MYL7	myosin light chain 7
3.600.415	-0.0781482	PTH	parathyroid hormone
3.599.949	-0.1907291	VAV2	vav guanine nucleotide exchange factor 2
3.598.901	-0.1676024	MYO10	myosin X
3.597.209	-0.4459699	NIPSNAP1	nipsnap homolog 1 (C. elegans)
3.596.475	-0.4990711		
3.595.886	-0.120662	TCL1B	T-cell leukemia/lymphoma 1B
3.595.474	-0.2536963	PHF10	PHD finger protein 10
359.536	-0.1394255	WASL	Wiskott-Aldrich syndrome like
3.594.417	-0.2089727	SCAP	SREBF chaperone
3.592.151	-0.211089	ZNF839	zinc finger protein 839
359.145	-0.1874953	ZNF574	zinc finger protein 574
3.590.448	-0.1949857	EFS	embryonal Fyn-associated substrate
3.590.294	-0.1858846	PRKCE	protein kinase C epsilon
3.586.074	-0.3617026	INSIG2	insulin induced gene 2
3.581.706	-0.124187	HERC2P8	hect domain and RLD 2 pseudogene 8
3.579.317	-0.1388192	ABCA2	ATP binding cassette subfamily A member 2
3.577.635	-0.1289624	EMX1	empty spiracles homeobox 1
3.577.205	-0.2628932	ESD	esterase D
3.575.023	-0.1075358	DRD2	dopamine receptor D2
3.570.516	0.2333313	GSS	glutathione synthetase
3.567.337	0.2757778	GOLGA5	golgin A5
3.561.826	-0.3760468	PID1	phosphotyrosine interaction domain containing
3.561.473	-0.1684049	C9orf78	chromosome 9 open reading frame 78
356.134	-0.1713461	DGCR8	DGCR8 microprocessor complex subunit
3.559.014	-0.1410035	PAPPA	pappalysin 1
3.557.368	-0.1277277	MAPK4	mitogen-activated protein kinase 4
3.555.041	0.2631943	ATP5C1	ATP synthase, H+ transporting, mitochondrial F
3.553.974	-0.290201	SLC25A11	solute carrier family 25 member 11
3.551.571	0.2467107	SNAP23	synaptosome associated protein 23
3.550.262	-0.1958006	CXorf21	chromosome X open reading frame 21
3.549.977	0.2373811	DGKZ	diacylglycerol kinase zeta
3.548.851	-0.2248725	TPK1	thiamin pyrophosphokinase 1
3.546.512	-0.1294008	FLJ14100	hypothetical protein FLJ14100
3.544.517	-0.1252153	CXorf36	chromosome X open reading frame 36
354.183	-0.1413014	NOX1	NADPH oxidase 1
3.541.542	0.2049975	RHOF	ras homolog family member F, filopodia associ
3.540.795	-0.1229019	RAB40C	RAB40C, member RAS oncogene family

3.538.159	-0.3041309	UAP1	UDP-N-acetylglucosamine pyrophosphorylase
3.531.223	0.3063781	MYO1D	myosin ID
3.531.218	-0.1103742		
3.530.899	0.2792263	SRSF3	serine and arginine rich splicing factor 3
3.527.639	-0.1517749	GFER	growth factor, augmenter of liver regeneration
3.526.569	0.1526491	FLOT1	flotillin 1
3.525.986	-0.095003	CNR1	cannabinoid receptor 1
3.521.601	-0.1032893	PAPPA2	pappalysin 2
3.520.772	0.1475112	GRK6	G protein-coupled receptor kinase 6
351.971	0.2133933	SLC25A17	solute carrier family 25 member 17
3.514.699	0.2487354	GPC5	glypican 5
3.514.462	0.2955789	RALGAPA1	Ral GTPase activating protein catalytic alpha su
3.508.939	0.2381616	PTBP1	polypyrimidine tract binding protein 1
3.508.261	-0.1262499	NTRK2	neurotrophic receptor tyrosine kinase 2
350.385	-0.0957309	TPT1	tumor protein, translationally-controlled 1
3.501.781	-0.1205791	CXCR5	C-X-C motif chemokine receptor 5
349.822	0.2332228	ZNF580	zinc finger protein 580
3.497.326	-0.1277576	FAM65B	family with sequence similarity 65 member B
3.496.078	0.2660917	WAPL	WAPL cohesin release factor
3.494.815	0.3119452	RB1	RB transcriptional corepressor 1
3.494.293	-0.1429335	SCRG1	stimulator of chondrogenesis 1
3.492.817	0.4929626	LAMB1	laminin subunit beta 1
3.489.862	-0.1035626	TRPM6	transient receptor potential cation channel sub
3.488.526	-0.2407437	SPOCK2	sparc/osteonectin, cwcv and kazal-like domain
3.486.994	0.1979779	LOC15756:	uncharacterized LOC157562
3.485.116	-0.0796002	FAM13C	family with sequence similarity 13 member C
3.480.388	0.3593544	ATP6V1A	ATPase H+ transporting V1 subunit A
3.477.427	0.7495761	GAGE2A///(	G antigen 2A///G antigen 2D///G antigen 12J///(
3.470.898	-0.0976372	NRP2	neuropilin 2
3.469.878	-0.1458827	KCNMB1	potassium calcium-activated channel subfamil
3.469.135	-0.1235607		
346.831	0.2755554	CDC42EP4	CDC42 effector protein 4
3.465.369	0.0959988		
3.459.474	-0.1149986	GYPB	glycophorin B (MNS blood group)
3.459.338	-0.2059372	ATF7IP	activating transcription factor 7 interacting prot
3.458.648	0.1664469	PPIE	peptidylprolyl isomerase E
3.458.527	-0.1034182	PRKCZ	protein kinase C zeta
3.441.353	-0.1929299	KCNAB1	potassium voltage-gated channel subfamily A n
3.440.739	0.1581216	RPL3	ribosomal protein L3
3.439.932	-0.1185482	SYT13	synaptotagmin 13
343.963	-0.1341104	ZNF749	zinc finger protein 749
3.435.828	0.2166129	PGAP1	post-GPI attachment to proteins 1
3.434.886	0.343054	C6orf62	chromosome 6 open reading frame 62
3.433.559	-0.1014028	SHOX	short stature homeobox

3.429.994	-0.1153284	SYNJ2	synaptojanin 2
3.429.422	-0.2758805	AKR1A1	aldo-keto reductase family 1 member A1
3.428.585	-0.105928		
3.428.137	-0.1124679	HTR3B	5-hydroxytryptamine receptor 3B
3.427.886	0.3050639	MFHAS1	malignant fibrous histiocytoma amplified seque
3.422.056	-0.1534522	CHRNB2	cholinergic receptor nicotinic beta 2 subunit
3.420.067	-0.1558014	PPP1R16B	protein phosphatase 1 regulatory subunit 16B
3.416.612	-0.3301148	EMP2	epithelial membrane protein 2
3.415.484	-0.1085634	PNLIP	pancreatic lipase
3.412.376	-0.1065521	ITSN2	intersectin 2
3.398.601	0.322062	MAGEB2	MAGE family member B2
338.784	0.2861322	PICALM	phosphatidylinositol binding clathrin assembly
3.374.928	-0.1289778	RUNX2	runt related transcription factor 2
3.373.546	-0.0843675	MARK2	microtubule affinity regulating kinase 2
3.370.351	-0.4970926	TTPA	alpha tocopherol transfer protein
3.369.742	-0.1258788	CD84	CD84 molecule
3.369.705	-0.1276676	GPR32	G protein-coupled receptor 32
3.369.158	0.2508531	RACK1	receptor for activated C kinase 1
3.367.042	0.2273282	MRPL23	mitochondrial ribosomal protein L23
3.366.567	0.5256228	NPTX2	neuronal pentraxin 2
3.364.062	0.3457177	ZMYND8	zinc finger MYND-type containing 8
3.358.947	-0.1240466	HSPA12A	heat shock protein family A (Hsp70) member 12
3.357.217	-0.1054494		
3.355.383	0.1801024	DSC2	desmocollin 2
3.353.579	-0.1387389	FLJ21369	uncharacterized protein FLJ21369
3.346.943	0.2125317	IMPACT	impact RWD domain protein
3.339.324	0.1958991	CAPZB	capping actin protein of muscle Z-line beta sub
3.338.957	-0.2309484	WDFY3	WD repeat and FYVE domain containing 3
3.338.197	0.3543143	SLC12A1	solute carrier family 12 member 1
3.337.902	-0.2040665		
3.336.748	-0.2199671	PDE3B	phosphodiesterase 3B
3.336.253	-0.1389476	CSHL1	chorionic somatomammotropin hormone like 1
3.335.319	-0.1218619	TUBBP5///T	tubulin beta pseudogene 5///tubulin beta 8 clas
3.332.307	0.2360812	SALL2	spalt like transcription factor 2
3.331.831	-0.3033719	SH3BP5	SH3 domain binding protein 5
3.330.464	-0.1005569	PLA2G2E	phospholipase A2 group IIE
3.330.233	-0.1198027	CDSN	corneodesmosin
3.329.297	-0.3797687		
3.328.846	-0.1296891	AQP5	aquaporin 5
3.328.229	0.3540054	FXR1	FMR1 autosomal homolog 1
3.322.467	-0.1005679	NAV1	neuron navigator 1
3.318.804	0.1632857	CDC42BPA	CDC42 binding protein kinase alpha
3.316.439	-0.1538761	CACNB1	calcium voltage-gated channel auxiliary subuni
3.316.203	0.5886254	GAGE3	G antigen 3

331.516	-0.1212858	UTP14A	UTP14A small subunit processome component
331.108	0.4119568	BEX4	brain expressed X-linked 4
3.310.507	-0.0934114	EPX	eosinophil peroxidase
3.307.286	-0.1420088	FOXO4	forkhead box O4
3.303.326	-0.1168999	WISP1	WNT1 inducible signaling pathway protein 1
3.299.121	-0.1087363	ZNF287	zinc finger protein 287
3.298.661	-0.1109255	PTPN1	protein tyrosine phosphatase, non-receptor typ
3.293.554	-0.0950816	TEF	TEF, PAR bZIP transcription factor
3.291.183	-0.2774787	SPANXC///I	SPANX family member C///deleted in lymphocy
3.287.266	-0.1563847	ATP4B	ATPase H+/K+ transporting beta subunit
3.284.495	-0.0984976	FLJ10232	hypothetical protein FLJ10232
3.282.345	-0.1053905	RPL41	ribosomal protein L41
3.281.613	-0.1172096	PIGO	phosphatidylinositol glycan anchor biosynthesi
3.279.571	-0.1434734	STAT2	signal transducer and activator of transcription
3.279.115	0.2109137	FUT2	fucosyltransferase 2
3.278.185	-0.2624252	DEXI	Dexi homolog
3.276.943	-0.2814456	HSDL2	hydroxysteroid dehydrogenase like 2
3.271.852	-0.1121047	VPS16	VPS16, CORVET/HOPS core subunit
3.270.778	-0.2945501	KLHL3	kelch like family member 3
3.270.287	-0.208397	ABHD10	abhydrolase domain containing 10
3.269.867	-0.1142214	CD84	CD84 molecule
326.875	0.2366618	SUGP2	SURP and G-patch domain containing 2
325.757	-0.1404847	CASR	calcium sensing receptor
3.257.009	-0.1003667	NPHS1	NPHS1, nephrin
325.573	-0.2237978	ARHGEF12	Rho guanine nucleotide exchange factor 12
3.255.374	-0.1198749	OR7C2	olfactory receptor family 7 subfamily C membe
3.253.587	-0.2628365	FGFR1	fibroblast growth factor receptor 1
3.251.633	0.2404704	ANP32B	acidic nuclear phosphoprotein 32 family memb
3.250.115	-0.093196	INPP4B	inositol polyphosphate-4-phosphatase type II B
3.244.811	-0.1340538	ARMC7	armadillo repeat containing 7
3.243.845	0.1603917	MAPKAPK2	mitogen-activated protein kinase-activated pro
324.154	-0.10574	HOXC5	homeobox C5
3.237.415	0.36294	LOX	lysyl oxidase
3.234.363	-0.1668551	MMP25	matrix metallopeptidase 25
3.231.431	-0.1011069	YES1	YES proto-oncogene 1, Src family tyrosine kinas
3.230.518	-0.1191961	SPTAN1	spectrin alpha, non-erythrocytic 1
3.229.248	0.238706	ITGB5	integrin subunit beta 5
3.228.985	-0.1386613	HNRNPUL2	heterogeneous nuclear ribonucleoprotein U like
3.228.299	-0.1255762	GK2	glycerol kinase 2
3.227.961	0.2721831	ZNF573	zinc finger protein 573
3.227.413	-0.0934564	GATA2	GATA binding protein 2
3.227.405	-0.1101553	CDK5R2	cyclin dependent kinase 5 regulatory subunit 2
3.214.937	-0.0990327	ENPP1	ectonucleotide pyrophosphatase/phosphodies
3.214.391	-0.2207232	TFB1M	transcription factor B1, mitochondrial

3.213.141	0.2156329	KRIT1	KRIT1, ankyrin repeat containing
321.048	-0.5218619	SRGN	serglycin
3.209.471	0.1865763	CDKN2C	cyclin dependent kinase inhibitor 2C
3.207.537	0.1757506	KDM4C	lysine demethylase 4C
3.207.056	0.1138199	OTUB2	OTU deubiquitinase, ubiquitin aldehyde binding
3.205.919	0.142373	SPATA5L1	spermatogenesis associated 5 like 1
320.512	-0.1114423	NOP14-AS1	NOP14 antisense RNA 1
3.204.705	-0.1166135	GABRB3	gamma-aminobutyric acid type A receptor beta
3.202.086	-0.0892597	TBC1D2B	TBC1 domain family member 2B
3.200.777	-0.0963725	RFC1	replication factor C subunit 1
3.199.857	-0.1007252	CNGB1	cyclic nucleotide gated channel beta 1
3.194.365	-0.102242	DCHS2	dachsous cadherin-related 2
3.193.971	-0.0847825	HEXA-AS1	HEXA antisense RNA 1
3.192.923	-0.2221571	NPR3	natriuretic peptide receptor 3
3.190.662	-0.2726951	B4GALT1	beta-1,4-galactosyltransferase 1
3.185.584	0.2739026	KIF21B	kinesin family member 21B
3.185.107	-0.2505396	CPOX	coproporphyrinogen oxidase
3.174.437	-0.1046995	HOXD9	homeobox D9
3.173.721	-0.1703286	STARD8	StAR related lipid transfer domain containing 8
3.168.254	0.2286006	TIAL1	TIA1 cytotoxic granule-associated RNA binding
3.167.282	-0.0832677	OR1A2	olfactory receptor family 1 subfamily A member
3.166.755	0.3134327	TGIF1	TGFB induced factor homeobox 1
3.166.408	0.2911118	AGRN	agrin
3.162.685	0.2679871	ACSL6	acyl-CoA synthetase long-chain family member
3.161.633	-0.2699969	NR2F1	nuclear receptor subfamily 2 group F member 1
3.159.785	0.2433762	PTP4A2	protein tyrosine phosphatase type IVA, member
3.159.382	-0.3387527	GSTP1	glutathione S-transferase pi 1
3.156.801	0.1307528	CCDC85C	coiled-coil domain containing 85C
3.154.976	-0.2194183	KLHL20	kelch like family member 20
3.147.483	-0.1185696	KCNJ5	potassium voltage-gated channel subfamily J m
3.145.153	-0.0936467	MMP11	matrix metallopeptidase 11
3.144.688	-0.1790037	HRASLS2	HRAS like suppressor 2
3.142.081	0.2004694	CLCC1	chloride channel CLIC like 1
3.140.042	-0.1193287	BTBD7	BTB domain containing 7
3.139.464	-0.1308709	ITGB3	integrin subunit beta 3
3.138.385	0.1744511	LANCL1	LanC like 1
3.136.414	0.379254	CTAG1A///(	cancer/testis antigen 1A///cancer/testis antiger
3.131.994	0.1695734	C2orf49	chromosome 2 open reading frame 49
3.129.681	-0.2091622	ARHGAP35	Rho GTPase activating protein 35
3.129.359	0.3114671	NRXN3	neurexin 3
3.126.391	-0.1271772	CACNB1	calcium voltage-gated channel auxiliary subuni
3.125.087	0.3356646	RPL31P4///	ribosomal protein L31 pseudogene 4///ribosom
3.124.878	-0.353046	TIMP3	TIMP metallopeptidase inhibitor 3
3.122.231	-0.2021462	KLHDC4	kelch domain containing 4

3.118.898	0.3994559	MSN	moesin
3.116.164	-0.0947835	FAM189A1	family with sequence similarity 189 member A1
3.115.815	-0.135297	NENF	neudesin neurotrophic factor
3.115.532	-0.1260878	SLITRK5	SLIT and NTRK like family member 5
3.114.862	-0.1071839	ZNF335	zinc finger protein 335
3.114.676	0.2211858	PPM1B	protein phosphatase, Mg2+/Mn2+ dependent 1
3.114.579	-0.1467079		
3.113.732	0.2572346	LOC44043	aminopeptidase puromycin sensitive pseudoge
3.109.891	0.2978783	SEPT11	septin 11
3.109.154	-0.2597535	MMP24	matrix metallopeptidase 24
3.102.721	-0.2355127	VPS13A	vacuolar protein sorting 13 homolog A
3.099.957	0.1377073	MVD	mevalonate diphosphate decarboxylase
3.096.107	-0.2181397	KCNH7	potassium voltage-gated channel subfamily H r
3.095.436	-0.1406646	MPP2	membrane palmitoylated protein 2
3.090.103	-0.09765	ATF7	activating transcription factor 7
3.088.709	0.2703294	LOC38782	DnaJ heat shock protein family (Hsp40) membe
3.088.351	0.3784318	STAT1	signal transducer and activator of transcription
3.085.982	0.3390548	POPDC3	popeye domain containing 3
3.084.527	-0.1474683	WT1	Wilms tumor 1
3.081.174	0.1807853	KRBOX4	KRAB box domain containing 4
3.079.997	0.2079943	PRPF40A	pre-mRNA processing factor 40 homolog A
3.073.339	0.2693891	TNIK	TRAF2 and NCK interacting kinase
307.188	-0.3657267	GGCX	gamma-glutamyl carboxylase
3.071.519	-0.1198753	TP53AIP1	tumor protein p53 regulated apoptosis inducing
3.071.476	-0.1059182	PDE4C	phosphodiesterase 4C
3.067.091	0.2023763	BIK	BCL2 interacting killer
3.064.669	0.1712584	HPCAL1	hippocalcin like 1
3.062.261	-0.1830262	MARCH1	membrane associated ring-CH-type finger 1
3.061.567	-0.178391	PYY	peptide YY
3.061.022	0.1579011	TFAM	transcription factor A, mitochondrial
3.058.881	-0.1087111	PRKCA	protein kinase C alpha
305.352	-0.2257851	CTSB	cathepsin B
3.048.995	-0.0939113	PDIA2	protein disulfide isomerase family A member 2
3.047.882	-0.0962667	ZBTB7B	zinc finger and BTB domain containing 7B
3.047.199	-0.1587879	LDB3	LIM domain binding 3
3.046.718	-0.1167281	MYOZ2	myozenin 2
3.045.744	0.1390045	ZBTB22	zinc finger and BTB domain containing 22
3.044.873	-0.1022359	IGSF9B	immunoglobulin superfamily member 9B
3.044.163	-0.1753701	MVK	mevalonate kinase
303.956	0.2259238	LUC7L3	LUC7 like 3 pre-mRNA splicing factor
3.038.182	-0.1497398	FOXD4L1//	forkhead box D4-like 1///forkhead box D4
303.568	0.3686365	ABCC1	ATP binding cassette subfamily C member 1
303.529	-0.1302082	AMELY	amelogenin, Y-linked
3.032.327	-0.2816576	NFE2L2	nuclear factor, erythroid 2 like 2

3.031.994	0.2590842	USP33	ubiquitin specific peptidase 33
3.029.092	-0.2470967	MFN2	mitofusin 2
3.027.715	-0.1569707	SGF29	SAGA complex associated factor 29
302.487	-0.2195468	KCNAB2	potassium voltage-gated channel subfamily A r
3.023.963	-0.12186	SEPT5///GF	septin 5///glycoprotein Ib platelet beta subunit
3.022.197	-0.3921929	ITGBL1	integrin subunit beta like 1
3.017.819	-0.1321341	EXOSC1	exosome component 1
3.015.602	-0.1195494	CDK6	cyclin dependent kinase 6
3.011.494	-0.0920862	GH2///GH1	growth hormone 2///growth hormone 1///chorio
3.011.259	0.1133801	BBS9	Bardet-Biedl syndrome 9
3.010.423	-0.2693194	ADAMTS5	ADAM metallopeptidase with thrombospondin t
3.008.354	0.14674	SGSM3	small G protein signaling modulator 3
3.005.753	0.2036012	ZNF43	zinc finger protein 43
3.003.469	-0.1187298	KCNC4	potassium voltage-gated channel subfamily C r
3.002.295	-0.1083424	ZNF117	zinc finger protein 117
2.989.465	-0.1343639	NKX3-1	NK3 homeobox 1
2.987.172	-0.1189811	SBF1	SET binding factor 1
2.986.753	-0.2376656	HNRNPDL	heterogeneous nuclear ribonucleoprotein D like
2.983.291	-0.1698939	SH3D21	SH3 domain containing 21
2.980.401	0.1501389	FBXL15	F-box and leucine rich repeat protein 15
2.974.025	0.1986245	PBXIP1	PBX homeobox interacting protein 1
2.972.488	0.1521397	MAPK3	mitogen-activated protein kinase 3
2.971.779	-0.3141865	KALRN	kalirin, RhoGEF kinase
2.970.319	-0.1150778	RANBP3	RAN binding protein 3
2.970.043	0.0863104	PRR7	proline rich 7 (synaptic)
2.969.427	-0.2233099	ABCD4	ATP binding cassette subfamily D member 4
296.926	0.173091	IDH3B	isocitrate dehydrogenase 3 (NAD(+)) beta
2.968.657	0.1523414	WDR77	WD repeat domain 77
2.968.453	0.235593	GGA1	golgi associated, gamma adaptin ear containing
2.965.092	0.2009963	PELP1	proline, glutamate and leucine rich protein 1
2.963.277	0.2509898	LOC65012	similar to choline kinase alpha isoform a///chol
2.963.233	0.1457866		
2.960.887	-0.1079176	HPCA	hippocalcin
2.960.716	-0.1197391	SPATA31A7	SPATA31 subfamily A member 7
2.957.794	-0.1923925	LMBR1L///S	limb development membrane protein 1 like///sy
2.947.455	-0.1243687	NDUFB2-AS	NDUFB2 antisense RNA 1
2.945.728	-0.1150222	GPR22	G protein-coupled receptor 22
2.945.319	-0.1371106	ADAMTS7	ADAM metallopeptidase with thrombospondin t
2.944.734	-0.0816175	EPHA7	EPH receptor A7
2.943.549	-0.1332807	MLX	MLX, MAX dimerization protein
2.942.631	-0.0932197	RLN1	relaxin 1
2.938.567	-0.133042	NPY2R	neuropeptide Y receptor Y2
2.937.733	-0.1396518	SPTBN1	spectrin beta, non-erythrocytic 1
2.936.566	-0.3806535	MTUS1	microtubule associated tumor suppressor 1

293.626	0.1221091	ZNF350	zinc finger protein 350
2.933.496	-0.1487076	PTAFR	platelet activating factor receptor
2.929.257	-0.1168266	ADAMTS7	ADAM metallopeptidase with thrombospondin t
292.801	0.1500189	PIDD1	p53-induced death domain protein 1
2.917.784	-0.1313075	ATXN7L1	ataxin 7 like 1
2.917.558	-0.1032271	ERBB2	erb-b2 receptor tyrosine kinase 2
2.914.053	-0.1254911	KLHL1	kelch like family member 1
2.913.886	-0.1064413	UCP1	uncoupling protein 1
2.911.972	-0.1067306	EDN2	endothelin 2
29.085	-0.1601063	ELSPBP1	epididymal sperm binding protein 1
2.908.296	0.2224982	LCMT2	leucine carboxyl methyltransferase 2
2.908.084	-0.3356939	STAP2	signal transducing adaptor family member 2
290.666	-0.8694478	DHRS2	dehydrogenase/reductase 2
2.905.075	0.1028775	RAB3B	RAB3B, member RAS oncogene family
2.901.577	0.3089645	ABLIM1	actin binding LIM protein 1
2.900.098	0.2368014	ARIH1	ariadne RBR E3 ubiquitin protein ligase 1
2.895.919	-0.1221146	GAD2	glutamate decarboxylase 2
2.890.941	-0.1994078	SNAPC3	small nuclear RNA activating complex polypept
2.888.964	-0.1361464	FHL1	four and a half LIM domains 1
2.886.754	0.2715774	MICU2	mitochondrial calcium uptake 2
2.881.594	0.34694	TMEM243	transmembrane protein 243
2.880.959	0.097878	ZNF35	zinc finger protein 35
288.071	-0.4442095		
2.877.486	0.2498184	PSTPIP2	proline-serine-threonine phosphatase interacti
2.876.717	-0.0985048	FUT5	fucosyltransferase 5
2.875.904	-0.0877204	ANGPT4	angiopoietin 4
2.869.446	-0.1039826	CPSF1	cleavage and polyadenylation specific factor 1
2.869.439	-0.0945261	POMC	proopiomelanocortin
2.866.655	-0.1888057	MCL1	BCL2 family apoptosis regulator
2.863.699	-0.0977884	TLX2	T-cell leukemia homeobox 2
2.857.305	0.4888215	SCD	stearoyl-CoA desaturase
2.857.182	0.1525425	KCNE5	potassium voltage-gated channel subfamily E r
2.856.732	-0.2171119	HELZ	helicase with zinc finger
2.855.503	0.141897	PSMC3IP	PSMC3 interacting protein
285.301	-0.1583568	TAOK2	TAO kinase 2
2.842.513	-0.1270579	ZZEF1	zinc finger ZZ-type and EF-hand domain contair
2.834.633	0.1753779	LTN1	listerin E3 ubiquitin protein ligase 1
2.830.176	-0.1286316	KIRREL	kin of IRRE like (Drosophila)
2.829.249	-0.102736	ST8SIA2	ST8 alpha-N-acetyl-neuraminide alpha-2,8-sial
282.824	0.2232789	ESYT1	extended synaptotagmin 1
2.827.536	-0.1583702	KAT6B	lysine acetyltransferase 6B
2.821.073	-0.1151128	GPLD1	glycosylphosphatidylinositol specific phosphol
2.820.011	-0.1494721	ATRX	ATRX, chromatin remodeler
2.818.625	0.1063767	ANXA2P3	annexin A2 pseudogene 3

2.818.268	0.2599135	CDS2	CDP-diacylglycerol synthase 2
2.816.721	0.1845259	SCMH1	sex comb on midleg homolog 1 (Drosophila)
2.815.857	0.5617506	HIST1H1C	histone cluster 1, H1c
2.805.216	-0.1432132	NPFF	neuropeptide FF-amide peptide precursor
2.801.879	-0.1349862	ARTN	artemin
2.801.304	-0.1243446	GM2A	GM2 ganglioside activator
2.800.685	-0.1223987	HCRTR2	hypocretin receptor 2
2.796.408	0.3746275	CNN3	calponin 3
2.793.379	-0.1852177	PTPN18	protein tyrosine phosphatase, non-receptor typ
2.793.032	0.2013424	SCGB1D2	secretoglobin family 1D member 2
2.787.175	-0.1506707		
2.785.911	0.1234576	ALKBH4	alkB homolog 4, lysine demethylase
2.785.286	-0.2030276	GRB7	growth factor receptor bound protein 7
2.784.346	-0.1080561	FRS2	fibroblast growth factor receptor substrate 2
2.781.518	-0.1140123	PNLIPRP1	pancreatic lipase related protein 1
2.780.701	0.2936323	DAZAP2	DAZ associated protein 2
2.771.159	-0.1399743	ITGB3	integrin subunit beta 3
2.770.589	0.2318544	C14orf2	chromosome 14 open reading frame 2
2.769.837	0.1336292	BRCA2	BRCA2, DNA repair associated
2.767.704	0.3321508	HIST1H2BD	histone cluster 1, H2bd
2.767.312	0.4031445	TM4SF20	transmembrane 4 L six family member 20
2.766.747	-0.1329076	F2RL2	coagulation factor II thrombin receptor like 2
2.764.485	-0.0960781	IGLV6-57	immunoglobulin lambda variable 6-57
2.762.101	-0.0897134	HUWE1	HECT, UBA and WWE domain containing 1, E3 u
2.761.569	-0.1060117	IL5RA	interleukin 5 receptor subunit alpha
276.091	0.1475159	RPL3P7	ribosomal protein L3 pseudogene 7
2.760.246	-0.1133701	NCR2	natural cytotoxicity triggering receptor 2
2.758.664	0.1413974	CCDC88A	coiled-coil domain containing 88A
2.758.403	-0.1389825		
2.756.964	-0.2622881	HBEGF	heparin binding EGF like growth factor
2.756.222	0.6030809	GPR37	G protein-coupled receptor 37
2.754.799	-0.1494411	CLCN7	chloride voltage-gated channel 7
2.748.533	-0.1282276	MAP2K7	mitogen-activated protein kinase kinase 7
2.741.645	0.2723712	GRSF1	G-rich RNA sequence binding factor 1
2.738.668	0.2818087	TOR1AIP1	torsin 1A interacting protein 1
2.737.105	0.2881496	SLCO2A1	solute carrier organic anion transporter family r
2.731.054	0.2678065	MTRR	5-methyltetrahydrofolate-homocysteine methy
2.729.658	0.1901166	PANK4	pantothenate kinase 4
2.726.428	-0.160588	TBX2	T-box 2
2.726.127	-0.1367974	NCOR2	nuclear receptor corepressor 2
2.725.399	-0.1043051	EGR4	early growth response 4
2.724.746	-0.1134119	MEG3	maternally expressed 3 (non-protein coding)
2.723.934	-0.1139991	NEU3	neuraminidase 3
272.184	0.2365628	EP300	E1A binding protein p300

2.718.859	-0.2946849		CXCL8	C-X-C motif chemokine ligand 8
2.714.199	-0.1907904		VSTM4	V-set and transmembrane domain containing 4
2.713.543	-0.208759		TXN2	thioredoxin 2
2.713.203	-0.1490896		SEZ6L	seizure related 6 homolog like
2.710.914	0.6094768		TMED2	transmembrane p24 trafficking protein 2
2.709.348	-0.1103881		MTMR8	myotubularin related protein 8
2.708.849	-0.3422682		ITPR2	inositol 1,4,5-trisphosphate receptor type 2
2.707.316	-0.1166144		FZD8	frizzled class receptor 8
2.705.873	0.3576149		MALL	mal, T-cell differentiation protein like
2.704.236	-0.1594633		YBX3	Y-box binding protein 3
2.704.128	-0.1058971		LOC10669	uncharacterized LOC106699570
269.499	0.1792134		ZNF557///M	zinc finger protein 557///migration and invasion
2.693.995	-0.1479766		KLK1	kallikrein 1
2.685.957	-0.1053225		FPR2	formyl peptide receptor 2
2.684.704	-0.1000111		KLK2	kallikrein related peptidase 2
2.683.898	-0.0838629		CLIC1P1	chloride intracellular channel 1 pseudogene 1
2.683.531	-0.1717463		ZRSR2	zinc finger CCCH-type, RNA binding motif and s
2.682.439	0.1287371		SGSM3	small G protein signaling modulator 3
2.680.328	-0.1219863		GP2	glycoprotein 2
2.678.898	-0.2092709			
2.678.508	-0.0887569		LOC51145	uncharacterized LOC51145
2.676.239		-10.205.752	INS-IGF2///	INS-IGF2 readthrough///insulin like growth fact
2.674.351	-0.1237214		SBNO1	strawberry notch homolog 1
267.297	-0.331058		TAPBPL	TAP binding protein like
2.672.171	-0.0953195		LOC72997	nuclear pore complex interacting protein family
2.668.359	0.1302435		POTEKP	POTE ankyrin domain family member K, pseudo
2.666.467	0.2739912		TRIOBP	TRIO and F-actin binding protein
2.664.974	-0.3576122		ADGRL2	adhesion G protein-coupled receptor L2
2.664.341	0.1023598		NUS1P3	NUS1 dehydrodolichyl diphosphate synthase s
2.663.457	-0.4505043		NNT	nicotinamide nucleotide transhydrogenase
2.661.423	0.2342799		UBE2W	ubiquitin conjugating enzyme E2 W (putative)
2.661.406	-0.2682363		SLC25A4	solute carrier family 25 member 4
2.660.962	-0.2700202		CLIC4///AD	chloride intracellular channel 4///adducin 3
2.656.776	0.4902765		JAG1	jagged 1
2.655.842	-0.0875149		SDK2	sidekick cell adhesion molecule 2
2.654.753	-0.1331284		MEPE	matrix extracellular phosphoglycoprotein
2.651.417	0.2783172		EIF4A1	eukaryotic translation initiation factor 4A1
2.649.255	-0.1440454		MT3	metallothionein 3
2.645.765	-0.0928722		CCR3	C-C motif chemokine receptor 3
2.644.979	-0.2806668			
2.641.178	-0.0873013		CBLB	Cbl proto-oncogene B
2.637.496	-0.1289908		ALX3	ALX homeobox 3
2.636.293	-0.2759116		HLA-DRB6	major histocompatibility complex, class II, DR b
2.632.562	-0.1234649		SLC25A17	solute carrier family 25 member 17

2.632.248	0.2311798	TPTE	transmembrane phosphatase with tensin homo
2.626.024	-0.1009102	MARCH6	membrane associated ring-CH-type finger 6
2.625.395	-0.1175293	MYBPH	myosin binding protein H
2.621.666	0.2010938	CA12	carbonic anhydrase 12
2.618.032	-0.0959773		
2.617.385	-0.1264593	KCNJ5	potassium voltage-gated channel subfamily J m
2.613.762	-0.0713374	RBMS2	RNA binding motif single stranded interacting p
2.611.604	-0.1171758	GULP1	GULP, engulfment adaptor PTB domain contain
2.611.382	-0.1043765	MCHR1	melanin concentrating hormone receptor 1
2.607.381	0.1390484	C22orf29	chromosome 22 open reading frame 29
260.503	-0.1201643	ARPP21	cAMP regulated phosphoprotein 21
2.601.758	-0.1057359	FLJ11710	uncharacterized protein FLJ11710
260.153	-0.1034599	DRD1	dopamine receptor D1
2.599.465	-0.1078269	HES2	hes family bHLH transcription factor 2
2.599.212	-0.1347329	SIGLEC8	sialic acid binding Ig like lectin 8
2.592.085	-0.1004029	GYPA	glycophorin A (MNS blood group)
2.586.729	0.1321912	PPFIA4	PTPRF interacting protein alpha 4
2.586.568	-0.1282928	CDKL3	cyclin dependent kinase like 3
2.584.716	-0.1049628	ME1	malic enzyme 1
258.178	-0.3139058	AIF1	allograft inflammatory factor 1
258.139	0.2051685	EMCN	endomucin
2.573.498	0.1166742	TBX19	T-box 19
2.572.191	-0.0750036		
2.571.887	-0.1318206	DNAH17	dynein axonemal heavy chain 17
2.571.014	-0.0930623	BMPR1B	bone morphogenetic protein receptor type 1B
2.566.785	-0.2065751	HP1BP3	heterochromatin protein 1 binding protein 3
2.558.486	-0.0871099		
2.557.557	-0.0932849	MYOC	myocilin
2.556.501	0.1056501	KATNB1	katanin regulatory subunit B1
2.554.218	-0.1222374	RFXAP	regulatory factor X associated protein
255.334	-0.5958085	KDM5D	lysine demethylase 5D
2.549.189	0.1104232	ZNF273	zinc finger protein 273
2.545.952	0.363757	CTAG2	cancer/testis antigen 2
2.543.999	-0.1128368	GABRA3	gamma-aminobutyric acid type A receptor alph
2.542.599	0.1585546	CAMK2G	calcium/calmodulin dependent protein kinase
2.536.235	-0.1267945	ITGB8	integrin subunit beta 8
2.535.071	0.1766427	BAG5	BCL2 associated athanogene 5
2.532.068	-0.0923828	IFNA6	interferon alpha 6
2.530.292	-0.0954271	LRCH1	leucine rich repeats and calponin homology do
2.529.825	-0.1221433	PKNOX2	PBX/knotted 1 homeobox 2
2.529.452	-0.1587631	CAMKK2	calcium/calmodulin dependent protein kinase
2.528.206	-0.1439952	LAX1	lymphocyte transmembrane adaptor 1
252.772	-0.135769	LILRA6	leukocyte immunoglobulin like receptor A6
2.527.046	0.3258841	RBPMS	RNA binding protein with multiple splicing

2.526.219	-0.1801985	DNAJC7	DnaJ heat shock protein family (Hsp40) membe
2.522.705	0.2129631	NFYC	nuclear transcription factor Y subunit gamma
252.232	-0.2711969	USO1///PAI	USO1 vesicle transport factor///p21 (RAC1) act
2.515.614	-0.1179921	CCDC181	coiled-coil domain containing 181
2.514.382	-0.0974744	DSCC1	DNA replication and sister chromatid cohesion
2.514.302	-0.1150155	GH2	growth hormone 2
2.513.399	-0.0949008	SPRR2C	small proline rich protein 2C (pseudogene)
2.510.291	-0.1326189	NRP2	neuropilin 2
2.509.482	-0.1085562	IL2	interleukin 2
2.508.797	0.1376087	RAD51D	RAD51 paralog D
2.506.795	-0.2480946	SASH3	SAM and SH3 domain containing 3
2.501.839	0.1622056	SKP2	S-phase kinase-associated protein 2, E3 ubiqui
2.500.891	-0.1022492	EPB41L4A	erythrocyte membrane protein band 4.1 like 4A
249.855	0.2139924	ARF5	ADP ribosylation factor 5
2.498.011	-0.1877454	POLE	DNA polymerase epsilon, catalytic subunit
2.497.479	-0.3092423	TAPBPL	TAP binding protein like
2.496.227	-0.1447002	RGS4	regulator of G-protein signaling 4
2.495.652	-0.0968663	PCNX2	pecanex homolog 2 (Drosophila)
2.487.232	-0.1276207	ZNRF4	zinc and ring finger 4
2.486.661	-0.1394266	CDK13	cyclin dependent kinase 13
2.485.765	-0.5366932	PLA1A	phospholipase A1 member A
2.484.242	-0.1188884	RAB11FIP3	RAB11 family interacting protein 3
2.481.987	-0.0737017	PTPN21	protein tyrosine phosphatase, non-receptor typ
2.477.174	-0.1351139	DPP6	dipeptidyl peptidase like 6
2.476.826	-0.1037138	FUT7	fucosyltransferase 7
2.473.515	-0.117153	FRMD8	FERM domain containing 8
2.469.415	-0.439718	MYL9	myosin light chain 9
2.469.259	0.1962532	MRPL4	mitochondrial ribosomal protein L4
2.468.335	0.1443894	C11orf80	chromosome 11 open reading frame 80
2.466.662	-0.0996744	KLK13	kallikrein related peptidase 13
246.464	0.2236293	ENTPD1	ectonucleoside triphosphate diphosphohydrola
2.462.189	-0.1044259	ZFP64	ZFP64 zinc finger protein
2.457.818	0.2190859	TPI1	triosephosphate isomerase 1
245.306	-0.1241862	ADAM28	ADAM metallopeptidase domain 28
244.908	0.2409108	TUBB2B	tubulin beta 2B class IIb
2.440.609	-0.1177653	ACRV1	acrosomal vesicle protein 1
244.023	-0.1042992	ADGRG1	adhesion G protein-coupled receptor G1
2.431.324	-0.1368633	FLJ13769	hypothetical protein FLJ13769
2.430.991	-0.200034	EHD1	EH domain containing 1
2.421.829	0.2800892	MRPS15///I	mitochondrial ribosomal protein S15///erythroc
2.420.331	-0.2510295	SAV1	salvador family WW domain containing protein
2.418.485	0.1533242	EFCAB14	EF-hand calcium binding domain 14
2.413.806	-0.1193567	PODNL1	podocan like 1
2.406.173	-0.1145456	ATRNL1	attractin like 1

2.401.796	-0.2104586	PNISR	PNN interacting serine and arginine rich protein
23.971	0.1505183	SUGP1	SURP and G-patch domain containing 1
239.628	-0.1956731	RPL13	ribosomal protein L13
2.394.375	-0.1145906	SPO11	SPO11, initiator of meiotic double stranded bre
239.347	0.1237609	SENP7	SUMO1/sentrin specific peptidase 7
239.126	-0.1179659	IBA57	IBA57 homolog, iron-sulfur cluster assembly
2.390.651	-0.1084462	CARD9	caspase recruitment domain family member 9
2.383.472	-0.1456779	IGFBP5	insulin like growth factor binding protein 5
2.381.825	0.1965799	EP300	E1A binding protein p300
2.380.276	-0.2973519	GYG2	glycogenin 2
2.374.955	-0.1176414	DLGAP4	DLG associated protein 4
237.448	-0.1310363	MBD2	methyl-CpG binding domain protein 2
237.342	-0.1070982	ATP10A	ATPase phospholipid transporting 10A (putative
2.372.207	-0.2026934	CNN1	calponin 1
237.056	-0.1144821		
2.368.966	-0.1513572	TNK1	tyrosine kinase non receptor 1
2.368.278	0.2041442	PTEN	phosphatase and tensin homolog
2.358.659	-0.1533212	HECW1	HECT, C2 and WW domain containing E3 ubiqu
235.773	0.2492595	RBM39	RNA binding motif protein 39
2.355.171	-0.2212331	USO1///PAI	USO1 vesicle transport factor///p21 (RAC1) act
2.354.654	-0.2051864	HEY2	hes related family bHLH transcription factor wit
2.352.345	-0.1222911	PDE1C	phosphodiesterase 1C
2.351.853	-0.1070986	CRTAM	cytotoxic and regulatory T-cell molecule
235.178	-0.0733505	KITLG	KIT ligand
2.348.839	-0.1516062	BMP7	bone morphogenetic protein 7
2.348.561	-0.1169978	CEACAM21	carcinoembryonic antigen related cell adhesion
2.342.959	0.1635909	PLEKHG6	pleckstrin homology and RhoGEF domain conta
2.339.933	-0.1196942	PTGER2	prostaglandin E receptor 2
233.601	0.2582849	PDCD4	programmed cell death 4 (neoplastic transform
2.332.548	-0.1668785	PEX16	peroxisomal biogenesis factor 16
233.074	-0.1657354	LOC72916	hCG1732469
2.327.621	0.2465812	TFAM	transcription factor A, mitochondrial
2.327.153	-0.2998204	BTD	biotinidase
2.320.799	0.1508706	TPRA1	transmembrane protein adipocyte associated 1
2.320.508	-0.2671794	IL1RN	interleukin 1 receptor antagonist
2.320.481	-0.3829921	SLC16A7	solute carrier family 16 member 7
231.821	0.5219482	FASN	fatty acid synthase
2.316.666	0.2865159	SRSF10	serine and arginine rich splicing factor 10
231.564	0.3160497	PAK3	p21 (RAC1) activated kinase 3
2.315.619	-0.3612688	CEBPD	CCAAT/enhancer binding protein delta
2.312.414	-0.1290583	BRF1	BRF1, RNA polymerase III transcription initiation
2.309.716	-0.1013574	CSPG4P1Y	chondroitin sulfate proteoglycan 4 pseudogene
2.309.544	-0.1448338	C22orf24	chromosome 22 open reading frame 24
230.675	-0.0999502	CHRNE	cholinergic receptor nicotinic epsilon subunit

2.306.672	0.0994045	DTNA	dystrobrevin alpha
2.306.494	-0.1156899	RBM26	RNA binding motif protein 26
2.305.883	-0.0949139	CCL24	C-C motif chemokine ligand 24
2.302.475	0.118637	GNB1L	G protein subunit beta 1 like
2.298.855	-0.113214	MYCL	v-myc avian myelocytomatosis viral oncogene l
2.293.072	-0.1496652	MUC5B	mucin 5B, oligomeric mucus/gel-forming
2.287.031	0.1638057	AIP	aryl hydrocarbon receptor interacting protein
2.284.003	-0.0857864		
2.283.546	0.2473729	ZNF22	zinc finger protein 22
2.278.592	-0.3054232	RAB27A	RAB27A, member RAS oncogene family
2.273.683	-0.0934029	LOC64927¹	similar to wingless-type MMTV integration site f
227.346	0.2009103	WSCD1	WSC domain containing 1
2.270.212	-0.1179371	SLC11A1	solute carrier family 11 member 1
2.267.289	-0.0805231	LOC10028¹	immunoglobin superfamily member 21 pseudo
226.599	-0.0929356	PFKFB4	6-phosphofructo-2-kinase/fructose-2,6-biphos
2.264.653	-0.0925833	MFAP3L	microfibrillar associated protein 3 like
2.264.181	-0.4823377	IL6R	interleukin 6 receptor
2.264.087	-0.0853284	CACNA1A	calcium voltage-gated channel subunit alpha1
2.263.226	-0.1832882	TLE2	transducin like enhancer of split 2
2.261.665	0.1765526	NUDT21	nudix hydrolase 21
2.260.599	0.2148517	ABHD14A	abhydrolase domain containing 14A
2.260.121	-0.083957	HOXD1	homeobox D1
2.256.616	-0.1144165	CIITA	class II major histocompatibility complex trans
2.256.416	0.1596694	RPS28P6	ribosomal protein S28 pseudogene 6
2.251.536	-0.0937104	PRKD1	protein kinase D1
2.249.769	-0.1273273	C14orf79	chromosome 14 open reading frame 79
2.248.309	-0.1103553	IL36RN	interleukin 36 receptor antagonist
224.387	-0.1219856	RANBP3	RAN binding protein 3
2.242.508	-0.2997071	LINC00667	long intergenic non-protein coding RNA 667
2.241.293	-0.1373444		
2.239.353	0.1577431	AMBRA1	autophagy and beclin 1 regulator 1
2.239.226	-0.1063146	AJAP1	adherens junctions associated protein 1
2.238.223	-0.0765964	GRIA1	glutamate ionotropic receptor AMPA type subu
2.238.196	-0.3346934	EVL	Enah/Vasp-like
2.238.063	0.1608791	TNPO3	transportin 3
2.236.336	-0.2667832	TCF7L2	transcription factor 7 like 2
2.229.346	0.1495148	PSMC1	proteasome 26S subunit, ATPase 1
2.228.821	0.1693786	GMPPB	GDP-mannose pyrophosphorylase B
2.226.788	0.5019142	CD36	CD36 molecule
2.221.831	-0.1292063	MYF5	myogenic factor 5
2.220.734	0.3516162	ISG20	interferon stimulated exonuclease gene 20
2.219.484	-0.2701573	FCGR3B	Fc fragment of IgG receptor IIIb
2.219.086	-0.2486712		
2.217.294	-0.1293114	KHDRBS2	KH RNA binding domain containing, signal trans

2.217.279	0.1733757	RBM38	RNA binding motif protein 38
2.214.785	0.3432535	ANXA9	annexin A9
2.213.651	-0.0981707	DSC1	desmocollin 1
2.211.961	-0.1617536	IL1A	interleukin 1 alpha
2.208.537	-0.2390669	PARVA	parvin alpha
2.206.488	0.212758	WIPI2	WD repeat domain, phosphoinositide interacti
2.206.443	-0.1521577	PCDH9	protocadherin 9
2.206.357	0.1739245	ACIN1	apoptotic chromatin condensation inducer 1
2.204.358	-0.135114	EPHB3	EPH receptor B3
2.202.542	-0.1186618	ZBTB17	zinc finger and BTB domain containing 17
220.221	-0.1097358	SH3GL3	SH3 domain containing GRB2 like 3, endophilin
2.200.111	-0.2215099	AGAP13P//	ArfGAP with GTPase domain, ankyrin repeat an
2.197.727	-0.0938065	TRIO	trio Rho guanine nucleotide exchange factor
2.193.419	0.3747016	MYO6	myosin VI
2.192.136	0.1046001	L3MBTL1	l(3)mbt-like 1 (Drosophila)
2.191.368	0.2019378	SAR1A	secretion associated Ras related GTPase 1A
2.191.264	-0.4336324	HLA-DPA1	major histocompatibility complex, class II, DP a
2.189.689	0.2677731	KIAA1033	KIAA1033
2.189.305	0.1448033	CENPI	centromere protein I
2.183.994	-0.1943191	FBLN1	fibulin 1
2.183.472	-0.081542		
2.182.845	0.3627728	SCCPDH	saccharopine dehydrogenase (putative)
2.181.285	0.2787299	GUCY2C	guanylate cyclase 2C
21.812	-0.1439518	DOCK5	dedicator of cytokinesis 5
2.179.627	-0.0931151	PRKY	protein kinase, Y-linked, pseudogene
217.955	0.343038	CD59	CD59 molecule
2.169.517	-0.1565545	TPSAB1	tryptase alpha/beta 1
2.168.197	0.1929384	PACS2	phosphofurin acidic cluster sorting protein 2
2.167.811	-0.1111622	SPRED2	sprouty related EVH1 domain containing 2
2.165.446	0.1497623	VPS52	VPS52, GARP complex subunit
2.164.279	-0.1110986		
2.163	-0.1266807	NRP2	neuropilin 2
2.161.362	-0.1125997	DENND2A	DENN domain containing 2A
2.161.035	-0.214961	SNX19	sorting nexin 19
2.159.138	0.2413645	PPIG	peptidylprolyl isomerase G
2.154.551	-0.4688589	HLA-DPA1	major histocompatibility complex, class II, DP a
2.154.157	-0.2754169		
2.150.834	-0.1563664	CDRT4	CMT1A duplicated region transcript 4
2.149.827	0.1527157	TMEM132A	transmembrane protein 132A
2.149.565	0.2685665	PRPF4	pre-mRNA processing factor 4
2.147.418	-0.1505561	PRAMEF2//	PRAME family member 2///PRAME family memb
2.147.417	0.154334	SUPT7L	SPT7-like STAGA complex gamma subunit
214.597	-0.2874726	GSTT2	glutathione S-transferase theta 2 (gene/pseudo
2.142.297	-0.0765379	CFTR	cystic fibrosis transmembrane conductance re

2.141.385	-0.1288459	BAZ2A	bromodomain adjacent to zinc finger domain 2/
2.140.238	0.2028506	TM9SF4	transmembrane 9 superfamily member 4
2.137.624	0.1781392	KLF13	Kruppel like factor 13
2.135.837	0.2771312	RDX	radixin
2.135.764	-0.2504999	GSE1	Gse1 coiled-coil protein
2.129.426	0.1443873	RBM14	RNA binding motif protein 14
2.128.193	-0.1278186		
2.125.936	-0.1399792	PTPRT	protein tyrosine phosphatase, receptor type T
2.125.737	0.1659056	SUPT20H	SPT20 homolog, SAGA complex component
2.123.867	-0.2638302	BNIP3	BCL2 interacting protein 3
2.122.647	-0.1548791	MMP24	matrix metallopeptidase 24
2.121.256	-0.1382784	SYT12	synaptotagmin 12
211.745	-0.3912719	CD74	CD74 molecule
2.116.803	0.2056326	ETV5	ETS variant 5
2.111.979	0.2132478	USP22	ubiquitin specific peptidase 22
2.111.844	0.2718155	CTNNA2	catenin alpha 2
2.109.179	-0.1093976	ADAM18	ADAM metallopeptidase domain 18
2.108.512	-0.0867888	DUS2	dihydrouridine synthase 2
2.106.021	-0.0852347	CHRD	chordin
2.104.816	0.1018219	ROBO2	roundabout guidance receptor 2
2.104.412	-0.2268529	EGFR	epidermal growth factor receptor
2.104.173	-0.0768885		
2.098.448	-0.1175718	POU2F2	POU class 2 homeobox 2
2.096.544	0.2436765	MAGEC1	MAGE family member C1
209.559	-0.1212156	ITGB3	integrin subunit beta 3
2.095.501	0.2800784	SH3BGRL3	SH3 domain binding glutamate rich protein like
209.466	0.1091521	BFSP1	beaded filament structural protein 1
2.088.377	-0.1167101	AP2A2	adaptor related protein complex 2 alpha 2 subu
2.087.485	-0.0966725		
2.087.072	-0.1314993	NR2E3	nuclear receptor subfamily 2 group E member 3
2.080.343	0.1582553	RPL13P5	ribosomal protein L13 pseudogene 5
2.079.967	-0.1229341	BTBD18	BTB domain containing 18
2.079.762	0.3067538	MAN2A1	mannosidase alpha class 2A member 1
2.078.854	0.1782727	SPTLC1	serine palmitoyltransferase long chain base sul
2.078.684	0.1713692	GLTSCR1L	GLTSCR1 like
2.075.756	0.1565426	C14orf2	chromosome 14 open reading frame 2
2.065.053	-0.1278578	PAWR	pro-apoptotic WT1 regulator
2.063.164	-0.1076688	ARSD	arylsulfatase D
2.053.352	-0.2129024	PRKRA	protein activator of interferon induced protein k
2.052.138	-0.0726566	PTPN22	protein tyrosine phosphatase, non-receptor typ
2.049.783	-0.1051662	FARS2	phenylalanyl-tRNA synthetase 2, mitochondrial
204.621	0.2440514	LOC73236(	G/T mismatch-specific thymine DNA glycosylas
2.040.621	-0.094382	ADGB	androglobin
2.038.443	0.0834167	BBS7	Bardet-Biedl syndrome 7

202.923	-0.1088811		TTLL5	tubulin tyrosine ligase like 5
2.027.856	-0.1181743		LOC39255!	MAGE family member C2 pseudogene
2.026.991	0.1971034		MAPK12	mitogen-activated protein kinase 12
2.025.932	-0.4232576		PGRMC1	progesterone receptor membrane component 1
2.022.309	-0.0788048		TSSK1B	testis specific serine kinase 1B
2.019.727	0.2564179		EAPP	E2F associated phosphoprotein
2.017.987	0.3142328		VAMP7	vesicle associated membrane protein 7
201.675	-0.2844965		PHLPP1	PH domain and leucine rich repeat protein phos
2.015.209	0.515821		AKAP12	A-kinase anchoring protein 12
201.309	0.3699738		C14orf1	chromosome 14 open reading frame 1
2.010.744	-0.1918441		STUM	stum, mechanosensory transduction mediator
2.010.307	0.188186		SKP1	S-phase kinase-associated protein 1
2.009.017	0.3322219		CD59	CD59 molecule
2.005.984	0.170153		PPP2R5D	protein phosphatase 2 regulatory subunit B'delt
2.003.883	-0.285598		ZCCHC14	zinc finger CCHC-type containing 14
2.000.715	0.2124137		NDST1	N-deacetylase and N-sulfotransferase 1
1.998.996	-0.1717684		SH2B2	SH2B adaptor protein 2
1.996.853	0.1541908		SPATA2	spermatogenesis associated 2
1.995.568	0.0795837		LRRC23	leucine rich repeat containing 23
1.990.831	-0.1041057		PTCH2	patched 2
1.989.425	-0.2115966		SENP6	SUMO1/sentrin specific peptidase 6
1.988.664	0.3694677		RTP4	receptor transporter protein 4
1.986.907	-0.1426248		TFPI	tissue factor pathway inhibitor
1.982.365	-0.1280683		COBLL1	cordon-bleu WH2 repeat protein like 1
1.981.643	-0.1433663			
1.980.814	-0.1069078		FBP2	fructose-bisphosphatase 2
1.973.728	-0.1339981		AGFG2	ArfGAP with FG repeats 2
1.971.992	-0.2183063		TAOK1	TAO kinase 1
1.970.037	0.2755804		KIAA0930	KIAA0930
1.969.436	-0.1276613		ELP6	elongator acetyltransferase complex subunit 6
1.967.741	0.1352199		SAP30	Sin3A associated protein 30
1.965.196	-0.2274701		AKAP17A	A-kinase anchoring protein 17A
1.964.355	-0.0898676		TRPA1	transient receptor potential cation channel sub
1.962.753	-0.2851418		C2orf72	chromosome 2 open reading frame 72
196.218	-0.1370505		EGFL8	EGF like domain multiple 8
1.953.768	-0.1018353		LOC65187(	similar to HLA class II histocompatibility antiger
1.952.122	0.1342205		BAZ1B	bromodomain adjacent to zinc finger domain 1I
1.951.451	0.1967851		BCS1L	BCS1 homolog, ubiquinol-cytochrome c reduct
1.945.155	-0.1227643		ITGB4	integrin subunit beta 4
1.941.157	-0.1060735			
1.940.398	0.2057134		CAPZB	capping actin protein of muscle Z-line beta sub
193.991	-0.3390295		WIPF1	WAS/WASL interacting protein family member 1
1.936.682	-0.2857734		NADK	NAD kinase
1.935.164	-0.0882919		ZFY	zinc finger protein, Y-linked

1.934.577	-0.0970488	ACAN	aggrecan
1.933.622	-0.3968498	CBR1	carbonyl reductase 1
1.931.617	-0.1130959	OPN1MW2,	opsin 1 (cone pigments), medium-wave-sensiti
1.928.945	-0.0837513	DLGAP2	DLG associated protein 2
1.924.275	0.2931426	TM9SF2	transmembrane 9 superfamily member 2
1.923.885	-0.0873122	PRY	PTPN13-like, Y-linked
1.923.399	-0.0676606	MS4A3	membrane spanning 4-domains A3
1.922.053	0.3014981	ZMYND8	zinc finger MYND-type containing 8
1.920.419	0.2363987	ICE2	interactor of little elongation complex ELL subu
1.919.565	-0.0947773	GLRA1	glycine receptor alpha 1
1.918.234	-0.1083833	PAPOLB	poly(A) polymerase beta
1.916.078	-0.2062005	NPIPA2///N	nuclear pore complex interacting protein family
1.914.999	0.1831393	MRPS22	mitochondrial ribosomal protein S22
1.912.024	-0.0966474	HIST1H3I	histone cluster 1, H3i
1.911.523	0.1907337	ARFGAP1	ADP ribosylation factor GTPase activating prote
1.905.585	-0.1248295	MAST1	microtubule associated serine/threonine kinas
1.899.882	-0.134424	BMP6	bone morphogenetic protein 6
1.899.002	0.1422835	SLC7A1	solute carrier family 7 member 1
1.897.425	0.2107458	C6orf106	chromosome 6 open reading frame 106
1.896.327	0.2867076	RER1	retention in endoplasmic reticulum sorting rece
189.412	0.1650073	APC	APC, WNT signaling pathway regulator
1.893.239	0.2201566	PEX6	peroxisomal biogenesis factor 6
1.887.958	-0.0927951		
1.887.098	-0.2068795	TCF7	transcription factor 7 (T-cell specific, HMG-box
1.885.858	0.2207434	RALGPS1	Ral GEF with PH domain and SH3 binding motif
1.885.435	0.2897698	WIPI1	WD repeat domain, phosphoinositide interactir
1.885.367	-0.4645092	INSIG1	insulin induced gene 1
1.883.828	-0.1652928	DIAPH1	diaphanous related formin 1
1.875.564	0.2350259	FBXL5	F-box and leucine rich repeat protein 5
1.872.369	-0.1452998	LPAR1	lysophosphatidic acid receptor 1
1.870.176	0.365269	RARRES1	retinoic acid receptor responder 1
1.870.165	-0.2576753	TMEM176A	transmembrane protein 176A
1.869.174	0.1330393	ERCC2	ERCC excision repair 2, TFIIH core complex heli
1.868.799	-0.121068	CD96	CD96 molecule
1.867.546	0.1782587	USP7	ubiquitin specific peptidase 7
1.864.012	0.1636639	KIAA0368	KIAA0368
1.861.483	-0.1863291	PLCD1	phospholipase C delta 1
1.857.126	0.3423302	BCLAF1	BCL2 associated transcription factor 1
1.853.715	-0.2085796	SLC25A28	solute carrier family 25 member 28
1.853.488	-0.0872216	FAM135A	family with sequence similarity 135 member A
1.851.811	-0.1195232	AIPL1	aryl hydrocarbon receptor interacting protein lik
1.851.562	0.244256	PIK3CA	phosphatidylinositol-4,5-bisphosphate 3-kinas
1.849.184	0.424007	PAGE4	PAGE family member 4
1.848.376	-0.144083	SOX15	SRY-box 15

1.846.187	-0.359961	CDKN1A	cyclin dependent kinase inhibitor 1A
1.845.506	-0.1656368	ANK2	ankyrin 2, neuronal
184.135	-0.0831674	FUT9	fucosyltransferase 9
1.841.317	-0.1139237	CTRC	chymotrypsin C
1.841.305	-0.1076461	FEV	FEV, ETS transcription factor
1.840.387	-0.2983348	PTER	phosphotriesterase related
1.840.157	-0.3834076	HLA-C	major histocompatibility complex, class I, C
1.835.352	-0.0884183	ASAP1	ArfGAP with SH3 domain, ankyrin repeat and PH
1.833.213	0.35502	GALNT7	polypeptide N-acetylgalactosaminyltransferase
1.831.741	0.250718	ISCA1	iron-sulfur cluster assembly 1
1.831.213	-0.1526817	TMEM59L	transmembrane protein 59 like
1.830.596	-0.2104389	KLF7	Kruppel like factor 7
1.829.848	-0.3869508	NRIP1	nuclear receptor interacting protein 1
1.829.131	0.1867362	COLEC12	collectin subfamily member 12
1.826.366	0.3366153	SCAMP1	secretory carrier membrane protein 1
1.826.265	-0.1454563	HSD3B2	hydroxy-delta-5-steroid dehydrogenase, 3 beta-
182.118	-0.1862736		
1.817.467	0.2020992	DIDO1	death inducer-obliterator 1
181.704	-0.0907091	MS4A2	membrane spanning 4-domains A2
1.816.174	-0.1234895	KIF3C	kinesin family member 3C
1.807.905	-0.0720969	HIPK3	homeodomain interacting protein kinase 3
1.804.221	-0.460451	IFI44	interferon induced protein 44
1.798.194	0.2103717	BIN1	bridging integrator 1
1.798.141	0.1768463	ETV4	ETS variant 4
1.793.263	0.2239966	CDC42BPB	CDC42 binding protein kinase beta
1.782.186	-0.0958751	RNF185	ring finger protein 185
1.778.435	-0.1196811	NEB	nebulin
1.778.287	-0.1426574	DUX4L2///D	double homeobox 4 like 2///double homeobox 4
1.777.504	-0.1021243		
1.776.722	-0.1236038	CACNA1C	calcium voltage-gated channel subunit alpha1 C
1.771.315	-0.1463893	SLC11A1	solute carrier family 11 member 1
1.768.416	0.1635168	SUMO1	small ubiquitin-like modifier 1
1.767.305	-0.1419824	SNPH	syntaphilin
1.766.637	-0.1059063		
176.435	0.2329028	ATP5G3	ATP synthase, H+ transporting, mitochondrial F
1.764.187	0.2267725	PTPRK	protein tyrosine phosphatase, receptor type K
1.761.908	-0.2171629	TREX1	three prime repair exonuclease 1
1.757.729	-0.0920586	SEMG2	semenogelin II
1.755.699	-0.1224065	CDH22	cadherin 22
1.753.703	-0.2225555	ATMIN	ATM interactor
1.752.277	-0.1013929	CR1	complement component 3b/4b receptor 1 (Kno
1.751.682	-0.2661147	PNPLA4	patatin like phospholipase domain containing 4
1.750.899	0.2403909	ACTN2	actinin alpha 2
1.748.257	0.2386828	MARCH7	membrane associated ring-CH-type finger 7

1.737.787	-0.1167491	FLJ12616	hypothetical LOC196707
1.735.065	-0.1087147	PRMT8	protein arginine methyltransferase 8
1.734.982	-0.0985448	DIO2	deiodinase, iodothyronine, type II
1.729.747	-0.1779848	RPS6KA1	ribosomal protein S6 kinase A1
1.727.724	-0.1059871	KIF5A	kinesin family member 5A
1.727.623	-0.2385704	HERC6	HECT and RLD domain containing E3 ubiquitin
1.726.028	0.2775909	MEF2C	myocyte enhancer factor 2C
1.723.331	-0.1171684	LOC65405	similar to Signal-regulatory protein beta-1 precu
1.717.894	-0.1867175	COX4I1	cytochrome c oxidase subunit 4I1
1.716.222	-0.1284043	FPR3	formyl peptide receptor 3
1.715.539	-0.2621966	UBE2G2	ubiquitin conjugating enzyme E2 G2
1.715.464	-0.0880089	PTGER3	prostaglandin E receptor 3
171.187	-0.1136982	NR1D1///TI	nuclear receptor subfamily 1 group D member
1.711.852	0.1772496	TGIF2	TGFB induced factor homeobox 2
1.710.849	-0.1988774	SLC14A2	solute carrier family 14 member 2
1.710.529	-0.3677686	FBXO2	F-box protein 2
1.707.644	-0.0900464	CXCL8	C-X-C motif chemokine ligand 8
1.705.383	-0.1010897	NDST4	N-deacetylase and N-sulfotransferase 4
170.263	0.1654996	CAMK2G	calcium/calmodulin dependent protein kinase
170.252	-0.1558079	PTPN21	protein tyrosine phosphatase, non-receptor typ
170.049	-0.4505059	PPIF	peptidylprolyl isomerase F
1.700.214	0.1520275	ZNF133	zinc finger protein 133
1.698.792	0.1716979	MLLT10	myeloid/lymphoid or mixed-lineage leukemia; t
1.698.775	0.2403986	ARL2BP	ADP ribosylation factor like GTPase 2 binding p
1.698.684	0.2560434	EIF4A1	eukaryotic translation initiation factor 4A1
1.697.685	-0.3719585	CITED2	Cbp/p300 interacting transactivator with Glu/A
1.694.689	0.2492861	WDR19	WD repeat domain 19
169.396	-0.1094203		
1.693.009	-0.0899143	GNAS	GNAS complex locus
1.692.206	0.2616726	ARF3	ADP ribosylation factor 3
1.692.175	0.183074	SUGP2	SURP and G-patch domain containing 2
1.690.792	0.1984981	POLR2E	RNA polymerase II subunit E
1.688.278	0.1370069	RPL3	ribosomal protein L3
1.686.607	-0.2288673	PITPNA	phosphatidylinositol transfer protein alpha
168.588	-0.1357636	POU1F1	POU class 1 homeobox 1
1.685.515	-0.139266	AMPH	amphiphysin
168.374	-0.1017281	SZT2	seizure threshold 2 homolog (mouse)
1.681.445	0.1530464	STC2	stanniocalcin 2
1.679.447	-0.1190828	ALOXE3	arachidonate lipoxygenase 3
1.674.423	-0.113804	GPR85	G protein-coupled receptor 85
1.673.726	-0.0927821		
1.667.753	-0.1054463	HOXD13	homeobox D13
166.498	-0.1508008	FCN1	ficolin 1
1.663.268	-0.1094791	CARD10	caspase recruitment domain family member 1C

1.661.223	-0.0862807	KLF8	Kruppel like factor 8
1.658.752	-0.1261356	PRKG1	protein kinase, cGMP-dependent, type I
1.650.893	-0.1613287	RGS4	regulator of G-protein signaling 4
1.645.055	-0.0782951		
1.644.636	0.2447382	TRMT1L	tRNA methyltransferase 1 like
1.639.707	-0.0973384	HCG2P7	HLA complex group 2 pseudogene 7
162.727	-0.1578232	PDK2	pyruvate dehydrogenase kinase 2
1.626.887	0.2278965	SLC30A9	solute carrier family 30 member 9
162.627	-0.2486901	MZT2B	mitotic spindle organizing protein 2B
1.625.084	0.1387655	ZFX	zinc finger protein, X-linked
1.623.027	-0.0911614	GP1BA	glycoprotein Ib platelet alpha subunit
1.622.462	0.21831	IL7	interleukin 7
1.621.716	0.1879654	FAM120A	family with sequence similarity 120A
1.620.297	0.0964564	HDDC2	HD domain containing 2
1.620.263	-0.1885298	ATPIF1	ATPase inhibitory factor 1
1.620.261	0.1162151	DZIP3	DAZ interacting zinc finger protein 3
1.616.929	-0.0953458	PIK3C3	phosphatidylinositol 3-kinase catalytic subunit
1.614.343	0.2619551	KHNYN	KH and NYN domain containing
1.612.357	-0.172411	ERG	ERG, ETS transcription factor
1.612.096	0.3069271	BMP4	bone morphogenetic protein 4
1.610.196	-0.1103498	PDGFB	platelet derived growth factor subunit B
1.609.906	-0.0892341	ACRV1	acrosomal vesicle protein 1
160.694	-0.0939417	CADM3	cell adhesion molecule 3
1.606.629	-0.0764832	LHCGR	luteinizing hormone/choriogonadotropin recept
1.606.233	-0.208126	TM7SF3	transmembrane 7 superfamily member 3
1.600.585	-0.2012211	OLFM1	olfactomedin 1
1.596.459	-0.0959242	TRMU///PP	tRNA 5-methylaminomethyl-2-thiouridylate me
159.644	-0.2642801	CMTM6	CKLF like MARVEL transmembrane domain con
1.595.751	0.1969891	ARHGAP17	Rho GTPase activating protein 17
159.514	-0.1428492	PSG7	pregnancy specific beta-1-glycoprotein 7 (gene
1.594.373	-0.1015803	NDNF	neuron derived neurotrophic factor
1.593.155	-0.2098187	CPSF7	cleavage and polyadenylation specific factor 7
1.592.306	-0.1192308	ESR1	estrogen receptor 1
1.591.536	-0.147162	ADAM8	ADAM metallopeptidase domain 8
1.589.647	-0.1755433	DENND3	DENN domain containing 3
1.589.423	-0.1366327	RABEP1	rabaptin, RAB GTPase binding effector protein 1
158.844	-0.0948994	SNTG1	syntrophin gamma 1
1.587.239	0.1537785	SPOUT1	SPOUT domain containing methyltransferase 1
1.587.211	0.2056026	PEX7	peroxisomal biogenesis factor 7
1.585.622	-0.1479348	RUNX1	runt related transcription factor 1
1.583.055	-0.1046788		
1.576.135	-0.2183211	KIF22	kinesin family member 22
1.572.042	0.3517563	HPRT1	hypoxanthine phosphoribosyltransferase 1
1.567.264	-0.1243389	KCNG1	potassium voltage-gated channel modifier subf

1.566	-0.1237761	ACAA2	acetyl-CoA acyltransferase 2
1.565.419	0.2215192	ERAP1	endoplasmic reticulum aminopeptidase 1
1.565.183	0.1968681	LRRC41	leucine rich repeat containing 41
1.565.005	0.7587628	GPR88	G protein-coupled receptor 88
1.563.224	-0.2113587	SETD6	SET domain containing 6
1.563.167	-0.0977664	DLEC1	deleted in lung and esophageal cancer 1
1.553.726	-0.0982951	SYNJ2	synaptojanin 2
1.552.865	-0.1103243	CEACAM3	carcinoembryonic antigen related cell adhesior
1.545.587	0.2854788	EFNB2	ephrin B2
1.544.885	-0.2294688	PIK3R4	phosphoinositide-3-kinase regulatory subunit 4
1.544.295	0.297817	DNAJB1	DnaJ heat shock protein family (Hsp40) membe
1.538.178	-0.1891463	IRF4	interferon regulatory factor 4
1.537.559	0.1635352	TNFRSF25	TNF receptor superfamily member 25
1.537.527	0.1562041	NKRF	NFKB repressing factor
1.535.091	-0.3853935	CECR1	cat eye syndrome chromosome region, candida
1.535.003	-0.4213508	HMOX1	heme oxygenase 1
1.534.753	-0.1152453	RBM17	RNA binding motif protein 17
1.534.141	0.1689706	PRR3	proline rich 3
1.529.029	-0.2135466	HIPK2	homeodomain interacting protein kinase 2
1.527.426	0.2158918	RALB	RALB Ras like proto-oncogene B
1.527.405	-0.1033435	FETUB	fetuin B
1.525.212	-0.1605548	MMP25	matrix metallopeptidase 25
1.518.869	-0.0997622	SCNN1B	sodium channel epithelial 1 beta subunit
1.508.038	-0.0867129	LOC64418:	similar to 3 beta-hydroxysteroid dehydrogenase
1.507.697	0.1520351	TMEM268	transmembrane protein 268
1.506.991	0.1600519	NSUN3	NOP2/Sun RNA methyltransferase family meml
1.506.272	0.3111138	TERT	telomerase reverse transcriptase
1.504.873	0.1203422	FAM155B	family with sequence similarity 155 member B
1.503.036	-0.1094995	L3MBTL1	l(3)mbt-like 1 (Drosophila)
1.499.771	-0.2120487	VPS13D	vacuolar protein sorting 13 homolog D
149.748	-0.0980302	IGHA1	immunoglobulin heavy constant alpha 1
1.494.567	-0.0904255	FGF20	fibroblast growth factor 20
1.494.205	-0.1060632	CR1	complement component 3b/4b receptor 1 (Kno
1.492.943	-0.0975965	KRT8P17	keratin 8 pseudogene 17
1.491.804	-0.114613	MTERF4	mitochondrial transcription termination factor 4
149.162	-0.108101	PDCD1	programmed cell death 1
1.491.325	-0.1697228	COX4I1	cytochrome c oxidase subunit 4I1
149.055	-0.0914328	DPYSL4	dihydropyrimidinase like 4
1.484.795	-0.1667707	CDK11A///(	cyclin dependent kinase 11A///cyclin depender
148.447	-0.2627425	CHIC2	cysteine rich hydrophobic domain 2
1.484.008	-0.0963053	DOCK9	dedicator of cytokinesis 9
1.482.973	-0.1427692	VGF	VGF nerve growth factor inducible
1.481.732	0.2613278	PTP4A2	protein tyrosine phosphatase type IVA, member
1.478.566	0.2620785	ACTN1	actinin alpha 1

1.477.993	0.2694266	SHANK2	SH3 and multiple ankyrin repeat domains 2
1.477.525	-0.3409618	NFIB	nuclear factor I B
1.471.065	0.2178318	NR2F6	nuclear receptor subfamily 2 group F member 6
1.466.751	-0.0956662	SPANXC	SPANX family member C
146.613	-0.0988232	NR5A1	nuclear receptor subfamily 5 group A member 1
1.464.319	-0.0823164	PTGIS	prostaglandin I2 (prostacyclin) synthase
1.464.136	-0.2786366	MBNL2	muscleblind like splicing regulator 2
1.462.966	-0.3662345	PPP2R1B	protein phosphatase 2 scaffold subunit Abeta
1.459.544	-0.095852	FGFR2	fibroblast growth factor receptor 2
1.458.313	0.1501536	CDK14	cyclin dependent kinase 14
1.455.456	0.2204211	NAP1L2	nucleosome assembly protein 1 like 2
1.454.151	0.3451089	DEPTOR	DEP domain containing MTOR-interacting prote
1.452.483	0.1452081	CTDSPL	CTD small phosphatase like
1.451.837	-0.1056805		
1.449.565	-0.2775952	MYO15B	myosin XVB
1.447.478	0.186803	DGUOK	deoxyguanosine kinase
1.444.914	-0.213457	PPP1R12B	protein phosphatase 1 regulatory subunit 12B
1.444.671	-0.2870279	TMEM140	transmembrane protein 140
1.444.219	0.1525987	TIMM17A	translocase of inner mitochondrial membrane 1
1.443.445	-0.0957446		
1.442.114	0.1674596	FUT1	fucosyltransferase 1 (H blood group)
1.441.323	-0.2732264	MPP1	membrane palmitoylated protein 1
1.440.943	-0.3741037	PALMD	palmdelphin
1.439.112	0.1157073	TUBGCP5	tubulin gamma complex associated protein 5
1.438.303	-0.20608	ZNF688	zinc finger protein 688
1.437.716	0.0919162	RNMT	RNA guanine-7 methyltransferase
1.433.946	-0.1075217	TNFSF18	tumor necrosis factor superfamily member 18
1.433.418	-0.2727778	CREM	cAMP responsive element modulator
1.431.396	-0.4990721	ARMCX3	armadillo repeat containing, X-linked 3
1.427.786	0.1490993	DCTN5	dynactin subunit 5
1.426.342	0.1319884	SOGA1	suppressor of glucose, autophagy associated 1
1.421.952	-0.1947462	MORC3	MORC family CW-type zinc finger 3
1.421.183	-0.1295225	GSTTP1	glutathione S-transferase theta pseudogene 1
1.420.467	-0.0788678	BMX	BMX non-receptor tyrosine kinase
1.416.998	0.1204654	NUP62CL	nucleoporin 62 C-terminal like
1.414.523	0.2633717	FIGF	c-fos induced growth factor
1.414.362	-0.2812427	USP12	ubiquitin specific peptidase 12
1.413.934	0.1571341	EIF2S1	eukaryotic translation initiation factor 2 subunit
1.413.453	-0.1032363	DNAH7	dynein axonemal heavy chain 7
1.413.274	-0.2932236	SLC43A1	solute carrier family 43 member 1
1.411.767	0.2025582	GNAS	GNAS complex locus
1.411.556	-0.1227902	ESR1	estrogen receptor 1
1.411.102	-0.103738	CCR4	C-C motif chemokine receptor 4
1.411.077	0.1882494	ATP5A1	ATP synthase, H+ transporting, mitochondrial F

141.082	0.1441631	TMEM39B	transmembrane protein 39B
1.407.848	0.1911681	SMTN	smoothelin
140.703	-0.1141536	CAMKK2	calcium/calmodulin dependent protein kinase
1.402.249	-0.1201997	KLRG1	killer cell lectin like receptor G1
140.066	-0.146224	HRASLS2	HRAS like suppressor 2
1.399.521	-0.0983286	TNK2	tyrosine kinase non receptor 2
1.398.021	-0.321523	PXDN	peroxidasin
1.396.487	-0.1314006	ITGA10	integrin subunit alpha 10
1.396.191	-0.4401983		
1.395.931	-0.1342235	FGF17	fibroblast growth factor 17
1.390.374	-0.129722	HOXD10	homeobox D10
1.389.505	0.1942502	RGS19	regulator of G-protein signaling 19
1.388.727	0.3790644	GULP1	GULP, engulfment adaptor PTB domain contain
1.385.547	0.1327564	KAT5	lysine acetyltransferase 5
1.385.335	0.2721351	RYBP	RING1 and YY1 binding protein
138.498	0.1646993	CEP290	centrosomal protein 290
1.383.075	-0.1030285	LILRP2	leukocyte immunoglobulin-like receptor pseud
1.380.892	0.1833836	PDXK	pyridoxal (pyridoxine, vitamin B6) kinase
1.378.822	-0.217448	ZFYVE21	zinc finger FYVE-type containing 21
1.376.002	-0.2953378	LBH	limb bud and heart development
137.348	-0.1152508	BCL2	BCL2, apoptosis regulator
137.069	-0.1331334	TSR3	TSR3, acp transferase ribosome maturation fac
1.370.344	-0.0945739	AP4E1	adaptor related protein complex 4 epsilon 1 sub
1.367.428	-0.1051208	OR52A1	olfactory receptor family 52 subfamily A memb
1.366.585	-0.0914674	ZNF460	zinc finger protein 460
1.366.096	-0.4539916	THBS1	thrombospondin 1
1.363.481	-0.078771	CAPN11	calpain 11
1.360.173	0.1053811	SAR1A	secretion associated Ras related GTPase 1A
1.357.045	-0.1587441	PTGER1	prostaglandin E receptor 1
135.354	-0.0995147	POU3F3	POU class 3 homeobox 3
1.345.083	-0.1191161	TRIM49B///	tripartite motif containing 49B///tripartite motif
1.343.718	-0.137991	CACNA1G	calcium voltage-gated channel subunit alpha1
1.343.505	-0.0813688	KIR3DL1	killer cell immunoglobulin like receptor, three Ig
1.342.355	-0.1480183	AFDN	afadin, adherens junction formation factor
1.342.332	-0.2401533	HCK	HCK proto-oncogene, Src family tyrosine kinase
1.341.989	0.1730423	CEP68	centrosomal protein 68
133.984	-0.2771391	DENND2D	DENN domain containing 2D
1.337.208	0.2211618	RBM8A	RNA binding motif protein 8A
1.332.403	0.1725124	FAM192A	family with sequence similarity 192 member A
1.330.062	0.1679791	LRRFIP2	LRR binding FLII interacting protein 2
1.328.766	-0.1017821	PAX3	paired box 3
1.328.112	0.1784074	C7orf26	chromosome 7 open reading frame 26
1.326.814	0.3312624	SPOCK1	sparc/osteonectin, cwcv and kazal-like domain
1.326.607	-0.2482253	GTF2H3	general transcription factor IIH subunit 3

1.326.583	-0.1020169	ABI3BP	ABI family member 3 binding protein
1.325.056	0.1643362	TNPO3	transportin 3
1.324.876	-0.1138887	NF2	neurofibromin 2
1.322.503	0.2249889	UBXN7	UBX domain protein 7
1.321.041	-0.0933657		
1.319.804	-0.1714742	TMEM63A	transmembrane protein 63A
1.318.898	-0.1162128	UBB	ubiquitin B
1.309.804	-0.1011902	LINC00663	long intergenic non-protein coding RNA 663
1.307.135	0.2365345	ZNF22	zinc finger protein 22
1.305.657	0.2814978	MRPS18B	mitochondrial ribosomal protein S18B
1.302.004	0.3288379	ILF3	interleukin enhancer binding factor 3
130.183	0.1663743	RAP2B///RA	RAP2B, member of RAS oncogene family///RAP
1.300.545	-0.3140534	AK4P3	adenylate kinase 4 pseudogene 3
1.299.042	0.1284035	STX1A	syntaxin 1A
1.294.719	-0.1069382	NOVA2	NOVA alternative splicing regulator 2
1.293.379	0.2296376	RPL31	ribosomal protein L31
1.285.374	-0.1498969	WIPF1	WAS/WASL interacting protein family member 1
1.283.396	0.1229523	PKNOX1	PBX/knotted 1 homeobox 1
1.281.401	-0.1961608	UBR2	ubiquitin protein ligase E3 component n-recogr
1.280.256	0.1739866	HNRNPA0	heterogeneous nuclear ribonucleoprotein A0
128.001	-0.0680016		
127.916	-0.1523156	NPR3	natriuretic peptide receptor 3
1.277.474	0.3265541	SNCAIP	synuclein alpha interacting protein
1.274.995	0.1406771	HUS1	HUS1 checkpoint clamp component
1.273.864	-0.1764084	SEMA6C	semaphorin 6C
1.273.222	0.2241255	MAX	MYC associated factor X
1.272.254	-0.1747641		
1.269.485	-0.1337664	MUC5AC	mucin 5AC, oligomeric mucus/gel-forming
1.266.852	0.1871422	RNF114	ring finger protein 114
126.641	-0.2548486	KCNB1	potassium voltage-gated channel subfamily B r
1.266.183	0.249684	BTNL8	butyrophilin like 8
1.265.665	-0.133732	DLX2	distal-less homeobox 2
1.264.979	-0.119858	CD47	CD47 molecule
1.264.707	-0.0808228	BRINP1	BMP/retinoic acid inducible neural specific 1
1.264.261	0.2653479	RNF14	ring finger protein 14
1.263.066	-0.1319783	NTN3	netrin 3
1.256.377	-0.1619796	CCR7	C-C motif chemokine receptor 7
1.254.066	-0.134686	IL9RP3///IL	interleukin 9 receptor pseudogene 3///interleuk
1.252.345	-0.2198604	MGRN1	mahogunin ring finger 1
1.251.654	-0.1101735	GPR45	G protein-coupled receptor 45
1.251.625	-0.115482		
1.250.183	-0.0990807	HRH3	histamine receptor H3
1.246.552	-0.289334	KANK1	KN motif and ankyrin repeat domains 1
1.246.134	0.2266478	STK38	serine/threonine kinase 38

1.243.557	0.4900005	ZNF331	zinc finger protein 331
1.240.904	-0.1348816	ALDH3B1	aldehyde dehydrogenase 3 family member B1
1.240.564	-0.1000483	HYDIN	HYDIN, axonemal central pair apparatus protei
1.238.886	-0.0870606	C15orf31	chromosome 15 open reading frame 31
1.236.145	-0.0787429		
1.230.836	-0.1041716	GPATCH4	G-patch domain containing 4
1.227.709	-0.1114499	DCSTAMP	dendrocyte expressed seven transmembrane p
1.225.898	0.1864178	BIRC2	baculoviral IAP repeat containing 2
1.224.234	-0.1203535	RCC1	regulator of chromosome condensation 1
122.123	-0.1517038	MAP4K1	mitogen-activated protein kinase kinase kinase
1.219.925	-0.1118344	TREML2	triggering receptor expressed on myeloid cells l
1.218.137	-0.1409774	ERCC4	ERCC excision repair 4, endonuclease catalytic
1.216.862	0.2122402	PSMD7	proteasome 26S subunit, non-ATPase 7
1.212.996	-0.3234801	SRSF11	serine and arginine rich splicing factor 11
1.211.272	-0.0956789	EXO5	exonuclease 5
1.210.392	-0.1533166	PDPN	podoplanin
1.205.308	-0.0939085	CSNK1G1	casein kinase 1 gamma 1
1.203.871	0.2095984	KRR1	KRR1, small subunit processome component h
1.201.219	0.2215234	TRAK1	trafficking kinesin protein 1
1.199.678	-0.1860329	PRDX6	peroxiredoxin 6
119.945	0.3011645	NFYB	nuclear transcription factor Y subunit beta
1.197.748	0.1817743	GIT2	GIT ArfGAP 2
1.195.244	-0.1362616	ASCL1	achaete-scute family bHLH transcription factor
1.191.369	-0.0816058	ABHD6	abhydrolase domain containing 6
1.190.843	-0.1006079	ENO1B	enolase alpha, lung-specific
1.189.521	-0.0937358	SHH	sonic hedgehog
1.189.341	-0.1663087	RAPGEF2	Rap guanine nucleotide exchange factor 2
1.188.714	-0.0959434	SLC2A3	solute carrier family 2 member 3
1.183.869	0.1933268	CA12	carbonic anhydrase 12
1.183.427	-0.0923793	HLA-C	major histocompatibility complex, class I, C
1.182.488	-0.1211232	STK38	serine/threonine kinase 38
1.182.441	0.1738675	MXD4	MAX dimerization protein 4
1.178.573	0.2275085	GMPR2	guanosine monophosphate reductase 2
1.178.181	-0.0837044	EFR3B	EFR3 homolog B
1.177.673	0.184897	NCOA1	nuclear receptor coactivator 1
1.175.722	-0.1180694	FOSL2	FOS like 2, AP-1 transcription factor subunit
117.256	-0.2140717	PPP3CA	protein phosphatase 3 catalytic subunit alpha
1.171.151	-0.0787303	TRIM24	tripartite motif containing 24
1.169.159	0.2374659	ATP6V1G1	ATPase H+ transporting V1 subunit G1
1.158.265	-0.1386695	SMURF1	SMAD specific E3 ubiquitin protein ligase 1
1.154.808	0.1227156	HIVEP2	human immunodeficiency virus type I enhance
1.153.484	-0.0894804	KCNAB3	potassium voltage-gated channel subfamily A r
1.152	-0.1328072	ADGRB3	adhesion G protein-coupled receptor B3
1.149.114	-0.0808115	DR1	down-regulator of transcription 1

1.148.838	-0.384702	PRNP	prion protein
1.146.638	-0.0805592	NCR2	natural cytotoxicity triggering receptor 2
1.142.312	-0.1093411	C19orf57	chromosome 19 open reading frame 57
1.140.474	0.3699558	PLPP1	phospholipid phosphatase 1
11.386	-0.1004688	DLGAP1	DLG associated protein 1
1.136.536	-0.091026	AREG	amphiregulin
1.131.849	0.2377948	BAZ1A	bromodomain adjacent to zinc finger domain 1/
1.129.146	-0.2590785	CC2D1A	coiled-coil and C2 domain containing 1A
1.128.866	-0.0972852	AKAP1	A-kinase anchoring protein 1
1.127.522	-0.1140495	VPS13C	vacuolar protein sorting 13 homolog C
1.126.426	-0.0884964	CTDSPL	CTD small phosphatase like
1.123.325	0.1128863	E2F1	E2F transcription factor 1
1.122.959	-0.1099571	MAU2	MAU2 sister chromatid cohesion factor
1.122.543	-0.0916768	LEFTY2	left-right determination factor 2
1.119.947	-0.3752101	IGFBP7	insulin like growth factor binding protein 7
1.119.029	-0.0794404	LRRC37A4I	leucine rich repeat containing 37 member A4, p
1.118.821	-0.1931198	SERPINB8	serpin family B member 8
1.115.545	0.27415	NTAN1	N-terminal asparagine amidase
1.115.023	0.2695425	TACC1	transforming acidic coiled-coil containing prote
111.449	0.2221903	SPSB1	splA/ryanodine receptor domain and SOCS box
1.112.619	-0.2298138	SRPRA	SRP receptor alpha subunit
1.110.225	-0.2315938	DCAF8	DDB1 and CUL4 associated factor 8
1.100.823	-0.1050282	CDH8	cadherin 8
1.098.721	-0.1049203	PARK2	parkin RBR E3 ubiquitin protein ligase
1.095.901	-0.1767969	NDUFC1	NADH:ubiquinone oxidoreductase subunit C1
1.094.387	0.2902133	SYT1	synaptotagmin 1
1.093.194	-0.1030977	SLC6A4	solute carrier family 6 member 4
1.091.572	0.1944672	MAN2B1	mannosidase alpha class 2B member 1
1.088.077	-0.141252	CLSPN	claspin
1.087.219	-0.174381	PCNX1	pecanex homolog 1 (Drosophila)
1.086.551	0.1237279	ZBED4	zinc finger BED-type containing 4
1.085.504	-0.0799301	LOC10192(	uncharacterized LOC101926997
1.085.185	-0.2536419	SEC16A	SEC16 homolog A, endoplasmic reticulum expo
1.084.948	0.2309735	PIKFYVE	phosphoinositide kinase, FYVE-type zinc finger
1.084.479	-0.0922802	FPR1	formyl peptide receptor 1
1.079.065	0.1840712	IPP	intracisternal A particle-promoted polypeptide
1.077.984	-0.1331868	DES	desmin
107.648	-0.1436064	BLK	BLK proto-oncogene, Src family tyrosine kinase
1.070.607	-0.1069276	TBXA2R	thromboxane A2 receptor
1.068.931	-0.1334802	SH3BP1	SH3 domain binding protein 1
1.066.976	-0.0956337	NNT	nicotinamide nucleotide transhydrogenase
1.061.377	-0.1190346	ZNF287	zinc finger protein 287
1.060.593	0.088001	CBFA2T2	CBFA2/RUNX1 translocation partner 2
1.060.298	0.2556419	MBD4	methyl-CpG binding domain 4, DNA glycosylase

1.058.843	-0.0884289	DNM2	dynamin 2
1.057.423	-0.1168385	RGS10	regulator of G-protein signaling 10
1.051.922	-0.4892002	EIF5A	eukaryotic translation initiation factor 5A
1.051.356	-0.077161	KCNJ12	potassium voltage-gated channel subfamily J m
1.047.956	0.3035771	OXCT1	3-oxoacid CoA-transferase 1
10.471	0.0861075	HIST1H3G	histone cluster 1, H3g
1.045.693	-0.1260065	CLEC4A	C-type lectin domain family 4 member A
1.033.045	0.2798433	NEDD9	neural precursor cell expressed, developmenta
1.032.524	-0.1114706	CGB7///CG	chorionic gonadotropin beta subunit 7///chorio
1.027.563	-0.1036007	FLJ12547	hypothetical protein FLJ12547
102.721	-0.0834318	BRD8	bromodomain containing 8
1.026.062	-0.1158825	FAM46C	family with sequence similarity 46 member C
1.025.853	-0.1312348		
1.025.243	-0.1698602	TBCD	tubulin folding cofactor D
1.023.928	-0.2488749	LRRFIP1	LRR binding FLII interacting protein 1
1.020.161	-0.1416342	ETV7	ETS variant 7
1.017.763	-0.23342	PHACTR4	phosphatase and actin regulator 4
1.010.599	0.2474244	PAICS	phosphoribosylaminoimidazole carboxylase; pl
1.006.295	-0.1040058	MYCN	v-myc avian myelocytomatosis viral oncogene r
1.006.035	-0.1057126	NUP188	nucleoporin 188
1.005.775	-0.2316262	AK2	adenylate kinase 2
1.005.589	0.1814867	DSTN	destrin, actin depolymerizing factor
1.005.153	0.1205497	C15orf39	chromosome 15 open reading frame 39
1.004.626	-0.0876294	RHAG	Rh-associated glycoprotein
1.004.118	-0.1048602	HIC1	hypermethylated in cancer 1
1.001.264	0.3194424	AGPAT5	1-acylglycerol-3-phosphate O-acyltransferase !
0.999418	-0.1040344	SLC15A2	solute carrier family 15 member 2
0.99794	0.4202061	FBN1	fibrillin 1
0.997118	0.1900508	DCP1A	decapping mRNA 1A
0.994148	-0.1110528		
0.993939	-0.2621442	TBL1X	transducin (beta)-like 1X-linked
0.993803	0.2175906	TOR1AIP1	torsin 1A interacting protein 1
0.993435	-0.2202892	ABTB2	ankyrin repeat and BTB domain containing 2
0.985627	-0.0908004	GABRA5	gamma-aminobutyric acid type A receptor alph
0.984722	-0.0932451	TAF6L	TATA-box binding protein associated factor 6 lik
0.982275	-0.1040726	KRT1	keratin 1
0.981502	0.2256184		
0.980833	-0.2543294	APOB	apolipoprotein B
0.979347	-0.1113081	CCDC71	coiled-coil domain containing 71
0.978988	0.2275776	CHMP1B	charged multivesicular body protein 1B
0.977846	-0.134928	SS18	SS18, nBAF chromatin remodeling complex sub
0.977495	-0.1078601	KCNC3	potassium voltage-gated channel subfamily C r
0.969996	-0.1180247	PCSK6	proprotein convertase subtilisin/kexin type 6
0.969145	-0.2224744	GK3P	glycerol kinase 3 pseudogene

0.968632	0.188497	WDR1	WD repeat domain 1
0.968306	-0.0846987	ADAM2	ADAM metallopeptidase domain 2
0.965888	-0.1233034	TM6SF2	transmembrane 6 superfamily member 2
0.9648	0.1449856	CCDC94	coiled-coil domain containing 94
0.963974	-0.1565316	PLSCR3	phospholipid scramblase 3
0.962282	-0.0900858	TRIM2	tripartite motif containing 2
0.962225	0.1773704	PATZ1	POZ/BTB and AT hook containing zinc finger 1
0.959492	-0.1070993	NCAPH2	non-SMC condensin II complex subunit H2
0.957908	-0.0964516	IL1R1	interleukin 1 receptor type 1
0.955952	0.1655577	TBC1D10B	TBC1 domain family member 10B
0.955329	-0.0879689	RBM48	RNA binding motif protein 48
0.949731	-0.2107762	PHB2	prohibitin 2
0.948579	0.2378523	HLA-A	major histocompatibility complex, class I, A
0.943767	-0.0981107		
0.943078	-0.3193804		
0.94232	-0.2276491	MPST	mercaptopyruvate sulfurtransferase
0.941371	-0.0805331	MEF2C	myocyte enhancer factor 2C
0.940295	-0.2820208	KLHDC10	kelch domain containing 10
0.940245	0.2661823	KPNA3	karyopherin subunit alpha 3
0.938124	0.1447556	TDRD3	tudor domain containing 3
0.936793	-0.1753299	CALML4	calmodulin like 4
0.936726	0.2175212	LOX	lysyl oxidase
0.936266	-0.3710219	NFIL3	nuclear factor, interleukin 3 regulated
0.935471	0.2590587	PNN	pinin, desmosome associated protein
0.932789	0.1051821	B3GALNT1	beta-1,3-N-acetylgalactosaminyltransferase 1 (
0.931401	-0.1264832	CA11	carbonic anhydrase 11
0.931073	-0.0874871	FGF8	fibroblast growth factor 8
0.927209	0.2007042	BCCIP	BRCA2 and CDKN1A interacting protein
0.926097	-0.086234	PSG2	pregnancy specific beta-1-glycoprotein 2
0.924327	-0.085067	PCDHGB6	protocadherin gamma subfamily B, 6
0.923871	0.1363264	TRIM31	tripartite motif containing 31
0.923122	0.2101311	MSL2	male-specific lethal 2 homolog (Drosophila)
0.922643	-0.291549	UBE2L6	ubiquitin conjugating enzyme E2 L6
0.919308	-0.1827725	CELF2	CUGBP, Elav-like family member 2
0.91809	0.2179895	IFITM3	interferon induced transmembrane protein 3
0.917505	0.1699159	ZMYM4	zinc finger MYM-type containing 4
0.916153	0.1965854	APOOL	apolipoprotein O like
0.914796	-0.1569655	IGLL1	immunoglobulin lambda like polypeptide 1
0.91308	-0.1508243	MARCH6	membrane associated ring-CH-type finger 6
0.911699	-0.1191625	TRIM3	tripartite motif containing 3
0.904844	0.2089837	IFT20	intraflagellar transport 20
0.903298	-0.1072293	DRD2	dopamine receptor D2
0.901294	0.1982073	ST6GALNA(	ST6 N-acetylgalactosaminide alpha-2,6-sialyltr
0.900446	0.2307151	PPFIA1	PTPRF interacting protein alpha 1

0.89784	0.1443023	ZBTB17	zinc finger and BTB domain containing 17
0.896264	-0.1321325		
0.895986	0.0882133	TTC26	tetratricopeptide repeat domain 26
0.893084	0.2004564	ATG4B	autophagy related 4B cysteine peptidase
0.891812	-0.1168548	ZNF556	zinc finger protein 556
0.891804	-0.1127826	GYPA	glycophorin A (MNS blood group)
0.887311	-0.3362096	CALD1	caldesmon 1
0.887305	-0.1364503		
0.886077	0.130178	KAT5	lysine acetyltransferase 5
0.885764	-0.0921235	SLURP1	secreted LY6/PLAUR domain containing 1
0.884598	0.2249689	GART	phosphoribosylglycinamide formyltransferase,
0.883491	0.2200643	SNAP23	synaptosome associated protein 23
0.882286	0.1216866	KIF2A	kinesin family member 2A
0.881121	-0.1369981		
0.880185	-0.2539095	IFI44	interferon induced protein 44
0.880085	0.1885015	OXSR1	oxidative stress responsive 1
0.879988	0.1504877	EAF2	ELL associated factor 2
0.879783	0.8878067	EPCAM	epithelial cell adhesion molecule
0.879069	-0.2184014	AP1G1	adaptor related protein complex 1 gamma 1 sul
0.875902	0.2671342	NFYB	nuclear transcription factor Y subunit beta
0.875322	-0.1109689	SCN2B	sodium voltage-gated channel beta subunit 2
0.872859	-0.0827209	GHRH	growth hormone releasing hormone
0.870049	-0.4469453	IL6ST	interleukin 6 signal transducer
0.869531	-0.0874895	TRAF3IP2	TRAF3 interacting protein 2
0.868497	-0.3727079	MMP2	matrix metallopeptidase 2
0.866572	-0.2630516	PRKCH	protein kinase C eta
0.864987	0.1411731	SLC16A3	solute carrier family 16 member 3
0.864552	-0.1809479	PDE1A	phosphodiesterase 1A
0.864316	-0.1025748	CRISP1	cysteine rich secretory protein 1
0.863924	0.1841229	SPG11	spastic paraplegia 11 (autosomal recessive)
0.860886	-0.1023861	ADORA1	adenosine A1 receptor
0.860398	-0.0827476	ZIC4	Zic family member 4
0.860198	0.1327978	LRIG2	leucine rich repeats and immunoglobulin like d
0.855037	-0.1010578	B4GALT1	beta-1,4-galactosyltransferase 1
0.853318	-0.2270085	ARHGAP29	Rho GTPase activating protein 29///myosin hea
0.851595	-0.1100928	SHPK///TRF	sedoheptulokinase///transient receptor potenti
0.848622	-0.0935002	PPFIBP1	PPFIA binding protein 1
0.845794	0.4716297	ACTB	actin beta
0.840465	0.1311848	TBC1D30	TBC1 domain family member 30
0.836742	0.2110866	ANKRA2	ankyrin repeat family A member 2
0.835102	-0.1026577	NRAP	nebulin related anchoring protein
0.834486	-0.1119691	RAD50	RAD50 double strand break repair protein
0.832838	-0.1102021	C14orf113	chromosome 14 open reading frame 113
0.832013	0.1129581	L3MBTL1	l(3)mbt-like 1 (Drosophila)

0.8315	0.261908	QPCT	glutaminyl-peptide cyclotransferase
0.829191	-0.1706681	PLEKHM1P	pleckstrin homology and RUN domain containi
0.826632	-0.1110474	VPREB3	pre-B lymphocyte 3
0.821774	-0.07888	FLJ10232	hypothetical protein FLJ10232
0.82117	-0.1144234	HOXA4	homeobox A4
0.820762	-0.136242	CCL22	C-C motif chemokine ligand 22
0.818523	-0.0719606	PCCA	propionyl-CoA carboxylase alpha subunit
0.818056	-0.1145335	DAB1	DAB1, reelin adaptor protein
0.815248	-0.2810602	CEACAM6	carcinoembryonic antigen related cell adhesio
0.814874	0.0872079	ANXA2P1	annexin A2 pseudogene 1
0.814445	-0.0752159	GLP1R	glucagon like peptide 1 receptor
0.811027	-0.1241054	CEMP1	cementum protein 1
0.809615	0.1552113	ATP7A	ATPase copper transporting alpha
0.807337	0.2903594	FLNC	filamin C
0.799266	0.2182368	ADAM23	ADAM metallopeptidase domain 23
0.796524	-0.1020644	TNKS	tankyrase
0.795348	0.1259382	PRKAA2	protein kinase AMP-activated catalytic subunit
0.794834	-0.1508277	TMEM222	transmembrane protein 222
0.793346	-0.0516535	GUCA1A	guanylate cyclase activator 1A
0.793282	-0.1448812	RNF126P1	ring finger protein 126 pseudogene 1
0.792789	-0.3124053	CYBB	cytochrome b-245 beta chain
0.79261	0.1399662	DPY19L2P2	DPY19L2 pseudogene 2
0.791874	-0.093428	SYN1	synapsin I
0.791093	-0.0798968	WSB1	WD repeat and SOCS box containing 1
0.789822	0.2210089	ITPR1	inositol 1,4,5-trisphosphate receptor type 1
0.784439	-0.1204339	PART1	prostate androgen-regulated transcript 1 (non-[illegible]
0.783542	-0.0926706	PELI1	pellino E3 ubiquitin protein ligase 1
0.783292	-0.0975487	GRIN1	glutamate ionotropic receptor NMDA type subu
0.783291	0.1994222	RBPJ	recombination signal binding protein for immu
0.778131	0.2530121	SEC14L1	SEC14 like lipid binding 1
0.77616	-0.116964	KCNJ5	potassium voltage-gated channel subfamily J m
0.775073	0.2252096	SSR4	signal sequence receptor subunit 4
0.774197	-0.0904205	PTGDR2	prostaglandin D2 receptor 2
0.773953	-0.0724538		
0.773859	0.1853203	NSMCE2	NSE2/MMS21 homolog, SMC5-SMC6 complex [illegible]
0.771436	0.1682988	KLHL22	kelch like family member 22
0.768964	0.2109098	HCAR3	hydroxycarboxylic acid receptor 3
0.767448	-0.0894804	EFCAB1	EF-hand calcium binding domain 1
0.76529	-0.3679759	ITPR2	inositol 1,4,5-trisphosphate receptor type 2
0.765174	-0.1331087	FLJ10246	FLJ10246
0.761579	-0.2107813	CALD1	caldesmon 1
0.76002	0.08517	COX11P1//	COX11, cytochrome c oxidase copper chapero
0.758927	-0.1338421	POU5F1P4/	POU class 5 homeobox 1 pseudogene 4///POU
0.758733	-0.0933788	GNAT1	G protein subunit alpha transducin 1

0.758672	-0.1199598	CLDN6	claudin 6
0.757299	-0.260767	MICAL2	microtubule associated monooxygenase, calpc
0.755362	0.1664542	NFATC4	nuclear factor of activated T-cells 4
0.753809	-0.0912792	NHLH1	nescient helix-loop-helix 1
0.750802	0.3125718	C1QL1	complement C1q like 1
0.749566	-0.1435313	GOSR1	golgi SNAP receptor complex member 1
0.748504	0.2157791	UQCRB	ubiquinol-cytochrome c reductase binding prot
0.748009	-0.0934364	SPTB	spectrin beta, erythrocytic
0.74671	0.2141701	GPS2	G protein pathway suppressor 2
0.746629	0.159162	DEAF1	DEAF1, transcription factor
0.745666	0.2584319	MCFD2	multiple coagulation factor deficiency 2
0.744597	-0.1360586	SPDEF	SAM pointed domain containing ETS transcripti
0.738005	0.1962863	OSR2	odd-skipped related transciption factor 2
0.737774	0.3734791	TM4SF5	transmembrane 4 L six family member 5
0.736467	-0.0819729	APOBEC3F	apolipoprotein B mRNA editing enzyme catalyti
0.734704	-0.0738278	DGKB	diacylglycerol kinase beta
0.729918	0.1379751	PRPF38B	pre-mRNA processing factor 38B
0.724118	-0.0905597	RARB	retinoic acid receptor beta
0.723447	-0.2523316	NR1D1///TI	nuclear receptor subfamily 1 group D member 1
0.7215	0.2260192	HNRNPUL2	heterogeneous nuclear ribonucleoprotein U like
0.719507	-0.1041148	TSPY1	testis specific protein, Y-linked 1
0.71369	-0.0782713	CASP4	caspase 4
0.711726	-0.129096	TBC1D30	TBC1 domain family member 30
0.709253	-0.13829	SUPT20H	SPT20 homolog, SAGA complex component
0.709048	0.1785483	KIDINS220	kinase D-interacting substrate 220kDa
0.706191	-0.087544	GJA5	gap junction protein alpha 5
0.704229	-0.2243489	LOC73121(	similar to Protein MICAL-3///microtubule assoc
0.703751	-0.2819357	ALAD	aminolevulinate dehydratase
0.698778	-0.1887547		
0.698093	-0.2684222	OAS2	2'-5'-oligoadenylate synthetase 2
0.69623	-0.1026771	MAGI2	membrane associated guanylate kinase, WW a
0.694347	-0.147082	WWP2	WW domain containing E3 ubiquitin protein liga
0.692656	-0.075873	GPC1	glypican 1
0.692543	0.2523987	GALK1	galactokinase 1
0.69082	-0.0904921	TP73	tumor protein p73
0.684845	-0.1023986	C16orf72	chromosome 16 open reading frame 72
0.68242	-0.1226204	SMC1A	structural maintenance of chromosomes 1A
0.680622	0.1554174	TMEM255A	transmembrane protein 255A
0.673581	-0.1773322	TCAF1	TRPM8 channel associated factor 1
0.672912	-0.1584956	MAPK8	mitogen-activated protein kinase 8
0.67247	-0.1318191	IBTK	inhibitor of Bruton tyrosine kinase
0.672058	0.1355162	GPATCH3	G-patch domain containing 3
0.66955	0.1834021	FNBP1	formin binding protein 1
0.669527	0.175493	BCAS1	breast carcinoma amplified sequence 1

0.663498	-0.1063671	TRA	T-cell receptor alpha locus
0.66186	-0.084608	CYB561	cytochrome b561
0.659205	0.1698405	MAP2	microtubule associated protein 2
0.655825	-0.0846736	FCRL2	Fc receptor like 2
0.655051	0.1799137	BCL7C	BCL tumor suppressor 7C
0.654632	-0.1230769	CRYGA	crystallin gamma A
0.654206	-0.0978285	RAB11FIP3	RAB11 family interacting protein 3
0.653961	-0.0770114	GPR50	G protein-coupled receptor 50
0.653858	0.2554869	FBXL7	F-box and leucine rich repeat protein 7
0.653269	-0.0981742	NGB	neuroglobin
0.65027	0.3046981	C1QBP	complement C1q binding protein
0.649524	-0.1118748	CACNB1	calcium voltage-gated channel auxiliary subuni
0.648679	-0.1893971		
0.647065	0.2087998	DNASE2	deoxyribonuclease 2, lysosomal
0.642236	-0.1855089	KIAA0319L	KIAA0319 like
0.641482	0.0988768	ZNF74	zinc finger protein 74
0.639064	-0.2364533	KLF4	Kruppel like factor 4
0.638206	-0.2270616	ILVBL	ilvB acetolactate synthase like
0.637815	-0.0869305	E2F2	E2F transcription factor 2
0.636246	-0.1349427	BMP8B	bone morphogenetic protein 8b
0.634885	-0.2059238	SHB	SH2 domain containing adaptor protein B
0.634825	-0.2443901	ETFB	electron transfer flavoprotein beta subunit
0.62833	0.1095765	NPAS2	neuronal PAS domain protein 2
0.627979	-0.0950393	ABI2	abl-interactor 2
0.625247	0.6093236	MAFF	MAF bZIP transcription factor F
0.624563	-0.1635369	ARHGEF1	Rho guanine nucleotide exchange factor 1
0.624388	-0.2047369	WDR7	WD repeat domain 7
0.623536	0.3799682	PSMB9	proteasome subunit beta 9
0.622452	-0.0852956	DLGAP1	DLG associated protein 1
0.618154	-0.0878278	PSG5	pregnancy specific beta-1-glycoprotein 5
0.617224	-0.1424447	ABCA4	ATP binding cassette subfamily A member 4
0.616649	-0.1057873	DCLK2	doublecortin like kinase 2
0.615929	-0.0961452	RPL21P18/	ribosomal protein L21 pseudogene 18///riboso
0.610357	0.2052148	SATL1	spermidine/spermine N1-acetyl transferase-lik
0.609109	-0.0943401	STXBP6	syntaxin binding protein 6
0.608746	-0.0845084	CEACAM4	carcinoembryonic antigen related cell adhesior
0.607978	-0.179694	CNBP	CCHC-type zinc finger nucleic acid binding prot
0.605477	-0.1827203	ABHD5	abhydrolase domain containing 5
0.602073	-0.1253298	GDPD2	glycerophosphodiester phosphodiesterase don
0.599023	-0.1208652	SCN3B	sodium voltage-gated channel beta subunit 3
0.598871	-0.1105863	SNX1	sorting nexin 1
0.596599	0.1971422	SMARCD2	SWI/SNF related, matrix associated, actin depe
0.593604	-0.1342949	BMS1P20//	BMS1, ribosome biogenesis factor pseudogene
0.593549	0.1588066	SIAH1	siah E3 ubiquitin protein ligase 1

0.592268	-0.1588157	LAMC2	laminin subunit gamma 2
0.590001	-0.1845381	NTN3	netrin 3
0.588624	-0.1185055	RRP1B	ribosomal RNA processing 1B
0.586882	-0.0878956		
0.586501	-0.1312444		
0.581422	-0.1229985	MUC3A	mucin 3A, cell surface associated
0.579384	-0.3883967	ST6GAL1	ST6 beta-galactoside alpha-2,6-sialyltransferas
0.578779	-0.0816926	CAMK2D	calcium/calmodulin dependent protein kinase
0.576555	-0.2228718	HHLA3	HERV-H LTR-associating 3
0.572498	-0.1596614	ADRA2A	adrenoceptor alpha 2A
0.572035	0.3298132	NRP1	neuropilin 1
0.565736	-0.0821475	PTGER3	prostaglandin E receptor 3
0.564768	-0.2744035	PPP1R10	protein phosphatase 1 regulatory subunit 10
0.563115	-0.1377837	ST7L	suppression of tumorigenicity 7 like
0.560889	-0.1305825	PAFAH2	platelet activating factor acetylhydrolase 2
0.560166	0.0817757	PKNOX1	PBX/knotted 1 homeobox 1
0.559282	-0.2091221	LOC64529	hypothetical LOC645291///C-terminal binding
0.559124	0.0840928	LNPEP	leucyl and cystinyl aminopeptidase
0.557783	-0.2283379	SIK2	salt inducible kinase 2
0.55611	0.1282836	ARIH2	ariadne RBR E3 ubiquitin protein ligase 2
0.555255	-0.1417182	RASSF9	Ras association domain family member 9
0.554455	-0.1068002		
0.5511	0.1732308	IMPDH1	inosine monophosphate dehydrogenase 1
0.548927	-0.089438	LOC10192	uncharacterized LOC101926997
0.54611	-0.2300386	SLIT2	slit guidance ligand 2
0.5447	-0.0954	LOC65214	u5 small nuclear ribonucleoprotein 200 kDa he
0.54448	0.2319567	PPIE	peptidylprolyl isomerase E
0.544431	0.2259713	VPS13C	vacuolar protein sorting 13 homolog C
0.543207	-0.0923731	SLC12A6	solute carrier family 12 member 6
0.536692	-0.1657023	CACFD1	calcium channel flower domain containing 1
0.533986	-0.262661	MTCP1	mature T-cell proliferation 1
0.533492	-0.0779962	LIMK1	LIM domain kinase 1
0.533197	-0.0782877	MYRF	myelin regulatory factor
0.532735	0.3939762	CDH11	cadherin 11
0.532713	-0.2105886	AGAP5///A	ArfGAP with GTPase domain, ankyrin repeat an
0.531429	0.1574941	ZSCAN26	zinc finger and SCAN domain containing 26
0.530309	-0.0894487	KIAA0319	KIAA0319
0.529996	0.2720073	SIRPA	signal regulatory protein alpha
0.529712	-0.2014415	FBXO31	F-box protein 31
0.529202	0.2871771	OXR1	oxidation resistance 1
0.528459	-0.0838936	RUNX1T1	RUNX1 translocation partner 1
0.526169	-0.1102399	ADCY3	adenylate cyclase 3
0.526096	-0.0967944	MMP26	matrix metallopeptidase 26
0.524848	-0.1017134	IL5	interleukin 5

0.523672	-0.1604918	TRIM2	tripartite motif containing 2
0.521106	-0.1082403	SAG	S-antigen visual arrestin
0.520841	0.1302704	MAP4	microtubule associated protein 4
0.51948	0.1225587	KRT8P12	keratin 8 pseudogene 12
0.518858	-0.0922489	GJB3	gap junction protein beta 3
0.517081	-0.1036989	RARA	retinoic acid receptor alpha
0.511664	-0.1017119	SLC14A1	solute carrier family 14 member 1 (Kidd blood g
0.510583	-0.1358635	AKT2	AKT serine/threonine kinase 2
0.508959	0.1811408	PCGF2	polycomb group ring finger 2
0.505597	-0.1435206	ARID5A	AT-rich interaction domain 5A
0.502952	0.1281233	NPEPL1	aminopeptidase-like 1
0.502519	-0.0843425	KCNJ10	potassium voltage-gated channel subfamily J m
0.502209	-0.0754634	GLRA3	glycine receptor alpha 3
0.501034	-0.1070359	CYSLTR2	cysteinyl leukotriene receptor 2
0.500988	-0.1102491	TENM4	teneurin transmembrane protein 4
0.49625	-0.3095452	PYGL	phosphorylase, glycogen, liver
0.495511	-0.1186401	MC2R	melanocortin 2 receptor
0.493923	-0.1023234	GLI1	GLI family zinc finger 1
0.491892	-0.1042394	FAM105A	family with sequence similarity 105 member A
0.487203	0.5102427	MYH4	myosin heavy chain 4
0.483516	0.2350352	TRA2B	transformer 2 beta homolog (Drosophila)
0.481593	-0.1020385	GLP1R	glucagon like peptide 1 receptor
0.479352	-0.1123682	SRRT	serrate, RNA effector molecule
0.479095	-0.0977864	CER1	cerberus 1, DAN family BMP antagonist
0.476249	0.4827893	FKBP1B	FK506 binding protein 1B
0.476128	0.210521	ZNF140	zinc finger protein 140
0.47596	-0.0930088	R3HDM1	R3H domain containing 1
0.475243	0.2308053	PTEN	phosphatase and tensin homolog
0.475238	0.2746426	LIMA1	LIM domain and actin binding 1
0.473983	0.1578959	CT55	cancer/testis antigen 55
0.466397	0.1736773	VAPB	VAMP associated protein B and C
0.463171	0.2388372	ASAH1	N-acylsphingosine amidohydrolase 1
0.461566	-0.1472289	RPS28	ribosomal protein S28
0.459255	-0.098774	TTTY1	testis-specific transcript, Y-linked 1 (non-protei
0.458287	-0.0984333	LEP	leptin
0.45786	0.3110428	EPB41L1	erythrocyte membrane protein band 4.1 like 1
0.454751	-0.1196224		
0.453437	-0.2028045	IL6	interleukin 6
0.45274	-0.1371984	ABO	ABO blood group (transferase A, alpha 1-3-N-ac
0.452317	-0.0989831		
0.451282	-0.1018515	MTUS2	microtubule associated tumor suppressor canc
0.448992	0.2632658	ITGB5	integrin subunit beta 5
0.448366	-0.0710263	FPR2	formyl peptide receptor 2
0.441623	-0.0865161		

0.44151	-0.0883758	HNRNPF	heterogeneous nuclear ribonucleoprotein F
0.440334	-0.20085	CAMK1D	calcium/calmodulin dependent protein kinase
0.439512	-0.0672913	IFNA10	interferon alpha 10
0.438143	-0.1076953	GPR12	G protein-coupled receptor 12
0.437931	-0.6759745		
0.437443	-0.0964818	CDK20	cyclin dependent kinase 20
0.43739	0.1440676	RAB3GAP1	RAB3 GTPase activating protein catalytic subun
0.435325	-0.116003	NOTCH2	notch 2
0.43495	0.1072729	COQ7	coenzyme Q7, hydroxylase
0.433717	0.2289997	AK2	adenylate kinase 2
0.432684	0.1493361	WHRN	whirlin
0.432365	-0.0876298	C22orf31	chromosome 22 open reading frame 31
0.432138	-0.1211449	GPR157	G protein-coupled receptor 157
0.42885	0.1728087	NAALADL1	N-acetylated alpha-linked acidic dipeptidase-li
0.425565	-0.1072732	SLC12A4	solute carrier family 12 member 4
0.424795	0.2035136	HNRNPF	heterogeneous nuclear ribonucleoprotein F
0.423955	-0.1486404	PLA2G5	phospholipase A2 group V
0.423219	-0.0912582	IRX4	iroquois homeobox 4
0.422099	-0.1542522		
0.421549	-0.1200519	VWA8	von Willebrand factor A domain containing 8
0.420222	0.1149286	ZNF135	zinc finger protein 135
0.418015	-0.1221683	LDB3	LIM domain binding 3
0.417323	-0.0694683	WFDC8	WAP four-disulfide core domain 8
0.41693	-0.1399846	HOXB8	homeobox B8
0.416593	-0.0801293	TUBB4BP7	tubulin beta 4B class IVb pseudogene 7
0.415648	0.205817	TXNDC15	thioredoxin domain containing 15
0.410002	0.275337	PYROXD1	pyridine nucleotide-disulphide oxidoreductase
0.409976	-0.0992437	HEYL	hes related family bHLH transcription factor wit
0.409459	0.0833095	SHC1P1	SHC adaptor protein 1 pseudogene 1
0.408289	-0.1347109	FGFR2	fibroblast growth factor receptor 2
0.406269	-0.2278843	LZTFL1	leucine zipper transcription factor like 1
0.405661	-0.1134535	ZNF576	zinc finger protein 576
0.405246	-0.1323943	ADCY9	adenylate cyclase 9
0.403699	0.4244441	FABP4	fatty acid binding protein 4
0.403301	0.1051388	BNIP1	BCL2 interacting protein 1
0.403067	0.1191985	EEF1A1	eukaryotic translation elongation factor 1 alpha
0.402182	-0.1011382	PHF7	PHD finger protein 7
0.402089	-0.2182698	KMT2A	lysine methyltransferase 2A
0.401305	-0.1109084		
0.400839	0.0983034	GPR137	G protein-coupled receptor 137
0.397326	0.2105921	FBXO11	F-box protein 11
[truncated: 690,662 more chars]
